# A genotyping array for the globally invasive vector mosquito, *Aedes albopictus*

**DOI:** 10.1186/s13071-024-06158-z

**Published:** 2024-03-04

**Authors:** Luciano Veiga Cosme, Margaret Corley, Thomas Johnson, Dave W. Severson, Guiyun Yan, Xiaoming Wang, Nigel Beebe, Andrew Maynard, Mariangela Bonizzoni, Ayda Khorramnejad, Ademir Jesus Martins, José Bento Pereira Lima, Leonard E. Munstermann, Sinnathamby N. Surendran, Chun-Hong Chen, Kevin Maringer, Isra Wahid, Shomen Mukherjee, Jiannon Xu, Michael C. Fontaine, Elizabet L. Estallo, Marina Stein, Todd Livdahl, Patricia Y. Scaraffia, Brendan H. Carter, Motoyoshi Mogi, Nobuko Tuno, James W. Mains, Kim A. Medley, David E. Bowles, Richard J. Gill, Roger Eritja, Ranulfo González-Obando, Huynh T. T. Trang, Sébastien Boyer, Ann-Marie Abunyewa, Kayleigh Hackett, Tina Wu, Justin Nguyễn, Jiangnan Shen, Hongyu Zhao, Jacob E. Crawford, Peter Armbruster, Adalgisa Caccone

**Affiliations:** 1 Department of Ecology and Evolutionary Biology, Yale University, New Haven, CT 06520-8105 USA; 2 Department of Biological Sciences, University of Notre Dame, Notre Dame, IN USA; 3 Department of Population Health and Disease Prevention, University of California, Irvine, CA USA; 4 School of the Environment, University of Queensland Australia, St Lucia, Australia; 5 Department of Biology and Biotechnology “Lazzaro Spallanzani”, University of Pavia, Pavia, Italy; 6 Laboratório de Fisiologia e Controle de Artrópodes Vetores, Instituto Oswaldo Cruz, FIOCRUZ, Rio de Janeiro, RJ Brazil; 7 Yale School of Public Health and Yale Peabody Museum, Yale University, New Haven, CT USA; 8 Department of Zoology, Faculty of Science, University of Jaffna, Jaffna, Sri Lanka; 9 National Health Research Institutes, National Mosquito-Borne Disease Control Research Center & National Institute of Infectious Diseases and Vaccinology, Miaoli, Taiwan; 10 Pirbright Institute, Pirbright, England UK; 11 Center for Zoonotic and Emerging Diseases, Hasanuddin University Medical Research Centre (HUMRC), Makassar, Indonesia; 12 Mitrani Department of Desert Ecology, Jacob Blaustein Institutes of Desert Research, Ben-Gurion University of the Negev, Midreshet Ben-Gurion, Israel; 13 Biological and Life Sciences Division, School of Arts and Sciences, Ahmedabad University, Ahmedabad, Gujarat India; 14 Department of Biology, New Mexico State University, Las Cruces, NM USA; 15 MIVEGEC, Université de Montpellier, CNRS, IRD, Montpellier, France; 16 University of Groningen, Groningen Institute for Evolutionary Life Sciences, Groningen, The Netherlands; 17 Facultad de Ciencias Exactas, Físicas y Naturales, Centro de Investigaciones Entomológicas de Córdoba, Universidad Nacional de Córdoba, Córdoba, Argentina; 18 Instituto de Investigaciones Biológicas y Tecnológicas, Consejo Nacional de Investigaciones Científicas y Técnicas, Universidad Nacional de Córdoba, Córdoba, Argentina; 19 Instituto de Medicina Regional, Universidad Nacional del Nordeste, CONICET CCT Nordeste, Resistencia, Argentina; 20 Clark University, Worcester, MA USA; 21 School of Public Health and Tropical Medicine, Tulane University, New Orleans, LA USA; 22 Division of Parasitology, Faculty of Medicine, Saga University, Nabeshima, Saga, Japan; 23 Laboratory of Ecology, Graduate School of Natural Science and Technology, Kanazawa University, Kanazawa, Japan; 24 MosquitoMate Inc., Lexington, KY USA; 25 Tyson Research Center, Washington University in St. Louis, St. Louis, USA; 26 US National Park Service, Washington, USA; 27 Department of Life Sciences, Georgina Mace Centre for the Living Planet, Imperial College London, Berkshire, UK; 28 Centre d’Estudis Avançats de Blanes, Consejo Superior de Investigaciones Científicas, Blanes, Spain; 29 Department of Biology, Universidad del Valle, Calle, Colombia; 30 Department of Medical Entomology and Zoonotics, Pasteur Institute in Ho Chi Minh City, Ho Chi Minh City, Vietnam; 31 Medical Entomology Unit, Institut Pasteur du Cambodge, Phnom Penh, Cambodia; 32 Department of Biostatistics, Yale School of Public Health, New Haven, CT 06510 USA; 33 Department of Genetics, Yale University School of Medicine, New Haven, CT 06510 USA; 34 Verily Life Sciences, San Francisco, CA USA; 35 Department of Biology, Georgetown University, Washington, DC USA

**Keywords:** *Aedes albopictus*, SNP chip, Validation, Population genomics

## Abstract

**Background:**

Although whole-genome sequencing (WGS) is the preferred genotyping method for most genomic analyses, limitations are often experienced when studying genomes characterized by a high percentage of repetitive elements, high linkage, and recombination deserts. The Asian tiger mosquito (*Aedes albopictus*), for example, has a genome comprising up to 72% repetitive elements, and therefore we set out to develop a single-nucleotide polymorphism (SNP) chip to be more cost-effective. *Aedes albopictus* is an invasive species originating from Southeast Asia that has recently spread around the world and is a vector for many human diseases. Developing an accessible genotyping platform is essential in advancing biological control methods and understanding the population dynamics of this pest species, with significant implications for public health.

**Methods:**

We designed a SNP chip for *Ae. albopictus* (Aealbo chip) based on approximately 2.7 million SNPs identified using WGS data from 819 worldwide samples. We validated the chip using laboratory single-pair crosses, comparing technical replicates, and comparing genotypes of samples genotyped by WGS and the SNP chip. We then used the chip for a population genomic analysis of 237 samples from 28 sites in the native range to evaluate its usefulness in describing patterns of genomic variation and tracing the origins of invasions.

**Results:**

Probes on the Aealbo chip targeted 175,396 SNPs in coding and non-coding regions across all three chromosomes, with a density of 102 SNPs per 1 Mb window, and at least one SNP in each of the 17,461 protein-coding genes. Overall, 70% of the probes captured the genetic variation. Segregation analysis found that 98% of the SNPs followed expectations of single-copy Mendelian genes. Comparisons with WGS indicated that sites with genotype disagreements were mostly heterozygotes at loci with WGS read depth < 20, while there was near complete agreement with WGS read depths > 20, indicating that the chip more accurately detects heterozygotes than low-coverage WGS. Sample sizes did not affect the accuracy of the SNP chip genotype calls. Ancestry analyses identified four to five genetic clusters in the native range with various levels of admixture.

**Conclusions:**

The Aealbo chip is highly accurate, is concordant with genotypes from WGS with high sequence coverage, and may be more accurate than low-coverage WGS.

**Graphical Abstract:**

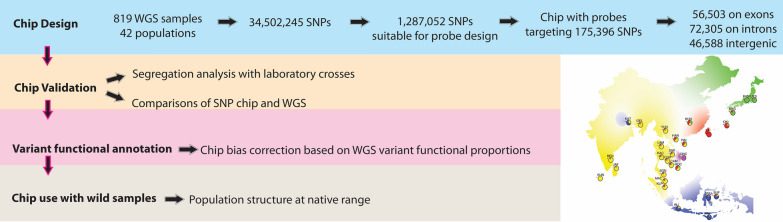

**Supplementary Information:**

The online version contains supplementary material available at 10.1186/s13071-024-06158-z.

## Background

*Aedes albopictus*, the Asian tiger mosquito, is native to Asia [1]. This species is known for its aggressive daytime biting behavior and adaptability to various environments, including temperate climates, facilitated by its ability to diapause during winter [1]. Over the past few decades, it has spread to other parts of the world, including the Americas, Africa, and Europe, primarily due to human activities [2].

*Aedes albopictus* is a public health concern, given its ability to act as a vector for a wide variety of viruses, including dengue, Zika, chikungunya, and West Nile viruses [3]. Public health authorities have implemented several control measures to reduce this species’ populations and prevent the spread of vector-borne illnesses [2]. These include monitoring its presence and tracing origins of invasive strains, since vectorial capacities and the ability to persist in temperate climates via diapause differ among strains [4].

To help support these control and monitoring goals, we developed a single-nucleotide polymorphism (SNP) chip array for *Ae. albopictus*. A SNP chip is a high-throughput genotyping technology that detects variations in DNA sequences, involving a change in a single nucleotide within the DNA sequence [5], the most common type of genetic variation. Researchers use various types of genomic data to select SNPs for developing a SNP chip: from whole-genome sequencing (WGS) that captures both common and rare SNPs across the whole genome, to double digest restriction site-associated DNA sequencing (ddRAD), transcriptome sequencing, and exome sequencing that select SNPs using only part of the genomic variation.

The development of a 50-K SNP chip for *Ae. aegypti* marked a significant advancement in our understanding of the mosquito's evolutionary history on a global scale [6–14]. This high-density SNP chip not only shed light on the complex evolutionary pathways of *Ae. aegypti* but also proved instrumental in conducting genome-wide association studies (GWAS) [15]. The Aealbo chip encompasses an even more significant number of markers, offering enhanced potential for detailed genomic analysis. This increased marker density in the Aealbo chip promises more comprehensive insights than those obtained from the *Ae. aegypti* chip, paving the way for deeper exploration into the genetic intricacies of these mosquito species.

Based on our results, the chip provides a notable improvement over previous tools such as allozymes, microsatellites, mitochondrial DNA, and restriction site-associated sequencing (RADseq) [16–19] in swiftly pinpointing the origins of new invasions. The chip facilitates the genetic mapping of critical traits such as vector competence, insecticide resistance, and diapause, which are paramount in combating the spread and impact of this species. Moreover, the SNP chip array is not only cost-effective but also simplifies the process, eliminating the need for extensive bioinformatics workflows typically associated with high-performance computing environments [20], which might be available in developing countries. With this chip, the user gets a streamlined, efficient solution that accelerates research and response strategies against one of the world’s most notorious invasive vectors.

Developing a dedicated SNP chip represents a significant advancement to address the critical challenges posed by *Ae. albopictus*. This technology is a pivotal tool for unraveling the complex genetic diversity and population structure of *Ae. albopictus*. Its application extends beyond fundamental scientific inquiry because of its implications for vector control strategies and disease transmission studies. By facilitating detailed genetic analysis, the SNP chip will enable researchers to track the spread of this species and understand its adaptation mechanisms. Integrating this technology into ecological monitoring and management programs will enhance our ability to predict and mitigate the impacts of this globally invasive species.

We report the development of a SNP chip for *Ae. albopictus* using WGS data from populations of mosquitoes collected worldwide, its validation by carrying out technical replicates, segregation analyses on multiple families to test Mendelian inheritance consistent with single-copy genes, and a comparison with WGS data from the same individuals. We then used the chip for a population genomic analysis of samples from across the species’ native range to evaluate the SNP chip’s performance.

## Methods

**Fig. 1:**
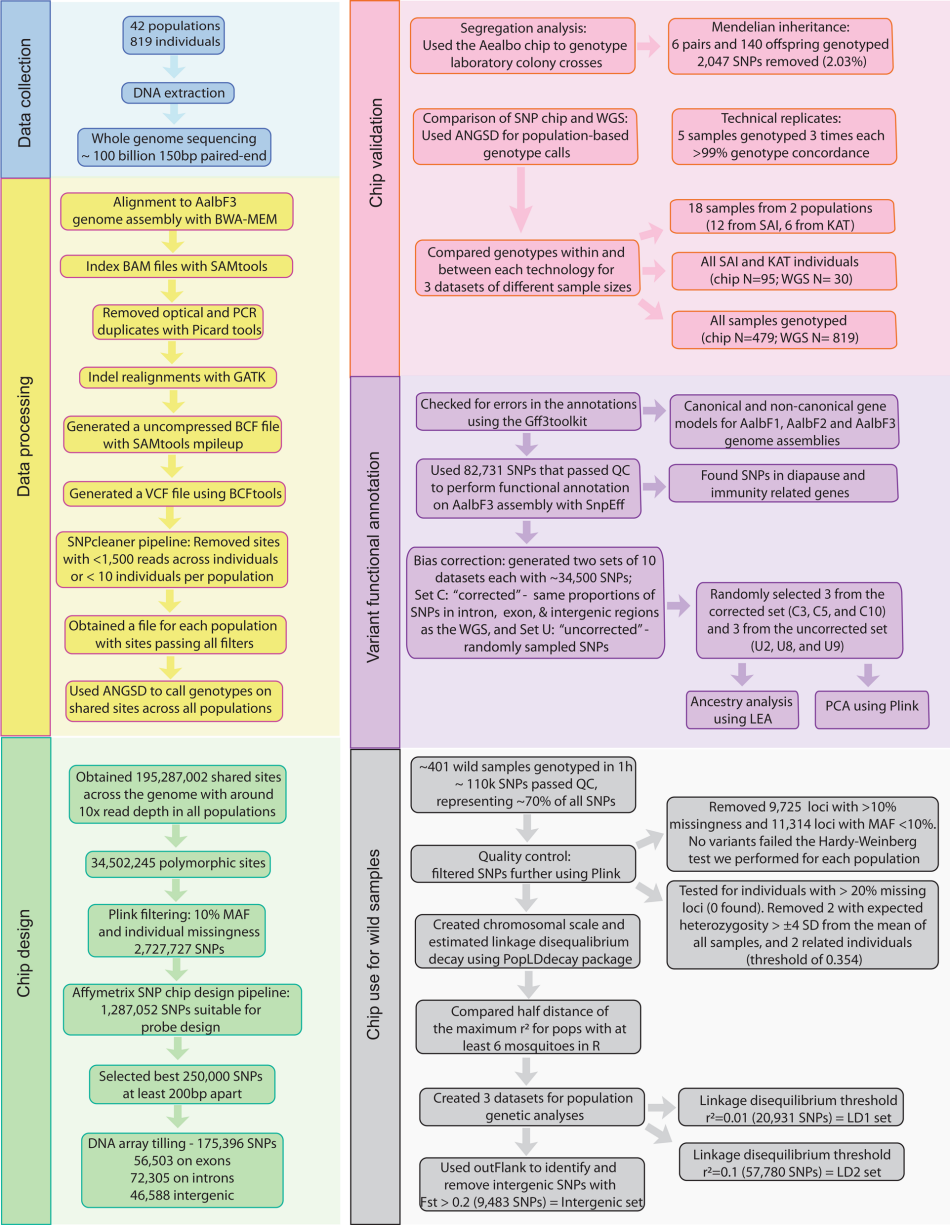
Pipeline describing the process to produce, validate, and use the Aealbo SNP chip. Different colors identify significant steps. Arrows provide the direction of the workflow. Steps include the method used, the number of SNPs obtained and/or removed at each step, the number of samples used, and dataset types used for the different analyses. Details are in the main text and supplementary materials files

The overall process for our chip design and validation is described in Fig. 1. We provide all the data and Markdown/HTML files (Additional files 1, 2, 3, 4, 5, 6, 7, 8, 9, 10, 11, 12, 13, 14, 15, 16: Files S1–S16 and Additional file 26: Table S1) showing the step-by-step procedure for all analyses and the results of ancestry analyses run on the Yale High-Performance Computing (HPC) clusters to facilitate analysis replication. The raw data and all the files required to replicate analyses are available in Zenodo (https://doi.org/10.5281/zenodo.10048029), and the code is also available in GitHub (link).

### Samples and dataset

**Fig. 2:**
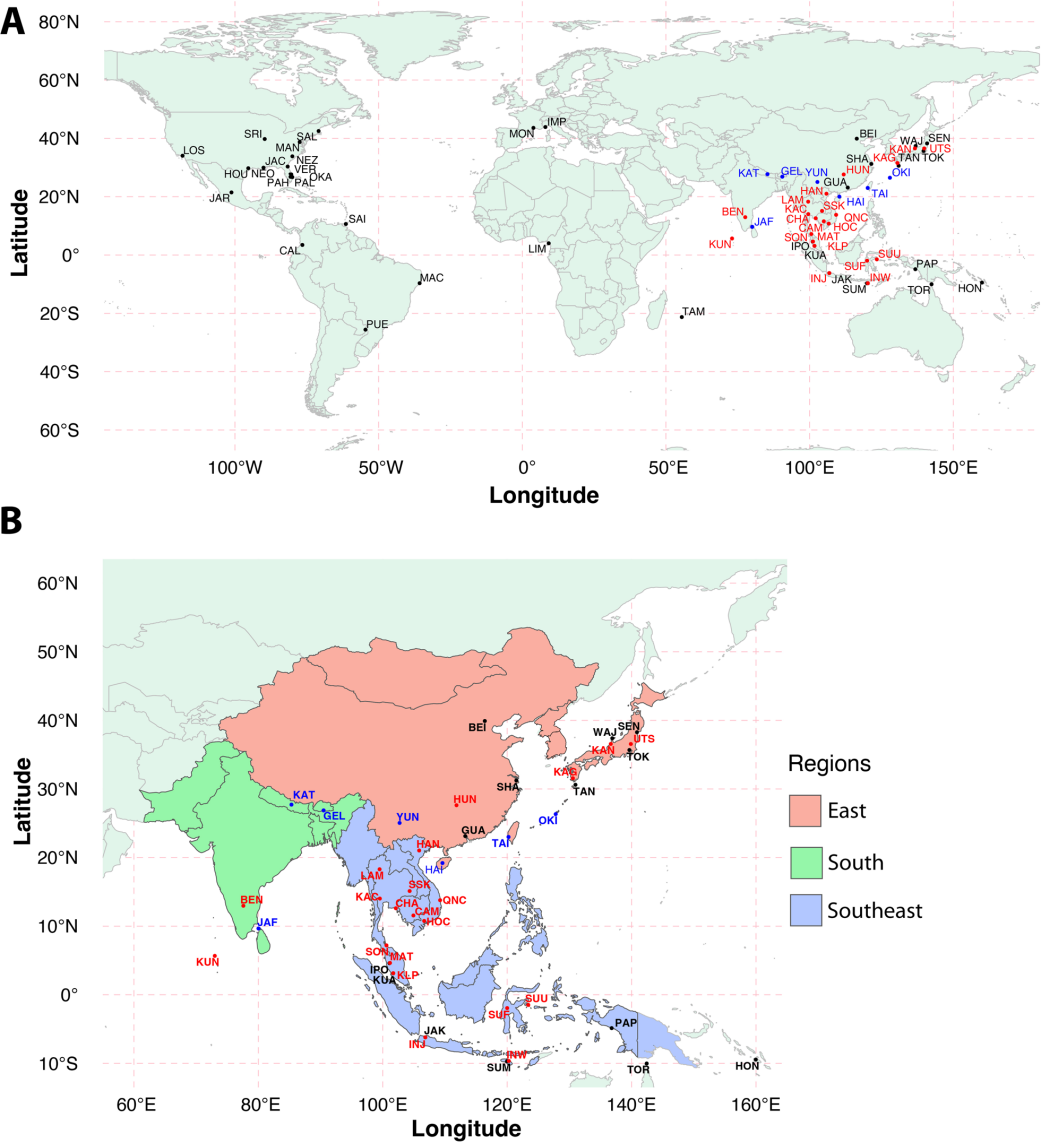
Locations of the samples used to identify the SNPs **A** using WGS data (black dots) and genotyped using the chip (red dots) or both (blue dots). A three-letter code identifies each locality. **B** Details of the Asian sampling localities. The shaded areas (East, South, and Southwest) identify the main geographical regions. Details on the sample localities, abbreviations, and number of samples per locality are reported in Additional file 26: Tables S2 and S3

To identify variable SNPs, we used WGS data from 819 individuals worldwide (see Additional file 26: Table S2, Fig. 2) that were sequenced as part of the Aalbo1200 genomes project [21]. Overall, we genotyped 401 samples with the chip, including single-pair matings from two laboratory colonies (*N* = 152; two crosses within an invasive North American population—“MAN”, three crosses from a native Malaysian sample—“KLP”, one cross between MAN × KLP) (Additional file 25: Fig. S1) and wild-caught animals (*N* = 261) (Additional file 26: Table S3). For the technical replicates analyses, we used three replicates for five randomly selected samples, genotyping the same samples three times (Additional file 26: Tables S4 and S5). For the comparison between the SNP chip and WGS, we created different datasets to compare the impact of different sample sizes (Additional file 25: Fig. S2). Datasets a and y included 18 samples from two sampling sites, Kathmandu, Nepal (KAT, *N* = 6) and Saint Augustine, Trinidad and Tobago (SAI, *N* = 12; Tables 1 and 2) that were genotyped using both platforms. Datasets b and x include all the samples that were genotyped from the two sampling sites above (SNP chip *N* = 95; WGS *N* = 30). Datasets c and w included all the samples genotyped with both methods (SNP chip *N* = 479; WGS *N* = 819). For the geographical population genomic analyses, we used 237 samples from 28 sampling sites (Fig. 2, Additional file 26: Table S3) from the native range of the species.

**Table 1:** Summary of the SNP mismatch percentage (zygosity) for individuals from two populations (KAT and SAI)

Population	Sample ID	ab	ac	bc	yx	yw	xw	ay	bx	cw
KAT	7	0.91	1.00	0.30	1.28	2.51	2.61	5.51	4.72	4.60
KAT	8	1.01	1.09	0.31	1.27	2.52	2.66	5.96	5.13	4.94
KAT	9	0.87	0.95	0.26	1.09	2.03	2.15	5.07	4.27	4.19
KAT	10	0.88	0.99	0.31	1.31	2.63	2.74	5.52	4.78	4.63
KAT	11	1.00	1.12	0.32	1.19	2.39	2.48	6.30	5.43	5.25
KAT	12	1.01	1.12	0.31	1.31	2.54	2.68	5.32	4.36	4.17
KAT mean		0.95	1.05	0.30	1.24	2.44	2.55	5.61	4.78	4.63
SAI	1	1.41	1.55	0.49	0.97	2.27	2.30	9.03	7.83	7.34
SAI	2	1.29	1.50	0.49	1.08	2.61	2.65	9.00	7.91	7.39
SAI	3	1.15	1.34	0.44	1.83	4.31	4.42	9.74	8.79	8.12
SAI	4	1.42	1.59	0.50	1.94	4.51	4.62	10.37	9.43	8.69
SAI	5	1.17	1.30	0.41	2.30	5.37	5.56	10.90	10.00	9.20
SAI	12	1.23	1.36	0.42	0.87	1.99	2.06	8.43	7.31	6.86
SAI	13	1.23	1.38	0.47	1.57	3.60	3.70	9.48	8.46	7.93
SAI	14	1.43	1.56	0.50	1.20	2.85	2.90	9.62	8.43	7.80
SAI	15	1.20	1.32	0.44	0.58	1.38	1.42	8.31	7.32	6.83
SAI	16	1.21	1.42	0.45	1.07	2.40	2.44	8.24	7.12	6.65
SAI	17	1.19	1.34	0.42	1.86	4.33	4.46	9.64	8.69	8.10
SAI	18	1.23	1.38	0.46	1.54	3.65	3.77	9.48	8.51	7.86
SAI mean		1.42	0.46	1.40	3.36	3.27	3.36	9.35	8.31	7.73
Overall mean		1.16	1.30	0.41	1.35	2.99	3.09	8.11	7.14	6.70

Two types of pairwise comparisons are presented: (1) the same methodology (WGS or SNP chip) but between datasets with different sample sizes (datasets a, b, and c for the SNP chip data and y, x and w for the WGS data; Additional file 25: Fig. S2), and (2) between methodologies (ay, bx, cw). The second column lists the individuals within each population, numbered by population. The following columns report the percent mismatches within and between datasets for that individual. “Mean” refers to the mean values per population. “Overall mean” refers to the mean for all the samples (last line)

**Table 2:** Cumulative SNP mismatch percentage (zygosity) across each population in pairwise comparisons

Population	Times	ab	ac	bc	yx	yw	xw	ay	bx	Cw
KAT	1	3.06	3.36	1.02	2.99	6.35	6.07	18.54	16.56	16.14
KAT	2	1.24	1.38	0.37	1.95	4.01	3.73	9.93	8.89	8.61
KAT	3	0.65	0.72	0.19	1.32	2.66	2.46	6.07	5.51	5.39
KAT	4	0.36	0.40	0.09	0.85	1.70	1.59	3.54	3.27	3.32
KAT	5	0.17	0.18	0.05	0.49	1.02	0.95	1.76	1.85	1.91
KAT	6	0.10	0.11	0.03	0.21	0.50	0.50	1.00	1.16	1.25
SAI	1	8.32	9.51	3.19	5.19	12.91	12.46	53.31	48.64	45.80
SAI	2	3.19	3.58	1.06	3.43	8.56	8.16	31.82	28.23	25.84
SAI	3	1.53	1.71	0.48	2.60	6.38	6.06	19.09	16.78	15.10
SAI	4	0.78	0.88	0.26	1.98	4.81	4.56	11.39	10.04	9.06
SAI	5	0.41	0.46	0.14	1.47	3.60	3.42	6.81	6.17	5.64
SAI	6	0.24	0.26	0.08	1.06	2.64	2.53	4.12	3.87	3.72
SAI	7	0.14	0.14	0.05	0.74	1.85	1.80	2.49	2.55	2.57
SAI	8	0.10	0.10	0.03	0.48	1.25	1.24	1.50	1.73	1.84
SAI	9	0.07	0.06	0.03	0.28	0.76	0.80	0.92	1.22	1.40
SAI	10	0.06	0.05	0.02	0.14	0.38	0.44	0.57	0.90	1.05
SAI	11	0.05	0.04	0.02	0.06	0.14	0.21	0.31	0.56	0.71
SAI	12	0.03	0.02	0.01	0.01	0.02	0.05	0.13	0.29	0.40

Times indicate different genotype calls across all samples within the two populations KAT (*n* = 6) and SAI (*n* = 12), both within (columns ab, ac, bc, yx, yw, xw) and between (columns ay, bx, cw) using the SNP chip (datasets a, b, and c) or the WGS datasets (y, x, and w)

### Genetic analyses

DNA was extracted from either whole or partial ethanol-preserved adults or larvae using the DNeasy Blood and Tissue kit (Qiagen), following the manufacturer’s instructions for purification of total DNA from insects, with the following modifications: instead of a mortar and pestle or electric homogenizer, we used a bead beater and then lysed samples overnight on a Thermomixer. We eluted the samples in 100–200 μl of 1% TE buffer in the final step. We also performed an additional step of treating our samples with 4 μl of RNase A (Qiagen). We stored all samples at −20 °C, and then we concentrated and purified them using Amicon^®^ Ultra 30 kDA centrifugal filter devices (Millipore), according to the manufacturer's instructions. We obtained approximately 23 μl of elute. Next, we checked the genomic DNA concentration using Qubit 1X dsDNA [double-stranded DNA] HS Assay Kits (Invitrogen).

For larvae and other samples that could not be identified to species using morphology (*N* = 75), we checked species assignments by sequencing a 1537-base-pair (bp) fragment of the mitochondrial cytochrome oxidase subunit 1 *COI* gene [22] (Additional file 26: Table S7) and comparing it to the species known sequences for the same fragment. Protocols for polymerase chain reaction (PCR) amplification and sequencing are in supplementary material (Additional file 17: File S17).

### Chip design

We used 819 WGS samples from populations worldwide (Fig. 2, Additional file 26: Table S2) to identify SNPs and design probes for the chip using these data. We then confirmed the accuracy of the chip through laboratory crosses, segregation studies, and technical replicates. We also compared the WGS and SNP chip data to identify and rectify possible inconsistencies (Additional file 25: Fig. S2).

*SNP discovery*: We processed 819 WGS datasets generated by Verily Life Sciences to discover SNP in the *Ae. albopictus* AalbF3 genome assembly [23]. Our objective was to carry out definitive, unambiguous genotype determinations—known as hard genotype calls—at SNP sites that met a minimum coverage of 10×. The data were exported in Plink format [24] for downstream analyses. The step-by-step methodology for selecting SNPs for the chip is detailed in the supplementary material (Additional file 1: File S1).

The WGS sample, detailed in Additional file 26: Table S2, averaged 100 million 150-bp paired-end reads per sample, with an average coverage of 12×. Procedures for the WGS data collection are reported in the Aalbo1200 genome project [21].

*SNP selection for probe design*: SNPs suitable for chip design must possess a high minor allele frequency, be distributed evenly across the genome, represent the geographical variation in the target taxa, or be near or within coding regions (if one of the goals is to screen for adaptive variation). Furthermore, it is important that selected SNPs are located in regions with good kinetic hybridization properties to ensure reliable and accurate genotyping. For probe design, we used a minor allele frequency (MAF) filter of 10% to select SNPs, allowing missingness of 10% for both SNPs and individuals; these criteria led to identification of ~ 2.7 million SNPs. All SNPs were scored for probe design using the Affymetrix SNP chip design pipeline (Affymetrix, Santa Clara, CA, USA), in consideration of which probe pairs would perform well based on thermodynamics, self-hybridization, and copy number present in the reference genome (Fig. 1). This process identified ~ 1.3 million SNPs. Of these, we selected 250,000 with the highest “pconvert” (an overall metric of probe performance) for array tilling. We used a mix of bash commands, Bedops [25], and the R package GenomicRanges [26] to select approximately 30% of the SNPs to be located on exons, introns, and intergenic regions, with those on coding regions set to be at least 200 bp apart. We also selected monomorphic sites within our samples for the Dish quality control (DQC) metric, a control method that measures signals at invariable sites to differentiate true signals from background.

*Identifying and mapping probe sequences to all Ae. albopictus genome assemblies*: Probes in SNP chip design are short DNA sequences, 71 nucleotides long, that match the flanking sequence of the target SNP based on a specific genome assembly. These probes bind specifically to the corresponding SNP location on a sample’s DNA. Thus, a probe’s hybridization efficiency and specificity set the accuracy of SNP detection, which often features multiple probes for each SNP. For this step, having an accurate genome assembly is essential, as it serves as a reference for probe design and SNP location. Given that for this species there are five genome assemblies from the National Center for Biotechnology Information (NCBI) and/or VectorBase, we mapped the probe sequences to each one of them (AalbF1 [27], AalbF2 [28], AalbF3 [23], AalbCell [29], AalbRimini [30]) and also mapped the probe sequences to the high-quality genome assembly of *Ae. aegypti* (AaegL5 [31]). Additional file 2: File S2 describes the step-by-step procedure to map the probe sequences using BWA(Burrows–Wheeler Aligner). In Additional files 2, 26: File S2 and Table S7, we provide an overview of the quality and characteristics of several genome assemblies used for probe sequence mapping.

*Chip manufacturing*: The physical chip was manufactured by Affymetrix using the selected probes replicated 2–4 times on the array, using a 96-well format. A total of 404,514 probes were tiled in each chip targeting the 175,396 polymorphic sites (Additional file 18: File S18). Each chip can genotype 95 samples, with one well used as control.

*SNP chip genotyping*: Before sending samples for genotyping on the chip, we normalized their DNA concentrations to 20 ng/μl and placed 20 μl of sample into each well. To assess performance of the probes after design, the Affymetrix’s bioinformatics team used samples of known genealogical relationship to create the so-called library files, including SNP-specific priors, which optimize the array genotyping calling algorithm. To do this, they used genotype calls from six of the crosses we performed (*N* = 152, Additional files 25, 26: Fig. S1 and Table S3) along with information describing the samples and their relationships. Using these data, the Affymetrix team developed the “library files” necessary to do genotype calls with the freely available Axiom Analysis Suite Software v.5.1.1.

### Chip validation

*Segregation analysis*: We conducted a segregation analysis using six laboratory crosses to evaluate genetic inheritance patterns and potential discrepancies (Additional file 25: Fig. S1). We genotyped these crosses (*N* = 152), testing SNPs for which at least one parent showed heterozygosity to bolster our chances of pinpointing segregation errors. Subsequently, we predicted the allele frequency in the offspring from each cross, given the parental genotypes. This approach enabled us to scrutinize varying SNP sets within each cross. Using Plink [18], we quantified the actual allele frequency observed in the offspring. We processed the data in R to compare the expected versus observed frequencies, considering any missing data among the offspring to estimate the frequencies, and using a Chi-square test to evaluate the significance of their differences. We used the Fisher method to combine the *P*-values of SNPs that appeared in multiple crosses and applied the Holmes correction for multiple testing [32]. SNPs with adjusted *P*-values below 0.05 failed the segregation test, indicating a significant difference between expected and observed allele frequencies. File S3 contains the step-by-step procedure along with the associated code and data.

*Comparing technical replicates*: To assess the accuracy of the chip and genotype parameters, we genotyped one sample from five populations three times (Additional file 26: Table S4). We then conducted pairwise comparisons for each technical replicate (Additional file 26: Table S5). The step-by-step procedure for these analyses is described in Additional file 5: File S5.

*Comparison of WGS and chip genotypes*: To ascertain potential congruence in genotype calls between the WGS and SNP chip, we genotyped samples using both methods. We also compared three datasets of different sizes genotyped using both methods (Additional file 25: Fig. S2) to evaluate the impact of sample size on the estimates of genotype frequencies, identify factors causing possible genotype mismatches, and assess the impact of those discrepancies on downstream analyses. We compared WGS sample sizes of 18, 30, and 819, as this could reveal shifts in allele-specific read depth that could be sample size-dependent. For instance, the small sample size set (*N* = 18) may have inconsistent read depths among individuals due to sequencing anomalies or individual-specific biases, while the 30 samples set is likely to provide a more balanced read depth. We used ANGSD, a population-based algorithm, where the sample size does affect the genotype likelihoods or genotypes called. In the large dataset (*N* = 819), allele-specific read depth should have the highest reliability, as individual variances average out, most accurately reflecting allele frequencies at common sites. For the SNP chip datasets of different sizes, similar considerations hold as the one indicated above for the WGS datasets. For all analyses. we only used the 175,396 SNP sites that were in common between the WGS and the SNP chip datasets and were selected for array tilling. We use ANGSD [33] to perform the population-based genotype calls using the Samtools model [34]. We compared the genotypes within and between each technology using a combination of python and R scripts. We used Samtools [34] to obtain the number of reads mapping to each allele from the cram files (a compressed format used for storing aligned reads data), testing different numbers of samples, and then checked whether there was a correlation between read depth and genotype mismatches. The step-by-step analysis and comparisons are described in Additional file 4: File S4. Once we identified the SNPs contributing to genotype mismatches using the two methods, we removed them and performed principal component analysis (PCA) with Plink, to see whether that increased the result congruence.

### Variant functional annotation and SNP density

To perform the functional annotation of the SNPs that genotyped well on the wild samples, we used SnpEff [35] with the AalbF3 [11] annotation. We used the Gff3 toolkit [36] to check for errors in the annotation file for the AalbF3 since it was derived from the previous genome assembly annotation, AalbF2. To do this, we used canonical and non-canonical gene models. Canonical gene models represent the standard or primary form of a gene, detailing its most common arrangement of exons and introns. While genes can produce various transcripts through alternative splicing, the canonical transcript serves as the main or most representative version. Non-canonical gene models depict alternative, less common forms of genes and their transcripts, which might arise due to various genetic processes or annotations. These non-standard models provide insights into the diversity and complexity of gene expression and regulation in an organism. We provide the step-by-step of analyses in Additional file 6: File S6. For two sets of genes of interest, diapause- and immunity-related genes [23, 28], we checked how many of the SNPs on the chip were on these genes.

To find possible bias in the chip, we determined whether SNPs from the chip were proportionally represented in the same gene categories as SNPs from WGS. We then created datasets to test whether having different proportions had impacts on downstream analyses. During the chip design, we aimed to select at least one SNP in each exon or gene. This is useful for some of the chip applications, especially GWAS. Therefore, we designed the chip to have an overrepresentation of SNPs from coding regions. To obtain equal representation of SNPs between the chip and WGS data across different types of genomic features, we designed an analysis pipeline to sample SNPs in the chip data that match the same proportions of SNPs in intron, exons, and intergenic regions as in the WGS data. In our study, we initially created 10 distinct SNP sets. For detailed comparative analysis, we randomly selected two of these sets, with each set comprising around 34,500 SNPs. The first set was designed to sample SNP variants in the chip that, according to their functional annotation, followed the same proportions found in each WGS dataset. Of these, we selected three sets: C3, C5, and C10. The second set included randomly sampled SNPs. Among these, we selected three datasets: U2, U8, and U9 (Additional file 26: Table S8). On these six datasets, we carried out ancestry analysis and PCA to evaluate whether there was a bias when using the SNP chip datasets corrected to reflect the same proportions of SNPs in different genomic features as in the WGS dataset compared to the uncorrected ones (Additional file 26: Table S8).

### Analyses of wild samples

*Quality control for chip data*: Once we genotyped all the samples, we further reduced the number of SNPs for downstream analyses, using stringent quality control measures to filter the SNPs dataset using Plink v 1.9 or v 2.0. We discarded either samples or SNPs that failed these quality control steps, which are described in detail in Additional file 7: File S7. Briefly, quality control filtering consisted of removing (1) loci with more than 10% missingness, (2) individuals with more than 20% missing loci, (3) loci that failed Hardy–Weinberg tests, with a threshold of 0.00001 for each population, (4) loci with a minor allele frequency smaller than 10%, (5) samples whose expected heterozygosity values deviated more than ±4 standard deviations from the mean of all samples, which might indicate low DNA quality, contamination, or high inbreeding [18], and (6) related individuals using a relatedness score of 0.354 to identify monozygotic twins and duplicate samples.

*Linkage analysis*: First, we created a chromosomal scale by merging the scaffolds into chromosomes, following the order provided in the AalbF3 genome assembly [23] (Additional file 8: File S8). Next, we utilized the PopLDdecay package [37] to estimate linkage disequilibrium (LD) (that is, non-random association of alleles at different loci) for populations with at least six individuals, applying parameters of a minor allele frequency (MAF) up to 1% and allowing for a genotype missingness of up to 20% (Additional file 26: Table S9). Subsequently, we calculated the LD half-life or half-distance in R to estimate the genetic distance at which the correlation between allele pairs reduces to half its original value. We then compared the LD half-life estimates across chromosomes to evaluate possible differences among chromosomes and looked at the correlation between sample size and LD half-life. Because the estimates were done for each population separately, the number of SNPs varied among comparisons (Additional file 26: Table S9).

*Creating datasets for population genetic analyses*: We evaluated how adjusting the LD thresholds for pruning impacted the outcomes of downstream analyses. Different LD levels modify the distribution of allele frequencies, potentially altering fixation index (F_ST_) calculations and skewing genetic differentiation interpretations. Linked loci, due to their proximity on a chromosome, often carry similar genetic information. This redundancy can obscure the true genetic variation within a dataset. By employing two different LD pruning strategies, we aimed to assess the extent to which this redundancy might affect the patterns observed in PCA. For ancestry analysis, the choice of LD threshold can influence the set of SNPs used, and this selection can shift ancestry proportions and assignments among individuals.

The *r*^2^ statistic in LD analysis represents the squared correlation coefficient between two alleles at different loci. It quantifies the strength and direction of the association between these alleles. When *r*^2^ approaches 1, there is a strong association, implying that knowing the allele at one locus can accurately predict the allele at the other. On the contrary, an *r*^2^ value close to 0 indicates a weak association, suggesting the alleles segregate independently. This measure is crucial in genetic studies, as it can identify genomic regions where variants are inherited together, possibly highlighting chromosomal regions containing genes under strong selection.

We used two different *r*^2^ values, 0.01 and 0.1, in our analyses. The rationale behind using a threshold of 0.01 is to ensure finer granularity to capture weak linkage disequilibria, while the 0.1 threshold removes highly correlated SNPs while preserving essential genetic information. Ultimately, the selected LD thresholds strike a balance, ensuring we neither lose critical data nor introduce biases from highly correlated variants. These thresholds also enhance computational efficiency, minimize potential biases, and align with standard practices for more straightforward comparisons with other studies. Finally, for our population structure analyses, we followed the recommendations of the algorithm’s manuals. For example, we used the recommended *r*^2^ threshold of 0.01 for Neural Admixture [38], and the recommended threshold of 0.1 for Admixture [39].

To evaluate the impact of different LD parameters on downstream population genomic analyses, we created two datasets with SNPs obtained using both *r*^2^ values: 0.01 (LD1: 20,931 SNPs) and 0.1 (LD2: 57,780 SNPs) (Additional file 26: Table S10). Using outFlank [19], we also created a dataset with quasi-neutral SNPs (intergenic: 9483 SNPs) by excluding intergenic SNPs with high genetic differentiation values, as measured by F_ST_ values (F_ST_ > 0.2; Additional file 7: File S7). These three SNP datasets (LD1, LD2, and intergenic) were used in the population genomic analyses to evaluate how using different genomic regions would impact results.

### Genetic ancestry, population structure, and differentiation

For ancestry analyses, we used four algorithms on each of the three SNP datasets (intergenic, LD1, and LD2)—Admixture [39], fastStructure [40], *sNMF-*LEA [41, 42], and Neural Admixture [38] (Additional file 26: Table S11)—to cross-check consistency across methodologies and validate the SNP chip data's effectiveness in ancestral analysis. Admixture and fastStructure differ in optimization procedures and priors; sNMF aligns closely with PCA methodology, and Neural Admixture is based on machine learning. We ran Admixture first with a wide range of *K* values (the number of subpopulations in structured populations; *K* = 1–25) to explore a wide spectrum of potential ancestral populations and identify potential substructures in the data that might be overlooked at smaller or larger *K* values. From the results of this analysis, we selected nine populations with low admixture percentages (YUN, OKI, KAN, UTS, TAI, BEN, INW, INJ, and QNC, Additional file 26: Table S3) to train the program Neural Admixture [38]. The exception was the population OKI, which showed admixture with two genetic clusters. We then used the trained data for inference with the entire dataset in Neural Admixture. We also reran Admixture with the populations we used to train Neural Admixture. We parsed the runs using pong [43] to find which runs had the most common mode. Two additional methods, implemented in the programs *sNMF*-LEA [41] and fastStructure [40], were also run on the full dataset. We describe the step-by-step procedure for each algorithm, including the number of runs and parameters, in Additional file 9: File S9 (Admixture), Additional file 10: File S10 (*sNMF*-LEA), Additional file 11: File S11 (fastStructure), and Additional file 12: File S12 (Neural Admixture). We also provide a summary in Additional file 26: Table S11. To provide a geographical visualization of the patterns of genomic differentiation, we used the R package tess3r [44] to interpolate the Q matrices from each algorithm over a map of Asia (Additional file 13: File S13). The Q matrix, derived from the algorithms, represents individual ancestry proportions for different numbers of ancestral groups. The Q matrix breaks down an individual’s genome into estimated fractions from various ancestral populations. Each row in this matrix corresponds to an individual, and each column represents an ancestral population. The values in the matrix, ranging from 0 to 1, represent the proportion of an individual's genome that can be attributed to each ancestral group. This matrix provides insights into population structure, migration patterns, and the admixture history of groups and individuals.

We also ran PCA to visualize patterns of genetic variation within and between groups, helping pinpoint significant axes of differentiation to complement the ancestry/clustering analyses described above. These analyses were run using the R package LEA [45], Plink [24], and adegenet [46, 47] (Additional files 7, 10: Files S7 and S10). We ran the PCA with all three SNP sets (intergenic, LD1, and LD2) to evaluate the reproducibility of the results.

We also ran PCA and clustering (LEA) analyses on the six SNP sets created to assess potential biases in the chip's coding regions (“corrected”: C3, C5, and C10, and “uncorrected”: U5, U8, and U9), described above in the section on variant functional annotation (Additional file 26: Table S8). We visualized all SNP sets together for PCA in a faceted plot crafted with ggplot2. Methods for the LEA analysis for the same six datasets are detailed in Additional file 14: File S14.

To quantify levels of genetic differentiation among sampling sites, we estimated the pairwise genetic distance (F_ST_) using the R package StTAMPP [48] for all three SNP datasets (intergenic, LD1, LD2). We calculated the F_ST_ values across each locus based on allele frequency and the level of heterozygosity, according to Weir and Cockerham [49], taking into account the population size. We used 100 bootstraps to estimate *P*-values and confidence intervals. The step-by-step procedure is described in Additional file 14: File S14.

We calculated the geographical distance (km) between sampling sites using the R package geosphere [50] and used the F_ST_ estimates from R package StTAMP [48] to evaluate whether there were significant correlations for all datasets (LD1, LD2, and intergenic). We fitted a linear regression model to the estimated values, where we predicted each F_ST_ variable based on the distance variable. After fitting the model for each country with at least three sampling localities, we extracted and computed the equation of the regression line and the coefficient of determination (*R*^2^). Next, we used the R package adegenet [47] to evaluate isolation by distance for all populations with at least four mosquitoes. We used the LD2 SNP set for this estimate and performed 999 random permutations between the genetic distance and the geographical distance (Additional file 14: File S14).

## Results

### Chip design

*SNP discovery and probe design*: The 819 WGS samples from 42 populations collected worldwide produced 195,287,002 shared sites across the genome with an average 10× read coverage across populations. The population-based genotype calls with ANGSD resulted in 34,502,245 polymorphic sites. After filtering with Plink for SNP and individual missingness (10%) and minor allele frequency (10%), we obtained 2,727,727 high-quality bi-allelic polymorphic sites for probe design (Additional file 23: File S23).

Affymetrix identified 1,287,052 SNPs suitable for the chip design. From these we selected the top 250,000 SNPs based on their genomic location and functional annotation, making sure the SNPs were at least 200 bp apart and aiming to have at least one SNP per exon. Out of this SNP set, Affymetrix tiled 175,396 SNP probes on the chip (56,503 SNPs on exons, 72,305 on introns, and 46,588 intergenic). The list of SNPs, their genomic location, and probe sequences are in Additional file 18: File S18. The p-convert is a metric that estimates the probability of successful SNP genotyping considering the probe thermodynamics and genomic alignment metrics. The mean p-convert for the probes in the chip was 0.71, suggesting the chip is likely to perform well.

*Mapping probe sequences to genome assemblies*: We selected the probe sequences identified by both algorithms BWA-MEM and BWA-ALN as having unique alignments in the AalbF3 genome assembly, with mapping quality > 20 and no secondary alignments (Additional file 25: Fig. S3), and compared this finding with alignment results from the other assemblies [21, 23, 24]. A total of 175,396 probes mapped with unique alignments to the AalbF3 assembly. Of these, ~ 96,000 probes mapped with unique alignments to the previous genome assembly (AalbF2), while 30 to 40% of the probes that aligned uniquely in AalbF3 had secondary alignments in the AalbF2 assembly. The genome assembly from the cell line [23] (AalbCell) had the lowest unique alignments, while this number increased with the most recent assemblies (Additional file 26: Tables S12 and S13).

### Chip validation

*Segregation analysis*: All 152 samples from the six crosses passed our quality control (Additional file 19: File S19). A total of 123,964 SNPs were recommended by the Best Practice Workflow (70.68% of the SNPs on the chip). Out of these, 101,376 SNPs were heterozygous in at least one parent in each family, allowing us to test ~ 50,000 SNPs per family (Additional file 26: Table S14), and 5249 SNPs across all families (Additional file 25: Fig. S4). After adjusting for multiple tests using the Holmes correction, 2047 SNPs failed the segregation test, which represents 2.03% of the tested SNPs (Additional file 19: File S19).

*Comparing technical replicates*: SNP calls were highly reproducible. The genotypic concordance within the four technical replicates was high (99.32%), while non-replicate samples shared just 52.74% of the genotypes (Table 3). The pairwise comparisons of the genotypes of each technical replicate using a custom code (Additional files 5, 26: File S5, Table S15) confirmed high genotypic concordance, with error estimates ranging from 0.33 to 1.02% (Table 3).

**Table 3:** Pairwise concordance analysis for technical replicates genotyped using the SNP chip

Technical replicate 1	Technical replicate 2	Concordance (%)
1a	1b	99.52
1a	1c	99.31
1b	1c	99.67
2a	2b	98.46
2a	2c	99.33
2b	2c	98.40
3a	3b	99.59
3a	3c	99.59
3b	3c	99.53
4a	4b	99.61
4a	4c	99.52
4b	4c	99.41
Mean		99.32

	Random samples	
1a	2b	52.33
3c	2a	50.86
1a	4a	54.43
3b	1a	53.33
Mean		52.74

Replicate	Mean error rate per replicate (%)
1	0.50
2	1.27
3	0.43
4	0.49
Mean	0.67

The first two columns list the two replicates. The third column shows the percent of times two replicates shared identical genotypes or the average error rate per replicate. “Random samples” refers to the percentage of times two randomly picked individuals share identical genotypes but are not technical replicates. The names of the genotyping files are described in Additional file 26: Table S5

*Comparison of WGS and chip genotypes*: The analysis of genotype data using either WGS or SNP chip methodologies across three distinct dataset sizes (labeled a, b, c for SNP chips and y, x, w for WGS) is shown in Additional file 25: Fig. S2, performing one genotype call for each dataset. Additional file 25: Fig. S5 shows that for both methods, the genotype error rate decreases as the sample size increases. Specifically, for the SNP chip datasets, error rates declined from 1.16% to 0.41% (Table 1). In contrast, error rates for the WGS datasets range from 1.35% (yx) to 3.09% (xw) (Table 1). Across both platforms, the SAI samples from Saint Augustine, Trinidad and Tobago, displayed slightly elevated error rates compared to other samples, ranging from 0.46% to 1.42% for SNP chips and 3.36% to 9.25% for WGS.

**Fig. 3:**
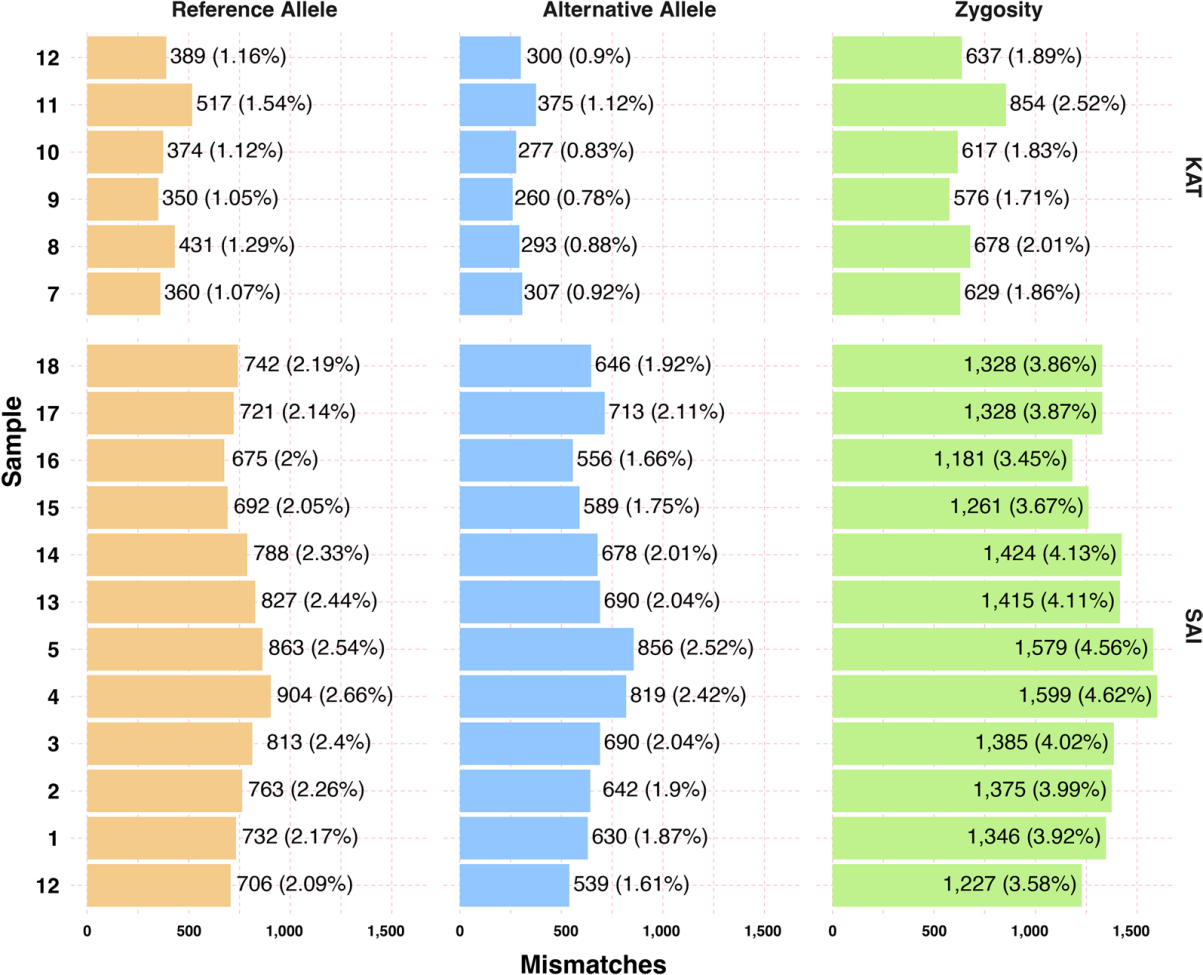
Pairwise comparison of genotype mismatch for 18 samples genotyped with the SNP chip or with WGS. The *y*-axis identifies each individual mosquito with a number from 1 to 12 for KAT and 1 to 18 for SAI; their population of origin is reported on the far right of the panels (KAT and SAI). The *x*-axis shows the number of SNPs with mismatches. The three different colored panels show the number of SNPs for which the reference allele did not match between the two datasets (orange panel), while the other two panels display the number of SNPs where the alternative allele (blue panel) or the zygosity (green panel) did not match. The actual numbers of mismatches (with percentages in parenthesis) are reported within each rectangle

The average mismatch rate was 8.11% between the SNP chip and WGS when analyzing the same 18 individuals (ay, Table 1). This rate decreased to 6.70% upon increasing the sample count in genotype calls (Table 1, Additional file 25: Figs. S6 and S7). The within-population comparisons showed that the error rate varied depending on the genetic make-up of each population and the sample size. For example, the mismatch rate for KAT decreased from 5.61 to 4.63% as we increased the sample size (ay and cw, Table 1). We observed a similar pattern for SAI; however, the mismatch rates were higher, decreasing from 9.35 to 7.73. Some samples showed error rates as high as 10.90% (Table 1, column ay, sample SAI 5). When we examined how many times a SNP genotype did not match between both technologies, we observed that most SNPs showed genotype errors only in a few samples (Table 2). For example, KAT, which had six samples, showed that up to 18.54% of the mismatches appear only once, while for SAI, up to 53.31% of the errors appear only once, indicating the randomness of most errors (Fig. 3).

Within each population subset, larger sample sizes correlate with more sites exhibiting mismatches within each population since most errors appear only in a few samples (Table 1). For instance, the SAI population, comprising 12 individuals, showed a higher mismatch rate of 1.42% compared to the KAT's 0.95%, which only contains six individuals (Additional file 25: Fig. S7). Comprehensive evaluations of larger datasets (labeled “bc” for chips and “xw” for WGS, as illustrated in Additional file 25: Fig. S2), highlight the mismatch rates of 0.41% for chips and 3.09% for WGS (Table 1). Independently of the comparison, when we look at the percentage of SNPs with errors in six samples, SAI consistently have higher percentages of mismatches, indicating the role of population genome architecture if there is genome size variation across the globe, or the potential influence of the DNA quality in the performance of both technologies. For KAT, the values vary from 0.03 to 1.25%, while for SAI it varies from 0.08 up to 4.12% (Table 2).

On a broader scale, the SNP chip genotype data stay consistently below an error rate of 1.30%, irrespective of the sample size in question. Meanwhile, the WGS data oscillate, exhibiting error rates between 1.35 and 3.09%, contingent on the dataset size. The average error rate rises to between 6.70 and 8.11% when cross-comparing genotype calls from the two platforms (Additional file 25: Fig. S5, Table 1). A pattern emerges where reduced read depths in WGS data correlate with heightened mismatch rates between methods (Additional file 25: Fig. S8). Array genotyping metrics like Fisher linear discriminant (FLD) and call rate (CR) correlate with mismatches and could be used to further filter out problematic probe sets on the array (Additional file 25: Figs. S9 and S10).

**Fig. 4:**
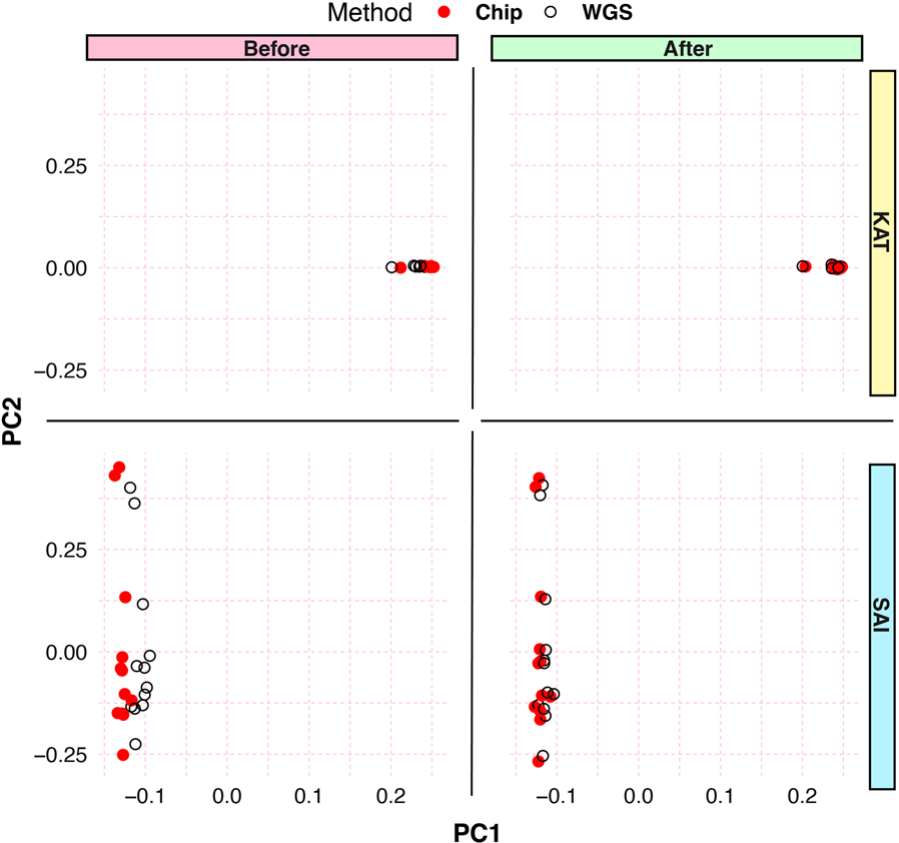
Principal component analyses using the same 18 samples from the KAT and SAI populations (far right) genotyped using WGS (black open circles dots) or the SNP chip (red dots) before (left) and after (right) filtering SNPs for FLD ≥ 6, CR ≥ 98.5%, and read count per site ≥ 20. The analyses were done using PCA from Plink and plotted with ggplot2

PCA was conducted to compare the WGS and SNP chip data for the 18-sample dataset, post-SNP filtration, based on criteria including FLD, CR, and a read depth greater than 20. The latter factors were deduced to correlate with mismatch rates (Fig. 4, Additional file 25: Figs. S9 and S10). The PCA displays a significant overlap between WGS and chip samples (Fig. 4). Therefore, while sample size exerts minimal influence on error rates at a sequencing depth of about 20×, depth combined with chip-specific metrics (FLD and CR) significantly dictates genotype consistency.

*Variant functional annotation and SNP density*: We observed the lowest number of errors in the AalbF3 annotation using canonical or non-canonical models when we performed the variant annotation using the 92,693 SNPs that genotyped well on the wild samples (Additional file 26: Tables S3 and S16) for the chip data and the ~ 2.7 million SNPs for the WGS data used in the chip design.

We confirmed that the 404,514 probes used in the chip target polymorphic sites in 17,461 protein coding genes and other genes (Additional file 26: Table S17), with 915 SNPs in genes associated with diapause or immunity (Additional file 26: Table S18). Lists of SNPs found on diapause and immunity genes are also provided (Additional file 26: Tables S19 and S20).

In the variant functional annotation, we categorized the genetic variants into six types: intron, intergenic, 5′ untranslated region (5′ UTR), 3′ UTR, and coding exons (including both synonymous and non-synonymous). The chip bias is especially pronounced in the overrepresentation of variants in functionally significant regions like coding exons (synonymous and non-synonymous) and UTRs. To correct for this, we turned to WGS data as a gold standard for unbiased variant representation. We performed a comprehensive functional annotation on both the WGS and SNP chip datasets to deepen our understanding of the variant landscape. This allowed us to categorize each SNP based on its genomic position or functional annotation category as described above. We then compared the proportions of SNPs across these categories between the two datasets.

Regarding intronic and intergenic variants, the chip generally underperforms WGS, as evidenced by negative bias percentages of −5.32% and −12.47%, respectively. However, the chip outperforms WGS in detecting the targeted synonymous, 5′ UTR, and 3′ UTR variants, with positive bias percentages of 9.75%, 3.53%, and 3.72%, respectively (Table 4), when comparing SNPs in common between the two technologies, as WGS captures millions of variants that we could not use in this comparison. The difference is marginal for non-synonymous variants, indicated by a minimal positive bias of 0.79%. The data suggest that the chip may be more sensitive to specific SNP functional annotations, while WGS provides a more balanced detection across all categories. Given this asymmetry, we calculated the proportion of SNPs from the chip that we could use to replicate the proportions observed with the WGS data and found that using ~ 65% (39,591 out of 61,749 SNPs) of the SNPs reflects the same variant distribution as the WGS data (Table 4).

**Table 4:** Variant functional annotation of SNPs using SnpEff for WGS and SNP chip data, with results of bias correction

Variant type	WGS (*N*)	WGS (%)	Chip (*N*)	Chip (%)	Chip bias (%)	Chip possible (*N*)	Chip corrected (%)
Intron	1,281,482	44.81	24,387	39.49	−5.32	17,741	44.81
Intergenic	993,796	34.75	13,758	22.28	−12.47	13,758	34.75
Synonymous	384,482	13.45	14,323	23.20	9.75	5325	13.45
Non-synonymous	67,217	2.35	1942	3.14	0.79	930	2.35
5′ UTR	67,744	2.37	3642	5.90	3.53	938	2.37
3′ UTR	64,912	2.27	3697	5.99	3.72	899	2.27
Total	2,859,633	100.00	61,749	100.00	0.00	39,591	100.00

The number of SNPs and their percentage in each category for the two methods are listed in columns 2 to 5. Column 6 shows the number of SNPs used to match the proportions in the WGS data (chip possible). The final column shows the new percentage for each category of the chip data after bias correction (chip corrected)

Initially, the SNP chip included ~ 102 SNPs per 1 Mb window across the *Ae. albopictus* genome with SNPs on 549 of the 574 scaffolds (no sites were found in the remaining 25 small scaffolds) from the AalbF3 assembly (Additional file 26: Table S7). Once we genotyped the wild samples and performed quality control, the SNPs dataset was reduced to 82,731 SNPs, averaging 57 SNPs per 1 Mb window (Additional file 26: Table S21), with a relatively even distribution across the genome (Additional file 25: Fig. S12).

### Quality control for wild samples

A summary of the analysis produced by the Axiom Analysis Suite software with all the thresholds used for quality control and genotyping is included in Additional file 20: File S20. A total of 243 samples passed quality control with 115,346 SNPs “recommended” by the “Best Practices Workflow,” representing 65.76% of all the variants in the chip. From this set, Plink removed 9725 SNPs missing in more than 20% of the individuals and 11,314 SNPs with a minor allele frequency less than 10%. All SNPs passed the Hardy-Weinberg Equilibrium (HWE) test. We removed two mosquitoes because their mean heterozygosity deviated from the overall mean heterozygosity by a standard deviation (SD) greater than 4, and two other mosquitoes because of high relatedness (> 0.354, Additional file 7: File S7). Thus, 237 samples passed the quality control test and were used in subsequent analyses.

Twenty-three samples did not pass the DQC threshold of 0.82. Twelve samples, all from Ho Chi Minh (HOC), Vietnam, failed DQC, indicating possible species misidentification prior to DNA extraction, which was confirmed by PCR, as they failed to amplify using the *Ae. albopictus* cytochrome oxidase I-specific primers (Additional files 17, 26: File S17, Table S6). These samples were thus not included in Table 2 and subsequent analyses. Additional samples from Ho Chi Minh from a different collection time, confirmed to be *Ae. albopictus*, were subsequently genotyped (Additional file 26: Table S3) and added to the dataset used for population genomic analyses. All the other samples from this collection were confirmed to be *Ae. albopictus* by PCR.

**Fig. 5:**
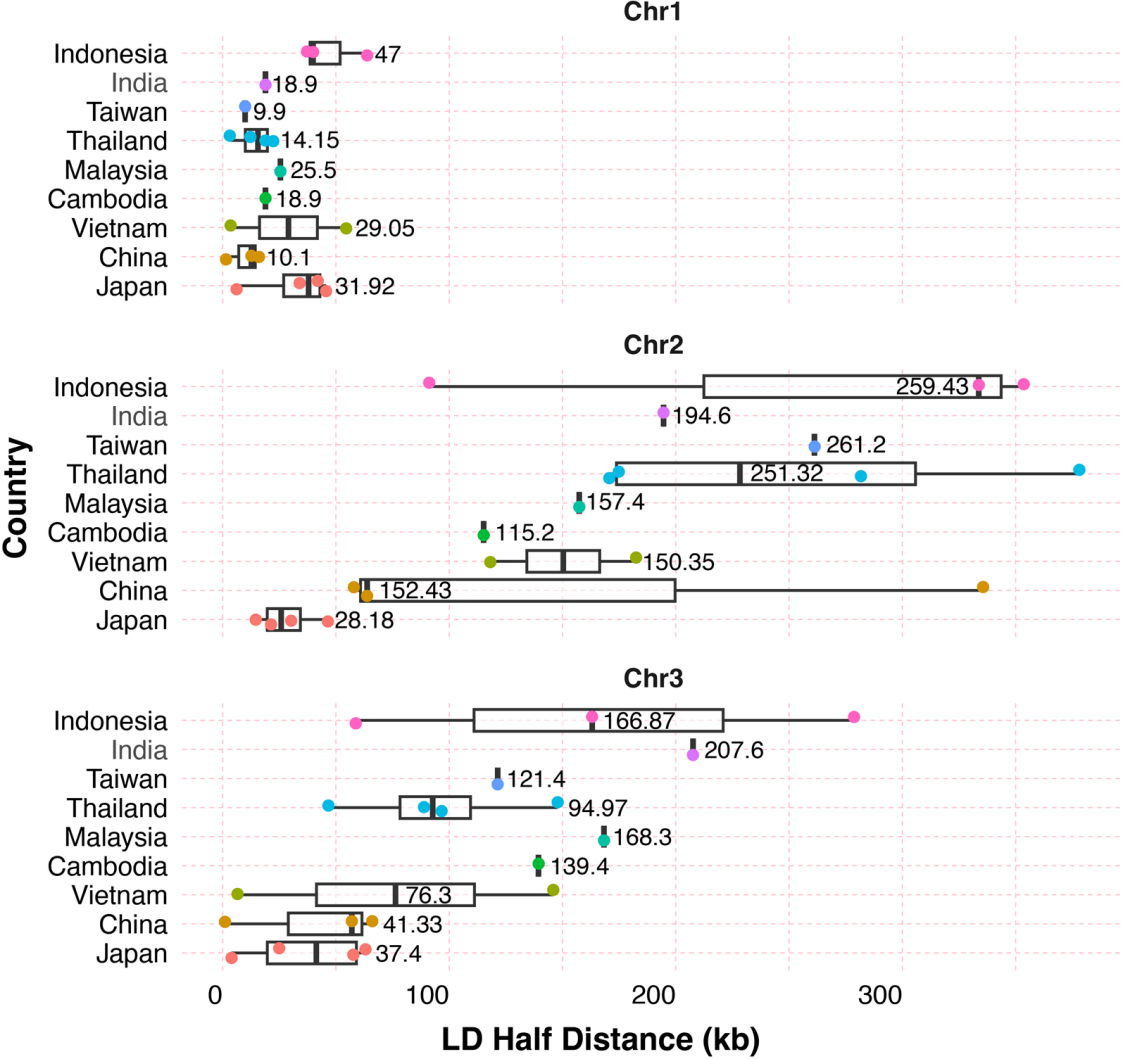
The linkage disequilibrium (LD) half-distance values in kilobases (kb) for the *Ae. albopictus* mosquitoes across Asian countries, grouped by chromosome (Chr1, Chr2, and Chr3). Each boxplot displays the interquartile range of the LD half-distance values, *r*^2^, with the vertical black line in the box marking the median. Colored points represent individual data points, with each color corresponding to a country. The number on each line indicates the *r*^2^ for each country–chromosome combination. Comparisons of values among countries are not appropriate given the different sampling intensity and country sizes. Additional file 25: Fig. S13 shows values for all populations

*Linkage disequilibrium analysis*: We compared the half distance of maximum *r*^2^ value for populations with at least six mosquitoes (Additional file 25: Fig. S13, Additional file 26: Tables S9 and S22) after estimating LD decay with the package PopLDdecay [37]. The LD half-life estimation of *Ae. albopictus* within its native range reveals varying degrees of LD across different chromosomes. Chromosomes 1 and 3 show a rapid decay in LD compared to chromosome 2, highlighting differences in their evolutionary histories or recombination events (Fig. 5) or potential misplacement of scaffolds in the genome assembly. We also observed a correlation between the sample size and the LD estimates (Additional file 26: Fig. S14). For chromosome 2, the correlation coefficient (*R*^2^) was 0.64 with a negative slope, indicating that the LD half-life estimates decreased as the sample size increased. The slope was also negative for the other two chromosomes, but the *R*^2^ was 0.23 and 0.11 for chromosomes 1 and 3, respectively. When we summarize the LD decay by country, it becomes more evident that there is rapid decay for chromosome 1 with relatively low variability between populations from the same country. The LD estimates for chromosomes 2 and 3 exhibit greater variability (Fig. 5). Due to the limited sampling size, it is not possible to draw any patterns for comparisons among the countries or geographical regions.

### Genetic ancestry, population structure, and differentiation among native populations

**Fig. 6:**
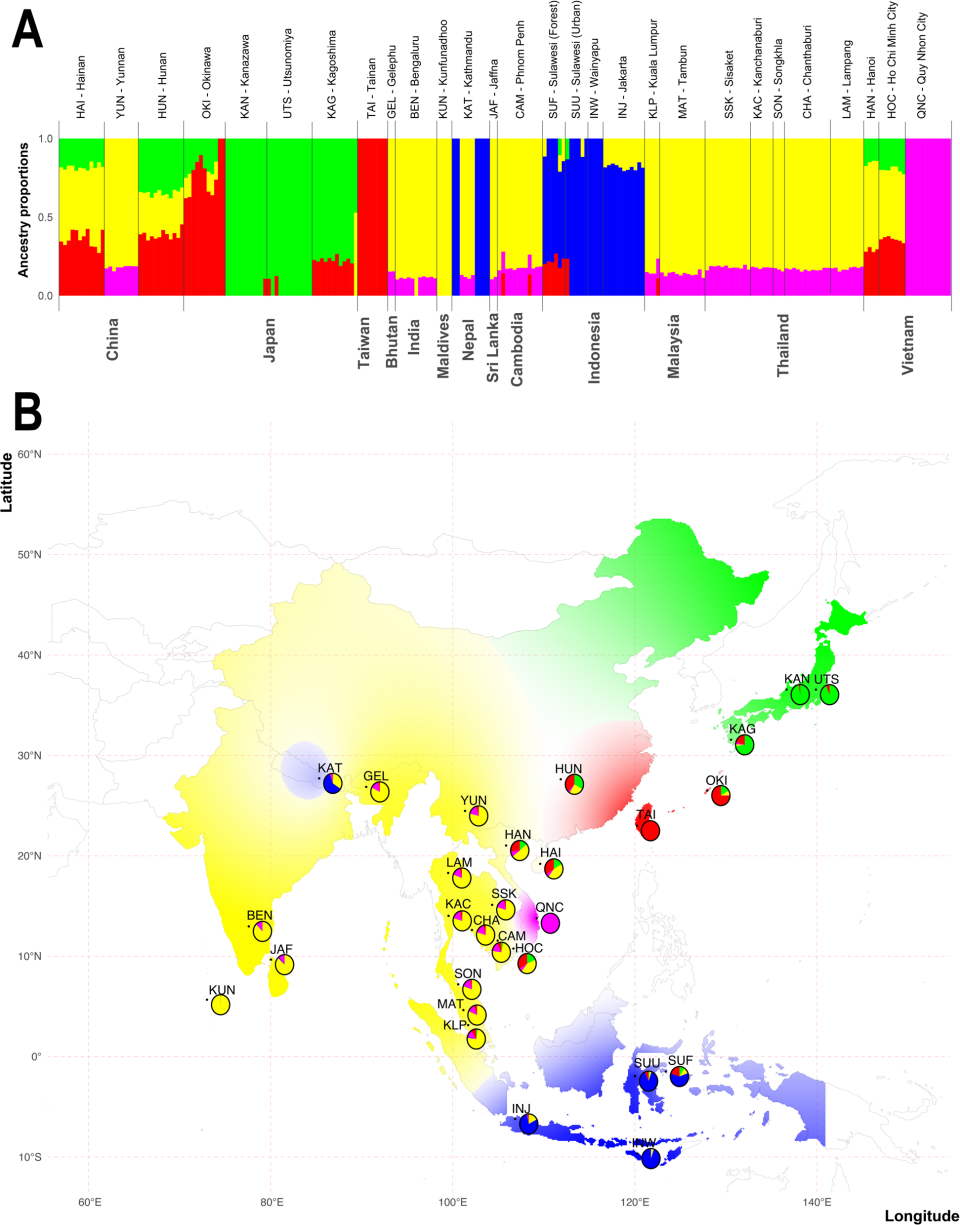
Population structure of *Ae. albopictus* in the native range using 57,780 SNPs (set LD2). Panel A shows the results of Admixture analyses on 237 samples for *K* = 5. Each bar represents a mosquito. The country of origin is listed on the bottom, while the population code and the name of the sampling locality are on the top. Each bar color and height represent the probability of the mosquito being assigned to an ancestral group. On the *y*-axis are the admixture proportions for each individual. Panel B shows a map where the ancestry matrix is interpolated over the entire region from which samples with different shades of the same color reflect different ancestry coefficients. The map was obtained using the R package tess3r. The colored pies at each sampling site reflect the proportion of the clusters found at that site. Plots for the other SNP sets and other algorithms are shown in Additional file 12: File S12, and Additional file 25: Figs. S15 and S16

All runs of Admixture with each of the three SNP sets (intergenic, LD1, or LD2) indicated a *K* value of 5 as the number of ancestral clusters (Figs. 6, Additional file 26: Figs. S16 and S17). The genetic cluster with the green color primarily covers Japan, indicating a prevalent genetic component in this region. The red cluster is predominantly found in Taiwan and Okinawa Island in southern Japan, with some genetic admixture detected in East China (HUN and HAN populations). The blue cluster is mainly concentrated in Indonesia but also observed in Nepal (KAT). However, it is important to note that some of the KAT mosquitoes were from a laboratory strain, which may have influenced the genetic composition. The magenta cluster is most notable in the central region of Vietnam, particularly in Quang Nam Province (QNC). The yellow cluster has the widest distribution, spanning across north Malaysia, the northern and southern regions of Vietnam, Sri Lanka, Cambodia, Thailand, the Maldives, Bhutan, India, and western China. Two of the other algorithms (fastStructure and LEA) suggested up to nine ancestral populations (Additional files 9, 10: Files S9 and S10). The differences were mostly due to some of the island samples (Taiwan and Okinawa) forming their own clusters using these methods rather than being included in other clusters.

Because of the consistency of the Admixture results with the three SNP datasets, we chose *K* = 5 to compare all the algorithms by comparing the structure plots directly (Additional file 25: Fig. S15) and by interpolating the Q matrices over the range of the sampling localities (Fig. 6 and Additional file 25: Fig. S16).

We trained Neural Admixture with nine populations representing the five genetic clusters using the three datasets (intergenic, LD1, and LD2). After the training, we plotted the Q matrix for *K* = 5 for each SNP set and observed that when using the intergenic SNP set, Neural Admixture correctly assigned the populations into each known genetic cluster and detected the known admixture for the OKI population (Additional file 25: Fig. S15). For the LD1 SNP set, which followed the authors’ recommendation for linkage pruning with *r*^2^ of 0.01, Neural Admixture correctly identified the five genetic clusters, but did not identify the admixture in the OKI population during training; however, it did identify admixture when performing the inference with all populations (Additional file 25: Figs. S16 and S17). For the LD2 SNP set, for which linkage pruning did not follow the authors recommendations with *r*^2^ of 0.1, Neural Admixture did not identify the five genetic clusters after the other three SNP data populations and inferred the ancestry of the 28 populations (Additional file 25: Figs. S15–S17).

The advantage of using the Neural Admixture is its speed relative to the other programs. It took only seconds for training and inference using two central processing units (CPUs) and graphical processing units (GPUs) on the HPC cluster, while for the other methods, running times were longer (hours to days), using approximately 300 CPUs. For example, FastStructure using the logistic prior took the longest time (up to 8 days).

**Fig. 7:**
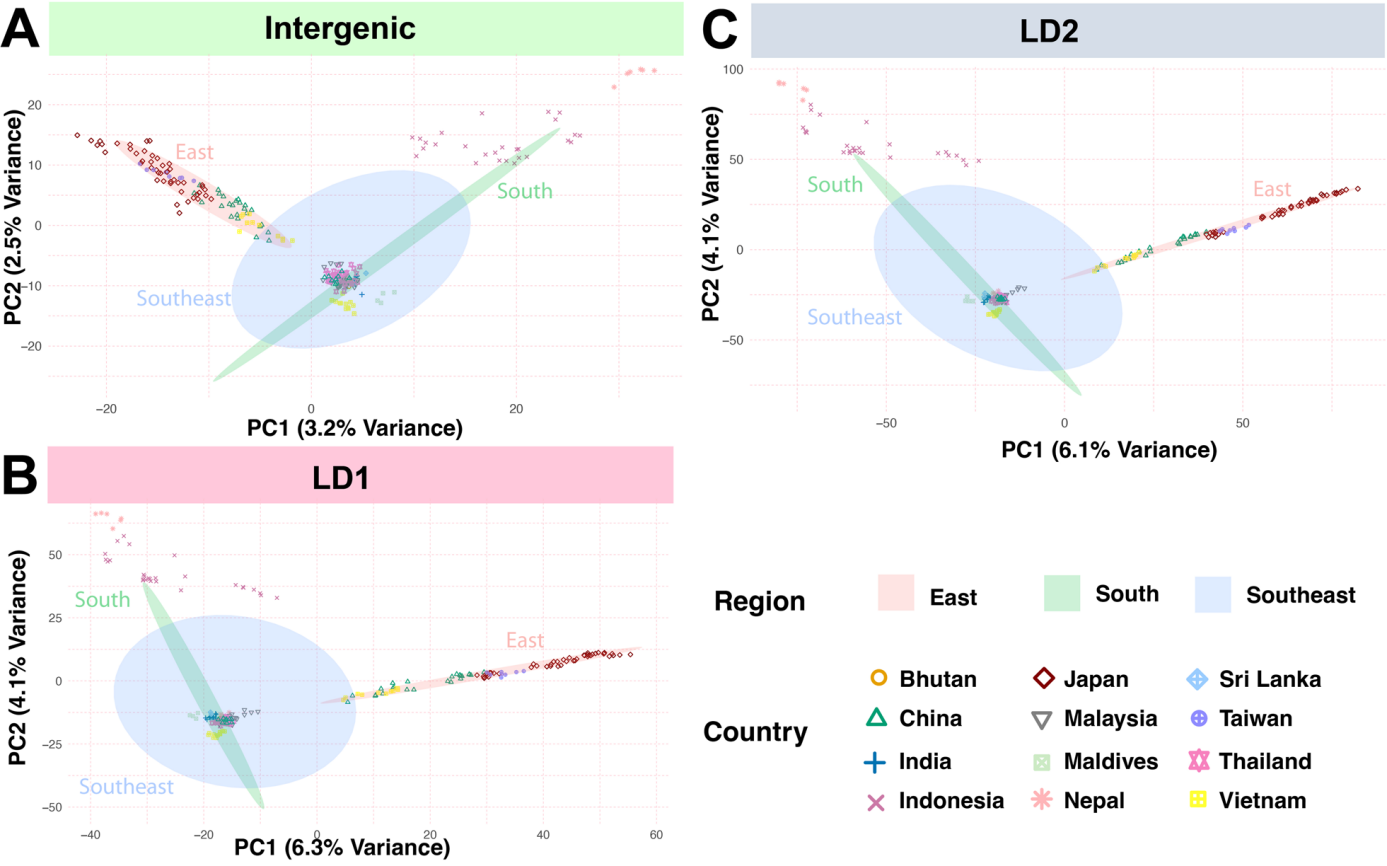
Principal component analysis for 237 samples from Asia for three SNP datasets (**A**: intergenic SNPs, **B**: LD1, and **C**: LD2). The analyses were carried out using LEA. For each PCA, the *x*- and *y*-axis refer to the results of the first two principal components (PC1 and PC2, respectively), with each percent variance reported in parentheses. The dots identify the samples. The color of the dots refers to the country of origin (see legend on bottom right). The ellipses on each panel mark each geographical region in Asia, covering 80% of the samples. The colors of the ellipses identify the region (see legend on bottom right)

To evaluate the robustness of the grouping retrieved from the above analyses, we also ran PCA and clustering analyses using the three datasets (intergenic, LD1, and LD2). Although PCA analyses did not show different clustering among datasets (Fig. 7), the variance of the SNP set with the intergenic SNPs was lower than the variance found using the two sets with different *r*^2^.

The PCAs using the same three SNP datasets identify three major clusters rather than five retrieved by the admixture analyses when we used Plink (Fig. 7), which might be due the use of two principal component axes. However, the clusters in the PCA space followed similar patterns from the admixture analysis. Some samples from Nepal clustered with the samples from Indonesia (Fig. 6). The samples from East Asia (Additional file 26: Table S3, Fig. 2) clustered along PC1, with samples from mainland Japan clustered at the right (LD1 and LD2), followed by samples from Taiwan, and then the samples from the Japanese island Okinawa. Next, the Chinese samples clustered with the Vietnamese samples at the left of the East Asia cluster. The admixture analysis indicated that samples from mainland Japan belong to a different ancestral group than those from Okinawa (Fig. 6), while samples from Taiwan clustered with Okinawa along with samples from East China. Therefore, although the East Asia samples clustered along the PC1, they followed the clustering patterns observed from the admixture analyses for the clusters “green” and “red” in Fig. 6.

**Fig. 8:**
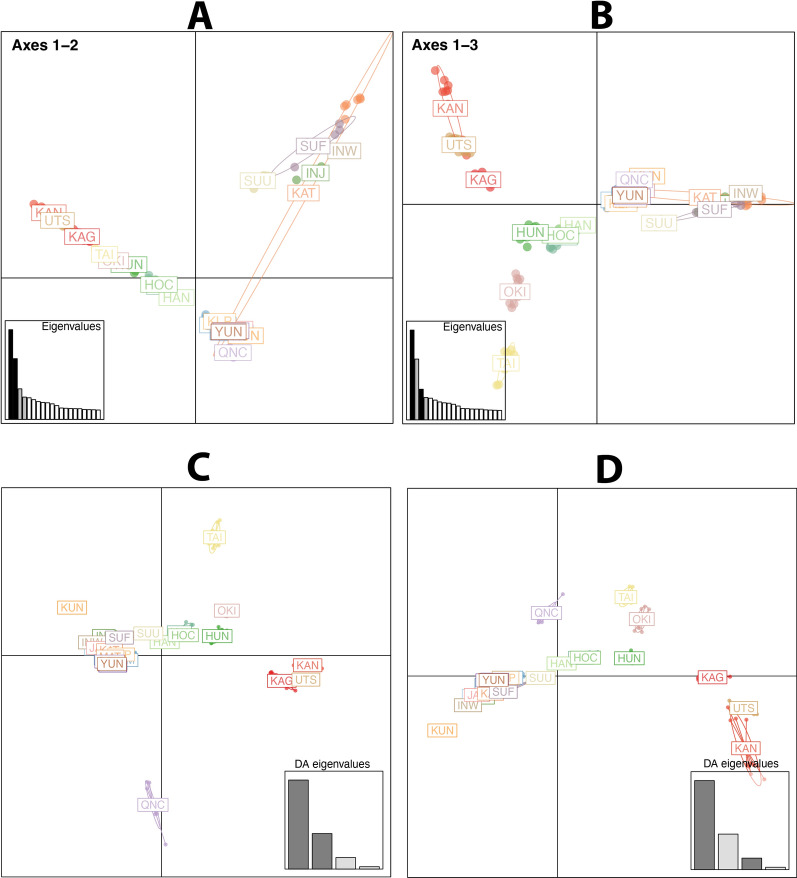
Comparative discriminant analysis of principal components (DAPC) across multiple populations. **A** The scatter plot illustrates genetic differentiation among populations along the first two principal axes (axes 1 and 2). Each population is represented by a unique color and is plotted based on its discriminant scores, which reflect the genetic distances and relationships between populations. The connected lines suggest a genetic trajectory or gradient among some of the populations. **B** Genetic variance among populations along the first and third principal axes (axes 1 and 3), offering a different perspective of genetic differentiation that might capture variance not evident in A. In both panels, the inset bar plots display the eigenvalues associated with each discriminant axis, indicating the proportion of genetic variance captured by each axis. Larger eigenvalues correspond to axes that explain a greater amount of genetic differentiation among populations. **C** and **D** Biplot of the principal components derived from a DAPC, highlighting the genetic differentiation among populations. Each point represents the genetic profile of a population, plotted according to its scores on the discriminant functions. The scatter plot's axes are scaled to the eigenvalues of the discriminant functions, as shown in the inset bar graph, which represents the relative contribution of each function to the total genetic variance observed

The PCA results using the R package adegenet correctly identified the five genetic clusters (Fig. 8). The clustering using axes 1 and 3 aligned with the Admixture analysis but failed to separate the QNC population. However, scatter plots of the discriminant functions provided a clear delineation among the predefined population groups (Fig. 8). These discriminant functions are particularly tailored to accentuate the genetic differences between groups and have successfully captured the expected population structure. Given that PCA maximizes total variance without regard to group labels, the overlapping clusters observed in the PCA plots could reflect a more continuous genetic variation across populations or the presence of shared genetic polymorphisms that PCA is sensitive to but are not informative for group differentiation.

We estimated the genetic differentiation (F_ST_) using the three SNP sets and fitted a linear regression to our estimates. As expected, the F_ST_ estimates for the intergenic SNP set were lower, but the overall patterns were similar for the LD1 and LD2 SNP sets: all three datasets had positive slopes (Additional file 25: Fig. S18). Next, after estimating the geographical distance between the sampling sites (km), we fitted a linear regression for estimates by country with at least three sampling localities [*lm* = *F*_ST_/(1 – *F*_ST_) ~ log(distance)] (Additional file 25: Fig. S19). The correlation coefficient (*R*^2^) was higher for China and Thailand, 1 and 0.38, respectively, indicating isolation by distance. However, we have a small number of sampling localities. When we used all 28 populations, the *R*^2^ was 0.00.

We created a matrix with the F_ST_ value (upper) and the geographical distance in kilometers (lower) for all sampling sites, sorting the matrix by distance, and observed that the F_ST_ values were higher as the distance between the sampling sites increased (Additional file 26: Table S23). Next, we used the R package adegenet [46, 47] to evaluate isolation by distance using populations with at least four mosquitoes (Additional file 26: Table S3) using the LD2 SNP set. The Mantel test indicated a correlation of 0.20, which suggests no deviation from the random expectation (*P* < 0.053) (Additional file 25: Fig. S20). The coefficient of correlation (*R*^2^) value of 0.05 when we fitted a linear regression model indicated that approximately 5% of the variation in genetic distance can be explained by geographical distance (Additional file 25: Fig. S20). The positive slope of the regression line supports the isolation-by-distance hypothesis, with greater geographical distances correlating with greater genetic distances at the native range of *Ae. albopictus*. However, a small proportion of the genetic distance is explained by geographical distance.

Among the five genetic groups, the F_ST_ values are higher for the “blue,” “red,” and “pink” clusters, while the admixture proportions are lower (Additional file 25: Fig. S21 and Fig. 6). The “blue” cluster is in South Asia, covering Indonesia, while the “red” cluster is mainly in Taiwan, Okinawa Island, and East China. The “pink” cluster is mainly in Vietnam, specifically in Qui N hon City (QNC), but populations from South Asia show admixture with this ancestral group. The “yellow” cluster shows the highest admixture and lowest F_ST_ values, and it covers South and Southeast Asia (Figs. 2, 6, and Additional file 25: S21).

The three Japanese populations above latitude 30° N are more genetically differentiated than those below latitude 30° N (average F_ST_ = 0.12 vs. F_ST_ = 0.09); interestingly, these populations are known to undergo photoperiodic diapause, while the others do not (Additional file 26: Table S24). The F_ST_ estimates for samples from South Asia (India, Maldives, Malaysia, and Nepal) were the highest (average F_ST_  = 0.12 for the region), followed by the samples from Southeast and East Asia.

To evaluate the chip bias effect, we used six SNP dataset sets. Three (C10, C3, and C5) were corrected by sampling the SNPs based on their functional category and replicating the proportions observed in the WGS data (Additional file 26: Table S8). The other three SNP datasets (U2, U8, and U9) included the same number of randomly sampled SNPs. The main difference between these sets of SNPs were the number of shared SNPs: the three SNP sets with corrected bias (the C set) shared 48.02% of the SNPs, whereas the SNP sets without bias correction (the U set) shared only 8.35% of the SNPs (Additional file 25: Fig. S22). The sNMF-LEA analyses with corrected bias (the C set) generated the same overall patterns observed with the other three SNPs sets (intergenic, LD1, and LD2, Additional file 25: Figs. S16 and S17). However, for the SNP sets without bias correction (the U set), the clustering of QNC and TAI in two runs was different, with their placement being swapped (Additional file 25: Figs. S23 and S24). Therefore, the correction of bias improved the performance of LEA in detecting the five genetic clusters correctly without any population being misidentified.

The clustering of the samples on the PCA space was very similar for all datasets using only the first two principal components, but the SNP sets with bias correction were more uniform since the variance explained by PC1 and PC2 was lower (Additional file 25: Fig. S25). For the SNP sets without bias correction (the U set), the PC1 variance was approximately 10% higher than the bias-corrected set (the C set). Additionally, the direction of the clusters in the PCA was not uniform for the sets without bias correction (Additional file 25: Fig. S25).

## Discussion

### Chip design

*SNP discovery and selection*: Using WGS SNP data, we designed an Aealbo chip which contains 175,396 SNPs; these SNPs were selected from the 2.7 million SNPs distributed across the AalbF3 genome assembly, which were screened for probe design suitability. We confirmed that the probes on our chip perform as expected by using tests for Mendelian segregation, extensive comparisons with WGS data, and prediction algorithms to calculate the p-convert metric. This metric estimates the probability of successful SNP genotyping by providing a confidence level for each genotype call associated with SNPs [51, 52]. Generally, if a SNP has a higher p-convert score, it is more likely to be correctly genotyped, while a lower score indicates potential inaccuracies in genotyping. The mean p-convert value for the probes in the Aealbo chip is 0.71 (Additional file 18: File S18), which is relatively high compared to other chips [6, 53].

Our analysis revealed that high-quality genotype calls were obtained for approximately 70% of the markers on the SNP chip, as indicated by the expected fluorescence signals (Additional files 19, 20: Files S19 and S20). Possible reasons for not having a larger percentage of SNPs performing well could be that the current genome assembly may be incomplete, and/or that we did not capture enough genetic variation in the species, or that there exists genome size variation [54, 55] or structural variations. Since the mapping of the probes in the different genome assemblies revealed problems in the first assemblies (Additional file 26: Table S12), the problem potentially lies in the current genome assembly, which is still underperforming and missing genomic regions that the probes are binding to, making them suitable for genotyping. Improvements on the quality of the species’ genome assembly should increase the percentage of SNPs performing well.

Our selection process for SNPs in probe design utilized a minor allele frequency (MAF) threshold of 10%, yet this threshold did not preclude us from obtaining SNPs with lower frequencies (< 10%). For instance, applying this MAF threshold in our current dataset led to excluding 11,314 SNPs from the initial set of 104,139. This refinement underscores the ability of the chip in capturing a comprehensive genetic snapshot while focusing on alleles with substantial representation in the population.

*Mapping probe sequences to genome assemblies*: Despite limitations due to the currently available genome assembly for *Ae. albopictus*, the mapping of the probes to the available assemblies suggests that they progressively improved, as the number of probes with unique alignments increased from older to newer assemblies (Additional file 26: Tables S12 and S13). Mapping the probe sequences to previous genome assemblies revealed duplication problems in these previous assemblies, with only 57% of the probes (~ 100,000) mapping uniquely to the AalbF2 assembly, while 99% mapped uniquely to the AalbF3 one (Additional file 26: Tables S12 and S13).

### Chip validation

*Segregation analyses*: The segregation analysis using six laboratory crosses indicated relatively few segregation errors: of the 101,376 SNPs examined, only 2.03% (2,047 SNPs) did not pass the criteria (*P* < 0.05) (Additional file 21: File S21). Segregation errors in SNPs can arise due to a variety of factors, such as procedural errors during the genotyping process, challenges in accurately measuring fluorescence signals from the probes, unintended hybridization of probes with non-specific DNA fragments, and compromised DNA sample quality. The errors detected could be due to rare genetic variants, such as unique point mutations or copy number variations, which influence the intensity readings of SNP assays, and/or to the presence of alleles that occur infrequently in the population. Lastly, unaccounted underlying population structures in family-based studies, such as gene and genome duplication events, could be responsible for some of the segregation errors, as well as variations in genome size in *Ae. albopictus* [54, 55] among the native and invasive populations, and therefore an incomplete genome assembly. If there is variation in genome size among the laboratory colonies, especially between the native and invasive crosses, or at the individual level, the segregation errors would vary among the families as we observed.

*Technical replicates*: The technical replicates showed high concordance, with an average of 99.32% using Axiom Analysis Suite or 99.46% using custom scripts (Additional file 5: File S5, Table 3), which is similar for other SNP chips [6, 53]. Several factors can produce small differences among replicates, including DNA sample quality, chip manufacturing inconsistencies, hybridization conditions, software difficulties in interpreting raw signals of different intensities, especially when signal levels are low, and the possibility of potential errors during DNA sample handling, processing, or normalization. Nonetheless, the high concordance found among replicates indicates the chip’s suitability for consistently genotyping *Ae. albopictus* samples.

*Comparison between WGS and SNP chip genotype calls*: Comparison of the two genotyping methods revealed that, while results from WGS and our chip were similar, read depth for the WGS data, sample size, and chip filtering thresholds influenced mismatch rates. We had three different sized datasets for each method (Additional file 25: Fig. S2).

In both chip and WGS genotyping methods, the error rate in genotypes increased as more samples were included in the study. For example, among 18 individuals genotyped with the chip, the 12 SAI samples from Saint Augustine (Trinidad and Tobago) had a higher mismatch rate of 1.42%. This was greater than the 0.95% mismatch rate in the six KAT samples from Kathmandu, Nepal (Tables 1 and 2). While the higher error rate observed in SAI samples could be influenced by factors such as their invasive range and island origin, potentially leading to allele loss due to drift, we must also consider the limited sample size and the likelihood that differences in DNA quality, possibly resulting from shipping issues, played a significant role.

Error rates for SNP chip data remained consistently under 1.3% regardless of the size of the datasets. In contrast, the error rates for WGS datasets fluctuated, as depicted in Additional file 25: Fig. S5. A notable negative relationship exists between the site read depth and genotype errors. Specifically, as the read depth decreases, mismatch rates rise. This suggests, not unexpectedly, that WGS data are more unreliable in identifying heterozygotes as read depth decreases.

In the case of SNP chip data, metrics such as the FLD and the CR have significant effects on these rates, as illustrated in Additional file 25: Figs. S8, S9, and S10. Importantly, after filtering out SNPs from both datasets based on the identified important metrics (FLD > 6, CR > 98.5, and read depth > 20), the multivariate analysis results, specifically PCA, for both the WGS and chip data from the 18-sample set were strikingly alike (Fig. 4).

### Variant functional annotation and SNP density

A primary challenge in identifying gene-specific SNPs was the quality of the existing genome assembly and annotation. To obtain the current genome assembly (AalbF3), the AalbF2 was deduplicated, removing redundant sequences originating from duplicated regions, repeats, or sequencing errors [23]. While the gene annotations lifted from AalbF2 to AalbF3 had the most negligible errors (Additional file 26: Table S16), deduplication in AalbF3 introduced challenges such as orphan exons and some transcripts missing start or stop codons. Despite these problems, we identified 915 SNPs in immunity- and diapause-related genes (Additional file 26: Table S18) that performed robustly in the test samples (Additional file 26: Tables S17–S20).

Having an improved genome assembly could improve the number of SNPs retained for downstream analyses, allowing us to maintain the density of SNPs we estimated at the initial chip design. The chip’s SNP density dropped from an initial 102 SNPs per 1 Mb window to 57 SNPs per 1 Mb post-quality control (Additional file 26: Table S21). This drop in the density is due mainly to the fact that only 70% of the probes generate the expected fluorescence signals.

As we had an overrepresentation of variants in coding regions on the SNP chip compared to the WGS dataset (Additional file 25: Fig. S11), we tested our ability to correct for this bias using SNPs from the chip data that followed the same functional proportions as those in the WGS data by comparing clustering and PCA results between “corrected” and “uncorrected” chip datasets (Table 4). Ancestry analyses using sNMF-LEA consistently produced similar results for both datasets (Additional file 25: Figs. S23 and S24), and similar clustering of groups were obtained from multivariate methods using PCA (Additional file 25: Fig. S25). The observed differences between the datasets likely stem from the uncorrected chip sets sharing fewer SNPs (8.35%), due primarily to the few intergenic SNPs on the chip. In contrast, the corrected chip sets share a much higher percentage of SNPs (48.02%) (Additional file 25: Fig. S22). This significant difference in shared SNP percentages means that most SNPs in the uncorrected sets come from varying genomic regions. In contrast, nearly half of the SNPs in the corrected sets represent the same genomic areas. Such variations in SNP distribution and overlap could influence the analysis outcomes.

### Analyses of wild samples

#### Time efficiency

Genotyping with the Aealbo chip was much faster than obtaining WGS data. While the WGS analysis for the 819 samples we analyzed required weeks of processing on the Yale HPC cluster, the genotype calls for the chip were done on a desktop computer, and results in “vcf” or “bed” format were generated within only a few hours. This relatively short computing time included the time it took to run several quality control measures. We ran the off-target variant tool from Axiom Analysis Suite to flag and remove any loci for which the clustering with other samples differed from the predicted fluorescence signal. Additionally, we were able to adjust for genetic variation that we did not account for during the chip design by using the SSTool (available for the Axiom Suite software). This allowed us to adjust the default library prior, which was built by the Thermo Fisher bioinformatics team with data from just the first five genotyped plates. We also only analyzed the SNPs flagged by the Axiom Suite pipeline as "recommended,” which were SNPs passing all the default quality control steps. Overall, these methods enabled us to quickly obtain quality-controlled genotype calls for subsequent analyses.

#### Linkage disequilibrium

We constructed a chromosomal-level assembly by integrating the 574 scaffolds based on the sequence order from the AalbF3 genome assembly (Additional file 8: File S8). It allowed us to assess linkage decay for each chromosome. LD half-distance estimates exhibited variability across chromosomes. Specifically, chromosome 2 displayed a distance extending up to 378 kilobases (kb). In contrast, chromosome 1 had consistently lower estimates across populations. Chromosome 3’s LD half-distance estimates were intermediate between chromosomes 1 and 2 (Fig. 5 and Additional files 25, 26: Fig. S13, Table S22). This variation in LD half-life across chromosomes can provide insights into the evolutionary dynamics, selection pressures, and historical recombination events unique to each chromosome. Differences in LD half-life across chromosomes can arise from various evolutionary processes such as selective sweeps, variation in recombination rates, and/or recent admixture. Low rates of recombination may be due to cryptic structural variants, such as inversions. Age of populations may also be involved since older populations have more time for recombination to randomly mix genomes. These factors could explain the low levels of LD in populations from Japan (Fig. 5 and Additional file 25: Fig. S13). These populations are likely old, being from the ancestral range, as well as having low admixture (Fig. 6), possibly due to limited gene flow among northern and southern populations, which differ in their propensity for photoperiodic diapause. Overall, several combined evolutionary forces, acting distinctly on each chromosome, can lead to the observed variations in LD half-life that we observed (Fig. 5).

When looking at LD half-distance data in different populations across Asia, we found variation among chromosomes (Additional files 25, 26: Fig. S13, Table S22) as well as variation due to sample size (Additional file 25: Fig. S14). Populations from China display a broad range of LD, with Yunnan having a high LD half-distance of 335.6 kb for Chr2, which contrasts sharply with the smaller values in other Chinese sites, such as Hainan (Chr2: 63.8 kb). Samples from Japan consistently show lower LD half-distances across all chromosomes, compared to samples from other regions (Additional file 26: Table S22), suggesting that different evolutionary histories or recombination events may be at play, including the occurrence of a sharp adaptive divide between northern diapausing and southern non-diapausing populations (see above).

#### Chip use with wild samples

We assessed the impact of bias in the chip due to the higher number of SNPs in coding regions (Table 4). To examine this, we used PCA and ancestral analysis with LEA. Neither test showed significant differences in clustering patterns (Additional file 25: Figs. S23 and S24). The primary variation involved the position of one ancestral group, either in Vietnam (QNC) or Taiwan (TAI), which could be due to the different sets of SNPs used for the bias-corrected (set C) and the bias-uncorrected (set U) analysis. For the U set, the number of shared SNPs was below 10%, while the C set shared near 50% (Additional file 25: Fig. S22).

*Genetic ancestry, population structure, and differentiation*: The SNP chip data, which included individuals from 28 sampling sites, allowed us to explore the genetic ancestry and the amount and patterns of differentiation across the native range of *Ae. albopictus*, from Southern Asia, incorporating India, through tropical Eastern Asia, including Indonesia, and northwards to East China and Japan [16].

Samples from the native range of *Ae. albopictus* were grouped into five major genetic ancestral groups (Fig. 6), although the exact number of genetic clusters varied depending on the algorithm used. Island samples were most sensitive to clustering algorithms.

To evaluate the robustness of our findings, we created three SNP datasets: one with only intergenic SNPs (intergenic) and two pruned using different linkage criteria, *R*^2^ = 0.01 (LD1) and *R*^2^ = 0.1 (LD2) (Additional file 26: Table S10). All Admixture runs across the three datasets consistently pointed to five ancestral groups (Fig. 6). In contrast, LEA and fastStructure, employing simple and logistic priors, detected more ancestral groups, especially with the island samples.

We reviewed the literature to evaluate how our results compare to previous studies that analyzed the genetic structure of *Ae. albopictus* in the native range. We focused only on studies where sampling localities in Asia and the Indian Ocean were used for the first time, and selectively review pertinent literature in greater detail in the supplemental Additional file 22: File S22. We acknowledge the challenges in comparing studies with different sampling density and molecular markers, and our comparisons were determined by the availability of data from different makers in Asia. Relevant papers for the native range, such as those by Black et al. [56], Kambhampati et al. [57], and Urbanelli et al. [58], found variable numbers of genetic clusters in regions like Malaysia, Borneo, Japan, and Indonesia. Our findings agreed with some of these clusters but revealed notable differences, particularly in China and Indonesia. While some researchers, such as Birungi and Munstermann [59], detected genetic similarities across regions, others, such as Duong et al. [60], pinpointed structure within a single country. Recent studies, such as Kotsakiozi et al. [18], expanded this genetic understanding for *Ae. albopictus*, but identified only one cluster in Asia, probably due to limited sampling. Our research, compared to previous studies, emphasizes the rich genetic diversity and evolving insights into *Ae. albopictus* in Asia and highlights how the specific markers and different sampling locations utilized can influence results.

We reached consistent ancestral population counts with different algorithms through meticulous parameter adjustments in LEA, fastStructure, and Admixture. This highlights the importance of algorithm fine-tuning and the utility of applying multiple algorithms in such studies. Neural Admixture [38] outperformed other algorithms in terms of efficiency and speed. Processing around 60,000 SNPs took under a minute using two CPUs and one GPU, a significant time-saver compared to other algorithms that required several hours or days using 300 CPUs. As more samples are added to the dataset, efficiency in compute time becomes more important.

The PCA identified the main clusters in the native range (Figs. 1 and 8). East Asia formed one group, while South and Southeast Asian samples were grouped together, and Indonesian samples formed their own cluster. Overall, the PCA results indicated a distribution of samples from East Asia along PC1, following a latitudinal order, with Japanese samples on the right and Chinese at the left (Fig. 7). The samples from mainland Japan clustered at the right of PC1, matching the clustering pattern from the ancestral analysis (“green” cluster Fig. 6). Finally, DAPC results with two discriminant functions correctly identified the five genetic clusters, supporting the differentiation of the QNC population (Vietnam), but also indicated that the KUN population (Maldives) may be genetically differentiated from the other populations from South Asia (Fig. 8).

The F_ST_ shows distinct trends across different countries for our intergenic (gray), LD1 (blue), and LD2 (orange) datasets (Additional file 25: Fig. S18). The intergenic SNP set displays a relatively uniform pattern across the countries. On the other hand, LD1 shows a gradual increase in genetic differentiation. Similarly, LD2, while ascending, is more similar to the intergenic set. These varying trends underscore the distinct genetic nuances that each SNP set captures across diverse populations.

Isolation by distance analyses revealed a correlation with geographical distance in China and Thailand (Additional file 25: Fig. S19). This correlation, however, varies in strength, with China’s high *R*^2^ value of 1 indicating a negative robust relationship, whereas the combined data for all populations displays a weaker *R*^2^ value of 0.00. In Thailand we observed a positive slope, with genetic distance increasing with geographical distance. This variation underscores the importance of local factors in shaping genetic diversity and differentiation within this mosquito.

With the Mantel test (Additional file 25: Fig. S20), the relationship between genetic and geographical distances in Asia becomes more apparent, with a correlation coefficient of 0.23 (panel A of Additional file 25: Fig. S20). Regression of genetics on geographical (log) distance indicated that around 5% of the genetic variation (*R*^2^ = 0.05) aligns with the geographical distance. While the test was not significant (*P* = 0.053), a more uniform sampling across Asia is necessary for a definitive conclusion. The lack of significant correlation between the genetic and geographical distance could be due to long-range invasions or migrations, facilitated by human transport [61–65].

We evaluated the relationship between admixture proportions and F_ST_ values among five genetic clusters (Additional file 25: Fig. S21). Countries like Vietnam and Thailand exhibit distinct genetic proportions, with values of 0.55 and 0.79, respectively. These admixture levels contrast with F_ST_ values, such as 0.14 between the “yellow” and “green” clusters, indicating genetic differentiation. Essentially, the data suggest that regions with lower admixture proportions display significant genetic differentiation from other clusters, hinting at the complex interplay of evolutionary histories and migrations in these populations.

We anticipate that our SNP chip, with its coverage of nearly all genes, will effectively distinguish between male and female mosquitoes. By leveraging the frequency of SNPs within genes that govern sex determination, we can develop a pipeline specifically tailored for sex determination. This targeted approach promises to enhance our understanding of the genetic basis of sex within mosquito populations, providing a crucial tool for research and vector control strategies.

In the evolving landscape of genomic research, the SNP chip designed for *Ae. albopictus* holds distinct advantages and applications compared to other techniques such as WGS, ddRAD, and RNA sequencing (RNASeq). While WGS provides comprehensive genomic information, it is often resource-intensive regarding data storage, computational requirements, and cost, especially for large sample sizes (Additional file 24: File S24). With its targeted approach, the SNP chip offers a more cost-effective and efficient solution for large-scale genotyping, sacrificing some genomic detail for broader applicability. Compared to ddRAD, which balances resolution and throughput, the SNP chip still stands out for its higher throughput and consistency, which is particularly useful in population-level studies. Unlike RNASeq, which is an important tool for expression profiling and functional genomics, the SNP chip specifically addresses genetic variation at the DNA level, with the added advantage of being able to analyze field samples not specifically preserved for RNA-based studies, which can be hard to do when samples are collected in remote locations with little access to the equipment or consumables necessary for RNA preservation. The SNP chip is particularly effective for studying population structure, phylogenetics, and marker trait associations, where stable DNA-based markers are required. Each technique has its niche, and the SNP chip fills a vital gap, providing a practical and reliable tool for genetic analysis in vector biology and public health research.

The SNP chip we developed presents a highly valuable tool for genetic research, particularly in studies focused on population genetics, evolutionary biology, and ecological monitoring. Its foremost advantage lies in its capacity for rapid genotyping, enabling the efficient processing of large sample sizes, which is a game-changer for large-scale genetic surveys and time-sensitive projects. Despite being an intermediate option in terms of cost, falling between ddRAD and WGS, the SNP chip offers a unique balance of efficiency, specificity, and cost-effectiveness. It excels in identifying known genetic markers and assessing broad-scale genetic diversity, making it an ideal choice for researchers aiming to understand genetic variations within well-characterized genomes or across large populations. While it may not be suited for uncovering new genetic variations or conducting fine-scale genetic analyses, its precision in targeted SNP genotyping makes it an indispensable tool for many genetic research applications. Overall, the SNP chip represents a significant step forward in genetic analysis, offering researchers a robust and efficient means to advance their studies in genetics and ecology.

## Conclusions

The newly developed SNP chip offers several benefits over WGS and represents a powerful tool for future research in *Ae. albopictus*. First, the chip is cost-effective, time-efficient, and accessible without genomics expertise and extensive computational resources. Secondly, the presence of 100,000 polymorphic sites in our study ensures high resolution and accuracy, making this approach highly effective in detecting population structure and regional differentiation. For example, in combination with information on geographical structure in the ancestral range presented in this paper and ongoing population genomics analyses, the chip will provide a new tool for efficiently determining the origin of newly invasive populations. Because of their large number, density, and relatively even distribution across the genome, the SNPs detected by chip will be useful for GWAS of traits related to vector biology and geographical adaptation such as insecticide resistance and vector competence. Thus, although WGS may be necessary for some applications that the chip is not well suited for, such as reconstruction of historical demography, the newly developed SNP chip can be used to address a wide range of questions of both applied and fundamental significance.

### Supplementary Information

**Additional file 1. **WGS analyses and SNP discovery.

**Additional file 2. **Mapping probes to reference genomes.

**Additional file 3. **Segregation analysis from laboratory crosses.

**Additional file 4. **Comparing genotypes of samples genotypes with WGS and chip.

**Additional file 5.** Comparing the genotypes of replicate samples.

**Additional file 6.** Functional annotation of SNPs and chip bias evaluation.

**Additional file 7.** Quality control for wild samples genotyped with the chip.

**Additional file 8.** Linkage analysis with PopLDdecay.

**Additional file 9.** Admixture analysis.

**Additional file 10.** LEA analysis.

**Additional file 11.** fastStructure analysis.

**Additional file 12.** Neural Admixture analysis.

**Additional file 13.** Interpolation of admixture matrices over Asia.

**Additional file 14.** Evaluating the impact of chip bias with LEA and PCA.

**Additional file 15.** Fst analysis.

**Additional file 16.** Create map with samples.

**Additional file 17.** Microsoft Word file with supplemental methods.

**Additional file 18.** Microsoft Excel file with probe sequences.

**Additional file 19.** PDF file from Axion Suite for the crosses genotype call.

**Additional file 20.** PDF file from Axion Suite for the wild genotype call.

**Additional file 21.** Microsoft Excel file with the result of the segregation test.

**Additional file 22.** Microsoft Word file with the literature review of the population structure of Ae. albopictus in Asia.

**Additional file 23.** Compressed text file with the scores of each polymorphic site for probe design.

**Additional file 24.** Cost estimate for the chip.

**Additional file 25.** Supplementary Figures.

**Additional file 26.** Supplementary Tables.

## Data Availability

The code describing the step-by-step of all analyses is available in GitHub (GitHub page or GitHub project). The raw data, the genotype call files, and all the files required to reproduce the analyses are available in Zenodo (https://doi.org/10.5281/zenodo.10048029).
