# Supplementary material for: A genotyping array for the globally invasive vector mosquito, Aedes albopictus
Source: Parasit Vectors. 2024 Mar 4;17:106. doi: 10.1186/s13071-024-06158-z (PMC10910840; doi:10.1186/s13071-024-06158-z)
Supplement: Supplementary file 1 — Additional file 1. WGS analyses and SNP discovery. [file 13071_2024_6158_MOESM1_ESM.html]

Aedes albopictus SNP chip - SNP discovery


# Aedes albopictus SNP chip - SNP discovery

#### Luciano V Cosme

#### 2023-08-23

- 1. Convert bam files to
  fastq
- 2. Map
  sequences to reference genome using BWA MEM
- 3. Index bam files with
  samtools
- 4. Mark
  PCR duplicates
- 5. Index bam files with
  samtools
- 6. Create
  headers
- 6. Create indel
  intervals for realignment
- 7. Perform indel realignment
- 8. Run SNP Cleaner
  pipeline to find sites
- 9. Get sites for genotype
  calls
- 10. Genotype calls using
  the sites files

In this markdown I write out the bash scripts I used on the cluster
to align sequences to the reference genome, do quality control on the
bam files, and select sites to do a hard genotype call using 819
samples.

## 1. Convert bam files to fastq

I received bam files aligned to AalbF2 and I needed to map the reads
to the AalBF3 genome assembly.

```
#!/bin/sh
#SBATCH --mail-type=BEGIN,END,FAIL          
#SBATCH --mail-user=luciano.cosme@yale.edu 
#SBATCH --ntasks=1
#SBATCH --cpus-per-task=4             
#SBATCH --mem-per-cpu=20gb                 
#SBATCH --time=120:00:00                         
#SBATCH --array=1-819
#SBATCH --job-name=bam2fastq
#SBATCH -o picard.%A_%a.bam2fastq.o.txt
#SBATCH -e picard.%A_%a.bam2fastq.ERROR.txt

module load picard/2.9.0-Java-1.8.0_121
cd /ycga-gpfs/scratch60/caccone/lvc26/fastq

samplesheet="bams5.txt"

threads=$SLURM_JOB_CPUS_PER_NODE

name=`sed -n "$SLURM_ARRAY_TASK_ID"p $samplesheet |  awk '{print $1}'`
r1=`sed -n "$SLURM_ARRAY_TASK_ID"p $samplesheet |  awk '{print $2}'`

java -Xmx80g -jar /$EBROOTPICARD/picard.jar SamToFastq VALIDATION_STRINGENCY=LENIENT \
I=$r1 \
FASTQ=$name\.R1.fastq.gz SECOND_END_FASTQ=$name\.R2.fastq.gz \
UNPAIRED_FASTQ=$name\.0.fastq.gz
```

## 2. Map sequences to reference genome using BWA MEM

```
#!/bin/sh
#SBATCH --mail-type=BEGIN,END,FAIL          
#SBATCH --mail-user=luciano.cosme@yale.edu 
#SBATCH --ntasks=1
#SBATCH --cpus-per-task=10            
#SBATCH --mem-per-cpu=4gb                 
#SBATCH --time=120:00:00                         
#SBATCH --array=1-819
#SBATCH --job-name=bwaMEM
#SBATCH -o bwa.%A_%a.bwa.o.txt
#SBATCH -e bwa.%A_%a.bwa.ERROR.txt
#SBATCH --requeue
 
module load BWA/0.7.17-foss-2016b
module load HTSlib/1.4.1-foss-2016a
module load SAMtools/1.9-foss-2016b

cd /gpfs/ycga/project/caccone/lvc26/september_2020/bams

samplesheet="fastq.txt"

threads=$SLURM_JOB_CPUS_PER_NODE

name=`sed -n "$SLURM_ARRAY_TASK_ID"p $samplesheet |  awk '{print $1}'`
r1=`sed -n "$SLURM_ARRAY_TASK_ID"p $samplesheet |  awk '{print $2}'` 
r2=`sed -n "$SLURM_ARRAY_TASK_ID"p $samplesheet |  awk '{print $3}'`

bwa mem -t 20 /gpfs/ycga/project/caccone/lvc26/september_2020/genome/aedes_albopictus_LA2_20200826.fasta \
$r1 $r2 | samtools view -@ 20 -bS - | samtools sort -m 1G -@ 20 -O BAM -o $name\.sorted.bam -
```

## 3. Index bam files with samtools

I know, I could have piped it with the last script but I did it
afterwards.

```
#!/bin/sh
#SBATCH --mail-type=BEGIN,END,FAIL          
#SBATCH --mail-user=luciano.cosme@yale.edu 
#SBATCH --ntasks=1
#SBATCH --cpus-per-task=10            
#SBATCH --mem-per-cpu=4gb                 
#SBATCH --time=120:00:00                         
#SBATCH --array=1-819
#SBATCH --job-name=INDEX
#SBATCH -o samtools.%A_%a.samtools.o.txt
#SBATCH -e samtools.%A_%a.samtools.ERROR.txt
#SBATCH --requeue                 

module load SAMtools/1.9-foss-2016b

cd /gpfs/ycga/scratch60/caccone/lvc26/dedup

samplesheet="bams4.txt"

threads=$SLURM_JOB_CPUS_PER_NODE

r1=`sed -n "$SLURM_ARRAY_TASK_ID"p $samplesheet |  awk '{print $2}'`

samtools index -@ 20 $r1
```

## 4. Mark PCR duplicates

```
#!/bin/sh
#SBATCH --mail-type=BEGIN,END,FAIL          
#SBATCH --mail-user=luciano.cosme@yale.edu 
#SBATCH --ntasks=1
#SBATCH --cpus-per-task=10             
#SBATCH --mem-per-cpu=9gb                 
#SBATCH --time=120:00:00                         
#SBATCH --array=1-819
#SBATCH --job-name=PICARD
#SBATCH -o picard.%A_%a.picard.o.txt
#SBATCH -e picard.%A_%a.picard.ERROR.txt
#SBATCH --requeue

pwd; hostname; date 

module load picard/2.9.0-Java-1.8.0_121

cd /gpfs/ycga/scratch60/caccone/lvc26/dedup

samplesheet="bams4.txt"

threads=$SLURM_JOB_CPUS_PER_NODE

name=`sed -n "$SLURM_ARRAY_TASK_ID"p $samplesheet |  awk '{print $1}'`
r1=`sed -n "$SLURM_ARRAY_TASK_ID"p $samplesheet |  awk '{print $2}'`

java -Xmx90G -jar $EBROOTPICARD/picard.jar MarkDuplicates \
I=$r1 O=$name\.dedup.bam M=$name\.dedup.txt VALIDATION_STRINGENCY=LENIENT
```

## 5. Index bam files with samtools

```
#!/bin/sh
#SBATCH --mail-type=BEGIN,END,FAIL          
#SBATCH --mail-user=luciano.cosme@yale.edu 
#SBATCH --ntasks=1
#SBATCH --cpus-per-task=2            
#SBATCH --mem-per-cpu=20gb                 
#SBATCH --time=120:00:00                         
#SBATCH --array=1-819
#SBATCH --job-name=INDEX
#SBATCH -o samtools.%A_%a.samtools.o.txt
#SBATCH -e samtools.%A_%a.samtools.ERROR.txt
#SBATCH --requeue                 

module load SAMtools/1.9-foss-2016b

cd /gpfs/ycga/scratch60/caccone/lvc26/dedup

samplesheet="bams4.txt"

threads=$SLURM_JOB_CPUS_PER_NODE

r1=`sed -n "$SLURM_ARRAY_TASK_ID"p $samplesheet |  awk '{print $2}'`

samtools index -@ 4 $r1
```

## 6. Create headers

```
#!/bin/sh
#SBATCH --mail-type=BEGIN,END,FAIL          
#SBATCH --mail-user=luciano.cosme@yale.edu 
#SBATCH --ntasks=1
#SBATCH --cpus-per-task=2           
#SBATCH --mem-per-cpu=5gb                 
#SBATCH --time=120:00:00                         
#SBATCH --array=1-819
#SBATCH --job-name=a.PICARD
#SBATCH -o picard.%A_%a.picard.o.txt
#SBATCH -e picard.%A_%a.picard.ERROR.txt
#SBATCH --requeue

pwd; hostname; date

module load picard/2.9.0-Java-1.8.0_121

cd /gpfs/ycga/scratch60/caccone/lvc26/header

samplesheet="bams4.txt"

threads=$SLURM_JOB_CPUS_PER_NODE

name=`sed -n "$SLURM_ARRAY_TASK_ID"p $samplesheet |  awk '{print $1}'`
r1=`sed -n "$SLURM_ARRAY_TASK_ID"p $samplesheet |  awk '{print $2}'`

java -Xmx90G -jar $EBROOTPICARD/picard.jar AddOrReplaceReadGroups \
I=$r1 \
O=$name\.header.bam \
RGSM=$name RGLB=wgs RGPL=illumina RGPU=none VALIDATION_STRINGENCY=LENIENT
```

## 6. Create indel intervals for realignment

```
#!/bin/sh
#SBATCH --mail-type=BEGIN,END,FAIL          
#SBATCH --mail-user=luciano.cosme@yale.edu 
#SBATCH --ntasks=1
#SBATCH --cpus-per-task=1           
#SBATCH --mem-per-cpu=10gb                 
#SBATCH --time=120:00:00                         
#SBATCH --array=1-819
#SBATCH --job-name=gatk3_indel
#SBATCH -o gatk3.%A_%a.gatk.o.txt
#SBATCH -e gatk3.%A_%a.gatk.ERROR.txt

module load GATK/3.8-0-Java-1.8.0_121

cd /ycga-gpfs/scratch60/caccone/lvc26/indel

samplesheet="bams6.txt"

threads=$SLURM_JOB_CPUS_PER_NODE

name=`sed -n "$SLURM_ARRAY_TASK_ID"p $samplesheet |  awk '{print $1}'` 
r1=`sed -n "$SLURM_ARRAY_TASK_ID"p $samplesheet |  awk '{print $2}'` 

java -Xmx20g -jar $EBROOTGATK/GenomeAnalysisTK.jar \
-T RealignerTargetCreator \
-R /gpfs/ycga/project/caccone/lvc26/september_2020/genome/aedes_albopictus_LA2_20200826.fasta \
-I $r1 \
-o $name\.realigner.intervals
```

## 7. Perform indel realignment

```
#!/bin/sh
#SBATCH --mail-type=BEGIN,END,FAIL          
#SBATCH --mail-user=luciano.cosme@yale.edu 
#SBATCH --ntasks=1
#SBATCH --cpus-per-task=2           
#SBATCH --mem-per-cpu=10gb                 
#SBATCH --time=120:00:00                         
#SBATCH --array=1-819
#SBATCH --job-name=GATK_IR
#SBATCH -o gatk3.IR.%A_%a.gatk.o.txt
#SBATCH -e gatk3.IR.%A_%a.gatk.ERROR.txt

module load GATK/3.8-1-0-gf15c1c3ef-Java-1.8

cd /ycga-gpfs/scratch60/caccone/lvc26/indel

samplesheet="bams.txt"

threads=$SLURM_JOB_CPUS_PER_NODE

name=`sed -n "$SLURM_ARRAY_TASK_ID"p $samplesheet |  awk '{print $1}'` 
r1=`sed -n "$SLURM_ARRAY_TASK_ID"p $samplesheet |  awk '{print $2}'` 
r2=`sed -n "$SLURM_ARRAY_TASK_ID"p $samplesheet |  awk '{print $3}'` 

java -Xmx80g -jar $EBROOTGATK/GenomeAnalysisTK.jar \
-T IndelRealigner \
-R /gpfs/ycga/project/caccone/lvc26/september_2020/genome/aedes_albopictus_LA2_20200826.fasta \
-I $r1 \
-targetIntervals $r2 \
-o $name\.realigned.bam
```

## 8. Run SNP Cleaner pipeline to find sites

I used this R script below along with the SNP cleaner script to find
sites with good sequence coverage. I did run it for each population and
changed the NUM\_SAMS and MIN\_IND according to the number of mosquitoes
in each population.

After running this for each population, it created 4 directories with
a list of sites “excl”, “filt”, “unfilt”, and “keeps”. Because we divide
the calls into chunks of 10Mb, I concatenate the keeps sites in the next
step.

```
#############################################################################
# Make a commands file to identify set of 'robust' sites for analysis with 
# ANGSD using samtools -> bcftools -> SNPcleaner
# 
# OUTPUT: .sh text file with one commands per line.  Execute to produce sites
# files in chunks for use with ANGSD.  
#
# Execute commands using GNU parallel, for example with 16 jobs in parallel:
# parallel -j 16 -a commands.sh
# (note that the pbzip commands are multithreaded so each command uses >1 CPU) 
#
# Combine sites files in whatever group desired
# POP=Tampon
# cat ./keeps/$POP.pileup.FILT.*.chnk.*.sites | sort -k 2,2n > ./$POP.pileup.FILT.sites 
#
## Dependencies #######
# snpCleaner.pl
# samtools added to $PATH
# pbzip2 installed
# bcftools added to $PATH
# module load R/3.5.0-foss-2016b-avx2
# module load dSQ/0.96
#############################################################################

## Name of population for processing
POP='Name_of_population_here' 

## Today's date, to keep records if different runs/versions are required
date = 'December_5_2020'

#############################################################################
## Define file handles and make directories
# Path to text file with full path to bams, one per line
#BAM_FILES=paste('/path/to/bam/File.',POP,'.bams', sep = '')
BAM_FILES = paste('/ycga-gpfs/project/caccone/lvc26/september_2020/pops/bams.',
                  POP,
                  '.txt',
                  sep = '')

## Output commands file name
OUTPUT_FILE = paste(
  '/ycga-gpfs/project/caccone/lvc26/september_2020/sites/',
  POP,
  '.mpile.snpcln.',
  date,
  '.txt',
  sep = ''
)

## Sites file directory and prefix
DIRS = paste('/ycga-gpfs/project/caccone/lvc26/september_2020/sites/',
             POP,
             '/sites',
             sep = '')

## make subdirs
system(paste('mkdir -p -m 777 ', DIRS, sep = ''))
system(paste('mkdir -m 777 ', DIRS, '/excl', sep = '')) # for excluded sites
system(paste('mkdir -m 777 ', DIRS, '/filt', sep = ''))
system(paste('mkdir -m 777 ', DIRS, '/unfilt', sep = ''))
system(paste('mkdir -m 777 ', DIRS, '/keeps', sep = ''))

#############################################################################
## Set variables

REF='/ycga-gpfs/project/caccone/lvc26/september_2020/genome/aedes_albopictus_LA2_20200826.fasta'
FAI = read.table(paste(REF,'.fai',sep = ''), header = F) ## samtools fasta index
#SNPCLNR='/gpfs/ycga/project/caccone/lvc26/genome/snpCleaner.pl'
SNPCLNR='/ycga-gpfs/project/caccone/lvc26/september_2020/snpCleaner.pl'
## MIN_IND: Minimum number indivuals to include (specifically, only analyze sites with at least MIN_IND individuals with at least 2 reads)
## MAXD: And maximum depth combined across individuals per site
NUM_SAMS = as.numeric(nrow(read.table(BAM_FILES, header = F)))
if (NUM_SAMS <= 9) {
  MIN_IND = NUM_SAMS
  MAXD = MIN_IND * 3 * 15
} else if (NUM_SAMS > 9) {
  MIN_IND = 9
  MAXD = (NUM_SAMS * 0.8) * 3 * 15
}

# SNPcleaner thresholds
f = 1e-4 # min p-value for map quality bias
b = 1e-10 # min p-value for base quality bias
L = 1e-6 # min p-value for exact test of heterozygote deficit
H = 1e-6 # min p-value for exact test of heterozygote excess
a = 0 # minimum number of alternate alleles for site
u = 2 # minimum read depth for an individual (required by -k)

## Unchanged defaults in SNPcleaner
# S = 0.0001 # min p-value for strand bias
# Q = 10 # minimum RMS mapping quality for SNPS
# d = 2 # minium site read depth
# e = 0.0001 # min p-value for end distance bias 
# h = 0.0001 # min p-value for exact test of HWE
# F = 0 # inbreeding coefficient value
# v # process non-variant sites


#############################################################################
## Make commands file
# on Farnam  export PATH=$PATH:/home/lvc26/project/samtools/samtools-1.3.1
MOD = 'export PATH=$PATH:/home/lvc26/project/software/pbzip2-1.1.13; module load SAMtools/1.3.1-foss-2016b BCFtools/1.5-foss-2016b;'
## Start with a clean file
try(system(paste('rm ', file, sep = '')), silent = TRUE)

## loop over scaffolds
for (i in 1:nrow(FAI)) {
  if (i %% 1000 == 0)
    cat(' --> Now on scaff ', i, '\n')
  
  
  ## Define
  chr = as.character(FAI[i, 1])
  
  
  ## Break up large scaffolds into 10MB chunks
  chnks = floor(as.numeric(as.character(FAI[i, 2])) / 10e6) + 1
  
  ## Loop over chunks
  for (c in 1:chnks) {
    ## Define start and stop of chunk
    end = c * 10e6
    strt = end - 10e6 + 1
    
    ## Construct full command
    cmmnd = paste(
      MOD,
      ' samtools mpileup -f ',
      REF,
      ' -b ',
      BAM_FILES,
      ' -q 10 -Q 20 -I -u -t SP,DP -r \'',
      chr,
      ':',
      as.integer(strt),
      '-',
      as.integer(end),
      '\' | bcftools call -f GQ -c - | pbzip2 -p3 -cz --best > ',
      DIRS,
      '/unfilt/',
      POP,
      '.pileup.unfilt.',
      chr,
      '.chnk.',
      c,
      '.vcf.bz2 && bzcat ',
      DIRS,
      '/unfilt/',
      POP,
      '.pileup.unfilt.',
      chr,
      '.chnk.',
      c,
      '.vcf.bz2 |perl ',
      SNPCLNR,
      ' -v -k ',
      MIN_IND,
      ' -u ',
      u,
      ' -D ',
      MAXD,
      ' -a ',
      a,
      ' -f ',
      f,
      ' -H ',
      H,
      ' -L ',
      L,
      ' -b ',
      b,
      ' -p ',
      DIRS,
      '/excl/',
      POP,
      '.pileup.EXCL.',
      chr,
      '.chnk.',
      c,
      '.vcf.bz2 | pbzip2 -p3 -cz --best > ',
      DIRS,
      '/filt/',
      POP,
      '.pileup.FILT.',
      chr,
      '.chnk.',
      c,
      '.vcf.bz2 && bzcat ',
      DIRS,
      '/filt/',
      POP,
      '.pileup.FILT.',
      chr,
      '.chnk.',
      c,
      '.vcf.bz2 | grep \'DP=\' |cut -f1,2 > ',
      DIRS,
      '/keeps/',
      POP,
      '.pileup.FILT.',
      chr,
      '.chnk.',
      c,
      '.sites',
      sep = ''
    )
    
    ## Append command (cmmnd) as new line on OUTPUT_FILE
    write.table(
      cmmnd,
      file = OUTPUT_FILE,
      append = T,
      quote = F,
      col.names = F,
      row.names = F
    )
    
  }
}
```

After running this R code above it creates a file with 2198 lines
(one for each chunk). I used dSQ (dead simple queue) tool to submit as
sbatch. Below are the first two lines of one population as an
example.

```
# This is only an example
samtools mpileup -f /home/lvc26/project/albopictus/genome/albo.genome.fasta -b /home/lvc26/project/albopictus/bams/bams.jp.txt -q 10 -Q 20 -I -u -t SP,DP -r 'NW_021837045.1:190000001-200000000' | bcftools call -f GQ -c - | pbzip2 -p3 -cz --best > /home/lvc26/project/albopictus/angsd/jp/sites/unfilt/jp.pileup.unfilt.NW_021837045.1.chnk.20.vcf.bz2 && bzcat /home/lvc26/project/albopictus/angsd/jp/sites/unfilt/jp.pileup.unfilt.NW_021837045.1.chnk.20.vcf.bz2 |perl /home/lvc26/project/albopictus/angsd/snpCleaner.pl -v -k 12 -u 2 -D 864 -a 0 -f 1e-04 -H 1e-06 -L 1e-06 -b 1e-10 -p /home/lvc26/project/albopictus/angsd/jp/sites/excl/jp.pileup.EXCL.NW_021837045.1.chnk.20.vcf.bz2 | pbzip2 -p3 -cz --best > /home/lvc26/project/albopictus/angsd/jp/sites/filt/jp.pileup.FILT.NW_021837045.1.chnk.20.vcf.bz2 && bzcat /home/lvc26/project/albopictus/angsd/jp/sites/filt/jp.pileup.FILT.NW_021837045.1.chnk.20.vcf.bz2 | grep 'DP=' |cut -f1,2 > /home/lvc26/project/albopictus/angsd/jp/sites/keeps/jp.pileup.FILT.NW_021837045.1.chnk.20.sites
samtools mpileup -f /home/lvc26/project/albopictus/genome/albo.genome.fasta -b /home/lvc26/project/albopictus/bams/bams.jp.txt -q 10 -Q 20 -I -u -t SP,DP -r 'NW_021837046.1:60000001-70000000' | bcftools call -f GQ -c - | pbzip2 -p3 -cz --best > /home/lvc26/project/albopictus/angsd/jp/sites/unfilt/jp.pileup.unfilt.NW_021837046.1.chnk.7.vcf.bz2 && bzcat /home/lvc26/project/albopictus/angsd/jp/sites/unfilt/jp.pileup.unfilt.NW_021837046.1.chnk.7.vcf.bz2 |perl /home/lvc26/project/albopictus/angsd/snpCleaner.pl -v -k 12 -u 2 -D 864 -a 0 -f 1e-04 -H 1e-06 -L 1e-06 -b 1e-10 -p /home/lvc26/project/albopictus/angsd/jp/sites/excl/jp.pileup.EXCL.NW_021837046.1.chnk.7.vcf.bz2 | pbzip2 -p3 -cz --best > /home/lvc26/project/albopictus/angsd/jp/sites/filt/jp.pileup.FILT.NW_021837046.1.chnk.7.vcf.bz2 && bzcat /home/lvc26/project/albopictus/angsd/jp/sites/filt/jp.pileup.FILT.NW_021837046.1.chnk.7.vcf.bz2 | grep 'DP=' |cut -f1,2 > /home/lvc26/project/albopictus/angsd/jp/sites/keeps/jp.pileup.FILT.NW_021837046.1.chnk.7.sites
```

Below is a dsq file as an example

```
#!/bin/bash
#SBATCH --partition=scavenge
#SBATCH --array 0-2198
#SBATCH --output /dev/null
#SBATCH --job-name Foshan
#SBATCH --mem-per-cpu 6g -t 20:00:00 --mail-type ALL

# DO NOT EDIT LINE BELOW
/ycga-gpfs/apps/hpc/software/dSQ/0.96/dSQBatch.py /gpfs/ycga/project/caccone/lvc26/september_2020/sites/Foshan.mpile.snpcln.October_20_2020.txt /gpfs/ycga/project/caccone/lvc26/september_2020/sites
```

Below is the SNP cleaner script (snpCleaner.pl)

```
#!/usr/bin/perl

# SNPcleaner.pl
# Author: Tyler Linderoth, tylerp.linderoth@gmail.com
my $version = "2.4.1";

# TODO:
#    Add argument check
#    Output smaller BED file (join contiguous regions)

use strict;
use warnings;
use Getopt::Std;
use IO::Compress::Bzip2;

my %opts = ('?'=> 0, 'd'=>2, 'D'=>1000000, 'k'=>1, 'u'=>0, 'a'=>2, 'Q'=>10, 'S'=>1e-4, 'b'=>1e-100, 'f'=>0, 'e'=>1e-4, 'h'=>1e-4, 'F'=>0, 'H'=>0, 'L'=>0, 'A'=>undef,
        'M'=>undef, 'B'=>undef, 'p'=>undef, 'r'=>undef, 'X'=>undef, 't'=>undef, 'g'=>undef, 'v'=>undef, '2'=>undef, 'q'=>2);

getopts('?2d:D:k:u:a:Q:S:b:f:e:h:F:H:L:A:M:B:p:r:X:q:tgv', \%opts);

die (qq/
#####################
# SNPcleaner v$version #
#####################
This scripts works with bcftools vcf file format to filter SNPs. This script is only for 
SNP filtering and will ignore INDELs. 

#######################################
Usage: snpCleaner.pl [options] <in.vcf>
or
cat <in.vcf> | snpCleaner.pl [options]

Options:
-?   help
-2   keep non-biallelic sites
-q INT   ploidy [$opts{q}]
-d INT   minimum site read depth [$opts{d}]
-D INT   maximum site read depth [$opts{D}]
-k INT   minimum number of 'covered' individuals (requires -u) [$opts{k}] 
-u INT   minimum read depth for an individual to be considered 'covered' (requires -k) [$opts{u}]
-a INT   minimum number of alternate alleles for site [$opts{a}]
-Q INT   minimum RMS mapping quality for SNPS [$opts{Q}]
-S FLOAT min p-value for strand bias [$opts{S}]
-b FLOAT min p-value for base quality bias [$opts{b}]
-f FLOAT min p-value for map quality bias [$opts{f}]
-e FLOAT min p-value for end distance bias [$opts{e}]
-h FLOAT min p-value for exact test of HWE [$opts{h}]
-F FLOAT inbreeding coefficient value [$opts{F}]
-H FLOAT min p-value for exact test of excess of heterozygous [$opts{H}]
-L FLOAT min p-value for exact test of defect of heterozygous [$opts{L}]
-A FILE  ANCESTRAL fasta file (with FAI on same folder)
-M CHAR  mutation type(s) to remove (ex. 'CT_GA') (requires -A)
-B CHAR  name of BED file for kept SNP positions
-p CHAR  name of file to dump sites that failed filters (bziped)
-r FILE  list of contigs to exclude
-X FILE  BED file of exonic regions (sorted from lowest to highest contig)
-t       filter non-exonic sites (requires -X)
-g   filter exons with SNPs out of HWE (requires -X)
-v   process nonvariants

Generating input VCF with BCFtools:
-run 'bcftools mpileup' with '-a SP,DP' to provide depth and strand bias info
-run 'bcftools call' using '-f GC -c'

An example for how to generate the input VCF file:
bcftools mpileup -f <reference fasta> -b <bam list> -a SP,DP | bcftools call -f GQ -c - > unfiltered_sites.vcf 

Consider using 'bcftools call --skip-variants indels' since these sites will
be ignored by snpCleaner anyways.

Output Notes:
-bed format file is zero-based.
-Characters preceeding filtered sites (dumped with option -p) indicate filters that the sites
 failed to pass and correspond to the option flags (e.g. 'S' indicates strand bias).
\n/) if ($opts{'?'} || (!$ARGV[0] && -t STDIN));

# Argument check
if($opts{t} || $opts{g}) {
    die(qq/option -X (exonic region BED file) is required for options -t and -g\n/) unless ($opts{X});
}
if($opts{M}) {
    die(qq/option -A (ANCESTRAL fasta file) is required for option -M\n/) unless ($opts{A});
}
if($opts{k} !~ m/^\d+$/g || $opts{u} !~ m/^\d+$/g) {
    die(qq/option -k and -u are inter-dependent (min number of -k INT individuals with less than -u INT coverage)\n/);
}

if ($opts{q} < 1) {
    die(qq/Invalid ploidy option -q: $opts{q} ... ploidy should be >= 1\n/);
}
else
{
    if ($opts{q} != 2 && ($opts{h} + $opts{H} + $opts{L}) > 0) {
        print STDERR "Warning: Hardy-Weinberg filtering only applicable for diploid data (-q 2)\n";
    }
}

my ($excont, @t, @seq, @buffer, %exons, %ancestral);
my ($n_sites, $ind_dp, $prev_format, @format, @ind_depth) = (0,0,"");

#open some necessary filehandles
open(BED, '>', $opts{B}) or die("ERROR: could not create BED file: $!") if($opts{B});
my $bz2 = new IO::Compress::Bzip2($opts{p}, 'Append' => 0) if($opts{p});


# Read file with list of excluded contigs
if($opts{r}) {
    open(RMV, '<', $opts{r}) or die("ERROR: could not open excluded CONTIGS file: $!");
    $excont = join("\t", <RMV>);
    close(RMV);
}

# Read FASTA index file
if($opts{A}) {
    open(FASTA, '<', $opts{A}) or die("ERROR: could not open FASTA file: $!");
    binmode(FASTA);
    
    open(FAI, '<', $opts{A}.".fai") or die("ERROR: could not open FAI file: $!");
    while(<FAI>){
    chomp;
    my @fai = split(/\t/);
    $ancestral{$fai[0]}{'length'} = $fai[1];
    $ancestral{$fai[0]}{'start'} = $fai[2];
    $ancestral{$fai[0]}{'n_chars'} = $fai[3];
    $ancestral{$fai[0]}{'n_bytes'} = $fai[4];
    }
    close(FAI);
}

# Read exon/intron information file
if($opts{X}) {
    open(EXON, '<', $opts{X}) or die("ERROR: could not open EXON file: $!");
    while (<EXON>) {
    my @exon = split(/\s+/);
    push( @{$exons{$exon[0]}}, {'start' => $exon[1], 'end' => $exon[2], 'HWE' => 0} );
    }
    close(EXON);
}


my ($prev_contig, $cur_contig, $pos) = ('start','start',0);
$" = "\t"; #for formatting printed output

# check if input VCF is from @ARGV or STDIN
if (-t STDIN)
{
    die ("No input VCF\n") unless @ARGV;
}

# The core loop
while (<>) {
    my $violate = ''; # for flagging filter violations

    @t = split;

    # Skip (and print) header lines
    (print, next) if(m/^#/);

    # Update position
    $pos = $t[1];

    # If contig changed
    if($t[0] ne $cur_contig){
    $prev_contig = $cur_contig;
    $cur_contig = $t[0];
    }

    # Skip sites with unknown ref
    $violate .= 'N' if ($t[3] eq 'N');

    # Skip non-variable sites
    $violate .= 'v' if ($t[4] eq '.' && !$opts{v});

    # Skip Indels
    if (length($t[3]) > 1 || length($t[4]) > 1) {
    $violate .= 'I';
    next;
    }

    # Skip sites from excluded contigs
    $violate .= 'r' if ($opts{r} && $excont =~ /\b$cur_contig\b/);

    # Skip non-biallelic Sites

    if( scalar(split(/,/,$t[4])) > 1 && !$opts{2} ) {
    $violate .= '2';
    }

    # Read ANC base from FASTA
    my $anc_base;
    if($opts{A}) {
    my $n_lines = int($pos / $ancestral{$cur_contig}{'n_chars'} - 1e-6);
    my $extra_bytes_per_line = $ancestral{$cur_contig}{'n_bytes'} - $ancestral{$cur_contig}{'n_chars'};
    seek(FASTA, $ancestral{$cur_contig}{'start'} + $pos - 1 + $n_lines*$extra_bytes_per_line, 0);
    read(FASTA, $anc_base, 1);
    $anc_base = 'N' if($anc_base =~ m/[RYSWKMBDHV]/i);
    warn("WARNING: invalid ancestral base at ",$cur_contig,", pos ",$pos,": ",$anc_base,".\n") if($anc_base !~ m/[ACTGN]/i);

    # Skip non-biallelic sites (major and minor differ from ANCESTRAL)
    $violate .= "m($anc_base)" if($anc_base !~ m/\Q$t[3]\E|\Q$t[4]\E|N/i && $t[4] ne '.');
    }

    # Skip sites with specified mutation types
    if ($opts{M} && $t[3] =~ m/[ATCG]/i && $t[4] =~ m/[ATCG]/i) { # if site is variable
    my $refalt=$t[3].$t[4];
    my $altref=$t[4].$t[3];

    if ($opts{M} =~ /($refalt|$altref)/i) {
        my @mutype = split("", $1);
        $violate .= "M" if ($anc_base !~ m/$mutype[1]/i);
    }   
    }
    
    # Eveness across individuals coverage filter
    # check where in vcf FORMAT the DP ID is    
    if( $prev_format ne $t[8] ){
    $ind_dp = 0;
    $prev_format = $t[8];
    @format = split(":",$t[8]);
    foreach (@format) {
        $ind_dp++ if($_ ne 'DP');
        last if($_ eq 'DP');
    }
    }

    # count how many individuals have coverage >= $opts{u}
    my $covcount = 0;
    my @genoinfo = @t[9 .. $#t];
    if( $ind_dp <= $#format ) { # if DP is missing in vcf skip even coverage filter
    for(my $i=0; $i <= $#genoinfo; $i++) {
        my @ind_info = split(":", $genoinfo[$i]);
        $covcount++ if ($ind_info[$ind_dp] >= $opts{u});
        $ind_depth[$i] += $ind_info[$ind_dp];
    }
    $violate .= 'k' if ($covcount < $opts{k});  
    }else {
    warn("WARNING: no individual depth information at ",$cur_contig,", pos ",$pos,". Check for \"samtools mpileup -D\" option.");
    }

    # Site coverage 
    my ($dp, $dp_alt) = (0,0);
    if ($t[7] =~ m/DP4=(\d+),(\d+),(\d+),(\d+)/i) {
    $dp = $1 + $2 + $3 + $4;
    $dp_alt = $3 + $4;
    }
    $violate .= 'd' if ($dp < $opts{d});
    $violate .= 'D' if ($dp > $opts{D});
    $violate .= 'a' if ($dp > 0 && $dp_alt < $opts{a});

    # Root-mean-square mapping quality of covering reads
    my $mq = $1 if ($t[7] =~ m/MQ=(\d+)/i);
    $violate .= 'Q' if ($mq && $mq < $opts{Q});

    # Strand, baseQ, mapQ, and tail distance bias
    my ($strand, $baseqb, $mapqb, $tail_dist);
    if ($t[7] =~ m/PV4=([^,]+),([^,]+),([^,]+),([^,;\t]+)/) {
    $strand = $1;
    $baseqb = $2;
    $mapqb = $3;
    $tail_dist = $4;
    }
    $violate .= 'S' if ($strand && $strand < $opts{S});
    $violate .= 'b' if ($baseqb && $baseqb < $opts{b});
    $violate .= 'f' if ($mapqb && $mapqb < $opts{f});
    $violate .= 'e' if ($tail_dist && $tail_dist < $opts{e});

    # Identify non-exonic regions
    my $exon_id = -1;
    if ($opts{X})  {
    my $n_exons = scalar(@{$exons{$cur_contig}});
    for($exon_id = 0; $exon_id < $n_exons; $exon_id++) {
        last if ($pos >= $exons{$cur_contig}[$exon_id]{'start'} && 
             $pos <= $exons{$cur_contig}[$exon_id]{'end'});
    }
    $exon_id = -1 if($exon_id >= $n_exons);
    $violate .= 't' if ( $opts{t} && $exon_id < 0 );
    }
    
    # HWE exact test
    if ($opts{q} == 2 && $t[4] ne '.') {
    my %genocount = (homoa => 0, homob => 0, het => 0);
    foreach (@genoinfo) {
        if (/0\/0:/) {
        $genocount{homoa}++;
        } elsif (/1\/1:/) {
        $genocount{homob}++;
        } elsif (/0\/1:|1\/0:/) {
        $genocount{het}++;
        }
    }

    my ($pHWE, $pHI, $pLOW) = hwe_exact($genocount{het},$genocount{homoa},$genocount{homob},$opts{F});
    die(qq/Genotype counts less than 0\n/) if $pHWE == -1;
    if ($pHWE < $opts{h}) {
        $violate .= "h(p=$pHWE)";
        $exons{$cur_contig}[$exon_id]{'HWE'} = 1 if($exon_id > -1);
    }
    if ($pHI < $opts{H}) {
        $violate .= "H(p=$pHI)";
        $exons{$cur_contig}[$exon_id]{'HWE'} = 1 if($exon_id > -1);
    }
    if ($pLOW < $opts{L}) {
        $violate .= "L(p=$pLOW)";
        $exons{$cur_contig}[$exon_id]{'HWE'} = 1 if($exon_id > -1);
    }
    }

    # remove exons with SNPs out of HWE
    unless( $#buffer < 0 || 
        ($exon_id >= 0 &&
         $cur_contig eq $buffer[0]{'contig'} && 
         $exon_id == $buffer[0]{'exon_id'}) ) {

    print_buffer($opts{g}, \@buffer, \%exons);
    undef @buffer;
    }
    
    push( @buffer, {'contig' => $cur_contig, 'exon_id' => $exon_id, 'pos' => $pos, 'violate' => $violate, 'vcf' => join("\t",@t)} );
    $n_sites++;
}
# Force printing of last entry
print_buffer($opts{g}, \@buffer, \%exons);

print(STDERR $n_sites." sites processed!\n");
# Print per-individual depth
for(my $i=0; $i <= $#ind_depth; $i++) {
    print(STDERR "Ind ".($i+1)." depth:\t".($ind_depth[$i]/$n_sites)."\n");
}


close FASTA if($opts{A});
close BED if($opts{B});
$bz2->close if($opts{p});


exit(0);


#################
### Functions ###
#################
sub print_buffer {
    my ($opts_g, $buffer, $exons) = @_;

    foreach my $g (@$buffer) {
    $g->{'violate'} .= 'g' if($opts_g && $g->{'exon_id'} >= 0 && $exons->{$g->{'contig'}}[$g->{'exon_id'}]{'HWE'} == 1);
    
    if( !$g->{'violate'} ){
        print($g->{'vcf'}."\n");
        print(BED $g->{'contig'}."\t".($g->{'pos'}-1)."\t".$g->{'pos'}."\n") if $opts{B};
    }else{
        $bz2->print($g->{'violate'}."\t".$g->{'vcf'}."\n") if($opts{p});
    }
    }
}


sub hwe_exact {

# Citation:
# Implements an exact SNP test of Hardy-Weinberg Equilibrium as described in Wigginton et al. 2005
# note that probabilities are calculated from the midpoint in order to take advantage of the recurrence
# relationships recognized in Guo and Thompson (1992) in the implementation of their MCMC sampler

    my ($obs_hets, $obs_homa, $obs_homb, $F) = @_;

    return(-1) if ($obs_hets < 0 || $obs_homa < 0 || $obs_homb < 0);

    my $obs_homr; #rare homozygote
    my $obs_homc; #commmon homozygote
    my $n = $obs_homa + $obs_homb + $obs_hets; # total number genotypes

    # define common and rare homozygotes
    if ($obs_homa > $obs_homb) {
    $obs_homc = $obs_homa;
    $obs_homr = $obs_homb;
    } elsif ($obs_homa < $obs_homb) {
    $obs_homc = $obs_homb;
    $obs_homr = $obs_homa;
    } elsif ($obs_homa == $obs_homb) { # need to check how matching number homos affects algorithm
    $obs_homc = $obs_homa;
    $obs_homr = $obs_homb;
    }

    my $rare = 2 * $obs_homr + $obs_hets; # number of minor alleles

    # theta for inbreeding HWE calculations
    my $pc = 1 - $rare/(2*$n);
    my $pr = 1 - $pc;
    my $pCC = $pc**2 + $pc*$pr*$F;
    my $pCR = 2*$pc*$pr - 2*$pc*$pr*$F;
    my $pRR = $pr**2 + $pc*$pr*$F;
    $pRR = 1e-6 if($pRR == 0);
    my $theta = ($pCR**2)/($pCC*$pRR);
    $theta = 1e-6 if($theta == 0);

    # initialize heterozygote probability array
    my @probs;
    for (my $i = 0; $i <= $rare; $i++) {
    $probs[$i] = 0.0;
    }

    # find midpoint of the minor allele count distribution
    my $mid = int($rare * (2 * $n - $rare) / (2 * $n));
    $mid = $mid + 1 if ( ($mid % 2) != ($rare % 2) ); # ensures number minor alleles and midpoint have parity

    my $curr_hets = $mid;
    my $curr_homr = ($rare - $mid) / 2;
    my $curr_homc = $n - $curr_hets - $curr_homr;

    $probs[$mid] = 1.0;
    my $sum = $probs[$mid];

    # calculate probabilities from midpoint down 
    for ($curr_hets = $mid; $curr_hets > 1; $curr_hets -= 2) {
    $probs[$curr_hets - 2] = $probs[$curr_hets] * $curr_hets * ($curr_hets - 1) / 
        ($theta * ($curr_homr + 1) * ($curr_homc + 1));
    $sum += $probs[$curr_hets - 2];

    # 2 fewer heterozygotes for next iteration -> add one rare, one common homozygote
    $curr_homr++;
    $curr_homc++;
    }

    # calculate  probabilities from midpoint up
    $curr_hets = $mid;
    $curr_homr = ($rare - $mid) / 2;
    $curr_homc = $n - $curr_hets - $curr_homr;

    for ($curr_hets = $mid; $curr_hets <= $rare - 2; $curr_hets += 2) {
    $probs[$curr_hets + 2] = $probs[$curr_hets] * $theta * $curr_homr * $curr_homc /
        (($curr_hets + 2) * ($curr_hets + 1));
    $sum += $probs[$curr_hets + 2];
    
    # add 2 heterozygotes for next interation -> subtract one rare, one common homozygote
    $curr_homr--;
    $curr_homc--;
    }
    for (my $i = 0; $i <= $rare; $i++) {
    $probs[$i] /= $sum;
    }

    # p-value calculation for hwe
    my $p_hwe = 0.0;
    for (my $i = 0; $i <= $rare; $i++) {
    next if ($probs[$i] > $probs[$obs_hets]);
    $p_hwe += $probs[$i];
    }
    $p_hwe = 1.0 if ($p_hwe > 1);

    # alternate p-value calculation for p_hi/p_low heterozygous
    my $p_hi = $probs[$obs_hets];
    for (my $i = $obs_hets + 1; $i <= $rare; $i++) {
    $p_hi += $probs[$i];
    }
    my $p_low = $probs[$obs_hets];
    for (my $i = $obs_hets - 1; $i >= 0; $i--) {
    $p_low += $probs[$i];
    }
#    my $p_hi_low;
#    if ($p_hi < $p_low) {
#   $p_hi_low = 2 * $p_hi;
#    } else {
#   $p_hi_low = 2 * $p_low
#    }

    return($p_hwe, $p_hi, $p_low);
}
```

How I submmited the dsq jobs

```
# for dsq
module load dSQ/0.96

##############################################
                 Beijing_China
##############################################

dsq --job-file Beijing_China.mpile.snpcln.October_12_2020.txt --output /dev/null --mem-per-cpu 4g -t 20:00:00 --mail-type ALL --job-name Beijing_China 
dsqa Beijing_China.mpile.snpcln.October_12_2020.txt job_10367110_status.tsv > failedjobs.txt 2> report.txt
# dsq --job-file failedjobs.txt -o /dev/null --mem-per-cpu 4g -t 20:00:00 --mail-type ALL --job-name foshan 
# dsqa failedjobs.txt job_9361930_status.tsv > failedjobs2.txt 2> report2.txt
# dsq --job-file failedjobs2.txt -o /dev/null --mem-per-cpu 4g -t 20:00:00 --mail-type ALL --job-name foshan


##############################################
                 Guangzhou_China
##############################################

dsq --job-file Guangzhou_China.mpile.snpcln.November_16_2020.txt --output /dev/null --mem-per-cpu 4g -t 20:00:00 --mail-type ALL --job-name Guangzhou_China 
dsqa Guangzhou_China.mpile.snpcln.November_16_2020.txt job_10500031_status.tsv > failedjobs.txt 2> report.txt
# dsq --job-file failedjobs.txt -o /dev/null --mem-per-cpu 4g -t 20:00:00 --mail-type ALL --job-name foshan 
# dsqa failedjobs.txt job_9361930_status.tsv > failedjobs2.txt 2> report2.txt
# dsq --job-file failedjobs2.txt -o /dev/null --mem-per-cpu 4g -t 20:00:00 --mail-type ALL --job-name foshan

##############################################
                 Hainan_China
##############################################

dsq --job-file Hainan_China.mpile.snpcln.November_16_2020.txt --output /dev/null --mem-per-cpu 4g -t 20:00:00 --mail-type ALL --job-name Hainan_China 
dsqa Hainan_China.mpile.snpcln.November_16_2020.txt job_10500332_status.tsv > failedjobs.txt 2> report.txt
# dsq --job-file failedjobs.txt -o /dev/null --mem-per-cpu 4g -t 20:00:00 --mail-type ALL --job-name foshan 
# dsqa failedjobs.txt job_9361930_status.tsv > failedjobs2.txt 2> report2.txt
# dsq --job-file failedjobs2.txt -o /dev/null --mem-per-cpu 4g -t 20:00:00 --mail-type ALL --job-name foshan


##############################################
                 Honiara_Solomon_Islands
##############################################

dsq --job-file Honiara_Solomon_Islands.mpile.snpcln.October_13_2020.txt --output /dev/null --mem-per-cpu 5g -t 20:00:00 --mail-type ALL --job-name Honiara_Solomon_Islands 
dsqa Honiara_Solomon_Islands.mpile.snpcln.October_13_2020.txt job_10368448_status.tsv > failedjobs.txt 2> report.txt
# dsq --job-file failedjobs.txt -o /dev/null --mem-per-cpu 4g -t 20:00:00 --mail-type ALL --job-name foshan 
# dsqa failedjobs.txt job_9361930_status.tsv > failedjobs2.txt 2> report2.txt
# dsq --job-file failedjobs2.txt -o /dev/null --mem-per-cpu 4g -t 20:00:00 --mail-type ALL --job-name foshan


##############################################
                 Houston18_USA
##############################################


dsq --job-file Houston18_USA.mpile.snpcln.October_13_2020.txt --output /dev/null --mem-per-cpu 5g -t 20:00:00 --mail-type ALL --job-name Houston18_USA 
dsqa Houston18_USA.mpile.snpcln.October_13_2020.txt job_10369429_status.tsv > failedjobs.txt 2> report.txt
# dsq --job-file failedjobs.txt -o /dev/null --mem-per-cpu 4g -t 20:00:00 --mail-type ALL --job-name foshan 
# dsqa failedjobs.txt job_9361930_status.tsv > failedjobs2.txt 2> report2.txt
# dsq --job-file failedjobs2.txt -o /dev/null --mem-per-cpu 4g -t 20:00:00 --mail-type ALL --job-name foshan


##############################################
                 Ipoh_Malaysia
##############################################


dsq --job-file Ipoh_Malaysia.mpile.snpcln.October_13_2020.txt --output /dev/null --mem-per-cpu 5g -t 20:00:00 --mail-type ALL --job-name Ipoh_Malaysia 
dsqa Ipoh_Malaysia.mpile.snpcln.October_13_2020.txt job_10369819_status.tsv > failedjobs.txt 2> report.txt
# dsq --job-file failedjobs.txt -o /dev/null --mem-per-cpu 4g -t 20:00:00 --mail-type ALL --job-name foshan 
# dsqa failedjobs.txt job_9361930_status.tsv > failedjobs2.txt 2> report2.txt
# dsq --job-file failedjobs2.txt -o /dev/null --mem-per-cpu 4g -t 20:00:00 --mail-type ALL --job-name foshan


##############################################
                 Jacksonville_Florida_USA
##############################################


dsq --job-file Jacksonville_Florida_USA.mpile.snpcln.October_13_2020.txt --output /dev/null --mem-per-cpu 5g -t 20:00:00 --mail-type ALL --job-name Jacksonville_Florida_USA 
dsqa Jacksonville_Florida_USA.mpile.snpcln.October_13_2020.txt job_10369939_status.tsv > failedjobs.txt 2> report.txt
# dsq --job-file failedjobs.txt -o /dev/null --mem-per-cpu 4g -t 20:00:00 --mail-type ALL --job-name foshan 
# dsqa failedjobs.txt job_9361930_status.tsv > failedjobs2.txt 2> report2.txt
# dsq --job-file failedjobs2.txt -o /dev/null --mem-per-cpu 4g -t 20:00:00 --mail-type ALL --job-name foshan


##############################################
                 Jakarta_Indonesia
##############################################


dsq --job-file Jakarta_Indonesia.mpile.snpcln.October_13_2020.txt --output /dev/null --mem-per-cpu 5g -t 20:00:00 --mail-type ALL --job-name Jakarta_Indonesia 
dsqa Jakarta_Indonesia.mpile.snpcln.October_13_2020.txt job_10369961_status.tsv > failedjobs.txt 2> report.txt
# dsq --job-file failedjobs.txt -o /dev/null --mem-per-cpu 4g -t 20:00:00 --mail-type ALL --job-name foshan 
# dsqa failedjobs.txt job_9361930_status.tsv > failedjobs2.txt 2> report2.txt
# dsq --job-file failedjobs2.txt -o /dev/null --mem-per-cpu 4g -t 20:00:00 --mail-type ALL --job-name foshan


##############################################
                 Jardin_Panteon
##############################################


dsq --job-file Jardin_Panteon.mpile.snpcln.October_13_2020.txt --output /dev/null --mem-per-cpu 5g -t 20:00:00 --mail-type ALL --job-name Jardin_Panteon 
dsqa Jardin_Panteon.mpile.snpcln.October_13_2020.txt job_10369987_status.tsv > failedjobs.txt 2> report.txt
# dsq --job-file failedjobs.txt -o /dev/null --mem-per-cpu 4g -t 20:00:00 --mail-type ALL --job-name foshan 
# dsqa failedjobs.txt job_9361930_status.tsv > failedjobs2.txt 2> report2.txt
# dsq --job-file failedjobs2.txt -o /dev/null --mem-per-cpu 4g -t 20:00:00 --mail-type ALL --job-name foshan


##############################################
                 Kathmandu_Nepal
##############################################


dsq --job-file Kathmandu_Nepal.mpile.snpcln.October_13_2020.txt --output /dev/null --mem-per-cpu 5g -t 20:00:00 --mail-type ALL --job-name Kathmandu_Nepal 
dsqa Kathmandu_Nepal.mpile.snpcln.October_13_2020.txt job_10370052_status.tsv > failedjobs.txt 2> report.txt
# dsq --job-file failedjobs.txt -o /dev/null --mem-per-cpu 4g -t 20:00:00 --mail-type ALL --job-name foshan 
# dsqa failedjobs.txt job_9361930_status.tsv > failedjobs2.txt 2> report2.txt
# dsq --job-file failedjobs2.txt -o /dev/null --mem-per-cpu 4g -t 20:00:00 --mail-type ALL --job-name foshan


##############################################
                 Kin_Village_Okinawa_Japan
##############################################


dsq --job-file Kin_Village_Okinawa_Japan.mpile.snpcln.October_13_2020.txt --output /dev/null --mem-per-cpu 5g -t 20:00:00 --mail-type ALL --job-name Kin_Village_Okinawa_Japan 
dsqa Kin_Village_Okinawa_Japan.mpile.snpcln.October_13_2020.txt job_10370084_status.tsv > failedjobs.txt 2> report.txt
# dsq --job-file failedjobs.txt -o /dev/null --mem-per-cpu 4g -t 20:00:00 --mail-type ALL --job-name foshan 
# dsqa failedjobs.txt job_9361930_status.tsv > failedjobs2.txt 2> report2.txt
# dsq --job-file failedjobs2.txt -o /dev/null --mem-per-cpu 4g -t 20:00:00 --mail-type ALL --job-name foshan


##############################################
                 Kuala_Lumpur
##############################################


dsq --job-file Kuala_Lumpur.mpile.snpcln.October_13_2020.txt --output /dev/null --mem-per-cpu 5g -t 20:00:00 --mail-type ALL --job-name Kuala_Lumpur 
dsqa Kuala_Lumpur.mpile.snpcln.October_13_2020.txt job_10370117_status.tsv > failedjobs.txt 2> report.txt
# dsq --job-file failedjobs.txt -o /dev/null --mem-per-cpu 4g -t 20:00:00 --mail-type ALL --job-name foshan 
# dsqa failedjobs.txt job_9361930_status.tsv > failedjobs2.txt 2> report2.txt
# dsq --job-file failedjobs2.txt -o /dev/null --mem-per-cpu 4g -t 20:00:00 --mail-type ALL --job-name foshan


##############################################
                 Limbe_Cameroon
##############################################


dsq --job-file Limbe_Cameroon.mpile.snpcln.October_13_2020.txt --output /dev/null --mem-per-cpu 5g -t 20:00:00 --mail-type ALL --job-name Limbe_Cameroon 
dsqa Limbe_Cameroon.mpile.snpcln.October_13_2020.txt job_10370139_status.tsv > failedjobs.txt 2> report.txt
# dsq --job-file failedjobs.txt -o /dev/null --mem-per-cpu 4g -t 20:00:00 --mail-type ALL --job-name foshan 
# dsqa failedjobs.txt job_9361930_status.tsv > failedjobs2.txt 2> report2.txt
# dsq --job-file failedjobs2.txt -o /dev/null --mem-per-cpu 4g -t 20:00:00 --mail-type ALL --job-name foshan


##############################################
                 LosAngeles_USA
##############################################


dsq --job-file LosAngeles_USA.mpile.snpcln.October_13_2020.txt --output /dev/null --mem-per-cpu 5g -t 20:00:00 --mail-type ALL --job-name LosAngeles_USA 
dsqa LosAngeles_USA.mpile.snpcln.October_13_2020.txt job_10370160_status.tsv > failedjobs.txt 2> report.txt
# dsq --job-file failedjobs.txt -o /dev/null --mem-per-cpu 4g -t 20:00:00 --mail-type ALL --job-name foshan 
# dsqa failedjobs.txt job_9361930_status.tsv > failedjobs2.txt 2> report2.txt
# dsq --job-file failedjobs2.txt -o /dev/null --mem-per-cpu 4g -t 20:00:00 --mail-type ALL --job-name foshan


##############################################
                 Maceio_Brazil
##############################################


dsq --job-file Maceio_Brazil.mpile.snpcln.October_13_2020.txt --output /dev/null --mem-per-cpu 5g -t 20:00:00 --mail-type ALL --job-name Maceio_Brazil 
dsqa Maceio_Brazil.mpile.snpcln.October_13_2020.txt job_10370193_status.tsv > failedjobs.txt 2> report.txt
# dsq --job-file failedjobs.txt -o /dev/null --mem-per-cpu 4g -t 20:00:00 --mail-type ALL --job-name foshan 
# dsqa failedjobs.txt job_9361930_status.tsv > failedjobs2.txt 2> report2.txt
# dsq --job-file failedjobs2.txt -o /dev/null --mem-per-cpu 4g -t 20:00:00 --mail-type ALL --job-name foshan


##############################################
                 Manassas_VA_USA
##############################################


dsq --job-file Manassas_VA_USA.mpile.snpcln.October_13_2020.txt --output /dev/null --mem-per-cpu 5g -t 20:00:00 --mail-type ALL --job-name Manassas_VA_USA 
dsqa Manassas_VA_USA.mpile.snpcln.October_13_2020.txt job_10370223_status.tsv > failedjobs.txt 2> report.txt
# dsq --job-file failedjobs.txt -o /dev/null --mem-per-cpu 4g -t 20:00:00 --mail-type ALL --job-name foshan 
# dsqa failedjobs.txt job_9361930_status.tsv > failedjobs2.txt 2> report2.txt
# dsq --job-file failedjobs2.txt -o /dev/null --mem-per-cpu 4g -t 20:00:00 --mail-type ALL --job-name foshan


##############################################
                 Montpelier_France
##############################################


dsq --job-file Montpelier_France.mpile.snpcln.October_14_2020.txt --output /dev/null --mem-per-cpu 5g -t 20:00:00 --mail-type ALL --job-name Montpelier_France 
dsqa Montpelier_France.mpile.snpcln.October_14_2020.txt job_10378126_status.tsv > failedjobs.txt 2> report.txt
# dsq --job-file failedjobs.txt -o /dev/null --mem-per-cpu 4g -t 20:00:00 --mail-type ALL --job-name foshan 
# dsqa failedjobs.txt job_9361930_status.tsv > failedjobs2.txt 2> report2.txt
# dsq --job-file failedjobs2.txt -o /dev/null --mem-per-cpu 4g -t 20:00:00 --mail-type ALL --job-name foshan


##############################################
                 New_Zion_South_Carolina_USA
##############################################


dsq --job-file New_Zion_South_Carolina_USA.mpile.snpcln.October_14_2020.txt --output /dev/null --mem-per-cpu 5g -t 20:00:00 --mail-type ALL --job-name New_Zion_South_Carolina_USA 
dsqa New_Zion_South_Carolina_USA.mpile.snpcln.October_14_2020.txt job_10378447_status.tsv > failedjobs.txt 2> report.txt
# dsq --job-file failedjobs.txt -o /dev/null --mem-per-cpu 4g -t 20:00:00 --mail-type ALL --job-name foshan 
# dsqa failedjobs.txt job_9361930_status.tsv > failedjobs2.txt 2> report2.txt
# dsq --job-file failedjobs2.txt -o /dev/null --mem-per-cpu 4g -t 20:00:00 --mail-type ALL --job-name foshan


##############################################
                 Oak_Hill_Florida_USA
##############################################


dsq --job-file Oak_Hill_Florida_USA.mpile.snpcln.October_14_2020.txt --output /dev/null --mem-per-cpu 5g -t 20:00:00 --mail-type ALL --job-name Oak_Hill_Florida_USA 
dsqa Oak_Hill_Florida_USA.mpile.snpcln.October_14_2020.txt job_10378491_status.tsv > failedjobs.txt 2> report.txt
# dsq --job-file failedjobs.txt -o /dev/null --mem-per-cpu 4g -t 20:00:00 --mail-type ALL --job-name foshan 
# dsqa failedjobs.txt job_9361930_status.tsv > failedjobs2.txt 2> report2.txt
# dsq --job-file failedjobs2.txt -o /dev/null --mem-per-cpu 4g -t 20:00:00 --mail-type ALL --job-name foshan


##############################################
                 Pahokee_FL_USA
##############################################


dsq --job-file Pahokee_FL_USA.mpile.snpcln.October_14_2020.txt --output /dev/null --mem-per-cpu 5g -t 20:00:00 --mail-type ALL --job-name Pahokee_FL_USA 
dsqa Pahokee_FL_USA.mpile.snpcln.October_14_2020.txt job_10378543_status.tsv > failedjobs.txt 2> report.txt
# dsq --job-file failedjobs.txt -o /dev/null --mem-per-cpu 4g -t 20:00:00 --mail-type ALL --job-name foshan 
# dsqa failedjobs.txt job_9361930_status.tsv > failedjobs2.txt 2> report2.txt
# dsq --job-file failedjobs2.txt -o /dev/null --mem-per-cpu 4g -t 20:00:00 --mail-type ALL --job-name foshan


##############################################
                 Palm_Beach_Florida_USA
##############################################


dsq --job-file Palm_Beach_Florida_USA.mpile.snpcln.October_14_2020.txt --output /dev/null --mem-per-cpu 5g -t 20:00:00 --mail-type ALL --job-name Palm_Beach_Florida_USA 
dsqa Palm_Beach_Florida_USA.mpile.snpcln.October_14_2020.txt job_10378560_status.tsv > failedjobs.txt 2> report.txt
# dsq --job-file failedjobs.txt -o /dev/null --mem-per-cpu 4g -t 20:00:00 --mail-type ALL --job-name foshan 
# dsqa failedjobs.txt job_9361930_status.tsv > failedjobs2.txt 2> report2.txt
# dsq --job-file failedjobs2.txt -o /dev/null --mem-per-cpu 4g -t 20:00:00 --mail-type ALL --job-name foshan


##############################################
                 Papua_Indonesia
##############################################


dsq --job-file Papua_Indonesia.mpile.snpcln.October_14_2020.txt --output /dev/null --mem-per-cpu 5g -t 20:00:00 --mail-type ALL --job-name Papua_Indonesia 
dsqa Papua_Indonesia.mpile.snpcln.October_14_2020.txt job_10378614_status.tsv > failedjobs.txt 2> report.txt
# dsq --job-file failedjobs.txt -o /dev/null --mem-per-cpu 4g -t 20:00:00 --mail-type ALL --job-name foshan 
# dsqa failedjobs.txt job_9361930_status.tsv > failedjobs2.txt 2> report2.txt
# dsq --job-file failedjobs2.txt -o /dev/null --mem-per-cpu 4g -t 20:00:00 --mail-type ALL --job-name foshan


##############################################
                 Prto_Igu_Msns_Argentina
##############################################


dsq --job-file Prto_Igu_Msns_Argentina.mpile.snpcln.October_14_2020.txt --output /dev/null --mem-per-cpu 5g -t 20:00:00 --mail-type ALL --job-name Prto_Igu_Msns_Argentina 
dsqa Prto_Igu_Msns_Argentina.mpile.snpcln.October_14_2020.txt job_10378656_status.tsv > failedjobs.txt 2> report.txt
# dsq --job-file failedjobs.txt -o /dev/null --mem-per-cpu 4g -t 20:00:00 --mail-type ALL --job-name foshan 
# dsqa failedjobs.txt job_9361930_status.tsv > failedjobs2.txt 2> report2.txt
# dsq --job-file failedjobs2.txt -o /dev/null --mem-per-cpu 4g -t 20:00:00 --mail-type ALL --job-name foshan


##############################################
                 Salem_New_Jersey_USA
##############################################


dsq --job-file Salem_New_Jersey_USA.mpile.snpcln.October_14_2020.txt --output /dev/null --mem-per-cpu 5g -t 20:00:00 --mail-type ALL --job-name Salem_New_Jersey_USA 
dsqa Salem_New_Jersey_USA.mpile.snpcln.October_14_2020.txt job_10378690_status.tsv > failedjobs.txt 2> report.txt
# dsq --job-file failedjobs.txt -o /dev/null --mem-per-cpu 4g -t 20:00:00 --mail-type ALL --job-name foshan 
# dsqa failedjobs.txt job_9361930_status.tsv > failedjobs2.txt 2> report2.txt
# dsq --job-file failedjobs2.txt -o /dev/null --mem-per-cpu 4g -t 20:00:00 --mail-type ALL --job-name foshan


##############################################
                 Sendai_Japan
##############################################


dsq --job-file Sendai_Japan.mpile.snpcln.November_17_2020.txt --output /dev/null --mem-per-cpu 5g -t 20:00:00 --mail-type ALL --job-name Sendai_Japan 
dsqa Sendai_Japan.mpile.snpcln.November_17_2020.txt job_10503588_status.tsv > failedjobs.txt 2> report.txt
# dsq --job-file failedjobs.txt -o /dev/null --mem-per-cpu 4g -t 20:00:00 --mail-type ALL --job-name foshan 
# dsqa failedjobs.txt job_9361930_status.tsv > failedjobs2.txt 2> report2.txt
# dsq --job-file failedjobs2.txt -o /dev/null --mem-per-cpu 4g -t 20:00:00 --mail-type ALL --job-name foshan


##############################################
                 Shanghai_China
##############################################


dsq --job-file Shanghai_China.mpile.snpcln.October_16_2020.txt --output /dev/null --mem-per-cpu 5g -t 20:00:00 --mail-type ALL --job-name Shanghai_China 
dsqa Shanghai_China.mpile.snpcln.October_16_2020.txt job_10388110_status.tsv > failedjobs.txt 2> report.txt
# dsq --job-file failedjobs.txt -o /dev/null --mem-per-cpu 4g -t 20:00:00 --mail-type ALL --job-name foshan 
# dsqa failedjobs.txt job_9361930_status.tsv > failedjobs2.txt 2> report2.txt
# dsq --job-file failedjobs2.txt -o /dev/null --mem-per-cpu 4g -t 20:00:00 --mail-type ALL --job-name foshan


##############################################
                 StAugustine_Trinidad
##############################################


dsq --job-file StAugustine_Trinidad.mpile.snpcln.October_16_2020.txt --output /dev/null --mem-per-cpu 5g -t 20:00:00 --mail-type ALL --job-name StAugustine_Trinidad 
dsqa StAugustine_Trinidad.mpile.snpcln.October_16_2020.txt job_10387744_status.tsv > failedjobs.txt 2> report.txt
# dsq --job-file failedjobs.txt -o /dev/null --mem-per-cpu 4g -t 20:00:00 --mail-type ALL --job-name foshan 
# dsqa failedjobs.txt job_9361930_status.tsv > failedjobs2.txt 2> report2.txt
# dsq --job-file failedjobs2.txt -o /dev/null --mem-per-cpu 4g -t 20:00:00 --mail-type ALL --job-name foshan


##############################################
                 Sumba_Indonesia
##############################################


dsq --job-file Sumba_Indonesia.mpile.snpcln.October_16_2020.txt --output /dev/null --mem-per-cpu 5g -t 20:00:00 --mail-type ALL --job-name Sumba_Indonesia 
dsqa Sumba_Indonesia.mpile.snpcln.October_16_2020.txt job_10388809_status.tsv > failedjobs.txt 2> report.txt
# dsq --job-file failedjobs.txt -o /dev/null --mem-per-cpu 4g -t 20:00:00 --mail-type ALL --job-name foshan 
# dsqa failedjobs.txt job_9361930_status.tsv > failedjobs2.txt 2> report2.txt
# dsq --job-file failedjobs2.txt -o /dev/null --mem-per-cpu 4g -t 20:00:00 --mail-type ALL --job-name foshan


##############################################
                 Tampon
##############################################


dsq --job-file Tampon.mpile.snpcln.October_16_2020.txt --output /dev/null --mem-per-cpu 5g -t 20:00:00 --mail-type ALL --job-name Tampon 
dsqa Tampon.mpile.snpcln.October_16_2020.txt job_10388978_status.tsv > failedjobs.txt 2> report.txt
# dsq --job-file failedjobs.txt -o /dev/null --mem-per-cpu 4g -t 20:00:00 --mail-type ALL --job-name foshan 
# dsqa failedjobs.txt job_9361930_status.tsv > failedjobs2.txt 2> report2.txt
# dsq --job-file failedjobs2.txt -o /dev/null --mem-per-cpu 4g -t 20:00:00 --mail-type ALL --job-name foshan


##############################################
                 Tanegashima_Japan
##############################################


dsq --job-file Tanegashima_Japan.mpile.snpcln.October_16_2020.txt --output /dev/null --mem-per-cpu 5g -t 20:00:00 --mail-type ALL --job-name Tanegashima_Japan 
dsqa Tanegashima_Japan.mpile.snpcln.October_16_2020.txt job_10389175_status.tsv > failedjobs.txt 2> report.txt
# dsq --job-file failedjobs.txt -o /dev/null --mem-per-cpu 4g -t 20:00:00 --mail-type ALL --job-name foshan 
# dsqa failedjobs.txt job_9361930_status.tsv > failedjobs2.txt 2> report2.txt
# dsq --job-file failedjobs2.txt -o /dev/null --mem-per-cpu 4g -t 20:00:00 --mail-type ALL --job-name foshan


##############################################
                 Tokyo_Japan
##############################################


dsq --job-file Tokyo_Japan.mpile.snpcln.October_16_2020.txt --output /dev/null --mem-per-cpu 5g -t 20:00:00 --mail-type ALL --job-name Tokyo_Japan 
dsqa Tokyo_Japan.mpile.snpcln.October_16_2020.txt job_10389216_status.tsv > failedjobsTokyo_Japan.txt 2> report.txt
# dsq --job-file failedjobs.txt -o /dev/null --mem-per-cpu 4g -t 20:00:00 --mail-type ALL --job-name foshan 
# dsqa failedjobs.txt job_9361930_status.tsv > failedjobs2.txt 2> report2.txt
# dsq --job-file failedjobs2.txt -o /dev/null --mem-per-cpu 4g -t 20:00:00 --mail-type ALL --job-name foshan


##############################################
                 Vero_Beach_FL_USA
##############################################


dsq --job-file Vero_Beach_FL_USA.mpile.snpcln.October_16_2020.txt --output /dev/null --mem-per-cpu 5g -t 20:00:00 --mail-type ALL --job-name Vero_Beach_FL_USA 
dsqa Vero_Beach_FL_USA.mpile.snpcln.October_16_2020.txt job_10389432_status.tsv > failedjobsVeroBeach.txt 2> report.txt
# dsq --job-file failedjobs.txt -o /dev/null --mem-per-cpu 4g -t 20:00:00 --mail-type ALL --job-name foshan 
# dsqa failedjobs.txt job_9361930_status.tsv > failedjobs2.txt 2> report2.txt
# dsq --job-file failedjobs2.txt -o /dev/null --mem-per-cpu 4g -t 20:00:00 --mail-type ALL --job-name foshan


##############################################
                 Wajima_Japan
##############################################


dsq --job-file Wajima_Japan.mpile.snpcln.October_19_2020.txt --output /dev/null --mem-per-cpu 5g -t 20:00:00 --mail-type ALL --job-name Wajima_Japan 
dsqa Wajima_Japan.mpile.snpcln.October_19_2020.txt job_10398951_status.tsv > failedjobsWajima_Japan.txt 2> report.txt
#dsq --job-file failedjobsWajima_Japan.txt -o /dev/null --mem-per-cpu 5g -t 20:00:00 --mail-type ALL --job-name Wajima_Japan2 
#dsqa failedjobsWajima_Japan.txt job_10396375_status.tsv > failedjobs2.txt 2> report2.txt
# dsq --job-file failedjobs2.txt -o /dev/null --mem-per-cpu 4g -t 20:00:00 --mail-type ALL --job-name foshan


##############################################
                 Yunnan_China
##############################################


dsq --job-file Yunnan_China.mpile.snpcln.October_19_2020.txt --output /dev/null --mem-per-cpu 5g -t 20:00:00 --mail-type ALL --job-name Yunnan_China 
dsqa Yunnan_China.mpile.snpcln.October_19_2020.txt job_10399257_status.tsv > failedjobsYunnan_China.txt 2> report.txt
#dsq --job-file failedjobs.txt -o /dev/null --mem-per-cpu 5g -t 20:00:00 --mail-type ALL --job-name Yunnan_China2 
#dsqa failedjobs.txt job_10396305_status.tsv > failedjobs2.txt 2> report.txt
# dsq --job-file failedjobs2.txt -o /dev/null --mem-per-cpu 4g -t 20:00:00 --mail-type ALL --job-name foshan


##############################################
                 Foshan
##############################################


dsq --job-file Foshan.mpile.snpcln.October_20_2020.txt --output /dev/null --mem-per-cpu 6g -t 20:00:00 --mail-type ALL --job-name Foshan 
dsqa Foshan.mpile.snpcln.October_20_2020.txt job_10406738_status.tsv > failedjobsFoshan.txt 2> report.txt
dsq --job-file failedjobsFoshan.txt -o /dev/null --mem-per-cpu 10g -t 20:00:00 --mail-type ALL --job-name foshan2 
dsqa failedjobsFoshan.txt job_10408871_status.tsv > failedjobsFoshan2.txt 2> report.txt
#dsq --job-file failedjobsFoshan2.txt -o /dev/null --mem-per-cpu 20g -t 120:00:00 --mail-type ALL --job-name foshan3
#dsqa failedjobsFoshan2.txt job_10404753_status.tsv > failedjobsFoshan2.txt 2> report.txt


##############################################
                 Torres_Strait_Islands
##############################################


dsq --job-file Torres_Strait_Islands.mpile.snpcln.October_20_2020.txt --output /dev/null --mem-per-cpu 10g -t 20:00:00 --mail-type ALL --job-name Torres_Strait_Islands 
dsqa Torres_Strait_Islands.mpile.snpcln.October_20_2020.txt job_10407048_status.tsv > failedjobsTorres_Strait_Islands.txt 2> report.txt
dsq --job-file failedjobsTorres_Strait_Islands.txt -o /dev/null --mem-per-cpu 10g -t 20:00:00 --mail-type ALL --job-name Torres_Strait_Islands2
dsqa failedjobsTorres_Strait_Islands.txt job_10409688_status.tsv > Torres_Strait_IslandsFailed2.txt 2> report.txt
#dsq --job-file Torres_Strait_IslandsFailed2.txt -o /dev/null --mem-per-cpu 20g -t 120:00:00 --mail-type ALL --job-name Torres_Strait_Islands
#dsqa Torres_Strait_IslandsFailed2.txt job_10404749_status.tsv > Torres_Strait_IslandsFailed3.txt 2> report.txt


##############################################
                 Cali_Colombia
##############################################


dsq --job-file Cali_Colombia.mpile.snpcln.December_1_2020.txt --output /dev/null --mem-per-cpu 5g -t 20:00:00 --mail-type ALL --job-name Cali_Colombia 
dsqa Cali_Colombia.mpile.snpcln.December_1_2020.txt job_10559838_status.tsv > failedjobs.txt 2> report.txt

##############################################
                 Gelephu_Bhutan
##############################################


dsq --job-file Gelephu_Bhutan.mpile.snpcln.December_1_2020.txt --output /dev/null --mem-per-cpu 5g -t 20:00:00 --mail-type ALL --job-name Gelephu_Bhutan 
dsqa Gelephu_Bhutan.mpile.snpcln.December_1_2020.txt job_10560143_status.tsv > failedjobs.txt 2> report.txt


##############################################
                 Jaffna_SriLanka
##############################################


dsq --job-file Jaffna_SriLanka.mpile.snpcln.December_1_2020.txt --output /dev/null --mem-per-cpu 5g -t 20:00:00 --mail-type ALL --job-name Jaffna_SriLanka 
dsqa Jaffna_SriLanka.mpile.snpcln.December_1_2020.txt job_10560263_status.tsv > failedjobs.txt 2> report.txt


##############################################
                 Springfield_MO_USA
##############################################


dsq --job-file Springfield_MO_USA.mpile.snpcln.December_1_2020.txt --output /dev/null --mem-per-cpu 5g -t 20:00:00 --mail-type ALL --job-name Springfield_MO_USA 
dsqa Springfield_MO_USA.mpile.snpcln.December_1_2020.txt job_10560285_status.tsv > failedjobs.txt 2> report.txt


##############################################
                 New_Orleans
##############################################


dsq --job-file New_Orleans.mpile.snpcln.December_1_2020.txt --output /dev/null --mem-per-cpu 5g -t 20:00:00 --mail-type ALL --job-name New_Orleans 
dsqa New_Orleans.mpile.snpcln.December_1_2020.txt job_10560316_status.tsv > failedjobs.txt 2> report.txt


##############################################
                 Tainan_Taiwan
##############################################
dsq --job-file Tainan_Taiwan.mpile.snpcln.December_1_2020.txt --output /dev/null --mem-per-cpu 5g -t 20:00:00 --mail-type ALL --job-name Tainan_Taiwan 
dsqa Tainan_Taiwan.mpile.snpcln.December_1_2020.txt job_10560349_status.tsv > failedjobs.txt 2> report.txt


##############################################
                 Imperia_Italy
##############################################


dsq --job-file Imperia_Italy.mpile.snpcln.December_5_2020.txt --output /dev/null --mem-per-cpu 5g -t 20:00:00 --mail-type ALL --job-name Imperia_Italy 
dsqa Imperia_Italy.mpile.snpcln.December_5_2020.txt job_10575997_status.tsv > failedjobs.txt 2> report.txt
```

## 9. Get sites for genotype calls

In this step I concatenate the sites from the snpCleaner pipeline.
Below is an a code used for one population.

```
# get interactive session
# srun --pty -t 24:00:00 -c 4 --mem=10G -p interactive bash

# load Anaconda
# module load Anaconda2/4.2.0

#concatenate files
from path import Path
d = Path('/ycga-gpfs/project/caccone/lvc26/september_2020/sites/Tainan_Taiwan/sites/keeps/')
with open('/ycga-gpfs/project/caccone/lvc26/september_2020/sites/cat/new_pops/Tainan_Taiwan.txt', 'w') as f_out:
    for file_name in d.walk('*.sites'):
        with open(file_name) as f_in:
            data = f_in.read()
            f_out.write(data)
```

Next I sorted the sites files. I created a python script to sort the
sites files since some had over a billion lines.

Example of batch script to sort files

```
#!/bin/bash
#SBATCH --ntasks=1
#SBATCH --cpus-per-task=1           
#SBATCH --mem-per-cpu=70gb       
#SBATCH --time=120:00:00                         
#SBATCH --job-name=sort
#SBATCH -o sort.%A_%a.sort.o.csv
#SBATCH -e sort.%A_%a.sort.ERROR.csv
#SBATCH --mail-type ALL

module load Anaconda2/4.2.0

#cd /ycga-gpfs/project/caccone/lvc26/september_2020/sites/cat/new_pops/
cd /gpfs/ycga/project/caccone/lvc26/september_2020/sites/cat/intersects/shared
python sort2.py
```

The python script

```
import os
import glob
import pandas as pd

files = glob.glob('/gpfs/ycga/project/caccone/lvc26/september_2020/sites/cat/intersects/shared/*.txt')
for file in files:
    df = pd.read_csv(file, sep='\t', skipinitialspace=True, header=None) # read file
    df.sort_values(by=[0], inplace=True) # sort file
    df.to_csv(file + '_sorted.txt',  index=None, header=None, sep='\t') # write file
```

The next step was to get an intersection of sites that had good read
coverage across all populations. I used a python script to get the
intersection.

Example of a batch script submitted. It needed a lot of memory
because files had around a billion lines. These sites files are just a
text file with two columns. One is the scaffold and the other is the
position. We use it later with angsd to do the genotype calls.

```
#!/bin/bash
#SBATCH --partition=scavenge
#SBATCH --ntasks=1
#SBATCH --cpus-per-task=1           
#SBATCH --mem-per-cpu=200gb       
#SBATCH --time=120:00:00                         
#SBATCH --job-name=sort
#SBATCH -o intersection_12.%A_%a.intersection_12.o.log
#SBATCH -e intersection_12.%A_%a.sort.intersection_12.log
#SBATCH --mail-type ALL

module load Anaconda2/4.2.0

cd /gpfs/ycga/project/caccone/lvc26/september_2020/sites/cat

python intersection.py
```

The python script

```
import glob

files = glob.glob("*.txt")
combine = set(open(files[0]))

print("Warning: assumes that here is a new line at the end of every file")
print("finding intersection of:")

for file in files:
    print(str(file))
    fp = open(file)
    combine = combine & set(fp)
    fp.close()

with open('results.txt', 'w+') as f:
    for item in combine:
        f.write("%s" % item)
```

## 10. Genotype calls using the sites files

Once I obtained the sites that we had good read coverage across all
populations, I indexed it with angsd and did a genotype call, exporting
as tped format.

```
#!/bin/sh
#SBATCH --mail-type=BEGIN,END,FAIL          
#SBATCH --mail-user=luciano.cosme@yale.edu 
#SBATCH --ntasks=1
#SBATCH --cpus-per-task=20          
#SBATCH --mem-per-cpu=6gb                  
#SBATCH --time=120:00:00 
#SBATCH --array=1-819
#SBATCH --job-name=angsd_chr
#SBATCH -o angsd_chr.%A_%a.o.txt
#SBATCH -e angsd_chr.%A_%a.ERROR.txt

cd /gpfs/ycga/project/caccone/lvc26/september_2020/snp_calls/chunk_calls

samplesheet="scaffolds.txt"

threads=$SLURM_JOB_CPUS_PER_NODE

name=`sed -n "$SLURM_ARRAY_TASK_ID"p $samplesheet |  awk '{print $1}'`
####r1=`sed -n "$SLURM_ARRAY_TASK_ID"p $samplesheet |  awk '{print $2}'`

/home/lvc26/project/angsd/angsd \
-ref /gpfs/ycga/project/caccone/lvc26/september_2020/genome/aedes_albopictus_LA2_20200826.fasta \
-bam /gpfs/ycga/project/caccone/lvc26/september_2020/snp_calls/bams.txt \
-nThreads 40 \
-r $name \
-gl 1 \
-dopost 1 \
-doMaf 2 \
-doMajorMinor 4 \
-minMapQ 20 \
-minQ 10 \
-remove_bads 1 \
-uniqueOnly 1 \
-sites /gpfs/ycga/project/caccone/lvc26/september_2020/sites/cat/intersects/shared/shared_sites.txt \
-doCounts 1 \
-setMinDepthInd 10 \
-minInd 2 \
-SNP_pval 1e-6 \
-doPlink 2 \
-doGeno 4 \
-capDepth 45 \
-minMaf 0.01 \
-out $name
```

Then I merged the files

```
#!/bin/sh
#SBATCH --partition=scavenge
#SBATCH --mail-type=BEGIN,END,FAIL          
#SBATCH --mail-user=luciano.cosme@yale.edu 
#SBATCH --ntasks=1
#SBATCH --cpus-per-task=10          
#SBATCH --mem-per-cpu=100gb                  
#SBATCH --time=120:00:00 
#SBATCH --job-name=plink_merge
#SBATCH -o plink_merge.%A_%a.o.txt
#SBATCH -e plink_merge.%A_%a.ERROR.txt

module load PLINK/1.90-beta4.4

cd /gpfs/ycga/project/caccone/lvc26/september_2020/snp_calls/chunk_calls/plink
plink --merge-list bed_files.txt --allow-extra-chr --make-bed --out merged --memory 1000000
```
