# Supplementary material for: A genotyping array for the globally invasive vector mosquito, Aedes albopictus
Source: Parasit Vectors. 2024 Mar 4;17:106. doi: 10.1186/s13071-024-06158-z (PMC10910840; doi:10.1186/s13071-024-06158-z)
Supplement: Supplementary file 2 — Additional file 2. Mapping probes to reference genomes. [file 13071_2024_6158_MOESM2_ESM.html]

Aedes albopictus SNP chip: Mapping probe sequences to reference genomes


# Aedes albopictus SNP chip: Mapping probe sequences to reference genomes

#### Luciano V Cosme

#### 2023-08-23

- Overview
  - 1. R libraries and software
  - 2. Generate sequences to map
  - 3. The
    genome sequences
    - 3.1 AalbF1\_ncbi
    - 3.2 AalbF1\_vb
    - 3.3 AalbF2\_ncbi
    - 3.4 AalbF2\_vb
    - 3.5 AalbF3 - used for chip
      design
    - 3.6
      AalbCell\_ncbi
    - 3.7 AalbCell\_vb
    - 3.5 AalbRamini
    - 3.6.
      Aedes aegypti genome
      - 3.6.1
        AaegL5\_ncbi
      - 3.6.2 AaegL5\_vb
  - 4. Mapping sequences on the
    HPC
    - 4.1 Transfer genomes file to
      cluster
    - 4.2 Transfer sequences file
      to cluster
    - 4.3 Index the
      reference genomes with BWA and bowtie2
      - 4.3.1 BWA index
      - 4.2.2 Bowtie
        index
  - 5. Mapping
    probe sequences with BWA on the cluster
    - 5.1 Map with the
      default bwa mem parameters
      - 5.1.1 Inspect the alignments
        - 5.1.1.1 AalbF3
        - 5.1.1.2 AalbF2
      - 5.1.2 Import bwa mem summary
        results
    - 5.2 Map with the
      default bwa aln parameters
      - 5.1.2 Import bwa aln summary
        results
    - 5.3 Select SNPs
      for genotype call with Axiom Suite
      - 5.3.1
        AalbF2 unique alignments and no mismatches for sequences with reference
        allele
      - 5.3.2
        AalbF3 unique alignments and no mismatches for sequences with reference
        allele
      - 5.3.3 Create Venn diagram
        AalbF3
      - 5.3.4 Create Venn diagram
        AalbF2
      - 5.3.5 Create Venn diagram
        AalbF2 and AalbF3
      - 5.3.6
        AalbF2 unique alignments and one mismatches for sequences with reference
        allele
      - 5.3.7
        AalbF3 unique alignments and one mismatches for sequences with reference
        allele

# Overview

In this RMarkdown we will overview the probe sequences from the Aedes
albopictus SNP chip, generate sequences for mapping with BWA and parse
the output of the mapping.

## 1. R libraries and software

```
library(tidyverse)
library(colorout)
library(here)
library(ggplot2)
library(Biostrings)
library(scales)
library(reticulate)
library(flextable)
library(officer)
library(rmdformats)
library(extrafont)
library(forcats)
library(ggrepel)
library(ggvenn)
```

## 2. Generate sequences to map

Clear our environment and memory

```
# Clear global environment
rm(list = ls())
# Clear memory using garbage collection
gc()
```

```
##           used  (Mb) gc trigger  (Mb) limit (Mb) max used  (Mb)
## Ncells 4407398 235.4    7463438 398.6         NA  7370225 393.7
## Vcells 7486949  57.2   12255594  93.6      32768 10146329  77.5
```

Import probe information

```
# import the data
probes_albopictus <-
  read_delim(
    here(
      "data", "probes", "tilled.csv"
    ),
    delim = ",",
    col_names = TRUE,
    show_col_types = FALSE
  ) |>
  mutate(
    Scaffold = str_replace(cust_chr, "chr", "")
  )

# check it out
head(probes_albopictus)
```

```
## # A tibble: 6 × 24
##   ReporterKey `Affx ID`       Name  Sequence SNPType ProbeCount  Tile Replicates
##         <dbl> <chr>           <chr> <chr>    <chr>        <dbl> <dbl>      <dbl>
## 1   536823060 Affx-1249419468 AX-5… CCACCCA… Y                2     1          2
## 2   535746209 Affx-1248904337 AX-5… CCAAAGG… W                4     1          2
## 3   537507869 Affx-1249761675 AX-5… CAAAATG… Y                2     1          2
## 4   533540163 Affx-1250822361 AX-5… CCTTCTG… W                4     1          2
## 5   536958410 Affx-1249488406 AX-5… ATAAGTT… R                2     1          2
## 6   537661563 Affx-1249840192 AX-5… GCATGAT… W                4     1          2
## # ℹ 16 more variables: DesignNumber <dbl>, Rank <dbl>, Strand <chr>,
## #   SetBack <lgl>, taxonomy_key <dbl>, organism <chr>, cust_id <chr>,
## #   cust_chr <chr>, cust_pos <dbl>, cust_ref <chr>, cust_alt <chr>,
## #   importance <chr>, pconvert <dbl>, recommendation <chr>, Error <lgl>,
## #   Scaffold <chr>
```

Function to get the reverse complement of the sequences that are 3’
to 5’

```
# create function to get the reverse complement
reverse_complement_vector <- function(dna_seqs) {
  DNAStringSet(dna_seqs) |> reverseComplement() |> as.character()
}

# save the function to source it later
dump(
  "reverse_complement_vector",
  here(
    "scripts", "analysis", "reverse_complement_vector.R")
)
```

Generage the sequences for mapping

```
# split the sequence into probe_left and probe_right based on the strand column
albopictus_seq <- 
  probes_albopictus |>
  mutate(
    prob_5a = ifelse(
      Strand == "f", gsub("\\[.*", "", Sequence), ""
    ),
    prob_5b = ifelse(
      Strand == "f", gsub(".*\\]", "", Sequence), ""
    ),
    prob_3b = ifelse(
      Strand == "r", gsub("\\[.*", "", Sequence), ""
    ),
    prob_3a = ifelse(
      Strand == "r", gsub(".*\\]", "", Sequence), ""
    ),
    prob_3b = reverse_complement_vector(prob_3b),
    prob_3a = reverse_complement_vector(prob_3a)
  ) |>
  unite(probe_left, prob_5a, prob_3a, sep = "") |>
  unite(probe_right, prob_5b, prob_3b, sep = "")

# add the alleles to each probe sequence
albopictus_seq_01 <- 
  albopictus_seq |>
  mutate(
    refA_forwad_strand = ifelse(Strand == "r", reverse_complement_vector(cust_ref), cust_ref),
    probe_left_ref = paste0(probe_left, refA_forwad_strand),
    
    altA_forwad_strand = ifelse(Strand == "r", reverse_complement_vector(cust_alt), cust_alt),
    probe_left_alt = paste0(probe_left, altA_forwad_strand),
    
    refA_forwad_strand = ifelse(Strand == "r", reverse_complement_vector(cust_ref), cust_ref),
    probe_right_ref = paste0(refA_forwad_strand, probe_right),
    
    altA_forwad_strand = ifelse(Strand == "r", reverse_complement_vector(cust_alt), cust_alt),
    probe_right_alt = paste0(altA_forwad_strand, probe_right)
  )

# create 71bp sequences
albopictus_seq_02 <-
  albopictus_seq_01 |>
  mutate(
    ref_allele_F = paste0(probe_left, refA_forwad_strand, probe_right),
    alt_allele_F = paste0(probe_left, altA_forwad_strand, probe_right),
    N_allele_F = paste0(probe_left, "N", probe_right)
  ) |>
  mutate(
    ref_allele_R = reverse_complement_vector(ref_allele_F),
    alt_allele_R = reverse_complement_vector(alt_allele_F),
    N_allele_R = reverse_complement_vector(N_allele_F)
  ) |>
  select(
    Name, Strand, Scaffold, cust_pos, cust_ref, cust_alt, ref_allele_F, ref_allele_R, alt_allele_F, alt_allele_R, N_allele_F, N_allele_R
  )

# create sequence names
albopictus_seq_02 <-
  albopictus_seq_02 |>
  mutate( # can can generate the name with only the SNP and the direction of the sequences (F for 5' to 3' and R for 3' to 5', this is independent of the target strand)
    fasta_name_F = paste0(">", Name, "_F"),
    fasta_name_R = paste0(">", Name, "_R")
  )

head(albopictus_seq_02)
```

```
## # A tibble: 6 × 14
##   Name      Strand Scaffold cust_pos cust_ref cust_alt ref_allele_F ref_allele_R
##   <chr>     <chr>  <chr>       <dbl> <chr>    <chr>    <chr>        <chr>       
## 1 AX-58338… r      1.144     5714162 T        C        TTTATGTGCAT… CCACCCAGTTG…
## 2 AX-58232… f      3.70      2807204 T        A        CCAAAGGATAT… TGTTTAACTGA…
## 3 AX-58407… r      1.85     22130239 C        T        CAAACATACGT… CAAAATGTTGA…
## 4 AX-58015… r      2.87     30095490 T        A        AAACATGTATG… CCTTCTGTGGA…
## 5 AX-58352… f      1.152     5192418 G        A        ATAAGTTATGG… TCTTCTTGCAT…
## 6 AX-58422… f      1.91      1315178 A        T        GCATGATTTGT… CTATGGCAGAA…
## # ℹ 6 more variables: alt_allele_F <chr>, alt_allele_R <chr>, N_allele_F <chr>,
## #   N_allele_R <chr>, fasta_name_F <chr>, fasta_name_R <chr>
```

Sanity check - check if the sequences are generated correctly:

AX-583388258 (reverse)
CCACCCAGTTGAAATTGTATCGGAACGGTGCATTA[C/T]TCATTTTAACGCTTTTTATCACAGATGCACATAAA
T C : A G AX-582323173 (forward)
CCAAAGGATATTTGGTCAGTACCAATAGTAAAAGT[A/T]TCGTCTGAAGTAAATGAAACCTCTTCAGTTAAACA
T A : T A

probe\_left\_ref TTTATGTGCATCTGTGATAAAAAGCGTTAAAATGA[A]
CCAAAGGATATTTGGTCAGTACCAATAGTAAAAGT[T]

probe\_left\_alt TTTATGTGCATCTGTGATAAAAAGCGTTAAAATGA[G]
CCAAAGGATATTTGGTCAGTACCAATAGTAAAAGT[A]

probe\_right\_ref [A]TAATGCACCGTTCCGATACAATTTCAACTGGGTGG
[T]TCGTCTGAAGTAAATGAAACCTCTTCAGTTAAACA

probe\_right\_alt [G]TAATGCACCGTTCCGATACAATTTCAACTGGGTGG
[A]TCGTCTGAAGTAAATGAAACCTCTTCAGTTAAACA

ref\_allele\_F AX-583388258\_F
TTTATGTGCATCTGTGATAAAAAGCGTTAAAATGA[A]TAATGCACCGTTCCGATACAATTTCAACTGGGTGG
AX-582323173\_F
CCAAAGGATATTTGGTCAGTACCAATAGTAAAAGT[T]TCGTCTGAAGTAAATGAAACCTCTTCAGTTAAACA

ref\_allele\_R AX-583388258\_R
CCACCCAGTTGAAATTGTATCGGAACGGTGCATTA[T]TCATTTTAACGCTTTTTATCACAGATGCACATAAA
AX-582323173\_R
TGTTTAACTGAAGAGGTTTCATTTACTTCAGACGA[A]ACTTTTACTATTGGTACTGACCAAATATCCTTTGG

alt\_allele\_F AX-583388258\_F
TTTATGTGCATCTGTGATAAAAAGCGTTAAAATGA[G]TAATGCACCGTTCCGATACAATTTCAACTGGGTGG
AX-583388258\_F
CCAAAGGATATTTGGTCAGTACCAATAGTAAAAGT[A]TCGTCTGAAGTAAATGAAACCTCTTCAGTTAAACA

alt\_allele\_R AX-583388258\_R
CCACCCAGTTGAAATTGTATCGGAACGGTGCATTA[C]TCATTTTAACGCTTTTTATCACAGATGCACATAAA
AX-583388258\_R
TGTTTAACTGAAGAGGTTTCATTTACTTCAGACGA[T]ACTTTTACTATTGGTACTGACCAAATATCCTTTGG

Let create the directories for the outputs

```
# create output directories 
dir_names <- c(
  "sequences"
)

for (dir_name in dir_names) {
  here("output", "probes", dir_name) |>
    dir.create(recursive = TRUE, showWarnings = FALSE)
}
```

Check one SNP again to make sure all sequences are correct

```
# check the sequences of the first snps
albopictus_seq_02 |>
  filter(
    Name == "AX-583388258"
  )
```

```
## # A tibble: 1 × 14
##   Name      Strand Scaffold cust_pos cust_ref cust_alt ref_allele_F ref_allele_R
##   <chr>     <chr>  <chr>       <dbl> <chr>    <chr>    <chr>        <chr>       
## 1 AX-58338… r      1.144     5714162 T        C        TTTATGTGCAT… CCACCCAGTTG…
## # ℹ 6 more variables: alt_allele_F <chr>, alt_allele_R <chr>, N_allele_F <chr>,
## #   N_allele_R <chr>, fasta_name_F <chr>, fasta_name_R <chr>
```

Sanity check:

AX-583388258
CCACCCAGTTGAAATTGTATCGGAACGGTGCATTA[C/T]TCATTTTAACGCTTTTTATCACAGATGCACATAAA
r: the reference allele is a A (5’ to 3”) or a T (3’ to 5’) ref\_allele\_F
TTTATGTGCATCTGTGATAAAAAGCGTTAAAATGA[A]TAATGCACCGTTCCGATACAATTTCAACTGGGTGG
ref\_allele\_R
CCACCCAGTTGAAATTGTATCGGAACGGTGCATTA[T]TCATTTTAACGCTTTTTATCACAGATGCACATAAA

alt\_allele\_F
TTTATGTGCATCTGTGATAAAAAGCGTTAAAATGA[G]TAATGCACCGTTCCGATACAATTTCAACTGGGTGG
alt\_allele\_R
CCACCCAGTTGAAATTGTATCGGAACGGTGCATTA[C]TCATTTTAACGCTTTTTATCACAGATGCACATAAA

N\_allele\_F
TTTATGTGCATCTGTGATAAAAAGCGTTAAAATGA[N]TAATGCACCGTTCCGATACAATTTCAACTGGGTGG
N\_allele\_R
CCACCCAGTTGAAATTGTATCGGAACGGTGCATTA[N]TCATTTTAACGCTTTTTATCACAGATGCACATAAA

We can create a list of sequences that we want to map

albopictus\_Ref\_F

```
# pivot the data frame to a longer format and extract the values as a vector
albopictus_Ref_F <- 
  albopictus_seq_02 |>
  pivot_longer(cols = c(fasta_name_F, ref_allele_F), names_to = "fasta_name") |>
  arrange(row_number(), fasta_name) |>  # sort by row number and column
  pull(value)

# Write the character vector to a file with no row or column names, and no quotes
writeLines(
  albopictus_Ref_F, here(
    "output", "probes", "sequences", "albopictus_Ref_F.fasta"
  )
)

head(albopictus_Ref_F)
```

```
## [1] ">AX-583388258_F"                                                        
## [2] "TTTATGTGCATCTGTGATAAAAAGCGTTAAAATGAATAATGCACCGTTCCGATACAATTTCAACTGGGTGG"
## [3] ">AX-582323173_F"                                                        
## [4] "CCAAAGGATATTTGGTCAGTACCAATAGTAAAAGTTTCGTCTGAAGTAAATGAAACCTCTTCAGTTAAACA"
## [5] ">AX-584070262_F"                                                        
## [6] "CAAACATACGTTTTGTCGTAAGTGAAATCGGTATAGAAAATTTCAGAAAATGATTATTTTTCAACATTTTG"
```

Count how many sequences (they should have 2 x 175,396 = 350,792)

```
echo "How many lines:"
wc -l output/probes/sequences/albopictus_Ref_F.fasta;

echo "
How many sequences:"
grep -c ">"  output/probes/sequences/albopictus_Ref_F.fasta;

echo "
Check the first lines:"
head output/probes/sequences/albopictus_Ref_F.fasta
```

```
## How many lines:
##   350792 output/probes/sequences/albopictus_Ref_F.fasta
## 
## How many sequences:
## 175396
## 
## Check the first lines:
## >AX-583388258_F
## TTTATGTGCATCTGTGATAAAAAGCGTTAAAATGAATAATGCACCGTTCCGATACAATTTCAACTGGGTGG
## >AX-582323173_F
## CCAAAGGATATTTGGTCAGTACCAATAGTAAAAGTTTCGTCTGAAGTAAATGAAACCTCTTCAGTTAAACA
## >AX-584070262_F
## CAAACATACGTTTTGTCGTAAGTGAAATCGGTATAGAAAATTTCAGAAAATGATTATTTTTCAACATTTTG
## >AX-580158081_F
## AAACATGTATGGAGTCTAAGGACAAAATGTACAATACTAACCCACATCTTGTTCGTCCAGTCCACAGAAGG
## >AX-583523242_F
## ATAAGTTATGGGTTTAGACAAATACACGAAATGAGGATAACCAACCATAATAATTTCCAGATGCAAGAAGA
```

albopictus\_Ref\_R

```
# Pivot the data frame to a longer format and extract the values as a vector
albopictus_Ref_R <- 
  albopictus_seq_02 |>
  pivot_longer(cols = c(fasta_name_R, ref_allele_R), names_to = "fasta_name") |>
  arrange(row_number(), fasta_name) |>  # sort by row number and column
  pull(value)

# Write the character vector to a file with no row or column names, and no quotes
writeLines(
  albopictus_Ref_R, here(
    "output", "probes", "sequences", "albopictus_Ref_R.fasta"
  )
)

head(albopictus_Ref_R)
```

```
## [1] ">AX-583388258_R"                                                        
## [2] "CCACCCAGTTGAAATTGTATCGGAACGGTGCATTATTCATTTTAACGCTTTTTATCACAGATGCACATAAA"
## [3] ">AX-582323173_R"                                                        
## [4] "TGTTTAACTGAAGAGGTTTCATTTACTTCAGACGAAACTTTTACTATTGGTACTGACCAAATATCCTTTGG"
## [5] ">AX-584070262_R"                                                        
## [6] "CAAAATGTTGAAAAATAATCATTTTCTGAAATTTTCTATACCGATTTCACTTACGACAAAACGTATGTTTG"
```

albopictus\_Alt\_F

```
# Pivot the data frame to a longer format and extract the values as a vector
albopictus_Alt_F <- 
  albopictus_seq_02 |>
  pivot_longer(cols = c(fasta_name_F, alt_allele_F), names_to = "fasta_name") |>
  arrange(row_number(), fasta_name) |>  # sort by row number and column
  pull(value)

# Write the character vector to a file with no row or column names, and no quotes
writeLines(
  albopictus_Alt_F, here(
    "output", "probes", "sequences", "albopictus_Alt_F.fasta"
  )
)

head(albopictus_Alt_F)
```

```
## [1] ">AX-583388258_F"                                                        
## [2] "TTTATGTGCATCTGTGATAAAAAGCGTTAAAATGAGTAATGCACCGTTCCGATACAATTTCAACTGGGTGG"
## [3] ">AX-582323173_F"                                                        
## [4] "CCAAAGGATATTTGGTCAGTACCAATAGTAAAAGTATCGTCTGAAGTAAATGAAACCTCTTCAGTTAAACA"
## [5] ">AX-584070262_F"                                                        
## [6] "CAAACATACGTTTTGTCGTAAGTGAAATCGGTATAAAAAATTTCAGAAAATGATTATTTTTCAACATTTTG"
```

albopictus\_Alt\_R

```
# Pivot the data frame to a longer format and extract the values as a vector
albopictus_Alt_R <- 
  albopictus_seq_02 |>
  pivot_longer(cols = c(fasta_name_R, alt_allele_R), names_to = "fasta_name") |>
  arrange(row_number(), fasta_name) |>  # sort by row number and column
  pull(value)

# Write the character vector to a file with no row or column names, and no quotes
writeLines(
  albopictus_Alt_R, here(
    "output", "probes", "sequences", "albopictus_Alt_R.fasta"
  )
)

head(albopictus_Alt_R)
```

```
## [1] ">AX-583388258_R"                                                        
## [2] "CCACCCAGTTGAAATTGTATCGGAACGGTGCATTACTCATTTTAACGCTTTTTATCACAGATGCACATAAA"
## [3] ">AX-582323173_R"                                                        
## [4] "TGTTTAACTGAAGAGGTTTCATTTACTTCAGACGATACTTTTACTATTGGTACTGACCAAATATCCTTTGG"
## [5] ">AX-584070262_R"                                                        
## [6] "CAAAATGTTGAAAAATAATCATTTTCTGAAATTTTTTATACCGATTTCACTTACGACAAAACGTATGTTTG"
```

albopictus\_N\_F

```
# Pivot the data frame to a longer format and extract the values as a vector
albopictus_N_F <- 
  albopictus_seq_02 |>
  pivot_longer(cols = c(fasta_name_F, N_allele_F), names_to = "fasta_name") |>
  arrange(row_number(), fasta_name) |>  # sort by row number and column
  pull(value)

# Write the character vector to a file with no row or column names, and no quotes
writeLines(
  albopictus_N_F, here(
    "output", "probes", "sequences", "albopictus_N_F.fasta"
  )
)

head(albopictus_N_F)
```

```
## [1] ">AX-583388258_F"                                                        
## [2] "TTTATGTGCATCTGTGATAAAAAGCGTTAAAATGANTAATGCACCGTTCCGATACAATTTCAACTGGGTGG"
## [3] ">AX-582323173_F"                                                        
## [4] "CCAAAGGATATTTGGTCAGTACCAATAGTAAAAGTNTCGTCTGAAGTAAATGAAACCTCTTCAGTTAAACA"
## [5] ">AX-584070262_F"                                                        
## [6] "CAAACATACGTTTTGTCGTAAGTGAAATCGGTATANAAAATTTCAGAAAATGATTATTTTTCAACATTTTG"
```

albopictus\_N\_R

```
# Pivot the data frame to a longer format and extract the values as a vector
albopictus_N_R <- 
  albopictus_seq_02 |>
  pivot_longer(cols = c(fasta_name_R, N_allele_R), names_to = "fasta_name") |>
  arrange(row_number(), fasta_name) |>  # sort by row number and column
  pull(value)

# Write the character vector to a file with no row or column names, and no quotes
writeLines(
  albopictus_N_R, here(
    "output", "probes", "sequences", "albopictus_N_R.fasta"
  )
)

head(albopictus_N_R)
```

```
## [1] ">AX-583388258_R"                                                        
## [2] "CCACCCAGTTGAAATTGTATCGGAACGGTGCATTANTCATTTTAACGCTTTTTATCACAGATGCACATAAA"
## [3] ">AX-582323173_R"                                                        
## [4] "TGTTTAACTGAAGAGGTTTCATTTACTTCAGACGANACTTTTACTATTGGTACTGACCAAATATCCTTTGG"
## [5] ">AX-584070262_R"                                                        
## [6] "CAAAATGTTGAAAAATAATCATTTTCTGAAATTTTNTATACCGATTTCACTTACGACAAAACGTATGTTTG"
```

Sanity check: Let’s check the sequences again to make sure our code
to create the fasta files worked we planned. The first sequence:
AX\_583388258\_r\_1\_144\_5714162\_T\_C (reverse strand) albopictus\_Ref\_F
TTTATGTGCATCTGTGATAAAAAGCGTTAAAATGA[A]TAATGCACCGTTCCGATACAATTTCAACTGGGTGG
albopictus\_Alt\_F
TTTATGTGCATCTGTGATAAAAAGCGTTAAAATGA[G]TAATGCACCGTTCCGATACAATTTCAACTGGGTGG
albopictus\_N\_F
TTTATGTGCATCTGTGATAAAAAGCGTTAAAATGA[N]TAATGCACCGTTCCGATACAATTTCAACTGGGTGG

Check the probe sequences with reference allele

```
head output/probes/sequences/albopictus_Ref_F.fasta
```

```
## >AX-583388258_F
## TTTATGTGCATCTGTGATAAAAAGCGTTAAAATGAATAATGCACCGTTCCGATACAATTTCAACTGGGTGG
## >AX-582323173_F
## CCAAAGGATATTTGGTCAGTACCAATAGTAAAAGTTTCGTCTGAAGTAAATGAAACCTCTTCAGTTAAACA
## >AX-584070262_F
## CAAACATACGTTTTGTCGTAAGTGAAATCGGTATAGAAAATTTCAGAAAATGATTATTTTTCAACATTTTG
## >AX-580158081_F
## AAACATGTATGGAGTCTAAGGACAAAATGTACAATACTAACCCACATCTTGTTCGTCCAGTCCACAGAAGG
## >AX-583523242_F
## ATAAGTTATGGGTTTAGACAAATACACGAAATGAGGATAACCAACCATAATAATTTCCAGATGCAAGAAGA
```

Check the probe sequences with alternative allele allele

```
head output/probes/sequences/albopictus_Alt_F.fasta
```

```
## >AX-583388258_F
## TTTATGTGCATCTGTGATAAAAAGCGTTAAAATGAGTAATGCACCGTTCCGATACAATTTCAACTGGGTGG
## >AX-582323173_F
## CCAAAGGATATTTGGTCAGTACCAATAGTAAAAGTATCGTCTGAAGTAAATGAAACCTCTTCAGTTAAACA
## >AX-584070262_F
## CAAACATACGTTTTGTCGTAAGTGAAATCGGTATAAAAAATTTCAGAAAATGATTATTTTTCAACATTTTG
## >AX-580158081_F
## AAACATGTATGGAGTCTAAGGACAAAATGTACAATTCTAACCCACATCTTGTTCGTCCAGTCCACAGAAGG
## >AX-583523242_F
## ATAAGTTATGGGTTTAGACAAATACACGAAATGAGAATAACCAACCATAATAATTTCCAGATGCAAGAAGA
```

Check the probe sequences with N as allele

```
head output/probes/sequences/albopictus_N_F.fasta
```

```
## >AX-583388258_F
## TTTATGTGCATCTGTGATAAAAAGCGTTAAAATGANTAATGCACCGTTCCGATACAATTTCAACTGGGTGG
## >AX-582323173_F
## CCAAAGGATATTTGGTCAGTACCAATAGTAAAAGTNTCGTCTGAAGTAAATGAAACCTCTTCAGTTAAACA
## >AX-584070262_F
## CAAACATACGTTTTGTCGTAAGTGAAATCGGTATANAAAATTTCAGAAAATGATTATTTTTCAACATTTTG
## >AX-580158081_F
## AAACATGTATGGAGTCTAAGGACAAAATGTACAATNCTAACCCACATCTTGTTCGTCCAGTCCACAGAAGG
## >AX-583523242_F
## ATAAGTTATGGGTTTAGACAAATACACGAAATGAGNATAACCAACCATAATAATTTCCAGATGCAAGAAGA
```

Sanity check: Combine the forward and reverse sequences in one file
we can use the following strategy:

We have two files: file1 head
output/probes/sequences/albopictus\_Ref\_F.fasta file1\_line1 (A)
file1\_line2 (B) file1\_line3 (C) file1\_line4 (D)

file2 head output/probes/sequences/albopictus\_Ref\_R.fasta file2\_line1
(a) file2\_line2 (b) file2\_line3 (c) file2\_line4 (d)

the output we need: head output/probes/sequences/albopictus\_Ref.fasta
file1\_line1 (A) file1\_line2 (B) file2\_line1 (a) file2\_line2 (b)
file1\_line3 (C) file1\_line4 (D) file2\_line3 (c) file2\_line4 (d)

Forward

```
head output/probes/sequences/albopictus_Ref_F.fasta
```

```
## >AX-583388258_F
## TTTATGTGCATCTGTGATAAAAAGCGTTAAAATGAATAATGCACCGTTCCGATACAATTTCAACTGGGTGG
## >AX-582323173_F
## CCAAAGGATATTTGGTCAGTACCAATAGTAAAAGTTTCGTCTGAAGTAAATGAAACCTCTTCAGTTAAACA
## >AX-584070262_F
## CAAACATACGTTTTGTCGTAAGTGAAATCGGTATAGAAAATTTCAGAAAATGATTATTTTTCAACATTTTG
## >AX-580158081_F
## AAACATGTATGGAGTCTAAGGACAAAATGTACAATACTAACCCACATCTTGTTCGTCCAGTCCACAGAAGG
## >AX-583523242_F
## ATAAGTTATGGGTTTAGACAAATACACGAAATGAGGATAACCAACCATAATAATTTCCAGATGCAAGAAGA
```

Reverse

```
head output/probes/sequences/albopictus_Ref_R.fasta
```

```
## >AX-583388258_R
## CCACCCAGTTGAAATTGTATCGGAACGGTGCATTATTCATTTTAACGCTTTTTATCACAGATGCACATAAA
## >AX-582323173_R
## TGTTTAACTGAAGAGGTTTCATTTACTTCAGACGAAACTTTTACTATTGGTACTGACCAAATATCCTTTGG
## >AX-584070262_R
## CAAAATGTTGAAAAATAATCATTTTCTGAAATTTTCTATACCGATTTCACTTACGACAAAACGTATGTTTG
## >AX-580158081_R
## CCTTCTGTGGACTGGACGAACAAGATGTGGGTTAGTATTGTACATTTTGTCCTTAGACTCCATACATGTTT
## >AX-583523242_R
## TCTTCTTGCATCTGGAAATTATTATGGTTGGTTATCCTCATTTCGTGTATTTGTCTAAACCCATAACTTAT
```

Create sequences with forward and reverse sequences for each allele
(they should map at the same genomic location)

Reference allele

```
with open("output/probes/sequences/albopictus_Ref_F.fasta") as f1, open("output/probes/sequences/albopictus_Ref_R.fasta") as f2, open("output/probes/sequences/albopictus_Ref.fasta", "w") as out:
    while True:
        try:
            f1_lines = [next(f1) for _ in range(2)] # read 2 lines from file1
            f2_lines = [next(f2) for _ in range(2)] # read 2 lines from file2
            out.writelines(f1_lines + f2_lines) # write 4 lines to output file
        except StopIteration:
            break
```

To remove all data from python

```
# python
py_run_string("import gc; gc.collect()")
```

Sanity check: check the probe sequences with reference allele

```
head output/probes/sequences/albopictus_Ref.fasta
```

```
## >AX-583388258_F
## TTTATGTGCATCTGTGATAAAAAGCGTTAAAATGAATAATGCACCGTTCCGATACAATTTCAACTGGGTGG
## >AX-583388258_R
## CCACCCAGTTGAAATTGTATCGGAACGGTGCATTATTCATTTTAACGCTTTTTATCACAGATGCACATAAA
## >AX-582323173_F
## CCAAAGGATATTTGGTCAGTACCAATAGTAAAAGTTTCGTCTGAAGTAAATGAAACCTCTTCAGTTAAACA
## >AX-582323173_R
## TGTTTAACTGAAGAGGTTTCATTTACTTCAGACGAAACTTTTACTATTGGTACTGACCAAATATCCTTTGG
## >AX-584070262_F
## CAAACATACGTTTTGTCGTAAGTGAAATCGGTATAGAAAATTTCAGAAAATGATTATTTTTCAACATTTTG
```

Alternative allele

```
with open("output/probes/sequences/albopictus_Alt_F.fasta") as f1, open("output/probes/sequences/albopictus_Alt_R.fasta") as f2, open("output/probes/sequences/albopictus_Alt.fasta", "w") as out:
    while True:
        try:
            f1_lines = [next(f1) for _ in range(2)] # read 2 lines from file1
            f2_lines = [next(f2) for _ in range(2)] # read 2 lines from file2
            out.writelines(f1_lines + f2_lines) # write 4 lines to output file
        except StopIteration:
            break
```

To remove all data from python

```
# python
py_run_string("import gc; gc.collect()")
```

Sanity check: the probe sequences with alternative allele

```
head output/probes/sequences/albopictus_Alt.fasta
```

```
## >AX-583388258_F
## TTTATGTGCATCTGTGATAAAAAGCGTTAAAATGAGTAATGCACCGTTCCGATACAATTTCAACTGGGTGG
## >AX-583388258_R
## CCACCCAGTTGAAATTGTATCGGAACGGTGCATTACTCATTTTAACGCTTTTTATCACAGATGCACATAAA
## >AX-582323173_F
## CCAAAGGATATTTGGTCAGTACCAATAGTAAAAGTATCGTCTGAAGTAAATGAAACCTCTTCAGTTAAACA
## >AX-582323173_R
## TGTTTAACTGAAGAGGTTTCATTTACTTCAGACGATACTTTTACTATTGGTACTGACCAAATATCCTTTGG
## >AX-584070262_F
## CAAACATACGTTTTGTCGTAAGTGAAATCGGTATAAAAAATTTCAGAAAATGATTATTTTTCAACATTTTG
```

N as allele

```
with open("output/probes/sequences/albopictus_N_F.fasta") as f1, open("output/probes/sequences/albopictus_N_R.fasta") as f2, open("output/probes/sequences/albopictus_N.fasta", "w") as out:
    while True:
        try:
            f1_lines = [next(f1) for _ in range(2)] # read 2 lines from file1
            f2_lines = [next(f2) for _ in range(2)] # read 2 lines from file2
            out.writelines(f1_lines + f2_lines) # write 4 lines to output file
        except StopIteration:
            break
```

To remove all data from python

```
# python
py_run_string("import gc; gc.collect()")
```

Sanity check: the probe sequences with alternative allele

```
head output/probes/sequences/albopictus_N.fasta
```

```
## >AX-583388258_F
## TTTATGTGCATCTGTGATAAAAAGCGTTAAAATGANTAATGCACCGTTCCGATACAATTTCAACTGGGTGG
## >AX-583388258_R
## CCACCCAGTTGAAATTGTATCGGAACGGTGCATTANTCATTTTAACGCTTTTTATCACAGATGCACATAAA
## >AX-582323173_F
## CCAAAGGATATTTGGTCAGTACCAATAGTAAAAGTNTCGTCTGAAGTAAATGAAACCTCTTCAGTTAAACA
## >AX-582323173_R
## TGTTTAACTGAAGAGGTTTCATTTACTTCAGACGANACTTTTACTATTGGTACTGACCAAATATCCTTTGG
## >AX-584070262_F
## CAAACATACGTTTTGTCGTAAGTGAAATCGGTATANAAAATTTCAGAAAATGATTATTTTTCAACATTTTG
```

## 3. The genome sequences

NCBI genomes at HERE
Vector Base genomes at HERE

We can create the directories for the genomes we want to test.

```
# create output directories 
dir_names <- c(
  "genomes"
)

for (dir_name in dir_names) {
  here("output", "probes", dir_name) |>
    dir.create(recursive = TRUE, showWarnings = FALSE)
}
```

### 3.1 AalbF1\_ncbi

```
# run the code on the terminal
# I saved it on my external SSD

# go to dir
cd /Volumes/evo2/probes_genomes

# download it
curl -OJX GET "https://api.ncbi.nlm.nih.gov/datasets/v2alpha/genome/accession/GCA_001444175.2/download?include_annotation_type=GENOME_FASTA,GENOME_GFF,RNA_FASTA,CDS_FASTA,PROT_FASTA,SEQUENCE_REPORT&filename=GCA_001444175.2.zip" -H "Accept: application/zip"

# unzip it
unzip GCA_001444175.2.zip

# rename the unziped data
mv ncbi_dataset AalbF1_ncbi

# check the scaffold names
head -n 1 AalbF1_ncbi/data/GCA_001444175.2/GCA_001444175.2_A.albopictus_v1.1_genomic.fna
# >KQ560100.1 Aedes albopictus isolate Foshan unplaced genomic scaffold scaffold18, whole genome shotgun sequence

# rename the other scaffolds
sed 's/^>\([^[:space:]]*\)\.1.*/>\1/' AalbF1_ncbi/data/GCA_001444175.2/GCA_001444175.2_A.albopictus_v1.1_genomic.fna > AalbF1_ncbi.fasta

# check it
grep ">" AalbF1_ncbi.fasta | head -n 5
# >KQ560100
# >KQ560101
# >KQ560102
# >KQ560103
# >KQ560104

# now the reference genome is ready for indexing. I will do it later on the cluster
```

Assembly statistics

GenBank Genome size 1.9 Gb Total ungapped length 1.8 Gb Number of
scaffolds 154,782 Scaffold N50 201 kb Scaffold L50 2,851 Number of
contigs 355,061 Contig N50 18.4 kb Contig L50 28,288 GC percent 40
Genome coverage 229.0x Assembly level Scaffold

### 3.2 AalbF1\_vb

```
# go to dir
cd /Volumes/evo2/probes_genomes

# download it
wget https://vectorbase.org/common/downloads/release-62/AalbopictusFoshan/fasta/data/VectorBase-62_AalbopictusFoshan_Genome.fasta

# check the scaffold names
grep ">" VectorBase-62_AalbopictusFoshan_Genome.fasta | head -n 5
# >JXUM01S000001 | organism=Aedes_albopictus_Foshan | version=AaloF1 | length=879215 | SO=supercontig
# >JXUM01S000002 | organism=Aedes_albopictus_Foshan | version=AaloF1 | length=913318 | SO=supercontig
# >JXUM01S000003 | organism=Aedes_albopictus_Foshan | version=AaloF1 | length=949118 | SO=supercontig
# >JXUM01S000004 | organism=Aedes_albopictus_Foshan | version=AaloF1 | length=917017 | SO=supercontig
# >JXUM01S000005 | organism=Aedes_albopictus_Foshan | version=AaloF1 | length=885085 | SO=supercontig

# rename the other scaffolds
sed 's/^>\([^[:space:]]*\).*/>\1/' VectorBase-62_AalbopictusFoshan_Genome.fasta > AalbF1_vb.fasta

# check it
grep ">" AalbF1_vb.fasta | head -n 5
# >JXUM01S000001
# >JXUM01S000002
# >JXUM01S000003
# >JXUM01S000004
# >JXUM01S000005

# now the reference genome is ready for indexing. I will do it later on the cluster
```

### 3.3 AalbF2\_ncbi

```
# go to dir
cd /Volumes/evo2/probes_genomes

# download it
curl -OJX GET "https://api.ncbi.nlm.nih.gov/datasets/v2alpha/genome/accession/GCF_006496715.2/download?include_annotation_type=GENOME_FASTA,GENOME_GFF,RNA_FASTA,CDS_FASTA,PROT_FASTA,SEQUENCE_REPORT&filename=GCF_006496715.2.zip" -H "Accept: application/zip"

# unzip it
unzip GCF_006496715.2.zip

# rename the unziped data
mv ncbi_dataset AalbF2_ncbi

# check the scaffold names
head -n 1 AalbF2_ncbi/data/GCF_006496715.2/GCF_006496715.2_Aalbo_primary.1_genomic.fna
# >NW_021837045.1 Aedes albopictus isolate FPA unplaced genomic scaffold, Aalbo_primary.1 scaffold_1, whole genome shotgun sequence

# rename the other scaffolds
sed 's/^>\([^[:space:]]*\)\.1.*/>\1/' AalbF2_ncbi/data/GCF_006496715.2/GCF_006496715.2_Aalbo_primary.1_genomic.fna > AalbF2_ncbi.fasta

# check it
grep ">" AalbF2_ncbi.fasta | head -n 5
# >NW_021837045
# >NW_021837046
# >NW_021837047
# >NW_021837048
# >NW_021837049

# now the reference genome is ready for indexing. I will do it later on the cluster
```

Assembly statistics

RefSeq GenBank Genome size 2.5 Gb 2.5 Gb Total ungapped length 2.5 Gb
2.5 Gb Number of organelles 1  
Number of scaffolds 2,113 2,196 Scaffold N50 55.7 Mb 55.7 Mb Scaffold
L50 13 13 Number of contigs 5,472 5,555 Contig N50 1.2 Mb 1.2 Mb Contig
L50 433 434 GC percent 40 40 Genome coverage 75.0x 75.0x Assembly level
Scaffold Scaffold

### 3.4 AalbF2\_vb

```
# go to dir
cd /Volumes/evo2/probes_genomes

# download it
wget https://vectorbase.org/common/downloads/release-62/AalbopictusFoshanFPA/fasta/data/VectorBase-62_AalbopictusFoshanFPA_Genome.fasta

# check the scaffold names
grep ">" VectorBase-62_AalbopictusFoshanFPA_Genome.fasta | head -n 5
# >SWKY01000001 | organism=Aedes_albopictus_Foshan_FPA | version=2019-06-28 | length=196395033 | SO=supercontig
# >SWKY01000002 | organism=Aedes_albopictus_Foshan_FPA | version=2019-06-28 | length=168827982 | SO=supercontig
# >SWKY01000003 | organism=Aedes_albopictus_Foshan_FPA | version=2019-06-28 | length=135305655 | SO=supercontig
# >SWKY01000004 | organism=Aedes_albopictus_Foshan_FPA | version=2019-06-28 | length=122869687 | SO=supercontig
# >SWKY01000005 | organism=Aedes_albopictus_Foshan_FPA | version=2019-06-28 | length=99254364 | SO=supercontig

# rename the other scaffolds
sed 's/^>\([^[:space:]]*\).*/>\1/' VectorBase-62_AalbopictusFoshanFPA_Genome.fasta > AalbF2_vb.fasta

# check it
grep ">" AalbF2_vb.fasta | head -n 5
# >SWKY01000001
# >SWKY01000002
# >SWKY01000003
# >SWKY01000004
# >SWKY01000005

# now the reference genome is ready for indexing. I will do it later on the cluster
```

### 3.5 AalbF3 - used for chip design

```
# go to dir
cd /Volumes/evo2/probes_genomes

# download it
curl -OJX GET curl -OJX GET "https://api.ncbi.nlm.nih.gov/datasets/v2alpha/genome/accession/GCA_018104305.1/download?include_annotation_type=GENOME_FASTA,GENOME_GFF,RNA_FASTA,CDS_FASTA,PROT_FASTA,SEQUENCE_REPORT&filename=GCA_018104305.1.zip" -H "Accept: application/zip"

# unzip it
unzip GCA_018104305.1.zip

# rename the unziped data
mv ncbi_dataset AalbF3_ncbi

# check the scaffold names
head -n 1 AalbF3_ncbi/data/GCA_018104305.1/GCA_018104305.1_AalbF3_genomic.fna
# >JAFDOQ010000574.1 Aedes albopictus isolate FPA chromosome 1 chr1.1, whole genome shotgun sequence

# rename the other scaffolds - this time keep the scaffold numbers only
# for example, the first scaffold name is:
# >JAFDOQ010000574.1 Aedes albopictus isolate FPA chromosome 1 chr1.1, whole genome shotgun sequence
# it will change to:
# >1.1
# we need to do that to match the SNP chip scaffold names
sed 's/^>\([^[:space:]]*\).*chr\([[:digit:]]*\.[[:digit:]]*\),.*/>\2/' AalbF3_ncbi/data/GCA_018104305.1/GCA_018104305.1_AalbF3_genomic.fna > AalbF3.fasta

# check it
grep ">" AalbF3.fasta | head -n 5
# >1.1
# >1.10
# >1.100
# >1.101
# >1.102

# now the reference genome is ready for indexing. I will do it later on the cluster
```

Assembly statistics

GenBank Genome size 1.5 Gb Total ungapped length 1.5 Gb Number of
chromosomes 3 Number of scaffolds 574 Scaffold N50 10.1 Mb Scaffold L50
43 Number of contigs 1,134 Contig N50 7.5 Mb Contig L50 56 GC percent 40
Genome coverage 75.0x Assembly level Scaffold

Vector Base does not have the AalbF3 genome assembly.

### 3.6 AalbCell\_ncbi

```
# go to dir
cd /Volumes/evo2/probes_genomes

# download it
curl -OJX GET "https://api.ncbi.nlm.nih.gov/datasets/v2alpha/genome/accession/GCF_001876365.2/download?include_annotation_type=GENOME_FASTA,GENOME_GFF,RNA_FASTA,CDS_FASTA,PROT_FASTA,SEQUENCE_REPORT&filename=GCF_001876365.2.zip" -H "Accept: application/zip"

# unzip it
unzip GCF_001876365.2.zip

# rename the unziped data
mv ncbi_dataset AalbCell_ncbi

# check the scaffold names
head -n 1 AalbCell_ncbi/data/GCF_001876365.2/GCF_001876365.2_canu_80X_arrow2.2_genomic.fna
# >NW_017855997.1 Aedes albopictus isolate C6/36 unplaced genomic scaffold, canu_80X_arrow2.2, whole genome shotgun sequence

# rename the other scaffolds
sed 's/^>\([^[:space:]]*\)\.1.*/>\1/' AalbCell_ncbi/data/GCF_001876365.2/GCF_001876365.2_canu_80X_arrow2.2_genomic.fna > AalbCell_ncbi.fasta


# check it
grep ">" AalbCell_ncbi.fasta | head -n 5
# >NW_017855997
# >NW_017855998
# >NW_017855999
# >NW_017856000
# >NW_017856001
```

Assembly statistics

RefSeq GenBank Genome size 2.2 Gb 2.2 Gb Total ungapped length 2.2 Gb
2.2 Gb Number of organelles 1  
Number of contigs 2,434 2,434 Contig N50 3.3 Mb 3.3 Mb Contig L50 203
203 GC percent 40 Genome coverage 80.0x 80.0x Assembly level Contig
Contig

### 3.7 AalbCell\_vb

```
# go to dir
cd /Volumes/evo2/probes_genomes

# download it
wget https://vectorbase.org/common/downloads/release-62/AalbopictusC6-36/fasta/data/VectorBase-62_AalbopictusC6-36_Genome.fasta

# check the scaffold names
grep ">" VectorBase-62_AalbopictusC6-36_Genome.fasta | head -n 5
# >AY072044 | organism=Aedes_albopictus_C6/36_cell_line | version=2017-02-03 | length=16665 | SO=mitochondrial_chromosome
# >MNAF02000001 | organism=Aedes_albopictus_C6/36_cell_line | version=2017-02-03 | length=44730 | SO=supercontig
# >MNAF02000002 | organism=Aedes_albopictus_C6/36_cell_line | version=2017-02-03 | length=1627547 | SO=supercontig
# >MNAF02000003 | organism=Aedes_albopictus_C6/36_cell_line | version=2017-02-03 | length=841259 | SO=supercontig
# >MNAF02000004 | organism=Aedes_albopictus_C6/36_cell_line | version=2017-02-03 | length=27940 | SO=supercontig

# rename MT scaffold and other scaffolds
sed 's/^>AY072044 .*/>MT/' VectorBase-62_AalbopictusC6-36_Genome.fasta | sed 's/^>\([^[:space:]]*\).*/>\1/' > AalbCell_vb.fasta

# check it
grep ">" AalbCell_vb.fasta | head -n 5
# >MT
# >MNAF02000001
# >MNAF02000002
# >MNAF02000003
# >MNAF02000004
```

### 3.5 AalbRamini

There is a warning on NCBI “Warning: contaminated”. There is no
information about what the contamination it is. So, we have to be
careful when checking the results.

```
# go to dir
cd /Volumes/evo2/probes_genomes

# download it
curl -OJX GET "https://api.ncbi.nlm.nih.gov/datasets/v2alpha/genome/accession/GCA_001574995.1/download?include_annotation_type=GENOME_FASTA,GENOME_GFF,RNA_FASTA,CDS_FASTA,PROT_FASTA,SEQUENCE_REPORT&filename=GCA_001574995.1.zip" -H "Accept: application/zip"

# unzip it
unzip GCA_001574995.1.zip

# rename the unziped data
mv ncbi_dataset AalbRamini

# check the scaffold names
head -n 1 AalbRamini/data/GCA_001574995.1/GCA_001574995.1_ASM157499v1_genomic.fna
# >LMAV010000001.1 Aedes albopictus isolate Rimini 3965547, whole genome shotgun sequence

# rename the other scaffolds
sed 's/^>\([^[:space:]]*\)\.1.*/>\1/' AalbRamini/data/GCA_001574995.1/GCA_001574995.1_ASM157499v1_genomic.fna > AalbRamini.fasta

# check it
grep ">" AalbRamini.fasta | head -n 5
# >LMAV010000001
# >LMAV010000002
# >LMAV010000003
# >LMAV010000004
# >LMAV010000005
```

Assembly statistics

GenBank Genome size 1.3 Gb Total ungapped length 1.3 Gb Number of
contigs 3,342,920 Contig N50 397 bp Contig L50 994,094 GC percent 40
Genome coverage 60.0x Assembly level Contig

This genome assembly is not available on Vector Base.

### 3.6. Aedes aegypti genome

We can also map the sequences to the reference genome of Aedes
aegypti genomes and most of them will not map uniquely (as a sanity
check).

#### 3.6.1 AaegL5\_ncbi

Available HERE

```
# go to dir
cd /Volumes/evo2/probes_genomes

# download it
curl -v -OJX GET "https://api.ncbi.nlm.nih.gov/datasets/v2alpha/genome/accession/GCF_002204515.2/download?include_annotation_type=GENOME_FASTA,GENOME_GFF,RNA_FASTA,CDS_FASTA,PROT_FASTA,SEQUENCE_REPORT&filename=GCF_002204515.2.zip" -H "Accept: application/zip";

# unzip it
unzip GCF_002204515.2.zip

# rename the unziped data
mv ncbi_dataset AaegL5_ncbi

# check the scaffold names
head -n 1 AaegL5_ncbi/data/GCF_002204515.2/GCF_002204515.2_AaegL5.0_genomic.fna
# >NC_035107.1 Aedes aegypti strain LVP_AGWG chromosome 1, AaegL5.0 Primary Assembly, whole genome shotgun sequence

# get the 3 chromosomes names
grep "chromosome" AaegL5_ncbi/data/GCF_002204515.2/GCF_002204515.2_AaegL5.0_genomic.fna | head
# >NC_035107.1 Aedes aegypti strain LVP_AGWG chromosome 1, AaegL5.0 Primary Assembly, whole genome shotgun sequence
# >NC_035108.1 Aedes aegypti strain LVP_AGWG chromosome 2, AaegL5.0 Primary Assembly, whole genome shotgun sequence
# >NC_035109.1 Aedes aegypti strain LVP_AGWG chromosome 3, AaegL5.0 Primary Assembly, whole genome shotgun sequence
# MT    MF194022.1  NC_035159.1 

# rename the remaining scaffolds
awk '/^>NC_035107\.1/{print ">1";next} /^>NC_035108\.1/{print ">2";next} /^>NC_035109\.1/{print ">3";next} /^>NC_035159\.1/{print ">MT";next} 1' AaegL5_ncbi/data/GCF_002204515.2/GCF_002204515.2_AaegL5.0_genomic.fna > AaegL5_ncbi.fasta

# check the output
grep ">" AaegL5_ncbi.fasta | head -n 5
# >1
# >2
# >3
# >NW_018734407.1 Aedes aegypti strain LVP_AGWG unplaced genomic scaffold, AaegL5.0 Primary Assembly AGWG_AaegL5_hic_scaff_1000_PBJ_arrow, whole genome shotgun sequence
# >NW_018734408.1 Aedes aegypti strain LVP_AGWG unplaced genomic scaffold, AaegL5.0 Primary Assembly AGWG_AaegL5_hic_scaff_1001_PBJ_arrow, whole genome shotgun sequence

# rename the other scaffolds
sed 's/^>\([^[:space:]]*\)\.1.*/>\1/' AaegL5_ncbi.fasta > AaegL5_ncbi.fasta.tmp && mv AaegL5_ncbi.fasta.tmp AaegL5_ncbi.fasta

# check it
 grep ">"  AaegL5_ncbi.fasta | head -n 5
# >1
# >2
# >3
# >NW_018734407
# >NW_018734408

# now the reference genome is ready for indexing. I will do it later on the cluster
```

Assembly statistics

RefSeq GenBank Genome size 1.3 Gb 1.3 Gb Total ungapped length 1.3 Gb
1.3 Gb Number of chromosomes 3 3 Number of organelles 1 1 Number of
scaffolds 2,309 2,309 Scaffold N50 409.8 Mb 409.8 Mb Scaffold L50 2 2
Number of contigs 2,538 2,538 Contig N50 11.8 Mb 11.8 Mb Contig L50 30
30 GC percent 38 38 Genome coverage 110.0x 110.0x Assembly level
Chromosome Chromosome Note: there are 2306 unplaced scaffolds in this
assembly Chromosomes and MT scaffolds: GenBank RefSeq 1 CM008043.1
NC\_035107.1 2 CM008044.1 NC\_035108.1 3 CM008045.1 NC\_035109.1 MT
MF194022.1 NC\_035159.1

#### 3.6.2 AaegL5\_vb

The genome from Vector Base

Available HERE

```
# run the code on the terminal
# I will save it on my external SSD

# go to dir
cd /Volumes/evo2/probes_genomes

# download it
wget https://vectorbase.org/common/downloads/release-62/AaegyptiLVP_AGWG/fasta/data/VectorBase-62_AaegyptiLVP_AGWG_Genome.fasta

# check the scaffold names
grep ">" VectorBase-62_AaegyptiLVP_AGWG_Genome.fasta | head -n 5
# >AaegL5_1 | organism=Aedes_aegypti_LVP_AGWG | version=AaegL5 | length=310827022 | SO=chromosome
# >AaegL5_2 | organism=Aedes_aegypti_LVP_AGWG | version=AaegL5 | length=474425716 | SO=chromosome
# >AaegL5_3 | organism=Aedes_aegypti_LVP_AGWG | version=AaegL5 | length=409777670 | SO=chromosome
# >AaegL5_MT | organism=Aedes_aegypti_LVP_AGWG | version=AaegL5 | length=16790 | SO=mitochondrial_chromosome
# >NIGP01000004 | organism=Aedes_aegypti_LVP_AGWG | version=AaegL5 | length=30735 | SO=supercontig

# rename the remaining scaffolds
awk '/^>AaegL5_1/{print ">1"} /^>AaegL5_2/{print ">2"} /^>AaegL5_3/{print ">3"} /^>AaegL5_MT/{print ">MT"} !/^>AaegL5_1|^>AaegL5_2|^>AaegL5_3|^>AaegL5_MT/{print}' VectorBase-62_AaegyptiLVP_AGWG_Genome.fasta > AaegL5_vb.fasta


# check the output
grep ">" AaegL5_vb.fasta | head -n 5
# >1
# >2
# >3
# >MT
# >NIGP01000004 | organism=Aedes_aegypti_LVP_AGWG | version=AaegL5 | length=30735 | SO=supercontig

# rename the other scaffolds
sed 's/^>\([^[:space:]]*\).*/>\1/' AaegL5_vb.fasta > AaegL5_vb.fasta.tmp && mv AaegL5_vb.fasta.tmp AaegL5_vb.fasta

# check it
cat AaegL5_vb.fasta | grep ">"  | head -n 5
# >1
# >2
# >3
# >MT
# >NIGP01000004

# now the reference genome is ready for indexing. I will do it later on the cluster
```

Compress genomes and put in project directory

```
for file in /Volumes/evo2/probes_genomes/*.fasta; do bgzip --threads 6 "$file" -c > /Users/lucianocosme/Library/CloudStorage/Dropbox/Albopictus/manuscript_chip/data/no_autogenous/albo_chip/output/probes/genomes/"$(basename "$file").gz"; done
```

We can check one genome to see if it worked

```
(cat output/probes/genomes/AalbF3.fasta.gz | bgzip -d |  grep ">" |head -n 5) 2>/dev/null
```

```
## >1.1
## >1.10
## >1.100
## >1.101
## >1.102
```

## 4. Mapping sequences on the HPC

### 4.1 Transfer genomes file to cluster

```
# transfer genomes
rsync -chavzP --stats /Users/lucianocosme/Library/CloudStorage/Dropbox/Albopictus/manuscript_chip/data/no_autogenous/albo_chip/output/probes/genomes lvc26@mccleary.ycrc.yale.edu:/ycga-gpfs/project/caccone/lvc26/probes
```

Decompress genomes

```
# create interactive session with 1 CPU
salloc --partition devel --time=06:00:00 --nodes=1 --ntasks=1 --cpus-per-task=1 --mem-per-cpu=10Gb zsh

# decompress
for file in genomes/*.gz; do gzip -d "$file"; done
```

Index with Samtools

```
# load Samtools
module load SAMtools/1.16-GCCcore-10.2.0

# index the genomes
for file in /ycga-gpfs/project/caccone/lvc26/probes/genomes/*.fasta
do
    samtools faidx "$file"
done
```

### 4.2 Transfer sequences file to cluster

```
# next we can download the files to our computer, run the command below in your computer, not in the cluster.
rsync -chavzP --stats /Users/lucianocosme/Library/CloudStorage/Dropbox/Albopictus/manuscript_chip/data/no_autogenous/albo_chip/output/probes/sequences lvc26@mccleary.ycrc.yale.edu:/ycga-gpfs/project/caccone/lvc26/probes
```

### 4.3 Index the reference genomes with BWA and bowtie2

If you want to get BWA on the cluster or your computer

```
# go to dir
cd /ycga-gpfs/project/caccone/lvc26/probes

# download 
wget https://github.com/lh3/bwa/releases/download/v0.7.17/bwa-0.7.17.tar.bz2

# decompress
tar -xjf bwa-0.7.17.tar.bz2

# go to dir
cd bwa-0.7.17

# compile
make

# to use it on the scripts
export PATH=/ycga-gpfs/project/caccone/lvc26/probes/bwa-0.7.17:$PATH
```

Get bowtie2

```
# download it
wget https://github.com/BenLangmead/bowtie2/releases/download/v2.4.4/bowtie2-2.4.4-linux-x86_64.zip

# unzip
unzip bowtie2-2.4.4-linux-x86_64.zip

# export path on the batch scripts
export PATH=/ycga-gpfs/project/caccone/lvc26/probes/bowtie2-2.4.4-linux-x86_64:$PATH
```

Download and install Samtools

Dependency: install libncurses5-dev

```
# download
wget https://invisible-mirror.net/archives/ncurses/ncurses-6.3.tar.gz

# extract
tar -xzf ncurses-6.3.tar.gz

# move to dir
cd ncurses-6.3

# configure
./configure --prefix=/gpfs/ycga/project/caccone/lvc26/probes/ncurses-6.3

# compile
make

# install
make install

# export path
export PATH=/gpfs/ycga/project/caccone/lvc26/probes/ncurses-6.3:$PATH

# add the ncurses library directory to your LD_LIBRARY_PATH variable
export LD_LIBRARY_PATH=/gpfs/ycga/project/caccone/lvc26/probes/ncurses-6.3/lib:$LD_LIBRARY_PATH
```

Samtools

```
# download it
wget https://github.com/samtools/samtools/releases/download/1.13/samtools-1.13.tar.bz2

# unzip
tar -xjf samtools-1.13.tar.bz2

# go to dir
cd samtools-1.13


# Check if the libbz2 library is installed
ldconfig -p | grep libbz2

module load GCCcore/12.2.0
# configure it
./configure --prefix=/ycga-gpfs/project/caccone/lvc26/probes/samtools-1.13 --with-bz2=/lib64

# install it 
make install

# export path on the batch scripts
export PATH=/ycga-gpfs/project/caccone/lvc26/probes/samtools-1.13:$PATH
```

Create interactive session, load modules, and test them

```
# create interactive session with 1 CPU
salloc --partition devel --time=06:00:00 --nodes=1 --ntasks=1 --cpus-per-task=1 --mem-per-cpu=5120 zsh

# create interactive session with 4 CPUs
salloc --partition devel --time=06:00:00 --nodes=1 --ntasks=1 --cpus-per-task=4 --mem-per-cpu=5120 zsh

# modules
module load BWA/0.7.17-GCCcore-10.2.0 # I am having problems with it
module load Bowtie2/2.4.2-GCCcore-1
module load SAMtools/1.16-GCCcore-10.2.0

# check if they are working or check their versions. I had issues with them and I decided to install my own versions.
```

Create files to use with Slurm

```
# go to dir
cd /ycga-gpfs/project/caccone/lvc26/probes/scripts

# create genome files (10 genomes)
ls -1 /ycga-gpfs/project/caccone/lvc26/probes/genomes/*.fasta > genomes.txt
ls -1 /ycga-gpfs/project/caccone/lvc26/probes/genomes/*.fasta | sed 's/.*\/\([^\/]*\)\.fasta/\1/' > genome_names.txt

# create sample files (9 sequences)
ls -1 /ycga-gpfs/project/caccone/lvc26/probes/sequences/albopictus*.fasta > sequences.txt
ls -1 /ycga-gpfs/project/caccone/lvc26/probes/sequences/albopictus*.fasta | sed 's/.*\/\([^\/]*\)\.fasta/\1/' > sequence_names.txt
```

#### 4.3.1 BWA index

Index with bwa

```
#!/bin/bash
#SBATCH --mail-type=END,FAIL
#SBATCH --mail-user=luciano.cosme@yale.edu
#SBATCH --job-name=bwa
#SBATCH --partition=day
#SBATCH --time=12:00:00
#SBATCH --cpus-per-task=1
#SBATCH --mem-per-cpu=20Gb
#SBATCH --array=1-10
#SBATCH -o /ycga-gpfs/project/caccone/lvc26/probes/scripts/bwa_logs/bwa_index_%A_%a.o.txt
#SBATCH -e /ycga-gpfs/project/caccone/lvc26/probes/scripts/bwa_logs/bwa_index_%A_%a.ERROR.txt

# load modules (twice the module was not working and I had to contact HPC support)
module load BWA/0.7.17-GCCcore-10.2.0

# or use my own
# export PATH=/ycga-gpfs/project/caccone/lvc26/probes/bwa-0.7.17:$PATH

# go to dir
cd /ycga-gpfs/project/caccone/lvc26/probes/scripts

# Set job name, output, and error environment variables
genomes="/ycga-gpfs/project/caccone/lvc26/probes/scripts/genomes.txt"
gnames="/ycga-gpfs/project/caccone/lvc26/probes/scripts/genome_names.txt"

threads=$SLURM_CPUS_PER_TASK

genomes_count=$(wc -l < "$genomes")

genome_idx=$((($SLURM_ARRAY_TASK_ID - 1) % $genomes_count + 1))

genome=$(sed -n "${genome_idx}p" "$genomes" | awk '{print $1}')
gname=$(sed -n "${genome_idx}p" "$gnames" | awk '{print $1}')

# sanity checks
echo "SLURM_ARRAY_TASK_ID: $SLURM_ARRAY_TASK_ID"
echo "Genome file: $genome"
echo "Genome name: $gname"

# create index
bwa index $genome;

# create file that maps the task ID to the genome and sequence names
gname=$(sed -n "${genome_idx}p" /ycga-gpfs/project/caccone/lvc26/probes/scripts/genome_names.txt | awk '{print $1}')
echo "$SLURM_ARRAY_TASK_ID:$gname" >> /gpfs/ycga/project/caccone/lvc26/probes/scripts/bwa_logs/bwa_index_task_id_mapping.txt
```

Update logs

```
#!/bin/sh
#SBATCH --mail-type=END,FAIL
#SBATCH --mail-user=luciano.cosme@yale.edu
#SBATCH --partition=day
#SBATCH --ntasks=1
#SBATCH --cpus-per-task=1
#SBATCH --mem-per-cpu=5G
#SBATCH --time=12:00:00
#SBATCH --job-name=bwa_indexL
#SBATCH -o /ycga-gpfs/project/caccone/lvc26/probes/scripts/bwa_logs/bwa_index_%A_%a.o.txt
#SBATCH -e /ycga-gpfs/project/caccone/lvc26/probes/scripts/bwa_logs/bwa_index_%A_%a.ERROR.txt

# go to dir
cd /gpfs/ycga/project/caccone/lvc26/probes/scripts/bwa_logs

# rename the log files
while IFS=: read -r task_id gname; do
  for ext in o.txt ERROR.txt; do
    for old_file in bwa_index_*_${task_id}.${ext}; do
      if [ -e "$old_file" ]; then
        new_file="bwa_index_${gname}.${ext}"
        mv "$old_file" "$new_file"
      fi
    done
  done
done < "bwa_index_task_id_mapping.txt"
```

Job wrapper for bwa index jobs

```
#!/bin/sh
#SBATCH --mail-type=END,FAIL
#SBATCH --mail-user=luciano.cosme@yale.edu
#SBATCH --partition=day
#SBATCH --ntasks=1
#SBATCH --cpus-per-task=1
#SBATCH --mem-per-cpu=1G
#SBATCH --time=12:00:00
#SBATCH --job-name=bwa_indexW
#SBATCH -o /ycga-gpfs/project/caccone/lvc26/probes/scripts/bwa_logs/bwa_wrapper_%A_%a.o.txt
#SBATCH -e /ycga-gpfs/project/caccone/lvc26/probes/scripts/bwa_logs/bwa_wrapper_%A_%a.ERROR.txt

# go to dir
cd /gpfs/ycga/project/caccone/lvc26/probes/scripts

# Submit job 1a
job_A_id=$(sbatch 1a.bwa_index.sh | awk '{print $4}')

# Submit job 1b with a dependency on the successful completion of job 1a
job_B_id=$(sbatch --dependency=afterok:${job_A_id} 1b.bwa_index.sh | awk '{print $4}')
```

Function to save the chunks

```
# Define a custom function to write the content of the chunk with the specified label
write_chunk <- function(rmd_file_path, label, output_directory, script_name) {
  # Read the Rmd file
  rmd_lines <- readLines(rmd_file_path)
  
  # Find the line numbers of the chunk start and end
  start_line <- grep(paste0("```\\{bash ", label), rmd_lines)
  end_line <- grep("```", rmd_lines[(start_line + 1):length(rmd_lines)]) + start_line
  
  # Extract the content of the chunk
  chunk_content <- rmd_lines[(start_line + 1):(end_line - 1)]
  
  # Save the content of the chunk to a file
  file_path <- file.path(output_directory, script_name)
  cat(chunk_content, sep = "\n", file = file_path)
  message("Bash script saved as: ", file_path)
}

# we can save the function to source it later
dump(
  "write_chunk",
  here(
    "scripts", "analysis", "write_chunk.R")
)
```

Now we can save the bash chunks. Save the RMarkdown before running
the code below:

```
# Set the R Markdown file path
rmd_file_path <- "scripts/RMarkdown_files/09.mapping_probes.Rmd"

# Set the output directory
output_directory <- "output/probes/scripts"

# Write the content of the chunk with the specified label
write_chunk(rmd_file_path, "1a.bwa_index.sh", output_directory, "1a.bwa_index.sh")
```

```
## Bash script saved as: output/probes/scripts/1a.bwa_index.sh
```

```
write_chunk(rmd_file_path, "1b.bwa_index.sh", output_directory, "1b.bwa_index.sh")
```

```
## Bash script saved as: output/probes/scripts/1b.bwa_index.sh
```

```
write_chunk(rmd_file_path, "1.bwa_index.sh", output_directory, "1.bwa_index.sh")
```

```
## Bash script saved as: output/probes/scripts/1.bwa_index.sh
```

#### 4.2.2 Bowtie index

Index with bowtie2

```
#!/bin/bash
#SBATCH --mail-type=END,FAIL
#SBATCH --mail-user=luciano.cosme@yale.edu
#SBATCH --job-name=bowtie2
#SBATCH --partition=day
#SBATCH --time=12:00:00
#SBATCH --cpus-per-task=4
#SBATCH --mem-per-cpu=5Gb
#SBATCH --array=1-10
#SBATCH -o /ycga-gpfs/project/caccone/lvc26/probes/scripts/bowtie_logs/bowtie_index_%A_%a.o.txt
#SBATCH -e /ycga-gpfs/project/caccone/lvc26/probes/scripts/bowtie_logs/bowtie_index_%A_%a.ERROR.txt

# load modules
# module load Bowtie2/2.4.2-GCCcore-10.2.0

# the module was not indexing the genome when I tried. I installed bowtie2
export PATH=/ycga-gpfs/project/caccone/lvc26/probes/bowtie2-2.4.4-linux-x86_64:$PATH

# go to dir
cd /ycga-gpfs/project/caccone/lvc26/probes/genomes

# Set job name, output, and error environment variables
genomes="/ycga-gpfs/project/caccone/lvc26/probes/scripts/genomes.txt"
gnames="/ycga-gpfs/project/caccone/lvc26/probes/scripts/genome_names.txt"

threads=$SLURM_CPUS_PER_TASK

genomes_count=$(wc -l < "$genomes")

genome_idx=$((($SLURM_ARRAY_TASK_ID - 1) % $genomes_count + 1))

genome=$(sed -n "${genome_idx}p" "$genomes" | awk '{print $1}')
gname=$(sed -n "${genome_idx}p" "$gnames" | awk '{print $1}')

# sanity checks
echo "SLURM_ARRAY_TASK_ID: $SLURM_ARRAY_TASK_ID"
echo "Genome file: $genome"
echo "Genome name: $gname"

# create index
bowtie2-build --threads 4 --verbose --large-index $genome $gname

# create file that maps the task ID to the genome and sequence names
gname=$(sed -n "${genome_idx}p" /ycga-gpfs/project/caccone/lvc26/probes/scripts/genome_names.txt | awk '{print $1}')
echo "$SLURM_ARRAY_TASK_ID:$gname" >> /ycga-gpfs/project/caccone/lvc26/probes/scripts/bowtie_logs/bowtie_index_task_id_mapping.txt
```

Update logs

```
#!/bin/sh
#SBATCH --mail-type=END,FAIL
#SBATCH --mail-user=luciano.cosme@yale.edu
#SBATCH --partition=day
#SBATCH --ntasks=1
#SBATCH --cpus-per-task=1
#SBATCH --mem-per-cpu=5G
#SBATCH --time=12:00:00
#SBATCH --job-name=bowtie_indexL
#SBATCH -o /ycga-gpfs/project/caccone/lvc26/probes/scripts/bowtie_logs/bowtie_index_%A_%a.o.txt
#SBATCH -e /ycga-gpfs/project/caccone/lvc26/probes/scripts/bowtie_logs/bowtie_index_%A_%a.ERROR.txt

# go to dir
cd /ycga-gpfs/project/caccone/lvc26/probes/scripts/bowtie_logs

# rename the log files
while IFS=: read -r task_id gname; do
  for ext in o.txt ERROR.txt; do
    for old_file in bowtie_index_*_${task_id}.${ext}; do
      if [ -e "$old_file" ]; then
        new_file="bowtie_index_${gname}.${ext}"
        mv "$old_file" "$new_file"
      fi
    done
  done
done < "bowtie_index_task_id_mapping.txt"
```

Job wrapper for bwa index jobs

```
#!/bin/sh
#SBATCH --mail-type=END,FAIL
#SBATCH --mail-user=luciano.cosme@yale.edu
#SBATCH --partition=day
#SBATCH --ntasks=1
#SBATCH --cpus-per-task=1
#SBATCH --mem-per-cpu=1G
#SBATCH --time=12:00:00
#SBATCH --job-name=bowtie_indexW
#SBATCH -o /ycga-gpfs/project/caccone/lvc26/probes/scripts/bowtie_logs/bowtie_indexW_%A_%a.o.txt
#SBATCH -e /ycga-gpfs/project/caccone/lvc26/probes/scripts/bowtie_logs/bowtie_indexW_%A_%a.ERROR.txt

# go to dir
cd /gpfs/ycga/project/caccone/lvc26/probes/scripts

# Submit job 2a
job_A_id=$(sbatch 2a.bowtie_index.sh | awk '{print $4}')

# Submit job 2b with a dependency on the successful completion of job 2a
job_B_id=$(sbatch --dependency=afterok:${job_A_id} 2b.bowtie_index.sh | awk '{print $4}')
```

Save the RMarkdown before running the code below:

```
# Set the R Markdown file path
rmd_file_path <- "scripts/RMarkdown_files/09.mapping_probes.Rmd"

# Set the output directory
output_directory <- "output/probes/scripts"

# Write the content of the chunk with the specified label
write_chunk(rmd_file_path, "2a.bowtie_index.sh", output_directory, "2a.bowtie_index.sh")
```

```
## Bash script saved as: output/probes/scripts/2a.bowtie_index.sh
```

```
write_chunk(rmd_file_path, "2b.bowtie_index.sh", output_directory, "2b.bowtie_index.sh")
```

```
## Bash script saved as: output/probes/scripts/2b.bowtie_index.sh
```

```
write_chunk(rmd_file_path, "2.bowtie_index.sh", output_directory, "2.bowtie_index.sh")
```

```
## Bash script saved as: output/probes/scripts/2.bowtie_index.sh
```

Transfer files to cluster

```
# next we can download the files to our computer, run the command below in your computer, not in the cluster.
rsync -chavzP --stats /Users/lucianocosme/Library/CloudStorage/Dropbox/Albopictus/manuscript_chip/data/no_autogenous/albo_chip/output/probes/scripts lvc26@mccleary.ycrc.yale.edu:/ycga-gpfs/project/caccone/lvc26/probes
```

Submit jobs

```
cd /gpfs/ycga/project/caccone/lvc26/probes/scripts;
sbatch 1.bwa_index.sh;
# Submitted batch job 450295
sbatch 2.bowtie_index.sh
# Submitted batch job 450499
```

Check job progress

```
sacct -j 450499 --format=JobID,JobName,Partition,AllocCPUS,State,ExitCode,Start,End,Elapsed,UserCPU,SystemCPU,TotalCPU,MaxRSS
```

## 5. Mapping probe sequences with BWA on the cluster

We will use the HPC cluster to speed up our alignments.

### 5.1 Map with the default bwa mem parameters

Check the manual at https://bio-bwa.sourceforge.net/bwa.shtml

bwa mem [-aCHMpP] [-t nThreads] [-k minSeedLen] [-w bandWidth] [-d
zDropoff] [-r seedSplitRatio] [-c maxOcc] [-A matchScore] [-B mmPenalty]
[-O gapOpenPen] [-E gapExtPen] [-L clipPen] [-U unpairPen] [-R RGline]
[-v verboseLevel] db.prefix reads.fq [mates.fq] Align 70bp-1Mbp query
sequences with the BWA-MEM algorithm. Briefly, the algorithm works by
seeding alignments with maximal exact matches (MEMs) and then extending
seeds with the affine-gap Smith-Waterman algorithm (SW).

Usage: bwa mem [options]  <in1.fq> [in2.fq]

Algorithm options:

```
   -t INT        number of threads [1]
   -k INT        minimum seed length [19]
   -w INT        band width for banded alignment [100]
   -d INT        off-diagonal X-dropoff [100]
   -r FLOAT      look for internal seeds inside a seed longer than {-k} * FLOAT [1.5]
   -y INT        seed occurrence for the 3rd round seeding [20]
   -c INT        skip seeds with more than INT occurrences [500]
   -D FLOAT      drop chains shorter than FLOAT fraction of the longest overlapping chain [0.50]
   -W INT        discard a chain if seeded bases shorter than INT [0]
   -m INT        perform at most INT rounds of mate rescues for each read [50]
   -S            skip mate rescue
   -P            skip pairing; mate rescue performed unless -S also in use
```

Scoring options:

```
   -A INT        score for a sequence match, which scales options -TdBOELU unless overridden [1]
   -B INT        penalty for a mismatch [4]
   -O INT[,INT]  gap open penalties for deletions and insertions [6,6]
   -E INT[,INT]  gap extension penalty; a gap of size k cost '{-O} + {-E}*k' [1,1]
   -L INT[,INT]  penalty for 5'- and 3'-end clipping [5,5]
   -U INT        penalty for an unpaired read pair [17]

   -x STR        read type. Setting -x changes multiple parameters unless overridden [null]
                 pacbio: -k17 -W40 -r10 -A1 -B1 -O1 -E1 -L0  (PacBio reads to ref)
                 ont2d: -k14 -W20 -r10 -A1 -B1 -O1 -E1 -L0  (Oxford Nanopore 2D-reads to ref)
                 intractg: -B9 -O16 -L5  (intra-species contigs to ref)
```

Input/output options:

```
   -p            smart pairing (ignoring in2.fq)
   -R STR        read group header line such as '@RG\tID:foo\tSM:bar' [null]
   -H STR/FILE   insert STR to header if it starts with @; or insert lines in FILE [null]
   -o FILE       sam file to output results to [stdout]
   -j            treat ALT contigs as part of the primary assembly (i.e. ignore <idxbase>.alt file)
   -5            for split alignment, take the alignment with the smallest coordinate as primary
   -q            don't modify mapQ of supplementary alignments
   -K INT        process INT input bases in each batch regardless of nThreads (for reproducibility) []

   -v INT        verbosity level: 1=error, 2=warning, 3=message, 4+=debugging [3]
   -T INT        minimum score to output [30]
   -h INT[,INT]  if there are <INT hits with score >80% of the max score, output all in XA [5,200]
   -a            output all alignments for SE or unpaired PE
   -C            append FASTA/FASTQ comment to SAM output
   -V            output the reference FASTA header in the XR tag
   -Y            use soft clipping for supplementary alignments
   -M            mark shorter split hits as secondary

   -I FLOAT[,FLOAT[,INT[,INT]]]
                 specify the mean, standard deviation (10% of the mean if absent), max
                 (4 sigma from the mean if absent) and min of the insert size distribution.
                 FR orientation only. [inferred]
```

Note: Please read the man page for detailed description of the
command line and options.

First we will use the default options, except we will use -t 10
(threads) and -a (output all found alignments for single-end or unpaired
paired-end reads. These alignments will be flagged as secondary
alignments).

```
#!/bin/sh
#SBATCH --mail-type=END,FAIL
#SBATCH --mail-user=luciano.cosme@yale.edu
#SBATCH --partition=day
#SBATCH --ntasks=1
#SBATCH --cpus-per-task=10
#SBATCH --mem-per-cpu=5G
#SBATCH --time=12:00:00
#SBATCH --array=1-90
#SBATCH --job-name=bwa_default
#SBATCH -o /ycga-gpfs/project/caccone/lvc26/probes/scripts/bwa_logs/bwa_mem/default/bwa_default_%A_%a.o.txt
#SBATCH -e /ycga-gpfs/project/caccone/lvc26/probes/scripts/bwa_logs/bwa_mem/default/bwa_default_%A_%a.ERROR.txt

# load modules
module load BWA/0.7.17-GCCcore-10.2.0
module load SAMtools/1.16-GCCcore-10.2.0

# use my own installation
# bwa
# export PATH=/ycga-gpfs/project/caccone/lvc26/probes/bwa-0.7.17:$PATH
# samtools
# export LD_LIBRARY_PATH=/gpfs/ycga/project/caccone/lvc26/probes/ncurses-6.3/lib:$LD_LIBRARY_PATH
# export PATH=/gpfs/ycga/project/caccone/lvc26/probes/ncurses-6.3:$PATH
# export PATH=/ycga-gpfs/project/caccone/lvc26/probes/samtools-1.13:$PATH

# go to dir
cd /ycga-gpfs/project/caccone/lvc26/probes/scripts

# Set job name, output, and error environment variables
genomes="/ycga-gpfs/project/caccone/lvc26/probes/scripts/genomes.txt"
gnames="/ycga-gpfs/project/caccone/lvc26/probes/scripts/genome_names.txt"
sequences="/ycga-gpfs/project/caccone/lvc26/probes/scripts/sequences.txt"
snames="/ycga-gpfs/project/caccone/lvc26/probes/scripts/sequence_names.txt"

threads=$SLURM_CPUS_PER_TASK

genomes_count=$(wc -l < "$genomes")
sequences_count=$(wc -l < "$sequences")

genome_idx=$((($SLURM_ARRAY_TASK_ID - 1) % $genomes_count + 1))
sequence_idx=$((($SLURM_ARRAY_TASK_ID - 1) / $genomes_count + 1))

genome=$(sed -n "${genome_idx}p" "$genomes" | awk '{print $1}')
gname=$(sed -n "${genome_idx}p" "$gnames" | awk '{print $1}')
sequence=$(sed -n "${sequence_idx}p" "$sequences" |  awk '{print $1}')
sname=$(sed -n "${sequence_idx}p" "$snames" | awk '{print $1}')

# sanity checks
echo "SLURM_ARRAY_TASK_ID: $SLURM_ARRAY_TASK_ID"
echo "Genome file: $genome"
echo "Genome name: $gname"
echo "Sequence file: $sequence"
echo "Sequence name: $sname"

# set output directory
output_dir="/ycga-gpfs/project/caccone/lvc26/probes/bwa_mem_default_crams/"

# map with bwa mem, convert to cram, sort, and index cram files
bwa mem -t 10 -a $genome $sequence | samtools view -@ 10 -C -T $genome - | samtools sort -m 1G -@ 10 -O CRAM -o $output_dir$gname\.$sname\.cram - && samtools index $output_dir$gname\.$sname\.cram

# create file that maps the task ID to the genome and sequence names
gname=$(sed -n "${genome_idx}p" /ycga-gpfs/project/caccone/lvc26/probes/scripts/genome_names.txt | awk '{print $1}')
sname=$(sed -n "${sequence_idx}p" /ycga-gpfs/project/caccone/lvc26/probes/scripts/sequence_names.txt | awk '{print $1}')
echo "$SLURM_ARRAY_TASK_ID:$gname:$sname" >> /ycga-gpfs/project/caccone/lvc26/probes/scripts/bwa_logs/bwa_mem/default/bwa_default_task_id_mapping.txt
```

Update logs

```
#!/bin/sh
#SBATCH --mail-type=END,FAIL
#SBATCH --mail-user=luciano.cosme@yale.edu
#SBATCH --partition=day
#SBATCH --ntasks=1
#SBATCH --cpus-per-task=1
#SBATCH --mem-per-cpu=5G
#SBATCH --time=12:00:00
#SBATCH --job-name=bwa_defaultL
#SBATCH -o /ycga-gpfs/project/caccone/lvc26/probes/scripts/bwa_logs/bwa_mem/default/bwa_defaultL_%A_%a.o.txt
#SBATCH -e /ycga-gpfs/project/caccone/lvc26/probes/scripts/bwa_logs/bwa_mem/default/bwa_defaultL_%A_%a.ERROR.txt

# go to dir
cd /ycga-gpfs/project/caccone/lvc26/probes/scripts/bwa_logs/bwa_mem/default

# rename the log files
while IFS=: read -r task_id gname sname; do
  for ext in o.txt ERROR.txt; do
    for old_file in bwa_default_*_${task_id}.${ext}; do
      if [ -e "$old_file" ]; then
        new_file="bwa_default_${gname}_${sname}.${ext}"
        mv "$old_file" "$new_file"
      fi
    done
  done
done < "/ycga-gpfs/project/caccone/lvc26/probes/scripts/bwa_logs/bwa_mem/default/bwa_default_task_id_mapping.txt"
```

Script to parse the cram files

```
#!/bin/bash

cram=$1
task_id=$2

# Get the name of the cram file
file_name=$(basename ${cram} .cram)
#   ____________________________________________________________________________
#   1. sequences with zero mismatch alignments                              ####
#   a. count
zero_mismatch_count=$(samtools view -@ 10 -q 30 ${cram} | awk '{ for (i=1; i<=NF; i++) { if ($i ~ /^MD:Z:/) { md_tag=$i } } } { if (md_tag ~ /^MD:Z:[0-9]+$/) { print } }' | wc -l)
#   b. save the sequence IDs to file
samtools view -@ 10 -q 30 ${cram} | awk '{ for (i=1; i<=NF; i++) { if ($i ~ /^MD:Z:/) { md_tag=$i } } } { if (md_tag ~ /^MD:Z:[0-9]+$/) { print $1 } }' > "/ycga-gpfs/project/caccone/lvc26/probes/bwa_mem_default_results/${file_name}_zero_mismatch_ids.txt"

#   ____________________________________________________________________________
#   2. sequences with one mismatch alignments                               ####
#   a. count
one_mismatch_count=$(samtools view -@ 10 -q 30 ${cram} | awk '{ for (i=1; i<=NF; i++) { if ($i ~ /^MD:Z:/) { md_tag=$i } } } { if (md_tag ~ /^MD:Z:[0-9]+[ACTG][0-9]*$/) { print } }' | wc -l)
#   b. save the sequence IDs to file
samtools view -@ 10 -q 30 ${cram} | awk '{ for (i=1; i<=NF; i++) { if ($i ~ /^MD:Z:/) { md_tag=$i } } } { if (md_tag ~ /^MD:Z:[0-9]+[ACTG][0-9]*$/) { print $1 } }' > "/ycga-gpfs/project/caccone/lvc26/probes/bwa_mem_default_results/${file_name}_one_mismatch_ids.txt"
#   c. save the sequence IDs with one mismatch, mismatch position, and SNP position to a file
# save the sequence IDs with one mismatch, mismatch position, and SNP position to a file
samtools view -@ 10 -q 30 ${cram} | awk '{
  start=$4; end=$4+length($10)-1;
  snp=start+calculate_snp_position($6, 35);
  md_tag=""; cigar=$6;
  strand=and($2, 16) ? "-" : "+";
  for (i=1; i<=NF; i++) { if ($i ~ /^MD:Z:/) { md_tag=$i } }
  if (md_tag ~ /^MD:Z:([0-9]+)[ACTG][0-9]*$/) {
    md_tag_split = split(md_tag, md_parts, ":");
    md_value = md_parts[3];
    split(md_value, mismatch_parts, /[ACTG]/);
    mismatch_position = mismatch_parts[1] + 1;
    print $1, mismatch_position, snp;
  }
}
function calculate_snp_position(cigar, snp_offset) {
  num_matched_bases = 0;
  num_chars = split(cigar, chars, "");
  current_num = "";
  for (i = 1; i <= num_chars; ++i) {
      if (chars[i] ~ /[0-9]/) {
          current_num = current_num chars[i];
      } else {
          if (chars[i] == "M") {
              num_matched_bases += strtonum(current_num);
              if (num_matched_bases >= snp_offset) {
                  return snp_offset - (num_matched_bases - strtonum(current_num));
              }
          }
          current_num = "";
      }
  }
  return snp_offset;
}' > "/ycga-gpfs/project/caccone/lvc26/probes/bwa_mem_default_results/${file_name}_one_mismatch_positions.txt"


#   ____________________________________________________________________________
#   3. sequences with alignments with quality > 30                          ####
#   a. count
high_quality_count=$(samtools view -@ 10 -q 30 ${cram} | wc -l)

#   ____________________________________________________________________________
#   4. sequences with unique mapping                                        ####
#   a. count
unique_count=$(samtools view -@ 10 -q 30 ${cram} | awk '{ if (!and($2, 0x100) && !and($2, 0x800) && !and($2, 0x400)) { if ($1 in seen) { next } else { seen[$1] = 1; count++ } } } END { print count }')
#   b. save the sequence IDs to a file
samtools view -@ 10 -q 30 ${cram} | awk '{ if (!and($2, 0x100) && !and($2, 0x800) && !and($2, 0x400)) { if ($1 in seen) { next } else { seen[$1] = 1; print $1 } } }' > "/ycga-gpfs/project/caccone/lvc26/probes/bwa_mem_default_results/${file_name}_unique_mapping_ids.txt"

#   ____________________________________________________________________________
#   5. sequences with alignments with zero mismatches                       ####
#   a. count 
unique_count_no_mismatches=$(samtools view -@ 10 -F 384 -q 30 ${cram} | awk '{ if ($6 !~ /^[0-9]+M$/) { next } } { if ($1 in seen) { next } else { seen[$1] = 1; mdtag=""; for (i=12; i<=NF; i++) { if ($i ~ /^MD:Z:/) { mdtag=$i } } if (mdtag == "" || mdtag ~ /^MD:Z:[0-9]+$/) { print $1; next } } }' | sort | uniq | wc -l)
#   b. save the sequence IDs to a file
samtools view -@ 10 -F 384 -q 30 ${cram} | awk '{ if ($6 !~ /^[0-9]+M$/) { next } } { if ($1 in seen) { next } else { seen[$1] = 1; mdtag=""; for (i=12; i<=NF; i++) { if ($i ~ /^MD:Z:/) { mdtag=$i } } if (mdtag == "" || mdtag ~ /^MD:Z:[0-9]+$/) { print $1; next } } }' | sort | uniq > "/ycga-gpfs/project/caccone/lvc26/probes/bwa_mem_default_results/${file_name}_unique_no_mismatches_ids.txt"

#   ____________________________________________________________________________
#   6. sequences with multiple mappings                                     ####
#   a. count 
multiple_count=$(samtools view -@ 10 -F 0x900 ${cram} | awk '$5 < 20 {print}' | wc -l)
#   b. save the sequence IDs to a file
samtools view -@ 10 -F 0x900 ${cram} | awk '$5 < 20 {print}' "/ycga-gpfs/project/caccone/lvc26/probes/bwa_mem_default_results/${file_name}_multiple_alignments_ids.txt"

#   ____________________________________________________________________________
#   7. sequences with secondary alignments                                  ####
#   a. count 
secondary_count=$(samtools view -@ 10 ${cram} |  awk '$2 == 256' | wc -l)
#   b. save the sequence IDs to a file
samtools view -@ 10 ${cram} |  awk '$2 == 256' "/ycga-gpfs/project/caccone/lvc26/probes/bwa_mem_default_results/${file_name}_secondary_alignments_ids.txt"

#   ____________________________________________________________________________
#   8. sequences with supplementary alignments                              ####
#   a. count 
supplementary_count=$(samtools view -@ 10 -f 0x800 -F 0x100 ${cram} | wc -l)


#   ____________________________________________________________________________
#   9. create output file for each cram file                                ####
#   a. extract the name, mapping start position, mapping end position, SNP position, strand, mapping quality, etc.
echo "Read_Name Ref_Name Start End SNP Strand Mapping_Quality Mismatches Indels" > "/ycga-gpfs/project/caccone/lvc26/probes/bwa_mem_default_results/${file_name}.txt"
# wrote Read_Name Ref_Name Start End SNP_position Strand Mapping_Quality Mismatches Indels
samtools view -@ 10 ${cram} | awk '{
  start=$4; end=$4+length($10)-1;
  snp=start+calculate_snp_position($6, 35);
  md_tag=""; cigar=$6;
  strand=and($2, 16) ? "-" : "+";
  for (i=1; i<=NF; i++) { if ($i ~ /^MD:Z:/) { md_tag=$i } }
  mismatches=gsub(/[ACGTN^]/, "", md_tag);
  indels=gsub(/[ID]/, "", cigar);
  print $1,$3,start,end,snp,strand,$5,mismatches,indels
}
function calculate_snp_position(cigar, snp_offset) {
  num_matched_bases = 0;
  num_chars = split(cigar, chars, "");
  current_num = "";
  for (i = 1; i <= num_chars; ++i) {
      if (chars[i] ~ /[0-9]/) {
          current_num = current_num chars[i];
      } else {
          if (chars[i] == "M") {
              num_matched_bases += strtonum(current_num);
              if (num_matched_bases >= snp_offset) {
                  return snp_offset - (num_matched_bases - strtonum(current_num));
              }
          }
          current_num = "";
      }
  }
  return snp_offset;
}' >> "/ycga-gpfs/project/caccone/lvc26/probes/bwa_mem_default_results/${file_name}.txt"


#   ____________________________________________________________________________
#   10. write counts to file                                                ####
echo "File: $file_name" >> "/ycga-gpfs/project/caccone/lvc26/probes/bwa_mem_default_results/${task_id}_bwa_default_results.txt"
echo "Zero mismatches: $zero_mismatch_count" >> "/ycga-gpfs/project/caccone/lvc26/probes/bwa_mem_default_results/${task_id}_bwa_default_results.txt"
echo "One mismatch: $one_mismatch_count" >> "/ycga-gpfs/project/caccone/lvc26/probes/bwa_mem_default_results/${task_id}_bwa_default_results.txt"
echo "Unique mappings: $unique_count" >> "/ycga-gpfs/project/caccone/lvc26/probes/bwa_mem_default_results/${task_id}_bwa_default_results.txt"
echo "Unique map 0 mismatches: $unique_count_no_mismatches" >> "/ycga-gpfs/project/caccone/lvc26/probes/bwa_mem_default_results/${task_id}_bwa_default_results.txt"
echo "High quality: $high_quality_count" >> "/ycga-gpfs/project/caccone/lvc26/probes/bwa_mem_default_results/${task_id}_bwa_default_results.txt"
echo "Multiple mappings: $multiple_count" >> "/ycga-gpfs/project/caccone/lvc26/probes/bwa_mem_default_results/${task_id}_bwa_default_results.txt"
echo "Secondary alignments: $secondary_count" >> "/ycga-gpfs/project/caccone/lvc26/probes/bwa_mem_default_results/${task_id}_bwa_default_results.txt"
echo "Supplementary alignments: $supplementary_count" >> "/ycga-gpfs/project/caccone/lvc26/probes/bwa_mem_default_results/${task_id}_bwa_default_results.txt"
```

Create a submission script Note: if you change –cpus-per-task=10, you
need to update bwa\_analysis\_default.sh (samtools view -@ 10)

```
#!/bin/sh
#SBATCH --mail-type=END,FAIL
#SBATCH --mail-user=luciano.cosme@yale.edu
#SBATCH --partition=day
#SBATCH --ntasks=1
#SBATCH --cpus-per-task=10
#SBATCH --mem-per-cpu=1G
#SBATCH --time=12:00:00
#SBATCH --array=1-90
#SBATCH --job-name=bwa_parseA
#SBATCH -o /ycga-gpfs/project/caccone/lvc26/probes/scripts/bwa_logs/bwa_mem/default/bwa_parseA_%A_%a.o.txt
#SBATCH -e /ycga-gpfs/project/caccone/lvc26/probes/scripts/bwa_logs/bwa_mem/default/bwa_parseA_%A_%a.ERROR.txt

# go to dir
cd /ycga-gpfs/project/caccone/lvc26/probes/scripts

# load Samtools
module load SAMtools/1.16-GCCcore-10.2.0

crams=(/gpfs/ycga/project/caccone/lvc26/probes/bwa_mem_default_crams/*.cram)
cram=${crams[$SLURM_ARRAY_TASK_ID - 1]}
file_name=$(basename "${cram}" .cram)

# Add the task_id and cram name to the log file
echo "${SLURM_ARRAY_TASK_ID}:${file_name}" >> /ycga-gpfs/project/caccone/lvc26/probes/scripts/bwa_logs/bwa_mem/default/bwa_defaultA_task_id_mapping.txt

# make parsing script executable
chmod u+x bwa_mem_analysis_default.sh

# run the parsing script for each cram file
./bwa_mem_analysis_default.sh "${cram}" "${SLURM_ARRAY_TASK_ID}"
```

Update the parsing logs

```
#!/bin/sh
#SBATCH --mail-type=END,FAIL
#SBATCH --mail-user=luciano.cosme@yale.edu
#SBATCH --partition=day
#SBATCH --ntasks=1
#SBATCH --cpus-per-task=1
#SBATCH --mem-per-cpu=5G
#SBATCH --time=12:00:00
#SBATCH --job-name=bwa_defaultL
#SBATCH -o /ycga-gpfs/project/caccone/lvc26/probes/scripts/bwa_logs/bwa_mem/default/bwa_defaultAL_%A_%a.o.txt
#SBATCH -e /ycga-gpfs/project/caccone/lvc26/probes/scripts/bwa_logs/bwa_mem/default/bwa_defaultAL_%A_%a.ERROR.txt

# go to dir
cd /ycga-gpfs/project/caccone/lvc26/probes/scripts/bwa_logs/bwa_mem/default

# rename the log files
while IFS=: read -r task_id cram_name; do
  for ext in o.txt ERROR.txt; do
    for old_file in bwa_parseA_*_${task_id}.${ext}; do
      if [ -e "$old_file" ]; then
        new_file="bwa_parseA_${cram_name}.${ext}"
        mv "$old_file" "$new_file"
      fi
    done
  done
done < "/ycga-gpfs/project/caccone/lvc26/probes/scripts/bwa_logs/bwa_mem/default/bwa_defaultA_task_id_mapping.txt"
```

Now we can join all the outputs and create a table

```
#!/bin/sh
#SBATCH --mail-type=END,FAIL
#SBATCH --mail-user=luciano.cosme@yale.edu
#SBATCH --partition=day
#SBATCH --ntasks=1
#SBATCH --cpus-per-task=1
#SBATCH --mem-per-cpu=10G
#SBATCH --time=12:00:00
#SBATCH --job-name=bwa_combine
#SBATCH -o /ycga-gpfs/project/caccone/lvc26/probes/scripts/bwa_logs/bwa_mem/default/bwa_parseC_%A_%a.o.txt
#SBATCH -e /ycga-gpfs/project/caccone/lvc26/probes/scripts/bwa_logs/bwa_mem/default/bwa_parseC_%A_%a.ERROR.txt

cd /ycga-gpfs/project/caccone/lvc26/probes

# Remove any previous combined results file
rm -f /ycga-gpfs/project/caccone/lvc26/probes/bwa_mem_default_results/bwa_default_results_combined.txt

# Combine the individual result files into a single file
for result in /ycga-gpfs/project/caccone/lvc26/probes/bwa_mem_default_results/*_bwa_default_results.txt; do
  cat "${result}" >> /ycga-gpfs/project/caccone/lvc26/probes/bwa_mem_default_results/bwa_default_results_combined.txt
  echo "" >> /ycga-gpfs/project/caccone/lvc26/probes/bwa_mem_default_results/bwa_default_results_combined.txt
done

# make a table
awk 'BEGIN {
    print "file\tzero_mismatches\tone_mismatch\tunique_mappings\tunique_map_0_mismatches\thigh_quality\tmultiple_mappings\tsecondary_alignments\tsupplementary_alignments"
} {
    if (/^File:/) {file=$2}
    else if (/^Zero mismatches:/) {zero_mismatches=$3}
    else if (/^One mismatch:/) {one_mismatch=$3}
    else if (/^Unique mappings:/) {unique_mappings=$3}
    else if (/^Unique map 0 mismatches:/) {unique_map_0_mismatches=$5}
    else if (/^High quality:/) {high_quality=$3}
    else if (/^Multiple mappings:/) {multiple_mappings=$3}
    else if (/^Secondary alignments:/) {secondary_alignments=$3}
    else if (/^Supplementary alignments:/) {supplementary_alignments=$3; print file"\t"zero_mismatches"\t"one_mismatch"\t"unique_mappings"\t"unique_map_0_mismatches"\t"high_quality"\t"multiple_mappings"\t"secondary_alignments"\t"supplementary_alignments}
}' /ycga-gpfs/project/caccone/lvc26/probes/bwa_mem_default_results/bwa_default_results_combined.txt > /ycga-gpfs/project/caccone/lvc26/probes/bwa_mem_default_results/bwa_mem_default_results_table.txt
```

Job wrapper

```
#!/bin/sh
#SBATCH --mail-type=END,FAIL
#SBATCH --mail-user=luciano.cosme@yale.edu
#SBATCH --partition=day
#SBATCH --ntasks=1
#SBATCH --cpus-per-task=1
#SBATCH --mem-per-cpu=1G
#SBATCH --time=12:00:00
#SBATCH --job-name=bwa_defaultW
#SBATCH -o /ycga-gpfs/project/caccone/lvc26/probes/scripts/bwa_logs/bwa_mem/default/bwa_defaultW_%A_%a.o.txt
#SBATCH -e /ycga-gpfs/project/caccone/lvc26/probes/scripts/bwa_logs/bwa_mem/default/bwa_defaultW_%A_%a.ERROR.txt

# go to dir
cd /gpfs/ycga/project/caccone/lvc26/probes/scripts

# Submit job a
job_A_id=$(sbatch 3a.bwa_mem_default_mapping.sh | awk '{print $4}')

# Submit job b with a dependency on the successful completion of job a
job_B_id=$(sbatch --dependency=afterok:${job_A_id} 3b.bwa_mem_default_logs.sh | awk '{print $4}')

# Submit job c with a dependency on the successful completion of job b
job_C_id=$(sbatch --dependency=afterok:${job_B_id} 3c.bwa_mem_default_parse_crams.sh | awk '{print $4}')

# Submit job d with a dependency on the successful completion of job c
job_D_id=$(sbatch --dependency=afterok:${job_C_id} 3d.bwa_mem_default_update_parse_logs.sh | awk '{print $4}')

# Submit job e with a dependency on the successful completion of job d
sbatch --dependency=afterok:${job_D_id} 3e.bwa_mem_default_join_results.sh
```

Create dir

```
# create output directories 
dir_names <- c(
  "scripts/bwa_mem_scripts",
  "results_cluster/bwa",
  "results_cluster/bwa/bwa_mem_default_crams",
  "results_cluster/bwa/bwa_mem_default_results"
)

for (dir_name in dir_names) {
  here("output", "probes", dir_name) |>
    dir.create(recursive = TRUE, showWarnings = FALSE)
}
```

Save the batch scripts

```
# save the changes before running the code below

# Set the R Markdown file path
rmd_file_path <- "scripts/RMarkdown_files/12.mapping_probes_albopicuts.Rmd"

# Set the output directory
output_directory <- "output/probes/scripts/bwa_mem_scripts"


# source function to write chunks
source(
  here(
    "scripts", "analysis", "write_chunk.R")
)

# Write the content of the chunk with the specified label
write_chunk(rmd_file_path, "3a.bwa_mem_default_mapping.sh", output_directory, "3a.bwa_mem_default_mapping.sh")
```

```
## Bash script saved as: output/probes/scripts/bwa_mem_scripts/3a.bwa_mem_default_mapping.sh
```

```
write_chunk(rmd_file_path, "3b.bwa_mem_default_logs.sh", output_directory, "3b.bwa_mem_default_logs.sh")
```

```
## Bash script saved as: output/probes/scripts/bwa_mem_scripts/3b.bwa_mem_default_logs.sh
```

```
write_chunk(rmd_file_path, "3c.bwa_mem_default_parse_crams.sh", output_directory, "3c.bwa_mem_default_parse_crams.sh")
```

```
## Bash script saved as: output/probes/scripts/bwa_mem_scripts/3c.bwa_mem_default_parse_crams.sh
```

```
write_chunk(rmd_file_path, "3d.bwa_mem_default_update_parse_logs.sh", output_directory, "3d.bwa_mem_default_update_parse_logs.sh")
```

```
## Bash script saved as: output/probes/scripts/bwa_mem_scripts/3d.bwa_mem_default_update_parse_logs.sh
```

```
write_chunk(rmd_file_path, "3e.bwa_mem_default_join_results.sh", output_directory, "3e.bwa_mem_default_join_results.sh")
```

```
## Bash script saved as: output/probes/scripts/bwa_mem_scripts/3e.bwa_mem_default_join_results.sh
```

```
write_chunk(rmd_file_path, "bwa_mem_analysis_default.sh", output_directory, "bwa_mem_analysis_default.sh")
```

```
## Bash script saved as: output/probes/scripts/bwa_mem_scripts/bwa_mem_analysis_default.sh
```

```
write_chunk(rmd_file_path, "3.bwa_mem_default.sh", output_directory, "3.bwa_mem_default.sh")
```

```
## Bash script saved as: output/probes/scripts/bwa_mem_scripts/3.bwa_mem_default.sh
```

Transfer files to cluster

```
rsync -chavzP --stats --existing --ignore-non-existing /Users/lucianocosme/Library/CloudStorage/Dropbox/Albopictus/manuscript_chip/data/no_autogenous/albo_chip/output/probes/scripts/bwa_mem_scripts/* lvc26@mccleary.ycrc.yale.edu:/ycga-gpfs/project/caccone/lvc26/probes/scripts
```

Submit jobs

```
cd /gpfs/ycga/project/caccone/lvc26/probes/scripts;
sbatch 3.bwa_mem_default.sh
```

Check job progress

```
sacct -j 776580 --format=JobID,JobName,Partition,AllocCPUS,State,ExitCode,Start,End,Elapsed,UserCPU,SystemCPU,TotalCPU,MaxRSS
```

#### 5.1.1 Inspect the alignments

Create dir

```
# create output directories 
dir_names <- c(
  "results_cluster/bwa",
  "results_cluster/bwa/bwa_default_crams",
  "results_cluster/bwa/bwa_default_results",
  "results_cluster/bwa/bwa_seeds_crams",
  "results_cluster/bwa/bwa_seeds_results"
)

for (dir_name in dir_names) {
  here("output", "probes", dir_name) |>
    dir.create(recursive = TRUE, showWarnings = FALSE)
}
```

Download files from cluster

```
# next we can download the files to our computer, run the command below in your computer, not in the cluster
# crams
rsync -chavzP --stats lvc26@mccleary.ycrc.yale.edu:/ycga-gpfs/project/caccone/lvc26/probes/bwa_default_crams/ /Users/lucianocosme/Library/CloudStorage/Dropbox/Albopictus/manuscript_chip/data/no_autogenous/albo_chip/output/probes/results_cluster/bwa
```

Create interactive session on the cluster and load Samtools

```
# create interactive session with 1 CPU
salloc --partition devel --time=06:00:00 --nodes=1 --ntasks=1 --cpus-per-task=1 --mem-per-cpu=10Gb zsh

# load samtools
module load SAMtools/1.16-GCCcore-10.2.0
```

##### 5.1.1.1 AalbF3

Check the first 10 alignments

```
# on the cluster
samtools view /gpfs/ycga/project/caccone/lvc26/probes/bwa_default_crams/AalbF3.albopictus_Ref.cram 2>&1 | head -n 10
# AX-584593832_R    256 1.1 81217   0   37M34H  *   0   0   *   *   NM:i:0  MD:Z:37 AS:i:37
# AX-583656114_R    256 1.1 92786   0   71M *   0   0   *   *   NM:i:2  MD:Z:40A8T21    AS:i:61
# AX-583656114_F    272 1.1 92786   0   71M *   0   0   *   *   NM:i:2  MD:Z:40A8T21    AS:i:61
# AX-582033422_F    256 1.1 97460   0   71M *   0   0   *   *   NM:i:2  MD:Z:17A1G51    AS:i:61
# AX-582033422_R    272 1.1 97460   0   71M *   0   0   *   *   NM:i:2  MD:Z:17A1G51    AS:i:61
# AX-581444870_R    0   1.1 97821   10  71M *   0   0   AAGGAATTGTAGTGCGCGCGCGAATAGTGTAATAATTCGAGGTTAGAATAATTGTTAAAGAGAAACCTCTT *   AS:i:71 XS:i:66 MD:Z:71 NM:i:0
# AX-581444870_F    16  1.1 97821   10  71M *   0   0   AAGGAATTGTAGTGCGCGCGCGAATAGTGTAATAATTCGAGGTTAGAATAATTGTTAAAGAGAAACCTCTT *   AS:i:71 XS:i:66 MD:Z:71 NM:i:0
# AX-583250556_R    256 1.1 103230  0   71M *   0   0   *   *   NM:i:3  MD:Z:43A5T5T15  AS:i:56
# AX-583250556_F    272 1.1 103230  0   71M *   0   0   *   *   NM:i:3  MD:Z:43A5T5T15  AS:i:56
# AX-583033226_F    0   1.1 161694  60  71M *   0   0   GGGAAAAATCGGAGGAATTTAAATCAAAGTTTCCAGAAAAATTGTAATAATCTATACAATATTTCCAATAT *   AS:i:71 XS:i:43 MD:Z:71 NM:i:0
```

Check a cram file to see the quality scores

```
# on the cluster
# SNP = AX-585090020
# scaffold_name = "2.150"
# start_position = 22827
# $end_position = 22898
samtools view /gpfs/ycga/project/caccone/lvc26/probes/bwa_default_crams/AalbF3.albopictus_Ref.cram 2.150:22827-22898
# AX-585090020_R    0     2.150 22828   60  71M *   0   0   TTGTCAGATCTATTCGTCTGGTAGTTGTTGAACACGGCAAAGTCCTGTGAAAATACAATTAGCAATACGCA *   AS:i:71 XS:i:0  MD:Z:71 NM:i:0
# AX-585090020_F    16  2.150   22828   60  71M *   0   0   TTGTCAGATCTATTCGTCTGGTAGTTGTTGAACACGGCAAAGTCCTGTGAAAATACAATTAGCAATACGCA *   AS:i:71 XS:i:0  MD:Z:71 NM:i:0
```

Explanation of the alignment flags

Each line represents a single read, and the fields are tab-separated.
Here’s a breakdown of the fields in each line:

```
Column 1: Read ID (AX-585090020_R or AX-585090020_F, indicating the reverse and forward strands of the same read pair)
Column 2: Flags (16 in the second line indicates the read is the reverse complement of the reference sequence - AX-585090020_F)
Column 3: Reference sequence name or ID (2.150 in both lines, which likely refers to the chromosome or contig number)
Column 4: 1-based leftmost position of the read on the reference sequence (22828 in both lines)
Column 5: Mapping quality (60 in both lines, which is the Phred-scaled probability that the alignment is incorrect)
Column 6: CIGAR string (71M in both lines, indicating that the read is aligned as a perfect match for 71 bases)
Column 7: Reference sequence name or ID of the mate/second read (in this case, * since this read is not paired)
Column 8: 1-based leftmost position of the mate/second read (in this case, * since this read is not paired)
Column 9: Template length (in this case, 0 since this read is not paired)
Column 10: Read sequence (TTGTCAGATCTATTCGTCTGGTAGTTGTTGAACACGGCAAAGTCCTGTGAAAATACAATTAGCAATACGCA - BWA created the reverse complement of reverse read)
Column 11: Quality scores for the read sequence (in this case, * since quality scores are not provided in this file - they are fasta sequences and not fastq)
Column 12-14: Optional fields (AS:i:71 indicates the alignment score, XS:i:0 indicates the suboptimal alignment score, MD:Z:71 indicates the string for mismatching positions and their corresponding reference bases, and NM:i:0 indicates the number of mismatches in the alignment)
```

Note about paired reads: 1) They must be derived from opposite ends
of the same DNA fragment. This means that the distance between the two
reads should be consistent with the expected fragment size, and the
orientation of the reads should be consistent with the direction of the
fragment. 2) They must be in the same relative orientation as each other
with respect to the reference genome. This means that the forward read
should be in the 5’ to 3’ direction and the reverse read should be in
the 3’ to 5’ direction.

I have forward and reverse sequences in one file but they are not
paired.

Let’s check a sequence with the reference allele (compare the flags
from the alignment)

```
samtools view -h /gpfs/ycga/project/caccone/lvc26/probes/bwa_default_crams/AalbF3.albopictus_Ref_F.cram | awk -v id="AX-585090020_F" '$1 == id'
# AX-585090020_F    16  2.150   22828   60  71M *   0   0   TTGTCAGATCTATTCGTCTGGTAGTTGTTGAACACGGCAAAGTCCTGTGAAAATACAATTAGCAATACGCA *   AS:i:71 XS:i:0  MD:Z:71 NM:i:0
# this read is the reverse complement of the reference sequence, is aligned perfectly for 71 bases, has no available mate/second read information, and has an alignment score of 71 with no mismatches in the alignment (as indicated by the MD:Z:71 and NM:i:0 fields). The XS:i:0 field indicates that this is the primary alignment for this read, rather than a secondary or alternative alignment.
```

Let’s check a sequence with the alternative allele

```
samtools view -h /gpfs/ycga/project/caccone/lvc26/probes/bwa_default_crams/AalbF3.albopictus_Alt_F.cram | awk -v id="AX-585090020_F" '$1 == id'
# AX-585090020_F    16  2.150   22828   60  71M *   0   0   TTGTCAGATCTATTCGTCTGGTAGTTGTTGAACACAGCAAAGTCCTGTGAAAATACAATTAGCAATACGCA *   AS:i:66 XS:i:0  MD:Z:35G35  NM:i:1
# this read is the reverse complement of the reference sequence, is aligned perfectly for 71 bases, has no available mate/second read information, and has an alignment score of 66 with one mismatch at position 36 (where the reference base is G and the read base is A).
```

Lets check a sequence with “N”

```
samtools view -h /gpfs/ycga/project/caccone/lvc26/probes/bwa_default_crams/AalbF3.albopictus_N_F.cram | awk -v id="AX-585090020_F" '$1 == id'
# AX-585090020_F    16  2.150   22828   60  71M *   0   0   TTGTCAGATCTATTCGTCTGGTAGTTGTTGAACACNGCAAAGTCCTGTGAAAATACAATTAGCAATACGCA *   AS:i:69 XS:i:0  MD:Z:35G35  NM:i:1
# this read is the reverse complement of the reference sequence, is aligned perfectly for 71 bases, has no available mate/second read information, and has an alignment score of 69 with one mismatch at position 36 (where the reference base is G and the read base is N). The MD:Z:35G35 field indicates that the first 35 bases of the read match perfectly to the reference, while the remaining 36th base is a mismatch. The NM:i:1 field confirms that there is one mismatch in the alignment. The XS:i:0 field indicates that this is the primary alignment for this read, rather than a secondary or alternative alignment.
```

##### 5.1.1.2 AalbF2

Now we can check the previous genome assembly, AalbF2 (2.45Gb), which
was used to create AalbF3 (1.45Gb).

Let’s check the sequence with the reference allele (compare the flags
from the alignment) using the NCBI genome

```
samtools view -h /gpfs/ycga/project/caccone/lvc26/probes/bwa_default_crams/AalbF2_ncbi.albopictus_Ref_F.cram | awk -v id="AX-585090020_F" '$1 == id'
# AX-585090020_F    0   NW_021837208    61127   45  71M *   0   0   TGCGTATTGCTAATTGTATTTTCACAGGACTTTGCCGTGTTCAACAACTACCAGACGAATAGATCTGACAA *   AS:i:71 XS:i:56 MD:Z:71 NM:i:0
# AX-585090020_F    256 NW_021838470    325982  0   47M1I23M    *   0   0   *   *   NM:i:3  MD:Z:1A7G60 AS:i:56

# the sequences is mapped to two different genomic positions:
# in the first line, the read is mapped to the reference sequence "NW_021837208" starting at position 61127 with a mapping quality of 45. The read is aligned perfectly for 71 bases with an alignment score of 71 and no mismatches.
# in the second line, the same read is mapped to a different reference sequence "NW_021838470" starting at position 325982 with a mapping quality of 0. The CIGAR string (47M1I23M) indicates that the read is aligned as a perfect match for the first 47 bases, followed by an insertion of 1 base, and then a match for the final 23 bases. The alignment score is 56 and there are 3 mismatches (indicated by the NM:i:3 field) at positions 1, 8, and 68, with a substitution of A, G, and G, respectively.
# not what we want. We will have to remove this SNP from our chip. We will check all the SNPs and remove all those with similar mapping.
```

Let’s check the sequence with the reference allele (compare the flags
from the alignment) using the VectorBase genome

```
samtools view -h /gpfs/ycga/project/caccone/lvc26/probes/bwa_default_crams/AalbF2_vb.albopictus_Ref_F.cram | awk -v id="AX-585090020_F" '$1 == id'
# AX-585090020_F    256 SWKY01000303    325982  0   47M1I23M    *   0   0   *   *   NM:i:3  MD:Z:1A7G60 AS:i:56
# AX-585090020_F    0   SWKY01001144    61127   45  71M *   0   0   TGCGTATTGCTAATTGTATTTTCACAGGACTTTGCCGTGTTCAACAACTACCAGACGAATAGATCTGACAA *   AS:i:71 XS:i:56 MD:Z:71 NM:i:0

# similar to NCBI genome, two mappings
# in the first line, the read is mapped to the reference sequence "SWKY01000303" starting at position 325982 with a mapping quality of 0. The CIGAR string (47M1I23M) indicates that the read is aligned as a perfect match for the first 47 bases, followed by an insertion of 1 base, and then a match for the final 23 bases. The alignment score is 56, and there are 3 mismatches (indicated by the NM:i:3 field) at positions 1, 8, and 68, with a substitution of A, G, and G, respectively.
# in the second line, the same read is mapped to a different reference sequence "SWKY01001144" starting at position 61127 with a mapping quality of 45. The read is aligned perfectly for 71 bases with an alignment score of 71 and no mismatches.
```

#### 5.1.2 Import bwa mem summary results

```
# next we can download the files to our computer, run the command below in your computer, not in the cluster
rsync -chavzP --stats lvc26@mccleary.ycrc.yale.edu:/ycga-gpfs/project/caccone/lvc26/probes/bwa_mem_default_results/bwa_mem_default_results_table.txt /Users/lucianocosme/Library/CloudStorage/Dropbox/Albopictus/manuscript_chip/data/no_autogenous/albo_chip/output/probes/results_cluster/bwa/bwa_mem_default_results
```

Import summary results of BWA mapping

```
# import data, create new columns, and sort
df_bwa_mem_default <-
  read_delim(
    here(
      "output", "probes", "results_cluster", "bwa", "bwa_mem_default_results", "bwa_mem_default_results_table.txt"
    ),
    delim = "\t",
    col_names = TRUE,
    show_col_types = FALSE
  ) |>
  separate(file, c("genome", "sequence"), sep = "\\.") |>
  arrange(match(genome, c("AalbF3", "AalbF2_ncbi", "AalbF2_vb", "AalbF1_ncbi", "AalbF1_vb", "AalbCell_ncbi", "AalbCell_vb", "AalbRamini", "AaegL5_ncbi", "AaegL5_vb")),
          match(sequence, c("albopictus_Ref", "albopictus_Ref_F", "albopictus_Ref_R", "albopictus_Alt", "albopictus_Alt_F", "albopictus_Alt_R", "albopictus_N", "albopictus_N_F", "albopictus_N_R"))) |>
  relocate(high_quality, .after = sequence)

# check the result
head(df_bwa_mem_default)
```

```
## # A tibble: 6 × 10
##   genome sequence      high_quality zero_mismatches one_mismatch unique_mappings
##   <chr>  <chr>                <dbl>           <dbl>        <dbl>           <dbl>
## 1 AalbF3 albopictus_R…       332228          332228            0          332228
## 2 AalbF3 albopictus_R…       166111          166111            0          166111
## 3 AalbF3 albopictus_R…       166117          166117            0          166117
## 4 AalbF3 albopictus_A…       328198               2       328196          328198
## 5 AalbF3 albopictus_A…       164095               1       164094          164095
## 6 AalbF3 albopictus_A…       164103               1       164102          164103
## # ℹ 4 more variables: unique_map_0_mismatches <dbl>, multiple_mappings <dbl>,
## #   secondary_alignments <dbl>, supplementary_alignments <dbl>
```

We can split the sequences by genome to make it easier to compare.
Besides, the metrics are created for all cram files, but some of them
are for sanity checks.

Ae. albopictus

```
# albopictus chip
albo_bwa_mem_default <-
  df_bwa_mem_default |>
  filter(str_detect(genome, "^Aalb")) |> # inside the filter() function, the str_detect() function from the stringr package (which is loaded automatically by dplyr) is used to detect which rows of the genome column start with "Aalb". The ^ character inside the regular expression ^Aalb specifies that the string should start with "Aalb". The filter() function then returns only the rows that match this condition.
  filter(str_detect(sequence, "^albopictus.*_F$")) |> # inside the filter() function, the str_detect() function is used to detect which rows of the sequence column start with "albopictus" and end with "_F". The ^ character inside the regular expression ^albopictus specifies that the string should start with "albopictus", and the $ character inside the regular expression .*_F$ specifies that the string should end with "_F". The filter() function then returns only the rows that match this condition.
  mutate(probe_allele = case_when(
    str_detect(sequence, "^albopictus_Ref_F$") ~ "Reference",
    str_detect(sequence, "^albopictus_Alt_F$") ~ "Alternative",
    str_detect(sequence, "^albopictus_N_F$") ~ "N"
  )) |>
  select(genome, probe_allele, everything()) |>
  select(-sequence)
# check the output
head(albo_bwa_mem_default)
```

```
## # A tibble: 6 × 10
##   genome  probe_allele high_quality zero_mismatches one_mismatch unique_mappings
##   <chr>   <chr>               <dbl>           <dbl>        <dbl>           <dbl>
## 1 AalbF3  Reference          166111          166111            0          166111
## 2 AalbF3  Alternative        164095               1       164094          164095
## 3 AalbF3  N                  164194               0       164194          164194
## 4 AalbF2… Reference          115033          115029            0          115033
## 5 AalbF2… Alternative         96906              20        96883           96906
## 6 AalbF2… N                  100803               0       100800          100803
## # ℹ 4 more variables: unique_map_0_mismatches <dbl>, multiple_mappings <dbl>,
## #   secondary_alignments <dbl>, supplementary_alignments <dbl>
```

We can make a table and save it

```
# Create a flextable from the data
ft_bwa_mem_default_albo <- regulartable(albo_bwa_mem_default)

# Export the flextable as a Word document
doc <- read_docx()
doc <- body_add_flextable(doc, value = ft_bwa_mem_default_albo)
print(doc, target = here("output", "probes", "results_cluster", "bwa","bwa_mem_default_results", "albopictus_bwa_mem_default_results_table.docx"))

# Display the flextable in the R Markdown document
ft_bwa_mem_default_albo
```

| genome | probe\_allele | high\_quality | zero\_mismatches | one\_mismatch | unique\_mappings | unique\_map\_0\_mismatches | multiple\_mappings | secondary\_alignments | supplementary\_alignments |
| --- | --- | --- | --- | --- | --- | --- | --- | --- | --- |
| AalbF3 | Reference | 166,111 | 166,111 | 0 | 166,111 | 166,111 | 8,621 | 22,070 | 0 |
| AalbF3 | Alternative | 164,095 | 1 | 164,094 | 164,095 | 1 | 8,782 | 39,032 | 0 |
| AalbF3 | N | 164,194 | 0 | 164,194 | 164,194 | 0 | 8,699 | 61,722 | 0 |
| AalbF2\_ncbi | Reference | 115,033 | 115,029 | 0 | 115,033 | 115,029 | 56,912 | 84,164 | 0 |
| AalbF2\_ncbi | Alternative | 96,906 | 20 | 96,883 | 96,906 | 20 | 66,540 | 115,993 | 0 |
| AalbF2\_ncbi | N | 100,803 | 0 | 100,800 | 100,803 | 0 | 61,887 | 148,532 | 0 |
| AalbF2\_vb | Reference | 115,032 | 115,028 | 0 | 115,032 | 115,028 | 56,912 | 84,093 | 0 |
| AalbF2\_vb | Alternative | 96,905 | 20 | 96,882 | 96,905 | 20 | 66,540 | 115,980 | 0 |
| AalbF2\_vb | N | 100,803 | 0 | 100,800 | 100,803 | 0 | 61,887 | 148,506 | 0 |
| AalbF1\_ncbi | Reference | 113,962 | 56,689 | 18,939 | 113,873 | 53,623 | 54,852 | 81,695 | 177 |
| AalbF1\_ncbi | Alternative | 105,778 | 6,526 | 53,857 | 105,695 | 5,016 | 59,453 | 89,835 | 149 |
| AalbF1\_ncbi | N | 106,494 | 327 | 54,983 | 106,410 | 0 | 58,561 | 120,831 | 152 |
| AalbF1\_vb | Reference | 113,962 | 56,689 | 18,939 | 113,873 | 53,623 | 54,852 | 81,695 | 177 |
| AalbF1\_vb | Alternative | 105,778 | 6,526 | 53,857 | 105,695 | 5,016 | 59,453 | 89,835 | 149 |
| AalbF1\_vb | N | 106,494 | 327 | 54,983 | 106,410 | 0 | 58,561 | 120,831 | 152 |
| AalbCell\_ncbi | Reference | 61,734 | 17,691 | 13,669 | 61,677 | 15,205 | 98,651 | 127,218 | 183 |
| AalbCell\_ncbi | Alternative | 55,438 | 4,991 | 14,932 | 55,398 | 3,877 | 102,692 | 128,574 | 147 |
| AalbCell\_ncbi | N | 55,345 | 228 | 14,603 | 55,298 | 0 | 102,741 | 167,040 | 156 |
| AalbCell\_vb | Reference | 61,734 | 17,691 | 13,669 | 61,677 | 15,205 | 98,651 | 127,218 | 183 |
| AalbCell\_vb | Alternative | 55,438 | 4,991 | 14,932 | 55,398 | 3,877 | 102,692 | 128,574 | 147 |
| AalbCell\_vb | N | 55,345 | 228 | 14,603 | 55,298 | 0 | 102,741 | 167,040 | 156 |
| AalbRamini | Reference | 102,505 | 35,493 | 23,409 | 102,269 | 29,610 | 66,238 | 82,493 | 379 |
| AalbRamini | Alternative | 99,646 | 9,652 | 38,675 | 99,409 | 6,437 | 68,214 | 80,777 | 371 |
| AalbRamini | N | 99,550 | 1,086 | 39,518 | 99,330 | 0 | 68,505 | 103,143 | 349 |

### 5.2 Map with the default bwa aln parameters

```
Usage:   bwa aln [options] <prefix> <in.fq>

Options: -n NUM    max #diff (int) or missing prob under 0.02 err rate (float) [0.04]
         -o INT    maximum number or fraction of gap opens [1]
         -e INT    maximum number of gap extensions, -1 for disabling long gaps [-1]
         -i INT    do not put an indel within INT bp towards the ends [5]
         -d INT    maximum occurrences for extending a long deletion [10]
         -l INT    seed length [32]
         -k INT    maximum differences in the seed [2]
         -m INT    maximum entries in the queue [2000000]
         -t INT    number of threads [1]
         -M INT    mismatch penalty [3]
         -O INT    gap open penalty [11]
         -E INT    gap extension penalty [4]
         -R INT    stop searching when there are >INT equally best hits [30]
         -q INT    quality threshold for read trimming down to 35bp [0]
         -f FILE   file to write output to instead of stdout
         -B INT    length of barcode
         -L        log-scaled gap penalty for long deletions
         -N        non-iterative mode: search for all n-difference hits (slooow)
         -I        the input is in the Illumina 1.3+ FASTQ-like format
         -b        the input read file is in the BAM format
         -0        use single-end reads only (effective with -b)
         -1        use the 1st read in a pair (effective with -b)
         -2        use the 2nd read in a pair (effective with -b)
         -Y        filter Casava-filtered sequences'
```

```
#!/bin/sh
#SBATCH --mail-type=END,FAIL
#SBATCH --mail-user=luciano.cosme@yale.edu
#SBATCH --partition=day
#SBATCH --ntasks=1
#SBATCH --cpus-per-task=10
#SBATCH --mem-per-cpu=5G
#SBATCH --time=12:00:00
#SBATCH --array=1-90
#SBATCH --job-name=bwa_default
#SBATCH -o /ycga-gpfs/project/caccone/lvc26/probes/scripts/bwa_logs/bwa_aln/default/bwa_default_%A_%a.o.txt
#SBATCH -e /ycga-gpfs/project/caccone/lvc26/probes/scripts/bwa_logs/bwa_aln/default/bwa_default_%A_%a.ERROR.txt

# load modules
module load BWA/0.7.17-GCCcore-10.2.0
module load SAMtools/1.16-GCCcore-10.2.0

# go to dir
cd /ycga-gpfs/project/caccone/lvc26/probes/scripts

# Set job name, output, and error environment variables
genomes="/ycga-gpfs/project/caccone/lvc26/probes/scripts/genomes.txt"
gnames="/ycga-gpfs/project/caccone/lvc26/probes/scripts/genome_names.txt"
sequences="/ycga-gpfs/project/caccone/lvc26/probes/scripts/sequences.txt"
snames="/ycga-gpfs/project/caccone/lvc26/probes/scripts/sequence_names.txt"

threads=$SLURM_CPUS_PER_TASK

genomes_count=$(wc -l < "$genomes")
sequences_count=$(wc -l < "$sequences")

genome_idx=$((($SLURM_ARRAY_TASK_ID - 1) % $genomes_count + 1))
sequence_idx=$((($SLURM_ARRAY_TASK_ID - 1) / $genomes_count + 1))

genome=$(sed -n "${genome_idx}p" "$genomes" | awk '{print $1}')
gname=$(sed -n "${genome_idx}p" "$gnames" | awk '{print $1}')
sequence=$(sed -n "${sequence_idx}p" "$sequences" |  awk '{print $1}')
sname=$(sed -n "${sequence_idx}p" "$snames" | awk '{print $1}')

# sanity checks
echo "SLURM_ARRAY_TASK_ID: $SLURM_ARRAY_TASK_ID"
echo "Genome file: $genome"
echo "Genome name: $gname"
echo "Sequence file: $sequence"
echo "Sequence name: $sname"

# set output directory
output_dir="/ycga-gpfs/project/caccone/lvc26/probes/bwa_aln_default_crams/"

# map with bwa aln, convert to cram, sort, and index cram files
bwa aln -t 10 $genome $sequence | bwa samse $genome - $sequence | samtools view -@ 10 -C -T $genome - | samtools sort -m 1G -@ 10 -O CRAM -o $output_dir$gname\.$sname\.cram - && samtools index $output_dir$gname\.$sname\.cram

# create file that maps the task ID to the genome and sequence names
gname=$(sed -n "${genome_idx}p" /ycga-gpfs/project/caccone/lvc26/probes/scripts/genome_names.txt | awk '{print $1}')
sname=$(sed -n "${sequence_idx}p" /ycga-gpfs/project/caccone/lvc26/probes/scripts/sequence_names.txt | awk '{print $1}')
echo "$SLURM_ARRAY_TASK_ID:$gname:$sname" >> /ycga-gpfs/project/caccone/lvc26/probes/scripts/bwa_logs/bwa_aln/default/bwa_default_task_id_mapping.txt
```

Update logs

```
#!/bin/sh
#SBATCH --mail-type=END,FAIL
#SBATCH --mail-user=luciano.cosme@yale.edu
#SBATCH --partition=day
#SBATCH --ntasks=1
#SBATCH --cpus-per-task=1
#SBATCH --mem-per-cpu=5G
#SBATCH --time=12:00:00
#SBATCH --job-name=bwa_defaultL
#SBATCH -o /ycga-gpfs/project/caccone/lvc26/probes/scripts/bwa_logs/bwa_aln/default/bwa_defaultL_%A_%a.o.txt
#SBATCH -e /ycga-gpfs/project/caccone/lvc26/probes/scripts/bwa_logs/bwa_aln/default/bwa_defaultL_%A_%a.ERROR.txt

# go to dir
cd /ycga-gpfs/project/caccone/lvc26/probes/scripts/bwa_logs/bwa_aln/default

# rename the log files
while IFS=: read -r task_id gname sname; do
  for ext in o.txt ERROR.txt; do
    for old_file in bwa_default_*_${task_id}.${ext}; do
      if [ -e "$old_file" ]; then
        new_file="bwa_default_${gname}_${sname}.${ext}"
        mv "$old_file" "$new_file"
      fi
    done
  done
done < "/ycga-gpfs/project/caccone/lvc26/probes/scripts/bwa_logs/bwa_aln/default/bwa_default_task_id_mapping.txt"
```

Script to parse the cram files

```
#!/bin/bash

cram=$1
task_id=$2

# Get the name of the cram file
file_name=$(basename ${cram} .cram)
#   ____________________________________________________________________________
#   1. sequences with zero mismatch alignments                              ####
#   a. count
zero_mismatch_count=$(samtools view -@ 10 -q 30 ${cram} | awk '{ for (i=1; i<=NF; i++) { if ($i ~ /^MD:Z:/) { md_tag=$i } } } { if (md_tag ~ /^MD:Z:[0-9]+$/) { print } }' | wc -l)
#   b. save the sequence IDs to file
samtools view -@ 10 -q 30 ${cram} | awk '{ for (i=1; i<=NF; i++) { if ($i ~ /^MD:Z:/) { md_tag=$i } } } { if (md_tag ~ /^MD:Z:[0-9]+$/) { print $1 } }' > "/ycga-gpfs/project/caccone/lvc26/probes/bwa_aln_default_results/${file_name}_zero_mismatch_ids.txt"

#   ____________________________________________________________________________
#   2. sequences with one mismatch alignments                               ####
#   a. count
one_mismatch_count=$(samtools view -@ 10 -q 30 ${cram} | awk '{ for (i=1; i<=NF; i++) { if ($i ~ /^MD:Z:/) { md_tag=$i } } } { if (md_tag ~ /^MD:Z:[0-9]+[ACTG][0-9]*$/) { print } }' | wc -l)
#   b. save the sequence IDs to file
samtools view -@ 10 -q 30 ${cram} | awk '{ for (i=1; i<=NF; i++) { if ($i ~ /^MD:Z:/) { md_tag=$i } } } { if (md_tag ~ /^MD:Z:[0-9]+[ACTG][0-9]*$/) { print $1 } }' > "/ycga-gpfs/project/caccone/lvc26/probes/bwa_aln_default_results/${file_name}_one_mismatch_ids.txt"
#   c. save the sequence IDs with one mismatch, mismatch position, and SNP position to a file
# save the sequence IDs with one mismatch, mismatch position, and SNP position to a file
samtools view -@ 10 -q 30 ${cram} | awk '{
  start=$4; end=$4+length($10)-1;
  snp=start+calculate_snp_position($6, 35);
  md_tag=""; cigar=$6;
  strand=and($2, 16) ? "-" : "+";
  for (i=1; i<=NF; i++) { if ($i ~ /^MD:Z:/) { md_tag=$i } }
  if (md_tag ~ /^MD:Z:([0-9]+)[ACTG][0-9]*$/) {
    md_tag_split = split(md_tag, md_parts, ":");
    md_value = md_parts[3];
    split(md_value, mismatch_parts, /[ACTG]/);
    mismatch_position = mismatch_parts[1] + 1;
    print $1, mismatch_position, snp;
  }
}
function calculate_snp_position(cigar, snp_offset) {
  num_matched_bases = 0;
  num_chars = split(cigar, chars, "");
  current_num = "";
  for (i = 1; i <= num_chars; ++i) {
      if (chars[i] ~ /[0-9]/) {
          current_num = current_num chars[i];
      } else {
          if (chars[i] == "M") {
              num_matched_bases += strtonum(current_num);
              if (num_matched_bases >= snp_offset) {
                  return snp_offset - (num_matched_bases - strtonum(current_num));
              }
          }
          current_num = "";
      }
  }
  return snp_offset;
}' > "/ycga-gpfs/project/caccone/lvc26/probes/bwa_aln_default_results/${file_name}_one_mismatch_positions.txt"


#   ____________________________________________________________________________
#   3. sequences with alignments with quality > 30                          ####
#   a. count
high_quality_count=$(samtools view -@ 10 -q 30 ${cram} | wc -l)

#   ____________________________________________________________________________
#   4. sequences with unique mapping                                        ####
#   a. count
unique_count=$(samtools view -@ 10 -q 30 ${cram} | awk '{ if (!and($2, 0x100) && !and($2, 0x800) && !and($2, 0x400)) { if ($1 in seen) { next } else { seen[$1] = 1; count++ } } } END { print count }')
#   b. save the sequence IDs to a file
samtools view -@ 10 -q 30 ${cram} | awk '{ if (!and($2, 0x100) && !and($2, 0x800) && !and($2, 0x400)) { if ($1 in seen) { next } else { seen[$1] = 1; print $1 } } }' > "/ycga-gpfs/project/caccone/lvc26/probes/bwa_aln_default_results/${file_name}_unique_mapping_ids.txt"

#   ____________________________________________________________________________
#   5. sequences with alignments with zero mismatches                       ####
#   a. count 
unique_count_no_mismatches=$(samtools view -@ 10 -F 384 -q 30 ${cram} | awk '{ if ($6 !~ /^[0-9]+M$/) { next } } { if ($1 in seen) { next } else { seen[$1] = 1; mdtag=""; for (i=12; i<=NF; i++) { if ($i ~ /^MD:Z:/) { mdtag=$i } } if (mdtag == "" || mdtag ~ /^MD:Z:[0-9]+$/) { print $1; next } } }' | sort | uniq | wc -l)
#   b. save the sequence IDs to a file
samtools view -@ 10 -F 384 -q 30 ${cram} | awk '{ if ($6 !~ /^[0-9]+M$/) { next } } { if ($1 in seen) { next } else { seen[$1] = 1; mdtag=""; for (i=12; i<=NF; i++) { if ($i ~ /^MD:Z:/) { mdtag=$i } } if (mdtag == "" || mdtag ~ /^MD:Z:[0-9]+$/) { print $1; next } } }' | sort | uniq > "/ycga-gpfs/project/caccone/lvc26/probes/bwa_aln_default_results/${file_name}_unique_no_mismatches_ids.txt"

#   ____________________________________________________________________________
#   6. sequences with multiple mappings                                     ####
#   a. count 
multiple_count=$(samtools view -@ 10 -F 0x900 ${cram} | awk '$5 < 20 {print}' | wc -l)
#   b. save the sequence IDs to a file
samtools view -@ 10 -F 0x900 ${cram} | awk '$5 < 20 {print}' "/ycga-gpfs/project/caccone/lvc26/probes/bwa_aln_default_results/${file_name}_multiple_alignments_ids.txt"

#   ____________________________________________________________________________
#   7. sequences with secondary alignments                                  ####
#   a. count 
secondary_count=$(samtools view -@ 10 ${cram} |  awk '$2 == 256' | wc -l)
#   b. save the sequence IDs to a file
samtools view -@ 10 ${cram} |  awk '$2 == 256' "/ycga-gpfs/project/caccone/lvc26/probes/bwa_aln_default_results/${file_name}_secondary_alignments_ids.txt"

#   ____________________________________________________________________________
#   8. sequences with supplementary alignments                              ####
#   a. count 
supplementary_count=$(samtools view -@ 10 -f 0x800 -F 0x100 ${cram} | wc -l)


#   ____________________________________________________________________________
#   9. create output file for each cram file                                ####
#   a. extract the name, mapping start position, mapping end position, SNP position, strand, mapping quality, etc.
echo "Read_Name Ref_Name Start End SNP Strand Mapping_Quality Mismatches Indels" > "/ycga-gpfs/project/caccone/lvc26/probes/bwa_aln_default_results/${file_name}.txt"
# wrote Read_Name Ref_Name Start End SNP_position Strand Mapping_Quality Mismatches Indels
samtools view -@ 10 ${cram} | awk '{
  start=$4; end=$4+length($10)-1;
  snp=start+calculate_snp_position($6, 35);
  md_tag=""; cigar=$6;
  strand=and($2, 16) ? "-" : "+";
  for (i=1; i<=NF; i++) { if ($i ~ /^MD:Z:/) { md_tag=$i } }
  mismatches=gsub(/[ACGTN^]/, "", md_tag);
  indels=gsub(/[ID]/, "", cigar);
  print $1,$3,start,end,snp,strand,$5,mismatches,indels
}
function calculate_snp_position(cigar, snp_offset) {
  num_matched_bases = 0;
  num_chars = split(cigar, chars, "");
  current_num = "";
  for (i = 1; i <= num_chars; ++i) {
      if (chars[i] ~ /[0-9]/) {
          current_num = current_num chars[i];
      } else {
          if (chars[i] == "M") {
              num_matched_bases += strtonum(current_num);
              if (num_matched_bases >= snp_offset) {
                  return snp_offset - (num_matched_bases - strtonum(current_num));
              }
          }
          current_num = "";
      }
  }
  return snp_offset;
}' >> "/ycga-gpfs/project/caccone/lvc26/probes/bwa_aln_default_results/${file_name}.txt"


#   ____________________________________________________________________________
#   10. write counts to file                                                ####
echo "File: $file_name" >> "/ycga-gpfs/project/caccone/lvc26/probes/bwa_aln_default_results/${task_id}_bwa_default_results.txt"
echo "Zero mismatches: $zero_mismatch_count" >> "/ycga-gpfs/project/caccone/lvc26/probes/bwa_aln_default_results/${task_id}_bwa_default_results.txt"
echo "One mismatch: $one_mismatch_count" >> "/ycga-gpfs/project/caccone/lvc26/probes/bwa_aln_default_results/${task_id}_bwa_default_results.txt"
echo "Unique mappings: $unique_count" >> "/ycga-gpfs/project/caccone/lvc26/probes/bwa_aln_default_results/${task_id}_bwa_default_results.txt"
echo "Unique map 0 mismatches: $unique_count_no_mismatches" >> "/ycga-gpfs/project/caccone/lvc26/probes/bwa_aln_default_results/${task_id}_bwa_default_results.txt"
echo "High quality: $high_quality_count" >> "/ycga-gpfs/project/caccone/lvc26/probes/bwa_aln_default_results/${task_id}_bwa_default_results.txt"
echo "Multiple mappings: $multiple_count" >> "/ycga-gpfs/project/caccone/lvc26/probes/bwa_aln_default_results/${task_id}_bwa_default_results.txt"
echo "Secondary alignments: $secondary_count" >> "/ycga-gpfs/project/caccone/lvc26/probes/bwa_aln_default_results/${task_id}_bwa_default_results.txt"
echo "Supplementary alignments: $supplementary_count" >> "/ycga-gpfs/project/caccone/lvc26/probes/bwa_aln_default_results/${task_id}_bwa_default_results.txt"
```

Create a submission script

```
#!/bin/sh
#SBATCH --mail-type=END,FAIL
#SBATCH --mail-user=luciano.cosme@yale.edu
#SBATCH --partition=day
#SBATCH --ntasks=1
#SBATCH --cpus-per-task=10
#SBATCH --mem-per-cpu=1G
#SBATCH --time=12:00:00
#SBATCH --array=1-90
#SBATCH --job-name=bwa_parseA
#SBATCH -o /ycga-gpfs/project/caccone/lvc26/probes/scripts/bwa_logs/bwa_aln/default/bwa_parseA_%A_%a.o.txt
#SBATCH -e /ycga-gpfs/project/caccone/lvc26/probes/scripts/bwa_logs/bwa_aln/default/bwa_parseA_%A_%a.ERROR.txt

# go to dir
cd /ycga-gpfs/project/caccone/lvc26/probes/scripts

# load Samtools
module load SAMtools/1.16-GCCcore-10.2.0

crams=(/gpfs/ycga/project/caccone/lvc26/probes/bwa_aln_default_crams/*.cram)
cram=${crams[$SLURM_ARRAY_TASK_ID - 1]}
file_name=$(basename "${cram}" .cram)

# Add the task_id and cram name to the log file
echo "${SLURM_ARRAY_TASK_ID}:${file_name}" >> /ycga-gpfs/project/caccone/lvc26/probes/scripts/bwa_logs/bwa_aln/default/bwa_defaultA_task_id_mapping.txt

# make parsing script executable
chmod u+x bwa_aln_analysis_default.sh

# run the parsing script for each cram file
./bwa_aln_analysis_default.sh "${cram}" "${SLURM_ARRAY_TASK_ID}"
```

Update the parsing logs

```
#!/bin/sh
#SBATCH --mail-type=END,FAIL
#SBATCH --mail-user=luciano.cosme@yale.edu
#SBATCH --partition=day
#SBATCH --ntasks=1
#SBATCH --cpus-per-task=1
#SBATCH --mem-per-cpu=5G
#SBATCH --time=12:00:00
#SBATCH --job-name=bwa_defaultL
#SBATCH -o /ycga-gpfs/project/caccone/lvc26/probes/scripts/bwa_logs/bwa_aln/default/bwa_defaultAL_%A_%a.o.txt
#SBATCH -e /ycga-gpfs/project/caccone/lvc26/probes/scripts/bwa_logs/bwa_aln/default/bwa_defaultAL_%A_%a.ERROR.txt

# go to dir
cd /ycga-gpfs/project/caccone/lvc26/probes/scripts/bwa_logs/bwa_aln/default

# rename the log files
while IFS=: read -r task_id cram_name; do
  for ext in o.txt ERROR.txt; do
    for old_file in bwa_parseA_*_${task_id}.${ext}; do
      if [ -e "$old_file" ]; then
        new_file="bwa_parseA_${cram_name}.${ext}"
        mv "$old_file" "$new_file"
      fi
    done
  done
done < "/ycga-gpfs/project/caccone/lvc26/probes/scripts/bwa_logs/bwa_aln/default/bwa_defaultA_task_id_mapping.txt"
```

Now we can join all the outputs and create a table

```
#!/bin/sh
#SBATCH --mail-type=END,FAIL
#SBATCH --mail-user=luciano.cosme@yale.edu
#SBATCH --partition=day
#SBATCH --ntasks=1
#SBATCH --cpus-per-task=1
#SBATCH --mem-per-cpu=10G
#SBATCH --time=12:00:00
#SBATCH --job-name=bwa_combine
#SBATCH -o /ycga-gpfs/project/caccone/lvc26/probes/scripts/bwa_logs/bwa_aln/default/bwa_parseC_%A_%a.o.txt
#SBATCH -e /ycga-gpfs/project/caccone/lvc26/probes/scripts/bwa_logs/bwa_aln/default/bwa_parseC_%A_%a.ERROR.txt

cd /ycga-gpfs/project/caccone/lvc26/probes

# Remove any previous combined results file
rm -f /ycga-gpfs/project/caccone/lvc26/probes/bwa_aln_default_results/bwa_default_results_combined.txt

# Combine the individual result files into a single file
for result in /ycga-gpfs/project/caccone/lvc26/probes/bwa_aln_default_results/*_bwa_default_results.txt; do
  cat "${result}" >> /ycga-gpfs/project/caccone/lvc26/probes/bwa_aln_default_results/bwa_default_results_combined.txt
  echo "" >> /ycga-gpfs/project/caccone/lvc26/probes/bwa_aln_default_results/bwa_default_results_combined.txt
done

# make a table
awk 'BEGIN {print "file\tzero_mismatches\tone_mismatch\tunique_mappings\tunique_map_0_mismatches\thigh_quality\tmultiple_mappings\tsecondary_alignments\tsupplementary_alignments"} {if (NR%9==1) {file=$2} else if (NR%9==2) {zero_mismatches=$3} else if (NR%9==3) {one_mismatch=$3} else if (NR%9==4) {unique_mappings=$3} else if (NR%9==5) {unique_map_0_mismatches=$5} else if (NR%9==6) {high_quality=$3} else if (NR%9==7) {multiple_mappings=$3} else if (NR%9==8) {secondary_alignments=$3} else if (NR%9==0) {supplementary_alignments=$3; print file"\t"zero_mismatches"\t"one_mismatch"\t"unique_mappings"\t"unique_map_0_mismatches"\t"high_quality"\t"multiple_mappings"\t"secondary_alignments"\t"supplementary_alignments} }' /ycga-gpfs/project/caccone/lvc26/probes/bwa_aln_default_results/bwa_default_results_combined.txt > /ycga-gpfs/project/caccone/lvc26/probes/bwa_aln_default_results/bwa_aln_default_results_table.txt
```

Job wrapper

```
#!/bin/sh
#SBATCH --mail-type=END,FAIL
#SBATCH --mail-user=luciano.cosme@yale.edu
#SBATCH --partition=day
#SBATCH --ntasks=1
#SBATCH --cpus-per-task=1
#SBATCH --mem-per-cpu=1G
#SBATCH --time=12:00:00
#SBATCH --job-name=bwa_defaultW
#SBATCH -o /ycga-gpfs/project/caccone/lvc26/probes/scripts/bwa_logs/bwa_aln/default/bwa_defaultW_%A_%a.o.txt
#SBATCH -e /ycga-gpfs/project/caccone/lvc26/probes/scripts/bwa_logs/bwa_aln/default/bwa_defaultW_%A_%a.ERROR.txt

# go to dir
cd /gpfs/ycga/project/caccone/lvc26/probes/scripts

# Submit job a
job_A_id=$(sbatch 4a.bwa_aln_default_mapping.sh | awk '{print $4}')

# Submit job b with a dependency on the successful completion of job a
job_B_id=$(sbatch --dependency=afterok:${job_A_id} 4b.bwa_aln_default_logs.sh | awk '{print $4}')

# Submit job c with a dependency on the successful completion of job b
job_C_id=$(sbatch --dependency=afterok:${job_B_id} 4c.bwa_aln_default_parse_crams.sh | awk '{print $4}')

# Submit job d with a dependency on the successful completion of job c
job_D_id=$(sbatch --dependency=afterok:${job_C_id} 4d.bwa_aln_default_update_parse_logs.sh | awk '{print $4}')

# Submit job e with a dependency on the successful completion of job d
sbatch --dependency=afterok:${job_D_id} 4e.bwa_aln_default_join_results.sh
```

Create dir

```
# create output directories 
dir_names <- c(
  "scripts/bwa_aln_scripts",
  "results_cluster/bwa/bwa_aln_default_crams",
  "results_cluster/bwa/bwa_aln_default_results"
)

for (dir_name in dir_names) {
  here("output", "probes", dir_name) |>
    dir.create(recursive = TRUE, showWarnings = FALSE)
}
```

Save the batch scripts

```
# save the changes before running the code below

# Set the R Markdown file path
rmd_file_path <- "scripts/RMarkdown_files/12.mapping_probes_albopicuts.Rmd"

# Set the output directory
output_directory <- "output/probes/scripts/bwa_aln_scripts"


# source function to write chunks
source(
  here(
    "scripts", "analysis", "write_chunk.R")
)

# Write the content of the chunk with the specified label
write_chunk(rmd_file_path, "4a.bwa_aln_default_mapping.sh", output_directory, "4a.bwa_aln_default_mapping.sh")
```

```
## Bash script saved as: output/probes/scripts/bwa_aln_scripts/4a.bwa_aln_default_mapping.sh
```

```
write_chunk(rmd_file_path, "4b.bwa_aln_default_logs.sh", output_directory, "4b.bwa_aln_default_logs.sh")
```

```
## Bash script saved as: output/probes/scripts/bwa_aln_scripts/4b.bwa_aln_default_logs.sh
```

```
write_chunk(rmd_file_path, "4c.bwa_aln_default_parse_crams.sh", output_directory, "4c.bwa_aln_default_parse_crams.sh")
```

```
## Bash script saved as: output/probes/scripts/bwa_aln_scripts/4c.bwa_aln_default_parse_crams.sh
```

```
write_chunk(rmd_file_path, "4d.bwa_aln_default_update_parse_logs.sh", output_directory, "4d.bwa_aln_default_update_parse_logs.sh")
```

```
## Bash script saved as: output/probes/scripts/bwa_aln_scripts/4d.bwa_aln_default_update_parse_logs.sh
```

```
write_chunk(rmd_file_path, "4e.bwa_aln_default_join_results.sh", output_directory, "4e.bwa_aln_default_join_results.sh")
```

```
## Bash script saved as: output/probes/scripts/bwa_aln_scripts/4e.bwa_aln_default_join_results.sh
```

```
write_chunk(rmd_file_path, "bwa_aln_analysis_default.sh", output_directory, "bwa_aln_analysis_default.sh")
```

```
## Bash script saved as: output/probes/scripts/bwa_aln_scripts/bwa_aln_analysis_default.sh
```

```
write_chunk(rmd_file_path, "4.bwa_aln_default.sh", output_directory, "4.bwa_aln_default.sh")
```

```
## Bash script saved as: output/probes/scripts/bwa_aln_scripts/4.bwa_aln_default.sh
```

Transfer files to cluster

```
# next we can download the files to our computer, run the command below in your computer, not in the cluster.
rsync -chavzP --stats /Users/lucianocosme/Library/CloudStorage/Dropbox/Albopictus/manuscript_chip/data/no_autogenous/albo_chip/output/probes/scripts/bwa_aln_scripts/* lvc26@mccleary.ycrc.yale.edu:/ycga-gpfs/project/caccone/lvc26/probes/scripts
```

Submit jobs

```
cd /gpfs/ycga/project/caccone/lvc26/probes/scripts;
sbatch 4.bwa_aln_default.sh
```

Check job progress

```
sacct -j 777036 --format=JobID,JobName,Partition,AllocCPUS,State,ExitCode,Start,End,Elapsed,UserCPU,SystemCPU,TotalCPU,MaxRSS
```

```
# next we can download the files to our computer, run the command below in your computer, not in the cluster
rsync -chavzP --stats lvc26@mccleary.ycrc.yale.edu:/ycga-gpfs/project/caccone/lvc26/probes/bwa_aln_default_results/bwa_aln_default_results_table.txt /Users/lucianocosme/Library/CloudStorage/Dropbox/Albopictus/manuscript_chip/data/no_autogenous/albo_chip/output/probes/results_cluster/bwa/bwa_aln_default_results
```

#### 5.1.2 Import bwa aln summary results

Import summary results of BWA mapping

```
# import data, create new columns, and sort
df_bwa_aln_default <-
  read_delim(
    here(
      "output", "probes", "results_cluster", "bwa", "bwa_aln_default_results", "bwa_aln_default_results_table.txt"
    ),
    delim = "\t",
    col_names = TRUE,
    show_col_types = FALSE
  ) |>
  separate(file, c("genome", "sequence"), sep = "\\.") |>
  arrange(match(genome, c("AalbF3", "AalbF2_ncbi", "AalbF2_vb", "AalbF1_ncbi", "AalbF1_vb", "AalbCell_ncbi", "AalbCell_vb", "AalbRamini", "AaegL5_ncbi", "AaegL5_vb")),
          match(sequence, c("albopictus_Ref", "albopictus_Ref_F", "albopictus_Ref_R", "albopictus_Alt", "albopictus_Alt_F", "albopictus_Alt_R", "albopictus_N", "albopictus_N_F", "albopictus_N_R"))) |>
  relocate(high_quality, .after = sequence)

# check the result
head(df_bwa_aln_default)
```

```
## # A tibble: 6 × 10
##   genome sequence      high_quality zero_mismatches one_mismatch unique_mappings
##   <chr>  <chr>                <dbl>           <dbl>        <dbl>           <dbl>
## 1 AalbF3 albopictus_R…       334970          334970            0          334970
## 2 AalbF3 albopictus_R…       167485          167485            0          167485
## 3 AalbF3 albopictus_R…       167485          167485            0          167485
## 4 AalbF3 albopictus_A…       334782               0       334782          334782
## 5 AalbF3 albopictus_A…       167391               0       167391          167391
## 6 AalbF3 albopictus_A…       167391               0       167391          167391
## # ℹ 4 more variables: unique_map_0_mismatches <dbl>, multiple_mappings <dbl>,
## #   secondary_alignments <dbl>, supplementary_alignments <dbl>
```

The BWA aln algorithm only reports the best alignment for each read,
which can be a single primary alignment or a primary alignment with
multiple equally good mappings (in which case, the primary alignment is
selected arbitrarily).

Ae. albopictus

```
# albopictus chip
albo_bwa_aln_default <-
  df_bwa_aln_default |>
  filter(str_detect(genome, "^Aalb")) |> # inside the filter() function, the str_detect() function from the stringr package (which is loaded automatically by dplyr) is used to detect which rows of the genome column start with "Aalb". The ^ character inside the regular expression ^Aalb specifies that the string should start with "Aalb". The filter() function then returns only the rows that match this condition.
  filter(str_detect(sequence, "^albopictus.*_F$")) |> # inside the filter() function, the str_detect() function is used to detect which rows of the sequence column start with "albopictus" and end with "_F". The ^ character inside the regular expression ^albopictus specifies that the string should start with "albopictus", and the $ character inside the regular expression .*_F$ specifies that the string should end with "_F". The filter() function then returns only the rows that match this condition.
  mutate(probe_allele = case_when(
    str_detect(sequence, "^albopictus_Ref_F$") ~ "Reference",
    str_detect(sequence, "^albopictus_Alt_F$") ~ "Alternative",
    str_detect(sequence, "^albopictus_N_F$") ~ "N"
  )) |>
  select(genome, probe_allele, everything()) |>
  select(-sequence, -secondary_alignments, -supplementary_alignments)
# check the output
head(albo_bwa_aln_default)
```

```
## # A tibble: 6 × 8
##   genome  probe_allele high_quality zero_mismatches one_mismatch unique_mappings
##   <chr>   <chr>               <dbl>           <dbl>        <dbl>           <dbl>
## 1 AalbF3  Reference          167485          167485            0          167485
## 2 AalbF3  Alternative        167391               0       167391          167391
## 3 AalbF3  N                  167434               0       167434          167434
## 4 AalbF2… Reference          122202          122199            0          122202
## 5 AalbF2… Alternative        114335               0       114333          114335
## 6 AalbF2… N                  117833               0       117831          117833
## # ℹ 2 more variables: unique_map_0_mismatches <dbl>, multiple_mappings <dbl>
```

We can make a table and save it

```
# Create a flextable from the data
ft_bwa_aln_default_albo <- regulartable(albo_bwa_aln_default)

# Export the flextable as a Word document
doc <- read_docx()
doc <- body_add_flextable(doc, value = ft_bwa_aln_default_albo)
print(doc, target = here("output", "probes", "results_cluster", "bwa","bwa_aln_default_results", "albopictus_bwa_aln_default_results_table.docx"))

# Display the flextable in the R Markdown document
ft_bwa_aln_default_albo
```

| genome | probe\_allele | high\_quality | zero\_mismatches | one\_mismatch | unique\_mappings | unique\_map\_0\_mismatches | multiple\_mappings |
| --- | --- | --- | --- | --- | --- | --- | --- |
| AalbF3 | Reference | 167,485 | 167,485 | 0 | 167,485 | 167,485 | 4,702 |
| AalbF3 | Alternative | 167,391 | 0 | 167,391 | 167,391 | 0 | 4,760 |
| AalbF3 | N | 167,434 | 0 | 167,434 | 167,434 | 0 | 4,739 |
| AalbF2\_ncbi | Reference | 122,202 | 122,199 | 0 | 122,202 | 122,199 | 34,505 |
| AalbF2\_ncbi | Alternative | 114,335 | 0 | 114,333 | 114,335 | 0 | 38,855 |
| AalbF2\_ncbi | N | 117,833 | 0 | 117,831 | 117,833 | 0 | 39,163 |
| AalbF2\_vb | Reference | 122,202 | 122,199 | 0 | 122,202 | 122,199 | 34,505 |
| AalbF2\_vb | Alternative | 114,335 | 0 | 114,333 | 114,335 | 0 | 38,855 |
| AalbF2\_vb | N | 117,833 | 0 | 117,831 | 117,833 | 0 | 39,163 |
| AalbF1\_ncbi | Reference | 99,429 | 56,630 | 18,064 | 99,429 | 55,761 | 56,507 |
| AalbF1\_ncbi | Alternative | 93,965 | 5,411 | 58,052 | 93,965 | 5,150 | 59,624 |
| AalbF1\_ncbi | N | 91,743 | 24 | 59,178 | 91,743 | 0 | 62,854 |
| AalbF1\_vb | Reference | 99,429 | 56,630 | 18,064 | 99,429 | 55,761 | 56,507 |
| AalbF1\_vb | Alternative | 93,965 | 5,411 | 58,052 | 93,965 | 5,150 | 59,624 |
| AalbF1\_vb | N | 91,743 | 24 | 59,178 | 91,743 | 0 | 62,854 |
| AalbCell\_ncbi | Reference | 59,704 | 17,538 | 16,657 | 59,704 | 16,875 | 80,135 |
| AalbCell\_ncbi | Alternative | 53,587 | 4,521 | 18,968 | 53,587 | 4,300 | 84,047 |
| AalbCell\_ncbi | N | 50,150 | 18 | 18,632 | 50,150 | 0 | 89,822 |
| AalbCell\_vb | Reference | 59,704 | 17,538 | 16,657 | 59,704 | 16,875 | 80,135 |
| AalbCell\_vb | Alternative | 53,587 | 4,521 | 18,968 | 53,587 | 4,300 | 84,047 |
| AalbCell\_vb | N | 50,150 | 18 | 18,632 | 50,150 | 0 | 89,822 |
| AalbRamini | Reference | 80,151 | 31,542 | 20,880 | 80,151 | 30,758 | 81,794 |
| AalbRamini | Alternative | 77,101 | 6,948 | 36,488 | 77,101 | 6,638 | 83,616 |
| AalbRamini | N | 73,552 | 21 | 37,149 | 73,552 | 0 | 86,916 |

### 5.3 Select SNPs for genotype call with Axiom Suite

We can download the results from the cluster to select SNPs whose
probes have unique mapping in AalbF2 and AalbF3. The parsing script
created several files for each cram file. For example, a list of SNPs
probe sequences with zero mismatches or 1 mismatch with unique
alignments.

The alignments statistics for genomes from NCBI and Vector-Base had
identical results. We use it to create the SNP positions for both
genomes, but we will use only one to select the SNPs for testing.

Files to download: 1) AalbF2

1. Reference allele AalbF2\_ncbi.albopictus\_Ref.txt
   AalbF2\_ncbi.albopictus\_Ref\_F.txt
   AalbF2\_ncbi.albopictus\_Ref\_F\_unique\_no\_mismatches\_ids.txt
2. Alternative allele AalbF2\_ncbi.albopictus\_Alt.txt
   AalbF2\_ncbi.albopictus\_Alt\_F.txt
   AalbF2\_ncbi.albopictus\_Alt\_F\_unique\_mapping\_ids.txt
   AalbF2\_ncbi.albopictus\_Alt\_F\_one\_mismatch\_ids.txt
   AalbF2\_ncbi.albopictus\_Alt\_F\_one\_mismatch\_positions.txt

2. AalbF3

1. Reference allele AalbF3.albopictus\_Ref.txt
   AalbF3.albopictus\_Ref\_F.txt
   AalbF3.albopictus\_Ref\_F\_unique\_no\_mismatches\_ids.txt
2. Alternative allele AalbF3.albopictus\_Alt.txt
   AalbF3.albopictus\_Alt\_F.txt
   AalbF3.albopictus\_Alt\_F\_unique\_mapping\_ids.txt
   AalbF3.albopictus\_Alt\_F\_one\_mismatch\_ids.txt
   AalbF3.albopictus\_Alt\_F\_one\_mismatch\_positions.txt

Create a list of files to download

```
echo 'AalbF2_ncbi.albopictus_Ref.txt
AalbF2_ncbi.albopictus_Ref_F.txt
AalbF2_ncbi.albopictus_Ref_F_unique_no_mismatches_ids.txt
AalbF2_ncbi.albopictus_Alt.txt
AalbF2_ncbi.albopictus_Alt_F.txt
AalbF2_ncbi.albopictus_Alt_F_unique_mapping_ids.txt
AalbF2_ncbi.albopictus_Alt_F_one_mismatch_ids.txt
AalbF2_ncbi.albopictus_Alt_F_one_mismatch_positions.txt
AalbF3.albopictus_Ref.txt
AalbF3.albopictus_Ref_F.txt
AalbF3.albopictus_Ref_F_unique_no_mismatches_ids.txt
AalbF3.albopictus_Alt.txt
AalbF3.albopictus_Alt_F.txt
AalbF3.albopictus_Alt_F_unique_mapping_ids.txt
AalbF3.albopictus_Alt_F_one_mismatch_ids.txt
AalbF3.albopictus_Alt_F_one_mismatch_positions.txt' > output/probes/results_cluster/bwa/list_of_files.txt
```

bwa mem

```
# download the files from the list_of_files.txt
rsync -chavzP --stats --files-from=output/probes/results_cluster/bwa/list_of_files.txt lvc26@mccleary.ycrc.yale.edu:/ycga-gpfs/project/caccone/lvc26/probes/bwa_mem_default_results/ /Users/lucianocosme/Library/CloudStorage/Dropbox/Albopictus/manuscript_chip/data/no_autogenous/albo_chip/output/probes/results_cluster/bwa/bwa_mem_default_results/
```

bwa aln

```
# next we can download the files to our computer, run the command below in your computer, not in the cluster
rsync -chavzP --stats --files-from=output/probes/results_cluster/bwa/list_of_files.txt lvc26@mccleary.ycrc.yale.edu:/ycga-gpfs/project/caccone/lvc26/probes/bwa_aln_default_results /Users/lucianocosme/Library/CloudStorage/Dropbox/Albopictus/manuscript_chip/data/no_autogenous/albo_chip/output/probes/results_cluster/bwa/bwa_aln_default_results
```

Check the files AalbF2

```
# this file list all SNPs sequences with the reference allele
head output/probes/results_cluster/bwa/bwa_aln_default_results/AalbF2_ncbi.albopictus_Ref_F.txt
```

```
## Read_Name Ref_Name Start End SNP Strand Mapping_Quality Mismatches Indels
## AX-582945972_F NW_021837045 11080 11150 11115 - 37 0 0
## AX-582943846_F NW_021837045 22286 22356 22321 + 37 0 0
## AX-582943848_F NW_021837045 22597 22667 22632 + 37 0 0
## AX-582943855_F NW_021837045 78569 78639 78604 + 20 0 0
## AX-582946029_F NW_021837045 133542 133612 133577 - 0 0 0
## AX-582943896_F NW_021837045 133743 133813 133778 + 37 0 0
## AX-582943911_F NW_021837045 153731 153801 153766 + 37 0 0
## AX-582943915_F NW_021837045 161325 161395 161360 + 37 0 0
## AX-582943937_F NW_021837045 197669 197739 197704 + 37 0 0
```

AalbF3

```
# this file list all SNPs sequences with the reference allele
head output/probes/results_cluster/bwa/bwa_aln_default_results/AalbF3.albopictus_Ref_F.txt
```

```
## Read_Name Ref_Name Start End SNP Strand Mapping_Quality Mismatches Indels
## AX-581444870_F 1.1 97821 97891 97856 - 20 0 0
## AX-583033226_F 1.1 161694 161764 161729 + 37 0 0
## AX-583035067_F 1.1 229605 229675 229640 - 0 0 0
## AX-583035083_F 1.1 236521 236591 236556 - 0 0 0
## AX-583035102_F 1.1 308089 308159 308124 - 0 0 0
## AX-583033340_F 1.1 311885 311955 311920 + 37 0 0
## AX-583033342_F 1.1 315024 315094 315059 + 23 0 0
## AX-583033356_F 1.1 315639 315709 315674 + 37 0 0
## AX-584624610_F 1.1 318424 318494 318459 - 0 0 0
```

#### 5.3.1 AalbF2 unique alignments and no mismatches for sequences with reference allele

bwa mem

```
# this file has the list of IDs with zero mismatches and unique alignments with MP>30
head output/probes/results_cluster/bwa/bwa_mem_default_results/AalbF2_ncbi.albopictus_Ref_F_unique_no_mismatches_ids.txt;

echo "
How many sequences with unique alignments and no mismatches:
"
wc -l output/probes/results_cluster/bwa/bwa_mem_default_results/AalbF2_ncbi.albopictus_Ref_F_unique_no_mismatches_ids.txt | awk '{print $1}'
```

```
## AX-579436016_F
## AX-579436089_F
## AX-579436102_F
## AX-579436125_F
## AX-579436149_F
## AX-579436196_F
## AX-579436214_F
## AX-579436243_F
## AX-579436253_F
## AX-579436298_F
## 
## How many sequences with unique alignments and no mismatches:
## 
## 115029
```

bwa aln unique alignments and no mismatches

```
# this file has the list of IDs with zero mismatches and unique alignments with MP>30
head output/probes/results_cluster/bwa/bwa_aln_default_results/AalbF2_ncbi.albopictus_Ref_F_unique_no_mismatches_ids.txt;

echo "
How many sequences with unique alignments and no mismatches:
"
wc -l output/probes/results_cluster/bwa/bwa_aln_default_results/AalbF2_ncbi.albopictus_Ref_F_unique_no_mismatches_ids.txt | awk '{print $1}'
```

```
## AX-579436016_F
## AX-579436089_F
## AX-579436102_F
## AX-579436125_F
## AX-579436149_F
## AX-579436196_F
## AX-579436214_F
## AX-579436243_F
## AX-579436253_F
## AX-579436298_F
## 
## How many sequences with unique alignments and no mismatches:
## 
## 122199
```

#### 5.3.2 AalbF3 unique alignments and no mismatches for sequences with reference allele

bwa mem

```
# this file has the list of IDs with zero mismatches and unique alignments with MP>30
head output/probes/results_cluster/bwa/bwa_mem_default_results/AalbF3.albopictus_Ref_F_unique_no_mismatches_ids.txt;

echo "
How many sequences with unique alignments and no mismatches:
"
wc -l output/probes/results_cluster/bwa/bwa_mem_default_results/AalbF3.albopictus_Ref_F_unique_no_mismatches_ids.txt | awk '{print $1}'
```

```
## AX-579436016_F
## AX-579436089_F
## AX-579436102_F
## AX-579436125_F
## AX-579436149_F
## AX-579436196_F
## AX-579436214_F
## AX-579436243_F
## AX-579436253_F
## AX-579436298_F
## 
## How many sequences with unique alignments and no mismatches:
## 
## 166111
```

bwa aln

```
# this file has the list of IDs with zero mismatches and unique alignments with MP>30
head output/probes/results_cluster/bwa/bwa_aln_default_results/AalbF3.albopictus_Ref_F_unique_no_mismatches_ids.txt;

echo "
How many sequences with unique alignments and no mismatches:
"
wc -l output/probes/results_cluster/bwa/bwa_aln_default_results/AalbF3.albopictus_Ref_F_unique_no_mismatches_ids.txt | awk '{print $1}'
```

```
## AX-579436016_F
## AX-579436089_F
## AX-579436102_F
## AX-579436125_F
## AX-579436149_F
## AX-579436196_F
## AX-579436214_F
## AX-579436243_F
## AX-579436253_F
## AX-579436298_F
## 
## How many sequences with unique alignments and no mismatches:
## 
## 167485
```

#### 5.3.3 Create Venn diagram AalbF3

```
# Function to read and process data
read_and_process_data <- function(subdir, filename) {
  data <- read_delim(
    here('output', 'probes', 'results_cluster', 'bwa', subdir, filename),
    delim = "\t",
    col_names = FALSE,
    show_col_types = FALSE
  ) |>
    pull() |>
    str_replace("_F$", "")
  
  return(data)
}

# Read in the two files as vectors
AalbF3_Ref_F_mem <-
  read_and_process_data(
    'bwa_mem_default_results',
    'AalbF3.albopictus_Ref_F_unique_no_mismatches_ids.txt'
  )
AalbF3_Ref_F_aln <-
  read_and_process_data(
    'bwa_aln_default_results',
    'AalbF3.albopictus_Ref_F_unique_no_mismatches_ids.txt'
  )

# Calculate shared values
AalbF3_Ref_F <- intersect(AalbF3_Ref_F_mem, AalbF3_Ref_F_aln)


# Create Venn diagram
venn_data <- list(
  "bwa mem" = AalbF3_Ref_F_mem,
  "bwa aln" = AalbF3_Ref_F_aln
)
venn_plot <- ggvenn(venn_data, fill_color = c("steelblue", "darkorange"), show_percentage = TRUE)

# Add a title
venn_plot <- venn_plot +
  ggtitle("Comparison of aligners for sequences mapped to AalbF3
          \n Reference allele") +
  theme(plot.title = element_text(hjust = .5))

# Display the Venn diagram
print(venn_plot)
```

```
# Save Venn diagram to PDF
output_path <- here("output", "probes", "results_cluster", "bwa", "AalbF3_Ref_F.pdf")
ggsave(output_path, venn_plot, height = 5, width = 5, dpi = 300)
```

#### 5.3.4 Create Venn diagram AalbF2

```
# Read in the two files as vectors
AalbF2_Ref_F_mem <-
  read_and_process_data(
    'bwa_mem_default_results',
    'AalbF2_ncbi.albopictus_Ref_F_unique_no_mismatches_ids.txt'
  )
AalbF2_Ref_F_aln <-
  read_and_process_data(
    'bwa_aln_default_results',
    'AalbF2_ncbi.albopictus_Ref_F_unique_no_mismatches_ids.txt'
  )

# Calculate shared values
AalbF2_Ref_F <- intersect(AalbF2_Ref_F_mem, AalbF2_Ref_F_aln)


# Create Venn diagram
venn_data2 <- list(
  "bwa mem" = AalbF2_Ref_F_mem,
  "bwa aln" = AalbF2_Ref_F_aln
)
venn_plot2 <- ggvenn(venn_data2, fill_color = c("steelblue", "darkorange"), show_percentage = TRUE)

# Add a title
venn_plot2 <- venn_plot2 +
  ggtitle("Comparison of aligners for sequences mapped to AalbF2
          \n Reference allele") +
  theme(plot.title = element_text(hjust = .5))

# Display the Venn diagram
print(venn_plot2)
```

```
# Save Venn diagram to PDF
output_path <- here("output", "probes", "results_cluster", "bwa", "AalbF2_Ref_F.pdf")
ggsave(output_path, venn_plot2, height = 5, width = 5, dpi = 300)
```

#### 5.3.5 Create Venn diagram AalbF2 and AalbF3

```
# Calculate shared values between AalbF2 and AalbF3
Ref_F <- intersect(AalbF2_Ref_F, AalbF3_Ref_F)

# Create Venn diagram
AalbF23_Ref_F <- list(
  "AalbF2" = AalbF2_Ref_F,
  "AalbF3" = AalbF3_Ref_F
)
venn_plot3 <- ggvenn(AalbF23_Ref_F, fill_color = c("steelblue", "darkorange"), show_percentage = TRUE)

# Add a title
venn_plot3 <- venn_plot3 +
  ggtitle("Comparison sequences mapped to AalbF2 and AalbF3
          \n Reference allele") +
  theme(plot.title = element_text(hjust = .5))

# Display the Venn diagram
print(venn_plot3)
```

```
# Save Venn diagram to PDF
output_path <- here("output", "probes", "results_cluster", "bwa", "AalbF23_Ref_F.pdf")
ggsave(output_path, venn_plot3, height = 5, width = 5, dpi = 300)
```

#### 5.3.6 AalbF2 unique alignments and one mismatches for sequences with reference allele

bwa mem

```
# this file has the list of IDs with 1 mismatches (they have the reference allele)
head output/probes/results_cluster/bwa/bwa_mem_default_results/AalbF2_ncbi.albopictus_Alt_F_one_mismatch_ids.txt;

echo "
How many sequences with alignments with 1 mismatches:
"
wc -l output/probes/results_cluster/bwa/bwa_mem_default_results/AalbF2_ncbi.albopictus_Alt_F_one_mismatch_ids.txt | awk '{print $1}'
```

```
## AX-582945972_F
## AX-582943848_F
## AX-582943896_F
## AX-582943915_F
## AX-582943937_F
## AX-582944020_F
## AX-582946265_F
## AX-582946304_F
## AX-582944169_F
## AX-582944173_F
## 
## How many sequences with alignments with 1 mismatches:
## 
## 96883
```

bwa aln alignments with 1 mismatches (sequences with alternative
allele)

```
# this file has the list of IDs with 1 mismatches
head output/probes/results_cluster/bwa/bwa_aln_default_results/AalbF2_ncbi.albopictus_Alt_F_one_mismatch_ids.txt;

echo "
How many sequences with alignments with 1 mismatches:
"
wc -l output/probes/results_cluster/bwa/bwa_aln_default_results/AalbF2_ncbi.albopictus_Alt_F_one_mismatch_ids.txt | awk '{print $1}'
```

```
## AX-582945972_F
## AX-582943846_F
## AX-582943848_F
## AX-582943896_F
## AX-582943915_F
## AX-582943937_F
## AX-582946090_F
## AX-582944020_F
## AX-582946265_F
## AX-582944133_F
## 
## How many sequences with alignments with 1 mismatches:
## 
## 114333
```

Get the overlapping sequences

```
# Function to read and process data
read_and_process_data <- function(subdir, filename) {
  data <- read_delim(
    here('output', 'probes', 'results_cluster', 'bwa', subdir, filename),
    delim = "\t",
    col_names = FALSE,
    show_col_types = FALSE
  ) |>
    pull() |>
    str_replace("_F$", "")
  
  return(data)
}

# Read in the two files as vectors
AalbF2_1ms_Alt_F_mem <-
  read_and_process_data(
    'bwa_mem_default_results',
    'AalbF2_ncbi.albopictus_Alt_F_one_mismatch_ids.txt'
  )
AalbF2_1ms_Alt_F_aln <-
  read_and_process_data(
    'bwa_aln_default_results',
    'AalbF2_ncbi.albopictus_Alt_F_one_mismatch_ids.txt'
  )

# Calculate shared values
AalbF2_1ms_Alt_F <- intersect(AalbF2_1ms_Alt_F_mem, AalbF2_1ms_Alt_F_aln)

# Create Venn diagram
venn_data4 <- list(
  "bwa mem" = AalbF2_1ms_Alt_F_mem,
  "bwa aln" = AalbF2_1ms_Alt_F_aln
)
venn_plot4 <- ggvenn(venn_data4, fill_color = c("steelblue", "darkorange"), show_percentage = TRUE)

# Add a title
venn_plot4 <- venn_plot4 +
  ggtitle("Comparison of aligners for sequences mapped to AalbF2 \n 1 mismatch - Alternative allele") +
  theme(plot.title = element_text(hjust = .5))

# Display the Venn diagram
print(venn_plot4)
```

```
# Save Venn diagram to PDF
output_path <- here("output", "probes", "results_cluster", "bwa", "AalbF2_alt_1ms_F.pdf")
ggsave(output_path, venn_plot4, height = 5, width = 5, dpi = 300)
```

Check the ids for the sequences with unique alignments bwa aln

```
# this file has the list of IDs with unique alignments
head output/probes/results_cluster/bwa/bwa_aln_default_results/AalbF2_ncbi.albopictus_Alt_F_unique_mapping_ids.txt;

echo "
How many sequences with unique alignments:
"
wc -l output/probes/results_cluster/bwa/bwa_aln_default_results/AalbF2_ncbi.albopictus_Alt_F_unique_mapping_ids.txt | awk '{print $1}'
```

```
## AX-582945972_F
## AX-582943846_F
## AX-582943848_F
## AX-582943896_F
## AX-582943915_F
## AX-582943937_F
## AX-582946090_F
## AX-582944020_F
## AX-582946265_F
## AX-582944133_F
## 
## How many sequences with unique alignments:
## 
## 114335
```

bwa mem

```
# this file has the list of IDs with unique alignments
head output/probes/results_cluster/bwa/bwa_mem_default_results/AalbF2_ncbi.albopictus_Alt_F_unique_mapping_ids.txt;

echo "
How many sequences with unique alignments:
"
wc -l output/probes/results_cluster/bwa/bwa_mem_default_results/AalbF2_ncbi.albopictus_Alt_F_unique_mapping_ids.txt | awk '{print $1}'
```

```
## AX-582945972_F
## AX-582943848_F
## AX-582943896_F
## AX-582943915_F
## AX-582943937_F
## AX-582944020_F
## AX-582946265_F
## AX-582946304_F
## AX-582944169_F
## AX-582944173_F
## 
## How many sequences with unique alignments:
## 
## 96906
```

Get the overlapping sequences

```
# Read in the two files as vectors
AalbF2_unique_Alt_F_mem <-
  read_and_process_data(
    'bwa_mem_default_results',
    'AalbF2_ncbi.albopictus_Alt_F_unique_mapping_ids.txt'
  )
AalbF2_unique_Alt_F_aln <-
  read_and_process_data(
    'bwa_aln_default_results',
    'AalbF2_ncbi.albopictus_Alt_F_unique_mapping_ids.txt'
  )

# Calculate shared values
AalbF2_unique_Alt_F <- intersect(AalbF2_unique_Alt_F_mem, AalbF2_unique_Alt_F_aln)


# Create Venn diagram
venn_data5 <- list(
  "bwa mem" = AalbF2_unique_Alt_F_mem,
  "bwa aln" = AalbF2_unique_Alt_F_aln
)
venn_plot5 <- ggvenn(venn_data5, fill_color = c("steelblue", "darkorange"), show_percentage = TRUE)

# Add a title
venn_plot5 <- venn_plot5 +
  ggtitle("Comparison of unique aligners for sequences mapped to AalbF2 \n Alternative allele") +
  theme(plot.title = element_text(hjust = .5))

# Display the Venn diagram
print(venn_plot5)
```

```
# Save Venn diagram to PDF
output_path <- here("output", "probes", "results_cluster", "bwa", "AalbF2_alt_unique_F.pdf")
ggsave(output_path, venn_plot5, height = 5, width = 5, dpi = 300)
```

Combine the unique and the 1 mismatch objects

```
# Calculate shared values
AalbF2_Alt_F <- intersect(AalbF2_1ms_Alt_F, AalbF2_unique_Alt_F)


# Create Venn diagram
venn_data6 <- list(
  "1 mismatch" = AalbF2_1ms_Alt_F,
  "Unique" = AalbF2_unique_Alt_F
)
venn_plot6 <- ggvenn(venn_data6, fill_color = c("steelblue", "darkorange"), show_percentage = TRUE)

# Add a title
venn_plot6 <- venn_plot6 +
  ggtitle("Unique + 1 mismatch alignments - AalbF2 \n Alternative allele") +
  theme(plot.title = element_text(hjust = .5))

# Display the Venn diagram
print(venn_plot6)
```

```
# Save Venn diagram to PDF
output_path <- here("output", "probes", "results_cluster", "bwa", "AalbF2_alt_unique_1ms_F.pdf")
ggsave(output_path, venn_plot6, height = 5, width = 5, dpi = 300)
```

#### 5.3.7 AalbF3 unique alignments and one mismatches for sequences with reference allele

bwa mem

```
# this file has the list of IDs with 1 mismatches (they have the reference allele)
head output/probes/results_cluster/bwa/bwa_mem_default_results/AalbF3.albopictus_Alt_F_one_mismatch_ids.txt;

echo "
How many sequences with alignments with 1 mismatches:
"
wc -l output/probes/results_cluster/bwa/bwa_mem_default_results/AalbF3.albopictus_Alt_F_one_mismatch_ids.txt | awk '{print $1}'
```

```
## AX-583033226_F
## AX-583033340_F
## AX-583033356_F
## AX-583033397_F
## AX-583035211_F
## AX-583035237_F
## AX-583033434_F
## AX-583035251_F
## AX-583035255_F
## AX-583035268_F
## 
## How many sequences with alignments with 1 mismatches:
## 
## 164094
```

bwa aln alignments with 1 mismatches (sequences with alternative
allele)

```
# this file has the list of IDs with 1 mismatches
head output/probes/results_cluster/bwa/bwa_aln_default_results/AalbF3.albopictus_Alt_F_one_mismatch_ids.txt;

echo "
How many sequences with alignments with 1 mismatches:
"
wc -l output/probes/results_cluster/bwa/bwa_aln_default_results/AalbF3.albopictus_Alt_F_one_mismatch_ids.txt | awk '{print $1}'
```

```
## AX-583033226_F
## AX-583033340_F
## AX-583033356_F
## AX-583033397_F
## AX-583035211_F
## AX-583035237_F
## AX-583033434_F
## AX-583035251_F
## AX-583035255_F
## AX-583035268_F
## 
## How many sequences with alignments with 1 mismatches:
## 
## 167391
```

Get the overlapping sequences

```
# Read in the two files as vectors
AalbF3_1ms_Alt_F_mem <-
  read_and_process_data(
    'bwa_mem_default_results',
    'AalbF3.albopictus_Alt_F_one_mismatch_ids.txt'
  )
AalbF3_1ms_Alt_F_aln <-
  read_and_process_data(
    'bwa_aln_default_results',
    'AalbF3.albopictus_Alt_F_one_mismatch_ids.txt'
  )

# Calculate shared values
AalbF3_1ms_Alt_F <- intersect(AalbF3_1ms_Alt_F_mem, AalbF3_1ms_Alt_F_aln)

# Create Venn diagram
venn_data7 <- list(
  "bwa mem" = AalbF3_1ms_Alt_F_mem,
  "bwa aln" = AalbF3_1ms_Alt_F_aln
)
venn_plot7 <- ggvenn(venn_data7, fill_color = c("steelblue", "darkorange"), show_percentage = TRUE)

# Add a title
venn_plot7 <- venn_plot7 +
  ggtitle("Comparison of aligners for sequences mapped to AalbF3 \n 1 mismatch - Alternative allele") +
  theme(plot.title = element_text(hjust = .5))

# Display the Venn diagram
print(venn_plot7)
```

```
# Save Venn diagram to PDF
output_path <- here("output", "probes", "results_cluster", "bwa", "AalbF3_alt_1ms_F.pdf")
ggsave(output_path, venn_plot7, height = 5, width = 5, dpi = 300)
```

Check the ids for the sequences with unique alignments bwa aln

```
# this file has the list of IDs with unique alignments
head output/probes/results_cluster/bwa/bwa_aln_default_results/AalbF3.albopictus_Alt_F_unique_mapping_ids.txt;

echo "
How many sequences with unique alignments:
"
wc -l output/probes/results_cluster/bwa/bwa_aln_default_results/AalbF3.albopictus_Alt_F_unique_mapping_ids.txt | awk '{print $1}'
```

```
## AX-583033226_F
## AX-583033340_F
## AX-583033356_F
## AX-583033397_F
## AX-583035211_F
## AX-583035237_F
## AX-583033434_F
## AX-583035251_F
## AX-583035255_F
## AX-583035268_F
## 
## How many sequences with unique alignments:
## 
## 167391
```

bwa mem

```
# this file has the list of IDs with unique alignments
head output/probes/results_cluster/bwa/bwa_mem_default_results/AalbF3.albopictus_Alt_F_unique_mapping_ids.txt;

echo "
How many sequences with unique alignments:
"
wc -l output/probes/results_cluster/bwa/bwa_mem_default_results/AalbF3.albopictus_Alt_F_unique_mapping_ids.txt | awk '{print $1}'
```

```
## AX-583033226_F
## AX-583033340_F
## AX-583033356_F
## AX-583033397_F
## AX-583035211_F
## AX-583035237_F
## AX-583033434_F
## AX-583035251_F
## AX-583035255_F
## AX-583035268_F
## 
## How many sequences with unique alignments:
## 
## 164095
```

Get the overlapping sequences

```
# Read in the two files as vectors
AalbF3_unique_Alt_F_mem <-
  read_and_process_data(
    'bwa_mem_default_results',
    'AalbF3.albopictus_Alt_F_unique_mapping_ids.txt'
  )
AalbF3_unique_Alt_F_aln <-
  read_and_process_data(
    'bwa_aln_default_results',
    'AalbF3.albopictus_Alt_F_unique_mapping_ids.txt'
  )

# Calculate shared values
AalbF3_unique_Alt_F <- intersect(AalbF3_unique_Alt_F_mem, AalbF3_unique_Alt_F_aln)


# Create Venn diagram
venn_data8 <- list(
  "bwa mem" = AalbF3_unique_Alt_F_mem,
  "bwa aln" = AalbF3_unique_Alt_F_aln
)
venn_plot8 <- ggvenn(venn_data8, fill_color = c("steelblue", "darkorange"), show_percentage = TRUE)

# Add a title
venn_plot8 <- venn_plot8 +
  ggtitle("Comparison of unique aligners for sequences mapped to AalbF3 \n Alternative allele") +
  theme(plot.title = element_text(hjust = .5))

# Display the Venn diagram
print(venn_plot8)
```

```
# Save Venn diagram to PDF
output_path <- here("output", "probes", "results_cluster", "bwa", "AalbF8_alt_unique_F.pdf")
ggsave(output_path, venn_plot8, height = 5, width = 5, dpi = 300)
```

Combine the unique and the 1 mismatch objects

```
# Calculate shared values
AalbF3_Alt_F <- intersect(AalbF3_1ms_Alt_F, AalbF3_unique_Alt_F)


# Create Venn diagram
venn_data9 <- list(
  "1 mismatch" = AalbF3_1ms_Alt_F,
  "Unique" = AalbF3_unique_Alt_F
)
venn_plot9 <- ggvenn(venn_data9, fill_color = c("steelblue", "darkorange"), show_percentage = TRUE)

# Add a title
venn_plot9 <- venn_plot9 +
  ggtitle("Unique + 1 mismatch alignments - AalbF3 \n Alternative allele") +
  theme(plot.title = element_text(hjust = .5))

# Display the Venn diagram
print(venn_plot9)
```

```
# Save Venn diagram to PDF
output_path <- here("output", "probes", "results_cluster", "bwa", "AalbF3_alt_unique_1ms_F.pdf")
ggsave(output_path, venn_plot9, height = 5, width = 5, dpi = 300)
```

Combine AalbF2 and AalbF3

```
# Calculate shared values
Alt_F <- intersect(AalbF3_Alt_F, AalbF2_Alt_F)

# Create Venn diagram
venn_data10 <- list(
  "AalbF2" = AalbF2_Alt_F,
  "AalbF3" = AalbF3_Alt_F
)
venn_plot10 <- ggvenn(venn_data10, fill_color = c("steelblue", "darkorange"), show_percentage = TRUE)

# Add a title
venn_plot10 <- venn_plot10 +
  ggtitle("AalbF2 vs. AalbF3 (Unique + 1 mismatch alignments) \n Alternative allele") +
  theme(plot.title = element_text(hjust = .5))

# Display the Venn diagram
print(venn_plot10)
```

```
# Save Venn diagram to PDF
output_path <- here("output", "probes", "results_cluster", "bwa", "AalbF23_alt_unique_1ms_F.pdf")
ggsave(output_path, venn_plot10, height = 5, width = 5, dpi = 300)
```

Now get the intersect of reference and alternative sets Combine
AalbF2 and AalbF3

```
# Calculate shared values
both_alleles <- intersect(Ref_F, Alt_F)

# save the vector to the output file
write_lines(
  both_alleles,
  file = here(
    "output",
    "probes",
    "results_cluster",
    "bwa",
    "SNPs_for_genotyping_bwa_default.txt"
  )
)


# Create Venn diagram
venn_data11 <- list(
  "Reference" = Ref_F,
  "Alternative" = Alt_F
)
venn_plot11 <- ggvenn(venn_data11, fill_color = c("steelblue", "darkorange"), show_percentage = TRUE)

# Add a title
venn_plot11 <- venn_plot11 +
  ggtitle("SNPs for genotyping - albopictus") +
  theme(plot.title = element_text(hjust = .5))

# Display the Venn diagram
print(venn_plot11)
```

```
# Save Venn diagram to PDF
output_path <- here("output", "probes", "results_cluster", "bwa", "SNPs_with_for_genotyping.pdf")
ggsave(output_path, venn_plot11, height = 5, width = 5, dpi = 300)
```

Next, we need to compare the coordinates of the SNPs and location of
the alignments for both sequences with reference and alternative allele.
We will keep only those that match. Most of them should have the same
coordinates.

AalbF3 Reference allele

```
# this file has the list alignments
head output/probes/results_cluster/bwa/bwa_mem_default_results/AalbF3.albopictus_Ref_F.txt;

echo "
How many alignments:
"
wc -l output/probes/results_cluster/bwa/bwa_mem_default_results/AalbF3.albopictus_Ref_F.txt | awk '{print $1}'
```

```
## Read_Name Ref_Name Start End SNP Strand Mapping_Quality Mismatches Indels
## AX-583656114_F 1.1 92786 92786 92821 - 0 2 0
## AX-582033422_F 1.1 97460 97460 97495 + 0 2 0
## AX-581444870_F 1.1 97821 97891 97856 - 10 0 0
## AX-583250556_F 1.1 103230 103230 103265 - 0 3 0
## AX-583033226_F 1.1 161694 161764 161729 + 60 0 0
## AX-580321895_F 1.1 221297 221297 221332 + 0 1 0
## AX-583035067_F 1.1 229605 229675 229640 - 0 0 0
## AX-583035102_F 1.1 234402 234402 234437 - 0 0 0
## AX-583035083_F 1.1 236521 236521 236556 - 0 0 0
## 
## How many alignments:
## 
## 219168
```

Alternative allele

```
# this file has the list alignments
head output/probes/results_cluster/bwa/bwa_mem_default_results/AalbF3.albopictus_Alt_F.txt;

echo "
How many alignments:
"
wc -l output/probes/results_cluster/bwa/bwa_mem_default_results/AalbF3.albopictus_Alt_F.txt | awk '{print $1}'
```

```
## Read_Name Ref_Name Start End SNP Strand Mapping_Quality Mismatches Indels
## AX-582395497_F 1.1 12020 12020 12055 - 0 0 0
## AX-584128854_F 1.1 14915 14915 14950 - 0 8 1
## AX-583691589_F 1.1 34280 34280 34315 - 0 0 0
## AX-583818014_F 1.1 46152 46152 46187 - 0 0 0
## AX-581137336_F 1.1 67672 67672 67707 - 0 3 0
## AX-583656114_F 1.1 92786 92786 92821 - 0 3 0
## AX-582317674_F 1.1 94572 94572 94607 + 0 3 0
## AX-582033422_F 1.1 97460 97460 97495 + 0 3 0
## AX-581444870_F 1.1 97821 97891 97856 - 9 1 0
## 
## How many alignments:
## 
## 253116
```

```
# this file has the list alignments
head output/probes/results_cluster/bwa/bwa_mem_default_results/AalbF3.albopictus_Alt_F_one_mismatch_positions.txt;

echo "
How many alignments:
"
wc -l output/probes/results_cluster/bwa/bwa_mem_default_results/AalbF3.albopictus_Alt_F_one_mismatch_positions.txt | awk '{print $1}'
```

```
## AX-583033226_F 36 161729
## AX-583033340_F 36 311920
## AX-583033356_F 36 315674
## AX-583033397_F 36 343391
## AX-583035211_F 36 345197
## AX-583035237_F 36 45785
## AX-583033434_F 36 49852
## AX-583035251_F 36 81816
## AX-583035255_F 36 90665
## AX-583035268_F 36 111746
## 
## How many alignments:
## 
## 164094
```

Now we can compare it with the original data.

Import probe information

```
# import the data
probes_albopictus <-
  read_delim(
    here(
      "data", "probes", "tilled.csv"
    ),
    delim = ",",
    col_names = TRUE,
    show_col_types = FALSE
  ) |>
  mutate(
    Scaffold = str_replace(cust_chr, "chr", "")
  ) |>
  select(
    Name, Scaffold, cust_pos
  ) |>
  arrange(
    Scaffold, cust_pos
  )

# check it out
head(probes_albopictus)
```

```
## # A tibble: 6 × 3
##   Name         Scaffold cust_pos
##   <chr>        <chr>       <dbl>
## 1 AX-581444870 1.1         97856
## 2 AX-583033226 1.1        161729
## 3 AX-583035067 1.1        229640
## 4 AX-583035083 1.1        305518
## 5 AX-583035102 1.1        308124
## 6 AX-583033340 1.1        311920
```

Import probe information

```
# import the data
alt_pos_map <-
  read_delim(
    here("output", "probes", "results_cluster", "bwa", "bwa_mem_default_results", "AalbF3.albopictus_Alt_F_one_mismatch_positions.txt"),
    delim = " ",
    col_names = FALSE,
    show_col_types = FALSE
  ) |>
  mutate_at(1, ~ str_remove(., "_F$"))

# rename columns
alt_pos_map <- alt_pos_map |>
  rename_at(
    vars(1:3), 
    ~ c("SNPs", "pos_seq", "pos_scaffold")
  )
# check it out
head(alt_pos_map)
```

```
## # A tibble: 6 × 3
##   SNPs         pos_seq pos_scaffold
##   <chr>          <dbl>        <dbl>
## 1 AX-583033226      36       161729
## 2 AX-583033340      36       311920
## 3 AX-583033356      36       315674
## 4 AX-583033397      36       343391
## 5 AX-583035211      36       345197
## 6 AX-583035237      36        45785
```

Join the objects

```
map_x_original <-
  left_join(
    probes_albopictus, alt_pos_map, by = c("Name" = "SNPs")
  ) |>
  drop_na()

# check if original and mapped data match
map_x_original |>
  mutate(match = (cust_pos == pos_scaffold)) |>
  group_by(match) |>
  summarise(count = n())
```

```
## # A tibble: 1 × 2
##   match  count
##   <lgl>  <int>
## 1 TRUE  164094
```

```
# all match, so the code to find the SNP position worked
```
