# Supplementary material for: A genotyping array for the globally invasive vector mosquito, Aedes albopictus
Source: Parasit Vectors. 2024 Mar 4;17:106. doi: 10.1186/s13071-024-06158-z (PMC10910840; doi:10.1186/s13071-024-06158-z)
Supplement: Supplementary file 3 — Additional file 3. Segregation analysis from laboratory crosses. [file 13071_2024_6158_MOESM3_ESM.html]

Aedes albopictus SNP chip - Analyse of allele segregation from laboratory crosses.


# Aedes albopictus SNP chip - Analyse of allele segregation from laboratory crosses.

#### Luciano V Cosme

#### 2023-08-28

- Validation
  of the *Aedes albopictus* chip using laboratory crosses
- Analytic
  approach
  - 1. Load
    libraries
  - 2. Import the
    data
    - 2.1 Use Plink2 to convert
      to bed format
    - 2.1.1 Use R to update the .fam
      file
  - 2. Check Mendel test with
    Plink
  - 3.
    Find segregating SNPs
    - 3.1
      Create bed files for each set of parents and offspring
      - 3.1.1 Parents
      - 3.1.1 Offspring
    - 3.2 Find the segregating
      SNPs using python
    - 3.3 Find the segregating SNPs
      using R
    - 3.4
      Sanity check: count segregating SNPs for parents in each family
    - 3.5 Create Venn diagram for
      one family
    - 3.6 Create Venn diagram
      for all families
    - 3.7
      Create six way Venn diagram for all families with the segregating
      SNPs
  - 4.
    Calculate the expected and observed allele frequencies or counts
    - 4.1 Estimate frequencies of
      parents
    - 4.2 Check the allele count
      of one family
    - 4.3 Check the minor
      allele count of one family
    - 4.4 Check the counts of each
      allele
    - 4.5 Estimate frequencies for
      offspring
    - 4.6 Clean the
      Plink output files to import into R
    - 4.7 Import frequency and
      counts
  - 5. Data
    Visualization
    - 5.1
      Get the unique total SNP count across all families that we can
      test
    - 5.2 Get the
      “shared” SNPs (genotyped in all 6 families)
    - 5.3 SNPs “Shared” among
      at least two families
    - 5.4 Plot
      allele counts
    - 5.5 Pie plot of the counts
    - 5.6 Check for
      NAs
  - 6. Tidying the
    data for statistical comparisons
    - 6.1 Remove NAs
    - 6.2 Sort by SNP
      id
  - 7. Power
    simulation
  - 8. Estimate power for our
    data
  - 9.
    Number of SNPs genotyped in the parents that could be tested
    - 9.1 Venn
      diagram
    - 9.2
      Parental loci with at least one parental genotype heterozygous
  - 10. Chi square test
    with Aedes albopictus data
    - 10.1 Remove SNPs with zeros
      or counts < 5
    - 10.2 Sanity checks after data
      tyding
    - 10.3
      Segregation test
  - 11. Sanity
    checks
  - 12. Segregation test with all
    SNPs
    - 12.1
      Estimate p-values using chi-square test (count > 5) or Fisher test
      (counts < 5)
    - 12.2 Save the data and load it
      back
    - 12.3 Combine and adjust
      p-values
    - 12.4 Select p-values columns
    - 12.5 Save the
      list of SNPs for futher genotype calls.

# Validation of the *Aedes albopictus* chip using laboratory crosses

To validate the *Aedes albopictus* chip we conducted six
crosses between native and invasive laboratory colonies.

# Analytic approach

1. Exported the recommended SNPs from the Axiom suite.
2. Utilized R and Python to identify the segregating SNPs, i.e., those
   that are heterozygous in one parent.
3. Performed comparison of only those SNPs where one parent was
   heterozygous. Besides, I estimated the power of a chi square test for
   each SNP.
4. Identified the SNPs shared or unique across the three families.
5. Estimated the frequency of the segregating SNPs in the
   offspring.
6. Based on the missingness in the offspring, I estimated the expected
   allele counts considering the genotypes of the parents.
7. Compared the expected and observed count of each of the two
   alleles.
8. Performed a chi-square test to examine the relationship between the
   observed and expected allele counts.
9. Excluded SNPs that had observed counts less than 5 to ensure
   statistical validity of the chi-square test.
10. If the SNP was present in more than one family, applied Fisher’s
    test to combine the p-values to gain an overall significance level.
11. If the SNP was present only in one family, utilized the single
    available p-value for statistical inference.
12. Performed Bonferroni correction to adjust the significance levels
    for multiple testing to control the family-wise error rate.

## 1. Load libraries

```
library(tidyverse)
library(here)
library(colorout)
library(flextable)
library(ggplot2)
library(scales)
library(reticulate)
library(extrafont)
library(stringr)
library(officer)
library(ggrepel)
library(readr)
library(pwr)
library(stats)
library(vcfR)
library(ggvenn)
library(RColorBrewer)
library(VariantAnnotation)
library(data.table)
library(doParallel)
library(dplyr)
library(gridExtra)
library(Cairo)
library(venn)
library(writexl)
```

## 2. Import the data

Check how many samples

```
# make sure you have all the .CEL samples in your family file - 152
bcftools query -l output/segregation/crosses_new_priors.vcf | wc -l
```

```
##      152
```

Check sample names

```
# make sure you have all the .CEL samples in your family file - 152
bcftools query -l output/segregation/crosses_new_priors.vcf | head
```

```
## 100_LabCross_MF71xMM15_M.CEL
## 101_LabCross_MF71xMM15_M.CEL
## 102_LabCross_MF71xMM15_M.CEL
## 103_LabCross_MF71xMM15_M.CEL
## 104_LabCross_MF71xMM15_M.CEL
## 105_LabCross_MF71xMM15_M.CEL
## 106_LabCross_MF71xMM15_M.CEL
## 107_LabCross_MF71xMM15_M.CEL
## 108_LabCross_MF71xMM15_M.CEL
## 109_LabCross_KF42xKM9_F.CEL
```

### 2.1 Use Plink2 to convert to bed format

```
# If you run this chunk you will have to open the file.fam in a text editor and set parents id and sex of each individual. I fix it using bash tools. You can start on the next chunk if you do not want to have to repeat what I did.
# we also check if the reference genome and the reference alleles match.
plink2 \
--allow-extra-chr \
--vcf output/segregation/crosses_new_priors.vcf \
--const-fid \
--make-bed \
--fa data/genome/albo.fasta.gz \
--ref-from-fa 'force' `# sets REF alleles when it can be done unambiguously, we use force to change the alleles` \
--out output/segregation/albopictus/file1 \
--silent;
# --keep-allele-order \ if you use Plink 1.9
grep "variants" output/segregation/albopictus/file1.log # to get the number of variants from the log file.
```

```
## --vcf: 123964 variants scanned.
## 123964 variants loaded from
## --ref-from-fa force: 0 variants changed, 123964 validated.
```

Check the headings of the the files we will work on.

```
head -n 5 output/segregation/albopictus/file1.fam
```

```
## 0    100_LabCross_MF71xMM15_M.CEL    0   0   0   -9
## 0    101_LabCross_MF71xMM15_M.CEL    0   0   0   -9
## 0    102_LabCross_MF71xMM15_M.CEL    0   0   0   -9
## 0    103_LabCross_MF71xMM15_M.CEL    0   0   0   -9
## 0    104_LabCross_MF71xMM15_M.CEL    0   0   0   -9
```

We need to update the family information, individual id, and sex of
each individual. We can use the same file we use with the Axiom Suite to
update our .fam file.

```
head -n 5 data/crosses/crosses_meta_data.txt
```

```
## Sample Filename  Family_ID   Individual_ID   Father_ID   Mother_ID   Sex Affection Status
## 1_LabCross_KF2.CEL   fam1    1   0   0   2   -9
## 2_LabCross_MF81.CEL  fam2    2   0   0   2   -9
## 3_LabCross_MF71.CEL  fam3    3   0   0   2   -9
## 4_LabCross_KF42.CEL  fam4    4   0   0   2   -9
```

### 2.1.1 Use R to update the .fam file

Import the fam file we use with Axiom Suite

```
# the order of the rows in this file does not matter
samples <-
  read.delim(
    file   = here(
      "data", "crosses", "crosses_meta_data.txt"
    ),
    header = TRUE
  )
head(samples)
```

```
##       Sample.Filename Family_ID Individual_ID Father_ID Mother_ID Sex
## 1  1_LabCross_KF2.CEL      fam1             1         0         0   2
## 2 2_LabCross_MF81.CEL      fam2             2         0         0   2
## 3 3_LabCross_MF71.CEL      fam3             3         0         0   2
## 4 4_LabCross_KF42.CEL      fam4             4         0         0   2
## 5 5_LabCross_KF57.CEL      fam5             5         0         0   2
## 6 6_LabCross_MF32.CEL      fam6             6         0         0   2
##   Affection.Status
## 1               -9
## 2               -9
## 3               -9
## 4               -9
## 5               -9
## 6               -9
```

Import .fam file we created once we created the bed file using
Plink2

```
# we will keep the order of the rows in this file
fam1 <-
  read.delim(
    file   = here(
      "output", "segregation", "albopictus", "file1.fam"
    ),
    header = FALSE,
    
  )
head(fam1)
```

```
##   V1                           V2 V3 V4 V5 V6
## 1  0 100_LabCross_MF71xMM15_M.CEL  0  0  0 -9
## 2  0 101_LabCross_MF71xMM15_M.CEL  0  0  0 -9
## 3  0 102_LabCross_MF71xMM15_M.CEL  0  0  0 -9
## 4  0 103_LabCross_MF71xMM15_M.CEL  0  0  0 -9
## 5  0 104_LabCross_MF71xMM15_M.CEL  0  0  0 -9
## 6  0 105_LabCross_MF71xMM15_M.CEL  0  0  0 -9
```

We can merge the tibbles.

```
# to keep the same order of the .fam file, we will first create an index based on the numbers of the samples, then use it too keep the order

# Extract the number part from the columns
fam1_temp <- fam1 |>
  mutate(num_id = as.numeric(str_extract(V2, "^\\d+")))

samples_temp <- samples |>
  mutate(num_id = as.numeric(str_extract(Sample.Filename, "^\\d+")))

# Perform the left join using the num_id columns and keep the order of fam1
df <- fam1_temp |>
  dplyr::left_join(samples_temp, by = "num_id") |>
  dplyr::select(-num_id) |>
  dplyr::select(8:13)

# check the data frame
head(df)
```

```
##   Family_ID Individual_ID Father_ID Mother_ID Sex Affection.Status
## 1      fam3           100        12         3   1               -9
## 2      fam3           101        12         3   1               -9
## 3      fam3           102        12         3   1               -9
## 4      fam3           103        12         3   1               -9
## 5      fam3           104        12         3   1               -9
## 6      fam3           105        12         3   1               -9
```

We can check how many samples we have in our file

```
nrow(df)
```

```
## [1] 152
```

Before you save the new fam file, you can change the original file to
a different name, to compare the order later. If you want to repeat the
steps above after you saving the new file1.fam, you will need to import
the vcf again.

```
#   ____________________________________________________________________________
#   save calculation to load later                                          ####
write.table(
  df,
  file      = here(
    "output", "segregation", "albopictus", "file1.fam"
  ),
  sep       = "\t",
  row.names = FALSE,
  col.names = FALSE,
  quote     = FALSE
)
```

Check the new .fam file to see if has the order and the sample
attributes we want.

```
# you can open the file on a text editor and double check the sample order and information.
head -n 5 output/segregation/albopictus/file1.fam
```

```
## fam3 100 12  3   1   -9
## fam3 101 12  3   1   -9
## fam3 102 12  3   1   -9
## fam3 103 12  3   1   -9
## fam3 104 12  3   1   -9
```

## 2. Check Mendel test with Plink

The command below set genotype missingness threshold to 20%, and
removed variants with any error in any trio (–me 0 0 var-first) or
arbitrary set allowed error rate (–me 0.1 0.02 var-first or –me 0.1 0.05
var-first)

```
plink \
--allow-extra-chr \
--bfile output/segregation/albopictus/file1 \
--mendel \
--me 0.1 0.02 var-first \
--me-exclude-one \
--out output/segregation/albopictus/file2 \
--make-bed \
--geno 0.2 \
--keep-allele-order \
--write-snplist \
--silent;
head -n 100 output/segregation/albopictus/file2.log
```

```
## PLINK v1.90b6.26 64-bit (2 Apr 2022)
## Options in effect:
##   --allow-extra-chr
##   --bfile output/segregation/albopictus/file1
##   --geno 0.2
##   --keep-allele-order
##   --make-bed
##   --me 0.1 0.02 var-first
##   --me-exclude-one
##   --mendel
##   --out output/segregation/albopictus/file2
##   --silent
##   --write-snplist
## 
## Hostname: LucianoCosme.wireless.yale.internal
## Working directory: /Users/lucianocosme/Library/CloudStorage/Dropbox/Albopictus/manuscript_chip/data/no_autogenous/albo_chip
## Start time: Wed Aug 23 11:08:18 2023
## 
## Random number seed: 1692803298
## 32768 MB RAM detected; reserving 16384 MB for main workspace.
## 123964 variants loaded from .bim file.
## 152 people (82 males, 70 females) loaded from .fam.
## Using 1 thread (no multithreaded calculations invoked).
## Before main variant filters, 12 founders and 140 nonfounders present.
## Calculating allele frequencies... done.
## Total genotyping rate is 0.974801.
## 2225 variants removed due to missing genotype data (--geno).
## --me/--mendel: 986705 Mendel errors detected.
## Reports written to output/segregation/albopictus/file2.mendel +
## output/segregation/albopictus/file2.imendel +
## output/segregation/albopictus/file2.fmendel +
## output/segregation/albopictus/file2.lmendel .
## 44101 variants and 0 people excluded.
## 77638 variants and 152 people pass filters and QC.
## Note: No phenotypes present.
## List of variant IDs written to output/segregation/albopictus/file2.snplist .
## --make-bed to output/segregation/albopictus/file2.bed +
## output/segregation/albopictus/file2.bim +
## output/segregation/albopictus/file2.fam ... done.
## 
## End time: Wed Aug 23 11:08:20 2023
```

## 3. Find segregating SNPs

The PLINK –freqx command generates a .frqx file which contains allele
frequencies for each SNP for each specific family.

### 3.1 Create bed files for each set of parents and offspring

We can subset our bed file to keep founders (parents) or non-founders
(offspring). We will create vcf files for each data set.

#### 3.1.1 Parents

```
# Define the list of family names
families=("fam1" "fam2" "fam3" "fam4" "fam5" "fam6")

# Iterate over each family
for fam_name in "${families[@]}"
do
    # Run Plink command for each family
    plink \
    --allow-extra-chr \
    --keep-fam <(echo "$fam_name") \
    --bfile output/segregation/albopictus/file1 \
    --out "output/segregation/albopictus/parents_family_$fam_name" \
    --recode vcf \
    --geno 0 \
    --keep-allele-order \
    --silent \
    --make-bed \
    --filter-founders
done
```

#### 3.1.1 Offspring

```
# Define the list of family IDs
families=("fam1" "fam2" "fam3" "fam4" "fam5" "fam6")

# Iterate over each family
for fam_id in "${families[@]}"
do
    # Run Plink command for each family
    plink \
    --allow-extra-chr \
    --keep-fam <(echo -e "$fam_id") \
    --bfile output/segregation/albopictus/file1 \
    --out output/segregation/albopictus/offspring_family_${fam_id} \
    --recode vcf \
    --keep-allele-order \
    --silent \
    --make-bed \
    --filter-nonfounders
done
```

### 3.2 Find the segregating SNPs using python

We can use vcf files that we created to find the SNPs that are
heterozygous in one parent.

```
import os
import pysam
import sys
import contextlib

# Define a context manager to suppress stdout and stderr
@contextlib.contextmanager
def suppress_stdout_stderr():
    with open(os.devnull, 'w') as null_file:
        with contextlib.redirect_stdout(null_file), contextlib.redirect_stderr(null_file):
            yield

# Redirect standard output to the null device
sys.stdout = open(os.devnull, 'w')

vcf_files = [
    "output/segregation/albopictus/parents_family_fam1.vcf",
    "output/segregation/albopictus/parents_family_fam2.vcf",
    "output/segregation/albopictus/parents_family_fam3.vcf",
    "output/segregation/albopictus/parents_family_fam4.vcf",
    "output/segregation/albopictus/parents_family_fam5.vcf",
    "output/segregation/albopictus/parents_family_fam6.vcf"
]

for vcf_file in vcf_files:
    # Open the VCF file
    vcf = pysam.VariantFile(vcf_file, "r")

    # Set output file name
    output_file_name = vcf_file.replace(".vcf", "_segregating_SNPs_python.txt")

    # Open output file
    with open(output_file_name, "w") as output_file:
        # Iterate over each record
        for rec in vcf.fetch():
            # For each SNP
            if len(rec.alleles) == 2:  # Check if it's biallelic
                sample_1_gt = rec.samples.values()[0]['GT']  # Individual 1 genotype
                sample_2_gt = rec.samples.values()[1]['GT']  # Individual 2 genotype
                
                # Check that they are not homozygous for the same allele
                if not ((sample_1_gt == (0, 0) and sample_2_gt == (0, 0)) or (sample_1_gt == (1, 1) and sample_2_gt == (1, 1))):
                    output_file.write(f"{rec.id}\n")

# Restore standard output
sys.stdout = sys.__stdout__
```

### 3.3 Find the segregating SNPs using R

```
# it is slower
vcf_files <- c(
    "output/segregation/albopictus/parents_family_fam1.vcf",
    "output/segregation/albopictus/parents_family_fam2.vcf",
    "output/segregation/albopictus/parents_family_fam3.vcf",
    "output/segregation/albopictus/parents_family_fam4.vcf",
    "output/segregation/albopictus/parents_family_fam5.vcf",
    "output/segregation/albopictus/parents_family_fam6.vcf"
)

for (vcf_file in vcf_files) {
  # Read the VCF file
  vcf <- readVcf(vcf_file)
  
  # Extract genotypes
  genotypes <- geno(vcf)$GT
  
  # Initialize the result vector
  result <- c()
  
  # Iterate over each record
  for (i in seq_len(nrow(genotypes))) {
    sample_1_gt <- genotypes[i, 1]
    sample_2_gt <- genotypes[i, 2]
    
    # Check that they are not homozygous for the same allele
    if (!(sample_1_gt == "0/0" &&
          sample_2_gt == "0/0") &&
        !(sample_1_gt == "1/1" && sample_2_gt == "1/1")) {
      result <- c(result, row.names(genotypes)[i])
    }
  }
  
  # Set output file name
  output_file_name <-
    gsub(".vcf", "_segregating_SNPs_R.txt", vcf_file)
  
  # Write the result to a file
  write.table(
    result,
    output_file_name,
    row.names = FALSE,
    col.names = FALSE,
    quote = FALSE
  )
}
```

Our goal: SNP can to be heterozygous in one sample if it is
homozygous in the other sample SNP cannot be homozygous for the same
allele in both samples (sample 1 = 0/0 and sample 2 = 0/0 or sample 1 =
1/1 and sample 2 = 2/2) SNP can be homozygous in both samples but for
different allele (sample 1 = 1/1 and sample 2 = 0/0 or vice versa)

Clean memory and environment

```
# python
py_run_string("import gc; gc.collect()")

# R
rm(list = ls())

# Clean up memory
gc()
```

```
##            used  (Mb) gc trigger  (Mb) limit (Mb) max used  (Mb)
## Ncells 10515291 561.6   18072101 965.2         NA 13852664 739.9
## Vcells 18192638 138.8   32644266 249.1      32768 32644266 249.1
```

### 3.4 Sanity check: count segregating SNPs for parents in each family

```
#ls output/segregation/albopictus/parents_family_*_segregating_SNPs_*.txt

files=(
  output/segregation/albopictus/parents_family_*_segregating_SNPs_R.txt
  output/segregation/albopictus/parents_family_*_segregating_SNPs_python.txt
)

echo "family code SNPs"
for file in "${files[@]}"; do
  line=$(wc -l "$file")
  snps=$(echo "$line" | awk '{print $1}')
  family=$(basename "$file" | cut -d "_" -f 3)
  code=$(basename "$file" | cut -d "_" -f 6 | cut -d "." -f 1)
  echo "$family $code $snps"
done
```

```
## family code SNPs
## fam1 R 42116
## fam2 R 54871
## fam3 R 55484
## fam4 R 42684
## fam5 R 42720
## fam6 R 55205
## fam1 python 42116
## fam2 python 54871
## fam3 python 55484
## fam4 python 42684
## fam5 python 42720
## fam6 python 55205
```

We can write the data to file and use flextable to make a table

```
files=(
  output/segregation/albopictus/parents_family_*_segregating_SNPs_R.txt
  output/segregation/albopictus/parents_family_*_segregating_SNPs_python.txt
)

echo "family,code,SNPs" > output/segregation/albopictus/per_family_segregating_SNPS.csv
for file in "${files[@]}"; do
  line=$(wc -l "$file")
  snps=$(echo "$line" | awk '{print $1}')
  family=$(basename "$file" | cut -d "_" -f 3)
  code=$(basename "$file" | cut -d "_" -f 6 | cut -d "." -f 1)
  echo "$family,$code,$snps" >> output/segregation/albopictus/per_family_segregating_SNPS.csv
done
```

Import the data

```
# Read the data
fam_SNPs_segragating <-
  read_csv(
  here(
  "output", "segregation", "albopictus", "per_family_segregating_SNPS.csv"
  ), 
  show_col_types = FALSE
)

# Create the flextable
ft <- 
  flextable(fam_SNPs_segragating)

# Print the table
ft
```

| family | code | SNPs |
| --- | --- | --- |
| fam1 | R | 42,116 |
| fam2 | R | 54,871 |
| fam3 | R | 55,484 |
| fam4 | R | 42,684 |
| fam5 | R | 42,720 |
| fam6 | R | 55,205 |
| fam1 | python | 42,116 |
| fam2 | python | 54,871 |
| fam3 | python | 55,484 |
| fam4 | python | 42,684 |
| fam5 | python | 42,720 |
| fam6 | python | 55,205 |

```
# save the table
# Initialize Word document
doc <- 
  read_docx() |>
  body_add_flextable(value = ft)

# Define the output path with 'here' library
output_path <- here(
  "output",
  "segregation", 
  "albopictus", 
  "per_family_segregating_SNPS.docx"
  )

# Save the Word document
print(doc, target = output_path)
```

The code worked as we expected.

### 3.5 Create Venn diagram for one family

Now compare the files for one family and see if they match

```
# Read in the two files as vectors
family_fam1_segregating_SNPs_python <-
  read_table(
    "output/segregation/albopictus/parents_family_fam1_segregating_SNPs_python.txt",
    col_names = FALSE
  )[[1]]

family_fam1_segregating_SNPs_R <-
  read_table(
    "output/segregation/albopictus/parents_family_fam1_segregating_SNPs_R.txt",
    col_names = FALSE
  )[[1]]

# Calculate shared values
family_fam1_segregating_SNPs <-
  intersect(
    family_fam1_segregating_SNPs_python,
    family_fam1_segregating_SNPs_R
  )


# Create Venn diagram
venn_data <-
  list(
    "python" = family_fam1_segregating_SNPs_python,
    "R" = family_fam1_segregating_SNPs_R
  )
venn_plot <-
  ggvenn(
    venn_data,
    fill_color = c("steelblue", "darkorange"),
    show_percentage = TRUE
  )

# Add a title
venn_plot <-
  venn_plot +
  ggtitle("SNPs fam1") +
  theme(plot.title = element_text(hjust = .5))

# Display the Venn diagram
print(venn_plot)
```

```
# Save Venn diagram to PDF
output_path <-
  here(
    "output",
    "segregation",
    "albopictus",
    "segregating_SNPs_parents_family_fam1.pdf"
  )
ggsave(
  output_path,
  venn_plot,
  height = 5,
  width = 5,
  dpi = 300
)
```

### 3.6 Create Venn diagram for all families

We can create a plot for all families

```
# Initialize a list to store the plots
plots <- list()

# List all families
families <- c("fam1", "fam2", "fam3", "fam4", "fam5", "fam6")

# Loop through each family
for (fam in families) {
  # Read in the two files as vectors
  family_segregating_SNPs_python <-
    read_table(
      paste0(
        "output/segregation/albopictus/parents_family_",
        fam,
        "_segregating_SNPs_python.txt"
      ),
      col_names = FALSE
    )[[1]]
  family_segregating_SNPs_R <-
    read_table(
      paste0(
        "output/segregation/albopictus/parents_family_",
        fam,
        "_segregating_SNPs_R.txt"
      ),
      col_names = FALSE
    )[[1]]
  
  # Calculate shared values
  family_segregating_SNPs <-
    intersect(family_segregating_SNPs_python, family_segregating_SNPs_R)
  
  # Create Venn diagram
  venn_data <-
    list("python" = family_segregating_SNPs_python, "R" = family_segregating_SNPs_R)
  venn_plot <-
    ggvenn(
      venn_data,
      fill_color = c("steelblue", "pink"),
      show_percentage = TRUE
    )
  
  # Add a title
  venn_plot <-
    venn_plot + ggtitle(paste0("SNPs ", fam)) + theme(plot.title = element_text(hjust = .5, vjust = 1.5, margin = margin(b = 5)))
  
  # Add the plot to the list
  plots[[fam]] <- venn_plot
}

# Combine the plots
combined_plot <- grid.arrange(grobs = plots,
                              ncol = 3,
                              layout_matrix = rbind(c(1, 2, 3),
                                                    c(4, 5, 6)))
```

```
# Save combined Venn diagram to PDF
output_path <-
  here(
    "output",
    "segregation",
    "albopictus",
    "segregating_SNPs_parents_all_families.pdf"
  )
ggsave(
  output_path,
  combined_plot,
  height = 15,
  width = 15,
  dpi = 300
)
```

### 3.7 Create six way Venn diagram for all families with the segregating SNPs

```
# List all families
families <- c("fam1", "fam2", "fam3", "fam4", "fam5", "fam6")

# Initialize a list to store the data
data_list <- list()

# Loop through each family
for (fam in families) {
  # Read in the file as a vector
  family_segregating_SNPs_R <-
    read_table(
      paste0(
        "output/segregation/albopictus/parents_family_",
        fam,
        "_segregating_SNPs_R.txt"
      ),
      col_names = FALSE
    )[[1]]
  
  # Add the data to the list
  data_list[[fam]] <- family_segregating_SNPs_R
}

# Define a vector of color-blind friendly colors
colors <-
  c("#E69F00",
    "#56B4E9",
    "#009E73",
    "#F0E442",
    "#000000",
    "#CC79A7")

# Save combined Venn diagram to PDF
output_path <-
  here("output",
       "segregation",
       "albopictus",
       "segregating_SNPs_parents_6_venn.pdf")

# Create a PDF device
pdf(output_path, width = 5, height = 5)
par(mar = c(5, 5, 7, 2) + 0.1)  # Adjust margins (bottom, left, top, right)

# Create the 6-way Venn diagram with filled sets
venn(data_list,
     zcolor = colors,
     opacity = 0.4,
     box = FALSE,
     ellipse = FALSE,
     lty = "blank")

# Adjust the title position and size
title(
  main = "6-way Venn Diagram of Segregating SNPs",
      line = -1,
      cex.main = 0.8
  )

# Close the PDF device
dev.off()
```

```
## quartz_off_screen 
##                 2
```

```
# show the plot here
venn(data_list,
     zcolor = colors,
     opacity = 0.4,
     box = FALSE,
     ellipse = FALSE,
     lty = "blank")
```

No, it is not what you are thinking. We do not have only 5,249 SNPs
shared across all six families. Remember, we compared the genotypes of
the parents. We have six pairs of mosquitoes. We selected SNPs that were
heterozygous in one of the parents. If a SNP was homozygous in both
parents, we did not seletct it for testing within each family. If one of
the parents had a missing genotype call for a loci, we did not include
this loci as well. So, what we see is that we have around 5,249 SNPs
that are heterozygous in the male or females across all six families.
Most of the SNPs do not follow this pattern, probably due to runs of
homozygosity.

Create segregation table

```
# List all families
families <- c("fam1", "fam2", "fam3", "fam4", "fam5", "fam6")

# Initialize a list to store the data
data_list <- list()

# Loop through each family
for (fam in families) {
  # Read in the file as a vector
  family_segregating_SNPs_R <-
    read.table(
      paste0(
        "output/segregation/albopictus/parents_family_",
        fam,
        "_segregating_SNPs_R.txt"
      ),
      colClasses = "character",
      col.names = FALSE
    )[[1]]
  
  # Add the data to the list
  data_list[[fam]] <- family_segregating_SNPs_R
}

# Calculate the pairwise comparisons
pairwise_comp <- matrix(0, nrow = length(families), ncol = length(families))
colnames(pairwise_comp) <- families
rownames(pairwise_comp) <- families

for (i in 1:(length(families) - 1)) {
  for (j in (i + 1):length(families)) {
    shared <- length(intersect(data_list[[families[i]]], data_list[[families[j]]]))
    pairwise_comp[i, j] <- shared
    pairwise_comp[j, i] <- shared
  }
}

# Calculate the total shared and total unique for each family
total_shared <- length(Reduce(intersect, data_list))
total_unique <- sapply(data_list, function(x) length(x) - sum(x %in% unlist(data_list[-which(names(data_list) == names(x))])))

# Create a data frame with the updated columns
table_data <- data.frame(Family = families,
                         t(pairwise_comp),
                         Total_Shared = total_shared,
                         Total_Unique = total_unique)

# Create a flextable object
ft <- flextable(table_data)

# Print the table
print(ft)
```

```
## a flextable object.
## col_keys: `Family`, `fam1`, `fam2`, `fam3`, `fam4`, `fam5`, `fam6`, `Total_Shared`, `Total_Unique` 
## header has 1 row(s) 
## body has 6 row(s) 
## original dataset sample: 
##      Family  fam1  fam2  fam3  fam4  fam5  fam6 Total_Shared Total_Unique
## fam1   fam1     0 20656 21023 27897 27404 20882         5249        42116
## fam2   fam2 20656     0 33581 20982 20929 36360         5249        54871
## fam3   fam3 21023 33581     0 21326 21265 35885         5249        55484
## fam4   fam4 27897 20982 21326     0 29677 21247         5249        42684
## fam5   fam5 27404 20929 21265 29677     0 21107         5249        42720
```

```
# save the table
# Initialize Word document
doc <-
  read_docx() |>
  body_add_flextable(value = ft)

# Define the output path with 'here' library
output_path <- here(
  "output",
  "segregation",
  "albopictus",
  "albopictus_segregating_SNPS.docx"
  )

# Save the Word document
print(doc, target = output_path)
```

## 4. Calculate the expected and observed allele frequencies or counts

Plink has several utilities that we can use to find the expected and
observed counts. I will go over some of them.

### 4.1 Estimate frequencies of parents

```
# Define the list of family names
families=("fam1" "fam2" "fam3" "fam4" "fam5" "fam6")

# Iterate over each family
for fam_id in "${families[@]}"
do
    # Run Plink command with --freq flag
    plink \
    --allow-extra-chr \
    --bfile output/segregation/albopictus/parents_family_${fam_id} \
    --out output/segregation/albopictus/parents_freq_family_${fam_id} \
    --keep-allele-order \
    --extract output/segregation/albopictus/parents_family_${fam_id}_segregating_SNPs_python.txt \
    --freq \
    --silent 

    # Run Plink command with --freqx flag
    plink \
    --allow-extra-chr \
    --bfile output/segregation/albopictus/parents_family_${fam_id} \
    --out output/segregation/albopictus/parents_freq_family_${fam_id} \
    --keep-allele-order \
    --extract output/segregation/albopictus/parents_family_${fam_id}_segregating_SNPs_python.txt \
    --freqx \
    --silent
    
    # Run Plink command with --freq counts flag
    plink \
    --allow-extra-chr \
    --bfile output/segregation/albopictus/parents_family_${fam_id} \
    --out output/segregation/albopictus/parents_freq_family_${fam_id} \
    --keep-allele-order \
    --extract output/segregation/albopictus/parents_family_${fam_id}_segregating_SNPs_python.txt \
    --freq counts \
    --silent 
done
```

### 4.2 Check the allele count of one family

From Plink documentation:

```
.frqx (genotype count report)

Produced by --freqx. Valid input for --read-freq.

A text file with a header line, and then one line per variant with the following ten fields:

CHR Chromosome code
SNP Variant identifier
A1  Allele 1 (usually minor)
A2  Allele 2 (usually major)
C(HOM A1)   A1 homozygote count
C(HET)  Heterozygote count
C(HOM A2)   A2 homozygote count
C(HAP A1)   Haploid A1 count (includes male X chromosome)
C(HAP A2)   Haploid A2 count
C(MISSING)  Missing genotype count
```

```
head output/segregation/albopictus/parents_freq_family_fam1.frqx
```

```
## CHR  SNP A1  A2  C(HOM A1)   C(HET)  C(HOM A2)   C(HAP A1)   C(HAP A2)   C(MISSING)
## 1.1  AX-583035083    A   G   0   1   1   0   0   0
## 1.1  AX-583033342    C   G   1   0   1   0   0   0
## 1.1  AX-583035163    A   G   1   0   1   0   0   0
## 1.10 AX-583035257    T   C   0   1   1   0   0   0
## 1.10 AX-583033504    C   T   0   1   1   0   0   0
## 1.10 AX-583036983    C   T   1   0   1   0   0   0
## 1.10 AX-583035263    C   T   0   1   1   0   0   0
## 1.10 AX-583037044    G   A   1   1   0   0   0   0
## 1.10 AX-583037240    G   A   1   1   0   0   0   0
```

### 4.3 Check the minor allele count of one family

From Plink documentation:

```
.frq (basic allele frequency report)
Produced by --freq. Valid input for --read-freq.

A text file with a header line, and then one line per variant with the following six fields:

CHR Chromosome code
SNP Variant identifier
A1  Allele 1 (usually minor)
A2  Allele 2 (usually major)
MAF Allele 1 frequency
NCHROBS Number of allele observations
```

```
head output/segregation/albopictus/parents_freq_family_fam1.frq
```

```
##  CHR            SNP   A1   A2          MAF  NCHROBS
##  1.1   AX-583035083    A    G         0.25        4
##  1.1   AX-583033342    C    G          0.5        4
##  1.1   AX-583035163    A    G          0.5        4
## 1.10   AX-583035257    T    C         0.25        4
## 1.10   AX-583033504    C    T         0.25        4
## 1.10   AX-583036983    C    T          0.5        4
## 1.10   AX-583035263    C    T         0.25        4
## 1.10   AX-583037044    G    A         0.75        4
## 1.10   AX-583037240    G    A         0.75        4
```

### 4.4 Check the counts of each allele

From Plink documentation:

```
.frq.count (basic allele count report)
Produced by "--freq counts". Valid input for --read-freq.

A text file with a header line, and then one line per variant with the following seven fields:

CHR Chromosome code
SNP Variant identifier
A1  Allele 1 (usually minor)
A2  Allele 2 (usually major)
C1  Allele 1 count
C2  Allele 2 count
G0  Missing genotype count (so C1 + C2 + 2 * G0 is constant on autosomal variants)
```

```
head output/segregation/albopictus/parents_freq_family_fam1.frq.counts
```

```
##  CHR            SNP   A1   A2     C1     C2     G0
##  1.1   AX-583035083    A    G      1      3      0
##  1.1   AX-583033342    C    G      2      2      0
##  1.1   AX-583035163    A    G      2      2      0
## 1.10   AX-583035257    T    C      1      3      0
## 1.10   AX-583033504    C    T      1      3      0
## 1.10   AX-583036983    C    T      2      2      0
## 1.10   AX-583035263    C    T      1      3      0
## 1.10   AX-583037044    G    A      3      1      0
## 1.10   AX-583037240    G    A      3      1      0
```

### 4.5 Estimate frequencies for offspring

We can use the flag –freq counts to get the counts for each allele
for all SNPs. It will also create the “NCHROBS” or “Number of allele
observations”. Then we can use the “NCHROBS” value for each SNP, to
calculate the expected counts for each parental allele.

```
# Define the list of family IDs
families=("fam1" "fam2" "fam3" "fam4" "fam5" "fam6")

# Iterate over each family
for fam_id in "${families[@]}"
do
    # Run Plink command with --freq flag
    plink \
    --allow-extra-chr \
    --bfile output/segregation/albopictus/offspring_family_${fam_id} \
    --out output/segregation/albopictus/offspring_freq_family_${fam_id} \
    --keep-allele-order \
    --extract output/segregation/albopictus/parents_family_${fam_id}_segregating_SNPs_python.txt \
    --freq \
    --nonfounders \
    --silent 

    # Run Plink command with --freqx flag
    plink \
    --allow-extra-chr \
    --bfile output/segregation/albopictus/offspring_family_${fam_id} \
    --out output/segregation/albopictus/offspring_freq_family_${fam_id} \
    --keep-allele-order \
    --extract output/segregation/albopictus/parents_family_${fam_id}_segregating_SNPs_python.txt \
    --freqx \
    --nonfounders \
    --silent
    
    # Run Plink command with --freq counts flag
    plink \
    --allow-extra-chr \
    --bfile output/segregation/albopictus/offspring_family_${fam_id} \
    --out output/segregation/albopictus/offspring_freq_family_${fam_id} \
    --keep-allele-order \
    --extract output/segregation/albopictus/parents_family_${fam_id}_segregating_SNPs_python.txt \
    --freq counts \
    --nonfounders \
    --silent
done
```

Check results

```
head output/segregation/albopictus/offspring_freq_family_fam1.frqx
```

```
## CHR  SNP A1  A2  C(HOM A1)   C(HET)  C(HOM A2)   C(HAP A1)   C(HAP A2)   C(MISSING)
## 1.1  AX-583035083    A   G   1   24  19  0   0   0
## 1.1  AX-583033342    C   G   0   44  0   0   0   0
## 1.1  AX-583035163    A   G   0   17  27  0   0   0
## 1.10 AX-583035257    T   C   0   20  24  0   0   0
## 1.10 AX-583033504    C   T   0   20  17  0   0   7
## 1.10 AX-583036983    C   T   0   44  0   0   0   0
## 1.10 AX-583035263    C   T   24  0   20  0   0   0
## 1.10 AX-583037044    G   A   24  20  0   0   0   0
## 1.10 AX-583037240    G   A   20  24  0   0   0   0
```

Minor allele allele count of one family

```
head output/segregation/albopictus/offspring_freq_family_fam1.frq
```

```
##  CHR            SNP   A1   A2          MAF  NCHROBS
##  1.1   AX-583035083    A    G       0.2955       88
##  1.1   AX-583033342    C    G          0.5       88
##  1.1   AX-583035163    A    G       0.1932       88
## 1.10   AX-583035257    T    C       0.2273       88
## 1.10   AX-583033504    C    T       0.2703       74
## 1.10   AX-583036983    C    T          0.5       88
## 1.10   AX-583035263    C    T       0.5455       88
## 1.10   AX-583037044    G    A       0.7727       88
## 1.10   AX-583037240    G    A       0.7273       88
```

Counts of each allele

```
head output/segregation/albopictus/offspring_freq_family_fam1.frq.counts
```

```
##  CHR            SNP   A1   A2     C1     C2     G0
##  1.1   AX-583035083    A    G     26     62      0
##  1.1   AX-583033342    C    G     44     44      0
##  1.1   AX-583035163    A    G     17     71      0
## 1.10   AX-583035257    T    C     20     68      0
## 1.10   AX-583033504    C    T     20     54      7
## 1.10   AX-583036983    C    T     44     44      0
## 1.10   AX-583035263    C    T     48     40      0
## 1.10   AX-583037044    G    A     68     20      0
## 1.10   AX-583037240    G    A     64     24      0
```

### 4.6 Clean the Plink output files to import into R

The code below will convert the .frq files into csv files. Plink put
uneven number of tabs or spaces between the variables.

```
for file in output/segregation/albopictus/*.frq
do
  awk '{$1=$1; OFS=","}1' "$file" > "${file%.frq}.clean.csv"
done
```

Check one file

```
head output/segregation/albopictus/offspring_freq_family_fam1.clean.csv
```

```
## CHR,SNP,A1,A2,MAF,NCHROBS
## 1.1,AX-583035083,A,G,0.2955,88
## 1.1,AX-583033342,C,G,0.5,88
## 1.1,AX-583035163,A,G,0.1932,88
## 1.10,AX-583035257,T,C,0.2273,88
## 1.10,AX-583033504,C,T,0.2703,74
## 1.10,AX-583036983,C,T,0.5,88
## 1.10,AX-583035263,C,T,0.5455,88
## 1.10,AX-583037044,G,A,0.7727,88
## 1.10,AX-583037240,G,A,0.7273,88
```

### 4.7 Import frequency and counts

Here is the summary of what the code below does:

1. **Define family names:** A list of family names to
   be analyzed is defined and stored in the family\_names variable.
2. **Initialize storage lists:** Two empty lists,
   family\_parents and family\_offspring, are created to store parental and
   offspring data respectively for each family.
3. **Read and store data:** A for loop is implemented
   to iterate over the family names. For each family:

   - **File path construction:** File paths for the
     parent and offspring data are constructed using string concatenation
     with the paste0 function.
   - **Data read:** Parent and offspring data are read
     from the constructed file paths using the read.csv function, specifying
     column names and types.
   - **Data storage:** The read parental data is added to
     the family\_parents list for further processing.
4. **Calculation and update of frequencies and
   counts:** Another for loop is started to iterate over the family
   names. For each family:

   - **Data retrieval:** Offspring’s NCHROBS data and the
     corresponding parental data are retrieved from the family\_offspring and
     family\_parents lists respectively.
   - **Calculate expected frequencies and counts:**
     Expected frequencies and counts based on Minor Allele Frequency (MAF)
     for the parental data are calculated using the mutate function, adding
     these as new columns to the parental data.
   - **Update parental data:** The parental data in the
     family\_parents list is updated with the new calculated columns.
   - **Calculate observed frequencies and counts:**
     Observed frequencies and counts based on MAF for the offspring data are
     calculated using the mutate function, adding these as new columns to the
     offspring data.
   - **Update offspring data:** The family\_offspring list
     is updated with these newly calculated columns.

In summary, the main purpose of this R script below is to process and
calculate the expected and observed frequencies and counts of SNPs for
multiple families.

```
# Define the list of family names
family_names <-
  c("fam1", "fam2", "fam3", "fam4", "fam5", "fam6")

# Create empty lists to store the data for each family
family_parents <- list()
family_offspring <- list()

# Read and process the data for each family
for (family_name in family_names) {
  # Parental and offspring data paths
  parent_file_path <-
    paste0(
      "output/segregation/albopictus/parents_freq_family_",
      family_name,
      ".clean.csv"
    )
  offspring_file_path <-
    paste0(
      "output/segregation/albopictus/offspring_freq_family_",
      family_name,
      ".clean.csv"
    )

  # Read the input files
  family_parent <-
    read.csv(
      parent_file_path,
      stringsAsFactors = FALSE,
      col.names = c("CHR", "SNP", "A1", "A2", "MAF", "NCHROBS"),
      colClasses = c(
        "character",
        "character",
        "character",
        "character",
        "numeric",
        "numeric"
      )
    )
  family_offspring[[family_name]] <-
    read.csv(
      offspring_file_path,
      stringsAsFactors = FALSE,
      col.names = c("CHR", "SNP", "A1", "A2", "MAF", "NCHROBS"),
      colClasses = c(
        "character",
        "character",
        "character",
        "character",
        "numeric",
        "numeric"
      )
    )

  # Add the processed parental data to the list
  family_parents[[family_name]] <- family_parent
}

# Iterate over each family to calculate the expected and observed frequencies and counts
for (family_name in family_names) {
  # Get the offspring NCHROBS for this family
  offspring_NCHROBS <- family_offspring[[family_name]]$NCHROBS

  # Get the parental data for this family
  parental_data <- family_parents[[family_name]]

  # Calculate the expected frequencies and counts based on MAF
  parental_data <- parental_data |>
    mutate(
      Expected_A1 = 1 - MAF,
      Expected_A2 = MAF,
      Expected_A1_count = Expected_A1 * offspring_NCHROBS,
      Expected_A2_count = Expected_A2 * offspring_NCHROBS
    )

  # Update the parental data
  family_parents[[family_name]] <- parental_data

  # Calculate the observed frequencies and counts
  family_offspring[[family_name]] <-
    family_offspring[[family_name]] |>
    mutate(
      Observed_A1 = 1 - MAF,
      Observed_A2 = MAF,
      Observed_A1_count = Observed_A1 * NCHROBS,
      Observed_A2_count = Observed_A2 * NCHROBS
    )
}
```

Check the output

```
str(family_offspring)
```

```
## List of 6
##  $ fam1:'data.frame':    42116 obs. of  10 variables:
##   ..$ CHR              : chr [1:42116] "1.1" "1.1" "1.1" "1.10" ...
##   ..$ SNP              : chr [1:42116] "AX-583035083" "AX-583033342" "AX-583035163" "AX-583035257" ...
##   ..$ A1               : chr [1:42116] "A" "C" "A" "T" ...
##   ..$ A2               : chr [1:42116] "G" "G" "G" "C" ...
##   ..$ MAF              : num [1:42116] 0.295 0.5 0.193 0.227 0.27 ...
##   ..$ NCHROBS          : num [1:42116] 88 88 88 88 74 88 88 88 88 88 ...
##   ..$ Observed_A1      : num [1:42116] 0.705 0.5 0.807 0.773 0.73 ...
##   ..$ Observed_A2      : num [1:42116] 0.295 0.5 0.193 0.227 0.27 ...
##   ..$ Observed_A1_count: num [1:42116] 62 44 71 68 54 ...
##   ..$ Observed_A2_count: num [1:42116] 26 44 17 20 20 ...
##  $ fam2:'data.frame':    54871 obs. of  10 variables:
##   ..$ CHR              : chr [1:54871] "1.1" "1.1" "1.1" "1.1" ...
##   ..$ SNP              : chr [1:54871] "AX-581444870" "AX-583035083" "AX-583035163" "AX-583033370" ...
##   ..$ A1               : chr [1:54871] "C" "A" "A" "G" ...
##   ..$ A2               : chr [1:54871] "T" "G" "G" "A" ...
##   ..$ MAF              : num [1:54871] 0.452 0.425 0.452 0.691 0.309 ...
##   ..$ NCHROBS          : num [1:54871] 42 40 42 42 42 42 42 42 40 42 ...
##   ..$ Observed_A1      : num [1:54871] 0.548 0.575 0.548 0.309 0.691 ...
##   ..$ Observed_A2      : num [1:54871] 0.452 0.425 0.452 0.691 0.309 ...
##   ..$ Observed_A1_count: num [1:54871] 23 23 23 13 29 ...
##   ..$ Observed_A2_count: num [1:54871] 19 17 19 29 13 ...
##  $ fam3:'data.frame':    55484 obs. of  10 variables:
##   ..$ CHR              : chr [1:55484] "1.1" "1.1" "1.10" "1.10" ...
##   ..$ SNP              : chr [1:55484] "AX-581444870" "AX-583033387" "AX-583033504" "AX-583035355" ...
##   ..$ A1               : chr [1:55484] "C" "C" "C" "G" ...
##   ..$ A2               : chr [1:55484] "T" "T" "T" "C" ...
##   ..$ MAF              : num [1:55484] 0.182 0.326 0.5 0.391 0.37 ...
##   ..$ NCHROBS          : num [1:55484] 44 46 44 46 46 46 46 46 46 46 ...
##   ..$ Observed_A1      : num [1:55484] 0.818 0.674 0.5 0.609 0.63 ...
##   ..$ Observed_A2      : num [1:55484] 0.182 0.326 0.5 0.391 0.37 ...
##   ..$ Observed_A1_count: num [1:55484] 36 31 22 28 29 ...
##   ..$ Observed_A2_count: num [1:55484] 8 15 22 18 17 ...
##  $ fam4:'data.frame':    42684 obs. of  10 variables:
##   ..$ CHR              : chr [1:42684] "1.1" "1.1" "1.1" "1.1" ...
##   ..$ SNP              : chr [1:42684] "AX-583033342" "AX-583033370" "AX-583033387" "AX-583035211" ...
##   ..$ A1               : chr [1:42684] "C" "G" "C" "C" ...
##   ..$ A2               : chr [1:42684] "G" "A" "T" "T" ...
##   ..$ MAF              : num [1:42684] 0.259 0.759 0.241 0.481 0.463 ...
##   ..$ NCHROBS          : num [1:42684] 54 54 54 54 54 50 54 54 54 54 ...
##   ..$ Observed_A1      : num [1:42684] 0.741 0.241 0.759 0.518 0.537 ...
##   ..$ Observed_A2      : num [1:42684] 0.259 0.759 0.241 0.481 0.463 ...
##   ..$ Observed_A1_count: num [1:42684] 40 13 41 28 29 ...
##   ..$ Observed_A2_count: num [1:42684] 14 41 13 26 25 ...
##  $ fam5:'data.frame':    42720 obs. of  10 variables:
##   ..$ CHR              : chr [1:42720] "1.1" "1.1" "1.1" "1.1" ...
##   ..$ SNP              : chr [1:42720] "AX-583035163" "AX-583033370" "AX-583035194" "AX-583033387" ...
##   ..$ A1               : chr [1:42720] "A" "G" "A" "C" ...
##   ..$ A2               : chr [1:42720] "G" "A" "G" "T" ...
##   ..$ MAF              : num [1:42720] 0.583 0.735 0.528 0.469 0.2 ...
##   ..$ NCHROBS          : num [1:42720] 36 34 36 32 30 36 36 36 34 36 ...
##   ..$ Observed_A1      : num [1:42720] 0.417 0.265 0.472 0.531 0.8 ...
##   ..$ Observed_A2      : num [1:42720] 0.583 0.735 0.528 0.469 0.2 ...
##   ..$ Observed_A1_count: num [1:42720] 15 9 17 17 24 ...
##   ..$ Observed_A2_count: num [1:42720] 21 25 19 15 6 ...
##  $ fam6:'data.frame':    55205 obs. of  10 variables:
##   ..$ CHR              : chr [1:55205] "1.1" "1.1" "1.1" "1.1" ...
##   ..$ SNP              : chr [1:55205] "AX-581444870" "AX-583035083" "AX-583033370" "AX-583035194" ...
##   ..$ A1               : chr [1:55205] "C" "A" "G" "A" ...
##   ..$ A2               : chr [1:55205] "T" "G" "A" "G" ...
##   ..$ MAF              : num [1:55205] 0.0833 0.4167 0.7857 0.2143 0.2857 ...
##   ..$ NCHROBS          : num [1:55205] 12 12 14 14 14 14 14 14 14 14 ...
##   ..$ Observed_A1      : num [1:55205] 0.917 0.583 0.214 0.786 0.714 ...
##   ..$ Observed_A2      : num [1:55205] 0.0833 0.4167 0.7857 0.2143 0.2857 ...
##   ..$ Observed_A1_count: num [1:55205] 11 7 3 11 10 ...
##   ..$ Observed_A2_count: num [1:55205] 1 5 11 3 4 ...
```

```
str(family_parents)
```

```
## List of 6
##  $ fam1:'data.frame':    42116 obs. of  10 variables:
##   ..$ CHR              : chr [1:42116] "1.1" "1.1" "1.1" "1.10" ...
##   ..$ SNP              : chr [1:42116] "AX-583035083" "AX-583033342" "AX-583035163" "AX-583035257" ...
##   ..$ A1               : chr [1:42116] "A" "C" "A" "T" ...
##   ..$ A2               : chr [1:42116] "G" "G" "G" "C" ...
##   ..$ MAF              : num [1:42116] 0.25 0.5 0.5 0.25 0.25 0.5 0.25 0.75 0.75 0.25 ...
##   ..$ NCHROBS          : num [1:42116] 4 4 4 4 4 4 4 4 4 4 ...
##   ..$ Expected_A1      : num [1:42116] 0.75 0.5 0.5 0.75 0.75 0.5 0.75 0.25 0.25 0.75 ...
##   ..$ Expected_A2      : num [1:42116] 0.25 0.5 0.5 0.25 0.25 0.5 0.25 0.75 0.75 0.25 ...
##   ..$ Expected_A1_count: num [1:42116] 66 44 44 66 55.5 44 66 22 22 66 ...
##   ..$ Expected_A2_count: num [1:42116] 22 44 44 22 18.5 44 22 66 66 22 ...
##  $ fam2:'data.frame':    54871 obs. of  10 variables:
##   ..$ CHR              : chr [1:54871] "1.1" "1.1" "1.1" "1.1" ...
##   ..$ SNP              : chr [1:54871] "AX-581444870" "AX-583035083" "AX-583035163" "AX-583033370" ...
##   ..$ A1               : chr [1:54871] "C" "A" "A" "G" ...
##   ..$ A2               : chr [1:54871] "T" "G" "G" "A" ...
##   ..$ MAF              : num [1:54871] 0.5 0.5 0.25 0.75 0.25 0.25 0.25 0.75 0.25 0.5 ...
##   ..$ NCHROBS          : num [1:54871] 4 4 4 4 4 4 4 4 4 4 ...
##   ..$ Expected_A1      : num [1:54871] 0.5 0.5 0.75 0.25 0.75 0.75 0.75 0.25 0.75 0.5 ...
##   ..$ Expected_A2      : num [1:54871] 0.5 0.5 0.25 0.75 0.25 0.25 0.25 0.75 0.25 0.5 ...
##   ..$ Expected_A1_count: num [1:54871] 21 20 31.5 10.5 31.5 31.5 31.5 10.5 30 21 ...
##   ..$ Expected_A2_count: num [1:54871] 21 20 10.5 31.5 10.5 10.5 10.5 31.5 10 21 ...
##  $ fam3:'data.frame':    55484 obs. of  10 variables:
##   ..$ CHR              : chr [1:55484] "1.1" "1.1" "1.10" "1.10" ...
##   ..$ SNP              : chr [1:55484] "AX-581444870" "AX-583033387" "AX-583033504" "AX-583035355" ...
##   ..$ A1               : chr [1:55484] "C" "C" "C" "G" ...
##   ..$ A2               : chr [1:55484] "T" "T" "T" "C" ...
##   ..$ MAF              : num [1:55484] 0.25 0.25 0.5 0.25 0.5 0.5 0.25 0.25 0.25 0.75 ...
##   ..$ NCHROBS          : num [1:55484] 4 4 4 4 4 4 4 4 4 4 ...
##   ..$ Expected_A1      : num [1:55484] 0.75 0.75 0.5 0.75 0.5 0.5 0.75 0.75 0.75 0.25 ...
##   ..$ Expected_A2      : num [1:55484] 0.25 0.25 0.5 0.25 0.5 0.5 0.25 0.25 0.25 0.75 ...
##   ..$ Expected_A1_count: num [1:55484] 33 34.5 22 34.5 23 23 34.5 34.5 34.5 11.5 ...
##   ..$ Expected_A2_count: num [1:55484] 11 11.5 22 11.5 23 23 11.5 11.5 11.5 34.5 ...
##  $ fam4:'data.frame':    42684 obs. of  10 variables:
##   ..$ CHR              : chr [1:42684] "1.1" "1.1" "1.1" "1.1" ...
##   ..$ SNP              : chr [1:42684] "AX-583033342" "AX-583033370" "AX-583033387" "AX-583035211" ...
##   ..$ A1               : chr [1:42684] "C" "G" "C" "C" ...
##   ..$ A2               : chr [1:42684] "G" "A" "T" "T" ...
##   ..$ MAF              : num [1:42684] 0.25 0.75 0.5 0.5 0.5 0.5 0.25 0.25 0.25 0.75 ...
##   ..$ NCHROBS          : num [1:42684] 4 4 4 4 4 4 4 4 4 4 ...
##   ..$ Expected_A1      : num [1:42684] 0.75 0.25 0.5 0.5 0.5 0.5 0.75 0.75 0.75 0.25 ...
##   ..$ Expected_A2      : num [1:42684] 0.25 0.75 0.5 0.5 0.5 0.5 0.25 0.25 0.25 0.75 ...
##   ..$ Expected_A1_count: num [1:42684] 40.5 13.5 27 27 27 25 40.5 40.5 40.5 13.5 ...
##   ..$ Expected_A2_count: num [1:42684] 13.5 40.5 27 27 27 25 13.5 13.5 13.5 40.5 ...
##  $ fam5:'data.frame':    42720 obs. of  10 variables:
##   ..$ CHR              : chr [1:42720] "1.1" "1.1" "1.1" "1.1" ...
##   ..$ SNP              : chr [1:42720] "AX-583035163" "AX-583033370" "AX-583035194" "AX-583033387" ...
##   ..$ A1               : chr [1:42720] "A" "G" "A" "C" ...
##   ..$ A2               : chr [1:42720] "G" "A" "G" "T" ...
##   ..$ MAF              : num [1:42720] 0.25 0.5 0.5 0.75 0.25 0.5 0.25 0.75 0.5 0.75 ...
##   ..$ NCHROBS          : num [1:42720] 4 4 4 4 4 4 4 4 4 4 ...
##   ..$ Expected_A1      : num [1:42720] 0.75 0.5 0.5 0.25 0.75 0.5 0.75 0.25 0.5 0.25 ...
##   ..$ Expected_A2      : num [1:42720] 0.25 0.5 0.5 0.75 0.25 0.5 0.25 0.75 0.5 0.75 ...
##   ..$ Expected_A1_count: num [1:42720] 27 17 18 8 22.5 18 27 9 17 9 ...
##   ..$ Expected_A2_count: num [1:42720] 9 17 18 24 7.5 18 9 27 17 27 ...
##  $ fam6:'data.frame':    55205 obs. of  10 variables:
##   ..$ CHR              : chr [1:55205] "1.1" "1.1" "1.1" "1.1" ...
##   ..$ SNP              : chr [1:55205] "AX-581444870" "AX-583035083" "AX-583033370" "AX-583035194" ...
##   ..$ A1               : chr [1:55205] "C" "A" "G" "A" ...
##   ..$ A2               : chr [1:55205] "T" "G" "A" "G" ...
##   ..$ MAF              : num [1:55205] 0.25 0.25 0.75 0.25 0.25 0.5 0.25 0.75 0.25 0.75 ...
##   ..$ NCHROBS          : num [1:55205] 4 4 4 4 4 4 4 4 4 4 ...
##   ..$ Expected_A1      : num [1:55205] 0.75 0.75 0.25 0.75 0.75 0.5 0.75 0.25 0.75 0.25 ...
##   ..$ Expected_A2      : num [1:55205] 0.25 0.25 0.75 0.25 0.25 0.5 0.25 0.75 0.25 0.75 ...
##   ..$ Expected_A1_count: num [1:55205] 9 9 3.5 10.5 10.5 7 10.5 3.5 10.5 3.5 ...
##   ..$ Expected_A2_count: num [1:55205] 3 3 10.5 3.5 3.5 7 3.5 10.5 3.5 10.5 ...
```

Here are a few possibilities to consider if our observed frequencies
are showing a wider range of values than expected:

**1. Sample Size:** - If our sample sizes are small,
this could result in greater variation in the observed frequencies due
to the inherent randomness in sampling.

**2. Sampling Bias:** - If the samples are not random,
or if certain genotypes are more likely to be sampled than others, this
could skew the observed frequencies.

**3. Genetic Drift:** - This is a random change in
allele frequencies that occurs. Over time, it can lead to large changes
in allele frequencies, especially if the population size is small.

**4. Genetic Linkage:** - If the SNPs are close to each
other on a chromosome, they might be inherited together more often than
would be expected by chance, leading to non-random associations between
alleles.

**5. Non-random Mating:** - If mating is not random
within the population, this could also skew the observed
frequencies.

## 5. Data Visualization

We can prepare the data for plotting with the code below. In summary,
the code does:

**1. Prepare tibbles for storing results:** - The
“result\_df\_all” and “result\_df\_shared” tibbles are initialized as empty
data structures to store the results.

**2. Define list names:** - A vector “list\_names” is
defined to store the names of the lists (e.g., family names) to be
analyzed.

**3. Define intervals and labels:** - The “intervals”
vector is defined as a sequence of values from 0 to 1 with a step of
0.1. - The “interval\_labels” vector is defined to store the labels for
each interval.

**4. Initialize epsilon value:** - The epsilon value is
set as a small value to avoid exact bin border values.

**5. Retrieve SNP lists from each family:** - SNP lists
from both parents and offspring are obtained for each family using the
“lapply” function and stored in “all\_offspring\_snps” and
“all\_parents\_snps” respectively.

**6. Find shared SNPs among families:** - The Reduce
function is used to find the SNPs that are common to all families by
taking the intersection of all offspring and parent SNP lists.

**7. Loop over list names:** - A loop is implemented to
iterate over each family in the “list\_names” vector.

**8. Offspring processing for all SNPs:** - Offspring
data is processed for all SNPs in the current family: - Observed allele
frequencies are binned into intervals using the “cut” function. - The
data is transformed to a longer format using “pivot\_longer”. - Grouping
and summarization are performed based on family, type, variable, and
interval using “group\_by” and “summarize”. - The processed data is added
to “tmp\_offspring\_all”.

**9. Parents processing for all SNPs:** - Parental data
is processed for all SNPs in the current family following a similar
process as in step 8.

**10. Combine all SNPs for analysis:** - The processed
offspring and parental data for all SNPs in the current family are
combined and added to “result\_df\_all”.

**11. Offspring processing for shared SNPs:** -
Offspring data is processed for shared SNPs among families (common
SNPs): - Filtering is applied to select only the shared SNPs. - Observed
allele frequencies are binned into intervals using the “cut” function. -
The data is transformed to a longer format using “pivot\_longer”. -
Grouping and summarization are performed based on family, type,
variable, and interval using “group\_by” and “summarize”. - The processed
data is added to “tmp\_offspring\_shared”.

**12. Parents processing for shared SNPs:** - Parental
data is processed for shared SNPs among families (common SNPs) following
a similar process as in step 11.

**13. Combine shared SNPs for analysis:** - The
processed offspring and parental data for shared SNPs in the current
family are combined and added to “result\_df\_shared”.

**14. Add a new variable for faceting:** - A new
variable “Family\_Type” is created by combining family and type
information in “result\_df\_all” and “result\_df\_shared”.

**15. Data wrangling steps before plotting:** - Variable
names in “result\_df\_all” and “result\_df\_shared” are modified by removing
the “Interval\_” prefix.

**16. Set desired order for variable levels:** - The
“desired\_order” vector is defined to specify the desired order of
variable levels. - Variable columns in “result\_df\_all” and
“result\_df\_shared” are converted to factors with the desired order using
the “factor” function.

```
# Prepare two empty tibbles to store the results
result_df_all <- tibble()
result_df_shared <- tibble()

# List names
list_names <-
  c(
    "fam1", "fam2", "fam3", "fam4", "fam5", "fam6"
  )

# Define intervals and labels
intervals <-
  seq(
    0, 1, 0.1
  )
interval_labels <-
  c(
    "0 ~ 0.1",
    "0.1 ~ 0.2",
    "0.2 ~ 0.3",
    "0.3 ~ 0.4",
    "0.4 ~ 0.5",
    "0.5 ~ 0.6",
    "0.6 ~ 0.7",
    "0.7 ~ 0.8",
    "0.8 ~ 0.9",
    "0.9 ~ 1"
  )

# Initialize a small epsilon value (we add this tiny tiny number to our values, so when we bin the data, we will not have values at the exact bin border)
epsilon <-
  .Machine$double.eps^0.5

# Get the SNP lists from each family in both parents and offspring
all_offspring_snps <-
  lapply(
    family_offspring, function(df) {
      df$SNP
    }
  )

all_parents_snps <- lapply(
  family_parents, function(df) {
    df$SNP
  }
)

# Find SNPs that are common to all families
shared_snps <-
  Reduce(
    intersect,
    c(
      all_offspring_snps, all_parents_snps
    )
  )

# Loop over both lists
for (i in seq_along(list_names)) {
  # Offspring
  tmp_offspring <- family_offspring[[i]]

  # Parents
  tmp_parents <- family_parents[[i]]

  # Offspring processing for all SNPs
  tmp_offspring_all <- tmp_offspring |>
    mutate(
      Interval_Observed_A1 = cut(
        Observed_A1,
        breaks = intervals + epsilon,
        labels = interval_labels
      ),
      Interval_Observed_A2 = cut(
        Observed_A2,
        breaks = intervals + epsilon,
        labels = interval_labels
      )
    ) |>
    pivot_longer(
      cols = starts_with("Interval"),
      names_to = "Variable",
      values_to = "Interval"
    ) |>
    group_by(
      Family = list_names[i],
      Type = "Offspring",
      Variable,
      Interval
    ) |>
    summarize(
      Count = n(), .groups = "drop"
    ) |>
    ungroup()

  # Parents processing for all SNPs
  tmp_parents_all <-
    tmp_parents |>
    mutate(
      Interval_Expected_A1 = cut(
        Expected_A1,
        breaks = intervals + epsilon,
        labels = interval_labels
      ),
      Interval_Expected_A2 = cut(
        Expected_A2,
        breaks = intervals + epsilon,
        labels = interval_labels
      )
    ) |>
    pivot_longer(
      cols = starts_with("Interval"),
      names_to = "Variable",
      values_to = "Interval"
    ) |>
    group_by(
      Family = list_names[i],
      Type = "Parents",
      Variable,
      Interval
    ) |>
    summarize(
      Count = n(), .groups = "drop"
    ) |>
    ungroup()

  # Combine all SNPs
  result_df_all <-
    bind_rows(
      result_df_all,
      tmp_offspring_all,
      tmp_parents_all
    )

  # Offspring processing for shared SNPs
  tmp_offspring_shared <-
    tmp_offspring |>
    filter(
      SNP %in% shared_snps
    ) |>
    mutate(
      Interval_Observed_A1 = cut(
        Observed_A1,
        breaks = intervals + epsilon,
        labels = interval_labels
      ),
      Interval_Observed_A2 = cut(
        Observed_A2,
        breaks = intervals + epsilon,
        labels = interval_labels
      )
    ) |>
    pivot_longer(
      cols = starts_with("Interval"),
      names_to = "Variable",
      values_to = "Interval"
    ) |>
    group_by(
      Family = list_names[i],
      Type = "Offspring",
      Variable,
      Interval
    ) |>
    summarize(
      Count = n(), .groups = "drop"
    ) |>
    ungroup()

  # Parents processing for shared SNPs
  tmp_parents_shared <-
    tmp_parents |>
    filter(
      SNP %in% shared_snps
    ) |>
    mutate(
      Interval_Expected_A1 = cut(
        Expected_A1,
        breaks = intervals + epsilon,
        labels = interval_labels
      ),
      Interval_Expected_A2 = cut(
        Expected_A2,
        breaks = intervals + epsilon,
        labels = interval_labels
      )
    ) |>
    pivot_longer(
      cols = starts_with("Interval"),
      names_to = "Variable",
      values_to = "Interval"
    ) |>
    group_by(
      Family = list_names[i],
      Type = "Parents",
      Variable,
      Interval
    ) |>
    summarize(
      Count = n(), .groups = "drop"
    ) |>
    ungroup()

  # Combine shared SNPs
  result_df_shared <-
    bind_rows(
      result_df_shared,
      tmp_offspring_shared,
      tmp_parents_shared
    )
}

# Add a new variable for faceting
result_df_all <-
  result_df_all |> mutate(
    Family_Type = paste(
      Family,
      Type,
      sep = "_"
    )
  )
result_df_shared <-
  result_df_shared |>
  mutate(
    Family_Type = paste(
      Family,
      Type,
      sep = "_"
    )
  )

# After data wrangling step before the plot
result_df_all$Variable <-
  str_remove(
    result_df_all$Variable,
    "Interval_"
  )
result_df_shared$Variable <-
  str_remove(
    result_df_shared$Variable,
    "Interval_"
  )

# Set desired order
desired_order <-
  c(
    "Expected_A1",
    "Observed_A1",
    "Expected_A2",
    "Observed_A2"
  )

# Convert Variable to a factor with desired levels
result_df_all$Variable <-
  factor(
    result_df_all$Variable,
    levels = desired_order
  )
result_df_shared$Variable <-
  factor(
    result_df_shared$Variable,
    levels = desired_order
  )
```

### 5.1 Get the unique total SNP count across all families that we can test

```
# count of SNPs across all families
offspring_SNPs <-
  unlist(
    lapply(
      family_offspring, function(df) {
        df$SNP
      }
    )
  )
parent_SNPs <- unlist(lapply(family_parents, function(df) {
  df$SNP
}))

# Combine offspring and parents SNPs
all_SNPs <- c(offspring_SNPs, parent_SNPs)

# Get the number of unique SNPs
num_unique_SNPs <- length(unique(all_SNPs))

print(num_unique_SNPs)
```

```
## [1] 101400
```

### 5.2 Get the “shared” SNPs (genotyped in all 6 families)

These are the loci for which one parent was heterozygous in all six
families. Most of the loci do not follow this trend. It could be due to
selection, drift, or other factors.

```
# Initialize shared_snps with the SNPs of the first family
shared_snps <-
  family_offspring[[1]]$SNP

# Loop over the rest of the lists
for (i in 2:length(list_names)) {
  # Get the intersection of shared_snps and the SNPs of the current family
  shared_snps <- intersect(shared_snps, family_offspring[[i]]$SNP)
}

# The length of shared_snps gives the total number of SNPs shared among all families
num_shared_snps <-
  length(shared_snps)

print(num_shared_snps)
```

```
## [1] 5249
```

### 5.3 SNPs “Shared” among at least two families

We can count how many loci we can test that are heterozygous in at
least one parent in two crosses

```
# Combine SNPs from all families into a single vector
all_snps <-
  c(family_offspring[[1]]$SNP,
    family_offspring[[2]]$SNP,
    family_offspring[[3]]$SNP)

# Count the occurrence of each SNP
snp_counts <- table(all_snps)

# Get the SNPs that occur in at least two families
shared_snps <- names(snp_counts[snp_counts >= 2])

# The length of shared_snps gives the total number of SNPs shared among at least two families
num_shared_snps <- length(shared_snps)

print(num_shared_snps)
```

```
## [1] 49390
```

### 5.4 Plot allele counts

Now we can plot the data with the allele counts

```
# source function theme
source(
  here(
    "scripts", "analysis", "my_theme.R"
  )
)

# create plot with the frequencies - use result_df_all for all SNPs or result_df_shared for the shared SNPs among the three families
ggplot(
  result_df_shared,
  aes(
    x = Interval,
    y = Count,
    fill = Variable
  )
) +
  geom_bar(
    stat = "identity",
    color = "lightgray",
    width = 0.9,
    position = "dodge"
  ) +
  geom_text(
    aes(
      label = scales::comma(Count)
    ),
    size = 2,
    position = position_dodge(width = 0.9)
  ) +
  facet_grid(
    Family ~ Variable,
    scales = "free_y", space = "free"
  ) +
  labs(
    x = "Frequency",
    y = "Count",
    title = "Binned allele counts for the 'recommended' SNPs from Axiom Suite",
    caption = "Allele counts of 101,400 SNPs across all families."
    # caption = "Allele counts of 5,249 SNPs shared among the families."
  ) +
  scale_fill_manual(
    values = c(
      "Expected_A1" = "#56B4E9",
      "Expected_A2" = "#E69F00",
      "Observed_A1" = "#009E73",
      "Observed_A2" = "#F0E442"
    ),
    guide = "none"
  ) +
  scale_y_continuous(
    labels = scales::comma, expand = c(0.15, 0.15)
  ) +
  scale_x_discrete(
    drop = FALSE
  ) +
  my_theme() +
  theme(
    plot.caption = element_text(
      hjust = 1,
      margin = margin(t = 10),
      face = "italic"
    ),
    legend.position = "none",
    # Remove the legend
    panel.spacing = unit(0.5, "lines"),
    axis.text.y = element_text(size = 8),
    plot.title = element_text(face = "bold")
  ) +
  coord_flip()
```

```
# save plot
ggsave(
  here(
    "output",
    "segregation",
    "albopictus",
    "allele_counts_all_SNPS.pdf"
    # "allele_frequencies_shared_SNPS.pdf"
  ),
  width = 9,
  height = 10,
  units = "in"
)
```

We see some NAs in the offspring. It is because we found the
segregating loci in each cross and extracted the list of SNPs from the
offspring. However, some of this SNPs had missing genotype calls in the
offspring, and therefore we have NAs (no counts). We need to remove them
later on in both parental and offspring data frames.

### 5.5 Pie plot of the counts

```
# Calculate percentages for each pie
result_df_pie <-
  result_df_shared |>
  group_by(
    Family, Type, Variable, Interval
  ) |>
  summarise(
    count = sum(Count), .groups = "drop"
  ) |>
  group_by(
    Family, Type, Variable
  ) |>
  mutate(
    percentage = count / sum(count) * 100
  ) |>
  ungroup() |>
  group_by(
    Family, Type, Variable
  ) |>
  mutate(
    total_count = sum(count)
  ) |>
  mutate(
    proportional_count = count / total_count
  ) |>
  ungroup()

# Generate palette with as many colors as intervals
color_palette <-
  colorRampPalette(brewer.pal(11, "Set3"))(length(unique(result_df_pie$Interval)))

# Plot
ggplot(
  result_df_pie, aes(x = "", y = count, fill = Interval)
) +
  geom_bar(
    width = 1, stat = "identity"
  ) +
  geom_text_repel(
    aes(
      y = count / 2,
      label = paste0(
        round(percentage, 1),
        "%"
      )
    ),
    position = position_stack(vjust = 0.0),
    size = 3,
    max.overlaps = Inf,
    force = 10
  ) + # Increase max.overlaps and force
  coord_polar(
    "y",
    start = 0
  ) +
  facet_grid(
    Family ~ Variable
  ) +
  my_theme() +
  theme(
    axis.title.x = element_blank(),
    axis.title.y = element_blank(),
    panel.background = element_blank(),
    axis.text.x = element_blank(),
    axis.ticks = element_blank(),
    strip.text.x = element_text(
      size = 8,
      colour = "black",
      face = "bold"
    ),
    strip.text.y = element_text(
      size = 8,
      colour = "black",
      face = "bold"
    )
  ) +
  scale_fill_manual(
    values = color_palette
  ) +
  labs(
    title = "Pie Chart of Allele Counts of 101,400 SNPs across all families",
    # title = "Pie Chart of Allele Counts of 5,249 SNPs genotyped in all families",
    fill = "Interval"
  )
```

```
# Save the plot
ggsave(
  here(
    "output",
    "segregation",
    "albopictus",
    "allele_counts_pie_plots_all_SNPs.pdf"
    # "allele_counts_pie_plots_shared_SNPs.pdf"
  ),
  width = 9,
  height = 10,
  units = "in"
)
```

### 5.6 Check for NAs

We see that we have some missing genotype calls for some SNPs in the
offspring. We can check how many we have with the code below.

```
# Count NAs in each column of family_parents
cat("NAs in family_data_parents:")
```

```
## NAs in family_data_parents:
```

```
print(colSums(sapply(family_parents, function(df) colSums(is.na(df)))))
```

```
## fam1 fam2 fam3 fam4 fam5 fam6 
##    0    0    0    0    0    0
```

```
# Count NAs in each column of family_offspring
cat("NAs in family_data_parents:")
```

```
## NAs in family_data_parents:
```

```
print(colSums(sapply(family_offspring, function(df) colSums(is.na(df)))))
```

```
## fam1 fam2 fam3 fam4 fam5 fam6 
##    5   40   55   55   65   55
```

## 6. Tidying the data for statistical comparisons

First we remove SNPs within each family that are missing in the
offspring

### 6.1 Remove NAs

```
# Remove NA values from family_data_parents
family_parents_2 <- lapply(family_parents, function(df) {
  df |>
    drop_na(SNP, starts_with("Expected"))
})

# Remove NA values from family_data_offspring
family_offspring_2 <- lapply(family_offspring, function(df) {
  df |>
    drop_na(SNP, starts_with("Observed"))
})
```

Check if it worked

```
# Count NAs in each column of family_parents
cat("NAs in family_data_parents:")
```

```
## NAs in family_data_parents:
```

```
print(colSums(sapply(family_parents_2, function(df) colSums(is.na(df)))))
```

```
## fam1 fam2 fam3 fam4 fam5 fam6 
##    0    0    0    0    0    0
```

```
# Count NAs in each column of family_offspring
cat("NAs in family_data_parents:")
```

```
## NAs in family_data_parents:
```

```
print(colSums(sapply(family_offspring_2, function(df) colSums(is.na(df)))))
```

```
## fam1 fam2 fam3 fam4 fam5 fam6 
##    0    0    0    0    0    0
```

Check the output

```
str(family_parents_2)
```

```
## List of 6
##  $ fam1:'data.frame':    42116 obs. of  10 variables:
##   ..$ CHR              : chr [1:42116] "1.1" "1.1" "1.1" "1.10" ...
##   ..$ SNP              : chr [1:42116] "AX-583035083" "AX-583033342" "AX-583035163" "AX-583035257" ...
##   ..$ A1               : chr [1:42116] "A" "C" "A" "T" ...
##   ..$ A2               : chr [1:42116] "G" "G" "G" "C" ...
##   ..$ MAF              : num [1:42116] 0.25 0.5 0.5 0.25 0.25 0.5 0.25 0.75 0.75 0.25 ...
##   ..$ NCHROBS          : num [1:42116] 4 4 4 4 4 4 4 4 4 4 ...
##   ..$ Expected_A1      : num [1:42116] 0.75 0.5 0.5 0.75 0.75 0.5 0.75 0.25 0.25 0.75 ...
##   ..$ Expected_A2      : num [1:42116] 0.25 0.5 0.5 0.25 0.25 0.5 0.25 0.75 0.75 0.25 ...
##   ..$ Expected_A1_count: num [1:42116] 66 44 44 66 55.5 44 66 22 22 66 ...
##   ..$ Expected_A2_count: num [1:42116] 22 44 44 22 18.5 44 22 66 66 22 ...
##  $ fam2:'data.frame':    54871 obs. of  10 variables:
##   ..$ CHR              : chr [1:54871] "1.1" "1.1" "1.1" "1.1" ...
##   ..$ SNP              : chr [1:54871] "AX-581444870" "AX-583035083" "AX-583035163" "AX-583033370" ...
##   ..$ A1               : chr [1:54871] "C" "A" "A" "G" ...
##   ..$ A2               : chr [1:54871] "T" "G" "G" "A" ...
##   ..$ MAF              : num [1:54871] 0.5 0.5 0.25 0.75 0.25 0.25 0.25 0.75 0.25 0.5 ...
##   ..$ NCHROBS          : num [1:54871] 4 4 4 4 4 4 4 4 4 4 ...
##   ..$ Expected_A1      : num [1:54871] 0.5 0.5 0.75 0.25 0.75 0.75 0.75 0.25 0.75 0.5 ...
##   ..$ Expected_A2      : num [1:54871] 0.5 0.5 0.25 0.75 0.25 0.25 0.25 0.75 0.25 0.5 ...
##   ..$ Expected_A1_count: num [1:54871] 21 20 31.5 10.5 31.5 31.5 31.5 10.5 30 21 ...
##   ..$ Expected_A2_count: num [1:54871] 21 20 10.5 31.5 10.5 10.5 10.5 31.5 10 21 ...
##  $ fam3:'data.frame':    55484 obs. of  10 variables:
##   ..$ CHR              : chr [1:55484] "1.1" "1.1" "1.10" "1.10" ...
##   ..$ SNP              : chr [1:55484] "AX-581444870" "AX-583033387" "AX-583033504" "AX-583035355" ...
##   ..$ A1               : chr [1:55484] "C" "C" "C" "G" ...
##   ..$ A2               : chr [1:55484] "T" "T" "T" "C" ...
##   ..$ MAF              : num [1:55484] 0.25 0.25 0.5 0.25 0.5 0.5 0.25 0.25 0.25 0.75 ...
##   ..$ NCHROBS          : num [1:55484] 4 4 4 4 4 4 4 4 4 4 ...
##   ..$ Expected_A1      : num [1:55484] 0.75 0.75 0.5 0.75 0.5 0.5 0.75 0.75 0.75 0.25 ...
##   ..$ Expected_A2      : num [1:55484] 0.25 0.25 0.5 0.25 0.5 0.5 0.25 0.25 0.25 0.75 ...
##   ..$ Expected_A1_count: num [1:55484] 33 34.5 22 34.5 23 23 34.5 34.5 34.5 11.5 ...
##   ..$ Expected_A2_count: num [1:55484] 11 11.5 22 11.5 23 23 11.5 11.5 11.5 34.5 ...
##  $ fam4:'data.frame':    42684 obs. of  10 variables:
##   ..$ CHR              : chr [1:42684] "1.1" "1.1" "1.1" "1.1" ...
##   ..$ SNP              : chr [1:42684] "AX-583033342" "AX-583033370" "AX-583033387" "AX-583035211" ...
##   ..$ A1               : chr [1:42684] "C" "G" "C" "C" ...
##   ..$ A2               : chr [1:42684] "G" "A" "T" "T" ...
##   ..$ MAF              : num [1:42684] 0.25 0.75 0.5 0.5 0.5 0.5 0.25 0.25 0.25 0.75 ...
##   ..$ NCHROBS          : num [1:42684] 4 4 4 4 4 4 4 4 4 4 ...
##   ..$ Expected_A1      : num [1:42684] 0.75 0.25 0.5 0.5 0.5 0.5 0.75 0.75 0.75 0.25 ...
##   ..$ Expected_A2      : num [1:42684] 0.25 0.75 0.5 0.5 0.5 0.5 0.25 0.25 0.25 0.75 ...
##   ..$ Expected_A1_count: num [1:42684] 40.5 13.5 27 27 27 25 40.5 40.5 40.5 13.5 ...
##   ..$ Expected_A2_count: num [1:42684] 13.5 40.5 27 27 27 25 13.5 13.5 13.5 40.5 ...
##  $ fam5:'data.frame':    42720 obs. of  10 variables:
##   ..$ CHR              : chr [1:42720] "1.1" "1.1" "1.1" "1.1" ...
##   ..$ SNP              : chr [1:42720] "AX-583035163" "AX-583033370" "AX-583035194" "AX-583033387" ...
##   ..$ A1               : chr [1:42720] "A" "G" "A" "C" ...
##   ..$ A2               : chr [1:42720] "G" "A" "G" "T" ...
##   ..$ MAF              : num [1:42720] 0.25 0.5 0.5 0.75 0.25 0.5 0.25 0.75 0.5 0.75 ...
##   ..$ NCHROBS          : num [1:42720] 4 4 4 4 4 4 4 4 4 4 ...
##   ..$ Expected_A1      : num [1:42720] 0.75 0.5 0.5 0.25 0.75 0.5 0.75 0.25 0.5 0.25 ...
##   ..$ Expected_A2      : num [1:42720] 0.25 0.5 0.5 0.75 0.25 0.5 0.25 0.75 0.5 0.75 ...
##   ..$ Expected_A1_count: num [1:42720] 27 17 18 8 22.5 18 27 9 17 9 ...
##   ..$ Expected_A2_count: num [1:42720] 9 17 18 24 7.5 18 9 27 17 27 ...
##  $ fam6:'data.frame':    55205 obs. of  10 variables:
##   ..$ CHR              : chr [1:55205] "1.1" "1.1" "1.1" "1.1" ...
##   ..$ SNP              : chr [1:55205] "AX-581444870" "AX-583035083" "AX-583033370" "AX-583035194" ...
##   ..$ A1               : chr [1:55205] "C" "A" "G" "A" ...
##   ..$ A2               : chr [1:55205] "T" "G" "A" "G" ...
##   ..$ MAF              : num [1:55205] 0.25 0.25 0.75 0.25 0.25 0.5 0.25 0.75 0.25 0.75 ...
##   ..$ NCHROBS          : num [1:55205] 4 4 4 4 4 4 4 4 4 4 ...
##   ..$ Expected_A1      : num [1:55205] 0.75 0.75 0.25 0.75 0.75 0.5 0.75 0.25 0.75 0.25 ...
##   ..$ Expected_A2      : num [1:55205] 0.25 0.25 0.75 0.25 0.25 0.5 0.25 0.75 0.25 0.75 ...
##   ..$ Expected_A1_count: num [1:55205] 9 9 3.5 10.5 10.5 7 10.5 3.5 10.5 3.5 ...
##   ..$ Expected_A2_count: num [1:55205] 3 3 10.5 3.5 3.5 7 3.5 10.5 3.5 10.5 ...
```

```
str(family_offspring_2)
```

```
## List of 6
##  $ fam1:'data.frame':    42115 obs. of  10 variables:
##   ..$ CHR              : chr [1:42115] "1.1" "1.1" "1.1" "1.10" ...
##   ..$ SNP              : chr [1:42115] "AX-583035083" "AX-583033342" "AX-583035163" "AX-583035257" ...
##   ..$ A1               : chr [1:42115] "A" "C" "A" "T" ...
##   ..$ A2               : chr [1:42115] "G" "G" "G" "C" ...
##   ..$ MAF              : num [1:42115] 0.295 0.5 0.193 0.227 0.27 ...
##   ..$ NCHROBS          : num [1:42115] 88 88 88 88 74 88 88 88 88 88 ...
##   ..$ Observed_A1      : num [1:42115] 0.705 0.5 0.807 0.773 0.73 ...
##   ..$ Observed_A2      : num [1:42115] 0.295 0.5 0.193 0.227 0.27 ...
##   ..$ Observed_A1_count: num [1:42115] 62 44 71 68 54 ...
##   ..$ Observed_A2_count: num [1:42115] 26 44 17 20 20 ...
##  $ fam2:'data.frame':    54863 obs. of  10 variables:
##   ..$ CHR              : chr [1:54863] "1.1" "1.1" "1.1" "1.1" ...
##   ..$ SNP              : chr [1:54863] "AX-581444870" "AX-583035083" "AX-583035163" "AX-583033370" ...
##   ..$ A1               : chr [1:54863] "C" "A" "A" "G" ...
##   ..$ A2               : chr [1:54863] "T" "G" "G" "A" ...
##   ..$ MAF              : num [1:54863] 0.452 0.425 0.452 0.691 0.309 ...
##   ..$ NCHROBS          : num [1:54863] 42 40 42 42 42 42 42 42 40 42 ...
##   ..$ Observed_A1      : num [1:54863] 0.548 0.575 0.548 0.309 0.691 ...
##   ..$ Observed_A2      : num [1:54863] 0.452 0.425 0.452 0.691 0.309 ...
##   ..$ Observed_A1_count: num [1:54863] 23 23 23 13 29 ...
##   ..$ Observed_A2_count: num [1:54863] 19 17 19 29 13 ...
##  $ fam3:'data.frame':    55473 obs. of  10 variables:
##   ..$ CHR              : chr [1:55473] "1.1" "1.1" "1.10" "1.10" ...
##   ..$ SNP              : chr [1:55473] "AX-581444870" "AX-583033387" "AX-583033504" "AX-583035355" ...
##   ..$ A1               : chr [1:55473] "C" "C" "C" "G" ...
##   ..$ A2               : chr [1:55473] "T" "T" "T" "C" ...
##   ..$ MAF              : num [1:55473] 0.182 0.326 0.5 0.391 0.37 ...
##   ..$ NCHROBS          : num [1:55473] 44 46 44 46 46 46 46 46 46 46 ...
##   ..$ Observed_A1      : num [1:55473] 0.818 0.674 0.5 0.609 0.63 ...
##   ..$ Observed_A2      : num [1:55473] 0.182 0.326 0.5 0.391 0.37 ...
##   ..$ Observed_A1_count: num [1:55473] 36 31 22 28 29 ...
##   ..$ Observed_A2_count: num [1:55473] 8 15 22 18 17 ...
##  $ fam4:'data.frame':    42673 obs. of  10 variables:
##   ..$ CHR              : chr [1:42673] "1.1" "1.1" "1.1" "1.1" ...
##   ..$ SNP              : chr [1:42673] "AX-583033342" "AX-583033370" "AX-583033387" "AX-583035211" ...
##   ..$ A1               : chr [1:42673] "C" "G" "C" "C" ...
##   ..$ A2               : chr [1:42673] "G" "A" "T" "T" ...
##   ..$ MAF              : num [1:42673] 0.259 0.759 0.241 0.481 0.463 ...
##   ..$ NCHROBS          : num [1:42673] 54 54 54 54 54 50 54 54 54 54 ...
##   ..$ Observed_A1      : num [1:42673] 0.741 0.241 0.759 0.518 0.537 ...
##   ..$ Observed_A2      : num [1:42673] 0.259 0.759 0.241 0.481 0.463 ...
##   ..$ Observed_A1_count: num [1:42673] 40 13 41 28 29 ...
##   ..$ Observed_A2_count: num [1:42673] 14 41 13 26 25 ...
##  $ fam5:'data.frame':    42707 obs. of  10 variables:
##   ..$ CHR              : chr [1:42707] "1.1" "1.1" "1.1" "1.1" ...
##   ..$ SNP              : chr [1:42707] "AX-583035163" "AX-583033370" "AX-583035194" "AX-583033387" ...
##   ..$ A1               : chr [1:42707] "A" "G" "A" "C" ...
##   ..$ A2               : chr [1:42707] "G" "A" "G" "T" ...
##   ..$ MAF              : num [1:42707] 0.583 0.735 0.528 0.469 0.2 ...
##   ..$ NCHROBS          : num [1:42707] 36 34 36 32 30 36 36 36 34 36 ...
##   ..$ Observed_A1      : num [1:42707] 0.417 0.265 0.472 0.531 0.8 ...
##   ..$ Observed_A2      : num [1:42707] 0.583 0.735 0.528 0.469 0.2 ...
##   ..$ Observed_A1_count: num [1:42707] 15 9 17 17 24 ...
##   ..$ Observed_A2_count: num [1:42707] 21 25 19 15 6 ...
##  $ fam6:'data.frame':    55194 obs. of  10 variables:
##   ..$ CHR              : chr [1:55194] "1.1" "1.1" "1.1" "1.1" ...
##   ..$ SNP              : chr [1:55194] "AX-581444870" "AX-583035083" "AX-583033370" "AX-583035194" ...
##   ..$ A1               : chr [1:55194] "C" "A" "G" "A" ...
##   ..$ A2               : chr [1:55194] "T" "G" "A" "G" ...
##   ..$ MAF              : num [1:55194] 0.0833 0.4167 0.7857 0.2143 0.2857 ...
##   ..$ NCHROBS          : num [1:55194] 12 12 14 14 14 14 14 14 14 14 ...
##   ..$ Observed_A1      : num [1:55194] 0.917 0.583 0.214 0.786 0.714 ...
##   ..$ Observed_A2      : num [1:55194] 0.0833 0.4167 0.7857 0.2143 0.2857 ...
##   ..$ Observed_A1_count: num [1:55194] 11 7 3 11 10 ...
##   ..$ Observed_A2_count: num [1:55194] 1 5 11 3 4 ...
```

### 6.2 Sort by SNP id

```
# Sort family_offspring_2 by SNP column
family_offspring_2 <- lapply(family_offspring_2, function(df) df[order(df$SNP), ])

# Sort family_parents_2 by SNP column
family_parents_2 <- lapply(family_parents_2, function(df) df[order(df$SNP), ])
```

Sanity checks

Check for differences in the SNPs within family, comparing paretal
and offspring SNPs

```
# Create an empty vector to store families with discrepancies
families_with_discrepancies <- c()

# Loop over each family in the parents and offspring list
for (family in names(family_parents_2)) {
  
  # Extract the parent and offspring data for the current family
  parent_data <- family_parents_2[[family]]
  offspring_data <- family_offspring_2[[family]]
  
  # Check if the number of rows (observations) is the same for both parents and offspring
  if (nrow(parent_data) != nrow(offspring_data)) {
    cat(paste("Number of observations differs for family", family, "\n"))
  }
  
  # Check if the SNP ids are the same for both parents and offspring and are in the same order
  if (!identical(parent_data$SNP, offspring_data$SNP)) {
    # Calculate number of discrepancies
    discrepancies <- sum(parent_data$SNP != offspring_data$SNP)
    cat(paste("SNP ids and/or order differ for family", family, "with", discrepancies, "discrepancies. \n"))
    # Append the family name to the vector
    families_with_discrepancies <- c(families_with_discrepancies, family)
  }
}
```

```
## Number of observations differs for family fam1
```

```
## Warning in parent_data$SNP != offspring_data$SNP: longer object length is not a
## multiple of shorter object length
```

```
## SNP ids and/or order differ for family fam1 with 23224 discrepancies. 
## Number of observations differs for family fam2
```

```
## Warning in parent_data$SNP != offspring_data$SNP: longer object length is not a
## multiple of shorter object length
```

```
## SNP ids and/or order differ for family fam2 with 40816 discrepancies. 
## Number of observations differs for family fam3
```

```
## Warning in parent_data$SNP != offspring_data$SNP: longer object length is not a
## multiple of shorter object length
```

```
## SNP ids and/or order differ for family fam3 with 43748 discrepancies. 
## Number of observations differs for family fam4
```

```
## Warning in parent_data$SNP != offspring_data$SNP: longer object length is not a
## multiple of shorter object length
```

```
## SNP ids and/or order differ for family fam4 with 30621 discrepancies. 
## Number of observations differs for family fam5
```

```
## Warning in parent_data$SNP != offspring_data$SNP: longer object length is not a
## multiple of shorter object length
```

```
## SNP ids and/or order differ for family fam5 with 40942 discrepancies. 
## Number of observations differs for family fam6
```

```
## Warning in parent_data$SNP != offspring_data$SNP: longer object length is not a
## multiple of shorter object length
```

```
## SNP ids and/or order differ for family fam6 with 42804 discrepancies.
```

```
# Output families with discrepancies
families_with_discrepancies
```

```
## [1] "fam1" "fam2" "fam3" "fam4" "fam5" "fam6"
```

We can remove the SNPs are are missing between the parents and
offspring. We did remove those SNPs with counts below 5, so we need to
make sure we have the same SNPs in the same order.

```
# Initialize two empty lists to store the resulting data frames
family_parents_3 <- list()
family_offspring_3 <- list()

# Loop over each family
for (family in names(family_parents_2)) {
  
  # Extract the parent and offspring data for the current family
  parent_data <- family_parents_2[[family]]
  offspring_data <- family_offspring_2[[family]]
  
  # Perform an inner join based on SNP column to find common SNPs
  common_data <- merge(parent_data, offspring_data, by = "SNP")
  
  # Find the rows in the parent and offspring data that have the common SNPs
  parent_data_common <- parent_data[parent_data$SNP %in% common_data$SNP, ]
  offspring_data_common <- offspring_data[offspring_data$SNP %in% common_data$SNP, ]
  
  # Sort the data frames by the SNP column
  parent_data_common <- parent_data_common[order(parent_data_common$SNP), ]
  offspring_data_common <- offspring_data_common[order(offspring_data_common$SNP), ]
  
  # Store the resulting data frames in the corresponding lists
  family_parents_3[[family]] <- parent_data_common
  family_offspring_3[[family]] <- offspring_data_common
}
```

Now check again for discrepancies

```
# Create an empty vector to store families with discrepancies
families_with_discrepancies <- c()

# Loop over each family in the parents and offspring list
for (family in names(family_parents_3)) {
  
  # Extract the parent and offspring data for the current family
  parent_data <- family_parents_3[[family]]
  offspring_data <- family_offspring_3[[family]]
  
  # Check if the number of rows (observations) is the same for both parents and offspring
  if (nrow(parent_data) != nrow(offspring_data)) {
    cat(paste("Number of observations differs for family", family, "\n"))
  }
  
  # Check if the SNP ids are the same for both parents and offspring and are in the same order
  if (!identical(parent_data$SNP, offspring_data$SNP)) {
    # Calculate number of discrepancies
    discrepancies <- sum(parent_data$SNP != offspring_data$SNP)
    cat(paste("SNP ids and/or order differ for family", family, "with", discrepancies, "discrepancies. \n"))
    # Append the family name to the vector
    families_with_discrepancies <- c(families_with_discrepancies, family)
  }
}

# Output families with discrepancies
families_with_discrepancies
```

```
## NULL
```

Now all the SNP ids match and are in the same order. We can compare
the counts of one allele to see if it is correct in the new nested
list.

Compare some counts to see if they match before and after data
manipulation

```
# Initialize a list to store the dataframes for each family
df_list <- list()

# Iterate over each family
for (family in names(family_parents_2)) {
  # Get the initial and processed data for the current family
  parent_data_2 <- family_parents_2[[family]]
  parent_data_3 <- family_parents_3[[family]]

  # Extract the relevant columns
  parent_data_2_subset <- parent_data_2[, c("SNP", "Expected_A1_count")]
  parent_data_3_subset <- parent_data_3[, c("SNP", "Expected_A1_count")]

  # Rename the columns for clarity
  colnames(parent_data_2_subset) <- c("SNP_id", "family_parents_2")
  colnames(parent_data_3_subset) <- c("SNP_id", "family_parents_3")

  # Merge the two dataframes on the SNP_id column
  merged_data <- merge(parent_data_2_subset, parent_data_3_subset, by = "SNP_id")

  # Add the merged dataframe to the list
  df_list[[family]] <- merged_data
}

# Combine all the dataframes in the list into a single dataframe
final_table <- do.call("rbind", df_list)

# Check the output
head(final_table)
```

```
##              SNP_id family_parents_2 family_parents_3
## fam1.1 AX-579436243               44               44
## fam1.2 AX-579436308               57               57
## fam1.3 AX-579436521               63               63
## fam1.4 AX-579436647               22               22
## fam1.5 AX-579436698               66               66
## fam1.6 AX-579436709               22               22
```

Everything looks good.

## 7. Power simulation

We can simulate how much power we would have with different
parameters. Summary of the code below: 1. **Define the range of
significance levels, effect sizes, and sample sizes:** - The
vector “significance\_levels” contains different significance levels to
be analyzed. - The vector “effect\_sizes” contains different effect sizes
to be analyzed. - The vector “sample\_sizes” contains different sample
sizes to be analyzed.

2. **Create an empty data frame to store the results:**
   - The data frame “df” is created to store the results of the power
     analysis.
3. **Perform power analysis for each combination of
   parameters:**
   - Two nested “for” loops are used to iterate over each combination of
     significance level and sample size.
   - For each combination, a data frame “df\_comb” is created to store the
     results for that specific combination.
   - The columns “effect\_size”, “significance\_level”, “sample\_size”, and
     “power” are initialized in “df\_comb”.
4. **Perform power analysis for each effect size in the current
   combination:**
   - Another “for” loop is used to iterate over each effect size in the
     current combination.
   - For each effect size, the power analysis is performed using the
     “pwr.chisq.test” function.
   - The resulting power value is stored in the corresponding row of
     “df\_comb”.
5. **Append the data frame for the current combination to the
   overall data frame:**
   - After performing the power analysis for all effect sizes in the
     current combination, “df\_comb” is appended to the overall data frame
     “df” using the “rbind” function.

The code iterates over all combinations of significance levels,
effect sizes, and sample sizes, and calculates the power for each
combination using the “pwr.chisq.test” function. The results are stored
in the data frame “df”, which will contain the effect size, significance
level, sample size, and corresponding power for each combination.

```
# Define the range of significance levels, effect sizes, and sample sizes
significance_levels <- c(0.001, 0.01, 0.05, 0.1)
effect_sizes <- c(0.01, 0.05, 0.5)
sample_sizes <- c(10, 20, 30, 40, 50, 60, 70) # we have 30 to 32 offspring per family.

# Create an empty data frame to store the results
df <- data.frame()

# Perform power analysis for each combination of parameters
for (significance_level in significance_levels) {
  for (sample_size in sample_sizes) {
    # Create a data frame for the current combination of significance level and sample size
    df_comb <- data.frame(
      effect_size = effect_sizes,
      significance_level = rep(significance_level, length(effect_sizes)),
      sample_size = rep(sample_size, length(effect_sizes)),
      power = rep(NA, length(effect_sizes))
    )

    # Perform power analysis for each effect size in the current combination
    for (i in seq_along(effect_sizes)) {
      effect_size <- effect_sizes[i]
      power <-
        pwr.chisq.test(
          w = effect_size,
          N = sample_size,
          df = 1,
          sig.level = significance_level,
          power = NULL
        )$power
      df_comb$power[i] <- power
    }

    # Append the data frame for the current combination to the overall data frame
    df <- rbind(df, df_comb)
  }
}

# Convert head(results) to a tibble
table_result <- as_tibble(df)

# Create a flextable object
flex_table <- flextable(table_result)

# Set the formatting options if needed
# flex_table <- flex_table %>%
#   theme_box()  # Example of adding a border around the table

# Print the flextable
flex_table
```

| effect\_size | significance\_level | sample\_size | power |
| --- | --- | --- | --- |
| 0.01 | 0.001 | 10 | 0.001005852 |
| 0.05 | 0.001 | 10 | 0.001148587 |
| 0.50 | 0.001 | 10 | 0.043690114 |
| 0.01 | 0.001 | 20 | 0.001011711 |
| 0.05 | 0.001 | 20 | 0.001301979 |
| 0.50 | 0.001 | 20 | 0.145836484 |
| 0.01 | 0.001 | 30 | 0.001017578 |
| 0.05 | 0.001 | 30 | 0.001460212 |
| 0.50 | 0.001 | 30 | 0.290503658 |
| 0.01 | 0.001 | 40 | 0.001023453 |
| 0.05 | 0.001 | 40 | 0.001623319 |
| 0.50 | 0.001 | 40 | 0.448975934 |
| 0.01 | 0.001 | 50 | 0.001029335 |
| 0.05 | 0.001 | 50 | 0.001791338 |
| 0.50 | 0.001 | 50 | 0.596774562 |
| 0.01 | 0.001 | 60 | 0.001035225 |
| 0.05 | 0.001 | 60 | 0.001964301 |
| 0.50 | 0.001 | 60 | 0.719870422 |
| 0.01 | 0.001 | 70 | 0.001041122 |
| 0.05 | 0.001 | 70 | 0.002142244 |
| 0.50 | 0.001 | 70 | 0.814010733 |
| 0.01 | 0.010 | 10 | 0.010037257 |
| 0.05 | 0.010 | 10 | 0.010938185 |
| 0.50 | 0.010 | 10 | 0.159959540 |
| 0.01 | 0.010 | 20 | 0.010074537 |
| 0.05 | 0.010 | 20 | 0.011890401 |
| 0.50 | 0.010 | 20 | 0.367018885 |
| 0.01 | 0.010 | 30 | 0.010111839 |
| 0.05 | 0.010 | 30 | 0.012856577 |
| 0.50 | 0.010 | 30 | 0.564655597 |
| 0.01 | 0.010 | 40 | 0.010149164 |
| 0.05 | 0.010 | 40 | 0.013836638 |
| 0.50 | 0.010 | 40 | 0.721212877 |
| 0.01 | 0.010 | 50 | 0.010186511 |
| 0.05 | 0.010 | 50 | 0.014830512 |
| 0.50 | 0.010 | 50 | 0.831398047 |
| 0.01 | 0.010 | 60 | 0.010223881 |
| 0.05 | 0.010 | 60 | 0.015838125 |
| 0.50 | 0.010 | 60 | 0.902710905 |
| 0.01 | 0.010 | 70 | 0.010261273 |
| 0.05 | 0.010 | 70 | 0.016859402 |
| 0.50 | 0.010 | 70 | 0.946024438 |
| 0.01 | 0.050 | 10 | 0.050114558 |
| 0.05 | 0.050 | 10 | 0.052868733 |
| 0.50 | 0.050 | 10 | 0.352608082 |
| 0.01 | 0.050 | 20 | 0.050229133 |
| 0.05 | 0.050 | 20 | 0.055747250 |
| 0.50 | 0.050 | 20 | 0.608779485 |
| 0.01 | 0.050 | 30 | 0.050343723 |
| 0.05 | 0.050 | 30 | 0.058635296 |
| 0.50 | 0.050 | 30 | 0.781907999 |
| 0.01 | 0.050 | 40 | 0.050458329 |
| 0.05 | 0.050 | 40 | 0.061532618 |
| 0.50 | 0.050 | 40 | 0.885379141 |
| 0.01 | 0.050 | 50 | 0.050572952 |
| 0.05 | 0.050 | 50 | 0.064438968 |
| 0.50 | 0.050 | 50 | 0.942437543 |
| 0.01 | 0.050 | 60 | 0.050687590 |
| 0.05 | 0.050 | 60 | 0.067354096 |
| 0.50 | 0.050 | 60 | 0.972127216 |
| 0.01 | 0.050 | 70 | 0.050802244 |
| 0.05 | 0.050 | 70 | 0.070277759 |
| 0.50 | 0.050 | 70 | 0.986903430 |
| 0.01 | 0.100 | 10 | 0.100169639 |
| 0.05 | 0.100 | 10 | 0.104238439 |
| 0.50 | 0.100 | 10 | 0.475226345 |
| 0.01 | 0.100 | 20 | 0.100339269 |
| 0.05 | 0.100 | 20 | 0.108471468 |
| 0.50 | 0.100 | 20 | 0.722863626 |
| 0.01 | 0.100 | 30 | 0.100508892 |
| 0.05 | 0.100 | 30 | 0.112698883 |
| 0.50 | 0.100 | 30 | 0.862975530 |
| 0.01 | 0.100 | 40 | 0.100678505 |
| 0.05 | 0.100 | 40 | 0.116920482 |
| 0.50 | 0.100 | 40 | 0.935420936 |
| 0.01 | 0.100 | 50 | 0.100848111 |
| 0.05 | 0.100 | 50 | 0.121136070 |
| 0.50 | 0.100 | 50 | 0.970666592 |
| 0.01 | 0.100 | 60 | 0.101017708 |
| 0.05 | 0.100 | 60 | 0.125345452 |
| 0.50 | 0.100 | 60 | 0.987064084 |
| 0.01 | 0.100 | 70 | 0.101187296 |
| 0.05 | 0.100 | 70 | 0.129548439 |
| 0.50 | 0.100 | 70 | 0.994432712 |

Create plot

```
# Create a line plot with points for each combination of significance level and sample size
ggplot(
  df,
  aes(
    x = effect_size,
    y = power,
    group = sample_size
  )
) +
  geom_line() +
  geom_point() +
  facet_grid(
    significance_level ~ sample_size,
    scales = "free"
  ) +
  labs(
    x = "Effect Size",
    y = "Power"
  ) +
  ggtitle(
    "Effect Size vs. Power by Significance Level and Sample Size"
  ) +
  my_theme() +
  theme(
    panel.spacing.x = unit(1, "lines")
  ) +
  scale_x_log10(
    breaks = c(0.01, 0.05, 0.5)
  ) +
  ylim(0, 1)
```

```
# save plot
ggsave(
  here(
    "output", "segregation", "albopictus", "power_segregation.pdf"
  ),
  width = 8,
  height = 5,
  units = "in"
)
```

## 8. Estimate power for our data

The power estimation code uses Cohen’s w (effect size measure for
chi-square test), which is calculated based on the difference between
observed and expected frequencies. This does not directly apply the
chi-square test, but uses the calculation based on chi-square
distribution to estimate power.

Here is a summary of what the code below is doing:

1. **Define parameters for power analysis:**
   - The desired effect size is set to 0.01.
   - The chosen significance level is set to 0.05.
   - An empty vector sample\_sizes is created to store the sample
     sizes.
2. **Read the .fam files and calculate sample sizes:**
   - A loop is implemented over the family\_names vector.
   - For each family, the corresponding .fam file is read using the
     read.table function.
   - The number of offspring is determined by counting the rows in the
     fam\_data table.
   - The sample size is appended to the sample\_sizes vector.
3. **Perform power analysis for each family:**
   - Another loop is implemented over the sample\_sizes vector.
   - The current family name and sample size are retrieved.
   - The parental and offspring data for the current family are assigned
     from family\_parents\_2 and family\_offspring\_2 respectively.
   - Common SNPs between parents and offspring for the current family are
     identified using the intersect function.
   - The parental and offspring data frames are subsetted to include only
     the common SNPs.
   - Information about the family name, number of offspring, observed
     genotype frequencies, and expected genotype frequencies is printed.
   - The validity of the observed genotype frequencies and expected
     genotype frequencies is checked.
   - Power analysis is performed using the pwr.chisq.test function to
     calculate the power based on the observed and expected genotype
     frequencies.
   - The calculated power is printed.

```
# For the first 100 SNPs
# Define parameters for power analysis
effect_size <- 0.01  # Desired effect size
significance_level <- 0.05  # Chosen significance level

# Create an empty tibble to store the results
results <- tibble(
  Family = character(),
  SNP = character(),
  Expected_A1_count = numeric(),
  Observed_A1_count = numeric(),
  Power_A1 = numeric(),
  Expected_A2_count = numeric(),
  Observed_A2_count = numeric(),
  Power_A2 = numeric()
)

# Perform power analysis for each family
for (i in 1:length(family_names)) {
  family <- family_names[i]
  
  family_parents <- family_parents_2[[i]]
  family_offspring <- family_offspring_2[[i]]
  
  # Find common SNPs between parents and offspring for the current family
  common_snps <- intersect(family_parents$SNP, family_offspring$SNP)
  
  # Select only the first 100 common SNPs for testing
  common_snps <- head(common_snps, 100) # comment out this line if you want to test all SNPs, it takes a long time

  # Iterate over each common SNP
  for (snp in common_snps) {
    # Subset parents and offspring data frames to include only the current SNP
    family_parents_snp <- subset(family_parents, SNP == snp)
    family_offspring_snp <- subset(family_offspring, SNP == snp)
    
    # Check if the subset resulted in any rows
    if (nrow(family_parents_snp) == 0 || nrow(family_offspring_snp) == 0) {
      next  # Skip to the next SNP
    }
    
    # Retrieve observed genotype frequencies in offspring
    observed_A1 <- family_offspring_snp$Observed_A1_count
    observed_A2 <- family_offspring_snp$Observed_A2_count
    
    # Retrieve expected genotype frequencies in parents
    expected_A1 <- family_parents_snp$Expected_A1_count
    expected_A2 <- family_parents_snp$Expected_A2_count
    
    # Perform power analysis for genotype A1
    power_A1 <- pwr.chisq.test(
      w = sum((observed_A1 - expected_A1)^2 / expected_A1),
      N = family_offspring_snp$NCHROBS[1],
      df = 1,
      sig.level = significance_level,
      power = NULL
    )$power
    
    # Perform power analysis for genotype A2
    power_A2 <- pwr.chisq.test(
      w = sum((observed_A2 - expected_A2)^2 / expected_A2),
      N = family_offspring_snp$NCHROBS[1],
      df = 1,
      sig.level = significance_level,
      power = NULL
    )$power
    
    # Store the results in the tibble
    result <- tibble(
      Family = family,
      SNP = snp,
      Expected_A1_count = sum(expected_A1),
      Observed_A1_count = sum(observed_A1),
      Power_A1 = power_A1,
      Expected_A2_count = sum(expected_A2),
      Observed_A2_count = sum(observed_A2),
      Power_A2 = power_A2
    )
    results <- bind_rows(results, result)
  }
}

# view the results
# head(results)

# Convert head(results) to a tibble
table_result <- as_tibble(head(results))

# Create a flextable object
flex_table <- flextable(table_result)

# Set the formatting options if needed
# flex_table <- flex_table %>%
#   theme_box()  # Example of adding a border around the table

# Print the flextable
flex_table
```

| Family | SNP | Expected\_A1\_count | Observed\_A1\_count | Power\_A1 | Expected\_A2\_count | Observed\_A2\_count | Power\_A2 |
| --- | --- | --- | --- | --- | --- | --- | --- |
| fam1 | AX-579436243 | 44 | 23.0032 | 1.00000000 | 44 | 64.9968 | 1.00000000 |
| fam1 | AX-579436308 | 57 | 58.9988 | 0.09375110 | 19 | 17.0012 | 0.44960797 |
| fam1 | AX-579436521 | 63 | 64.9992 | 0.08954243 | 21 | 19.0008 | 0.41474907 |
| fam1 | AX-579436647 | 22 | 20.9968 | 0.07135068 | 66 | 67.0032 | 0.05234725 |
| fam1 | AX-579436698 | 66 | 67.0032 | 0.05234725 | 22 | 20.9968 | 0.07135068 |
| fam1 | AX-579436709 | 22 | 23.0032 | 0.07135068 | 66 | 64.9968 | 0.05234725 |

We observe that the power is different from family to family, and
from SNP to SNP. The mean power is 0.5952 and the min is 0.01.

```
summary(results)
```

```
##     Family              SNP            Expected_A1_count Observed_A1_count
##  Length:600         Length:600         Min.   : 3.0      Min.   : 0.00    
##  Class :character   Class :character   1st Qu.:11.5      1st Qu.:13.00    
##  Mode  :character   Mode  :character   Median :23.0      Median :22.00    
##                                        Mean   :25.0      Mean   :25.48    
##                                        3rd Qu.:34.5      3rd Qu.:33.00    
##                                        Max.   :66.0      Max.   :88.00    
##     Power_A1       Expected_A2_count Observed_A2_count    Power_A2      
##  Min.   :0.05000   Min.   : 3.00     Min.   : 0.0      Min.   :0.05000  
##  1st Qu.:0.05091   1st Qu.:10.50     1st Qu.:11.0      1st Qu.:0.05216  
##  Median :0.17843   Median :19.25     Median :17.5      Median :0.28395  
##  Mean   :0.43607   Mean   :20.78     Mean   :20.3      Mean   :0.46218  
##  3rd Qu.:0.99782   3rd Qu.:27.00     3rd Qu.:27.0      3rd Qu.:1.00000  
##  Max.   :1.00000   Max.   :66.00     Max.   :71.0      Max.   :1.00000
```

Overall, the power analysis suggests that for all three families, the
sample sizes and observed genotype frequencies provide sufficient
statistical power to detect an effect of the desired size (effect size =
0.01) at a significance level of 0.05.

## 9. Number of SNPs genotyped in the parents that could be tested

Each family is a cross of different populations. They have different
sets of SNPs genotyped as heterozygous in one parent.

### 9.1 Venn diagram

We can make a Venn diagram to represent the shared and unique SNPs
across all families.

```
# List all families
families <- c("fam1", "fam2", "fam3", "fam4", "fam5", "fam6")

# Initialize a list to store the data
data_list <- list()

# Loop through each family
for (fam in families) {
  # Read in the file as a vector
  family_segregating_SNPs_R <-
    read_table(
      paste0(
        "output/segregation/albopictus/parents_family_",
        fam,
        "_segregating_SNPs_R.txt"
      ),
      col_names = FALSE
    )[[1]]
  
  # Add the data to the list
  data_list[[fam]] <- family_segregating_SNPs_R
}

# Define a vector of color-blind friendly colors
colors <-
  c("#E69F00",
    "#56B4E9",
    "#009E73",
    "#F0E442",
    "#000000",
    "#CC79A7")

# Save combined Venn diagram to PDF
output_path <-
  here("output",
       "segregation",
       "albopictus",
       "segregating_SNPs_parents_6_venn.pdf")

# Create a PDF device
pdf(output_path, width = 5, height = 5)
par(mar = c(5, 5, 7, 2) + 0.1)  # Adjust margins (bottom, left, top, right)

# Create the 6-way Venn diagram with filled sets
venn(data_list,
     zcolor = colors,
     opacity = 0.4,
     box = FALSE,
     ellipse = FALSE,
     lty = "blank")

# Adjust the title position and size
title(
  main = "6-way Venn Diagram of Segregating SNPs",
      line = -1,
      cex.main = 0.8
  )

# Close the PDF device
dev.off()
```

```
## quartz_off_screen 
##                 2
```

```
# show the plot here
venn(data_list,
     zcolor = colors,
     opacity = 0.4,
     box = FALSE,
     ellipse = FALSE,
     lty = "blank")
```

### 9.2 Parental loci with at least one parental genotype heterozygous

```
# Calculate shared SNPs among all families
shared_snps <- Reduce(intersect, lapply(family_parents_3, function(df) df$SNP))

# Calculate the number of shared SNPs
n_shared_SNPs <- length(shared_snps)

# Calculate the number of unique SNPs for each family
n_unique_SNPs <- sapply(family_names, function(family) {
  unique_SNPs <- family_parents_3[[family]]$SNP
  for (other_family in family_names[family_names != family]) {
    unique_SNPs <- setdiff(unique_SNPs, family_parents_3[[other_family]]$SNP)
  }
  length(unique_SNPs)
})

# Calculate the total number of SNPs for each family
n_total_SNPs <- sapply(family_parents_3, function(df) length(unique(df$SNP)))

# Calculate the percentage of shared SNPs for each family
p_shared_SNPs <- round(n_shared_SNPs / n_total_SNPs * 100, 2)

# Calculate the percentage of unique SNPs for each family
p_unique_SNPs <- round(n_unique_SNPs / n_total_SNPs * 100, 2)

# Create a data frame with the data
table_data <- data.frame(
  Family = family_names,
  n_total_SNPs = n_total_SNPs,
  n_shared_SNPs = n_shared_SNPs,
  p_shared_SNPs = p_shared_SNPs,
  n_unique_SNPs = n_unique_SNPs,
  p_unique_SNPs = p_unique_SNPs
)

# Create a flextable object
flex_table <- flextable::flextable(table_data)

# Set the table caption
table_caption <- "Table 1. Parental loci with at least one parental genotype heterozygous"
flex_table <- flextable::set_caption(flex_table, caption = table_caption)

# Print the table
flex_table
```

Table 1. Parental loci with at least one parental genotype heterozygous

| Family | n\_total\_SNPs | n\_shared\_SNPs | p\_shared\_SNPs | n\_unique\_SNPs | p\_unique\_SNPs |
| --- | --- | --- | --- | --- | --- |
| fam1 | 42,115 | 5,249 | 12.46 | 2,280 | 5.41 |
| fam2 | 54,863 | 5,249 | 9.57 | 4,526 | 8.25 |
| fam3 | 55,473 | 5,249 | 9.46 | 4,975 | 8.97 |
| fam4 | 42,673 | 5,249 | 12.30 | 1,840 | 4.31 |
| fam5 | 42,707 | 5,249 | 12.29 | 2,097 | 4.91 |
| fam6 | 55,194 | 5,249 | 9.51 | 3,495 | 6.33 |

```
# Save the Word document
flextable::save_as_docx(
  flex_table,
  path = here(
    "output",
    "segregation",
    "albopictus",
    "segregating_SNPs_table2.docx"
  )
)
```

Pairwise comparison of parental genotypes

```
# Create an empty matrix to store the counts
shared_snps_matrix <- matrix(0, nrow = length(family_names), ncol = length(family_names), dimnames = list(family_names, family_names))

# Calculate the shared SNPs for each pairwise comparison
for (i in 1:length(family_names)) {
  for (j in 1:length(family_names)) {
    if (i != j) {
      shared_snps <- intersect(family_parents_3[[i]]$SNP, family_parents_2[[j]]$SNP)
      shared_snps_matrix[i, j] <- length(shared_snps)
    }
  }
}

# Convert the shared SNP matrix to a data frame
shared_snps_df <- as.data.frame(shared_snps_matrix)

# Create a flextable object for the shared SNP matrix
flex_table_shared_snps_matrix <- flextable::flextable(shared_snps_df)

# Set the table caption
table_caption_shared_snps_matrix <- "Table 3. Number of SNPs shared among families"
flex_table_shared_snps_matrix <- flextable::set_caption(flex_table_shared_snps_matrix, caption = table_caption_shared_snps_matrix)

flex_table_shared_snps_matrix
```

Table 3. Number of SNPs shared among families

| fam1 | fam2 | fam3 | fam4 | fam5 | fam6 |
| --- | --- | --- | --- | --- | --- |
| 0 | 20,656 | 21,022 | 27,897 | 27,404 | 20,882 |
| 20,656 | 0 | 33,581 | 20,982 | 20,929 | 36,359 |
| 21,022 | 33,578 | 0 | 21,325 | 21,263 | 35,883 |
| 27,896 | 20,978 | 21,320 | 0 | 29,676 | 21,244 |
| 27,403 | 20,924 | 21,262 | 29,676 | 0 | 21,104 |
| 20,875 | 36,358 | 35,881 | 21,242 | 21,102 | 0 |

```
# Save the Word document
flextable::save_as_docx(
  flex_table_shared_snps_matrix,
  path = here(
    "output",
    "segregation",
    "albopictus",
    "shared_SNPs_matrix.docx"
  )
)
```

We can check how many times we can test each SNP. For example, the
SNP was genotyped in both parents and is heterozygous in one or both
parents. I removed SNPs that were homozygous in both parents.

```
# Combine SNP IDs from all families into a single vector
all_snps <- unlist(lapply(family_parents_3, function(df) df$SNP))

# Count the occurrences of each SNP in all six families
snp_counts <- table(all_snps)

# Calculate the number of times each SNP is seen in 1, 2, 3, 4, 5, or 6 families
unique_counts <- table(snp_counts)

# Create a data frame with the counts
shared_snps_data <- data.frame(
  "Times_seen" = 1:length(unique_counts),
  "n_SNPs" = as.vector(unique_counts)
)

# Create a flextable object
flex_table_shared_snps <- flextable::flextable(shared_snps_data)

# Set the table caption
table_caption_shared_snps <- "Table 2. Count of SNPs seen in different numbers of families"
flex_table_shared_snps <- flextable::set_caption(flex_table_shared_snps, caption = table_caption_shared_snps)

# Print the table
flex_table_shared_snps
```

Table 2. Count of SNPs seen in different numbers of families

| Times\_seen | n\_SNPs |
| --- | --- |
| 1 | 19,213 |
| 2 | 25,124 |
| 3 | 25,922 |
| 4 | 15,036 |
| 5 | 10,832 |
| 6 | 5,249 |

```
# Save the Word document
flextable::save_as_docx(
  flex_table_shared_snps,
  path = here(
    "output",
    "segregation",
    "albopictus",
    "shared_SNPs_table.docx"
  )
)
```

Out of the more than 100k SNPs, 19,213 can be tested only in one
family but the others can be tested in more than one family.

We can save the data as RDS format to load it later if necessary. To
save the data

```
# Save the data
saveRDS(
  family_parents_3,
  file = here(
    "output", "segregation", "albopictus", "family_parents_2.rds"
  )
)
saveRDS(
  family_offspring_3,
  file = here(
    "output", "segregation", "albopictus", "family_offspring_2.rds"
  )
)
```

To load the data

```
# Load the data later
family_parents_3 <-
  readRDS(
    file = here(
      "output", "segregation", "albopictus", "family_parents_2.rds"
    )
  )
family_offspring_3 <-
  readRDS(
    file = here(
      "output", "segregation", "albopictus", "family_offspring_2.rds"
    )
  )
```

## 10. Chi square test with Aedes albopictus data

Do a chi square test for each family, obtain p values, store them in
a tibble, use Fisher test to combine the p values across the families.
However, the number of SNPs in each family is different. So, if a SNP is
in only one family, it is okay to have only one p values. Let’s do the
chi square and the fisher test to combine the p values.

### 10.1 Remove SNPs with zeros or counts < 5

We can remove SNPs with zero and <5 counts if you want. Check
below how many SNPs we would remove. I did not remove it for the test. I
just wanted to know how many SNPs we would remove.

```
# Creating new data sets excluding rows with counts less than 5 in parents and offspring
family_parents_5 <-
  lapply(
    family_parents_3, function(df) {
      df[!(df$Expected_A1_count < 5 | df$Expected_A2_count < 5), ]
    }
  )

family_offspring_5 <-
  lapply(
    family_offspring_3, function(df) {
      df[!(df$Observed_A1_count < 5 | df$Observed_A2_count < 5), ]
    }
  )
```

### 10.2 Sanity checks after data tyding

Now check again for discrepancies, we will use the data set without
counts smaller than 5

```
# Create an empty vector to store families with discrepancies
families_with_discrepancies <- c()

# Loop over each family in the parents and offspring list
for (family in names(family_parents_5)) {
  
  # Extract the parent and offspring data for the current family
  parent_data <- family_parents_5[[family]]
  offspring_data <- family_offspring_5[[family]]
  
  # Check if the number of rows (observations) is the same for both parents and offspring
  if (nrow(parent_data) != nrow(offspring_data)) {
    cat(paste("Number of observations differs for family", family, "\n"))
  }
  
  # Check if the SNP ids are the same for both parents and offspring and are in the same order
  if (!identical(parent_data$SNP, offspring_data$SNP)) {
    # Calculate number of discrepancies
    discrepancies <- sum(parent_data$SNP != offspring_data$SNP)
    cat(paste("SNP ids and/or order differ for family", family, "with", discrepancies, "discrepancies. \n"))
    # Append the family name to the vector
    families_with_discrepancies <- c(families_with_discrepancies, family)
  }
}
```

```
## Number of observations differs for family fam1
```

```
## Warning in parent_data$SNP != offspring_data$SNP: longer object length is not a
## multiple of shorter object length
```

```
## SNP ids and/or order differ for family fam1 with 42056 discrepancies. 
## Number of observations differs for family fam2
```

```
## Warning in parent_data$SNP != offspring_data$SNP: longer object length is not a
## multiple of shorter object length
```

```
## SNP ids and/or order differ for family fam2 with 54721 discrepancies. 
## Number of observations differs for family fam3
```

```
## Warning in parent_data$SNP != offspring_data$SNP: longer object length is not a
## multiple of shorter object length
```

```
## SNP ids and/or order differ for family fam3 with 55348 discrepancies. 
## Number of observations differs for family fam4
```

```
## Warning in parent_data$SNP != offspring_data$SNP: longer object length is not a
## multiple of shorter object length
```

```
## SNP ids and/or order differ for family fam4 with 42628 discrepancies. 
## Number of observations differs for family fam5
```

```
## Warning in parent_data$SNP != offspring_data$SNP: longer object length is not a
## multiple of shorter object length
```

```
## SNP ids and/or order differ for family fam5 with 42570 discrepancies. 
## Number of observations differs for family fam6
```

```
## Warning in parent_data$SNP != offspring_data$SNP: longer object length is not a
## multiple of shorter object length
```

```
## SNP ids and/or order differ for family fam6 with 20461 discrepancies.
```

```
# Output families with discrepancies
families_with_discrepancies
```

```
## [1] "fam1" "fam2" "fam3" "fam4" "fam5" "fam6"
```

Because we remove the SNPs with counts below 5, the length and the
SNPs between parental and offspring data sets is different. We can fix
it with the code we used before.

```
# Initialize two empty lists to store the resulting data frames
family_parents_6 <- list()
family_offspring_6 <- list()

# Loop over each family
for (family in names(family_parents_5)) {
  
  # Extract the parent and offspring data for the current family
  parent_data <- family_parents_5[[family]]
  offspring_data <- family_offspring_5[[family]]
  
  # Perform an inner join based on SNP column to find common SNPs
  common_data <- merge(parent_data, offspring_data, by = "SNP")
  
  # Find the rows in the parent and offspring data that have the common SNPs
  parent_data_common <- parent_data[parent_data$SNP %in% common_data$SNP, ]
  offspring_data_common <- offspring_data[offspring_data$SNP %in% common_data$SNP, ]
  
  # Sort the data frames by the SNP column
  parent_data_common <- parent_data_common[order(parent_data_common$SNP), ]
  offspring_data_common <- offspring_data_common[order(offspring_data_common$SNP), ]
  
  # Store the resulting data frames in the corresponding lists
  family_parents_6[[family]] <- parent_data_common
  family_offspring_6[[family]] <- offspring_data_common
}
```

Now check again for discrepancies again

```
# Create an empty vector to store families with discrepancies
families_with_discrepancies <- c()

# Loop over each family in the parents and offspring list
for (family in names(family_parents_6)) {
  
  # Extract the parent and offspring data for the current family
  parent_data <- family_parents_6[[family]]
  offspring_data <- family_offspring_6[[family]]
  
  # Check if the number of rows (observations) is the same for both parents and offspring
  if (nrow(parent_data) != nrow(offspring_data)) {
    cat(paste("Number of observations differs for family", family, "\n"))
  }
  
  # Check if the SNP ids are the same for both parents and offspring and are in the same order
  if (!identical(parent_data$SNP, offspring_data$SNP)) {
    # Calculate number of discrepancies
    discrepancies <- sum(parent_data$SNP != offspring_data$SNP)
    cat(paste("SNP ids and/or order differ for family", family, "with", discrepancies, "discrepancies. \n"))
    # Append the family name to the vector
    families_with_discrepancies <- c(families_with_discrepancies, family)
  }
}

# Output families with discrepancies
families_with_discrepancies
```

```
## NULL
```

We can compare how many SNPs we are removing from data sets once we
remove the SNPs with counts below 5. Since we have the same SNPs in
parents and offspring, we can check only one of the data sets before and
after removing the SNPs with low allele counts.

```
# Calculate the number of removed SNPs for each family
removed_snps <- 
  sapply(seq_along(family_parents_5), function(i) {
  nrow(family_parents_5[[i]]) - nrow(family_parents_6[[i]])
})

# Calculate total unique SNPs before and after filtering
unique_snps_before <-
  unique(unlist(lapply(family_parents_5, `[[`, "SNP")))
unique_snps_after <-
  unique(unlist(lapply(family_parents_6, `[[`, "SNP")))

# Calculate number of SNPs before and after for each family
snps_before <- 
  sapply(family_parents_5, function(df)
  nrow(df))
snps_after <- 
  sapply(family_parents_6, function(df)
  nrow(df))

# Calculate percentage of SNPs removed for each family and across all families
percentage_removed <-
  removed_snps / snps_before * 100
percentage_removed_all <-
  length(setdiff(unique_snps_before, unique_snps_after)) / length(unique_snps_before) * 100

# Format percentage values with two decimal places
percentage_removed <- 
  sprintf("%.2f", percentage_removed)
percentage_removed_all <-
  sprintf("%.2f", percentage_removed_all)

# Create a tibble with the results
results <- 
  tibble(
  Family = as.character(1:length(removed_snps)),
  SNPs_Before = snps_before,
  SNPs_After = snps_after,
  SNPs_Removed = removed_snps,
  Percentage_Removed = percentage_removed
)

# Calculate the total number of unique SNPs removed across all families
total_snps_removed <-
  length(setdiff(unique_snps_before, unique_snps_after))

# Add total row to the tibble
total_row <- 
  tibble(
  Family = "Total",
  SNPs_Before = length(unique_snps_before),
  SNPs_After = length(unique_snps_after),
  SNPs_Removed = total_snps_removed,
  Percentage_Removed = percentage_removed_all
)
# Bind objects
results <- 
  bind_rows(results, total_row)

# Set theme
set_flextable_defaults(
  font.family = "Arial",
  font.size = 9,
  big.mark = ",",
  theme_fun = "theme_zebra" # try the themes: theme_alafoli(), theme_apa(), theme_booktabs(), theme_box(), theme_tron_legacy(), theme_tron(), theme_vader(), theme_vanilla(), theme_zebra()
)

# Create the flextable object to save as .docx
flex_table <- 
  flextable(results) |>
  set_caption(caption = as_paragraph(
    as_chunk(
      "Table 5. Number of SNPs before and after removing SNPs with allele count below 5.",
      props = fp_text_default(color = "#000000", font.size = 12)
    )
  ),
  fp_p = fp_par(text.align = "center", padding = 5))

# Print the table Markdown
flex_table
```

Table 5. Number of SNPs before and after removing SNPs with allele count below 5.

| Family | SNPs\_Before | SNPs\_After | SNPs\_Removed | Percentage\_Removed |
| --- | --- | --- | --- | --- |
| 1 | 42,108 | 41,434 | 674 | 1.60 |
| 2 | 54,811 | 53,407 | 1,404 | 2.56 |
| 3 | 55,441 | 54,099 | 1,342 | 2.42 |
| 4 | 42,634 | 41,366 | 1,268 | 2.97 |
| 5 | 42,571 | 40,425 | 2,146 | 5.04 |
| 6 | 20,469 | 12,373 | 8,096 | 39.55 |
| Total | 98,654 | 96,378 | 2,276 | 2.31 |

```
# Save the Word document
flextable::save_as_docx(flex_table,
                        path = here(
                          "output",
                          "segregation",
                          "albopictus",
                          "filtering_results.docx"
                        ))
```

We remove a total of 2,276 from our testing because they had counts
below 5. It means we will not test their segregation because their
counts were below 5. We see that fam6 which had only 6 offspring had
around 40% of the SNPs with counts below 5. There was a problem with the
DNA yield of the fam6 and we did not have too many individuals to
genotype. However, most of SNPs from fam6 are tested in other families.
So, we lose some power and some SNPs, but there is not much we can do.
Perhaps keep the low counts but we know it will be breaking one
assumption of the chi square test.

Now we can do our test. Since we have thousands of SNPs, lets subset
to create a small data set to test the code for the statistical
analyses

```
# Subset the data to get the first 100 SNPs
family_parents_6_subset_no_zeros <- lapply(family_parents_6, function(df) df[1:100, ])
family_offspring_6_subset_no_zeros <- lapply(family_offspring_6, function(df) df[1:100, ])

# Verify the result data
# str(family_parents_6_subset_no_zeros)
# str(family_offspring_6_subset_no_zeros)
```

We can save the data as RDS format to load it later if necessary. To
save the data

```
# Save the data
saveRDS(
  family_parents_6_subset_no_zeros,
  file = here(
    "output", "segregation", "albopictus", "family_parents_6_subset_no_zeros.rds"
  )
)
saveRDS(
  family_offspring_6_subset_no_zeros,
  file = here(
    "output", "segregation", "albopictus", "family_offspring_6_subset_no_zeros.rds"
  )
)
```

We can also subset the original data and keep the counts below 5.

```
# Subset the data to get the first 100 SNPs
family_parents_6_subset_with_zeros <- lapply(family_parents_3, function(df) df[1:100, ])
family_offspring_6_subset_with_zeros <- lapply(family_offspring_3, function(df) df[1:100, ])

# Verify the subsetted data
# str(family_parents_6_subset_with_zeros)
# str(family_offspring_6_subset_with_zeros)
```

Check for discrepancies

```
# Create an empty vector to store families with discrepancies
families_with_discrepancies <- c()

# Loop over each family in the parents and offspring list
for (family in names(family_parents_6_subset_with_zeros)) {
  
  # Extract the parent and offspring data for the current family
  parent_data <- family_parents_6_subset_with_zeros[[family]]
  offspring_data <- family_offspring_6_subset_with_zeros[[family]]
  
  # Check if the number of rows (observations) is the same for both parents and offspring
  if (nrow(parent_data) != nrow(offspring_data)) {
    cat(paste("Number of observations differs for family", family, "\n"))
  }
  
  # Check if the SNP ids are the same for both parents and offspring and are in the same order
  if (!identical(parent_data$SNP, offspring_data$SNP)) {
    # Calculate number of discrepancies
    discrepancies <- sum(parent_data$SNP != offspring_data$SNP)
    cat(paste("SNP ids and/or order differ for family", family, "with", discrepancies, "discrepancies. \n"))
    # Append the family name to the vector
    families_with_discrepancies <- c(families_with_discrepancies, family)
  }
}

# Output families with discrepancies
families_with_discrepancies
```

```
## NULL
```

```
# Save the data
saveRDS(
  family_parents_6_subset_with_zeros,
  file = here(
    "output", "segregation", "albopictus", "family_parents_6_subset_with_zeros.rds"
  )
)
saveRDS(
  family_offspring_6_subset_with_zeros,
  file = here(
    "output", "segregation", "albopictus", "family_offspring_6_subset_with_zeros.rds"
  )
)
```

Clean environment and memory

```
rm(list = ls())
gc()
```

```
##            used  (Mb) gc trigger  (Mb) limit (Mb) max used  (Mb)
## Ncells 10618106 567.1   18380167 981.7         NA 13917246 743.3
## Vcells 19681551 150.2   68876258 525.5      32768 66644959 508.5
```

To load the data

```
# Load the data with no counts below 5
family_parents_6_subset_no_zeros <-
  readRDS(
    file = here(
      "output", "segregation", "albopictus", "family_parents_6_subset_no_zeros.rds"
    )
  )
family_offspring_6_subset_no_zeros <-
  readRDS(
    file = here(
      "output", "segregation", "albopictus", "family_offspring_6_subset_no_zeros.rds"
    )
  )

# Load the data with counts below 5 and zeros
family_parents_6_subset_with_zeros <-
  readRDS(
    file = here(
      "output", "segregation", "albopictus", "family_parents_6_subset_with_zeros.rds"
    )
  )
family_offspring_6_subset_with_zeros <-
  readRDS(
    file = here(
      "output", "segregation", "albopictus", "family_offspring_6_subset_with_zeros.rds"
    )
  )
```

### 10.3 Segregation test

We will use the data without filtering.

**Code Explanation:**

1. **Data Frame Initialization:** Initializes an empty
   data frame named “combined\_df” with specific column names and data
   types.
2. **Unique SNP Identification:** Identifies all unique
   Single Nucleotide Polymorphisms (SNPs) across all families.
3. **SNP Analysis Loop:** Begins a loop to perform
   statistical analysis for each SNP.
4. **Family Iteration:** Inside this loop, another loop
   is created to go through each family.
5. **SNP Presence Check:** For each family, the code
   checks if the current SNP is present in both parents and
   offspring.
6. **Statistical Tests:** Conducts Fisher’s exact test
   or Chi-square test depending on the observed and expected counts for the
   SNP alleles.
7. **P-Value Storage:** Stores the p-value from the
   statistical test.
8. **Data Frame Update:** Adds the details to the
   “combined\_df” data frame for each family.
9. **Data Frame Transformation:** Uses “pivot\_wider” to
   transform the data frame from a long format to a wide format, creating
   separate columns for each family and renaming them.
10. **Rows Merging:** Merges rows with same SNP, while
    preserving unique values in each family column, and places the count of
    families and combined p-value in the final data frame.
11. **Output Order Setting:** Sets the order of the
    columns in the output data frame.
12. **P-Value Combination:** Combines the p-values for
    each SNP across all families using a chi-square based method.
13. **Adjusted P-Values Calculation:** Calculates
    adjusted p-values using the Holm-Bonferroni method.
14. **P-Values Formatting:** Applies a custom function
    to format the p-values in a certain manner.
15. **Count Columns Rounding:** Rounds count columns to
    2 decimal places.
16. **P-Values Conversion:** Converts the p-values to
    numeric and then applies custom formatting to these columns.
17. **Family Count Update:** Updates the count of
    families for each SNP.
18. **Results Conversion to Tibble:** Converts the final
    results data frame to a tibble for displaying.
19. **Flextable Creation:** Creates a flextable object
    to display the head of the results in a stylized format.

```
# Initialize the combined data frame
combined_df <-
  data.frame(
    Family = character(),
    SNP = character(),
    Expected_A1_count = numeric(),
    Expected_A2_count = numeric(),
    Observed_A1_count = numeric(),
    Observed_A2_count = numeric(),
    P_Value = numeric(),
    Combined_P_Value = numeric(),
    Family_Count = integer(),
    stringsAsFactors = FALSE
  )

# Get all unique SNPs across all families - use _with_zeros or no_zeros to see how many SNPs
all_snps <-
  unique(
    unlist(
      lapply(
        family_parents_6_subset_with_zeros, function(x) {
          x$SNP
        }
      )
    )
  )

# Perform the analysis for each SNP
for (snp in all_snps) {
  p_values <- numeric()
  family_count <- 0
  families <- c()

  # Go through each family
  for (i in 1:length(family_parents_6_subset_with_zeros)) {
    family_parents <- family_parents_6_subset_with_zeros[[i]]
    family_offspring <- family_offspring_6_subset_with_zeros[[i]]

    # Check if the SNP is in the family
    if (snp %in% family_parents$SNP &&
      snp %in% family_offspring$SNP) {
      family_count <- family_count + 1
      families <- c(families, names(family_parents_6_subset_with_zeros)[i])

      # Conduct the chi-square test
      observed_counts <-
        c(
          family_offspring[family_offspring$SNP == snp, "Observed_A1_count"],
          family_offspring[family_offspring$SNP == snp, "Observed_A2_count"]
        )
      expected_counts <-
        c(
          family_parents[family_parents$SNP == snp, "Expected_A1_count"],
          family_parents[family_parents$SNP == snp, "Expected_A2_count"]
        )
      if (min(observed_counts, expected_counts) < 5) {
        # If either of the counts is less than 5, use Fisher's Exact Test
        test_result <- fisher.test(matrix(c(observed_counts, expected_counts), nrow = 2))$p.value
      } else {
        # Otherwise use Chi-Square Test
        test_result <- chisq.test(observed_counts, p = expected_counts / sum(expected_counts))$p.value
      }

      # Store the p-value
      p_values <- c(p_values, test_result)

      # Add the details to the data frame for each family
      combined_df <- rbind(
        combined_df,
        data.frame(
          Family = names(family_parents_6_subset_with_zeros)[i],
          SNP = snp,
          Expected_A1_count = family_parents[family_parents$SNP == snp, "Expected_A1_count"],
          Observed_A1_count = family_offspring[family_offspring$SNP == snp, "Observed_A1_count"],
          Expected_A2_count = family_parents[family_parents$SNP == snp, "Expected_A2_count"],
          Observed_A2_count = family_offspring[family_offspring$SNP == snp, "Observed_A2_count"],
          P_Value = test_result,
          Family_Count = family_count,
          # Assign the Family_Count here
          Combined_P_Value = NA,
          # As we haven't calculated combined p-value yet
          stringsAsFactors = FALSE
        )
      )
    }
  }

  # Skip if there's no SNP found in any family
  if (family_count == 0) {
    next
  }
}

new_df <- combined_df|>
  pivot_wider(names_from = Family, values_from = c(Expected_A1_count, Observed_A1_count, Expected_A2_count, Observed_A2_count, P_Value)) |>
  rename_with(~ gsub("(\\d+)_(Expected|Observed)", paste0("fam\\1_", "\\2"), .), starts_with("Expected")) |>
  rename_with(~ gsub("(\\d+)_(Expected|Observed)", paste0("fam\\1_", "\\2"), .), starts_with("Observed"))

# Merge rows
merged_df <-
  new_df |>
  group_by(SNP) %>%
  summarise(
    across(
      starts_with("Expected_A1_count_fam"),
      ~ ifelse(all(is.na(.)), NA, unique(.[!is.na(.)])[1])
    ),
    across(
      starts_with("Observed_A1_count_fam"),
      ~ ifelse(all(is.na(.)), NA, unique(.[!is.na(.)])[1])
    ),
    across(
      starts_with("Expected_A2_count_fam"),
      ~ ifelse(all(is.na(.)), NA, unique(.[!is.na(.)])[1])
    ),
    across(
      starts_with("Observed_A2_count_fam"),
      ~ ifelse(all(is.na(.)), NA, unique(.[!is.na(.)])[1])
    ),
    across(
      starts_with("P_Value_fam"),
      ~ ifelse(all(is.na(.)), NA, unique(.[!is.na(.)])[1])
    ),
    Family_Count = first(Family_Count),
    Combined_P_Value = first(Combined_P_Value)
  ) %>%
  ungroup() %>%
  dplyr::select(
    SNP,
    starts_with("Expected_A1_count_fam"),
    starts_with("Expected_A2_count_fam"),
    starts_with("Observed_A1_count_fam"),
    starts_with("Observed_A2_count_fam"),
    starts_with("P_Value_fam"),
    Family_Count,
    Combined_P_Value
  )

# set ouput order
merged_df <-
  merged_df |>
  dplyr::select(SNP,
         starts_with("Expected_A1_count_fam1"),
         starts_with("Observed_A1_count_fam1"),
         starts_with("Expected_A2_count_fam1"),
         starts_with("Observed_A2_count_fam1"),
         starts_with("P_Value_fam1"),
         starts_with("Expected_A1_count_fam2"),
         starts_with("Observed_A1_count_fam2"),
         starts_with("Expected_A2_count_fam2"),
         starts_with("Observed_A2_count_fam2"),
         starts_with("P_Value_fam2"),
         starts_with("Expected_A1_count_fam3"),
         starts_with("Observed_A1_count_fam3"),
         starts_with("Expected_A2_count_fam3"),
         starts_with("Observed_A2_count_fam3"),
         starts_with("P_Value_fam3"),
         starts_with("Expected_A1_count_fam4"),
         starts_with("Observed_A1_count_fam4"),
         starts_with("Expected_A2_count_fam4"),
         starts_with("Observed_A2_count_fam4"),
         starts_with("P_Value_fam4"),
         starts_with("Expected_A1_count_fam5"),
         starts_with("Observed_A1_count_fam5"),
         starts_with("Expected_A2_count_fam5"),
         starts_with("Observed_A2_count_fam5"),
         starts_with("P_Value_fam5"),
         starts_with("Expected_A1_count_fam6"),
         starts_with("Observed_A1_count_fam6"),
         starts_with("Expected_A2_count_fam6"),
         starts_with("Observed_A2_count_fam6"),
         starts_with("P_Value_fam6"),
         Family_Count,
         Combined_P_Value)

#
# Combine the p values 


# Option 1: using fisher.test()

# Create new columns for Combined_P_Value and Adjusted_P_Value
merged_df$Combined_P_Value <- NA
merged_df$Adjusted_P_Value <- NA

# Iterate over each row
# for (i in 1:nrow(merged_df)) {
#   p_values <- numeric()
# 
#   # Check P-values in each family column
#   for (j in 1:6) {
#     col_name <- paste0("P_Value_fam", j)
#     if (!is.na(merged_df[i, col_name])) {
#       p_value <- as.numeric(merged_df[i, col_name])
#       if (!is.na(p_value)) {
#         p_values <- c(p_values, p_value)
#       }
#     }
#   }
# 
#   # Combine P-values if more than one is present - using fisher.test
#   if (length(p_values) > 1) {
#     contingency_table <- matrix(c(sum(1 - p_values), sum(p_values), sum(p_values), sum(1 - p_values)), ncol = 2)
#     combined_p_value <- fisher.test(contingency_table)$p.value
#     merged_df[i, "Combined_P_Value"] <- combined_p_value
#   } else if (length(p_values) == 1) {
#     merged_df[i, "Combined_P_Value"] <- p_values
#   }
# }
#
# Option 2: using chi-square
# Iterate over each row
for (i in 1:nrow(merged_df)) {
  p_values <- numeric()

  # Check P-values in each family column
  for (j in 1:6) {
    col_name <- paste0("P_Value_fam", j)
    if (!is.na(merged_df[i, col_name])) {
      p_value <- as.numeric(merged_df[i, col_name])
      if (!is.na(p_value)) {
        p_values <- c(p_values, p_value)
      }
    }
  }

  # Combine P-values if more than one is present
  if (length(p_values) > 1) {
    combined_p_value <- pchisq(
      -2 * sum(log(p_values)),
      df = 2 * length(p_values),
      lower.tail = FALSE
    )
    merged_df[i, "Combined_P_Value"] <- combined_p_value
  } else if (length(p_values) == 1) {
    merged_df[i, "Combined_P_Value"] <- p_values
  }
}

# Calculate Adjusted P-values
adjusted_p_values <- p.adjust(merged_df$Combined_P_Value, method = "holm")
merged_df$Adjusted_P_Value <- adjusted_p_values


# Custom formatting function for p-values
format_pvalues <- function(x) {
  ifelse(x < 0.001, formatC(x, format = "e", digits = 2),
         ifelse(x >= 0.001 & x < 1, round(x, 3),
                ifelse(x == 1, "1", format(x, scientific = FALSE))))
}

# Apply custom formatting to Combined_P_Value and Adjusted_P_Value columns
merged_df[c("Combined_P_Value", "Adjusted_P_Value")] <- lapply(merged_df[c("Combined_P_Value", "Adjusted_P_Value")], format_pvalues)

# Get a vector of column names containing "_count"
count_columns <- grep("_count", colnames(merged_df), value = TRUE)

# Iterate over these columns, and round each to 2 decimal places
merged_df <- merged_df |>
  mutate(across(all_of(count_columns), ~ round(., digits = 2)))

# Get a vector of column names starting with "P_Value_fam"
pvalue_columns <- grep("^P_Value_fam", colnames(merged_df), value = TRUE)

# Convert P_Value columns to numeric
merged_df <- 
  merged_df |>
  mutate(across(all_of(pvalue_columns), as.numeric))

# Apply custom formatting to P_Value columns
merged_df[pvalue_columns] <-
  lapply(merged_df[pvalue_columns], format_pvalues)

# Update the counts
pvalue_columns <-
  grep("P_Value_fam", colnames(merged_df), value = TRUE)

merged_df <-
  merged_df |>
  mutate(
    Family_Count = rowSums(!is.na(across(all_of(pvalue_columns))))
  )

# Convert head(results) to a tibble
table_result <- 
  as_tibble(merged_df)

# Set theme if you want to use something different from the previous table
set_flextable_defaults(
  font.family = "Arial",
  font.size = 9,
  big.mark = ",",
  theme_fun = "theme_zebra" # try the themes: theme_alafoli(), theme_apa(), theme_booktabs(), theme_box(), theme_tron_legacy(), theme_tron(), theme_vader(), theme_vanilla(), theme_zebra()
)

# Then create the flextable object
flex_table <- 
flextable(head(table_result)) |>
  set_caption(caption = as_paragraph(
    as_chunk(
      "Table 6. Output of 100 SNPs to test our code (header) .",
      props = fp_text_default(color = "#000000", font.size = 14)
    )
  ),
  fp_p = fp_par(text.align = "center", padding = 5))

# Print the flextable
flex_table
```

Table 6. Output of 100 SNPs to test our code (header) .

| SNP | Expected\_A1\_count\_fam1 | Observed\_A1\_count\_fam1 | Expected\_A2\_count\_fam1 | Observed\_A2\_count\_fam1 | P\_Value\_fam1 | Expected\_A1\_count\_fam2 | Observed\_A1\_count\_fam2 | Expected\_A2\_count\_fam2 | Observed\_A2\_count\_fam2 | P\_Value\_fam2 | Expected\_A1\_count\_fam3 | Observed\_A1\_count\_fam3 | Expected\_A2\_count\_fam3 | Observed\_A2\_count\_fam3 | P\_Value\_fam3 | Expected\_A1\_count\_fam4 | Observed\_A1\_count\_fam4 | Expected\_A2\_count\_fam4 | Observed\_A2\_count\_fam4 | P\_Value\_fam4 | Expected\_A1\_count\_fam5 | Observed\_A1\_count\_fam5 | Expected\_A2\_count\_fam5 | Observed\_A2\_count\_fam5 | P\_Value\_fam5 | Expected\_A1\_count\_fam6 | Observed\_A1\_count\_fam6 | Expected\_A2\_count\_fam6 | Observed\_A2\_count\_fam6 | P\_Value\_fam6 | Family\_Count | Combined\_P\_Value | Adjusted\_P\_Value |
| --- | --- | --- | --- | --- | --- | --- | --- | --- | --- | --- | --- | --- | --- | --- | --- | --- | --- | --- | --- | --- | --- | --- | --- | --- | --- | --- | --- | --- | --- | --- | --- | --- | --- |
| AX-579436016 |  |  |  |  |  |  |  |  |  |  |  |  |  |  |  |  |  |  |  |  |  |  |  |  |  | 9.0 | 8 | 3.0 | 4 | 1 | 1 | 1 | 1 |
| AX-579436089 |  |  |  |  |  |  |  |  |  |  | 33 | 29 | 11 | 15 | 0.164 |  |  |  |  |  | 27 | 26 | 9 | 10 | 0.7 |  |  |  |  |  | 2 | 0.363 | 1 |
| AX-579436125 |  |  |  |  |  |  |  |  |  |  |  |  |  |  |  |  |  |  |  |  | 27 | 36 | 9 | 0 | 0.002 |  |  |  |  |  | 1 | 0.002 | 0.411 |
| AX-579436149 |  |  |  |  |  | 21 | 18 | 21 | 24 | 0.355 | 23 | 18 | 23 | 28 | 0.14 |  |  |  |  |  |  |  |  |  |  |  |  |  |  |  | 2 | 0.199 | 1 |
| AX-579436243 | 44 | 23 | 44 | 65 | 7.59e-06 |  |  |  |  |  |  |  |  |  |  | 40.5 | 33 | 13.5 | 21 | 0.018 |  |  |  |  |  | 10.5 | 11 | 3.5 | 3 | 1 | 3 | 1.97e-05 | 0.004 |
| AX-579436298 |  |  |  |  |  | 21 | 21 | 21 | 21 | 1 | 23 | 23 | 23 | 23 | 1 |  |  |  |  |  |  |  |  |  |  | 7.0 | 7 | 7.0 | 7 | 1 | 3 | 1 | 1 |

Select p values columns

```
# Select columns with "SNP" and "P_Value" in the column name
selected_columns <- c("SNP", grep("P_Value", colnames(merged_df), value = TRUE), "Family_Count")

# Create a new dataframe with selected columns
selected_df <- merged_df[selected_columns]

# Reorder the columns in selected_df
selected_df <- selected_df[, c(selected_columns[1:7], selected_columns[10], selected_columns[8], selected_columns[9])]

# Set theme if you want to use something different from the previous table
set_flextable_defaults(
  font.family = "Arial",
  font.size = 9,
  big.mark = ",",
  theme_fun = "theme_zebra" # try the themes: theme_alafoli(), theme_apa(), theme_booktabs(), theme_box(), theme_tron_legacy(), theme_tron(), theme_vader(), theme_vanilla(), theme_zebra()
)

# Then create the flextable object
flex_table <- flextable(head(selected_df)) |>
  set_caption(caption = as_paragraph(
    as_chunk(
      "Table 6. Output of 100 SNPs to test our code (header).",
      props = fp_text_default(color = "#000000", font.size = 14)
    )
  ),
  fp_p = fp_par(text.align = "center", padding = 5))

# Print the flextable
flex_table
```

Table 6. Output of 100 SNPs to test our code (header).

| SNP | P\_Value\_fam1 | P\_Value\_fam2 | P\_Value\_fam3 | P\_Value\_fam4 | P\_Value\_fam5 | P\_Value\_fam6 | Family\_Count | Combined\_P\_Value | Adjusted\_P\_Value |
| --- | --- | --- | --- | --- | --- | --- | --- | --- | --- |
| AX-579436016 |  |  |  |  |  | 1 | 1 | 1 | 1 |
| AX-579436089 |  |  | 0.164 |  | 0.7 |  | 2 | 0.363 | 1 |
| AX-579436125 |  |  |  |  | 0.002 |  | 1 | 0.002 | 0.411 |
| AX-579436149 |  | 0.355 | 0.14 |  |  |  | 2 | 0.199 | 1 |
| AX-579436243 | 7.59e-06 |  |  | 0.018 |  | 1 | 3 | 1.97e-05 | 0.004 |
| AX-579436298 |  | 1 | 1 |  |  | 1 | 3 | 1 | 1 |

Save the results in Excel format

```
# Specify the path and file name for the Excel file
excel_file <-
  here("output",
       "segregation",
       "albopictus",
       "results_pvalues_100_SNPs_01.xlsx")

# Create an empty Excel workbook
wb <- openxlsx::createWorkbook()

# Create a worksheet in the workbook
openxlsx::addWorksheet(wb, sheetName = "Results")

# Write the data frame to the worksheet
openxlsx::writeData(wb, sheet = "Results", selected_df)

# Save the workbook as an Excel file
openxlsx::saveWorkbook(wb, file = excel_file, overwrite = TRUE)
```

We can check the range of the p-values

```
# Calculate the range for each column (excluding SNP)
column_ranges <-
  selected_df |>
  dplyr::select(-SNP) |>
  reframe(
    across(
      .cols = everything(),
      .fns = list(
        range = ~ range(as.numeric(.), na.rm = TRUE)
      ),
      .names = "{.col}_{.fn}"
    )
  )

# Print the column ranges
print(column_ranges)
```

```
## # A tibble: 2 × 9
##   P_Value_fam1_range P_Value_fam2_range P_Value_fam3_range P_Value_fam4_range
##                <dbl>              <dbl>              <dbl>              <dbl>
## 1           7.89e-17          0.0000147        0.000000793           1.86e-10
## 2           1   e+ 0          1                1                     1   e+ 0
## # ℹ 5 more variables: P_Value_fam5_range <dbl>, P_Value_fam6_range <dbl>,
## #   Family_Count_range <dbl>, Combined_P_Value_range <dbl>,
## #   Adjusted_P_Value_range <dbl>
```

We see values from 0 to 1. I created a format function to make zero
as 0 and not 00e+00, values from 0 to 0.001 will have scientific
notation, 0.001 to 1 will have 3 decimals (rounded).

Check how many SNPs we tested. Remember, each family has a different
set of SNPs. So, we end up testing more than 500 SNPs.

```
length(
  unique(
    selected_df$SNP
  )
)
```

```
## [1] 205
```

The p-value is a measure of the likelihood that the observed
difference from expected occurred due to random chance. A higher p-value
suggests the frequencies could be randomly distributed, while a lower
p-value suggests non-random distribution. The conventional threshold to
decide whether a result is statistically significant is often set at
0.05.

In this case, you could consider those SNPs as randomly distributed
whose adjusted p-value is more than 0.05 in all the families where they
appear.

We can use the adjusted p values to filter out SNPs

```
randomly_distributed_SNPs3 <-
  selected_df |>
  filter(Adjusted_P_Value > 0.05) |>
  pull(SNP) |>
  unique()

length(randomly_distributed_SNPs3)
```

```
## [1] 197
```

We tested 205 SNPs and 197 passed our test.

## 11. Sanity checks

Check the mim and max count values for each allele in each family

```
# Define the list of variables
vars <-
  c(
    "Expected_A1_count",
    "Expected_A2_count",
    "Observed_A1_count",
    "Observed_A2_count"
  )

# Define the lists of data frames
dfs_parents <- list(
  fam1 = family_parents_6_subset_with_zeros$fam1,
  fam2 = family_parents_6_subset_with_zeros$fam2,
  fam3 = family_parents_6_subset_with_zeros$fam3,
  fam4 = family_parents_6_subset_with_zeros$fam4,
  fam5 = family_parents_6_subset_with_zeros$fam5,
  fam6 = family_parents_6_subset_with_zeros$fam6
)

dfs_offspring <- list(
  fam1 = family_offspring_6_subset_with_zeros$fam1,
  fam2 = family_offspring_6_subset_with_zeros$fam2,
  fam3 = family_offspring_6_subset_with_zeros$fam3,
  fam4 = family_offspring_6_subset_with_zeros$fam4,
  fam5 = family_offspring_6_subset_with_zeros$fam5,
  fam6 = family_offspring_6_subset_with_zeros$fam6
)

# Initialize the result lists
max_vals <- list()
min_vals <- list()

# Iterate over all variables
for (var in vars) {
  # Calculate the maximum values
  max_vals[[var]] <-
    max(sapply(dfs_parents, function(x)
      max(x[[var]])),
      sapply(dfs_offspring, function(x)
        max(x[[var]])))
  
  # Calculate the minimum values
  min_vals[[var]] <-
    min(sapply(dfs_parents, function(x)
      min(x[[var]])),
      sapply(dfs_offspring, function(x)
        min(x[[var]])))
}

# Print the maximum values
print(max_vals)
```

```
## $Expected_A1_count
## [1] 66
## 
## $Expected_A2_count
## [1] 66
## 
## $Observed_A1_count
## [1] 88
## 
## $Observed_A2_count
## [1] 70.9984
```

```
# Print the minimum values
print(min_vals)
```

```
## $Expected_A1_count
## [1] 3
## 
## $Expected_A2_count
## [1] 3
## 
## $Observed_A1_count
## [1] 0
## 
## $Observed_A2_count
## [1] 0
```

Check summary statistics

```
# Define the function to calculate the mode
calc_mode <- function(x) {
  ux <- unique(x)
  ux[which.max(tabulate(match(x, ux)))]
}

# Define the function to calculate summary statistics
calc_summary_stats <- function(df, column) {
  summarise(
    df,
    Mean = mean(df[[column]], na.rm = TRUE),
    Median = median(df[[column]], na.rm = TRUE),
    Mode = calc_mode(df[[column]]), # Mode calculation updated
    Quantile_0_0.25 = quantile(df[[column]], 0.25, na.rm = TRUE),
    Quantile_0.25_0.5 = quantile(df[[column]], 0.5, na.rm = TRUE),
    Quantile_0.5_0.75 = quantile(df[[column]], 0.75, na.rm = TRUE),
    Quantile_0.75_1 = quantile(df[[column]], 1, na.rm = TRUE)
  )
}

# Initialize the table to store the results
table_result <- data.frame(
  Family = character(),
  Allele = character(),
  Mean = numeric(),
  Median = numeric(),
  Mode = numeric(),
  Quantile_0_0.25 = numeric(),
  Quantile_0.25_0.5 = numeric(),
  Quantile_0.5_0.75 = numeric(),
  Quantile_0.75_1 = numeric(),
  stringsAsFactors = FALSE
)


# Iterate over each family
for (i in names(family_parents_6_subset_with_zeros)) {
  family_parents <- family_parents_6_subset_with_zeros[[i]]
  family_offspring <- family_offspring_6_subset_with_zeros[[i]]
  
  # Get the SNPs shared between parents and offspring within the family
  shared_snps <- intersect(family_parents$SNP, family_offspring$SNP)
  
  # Filter the family dataset and offspring dataset to include only the shared SNPs
  family_parents_shared <- dplyr::filter(family_parents, SNP %in% shared_snps)
  family_offspring_shared <- dplyr::filter(family_offspring, SNP %in% shared_snps)
  
  # For each allele, calculate summary statistics and append them to the table
  for (allele in c("Expected_A1_count", "Observed_A1_count", "Expected_A2_count", "Observed_A2_count")) {
    if (grepl("Expected", allele)) {
      df <- family_parents_shared
    } else {
      df <- family_offspring_shared
    }
    
    summary_stats <- calc_summary_stats(df, allele)
    
    table_result <- bind_rows(
      table_result,
      data.frame(
        Family = i,
        Allele = allele,
        Mean = summary_stats$Mean,
        Median = summary_stats$Median,
        Mode = summary_stats$Mode,
        Quantile_0_0.25 = summary_stats$Quantile_0_0.25,
        Quantile_0.25_0.5 = summary_stats$Quantile_0.25_0.5,
        Quantile_0.5_0.75 = summary_stats$Quantile_0.5_0.75,
        Quantile_0.75_1 = summary_stats$Quantile_0.75_1
      )
    )
  }
}


# Create a flextable object
flex_table <- flextable(table_result)

# Print the flextable
flex_table
```

| Family | Allele | Mean | Median | Mode | Quantile\_0\_0.25 | Quantile\_0.25\_0.5 | Quantile\_0.5\_0.75 | Quantile\_0.75\_1 |
| --- | --- | --- | --- | --- | --- | --- | --- | --- |
| fam1 | Expected\_A1\_count | 47.305000 | 44.0000 | 44.0000 | 42.75000 | 44.0000 | 66.00000 | 66.00000 |
| fam1 | Observed\_A1\_count | 48.399895 | 46.4992 | 44.0000 | 36.25230 | 46.4992 | 64.99680 | 88.00000 |
| fam1 | Expected\_A2\_count | 38.315000 | 43.0000 | 44.0000 | 22.00000 | 43.0000 | 44.00000 | 66.00000 |
| fam1 | Observed\_A2\_count | 37.220105 | 39.9984 | 44.0000 | 20.99680 | 39.9984 | 47.00080 | 70.99840 |
| fam2 | Expected\_A1\_count | 21.825000 | 21.0000 | 21.0000 | 17.75000 | 21.0000 | 31.50000 | 31.50000 |
| fam2 | Observed\_A1\_count | 22.229966 | 21.0000 | 21.0000 | 13.99860 | 21.0000 | 30.00060 | 35.99820 |
| fam2 | Expected\_A2\_count | 19.335000 | 21.0000 | 21.0000 | 10.50000 | 21.0000 | 21.00000 | 31.50000 |
| fam2 | Observed\_A2\_count | 18.930034 | 20.5002 | 21.0000 | 11.99940 | 20.5002 | 23.99925 | 32.99940 |
| fam3 | Expected\_A1\_count | 23.510000 | 23.0000 | 23.0000 | 11.50000 | 23.0000 | 34.50000 | 34.50000 |
| fam3 | Observed\_A1\_count | 24.529846 | 28.9984 | 28.9984 | 15.00060 | 28.9984 | 30.99940 | 38.00060 |
| fam3 | Expected\_A2\_count | 22.170000 | 23.0000 | 23.0000 | 11.50000 | 23.0000 | 34.50000 | 34.50000 |
| fam3 | Observed\_A2\_count | 21.150154 | 17.0016 | 17.0016 | 15.00060 | 17.0016 | 30.25040 | 38.00060 |
| fam4 | Expected\_A1\_count | 31.195000 | 27.0000 | 40.5000 | 27.00000 | 27.0000 | 40.50000 | 40.50000 |
| fam4 | Observed\_A1\_count | 31.150106 | 32.4999 | 41.0022 | 26.00075 | 32.4999 | 39.99780 | 47.00160 |
| fam4 | Expected\_A2\_count | 22.085000 | 24.5000 | 13.5000 | 13.50000 | 24.5000 | 27.00000 | 40.50000 |
| fam4 | Observed\_A2\_count | 22.129894 | 21.0006 | 12.9978 | 14.00220 | 21.0006 | 27.00000 | 54.00000 |
| fam5 | Expected\_A1\_count | 18.425000 | 18.0000 | 18.0000 | 14.75000 | 18.0000 | 27.00000 | 27.00000 |
| fam5 | Observed\_A1\_count | 18.169880 | 18.0000 | 18.0000 | 13.00050 | 18.0000 | 24.25050 | 36.00000 |
| fam5 | Expected\_A2\_count | 16.695000 | 18.0000 | 18.0000 | 9.00000 | 18.0000 | 18.00000 | 27.00000 |
| fam5 | Observed\_A2\_count | 16.950120 | 18.0000 | 18.0000 | 11.00160 | 18.0000 | 20.25090 | 28.00080 |
| fam6 | Expected\_A1\_count | 7.770000 | 7.0000 | 10.5000 | 7.00000 | 7.0000 | 10.50000 | 10.50000 |
| fam6 | Observed\_A1\_count | 8.420065 | 9.0006 | 10.0002 | 6.75010 | 9.0006 | 10.00020 | 12.99998 |
| fam6 | Expected\_A2\_count | 6.090000 | 7.0000 | 3.5000 | 3.50000 | 7.0000 | 7.00000 | 10.50000 |
| fam6 | Observed\_A2\_count | 5.439935 | 4.9994 | 3.9998 | 3.74975 | 4.9994 | 7.00000 | 13.00040 |

Plot the stats

```
# Reshape data for ggplot
table_result_long <- tidyr::pivot_longer(
  table_result,
  c(
    "Mean",
    "Median",
    "Mode",
    "Quantile_0_0.25",
    "Quantile_0.25_0.5",
    "Quantile_0.5_0.75",
    "Quantile_0.75_1"
  ),
  names_to = "Statistic",
  values_to = "Value"
)

# Source function theme
source(here("scripts", "analysis", "my_theme.R"))

# Define the desired order of the factor levels
allele_order <- c("Expected_A1_count", "Observed_A1_count", "Expected_A2_count", "Observed_A2_count")

# Convert the Allele variable to a factor with the desired order
table_result_long$Allele <- factor(table_result_long$Allele, levels = allele_order)


# Generate boxplot
p3 <-
  ggplot(table_result_long,
         aes(
           x = Family,
           y = Value,
           fill = Allele,
           color = Allele
         )) +
  facet_wrap(~ Statistic, scales = "fixed") +
  geom_boxplot() +
  my_theme() +
  theme(axis.text.x = element_text(angle = 90),
        legend.position = "top") +
  labs(title = "Boxplot of Quantiles for each Family", x = "Family", y = "Value")

# check plot
p3
```

```
# Save Venn diagram to PDF
# output_path <-
#   here("output",
#        "segregation",
#        "albopictus",
#        "count_stats.pdf")
# ggsave(output_path,
#        p3,
#        height = 5,
#        width = 8,
#        dpi = 300)
```

The fam1 is the largest (44 offspring) while the fam6 is the smallest
(6 offspring)

## 12. Segregation test with all SNPs

I updated the code to use the library data.table and tried to make
the code more efficient. It still take a long time to run, so I saved
the output in .rds format.

Code summary:

**1. Initialize Data Table**

The script begins by initializing a ‘data.table’ named ‘combined\_dt’
with the specified columns: Family, SNP, Expected\_A1\_count,
Expected\_A2\_count, Observed\_A1\_count, Observed\_A2\_count, P\_Value,
Combined\_P\_Value, Family\_Count. These columns will hold specific types
of data (such as character, numeric, or integer).

**2. Extract Unique SNPs**

The ‘all\_snps’ vector is created by extracting all unique SNP values
from the list ‘family\_parents\_3’.

**3. Main Loop Over SNPs**

The main part of the script starts with a loop over all SNPs, in
which each SNP is analyzed separately.

**4. Identify Families with the SNP**

For each SNP, the script identifies families that contain this
SNP.

**5. Skip SNPs Not Present in Families**

If no family contains the SNP, the loop skips to the next SNP.

**6. Perform Statistical Tests**

If a family does contain the SNP, the script performs a statistical
test (either Fisher’s Exact Test or Chi-Square Test, depending on the
observed and expected counts) to compare the observed and expected
counts of two alleles (A1 and A2) in the parents and the offspring.

**7. Add Results to Data Table**

The result of the statistical test and the data for the SNP (expected
and observed counts, family name, etc.) are then added to the
‘combined\_dt’.

**8. Save the Data**

After the loop over SNPs is finished, the ‘combined\_dt’ is saved to
an .rds file for future use.

**9. Widen the Data Frame**

Next, the script transforms ‘combined\_dt’ into a wider format using
the ‘pivot\_wider()’ function. The new data frame ‘new\_df’ contains
separate columns for each family.

**10. Aggregate the Data**

Then, ‘new\_df’ is aggregated into ‘merged\_df’ by grouping by SNP and
summarizing the columns.

**11. Calculate Combined P-Values**

In the next step, the script calculates a combined P-value for each
SNP across all families. If more than one P-value is present for a SNP,
they are combined using the Fisher’s method; if only one P-value is
present, it is used as is.

**12. Adjust for Multiple Comparisons**

After that, the script adjusts the combined P-values for multiple
comparisons using the Holm method.

**13. Apply Custom Formatting and Round Counts**

Then, the script applies custom formatting to P-values and rounds
count values to 2 decimal places.

**14. Convert to Tibble**

After some more data transformations, the ‘merged\_df’ is converted
into a tibble ‘table\_result’.

**15. Visualize the Data**

Finally, ‘table\_result’ is visualized using the ‘flextable’ function
to create a nicely formatted table.

### 12.1 Estimate p-values using chi-square test (count > 5) or Fisher test (counts < 5)

```
# Initialize the data.table with the same columns as your data.frame
combined_dt <- data.table(
  Family = character(0),
  SNP = character(0),
  Expected_A1_count = numeric(0),
  Expected_A2_count = numeric(0),
  Observed_A1_count = numeric(0),
  Observed_A2_count = numeric(0),
  P_Value = numeric(0),
  Combined_P_Value = numeric(0),
  Family_Count = integer(0)
)


# Get all unique SNPs across all families - use _with_zeros or no_zeros to see how many SNPs
all_snps <-
  unique(unlist(lapply(family_parents_3, function(x) {
    x$SNP
  })))

# Perform the analysis for each SNP
for (snp in all_snps) {
  # Get the families that contain the SNP
  families_with_snp <- lapply(family_parents_3, function(family) {
    snp %in% family$SNP
  })
  families_with_snp_index <- unlist(families_with_snp)
  
  # Check if any family contains the SNP
  if (sum(families_with_snp_index) == 0) {
    next
  }
  
  # Go through each family that contains the SNP
  for (i in which(families_with_snp_index)) {
    family_parents <- family_parents_3[[i]]
    family_offspring <- family_offspring_3[[i]]
    
    # Check if the SNP is in the offspring
    if (snp %in% family_offspring$SNP) {
      # Conduct the chi-square test
      observed_counts <-
        c(family_offspring[family_offspring$SNP == snp, "Observed_A1_count"],
          family_offspring[family_offspring$SNP == snp, "Observed_A2_count"])
      expected_counts <-
        c(family_parents[family_parents$SNP == snp, "Expected_A1_count"],
          family_parents[family_parents$SNP == snp, "Expected_A2_count"])
      if (min(observed_counts, expected_counts) < 5) {
        # If either of the counts is less than 5, use Fisher's Exact Test
        test_result <-
          fisher.test(matrix(c(
            observed_counts, expected_counts
          ), nrow = 2))$p.value
      } else {
        # Otherwise use Chi-Square Test
        test_result <-
          chisq.test(observed_counts, p = expected_counts / sum(expected_counts))$p.value
      }
      
      # Add the details to the data table
      combined_dt <- rbindlist(list(
        combined_dt,
        data.table(
          Family = names(family_parents_3)[i],
          SNP = snp,
          Expected_A1_count = family_parents[family_parents$SNP == snp, "Expected_A1_count"],
          Observed_A1_count = family_offspring[family_offspring$SNP == snp, "Observed_A1_count"],
          Expected_A2_count = family_parents[family_parents$SNP == snp, "Expected_A2_count"],
          Observed_A2_count = family_offspring[family_offspring$SNP == snp, "Observed_A2_count"],
          P_Value = test_result,
          Combined_P_Value = NA,
          Family_Count = sum(families_with_snp_index)
        )
      ),
      fill = TRUE,
      use.names = TRUE)
    }
  }
}
```

### 12.2 Save the data and load it back

We will save the data and load it back since we used eval=FALSE in
the chunk to estimate the p-values.

```
# Save the data because it takes a long time to run, when kniting the document, comment it out
# saveRDS(
#   combined_dt,
#   file = here(
#     "output",
#     "segregation",
#     "albopictus",
#     "combined_dt_all_SNPs.rds"
#   )
# )

# Load the data
combined_dt <-
  readRDS(
    file = here(
      "output",
      "segregation",
      "albopictus",
      "combined_dt_all_SNPs.rds"
    )
  )
```

### 12.3 Combine and adjust p-values

```
# Create new data frame using pivot_wider()
new_df <-
  combined_dt |>
  pivot_wider(
    names_from = Family,
    values_from = c(
      Expected_A1_count,
      Observed_A1_count,
      Expected_A2_count,
      Observed_A2_count,
      P_Value
    )
  ) |>
  rename_with(~ gsub("(\\d+)_(Expected|Observed)", paste0("fam\\1_", "\\2"), .),
              starts_with("Expected")) |>
  rename_with(~ gsub("(\\d+)_(Expected|Observed)", paste0("fam\\1_", "\\2"), .),
              starts_with("Observed"))

# Merge rows
merged_df_2 <-
  new_df |>
  group_by(SNP) |>
  summarise(
    across(
      starts_with("Expected_A1_count_fam"),
      ~ ifelse(all(is.na(.)), NA, unique(.[!is.na(.)])[1])
    ),
    across(
      starts_with("Observed_A1_count_fam"),
      ~ ifelse(all(is.na(.)), NA, unique(.[!is.na(.)])[1])
    ),
    across(
      starts_with("Expected_A2_count_fam"),
      ~ ifelse(all(is.na(.)), NA, unique(.[!is.na(.)])[1])
    ),
    across(
      starts_with("Observed_A2_count_fam"),
      ~ ifelse(all(is.na(.)), NA, unique(.[!is.na(.)])[1])
    ),
    across(starts_with("P_Value_fam"),
           ~ ifelse(all(is.na(
             .
           )), NA, unique(.[!is.na(.)])[1])),
    Family_Count = dplyr::first(Family_Count),
    Combined_P_Value = dplyr::first(Combined_P_Value)
  ) |>
  ungroup() |>
  dplyr::select(
    SNP,
    starts_with("Expected_A1_count_fam"),
    starts_with("Expected_A2_count_fam"),
    starts_with("Observed_A1_count_fam"),
    starts_with("Observed_A2_count_fam"),
    starts_with("P_Value_fam"),
    Family_Count,
    Combined_P_Value
  )

# set output order
merged_df_2 <-
  merged_df_2 |>
  dplyr::select(
    SNP,
    starts_with("Expected_A1_count_fam1"),
    starts_with("Observed_A1_count_fam1"),
    starts_with("Expected_A2_count_fam1"),
    starts_with("Observed_A2_count_fam1"),
    starts_with("P_Value_fam1"),
    starts_with("Expected_A1_count_fam2"),
    starts_with("Observed_A1_count_fam2"),
    starts_with("Expected_A2_count_fam2"),
    starts_with("Observed_A2_count_fam2"),
    starts_with("P_Value_fam2"),
    starts_with("Expected_A1_count_fam3"),
    starts_with("Observed_A1_count_fam3"),
    starts_with("Expected_A2_count_fam3"),
    starts_with("Observed_A2_count_fam3"),
    starts_with("P_Value_fam3"),
    starts_with("Expected_A1_count_fam4"),
    starts_with("Observed_A1_count_fam4"),
    starts_with("Expected_A2_count_fam4"),
    starts_with("Observed_A2_count_fam4"),
    starts_with("P_Value_fam4"),
    starts_with("Expected_A1_count_fam5"),
    starts_with("Observed_A1_count_fam5"),
    starts_with("Expected_A2_count_fam5"),
    starts_with("Observed_A2_count_fam5"),
    starts_with("P_Value_fam5"),
    starts_with("Expected_A1_count_fam6"),
    starts_with("Observed_A1_count_fam6"),
    starts_with("Expected_A2_count_fam6"),
    starts_with("Observed_A2_count_fam6"),
    starts_with("P_Value_fam6"),
    Family_Count,
    Combined_P_Value
  )

# Combine the p values
# Iterate over each row
for (i in 1:nrow(merged_df_2)) {
  p_values <- numeric()
  
  # Check P-values in each family column
  for (j in 1:6) {
    col_name <- paste0("P_Value_fam", j)
    if (!is.na(merged_df_2[i, col_name])) {
      p_value <- as.numeric(merged_df_2[i, col_name])
      if (!is.na(p_value)) {
        p_values <- c(p_values, p_value)
      }
    }
  }
  
  # Combine P-values if more than one is present
  if (length(p_values) > 1) {
    combined_p_value <- pchisq(-2 * sum(log(p_values)),
                               df = 2 * length(p_values),
                               lower.tail = FALSE)
    merged_df_2[i, "Combined_P_Value"] <- combined_p_value
  } else if (length(p_values) == 1) {
    merged_df_2[i, "Combined_P_Value"] <- p_values
  }
}

# Calculate Adjusted P-values
adjusted_p_values <-
  p.adjust(merged_df_2$Combined_P_Value, method = "holm")
merged_df_2$Adjusted_P_Value <- adjusted_p_values


# Custom formatting function for p-values
format_pvalues <- function(x) {
  ifelse(x < 0.001,
         formatC(x, format = "e", digits = 2),
         ifelse(x >= 0.001 & x < 1, round(x, 3),
                ifelse(x == 1, "1", format(x, scientific = FALSE))))
}

# Apply custom formatting to Combined_P_Value and Adjusted_P_Value columns
merged_df_2[c("Combined_P_Value", "Adjusted_P_Value")] <-
  lapply(merged_df_2[c("Combined_P_Value", "Adjusted_P_Value")], format_pvalues)

# Get a vector of column names containing "_count"
count_columns <-
  grep("_count", colnames(merged_df_2), value = TRUE)

# Iterate over these columns, and round each to 2 decimal places
merged_df_2 <-
  merged_df_2 |>
  mutate(across(all_of(count_columns), ~ round(., digits = 2)))

# Get a vector of column names starting with "P_Value_fam"
pvalue_columns <-
  grep("^P_Value_fam", colnames(merged_df_2), value = TRUE)

# Convert P_Value columns to numeric
merged_df_2 <-
  merged_df_2 |>
  mutate(across(all_of(pvalue_columns), as.numeric))

# Apply custom formatting to P_Value columns
merged_df_2[pvalue_columns] <-
  lapply(merged_df_2[pvalue_columns], format_pvalues)

# Update the counts
pvalue_columns <-
  grep("P_Value_fam", colnames(merged_df_2), value = TRUE)

merged_df_2 <-
  merged_df_2 |>
  mutate(Family_Count = rowSums(!is.na(across(
    all_of(pvalue_columns)
  ))))

# Convert head(results) to a tibble
table_result <-
  as_tibble(merged_df_2)

# Set theme if you want to use something different from the previous table
set_flextable_defaults(
  font.family = "Arial",
  font.size = 9,
  big.mark = ",",
  theme_fun = "theme_zebra" # try the themes: theme_alafoli(), theme_apa(), theme_booktabs(), theme_box(), theme_tron_legacy(), theme_tron(), theme_vader(), theme_vanilla(), theme_zebra()
)

# Then create the flextable object
flex_table <-
  flextable(head(table_result)) |>
  set_caption(caption = as_paragraph(
    as_chunk(
      "Table 6. Output of all SNPs to test our code (header) .",
      props = fp_text_default(color = "#000000", font.size = 14)
    )
  ),
  fp_p = fp_par(text.align = "center", padding = 5))

# Print the flextable
flex_table
```

Table 6. Output of all SNPs to test our code (header) .

| SNP | Expected\_A1\_count\_fam1 | Observed\_A1\_count\_fam1 | Expected\_A2\_count\_fam1 | Observed\_A2\_count\_fam1 | P\_Value\_fam1 | Expected\_A1\_count\_fam2 | Observed\_A1\_count\_fam2 | Expected\_A2\_count\_fam2 | Observed\_A2\_count\_fam2 | P\_Value\_fam2 | Expected\_A1\_count\_fam3 | Observed\_A1\_count\_fam3 | Expected\_A2\_count\_fam3 | Observed\_A2\_count\_fam3 | P\_Value\_fam3 | Expected\_A1\_count\_fam4 | Observed\_A1\_count\_fam4 | Expected\_A2\_count\_fam4 | Observed\_A2\_count\_fam4 | P\_Value\_fam4 | Expected\_A1\_count\_fam5 | Observed\_A1\_count\_fam5 | Expected\_A2\_count\_fam5 | Observed\_A2\_count\_fam5 | P\_Value\_fam5 | Expected\_A1\_count\_fam6 | Observed\_A1\_count\_fam6 | Expected\_A2\_count\_fam6 | Observed\_A2\_count\_fam6 | P\_Value\_fam6 | Family\_Count | Combined\_P\_Value | Adjusted\_P\_Value |
| --- | --- | --- | --- | --- | --- | --- | --- | --- | --- | --- | --- | --- | --- | --- | --- | --- | --- | --- | --- | --- | --- | --- | --- | --- | --- | --- | --- | --- | --- | --- | --- | --- | --- |
| AX-579436016 |  |  |  |  |  |  |  |  |  |  |  |  |  |  |  |  |  |  |  |  |  |  |  |  |  | 9.0 | 8 | 3.0 | 4 | 1 | 1 | 1 | 1 |
| AX-579436089 |  |  |  |  |  |  |  |  |  |  | 33 | 29 | 11 | 15 | 0.164 |  |  |  |  |  | 27 | 26 | 9 | 10 | 0.7 |  |  |  |  |  | 2 | 0.363 | 1 |
| AX-579436125 |  |  |  |  |  |  |  |  |  |  |  |  |  |  |  |  |  |  |  |  | 27 | 36 | 9 | 0 | 0.002 |  |  |  |  |  | 1 | 0.002 | 1 |
| AX-579436149 |  |  |  |  |  | 21 | 18 | 21 | 24 | 0.355 | 23 | 18 | 23 | 28 | 0.14 |  |  |  |  |  |  |  |  |  |  |  |  |  |  |  | 2 | 0.199 | 1 |
| AX-579436243 | 44 | 23 | 44 | 65 | 7.59e-06 |  |  |  |  |  |  |  |  |  |  | 40.5 | 33 | 13.5 | 21 | 0.018 |  |  |  |  |  | 10.5 | 11 | 3.5 | 3 | 1 | 3 | 1.97e-05 | 1 |
| AX-579436298 |  |  |  |  |  | 21 | 21 | 21 | 21 | 1 | 23 | 23 | 23 | 23 | 1 |  |  |  |  |  |  |  |  |  |  | 7.0 | 7 | 7.0 | 7 | 1 | 3 | 1 | 1 |

### 12.4 Select p-values columns

Code explanation:

**1. Selecting Columns:** The script starts by creating
a vector ‘selected\_columns’ that contains the names of the columns to be
selected from the data frame ‘merged\_df’. These columns are “SNP”, all
the columns with “P\_Value” in their names, and “Family\_Count”.

**2. Creating a New Data Frame:** Then, a new data frame
‘selected\_df’ is created by subsetting ‘merged\_df’ with
‘selected\_columns’.

**3. Reordering Columns:** Next, the script reorders the
columns in ‘selected\_df’. The order of the columns is specified by
indexing ‘selected\_columns’.

**4. Setting Table Defaults:** Following that, the
script sets default parameters for the upcoming table creation with
‘set\_flextable\_defaults()’. This includes the font, font size, digit
grouping symbol (big.mark), and the theme.

**5. Creating a Flextable Object:** Then, the script
creates a flextable object ‘flex\_table’ from the first few rows of
‘selected\_df’ using the ‘flextable()’ and ‘head()’ functions.

**6. Adding a Caption:** The ‘set\_caption()’ function is
used to add a caption to ‘flex\_table’. The caption is formatted using
‘as\_paragraph()’ and ‘as\_chunk()’ functions, with specific text
properties set using ‘fp\_text\_default()’. The ‘fp\_par()’ function is
used to align the caption text to the center and add padding.

**7. Printing the Flextable:** Finally, the flextable
‘flex\_table’ is printed.

```
# Select columns with "SNP" and "P_Value" in the column name
selected_columns <-
  c("SNP", grep("P_Value", colnames(merged_df_2), value = TRUE), "Family_Count")

# Create a new dataframe with selected columns
selected_df_2 <- 
  merged_df_2[selected_columns]

# Reorder the columns in selected_df_2
selected_df_2 <-
  selected_df_2[, c(selected_columns[1:7],
                  selected_columns[10],
                  selected_columns[8],
                  selected_columns[9])]

# Set theme if you want to use something different from the previous table
set_flextable_defaults(
  font.family = "Arial",
  font.size = 9,
  big.mark = ",",
  theme_fun = "theme_zebra" # try the themes: theme_alafoli(), theme_apa(), theme_booktabs(), theme_box(), theme_tron_legacy(), theme_tron(), theme_vader(), theme_vanilla(), theme_zebra()
)

# Then create the flextable object
flex_table <- flextable(head(selected_df_2)) |>
  set_caption(caption = as_paragraph(
    as_chunk(
      "Table 6. Output of all SNPs to test our code (header).",
      props = fp_text_default(color = "#000000", font.size = 14)
    )
  ),
  fp_p = fp_par(text.align = "center", padding = 5))

# Print the flextable
flex_table
```

Table 6. Output of all SNPs to test our code (header).

| SNP | P\_Value\_fam1 | P\_Value\_fam2 | P\_Value\_fam3 | P\_Value\_fam4 | P\_Value\_fam5 | P\_Value\_fam6 | Family\_Count | Combined\_P\_Value | Adjusted\_P\_Value |
| --- | --- | --- | --- | --- | --- | --- | --- | --- | --- |
| AX-579436016 |  |  |  |  |  | 1 | 1 | 1 | 1 |
| AX-579436089 |  |  | 0.164 |  | 0.7 |  | 2 | 0.363 | 1 |
| AX-579436125 |  |  |  |  | 0.002 |  | 1 | 0.002 | 1 |
| AX-579436149 |  | 0.355 | 0.14 |  |  |  | 2 | 0.199 | 1 |
| AX-579436243 | 7.59e-06 |  |  | 0.018 |  | 1 | 3 | 1.97e-05 | 1 |
| AX-579436298 |  | 1 | 1 |  |  | 1 | 3 | 1 | 1 |

Save the results in Excel format

```
# Specify the path and file name for the Excel file
excel_file <-
  here("output",
       "segregation",
       "albopictus",
       "combined_dt_all_SNPs.xlsx")

# Create an empty Excel workbook
wb <- openxlsx::createWorkbook()

# Create a worksheet in the workbook
openxlsx::addWorksheet(wb, sheetName = "Results")

# Write the data frame to the worksheet
openxlsx::writeData(wb, sheet = "Results", selected_df_2)

# Save the workbook as an Excel file
openxlsx::saveWorkbook(wb, file = excel_file, overwrite = TRUE)
```

We can check the range of the p-values

```
# Calculate the range for each column (excluding SNP)
column_ranges <-
  selected_df_2 |>
  dplyr::select(-SNP) |>
  reframe(
    across(
      .cols = everything(),
      .fns = list(
        range = ~ range(as.numeric(.), na.rm = TRUE)
      ),
      .names = "{.col}_{.fn}"
    )
  )

# Print the column ranges
print(column_ranges)
```

```
## # A tibble: 2 × 9
##   P_Value_fam1_range P_Value_fam2_range P_Value_fam3_range P_Value_fam4_range
##                <dbl>              <dbl>              <dbl>              <dbl>
## 1           8.05e-35           5.58e-17           1.82e-19           6.38e-26
## 2           1   e+ 0           1   e+ 0           1   e+ 0           1   e+ 0
## # ℹ 5 more variables: P_Value_fam5_range <dbl>, P_Value_fam6_range <dbl>,
## #   Family_Count_range <dbl>, Combined_P_Value_range <dbl>,
## #   Adjusted_P_Value_range <dbl>
```

We see values from 0 to 1. I created a format function to make zero
as 0 and not 00e+00, values from 0 to 0.001 will have scientific
notation, 0.001 to 1 will have 3 decimals (rounded).

Check how many SNPs we tested. Remember, each family has a different
set of SNPs. So, we end up testing more than 500 SNPs.

```
length(
  unique(
    selected_df_2$SNP
  )
)
```

```
## [1] 101376
```

The p-value is a measure of the likelihood that the observed
difference from expected occurred due to random chance. A higher p-value
suggests the frequencies could be randomly distributed, while a lower
p-value suggests non-random distribution. The conventional threshold to
decide whether a result is statistically significant is often set at
0.05.

In this case, you could consider those SNPs as randomly distributed
whose adjusted p-value is more than 0.05 in all the families where they
appear.

We can use the adjusted p values to filter out SNPs

Pass

```
randomly_distributed_SNPs3 <-
  selected_df_2 |>
  filter(Adjusted_P_Value > 0.05) |>
  pull(SNP) |>
  unique()

length(randomly_distributed_SNPs3)
```

```
## [1] 99313
```

Fail

```
segregation_errors_SNPs <-
  selected_df_2 |>
  filter(Adjusted_P_Value < 0.05) |>
  pull(SNP) |>
  unique()

length(segregation_errors_SNPs)
```

```
## [1] 2047
```

We tested 101,376 SNPs and 99,313 passed our test and 2,047 did not
pass (2.03% error rate).

### 12.5 Save the list of SNPs for futher genotype calls.

Write the list of SNPs that passed our test to file

```
# Create object
pass_segregation_SNPs <- unique(selected_df_2$SNP)

# Define the file path
file_path <- here("output",
                  "segregation",
                  "albopictus",
                  "albopictus_SNPs_passed_segregation.txt")

# Write unique SNPs to the file
writeLines(pass_segregation_SNPs, con = file_path)
```

Write the list of SNPs that did not our test to file

```
# Create object
fail_segregation_SNPs <- unique(segregation_errors_SNPs)

# Define the file path
file_path <- here("output",
                  "segregation",
                  "albopictus",
                  "albopictus_SNPs_fail_segregation.txt")

# Write unique SNPs to the file
writeLines(fail_segregation_SNPs, con = file_path)
```
