# Supplementary material for: A genotyping array for the globally invasive vector mosquito, Aedes albopictus
Source: Parasit Vectors. 2024 Mar 4;17:106. doi: 10.1186/s13071-024-06158-z (PMC10910840; doi:10.1186/s13071-024-06158-z)
Supplement: Supplementary file 4 — Additional file 4. Comparing genotypes of samples genotypes with WGS and chip. [file 13071_2024_6158_MOESM4_ESM.html]

 

 

 

 
 
 


 

 

 Aedes albopictus SNP chip - Comparying the genotypes of samples via WGS and the chip. 

 
 
 
 
 
 
 
 
 
 
 

 


 
 
 


 


 


 

 

 


 

 


 


 


 Aedes albopictus SNP chip - Comparying the
genotypes of samples via WGS and the chip. 
 Luciano V Cosme 
 2023-08-28 

 

 
 
  Comparison
of genotypes obtained by WGS and the chip  
  Analytic
approach 
 
  1. Load
libraries  
  2.
Import the chip data 
 
  2.1 Use Plink2 to convert
to bed format  
  
  2.2 Use R to update the .fam
file  
  3.
Import the WGS data  
  4. Prepare vcf files for
comparisons  
  5.
Pairwise comparions 
 
  5.1 Compare priors
to test code for comparisons 
 
  5.1.1 Allele
counts  
  5.1.2 Reference and
alternative alleles  
  
  5.2 Compare default prior and
WGS 
 
  5.2.1 Allele
counts  
  5.2.2 Reference and
alternative alleles  
  
  5.3 Compare default prior and
WGS 
 
  5.3.1 Allele
counts  
  5.3.2 Reference and
alternative alleles  
  
  
  6. Across samples comparisons 
 
  6.1 Total discrepancies
across all samples  
  6.2 Within
KAT and SAI  
  6.3 Discrepancies per sample  
  6.4 Save the data to load
later  
  6.5 SNPs with errors in 2
or more samples  
  6.6 Venn diagram fail
Mendel and mismatches  
  6.8 PCA before and after
removing SNPs  
  
  7. New genotype
calls for WGS data on the cluster 
 
  7.1 Batch
scripts  
  7.2 Convert tped to bed on
the cluster  
  7.3 Check and update SNP ids
for wgs  
  7.4
Set reference allele  
  
  8. Data sets for comparisons 
 
  8.1 Setting labels for each
data set  
  8.2 Merge data
sets  
  8.3 WGS calls with
different alternative alleles  
  
  9. Create vcf files for all
comparison 
 
  9.1
Create all the vcfs  
  9.2 Pairwise comparisons
summary  
  9.3
Script to process vcf files and function to import csv files  
  
  10. Chip
comparisons 
 
  10.1 “ab” -
Genotype calls using 18 versus 95 samples  
  10.2 “ac” -
Genotype calls using 18 versus 500 samples  
  10.3 “bc” -
Genotype calls using 95 versus 500 samples  
  
  11. WGS
comparsions 
 
  11.1 “xy”
Genotyping calls with 18 versus 30 samples  
  11.2 “ey”
Genotyping calls with 18 versus 800 samples  
  11.3 “wx”
Genotyping calls with 30 versus 800 samples  
  
  12. Chip and WGS comparisons 
 
  12.1 “ay” - WGS and
chip calls with 18 samples  
  12.2
“bx” - WGS call with 30 samples and chip call with 95 samples  
  12.3
“cw” - WGS call with 800 samples and chip call with 500 samples  
  
  13. Statistical comparisons  
  14. Check how
many samples a SNP might have errors 
 
  14.1 For chip
(ac)  
  
  15. Get allele counts from
cram files 
 
  15.1 Use Samtools to get
allele read counts  
  15.2 Parse the pileup files  
  15.2
Parse the csv files  
  15.3 Update SNP
ids  
  15.4 Check if there are
indels at SNP sites  
  15.5
Check correlation between low allele read count and genotype
mismatches  
  15.6 WGS
“xy” - genotyping calls with 18 versus 30 samples  
  15.7 WGS vs
chip “ay” - WGS and chip calls with 18 samples  
  15.8
Filtering based on variables correlated with mismatches  
  15.9
PCA with WGS and chip data sets before and after filtering 
 
  15.9.1
Venn diagram between SNPs with mismatches and segregation
errors  
  15.9.2 Venn diagram
fail Mendel and mismatches  
  15.9.3 PCA before and
after removing SNPs  
  
  
  16. Conclusion  
  
 
 

 
 Comparison of genotypes obtained by WGS and the chip 
 We used the DNA left over from the library prep for WGS of 18
samples. The DNA was extracted but not used entirely for the library
prep. Here we will compare the genotypes of sites that are shared
between the two data sets. For the genotyping using the chip, we used
the recommended priors and the new priors we obtained using the “SSTool”
from Thermo Fisher using the crosses. The WGS data was used to design
the probe sequences for the chip. However, we used 819 genomes to design
the chip, and here we will take into consideration only 18 samples. We
used ANGSD to perform the genotype calls for all 819 samples together
and here we are looking at only a few samples. Therefore, although the
comparison can help us identify problematic loci, we are cautious about
the accuracy of each technology. The average sequence depth for the WGS
across the 819 samples was 12X. However, it is variable from sample to
sample, and across the genome. Therefore, we cannot precisely tell if
the discrepancies in the genotypes between the technologies are due to
sequence depth, sequencing errors, or with the chip. We aim to gather a
general overview of loci with discrepancies in zygosity or genomic
regions with higher than expected genotype discordancies. 
 
 
 Analytic approach 
 
  Data Preparation 
 
 Set the reference allele to match the ‘AalbF3’ genome assembly for
both WGS and SNP chip data. 
 Convert the genotyping data into VCF format, making sure to maintain
consistency between the two datasets. 
  
  Pairwise Comparisons 
 
 Develop Python and/or R scripts to perform pairwise comparisons of
the genotypes from each sample across the two technologies. 
 Check the concordance between the two genotyping methods for each
sample pair. 
  
  Results Summarization 
 
 Compile the results of the pairwise comparisons into a
comprehensible format (e.g., a table or graph). 
 Calculate summary statistics that capture the level of agreement or
discrepancy between the two technologies (e.g., percent agreement, kappa
coefficient). 
  
  Threshold Identification 
 
 If discrepancies exist, investigate possible thresholds or cutoffs
that might explain the difference. 
 Examine the relationship between these thresholds and other
characteristics of the data (e.g., minor allele frequency, call
rate). 
  
  Interpretation 
 
 Draw conclusions about the relative performance of the two
genotyping technologies based on your findings. 
 Consider any implications these findings might have for future
research or clinical applications. 
  
  Correlation between chip and WGS variables 
 
 Identify variables associated with increase in mismatch rate between
the genotyping technologies. 
 Try different thresholds for the variables associated with high
mismatch rate 
  
  Data filtering and PCA 
 
 Once the variables are identified, try different thresholds for
perfecting overlap of points in a PCA when comparing WGS and Chip. 
  
 
 
 1. Load libraries 
       library (tidyverse) 
    library (here) 
    library (colorout) 
    library (flextable) 
    library (ggplot2) 
    library (scales) 
    library (reticulate) 
    library (extrafont) 
    library (stringr) 
    library (readr) 
    library (dplyr) 
    library (data.table) 
    library (scales) 
    library (ggrepel) 
    library (flextable) 
    library (forcats) 
    library (officer) 
    library (ggvenn) 
    library (RColorBrewer) 
    library (ggstatsplot) 
    library (broom)    
 Note about the general approach We have data of 18 samples from 2
populations genotyped with both technologies: 6 samples from Nepal (KAT)
and 12 samples from Trinidad and Tobago (SAI) - we did not have enough
DNA left after library prep for all samples 
 3 genotyping calls: WGS -> 800+ samples, 30 samples (KAT 12
samples and SAI 18 samples), and 18 samples (KAT 6 samples and SAI 12
samples) 
 Chip -> 500 samples, 95 samples (1 plate with the 18 samples and
other wild samples), and 18 samples (KAT 6 samples and SAI 12
samples) 
 Since the WGS calls took longer, part of the code is written
comparing default and new prior generated using the crosses. The aim is
illustrative and to develop the code while waiting for the WGS calls to
finish. It is not a good idea to use a prior from lab crosses in
genotype calls using wild animals. 
 
 
 2. Import the chip data 
 Check how many samples 
       # make sure you have all the .CEL samples in your family file - 152  
    bcftools  query  -l  data/raw_data/albo/wgs_vs_chip/wgs_default_prior_recommended_june_16_2023.vcf  |   wc   -l     
  ##       18  
 Check sample names 
       # make sure you have all the .CEL samples in your family file - 152  
    bcftools  query  -l  data/raw_data/albo/wgs_vs_chip/wgs_default_prior_recommended_june_16_2023.vcf  |   head     
  ## 601_Debug027_A12.CEL
## 604_Debug027_B1.CEL
## 605_Debug027_B2.CEL
## 606_Debug027_B3.CEL
## 607_Debug027_B4.CEL
## 608_Debug027_B5.CEL
## 611_Debug028_G10.CEL
## 612_Debug028_G11.CEL
## 613_Debug028_G7.CEL
## 614_Debug028_G8.CEL  
 
 2.1 Use Plink2 to convert to bed format 
 Create output directory 
       # Create main directory  
    dir.create ( 
      here ( "output" ,  "wgs_vs_chip" ), 
      showWarnings =   FALSE , 
      recursive =   FALSE  
   )    
 Convert ‘vcf’ file from Axiom suite to ‘bed’ format 
       # I created a fam file with the information about each sample, but first we import the data and create a bed file setting the family id constant  
    plink2   \  
   --allow-extra-chr  \  
   --vcf data/raw_data/albo/wgs_vs_chip/wgs_default_prior_recommended_june_16_2023.vcf  \  
   --const-fid  \  
   --make-bed  \  
   --fa data/genome/albo.fasta.gz  \  
   --ref-from-fa  'force'   `  # sets REF alleles when it can be done unambiguously, we use force to change the alleles  `   \  
   --out output/wgs_vs_chip/chip_dp_01  `  # dp - default priors  `   \  
   --silent ;  
    # --keep-allele-order \ if you use Plink 1.9  
    grep   "variants"  output/wgs_vs_chip/chip_dp_01.log  # to get the number of variants from the log file.     
  ## --vcf: 105607 variants scanned.
## 105607 variants loaded from output/wgs_vs_chip/chip_dp_01-temporary.pvar.zst.
## --ref-from-fa force: 0 variants changed, 105607 validated.  
 Using the default priors we obtained 105,607 SNPs. All the reference
alleles matched the reference genome (AalbF3). 
       # I created a fam file with the information about each sample, but first we import the data and create a bed file setting the family id constant  
    plink2   \  
   --allow-extra-chr  \  
   --vcf data/raw_data/albo/wgs_vs_chip/wgs_new_prior_recommended_june_16_2023.vcf  \  
   --const-fid  \  
   --make-bed  \  
   --fa data/genome/albo.fasta.gz  \  
   --ref-from-fa  'force'   `  # sets REF alleles when it can be done unambiguously, we use force to change the alleles  `   \  
   --out output/wgs_vs_chip/chip_np_01  `  # np - new priors  `   \  
   --silent ;  
    # --keep-allele-order \ if you use Plink 1.9  
    grep   "variants"  output/wgs_vs_chip/chip_np_01.log  # to get the number of variants from the log file.     
  ## --vcf: 118408 variants scanned.
## 118408 variants loaded from output/wgs_vs_chip/chip_np_01-temporary.pvar.zst.
## --ref-from-fa force: 0 variants changed, 118408 validated.  
 Using the new priors we obtained 118,408 SNPs. All the reference
alleles matched the reference genome (AalbF3). 
 Check the headings of the the files we will work on. 
       head   -n  5 output/wgs_vs_chip/chip_np_01.fam    
  ## 0    601_Debug027_A12.CEL    0   0   0   -9
## 0    604_Debug027_B1.CEL 0   0   0   -9
## 0    605_Debug027_B2.CEL 0   0   0   -9
## 0    606_Debug027_B3.CEL 0   0   0   -9
## 0    607_Debug027_B4.CEL 0   0   0   -9  
 We need to update the family information, individual id, and sex of
each individual. We can use the same file we use with the Axiom Suite to
update our .fam file. 
       head   -n  5 data/raw_data/albo/wgs_vs_chip/sample_ped_info.txt    
  ## Sample Filename  Family_ID   Individual_ID   Father_ID   Mother_ID   Sex Affection Status
## 608_Debug027_B5.CEL  KAT 12a 0   0   2   -9
## 616_Debug028_H10.CEL SAI 16a 0   0   2   -9
## 615_Debug028_G9.CEL  SAI 3a  0   0   2   -9
## 607_Debug027_B4.CEL  KAT 11a 0   0   2   -9  
 
 
 
 2.2 Use R to update the .fam file 
 Import the fam file we use with Axiom Suite 
       # the order of the rows in this file does not matter  
   samples  <-  
      read.delim ( 
        file   =   here ( 
          "data" , 
          "raw_data" , 
          "albo" , 
          "wgs_vs_chip" , 
          "sample_ped_info.txt"  
       ), 
        header =   TRUE  
     ) 
    head (samples)    
  ##        Sample.Filename Family_ID Individual_ID Father_ID Mother_ID Sex
## 1  608_Debug027_B5.CEL       KAT           12a         0         0   2
## 2 616_Debug028_H10.CEL       SAI           16a         0         0   2
## 3  615_Debug028_G9.CEL       SAI            3a         0         0   2
## 4  607_Debug027_B4.CEL       KAT           11a         0         0   2
## 5  606_Debug027_B3.CEL       KAT           10a         0         0   2
## 6  614_Debug028_G8.CEL       SAI            2a         0         0   2
##   Affection.Status
## 1               -9
## 2               -9
## 3               -9
## 4               -9
## 5               -9
## 6               -9  
 Import .fam file we created once we created the bed file using
Plink2 
       # The fam file is the same for both data sets with the default or new priors  
   fam1  <-  
      read.delim ( 
        file   =   here ( 
          "output" ,  "wgs_vs_chip" ,  "chip_dp_01.fam"  
       ), 
        header =   FALSE , 
        
     ) 
    head (fam1)    
  ##   V1                   V2 V3 V4 V5 V6
## 1  0 601_Debug027_A12.CEL  0  0  0 -9
## 2  0  604_Debug027_B1.CEL  0  0  0 -9
## 3  0  605_Debug027_B2.CEL  0  0  0 -9
## 4  0  606_Debug027_B3.CEL  0  0  0 -9
## 5  0  607_Debug027_B4.CEL  0  0  0 -9
## 6  0  608_Debug027_B5.CEL  0  0  0 -9  
 We can merge the tibbles. 
       # to keep the same order of the .fam file, we will first create an index based on the numbers of the samples, then use it too keep the order  
    
    # Extract the number part from the columns  
   fam1_temp  <-  fam1  |>  
      mutate ( num_id =   as.numeric ( str_extract (V2,  "^  \\  d+" ))) 
    
   samples_temp  <-  samples  |>  
      mutate ( num_id =   as.numeric ( str_extract (Sample.Filename,  "^  \\  d+" ))) 
    
    # Perform the left join using the num_id columns and keep the order of fam1  
   df  <-  fam1_temp  |>  
     dplyr ::  left_join (samples_temp,  by =   "num_id" )  |>  
     dplyr ::  select ( - num_id)  |>  
     dplyr ::  select ( 8  :  13 ) 
    
    # check the data frame  
    head (df)    
  ##   Family_ID Individual_ID Father_ID Mother_ID Sex Affection.Status
## 1       KAT            7a         0         0   2               -9
## 2       KAT            8a         0         0   2               -9
## 3       KAT            9a         0         0   2               -9
## 4       KAT           10a         0         0   2               -9
## 5       KAT           11a         0         0   2               -9
## 6       KAT           12a         0         0   2               -9  
 We can check how many samples we have in our file 
       nrow (df)    
  ## [1] 18  
 Before you save the new fam file, you can change the original file to
a different name, to compare the order later. If you want to repeat the
steps above after you saving the new file1.fam, you will need to import
the vcf again. 
       # Save and override the .fam file for dp  
    write.table ( 
     df, 
      file      =   here ( 
        "output" ,  "wgs_vs_chip" ,  "chip_dp_01.fam"  
     ), 
      sep       =   "  \t  " , 
      row.names =   FALSE , 
      col.names =   FALSE , 
      quote     =   FALSE  
   ) 
    
    # Save and override the .fam file for np  
    # Fist we need to change the sample ids  
   df $ Individual_ID  <-   gsub ( "a" ,  "b" , df $ Individual_ID) 
    # Save it  
    write.table ( 
     df, 
      file      =   here ( 
        "output" ,  "wgs_vs_chip" ,  "chip_np_01.fam"  
     ), 
      sep       =   "  \t  " , 
      row.names =   FALSE , 
      col.names =   FALSE , 
      quote     =   FALSE  
   )    
 Check the new .fam file to see if has the order and the sample
attributes we want. 
       # you can open the file on a text editor and double check the sample order and information.  
    head   -n  5 output/wgs_vs_chip/chip_dp_01.fam    
  ## KAT  7a  0   0   2   -9
## KAT  8a  0   0   2   -9
## KAT  9a  0   0   2   -9
## KAT  10a 0   0   2   -9
## KAT  11a 0   0   2   -9  
       # you can open the file on a text editor and double check the sample order and information.  
    head   -n  5 output/wgs_vs_chip/chip_np_01.fam    
  ## KAT  7b  0   0   2   -9
## KAT  8b  0   0   2   -9
## KAT  9b  0   0   2   -9
## KAT  10b 0   0   2   -9
## KAT  11b 0   0   2   -9  
 
 
 3. Import the WGS data 
 The WGS data is already in the ‘bed’ format, we can create a new bed
file and check if the reference alleles match the reference genome. 
       # We can create a new bed file and check if the reference and alternative alleles are set correctly  
    # I manually added "w" to the sample names after creating the file  
    plink2   \  
   --allow-extra-chr  \  
   --bfile data/raw_data/albo/wgs_vs_chip/wgs  \  
   --make-bed  \  
   --fa data/genome/albo.fasta.gz  \  
   --ref-from-fa  'force'   \  
   --out output/wgs_vs_chip/wgs_01  \  
   --silent ;  
    # --keep-allele-order \ if you use Plink 1.9  
    grep   "variants\|samples"  output/wgs_vs_chip/wgs_01.log    
  ## 18 samples (0 females, 0 males, 18 ambiguous; 18 founders) loaded from
## 175360 variants loaded from data/raw_data/albo/wgs_vs_chip/wgs.bim.
## --ref-from-fa force: 0 variants changed, 175360 validated.  
 Now we have some considerations to make about which strategy to
follow to do a pairwise comparison of the 18 samples: 
 
   Single VCF for Each Technology:  We can create
two multi-sample VCFs, one for each technology (sequencing and SNP
chip). This approach could make it easier to manage and manipulate your
data, especially if the number of variants detected by each technology
is different.  
   Single VCF for Each Sample:  Having a separate
VCF for each sample could be useful if we plan to do a lot of
sample-specific processing. However, it could become difficult to manage
if we had a large number of samples.  
 
 I will create a vcf for each sample setting the missingness to
zero. 
 
 
 4. Prepare vcf files for comparisons 
 Create output directory 
       # Create subdirectories for default and new priors. We can put the WGS vcfs in both.  
   subdirs  <-   c ( "vcfs" ) 
    
    for  (subdir  in  subdirs) { 
      dir.create ( here ( "output" ,  "wgs_vs_chip" , subdir),  showWarnings =   FALSE ) 
   }    
 We can merge the WGS and Chip data sets 
       # Create list of files to merge: wgs with chip with default prior  
    echo   'output/wgs_vs_chip/wgs_01  
    output/wgs_vs_chip/chip_dp_01  
    output/wgs_vs_chip/chip_np_01'   >  output/wgs_vs_chip/merge_list.txt    
 Merge the data (wgs and both chip data sets) 
       plink   \  
   --allow-extra-chr  \  
   --keep-allele-order  \  
   --merge-list output/wgs_vs_chip/merge_list.txt  \  
   --out output/wgs_vs_chip/wgs_chip  \  
   --silent 
    
    grep   "variants\|samples"  output/wgs_vs_chip/wgs_chip.log    
  ## Performing single-pass merge (54 people, 175388 variants).  
 Now we can subset the samples and keep the pairs that we are
interested in. 
  Code Explanation:  
 
  Variable Initialization: 
 
 The code defines a variable “input_file” with the value
“output/wgs_vs_chip/wgs_chip.fam”. 
 It defines a variable “output_dir” with the value
“output/wgs_vs_chip/vcfs”. 
 It defines a variable “bfile” with the value
“output/wgs_vs_chip/wgs_chip”. 
  
  Create Output Directory: 
 
 The code creates the output directory if it does not exist using the
following command: mkdir -p $output_dir 
  
  Retrieve Unique Families: 
 
 It retrieves the unique families from the input file specified by
“input_file”. 
 The “awk” command extracts the first column from the input
file. 
 The “sort” command sorts the extracted column. 
 The “uniq” command filters out duplicate entries. 
 The resulting unique families are stored in the “families”
variable. 
  
  Loop Over Families: 
 
 The code enters a loop over each family (“famid”) in the “families”
variable. 
  
  Retrieve Base Sample IDs: 
 
 Within the family loop, it retrieves the base sample IDs (without
‘a’, ‘b’, or ‘w’ suffixes) for the current family. 
 The “grep” command filters the input file based on the current
family. 
 The “awk” command extracts the second column (base sample IDs) from
the filtered lines. 
 The “sed” command removes the ‘a’, ‘b’, or ‘w’ suffixes from the
base sample IDs. 
 The “uniq” command filters out duplicate entries. 
 The resulting base sample IDs are stored in the “base_iids”
variable. 
  
  Nested Loop Over Base Sample IDs and Combinations: 
 
 The code enters another loop over each base sample ID (“base_iid”)
in the “base_iids” variable. 
 Within the base sample ID loop, it enters a nested loop over three
combinations: “aw”, “ab”, and “bw”. 
  
  Check Sample Existence: 
 
 For each combination, the code checks if both samples exist in the
input file. 
 It uses the “grep” command with regular expressions and the “-q”
option to suppress output. 
 If both samples exist, it proceeds with the following steps. 
  
  Create Temporary File: 
 
 It creates a temporary file using the “mktemp” command to store the
relevant lines from the input file. 
  
  Extract Relevant Lines: 
 
 The code uses the “grep” command to extract the lines from the input
file that match the family, base sample ID, and current
combination. 
 The matching lines are appended to the temporary file. 
  
  Execute plink2: 
 
 It executes the “plink2” command with various options and arguments
to perform specific operations on the data. 
 The command performs tasks such as allowing extra chromosomes,
preserving allele order, using the specified binary file (“bfile”),
applying filters, and specifying the output format. 
 The output is saved to a VCF file with a name based on the family,
base sample ID, and combination. 
 The “–silent” option suppresses unnecessary output. 
  
  Remove Temporary File: 
 
 After executing “plink2”, it removes the temporary file using the
“rm” command. 
  
  Continuation of Nested Loops: 
 
 The code continues the nested loops until all combinations and base
sample IDs have been processed. 
  
 
       input_file  =  "output/wgs_vs_chip/wgs_chip.fam"  
    output_dir  =  "output/wgs_vs_chip/vcfs"  
    bfile  =  "output/wgs_vs_chip/wgs_chip"  
    
    # create the output directory if it does not exist  
    mkdir   -p   $output_dir  
    
    # get unique families  
    families  =  $(  awk   '{print $1}'   $input_file   |   sort   |   uniq  )  
    
    for  famid  in   $families  ;   do  
      # get the base sample ids (without a, b, w)  
      base_iids  =  $(  grep   "  $famid  "   $input_file   |   awk   '{print $2}'   |   sed   's/[abw]$//'   |   uniq  )  
      
      for  base_iid  in   $base_iids  ;   do  
        for  combination  in   "aw"   "ab"   "bw"  ;   do  
          # Check if both samples exist  
          if   grep   -qE   "  ${famid}  \s  ${base_iid}  [  ${combination  :  0  :  1  }  ]\s"   "  $input_file  "   &&   
             grep   -qE   "  ${famid}  \s  ${base_iid}  [  ${combination  :  1  :  1  }  ]\s"   "  $input_file  "  ;   then  
            # Create temporary file  
            tmp_file  =  $(  mktemp  )  
            grep   -E   "  ${famid}  \s  ${base_iid}  [  ${combination  :  0  :  1  }  ]\s"   "  $input_file  "   >   "  $tmp_file  "  
            grep   -E   "  ${famid}  \s  ${base_iid}  [  ${combination  :  1  :  1  }  ]\s"   "  $input_file  "   >>   "  $tmp_file  "  
      
            # Execute plink2  
            plink2   \  
            --allow-extra-chr   \  
            --keep-allele-order   \  
            --bfile   $bfile   \  
            --keep   "  $tmp_file  "   \  
            --recode  vcf-iid  \  
            --geno  0  \  
            --out   "  $output_dir  /  ${famid}  _  ${base_iid}${combination}  "   \  
            --silent  
      
            # Remove temporary file  
            rm   "  $tmp_file  "  
          fi  
        done  
      done  
    done     
 Check how many SNPs per vcf 
       # Define directory with the vcfs  
    output_dir  =  "output/wgs_vs_chip/vcfs"  
    # Count how many SNPs we have in each vcf file  
    for  file  in   ${output_dir} / * .vcf ;   do  
        echo   $(  basename   $file) :  $(  grep   -v   '^#'   $file   |   wc   -l  )  
    done     
  ## KAT_10ab.vcf: 88082
## KAT_10aw.vcf: 103266
## KAT_10bw.vcf: 112299
## KAT_11ab.vcf: 87696
## KAT_11aw.vcf: 102966
## KAT_11bw.vcf: 111933
## KAT_12ab.vcf: 87242
## KAT_12aw.vcf: 102463
## KAT_12bw.vcf: 110802
## KAT_7ab.vcf: 88070
## KAT_7aw.vcf: 103231
## KAT_7bw.vcf: 112281
## KAT_8ab.vcf: 87510
## KAT_8aw.vcf: 102794
## KAT_8bw.vcf: 111420
## KAT_9ab.vcf: 87797
## KAT_9aw.vcf: 103062
## KAT_9bw.vcf: 111759
## SAI_12ab.vcf: 87428
## SAI_12aw.vcf: 102797
## SAI_12bw.vcf: 112888
## SAI_13ab.vcf: 87351
## SAI_13aw.vcf: 102716
## SAI_13bw.vcf: 112654
## SAI_14ab.vcf: 87155
## SAI_14aw.vcf: 102598
## SAI_14bw.vcf: 112582
## SAI_15ab.vcf: 87550
## SAI_15aw.vcf: 102946
## SAI_15bw.vcf: 113085
## SAI_16ab.vcf: 87591
## SAI_16aw.vcf: 102931
## SAI_16bw.vcf: 113002
## SAI_17ab.vcf: 87443
## SAI_17aw.vcf: 102744
## SAI_17bw.vcf: 112943
## SAI_18ab.vcf: 87646
## SAI_18aw.vcf: 103116
## SAI_18bw.vcf: 113466
## SAI_1ab.vcf: 87267
## SAI_1aw.vcf: 102757
## SAI_1bw.vcf: 112835
## SAI_2ab.vcf: 87376
## SAI_2aw.vcf: 102681
## SAI_2bw.vcf: 112683
## SAI_3ab.vcf: 87600
## SAI_3aw.vcf: 102966
## SAI_3bw.vcf: 112968
## SAI_4ab.vcf: 87492
## SAI_4aw.vcf: 102970
## SAI_4bw.vcf: 113414
## SAI_5ab.vcf: 87469
## SAI_5aw.vcf: 102862
## SAI_5bw.vcf: 112823  
 Check sample names to see if our code created the vcfs with two
samples 
       # Define directory with the VCFs  
    output_dir  =  "output/wgs_vs_chip/vcfs"  
    
    # Iterate over each VCF file  
    for  file  in   "  ${output_dir}  " / * .vcf ;   do  
        # Extract the file name without the directory path  
        file_name  =  $(  basename   "  ${file}  "  )  
    
        # Use bcftools query to retrieve the sample names  
        sample_names  =  $(  bcftools  query  -l   "  ${file}  "  )  
        
        # Print the file name and the sample names  
        echo   "  ${file_name}  :   ${sample_names}  "  
    done     
  ## KAT_10ab.vcf: 10a
## 10b
## KAT_10aw.vcf: 10a
## 10w
## KAT_10bw.vcf: 10b
## 10w
## KAT_11ab.vcf: 11a
## 11b
## KAT_11aw.vcf: 11a
## 11w
## KAT_11bw.vcf: 11b
## 11w
## KAT_12ab.vcf: 12a
## 12b
## KAT_12aw.vcf: 12a
## 12w
## KAT_12bw.vcf: 12b
## 12w
## KAT_7ab.vcf: 7a
## 7b
## KAT_7aw.vcf: 7a
## 7w
## KAT_7bw.vcf: 7b
## 7w
## KAT_8ab.vcf: 8a
## 8b
## KAT_8aw.vcf: 8a
## 8w
## KAT_8bw.vcf: 8b
## 8w
## KAT_9ab.vcf: 9a
## 9b
## KAT_9aw.vcf: 9a
## 9w
## KAT_9bw.vcf: 9b
## 9w
## SAI_12ab.vcf: 12a
## 12b
## SAI_12aw.vcf: 12a
## 12w
## SAI_12bw.vcf: 12b
## 12w
## SAI_13ab.vcf: 13a
## 13b
## SAI_13aw.vcf: 13a
## 13w
## SAI_13bw.vcf: 13b
## 13w
## SAI_14ab.vcf: 14a
## 14b
## SAI_14aw.vcf: 14a
## 14w
## SAI_14bw.vcf: 14b
## 14w
## SAI_15ab.vcf: 15a
## 15b
## SAI_15aw.vcf: 15a
## 15w
## SAI_15bw.vcf: 15b
## 15w
## SAI_16ab.vcf: 16a
## 16b
## SAI_16aw.vcf: 16a
## 16w
## SAI_16bw.vcf: 16b
## 16w
## SAI_17ab.vcf: 17a
## 17b
## SAI_17aw.vcf: 17a
## 17w
## SAI_17bw.vcf: 17b
## 17w
## SAI_18ab.vcf: 18a
## 18b
## SAI_18aw.vcf: 18a
## 18w
## SAI_18bw.vcf: 18b
## 18w
## SAI_1ab.vcf: 1a
## 1b
## SAI_1aw.vcf: 1a
## 1w
## SAI_1bw.vcf: 1b
## 1w
## SAI_2ab.vcf: 2a
## 2b
## SAI_2aw.vcf: 2a
## 2w
## SAI_2bw.vcf: 2b
## 2w
## SAI_3ab.vcf: 3a
## 3b
## SAI_3aw.vcf: 3a
## 3w
## SAI_3bw.vcf: 3b
## 3w
## SAI_4ab.vcf: 4a
## 4b
## SAI_4aw.vcf: 4a
## 4w
## SAI_4bw.vcf: 4b
## 4w
## SAI_5ab.vcf: 5a
## 5b
## SAI_5aw.vcf: 5a
## 5w
## SAI_5bw.vcf: 5b
## 5w  
 Create new directories 
       # Create main directory  
    dir.create ( 
      here ( "output" ,  "wgs_vs_chip" ,  "scripts" ), 
      showWarnings =   FALSE , 
      recursive =   FALSE  
   )    
 Script to compare alleles between wgs and chip or chip priors 
 Code summary: The provided code performs the following steps: 
 
   Import the necessary libraries  The code imports
the required libraries: “allel”, “pandas”, “os”, and “numpy”.  
   Create an empty DataFrame  The code initializes
an empty DataFrame called “output_df” to store the output results
obtained from the analysis.  
   Specify the directory  The code defines the
directory path where the VCF files are located using the “dir_name”
variable.  
   Retrieve a list of VCF files  The code uses the
“os.listdir()” function and list comprehension to create a list of all
VCF files in the specified directory that end with ‘.vcf’.  
   Iterate over each VCF file  The code sets up a
loop to iterate over each VCF file found in the previous step.  
   Construct the file path  The code constructs the
full file path for the current VCF file by combining the directory path
and the file name using “os.path.join()”.  
   Read the VCF file  The code reads the VCF file
using “allel.read_vcf()” from the “allel” library, specifying to load
all available fields (’*’).  
   Extract the genotype data  The code extracts the
genotype data from the VCF file using
“allel.GenotypeArray(callset[‘calldata/GT’])”.  
   Check sample count  The code verifies if the VCF
file contains two samples by checking the shape of the genotype array
using the “assert” statement. If the shape doesn’t match the expected
number of samples, an assertion error is raised.  
   Count total SNPs  The code determines the total
number of SNPs in the genotype data by calculating the length of the
genotype array using “len(gt)”.  
   Calculate counts of homozygous and heterozygous
SNPs  The code uses “np.count_nonzero()” and relevant methods of
the “gt” object to count the number of homozygous reference, homozygous
alternate, and heterozygous SNPs for each sample.  
   Compute counts of mismatched homozygous and heterozygous
SNPs  The code compares the genotypes between the two samples
using “np.sum()” to calculate the counts of mismatched homozygous
reference, homozygous alternate, and heterozygous SNPs.  
   Extract reference and alternative alleles  The
code retrieves the reference and alternative alleles for each SNP from
the VCF file.  
   Count mismatching reference and alternative
alleles  The code compares the alleles between the two samples
and counts the number of SNPs with mismatching reference alleles and the
number of SNPs with mismatching alternative alleles.  
   Calculate counts of A, T, C, and G alleles  The
code computes the counts of A, T, C, and G alleles for each sample based
on the genotype data and the corresponding reference and alternative
alleles.  
   Create and append result to output dataframe  The
code creates a DataFrame called “result” to store the calculated
statistics for the current VCF file and appends it to the “output_df”
DataFrame using “pd.concat()”.  
   Repeat for each VCF file  The code repeats steps
5 to 16 for each VCF file in the directory, processing and appending the
results to the “output_df” DataFrame.  
   Write the output to a CSV file  The code writes
the final “output_df” DataFrame to a CSV file named
‘allele_comparison_stats_2.csv’ using the “to_csv()” method of
pandas.  
 
       import  allel 
    import  pandas  as  pd 
    import  os 
    import  numpy  as  np 
    
    # Initialize the output dataframe  
   output_df  =  pd.DataFrame() 
    
    # Directory with vcf files  
   dir_name  =   "output/wgs_vs_chip/vcfs/"  
    
    # Get list of all vcf files in the directory  
   vcf_files  =  [f  for  f  in  os.listdir(dir_name)  if  f.endswith( '.vcf' )] 
    
    # Iterate over VCF files  
    for  vcf_file  in  vcf_files: 
       file_path  =  os.path.join(dir_name, vcf_file) 
       callset  =  allel.read_vcf(file_path, fields = [ '*' ]) 
    
        # Get genotype  
       gt  =  allel.GenotypeArray(callset[ 'calldata/GT' ]) 
        
        # Verify the vcf contains two samples  
        assert  gt.shape[ 1 ]  ==   2 ,  f"Expected 2 samples in   { vcf_file }  , found   { gt . shape[ 1 ] }  "  
    
        # Count SNPs  
       n_snps  =   len (gt) 
    
        # Count homozygous and heterozygous SNPs for each sample  
       n_homo_ref  =  np.count_nonzero(gt.is_hom_ref(), axis =  0 ) 
       n_homo_alt  =  np.count_nonzero(gt.is_hom_alt(), axis =  0 ) 
       n_hetero  =  np.count_nonzero(gt.is_het(), axis =  0 ) 
        
        # Count homozygous and heterozygous SNPs mismatches  
       n_homo_ref_mismatch  =  np. sum (gt.is_hom_ref()[:,  0 ]  !=  gt.is_hom_ref()[:,  1 ]) 
       n_homo_alt_mismatch  =  np. sum (gt.is_hom_alt()[:,  0 ]  !=  gt.is_hom_alt()[:,  1 ]) 
       n_hetero_mismatch  =  np. sum (gt.is_het()[:,  0 ]  !=  gt.is_het()[:,  1 ]) 
    
        # Get alleles  
       ref_alleles  =  callset[ 'variants/REF' ] 
       alt_alleles  =  callset[ 'variants/ALT' ][:,  0 ]   # assuming bi-allelic  
    
        # Count mismatching reference and alternative alleles  
       n_snps_ref_mismatch  =  np.count_nonzero(ref_alleles[gt[:, 0 ]]  !=  ref_alleles[gt[:, 1 ]]) 
       n_snps_alt_mismatch  =  np.count_nonzero(alt_alleles[gt[:, 0 ]]  !=  alt_alleles[gt[:, 1 ]]) 
    
        # Count alleles for each sample  
       n_a  =   sum (np.count_nonzero(gt  ==  i, axis =  0 )  for  i  in   range ( 4 )  if  ref_alleles[i]  ==   'A'   or  alt_alleles[i]  ==   'A' ) 
       n_t  =   sum (np.count_nonzero(gt  ==  i, axis =  0 )  for  i  in   range ( 4 )  if  ref_alleles[i]  ==   'T'   or  alt_alleles[i]  ==   'T' ) 
       n_c  =   sum (np.count_nonzero(gt  ==  i, axis =  0 )  for  i  in   range ( 4 )  if  ref_alleles[i]  ==   'C'   or  alt_alleles[i]  ==   'C' ) 
       n_g  =   sum (np.count_nonzero(gt  ==  i, axis =  0 )  for  i  in   range ( 4 )  if  ref_alleles[i]  ==   'G'   or  alt_alleles[i]  ==   'G' ) 
    
        # Append results to the output dataframe  
       result  =  pd.DataFrame({ 
            'vcf_file' : [file_path], 
            'n_SNPs' : [n_snps], 
            'n_SNPs_ref_mismatch' : [n_snps_ref_mismatch], 
            'n_SNPs_alt_mismatch' : [n_snps_alt_mismatch], 
            'n_A' : [n_a], 
            'n_T' : [n_t], 
            'n_C' : [n_c], 
            'n_G' : [n_g], 
            'n_homo_ref' : [n_homo_ref], 
            'n_homo_alt' : [n_homo_alt], 
            'n_hetero' : [n_hetero], 
            'n_homo_ref_mismatch' : [n_homo_ref_mismatch], 
            'n_homo_alt_mismatch' : [n_homo_alt_mismatch], 
            'n_hetero_mismatch' : [n_hetero_mismatch] 
       }) 
    
       output_df  =  pd.concat([output_df, result]) 
    
    # Write the result to a csv file  
   output_df.to_csv( 'output/wgs_vs_chip/allele_comparison_stats_2.csv' , index =  False )    
 Clean env 
       # python  
    py_run_string ( "import gc; gc.collect()" )    
 Import the data 
      data  <-  
      read_delim ( 
        "output/wgs_vs_chip/allele_comparison_stats_2.csv" , 
        delim =   "," , 
        show_col_types =   FALSE  
     ) 
    
   data  <-  
     data  |>  
      mutate ( vcf_file =   str_remove (vcf_file,  "output/wgs_vs_chip/vcfs/" ))  |>  
      separate ( 
       vcf_file, 
        into =   c ( "Population" ,  "Sample_Comparison" ), 
        sep =   "_" , 
        extra =   "drop"  
     )  |>  
      separate ( 
       Sample_Comparison, 
        into =   c ( "Sample" ,  "Comparison" ), 
        sep =   "(?<=  \\  d)(?=[a-z])" , 
        convert =   TRUE  
     )  |>  
      mutate ( Comparison =   str_remove (Comparison,  ".vcf" ))  |>  
      arrange (Comparison) 
    
    # Split the "Comparison" column into "Sample1" and "Sample2"  
   data  <-   
     data  |>  
      separate ( 
       Comparison, 
        into =   c ( "Sample1" ,  "Sample2" ), 
        sep =   1 , 
        # because each comparison has two characters  
        remove =   FALSE  
     )  |>   # keep the original comparison column  
      relocate (Sample1, Sample2,  .after =  Comparison)  # move the new columns right after Comparison  
    
   cols_to_split  <-  
      c ( "n_A" , 
        "n_T" , 
        "n_C" , 
        "n_G" , 
        "n_homo_ref" , 
        "n_homo_alt" , 
        "n_hetero" ) 
    
    # Remove unwanted characters from the columns  
    for  (col_name  in  cols_to_split) { 
     data[[col_name]]  <-   gsub ( "  \\  [  \\  [|]  \\  n" ,  "" , data[[col_name]]) 
   } 
    
    # Split the columns  
    for  (col_name  in  cols_to_split) { 
      # Create new column names based on 'Sample1' and 'Sample2'  
     new_col_names  <-   paste0 (col_name,  "_sample" ,  1  :  2 ) 
      
     data  <-  data  |>  
        separate ( 
          col =  col_name, 
          into =  new_col_names, 
          sep =   " " , 
          extra =   "drop"  
       ) 
   } 
    
    # Clean the new columns  
   cols_to_clean  <-   
      grep ( "^n_" ,  names (data),  value =   TRUE ) 
    
    for  (col_name  in  cols_to_clean) { 
      # Remove unwanted characters '[', ']', and '\n'  
     data[[col_name]]  <-   gsub ( "  \\  [|]|  \\  n" ,  "" , data[[col_name]]) 
   } 
    
    # Split the column names into "Sample" and numeric value  
   data  <-   
     data  |>  
      separate ( 
        col =  Comparison, 
        into =   c ( "Sample1" ,  "Sample2" ), 
        sep =   1 , 
        remove =   FALSE  
     )  |>  
      relocate (Sample1, Sample2,  .after =  Comparison) 
    
    # Convert columns to numeric  
    # Specify the column names to convert to numeric  
   columns_to_convert  <-  
      c ( 
        # "Population",  
        "Sample" , 
        # "Comparison",  
        # "Sample1",  
        # "Sample2",  
        "n_SNPs" , 
        "n_SNPs_ref_mismatch" , 
        "n_SNPs_alt_mismatch" , 
        "n_A_sample1" , 
        "n_A_sample2" , 
        "n_T_sample1" , 
        "n_T_sample2" , 
        "n_C_sample1" , 
        "n_C_sample2" , 
        "n_G_sample1" , 
        "n_G_sample2" , 
        "n_homo_ref_sample1" , 
        "n_homo_ref_sample2" , 
        "n_homo_alt_sample1" , 
        "n_homo_alt_sample2" , 
        "n_hetero_sample1" , 
        "n_hetero_sample2" , 
        "n_homo_ref_mismatch" , 
        "n_homo_alt_mismatch" , 
        "n_hetero_mismatch"  
     ) 
    
    # Convert columns to numeric  
   data[columns_to_convert]  <-  
      lapply (data[columns_to_convert],  function (x) 
        as.numeric ( as.character (x))) 
    
    # Verify the column types  
    print ( sapply (data[columns_to_convert], class))    
  ##              Sample              n_SNPs n_SNPs_ref_mismatch n_SNPs_alt_mismatch 
##           "numeric"           "numeric"           "numeric"           "numeric" 
##         n_A_sample1         n_A_sample2         n_T_sample1         n_T_sample2 
##           "numeric"           "numeric"           "numeric"           "numeric" 
##         n_C_sample1         n_C_sample2         n_G_sample1         n_G_sample2 
##           "numeric"           "numeric"           "numeric"           "numeric" 
##  n_homo_ref_sample1  n_homo_ref_sample2  n_homo_alt_sample1  n_homo_alt_sample2 
##           "numeric"           "numeric"           "numeric"           "numeric" 
##    n_hetero_sample1    n_hetero_sample2 n_homo_ref_mismatch n_homo_alt_mismatch 
##           "numeric"           "numeric"           "numeric"           "numeric" 
##   n_hetero_mismatch 
##           "numeric"  
 Now we can subset the data to have more meaningful comparisons and
visualizations. 
 
 
 5. Pairwise comparions 
 First we can compare the priors to see if it is reasonable to
generate new priors using the SSTool. I am doing this first because the
genotype calls for the WGS data are still running. We can test our code
and later we use it to look at the comparisons of interest. I do not
think that the new prior generated with the crosses data should work
since the population have been in the lab for several generations and we
are using the priors with wild animals. 
 
 5.1 Compare priors to test code for comparisons 
 I create new priors using the SSToll from ThermoFisher and the
crosses data. We can compare the genotype calls using each priors. We
need to do some data tyding first. 
       # Filter rows containing "ab" in column "Comparison"  
   priors  <-  
     data  |>  
      filter ( 
       Comparison  ==   "ab"  
     ) 
    
    # The default priors is represented as "a" (Sample1) and the new priors are represented as "b" (Sample2)  
    
    # Change column names  
    colnames (priors)  <-   gsub ( "sample1" ,  "default_prior" ,  colnames (priors)) 
    colnames (priors)  <-   gsub ( "sample2" ,  "new_prior" ,  colnames (priors)) 
    
    # Verify the updated column names  
    print ( colnames (priors))    
  ##  [1] "Population"               "Sample"                  
##  [3] "Comparison"               "Sample1"                 
##  [5] "Sample2"                  "n_SNPs"                  
##  [7] "n_SNPs_ref_mismatch"      "n_SNPs_alt_mismatch"     
##  [9] "n_A_default_prior"        "n_A_new_prior"           
## [11] "n_T_default_prior"        "n_T_new_prior"           
## [13] "n_C_default_prior"        "n_C_new_prior"           
## [15] "n_G_default_prior"        "n_G_new_prior"           
## [17] "n_homo_ref_default_prior" "n_homo_ref_new_prior"    
## [19] "n_homo_alt_default_prior" "n_homo_alt_new_prior"    
## [21] "n_hetero_default_prior"   "n_hetero_new_prior"      
## [23] "n_homo_ref_mismatch"      "n_homo_alt_mismatch"     
## [25] "n_hetero_mismatch"  
 Sanity check 
       # Add a new column named allele_totals to sum n_A_new_prior, n_T_new_prior, n_C_new_prior, and n_G_new_prior  
   priors  <-  
     priors  |>  
      mutate ( 
        allele_total_new =  n_A_new_prior  +  n_T_new_prior  +  n_C_new_prior  +  n_G_new_prior, 
        allele_total_default =  n_A_default_prior  +  n_T_default_prior  +  n_C_default_prior  +  n_G_default_prior 
     ) 
    
    # Compare the allele totals with the number of SNPs  
    head (priors  |>  
     dplyr ::  select (Population, Sample, n_SNPs, allele_total_new, allele_total_default))    
  ## # A tibble: 6 × 5
##   Population Sample n_SNPs allele_total_new allele_total_default
##   <chr>       <dbl>  <dbl>            <dbl>                <dbl>
## 1 KAT            11  87696           175392               175392
## 2 KAT             9  87797           175594               175594
## 3 SAI            12  87428           174856               174856
## 4 SAI            16  87591           175182               175182
## 5 SAI            14  87155           174310               174310
## 6 KAT            12  87242           174484               174484  
 The sum of A, T, C and G is twice as the number of SNPs because we
have two samples in each comparison. Therefore, we need to divide by 2
when calculating the differences in allele counts. 
 
 5.1.1 Allele counts 
       # we can calculate how many counts of each allele (A, T, C and G) we have for each prior. Lets do difference = New - default prior  
   priors_allele_count  <-  
     priors  |>  
     dplyr ::  select ( 
       Population, 
       Sample, 
       n_SNPs, 
       n_A_default_prior, 
       n_A_new_prior, 
       n_T_default_prior, 
       n_T_new_prior, 
       n_C_default_prior, 
       n_C_new_prior, 
       n_G_default_prior, 
       n_G_new_prior, 
     )  |>  
      mutate ( 
        n_A_diff =  (n_A_new_prior  /   2   -  n_A_default_prior  /   2 ), 
        n_T_diff =  (n_T_new_prior  /   2   -  n_T_default_prior  /   2 ), 
        n_C_diff =  (n_C_new_prior  /   2   -  n_C_default_prior  /   2 ), 
        n_G_diff =  (n_G_new_prior  /   2   -  n_G_default_prior  /   2 ) 
     )  |>  
     dplyr ::  select (Population, 
                   Sample, 
                   n_SNPs, 
                   n_A_diff, 
                   n_T_diff, 
                   n_C_diff, 
                   n_G_diff)  |>  
      arrange (Population, Sample)  |>  
      mutate ( 
        n_A_diff =   paste0 ( 
          formatC ( 
           n_A_diff, 
            big.mark =   "," , 
            format =   "f" , 
            digits =   0  
         ), 
          " (" , 
          round ((n_A_diff  /  n_SNPs)  *   100 ,  2 ), 
          "%)"  
       ), 
        n_T_diff =   paste0 ( 
          formatC ( 
           n_T_diff, 
            big.mark =   "," , 
            format =   "f" , 
            digits =   0  
         ), 
          " (" , 
          round ((n_T_diff  /  n_SNPs)  *   100 ,  2 ), 
          "%)"  
       ), 
        n_C_diff =   paste0 ( 
          formatC ( 
           n_C_diff, 
            big.mark =   "," , 
            format =   "f" , 
            digits =   0  
         ), 
          " (" , 
          round ((n_C_diff  /  n_SNPs)  *   100 ,  2 ), 
          "%)"  
       ), 
        n_G_diff =   paste0 ( 
          formatC ( 
           n_G_diff, 
            big.mark =   "," , 
            format =   "f" , 
            digits =   0  
         ), 
          " (" , 
          round ((n_G_diff  /  n_SNPs)  *   100 ,  2 ), 
          "%)"  
       ) 
     )  |>  
      relocate (n_C_diff,  .after =  n_A_diff)  # move the new columns right after n_A_diff  
    
    # Convert head(results) to a tibble  
   table_result  <-  
      as_tibble (priors_allele_count) 
    
    # Set theme if you want to use something different from the previous table  
    set_flextable_defaults ( 
      font.family =   "Arial" , 
      font.size =   9 , 
      big.mark =   "," , 
      theme_fun =   "theme_zebra"   # try the themes: theme_alafoli(), theme_apa(), theme_booktabs(), theme_box(), theme_tron_legacy(), theme_tron(), theme_vader(), theme_vanilla(), theme_zebra()  
   ) 
    
    # Then create the flextable object  
   flex_table  <-  
      flextable (table_result)  |>  
      set_caption ( caption =   as_paragraph ( 
        as_chunk ( 
          "Table 1. Differences between the default and new priors from the crosses obtained using the SSTool." , 
          props =   fp_text_default ( color =   "#000000" ,  font.size =   14 ) 
       ) 
     ), 
      fp_p =   fp_par ( text.align =   "center" ,  padding =   5 )) 
    
   flex_table    
     Table 1. Differences between the default and new priors from the crosses obtained using the SSTool.       Population      Sample      n_SNPs      n_A_diff      n_C_diff      n_T_diff      n_G_diff          KAT      7      88,070      5,343 (6.07%)      -5,343 (-6.07%)      0 (0%)      0 (0%)        KAT      8      87,510      6,162 (7.04%)      -6,162 (-7.04%)      0 (0%)      0 (0%)        KAT      9      87,797      4,838 (5.51%)      -4,838 (-5.51%)      0 (0%)      0 (0%)        KAT      10      88,082      5,141 (5.84%)      -5,141 (-5.84%)      0 (0%)      0 (0%)        KAT      11      87,696      6,703 (7.64%)      -6,703 (-7.64%)      0 (0%)      0 (0%)        KAT      12      87,242      4,926 (5.65%)      -4,926 (-5.65%)      0 (0%)      0 (0%)        SAI      1      87,267      10,592 (12.14%)      -10,592 (-12.14%)      0 (0%)      0 (0%)        SAI      2      87,376      -10,104 (-11.56%)      10,104 (11.56%)      -10,104 (-11.56%)      10,104 (11.56%)        SAI      3      87,600      9,602 (10.96%)      -9,602 (-10.96%)      0 (0%)      0 (0%)        SAI      4      87,492      10,586 (12.1%)      -10,586 (-12.1%)      0 (0%)      0 (0%)        SAI      5      87,469      10,018 (11.45%)      -10,018 (-11.45%)      0 (0%)      0 (0%)        SAI      12      87,428      9,953 (11.38%)      -9,953 (-11.38%)      0 (0%)      0 (0%)        SAI      13      87,351      9,996 (11.44%)      -9,996 (-11.44%)      0 (0%)      0 (0%)        SAI      14      87,155      10,676 (12.25%)      -10,676 (-12.25%)      0 (0%)      0 (0%)        SAI      15      87,550      10,196 (11.65%)      -10,196 (-11.65%)      0 (0%)      0 (0%)        SAI      16      87,591      9,513 (10.86%)      -9,513 (-10.86%)      0 (0%)      0 (0%)        SAI      17      87,443      9,590 (10.97%)      -9,590 (-10.97%)      0 (0%)      0 (0%)        SAI      18      87,646      10,398 (11.86%)      -10,398 (-11.86%)      0 (0%)      0 (0%)       
 The main difference of the genotypes obtained from the different
priors are the transversions of A and C. T. The problem might be from
the fact we used priors from the crosses. What I can do is to run a
genotype call with the entire plate that has the samples we are
comparing and generate priors for them. The SSTool requires at least 1
plate to generate new priors and we have only 18 samples. I will do that
and add it to the comparisons we need to do. 
 
 
 5.1.2 Reference and alternative alleles 
 Lets do a sanity check and count how many homozygous and heterozygous
we have 
       # Add a new column named allele_totals to sum n_A_new_prior, n_T_new_prior, n_C_new_prior, and n_G_new_prior  
   priors  <-  
     priors  |>  
      mutate ( 
        n_hom_het_default =   rowSums ( 
          cbind ( 
           n_homo_ref_default_prior, 
           n_homo_alt_default_prior, 
           n_hetero_default_prior 
         ), 
          na.rm =   TRUE  
       ), 
        n_hom_het_new =   rowSums ( 
          cbind ( 
           n_homo_ref_new_prior, 
           n_homo_alt_new_prior, 
           n_hetero_new_prior 
         ), 
          na.rm =   TRUE  
       ) 
     ) 
    
    # Compare the allele totals with the number of SNPs  
    head (priors  |>  
     dplyr ::  select (Population, Sample, n_SNPs, n_hom_het_default, n_hom_het_new))    
  ## # A tibble: 6 × 5
##   Population Sample n_SNPs n_hom_het_default n_hom_het_new
##   <chr>       <dbl>  <dbl>             <dbl>         <dbl>
## 1 KAT            11  87696             87696         87696
## 2 KAT             9  87797             78120         87391
## 3 SAI            12  87428             87428         87428
## 4 SAI            16  87591             87591         87591
## 5 SAI            14  87155             87155         87155
## 6 KAT            12  87242             77390         86829  
 The total number of SNPs match the sum of homozygous and
heterozygous, so we do not have to divide by 2 as we did for the sum of
alleles 
       # we can select only one of the column since it is biallelic data  
   priors_ref_alt  <-  
     priors  |>  
     dplyr ::  select ( 
       Population, 
       Sample, 
       n_SNPs, 
       n_SNPs_ref_mismatch, 
       n_SNPs_alt_mismatch, 
       n_homo_ref_default_prior, 
       n_homo_ref_new_prior, 
       n_homo_ref_mismatch, 
       n_homo_alt_default_prior, 
       n_homo_alt_new_prior, 
       n_homo_alt_mismatch, 
       n_hetero_default_prior, 
       n_hetero_new_prior, 
       n_hetero_mismatch 
     )  |>  
      arrange ( 
       Population, Sample 
     ) 
    
    # We can select or rename columns to make our table easier to understand. We can create new columns since the alt and ref allele counts are the same because the alleles are swapped when we use the new priors.  
    
    # Get the number of SNPs with the alleles swapped. Remember, for 2 mosquitoes with 10 SNPs we have 40 alleles. When we want to calculate the percentages based on the number of SNPs, we need to divided the values by 2 (two samples)  
   priors_ref_alt  <-  
     priors_ref_alt  |>  
      mutate ( 
        alleles_swapped =  n_SNPs_ref_mismatch, 
        hom_ref_diff =  n_homo_ref_mismatch, 
        hom_ref_alt =  n_homo_alt_mismatch, 
        het_diff =  n_hetero_mismatch 
     )  |>  
     dplyr ::  select (Population, 
                   Sample, 
                   n_SNPs, 
                   alleles_swapped, 
                   hom_ref_diff, 
                   hom_ref_alt, 
                   het_diff)  |>  
      mutate ( 
        alleles_swapped =   paste0 ( 
          formatC (alleles_swapped,  big.mark =   "," ,  format =   "d" ), 
          " (" , 
          round ((alleles_swapped  /  n_SNPs)  *   100 ,  2 ), 
          "%)"  
       ), 
        hom_ref_diff =   paste0 ( 
          formatC (hom_ref_diff,  big.mark =   "," ,  format =   "d" ), 
          " (" , 
          round ((hom_ref_diff  /  n_SNPs)  *   100 ,  2 ), 
          "%)"  
       ), 
        hom_ref_alt =   paste0 ( 
          formatC (hom_ref_alt,  big.mark =   "," ,  format =   "d" ), 
          " (" , 
          round ((hom_ref_alt  /  n_SNPs)  *   100 ,  2 ), 
          "%)"  
       ), 
        het_diff =   paste0 ( 
          formatC (het_diff,  big.mark =   "," ,  format =   "d" ), 
          " (" , 
          round ((het_diff  /  n_SNPs)  *   100 ,  2 ), 
          "%)"  
       ) 
     ) 
    
    # Convert head(results) to a tibble  
   table_result  <-  
      as_tibble (priors_ref_alt) 
    
    # Set theme if you want to use something different from the previous table  
    set_flextable_defaults ( 
      font.family =   "Arial" , 
      font.size =   9 , 
      big.mark =   "," , 
      theme_fun =   "theme_zebra"   # try the themes: theme_alafoli(), theme_apa(), theme_booktabs(), theme_box(), theme_tron_legacy(), theme_tron(), theme_vader(), theme_vanilla(), theme_zebra()  
   ) 
    
    # Then create the flextable object  
   flex_table  <-  
      flextable (table_result)  |>  
      set_caption ( caption =   as_paragraph ( 
        as_chunk ( 
          "Table 2. Number of alleles with alleles swapped and differences in zygosity when default and new priors of the crosses." , 
          props =   fp_text_default ( color =   "#000000" ,  font.size =   14 ) 
       ) 
     ), 
      fp_p =   fp_par ( text.align =   "center" ,  padding =   5 )) 
    
    # Print the flextable  
   flex_table    
     Table 2. Number of alleles with alleles swapped and differences in zygosity when default and new priors of the crosses.       Population      Sample      n_SNPs      alleles_swapped      hom_ref_diff      hom_ref_alt      het_diff          KAT      7      88,070      1,500 (1.7%)      718 (0.82%)      782 (0.89%)      1,486 (1.69%)        KAT      8      87,510      1,573 (1.8%)      724 (0.83%)      849 (0.97%)      1,565 (1.79%)        KAT      9      87,797      1,500 (1.71%)      682 (0.78%)      818 (0.93%)      1,486 (1.69%)        KAT      10      88,082      1,417 (1.61%)      659 (0.75%)      758 (0.86%)      1,411 (1.6%)        KAT      11      87,696      1,559 (1.78%)      651 (0.74%)      908 (1.04%)      1,559 (1.78%)        KAT      12      87,242      1,655 (1.9%)      789 (0.9%)      866 (0.99%)      1,649 (1.89%)        SAI      1      87,267      1,702 (1.95%)      650 (0.74%)      1,052 (1.21%)      1,696 (1.94%)        SAI      2      87,376      1,696 (1.94%)      700 (0.8%)      996 (1.14%)      1,692 (1.94%)        SAI      3      87,600      1,530 (1.75%)      638 (0.73%)      892 (1.02%)      1,522 (1.74%)        SAI      4      87,492      1,727 (1.97%)      654 (0.75%)      1,073 (1.23%)      1,719 (1.96%)        SAI      5      87,469      1,592 (1.82%)      678 (0.78%)      914 (1.04%)      1,584 (1.81%)        SAI      12      87,428      1,628 (1.86%)      656 (0.75%)      972 (1.11%)      1,618 (1.85%)        SAI      13      87,351      1,651 (1.89%)      695 (0.8%)      956 (1.09%)      1,643 (1.88%)        SAI      14      87,155      1,761 (2.02%)      739 (0.85%)      1,022 (1.17%)      1,751 (2.01%)        SAI      15      87,550      1,589 (1.81%)      641 (0.73%)      948 (1.08%)      1,585 (1.81%)        SAI      16      87,591      1,639 (1.87%)      673 (0.77%)      966 (1.1%)      1,623 (1.85%)        SAI      17      87,443      1,577 (1.8%)      679 (0.78%)      898 (1.03%)      1,563 (1.79%)        SAI      18      87,646      1,589 (1.81%)      641 (0.73%)      948 (1.08%)      1,579 (1.8%)       
 
 
 
 5.2 Compare default prior and WGS 
 I create new priors using the SSToll from ThermoFisher and the
crosses data. We can compare the genotype calls using each priors. We
need to do some data tidying first. 
       # Filter rows containing "ab" in column "Comparison"  
   default_wgs  <-  
     data  |>  
      filter ( 
       Comparison  ==   "aw"  
     ) 
    
    # The default priors is represented as "a" (Sample1) and the new priors are represented as "b" (Sample2)  
    
    # Change column names  
    colnames (default_wgs)  <-   gsub ( "sample1" ,  "default_prior" ,  colnames (default_wgs)) 
    colnames (default_wgs)  <-   gsub ( "sample2" ,  "wgs" ,  colnames (default_wgs)) 
    
    # Verify the updated column names  
    print ( colnames (default_wgs))    
  ##  [1] "Population"               "Sample"                  
##  [3] "Comparison"               "Sample1"                 
##  [5] "Sample2"                  "n_SNPs"                  
##  [7] "n_SNPs_ref_mismatch"      "n_SNPs_alt_mismatch"     
##  [9] "n_A_default_prior"        "n_A_wgs"                 
## [11] "n_T_default_prior"        "n_T_wgs"                 
## [13] "n_C_default_prior"        "n_C_wgs"                 
## [15] "n_G_default_prior"        "n_G_wgs"                 
## [17] "n_homo_ref_default_prior" "n_homo_ref_wgs"          
## [19] "n_homo_alt_default_prior" "n_homo_alt_wgs"          
## [21] "n_hetero_default_prior"   "n_hetero_wgs"            
## [23] "n_homo_ref_mismatch"      "n_homo_alt_mismatch"     
## [25] "n_hetero_mismatch"  
 
 5.2.1 Allele counts 
       # we can calculate how many counts of each allele (A, T, C and G)  
   priors_allele_count_dw  <-  
     default_wgs  |>  
     dplyr ::  select ( 
       Population, 
       Sample, 
       n_SNPs, 
       n_A_default_prior, 
       n_A_wgs, 
       n_T_default_prior, 
       n_T_wgs, 
       n_C_default_prior, 
       n_C_wgs, 
       n_G_default_prior, 
       n_G_wgs, 
     )  |>  
      mutate ( 
        n_A_diff =  (n_A_wgs  /   2   -  n_A_default_prior  /   2 ), 
        n_T_diff =  (n_T_wgs  /   2   -  n_T_default_prior  /   2 ), 
        n_C_diff =  (n_C_wgs  /   2   -  n_C_default_prior  /   2 ), 
        n_G_diff =  (n_G_wgs  /   2   -  n_G_default_prior  /   2 ) 
     )  |>  
     dplyr ::  select (Population, 
                   Sample, 
                   n_SNPs, 
                   n_A_diff, 
                   n_T_diff, 
                   n_C_diff, 
                   n_G_diff)  |>  
      arrange (Population, Sample)  |>  
      mutate ( 
        n_A_diff =   paste0 ( 
          formatC ( 
           n_A_diff, 
            big.mark =   "," , 
            format =   "f" , 
            digits =   0  
         ), 
          " (" , 
          round ((n_A_diff  /  n_SNPs)  *   100 ,  2 ), 
          "%)"  
       ), 
        n_T_diff =   paste0 ( 
          formatC ( 
           n_T_diff, 
            big.mark =   "," , 
            format =   "f" , 
            digits =   0  
         ), 
          " (" , 
          round ((n_T_diff  /  n_SNPs)  *   100 ,  2 ), 
          "%)"  
       ), 
        n_C_diff =   paste0 ( 
          formatC ( 
           n_C_diff, 
            big.mark =   "," , 
            format =   "f" , 
            digits =   0  
         ), 
          " (" , 
          round ((n_C_diff  /  n_SNPs)  *   100 ,  2 ), 
          "%)"  
       ), 
        n_G_diff =   paste0 ( 
          formatC ( 
           n_G_diff, 
            big.mark =   "," , 
            format =   "f" , 
            digits =   0  
         ), 
          " (" , 
          round ((n_G_diff  /  n_SNPs)  *   100 ,  2 ), 
          "%)"  
       ) 
     )  |>  
      relocate (n_C_diff,  .after =  n_A_diff)  # move the new columns right after n_A_diff  
    
    # Convert head(results) to a tibble  
   table_result  <-  
      as_tibble (priors_allele_count_dw) 
    
    # Set theme if you want to use something different from the previous table  
    set_flextable_defaults ( 
      font.family =   "Arial" , 
      font.size =   9 , 
      big.mark =   "," , 
      theme_fun =   "theme_zebra"   # try the themes: theme_alafoli(), theme_apa(), theme_booktabs(), theme_box(), theme_tron_legacy(), theme_tron(), theme_vader(), theme_vanilla(), theme_zebra()  
   ) 
    
    # Then create the flextable object  
   flex_table  <-  
      flextable (table_result)  |>  
      set_caption ( caption =   as_paragraph ( 
        as_chunk ( 
          "Table 3. Differences between the default prior from the WGS data." , 
          props =   fp_text_default ( color =   "#000000" ,  font.size =   14 ) 
       ) 
     ), 
      fp_p =   fp_par ( text.align =   "center" ,  padding =   5 )) 
    
    # Print the flextable  
   flex_table    
     Table 3. Differences between the default prior from the WGS data.       Population      Sample      n_SNPs      n_A_diff      n_C_diff      n_T_diff      n_G_diff          KAT      7      103,231      6,742 (6.53%)      -6,742 (-6.53%)      0 (0%)      0 (0%)        KAT      8      102,794      7,750 (7.54%)      -7,750 (-7.54%)      0 (0%)      0 (0%)        KAT      9      103,062      6,186 (6%)      -6,186 (-6%)      0 (0%)      0 (0%)        KAT      10      103,266      6,574 (6.37%)      -6,574 (-6.37%)      0 (0%)      0 (0%)        KAT      11      102,966      8,372 (8.13%)      -8,372 (-8.13%)      0 (0%)      0 (0%)        KAT      12      102,463      6,241 (6.09%)      -6,241 (-6.09%)      0 (0%)      0 (0%)        SAI      1      102,757      12,930 (12.58%)      -12,930 (-12.58%)      0 (0%)      0 (0%)        SAI      2      102,681      0 (0%)      0 (0%)      -12,286 (-11.97%)      12,286 (11.97%)        SAI      3      102,966      11,771 (11.43%)      -11,771 (-11.43%)      0 (0%)      0 (0%)        SAI      4      102,970      12,924 (12.55%)      -12,924 (-12.55%)      0 (0%)      0 (0%)        SAI      5      102,862      12,219 (11.88%)      -12,219 (-11.88%)      0 (0%)      0 (0%)        SAI      12      102,797      12,152 (11.82%)      -12,152 (-11.82%)      0 (0%)      0 (0%)        SAI      13      102,716      12,126 (11.8%)      -12,126 (-11.8%)      0 (0%)      0 (0%)        SAI      14      102,598      13,013 (12.68%)      -13,013 (-12.68%)      0 (0%)      0 (0%)        SAI      15      102,946      12,514 (12.16%)      -12,514 (-12.16%)      0 (0%)      0 (0%)        SAI      16      102,931      11,548 (11.22%)      -11,548 (-11.22%)      0 (0%)      0 (0%)        SAI      17      102,744      11,670 (11.36%)      -11,670 (-11.36%)      0 (0%)      0 (0%)        SAI      18      103,116      12,732 (12.35%)      -12,732 (-12.35%)      0 (0%)      0 (0%)       
 The main difference of the genotypes obtained from the different
priors are the transversions of A and C. T. The problem might be from
the fact we used priors from the crosses. What I can do is to run a
genotype call with the entire plate that has the samples we are
comparing and generate priors for them. The SSTool requires at least 1
plate to generate new priors and we have only 18 samples. I will do that
and add it to the comparisons we need to do. 
 
 
 5.2.2 Reference and alternative alleles 
       # we can select only one of the column since it is biallelic data  
   priors_ref_alt_dw  <-  
     default_wgs  |>  
     dplyr ::  select ( 
       Population, 
       Sample, 
       n_SNPs, 
       n_SNPs_ref_mismatch, 
       n_SNPs_alt_mismatch, 
       n_homo_ref_default_prior, 
       n_homo_ref_wgs, 
       n_homo_ref_mismatch, 
       n_homo_alt_default_prior, 
       n_homo_alt_wgs, 
       n_homo_alt_mismatch, 
       n_hetero_default_prior, 
       n_hetero_wgs, 
       n_hetero_mismatch 
     )  |>  
      arrange ( 
       Population, Sample 
     ) 
    
    # We can select or rename columns to make our table easier to understand. We can create new columns since the alt and ref allele counts are the same because the alleles are swapped when we use the new priors.  
    # Set the display format to avoid scientific notation  
    options ( scipen =   999 ) 
    
    # Get the number of SNPs with the alleles swapped  
   priors_ref_alt_dw  <-  
     priors_ref_alt_dw  |>  
      mutate ( 
        alleles_swapped =  n_SNPs_ref_mismatch, 
        hom_ref_diff =  n_homo_ref_mismatch, 
        hom_ref_alt =  n_homo_alt_mismatch, 
        het_diff =  n_hetero_mismatch 
     )  |>  
     dplyr ::  select (Population, 
                   Sample, 
                   n_SNPs, 
                   alleles_swapped, 
                   hom_ref_diff, 
                   hom_ref_alt, 
                   het_diff)  |>  
      mutate ( 
        alleles_swapped =   paste0 ( 
          formatC (alleles_swapped,  big.mark =   "," ,  format =   "d" ), 
          " (" , 
          round ((alleles_swapped  /  n_SNPs)  *   100 ,  2 ), 
          "%)"  
       ), 
        hom_ref_diff =   paste0 ( 
          formatC (hom_ref_diff,  big.mark =   "," ,  format =   "d" ), 
          " (" , 
          round ((hom_ref_diff  /  n_SNPs)  *   100 ,  2 ), 
          "%)"  
       ), 
        hom_ref_alt =   paste0 ( 
          formatC (hom_ref_alt,  big.mark =   "," ,  format =   "d" ), 
          " (" , 
          round ((hom_ref_alt  /  n_SNPs)  *   100 ,  2 ), 
          "%)"  
       ), 
        het_diff =   paste0 ( 
          formatC (het_diff,  big.mark =   "," ,  format =   "d" ), 
          " (" , 
          round ((het_diff  /  n_SNPs)  *   100 ,  2 ), 
          "%)"  
       ) 
     ) 
    
    # Convert head(results) to a tibble  
   table_result  <-  
      as_tibble (priors_ref_alt_dw) 
    
    # Set theme if you want to use something different from the previous table  
    set_flextable_defaults ( 
      font.family =   "Arial" , 
      font.size =   9 , 
      big.mark =   "," , 
      theme_fun =   "theme_zebra"   # try the themes: theme_alafoli(), theme_apa(), theme_booktabs(), theme_box(), theme_tron_legacy(), theme_tron(), theme_vader(), theme_vanilla(), theme_zebra()  
   ) 
    
    # Then create the flextable object  
   flex_table  <-  
      flextable (table_result)  |>  
      set_caption ( caption =   as_paragraph ( 
        as_chunk ( 
          "Table 4. SNPs with alleles swapped and differences in zygosity comparing the default prior and WGS data." , 
          props =   fp_text_default ( color =   "#000000" ,  font.size =   14 ) 
       ) 
     ), 
      fp_p =   fp_par ( text.align =   "center" ,  padding =   5 )) 
    
    # Print the flextable  
   flex_table    
     Table 4. SNPs with alleles swapped and differences in zygosity comparing the default prior and WGS data.       Population      Sample      n_SNPs      alleles_swapped      hom_ref_diff      hom_ref_alt      het_diff          KAT      7      103,231      8,537 (8.27%)      4,785 (4.64%)      3,752 (3.63%)      6,051 (5.86%)        KAT      8      102,794      9,023 (8.78%)      4,959 (4.82%)      4,064 (3.95%)      6,539 (6.36%)        KAT      9      103,062      7,891 (7.66%)      4,438 (4.31%)      3,453 (3.35%)      5,555 (5.39%)        KAT      10      103,266      8,573 (8.3%)      4,844 (4.69%)      3,729 (3.61%)      6,025 (5.83%)        KAT      11      102,966      9,184 (8.92%)      5,055 (4.91%)      4,129 (4.01%)      6,864 (6.67%)        KAT      12      102,463      8,444 (8.24%)      4,741 (4.63%)      3,703 (3.61%)      5,742 (5.6%)        SAI      1      102,757      11,541 (11.23%)      6,258 (6.09%)      5,283 (5.14%)      9,839 (9.58%)        SAI      2      102,681      11,628 (11.32%)      6,322 (6.16%)      5,306 (5.17%)      9,788 (9.53%)        SAI      3      102,966      13,355 (12.97%)      7,493 (7.28%)      5,862 (5.69%)      10,475 (10.17%)        SAI      4      102,970      14,416 (14%)      8,084 (7.85%)      6,332 (6.15%)      11,322 (11%)        SAI      5      102,862      15,141 (14.72%)      8,644 (8.4%)      6,497 (6.32%)      11,563 (11.24%)        SAI      12      102,797      10,711 (10.42%)      5,842 (5.68%)      4,869 (4.74%)      9,165 (8.92%)        SAI      13      102,716      12,642 (12.31%)      6,909 (6.73%)      5,733 (5.58%)      10,204 (9.93%)        SAI      14      102,598      12,376 (12.06%)      6,829 (6.66%)      5,547 (5.41%)      10,502 (10.24%)        SAI      15      102,946      10,326 (10.03%)      5,519 (5.36%)      4,807 (4.67%)      9,042 (8.78%)        SAI      16      102,931      10,644 (10.34%)      5,822 (5.66%)      4,822 (4.68%)      8,882 (8.63%)        SAI      17      102,744      13,389 (13.03%)      7,579 (7.38%)      5,810 (5.65%)      10,367 (10.09%)        SAI      18      103,116      12,630 (12.25%)      7,045 (6.83%)      5,585 (5.42%)      10,270 (9.96%)       
       # Reset the display format to the default  
    options ( scipen =   0 )    
 
 
 
 5.3 Compare default prior and WGS 
 I create new priors using the SSToll from ThermoFisher and the
crosses data. We can compare the genotype calls using each priors. We
need to do some data tidying first. 
       # Filter rows containing "ab" in column "Comparison"  
   cross_prior_wgs  <-  
     data  |>  
      filter ( 
       Comparison  ==   "bw"  
     ) 
    
    # The default priors is represented as "a" (Sample1) and the new priors are represented as "b" (Sample2)  
    
    # Change column names  
    colnames (cross_prior_wgs)  <-   gsub ( "sample1" ,  "cross_prior" ,  colnames (cross_prior_wgs)) 
    colnames (cross_prior_wgs)  <-   gsub ( "sample2" ,  "wgs" ,  colnames (cross_prior_wgs)) 
    
    # Verify the updated column names  
    print ( colnames (cross_prior_wgs))    
  ##  [1] "Population"             "Sample"                 "Comparison"            
##  [4] "Sample1"                "Sample2"                "n_SNPs"                
##  [7] "n_SNPs_ref_mismatch"    "n_SNPs_alt_mismatch"    "n_A_cross_prior"       
## [10] "n_A_wgs"                "n_T_cross_prior"        "n_T_wgs"               
## [13] "n_C_cross_prior"        "n_C_wgs"                "n_G_cross_prior"       
## [16] "n_G_wgs"                "n_homo_ref_cross_prior" "n_homo_ref_wgs"        
## [19] "n_homo_alt_cross_prior" "n_homo_alt_wgs"         "n_hetero_cross_prior"  
## [22] "n_hetero_wgs"           "n_homo_ref_mismatch"    "n_homo_alt_mismatch"   
## [25] "n_hetero_mismatch"  
 
 5.3.1 Allele counts 
       # we can calculate how many counts of each allele (A, T, C and G)  
   priors_allele_count_nw  <-  
     cross_prior_wgs  |>  
     dplyr ::  select ( 
       Population, 
       Sample, 
       n_SNPs, 
       n_A_cross_prior, 
       n_A_wgs, 
       n_T_cross_prior, 
       n_T_wgs, 
       n_C_cross_prior, 
       n_C_wgs, 
       n_G_cross_prior, 
       n_G_wgs, 
     )  |>  
      mutate ( 
        n_A_diff =  (n_A_wgs  /   2   -  n_A_cross_prior  /   2 ), 
        n_T_diff =  (n_T_wgs  /   2   -  n_T_cross_prior  /   2 ), 
        n_C_diff =  (n_C_wgs  /   2   -  n_C_cross_prior  /   2 ), 
        n_G_diff =  (n_G_wgs  /   2   -  n_G_cross_prior  /   2 ) 
     )  |>  
     dplyr ::  select (Population, 
                   Sample, 
                   n_SNPs, 
                   n_A_diff, 
                   n_T_diff, 
                   n_C_diff, 
                   n_G_diff)  |>  
      arrange (Population, Sample)  |>  
      mutate ( 
        n_A_diff =   paste0 ( 
          formatC ( 
           n_A_diff, 
            big.mark =   "," , 
            format =   "f" , 
            digits =   0  
         ), 
          " (" , 
          round ((n_A_diff  /  n_SNPs)  *   100 ,  2 ), 
          "%)"  
       ), 
        n_T_diff =   paste0 ( 
          formatC ( 
           n_T_diff, 
            big.mark =   "," , 
            format =   "f" , 
            digits =   0  
         ), 
          " (" , 
          round ((n_T_diff  /  n_SNPs)  *   100 ,  2 ), 
          "%)"  
       ), 
        n_C_diff =   paste0 ( 
          formatC ( 
           n_C_diff, 
            big.mark =   "," , 
            format =   "f" , 
            digits =   0  
         ), 
          " (" , 
          round ((n_C_diff  /  n_SNPs)  *   100 ,  2 ), 
          "%)"  
       ), 
        n_G_diff =   paste0 ( 
          formatC ( 
           n_G_diff, 
            big.mark =   "," , 
            format =   "f" , 
            digits =   0  
         ), 
          " (" , 
          round ((n_G_diff  /  n_SNPs)  *   100 ,  2 ), 
          "%)"  
       ) 
     )  |>  
      relocate (n_C_diff,  .after =  n_A_diff)  # move the new columns right after n_A_diff  
    
    # Convert head(results) to a tibble  
   table_result  <-  
      as_tibble (priors_allele_count_nw) 
    
    # Set theme if you want to use something different from the previous table  
    set_flextable_defaults ( 
      font.family =   "Arial" , 
      font.size =   9 , 
      big.mark =   "," , 
      theme_fun =   "theme_zebra"   # try the themes: theme_alafoli(), theme_apa(), theme_booktabs(), theme_box(), theme_tron_legacy(), theme_tron(), theme_vader(), theme_vanilla(), theme_zebra()  
   ) 
    
    # Then create the flextable object  
   flex_table  <-  
      flextable (table_result)  |>  
      set_caption ( caption =   as_paragraph ( 
        as_chunk ( 
          "Table 5. Allele count differences between the crosses' prior from the WGS data." , 
          props =   fp_text_default ( color =   "#000000" ,  font.size =   14 ) 
       ) 
     ), 
      fp_p =   fp_par ( text.align =   "center" ,  padding =   5 )) 
    
    # Print the flextable  
   flex_table    
     Table 5. Allele count differences between the crosses' prior from the WGS data.       Population      Sample      n_SNPs      n_A_diff      n_C_diff      n_T_diff      n_G_diff          KAT      7      112,281      6,634 (5.91%)      -6,634 (-5.91%)      -6,634 (-5.91%)      6,634 (5.91%)        KAT      8      111,420      7,564 (6.79%)      -7,564 (-6.79%)      -7,564 (-6.79%)      7,564 (6.79%)        KAT      9      111,759      6,236 (5.58%)      -6,236 (-5.58%)      -6,236 (-5.58%)      6,236 (5.58%)        KAT      10      112,299      6,488 (5.78%)      -6,488 (-5.78%)      -6,488 (-5.78%)      6,488 (5.78%)        KAT      11      111,933      8,256 (7.38%)      -8,256 (-7.38%)      -8,256 (-7.38%)      8,256 (7.38%)        KAT      12      110,802      6,302 (5.69%)      -6,302 (-5.69%)      -6,302 (-5.69%)      6,302 (5.69%)        SAI      1      112,835      12,710 (11.26%)      -12,710 (-11.26%)      -12,710 (-11.26%)      12,710 (11.26%)        SAI      2      112,683      12,124 (10.76%)      -12,124 (-10.76%)      -12,124 (-10.76%)      12,124 (10.76%)        SAI      3      112,968      11,580 (10.25%)      -11,580 (-10.25%)      -11,580 (-10.25%)      11,580 (10.25%)        SAI      4      113,414      12,744 (11.24%)      -12,744 (-11.24%)      -12,744 (-11.24%)      12,744 (11.24%)        SAI      5      112,823      11,893 (10.54%)      -11,893 (-10.54%)      -11,893 (-10.54%)      11,893 (10.54%)        SAI      12      112,888      11,938 (10.57%)      -11,938 (-10.57%)      -11,938 (-10.57%)      11,938 (10.57%)        SAI      13      112,654      11,978 (10.63%)      -11,978 (-10.63%)      -11,978 (-10.63%)      11,978 (10.63%)        SAI      14      112,582      12,753 (11.33%)      -12,753 (-11.33%)      -12,753 (-11.33%)      12,753 (11.33%)        SAI      15      113,085      12,135 (10.73%)      -12,135 (-10.73%)      -12,135 (-10.73%)      12,135 (10.73%)        SAI      16      113,002      11,524 (10.2%)      -11,524 (-10.2%)      -11,524 (-10.2%)      11,524 (10.2%)        SAI      17      112,943      11,556 (10.23%)      -11,556 (-10.23%)      -11,556 (-10.23%)      11,556 (10.23%)        SAI      18      113,466      12,483 (11%)      -12,483 (-11%)      -12,483 (-11%)      12,483 (11%)       
 The main difference of the genotypes obtained from the different
priors are the transversions of A and C. T. The problem might be from
the fact we used priors from the crosses. What I can do is to run a
genotype call with the entire plate that has the samples we are
comparing and generate priors for them. The SSTool requires at least 1
plate to generate new priors and we have only 18 samples. I will do that
and add it to the comparisons we need to do. 
 
 
 5.3.2 Reference and alternative alleles 
       # we can select only one of the column since it is biallelic data  
   priors_ref_alt_nw  <-  
     cross_prior_wgs  |>  
     dplyr ::  select ( 
       Population, 
       Sample, 
       n_SNPs, 
       n_SNPs_ref_mismatch, 
       n_SNPs_alt_mismatch, 
       n_homo_ref_cross_prior, 
       n_homo_ref_wgs, 
       n_homo_ref_mismatch, 
       n_homo_alt_cross_prior, 
       n_homo_alt_wgs, 
       n_homo_alt_mismatch, 
       n_hetero_cross_prior, 
       n_hetero_wgs, 
       n_hetero_mismatch 
     )  |>  
      arrange ( 
       Population, Sample 
     ) 
    
    # We can select or rename columns to make our table easier to understand. We can create new columns since the alt and ref allele counts are the same because the alleles are swapped when we use the new priors.  
    # Set the display format to avoid scientific notation  
    options ( scipen =   999 ) 
    
    # Get the number of SNPs with the alleles swapped  
   priors_ref_alt_nw  <-  
     priors_ref_alt_nw  |>  
      mutate ( 
        alleles_swapped =  n_SNPs_ref_mismatch  /   2 , 
        hom_ref_diff =  n_homo_ref_mismatch  /   2 , 
        hom_ref_alt =  n_homo_alt_mismatch  /   2 , 
        het_diff =  n_hetero_mismatch  /   2  
     )  |>  
     dplyr ::  select (Population, 
                   Sample, 
                   n_SNPs, 
                   alleles_swapped, 
                   hom_ref_diff, 
                   hom_ref_alt, 
                   het_diff)  |>  
      mutate ( 
        alleles_swapped =   paste0 ( 
          formatC (alleles_swapped,  big.mark =   "," ,  format =   "d" ), 
          " (" , 
          round ((alleles_swapped  /  n_SNPs)  *   100 ,  2 ), 
          "%)"  
       ), 
        hom_ref_diff =   paste0 ( 
          formatC (hom_ref_diff,  big.mark =   "," ,  format =   "d" ), 
          " (" , 
          round ((hom_ref_diff  /  n_SNPs)  *   100 ,  2 ), 
          "%)"  
       ), 
        hom_ref_alt =   paste0 ( 
          formatC (hom_ref_alt,  big.mark =   "," ,  format =   "d" ), 
          " (" , 
          round ((hom_ref_alt  /  n_SNPs)  *   100 ,  2 ), 
          "%)"  
       ), 
        het_diff =   paste0 ( 
          formatC (het_diff,  big.mark =   "," ,  format =   "d" ), 
          " (" , 
          round ((het_diff  /  n_SNPs)  *   100 ,  2 ), 
          "%)"  
       ) 
     ) 
    
    # Convert head(results) to a tibble  
   table_result  <-  
      as_tibble (priors_ref_alt_nw) 
    
    # Set theme if you want to use something different from the previous table  
    set_flextable_defaults ( 
      font.family =   "Arial" , 
      font.size =   9 , 
      big.mark =   "," , 
      theme_fun =   "theme_zebra"   # try the themes: theme_alafoli(), theme_apa(), theme_booktabs(), theme_box(), theme_tron_legacy(), theme_tron(), theme_vader(), theme_vanilla(), theme_zebra()  
   ) 
    
    # Then create the flextable object  
   flex_table  <-  
      flextable (table_result)  |>  
      set_caption ( caption =   as_paragraph ( 
        as_chunk ( 
          "Table 6. SNPs with alleles swapped and differences in zygosity comparing crosses prior and WGS data." , 
          props =   fp_text_default ( color =   "#000000" ,  font.size =   14 ) 
       ) 
     ), 
      fp_p =   fp_par ( text.align =   "center" ,  padding =   5 )) 
    
    # Print the flextable  
   flex_table    
     Table 6. SNPs with alleles swapped and differences in zygosity comparing crosses prior and WGS data.       Population      Sample      n_SNPs      alleles_swapped      hom_ref_diff      hom_ref_alt      het_diff          KAT      7      112,281      6,608 (5.89%)      3,690 (3.29%)      2,918 (2.6%)      4,225 (3.76%)        KAT      8      111,420      6,810 (6.11%)      3,782 (3.39%)      3,027 (2.72%)      4,463 (4.01%)        KAT      9      111,759      6,221 (5.57%)      3,447 (3.08%)      2,773 (2.48%)      3,924 (3.51%)        KAT      10      112,299      6,602 (5.88%)      3,678 (3.28%)      2,924 (2.6%)      4,184 (3.73%)        KAT      11      111,933      6,866 (6.13%)      3,824 (3.42%)      3,042 (2.72%)      4,693 (4.19%)        KAT      12      110,802      6,499 (5.87%)      3,603 (3.25%)      2,895 (2.61%)      4,002 (3.61%)        SAI      1      112,835      8,107 (7.19%)      4,770 (4.23%)      3,337 (2.96%)      6,555 (5.81%)        SAI      2      112,683      8,127 (7.21%)      4,799 (4.26%)      3,327 (2.95%)      6,427 (5.7%)        SAI      3      112,968      8,935 (7.91%)      5,300 (4.69%)      3,634 (3.22%)      6,723 (5.95%)        SAI      4      113,414      9,449 (8.33%)      5,582 (4.92%)      3,867 (3.41%)      7,173 (6.33%)        SAI      5      112,823      9,759 (8.65%)      5,783 (5.13%)      3,975 (3.52%)      7,225 (6.4%)        SAI      12      112,888      7,633 (6.76%)      4,458 (3.95%)      3,175 (2.81%)      6,131 (5.43%)        SAI      13      112,654      8,580 (7.62%)      4,990 (4.43%)      3,590 (3.19%)      6,601 (5.86%)        SAI      14      112,582      8,412 (7.47%)      4,994 (4.44%)      3,417 (3.04%)      6,775 (6.02%)        SAI      15      113,085      7,529 (6.66%)      4,392 (3.88%)      3,137 (2.77%)      6,150 (5.44%)        SAI      16      113,002      7,638 (6.76%)      4,463 (3.95%)      3,174 (2.81%)      5,946 (5.26%)        SAI      17      112,943      8,922 (7.9%)      5,318 (4.71%)      3,604 (3.19%)      6,665 (5.9%)        SAI      18      113,466      8,634 (7.61%)      5,070 (4.47%)      3,564 (3.14%)      6,707 (5.91%)       
       # Reset the display format to the default  
    options ( scipen =   0 )    
 Now, I have to do the genotype call using the entire plate, generate
new priors, and then compare the data to the wgs data set. However, I
did not do any filtering. I could do some QC in the data before any
comparisons, but the total number of SNPs that I can compare will be
decreased. 
 
 
 
 
 6. Across samples comparisons 
 Comparing the two priors 
       import  allel 
    import  pandas  as  pd 
    import  os 
    import  numpy  as  np 
    import  warnings 
    
    # Ignore DtypeWarnings from pandas  
   warnings.filterwarnings( 'ignore' , category = pd.errors.DtypeWarning) 
    
    # Directory with vcf files  
   dir_name  =   "output/wgs_vs_chip/vcfs/"  
    
    # Get list of all vcf files in the directory  
    # vcf_files = [f for f in os.listdir(dir_name) if f.endswith('.vcf')]  
    # Get list of all vcf files in the directory with *_ab.vcf, *_aw.vcf or *_bw.vcf  
   vcf_files  =  [f  for  f  in  os.listdir(dir_name)  if  f.endswith( 'ab.vcf' )] 
    
   csv_output_files  =  [] 
    
    # Function to convert genotype indices to alleles  
    def  genotype_to_alleles(gt_indices, ref_allele, alt_alleles): 
       alleles  =  np.concatenate(([ref_allele], alt_alleles)) 
        return   " " .join(alleles[idx]  for  idx  in  gt_indices  if  idx !=-  1 )   # idx -1 means missing data  
    
    # Iterate over VCF files  
    for  vcf_file  in  vcf_files: 
       file_path  =  os.path.join(dir_name, vcf_file) 
       callset  =  allel.read_vcf(file_path, fields = [ '*' ]) 
    
        # Get genotype  
       gt  =  allel.GenotypeArray(callset[ 'calldata/GT' ]) 
    
        # Get sample names and add prefix from file name  
       sample_1, sample_2  =  callset[ 'samples' ] 
       prefix  =  vcf_file.split( "_" )[ 0 ]  +   "_"    # Added "_" after prefix  
       sample_1  =  prefix  +  sample_1 
       sample_2  =  prefix  +  sample_2 
    
        # Verify the vcf contains two samples  
        assert  gt.shape[ 1 ]  ==   2 ,  f"Expected 2 samples in   { vcf_file }  , found   { gt . shape[ 1 ] }  "  
    
        # Create DataFrame  
       df  =  pd.DataFrame({ 
            'SNP_id' : callset[ 'variants/ID' ], 
            f'  { sample_1 }  _geno' : [genotype_to_alleles(gt, callset[ 'variants/REF' ][i], callset[ 'variants/ALT' ][i])  for  i, gt  in   enumerate (gt[:,  0 ])], 
            f'  { sample_2 }  _geno' : [genotype_to_alleles(gt, callset[ 'variants/REF' ][i], callset[ 'variants/ALT' ][i])  for  i, gt  in   enumerate (gt[:,  1 ])], 
            f'  { sample_1 }  _  { sample_2 }  _gcomp' : np.where(gt[:,  0 ]  ==  gt[:,  1 ],  'match' ,  'mismatch' ).tolist(), 
            f'  { sample_1 }  _zygo' : np.where(gt.is_hom_ref()[:,  0 ],  'hom_ref' , np.where(gt.is_hom_alt()[:,  0 ],  'hom_alt' ,  'het' )).tolist(), 
            f'  { sample_2 }  _zygo' : np.where(gt.is_hom_ref()[:,  1 ],  'hom_ref' , np.where(gt.is_hom_alt()[:,  1 ],  'hom_alt' ,  'het' )).tolist(), 
            f'  { sample_1 }  _  { sample_2 }  _zcomp' : np.where(gt.is_hom()[:,  0 ]  ==  gt.is_hom()[:,  1 ],  'match' ,  'mismatch' ).tolist() 
       }) 
    
       output_file  =   f'output/wgs_vs_chip/  { os . path . basename(vcf_file) . replace( ".vcf" ,  "" ) }  _comparison_ab.csv'   # change the name here when you change the vcfs you are analyzing  
       df.to_csv(output_file, index =  False ) 
       csv_output_files.append(output_file) 
        
    # # Combine only the newly created CSVs into one  
    
    # Get the directory path where your files are located  
   dir_path  =   "output/wgs_vs_chip/"  
    
    # Get list of all CSV files in the directory that end with '_ab.csv'  
   csv_files  =  [os.path.join(dir_path, f)  for  f  in  os.listdir(dir_path)  if  f.endswith( '_ab.csv' )] 
    
    # Ensure that we have at least one such file  
    if   not  csv_files: 
        raise   ValueError ( "No CSV files found matching '_ab.csv'" ) 
    
    # Load the first CSV file  
   combined_csv  =  pd.read_csv(csv_files[ 0 ]) 
    
    # Merge the rest of the CSV files one by one  
    for  f  in  csv_files[ 1 :]: 
       df  =  pd.read_csv(f) 
       combined_csv  =  pd.merge(combined_csv, df, on =  'SNP_id' , how =  'outer' ) 
    
   combined_csv.to_csv(os.path.join(dir_path,  'combined_comparison_ab.csv' ), index =  False )    
 Compare the reference and alternative allele between the two
priors 
 Import the data and use “Tidyverse” to change column names. I left
the two codes to compare the output and make sure it is creating the
same object. 
      data_ab  <-  
      read_delim ( 
        "output/wgs_vs_chip/combined_comparison_ab.csv" , 
        delim =   "," , 
        show_col_types =   FALSE  
     ) 
    # Get all column names that end with '_gcomp'  
   gcomp_cols  <-   grep ( "_gcomp$" ,  names (data_ab),  value =   TRUE ) 
    
    # Iterate over those column names and for each, create new _ref and _alt columns  
    for  (col  in  gcomp_cols) { 
     data_ab  <-  data_ab  |>  
        separate (col,  into =   c ( paste0 (col,  "_ref" ),  paste0 (col,  "_alt" )),  sep =   "," )  |>  
        mutate ( across ( 
          starts_with ( paste0 (col,  "_" )), 
          ~   str_replace_all (.,  "  \\  [|  \\  ]|'|[:space:]" ,  "" ) 
       )) 
   } 
    
    # Renaming columns to match the reference and alternative alleles  
   data_ab  <-  
     data_ab  |>  
     dplyr ::  rename_with ( ~   str_replace_all (.,  "_gcomp_alt$" ,  "_ALT" ), 
                         ends_with ( "_gcomp_alt" ))  |>  
     dplyr ::  rename_with ( ~   str_replace_all (.,  "_gcomp_ref$" ,  "_REF" ), 
                         ends_with ( "_gcomp_ref" )) 
    
    # Now we can count how many times each SNP had errors within the 18 samples  
    # Check output  
    head (data_ab[,  c ( "SNP_id" ,  names (data_ab)[ grepl ( "_REF$|_ALT$" ,  names (data_ab))]),  with =   FALSE ])    
  ## # A tibble: 6 × 37
##   SNP_id       KAT_9a_KAT_9b_REF KAT_9a_KAT_9b_ALT SAI_15a_SAI_15b_REF
##   <chr>        <chr>             <chr>             <chr>              
## 1 AX-581444870 match             match             match              
## 2 AX-583035067 match             match             match              
## 3 AX-583033342 match             match             match              
## 4 AX-583035163 match             match             match              
## 5 AX-583035194 match             match             match              
## 6 AX-583033387 match             match             match              
## # ℹ 33 more variables: SAI_15a_SAI_15b_ALT <chr>, SAI_3a_SAI_3b_REF <chr>,
## #   SAI_3a_SAI_3b_ALT <chr>, KAT_12a_KAT_12b_REF <chr>,
## #   KAT_12a_KAT_12b_ALT <chr>, KAT_7a_KAT_7b_REF <chr>,
## #   KAT_7a_KAT_7b_ALT <chr>, SAI_2a_SAI_2b_REF <chr>, SAI_2a_SAI_2b_ALT <chr>,
## #   SAI_14a_SAI_14b_REF <chr>, SAI_14a_SAI_14b_ALT <chr>,
## #   KAT_8a_KAT_8b_REF <chr>, KAT_8a_KAT_8b_ALT <chr>,
## #   SAI_13a_SAI_13b_REF <chr>, SAI_13a_SAI_13b_ALT <chr>, …  
 Import the data and use “library(data.table) to change column
names 
       # Read the file with fread() function which is faster than read_delim()  
   data_ab_dt  <-  
      fread ( 
        here ( 
          "output" , 
          "wgs_vs_chip" ,  
          "combined_comparison_ab.csv"  
         ) 
       ) 
    
    # Get all column names that end with '_gcomp'  
   gcomp_cols  <-   grep ( "_gcomp$" ,  names (data_ab_dt),  value =   TRUE ) 
    
    # Convert data.frame to data.table  
    setDT (data_ab_dt) 
    
    # Iterate over those column names and for each, create new _REF and _ALT columns  
    for  (col  in  gcomp_cols) { 
      
      # Split each '_gcomp' column into '_REF' and '_ALT'  
     data_ab_dt[,  c ( paste0 (col,  "_REF" ),  paste0 (col,  "_ALT" ))  :=   tstrsplit ( get (col),  ", " ,  fixed=  TRUE )] 
      
      # Remove unwanted characters from each new column  
     data_ab_dt[, ( paste0 (col,  "_REF" ))  :=   gsub ( "  \\  [|  \\  ]|'" ,  "" ,  get ( paste0 (col,  "_REF" )))] 
     data_ab_dt[, ( paste0 (col,  "_ALT" ))  :=   gsub ( "  \\  [|  \\  ]|'" ,  "" ,  get ( paste0 (col,  "_ALT" )))] 
   } 
    
    # Renaming columns to remove _gcomp  
   new_names  <-   names (data_ab_dt) 
   new_names  <-   gsub ( "_gcomp_ALT$" ,  "_ALT" , new_names) 
   new_names  <-   gsub ( "_gcomp_REF$" ,  "_REF" , new_names) 
    setnames (data_ab_dt, new_names) 
    
    
    # Select and display only columns that match the criteria  
    head (data_ab_dt[,  c ( "SNP_id" ,  names (data_ab_dt)[ grepl ( "_REF$|_ALT$" ,  names (data_ab_dt))]),  with =   FALSE ])    
  ##          SNP_id KAT_9a_KAT_9b_REF KAT_9a_KAT_9b_ALT SAI_15a_SAI_15b_REF
## 1: AX-581444870             match             match               match
## 2: AX-583035067             match             match               match
## 3: AX-583033342             match             match               match
## 4: AX-583035163             match             match               match
## 5: AX-583035194             match             match               match
## 6: AX-583033387             match             match               match
##    SAI_15a_SAI_15b_ALT SAI_3a_SAI_3b_REF SAI_3a_SAI_3b_ALT KAT_12a_KAT_12b_REF
## 1:               match             match             match               match
## 2:               match             match             match               match
## 3:               match             match             match               match
## 4:               match             match             match               match
## 5:               match             match             match               match
## 6:               match             match             match               match
##    KAT_12a_KAT_12b_ALT KAT_7a_KAT_7b_REF KAT_7a_KAT_7b_ALT SAI_2a_SAI_2b_REF
## 1:               match             match             match              <NA>
## 2:               match             match             match             match
## 3:               match             match             match             match
## 4:               match             match             match             match
## 5:               match             match             match             match
## 6:               match             match             match             match
##    SAI_2a_SAI_2b_ALT SAI_14a_SAI_14b_REF SAI_14a_SAI_14b_ALT KAT_8a_KAT_8b_REF
## 1:              <NA>               match               match             match
## 2:             match               match               match             match
## 3:             match               match               match             match
## 4:             match               match               match             match
## 5:             match               match               match             match
## 6:             match               match               match             match
##    KAT_8a_KAT_8b_ALT SAI_13a_SAI_13b_REF SAI_13a_SAI_13b_ALT SAI_5a_SAI_5b_REF
## 1:             match               match               match             match
## 2:             match               match               match             match
## 3:             match               match               match             match
## 4:             match               match               match             match
## 5:             match               match               match             match
## 6:             match               match               match             match
##    SAI_5a_SAI_5b_ALT SAI_18a_SAI_18b_REF SAI_18a_SAI_18b_ALT
## 1:             match               match               match
## 2:             match               match               match
## 3:             match               match               match
## 4:             match               match               match
## 5:             match               match               match
## 6:             match               match               match
##    KAT_10a_KAT_10b_REF KAT_10a_KAT_10b_ALT SAI_1a_SAI_1b_REF SAI_1a_SAI_1b_ALT
## 1:               match               match              <NA>              <NA>
## 2:               match               match             match             match
## 3:               match               match             match             match
## 4:               match               match             match             match
## 5:               match               match             match             match
## 6:               match               match             match             match
##    SAI_17a_SAI_17b_REF SAI_17a_SAI_17b_ALT SAI_4a_SAI_4b_REF SAI_4a_SAI_4b_ALT
## 1:               match               match             match             match
## 2:               match               match             match             match
## 3:               match               match             match             match
## 4:               match               match             match             match
## 5:               match               match             match             match
## 6:               match               match             match             match
##    SAI_12a_SAI_12b_REF SAI_12a_SAI_12b_ALT KAT_11a_KAT_11b_REF
## 1:               match               match               match
## 2:               match               match               match
## 3:               match               match               match
## 4:               match               match               match
## 5:               match               match               match
## 6:               match               match               match
##    KAT_11a_KAT_11b_ALT SAI_16a_SAI_16b_REF SAI_16a_SAI_16b_ALT
## 1:               match               match               match
## 2:               match               match               match
## 3:               match               match               match
## 4:               match               match               match
## 5:               match               match               match
## 6:               match               match               match  
 Compare one sample to see if the counts match and mismatch are
correct using “Tidyverse” or “data.table” 
       table (data_ab $ KAT_11a_KAT_11b_REF)    
  ## 
##    match mismatch 
##    87506      546  
       table (data_ab_dt $ KAT_11a_KAT_11b_REF)    
  ## 
##    match mismatch 
##    87506      546  
 We can also count NAs 
       table (data_ab $ KAT_11a_KAT_11b_REF,  useNA =   "ifany" )    
  ## 
##    match mismatch     <NA> 
##    87506      546     2490  
       table (data_ab_dt $ KAT_11a_KAT_11b_REF,  useNA =   "ifany" )    
  ## 
##    match mismatch     <NA> 
##    87506      546     2490  
 The main difference between the objects is that we kept the original
columns in data_ab_dt but not in the data_ab. It is not important but we
can inspect the data for inconsistencies in our code. 
 Now we can change our code to get all the metrics we want. We have
too many column names in our data. Check column names 
       colnames (data_ab_dt)    
  ##   [1] "SNP_id"                "KAT_9a_geno"           "KAT_9b_geno"          
##   [4] "KAT_9a_KAT_9b_gcomp"   "KAT_9a_zygo"           "KAT_9b_zygo"          
##   [7] "KAT_9a_KAT_9b_zcomp"   "SAI_15a_geno"          "SAI_15b_geno"         
##  [10] "SAI_15a_SAI_15b_gcomp" "SAI_15a_zygo"          "SAI_15b_zygo"         
##  [13] "SAI_15a_SAI_15b_zcomp" "SAI_3a_geno"           "SAI_3b_geno"          
##  [16] "SAI_3a_SAI_3b_gcomp"   "SAI_3a_zygo"           "SAI_3b_zygo"          
##  [19] "SAI_3a_SAI_3b_zcomp"   "KAT_12a_geno"          "KAT_12b_geno"         
##  [22] "KAT_12a_KAT_12b_gcomp" "KAT_12a_zygo"          "KAT_12b_zygo"         
##  [25] "KAT_12a_KAT_12b_zcomp" "KAT_7a_geno"           "KAT_7b_geno"          
##  [28] "KAT_7a_KAT_7b_gcomp"   "KAT_7a_zygo"           "KAT_7b_zygo"          
##  [31] "KAT_7a_KAT_7b_zcomp"   "SAI_2a_geno"           "SAI_2b_geno"          
##  [34] "SAI_2a_SAI_2b_gcomp"   "SAI_2a_zygo"           "SAI_2b_zygo"          
##  [37] "SAI_2a_SAI_2b_zcomp"   "SAI_14a_geno"          "SAI_14b_geno"         
##  [40] "SAI_14a_SAI_14b_gcomp" "SAI_14a_zygo"          "SAI_14b_zygo"         
##  [43] "SAI_14a_SAI_14b_zcomp" "KAT_8a_geno"           "KAT_8b_geno"          
##  [46] "KAT_8a_KAT_8b_gcomp"   "KAT_8a_zygo"           "KAT_8b_zygo"          
##  [49] "KAT_8a_KAT_8b_zcomp"   "SAI_13a_geno"          "SAI_13b_geno"         
##  [52] "SAI_13a_SAI_13b_gcomp" "SAI_13a_zygo"          "SAI_13b_zygo"         
##  [55] "SAI_13a_SAI_13b_zcomp" "SAI_5a_geno"           "SAI_5b_geno"          
##  [58] "SAI_5a_SAI_5b_gcomp"   "SAI_5a_zygo"           "SAI_5b_zygo"          
##  [61] "SAI_5a_SAI_5b_zcomp"   "SAI_18a_geno"          "SAI_18b_geno"         
##  [64] "SAI_18a_SAI_18b_gcomp" "SAI_18a_zygo"          "SAI_18b_zygo"         
##  [67] "SAI_18a_SAI_18b_zcomp" "KAT_10a_geno"          "KAT_10b_geno"         
##  [70] "KAT_10a_KAT_10b_gcomp" "KAT_10a_zygo"          "KAT_10b_zygo"         
##  [73] "KAT_10a_KAT_10b_zcomp" "SAI_1a_geno"           "SAI_1b_geno"          
##  [76] "SAI_1a_SAI_1b_gcomp"   "SAI_1a_zygo"           "SAI_1b_zygo"          
##  [79] "SAI_1a_SAI_1b_zcomp"   "SAI_17a_geno"          "SAI_17b_geno"         
##  [82] "SAI_17a_SAI_17b_gcomp" "SAI_17a_zygo"          "SAI_17b_zygo"         
##  [85] "SAI_17a_SAI_17b_zcomp" "SAI_4a_geno"           "SAI_4b_geno"          
##  [88] "SAI_4a_SAI_4b_gcomp"   "SAI_4a_zygo"           "SAI_4b_zygo"          
##  [91] "SAI_4a_SAI_4b_zcomp"   "SAI_12a_geno"          "SAI_12b_geno"         
##  [94] "SAI_12a_SAI_12b_gcomp" "SAI_12a_zygo"          "SAI_12b_zygo"         
##  [97] "SAI_12a_SAI_12b_zcomp" "KAT_11a_geno"          "KAT_11b_geno"         
## [100] "KAT_11a_KAT_11b_gcomp" "KAT_11a_zygo"          "KAT_11b_zygo"         
## [103] "KAT_11a_KAT_11b_zcomp" "SAI_16a_geno"          "SAI_16b_geno"         
## [106] "SAI_16a_SAI_16b_gcomp" "SAI_16a_zygo"          "SAI_16b_zygo"         
## [109] "SAI_16a_SAI_16b_zcomp" "KAT_9a_KAT_9b_REF"     "KAT_9a_KAT_9b_ALT"    
## [112] "SAI_15a_SAI_15b_REF"   "SAI_15a_SAI_15b_ALT"   "SAI_3a_SAI_3b_REF"    
## [115] "SAI_3a_SAI_3b_ALT"     "KAT_12a_KAT_12b_REF"   "KAT_12a_KAT_12b_ALT"  
## [118] "KAT_7a_KAT_7b_REF"     "KAT_7a_KAT_7b_ALT"     "SAI_2a_SAI_2b_REF"    
## [121] "SAI_2a_SAI_2b_ALT"     "SAI_14a_SAI_14b_REF"   "SAI_14a_SAI_14b_ALT"  
## [124] "KAT_8a_KAT_8b_REF"     "KAT_8a_KAT_8b_ALT"     "SAI_13a_SAI_13b_REF"  
## [127] "SAI_13a_SAI_13b_ALT"   "SAI_5a_SAI_5b_REF"     "SAI_5a_SAI_5b_ALT"    
## [130] "SAI_18a_SAI_18b_REF"   "SAI_18a_SAI_18b_ALT"   "KAT_10a_KAT_10b_REF"  
## [133] "KAT_10a_KAT_10b_ALT"   "SAI_1a_SAI_1b_REF"     "SAI_1a_SAI_1b_ALT"    
## [136] "SAI_17a_SAI_17b_REF"   "SAI_17a_SAI_17b_ALT"   "SAI_4a_SAI_4b_REF"    
## [139] "SAI_4a_SAI_4b_ALT"     "SAI_12a_SAI_12b_REF"   "SAI_12a_SAI_12b_ALT"  
## [142] "KAT_11a_KAT_11b_REF"   "KAT_11a_KAT_11b_ALT"   "SAI_16a_SAI_16b_REF"  
## [145] "SAI_16a_SAI_16b_ALT"  
 Check the data 
       glimpse (data_ab_dt)    
  ## Rows: 90,542
## Columns: 145
## $ SNP_id                <chr> "AX-581444870", "AX-583035067", "AX-583033342", …
## $ KAT_9a_geno           <chr> "T T", "A T", "G C", "G G", "G G", "T C", "T T",…
## $ KAT_9b_geno           <chr> "T T", "A T", "G C", "G G", "G G", "T C", "T T",…
## $ KAT_9a_KAT_9b_gcomp   <chr> "['match', 'match']", "['match', 'match']", "['m…
## $ KAT_9a_zygo           <chr> "hom_ref", "het", "het", "hom_ref", "hom_ref", "…
## $ KAT_9b_zygo           <chr> "hom_ref", "het", "het", "hom_ref", "hom_ref", "…
## $ KAT_9a_KAT_9b_zcomp   <chr> "match", "match", "match", "match", "match", "ma…
## $ SAI_15a_geno          <chr> "T T", "A A", "G G", "G A", "G A", "T T", "T T",…
## $ SAI_15b_geno          <chr> "T T", "A A", "G G", "G A", "G A", "T T", "T T",…
## $ SAI_15a_SAI_15b_gcomp <chr> "['match', 'match']", "['match', 'match']", "['m…
## $ SAI_15a_zygo          <chr> "hom_ref", "hom_ref", "hom_ref", "het", "het", "…
## $ SAI_15b_zygo          <chr> "hom_ref", "hom_ref", "hom_ref", "het", "het", "…
## $ SAI_15a_SAI_15b_zcomp <chr> "match", "match", "match", "match", "match", "ma…
## $ SAI_3a_geno           <chr> "T T", "A A", "G G", "G G", "G A", "T T", "T T",…
## $ SAI_3b_geno           <chr> "T T", "A A", "G G", "G G", "G A", "T T", "T T",…
## $ SAI_3a_SAI_3b_gcomp   <chr> "['match', 'match']", "['match', 'match']", "['m…
## $ SAI_3a_zygo           <chr> "hom_ref", "hom_ref", "hom_ref", "hom_ref", "het…
## $ SAI_3b_zygo           <chr> "hom_ref", "hom_ref", "hom_ref", "hom_ref", "het…
## $ SAI_3a_SAI_3b_zcomp   <chr> "match", "match", "match", "match", "match", "ma…
## $ KAT_12a_geno          <chr> "T T", "A A", "G C", "G G", "G A", "T T", "T T",…
## $ KAT_12b_geno          <chr> "T T", "A A", "G C", "G G", "G A", "T T", "T T",…
## $ KAT_12a_KAT_12b_gcomp <chr> "['match', 'match']", "['match', 'match']", "['m…
## $ KAT_12a_zygo          <chr> "hom_ref", "hom_ref", "het", "hom_ref", "het", "…
## $ KAT_12b_zygo          <chr> "hom_ref", "hom_ref", "het", "hom_ref", "het", "…
## $ KAT_12a_KAT_12b_zcomp <chr> "match", "match", "match", "match", "match", "ma…
## $ KAT_7a_geno           <chr> "T T", "A T", "G C", "G G", "G G", "T T", "T T",…
## $ KAT_7b_geno           <chr> "T T", "A T", "G C", "G G", "G G", "T T", "T T",…
## $ KAT_7a_KAT_7b_gcomp   <chr> "['match', 'match']", "['match', 'match']", "['m…
## $ KAT_7a_zygo           <chr> "hom_ref", "het", "het", "hom_ref", "hom_ref", "…
## $ KAT_7b_zygo           <chr> "hom_ref", "het", "het", "hom_ref", "hom_ref", "…
## $ KAT_7a_KAT_7b_zcomp   <chr> "match", "match", "match", "match", "match", "ma…
## $ SAI_2a_geno           <chr> "", "A A", "G G", "G A", "G G", "T T", "T T", "C…
## $ SAI_2b_geno           <chr> "", "A A", "G G", "G A", "G G", "T T", "T T", "C…
## $ SAI_2a_SAI_2b_gcomp   <chr> "", "['match', 'match']", "['match', 'match']", …
## $ SAI_2a_zygo           <chr> "", "hom_ref", "hom_ref", "het", "hom_ref", "hom…
## $ SAI_2b_zygo           <chr> "", "hom_ref", "hom_ref", "het", "hom_ref", "hom…
## $ SAI_2a_SAI_2b_zcomp   <chr> "", "match", "match", "match", "match", "match",…
## $ SAI_14a_geno          <chr> "T T", "A A", "G G", "G G", "G G", "T T", "T T",…
## $ SAI_14b_geno          <chr> "T T", "A A", "G G", "G G", "G G", "T T", "T T",…
## $ SAI_14a_SAI_14b_gcomp <chr> "['match', 'match']", "['match', 'match']", "['m…
## $ SAI_14a_zygo          <chr> "hom_ref", "hom_ref", "hom_ref", "hom_ref", "hom…
## $ SAI_14b_zygo          <chr> "hom_ref", "hom_ref", "hom_ref", "hom_ref", "hom…
## $ SAI_14a_SAI_14b_zcomp <chr> "match", "match", "match", "match", "match", "ma…
## $ KAT_8a_geno           <chr> "T T", "A A", "G C", "G G", "G A", "T T", "T T",…
## $ KAT_8b_geno           <chr> "T T", "A A", "G C", "G G", "G A", "T T", "T T",…
## $ KAT_8a_KAT_8b_gcomp   <chr> "['match', 'match']", "['match', 'match']", "['m…
## $ KAT_8a_zygo           <chr> "hom_ref", "hom_ref", "het", "hom_ref", "het", "…
## $ KAT_8b_zygo           <chr> "hom_ref", "hom_ref", "het", "hom_ref", "het", "…
## $ KAT_8a_KAT_8b_zcomp   <chr> "match", "match", "match", "match", "match", "ma…
## $ SAI_13a_geno          <chr> "T T", "A A", "G G", "A A", "G G", "T T", "T T",…
## $ SAI_13b_geno          <chr> "T T", "A A", "G G", "A A", "G G", "T T", "T T",…
## $ SAI_13a_SAI_13b_gcomp <chr> "['match', 'match']", "['match', 'match']", "['m…
## $ SAI_13a_zygo          <chr> "hom_ref", "hom_ref", "hom_ref", "hom_alt", "hom…
## $ SAI_13b_zygo          <chr> "hom_ref", "hom_ref", "hom_ref", "hom_alt", "hom…
## $ SAI_13a_SAI_13b_zcomp <chr> "match", "match", "match", "match", "match", "ma…
## $ SAI_5a_geno           <chr> "T T", "A A", "G G", "G G", "G A", "T C", "T T",…
## $ SAI_5b_geno           <chr> "T T", "A A", "G G", "G G", "G A", "T C", "T T",…
## $ SAI_5a_SAI_5b_gcomp   <chr> "['match', 'match']", "['match', 'match']", "['m…
## $ SAI_5a_zygo           <chr> "hom_ref", "hom_ref", "hom_ref", "hom_ref", "het…
## $ SAI_5b_zygo           <chr> "hom_ref", "hom_ref", "hom_ref", "hom_ref", "het…
## $ SAI_5a_SAI_5b_zcomp   <chr> "match", "match", "match", "match", "match", "ma…
## $ SAI_18a_geno          <chr> "T T", "A A", "G G", "G A", "G A", "T T", "T T",…
## $ SAI_18b_geno          <chr> "T T", "A A", "G G", "G A", "G A", "T T", "T T",…
## $ SAI_18a_SAI_18b_gcomp <chr> "['match', 'match']", "['match', 'match']", "['m…
## $ SAI_18a_zygo          <chr> "hom_ref", "hom_ref", "hom_ref", "het", "het", "…
## $ SAI_18b_zygo          <chr> "hom_ref", "hom_ref", "hom_ref", "het", "het", "…
## $ SAI_18a_SAI_18b_zcomp <chr> "match", "match", "match", "match", "match", "ma…
## $ KAT_10a_geno          <chr> "T T", "A T", "C C", "G G", "G G", "T T", "T T",…
## $ KAT_10b_geno          <chr> "T T", "A T", "C C", "G G", "G G", "T T", "T T",…
## $ KAT_10a_KAT_10b_gcomp <chr> "['match', 'match']", "['match', 'match']", "['m…
## $ KAT_10a_zygo          <chr> "hom_ref", "het", "hom_alt", "hom_ref", "hom_ref…
## $ KAT_10b_zygo          <chr> "hom_ref", "het", "hom_alt", "hom_ref", "hom_ref…
## $ KAT_10a_KAT_10b_zcomp <chr> "match", "match", "match", "match", "match", "ma…
## $ SAI_1a_geno           <chr> "", "A A", "G G", "G G", "G A", "T T", "T T", "C…
## $ SAI_1b_geno           <chr> "", "A A", "G G", "G G", "G A", "T T", "T T", "C…
## $ SAI_1a_SAI_1b_gcomp   <chr> "", "['match', 'match']", "['match', 'match']", …
## $ SAI_1a_zygo           <chr> "", "hom_ref", "hom_ref", "hom_ref", "het", "hom…
## $ SAI_1b_zygo           <chr> "", "hom_ref", "hom_ref", "hom_ref", "het", "hom…
## $ SAI_1a_SAI_1b_zcomp   <chr> "", "match", "match", "match", "match", "match",…
## $ SAI_17a_geno          <chr> "T T", "A A", "G G", "G A", "G A", "T T", "T T",…
## $ SAI_17b_geno          <chr> "T T", "A A", "G G", "G A", "G A", "T T", "T T",…
## $ SAI_17a_SAI_17b_gcomp <chr> "['match', 'match']", "['match', 'match']", "['m…
## $ SAI_17a_zygo          <chr> "hom_ref", "hom_ref", "hom_ref", "het", "het", "…
## $ SAI_17b_zygo          <chr> "hom_ref", "hom_ref", "hom_ref", "het", "het", "…
## $ SAI_17a_SAI_17b_zcomp <chr> "match", "match", "match", "match", "match", "ma…
## $ SAI_4a_geno           <chr> "T T", "A A", "G G", "G G", "G A", "C C", "T C",…
## $ SAI_4b_geno           <chr> "T T", "A A", "G G", "G G", "G A", "C C", "T C",…
## $ SAI_4a_SAI_4b_gcomp   <chr> "['match', 'match']", "['match', 'match']", "['m…
## $ SAI_4a_zygo           <chr> "hom_ref", "hom_ref", "hom_ref", "hom_ref", "het…
## $ SAI_4b_zygo           <chr> "hom_ref", "hom_ref", "hom_ref", "hom_ref", "het…
## $ SAI_4a_SAI_4b_zcomp   <chr> "match", "match", "match", "match", "match", "ma…
## $ SAI_12a_geno          <chr> "T T", "A A", "G G", "G G", "G A", "T T", "T T",…
## $ SAI_12b_geno          <chr> "T T", "A A", "G G", "G G", "G A", "T T", "T T",…
## $ SAI_12a_SAI_12b_gcomp <chr> "['match', 'match']", "['match', 'match']", "['m…
## $ SAI_12a_zygo          <chr> "hom_ref", "hom_ref", "hom_ref", "hom_ref", "het…
## $ SAI_12b_zygo          <chr> "hom_ref", "hom_ref", "hom_ref", "hom_ref", "het…
## $ SAI_12a_SAI_12b_zcomp <chr> "match", "match", "match", "match", "match", "ma…
## $ KAT_11a_geno          <chr> "T T", "A T", "C C", "G G", "G G", "T T", "T T",…
## $ KAT_11b_geno          <chr> "T T", "A T", "C C", "G G", "G G", "T T", "T T",…
## $ KAT_11a_KAT_11b_gcomp <chr> "['match', 'match']", "['match', 'match']", "['m…
## $ KAT_11a_zygo          <chr> "hom_ref", "het", "hom_alt", "hom_ref", "hom_ref…
## $ KAT_11b_zygo          <chr> "hom_ref", "het", "hom_alt", "hom_ref", "hom_ref…
## $ KAT_11a_KAT_11b_zcomp <chr> "match", "match", "match", "match", "match", "ma…
## $ SAI_16a_geno          <chr> "T T", "A A", "G G", "G G", "G A", "T T", "T T",…
## $ SAI_16b_geno          <chr> "T T", "A A", "G G", "G G", "G A", "T T", "T T",…
## $ SAI_16a_SAI_16b_gcomp <chr> "['match', 'match']", "['match', 'match']", "['m…
## $ SAI_16a_zygo          <chr> "hom_ref", "hom_ref", "hom_ref", "hom_ref", "het…
## $ SAI_16b_zygo          <chr> "hom_ref", "hom_ref", "hom_ref", "hom_ref", "het…
## $ SAI_16a_SAI_16b_zcomp <chr> "match", "match", "match", "match", "match", "ma…
## $ KAT_9a_KAT_9b_REF     <chr> "match", "match", "match", "match", "match", "ma…
## $ KAT_9a_KAT_9b_ALT     <chr> "match", "match", "match", "match", "match", "ma…
## $ SAI_15a_SAI_15b_REF   <chr> "match", "match", "match", "match", "match", "ma…
## $ SAI_15a_SAI_15b_ALT   <chr> "match", "match", "match", "match", "match", "ma…
## $ SAI_3a_SAI_3b_REF     <chr> "match", "match", "match", "match", "match", "ma…
## $ SAI_3a_SAI_3b_ALT     <chr> "match", "match", "match", "match", "match", "ma…
## $ KAT_12a_KAT_12b_REF   <chr> "match", "match", "match", "match", "match", "ma…
## $ KAT_12a_KAT_12b_ALT   <chr> "match", "match", "match", "match", "match", "ma…
## $ KAT_7a_KAT_7b_REF     <chr> "match", "match", "match", "match", "match", "ma…
## $ KAT_7a_KAT_7b_ALT     <chr> "match", "match", "match", "match", "match", "ma…
## $ SAI_2a_SAI_2b_REF     <chr> NA, "match", "match", "match", "match", "match",…
## $ SAI_2a_SAI_2b_ALT     <chr> NA, "match", "match", "match", "match", "match",…
## $ SAI_14a_SAI_14b_REF   <chr> "match", "match", "match", "match", "match", "ma…
## $ SAI_14a_SAI_14b_ALT   <chr> "match", "match", "match", "match", "match", "ma…
## $ KAT_8a_KAT_8b_REF     <chr> "match", "match", "match", "match", "match", "ma…
## $ KAT_8a_KAT_8b_ALT     <chr> "match", "match", "match", "match", "match", "ma…
## $ SAI_13a_SAI_13b_REF   <chr> "match", "match", "match", "match", "match", "ma…
## $ SAI_13a_SAI_13b_ALT   <chr> "match", "match", "match", "match", "match", "ma…
## $ SAI_5a_SAI_5b_REF     <chr> "match", "match", "match", "match", "match", "ma…
## $ SAI_5a_SAI_5b_ALT     <chr> "match", "match", "match", "match", "match", "ma…
## $ SAI_18a_SAI_18b_REF   <chr> "match", "match", "match", "match", "match", "ma…
## $ SAI_18a_SAI_18b_ALT   <chr> "match", "match", "match", "match", "match", "ma…
## $ KAT_10a_KAT_10b_REF   <chr> "match", "match", "match", "match", "match", "ma…
## $ KAT_10a_KAT_10b_ALT   <chr> "match", "match", "match", "match", "match", "ma…
## $ SAI_1a_SAI_1b_REF     <chr> NA, "match", "match", "match", "match", "match",…
## $ SAI_1a_SAI_1b_ALT     <chr> NA, "match", "match", "match", "match", "match",…
## $ SAI_17a_SAI_17b_REF   <chr> "match", "match", "match", "match", "match", "ma…
## $ SAI_17a_SAI_17b_ALT   <chr> "match", "match", "match", "match", "match", "ma…
## $ SAI_4a_SAI_4b_REF     <chr> "match", "match", "match", "match", "match", "ma…
## $ SAI_4a_SAI_4b_ALT     <chr> "match", "match", "match", "match", "match", "ma…
## $ SAI_12a_SAI_12b_REF   <chr> "match", "match", "match", "match", "match", "ma…
## $ SAI_12a_SAI_12b_ALT   <chr> "match", "match", "match", "match", "match", "ma…
## $ KAT_11a_KAT_11b_REF   <chr> "match", "match", "match", "match", "match", "ma…
## $ KAT_11a_KAT_11b_ALT   <chr> "match", "match", "match", "match", "match", "ma…
## $ SAI_16a_SAI_16b_REF   <chr> "match", "match", "match", "match", "match", "ma…
## $ SAI_16a_SAI_16b_ALT   <chr> "match", "match", "match", "match", "match", "ma…  
 Although we have 129 columns, we have the comparison of each sample
using different priors or genotyping technology. Then, we have genotypes
of each sample, for example our first samples are “KAT_11a_geno” and
“KAT_11b_geno”. In this column we have the real genotype of the sample.
Here sample “a” and sample “b” are references to the two priors we are
comparing (a - default and b - new prior from the crosses). Later, I
will compare the default prior with the plate prior (I will create a
prior using the plate that had the 18 samples we are comparing). 
 The next columns are the comparison of the reference and alternative
alleles. The values in these columns are “match” and “mismatch”. Later
we can summarize the data by counting the strings “match” and “mismatch”
across the 18 samples. Or if we are curious, even compare the two
populations. 
 The next column are about the zygosity of each sample. As our first
samples we have the columns: “KAT_11a_zygo” “KAT_11b_zygo” and
“KAT_11a_KAT_11b_zcomp”. The values in the two first columns are
“hom_ref”, “hom_alt”, or “het”. The values for the column _zcomp are
“match” or “mismatch” as result of comparing the zygosity of the two
columns before it. 
 We can create two new columns comparing all the samples. 
       # Convert your data to a data.table (it is already)  
    setDT (data_ab_dt) 
    
    # Create columns for match and mismatch count for columns ending with _REF  
   cols_REF  <-   
      grep ( "_REF$" ,  names (data_ab_dt),  value =   TRUE ) 
    
    # Calculate the count of "match" or "mismatch" for each row  
   data_ab_dt[,  c ( "REF_match_count" ,  "REF_mismatch_count" )  :=  
             .( rowSums (.SD  ==   "match" ,  na.rm =   TRUE ), 
                rowSums (.SD  ==   "mismatch" ,  na.rm =   TRUE )), 
           .SDcols  =  cols_REF] 
    
    # Create columns for match and mismatch count for columns ending with _ALT  
   cols_ALT  <-   
      grep ( "_ALT$" ,  names (data_ab_dt),  value =   TRUE ) 
    
    # Calculate the count of "match" or "mismatch" for each row  
   data_ab_dt[,  c ( "ALT_match_count" ,  "ALT_mismatch_count" )  :=  
             .( rowSums (.SD  ==   "match" ,  na.rm =   TRUE ), 
                rowSums (.SD  ==   "mismatch" ,  na.rm =   TRUE )), 
           .SDcols  =  cols_ALT] 
    
    # Create columns for match and mismatch count for columns ending with _zcomp  
   cols_Zigo  <-  
      grep ( "_zcomp$" ,  names (data_ab_dt),  value =   TRUE ) 
    
    # Calculate the count of "match" or "mismatch" for each row  
   data_ab_dt[,  c ( "Zigo_match_count" ,  "Zigo_mismatch_count" )  :=  
             .( rowSums (.SD  ==   "match" ,  na.rm =   TRUE ), 
                rowSums (.SD  ==   "mismatch" ,  na.rm =   TRUE )), 
           .SDcols  =  cols_Zigo] 
    
    # Now, you can summarize this for each SNP_id  
   summary_18_samples  <-  
     data_ab_dt[, .( 
        REF_match =   sum (REF_match_count,  na.rm =   TRUE ), 
        REF_mismatch =   sum (REF_mismatch_count,  na.rm =   TRUE ), 
        ALT_match =   sum (ALT_match_count,  na.rm =   TRUE ), 
        ALT_mismatch =   sum (ALT_mismatch_count,  na.rm =   TRUE ), 
        Zigo_match =   sum (Zigo_match_count,  na.rm =   TRUE ), 
        Zigo_mismatch =   sum (Zigo_mismatch_count,  na.rm =   TRUE ) 
     ), 
     by  =  SNP_id] 
    
    # Sort data by SNP_id  
    setorder (summary_18_samples, SNP_id) 
    
    # Check the result  
    head (summary_18_samples)    
  ##          SNP_id REF_match REF_mismatch ALT_match ALT_mismatch Zigo_match
## 1: AX-579436125        18            0        18            0         18
## 2: AX-579436196        16            0        16            0         16
## 3: AX-579436243        15            3        18            0         15
## 4: AX-579436298        17            0        17            0         17
## 5: AX-579436308        16            0        16            0         16
## 6: AX-579436317        18            0        18            0         18
##    Zigo_mismatch
## 1:             0
## 2:             0
## 3:             3
## 4:             0
## 5:             0
## 6:             0  
 
 6.1 Total discrepancies across all samples 
 How many SNPs have discrepancies in the genotypes in 1 or more
samples (out of the 18 samples) 
       # Discrepancies in 1 or more samples  
    # How many SNPs we tested  
   tested_snps  <-   length ( unique (data_ab_dt $ SNP_id)) 
    cat ( "Number of SNPs tested:" , tested_snps,  "  \n  " )    
  ## Number of SNPs tested: 90542  
       # How many SNPs failed  
   failed_snpsR  <-  
      length ( 
        unique (data_ab_dt[data_ab_dt $ REF_mismatch_count  >=   1 ,] $ SNP_id 
              ) 
            ) 
    cat ( "REF mismatch at in 1 sample:" , failed_snpsR,  "  \n  " )    
  ## REF mismatch at in 1 sample: 6387  
       # How many SNPs failed  
   failed_snpsA  <-  
      length ( 
        unique (data_ab_dt[data_ab_dt $ ALT_mismatch_count  >=   1 ,,] $ SNP_id 
              ) 
            ) 
    cat ( "ALT mismatch at least in 1 sample:" , failed_snpsA,  "  \n  " )    
  ## ALT mismatch at least in 1 sample: 3464  
       # How many SNPs failed zygosity  
   failed_snps  <-  
      length ( 
        unique (data_ab_dt[data_ab_dt $ Zigo_mismatch_count  >=   1 ,,] $ SNP_id 
              ) 
            ) 
    cat ( "Zygosity mismatch in at least 1 sample:" , failed_snps,  "  \n  " )    
  ## Zygosity mismatch in at least 1 sample: 9309  
       # Calculate percentage  
   percentage_failed  <-   round (failed_snps  /  tested_snps  *   100 ,  2 ) 
    cat ( "Percentage of failed SNPs in 1 or more samples:" , percentage_failed,  "%  \n  " )    
  ## Percentage of failed SNPs in 1 or more samples: 10.28 %  
 We see 12,031 SNPs with discrepancies but most of them are only in 1
sample. Lets check how many have errors in two samples 
       # Discrepancies in 2 or more samples  
    # How many SNPs we tested  
   tested_snps  <-   length ( unique (data_ab_dt $ SNP_id)) 
    cat ( "Number of SNPs tested:" , tested_snps,  "  \n  " )    
  ## Number of SNPs tested: 90542  
       # How many SNPs failed  
   failed_snpsR  <-  
      length ( 
        unique (data_ab_dt[data_ab_dt $ REF_mismatch_count  >=   2 ,] $ SNP_id 
              ) 
            ) 
    cat ( "REF mismatch in 2 or more samples:" , failed_snpsR,  "  \n  " )    
  ## REF mismatch in 2 or more samples: 2657  
       # How many SNPs failed  
   failed_snpsA  <-  
      length ( 
        unique (data_ab_dt[data_ab_dt $ ALT_mismatch_count  >=   2 ,] $ SNP_id 
              ) 
            ) 
    cat ( "ALT mismatch in 2 or more samples:" , failed_snpsA,  "  \n  " )    
  ## ALT mismatch in 2 or more samples: 1286  
       # How many SNPs failed  
   failed_snps  <-  
      length ( 
        unique (data_ab_dt[data_ab_dt $ Zigo_mismatch_count  >=   2 ,] $ SNP_id 
              ) 
            ) 
    cat ( "Zygosity mismatch in 2 or more samples:" , failed_snps,  "  \n  " )    
  ## Zygosity mismatch in 2 or more samples: 3936  
       # Calculate percentage  
   percentage_failed  <-   round (failed_snps  /  tested_snps  *   100 ,  2 ) 
    cat ( "Percentage of failed SNPs in 2 or more samples:" , percentage_failed,  "%  \n  " )    
  ## Percentage of failed SNPs in 2 or more samples: 4.35 %  
 We see that half of the SNPs have mismatching genotypes in 1 sample
only and 6,061 SNPs show genotyping mismatches in 2 or more samples. 
       # Check how many SNPs with errors in only 1 sample  
    # How many SNPs we tested  
   tested_snps  <-   length ( unique (data_ab_dt $ SNP_id)) 
    cat ( "Number of SNPs tested:" , tested_snps,  "  \n  " )    
  ## Number of SNPs tested: 90542  
       # How many SNPs failed  
   failed_snpsR  <-  
      length ( 
        unique (data_ab_dt[data_ab_dt $ REF_mismatch_count  ==   1 ,] $ SNP_id 
              ) 
            ) 
    cat ( "REF mismatch in only 1 sample:" , failed_snpsR,  "  \n  " )    
  ## REF mismatch in only 1 sample: 3730  
       # How many SNPs failed  
   failed_snpsA  <-  
      length ( 
        unique (data_ab_dt[data_ab_dt $ ALT_mismatch_count  ==   1 ,] $ SNP_id 
              ) 
            ) 
    cat ( "ALT mismatch in only 1 sample:" , failed_snpsA,  "  \n  " )    
  ## ALT mismatch in only 1 sample: 2178  
       # How many SNPs failed  
   failed_snps  <-  
      length ( 
        unique (data_ab_dt[data_ab_dt $ Zigo_mismatch_count  ==   1 ,] $ SNP_id 
              ) 
            ) 
    cat ( "Zygosity mismatch in only 1 sample:" , failed_snps,  "  \n  " )    
  ## Zygosity mismatch in only 1 sample: 5373  
       # Calculate percentage  
   percentage_failed  <-   round (failed_snps  /  tested_snps  *   100 ,  2 ) 
    cat ( "Percentage of failed SNPs in only 1 sample:" , percentage_failed,  "%  \n  " )    
  ## Percentage of failed SNPs in only 1 sample: 5.93 %  
 We observe that 6,004 SNPs have genotype mismatches in only 1 sample
out of the 18 samples. Is it random or does it follow a pattern? 
 Nearly half of the SNPs that have discrepancies are from a single
sample genotype mismatch. 
 We can create a histogram of the number of errors or mismatches per
sample 
       # summary_18_samples is your data.table  
    setDT (summary_18_samples) 
    
    # Select only the relevant columns  
   dt  <-   
     summary_18_samples[, .(SNP_id, REF_mismatch, ALT_mismatch, Zigo_mismatch)] 
    
    # Reshape data to long format  
   dt_long  <-   
      melt (dt,  id.vars =   "SNP_id" ,  variable.name =   "type" ,  value.name =   "count" ) 
    
    # Convert to data.table if it's not already  
    setDT (dt_long) 
    
    # Convert to numeric if it's not already  
   dt_long[, count  :=   as.numeric (count)] 
    
    # Count occurrences per count value  
   dt_long  <-   
     dt_long[, .( n =  .N), by  =  .(type, count)] 
    
    # Calculate total count of unique SNPs  
   total_SNP  <-   
      length ( unique (dt $ SNP_id)) 
    
    # Add a new column for the percentage  
   dt_long[, perc  :=  n  /  total_SNP  *   100 ] 
    
    # Define new labels  
   new_labels  <-  
      c ( 
      "Reference Allele"   =   "REF_mismatch" , 
      "Alternative Allele"   =   "ALT_mismatch" , 
      "Zygosity Mismatch"   =   "Zigo_mismatch"  
   ) 
    
    # Apply new labels  
   dt_long $ type  <-  
      fct_recode (dt_long $ type,  !!! new_labels) 
    
    # import plotting theme  
    source ( 
      here ( 
        "scripts" , 
        "analysis" , 
        "my_theme2.R"   # choose my_theme.R (Roboto Condensed) or my_theme2.R (default font)  
     ) 
   ) 
    
    # Create facet histogram  
    ggplot (dt_long,  aes ( x =  count,  y =  n))  +  
      geom_bar ( 
        stat =   "identity" , 
        fill =   "#ffcae4" , 
        color =   ifelse ( 
         dt_long $ count  ==   0 , 
          "#CCFF00" , 
          ifelse (dt_long $ count  ==   1 ,  "#4169E1" ,  "#FF7F50" ) 
       ), 
        width =   0.6 , 
        linewidth =   1  
     )  +  
      geom_text_repel ( aes ( label =   paste0 ( 
       scales ::  comma (n),  " (" ,  round (perc,  2 ),  "%)"  
     )),  size =   2.7 ,  color =   "gray10" )  +  
      facet_wrap ( ~  type,  scales =   "free_y" )  +  
      labs ( 
        title =   "Histogram of SNP Mismatch Counts across the 18 samples" , 
        x =   "Count" , 
        y =   "Frequency" , 
        caption =   "Comparison of the genotypes of 90,834 SNPs using default and crosses priors.  \n   12,030 SNPs (13.24%) have discrepancies in at least 1 sample.  \n   Bar border colors: Electric Lime = no errors; Royal Blue =  1 error; Coral = more than 1 error"  
     )  +  
      scale_y_continuous ( labels =  scales :: comma)  +  
      scale_x_continuous ( breaks =   0  :  18 )  +  
      my_theme ()  +  
      coord_flip ()  +  
      theme ( plot.caption =   element_text ( 
        face =   "italic" , 
        size =   10 , 
        color =   "grey20"  
     ))    
   
       # save the plot  
    ggsave ( 
      here ( 
        "output" , 
        "wgs_vs_chip" , 
        "figures" , 
        "default_cross_priors_mismatches.pdf"  
     ), 
      width  =   8 , 
      height =   6 , 
      units  =   "in"  
   )    
 
 
 6.2 Within KAT and SAI 
 Now we can create columns to get the same statistics for each
population “SAI” and “KAT”. 
 Lets check the first SNP 
       # the original data  
   data_ab_dt  |>  
     dplyr ::  filter (SNP_id  ==   "AX-579436089" )    
  ## Empty data.table (0 rows and 151 cols): SNP_id,KAT_9a_geno,KAT_9b_geno,KAT_9a_KAT_9b_gcomp,KAT_9a_zygo,KAT_9b_zygo...  
       # or the data table  
   dt  |>  
     dplyr ::  filter (SNP_id  ==   "AX-579436089" )    
  ## Empty data.table (0 rows and 4 cols): SNP_id,REF_mismatch,ALT_mismatch,Zigo_mismatch  
 We have SAI_ and KAT_; we can subset the data and compare the two
populations. 
 Check SAI 
       # Convert your data to a data.table  
    # setDT(data_ab_dt)  
    
    # Extract SAI and KAT columns  
   SAI_cols  <-   grep ( "^SAI_" ,  names (data_ab_dt),  value =   TRUE ) 
   KAT_cols  <-   grep ( "^KAT_" ,  names (data_ab_dt),  value =   TRUE ) 
    
    # Subset the data into two data tables for SAI and KAT  
   data_SAI  <-  data_ab_dt[,  c ( 'SNP_id' , SAI_cols), with  =   FALSE ] 
   data_KAT  <-  data_ab_dt[,  c ( 'SNP_id' , KAT_cols), with  =   FALSE ] 
    
    # SAI  
    # Create columns for match and mismatch count for columns ending with _REF  
   cols_REF  <-  
      grep ( "_REF$" ,  names (data_SAI),  value =   TRUE ) 
    
    # Calculate the count of "match" or "mismatch" for each row  
   data_SAI[,  c ( "REF_match_count" ,  "REF_mismatch_count" )  :=  
              .( rowSums (.SD  ==   "match" ,  na.rm =   TRUE ), 
                 rowSums (.SD  ==   "mismatch" ,  na.rm =   TRUE )), 
            .SDcols  =  cols_REF] 
    
    # Create columns for match and mismatch count for columns ending with _ALT  
   cols_ALT  <-  
      grep ( "_ALT$" ,  names (data_SAI),  value =   TRUE ) 
    
    # Calculate the count of "match" or "mismatch" for each row  
   data_SAI[,  c ( "ALT_match_count" ,  "ALT_mismatch_count" )  :=  
              .( rowSums (.SD  ==   "match" ,  na.rm =   TRUE ), 
                 rowSums (.SD  ==   "mismatch" ,  na.rm =   TRUE )), 
            .SDcols  =  cols_ALT] 
    
    # Create columns for match and mismatch count for columns ending with _zcomp  
   cols_Zigo  <-  
      grep ( "_zcomp$" ,  names (data_SAI),  value =   TRUE ) 
    
    # Calculate the count of "match" or "mismatch" for each row  
   data_SAI[,  c ( "Zigo_match_count" ,  "Zigo_mismatch_count" )  :=  
              .( rowSums (.SD  ==   "match" ,  na.rm =   TRUE ), 
                 rowSums (.SD  ==   "mismatch" ,  na.rm =   TRUE )), 
            .SDcols  =  cols_Zigo] 
    
    # Now, you can summarize this for each SNP_id  
   summary_sai  <-  
     data_SAI[, .( 
        REF_match =   sum (REF_match_count,  na.rm =   TRUE ), 
        REF_mismatch =   sum (REF_mismatch_count,  na.rm =   TRUE ), 
        ALT_match =   sum (ALT_match_count,  na.rm =   TRUE ), 
        ALT_mismatch =   sum (ALT_mismatch_count,  na.rm =   TRUE ), 
        Zigo_match =   sum (Zigo_match_count,  na.rm =   TRUE ), 
        Zigo_mismatch =   sum (Zigo_mismatch_count,  na.rm =   TRUE ) 
     ), 
     by  =  SNP_id] 
    
    # Sort data by SNP_id  
    setorder (summary_sai, SNP_id) 
    
    # Check the result  
    head (summary_sai)    
  ##          SNP_id REF_match REF_mismatch ALT_match ALT_mismatch Zigo_match
## 1: AX-579436125        12            0        12            0         12
## 2: AX-579436196        10            0        10            0         10
## 3: AX-579436243        10            2        12            0         10
## 4: AX-579436298        11            0        11            0         11
## 5: AX-579436308        10            0        10            0         10
## 6: AX-579436317        12            0        12            0         12
##    Zigo_mismatch
## 1:             0
## 2:             0
## 3:             2
## 4:             0
## 5:             0
## 6:             0  
 Now KAT 
       # KAT  
    # Create columns for match and mismatch count for columns ending with _REF  
   cols_REF  <-  
      grep ( "_REF$" ,  names (data_KAT),  value =   TRUE ) 
    
    # Calculate the count of "match" or "mismatch" for each row  
   data_KAT[,  c ( "REF_match_count" ,  "REF_mismatch_count" )  :=  
              .( rowSums (.SD  ==   "match" ,  na.rm =   TRUE ), 
                 rowSums (.SD  ==   "mismatch" ,  na.rm =   TRUE )), 
            .SDcols  =  cols_REF] 
    
    # Create columns for match and mismatch count for columns ending with _ALT  
   cols_ALT  <-  
      grep ( "_ALT$" ,  names (data_KAT),  value =   TRUE ) 
    
    # Calculate the count of "match" or "mismatch" for each row  
   data_KAT[,  c ( "ALT_match_count" ,  "ALT_mismatch_count" )  :=  
              .( rowSums (.SD  ==   "match" ,  na.rm =   TRUE ), 
                 rowSums (.SD  ==   "mismatch" ,  na.rm =   TRUE )), 
            .SDcols  =  cols_ALT] 
    
    # Create columns for match and mismatch count for columns ending with _zcomp  
   cols_Zigo  <-  
      grep ( "_zcomp$" ,  names (data_KAT),  value =   TRUE ) 
    
    # Calculate the count of "match" or "mismatch" for each row  
   data_KAT[,  c ( "Zigo_match_count" ,  "Zigo_mismatch_count" )  :=  
              .( rowSums (.SD  ==   "match" ,  na.rm =   TRUE ), 
                 rowSums (.SD  ==   "mismatch" ,  na.rm =   TRUE )), 
            .SDcols  =  cols_Zigo] 
    
    # Now, you can summarize this for each SNP_id  
   summary_kat  <-  
     data_KAT[, .( 
        REF_match =   sum (REF_match_count,  na.rm =   TRUE ), 
        REF_mismatch =   sum (REF_mismatch_count,  na.rm =   TRUE ), 
        ALT_match =   sum (ALT_match_count,  na.rm =   TRUE ), 
        ALT_mismatch =   sum (ALT_mismatch_count,  na.rm =   TRUE ), 
        Zigo_match =   sum (Zigo_match_count,  na.rm =   TRUE ), 
        Zigo_mismatch =   sum (Zigo_mismatch_count,  na.rm =   TRUE ) 
     ), 
     by  =  SNP_id] 
    
    # Sort data by SNP_id  
    setorder (summary_kat, SNP_id) 
    
    # Check output  
    head (summary_kat)    
  ##          SNP_id REF_match REF_mismatch ALT_match ALT_mismatch Zigo_match
## 1: AX-579436125         6            0         6            0          6
## 2: AX-579436196         6            0         6            0          6
## 3: AX-579436243         5            1         6            0          5
## 4: AX-579436298         6            0         6            0          6
## 5: AX-579436308         6            0         6            0          6
## 6: AX-579436317         6            0         6            0          6
##    Zigo_mismatch
## 1:             0
## 2:             0
## 3:             1
## 4:             0
## 5:             0
## 6:             0  
 Make plot to visualize the output 
 First lets get statistics to add to the plot caption. I tried two
codes to make sure we get the right output: 
 How many SNPs have discrepancies in the genotypes in 1 or more
samples for KAT? 
 Code 1 
       # Discrepancies in 2 or more samples, we use or operator |  
   failed_kat_ab  <-  
     data_KAT  |>  
     dplyr ::  filter (REF_mismatch_count  >   0   |  
                     ALT_mismatch_count  >   0   |  Zigo_mismatch_count  >   0 ) 
    # How many SNPs we tested  
   tested_snps  <-  
      length ( unique (data_KAT $ SNP_id)) 
    cat ( "Number of SNPs tested:" , tested_snps,  "  \n  " )    
  ## Number of SNPs tested: 90542  
       # How many SNPs failed  
   failed_snps_kat_ab  <-  
      length ( unique (failed_kat_ab $ SNP_id)) 
    cat ( "Number of SNPs failed:" , failed_snps_kat_ab,  "  \n  " )    
  ## Number of SNPs failed: 2773  
       # Calculate percentage  
   percentage_failed_kat_ab  <-  
      round (failed_snps_kat_ab  /  tested_snps  *   100 ,  2 ) 
    cat ( "Percentage of failed SNPs:" , percentage_failed_kat_ab,  "%  \n  " )    
  ## Percentage of failed SNPs: 3.06 %  
 Code 2 
       # Discrepancies in 1 or more samples  
    # How many SNPs we tested  
   tested_snps  <-   length ( unique (data_KAT $ SNP_id)) 
    cat ( "Number of SNPs tested:" , tested_snps,  "  \n  " )    
  ## Number of SNPs tested: 90542  
       # How many SNPs failed  
   failed_kat_ab  <-  
      length ( unique (data_KAT[data_KAT $ REF_mismatch_count  >   0   |  
                              data_KAT $ ALT_mismatch_count  >   0   |  
                              data_KAT $ Zigo_mismatch_count  >   0 , ] $ SNP_id)) 
    cat ( "Number of SNPs failed:" , failed_kat_ab,  "  \n  " )    
  ## Number of SNPs failed: 2773  
       # Calculate percentage  
   percentage_failed  <-   round (failed_kat_ab  /  tested_snps  *   100 ,  2 ) 
    cat ( "Percentage of failed SNPs:" , percentage_failed,  "%  \n  " )    
  ## Percentage of failed SNPs: 3.06 %  
 How many SNPs have discrepancies in the genotypes in 1 or more
samples for SAI 
 Code 1 
       # Discrepancies in 2 or more samples, we use or operator |  
   failed_sai_ab  <-  
     data_SAI  |>  
     dplyr ::  filter (REF_mismatch_count  >   0   |  
                     ALT_mismatch_count  >   0   |  Zigo_mismatch_count  >   0 ) 
    
    # How many SNPs we tested  
   tested_snps  <-  
      length ( unique (data_SAI $ SNP_id)) 
    cat ( "Number of SNPs tested:" , tested_snps,  "  \n  " )    
  ## Number of SNPs tested: 90542  
       # How many SNPs failed  
   failed_snps_sai_ab  <-  
      length ( unique (failed_sai_ab $ SNP_id)) 
    cat ( "Number of SNPs failed:" , failed_snps_sai_ab,  "  \n  " )    
  ## Number of SNPs failed: 7532  
       # Calculate percentage  
   percentage_failed_sai_ab  <-  
      round (failed_snps_sai_ab  /  tested_snps  *   100 ,  2 ) 
    cat ( "Percentage of failed SNPs:" , percentage_failed_sai_ab,  "%  \n  " )    
  ## Percentage of failed SNPs: 8.32 %  
 Code 2 
       # Discrepancies in 1 or more samples  
    # How many SNPs we tested  
   tested_snps  <-  
      length ( unique (data_SAI $ SNP_id)) 
    cat ( "Number of SNPs tested:" , tested_snps,  "  \n  " )    
  ## Number of SNPs tested: 90542  
       # How many SNPs failed  
   failed_sai_ab  <-  
      length ( unique (data_SAI[data_SAI $ REF_mismatch_count  >   0   |  
                                data_SAI $ ALT_mismatch_count  >   0   |  
                                data_SAI $ Zigo_mismatch_count  >   0 ,] $ SNP_id)) 
    cat ( "Number of SNPs failed:" , failed_sai_ab,  "  \n  " )    
  ## Number of SNPs failed: 7532  
       # Calculate percentage  
   percentage_failed  <-   
      round (failed_sai_ab  /  tested_snps  *   100 ,  2 ) 
    cat ( "Percentage of failed SNPs:" , percentage_failed,  "%  \n  " )    
  ## Percentage of failed SNPs: 8.32 %  
 Both codes created the same output. 
 Data tidying and plotting 
       # import plotting theme  
    source ( 
      here ( 
        "scripts" , 
        "analysis" , 
        "my_theme2.R"   # choose my_theme.R (Roboto Condensed) or my_theme2.R (default font)  
     ) 
   ) 
    
    # Merge summary_sai and summary_kat  
   merged_sai_kat  <-  
      merge (summary_sai, 
           summary_kat, 
            by =   "SNP_id" , 
            suffixes =   c ( "_sai" ,  "_kat" )) 
    
    # Select only the relevant columns  
   dt  <-   
     merged_sai_kat[, .( 
     SNP_id, 
     REF_mismatch_sai, 
     ALT_mismatch_sai, 
     Zigo_mismatch_sai, 
     REF_mismatch_kat, 
     ALT_mismatch_kat, 
     Zigo_mismatch_kat 
   )] 
    
    
    # Reshape data to long format  
   dt_long  <-  
      melt (dt, 
           id.vars =   "SNP_id" , 
           variable.name =   "type" , 
           value.name =   "count" ) 
    
    # Convert to data.table if it's not already  
    setDT (dt_long) 
    
    # Extract the last part after "_" in the 'type' column to form 'group' column  
   dt_long[, group  :=   str_extract (type,  "(?<=_)[^_]+$" )] 
    
    # Extract the part before the first "_" in the 'type' column to form 'allele' column  
   dt_long[, allele  :=   str_extract (type,  "^[^_]+" )] 
    
    # Convert to numeric if it's not already  
   dt_long[, count  :=   as.numeric (count)] 
    
    # Count occurrences per count value  
   dt_long  <-  
     dt_long[, .( n =  .N), by  =  .(allele, group, count)] 
    # dt_long[, n := .N, by = .(allele, group, count)]  
    
    # Calculate total count of unique SNPs  
   total_SNP  <-  
      length ( unique (dt $ SNP_id)) 
    
    # Add a new column for the percentage  
   dt_long[, perc  :=  n  /  total_SNP  *   100 , by  =  group] 
    
    # Set levels for 'group' variable  
   dt_long $ group  <-  
      factor (dt_long $ group,  levels =   c ( "sai" ,  "kat" )) 
    
    # Set levels for 'allele' variable  
   dt_long $ allele  <-  
      factor (dt_long $ allele,  levels =   c ( "REF" ,  "ALT" ,  "Zigo" )) 
    
    # Modify levels for 'allele' variable  
    levels (dt_long $ allele)  <-  
      c ( "Reference Allele" ,  "Alternative Allele" ,  "Zygosity" ) 
    
    # Modify levels for 'group' variable  
    levels (dt_long $ group)  <-  
      c ( "SAI" ,  "KAT" ) 
    
   dt_long $ count  <-  
      as.numeric (dt_long $ count) 
    
    # Create plot  
    ggplot (dt_long,  aes ( x =  count,  y =  n))  +  
      geom_bar ( 
        stat =   "identity" , 
        fill =   "#ffcae4" , 
        color =   ifelse ( 
         dt_long $ count  ==   0 , 
          "#CCFF00" , 
          ifelse (dt_long $ count  ==   1 ,  "#4169E1" ,  "#FF7F50" ) 
       ), 
        width =   0.6 , 
        linewidth =   1  
     )  +  
      geom_text_repel ( aes ( label =   paste0 ( 
       scales ::  comma (n),  " (" ,  round (perc,  2 ),  "%)"  
     )),  size =   2.7 ,  color =   "gray10" )  +  
      facet_wrap ( ~  group  +  allele,  scales =   "free_y" ,  ncol =   3 )  +  
      labs ( 
        title =   "Histogram of SNP Mismatch Counts across all samples for each population" , 
        x =   "Count" , 
        y =   "Frequency" , 
        caption =   "Comparison of the genotypes of 90,834 SNPs using default and crosses priors.  \n   Number of genotype discordance in at least 1 sample for each sampling locality:  \n   KAT 6 samples from native range         SAI 12 samples from invasive range  \n   Bar border colors: Electric Lime = no errors; Royal Blue =  1 error; Coral = more than 1 error   \n  SAI: Saint Augustine, Trinidad and Tobago -> 9,619 SNPs (10.59%)  \n   KAT: Kathmandu, Nepal -> 4,165 SNPs (4.59%)"  
     )  +  
      coord_flip ()  +  
      my_theme ()  +  
      scale_y_continuous ( labels =  scales :: comma)  +  
      scale_x_continuous ( breaks =   0  :  18 )  +  
      theme ( plot.caption =   element_text ( 
        face =   "italic" , 
        size =   10 , 
        color =   "grey20"  
     ))    
   
       # save the plot  
    ggsave ( 
      here ( 
        "output" , 
        "wgs_vs_chip" , 
        "figures" , 
        "default_cross_priors_mismatches_SAI_KAT.pdf"  
     ), 
      width  =   8 , 
      height =   8 , 
      units  =   "in"  
   )    
 It seems that SAI has more mismatches but it has twice as many
samples than KAT. We can check the mismatches per sample. 
 
 
 6.3 Discrepancies per sample 
       # Initialize an empty list to hold the counts  
   count_list  <-   list () 
    
    # Select columns  
   matching_columns  <-   colnames (data_ab_dt)[ grepl ( pattern =   "(_REF$|_ALT$|_zcomp$)" ,  colnames (data_ab_dt))] 
    
    # Loop through each column  
    for  (column  in  matching_columns) { 
     match_count  <-  
        sum ( str_detect (data_ab_dt[[column]],  "match" ),  na.rm =   TRUE ) 
     mismatch_count  <-  
        sum ( str_detect (data_ab_dt[[column]],  "mismatch" ),  na.rm =   TRUE ) 
      
      # Create a data.table with counts for the current column  
     count_dt  <-  
        data.table ( Column =  column, 
                   Match =  match_count, 
                   Mismatch =  mismatch_count) 
      
      # Add the count data.table to the list  
     count_list[[column]]  <-  count_dt 
   } 
    
    # Combine all count data.tables into a single data.table  
   counts_all_columns  <-  
      rbindlist (count_list) 
    
    # Calculate total  
   counts_all_columns  <-  
     counts_all_columns  |>  
      mutate ( Total =  Match  +  Mismatch) 
    
    # Create new columns: Population, Sample, and Comparison  
   counts_all_columns  <-  
     counts_all_columns  |>  
      mutate ( 
        Population =   sub ( "^([^_]+).*" ,  "  \\  1" , Column), 
        Sample =   sub ( "^.*_(  \\  d+).*" ,  "  \\  1" , Column), 
        Comparison =   sub ( ".*_([^_]+)$" ,  "  \\  1" , Column) 
     ) 
    
    # Reorder the columns and create sample_id  
   counts_all_columns  <-  
     counts_all_columns  |>  
     dplyr ::  select (Population, Sample, Comparison, Match, Mismatch, Total) 
    
    # Calculate percentage columns  
   counts_all_columns  <-  
     counts_all_columns  |>  
      mutate ( Percent_Match =   round ((Match  /  Total)  *   100 ,  2 ), 
             Percent_Mismatch =   round ((Mismatch  /  Total)  *   100 ,  2 )) 
    
    # Replace zcomp with Zygosity  
   counts_all_columns $ Comparison  <-  
      gsub ( "zcomp" ,  "Zygosity" , counts_all_columns $ Comparison) 
    
    head (counts_all_columns)    
  ##    Population Sample Comparison Match Mismatch Total Percent_Match
## 1:        KAT      9   Zygosity 87964      775 88739         99.13
## 2:        SAI     15   Zygosity 87864     1064 88928         98.80
## 3:        SAI      3   Zygosity 87880     1025 88905         98.85
## 4:        KAT     12   Zygosity 87462      894 88356         98.99
## 5:        KAT      7   Zygosity 88177      810 88987         99.09
## 6:        SAI      2   Zygosity 87657     1148 88805         98.71
##    Percent_Mismatch
## 1:             0.87
## 2:             1.20
## 3:             1.15
## 4:             1.01
## 5:             0.91
## 6:             1.29  
 Make a plot 
       # import plotting theme  
    source ( 
      here ( 
        "scripts" , 
        "analysis" , 
        "my_theme2.R"   # choose my_theme.R (Roboto Condensed) or my_theme2.R (default font)  
     ) 
   ) 
    
    # Define color palette  
   color_palette  <-   c ( "#92C6FF" ,  "#f5cb8b" ,  "#bff28c" ) 
    
    # Convert Sample to numeric and sort samples numerically within each Population group  
   counts_all_columns $ Sample  <-  
      as.numeric (counts_all_columns $ Sample) 
   counts_all_columns  <-   
     counts_all_columns  |>  
      arrange (Population, Sample) 
    
    # Convert Sample column back to factor with sorted levels within each group  
   counts_all_columns $ Sample  <-  
      factor (counts_all_columns $ Sample, 
             levels =   unique (counts_all_columns $ Sample)) 
    
    
    # Rename and reorder Comparison column  
   counts_all_columns  <-  
     counts_all_columns  |>  
      mutate ( 
        Comparison_new =   recode ( 
         Comparison, 
          "REF"   =   "Reference Allele" , 
          "ALT"   =   "Alternative Allele" , 
          "Zygosity"   =   "Zygosity"  
       ) 
     )  |>  
      mutate ( Comparison_new =   factor ( 
       Comparison_new, 
        levels =   c ( "Reference Allele" ,  "Alternative Allele" ,  "Zygosity" ) 
     )) 
    
    # Create plot  
    ggplot (counts_all_columns, 
           aes ( x =  Sample,  y =  Mismatch,  fill =  Comparison))  +  
      geom_bar ( stat =   "identity" ,  position =   "dodge" )  +  
      facet_grid (Population  ~  Comparison_new, 
                 scales =   "free_y" , 
                 space =   "free" )  +  
      coord_flip ()  +  
      labs ( 
        title =   "SNP Mismatch Counts per Sample" , 
        x =   "Sample" , 
        y =   "Mismatches" , 
        caption =   "Genotyping errors per sample within each population using the default and the crosses priors."  
     )  +  
      # labs(x = "Sample", y = "Mismatch") +  
      theme ( panel.spacing =   unit ( 0.5 ,  "lines" ))  +  
      geom_text ( aes ( label =   paste0 ( 
       scales ::  comma (Mismatch),  " (" , Percent_Mismatch,  "%)"  
     )), 
      # position = position_dodge(width = 0.9),  
      hjust =   1 , 
      size =   2.5 )  +  
      scale_fill_manual ( values =  color_palette)  +  
      theme ( axis.text.x =   element_text ( angle =   45 ,  hjust =   1 ))  +  
      guides ( fill =   "none" )  +  
      my_theme ()  +  
      # theme(plot.margin = margin(10, 20, 10, 10)) +   # Increase right margin to prevent labels getting cut off  
      scale_y_continuous ( labels =  scales :: comma)  +    # Add thousands separator to y-axis labels  
      theme ( plot.caption =   element_text ( 
        face =   "italic" , 
        size =   10 , 
        color =   "grey20"  
     ))    
   
       # save the plot  
    ggsave ( 
      here ( 
        "output" , 
        "wgs_vs_chip" , 
        "figures" , 
        "default_cross_priors_mismatches_SAI_KAT_per_sample_stats.pdf"  
     ), 
      width  =   8 , 
      height =   7 , 
      units  =   "in"  
   )    
 We see that the number of mismatches are quite consistent across all
18 samples and there does not seem to be a bias towards native or
invasive ranges. What we have to decide now is what is random and we can
accept and what we need to filter out to avoid problems in our
downstream analyses. 
 
 
 6.4 Save the data to load later 
       # Save the data 18 samples  
    saveRDS ( 
     summary_18_samples, 
      file =   here ( 
        "output" , 
        "wgs_vs_chip" , 
        "summary_18_samples.rds"  
     ) 
   ) 
    
    # Save the data KAT  
    saveRDS ( 
     summary_kat, 
      file =   here ( 
        "output" , 
        "wgs_vs_chip" , 
        "summary_kat.rds"  
     ) 
   ) 
    
    
    # Save the data SAI  
    saveRDS ( 
     summary_sai, 
      file =   here ( 
        "output" , 
        "wgs_vs_chip" , 
        "summary_sai.rds"  
     ) 
   ) 
    
    
    # Save the data  
    saveRDS ( 
     counts_all_columns, 
      file =   here ( 
        "output" , 
        "wgs_vs_chip" , 
        "counts_all_columns.rds"  
     ) 
   ) 
    
    # Save the data  
    saveRDS ( 
     data_ab_dt, 
      file =   here ( 
        "output" , 
        "wgs_vs_chip" , 
        "data_ab_dt.rds"  
     ) 
   )    
 
 
 6.5 SNPs with errors in 2 or more samples 
 We can compare the SNP with 2 or more samples with discrepancies with
the SNPs that did not pass our segregation test. 
       # Load the data  
   data_ab_dt  <-  
      readRDS ( 
        file =   here ( 
          "output" , 
          "wgs_vs_chip" , 
          "data_ab_dt.rds"  
       ) 
     )    
 Get the SNPs that have errors in 2 or more samples 
       # Discrepancies in 2 or more samples  
    # How many SNPs we tested  
   tested_snps  <-   length ( unique (data_ab_dt $ SNP_id)) 
    cat ( "Number of SNPs tested:" , tested_snps,  "  \n  " )    
  ## Number of SNPs tested: 90542  
       # How many SNPs failed  
   failed_snpsR  <-  
      length ( 
        unique (data_ab_dt[data_ab_dt $ REF_mismatch_count  >=   2 ,] $ SNP_id 
              ) 
            ) 
    cat ( "REF mismatch at in 2 samples:" , failed_snpsR,  "  \n  " )    
  ## REF mismatch at in 2 samples: 2657  
       # How many SNPs failed  
   failed_snpsA  <-  
      length ( 
        unique (data_ab_dt[data_ab_dt $ ALT_mismatch_count  >=   2 ,] $ SNP_id 
              ) 
            ) 
    cat ( "ALT mismatch at least in 2 samples:" , failed_snpsA,  "  \n  " )    
  ## ALT mismatch at least in 2 samples: 1286  
       # How many SNPs failed zygosity  
   failed_snps  <-  
      length ( 
        unique (data_ab_dt[data_ab_dt $ Zigo_mismatch_count  >=   2 ,] $ SNP_id 
              ) 
            ) 
    cat ( "Zygosity mismatch in at least 2 samples:" , failed_snps,  "  \n  " )    
  ## Zygosity mismatch in at least 2 samples: 3936  
       # Calculate percentage  
   percentage_failed  <-   round (failed_snps  /  tested_snps  *   100 ,  2 ) 
    cat ( "Percentage of failed SNPs in 2 or more samples:" , percentage_failed,  "%  \n  " )    
  ## Percentage of failed SNPs in 2 or more samples: 4.35 %  
 Get the SNP ids 
      failed_snps_ids  <-  
      unique ( 
       data_ab_dt[data_ab_dt $ Zigo_mismatch_count  >=   2 , ] $ SNP_id 
       ) 
    
    # Define the file path  
   file_path  <-   here ( "output" , 
                      "wgs_vs_chip" , 
                      "SNPs_failed_2_samples.txt" ) 
    
    # Write unique SNPs to the file  
    writeLines (failed_snps_ids,  con =  file_path)    
 
 
 6.6 Venn diagram fail Mendel and mismatches 
 Create a Venn diagram between the SNPs with genotyping mismatches and
those that failed our segregation test 
       # Read in the two files as vectors  
   fail_mendel  <-  
      read_table ( 
        here ( 
         "output" ,  
         "segregation" , 
         "albopictus" , 
         "albopictus_SNPs_fail_segregation.txt"  
       ), 
        col_names =   FALSE , 
        show_col_types =   FALSE  
       )[[ 1 ]] 
    
   fail_geno  <-  
      read_table ( 
        here ( 
         "output" ,  
         "wgs_vs_chip" , 
         "SNPs_failed_2_samples.txt"  
       ), 
        col_names =   FALSE , 
        show_col_types =   FALSE  
       )[[ 1 ]] 
    
    # Calculate shared values  
   errors_SNPs  <-  
      intersect ( 
       fail_mendel, 
       fail_geno 
     ) 
    
    
    # Create Venn diagram  
   venn_data  <-  
      list ( 
        "Fail Mendel"   =  fail_mendel, 
        "Genotype Mismatches"   =  fail_geno 
     ) 
   venn_plot  <-  
      ggvenn ( 
       venn_data, 
        fill_color =   c ( "steelblue" ,  "darkorange" ), 
        show_percentage =   TRUE  
     ) 
    
    # Add a title  
   venn_plot  <-  
     venn_plot  +  
      ggtitle ( "Comparison of SNPs with errors" )  +  
      theme ( plot.title =   element_text ( hjust =  . 5 )) 
    
    # Display the Venn diagram  
    print (venn_plot)    
   
       # Save Venn diagram to PDF  
   output_path  <-  
      here ( 
        "output" , 
        "wgs_vs_chip" , 
        "figures" , 
        "Mendel_geno_priors.pdf"  
     ) 
    ggsave ( 
     output_path, 
     venn_plot, 
      height =   6 , 
      width =   6 , 
      dpi =   300  
   )    
 
 
 6.8 PCA before and after removing SNPs 
 We can prepare a PCA before and after removing the SNPs with errors.
First let’s combine the two vectors with the SNP ids with errors 
       # Combine vectors  
   combined_errors  <-  
      unique ( c (fail_mendel, 
              fail_geno)) 
    
    # Write to file  
    write.table ( 
     combined_errors, 
      file =   here ( 
        "output" , 
        "wgs_vs_chip" , 
        "SNPs_with_errors.txt"  
     ), 
      row.names =   FALSE , 
      col.names =   FALSE , 
      quote =   FALSE  
   )    
 Now use Plink to create PCA excluding only the SNPs that failed our
segregation test 
 Lets import our .fam file to filter the IDs we want to compare. 
       # Read the data  
   fam_data  <-  
      here ( "output" ,  "wgs_vs_chip" ,  "wgs_chip.fam" )  |>  
      read_delim ( 
        delim =   "  \t  " , 
        col_names =   FALSE , 
        show_col_types =   FALSE  
      )  |>  
      setNames ( 
        c ( 
          "FID" ,  "IID" ,  "PID" ,  "MID" ,  "Sex" ,  "Phenotype"  
         ) 
       ) 
    
    # Filter the data  
   filtered_data  <-  
     fam_data  |>  
     dplyr ::  filter (stringr ::  str_detect (IID,  "a$|b$" ))  |>  
     dplyr ::  select ( "FID" ,  "IID" ) 
    
    # Save to file  
    write.table ( 
     filtered_data, 
      file =   here ( "output" ,  "wgs_vs_chip" ,  "samples_priors.txt" ), 
      quote =   FALSE , 
      sep =   " " , 
      row.names =   FALSE , 
      col.names =   FALSE  
   )    
 Use Plink with only the samples we are comparing (priors) and remove
SNPs that failed Mendel test 
       # Before  
    plink   \  
   --allow-extra-chr  \  
   --keep-allele-order  \  
   --bfile output/wgs_vs_chip/wgs_chip  \  
   --exclude output/segregation/albopictus/albopictus_SNPs_fail_segregation.txt  \  
   --keep output/wgs_vs_chip/samples_priors.txt  \  
   --pca  \  
   --geno 0.1  \  
   --maf 0.05  \  
   --out output/wgs_vs_chip/priors_pca_1  \  
   --silent    
 Now do it again but remove both SNPs that failed Mendel test and that
have genotype mismatches in at least 2 samples (plus those with
segregation errors). 
       # After  
    plink   \  
   --allow-extra-chr  \  
   --keep-allele-order  \  
   --bfile output/wgs_vs_chip/wgs_chip  \  
   --exclude output/wgs_vs_chip/SNPs_with_errors.txt  \  
   --keep output/wgs_vs_chip/samples_priors.txt  \  
   --pca  \  
   --geno 0.1  \  
   --maf 0.05  \  
   --out output/wgs_vs_chip/priors_pca_2  \  
   --silent    
 Create PCA plot 
       # Load the PCA results  
   pca_1  <-  
      read.table ( here ( "output" ,  "wgs_vs_chip" ,  "priors_pca_1.eigenvec" ), 
                 header =   FALSE ) 
    colnames (pca_1)  <-   c ( "FID" ,  "IID" ,  paste0 ( "PC" ,  1  : ( ncol (pca_1)  -   2 ))) 
   pca_1 $ analysis  <-   "Before"  
   pca_1 $ group  <-   ifelse ( 
     stringr ::  str_detect (pca_1 $ IID,  "a$" ), 
      "a" , 
      ifelse (stringr ::  str_detect (pca_1 $ IID,  "b$" ),  "b" ,  "Other" ) 
   ) 
    
   pca_2  <-  
      read.table ( here ( "output" ,  "wgs_vs_chip" ,  "priors_pca_2.eigenvec" ), 
                 header =   FALSE ) 
    colnames (pca_2)  <-   c ( "FID" ,  "IID" ,  paste0 ( "PC" ,  1  : ( ncol (pca_2)  -   2 ))) 
   pca_2 $ analysis  <-   "After"  
   pca_2 $ group  <-   ifelse ( 
     stringr ::  str_detect (pca_2 $ IID,  "a$" ), 
      "a" , 
      ifelse (stringr ::  str_detect (pca_2 $ IID,  "b$" ),  "b" ,  "Other" ) 
   ) 
    
    # Combine the data  
   combined_pca  <-   rbind (pca_1, pca_2) 
    
    # import plotting theme  
    source ( 
      here ( 
        "scripts" , 
        "analysis" , 
        "my_theme2.R"  
     ) 
   ) 
    
    # Convert the 'analysis' column to a factor and specify the level order  
   combined_pca $ analysis  <-   
      factor (combined_pca $ analysis,  levels =   c ( "Before" ,  "After" )) 
    
    # Create a facet plot  
    ggplot (combined_pca,  aes ( x =  PC1,  y =  PC2,  color =  group,  shape =  group))  +  
      geom_point ( size =   2 )  +  
      facet_grid (FID  ~  analysis,  scales =   "free" )  +  
      # geom_text_repel(aes(label = IID), size = 3, max.overlaps = Inf) +   
      labs ( 
        x =   "PC1" , 
        y =   "PC2" , 
        title =   "The effect of SNPs with genotyping mismatches in 2 or more samples" , 
        colour =   "Prior" , 
        shape =   "Prior" , 
        caption =   "Removing SNPs with genotypes errors in at least 2 samples.   \n  'Before' with 71,144 SNPs 'After' with 66,485 SNPs (--maf 0.05 and --geno 0.1)."  
     )  +  
      my_theme ()  +  
      scale_color_manual ( 
        values =   c ( 
          "a"   =   "lightblue" , 
          "b"   =   "orange" , 
          "Other"   =   "black"  
       ), 
        labels =   c ( "a"   =   "Default" ,  "b"   =   "Crosses" ,  "Other"   =   "Other" ) 
     )  +  
      theme ( plot.caption =   element_text ( 
        face =   "italic" , 
        size =   10 , 
        color =   "grey20"  
     ), 
      legend.position =   "top" )  +  
      scale_shape_manual ( 
        values =   c ( 
          "a"   =   19 ,   # Filled circle  
          "b"   =   1 ,   # Open circle  
          "Other"   =   3    # Plus  
       ), 
        labels =   c ( "a"   =   "Default" ,  "b"   =   "Crosses" ,  "Other"   =   "Other" ) 
     )    
   
       # Save plot to PDF  
    ggsave ( 
      here ( 
        "output" , 
        "wgs_vs_chip" , 
        "figures" , 
        "PCA_before_after_remove_SNPs_errors_2_or_more_samples.pdf"  
     ), 
      height =   6 , 
      width =   6 , 
      dpi =   300  
   )    
 We can remove all SNPs with errors and then we would have a perfect
overlap of the points. The frequencies and genotypes would be all the
same. It is interesting to know that we can see the effect of few
thousand SNPs (~ 6k) that have 1 genotype wrong in 1 sample out of the
18 samples. 
 
 
 
 7. New genotype calls for WGS data on the cluster 
 Because we extracted the genotypes of the WGS samples from the output
of the genotype call using 819 genomes, with KAT and SAI having more
samples than we are analyzing here, I will re-do the genotype call using
only the samples we have here. Then we can compare the results. One
would think that it is okay to subset a dataset and compare it to
another one. However, since we used ANGSD doing the genotype calls using
all samples, we have the opportunity to compare the outcomes. 
 We can use the “filtered_data” object to get the sample IDs we
need. 
       # Removing 'a' and 'b' from IID column  
   samples_wgs  <-   
     filtered_data  |>  
      mutate ( IID =   str_remove_all (IID,  "[ab]" ))  |>  
     dplyr ::  select (FID, IID)  |>  
      distinct () 
    
    # Get the number of samples  
    length (samples_wgs $ IID)    
  ## [1] 18  
 Check the wgs samples 
      samples_wgs    
  ## # A tibble: 18 × 2
##    FID   IID  
##    <chr> <chr>
##  1 KAT   7    
##  2 KAT   8    
##  3 KAT   9    
##  4 KAT   10   
##  5 KAT   11   
##  6 KAT   12   
##  7 SAI   1    
##  8 SAI   2    
##  9 SAI   3    
## 10 SAI   4    
## 11 SAI   5    
## 12 SAI   12   
## 13 SAI   13   
## 14 SAI   14   
## 15 SAI   15   
## 16 SAI   16   
## 17 SAI   17   
## 18 SAI   18  
 We have a total of 30 samples for KAT + SAI 
       ls   -1   * .cram  |   wc   -l  
    # 30     
 The name of the wgs samples on the cluster 
       # all 30 samples for genotype call  
    # Kathmandu_Nepal_F_10.cram  
    # Kathmandu_Nepal_F_11.cram  
    # Kathmandu_Nepal_F_12.cram  
    # Kathmandu_Nepal_F_7.cram  
    # Kathmandu_Nepal_F_8.cram  
    # Kathmandu_Nepal_F_9.cram  
    # Kathmandu_Nepal_M_1.cram  
    # Kathmandu_Nepal_M_2.cram  
    # Kathmandu_Nepal_M_3.cram  
    # Kathmandu_Nepal_M_4.cram  
    # Kathmandu_Nepal_M_5.cram  
    # Kathmandu_Nepal_M_6.cram  
    # StAugustine_Trinidad_F_12.cram  
    # StAugustine_Trinidad_F_13.cram  
    # StAugustine_Trinidad_F_14.cram  
    # StAugustine_Trinidad_F_15.cram  
    # StAugustine_Trinidad_F_16.cram  
    # StAugustine_Trinidad_F_17.cram  
    # StAugustine_Trinidad_F_18.cram  
    # StAugustine_Trinidad_F_1.cram  
    # StAugustine_Trinidad_F_2.cram  
    # StAugustine_Trinidad_F_3.cram  
    # StAugustine_Trinidad_F_4.cram  
    # StAugustine_Trinidad_F_5.cram  
    # StAugustine_Trinidad_F_6.cram  
    # StAugustine_Trinidad_M_10.cram  
    # StAugustine_Trinidad_M_11.cram  
    # StAugustine_Trinidad_M_7.cram  
    # StAugustine_Trinidad_M_8.cram  
    # StAugustine_Trinidad_M_9.cram  
    
    
    # we will do a genotype call with the 18 samples  
    # Kathmandu_Nepal_F_10.cram  
    # Kathmandu_Nepal_F_11.cram  
    # Kathmandu_Nepal_F_12.cram  
    # Kathmandu_Nepal_F_7.cram  
    # Kathmandu_Nepal_F_8.cram  
    # Kathmandu_Nepal_F_9.cram  
    # StAugustine_Trinidad_F_1.cram  
    # StAugustine_Trinidad_F_2.cram  
    # StAugustine_Trinidad_F_3.cram  
    # StAugustine_Trinidad_F_4.cram  
    # StAugustine_Trinidad_F_5.cram  
    # StAugustine_Trinidad_F_12.cram  
    # StAugustine_Trinidad_F_13.cram  
    # StAugustine_Trinidad_F_14.cram  
    # StAugustine_Trinidad_F_15.cram  
    # StAugustine_Trinidad_F_16.cram  
    # StAugustine_Trinidad_F_17.cram  
    # StAugustine_Trinidad_F_18.cram     
 On the cluster the data is at
/ycga-gpfs/project/caccone/lvc26/september_2020/crams 
 We can do two genotype calls. One with all samples and one with the
samples (30) we genotyped with the chip (18). Then, we can compare the
results with the extracted genotypes of the 18 samples. We extracted it
from a file that we created using angsd and 819 samples. 
 We can use the same script that we used for the genotype calls, but
change the samples and the sites file (use only the one we have in the
chip). 
 To create a sites file we can use the .bim file of the wgs data with
all the sites we have in the chip (175k) 
 
 7.1 Batch scripts 
 Here is a batch script I used for the genotype calls 
       #!/bin/sh  
    #SBATCH --mail-type=  BEGIN  ,  END  ,FAIL            
    #SBATCH --mail-user=luciano.cosme@yale.edu   
    #SBATCH --ntasks=1  
    #SBATCH --cpus-per-task=20            
    #SBATCH --mem-per-cpu=6gb                    
    #SBATCH --time=120:00:00   
    #SBATCH --array=1-819  
    #SBATCH --job-name=angsd_chr  
    #SBATCH -o angsd_chr.%A_%a.o.txt  
    #SBATCH -e angsd_chr.%A_%a.ERROR.txt  
    
    cd  /gpfs/ycga/project/caccone/lvc26/september_2020/snp_calls/chunk_calls 
    
    samplesheet  =  "scaffolds.txt"  
    
    threads  =  $SLURM_JOB_CPUS_PER_NODE  
    
    name  =  `  sed   -n   "  $SLURM_ARRAY_TASK_ID  " p  $samplesheet   |    awk   '{print $1}'  `  
    
    /home/lvc26/project/angsd/angsd   \  
   -ref /gpfs/ycga/project/caccone/lvc26/september_2020/genome/aedes_albopictus_LA2_20200826.fasta  \  
   -bam /gpfs/ycga/project/caccone/lvc26/september_2020/snp_calls/bams.txt  \  
   -nThreads 40  \  
   -r  $name   \  
   -gl 1  \  
   -dopost 1  \  
   -doMaf 2  \  
   -doMajorMinor 4  \  
   -minMapQ 20  \  
   -minQ 10  \  
   -remove_bads 1  \  
   -uniqueOnly 1  \  
   -sites /gpfs/ycga/project/caccone/lvc26/september_2020/sites/cat/intersects/shared/shared_sites.txt  \  
   -doCounts 1  \  
   -setMinDepthInd 10  \  
   -minInd 2  \  
   -SNP_pval 1e-6  \  
   -doPlink 2  \  
   -doGeno 4  \  
   -capDepth 45  \  
   -minMaf 0.01  \  
   -out  $name     
 We need to create two lists of cram files and a new sites file. 
 Create new sites file. First, check the .bim file 
       head  output/wgs_vs_chip/wgs_01.bim    
  ## 1.1  AX-581444870    0   97856   C   T
## 1.1  AX-583033226    0   161729  A   G
## 1.1  AX-583035067    0   229640  T   A
## 1.1  AX-583035083    0   305518  A   G
## 1.1  AX-583035102    0   308124  A   G
## 1.1  AX-583033340    0   311920  G   A
## 1.1  AX-583033342    0   315059  C   G
## 1.1  AX-583035163    0   315386  A   G
## 1.1  AX-583033356    0   315674  C   T
## 1.1  AX-583033370    0   330057  G   A  
 We can get the first (chromosome) and forth column (position) to
create a sites file. Check how many SNPs we have in the .bim file 
       wc   -l  output/wgs_vs_chip/wgs_01.bim    
  ##   175360 output/wgs_vs_chip/wgs_01.bim  
 We can use “awk” to do what we need 
       awk   '{print "chr"$1, $4}'  output/wgs_vs_chip/wgs_01.bim  >  output/wgs_vs_chip/new_calls/wgs_sites.txt ;  
    head  output/wgs_vs_chip/new_calls/wgs_sites.txt    
  ## chr1.1 97856
## chr1.1 161729
## chr1.1 229640
## chr1.1 305518
## chr1.1 308124
## chr1.1 311920
## chr1.1 315059
## chr1.1 315386
## chr1.1 315674
## chr1.1 330057  
 The reference genome that I used had “chr” before the scaffold names.
We need to use it to match the genome. It is easy to remove or add
it. 
 We can create a file with the SNP id that ANGSD creates
(chromosome_position) 
  chr1.1 chr1.1_97856
chr1.1 chr1.1_161729
chr1.1 chr1.1_229640
chr1.1 chr1.1_305518
chr1.1 chr1.1_308124
chr1.1 chr1.1_311920
chr1.1 chr1.1_315059
chr1.1 chr1.1_315386  
       awk   -v  OFS= '\t'   '{$6="chr"$1 "_" $4; $7="chr" $1; print $1, $7, $4, $6, $2}'  output/wgs_vs_chip/wgs_01.bim  >  output/wgs_vs_chip/new_calls/wgs_snps_ids.txt ;  
    head  output/wgs_vs_chip/new_calls/wgs_snps_ids.txt    
  ## 1.1  chr1.1  97856   chr1.1_97856    AX-581444870
## 1.1  chr1.1  161729  chr1.1_161729   AX-583033226
## 1.1  chr1.1  229640  chr1.1_229640   AX-583035067
## 1.1  chr1.1  305518  chr1.1_305518   AX-583035083
## 1.1  chr1.1  308124  chr1.1_308124   AX-583035102
## 1.1  chr1.1  311920  chr1.1_311920   AX-583033340
## 1.1  chr1.1  315059  chr1.1_315059   AX-583033342
## 1.1  chr1.1  315386  chr1.1_315386   AX-583035163
## 1.1  chr1.1  315674  chr1.1_315674   AX-583033356
## 1.1  chr1.1  330057  chr1.1_330057   AX-583033370  
 We can use this file to replace the SNP ids that we will get with
ANGSD. 
 We can add the SNP ids (AX-) to our file to convert between the two
SNP names. We can use the position as reference when replacing the SNP
id that ANGSD creates and the ones we have in the chip. 
 Since we are using only 175k sites instead of over 300 million when
we did a genotype call, we do not need to split the genome into chunks
or scaffolds. We can do a genotype call for the entire genome. 
 Index the sites file with ANGSD on the cluster 
       /home/lvc26/project/angsd/angsd  sites index wgs_sites.txt    
 Now we create the list of cram files. 
       # Define path and file names  
   path  <-   "/ycga-gpfs/project/caccone/lvc26/september_2020/crams/"  
   samples_30  <-  
      c ( 
        "Kathmandu_Nepal_F_10.cram" , 
        "Kathmandu_Nepal_F_11.cram" , 
        "Kathmandu_Nepal_F_12.cram" , 
        "Kathmandu_Nepal_F_7.cram" , 
        "Kathmandu_Nepal_F_8.cram" , 
        "Kathmandu_Nepal_F_9.cram" , 
        "Kathmandu_Nepal_M_1.cram" , 
        "Kathmandu_Nepal_M_2.cram" , 
        "Kathmandu_Nepal_M_3.cram" , 
        "Kathmandu_Nepal_M_4.cram" , 
        "Kathmandu_Nepal_M_5.cram" , 
        "Kathmandu_Nepal_M_6.cram" , 
        "StAugustine_Trinidad_F_12.cram" , 
        "StAugustine_Trinidad_F_13.cram" , 
        "StAugustine_Trinidad_F_14.cram" , 
        "StAugustine_Trinidad_F_15.cram" , 
        "StAugustine_Trinidad_F_16.cram" , 
        "StAugustine_Trinidad_F_17.cram" , 
        "StAugustine_Trinidad_F_18.cram" , 
        "StAugustine_Trinidad_F_1.cram" , 
        "StAugustine_Trinidad_F_2.cram" , 
        "StAugustine_Trinidad_F_3.cram" , 
        "StAugustine_Trinidad_F_4.cram" , 
        "StAugustine_Trinidad_F_5.cram" , 
        "StAugustine_Trinidad_F_6.cram" , 
        "StAugustine_Trinidad_M_10.cram" , 
        "StAugustine_Trinidad_M_11.cram" , 
        "StAugustine_Trinidad_M_7.cram" , 
        "StAugustine_Trinidad_M_8.cram" , 
        "StAugustine_Trinidad_M_9.cram"  
     ) 
    
    
    # Combine path and file names  
   full_paths_30  <-   file.path (path, samples_30) 
    
    # Write to a text file  
    writeLines (full_paths_30,  here ( "output" , "wgs_vs_chip" ,  "new_calls" ,  "crams_30.txt" )) 
    
    # 18 samples  
   samples_18  <-  
      c ( 
        "Kathmandu_Nepal_F_10.cram" , 
        "Kathmandu_Nepal_F_11.cram" , 
        "Kathmandu_Nepal_F_12.cram" , 
        "Kathmandu_Nepal_F_7.cram" , 
        "Kathmandu_Nepal_F_8.cram" , 
        "Kathmandu_Nepal_F_9.cram" , 
        "StAugustine_Trinidad_F_1.cram" , 
        "StAugustine_Trinidad_F_2.cram" , 
        "StAugustine_Trinidad_F_3.cram" , 
        "StAugustine_Trinidad_F_4.cram" , 
        "StAugustine_Trinidad_F_5.cram" , 
        "StAugustine_Trinidad_F_12.cram" , 
        "StAugustine_Trinidad_F_13.cram" , 
        "StAugustine_Trinidad_F_14.cram" , 
        "StAugustine_Trinidad_F_15.cram" , 
        "StAugustine_Trinidad_F_16.cram" , 
        "StAugustine_Trinidad_F_17.cram" , 
        "StAugustine_Trinidad_F_18.cram"  
     ) 
    
    
    # Combine path and file names  
   full_paths_18  <-   file.path (path, samples_18) 
    
    # Write to a text file  
    writeLines (full_paths_18,  here ( "output" , "wgs_vs_chip" ,  "new_calls" ,  "crams_18.txt" ))    
 Now we have to create the batch scripts to submit in the cluster 
 30 samples 
       #!/bin/sh  
    #SBATCH --mail-type=  BEGIN  ,  END  ,FAIL            
    #SBATCH --mail-user=luciano.cosme@yale.edu   
    #SBATCH --ntasks=1  
    #SBATCH --cpus-per-task=20            
    #SBATCH --mem-per-cpu=5gb                    
    #SBATCH --time=120:00:00   
    #SBATCH --job-name=angsd_wgs_chip_30  
    #SBATCH -o angsd_wgs_chip_30%A_%a.o.txt  
    #SBATCH -e angsd_wgs_chip_30%A_%a.ERROR.txt  
    
    cd  /ycga-gpfs/project/caccone/lvc26/wgs_chip_calls 
    
    /home/lvc26/project/angsd/angsd   \  
   -ref /gpfs/ycga/project/caccone/lvc26/september_2020/genome/aedes_albopictus_LA2_20200826.fasta  \  
   -bam /ycga-gpfs/project/caccone/lvc26/wgs_chip_calls/crams_30.txt  \  
   -nThreads 40  \  
   -gl 1  \  
   -dopost 1  \  
   -doMaf 2  \  
   -doMajorMinor 4  \  
   -minMapQ 20  \  
   -minQ 10  \  
   -remove_bads 1  \  
   -uniqueOnly 1  \  
   -sites /ycga-gpfs/project/caccone/lvc26/wgs_chip_calls/wgs_sites.txt  \  
   -doCounts 1  \  
   -setMinDepthInd 10  \  
   -minInd 2  \  
   -SNP_pval 1e-6  \  
   -doPlink 2  \  
   -doGeno 4  \  
   -capDepth 45  \  
   -minMaf 0.01  \  
   -out /ycga-gpfs/project/caccone/lvc26/wgs_chip_calls/wgs_chip_30    
 18 samples 
       #!/bin/sh  
    #SBATCH --mail-type=  BEGIN  ,  END  ,FAIL            
    #SBATCH --mail-user=luciano.cosme@yale.edu   
    #SBATCH --ntasks=1  
    #SBATCH --cpus-per-task=20            
    #SBATCH --mem-per-cpu=5gb                    
    #SBATCH --time=120:00:00   
    #SBATCH --job-name=angsd_wgs_chip_18  
    #SBATCH -o angsd_wgs_chip_18%A_%a.o.txt  
    #SBATCH -e angsd_wgs_chip_18%A_%a.ERROR.txt  
    
    cd  /ycga-gpfs/project/caccone/lvc26/wgs_chip_calls 
    
    /home/lvc26/project/angsd/angsd   \  
   -ref /gpfs/ycga/project/caccone/lvc26/september_2020/genome/aedes_albopictus_LA2_20200826.fasta  \  
   -bam /ycga-gpfs/project/caccone/lvc26/wgs_chip_calls/crams_18.txt  \  
   -nThreads 40  \  
   -gl 1  \  
   -dopost 1  \  
   -doMaf 2  \  
   -doMajorMinor 4  \  
   -minMapQ 20  \  
   -minQ 10  \  
   -remove_bads 1  \  
   -uniqueOnly 1  \  
   -sites /ycga-gpfs/project/caccone/lvc26/wgs_chip_calls/wgs_sites.txt  \  
   -doCounts 1  \  
   -setMinDepthInd 10  \  
   -minInd 2  \  
   -SNP_pval 1e-6  \  
   -doPlink 2  \  
   -doGeno 4  \  
   -capDepth 45  \  
   -minMaf 0.01  \  
   -out /ycga-gpfs/project/caccone/lvc26/wgs_chip_calls/wgs_chip_18    
 
 
 7.2 Convert tped to bed on the cluster 
 Once the genotype calls are done we can convert the tped to bed file.
Our file has only the SNPs from the list we supplied. We can double
check and extract the SNP ids to see if everything works. 
       awk   -v  OFS= '\t'   '{$6="chr"$1 "_" $4; $7="chr" "_"$1; print $7, $6}'  output/wgs_vs_chip/wgs_01.bim  >  output/wgs_vs_chip/new_calls/SNPs_175k.txt ;  
    head  output/wgs_vs_chip/new_calls/SNPs_175k.txt    
 Now extract the SNPs and create new bed file 
       # Load Plink  
    module  load PLINK/1.90-beta4.4 
    
    cd  /ycga-gpfs/project/caccone/lvc26/wgs_chip_calls 
    
    # 30 samples  
    # Run Plink and extract the 175k SNPs  
    plink   \  
   --allow-extra-chr  \  
   --keep-allele-order  \  
   --tfile wgs_chip_30  \  
   --make-bed  \  
   --extract SNPs_175k.txt  \  
   --out wgs_chip_30 
    
    # 128238 MB RAM detected; reserving 64119 MB for main workspace.  
    # Processing .tped file... done.  
    # wgs_chip_30-temporary.bed + wgs_chip_30-temporary.bim +  
    # wgs_chip_30-temporary.fam written.  
    # 169798 variants loaded from .bim file.  
    # 30 people (0 males, 0 females, 30 ambiguous) loaded from .fam.  
    # Ambiguous sex IDs written to wgs_chip_30.nosex .  
    # --extract: 169798 variants remaining.  
    # Using 1 thread (no multithreaded calculations invoked).  
    # Before main variant filters, 30 founders and 0 nonfounders present.  
    # Calculating allele frequencies... done.  
    # 169798 variants and 30 people pass filters and QC.  
    # Note: No phenotypes present.  
    # --make-bed to wgs_chip_30.bed + wgs_chip_30.bim + wgs_chip_30.fam ... done.  
    
    # 18 samples  
    # Run Plink and extract the 175k SNPs  
    plink   \  
   --allow-extra-chr  \  
   --keep-allele-order  \  
   --tfile wgs_chip_18  \  
   --make-bed  \  
   --extract SNPs_175k.txt  \  
   --out wgs_chip_18 
    
    # 128238 MB RAM detected; reserving 64119 MB for main workspace.  
    # Processing .tped file... done.  
    # wgs_chip_18-temporary.bed + wgs_chip_18-temporary.bim +  
    # wgs_chip_18-temporary.fam written.  
    # 165104 variants loaded from .bim file.  
    # 18 people (0 males, 0 females, 18 ambiguous) loaded from .fam.  
    # Ambiguous sex IDs written to wgs_chip_18.nosex .  
    # --extract: 165104 variants remaining.  
    # Using 1 thread (no multithreaded calculations invoked).  
    # Before main variant filters, 18 founders and 0 nonfounders present.  
    # Calculating allele frequencies... done.  
    # 165104 variants and 18 people pass filters and QC.  
    # Note: No phenotypes present.  
    # --make-bed to wgs_chip_18.bed + wgs_chip_18.bim + wgs_chip_18.fam ... done.     
 
 
 7.3 Check and update SNP ids for wgs 
 The only last thing we need to adjust is to make sure our files have
the same IDs for chromosome and SNPs. The reference genome used for
mapping had “chr” before each scaffold name. When we do a genotype call
with the chip data, we do not have the extra string “chr” in each
scaffold name. Therefore, we need to adjust that to compare the samples.
I did remove the string “chr” from the reference genome. We can remove
it from our bed file using any tool. 
 In the past I did a genotype call for each population. We have 18
samples for SAI and 12 samples for KAT. We had DNA left over for 6
samples for KAT and 12 samples for SAI. That makes everything more
complicated to compare. We have to make sure that there is no
differences in the genotype calls based on the number of samples with
which we do the calls. 
 For now. I will compare the results of the wgs calls using the 819
samples (all populations), 30 samples (both populations, KAT and SAI),
and 18 samples (only the samples we have chip data). 
 For the chip calls, I did a call using only the 18 samples. Since I
did not have more samples, I did a genotype call using the entire plate
of samples where the 18 samples were (95 samples total). Finally, I did
a genotype call using all wild samples (native and invasive ranges) we
have in the manuscript with the 18 samples. 
 Therefore, we have 3 wgs calls and 3 chip calls. I decided not to
compare the priors. We can compare the priors separately. 
 Lets get the data in the same format. I download the data from the
cluster and put it in the dir “new_calls” 
 Check the .bim file after downloading it from the cluster 
       head  output/wgs_vs_chip/new_calls/wgs_chip_18.bim    
  ## 2.206    NA  0   14153   G   A
## 2.206    NA  0   41198   G   T
## 2.206    NA  0   46216   C   T
## 2.206    NA  0   46416   G   A
## 2.206    NA  0   47314   T   G
## 2.206    NA  0   64862   A   G
## 2.206    NA  0   67410   C   T
## 2.206    NA  0   69313   A   C
## 2.206    NA  0   71859   A   T
## 2.206    NA  0   72355   A   G  
 Now check how the chip data is different 
       head  output/wgs_vs_chip/chip_dp_01.bim    
  ## 1.1  AX-581444870    0   97856   C   T
## 1.1  AX-583035067    0   229640  T   A
## 1.1  AX-583035102    0   308124  A   G
## 1.1  AX-583033342    0   315059  C   G
## 1.1  AX-583035163    0   315386  A   G
## 1.1  AX-583035194    0   330265  A   G
## 1.1  AX-583033387    0   331288  C   T
## 1.1  AX-583035211    0   345197  C   T
## 1.10 AX-583035257    0   91677   T   C
## 1.10 AX-583033504    0   141489  C   T  
 We can see first they are not in the same order and that the SNP ids
are different. We can use the file we created earlier to update the
ids. 
 Check the file 
       head  output/wgs_vs_chip/new_calls/wgs_snps_ids.txt    
  ## 1.1  chr1.1  97856   chr1.1_97856    AX-581444870
## 1.1  chr1.1  161729  chr1.1_161729   AX-583033226
## 1.1  chr1.1  229640  chr1.1_229640   AX-583035067
## 1.1  chr1.1  305518  chr1.1_305518   AX-583035083
## 1.1  chr1.1  308124  chr1.1_308124   AX-583035102
## 1.1  chr1.1  311920  chr1.1_311920   AX-583033340
## 1.1  chr1.1  315059  chr1.1_315059   AX-583033342
## 1.1  chr1.1  315386  chr1.1_315386   AX-583035163
## 1.1  chr1.1  315674  chr1.1_315674   AX-583033356
## 1.1  chr1.1  330057  chr1.1_330057   AX-583033370  
 We can import the files, but make sure we keep the same order of the
“wgs_chip_18.bim”, we can create an index once we import. 
       # Define file paths using here  
   bim_file  <-  
      here ( "output" ,  "wgs_vs_chip" ,  "new_calls" ,  "wgs_chip_18.bim" ) 
   snp_ids_file  <-  
      here ( "output" ,  "wgs_vs_chip" ,  "new_calls" ,  "wgs_snps_ids.txt" ) 
   output_file  <-  
      here ( "output" , 
           "wgs_vs_chip" , 
           "new_calls" , 
           "wgs_chip_18_updated.bim" ) 
    
    # Import the .bim file  
   bim_data  <-   read_delim ( 
     bim_file, 
      delim =   "  \t  " , 
      show_col_types =   FALSE , 
      col_names =   c ( "chr" ,  "id_match" ,  "cm" ,  "bp" ,  "allele1" ,  "allele2" ), 
      col_types =   cols ( .default =   col_character ()) 
   ) 
    
    # Create an index column  
   bim_data  <-   
     bim_data  |>  
      mutate ( index =   row_number ())  |>  
      # Remove the string "chr" from the chr column  
      mutate ( chr =   str_remove (chr,  "chr" )) 
    
    # Import the .txt file  
   snp_ids  <-   read_delim ( 
     snp_ids_file, 
      delim =   "  \t  " , 
      show_col_types =   FALSE , 
      col_names =   c ( "chr_ref" ,  "id_ref" ,  "bp_ref" ,  "id_match" ,  "snp_id" ), 
      col_types =   cols ( .default =   col_character ()) 
   ) 
    
    # Merge the two data frames by matching chr and bp in bim_data with chr_ref and bp_ref in snp_ids  
   merged_data  <-  
      left_join (bim_data, snp_ids,  by =   "id_match" )  |>  
     dplyr ::  select ( 
       chr, snp_id, cm, bp, allele1, allele2 
     ) 
    
    # Check output  
    head (merged_data)    
  ## # A tibble: 6 × 6
##   chr   snp_id cm    bp    allele1 allele2
##   <chr> <chr>  <chr> <chr> <chr>   <chr>  
## 1 2.206 <NA>   0     14153 G       A      
## 2 2.206 <NA>   0     41198 G       T      
## 3 2.206 <NA>   0     46216 C       T      
## 4 2.206 <NA>   0     46416 G       A      
## 5 2.206 <NA>   0     47314 T       G      
## 6 2.206 <NA>   0     64862 A       G  
       # Write the updated data frame to a new .bim file without headers or quotes  
    write.table ( 
     merged_data, 
      file =  output_file, 
      sep =   "  \t  " , 
      quote =   FALSE , 
      row.names =   FALSE , 
      col.names =   FALSE  
   )    
 Now, add word “backup” to the current .bim file and then delete
“updated” from the new file we save. Then it replaces the current .bim
file 
 Compare both .bim files to see if they look okay 
 Before 
       head  output/wgs_vs_chip/new_calls/wgs_chip_18.bim    
  ## 2.206    NA  0   14153   G   A
## 2.206    NA  0   41198   G   T
## 2.206    NA  0   46216   C   T
## 2.206    NA  0   46416   G   A
## 2.206    NA  0   47314   T   G
## 2.206    NA  0   64862   A   G
## 2.206    NA  0   67410   C   T
## 2.206    NA  0   69313   A   C
## 2.206    NA  0   71859   A   T
## 2.206    NA  0   72355   A   G  
 After 
       head  output/wgs_vs_chip/new_calls/wgs_chip_18_updated.bim    
  ## 2.206    NA  0   14153   G   A
## 2.206    NA  0   41198   G   T
## 2.206    NA  0   46216   C   T
## 2.206    NA  0   46416   G   A
## 2.206    NA  0   47314   T   G
## 2.206    NA  0   64862   A   G
## 2.206    NA  0   67410   C   T
## 2.206    NA  0   69313   A   C
## 2.206    NA  0   71859   A   T
## 2.206    NA  0   72355   A   G  
 It looks okay. We can replace the original file with the new file 
       mv  output/wgs_vs_chip/new_calls/wgs_chip_18.bim output/wgs_vs_chip/new_calls/wgs_chip_18_backup.bim ;  
    mv  output/wgs_vs_chip/new_calls/wgs_chip_18_updated.bim output/wgs_vs_chip/new_calls/wgs_chip_18.bim ;     
 
 
 7.4 Set reference allele 
 We can check if everything is working by checking the reference
allele using the genome without the string ‘chr’ 
       plink2   \  
   --allow-extra-chr  \  
   --bfile output/wgs_vs_chip/new_calls/wgs_chip_18  \  
   --make-bed  \  
   --fa data/genome/albo.fasta.gz  \  
   --ref-from-fa  'force'   `  # sets REF alleles when it can be done unambiguously, we use force to change the alleles  `   \  
   --out output/wgs_vs_chip/new_calls/wgs_chip_18_samples  \  
   --silent ;  
    # --keep-allele-order \ if you use Plink 1.9  
    grep   "variants"  output/wgs_vs_chip/new_calls/wgs_chip_18_samples.log  # to get the number of variants from the log file.     
  ## 165104 variants loaded from output/wgs_vs_chip/new_calls/wgs_chip_18.bim.
## --ref-from-fa force: 35328 variants changed, 129772 validated.  
 We updated the alleles and now we can do the same operation for the
other file with the 30 samples. 
       # Define file paths using here  
   bim_file  <-  
      here ( "output" ,  "wgs_vs_chip" ,  "new_calls" ,  "wgs_chip_30.bim" ) 
   snp_ids_file  <-  
      here ( "output" ,  "wgs_vs_chip" ,  "new_calls" ,  "wgs_snps_ids.txt" ) 
   output_file  <-  
      here ( "output" , 
           "wgs_vs_chip" , 
           "new_calls" , 
           "wgs_chip_30_updated.bim" ) 
    
    # Import the .bim file  
   bim_data  <-   read_delim ( 
     bim_file, 
      delim =   "  \t  " , 
      show_col_types =   FALSE , 
      col_names =   c ( "chr" ,  "id_match" ,  "cm" ,  "bp" ,  "allele1" ,  "allele2" ), 
      col_types =   cols ( .default =   col_character ()) 
   ) 
    
    # Create an index column  
   bim_data  <-   
     bim_data  |>  
      mutate ( index =   row_number ())  |>  
      # Remove the string "chr" from the chr column  
      mutate ( chr =   str_remove (chr,  "chr" )) 
    
    # Import the .txt file  
   snp_ids  <-   read_delim ( 
     snp_ids_file, 
      delim =   "  \t  " , 
      show_col_types =   FALSE , 
      col_names =   c ( "chr_ref" ,  "id_ref" ,  "bp_ref" ,  "id_match" ,  "snp_id" ), 
      col_types =   cols ( .default =   col_character ()) 
   ) 
    
    # Merge the two data frames by matching chr and bp in bim_data with chr_ref and bp_ref in snp_ids  
   merged_data  <-  
      left_join (bim_data, snp_ids,  by =   "id_match" )  |>  
     dplyr ::  select ( 
       chr, snp_id, cm, bp, allele1, allele2 
     ) 
    
    # Check output  
    head (merged_data)    
  ## # A tibble: 6 × 6
##   chr   snp_id cm    bp    allele1 allele2
##   <chr> <chr>  <chr> <chr> <chr>   <chr>  
## 1 2.206 <NA>   0     14153 G       A      
## 2 2.206 <NA>   0     41198 G       T      
## 3 2.206 <NA>   0     46216 C       T      
## 4 2.206 <NA>   0     46416 G       A      
## 5 2.206 <NA>   0     47314 T       G      
## 6 2.206 <NA>   0     64862 A       G  
       # Write the updated data frame to a new .bim file without headers or quotes  
    write.table ( 
     merged_data, 
      file =  output_file, 
      sep =   "  \t  " , 
      quote =   FALSE , 
      row.names =   FALSE , 
      col.names =   FALSE  
   )    
 Compare both .bim files to see if they look okay 
 Before 
       head  output/wgs_vs_chip/new_calls/wgs_chip_30.bim    
  ## 2.206    NA  0   14153   G   A
## 2.206    NA  0   41198   G   T
## 2.206    NA  0   46216   C   T
## 2.206    NA  0   46416   G   A
## 2.206    NA  0   47314   T   G
## 2.206    NA  0   64862   A   G
## 2.206    NA  0   67410   C   T
## 2.206    NA  0   69313   A   C
## 2.206    NA  0   71859   A   T
## 2.206    NA  0   72355   A   G  
 After 
       head  output/wgs_vs_chip/new_calls/wgs_chip_30_updated.bim    
  ## 2.206    NA  0   14153   G   A
## 2.206    NA  0   41198   G   T
## 2.206    NA  0   46216   C   T
## 2.206    NA  0   46416   G   A
## 2.206    NA  0   47314   T   G
## 2.206    NA  0   64862   A   G
## 2.206    NA  0   67410   C   T
## 2.206    NA  0   69313   A   C
## 2.206    NA  0   71859   A   T
## 2.206    NA  0   72355   A   G  
 It looks okay. We can replace the original file with the new file 
       mv  output/wgs_vs_chip/new_calls/wgs_chip_30.bim output/wgs_vs_chip/new_calls/wgs_chip_30_backup.bim ;  
    mv  output/wgs_vs_chip/new_calls/wgs_chip_30_updated.bim output/wgs_vs_chip/new_calls/wgs_chip_30.bim    
 We can check if everything is working by checking the reference
allele using the genome without the string ‘chr’ 
       plink2   \  
   --allow-extra-chr  \  
   --bfile output/wgs_vs_chip/new_calls/wgs_chip_30  \  
   --make-bed  \  
   --fa data/genome/albo.fasta.gz  \  
   --ref-from-fa  'force'   `  # sets REF alleles when it can be done unambiguously, we use force to change the alleles  `   \  
   --out output/wgs_vs_chip/new_calls/wgs_chip_30_samples  \  
   --silent ;  
    # --keep-allele-order \ if you use Plink 1.9  
    grep   "variants"  output/wgs_vs_chip/new_calls/wgs_chip_30_samples.log  # to get the number of variants from the log file.     
  ## 169798 variants loaded from output/wgs_vs_chip/new_calls/wgs_chip_30.bim.
## --ref-from-fa force: 36269 variants changed, 133525 validated.  
 The bed file with the genotypes for the 18 samples extracted after a
genotype call with all 819 samples is in our directory. We already set
the reference alleles. Check the log of the file 
       head   -n  100 output/wgs_vs_chip/wgs_01.log    
  ## PLINK v2.00a3.3 64-bit (3 Jun 2022)
## Options in effect:
##   --allow-extra-chr
##   --bfile data/raw_data/albo/wgs_vs_chip/wgs
##   --fa data/genome/albo.fasta.gz
##   --make-bed
##   --out output/wgs_vs_chip/wgs_01
##   --ref-from-fa force
##   --silent
## 
## Hostname: LucianoCosme.wireless.yale.internal
## Working directory: /Users/lucianocosme/Library/CloudStorage/Dropbox/Albopictus/manuscript_chip/data/no_autogenous/albo_chip
## Start time: Mon Aug 28 10:26:14 2023
## 
## Random number seed: 1693232774
## 32768 MiB RAM detected; reserving 16384 MiB for main workspace.
## Using up to 12 threads (change this with --threads).
## 18 samples (0 females, 0 males, 18 ambiguous; 18 founders) loaded from
## data/raw_data/albo/wgs_vs_chip/wgs.fam.
## 175360 variants loaded from data/raw_data/albo/wgs_vs_chip/wgs.bim.
## Note: No phenotype data present.
## --ref-from-fa force: 0 variants changed, 175360 validated.
## Writing output/wgs_vs_chip/wgs_01.fam ... done.
## Writing output/wgs_vs_chip/wgs_01.bim ... done.
## Writing output/wgs_vs_chip/wgs_01.bed ... done.
## 
## End time: Mon Aug 28 10:26:17 2023  
 
 
 
 8. Data sets for comparisons 
 For the chip calls we will use only the default prior. We will have
the 3 data sets: call using 18 samples, call using a plate (95 samples),
and call using all wild samples (515 samples). 
 
 8.1 Setting labels for each data set 
 We need to make sure the sex is correct in all files. We can add
letters to separate each data set” 
 a - chip call with 18 samples b - chip call with plate (95 samples) c
- chip call with 500+ samples w - wgs call with 800+ samples x - wgs
call with 30 samples y - wgs call with 18 samples 
 Check the log of Plink when we set alleles for the data set with the
18 samples only. 
       head   -n  100 output/wgs_vs_chip/chip_dp_01.log    
  ## PLINK v2.00a3.3 64-bit (3 Jun 2022)
## Options in effect:
##   --allow-extra-chr
##   --const-fid
##   --fa data/genome/albo.fasta.gz
##   --make-bed
##   --out output/wgs_vs_chip/chip_dp_01
##   --ref-from-fa force
##   --silent
##   --vcf data/raw_data/albo/wgs_vs_chip/wgs_default_prior_recommended_june_16_2023.vcf
## 
## Hostname: LucianoCosme.wireless.yale.internal
## Working directory: /Users/lucianocosme/Library/CloudStorage/Dropbox/Albopictus/manuscript_chip/data/no_autogenous/albo_chip
## Start time: Mon Aug 28 10:26:09 2023
## 
## Random number seed: 1693232769
## 32768 MiB RAM detected; reserving 16384 MiB for main workspace.
## Using up to 12 threads (change this with --threads).
## --vcf: 105607 variants scanned.
## --vcf: output/wgs_vs_chip/chip_dp_01-temporary.pgen +
## output/wgs_vs_chip/chip_dp_01-temporary.pvar.zst +
## output/wgs_vs_chip/chip_dp_01-temporary.psam written.
## 18 samples (0 females, 0 males, 18 ambiguous; 18 founders) loaded from
## output/wgs_vs_chip/chip_dp_01-temporary.psam.
## 105607 variants loaded from output/wgs_vs_chip/chip_dp_01-temporary.pvar.zst.
## Note: No phenotype data present.
## --ref-from-fa force: 0 variants changed, 105607 validated.
## Writing output/wgs_vs_chip/chip_dp_01.fam ... done.
## Writing output/wgs_vs_chip/chip_dp_01.bim ... done.
## Writing output/wgs_vs_chip/chip_dp_01.bed ... done.
## 
## End time: Mon Aug 28 10:26:11 2023  
 Import the new results (95 and 515 samples). I used the default prior
for both. We have a different document where we compare the priors and
decided if it is worth using it. 
       # I created a fam file with the information about each sample, but first we import the data and create a bed file setting the family id constant  
    plink2   \  
   --allow-extra-chr  \  
   --vcf data/raw_data/albo/wgs_vs_chip/chip_wgs_plate_june_28_dp.vcf  \  
   --const-fid  \  
   --make-bed  \  
   --fa data/genome/albo.fasta.gz  \  
   --ref-from-fa  'force'   `  # sets REF alleles when it can be done unambiguously, we use force to change the alleles  `   \  
   --out output/wgs_vs_chip/chip_plate_dp_01  `  # dp - default priors  `   \  
   --silent ;  
    # --keep-allele-order \ if you use Plink 1.9  
    grep   "variants"  output/wgs_vs_chip/chip_plate_dp_01.log  # to get the number of variants from the log file.     
  ## --vcf: 104895 variants scanned.
## 104895 variants loaded from
## --ref-from-fa force: 0 variants changed, 104895 validated.  
 Now the chip calls using 500+ samples 
 Import the fam file we use with Axiom Suite 
       # the order of the rows in this file does not matter  
   samples  <-  
      read.delim ( 
        file   =   here ( 
          "data" , 
          "raw_data" , 
          "albo" , 
          "wgs_vs_chip" , 
          "sample_ped_info_2.txt"  
       ), 
        header =   TRUE  
     ) 
    head (samples)    
  ##     Sample.Filename Family_ID Individual_ID Father_ID Mother_ID Sex
## 1  8_MAN_Brazil.CEL       MAU             8         0         0   0
## 2  9_MAN_Brazil.CEL       MAU             9         0         0   0
## 3 16_MAN_Brazil.CEL       MAU            16         0         0   0
## 4 17_MAN_Brazil.CEL       MAU            17         0         0   0
## 5 18_MAN_Brazil.CEL       MAU            18         0         0   0
## 6 60_MAN_Brazil.CEL       MAU            60         0         0   0
##   Affection.Status
## 1               -9
## 2               -9
## 3               -9
## 4               -9
## 5               -9
## 6               -9  
 Import .fam file we created once we created the bed file using
Plink2 
       # The fam file is the same for both data sets with the default or new priors  
   fam1  <-  
      read.delim ( 
        file   =   here ( 
          "output" ,  "wgs_vs_chip" ,  "chip_plate_dp_01.fam"  
       ), 
        header =   FALSE , 
        
     ) 
    head (fam1)    
  ##   V1                   V2 V3 V4 V5 V6
## 1  0 601_Debug027_A12.CEL  0  0  0 -9
## 2  0  602_Debug027_A2.CEL  0  0  0 -9
## 3  0  603_Debug027_A5.CEL  0  0  0 -9
## 4  0  604_Debug027_B1.CEL  0  0  0 -9
## 5  0  605_Debug027_B2.CEL  0  0  0 -9
## 6  0  606_Debug027_B3.CEL  0  0  0 -9  
 We can merge the tibbles 
       # to keep the same order of the .fam file, we will first create an index based on the numbers of the samples, then use it too keep the order  
    
    # Extract the number part from the columns  
   fam1_temp  <-  fam1  |>  
      mutate ( num_id =   as.numeric ( str_extract (V2,  "^  \\  d+" ))) 
    
   samples_temp  <-  samples  |>  
      mutate ( num_id =   as.numeric ( str_extract (Sample.Filename,  "^  \\  d+" ))) 
    
    # Perform the left join using the num_id columns and keep the order of fam1  
   df  <-  fam1_temp  |>  
     dplyr ::  left_join (samples_temp,  by =   "num_id" )  |>  
     dplyr ::  select ( - num_id)  |>  
     dplyr ::  select ( 8  :  13 ) 
    
    # check the data frame  
    head (df)    
  ##   Family_ID Individual_ID Father_ID Mother_ID Sex Affection.Status
## 1       KAT             7         0         0   1               -9
## 2       GEL           602         0         0   0               -9
## 3       GEL           603         0         0   0               -9
## 4       KAT             8         0         0   1               -9
## 5       KAT             9         0         0   1               -9
## 6       KAT            10         0         0   1               -9  
 We can check how many samples we have in our file 
       nrow (df)    
  ## [1] 95  
 Before you save the new fam file, you can change the original file to
a different name, to compare the order later. If you want to repeat the
steps above after you save the new file1.fam, you will need to import
the vcf again. 
       # Save and override the .fam file for dp  
    write.table ( 
     df, 
      file      =   here ( 
        "output" ,  "wgs_vs_chip" ,  "chip_plate_dp_01.fam"  
     ), 
      sep       =   "  \t  " , 
      row.names =   FALSE , 
      col.names =   FALSE , 
      quote     =   FALSE  
   )    
 Now we have to subset the data set to keep only the samples form KAT
and SAI. We can create a file with the samples we have to keep using the
.fam file of our previous call. 
 Check the .fam file 
       head  output/wgs_vs_chip/chip_dp_01.fam    
  ## KAT  7a  0   0   2   -9
## KAT  8a  0   0   2   -9
## KAT  9a  0   0   2   -9
## KAT  10a 0   0   2   -9
## KAT  11a 0   0   2   -9
## KAT  12a 0   0   2   -9
## SAI  4a  0   0   2   -9
## SAI  5a  0   0   2   -9
## SAI  1a  0   0   2   -9
## SAI  2a  0   0   2   -9  
 We need to remove the “a” 
       awk   '{gsub("a", "", $2); print $1,$2}'  output/wgs_vs_chip/chip_dp_01.fam  >  output/wgs_vs_chip/chip_samples_subset.txt ;  
    head  output/wgs_vs_chip/chip_samples_subset.txt    
  ## KAT 7
## KAT 8
## KAT 9
## KAT 10
## KAT 11
## KAT 12
## SAI 4
## SAI 5
## SAI 1
## SAI 2  
 Now subset the samples 
       plink2   \  
   --allow-extra-chr  \  
   --bfile output/wgs_vs_chip/chip_plate_dp_01  \  
   --make-bed  \  
   --keep output/wgs_vs_chip/chip_samples_subset.txt  \  
   --out output/wgs_vs_chip/chip_plate_dp_02  \  
   --silent ;  
    # --keep-allele-order \ if you use Plink 1.9  
    grep   "variants\|samples"  output/wgs_vs_chip/chip_plate_dp_02.log  # to get the number of variants from the log file.     
  ##   --keep output/wgs_vs_chip/chip_samples_subset.txt
## 95 samples (21 females, 60 males, 14 ambiguous; 95 founders) loaded from
## 104895 variants loaded from output/wgs_vs_chip/chip_plate_dp_01.bim.
## --keep: 18 samples remaining.
## 18 samples (0 females, 18 males; 18 founders) remaining after main filters.  
 Check the new .fam file to see if has the order and the sample
attributes we want. 
 Check the fam file of the call with 18 samples 
       # you can open the file on a text editor and double check the sample order and information.  
    head   -n  5 output/wgs_vs_chip/chip_dp_01.fam    
  ## KAT  7a  0   0   2   -9
## KAT  8a  0   0   2   -9
## KAT  9a  0   0   2   -9
## KAT  10a 0   0   2   -9
## KAT  11a 0   0   2   -9  
 Check the plate data 
       # you can open the file on a text editor and double check the sample order and information.  
    head   -n  5 output/wgs_vs_chip/chip_plate_dp_02.fam    
  ## KAT  7   0   0   1   -9
## KAT  8   0   0   1   -9
## KAT  9   0   0   1   -9
## KAT  10  0   0   1   -9
## KAT  11  0   0   1   -9  
 We see inconsistency in the sex and that we could add a letter to the
fam file of “chip_plate_dp_02.fam”. Lets use awk to add the letter
“b” 
       # Run this only once  
    awk   '{$2 = $2 "b"; print $0}'  output/wgs_vs_chip/chip_plate_dp_02.fam  >  output/wgs_vs_chip/chip_plate_dp_02_new.fam  &&   mv  output/wgs_vs_chip/chip_plate_dp_02_new.fam output/wgs_vs_chip/chip_plate_dp_02.fam ;  
    
    # Check the output  
    head  output/wgs_vs_chip/chip_plate_dp_02.fam    
  ## KAT 7b 0 0 1 -9
## KAT 8b 0 0 1 -9
## KAT 9b 0 0 1 -9
## KAT 10b 0 0 1 -9
## KAT 11b 0 0 1 -9
## KAT 12b 0 0 1 -9
## SAI 4b 0 0 1 -9
## SAI 5b 0 0 1 -9
## SAI 1b 0 0 1 -9
## SAI 2b 0 0 1 -9  
 I fixed the sex manually and created new file 
       # Check the output  
    head  output/wgs_vs_chip/chip_plate_dp_03.fam    
  ## KAT 7b 0 0 2 -9
## KAT 8b 0 0 2 -9
## KAT 9b 0 0 2 -9
## KAT 10b 0 0 2 -9
## KAT 11b 0 0 2 -9
## KAT 12b 0 0 2 -9
## SAI 4b 0 0 2 -9
## SAI 5b 0 0 2 -9
## SAI 1b 0 0 2 -9
## SAI 2b 0 0 2 -9  
 We can use ‘c’ for the data set from the call with 500+ samples. 
 We can also update the .fam file of the wgs data, adding letters to
the samples. We will then merge the bed files and use code to create vcf
files with pairs of samples setting missingness to zero. 
 Check the wgs data 
       # you can open the file on a text editor and double check the sample order and information.  
    head   -n  5 output/wgs_vs_chip/new_calls/wgs_chip_30_samples.fam    
  ## 1    1   0   0   0   -9
## 2    1   0   0   0   -9
## 3    1   0   0   0   -9
## 4    1   0   0   0   -9
## 5    1   0   0   0   -9  
 ANGSD create the file with the samples following the order of the
samples in our list of crams files 
       head   -n  5 output/wgs_vs_chip/new_calls/crams_30.txt    
  ## /ycga-gpfs/project/caccone/lvc26/september_2020/crams/Kathmandu_Nepal_F_10.cram
## /ycga-gpfs/project/caccone/lvc26/september_2020/crams/Kathmandu_Nepal_F_11.cram
## /ycga-gpfs/project/caccone/lvc26/september_2020/crams/Kathmandu_Nepal_F_12.cram
## /ycga-gpfs/project/caccone/lvc26/september_2020/crams/Kathmandu_Nepal_F_7.cram
## /ycga-gpfs/project/caccone/lvc26/september_2020/crams/Kathmandu_Nepal_F_8.cram  
 I created a file with 3 columns: Family id, sex, individual id 
       head   -n  5 output/wgs_vs_chip/new_calls/crams_30_names_sex.txt    
  ## KAT 2 10
## KAT 2 11
## KAT 2 12
## KAT 2 7
## KAT 2 8  
 Now we can use the file with the name of the samples to replace
columns in the .fam file 
       # Create new fam  
    paste  output/wgs_vs_chip/new_calls/wgs_chip_30_samples.fam output/wgs_vs_chip/new_calls/crams_30_names_sex.txt |   awk   '{print $7, $9, $3, $4, $8, $6}'   >  output/wgs_vs_chip/new_calls/merged_30.fam ;  
    # Check it  
    head  output/wgs_vs_chip/new_calls/merged_30.fam ;  
    # Backup and replace  
    mv  output/wgs_vs_chip/new_calls/wgs_chip_30_samples.fam output/wgs_vs_chip/new_calls/wgs_chip_30_samples_backup.fam ;  
    mv  output/wgs_vs_chip/new_calls/merged_30.fam output/wgs_vs_chip/new_calls/wgs_chip_30_samples.fam    
  ## KAT 10 0 0 2 -9
## KAT 11 0 0 2 -9
## KAT 12 0 0 2 -9
## KAT 7 0 0 2 -9
## KAT 8 0 0 2 -9
## KAT 9 0 0 2 -9
## KAT 1 0 0 1 -9
## KAT 2 0 0 1 -9
## KAT 3 0 0 1 -9
## KAT 4 0 0 1 -9  
 We have to repeat it for the other wgs data sets 
 18 samples 
       # Create new fam  
    paste  output/wgs_vs_chip/new_calls/wgs_chip_18_samples.fam output/wgs_vs_chip/new_calls/crams_18_names_sex.txt |   awk   '{print $7, $9, $3, $4, $8, $6}'   >  output/wgs_vs_chip/new_calls/merged_18.fam ;  
    # Check it  
    head  output/wgs_vs_chip/new_calls/merged_18.fam ;  
    # Backup and replace  
    mv  output/wgs_vs_chip/new_calls/wgs_chip_18_samples.fam output/wgs_vs_chip/new_calls/wgs_chip_18_samples_backup.fam ;  
    mv  output/wgs_vs_chip/new_calls/merged_18.fam output/wgs_vs_chip/new_calls/wgs_chip_18_samples.fam    
  ## KAT 10 0 0 2 -9
## KAT 11 0 0 2 -9
## KAT 12 0 0 2 -9
## KAT 7 0 0 2 -9
## KAT 8 0 0 2 -9
## KAT 9 0 0 2 -9
## SAI 1 0 0 2 -9
## SAI 2 0 0 2 -9
## SAI 3 0 0 2 -9
## SAI 4 0 0 2 -9  
 Check the file extracted from the 819 samples genotype call 
       head  output/wgs_vs_chip/wgs_01.fam    
  ## SAI  5w  0   0   0   -9
## SAI  4w  0   0   0   -9
## SAI  3w  0   0   0   -9
## SAI  2w  0   0   0   -9
## SAI  1w  0   0   0   -9
## SAI  18w 0   0   0   -9
## SAI  17w 0   0   0   -9
## SAI  16w 0   0   0   -9
## SAI  15w 0   0   0   -9
## SAI  14w 0   0   0   -9  
 I created a new file and added the “w” 
       head  output/wgs_vs_chip/wgs_02.fam    
  ## SAI  5w  0   0   2   -9
## SAI  4w  0   0   2   -9
## SAI  3w  0   0   2   -9
## SAI  2w  0   0   2   -9
## SAI  1w  0   0   2   -9
## SAI  18w 0   0   2   -9
## SAI  17w 0   0   2   -9
## SAI  16w 0   0   2   -9
## SAI  15w 0   0   2   -9
## SAI  14w 0   0   2   -9  
 Lets make sure the sex is set the same in all files 
 Check “a” chip call with 18 samples 
       head  output/wgs_vs_chip/chip_dp_01.fam    
  ## KAT  7a  0   0   2   -9
## KAT  8a  0   0   2   -9
## KAT  9a  0   0   2   -9
## KAT  10a 0   0   2   -9
## KAT  11a 0   0   2   -9
## KAT  12a 0   0   2   -9
## SAI  4a  0   0   2   -9
## SAI  5a  0   0   2   -9
## SAI  1a  0   0   2   -9
## SAI  2a  0   0   2   -9  
 Check “b” chip call with plate 
       head  output/wgs_vs_chip/chip_plate_dp_03.fam    
  ## KAT 7b 0 0 2 -9
## KAT 8b 0 0 2 -9
## KAT 9b 0 0 2 -9
## KAT 10b 0 0 2 -9
## KAT 11b 0 0 2 -9
## KAT 12b 0 0 2 -9
## SAI 4b 0 0 2 -9
## SAI 5b 0 0 2 -9
## SAI 1b 0 0 2 -9
## SAI 2b 0 0 2 -9  
 Check “c” chip call with 500+ samples 
 We need to prepare the bed file first. 
       # I created a fam file with the information about each sample, but first we import the data and create a bed file setting the family id constant  
    plink2   \  
   --allow-extra-chr  \  
   --vcf data/raw_data/albo/wgs_vs_chip/manuscript_dp_june_28.vcf  \  
   --const-fid  \  
   --make-bed  \  
   --fa data/genome/albo.fasta.gz  \  
   --ref-from-fa  'force'   `  # sets REF alleles when it can be done unambiguously, we use force to change the alleles  `   \  
   --out output/wgs_vs_chip/chip_500_dp_01  `  # dp - default priors  `   \  
   --silent ;  
    # --keep-allele-order \ if you use Plink 1.9  
    grep   "variants"  output/wgs_vs_chip/chip_500_dp_01.log  # to get the number of variants from the log file.     
  ## --vcf: 107294 variants scanned.
## 107294 variants loaded from
## --ref-from-fa force: 0 variants changed, 107294 validated.  
 Import the fam file we use with Axiom Suite 
       # the order of the rows in this file does not matter  
   samples  <-  
      read.delim ( 
        file   =   here ( 
          "data" , 
          "raw_data" , 
          "albo" , 
          "wgs_vs_chip" , 
          "sample_ped_info_ALLPOPS_for_comparisons.txt"  
       ), 
        header =   TRUE  
     ) 
    head (samples)    
  ##     Sample.Filename Family_ID Individual_ID Father_ID Mother_ID Sex
## 1  8_MAN_Brazil.CEL       MAU             8         0         0   0
## 2  9_MAN_Brazil.CEL       MAU             9         0         0   0
## 3 16_MAN_Brazil.CEL       MAU            16         0         0   0
## 4 17_MAN_Brazil.CEL       MAU            17         0         0   0
## 5 18_MAN_Brazil.CEL       MAU            18         0         0   0
## 6 60_MAN_Brazil.CEL       MAU            60         0         0   0
##   Affection.Status
## 1               -9
## 2               -9
## 3               -9
## 4               -9
## 5               -9
## 6               -9  
 Import .fam file we created once we created the bed file using
Plink2 
       # The fam file is the same for both data sets with the default or new priors  
   fam1  <-  
      read.delim ( 
        file   =   here ( 
          "output" ,  "wgs_vs_chip" ,  "chip_500_dp_01.fam"  
       ), 
        header =   FALSE , 
        
     ) 
    head (fam1)    
  ##   V1           V2 V3 V4 V5 V6
## 1  0 1001_OKI.CEL  0  0  0 -9
## 2  0 1002_OKI.CEL  0  0  0 -9
## 3  0 1003_OKI.CEL  0  0  0 -9
## 4  0 1004_OKI.CEL  0  0  0 -9
## 5  0 1005_OKI.CEL  0  0  0 -9
## 6  0 1006_OKI.CEL  0  0  0 -9  
 We can merge the tibbles. 
       # to keep the same order of the .fam file, we will first create an index based on the numbers of the samples, then use it too keep the order  
    
    # Extract the number part from the columns  
   fam1_temp  <-  fam1  |>  
      mutate ( num_id =   as.numeric ( str_extract (V2,  "^  \\  d+" ))) 
    
   samples_temp  <-  samples  |>  
      mutate ( num_id =   as.numeric ( str_extract (Sample.Filename,  "^  \\  d+" ))) 
    
    # Perform the left join using the num_id columns and keep the order of fam1  
   df  <-  fam1_temp  |>  
     dplyr ::  left_join (samples_temp,  by =   "num_id" )  |>  
     dplyr ::  select ( - num_id)  |>  
     dplyr ::  select ( 8  :  13 ) 
    
    # check the data frame  
    head (df)    
  ##   Family_ID Individual_ID Father_ID Mother_ID Sex Affection.Status
## 1       OKI          1001         0         0   2               -9
## 2       OKI          1002         0         0   2               -9
## 3       OKI          1003         0         0   2               -9
## 4       OKI          1004         0         0   2               -9
## 5       OKI          1005         0         0   2               -9
## 6       OKI          1006         0         0   1               -9  
 We can check how many samples we have in our file 
       nrow (df)    
  ## [1] 479  
 Before you save the new fam file, you can change the original file to
a different name, to compare the order later. If you want to repeat the
steps above after you saving the new file1.fam, you will need to import
the vcf again. 
       # Save and override the .fam file for dp  
    write.table ( 
     df, 
      file      =   here ( "output" ,  "wgs_vs_chip" ,  "chip_500_dp_01.fam" ), 
      sep       =   "  \t  " , 
      row.names =   FALSE , 
      col.names =   FALSE , 
      quote     =   FALSE  
   )    
 Now we have to subset the data set to keep only the samples form KAT
and SAI. We can create a file with the samples we have to keep using the
.fam file of our previous call. 
 Check the .fam file 
       head  output/wgs_vs_chip/chip_500_dp_01.fam    
  ## OKI  1001    0   0   2   -9
## OKI  1002    0   0   2   -9
## OKI  1003    0   0   2   -9
## OKI  1004    0   0   2   -9
## OKI  1005    0   0   2   -9
## OKI  1006    0   0   1   -9
## OKI  1007    0   0   1   -9
## OKI  1008    0   0   1   -9
## OKI  1009    0   0   1   -9
## OKI  1010    0   0   1   -9  
 Now we have to select only the 18 samples for our comparisons. 
       plink2   \  
   --allow-extra-chr  \  
   --bfile output/wgs_vs_chip/chip_500_dp_01  \  
   --make-bed  \  
   --keep output/wgs_vs_chip/chip_samples_subset.txt  \  
   --out output/wgs_vs_chip/chip_500_dp_02  \  
   --silent ;  
    # --keep-allele-order \ if you use Plink 1.9  
    grep   "variants\|samples"  output/wgs_vs_chip/chip_500_dp_02.log  # to get the number of variants from the log file.     
  ##   --keep output/wgs_vs_chip/chip_samples_subset.txt
## 479 samples (138 females, 130 males, 211 ambiguous; 479 founders) loaded from
## 107294 variants loaded from output/wgs_vs_chip/chip_500_dp_01.bim.
## --keep: 18 samples remaining.
## 18 samples (0 females, 18 males; 18 founders) remaining after main filters.  
 Check the .fam file 
       head  output/wgs_vs_chip/chip_500_dp_02.fam    
  ## KAT  7   0   0   1   -9
## KAT  8   0   0   1   -9
## KAT  9   0   0   1   -9
## KAT  10  0   0   1   -9
## KAT  11  0   0   1   -9
## KAT  12  0   0   1   -9
## SAI  4   0   0   1   -9
## SAI  5   0   0   1   -9
## SAI  1   0   0   1   -9
## SAI  2   0   0   1   -9  
 After fixing the sex and add letter 
       head  output/wgs_vs_chip/chip_500_dp_03.fam    
  ## KAT  7c  0   0   2   -9
## KAT  8c  0   0   2   -9
## KAT  9c  0   0   2   -9
## KAT  10c 0   0   2   -9
## KAT  11c 0   0   2   -9
## KAT  12c 0   0   2   -9
## SAI  4c  0   0   2   -9
## SAI  5c  0   0   2   -9
## SAI  1c  0   0   2   -9
## SAI  2c  0   0   2   -9  
 Check “w” wgs call with 800+ samples 
       head  output/wgs_vs_chip/wgs_02.fam    
  ## SAI  5w  0   0   2   -9
## SAI  4w  0   0   2   -9
## SAI  3w  0   0   2   -9
## SAI  2w  0   0   2   -9
## SAI  1w  0   0   2   -9
## SAI  18w 0   0   2   -9
## SAI  17w 0   0   2   -9
## SAI  16w 0   0   2   -9
## SAI  15w 0   0   2   -9
## SAI  14w 0   0   2   -9  
 Check “x” wgs call with 30 samples (I added the x manually after
dupplicating the files and adding x) 
       head  output/wgs_vs_chip/new_calls/wgs_chip_30x.fam    
  ## KAT 10x 0 0 2 -9
## KAT 11x 0 0 2 -9
## KAT 12x 0 0 2 -9
## KAT 7x 0 0 2 -9
## KAT 8x 0 0 2 -9
## KAT 9x 0 0 2 -9
## KAT 1x 0 0 1 -9
## KAT 2x 0 0 1 -9
## KAT 3x 0 0 1 -9
## KAT 4x 0 0 1 -9  
 Check “y” wgs call with 18 samples 
       head  output/wgs_vs_chip/new_calls/wgs_chip_18y.fam    
  ## KAT 10y 0 0 2 -9
## KAT 11y 0 0 2 -9
## KAT 12y 0 0 2 -9
## KAT 7y 0 0 2 -9
## KAT 8y 0 0 2 -9
## KAT 9y 0 0 2 -9
## SAI 1y 0 0 2 -9
## SAI 2y 0 0 2 -9
## SAI 3y 0 0 2 -9
## SAI 4y 0 0 2 -9  
 
 
 8.2 Merge data sets 
 Now we can merge the files into a single bed file. We set all the
reference alleles to match the reference genome in every data set. This
is crucial for our comparisons. We also need to use –keep-allele-order
if we use Plink 1.9 
 We can create a list of the files to merge 
       # chip  
    echo   'output/wgs_vs_chip/chip_dp_01  
    output/wgs_vs_chip/chip_plate_dp_03  
    output/wgs_vs_chip/chip_500_dp_03  
    '   >  output/wgs_vs_chip/merge_list_2.txt 
    
    # wgs  
    echo   'output/wgs_vs_chip/wgs_02  
    output/wgs_vs_chip/new_calls/wgs_chip_30x  
    output/wgs_vs_chip/new_calls/wgs_chip_18y  
    '   >  output/wgs_vs_chip/merge_list_3.txt    
 Merge the chip bed files 
       plink   \  
   --allow-extra-chr  \  
   --keep-allele-order  \  
   --merge-list output/wgs_vs_chip/merge_list_2.txt  \  
   --out output/wgs_vs_chip/chip_3_datasets  \  
   --silent 
    
    grep   "variants\|samples"  output/wgs_vs_chip/chip_3_datasets.log    
 Merge the wgs bed files 
       plink   \  
   --allow-extra-chr  \  
   --keep-allele-order  \  
   --merge-list output/wgs_vs_chip/merge_list_3.txt  \  
   --out output/wgs_vs_chip/wgs_3_datasets  \  
   --silent 
    
    grep   "variants\|samples"  output/wgs_vs_chip/wgs_3_datasets.log    
 
 
 8.3 WGS calls with different alternative alleles 
 When we run Plink to merge the files, we get an error about sites
having three alleles. It happens because we did genotype calls using
only 18, 30 or 819 samples, we end up with different alleles. We used
angsd which is a population based algorithm for genotype calls. Plink
creates a list of SNPs that have more than 2 alleles. We can check it
later. Lets count how many SNPs: 
       wc   -l  output/wgs_vs_chip/wgs_3_datasets.missnp    
  ##     2755 output/wgs_vs_chip/wgs_3_datasets.missnp  
 Let’s see how many SNPs have this problem once we decrease the sample
size 
       # wgs 2 - 819 samples vs 30 samples  
    echo   'output/wgs_vs_chip/wgs_02  
    output/wgs_vs_chip/new_calls/wgs_chip_30x  
    '   >  output/wgs_vs_chip/merge_list_4.txt ;  
    
    # wgs 3 -  891 samples vs 18 samples  
    echo   'output/wgs_vs_chip/wgs_02  
    output/wgs_vs_chip/new_calls/wgs_chip_18y  
    '   >  output/wgs_vs_chip/merge_list_5.txt ;  
    
    # wgs 4 - 30 samples vc 18 samples  
    echo   'output/wgs_vs_chip/new_calls/wgs_chip_30x  
    output/wgs_vs_chip/new_calls/wgs_chip_18y  
    '   >  output/wgs_vs_chip/merge_list_6.txt ;     
 Now we can try to merge them to see how many SNPs have different
alleles 
 30 versus 819 samples 
       plink   \  
   --allow-extra-chr  \  
   --keep-allele-order  \  
   --merge-list output/wgs_vs_chip/merge_list_4.txt  \  
   --out output/wgs_vs_chip/wgs_800_vs_30_samples  \  
   --silent 
    
    grep   "variants\|samples"  output/wgs_vs_chip/wgs_800_vs_30_samples.log    
 We have 2,245 SNPs with 3+ alleles. It happens because the
alternative alleles are different in each data set 
       wc   -l  output/wgs_vs_chip/wgs_800_vs_30_samples.missnp    
  ##     2245 output/wgs_vs_chip/wgs_800_vs_30_samples.missnp  
 18 versus 819 samples 
       plink   \  
   --allow-extra-chr  \  
   --keep-allele-order  \  
   --merge-list output/wgs_vs_chip/merge_list_5.txt  \  
   --out output/wgs_vs_chip/wgs_800_vs_18_samples  \  
   --silent 
    
    grep   "variants\|samples"  output/wgs_vs_chip/wgs_800_vs_18_samples.log    
 We have 2,257 SNPs with 3+ alleles 
       wc   -l  output/wgs_vs_chip/wgs_800_vs_18_samples.missnp    
  ##     2257 output/wgs_vs_chip/wgs_800_vs_18_samples.missnp  
 18 versus 30 samples 
       plink   \  
   --allow-extra-chr  \  
   --keep-allele-order  \  
   --merge-list output/wgs_vs_chip/merge_list_6.txt  \  
   --out output/wgs_vs_chip/wgs_18_vs_30_samples  \  
   --silent 
    
    grep   "variants\|samples"  output/wgs_vs_chip/wgs_18_vs_30_samples.log    
 We have 882 SNPs with 3+ alleles 
       wc   -l  output/wgs_vs_chip/wgs_18_vs_30_samples.missnp    
  ##      882 output/wgs_vs_chip/wgs_18_vs_30_samples.missnp  
 We can get the list of SNPs 
       cat  output/wgs_vs_chip/wgs_800_vs_30_samples.missnp output/wgs_vs_chip/wgs_800_vs_18_samples.missnp output/wgs_vs_chip/wgs_18_vs_30_samples.missnp  |   awk   '!seen[$0]++'   >  output/wgs_vs_chip/SNPs_wgs_3_alleles.txt ;  
    wc   -l  output/wgs_vs_chip/SNPs_wgs_3_alleles.txt    
  ##     2755 output/wgs_vs_chip/SNPs_wgs_3_alleles.txt  
 We need to remove the 2,755 SNPs. 
 We can remove these SNPs and only compare the other ones. Lets double
check to make sure we have only bi-allelic data as well. Perhaps that is
why we see inconsistencies between the genotype calls from the chip and
wgs 
 Exclude from 18 samples 
       plink   \  
   --bfile output/wgs_vs_chip/new_calls/wgs_chip_18y  \  
   --allow-extra-chr  \  
   --keep-allele-order  \  
   --biallelic-only  \  
   --exclude output/wgs_vs_chip/SNPs_wgs_3_alleles.txt  \  
   --out output/wgs_vs_chip/new_calls/wgs_chip_18y_b  \  
   --make-bed  \  
   --silent 
    
    grep   "variants\|samples"  output/wgs_vs_chip/new_calls/wgs_chip_18y_b.log    
 Exclude from 30 samples 
       plink   \  
   --bfile output/wgs_vs_chip/new_calls/wgs_chip_30x  \  
   --allow-extra-chr  \  
   --keep-allele-order  \  
   --biallelic-only  \  
   --exclude output/wgs_vs_chip/SNPs_wgs_3_alleles.txt  \  
   --out output/wgs_vs_chip/new_calls/wgs_chip_30x_b  \  
   --make-bed  \  
   --silent 
    
    grep   "variants\|samples"  output/wgs_vs_chip/new_calls/wgs_chip_30x_b.log    
 Exclude from data subset with 819 samples 
       plink   \  
   --bfile output/wgs_vs_chip/wgs_02  \  
   --allow-extra-chr  \  
   --keep-allele-order  \  
   --biallelic-only  \  
   --exclude output/wgs_vs_chip/SNPs_wgs_3_alleles.txt  \  
   --out output/wgs_vs_chip/wgs_03  \  
   --make-bed  \  
   --silent 
    
    grep   "variants\|samples"  output/wgs_vs_chip/wgs_03.log    
 Exclude from data subset from 30 samples 
       plink   \  
   --bfile output/wgs_vs_chip/new_calls/wgs_chip_30x  \  
   --allow-extra-chr  \  
   --keep-allele-order  \  
   --biallelic-only  \  
   --exclude output/wgs_vs_chip/SNPs_wgs_3_alleles_b.txt  \  
   --out output/wgs_vs_chip/new_calls/wgs_chip_30x_c  \  
   --make-bed  \  
   --silent 
    
    grep   "variants\|samples"  output/wgs_vs_chip/new_calls/wgs_chip_30x_c.log    
 Exclude from data subset from 18 samples 
       plink   \  
   --bfile output/wgs_vs_chip/new_calls/wgs_chip_18y  \  
   --allow-extra-chr  \  
   --keep-allele-order  \  
   --biallelic-only  \  
   --exclude output/wgs_vs_chip/SNPs_wgs_3_alleles_b.txt  \  
   --out output/wgs_vs_chip/new_calls/wgs_chip_18y_c  \  
   --make-bed  \  
   --silent 
    
    grep   "variants\|samples"  output/wgs_vs_chip/new_calls/wgs_chip_18y_c.log    
 Now create new list to merge. We can merge all files (chip and wgs)
into one single file, but first lets create one file with the wgs
samples only 
       # wgs   
    echo   'output/wgs_vs_chip/wgs_03  
    output/wgs_vs_chip/new_calls/wgs_chip_30x_b  
    output/wgs_vs_chip/new_calls/wgs_chip_18y_b  
    '   >  output/wgs_vs_chip/merge_list_7.txt 
    
    # all  
    echo   'output/wgs_vs_chip/wgs_03  
    output/wgs_vs_chip/new_calls/wgs_chip_30x_b  
    output/wgs_vs_chip/new_calls/wgs_chip_18y_b  
    output/wgs_vs_chip/chip_dp_01  
    output/wgs_vs_chip/chip_plate_dp_03  
    output/wgs_vs_chip/chip_500_dp_03  
    '   >  output/wgs_vs_chip/merge_list_8.txt    
 WGS 
       plink   \  
   --allow-extra-chr  \  
   --keep-allele-order  \  
   --merge-list output/wgs_vs_chip/merge_list_7.txt  \  
   --out output/wgs_vs_chip/wgs_3_datasets_b  \  
   --silent ;  
    
    grep   "variants\|samples"  output/wgs_vs_chip/wgs_3_datasets_b.log    
 Merge all data sets 
       plink   \  
   --allow-extra-chr  \  
   --keep-allele-order  \  
   --merge-list output/wgs_vs_chip/merge_list_8.txt  \  
   --out output/wgs_vs_chip/wgs_chip_merged  \  
   --silent 
    
    grep   "variants\|samples"  output/wgs_vs_chip/wgs_chip_merged.log    
 Now, we have to set the reference allele to match the reference
genome: we remove SNPs with more than 1 alternative allele due to
genotype calls with low sample size, and create a single file. 
       head   -n  25 output/wgs_vs_chip/wgs_chip_merged.fam    
  ## KAT  1x  0   0   1   -9
## KAT  2x  0   0   1   -9
## KAT  3x  0   0   1   -9
## KAT  4x  0   0   1   -9
## KAT  5x  0   0   1   -9
## KAT  6x  0   0   1   -9
## KAT  7a  0   0   2   -9
## KAT  7b  0   0   2   -9
## KAT  7c  0   0   2   -9
## KAT  7w  0   0   2   -9
## KAT  7x  0   0   2   -9
## KAT  7y  0   0   2   -9
## KAT  8a  0   0   2   -9
## KAT  8b  0   0   2   -9
## KAT  8c  0   0   2   -9
## KAT  8w  0   0   2   -9
## KAT  8x  0   0   2   -9
## KAT  8y  0   0   2   -9
## KAT  9a  0   0   2   -9
## KAT  9b  0   0   2   -9
## KAT  9c  0   0   2   -9
## KAT  9w  0   0   2   -9
## KAT  9x  0   0   2   -9
## KAT  9y  0   0   2   -9
## KAT  10a 0   0   2   -9  
 
 
 
 9. Create vcf files for all comparison 
 The 18 samples were extracted when more samples were used for the
genotype call 
  a : chip - call with 18 samples  b :
chip - call with 95 samples (full plate)  c : chip - call
with 500+ samples  w : wgs - call with 800+ samples
 x : wgs - call with 30 samples (all wgs samples for both
populations)  y : wgs - call with 18 samples (only
samples with wgs and chip) 
 We do not need to do all pairwise comparisons. 
  Chip : ab, ac, bc  WGS : wx, wy, xy
 WGS versus chip : aw, ax, ay, bw, bx, by, cw, cx, cy 
 All comparisons (* those I will focus on) 
  Chip  ab - chip_18 vs chip_95 ac - chip_18 vs
chip_500 * bc - chip_95 vs chip_500 
  WGS  wx - wgs_800 vs wgs_30 wy - wgs_800 vs wgs_18 *
xy - wgs_18 vs wgs_30 
  Chip vs WGS  aw - chip_18 vs wgs_800   ax -
chip_18 vs wgs_30 ay - chip_18 vs wgs_18   bw - chip_95 vs wgs_800
bx - chip_95 vs wgs_30 by - chip_95 vs wgs_18 cw - chip_500 vs wgs_800
  cx - chip_500 vs wgs_30 cy - chip_500 vs wgs_18   
 
 9.1 Create all the vcfs 
       input_file  =  "output/wgs_vs_chip/wgs_chip_merged.fam"  
    output_dir  =  "output/wgs_vs_chip/vcfs2"  
    bfile  =  "output/wgs_vs_chip/wgs_chip_merged"  
    
    # create the output directory if it does not exist  
    mkdir   -p   $output_dir  
    
    # get unique families  
    families  =  $(  awk   '{print $1}'   $input_file   |   sort   |   uniq  )  
    
    for  famid  in   $families  ;   do  
      # get the base sample ids (without a, b, w)  
      base_iids  =  $(  grep   "  $famid  "   $input_file   |   awk   '{print $2}'   |   sed   's/[abcwxy]$//'   |   uniq  )  
      
      for  base_iid  in   $base_iids  ;   do  
        for  combination  in   "ab"   "ac"   "bc"   "wx"   "wy"   "xy"   "aw"   "ax"   "ay"   "bw"   "bx"   "by"   "cw"   "cx"   "cy"  ;   do  
          # Check if both samples exist  
          if   grep   -qE   "  ${famid}  \s  ${base_iid}  [  ${combination  :  0  :  1  }  ]\s"   "  $input_file  "   &&   
             grep   -qE   "  ${famid}  \s  ${base_iid}  [  ${combination  :  1  :  1  }  ]\s"   "  $input_file  "  ;   then  
            # Create temporary file  
            tmp_file  =  $(  mktemp  )  
            grep   -E   "  ${famid}  \s  ${base_iid}  [  ${combination  :  0  :  1  }  ]\s"   "  $input_file  "   >   "  $tmp_file  "  
            grep   -E   "  ${famid}  \s  ${base_iid}  [  ${combination  :  1  :  1  }  ]\s"   "  $input_file  "   >>   "  $tmp_file  "  
      
            # Execute plink2  
            plink2   \  
            --allow-extra-chr   \  
            --keep-allele-order   \  
            --bfile   $bfile   \  
            --keep   "  $tmp_file  "   \  
            --recode  vcf-iid  \  
            --geno  0  \  
            --out   "  $output_dir  /  ${famid}  _  ${base_iid}${combination}  "   \  
            --silent  
      
            # Remove temporary file  
            rm   "  $tmp_file  "  
          fi  
        done  
      done  
    done     
 Check how many SNPs per vcf 
       # Define directory with the vcfs  
    output_dir  =  "output/wgs_vs_chip/vcfs2"  
    # Count how many SNPs we have in each vcf file  
    for  file  in   ${output_dir} / * .vcf ;   do  
        echo   $(  basename   $file) :  $(  grep   -v   '^#'   $file   |   wc   -l  )  
    done     
  ## KAT_10ab.vcf: 88159
## KAT_10ac.vcf: 87404
## KAT_10aw.vcf: 101950
## KAT_10ax.vcf: 99196
## KAT_10ay.vcf: 96789
## KAT_10bc.vcf: 92187
## KAT_10bw.vcf: 100087
## KAT_10bx.vcf: 97433
## KAT_10by.vcf: 95133
## KAT_10cw.vcf: 100694
## KAT_10cx.vcf: 97956
## KAT_10cy.vcf: 95593
## KAT_10wx.vcf: 167052
## KAT_10wy.vcf: 162472
## KAT_10xy.vcf: 162349
## KAT_11ab.vcf: 88052
## KAT_11ac.vcf: 87326
## KAT_11aw.vcf: 101657
## KAT_11ax.vcf: 98913
## KAT_11ay.vcf: 96503
## KAT_11bc.vcf: 92556
## KAT_11bw.vcf: 100431
## KAT_11bx.vcf: 97766
## KAT_11by.vcf: 95463
## KAT_11cw.vcf: 101043
## KAT_11cx.vcf: 98301
## KAT_11cy.vcf: 95930
## KAT_11wx.vcf: 167052
## KAT_11wy.vcf: 162472
## KAT_11xy.vcf: 162349
## KAT_12ab.vcf: 87462
## KAT_12ac.vcf: 86666
## KAT_12aw.vcf: 101153
## KAT_12ax.vcf: 98413
## KAT_12ay.vcf: 95990
## KAT_12bc.vcf: 91243
## KAT_12bw.vcf: 99186
## KAT_12bx.vcf: 96579
## KAT_12by.vcf: 94294
## KAT_12cw.vcf: 99524
## KAT_12cx.vcf: 96821
## KAT_12cy.vcf: 94467
## KAT_12wx.vcf: 167052
## KAT_12wy.vcf: 162472
## KAT_12xy.vcf: 162349
## KAT_7ab.vcf: 88177
## KAT_7ac.vcf: 87404
## KAT_7aw.vcf: 101913
## KAT_7ax.vcf: 99167
## KAT_7ay.vcf: 96757
## KAT_7bc.vcf: 92119
## KAT_7bw.vcf: 100143
## KAT_7bx.vcf: 97489
## KAT_7by.vcf: 95188
## KAT_7cw.vcf: 100648
## KAT_7cx.vcf: 97909
## KAT_7cy.vcf: 95539
## KAT_7wx.vcf: 167052
## KAT_7wy.vcf: 162472
## KAT_7xy.vcf: 162349
## KAT_8ab.vcf: 87828
## KAT_8ac.vcf: 87045
## KAT_8aw.vcf: 101493
## KAT_8ax.vcf: 98756
## KAT_8ay.vcf: 96344
## KAT_8bc.vcf: 91879
## KAT_8bw.vcf: 99824
## KAT_8bx.vcf: 97180
## KAT_8by.vcf: 94881
## KAT_8cw.vcf: 100319
## KAT_8cx.vcf: 97577
## KAT_8cy.vcf: 95212
## KAT_8wx.vcf: 167052
## KAT_8wy.vcf: 162472
## KAT_8xy.vcf: 162349
## KAT_9ab.vcf: 87964
## KAT_9ac.vcf: 87230
## KAT_9aw.vcf: 101750
## KAT_9ax.vcf: 99001
## KAT_9ay.vcf: 96585
## KAT_9bc.vcf: 91906
## KAT_9bw.vcf: 99797
## KAT_9bx.vcf: 97158
## KAT_9by.vcf: 94873
## KAT_9cw.vcf: 100447
## KAT_9cx.vcf: 97711
## KAT_9cy.vcf: 95341
## KAT_9wx.vcf: 167052
## KAT_9wy.vcf: 162472
## KAT_9xy.vcf: 162349
## SAI_12ab.vcf: 87741
## SAI_12ac.vcf: 87510
## SAI_12aw.vcf: 101471
## SAI_12ax.vcf: 98706
## SAI_12ay.vcf: 96286
## SAI_12bc.vcf: 93098
## SAI_12bw.vcf: 100688
## SAI_12bx.vcf: 98024
## SAI_12by.vcf: 95713
## SAI_12cw.vcf: 102927
## SAI_12cx.vcf: 100107
## SAI_12cy.vcf: 97679
## SAI_12wx.vcf: 167052
## SAI_12wy.vcf: 162472
## SAI_12xy.vcf: 162349
## SAI_13ab.vcf: 87660
## SAI_13ac.vcf: 87456
## SAI_13aw.vcf: 101401
## SAI_13ax.vcf: 98651
## SAI_13ay.vcf: 96226
## SAI_13bc.vcf: 93075
## SAI_13bw.vcf: 100586
## SAI_13bx.vcf: 97927
## SAI_13by.vcf: 95618
## SAI_13cw.vcf: 102993
## SAI_13cx.vcf: 100179
## SAI_13cy.vcf: 97751
## SAI_13wx.vcf: 167052
## SAI_13wy.vcf: 162472
## SAI_13xy.vcf: 162349
## SAI_14ab.vcf: 87533
## SAI_14ac.vcf: 87237
## SAI_14aw.vcf: 101271
## SAI_14ax.vcf: 98518
## SAI_14ay.vcf: 96097
## SAI_14bc.vcf: 92921
## SAI_14bw.vcf: 100489
## SAI_14bx.vcf: 97823
## SAI_14by.vcf: 95505
## SAI_14cw.vcf: 102829
## SAI_14cx.vcf: 100004
## SAI_14cy.vcf: 97585
## SAI_14wx.vcf: 167052
## SAI_14wy.vcf: 162472
## SAI_14xy.vcf: 162349
## SAI_15ab.vcf: 87864
## SAI_15ac.vcf: 87468
## SAI_15aw.vcf: 101625
## SAI_15ax.vcf: 98868
## SAI_15ay.vcf: 96432
## SAI_15bc.vcf: 93104
## SAI_15bw.vcf: 100799
## SAI_15bx.vcf: 98133
## SAI_15by.vcf: 95815
## SAI_15cw.vcf: 102932
## SAI_15cx.vcf: 100132
## SAI_15cy.vcf: 97690
## SAI_15wx.vcf: 167052
## SAI_15wy.vcf: 162472
## SAI_15xy.vcf: 162349
## SAI_16ab.vcf: 87927
## SAI_16ac.vcf: 87597
## SAI_16aw.vcf: 101603
## SAI_16ax.vcf: 98843
## SAI_16ay.vcf: 96400
## SAI_16bc.vcf: 93231
## SAI_16bw.vcf: 100806
## SAI_16bx.vcf: 98139
## SAI_16by.vcf: 95831
## SAI_16cw.vcf: 103026
## SAI_16cx.vcf: 100200
## SAI_16cy.vcf: 97775
## SAI_16wx.vcf: 167052
## SAI_16wy.vcf: 162472
## SAI_16xy.vcf: 162349
## SAI_17ab.vcf: 87744
## SAI_17ac.vcf: 87447
## SAI_17aw.vcf: 101417
## SAI_17ax.vcf: 98666
## SAI_17ay.vcf: 96242
## SAI_17bc.vcf: 93112
## SAI_17bw.vcf: 100736
## SAI_17bx.vcf: 98062
## SAI_17by.vcf: 95751
## SAI_17cw.vcf: 102914
## SAI_17cx.vcf: 100092
## SAI_17cy.vcf: 97664
## SAI_17wx.vcf: 167052
## SAI_17wy.vcf: 162472
## SAI_17xy.vcf: 162349
## SAI_18ab.vcf: 87935
## SAI_18ac.vcf: 87564
## SAI_18aw.vcf: 101797
## SAI_18ax.vcf: 99029
## SAI_18ay.vcf: 96601
## SAI_18bc.vcf: 93301
## SAI_18bw.vcf: 101047
## SAI_18bx.vcf: 98377
## SAI_18by.vcf: 96048
## SAI_18cw.vcf: 103184
## SAI_18cx.vcf: 100357
## SAI_18cy.vcf: 97911
## SAI_18wx.vcf: 167052
## SAI_18wy.vcf: 162472
## SAI_18xy.vcf: 162349
## SAI_1ab.vcf: 87689
## SAI_1ac.vcf: 87385
## SAI_1aw.vcf: 101429
## SAI_1ax.vcf: 98673
## SAI_1ay.vcf: 96245
## SAI_1bc.vcf: 93177
## SAI_1bw.vcf: 100815
## SAI_1bx.vcf: 98143
## SAI_1by.vcf: 95812
## SAI_1cw.vcf: 103214
## SAI_1cx.vcf: 100379
## SAI_1cy.vcf: 97949
## SAI_1wx.vcf: 167052
## SAI_1wy.vcf: 162472
## SAI_1xy.vcf: 162349
## SAI_2ab.vcf: 87657
## SAI_2ac.vcf: 87426
## SAI_2aw.vcf: 101355
## SAI_2ax.vcf: 98620
## SAI_2ay.vcf: 96190
## SAI_2bc.vcf: 93082
## SAI_2bw.vcf: 100677
## SAI_2bx.vcf: 98014
## SAI_2by.vcf: 95688
## SAI_2cw.vcf: 103010
## SAI_2cx.vcf: 100205
## SAI_2cy.vcf: 97774
## SAI_2wx.vcf: 167052
## SAI_2wy.vcf: 162472
## SAI_2xy.vcf: 162349
## SAI_3ab.vcf: 87880
## SAI_3ac.vcf: 87578
## SAI_3aw.vcf: 101643
## SAI_3ax.vcf: 98887
## SAI_3ay.vcf: 96457
## SAI_3bc.vcf: 93174
## SAI_3bw.vcf: 100786
## SAI_3bx.vcf: 98121
## SAI_3by.vcf: 95804
## SAI_3cw.vcf: 103002
## SAI_3cx.vcf: 100179
## SAI_3cy.vcf: 97756
## SAI_3wx.vcf: 167052
## SAI_3wy.vcf: 162472
## SAI_3xy.vcf: 162349
## SAI_4ab.vcf: 87863
## SAI_4ac.vcf: 87521
## SAI_4aw.vcf: 101639
## SAI_4ax.vcf: 98872
## SAI_4ay.vcf: 96440
## SAI_4bc.vcf: 93447
## SAI_4bw.vcf: 101201
## SAI_4bx.vcf: 98511
## SAI_4by.vcf: 96177
## SAI_4cw.vcf: 103372
## SAI_4cx.vcf: 100533
## SAI_4cy.vcf: 98094
## SAI_4wx.vcf: 167052
## SAI_4wy.vcf: 162472
## SAI_4xy.vcf: 162349
## SAI_5ab.vcf: 87833
## SAI_5ac.vcf: 87542
## SAI_5aw.vcf: 101537
## SAI_5ax.vcf: 98775
## SAI_5ay.vcf: 96350
## SAI_5bc.vcf: 93216
## SAI_5bw.vcf: 100802
## SAI_5bx.vcf: 98141
## SAI_5by.vcf: 95826
## SAI_5cw.vcf: 102992
## SAI_5cx.vcf: 100175
## SAI_5cy.vcf: 97746
## SAI_5wx.vcf: 167052
## SAI_5wy.vcf: 162472
## SAI_5xy.vcf: 162349  
 Since we set genotyping missingness to zero within each pair of
samples, we see different number of SNPs in each vcf. 
 Check sample names to see if our code created the vcfs with two
samples 
       # Define directory with the VCFs  
    output_dir  =  "output/wgs_vs_chip/vcfs2"  
    
    # Iterate over each VCF file  
    for  file  in   "  ${output_dir}  " / * .vcf ;   do  
        # Extract the file name without the directory path  
        file_name  =  $(  basename   "  ${file}  "  )  
    
        # Use bcftools query to retrieve the sample names  
        sample_names  =  $(  bcftools  query  -l   "  ${file}  "  )  
        
        # Print the file name and the sample names  
        echo   "  ${file_name}  :   ${sample_names}  "  
    done     
  ## KAT_10ab.vcf: 10a
## 10b
## KAT_10ac.vcf: 10a
## 10c
## KAT_10aw.vcf: 10a
## 10w
## KAT_10ax.vcf: 10a
## 10x
## KAT_10ay.vcf: 10a
## 10y
## KAT_10bc.vcf: 10b
## 10c
## KAT_10bw.vcf: 10b
## 10w
## KAT_10bx.vcf: 10b
## 10x
## KAT_10by.vcf: 10b
## 10y
## KAT_10cw.vcf: 10c
## 10w
## KAT_10cx.vcf: 10c
## 10x
## KAT_10cy.vcf: 10c
## 10y
## KAT_10wx.vcf: 10w
## 10x
## KAT_10wy.vcf: 10w
## 10y
## KAT_10xy.vcf: 10x
## 10y
## KAT_11ab.vcf: 11a
## 11b
## KAT_11ac.vcf: 11a
## 11c
## KAT_11aw.vcf: 11a
## 11w
## KAT_11ax.vcf: 11a
## 11x
## KAT_11ay.vcf: 11a
## 11y
## KAT_11bc.vcf: 11b
## 11c
## KAT_11bw.vcf: 11b
## 11w
## KAT_11bx.vcf: 11b
## 11x
## KAT_11by.vcf: 11b
## 11y
## KAT_11cw.vcf: 11c
## 11w
## KAT_11cx.vcf: 11c
## 11x
## KAT_11cy.vcf: 11c
## 11y
## KAT_11wx.vcf: 11w
## 11x
## KAT_11wy.vcf: 11w
## 11y
## KAT_11xy.vcf: 11x
## 11y
## KAT_12ab.vcf: 12a
## 12b
## KAT_12ac.vcf: 12a
## 12c
## KAT_12aw.vcf: 12a
## 12w
## KAT_12ax.vcf: 12a
## 12x
## KAT_12ay.vcf: 12a
## 12y
## KAT_12bc.vcf: 12b
## 12c
## KAT_12bw.vcf: 12b
## 12w
## KAT_12bx.vcf: 12b
## 12x
## KAT_12by.vcf: 12b
## 12y
## KAT_12cw.vcf: 12c
## 12w
## KAT_12cx.vcf: 12c
## 12x
## KAT_12cy.vcf: 12c
## 12y
## KAT_12wx.vcf: 12w
## 12x
## KAT_12wy.vcf: 12w
## 12y
## KAT_12xy.vcf: 12x
## 12y
## KAT_7ab.vcf: 7a
## 7b
## KAT_7ac.vcf: 7a
## 7c
## KAT_7aw.vcf: 7a
## 7w
## KAT_7ax.vcf: 7a
## 7x
## KAT_7ay.vcf: 7a
## 7y
## KAT_7bc.vcf: 7b
## 7c
## KAT_7bw.vcf: 7b
## 7w
## KAT_7bx.vcf: 7b
## 7x
## KAT_7by.vcf: 7b
## 7y
## KAT_7cw.vcf: 7c
## 7w
## KAT_7cx.vcf: 7c
## 7x
## KAT_7cy.vcf: 7c
## 7y
## KAT_7wx.vcf: 7w
## 7x
## KAT_7wy.vcf: 7w
## 7y
## KAT_7xy.vcf: 7x
## 7y
## KAT_8ab.vcf: 8a
## 8b
## KAT_8ac.vcf: 8a
## 8c
## KAT_8aw.vcf: 8a
## 8w
## KAT_8ax.vcf: 8a
## 8x
## KAT_8ay.vcf: 8a
## 8y
## KAT_8bc.vcf: 8b
## 8c
## KAT_8bw.vcf: 8b
## 8w
## KAT_8bx.vcf: 8b
## 8x
## KAT_8by.vcf: 8b
## 8y
## KAT_8cw.vcf: 8c
## 8w
## KAT_8cx.vcf: 8c
## 8x
## KAT_8cy.vcf: 8c
## 8y
## KAT_8wx.vcf: 8w
## 8x
## KAT_8wy.vcf: 8w
## 8y
## KAT_8xy.vcf: 8x
## 8y
## KAT_9ab.vcf: 9a
## 9b
## KAT_9ac.vcf: 9a
## 9c
## KAT_9aw.vcf: 9a
## 9w
## KAT_9ax.vcf: 9a
## 9x
## KAT_9ay.vcf: 9a
## 9y
## KAT_9bc.vcf: 9b
## 9c
## KAT_9bw.vcf: 9b
## 9w
## KAT_9bx.vcf: 9b
## 9x
## KAT_9by.vcf: 9b
## 9y
## KAT_9cw.vcf: 9c
## 9w
## KAT_9cx.vcf: 9c
## 9x
## KAT_9cy.vcf: 9c
## 9y
## KAT_9wx.vcf: 9w
## 9x
## KAT_9wy.vcf: 9w
## 9y
## KAT_9xy.vcf: 9x
## 9y
## SAI_12ab.vcf: 12a
## 12b
## SAI_12ac.vcf: 12a
## 12c
## SAI_12aw.vcf: 12a
## 12w
## SAI_12ax.vcf: 12a
## 12x
## SAI_12ay.vcf: 12a
## 12y
## SAI_12bc.vcf: 12b
## 12c
## SAI_12bw.vcf: 12b
## 12w
## SAI_12bx.vcf: 12b
## 12x
## SAI_12by.vcf: 12b
## 12y
## SAI_12cw.vcf: 12c
## 12w
## SAI_12cx.vcf: 12c
## 12x
## SAI_12cy.vcf: 12c
## 12y
## SAI_12wx.vcf: 12w
## 12x
## SAI_12wy.vcf: 12w
## 12y
## SAI_12xy.vcf: 12x
## 12y
## SAI_13ab.vcf: 13a
## 13b
## SAI_13ac.vcf: 13a
## 13c
## SAI_13aw.vcf: 13a
## 13w
## SAI_13ax.vcf: 13a
## 13x
## SAI_13ay.vcf: 13a
## 13y
## SAI_13bc.vcf: 13b
## 13c
## SAI_13bw.vcf: 13b
## 13w
## SAI_13bx.vcf: 13b
## 13x
## SAI_13by.vcf: 13b
## 13y
## SAI_13cw.vcf: 13c
## 13w
## SAI_13cx.vcf: 13c
## 13x
## SAI_13cy.vcf: 13c
## 13y
## SAI_13wx.vcf: 13w
## 13x
## SAI_13wy.vcf: 13w
## 13y
## SAI_13xy.vcf: 13x
## 13y
## SAI_14ab.vcf: 14a
## 14b
## SAI_14ac.vcf: 14a
## 14c
## SAI_14aw.vcf: 14a
## 14w
## SAI_14ax.vcf: 14a
## 14x
## SAI_14ay.vcf: 14a
## 14y
## SAI_14bc.vcf: 14b
## 14c
## SAI_14bw.vcf: 14b
## 14w
## SAI_14bx.vcf: 14b
## 14x
## SAI_14by.vcf: 14b
## 14y
## SAI_14cw.vcf: 14c
## 14w
## SAI_14cx.vcf: 14c
## 14x
## SAI_14cy.vcf: 14c
## 14y
## SAI_14wx.vcf: 14w
## 14x
## SAI_14wy.vcf: 14w
## 14y
## SAI_14xy.vcf: 14x
## 14y
## SAI_15ab.vcf: 15a
## 15b
## SAI_15ac.vcf: 15a
## 15c
## SAI_15aw.vcf: 15a
## 15w
## SAI_15ax.vcf: 15a
## 15x
## SAI_15ay.vcf: 15a
## 15y
## SAI_15bc.vcf: 15b
## 15c
## SAI_15bw.vcf: 15b
## 15w
## SAI_15bx.vcf: 15b
## 15x
## SAI_15by.vcf: 15b
## 15y
## SAI_15cw.vcf: 15c
## 15w
## SAI_15cx.vcf: 15c
## 15x
## SAI_15cy.vcf: 15c
## 15y
## SAI_15wx.vcf: 15w
## 15x
## SAI_15wy.vcf: 15w
## 15y
## SAI_15xy.vcf: 15x
## 15y
## SAI_16ab.vcf: 16a
## 16b
## SAI_16ac.vcf: 16a
## 16c
## SAI_16aw.vcf: 16a
## 16w
## SAI_16ax.vcf: 16a
## 16x
## SAI_16ay.vcf: 16a
## 16y
## SAI_16bc.vcf: 16b
## 16c
## SAI_16bw.vcf: 16b
## 16w
## SAI_16bx.vcf: 16b
## 16x
## SAI_16by.vcf: 16b
## 16y
## SAI_16cw.vcf: 16c
## 16w
## SAI_16cx.vcf: 16c
## 16x
## SAI_16cy.vcf: 16c
## 16y
## SAI_16wx.vcf: 16w
## 16x
## SAI_16wy.vcf: 16w
## 16y
## SAI_16xy.vcf: 16x
## 16y
## SAI_17ab.vcf: 17a
## 17b
## SAI_17ac.vcf: 17a
## 17c
## SAI_17aw.vcf: 17a
## 17w
## SAI_17ax.vcf: 17a
## 17x
## SAI_17ay.vcf: 17a
## 17y
## SAI_17bc.vcf: 17b
## 17c
## SAI_17bw.vcf: 17b
## 17w
## SAI_17bx.vcf: 17b
## 17x
## SAI_17by.vcf: 17b
## 17y
## SAI_17cw.vcf: 17c
## 17w
## SAI_17cx.vcf: 17c
## 17x
## SAI_17cy.vcf: 17c
## 17y
## SAI_17wx.vcf: 17w
## 17x
## SAI_17wy.vcf: 17w
## 17y
## SAI_17xy.vcf: 17x
## 17y
## SAI_18ab.vcf: 18a
## 18b
## SAI_18ac.vcf: 18a
## 18c
## SAI_18aw.vcf: 18a
## 18w
## SAI_18ax.vcf: 18a
## 18x
## SAI_18ay.vcf: 18a
## 18y
## SAI_18bc.vcf: 18b
## 18c
## SAI_18bw.vcf: 18b
## 18w
## SAI_18bx.vcf: 18b
## 18x
## SAI_18by.vcf: 18b
## 18y
## SAI_18cw.vcf: 18c
## 18w
## SAI_18cx.vcf: 18c
## 18x
## SAI_18cy.vcf: 18c
## 18y
## SAI_18wx.vcf: 18w
## 18x
## SAI_18wy.vcf: 18w
## 18y
## SAI_18xy.vcf: 18x
## 18y
## SAI_1ab.vcf: 1a
## 1b
## SAI_1ac.vcf: 1a
## 1c
## SAI_1aw.vcf: 1a
## 1w
## SAI_1ax.vcf: 1a
## 1x
## SAI_1ay.vcf: 1a
## 1y
## SAI_1bc.vcf: 1b
## 1c
## SAI_1bw.vcf: 1b
## 1w
## SAI_1bx.vcf: 1b
## 1x
## SAI_1by.vcf: 1b
## 1y
## SAI_1cw.vcf: 1c
## 1w
## SAI_1cx.vcf: 1c
## 1x
## SAI_1cy.vcf: 1c
## 1y
## SAI_1wx.vcf: 1w
## 1x
## SAI_1wy.vcf: 1w
## 1y
## SAI_1xy.vcf: 1x
## 1y
## SAI_2ab.vcf: 2a
## 2b
## SAI_2ac.vcf: 2a
## 2c
## SAI_2aw.vcf: 2a
## 2w
## SAI_2ax.vcf: 2a
## 2x
## SAI_2ay.vcf: 2a
## 2y
## SAI_2bc.vcf: 2b
## 2c
## SAI_2bw.vcf: 2b
## 2w
## SAI_2bx.vcf: 2b
## 2x
## SAI_2by.vcf: 2b
## 2y
## SAI_2cw.vcf: 2c
## 2w
## SAI_2cx.vcf: 2c
## 2x
## SAI_2cy.vcf: 2c
## 2y
## SAI_2wx.vcf: 2w
## 2x
## SAI_2wy.vcf: 2w
## 2y
## SAI_2xy.vcf: 2x
## 2y
## SAI_3ab.vcf: 3a
## 3b
## SAI_3ac.vcf: 3a
## 3c
## SAI_3aw.vcf: 3a
## 3w
## SAI_3ax.vcf: 3a
## 3x
## SAI_3ay.vcf: 3a
## 3y
## SAI_3bc.vcf: 3b
## 3c
## SAI_3bw.vcf: 3b
## 3w
## SAI_3bx.vcf: 3b
## 3x
## SAI_3by.vcf: 3b
## 3y
## SAI_3cw.vcf: 3c
## 3w
## SAI_3cx.vcf: 3c
## 3x
## SAI_3cy.vcf: 3c
## 3y
## SAI_3wx.vcf: 3w
## 3x
## SAI_3wy.vcf: 3w
## 3y
## SAI_3xy.vcf: 3x
## 3y
## SAI_4ab.vcf: 4a
## 4b
## SAI_4ac.vcf: 4a
## 4c
## SAI_4aw.vcf: 4a
## 4w
## SAI_4ax.vcf: 4a
## 4x
## SAI_4ay.vcf: 4a
## 4y
## SAI_4bc.vcf: 4b
## 4c
## SAI_4bw.vcf: 4b
## 4w
## SAI_4bx.vcf: 4b
## 4x
## SAI_4by.vcf: 4b
## 4y
## SAI_4cw.vcf: 4c
## 4w
## SAI_4cx.vcf: 4c
## 4x
## SAI_4cy.vcf: 4c
## 4y
## SAI_4wx.vcf: 4w
## 4x
## SAI_4wy.vcf: 4w
## 4y
## SAI_4xy.vcf: 4x
## 4y
## SAI_5ab.vcf: 5a
## 5b
## SAI_5ac.vcf: 5a
## 5c
## SAI_5aw.vcf: 5a
## 5w
## SAI_5ax.vcf: 5a
## 5x
## SAI_5ay.vcf: 5a
## 5y
## SAI_5bc.vcf: 5b
## 5c
## SAI_5bw.vcf: 5b
## 5w
## SAI_5bx.vcf: 5b
## 5x
## SAI_5by.vcf: 5b
## 5y
## SAI_5cw.vcf: 5c
## 5w
## SAI_5cx.vcf: 5c
## 5x
## SAI_5cy.vcf: 5c
## 5y
## SAI_5wx.vcf: 5w
## 5x
## SAI_5wy.vcf: 5w
## 5y
## SAI_5xy.vcf: 5x
## 5y  
 
 
 9.2 Pairwise comparisons summary 
 Compare the two samples in each vcf file and create csv output across
all samples 
       import  allel 
    import  pandas  as  pd 
    import  os 
    import  numpy  as  np 
    
    # Initialize the output dataframe  
   output_df  =  pd.DataFrame() 
    
    # Directory with vcf files  
   dir_name  =   "output/wgs_vs_chip/vcfs2/"  
    
    # Get list of all vcf files in the directory  
   vcf_files  =  [f  for  f  in  os.listdir(dir_name)  if  f.endswith( '.vcf' )] 
    
    # Iterate over VCF files  
    for  vcf_file  in  vcf_files: 
       file_path  =  os.path.join(dir_name, vcf_file) 
       callset  =  allel.read_vcf(file_path, fields = [ '*' ]) 
    
        # Get genotype  
       gt  =  allel.GenotypeArray(callset[ 'calldata/GT' ]) 
        
        # Verify the vcf contains two samples  
        assert  gt.shape[ 1 ]  ==   2 ,  f"Expected 2 samples in   { vcf_file }  , found   { gt . shape[ 1 ] }  "  
    
        # Count SNPs  
       n_snps  =   len (gt) 
    
        # Count homozygous and heterozygous SNPs for each sample  
       n_homo_ref  =  np.count_nonzero(gt.is_hom_ref(), axis =  0 ) 
       n_homo_alt  =  np.count_nonzero(gt.is_hom_alt(), axis =  0 ) 
       n_hetero  =  np.count_nonzero(gt.is_het(), axis =  0 ) 
        
        # Count homozygous and heterozygous SNPs mismatches  
       n_homo_ref_mismatch  =  np. sum (gt.is_hom_ref()[:,  0 ]  !=  gt.is_hom_ref()[:,  1 ]) 
       n_homo_alt_mismatch  =  np. sum (gt.is_hom_alt()[:,  0 ]  !=  gt.is_hom_alt()[:,  1 ]) 
       n_hetero_mismatch  =  np. sum (gt.is_het()[:,  0 ]  !=  gt.is_het()[:,  1 ]) 
    
        # Get alleles  
       ref_alleles  =  callset[ 'variants/REF' ] 
       alt_alleles  =  callset[ 'variants/ALT' ][:,  0 ]   # assuming bi-allelic  
    
        # Count mismatching reference and alternative alleles  
       n_snps_ref_mismatch  =  np.count_nonzero(ref_alleles[gt[:, 0 ]]  !=  ref_alleles[gt[:, 1 ]]) 
       n_snps_alt_mismatch  =  np.count_nonzero(alt_alleles[gt[:, 0 ]]  !=  alt_alleles[gt[:, 1 ]]) 
    
        # Count alleles for each sample  
       n_a  =   sum (np.count_nonzero(gt  ==  i, axis =  0 )  for  i  in   range ( 4 )  if  ref_alleles[i]  ==   'A'   or  alt_alleles[i]  ==   'A' ) 
       n_t  =   sum (np.count_nonzero(gt  ==  i, axis =  0 )  for  i  in   range ( 4 )  if  ref_alleles[i]  ==   'T'   or  alt_alleles[i]  ==   'T' ) 
       n_c  =   sum (np.count_nonzero(gt  ==  i, axis =  0 )  for  i  in   range ( 4 )  if  ref_alleles[i]  ==   'C'   or  alt_alleles[i]  ==   'C' ) 
       n_g  =   sum (np.count_nonzero(gt  ==  i, axis =  0 )  for  i  in   range ( 4 )  if  ref_alleles[i]  ==   'G'   or  alt_alleles[i]  ==   'G' ) 
    
        # Append results to the output dataframe  
       result  =  pd.DataFrame({ 
            'vcf_file' : [file_path], 
            'n_SNPs' : [n_snps], 
            'n_SNPs_ref_mismatch' : [n_snps_ref_mismatch], 
            'n_SNPs_alt_mismatch' : [n_snps_alt_mismatch], 
            'n_A' : [n_a], 
            'n_T' : [n_t], 
            'n_C' : [n_c], 
            'n_G' : [n_g], 
            'n_homo_ref' : [n_homo_ref], 
            'n_homo_alt' : [n_homo_alt], 
            'n_hetero' : [n_hetero], 
            'n_homo_ref_mismatch' : [n_homo_ref_mismatch], 
            'n_homo_alt_mismatch' : [n_homo_alt_mismatch], 
            'n_hetero_mismatch' : [n_hetero_mismatch] 
       }) 
    
       output_df  =  pd.concat([output_df, result]) 
    
    # Write the result to a csv file  
   output_df.to_csv( 'output/wgs_vs_chip/vcfs2/allele_comparison_stats.csv' , index =  False )    
 Clean env 
       # python  
    py_run_string ( "import gc; gc.collect()" )    
 Import the data 
      data  <-  
      read_delim ( 
        "output/wgs_vs_chip/vcfs2/allele_comparison_stats.csv" , 
        delim =   "," , 
        show_col_types =   FALSE  
     )  
    
   data  <-  
     data  |>  
      mutate ( vcf_file =   str_remove (vcf_file,  "output/wgs_vs_chip/vcfs2/" ))  |>  
      separate ( 
       vcf_file, 
        into =   c ( "Population" ,  "Sample_Comparison" ), 
        sep =   "_" , 
        extra =   "drop"  
     )  |>  
      separate ( 
       Sample_Comparison, 
        into =   c ( "Sample" ,  "Comparison" ), 
        sep =   "(?<=  \\  d)(?=[a-z])" , 
        convert =   TRUE  
     )  |>  
      mutate ( Comparison =   str_remove (Comparison,  ".vcf" ))  |>  
      arrange (Comparison) 
    
    # Split the "Comparison" column into "Sample1" and "Sample2"  
   data  <-   
     data  |>  
      separate ( 
       Comparison, 
        into =   c ( "Sample1" ,  "Sample2" ), 
        sep =   1 , 
        # because each comparison has two characters  
        remove =   FALSE  
     )  |>   # keep the original comparison column  
      relocate (Sample1, Sample2,  .after =  Comparison)  # move the new columns right after Comparison  
    
   cols_to_split  <-  
      c ( "n_A" , 
        "n_T" , 
        "n_C" , 
        "n_G" , 
        "n_homo_ref" , 
        "n_homo_alt" , 
        "n_hetero" ) 
    
    # Remove unwanted characters from the columns  
    for  (col_name  in  cols_to_split) { 
     data[[col_name]]  <-   gsub ( "  \\  [  \\  [|]  \\  n" ,  "" , data[[col_name]]) 
   } 
    
    # Split the columns  
    for  (col_name  in  cols_to_split) { 
      # Create new column names based on 'Sample1' and 'Sample2'  
     new_col_names  <-   paste0 (col_name,  "_sample" ,  1  :  2 ) 
      
     data  <-  data  |>  
        separate ( 
          col =  col_name, 
          into =  new_col_names, 
          sep =   " " , 
          extra =   "drop"  
       ) 
   } 
    
    # Clean the new columns  
   cols_to_clean  <-   
      grep ( "^n_" ,  names (data),  value =   TRUE ) 
    
    for  (col_name  in  cols_to_clean) { 
      # Remove unwanted characters '[', ']', and '\n'  
     data[[col_name]]  <-   gsub ( "  \\  [|]|  \\  n" ,  "" , data[[col_name]]) 
   } 
    
    # Specify the column names to convert to numeric  
   columns_to_convert  <-  
      c ( 
        # "Population",  
        "Sample" , 
        # "Comparison",  
        # "Sample1",  
        # "Sample2",  
        "n_SNPs" , 
        "n_SNPs_ref_mismatch" , 
        "n_SNPs_alt_mismatch" , 
        "n_A_sample1" , 
        "n_A_sample2" , 
        "n_T_sample1" , 
        "n_T_sample2" , 
        "n_C_sample1" , 
        "n_C_sample2" , 
        "n_G_sample1" , 
        "n_G_sample2" , 
        "n_homo_ref_sample1" , 
        "n_homo_ref_sample2" , 
        "n_homo_alt_sample1" , 
        "n_homo_alt_sample2" , 
        "n_hetero_sample1" , 
        "n_hetero_sample2" , 
        "n_homo_ref_mismatch" , 
        "n_homo_alt_mismatch" , 
        "n_hetero_mismatch"  
     ) 
    
    # Convert columns to numeric  
   data[columns_to_convert]  <-  
      lapply (data[columns_to_convert],  function (x) 
        as.numeric ( as.character (x))) 
    
    # Verify the column types  
    print ( sapply (data[columns_to_convert], class))    
  ##              Sample              n_SNPs n_SNPs_ref_mismatch n_SNPs_alt_mismatch 
##           "numeric"           "numeric"           "numeric"           "numeric" 
##         n_A_sample1         n_A_sample2         n_T_sample1         n_T_sample2 
##           "numeric"           "numeric"           "numeric"           "numeric" 
##         n_C_sample1         n_C_sample2         n_G_sample1         n_G_sample2 
##           "numeric"           "numeric"           "numeric"           "numeric" 
##  n_homo_ref_sample1  n_homo_ref_sample2  n_homo_alt_sample1  n_homo_alt_sample2 
##           "numeric"           "numeric"           "numeric"           "numeric" 
##    n_hetero_sample1    n_hetero_sample2 n_homo_ref_mismatch n_homo_alt_mismatch 
##           "numeric"           "numeric"           "numeric"           "numeric" 
##   n_hetero_mismatch 
##           "numeric"  
 We can look over all the comparisons to see if we can see any
pattern 
       # Calculate the percentages for each category  
   data  <-  
     data  |>  
      mutate ( 
        Perc_n_homo_ref_mismatch =   round ((n_homo_ref_mismatch  /  n_SNPs)  *   100 ,  2 ), 
        Perc_n_homo_alt_mismatch =   round ((n_homo_alt_mismatch  /  n_SNPs)  *   100 ,  2 ), 
        Perc_n_hetero_mismatch =   round ((n_hetero_mismatch  /  n_SNPs)  *   100 ,  2 ) 
     ) 
    
    # Continue with the reshaping  
   data_long  <-  data  |>  
      pivot_longer ( cols =   starts_with ( "n_" ), 
                   names_to =   "Category" , 
                   values_to =   "Value" )  |>  
      pivot_longer ( cols =   starts_with ( "Perc_" ), 
                   names_to =   "Category_Perc" , 
                   values_to =   "Percentage" )  |>  
      mutate ( Category_Perc =   str_remove (Category_Perc,  "Perc_" ))  |>  
      filter (Category  ==  Category_Perc  |  
              Category  ==   "n_SNPs" )   # Remove Total_mismatch  
    
    # Define a color palette  
   color_palette  <-   c ( "#FF8C94" ,  "#FFE180" ,  "#9CE09C" ,  "#A391FF" ) 
    
    # Rename categories  
   data_long  <-  data_long  |>  
      mutate ( 
        Category =   recode ( 
         Category, 
          "n_SNPs"   =   "SNPs" , 
          "n_homo_ref_mismatch"   =   "Homozygous REF" , 
          "n_homo_alt_mismatch"   =   "Homozygous ALT" , 
          "n_hetero_mismatch"   =   "Heterozygous"  
       ) 
     ) 
    
    # Change the order of the Comparison variable (Chip, WGS, and Chip vs WGS)  
   data_long $ Comparison  <-  
      factor ( 
       data_long $ Comparison, 
        levels =   c ( 
          "ab" , 
          "ac" , 
          "bc" , 
          "wx" , 
          "wy" , 
          "xy" , 
          "aw" , 
          "ax" , 
          "ay" , 
          "bw" , 
          "bx" , 
          "by" , 
          "cw" , 
          "cx" , 
          "cy"  
       ) 
     ) 
    
    # Recode the levels of the "Comparison" variable  
   data_long $ Comparison  <-   recode ( 
     data_long $ Comparison, 
      "ab"   =   "chip_18 : chip_95" , 
      "ac"   =   "chip_18 : chip_500" , 
      "bc"   =   "chip_95 : chip_500" , 
      "wx"   =   "wgs_800 : wgs_30" , 
      "wy"   =   "wgs_800 : wgs_18" , 
      "xy"   =   "wgs_18 : wgs_30" , 
      "aw"   =   "chip_18 : wgs_800" , 
      "ax"   =   "chip_18 : wgs_30" , 
      "ay"   =   "chip_18 : wgs_18" , 
      "bw"   =   "chip_95 : wgs_800" , 
      "bx"   =   "chip_95 : wgs_30" , 
      "by"   =   "chip_95 : wgs_18" , 
      "cw"   =   "chip_500 : wgs_800" , 
      "cx"   =   "chip_500 : wgs_30" , 
      "cy"   =   "chip_500 : wgs_18"  
   ) 
    
    # Create the plot  
    ggplot (data_long,  aes ( x =  Category,  y =  Value,  fill =  Category))  +  
      geom_bar ( stat =   "identity" ,  position =   "dodge" )  +  
      facet_grid (Comparison  ~  Population,  scales =   "free_y" ,  space =   "free" )  +  
      coord_flip ()  +  
      labs ( 
        title =   "Mismatches of zygosity in pairwise comparisons" , 
        x =   "Category" , 
        y =   "Count" , 
        caption =   "The comparison are between SNPs genotyped in both samples.  \n   Each sample as genotyped with a different number of samples. Each pair of sample was subseted to  \n   a vcf file allowing no genotyping missingness. Next, with custom python script,  \n   the total number of genotypes matches and mismatches was stored in a csv file.   \n  The data was tidy and visualized in R.  \n  REF = Reference allele; ALT = Alternative allele  \n   At right are the number of samples used in the genotype calls  \n   for each data set comparison."  
     )  +  
      theme ( panel.spacing =   unit ( 1.5 ,  "lines" ))  +  
      geom_text ( 
        aes ( label =   ifelse ( 
         Category  ==   "SNPs" , 
         scales ::  comma (Value), 
          paste0 (scales ::  comma (Value),  " (" ,  sprintf ( "%.2f" , Percentage),  "%)" ) 
       )), 
        position =   position_dodge ( width =   0.7 ), 
        hjust =   0.9 , 
        vjust =   0.5 , 
        size =   2.5 , 
        check_overlap =   TRUE , 
        color =   "black"  
     )  +  
      scale_fill_manual ( values =  color_palette)  +  
      theme ( axis.text.x =   element_text ( angle =   45 ,  hjust =   1 ))  +  
      guides ( fill =   "none" )  +  
      my_theme ()  +  
      scale_y_continuous ( 
        labels =  scales :: comma, 
        trans =   "log10" , 
        breaks =   c ( 10 ,  100 ,  1000 ,  10000 ,  100000 ), 
        limits =   c ( 1 ,  NA ), 
        expand =   expansion ( mult =   c ( 0 ,  0.1 )) 
     )  +  
      theme ( 
        plot.caption =   element_text ( 
          face =   "italic" , 
          size =   8 , 
          color =   "grey20"  
       ), 
        plot.margin =   unit ( c ( 1 ,  2 ,  1 ,  1 ),  "cm" ), 
        axis.text.x =   element_text ( angle =   0 ,  hjust =   1 ), 
        axis.text =   element_text ( size =   7 ), 
        strip.text.y =   element_text ( angle =   360 ) 
     )    
   
       # save the plot  
    ggsave ( 
      here ( 
        "output" , 
        "wgs_vs_chip" , 
        "figures" , 
        "01.Pairwise_comparions_wgs_chip.pdf"  
     ), 
      width  =   8 , 
      height =   14 , 
      units  =   "in"  
   )    
 We see the lowest mismatch rate for the comparisons within each
technology was used independently to how many samples the sample was
genotyped with. The chip seems slightly better for smaller sample sizes.
The sample size seems not to affect the overall result of the
technologies comparisons. The mismatch rate in SAI (island invasive
range) is higher than KAT (continent native range), indicating the
presence of low frequency alleles that might might be difficult to
detect. 
 Next, we can look at the performance of the technologies across all
18 samples. Our next questions are a bit different. For example, how
many SNPs have mismatches in 1, 2 or more samples? Are there any SNPs
that have errors in more than 2 samples? Can we find a way to identify
them and remove them? 
 Since the sample size with which the genotype call was performed does
not affect the overall results, we can select a few comparisons to look
into in detail. We then can compare the read count for each allele from
the WGS with the mismatch rate. Do the SNPs with mismatches between the
technologies have a lower read count? If so, what is the error rate if
we remove SNPs that had 1 or a few reads? Does the output of the
comparison improve the concordance between the technologies? We can do
that selecting 1 or two data sets. For example, we can select chip_18:
wgs_18 (ay) and chip_500: wgs_800 (cw). Then we will compare the
genotypes of samples that were genotyped using only 18 samples or the
entire data set we had (around 500 samples for the chip and 800 for the
wgs data set). We extracted the 18 samples from our large data set for
comparisons. 
 We can also compare if the SNPs with mismatches are the same when the
sample size for the genotype call is large or small. What percentage of
SNPs have mismatches when varying the sample size? We can do the same
comparison for each technology. 
 Save the data first 
       # Save the data  
    saveRDS ( 
     data_long, 
      file =   here ( 
        "output" , 
        "wgs_vs_chip" , 
        "pairwise_comparison_long.rds"  
     ) 
   ) 
    
    # Clean environment and memory  
    rm ( list =   ls ()) 
    gc ()    
  ##            used  (Mb) gc trigger  (Mb) limit (Mb) max used  (Mb)
## Ncells 10847074 579.3   17143224 915.6         NA 15008399 801.6
## Vcells 19261980 147.0   37740698 288.0      32768 37739631 288.0  
       # # Load the data  
    # data_long <-  
    #   readRDS(  
    #     file = here(  
    #       "output",  
    #       "wgs_vs_chip",  
    #       "pairwise_comparison_long.rds"  
    #     )  
    #   )     
 
 
 9.3 Script to process vcf files and function to import csv
files 
 Python script to get the match and mismatches from a vcf file 
       import  argparse 
    import  allel 
    import  pandas  as  pd 
    import  os 
    import  numpy  as  np 
    import  warnings 
    
    # Ignore DtypeWarnings from pandas  
   warnings.filterwarnings( 'ignore' , category = pd.errors.DtypeWarning) 
    
    # Function to convert genotype indices to alleles  
    def  genotype_to_alleles(gt_indices, ref_allele, alt_alleles): 
       alleles  =  np.concatenate(([ref_allele], alt_alleles)) 
        return   " " .join(alleles[idx]  for  idx  in  gt_indices  if  idx !=-  1 )   # idx -1 means missing data  
    
    def  process_vcf_files(vcf_file_ending): 
       dir_name  =   "output/wgs_vs_chip/vcfs2/"  
       vcf_files  =  [f  for  f  in  os.listdir(dir_name)  if  f.endswith( f'  { vcf_file_ending }  .vcf' )] 
        
        if   not  vcf_files: 
            raise   ValueError ( f"No VCF files found matching '  { vcf_file_ending }  '" ) 
    
        for  vcf_file  in  vcf_files: 
           file_path  =  os.path.join(dir_name, vcf_file) 
           callset  =  allel.read_vcf(file_path, fields = [ '*' ]) 
    
            # Get genotype  
           gt  =  allel.GenotypeArray(callset[ 'calldata/GT' ]) 
    
            # Get sample names and add prefix from file name  
           sample_1, sample_2  =  callset[ 'samples' ] 
           prefix  =  vcf_file.split( "_" )[ 0 ]  +   "_"    # Added "_" after prefix  
           sample_1  =  prefix  +  sample_1 
           sample_2  =  prefix  +  sample_2 
    
            # Verify the vcf contains two samples  
            assert  gt.shape[ 1 ]  ==   2 ,  f"Expected 2 samples in   { vcf_file }  , found   { gt . shape[ 1 ] }  "  
    
            # Create DataFrame  
           df  =  pd.DataFrame({ 
                'SNP_id' : callset[ 'variants/ID' ], 
                f'  { sample_1 }  _geno' : [genotype_to_alleles(gt, callset[ 'variants/REF' ][i], callset[ 'variants/ALT' ][i])  for  i, gt  in   enumerate (gt[:,  0 ])], 
                f'  { sample_2 }  _geno' : [genotype_to_alleles(gt, callset[ 'variants/REF' ][i], callset[ 'variants/ALT' ][i])  for  i, gt  in   enumerate (gt[:,  1 ])], 
                f'  { sample_1 }  _  { sample_2 }  _gcomp' : np.where(gt[:,  0 ]  ==  gt[:,  1 ],  'match' ,  'mismatch' ).tolist(), 
                f'  { sample_1 }  _zygo' : np.where(gt.is_hom_ref()[:,  0 ],  'hom_ref' , np.where(gt.is_hom_alt()[:,  0 ],  'hom_alt' ,  'het' )).tolist(), 
                f'  { sample_2 }  _zygo' : np.where(gt.is_hom_ref()[:,  1 ],  'hom_ref' , np.where(gt.is_hom_alt()[:,  1 ],  'hom_alt' ,  'het' )).tolist(), 
                f'  { sample_1 }  _  { sample_2 }  _zcomp' : np.where(gt.is_hom()[:,  0 ]  ==  gt.is_hom()[:,  1 ],  'match' ,  'mismatch' ).tolist() 
           }) 
    
            # When you write your output file, use the input filename to create the corresponding output filename  
           output_file  =   f'output/wgs_vs_chip/  { os . path . basename(vcf_file) . replace( ".vcf" ,  "" ) }  _comparison.csv'  
           df.to_csv(output_file, index =  False ) 
    
    def  combine_csv_files(vcf_file_ending): 
        # Combine only the newly created CSVs into one  
       dir_path  =   "output/wgs_vs_chip/"  
       csv_files  =  [os.path.join(dir_path, f)  for  f  in  os.listdir(dir_path)  if  f.endswith( f'  { vcf_file_ending }  _comparison.csv' )] 
    
        # Ensure that we have at least one such file  
        if   not  csv_files: 
            raise   ValueError ( f"No CSV files found matching '  { vcf_file_ending }  _comparison.csv'" ) 
    
        # Load the first CSV file  
       combined_csv  =  pd.read_csv(csv_files[ 0 ]) 
    
        # Merge the rest of the CSV files one by one  
        for  f  in  csv_files[ 1 :]: 
           df  =  pd.read_csv(f) 
           combined_csv  =  pd.merge(combined_csv, df, on =  'SNP_id' , how =  'outer' ) 
    
       combined_csv.to_csv(os.path.join(dir_path,  f'combined_comparison_  { vcf_file_ending }  .csv' ), index =  False ) 
    
    def  main(): 
        # Initialize parser  
       parser  =  argparse.ArgumentParser(description =  "Process VCF files and output CSV comparison files" ) 
    
        # Add argument  
       parser.add_argument( 'vcf_file_ending' ,  type  =  str ,  help  =  "The ending for VCF files to be processed (e.g., 'ay')" ) 
    
        # Parse arguments  
       args  =  parser.parse_args() 
    
        # Remove '.vcf' from the ending, if present  
       vcf_file_ending  =  args.vcf_file_ending.replace( '.vcf' ,  '' ) 
    
        # Process VCF files and combine CSV files  
       process_vcf_files(vcf_file_ending) 
       combine_csv_files(vcf_file_ending) 
    
    if   __name__   ==   "__main__" : 
       main()    
 How to run the Python script 
       python  output/wgs_vs_chip/scripts/create_csv_from_vcfs.py ay    
 We can write a function to import and process the csv files our
python script generates 
      process_csv_files  <-   function (csv_file_ending) { 
      # Read the CSV file using fread() function  
     csv_file  <-   paste0 ( "output/wgs_vs_chip/combined_comparison_" , csv_file_ending,  ".csv" ) 
     data_dt  <-  data.table ::  fread (csv_file) 
      
      # Get all column names that end with '_gcomp'  
     gcomp_cols  <-   grep ( "_gcomp$" ,  names (data_dt),  value =   TRUE ) 
      
      # Convert data.frame to data.table  
      setDT (data_dt) 
      
      # Iterate over the '_gcomp' columns and create new '_REF' and '_ALT' columns  
      for  (col  in  gcomp_cols) { 
        # Split each '_gcomp' column into '_REF' and '_ALT'  
       ref_col  <-   paste0 (col,  "_REF" ) 
       alt_col  <-   paste0 (col,  "_ALT" ) 
       data_dt[,  c (ref_col, alt_col)  :=   tstrsplit ( get (col),  ", " ,  fixed =   TRUE )] 
        
        # Remove unwanted characters from each new column  
       data_dt[, (ref_col)  :=   gsub ( "  \\  [|  \\  ]|'" ,  "" ,  get (ref_col))] 
       data_dt[, (alt_col)  :=   gsub ( "  \\  [|  \\  ]|'" ,  "" ,  get (alt_col))] 
     } 
      
      # Rename columns to remove '_gcomp'  
     new_names  <-   names (data_dt) 
     new_names  <-   gsub ( "_gcomp_ALT$" ,  "_ALT" , new_names) 
     new_names  <-   gsub ( "_gcomp_REF$" ,  "_REF" , new_names) 
      setnames (data_dt, new_names) 
      setnames (data_dt, new_names) 
      
      # Return the processed data.table  
      return (data_dt) 
   } 
    
    # we can save the function to source it later  
    dump ( 
      "process_csv_files" , 
      here ( 
        "scripts" ,  "analysis" ,  "process_csv_files.R" ) 
   )    
 How to run the function to import the csv files 
      data_ay_dt  <-   process_csv_files ( "ay" ) 
    
    # Check and display only columns that match the criteria  
    head (data_ay_dt[,  c ( "SNP_id" ,  names (data_ay_dt)[ grepl ( "_REF$|_ALT$" ,  names (data_ay_dt))]),  with =   FALSE ])    
 Function to get summary of the mismatches 
      process_data_object  <-   function (object_name) { 
      # Get the data.table object based on the input name  
     data_dt  <-   get (object_name) 
      
      # Create columns for match and mismatch count for columns ending with _REF  
     cols_REF  <-   grep ( "_REF$" ,  names (data_dt),  value =   TRUE ) 
     data_dt[,  c ( "REF_match_count" ,  "REF_mismatch_count" )  :=  .( 
        rowSums (.SD  ==   "match" ,  na.rm =   TRUE ), 
        rowSums (.SD  ==   "mismatch" ,  na.rm =   TRUE ) 
     ), .SDcols  =  cols_REF] 
      
      # Create columns for match and mismatch count for columns ending with _ALT  
     cols_ALT  <-   grep ( "_ALT$" ,  names (data_dt),  value =   TRUE ) 
     data_dt[,  c ( "ALT_match_count" ,  "ALT_mismatch_count" )  :=  .( 
        rowSums (.SD  ==   "match" ,  na.rm =   TRUE ), 
        rowSums (.SD  ==   "mismatch" ,  na.rm =   TRUE ) 
     ), .SDcols  =  cols_ALT] 
      
      # Create columns for match and mismatch count for columns ending with _zcomp  
     cols_Zigo  <-   grep ( "_zcomp$" ,  names (data_dt),  value =   TRUE ) 
     data_dt[,  c ( "Zigo_match_count" ,  "Zigo_mismatch_count" )  :=  .( 
        rowSums (.SD  ==   "match" ,  na.rm =   TRUE ), 
        rowSums (.SD  ==   "mismatch" ,  na.rm =   TRUE ) 
     ), .SDcols  =  cols_Zigo] 
      
      # Summarize the data for each SNP_id  
     summary_dt  <-  data_dt[, .( 
        REF_match =   sum (REF_match_count,  na.rm =   TRUE ), 
        REF_mismatch =   sum (REF_mismatch_count,  na.rm =   TRUE ), 
        ALT_match =   sum (ALT_match_count,  na.rm =   TRUE ), 
        ALT_mismatch =   sum (ALT_mismatch_count,  na.rm =   TRUE ), 
        Zigo_match =   sum (Zigo_match_count,  na.rm =   TRUE ), 
        Zigo_mismatch =   sum (Zigo_mismatch_count,  na.rm =   TRUE ) 
     ), by  =  SNP_id] 
      
      # Sort the summarized data by SNP_id  
      setorder (summary_dt, SNP_id) 
      
      # Return the processed summary data.table  
      return (summary_dt) 
   } 
    
    # we can save the function to source it later  
    dump ( 
      "process_data_object" , 
      here ( 
        "scripts" ,  "analysis" ,  "process_data_object.R" ) 
   )    
 How to run the function 
      summary_ab  <-   process_data_object ( "data_ab_dt" )    
 Function to process the summaries for plotting 
      process_summary_object  <-   function (summary_object_name) { 
      # Select only the relevant columns  
     dt  <-   get (summary_object_name)[, .(SNP_id, REF_mismatch, ALT_mismatch, Zigo_mismatch)] 
      
      # Reshape data to long format  
     dt_long  <-  reshape2 ::  melt (dt,  id.vars =   "SNP_id" ,  variable.name =   "type" ,  value.name =   "count" ) 
      
      # Convert to data.table if it's not already  
      setDT (dt_long) 
      
      # Convert count to numeric if it's not already  
     dt_long[, count  :=   as.numeric (count)] 
      
      # Count occurrences per count value  
     dt_long  <-  dt_long[, .( n =  .N), by  =  .(type, count)] 
      
      # Calculate total count of unique SNPs  
     total_SNP  <-   length ( unique (dt $ SNP_id)) 
      
      # Add a new column for the percentage  
     dt_long[, perc  :=  n  /  total_SNP  *   100 ] 
      
      # Define new labels  
     new_labels  <-   c ( 
        "Reference Allele"   =   "REF_mismatch" , 
        "Alternative Allele"   =   "ALT_mismatch" , 
        "Zygosity Mismatch"   =   "Zigo_mismatch"  
     ) 
      
      # Apply new labels  
     dt_long $ type  <-  forcats ::  fct_recode (dt_long $ type,  !!! new_labels) 
      
      # Return the processed data.table  
      return (dt_long) 
   } 
    # we can save the function to source it later  
    dump ( 
      "process_summary_object" , 
      here ( 
        "scripts" ,  "analysis" ,  "process_summary_object.R" ) 
   )    
 How to run process_summary_objects function 
      dt_long_ab  <-   process_summary_object ( "summary_ab" )    
 Theme for plotting 
       # import plotting theme  
    source ( 
      here ( 
        "scripts" , 
        "analysis" , 
        "my_theme2.R"   # choose my_theme.R (Roboto Condensed) or my_theme2.R (default font)  
     ) 
   )    
 Function to errors per SNP per sample 
      plot_dt_long  <-   function (object_suffix) { 
      # Get the object name based on the suffix  
     object_name  <-   paste0 ( "dt_long_" , object_suffix) 
      
      # Get the corresponding data.table object  
     dt_long  <-   get (object_name) 
      
      # Create facet histogram  
     p  <-   ggplot (dt_long,  aes ( x =  count,  y =  n))  +  
        geom_bar ( 
          stat =   "identity" , 
          fill =   "#ffcae4" , 
          color =   ifelse ( 
           dt_long $ count  ==   0 , 
            "#CCFF00" , 
            ifelse (dt_long $ count  ==   1 ,  "#4169E1" ,  "#FF7F50" ) 
         ), 
          width =   0.6 , 
          linewidth =   1  
       )  +  
        geom_text ( 
          aes ( label =   paste0 ( 
           scales ::  comma (n),  " (" ,  round (perc,  2 ),  "%)"  
         )), 
          hjust =   ifelse (dt_long $ count  ==   0 , . 7 ,  0.01 ), 
          size =   2.3 , 
          color =   "gray10"  
       )  +  
        facet_wrap ( ~  type,  scales =   "free_y" )  +  
        labs ( 
          title =   paste ( "Histogram of SNP Mismatch Counts" , object_suffix), 
          x =   "Sample Count" , 
          y =   "SNP Count" , 
          caption =   paste (object_suffix,  "  \n   Bar border colors: Electric Lime = no errors; Royal Blue =  1 error; Coral = more than 1 error" ) 
       )  +  
        scale_y_continuous ( 
          breaks =   c ( 0 ,  25000 ,  50000 ,  75000 ,  100000 ,  125000 ,  150000 ,  175000 ), 
          labels =   function (x)  paste0 (x  /   1000 ,  "k" ), 
          expand =   expansion ( mult =   c ( 0 ,  0.2 )) 
       )  +  
        scale_x_continuous ( breaks =   0  :  18 ,  expand =   expansion ( add =   c ( 0.5 ,  0 )))  +  
        my_theme ()  +  
        coord_flip ()  +  
        theme ( 
          plot.caption =   element_text ( 
            face =   "italic" , 
            size =   10 , 
            color =   "grey20"  
         ), 
          panel.spacing =   unit ( 2 ,  "lines" ), 
          plot.margin =   unit ( c ( 1 ,  3 ,  1 ,  1 ),  "cm" ), 
          axis.text.x =   element_text ( size =   7 ,  angle =   0 )  
       ) 
      
      # Save the plot  
     output_file  <-   here ( "output" ,  "wgs_vs_chip" ,  "figures" ,  paste0 (object_suffix,  "_mismatches.pdf" )) 
      ggsave (output_file, p,  width =   8 ,  height =   6 ,  units =   "in" ) 
      
      # Return the plot object  
      return (p) 
   }    
 How to run the plotting function 
       plot_dt_long ( "ab" )    
 Function to get summary for each population 
      generate_summary  <-   function (data_dt, population, object_suffix) { 
      # Extract population columns  
     pop_cols  <-   grep ( paste0 ( "^" , population,  "_" ),  names (data_dt),  value =   TRUE ) 
      
      # Subset the data into population-specific data table  
     data_pop  <-  data_dt[,  c ( 'SNP_id' , pop_cols), with  =   FALSE ] 
      
      # Create columns for match and mismatch count for columns ending with _REF  
     cols_REF  <-   grep ( "_REF$" ,  names (data_pop),  value =   TRUE ) 
      
      # Calculate the count of "match" or "mismatch" for each row  
     data_pop[,  c ( "REF_match_count" ,  "REF_mismatch_count" )  :=  
                  .( rowSums (.SD  ==   "match" ,  na.rm =   TRUE ), 
                     rowSums (.SD  ==   "mismatch" ,  na.rm =   TRUE )), 
              .SDcols  =  cols_REF] 
      
      # Create columns for match and mismatch count for columns ending with _ALT  
     cols_ALT  <-   grep ( "_ALT$" ,  names (data_pop),  value =   TRUE ) 
      
      # Calculate the count of "match" or "mismatch" for each row  
     data_pop[,  c ( "ALT_match_count" ,  "ALT_mismatch_count" )  :=  
                  .( rowSums (.SD  ==   "match" ,  na.rm =   TRUE ), 
                     rowSums (.SD  ==   "mismatch" ,  na.rm =   TRUE )), 
              .SDcols  =  cols_ALT] 
      
      # Create columns for match and mismatch count for columns ending with _zcomp  
     cols_Zigo  <-   grep ( "_zcomp$" ,  names (data_pop),  value =   TRUE ) 
      
      # Calculate the count of "match" or "mismatch" for each row  
     data_pop[,  c ( "Zigo_match_count" ,  "Zigo_mismatch_count" )  :=  
                  .( rowSums (.SD  ==   "match" ,  na.rm =   TRUE ), 
                     rowSums (.SD  ==   "mismatch" ,  na.rm =   TRUE )), 
              .SDcols  =  cols_Zigo] 
      
      # Now, you can summarize this for each SNP_id  
     summary_pop  <-  data_pop[, .( 
        REF_match =   sum (REF_match_count,  na.rm =   TRUE ), 
        REF_mismatch =   sum (REF_mismatch_count,  na.rm =   TRUE ), 
        ALT_match =   sum (ALT_match_count,  na.rm =   TRUE ), 
        ALT_mismatch =   sum (ALT_mismatch_count,  na.rm =   TRUE ), 
        Zigo_match =   sum (Zigo_match_count,  na.rm =   TRUE ), 
        Zigo_mismatch =   sum (Zigo_mismatch_count,  na.rm =   TRUE ) 
     ), 
     by  =  SNP_id] 
      
      # Sort data by SNP_id  
      setorder (summary_pop, SNP_id) 
      
      # Assign the summary_pop object to a new variable based on the object_suffix  
     summary_pop_object_name  <-   paste0 ( "summary_" , population,  "_" , object_suffix) 
      assign (summary_pop_object_name, summary_pop,  envir =  .GlobalEnv) 
      
      # Return the summary_pop object  
      return (summary_pop) 
   }    
 How to run the functions 
      summary_sai_ay  <-   generate_summary (data_ay_dt,  "SAI" ,  "suffix" ) 
   summary_kat_ay  <-   generate_summary (data_ay_dt,  "KAT" ,  "suffix" ) 
   dt_long_2_ay  <-   merge_and_transform ( "ay" )    
 Function to merge the SAI and KAT summaries 
      merge_and_transform  <-   function (object_suffix) { 
      # Merge summary_sai and summary_kat  
     merged_sai_kat  <-   merge ( 
        get ( paste0 ( "summary_sai_" , object_suffix)), 
        get ( paste0 ( "summary_kat_" , object_suffix)), 
        by =   "SNP_id" , 
        suffixes =   c ( "_sai" ,  "_kat" ) 
     ) 
      
      # Select only the relevant columns  
     dt  <-  merged_sai_kat[, .( 
       SNP_id, 
       REF_mismatch_sai, 
       ALT_mismatch_sai, 
       Zigo_mismatch_sai, 
       REF_mismatch_kat, 
       ALT_mismatch_kat, 
       Zigo_mismatch_kat 
     )] 
      
      # Reshape data to long format  
     dt_long  <-   melt ( 
       dt, 
        id.vars =   "SNP_id" , 
        variable.name =   "type" , 
        value.name =   "count"  
     ) 
      
      # Convert to data.table if it's not already  
      setDT (dt_long) 
      
      # Extract the last part after "_" in the 'type' column to form 'group' column  
     dt_long[, group  :=   str_extract (type,  "(?<=_)[^_]+$" )] 
      
      # Extract the part before the first "_" in the 'type' column to form 'allele' column  
     dt_long[, allele  :=   str_extract (type,  "^[^_]+" )] 
      
      # Convert to numeric if it's not already  
     dt_long[, count  :=   as.numeric (count)] 
      
      # Count occurrences per count value  
     dt_long  <-  dt_long[, .( n =  .N), by  =  .(allele, group, count)] 
      
      # Calculate total count of unique SNPs  
     total_SNP  <-   length ( unique (dt $ SNP_id)) 
      
      # Add a new column for the percentage  
     dt_long[, perc  :=  n  /  total_SNP  *   100 , by  =  group] 
      
      # Set levels for 'group' variable  
     dt_long $ group  <-   factor (dt_long $ group,  levels =   c ( "sai" ,  "kat" )) 
      
      # Set levels for 'allele' variable  
     dt_long $ allele  <-   factor (dt_long $ allele,  levels =   c ( "REF" ,  "ALT" ,  "Zigo" )) 
      
      # Modify levels for 'allele' variable  
      levels (dt_long $ allele)  <-   c ( "Reference Allele" ,  "Alternative Allele" ,  "Zygosity" ) 
      
      # Modify levels for 'group' variable  
      levels (dt_long $ group)  <-   c ( "SAI" ,  "KAT" ) 
      
     dt_long $ count  <-   as.numeric (dt_long $ count) 
      
      # Assign the dt_long object to a new variable  
     dt_long_object_name  <-   paste0 ( "dt_long_" , object_suffix) 
      assign (dt_long_object_name, dt_long,  envir =  .GlobalEnv) 
      
      # Return the dt_long object  
      return (dt_long) 
   }    
 Function to create plot comparing the two populations 
      create_plot2  <-   function (object_suffix, output_path, dt_long) { 
      # Create plot  
     plot  <-   ggplot (dt_long,  aes ( x =  count,  y =  n))  +  
        geom_bar ( 
          stat =   "identity" , 
          fill =   "#ffcae4" , 
          color =   ifelse ( 
           dt_long $ count  ==   0 , 
            "#CCFF00" , 
            ifelse (dt_long $ count  ==   1 ,  "#4169E1" ,  "#FF7F50" ) 
         ), 
          width =   0.6 , 
          linewidth =   1  
       )  +  
        geom_text ( 
          aes ( label =   paste0 ( 
           scales ::  comma (n),  " (" ,  round (perc,  2 ),  "%)"  
         )), 
          hjust =   ifelse (dt_long $ count  ==   0 , . 7 ,  0.01 ), 
          size =   2.3 , 
          color =   "gray10"  
       )  +  
        facet_wrap ( ~  group  +  allele,  scales =   "free_y" ,  ncol =   3 )  +  
        labs ( 
          title =   paste ( "Histogram of SNP Mismatch Counts" , object_suffix), 
          x =   "Count" , 
          y =   "Frequency" , 
          caption =   paste ( 
           object_suffix, 
            "  \n   KAT 6 samples from native range         SAI 12 samples from invasive range  \n   Bar border colors: Electric Lime = no errors; Royal Blue =  1 error; Coral = more than 1 error"  
         ) 
       )  +  
        coord_flip ()  +  
        my_theme ()  +  
        # scale_y_continuous(labels = scales::comma) +  
        scale_y_continuous ( 
          breaks =   c ( 0 ,  25000 ,  50000 ,  75000 ,  100000 ,  125000 ,  150000 ,  175000 ), 
          labels =   function (x)  paste0 (x  /   1000 ,  "k" ), 
          expand =   expansion ( mult =   c ( 0 ,  0.2 )) 
       )  +  
        scale_x_continuous ( breaks =   0  :  18 )  +  
        theme ( 
          plot.caption =   element_text ( 
            face =   "italic" , 
            size =   10 , 
            color =   "grey20"  
         ), 
          panel.spacing =   unit ( 3 ,  "lines" ), 
          plot.margin =   unit ( c ( 1 ,  3 ,  1 ,  1 ),  "cm" ), 
          axis.text.x =   element_text ( size =   7 ,  angle =   0 )  
       ) 
      
      # Print the plot in RStudio  
      print (plot) 
      
      # Save the plot  
      ggsave ( 
       output_path, 
        plot =  plot, 
        width =   8 , 
        height =   8 , 
        units =   "in"  
     ) 
   }    
 How to run the functions 
      summary_sai_ay  <-   generate_summary_sai (data_ay_dt,  "ay" ) 
   summary_kat_ay  <-   generate_summary_kat (data_KAT,  "ay" ) 
   dt_long_2_ay  <-   merge_and_transform ( "ay" ) 
    create_plot2 ( "ay" ,  here ( "output" ,  "wgs_vs_chip" ,  "figures" ,  "ay_mismatches_SAI_KAT.pdf" ), dt_long_2_ay)    
 Function to get counts for pairwise comparison plot 
      calculate_counts  <-   function (data_dt) { 
      # Initialize an empty list to hold the counts  
     count_list  <-   list () 
    
      # Select columns  
     matching_columns  <-   colnames (data_dt)[ grepl ( pattern =   "(_REF$|_ALT$|_zcomp$)" ,  colnames (data_dt))] 
    
      # Loop through each column  
      for  (column  in  matching_columns) { 
       match_count  <-   sum ( str_detect (data_dt[[column]],  "match" ),  na.rm =   TRUE ) 
       mismatch_count  <-   sum ( str_detect (data_dt[[column]],  "mismatch" ),  na.rm =   TRUE ) 
    
        # Create a data.table with counts for the current column  
       count_dt  <-   data.table ( Column =  column,  Match =  match_count,  Mismatch =  mismatch_count) 
    
        # Add the count data.table to the list  
       count_list[[column]]  <-  count_dt 
     } 
    
      # Combine all count data.tables into a single data.table  
     counts  <-   rbindlist (count_list) 
    
      # Calculate total  
     counts  <-  counts  |>  
        mutate ( Total =  Match  +  Mismatch) 
    
      # Create new columns: Population, Sample, and Comparison  
     counts  <-  counts  |>  
        mutate ( 
          Population =   sub ( "^([^_]+).*" ,  "  \\  1" , Column), 
          Sample =   sub ( "^.*_(  \\  d+).*" ,  "  \\  1" , Column), 
          Comparison =   sub ( ".*_([^_]+)$" ,  "  \\  1" , Column) 
       ) 
    
      # Reorder the columns and create sample_id  
     counts  <-  counts  |>  
       dplyr ::  select (Population, Sample, Comparison, Match, Mismatch, Total) 
    
      # Calculate percentage columns  
     counts  <-  counts  |>  
        mutate ( 
          Percent_Match =   round ((Match  /  Total)  *   100 ,  2 ), 
          Percent_Mismatch =   round ((Mismatch  /  Total)  *   100 ,  2 ) 
       ) 
    
      # Replace zcomp with Zygosity  
     counts $ Comparison  <-   gsub ( "zcomp" ,  "Zygosity" , counts $ Comparison) 
    
      # Define color palette  
     color_palette  <-   c ( "#92C6FF" ,  "#f5cb8b" ,  "#bff28c" ) 
    
      # Convert Sample to numeric and sort samples numerically within each Population group  
     counts $ Sample  <-   as.numeric (counts $ Sample) 
     counts  <-  counts  |>   arrange (Population, Sample) 
    
      # Convert Sample column back to factor with sorted levels within each group  
     counts $ Sample  <-   factor (counts $ Sample,  levels =   unique (counts $ Sample)) 
    
      # Rename and reorder Comparison column  
     counts  <-  counts  |>   mutate ( 
        Comparison_new =   recode ( 
         Comparison, 
          "REF"   =   "Reference Allele" , 
          "ALT"   =   "Alternative Allele" , 
          "Zygosity"   =   "Zygosity"  
       ) 
     )  |>   mutate ( 
        Comparison_new =   factor ( 
         Comparison_new, 
          levels =   c ( "Reference Allele" ,  "Alternative Allele" ,  "Zygosity" ) 
       ) 
     ) 
    
      return (counts) 
   }    
 Pairwise plotting function 
      plot_counts  <-   function (counts,  output_file =   NULL ) { 
      library (ggplot2) 
    
      # Define color palette  
     color_palette  <-   c ( "#92C6FF" ,  "#f5cb8b" ,  "#bff28c" ) 
    
      # Create plot  
     plot  <-   ggplot (counts, 
                     aes ( x =  Sample,  y =  Mismatch,  fill =  Comparison))  +  
        geom_bar ( stat =   "identity" ,  position =   "dodge" )  +  
        facet_grid (Population  ~  Comparison_new, 
                   scales =   "free_y" , 
                   space =   "free" )  +  
        coord_flip ()  +  
        labs ( 
          title =   "SNP Mismatch Counts per Sample" , 
          x =   "Sample" , 
          y =   "Mismatches" , 
          caption =   "Genotyping errors per sample within each population."  
       )  +  
        my_theme ()  +  
        theme ( panel.spacing =   unit ( 0.5 ,  "lines" ))  +  
        geom_text ( aes ( label =   paste0 ( 
         scales ::  comma (Mismatch),  " (" , Percent_Mismatch,  "%)"  
       )), 
        hjust =   1 , 
        size =   2.5 )  +  
        scale_fill_manual ( values =  color_palette)  +  
        theme ( axis.text.x =   element_text ( angle =   0 ,  hjust =   1 ,  size =   7 ))  +  
        guides ( fill =   "none" )  +  
        theme ( plot.caption =   element_text ( 
          face =   "italic" , 
          size =   10 , 
          color =   "grey20"  
       ))  +  
        scale_y_continuous ( labels =  scales :: comma)   # Add thousands separator to y-axis labels  
    
      # Save the plot if output_file is provided  
      if  ( !  is.null (output_file)) { 
        ggsave (output_file, plot,  width =   8 ,  height =   7 ,  units =   "in" ) 
     } 
    
      # Return the plot  
      return (plot) 
   }    
 How to run the calculate_counts and plot_counts functions 
       # Call the function with data_*_dt as input  
   counts_ay  <-   calculate_counts (data_ay_dt) 
    plot_counts (counts_ay,  here ( "output" ,  "wgs_vs_chip" ,  "figures" ,  "ay_SAI_KAT_per_sample_stats.pdf" ))    
 The comparisons we will make: 
 Chip: “ab” - Genotyping calls using 18 versus 95 samples “ac” -
Genotyping calls using 18 versus 500 samples “bc” - Genotyping calls
using 95 versus 500 samples 
 WGS: “xy” Genotyping calls with 18 versus 30 samples “wy” Genotyping
calls with 18 versus 800 samples “wx” Genotyping calls with 30 versus
800 samples 
 Chip x WGS: “ay” - WGS and chip calls with 18 samples “bx” - WGS call
with 30 samples and chip call with 95 samples “cw” - WGS call with 800
samples and chip call with 500 samples 
 
 
 
 10. Chip comparisons 
 
 10.1 “ab” - Genotype calls using 18 versus 95 samples 
 Generate csv files 
       python  output/wgs_vs_chip/scripts/create_csv_from_vcfs.py ab    
 Import csv 
      data_ab_dt  <-   process_csv_files ( "ab" ) 
    
    # Check and display only columns that match the criteria  
    head (data_ab_dt[,  c ( "SNP_id" ,  names (data_ab_dt)[ grepl ( "_REF$|_ALT$" ,  names (data_ab_dt))]),  with =   FALSE ])    
  ##          SNP_id KAT_9a_KAT_9b_REF KAT_9a_KAT_9b_ALT SAI_15a_SAI_15b_REF
## 1: AX-581444870             match             match               match
## 2: AX-583035067             match             match               match
## 3: AX-583033342             match             match               match
## 4: AX-583035163             match             match               match
## 5: AX-583035194             match             match               match
## 6: AX-583033387             match             match               match
##    SAI_15a_SAI_15b_ALT SAI_3a_SAI_3b_REF SAI_3a_SAI_3b_ALT KAT_12a_KAT_12b_REF
## 1:               match             match             match               match
## 2:               match             match             match               match
## 3:               match             match             match               match
## 4:               match             match             match               match
## 5:               match             match             match               match
## 6:               match             match             match               match
##    KAT_12a_KAT_12b_ALT KAT_7a_KAT_7b_REF KAT_7a_KAT_7b_ALT SAI_2a_SAI_2b_REF
## 1:               match             match             match              <NA>
## 2:               match             match             match             match
## 3:               match             match             match             match
## 4:               match             match             match             match
## 5:               match             match             match             match
## 6:               match             match             match             match
##    SAI_2a_SAI_2b_ALT SAI_14a_SAI_14b_REF SAI_14a_SAI_14b_ALT KAT_8a_KAT_8b_REF
## 1:              <NA>               match               match             match
## 2:             match               match               match             match
## 3:             match               match               match             match
## 4:             match               match               match             match
## 5:             match               match               match             match
## 6:             match               match               match             match
##    KAT_8a_KAT_8b_ALT SAI_13a_SAI_13b_REF SAI_13a_SAI_13b_ALT SAI_5a_SAI_5b_REF
## 1:             match               match               match             match
## 2:             match               match               match             match
## 3:             match               match               match             match
## 4:             match               match               match             match
## 5:             match               match               match             match
## 6:             match               match               match             match
##    SAI_5a_SAI_5b_ALT SAI_18a_SAI_18b_REF SAI_18a_SAI_18b_ALT
## 1:             match               match               match
## 2:             match               match               match
## 3:             match               match               match
## 4:             match               match               match
## 5:             match               match               match
## 6:             match               match               match
##    KAT_10a_KAT_10b_REF KAT_10a_KAT_10b_ALT SAI_1a_SAI_1b_REF SAI_1a_SAI_1b_ALT
## 1:               match               match              <NA>              <NA>
## 2:               match               match             match             match
## 3:               match               match             match             match
## 4:               match               match             match             match
## 5:               match               match             match             match
## 6:               match               match             match             match
##    SAI_17a_SAI_17b_REF SAI_17a_SAI_17b_ALT SAI_4a_SAI_4b_REF SAI_4a_SAI_4b_ALT
## 1:               match               match             match             match
## 2:               match               match             match             match
## 3:               match               match             match             match
## 4:               match               match             match             match
## 5:               match               match             match             match
## 6:               match               match             match             match
##    SAI_12a_SAI_12b_REF SAI_12a_SAI_12b_ALT KAT_11a_KAT_11b_REF
## 1:               match               match               match
## 2:               match               match               match
## 3:               match               match               match
## 4:               match               match               match
## 5:               match               match               match
## 6:               match               match               match
##    KAT_11a_KAT_11b_ALT SAI_16a_SAI_16b_REF SAI_16a_SAI_16b_ALT
## 1:               match               match               match
## 2:               match               match               match
## 3:               match               match               match
## 4:               match               match               match
## 5:               match               match               match
## 6:               match               match               match  
 Get the summary 
      summary_ab  <-   process_data_object ( "data_ab_dt" ) 
    head (summary_ab)    
  ##          SNP_id REF_match REF_mismatch ALT_match ALT_mismatch Zigo_match
## 1: AX-579436125        18            0        18            0         18
## 2: AX-579436196        16            0        16            0         16
## 3: AX-579436243        15            3        18            0         15
## 4: AX-579436298        17            0        17            0         17
## 5: AX-579436308        16            0        16            0         16
## 6: AX-579436317        18            0        18            0         18
##    Zigo_mismatch
## 1:             0
## 2:             0
## 3:             3
## 4:             0
## 5:             0
## 6:             0  
 Check NAs, match and mismatch counts 
       table (data_ab_dt $ KAT_11a_KAT_11b_zcomp,  useNA =   "ifany" )    
  ## 
##             match mismatch 
##     2490    87163      889  
 Make data long format for plotting 
      dt_long_ab  <-   process_summary_object ( "summary_ab" ) 
    head (dt_long_ab)    
  ##                type count     n       perc
## 1: Reference Allele     0 84155 92.9458152
## 2: Reference Allele     3   622  0.6869740
## 3: Reference Allele     4   341  0.3766208
## 4: Reference Allele     2  1324  1.4623048
## 5: Reference Allele     1  3730  4.1196351
## 6: Reference Allele     5   174  0.1921760  
 Create plot of SNP error per sample 
       plot_dt_long ( "ab" )    
   
 Compare both populations 
      summary_sai_ab  <-   generate_summary (data_ab_dt,  "SAI" ,  "suffix" ) 
   summary_kat_ab  <-   generate_summary (data_ab_dt,  "KAT" ,  "suffix" ) 
   dt_long_2_ab  <-   merge_and_transform ( "ab" ) 
    create_plot2 ( "ab" ,  here ( "output" ,  "wgs_vs_chip" ,  "figures" ,  "ab_mismatches_SAI_KAT.pdf" ), dt_long_2_ab)    
   
 Counts plot 
       # Call the function with data_*_dt as input  
   counts_ab  <-   calculate_counts (data_ab_dt) 
    plot_counts (counts_ab,  here ( "output" ,  "wgs_vs_chip" ,  "figures" ,  "ab_SAI_KAT_per_sample_stats.pdf" ))    
   
 
 
 10.2 “ac” - Genotype calls using 18 versus 500 samples 
 Generate csv files 
       python  output/wgs_vs_chip/scripts/create_csv_from_vcfs.py ac    
 Import csv 
      data_ac_dt  <-   process_csv_files ( "ac" ) 
    
    # Check and display only columns that match the criteria  
    head (data_ac_dt[,  c ( "SNP_id" ,  names (data_ac_dt)[ grepl ( "_REF$|_ALT$" ,  names (data_ac_dt))]),  with =   FALSE ])    
  ##          SNP_id KAT_12a_KAT_12c_REF KAT_12a_KAT_12c_ALT SAI_3a_SAI_3c_REF
## 1: AX-583035067               match               match             match
## 2: AX-583033342               match               match             match
## 3: AX-583035194               match               match             match
## 4: AX-583033387               match               match             match
## 5: AX-583035211               match               match             match
## 6: AX-583035257               match               match             match
##    SAI_3a_SAI_3c_ALT KAT_9a_KAT_9c_REF KAT_9a_KAT_9c_ALT SAI_15a_SAI_15c_REF
## 1:             match             match             match               match
## 2:             match             match             match               match
## 3:             match             match             match               match
## 4:             match             match             match               match
## 5:             match             match             match               match
## 6:             match             match             match               match
##    SAI_15a_SAI_15c_ALT SAI_14a_SAI_14c_REF SAI_14a_SAI_14c_ALT
## 1:               match               match               match
## 2:               match               match               match
## 3:               match               match               match
## 4:               match               match               match
## 5:               match               match               match
## 6:               match               match               match
##    KAT_8a_KAT_8c_REF KAT_8a_KAT_8c_ALT SAI_2a_SAI_2c_REF SAI_2a_SAI_2c_ALT
## 1:             match             match             match             match
## 2:             match             match             match             match
## 3:             match             match             match             match
## 4:             match             match             match             match
## 5:             match             match             match             match
## 6:             match             match             match             match
##    KAT_7a_KAT_7c_REF KAT_7a_KAT_7c_ALT SAI_17a_SAI_17c_REF SAI_17a_SAI_17c_ALT
## 1:             match             match               match               match
## 2:             match             match               match               match
## 3:             match             match               match               match
## 4:             match             match               match               match
## 5:             match             match               match               match
## 6:             match             match               match               match
##    SAI_1a_SAI_1c_REF SAI_1a_SAI_1c_ALT KAT_10a_KAT_10c_REF KAT_10a_KAT_10c_ALT
## 1:             match             match               match               match
## 2:             match             match               match               match
## 3:             match             match               match               match
## 4:             match             match               match               match
## 5:             match             match               match               match
## 6:             match             match               match               match
##    SAI_18a_SAI_18c_REF SAI_18a_SAI_18c_ALT SAI_5a_SAI_5c_REF SAI_5a_SAI_5c_ALT
## 1:               match               match             match             match
## 2:               match               match             match             match
## 3:               match               match             match             match
## 4:               match               match             match             match
## 5:               match               match             match             match
## 6:               match               match             match             match
##    SAI_13a_SAI_13c_REF SAI_13a_SAI_13c_ALT SAI_16a_SAI_16c_REF
## 1:               match               match               match
## 2:               match               match               match
## 3:               match               match               match
## 4:               match               match               match
## 5:               match               match               match
## 6:               match               match               match
##    SAI_16a_SAI_16c_ALT KAT_11a_KAT_11c_REF KAT_11a_KAT_11c_ALT
## 1:               match               match               match
## 2:               match               match               match
## 3:               match               match               match
## 4:               match               match               match
## 5:               match               match               match
## 6:               match               match               match
##    SAI_12a_SAI_12c_REF SAI_12a_SAI_12c_ALT SAI_4a_SAI_4c_REF SAI_4a_SAI_4c_ALT
## 1:               match               match             match             match
## 2:               match               match             match             match
## 3:               match               match             match             match
## 4:               match               match             match             match
## 5:               match               match             match             match
## 6:               match               match             match             match  
 Get the summary 
      summary_ac  <-   process_data_object ( "data_ac_dt" ) 
    head (summary_ac)    
  ##          SNP_id REF_match REF_mismatch ALT_match ALT_mismatch Zigo_match
## 1: AX-579436089        13            1        14            0         13
## 2: AX-579436149        18            0        18            0         18
## 3: AX-579436196        16            0        16            0         16
## 4: AX-579436243        15            3        18            0         15
## 5: AX-579436298        17            0        17            0         17
## 6: AX-579436308        16            0        16            0         16
##    Zigo_mismatch
## 1:             1
## 2:             0
## 3:             0
## 4:             3
## 5:             0
## 6:             0  
 Make data long format for plotting 
      dt_long_ac  <-   process_summary_object ( "summary_ac" ) 
    head (dt_long_ac)    
  ##                type count     n       perc
## 1: Reference Allele     1  4327  4.7943536
## 2: Reference Allele     0 82923 91.8794043
## 3: Reference Allele     3   696  0.7711740
## 4: Reference Allele     2  1508  1.6708771
## 5: Reference Allele     4   371  0.4110712
## 6: Reference Allele     6   126  0.1396091  
 Create plot of SNP error per sample 
       plot_dt_long ( "ac" )    
   
 Compare both populations 
      summary_sai_ac  <-   generate_summary (data_ac_dt,  "SAI" ,  "suffix" ) 
   summary_kat_ac  <-   generate_summary (data_ac_dt,  "KAT" ,  "suffix" ) 
   dt_long_2_ac  <-   merge_and_transform ( "ac" ) 
    create_plot2 ( "ac" ,  here ( "output" ,  "wgs_vs_chip" ,  "figures" ,  "ac_mismatches_SAI_KAT.pdf" ), dt_long_2_ac)    
   
 Counts plot 
       # Call the function with data_*_dt as input  
   counts_ac  <-   calculate_counts (data_ac_dt) 
    plot_counts (counts_ac,  here ( "output" ,  "wgs_vs_chip" ,  "figures" ,  "ac_SAI_KAT_per_sample_stats.pdf" ))    
   
 
 
 10.3 “bc” - Genotype calls using 95 versus 500 samples 
 Generate csv files 
       python  output/wgs_vs_chip/scripts/create_csv_from_vcfs.py bc    
 Import csv 
      data_bc_dt  <-   process_csv_files ( "bc" ) 
    
    # Check and display only columns that match the criteria  
    head (data_bc_dt[,  c ( "SNP_id" ,  names (data_bc_dt)[ grepl ( "_REF$|_ALT$" ,  names (data_bc_dt))]),  with =   FALSE ])    
  ##          SNP_id SAI_3b_SAI_3c_REF SAI_3b_SAI_3c_ALT SAI_2b_SAI_2c_REF
## 1: AX-583035067             match             match             match
## 2: AX-583033342             match             match             match
## 3: AX-583033370             match             match             match
## 4: AX-583035194             match             match             match
## 5: AX-583033387             match             match             match
## 6: AX-583035211             match             match             match
##    SAI_2b_SAI_2c_ALT SAI_18b_SAI_18c_REF SAI_18b_SAI_18c_ALT SAI_1b_SAI_1c_REF
## 1:             match               match               match             match
## 2:             match               match               match             match
## 3:             match               match               match             match
## 4:             match               match               match             match
## 5:             match               match               match             match
## 6:             match               match               match             match
##    SAI_1b_SAI_1c_ALT SAI_4b_SAI_4c_REF SAI_4b_SAI_4c_ALT SAI_5b_SAI_5c_REF
## 1:             match             match             match             match
## 2:             match             match             match             match
## 3:             match             match             match             match
## 4:             match             match             match             match
## 5:             match             match             match             match
## 6:             match             match             match             match
##    SAI_5b_SAI_5c_ALT SAI_12b_SAI_12c_REF SAI_12b_SAI_12c_ALT
## 1:             match               match               match
## 2:             match               match               match
## 3:             match               match               match
## 4:             match               match               match
## 5:             match               match               match
## 6:             match               match               match
##    SAI_13b_SAI_13c_REF SAI_13b_SAI_13c_ALT SAI_17b_SAI_17c_REF
## 1:               match               match               match
## 2:               match               match               match
## 3:               match               match               match
## 4:               match               match               match
## 5:               match               match               match
## 6:               match               match               match
##    SAI_17b_SAI_17c_ALT SAI_16b_SAI_16c_REF SAI_16b_SAI_16c_ALT
## 1:               match               match               match
## 2:               match               match               match
## 3:               match               match               match
## 4:               match               match               match
## 5:               match               match               match
## 6:               match               match               match
##    SAI_15b_SAI_15c_REF SAI_15b_SAI_15c_ALT SAI_14b_SAI_14c_REF
## 1:               match               match               match
## 2:               match               match               match
## 3:               match               match               match
## 4:               match               match               match
## 5:               match               match               match
## 6:               match               match               match
##    SAI_14b_SAI_14c_ALT KAT_7b_KAT_7c_REF KAT_7b_KAT_7c_ALT KAT_11b_KAT_11c_REF
## 1:               match             match             match               match
## 2:               match             match             match               match
## 3:               match             match             match               match
## 4:               match             match             match               match
## 5:               match             match             match               match
## 6:               match             match             match               match
##    KAT_11b_KAT_11c_ALT KAT_10b_KAT_10c_REF KAT_10b_KAT_10c_ALT
## 1:               match               match               match
## 2:               match               match               match
## 3:               match               match               match
## 4:               match               match               match
## 5:               match               match               match
## 6:               match               match               match
##    KAT_8b_KAT_8c_REF KAT_8b_KAT_8c_ALT KAT_9b_KAT_9c_REF KAT_9b_KAT_9c_ALT
## 1:             match             match             match             match
## 2:             match             match             match             match
## 3:             match             match             match             match
## 4:             match             match             match             match
## 5:             match             match             match             match
## 6:             match             match             match             match
##    KAT_12b_KAT_12c_REF KAT_12b_KAT_12c_ALT
## 1:               match               match
## 2:               match               match
## 3:               match               match
## 4:               match               match
## 5:               match               match
## 6:               match               match  
 Get the summary 
      summary_bc  <-   process_data_object ( "data_bc_dt" ) 
    head (summary_bc)    
  ##          SNP_id REF_match REF_mismatch ALT_match ALT_mismatch Zigo_match
## 1: AX-579436196        16            0        16            0         16
## 2: AX-579436243        18            0        18            0         18
## 3: AX-579436298        17            0        17            0         17
## 4: AX-579436308        18            0        18            0         18
## 5: AX-579436317        18            0        18            0         18
## 6: AX-579436348        18            0        18            0         18
##    Zigo_mismatch
## 1:             0
## 2:             0
## 3:             0
## 4:             0
## 5:             0
## 6:             0  
 Make data long format for plotting 
      dt_long_bc  <-   process_summary_object ( "summary_bc" ) 
    head (dt_long_bc)    
  ##                type count     n        perc
## 1: Reference Allele     0 93283 97.19510289
## 2: Reference Allele     1  1741  1.81401407
## 3: Reference Allele     4    88  0.09169054
## 4: Reference Allele     2   498  0.51888513
## 5: Reference Allele     3   227  0.23651993
## 6: Reference Allele     6    41  0.04271946  
 Create plot of SNP error per sample 
       plot_dt_long ( "bc" )    
   
 Compare both populations 
      summary_sai_bc  <-   generate_summary (data_bc_dt,  "SAI" ,  "suffix" ) 
   summary_kat_bc  <-   generate_summary (data_bc_dt,  "KAT" ,  "suffix" ) 
   dt_long_2_bc  <-   merge_and_transform ( "bc" ) 
    create_plot2 ( "bc" ,  here ( "output" ,  "wgs_vs_chip" ,  "figures" ,  "bc_mismatches_SAI_KAT.pdf" ), dt_long_2_bc)    
   
 Counts plot 
       # Call the function with data_*_dt as input  
   counts_bc  <-   calculate_counts (data_bc_dt) 
    plot_counts (counts_bc,  here ( "output" ,  "wgs_vs_chip" ,  "figures" ,  "bc_SAI_KAT_per_sample_stats.pdf" ))    
   
 
 
 
 11. WGS comparsions 
 
 11.1 “xy” Genotyping calls with 18 versus 30 samples 
 Generate csv files 
       python  output/wgs_vs_chip/scripts/create_csv_from_vcfs.py xy    
 Import csv 
      data_xy_dt  <-   process_csv_files ( "xy" ) 
    
    # Check and display only columns that match the criteria  
    head (data_xy_dt[,  c ( "SNP_id" ,  names (data_xy_dt)[ grepl ( "_REF$|_ALT$" ,  names (data_xy_dt))]),  with =   FALSE ])    
  ##          SNP_id KAT_7x_KAT_7y_REF KAT_7x_KAT_7y_ALT SAI_2x_SAI_2y_REF
## 1: AX-583035067             match             match             match
## 2: AX-583035102             match             match             match
## 3: AX-583033340             match             match             match
## 4: AX-583033342             match             match             match
## 5: AX-583035163             match             match             match
## 6: AX-583033356             match             match             match
##    SAI_2x_SAI_2y_ALT KAT_8x_KAT_8y_REF KAT_8x_KAT_8y_ALT SAI_14x_SAI_14y_REF
## 1:             match             match             match               match
## 2:             match             match             match               match
## 3:             match             match             match               match
## 4:             match             match             match               match
## 5:             match             match             match               match
## 6:             match             match             match               match
##    SAI_14x_SAI_14y_ALT KAT_12x_KAT_12y_REF KAT_12x_KAT_12y_ALT
## 1:               match               match               match
## 2:               match               match               match
## 3:               match               match               match
## 4:               match               match               match
## 5:               match               match               match
## 6:               match               match               match
##    SAI_15x_SAI_15y_REF SAI_15x_SAI_15y_ALT KAT_9x_KAT_9y_REF KAT_9x_KAT_9y_ALT
## 1:               match               match             match             match
## 2:               match               match             match             match
## 3:               match               match             match             match
## 4:               match               match             match             match
## 5:               match               match             match             match
## 6:               match               match             match             match
##    SAI_3x_SAI_3y_REF SAI_3x_SAI_3y_ALT SAI_4x_SAI_4y_REF SAI_4x_SAI_4y_ALT
## 1:             match             match             match             match
## 2:             match             match             match             match
## 3:             match             match             match             match
## 4:             match             match             match             match
## 5:             match             match             match             match
## 6:             match             match             match             match
##    SAI_12x_SAI_12y_REF SAI_12x_SAI_12y_ALT SAI_16x_SAI_16y_REF
## 1:               match               match               match
## 2:               match               match               match
## 3:               match               match               match
## 4:               match               match               match
## 5:               match               match               match
## 6:               match               match               match
##    SAI_16x_SAI_16y_ALT KAT_11x_KAT_11y_REF KAT_11x_KAT_11y_ALT
## 1:               match               match               match
## 2:               match               match               match
## 3:               match               match               match
## 4:               match               match               match
## 5:               match               match               match
## 6:               match               match               match
##    SAI_18x_SAI_18y_REF SAI_18x_SAI_18y_ALT SAI_13x_SAI_13y_REF
## 1:               match               match               match
## 2:               match               match               match
## 3:               match               match               match
## 4:               match               match               match
## 5:               match               match               match
## 6:               match               match               match
##    SAI_13x_SAI_13y_ALT SAI_5x_SAI_5y_REF SAI_5x_SAI_5y_ALT SAI_1x_SAI_1y_REF
## 1:               match             match             match             match
## 2:               match             match             match             match
## 3:               match             match             match             match
## 4:               match             match             match             match
## 5:               match             match             match             match
## 6:               match             match             match             match
##    SAI_1x_SAI_1y_ALT SAI_17x_SAI_17y_REF SAI_17x_SAI_17y_ALT
## 1:             match               match               match
## 2:             match               match               match
## 3:             match               match               match
## 4:             match               match               match
## 5:             match               match               match
## 6:             match               match               match
##    KAT_10x_KAT_10y_REF KAT_10x_KAT_10y_ALT
## 1:               match               match
## 2:               match               match
## 3:               match               match
## 4:               match               match
## 5:               match               match
## 6:               match               match  
 Get the summary 
      summary_xy  <-   process_data_object ( "data_xy_dt" ) 
    head (summary_xy)    
  ##          SNP_id REF_match REF_mismatch ALT_match ALT_mismatch Zigo_match
## 1: AX-579436016        18            0        18            0         18
## 2: AX-579436089        18            0        18            0         18
## 3: AX-579436102        18            0        18            0         18
## 4: AX-579436125        18            0        18            0         18
## 5: AX-579436196        18            0        18            0         18
## 6: AX-579436214        18            0        18            0         18
##    Zigo_mismatch
## 1:             0
## 2:             0
## 3:             0
## 4:             0
## 5:             0
## 6:             0  
 Make data long format for plotting 
      dt_long_xy  <-   process_summary_object ( "summary_xy" ) 
    head (dt_long_xy)    
  ##                type count      n        perc
## 1: Reference Allele     0 159905 98.49460114
## 2: Reference Allele     1    836  0.51494004
## 3: Reference Allele     3    256  0.15768499
## 4: Reference Allele     4    165  0.10163290
## 5: Reference Allele     2    285  0.17554774
## 6: Reference Allele    15     17  0.01047127  
 Create plot of SNP error per sample 
       plot_dt_long ( "xy" )    
   
 Compare both populations 
      summary_sai_xy  <-   generate_summary (data_xy_dt,  "SAI" ,  "suffix" ) 
   summary_kat_xy  <-   generate_summary (data_xy_dt,  "KAT" ,  "suffix" ) 
   dt_long_2_xy  <-   merge_and_transform ( "xy" ) 
    create_plot2 ( "xy" ,  here ( "output" ,  "wgs_vs_chip" ,  "figures" ,  "xy_mismatches_SAI_KAT.pdf" ), dt_long_2_xy)    
   
 Counts plot 
       # Call the function with data_*_dt as input  
   counts_xy  <-   calculate_counts (data_xy_dt) 
    plot_counts (counts_xy,  here ( "output" ,  "wgs_vs_chip" ,  "figures" ,  "xy_SAI_KAT_per_sample_stats.pdf" ))    
   
 
 
 11.2 “ey” Genotyping calls with 18 versus 800 samples 
 Generate csv files 
       python  output/wgs_vs_chip/scripts/create_csv_from_vcfs.py wy    
 Import csv 
      data_wy_dt  <-   process_csv_files ( "wy" ) 
    
    # Check and display only columns that match the criteria  
    head (data_wy_dt[,  c ( "SNP_id" ,  names (data_wy_dt)[ grepl ( "_REF$|_ALT$" ,  names (data_wy_dt))]),  with =   FALSE ])    
  ##          SNP_id KAT_7w_KAT_7y_REF KAT_7w_KAT_7y_ALT KAT_8w_KAT_8y_REF
## 1: AX-583035067             match             match             match
## 2: AX-583035102             match             match             match
## 3: AX-583033340             match             match             match
## 4: AX-583033342             match             match             match
## 5: AX-583035163             match             match             match
## 6: AX-583033356             match             match             match
##    KAT_8w_KAT_8y_ALT SAI_14w_SAI_14y_REF SAI_14w_SAI_14y_ALT SAI_2w_SAI_2y_REF
## 1:             match               match               match             match
## 2:             match               match               match             match
## 3:             match               match               match             match
## 4:             match               match               match             match
## 5:             match               match               match             match
## 6:             match               match               match             match
##    SAI_2w_SAI_2y_ALT SAI_3w_SAI_3y_REF SAI_3w_SAI_3y_ALT SAI_15w_SAI_15y_REF
## 1:             match             match             match               match
## 2:             match             match             match               match
## 3:             match             match             match               match
## 4:             match             match             match               match
## 5:             match             match             match               match
## 6:             match             match             match               match
##    SAI_15w_SAI_15y_ALT KAT_9w_KAT_9y_REF KAT_9w_KAT_9y_ALT KAT_12w_KAT_12y_REF
## 1:               match             match             match               match
## 2:               match             match             match               match
## 3:               match             match             match               match
## 4:               match             match             match               match
## 5:               match             match             match               match
## 6:               match             match             match               match
##    KAT_12w_KAT_12y_ALT SAI_12w_SAI_12y_REF SAI_12w_SAI_12y_ALT
## 1:               match               match               match
## 2:               match               match               match
## 3:               match               match               match
## 4:               match               match               match
## 5:               match               match               match
## 6:               match               match               match
##    SAI_4w_SAI_4y_REF SAI_4w_SAI_4y_ALT KAT_11w_KAT_11y_REF KAT_11w_KAT_11y_ALT
## 1:             match             match               match               match
## 2:             match             match               match               match
## 3:             match             match               match               match
## 4:             match             match               match               match
## 5:             match             match               match               match
## 6:             match             match               match               match
##    SAI_16w_SAI_16y_REF SAI_16w_SAI_16y_ALT SAI_5w_SAI_5y_REF SAI_5w_SAI_5y_ALT
## 1:               match               match             match             match
## 2:               match               match             match             match
## 3:               match               match             match             match
## 4:               match               match             match             match
## 5:               match               match             match             match
## 6:               match               match             match             match
##    SAI_13w_SAI_13y_REF SAI_13w_SAI_13y_ALT SAI_18w_SAI_18y_REF
## 1:               match               match               match
## 2:               match               match               match
## 3:               match               match               match
## 4:               match               match               match
## 5:               match               match               match
## 6:               match               match               match
##    SAI_18w_SAI_18y_ALT KAT_10w_KAT_10y_REF KAT_10w_KAT_10y_ALT
## 1:               match               match               match
## 2:               match               match               match
## 3:               match               match               match
## 4:               match               match               match
## 5:               match               match               match
## 6:               match               match               match
##    SAI_17w_SAI_17y_REF SAI_17w_SAI_17y_ALT SAI_1w_SAI_1y_REF SAI_1w_SAI_1y_ALT
## 1:               match               match             match             match
## 2:               match               match             match             match
## 3:               match               match             match             match
## 4:               match               match             match             match
## 5:               match               match             match             match
## 6:               match               match             match             match  
 Get the summary 
      summary_wy  <-   process_data_object ( "data_wy_dt" ) 
    head (summary_wy)    
  ##          SNP_id REF_match REF_mismatch ALT_match ALT_mismatch Zigo_match
## 1: AX-579436016        18            0        18            0         18
## 2: AX-579436089        18            0        18            0         18
## 3: AX-579436102        18            0        18            0         18
## 4: AX-579436125        18            0        18            0         18
## 5: AX-579436196        18            0        18            0         18
## 6: AX-579436214        18            0        18            0         18
##    Zigo_mismatch
## 1:             0
## 2:             0
## 3:             0
## 4:             0
## 5:             0
## 6:             0  
 Make data long format for plotting 
      dt_long_wy  <-   process_summary_object ( "summary_wy" ) 
    head (dt_long_wy)    
  ##                type count      n         perc
## 1: Reference Allele     0 156689 96.440617460
## 2: Reference Allele     3    564  0.347136737
## 3: Reference Allele     1   2128  1.309764144
## 4: Reference Allele     2    814  0.501009405
## 5: Reference Allele     5    318  0.195726033
## 6: Reference Allele    16     16  0.009847851  
 Create plot of SNP error per sample 
       plot_dt_long ( "wy" )    
   
 Compare both populations 
      summary_sai_wy  <-   generate_summary (data_wy_dt,  "SAI" ,  "suffix" ) 
   summary_kat_wy  <-   generate_summary (data_wy_dt,  "KAT" ,  "suffix" ) 
   dt_long_2_wy  <-   merge_and_transform ( "wy" ) 
    create_plot2 ( "wy" ,  here ( "output" ,  "wgs_vs_chip" ,  "figures" ,  "wy_mismatches_SAI_KAT.pdf" ), dt_long_2_wy)    
   
 Counts plot 
       # Call the function with data_*_dt as input  
   counts_wy  <-   calculate_counts (data_wy_dt) 
    plot_counts (counts_wy,  here ( "output" ,  "wgs_vs_chip" ,  "figures" ,  "wy_SAI_KAT_per_sample_stats.pdf" ))    
   
 
 
 11.3 “wx” Genotyping calls with 30 versus 800 samples 
 Generate csv files 
       python  output/wgs_vs_chip/scripts/create_csv_from_vcfs.py wx    
 Import csv 
      data_wx_dt  <-   process_csv_files ( "wx" ) 
    
    # Check and display only columns that match the criteria  
    head (data_wy_dt[,  c ( "SNP_id" ,  names (data_wy_dt)[ grepl ( "_REF$|_ALT$" ,  names (data_wy_dt))]),  with =   FALSE ])    
  ##          SNP_id KAT_7w_KAT_7y_REF KAT_7w_KAT_7y_ALT KAT_8w_KAT_8y_REF
## 1: AX-583035067             match             match             match
## 2: AX-583035102             match             match             match
## 3: AX-583033340             match             match             match
## 4: AX-583033342             match             match             match
## 5: AX-583035163             match             match             match
## 6: AX-583033356             match             match             match
##    KAT_8w_KAT_8y_ALT SAI_14w_SAI_14y_REF SAI_14w_SAI_14y_ALT SAI_2w_SAI_2y_REF
## 1:             match               match               match             match
## 2:             match               match               match             match
## 3:             match               match               match             match
## 4:             match               match               match             match
## 5:             match               match               match             match
## 6:             match               match               match             match
##    SAI_2w_SAI_2y_ALT SAI_3w_SAI_3y_REF SAI_3w_SAI_3y_ALT SAI_15w_SAI_15y_REF
## 1:             match             match             match               match
## 2:             match             match             match               match
## 3:             match             match             match               match
## 4:             match             match             match               match
## 5:             match             match             match               match
## 6:             match             match             match               match
##    SAI_15w_SAI_15y_ALT KAT_9w_KAT_9y_REF KAT_9w_KAT_9y_ALT KAT_12w_KAT_12y_REF
## 1:               match             match             match               match
## 2:               match             match             match               match
## 3:               match             match             match               match
## 4:               match             match             match               match
## 5:               match             match             match               match
## 6:               match             match             match               match
##    KAT_12w_KAT_12y_ALT SAI_12w_SAI_12y_REF SAI_12w_SAI_12y_ALT
## 1:               match               match               match
## 2:               match               match               match
## 3:               match               match               match
## 4:               match               match               match
## 5:               match               match               match
## 6:               match               match               match
##    SAI_4w_SAI_4y_REF SAI_4w_SAI_4y_ALT KAT_11w_KAT_11y_REF KAT_11w_KAT_11y_ALT
## 1:             match             match               match               match
## 2:             match             match               match               match
## 3:             match             match               match               match
## 4:             match             match               match               match
## 5:             match             match               match               match
## 6:             match             match               match               match
##    SAI_16w_SAI_16y_REF SAI_16w_SAI_16y_ALT SAI_5w_SAI_5y_REF SAI_5w_SAI_5y_ALT
## 1:               match               match             match             match
## 2:               match               match             match             match
## 3:               match               match             match             match
## 4:               match               match             match             match
## 5:               match               match             match             match
## 6:               match               match             match             match
##    SAI_13w_SAI_13y_REF SAI_13w_SAI_13y_ALT SAI_18w_SAI_18y_REF
## 1:               match               match               match
## 2:               match               match               match
## 3:               match               match               match
## 4:               match               match               match
## 5:               match               match               match
## 6:               match               match               match
##    SAI_18w_SAI_18y_ALT KAT_10w_KAT_10y_REF KAT_10w_KAT_10y_ALT
## 1:               match               match               match
## 2:               match               match               match
## 3:               match               match               match
## 4:               match               match               match
## 5:               match               match               match
## 6:               match               match               match
##    SAI_17w_SAI_17y_REF SAI_17w_SAI_17y_ALT SAI_1w_SAI_1y_REF SAI_1w_SAI_1y_ALT
## 1:               match               match             match             match
## 2:               match               match             match             match
## 3:               match               match             match             match
## 4:               match               match             match             match
## 5:               match               match             match             match
## 6:               match               match             match             match  
 Get the summary 
      summary_wx  <-   process_data_object ( "data_wx_dt" ) 
    head (summary_wx)    
  ##          SNP_id REF_match REF_mismatch ALT_match ALT_mismatch Zigo_match
## 1: AX-579436016        18            0        18            0         18
## 2: AX-579436089        18            0        18            0         18
## 3: AX-579436102        18            0        18            0         18
## 4: AX-579436125        18            0        18            0         18
## 5: AX-579436196        18            0        18            0         18
## 6: AX-579436214        18            0        18            0         18
##    Zigo_mismatch
## 1:             0
## 2:             0
## 3:             0
## 4:             0
## 5:             0
## 6:             0  
 Make data long format for plotting 
      dt_long_wx  <-   process_summary_object ( "summary_wx" ) 
    head (dt_long_wx)    
  ##                type count      n        perc
## 1: Reference Allele     0 161444 96.64296147
## 2: Reference Allele     3    577  0.34540143
## 3: Reference Allele     2    824  0.49325958
## 4: Reference Allele     1   2117  1.26727007
## 5: Reference Allele     5    310  0.18557096
## 6: Reference Allele    16     24  0.01436678  
 Create plot of SNP error per sample 
       plot_dt_long ( "wx" )    
   
 Compare both populations 
      summary_sai_wx  <-   generate_summary (data_wx_dt,  "SAI" ,  "suffix" ) 
   summary_kat_wx  <-   generate_summary (data_wx_dt,  "KAT" ,  "suffix" ) 
   dt_long_2_wx  <-   merge_and_transform ( "wx" ) 
    create_plot2 ( "wx" ,  here ( "output" ,  "wgs_vs_chip" ,  "figures" ,  "wx_mismatches_SAI_KAT.pdf" ), dt_long_2_wx)    
   
 Counts plot 
       # Call the function with data_*_dt as input  
   counts_wx  <-   calculate_counts (data_wx_dt) 
    plot_counts (counts_wx,  here ( "output" ,  "wgs_vs_chip" ,  "figures" ,  "wx_SAI_KAT_per_sample_stats.pdf" ))    
   
 
 
 
 12. Chip and WGS comparisons 
 
 12.1 “ay” - WGS and chip calls with 18 samples 
 Generate csv files 
       python  output/wgs_vs_chip/scripts/create_csv_from_vcfs.py ay    
 Import csv 
      data_ay_dt  <-   process_csv_files ( "ay" ) 
    
    # Check and display only columns that match the criteria  
    head (data_ay_dt[,  c ( "SNP_id" ,  names (data_ay_dt)[ grepl ( "_REF$|_ALT$" ,  names (data_ay_dt))]),  with =   FALSE ])    
  ##          SNP_id KAT_11a_KAT_11y_REF KAT_11a_KAT_11y_ALT SAI_16a_SAI_16y_REF
## 1: AX-583035067               match            mismatch               match
## 2: AX-583035102               match               match            mismatch
## 3: AX-583033342               match               match               match
## 4: AX-583035163               match               match               match
## 5: AX-583035194               match               match               match
## 6: AX-583033387               match               match               match
##    SAI_16a_SAI_16y_ALT SAI_12a_SAI_12y_REF SAI_12a_SAI_12y_ALT
## 1:               match               match               match
## 2:               match            mismatch            mismatch
## 3:               match               match               match
## 4:               match               match               match
## 5:               match               match               match
## 6:               match               match               match
##    SAI_4a_SAI_4y_REF SAI_4a_SAI_4y_ALT KAT_10a_KAT_10y_REF KAT_10a_KAT_10y_ALT
## 1:             match             match               match               match
## 2:             match             match            mismatch               match
## 3:             match             match               match               match
## 4:             match             match               match               match
## 5:             match             match               match               match
## 6:             match             match               match               match
##    SAI_17a_SAI_17y_REF SAI_17a_SAI_17y_ALT SAI_1a_SAI_1y_REF SAI_1a_SAI_1y_ALT
## 1:               match               match             match             match
## 2:               match               match             match          mismatch
## 3:               match               match             match             match
## 4:               match               match             match             match
## 5:               match               match             match          mismatch
## 6:               match               match             match             match
##    SAI_5a_SAI_5y_REF SAI_5a_SAI_5y_ALT SAI_13a_SAI_13y_REF SAI_13a_SAI_13y_ALT
## 1:             match             match               match               match
## 2:             match          mismatch               match            mismatch
## 3:             match             match               match               match
## 4:             match             match            mismatch            mismatch
## 5:             match             match               match               match
## 6:             match             match               match               match
##    SAI_18a_SAI_18y_REF SAI_18a_SAI_18y_ALT KAT_8a_KAT_8y_REF KAT_8a_KAT_8y_ALT
## 1:               match               match             match             match
## 2:                <NA>                <NA>             match          mismatch
## 3:               match               match             match             match
## 4:               match               match             match             match
## 5:               match               match             match             match
## 6:               match               match             match             match
##    SAI_14a_SAI_14y_REF SAI_14a_SAI_14y_ALT SAI_2a_SAI_2y_REF SAI_2a_SAI_2y_ALT
## 1:               match               match             match             match
## 2:               match            mismatch             match          mismatch
## 3:               match               match             match             match
## 4:               match               match             match             match
## 5:               match               match             match             match
## 6:               match               match             match             match
##    KAT_7a_KAT_7y_REF KAT_7a_KAT_7y_ALT SAI_3a_SAI_3y_REF SAI_3a_SAI_3y_ALT
## 1:             match          mismatch             match             match
## 2:             match          mismatch          mismatch             match
## 3:             match             match             match             match
## 4:             match             match             match             match
## 5:             match             match             match             match
## 6:             match             match             match             match
##    SAI_15a_SAI_15y_REF SAI_15a_SAI_15y_ALT KAT_9a_KAT_9y_REF KAT_9a_KAT_9y_ALT
## 1:               match               match             match             match
## 2:               match            mismatch              <NA>              <NA>
## 3:               match               match             match             match
## 4:               match               match             match             match
## 5:               match               match             match             match
## 6:               match               match             match             match
##    KAT_12a_KAT_12y_REF KAT_12a_KAT_12y_ALT
## 1:               match               match
## 2:               match            mismatch
## 3:               match               match
## 4:               match               match
## 5:               match               match
## 6:               match               match  
 Get the summary 
      summary_ay  <-   process_data_object ( "data_ay_dt" ) 
    head (summary_ay)    
  ##          SNP_id REF_match REF_mismatch ALT_match ALT_mismatch Zigo_match
## 1: AX-579436089        15            0        15            0         15
## 2: AX-579436125        15            3        18            0         15
## 3: AX-579436196        14            2        16            0         14
## 4: AX-579436243        15            3        18            0         15
## 5: AX-579436298        16            1        12            5         13
## 6: AX-579436308        16            0        16            0         16
##    Zigo_mismatch
## 1:             0
## 2:             3
## 3:             2
## 4:             3
## 5:             4
## 6:             0  
 Make data long format for plotting 
      dt_long_ay  <-   process_summary_object ( "summary_ay" ) 
    head (dt_long_ay)    
  ##                type count     n       perc
## 1: Reference Allele     0 60897 61.5419597
## 2: Reference Allele     3  4609  4.6578139
## 3: Reference Allele     2  8737  8.8295335
## 4: Reference Allele     1 18417 18.6120543
## 5: Reference Allele     4  2586  2.6133883
## 6: Reference Allele     7   471  0.4759884  
 Create plot of SNP error per sample 
       plot_dt_long ( "ay" )    
   
 Compare both populations 
      summary_sai_ay  <-   generate_summary (data_ay_dt,  "SAI" ,  "suffix" ) 
   summary_kat_ay  <-   generate_summary (data_ay_dt,  "KAT" ,  "suffix" ) 
   dt_long_2_ay  <-   merge_and_transform ( "ay" ) 
    create_plot2 ( "ay" ,  here ( "output" ,  "wgs_vs_chip" ,  "figures" ,  "ay_mismatches_SAI_KAT.pdf" ), dt_long_2_ay)    
   
 Counts plot 
       # Call the function with data_*_dt as input  
   counts_ay  <-   calculate_counts (data_ay_dt) 
    plot_counts (counts_ay,  here ( "output" ,  "wgs_vs_chip" ,  "figures" ,  "ay_SAI_KAT_per_sample_stats.pdf" ))    
   
 
 
 12.2 “bx” - WGS call with 30 samples and chip call with 95
samples 
 Generate csv files 
       python  output/wgs_vs_chip/scripts/create_csv_from_vcfs.py bx    
 Import csv 
      data_bx_dt  <-   process_csv_files ( "bx" ) 
    
    # Check and display only columns that match the criteria  
    head (data_bx_dt[,  c ( "SNP_id" ,  names (data_bx_dt)[ grepl ( "_REF$|_ALT$" ,  names (data_bx_dt))]),  with =   FALSE ])    
  ##          SNP_id SAI_14b_SAI_14x_REF SAI_14b_SAI_14x_ALT KAT_8b_KAT_8x_REF
## 1: AX-583035067               match               match             match
## 2: AX-583035102               match            mismatch             match
## 3: AX-583033342               match               match             match
## 4: AX-583035163               match               match             match
## 5: AX-583033370               match               match             match
## 6: AX-583035194               match               match             match
##    KAT_8b_KAT_8x_ALT SAI_2b_SAI_2x_REF SAI_2b_SAI_2x_ALT KAT_7b_KAT_7x_REF
## 1:             match             match             match             match
## 2:          mismatch             match          mismatch             match
## 3:             match             match             match             match
## 4:             match             match             match             match
## 5:             match             match             match             match
## 6:             match             match             match             match
##    KAT_7b_KAT_7x_ALT KAT_12b_KAT_12x_REF KAT_12b_KAT_12x_ALT SAI_3b_SAI_3x_REF
## 1:          mismatch               match               match             match
## 2:          mismatch               match            mismatch          mismatch
## 3:             match               match               match             match
## 4:             match               match               match             match
## 5:             match               match               match          mismatch
## 6:             match               match               match             match
##    SAI_3b_SAI_3x_ALT KAT_9b_KAT_9x_REF KAT_9b_KAT_9x_ALT SAI_15b_SAI_15x_REF
## 1:             match             match             match               match
## 2:             match              <NA>              <NA>               match
## 3:             match             match             match               match
## 4:             match             match             match               match
## 5:             match             match             match               match
## 6:             match             match             match               match
##    SAI_15b_SAI_15x_ALT SAI_16b_SAI_16x_REF SAI_16b_SAI_16x_ALT
## 1:               match               match               match
## 2:            mismatch            mismatch               match
## 3:               match               match               match
## 4:               match               match               match
## 5:               match               match               match
## 6:               match               match               match
##    KAT_11b_KAT_11x_REF KAT_11b_KAT_11x_ALT SAI_12b_SAI_12x_REF
## 1:               match            mismatch               match
## 2:               match               match            mismatch
## 3:               match               match               match
## 4:               match               match               match
## 5:               match               match               match
## 6:               match               match               match
##    SAI_12b_SAI_12x_ALT SAI_4b_SAI_4x_REF SAI_4b_SAI_4x_ALT SAI_17b_SAI_17x_REF
## 1:               match             match             match               match
## 2:            mismatch             match             match               match
## 3:               match             match             match               match
## 4:               match             match             match               match
## 5:               match             match             match               match
## 6:               match             match             match               match
##    SAI_17b_SAI_17x_ALT SAI_1b_SAI_1x_REF SAI_1b_SAI_1x_ALT KAT_10b_KAT_10x_REF
## 1:               match             match             match               match
## 2:               match             match          mismatch            mismatch
## 3:               match             match             match               match
## 4:               match             match             match               match
## 5:               match             match             match               match
## 6:               match             match          mismatch               match
##    KAT_10b_KAT_10x_ALT SAI_18b_SAI_18x_REF SAI_18b_SAI_18x_ALT
## 1:               match               match               match
## 2:               match                <NA>                <NA>
## 3:               match               match               match
## 4:               match               match               match
## 5:               match               match               match
## 6:               match               match               match
##    SAI_5b_SAI_5x_REF SAI_5b_SAI_5x_ALT SAI_13b_SAI_13x_REF SAI_13b_SAI_13x_ALT
## 1:             match             match               match               match
## 2:             match          mismatch               match            mismatch
## 3:             match             match               match               match
## 4:             match             match            mismatch            mismatch
## 5:             match             match               match               match
## 6:             match             match               match               match  
 Get the summary 
      summary_bx  <-   process_data_object ( "data_bx_dt" ) 
    head (summary_bx)    
  ##          SNP_id REF_match REF_mismatch ALT_match ALT_mismatch Zigo_match
## 1: AX-579436125        15            3        18            0         15
## 2: AX-579436196        14            2        16            0         14
## 3: AX-579436243        18            0        18            0         18
## 4: AX-579436298        16            1        12            5         13
## 5: AX-579436308        18            0        18            0         18
## 6: AX-579436348        18            0        18            0         18
##    Zigo_mismatch
## 1:             3
## 2:             2
## 3:             0
## 4:             4
## 5:             0
## 6:             0  
 Make data long format for plotting 
      dt_long_bx  <-   process_summary_object ( "summary_bx" ) 
    head (dt_long_bx)    
  ##                type count     n      perc
## 1: Reference Allele     3  3861  3.830927
## 2: Reference Allele     2  7427  7.369152
## 3: Reference Allele     0 66782 66.261845
## 4: Reference Allele     1 16746 16.615568
## 5: Reference Allele     5  1168  1.158903
## 6: Reference Allele     4  2155  2.138215  
 Create plot of SNP error per sample 
       plot_dt_long ( "bx" )    
   
 Compare both populations 
      summary_sai_bx  <-   generate_summary (data_bx_dt,  "SAI" ,  "suffix" ) 
   summary_kat_bx  <-   generate_summary (data_bx_dt,  "KAT" ,  "suffix" ) 
   dt_long_2_bx  <-   merge_and_transform ( "bx" ) 
    create_plot2 ( "bx" ,  here ( "output" ,  "wgs_vs_chip" ,  "figures" ,  "bx_mismatches_SAI_KAT.pdf" ), dt_long_2_bx)    
   
 Counts plot 
       # Call the function with data_*_dt as input  
   counts_bx  <-   calculate_counts (data_bx_dt) 
    plot_counts (counts_bx,  here ( "output" ,  "wgs_vs_chip" ,  "figures" ,  "bx_SAI_KAT_per_sample_stats.pdf" ))    
   
 
 
 12.3 “cw” - WGS call with 800 samples and chip call with 500
samples 
 Generate csv files 
       python  output/wgs_vs_chip/scripts/create_csv_from_vcfs.py cw    
 Import csv 
      data_cw_dt  <-   process_csv_files ( "cw" ) 
    
    # Check and display only columns that match the criteria  
    head (data_cw_dt[,  c ( "SNP_id" ,  names (data_cw_dt)[ grepl ( "_REF$|_ALT$" ,  names (data_cw_dt))]),  with =   FALSE ])    
  ##          SNP_id KAT_9c_KAT_9w_REF KAT_9c_KAT_9w_ALT SAI_15c_SAI_15w_REF
## 1: AX-583035067             match             match               match
## 2: AX-583033342             match             match               match
## 3: AX-583033356             match             match               match
## 4: AX-583033370             match             match               match
## 5: AX-583035194             match             match               match
## 6: AX-583033387             match             match               match
##    SAI_15c_SAI_15w_ALT SAI_3c_SAI_3w_REF SAI_3c_SAI_3w_ALT KAT_12c_KAT_12w_REF
## 1:               match             match             match               match
## 2:               match             match             match               match
## 3:               match          mismatch             match               match
## 4:               match          mismatch             match               match
## 5:               match             match             match               match
## 6:               match             match             match               match
##    KAT_12c_KAT_12w_ALT KAT_7c_KAT_7w_REF KAT_7c_KAT_7w_ALT SAI_2c_SAI_2w_REF
## 1:               match             match          mismatch             match
## 2:               match             match             match             match
## 3:               match             match             match             match
## 4:               match             match             match             match
## 5:               match             match             match             match
## 6:               match             match             match             match
##    SAI_2c_SAI_2w_ALT SAI_14c_SAI_14w_REF SAI_14c_SAI_14w_ALT KAT_8c_KAT_8w_REF
## 1:             match               match               match             match
## 2:             match               match               match             match
## 3:             match               match               match             match
## 4:             match               match               match             match
## 5:             match               match               match             match
## 6:             match               match            mismatch             match
##    KAT_8c_KAT_8w_ALT SAI_13c_SAI_13w_REF SAI_13c_SAI_13w_ALT SAI_5c_SAI_5w_REF
## 1:             match               match               match             match
## 2:             match               match               match             match
## 3:             match               match               match             match
## 4:             match               match               match             match
## 5:             match               match               match             match
## 6:             match               match               match             match
##    SAI_5c_SAI_5w_ALT SAI_18c_SAI_18w_REF SAI_18c_SAI_18w_ALT
## 1:             match               match               match
## 2:             match               match               match
## 3:             match               match               match
## 4:             match               match               match
## 5:             match               match               match
## 6:             match               match               match
##    KAT_10c_KAT_10w_REF KAT_10c_KAT_10w_ALT SAI_1c_SAI_1w_REF SAI_1c_SAI_1w_ALT
## 1:               match               match             match             match
## 2:               match               match             match             match
## 3:                <NA>                <NA>             match             match
## 4:               match               match             match             match
## 5:               match               match             match          mismatch
## 6:               match               match             match          mismatch
##    SAI_17c_SAI_17w_REF SAI_17c_SAI_17w_ALT SAI_4c_SAI_4w_REF SAI_4c_SAI_4w_ALT
## 1:               match               match             match             match
## 2:               match               match             match             match
## 3:               match               match             match             match
## 4:               match               match             match             match
## 5:               match               match             match             match
## 6:               match            mismatch             match             match
##    SAI_12c_SAI_12w_REF SAI_12c_SAI_12w_ALT KAT_11c_KAT_11w_REF
## 1:               match               match               match
## 2:               match               match               match
## 3:               match               match                <NA>
## 4:               match               match               match
## 5:               match               match               match
## 6:               match            mismatch               match
##    KAT_11c_KAT_11w_ALT SAI_16c_SAI_16w_REF SAI_16c_SAI_16w_ALT
## 1:            mismatch               match               match
## 2:               match               match               match
## 3:                <NA>            mismatch               match
## 4:               match               match               match
## 5:               match               match               match
## 6:               match               match            mismatch  
 Get the summary 
      summary_cw  <-   process_data_object ( "data_cw_dt" ) 
    head (summary_cw)    
  ##          SNP_id REF_match REF_mismatch ALT_match ALT_mismatch Zigo_match
## 1: AX-579436089        15            1        16            0         15
## 2: AX-579436149        18            0        18            0         18
## 3: AX-579436196        15            2        17            0         15
## 4: AX-579436243        18            0        18            0         18
## 5: AX-579436298        16            1        14            3         13
## 6: AX-579436308        18            0        18            0         18
##    Zigo_mismatch
## 1:             1
## 2:             0
## 3:             2
## 4:             0
## 5:             4
## 6:             0  
 Make data long format for plotting 
      dt_long_cw  <-   process_summary_object ( "summary_cw" ) 
    head (dt_long_cw)    
  ##                type count     n       perc
## 1: Reference Allele     1 17110 16.1682022
## 2: Reference Allele     0 71793 67.8412473
## 3: Reference Allele     2  7336  6.9321994
## 4: Reference Allele     5  1105  1.0441767
## 5: Reference Allele     3  3758  3.5511458
## 6: Reference Allele     7   350  0.3307347  
 Create plot of SNP error per sample 
       plot_dt_long ( "cw" )    
   
 Compare both populations 
      summary_sai_cw  <-   generate_summary (data_cw_dt,  "SAI" ,  "suffix" ) 
   summary_kat_cw  <-   generate_summary (data_cw_dt,  "KAT" ,  "suffix" ) 
   dt_long_2_cw  <-   merge_and_transform ( "cw" ) 
    create_plot2 ( "cw" ,  here ( "output" ,  "wgs_vs_chip" ,  "figures" ,  "cw_mismatches_SAI_KAT.pdf" ), dt_long_2_cw)    
   
 Counts plot 
       # Call the function with data_*_dt as input  
   counts_cw  <-   calculate_counts (data_cw_dt) 
    plot_counts (counts_cw,  here ( "output" ,  "wgs_vs_chip" ,  "figures" ,  "cw_SAI_KAT_per_sample_stats.pdf" ))    
   
 
 
 
 13. Statistical comparisons 
 Function to get the Zygosity summary for each object 
      create_Zygosity_df  <-   function (counts_df) { 
     Zygosity_df  <-  counts_df  |>  
        filter (Comparison  ==   "Zygosity" )  |>  
       dplyr ::  select ( 
         Population, 
         Sample, 
         Total, 
         Match, 
         Percent_Match, 
         Mismatch, 
         Percent_Mismatch 
       ) 
      
      return (Zygosity_df) 
   }    
 Apply the function 
       # chip  
   Zygosity_ab  <-   create_Zygosity_df (counts_ab) 
   Zygosity_ac  <-   create_Zygosity_df (counts_ac) 
   Zygosity_bc  <-   create_Zygosity_df (counts_bc) 
    
    # wgs  
   Zygosity_xy  <-   create_Zygosity_df (counts_xy) 
   Zygosity_wy  <-   create_Zygosity_df (counts_wy) 
   Zygosity_wx  <-   create_Zygosity_df (counts_wx) 
    
    # wgs x chip  
   Zygosity_ay  <-   create_Zygosity_df (counts_ay) 
   Zygosity_bx  <-   create_Zygosity_df (counts_bx) 
   Zygosity_cw  <-   create_Zygosity_df (counts_cw)    
 Use library(ggstatsplot) to compare the mean error rate for Zygosity.
We classified each loci as homo_ref, homo_alt, and het. Then we checked
if they matched or not. 
       # Add source columns to each data frame  
   Zygosity_ab $ Source  <-   'ab'  
   Zygosity_ac $ Source  <-   'ac'  
   Zygosity_bc $ Source  <-   'bc'  
    
   Zygosity_xy $ Source  <-   'xy'  
   Zygosity_wy $ Source  <-   'wy'  
   Zygosity_wx $ Source  <-   'wx'  
    
   Zygosity_ay $ Source  <-   'ay'  
   Zygosity_bx $ Source  <-   'bx'  
   Zygosity_cw $ Source  <-   'cw'  
    
    
    # Combine all data frames  
   combined_data  <-  
      rbind ( 
       Zygosity_ab, 
       Zygosity_ac, 
       Zygosity_bc, 
       Zygosity_xy, 
       Zygosity_wy, 
       Zygosity_wx, 
       Zygosity_ay, 
       Zygosity_bx, 
       Zygosity_cw 
     )    
 For KAT 
       # Specify the desired order  
   desired_order  <-   c ( "ab" ,  "ac" ,  "bc" ,  "xy" ,  "wy" ,  "wx" ,  "ay" ,  "bx" ,  "cw" ) 
    
    # Convert the 'Source' column to a factor and specify the order of the levels  
   combined_data $ Source  <-   factor (combined_data $ Source,  levels =  desired_order) 
    
    # For KAT  
   data_KAT_t  <-   subset (combined_data, Population  ==   "KAT" ) 
    
    
    # first, assign the plot to a variable  
   plot_KAT_plot  <-   ggbetweenstats ( 
      data =  data_KAT_t, 
      x =  Source, 
      y =  Percent_Mismatch, 
      title =   "Genotyping mismatches for KAT (native)" , 
      type =   "nonparametric" ,  
      pairwise.comparisons =   TRUE ,  
      pairwise.display =   "significant" , 
      palette =   "RdYlBu" ,  # change to a different palette if you prefer  
      package =   "RColorBrewer"  
   ) 
    
   plot_KAT_plot    
   
       # Use here function to specify the path  
   output_path  <-   here ( "output" ,  "wgs_vs_chip" ,  "figures" ,  "stats_KAT.pdf" ) 
    
    # Save the plot  
    ggsave ( filename =  output_path,  plot =  plot_KAT_plot,  width =   10 ,  height =   7 ,  dpi =   300 )    
 For SAI 
       # For SAI  
   data_SAI_t  <-   subset (combined_data, Population  ==   "SAI" ) 
    
    # first, assign the plot to a variable  
   plot_SAI_plot  <-   ggbetweenstats ( 
      data =  data_SAI_t, 
      x =  Source, 
      y =  Percent_Mismatch, 
      title =   "Genotyping mismatches for SAI (invasive)" , 
      type =   "nonparametric" ,  
      pairwise.comparisons =   TRUE ,  
      pairwise.display =   "significant" , 
      palette =   "RdYlBu" ,  # change to a different palette if you prefer  
      package =   "RColorBrewer"  
   ) 
    
   plot_SAI_plot    
   
       # Use here function to specify the path  
   output_path  <-   here ( "output" ,  "wgs_vs_chip" ,  "figures" ,  "stats_SAI.pdf" ) 
    
    # Save the plot  
    ggsave ( filename =  output_path,  plot =  plot_SAI_plot,  width =   10 ,  height =   7 ,  dpi =   300 )    
 Comparison irrespective of population 
      plot_both_plot <-   ggbetweenstats ( 
      data =  combined_data,  # using the entire data here, not just KAT  
      x =  Source, 
      y =  Percent_Mismatch, 
      title =   "Comparison of mean percent mismatch between sources" , 
      type =   "nonparametric" , 
      pairwise.comparisons =   TRUE ,  
      pairwise.display =   "significant" , 
      palette =   "RdYlBu" , 
      package =   "RColorBrewer"  
   ) 
    
   plot_both_plot    
   
       # Use here function to specify the path  
   output_path  <-   here ( "output" ,  "wgs_vs_chip" ,  "figures" ,  "stats_both.pdf" ) 
    
    # Save the plot  
    ggsave ( filename =  output_path,  plot =  plot_both_plot,  width =   10 ,  height =   7 ,  dpi =   300 )    
 We can use library broom to get a table 
       set.seed ( 123 ) 
    # I put warning=FALSE because some of the values are close. In the next chunk we add some jitter and we will not get warnings.  
    
    # Conduct pairwise Wilcoxon test  
   result  <-   pairwise.wilcox.test ( 
       combined_data $ Percent_Mismatch, 
       combined_data $ Source, 
        p.adjust.method =   "holm"  
   ) 
    
    # Tidy the result to a dataframe  
   result_tidy  <-  broom ::  tidy (result) 
    
    # Print the result  
    print (result_tidy)    
  ## # A tibble: 36 × 3
##    group1 group2   p.value
##    <chr>  <chr>      <dbl>
##  1 ac     ab     0.384    
##  2 bc     ab     0.0000101
##  3 bc     ac     0.0000101
##  4 xy     ab     0.694    
##  5 xy     ac     1        
##  6 xy     bc     0.0000101
##  7 wy     ab     0.0000101
##  8 wy     ac     0.0000101
##  9 wy     bc     0.0000101
## 10 wy     xy     0.0000116
## # ℹ 26 more rows  
 Add jitters 
       set.seed ( 123 ) 
    # If we add jitters the p-values are slightly different.  
   combined_data $ Percent_Mismatch_jitter  <-   jitter (combined_data $ Percent_Mismatch,  amount =   1e-9 ) 
    
   result  <-   pairwise.wilcox.test ( 
       combined_data $ Percent_Mismatch_jitter, 
       combined_data $ Source, 
        p.adjust.method =   "holm"  
   ) 
    
    # Tidy the result to a dataframe  
   result_tidy  <-  broom ::  tidy (result) 
    
    # Print the result  
    print (result_tidy)    
  ## # A tibble: 36 × 3
##    group1 group2       p.value
##    <chr>  <chr>          <dbl>
##  1 ac     ab     0.382        
##  2 bc     ab     0.00000000793
##  3 bc     ac     0.00000000793
##  4 xy     ab     0.644        
##  5 xy     ac     1            
##  6 xy     bc     0.00000000793
##  7 wy     ab     0.00000000837
##  8 wy     ac     0.0000000423 
##  9 wy     bc     0.00000000793
## 10 wy     xy     0.000000278  
## # ℹ 26 more rows  
 Create table 
       # Calculate mean  
   mean_df  <-  combined_data  |>  
      group_by (Source)   |>  
      summarise ( Mean_Percent_Mismatch =   mean (Percent_Mismatch,  na.rm =   TRUE )) 
    
    # Calculate median  
   median_df  <-  combined_data  |>  
      group_by (Source)  |>  
      summarise ( Median_Percent_Mismatch =   median (Percent_Mismatch,  na.rm =   TRUE )) 
    
    # Pairwise Wilcoxon test  
   result  <-   pairwise.wilcox.test ( 
       combined_data $ Percent_Mismatch, 
       combined_data $ Source, 
        p.adjust.method =   "holm"  
   ) 
    
    # # Extract p-values and tidy the result into a data frame  
   pvalues_df  <-   as.data.frame (result $ p.value)  |>  
      rownames_to_column ( "Source" )  |>  
     dplyr ::  rename ( P_Value =   2 ) 
    # pvalues_df <-  
    #   tibble::rownames_to_column(as.data.frame(result$p.value), "Source") %>%  
    #   dplyr::rename(P_Value = 2)  
    
    # Merge mean, median and p-values into one table  
   summary_df  <-   full_join (mean_df, median_df,  by =   "Source" )  |>  
      full_join (pvalues_df,  by =   "Source" ) 
    
    # Rename "Source" column  
   summary_df  <-  dplyr ::  rename (summary_df,  Comparison =  Source) 
    
    # Function to format data for mean and median  
   format_mean_median  <-   function (x) { 
      round (x,  2 ) 
   } 
    
    # Function to format data for p-values  
   format_pvalue  <-   function (x) { 
     formatted_x  <-   ifelse ( abs (x)  <   1e-4 ,  formatC (x,  format =   "e" ,  digits =   4 ),  round (x,  4 )) 
      # Append an asterisk for p-values below 0.05  
      ifelse (x  <   0.05 ,  paste0 (formatted_x,  "*" ), formatted_x) 
   } 
    
    
    # Apply the function to each column as needed  
   summary_df $ Mean_Percent_Mismatch  <-   format_mean_median (summary_df $ Mean_Percent_Mismatch) 
   summary_df $ Median_Percent_Mismatch  <-   format_mean_median (summary_df $ Median_Percent_Mismatch) 
    
    # Apply the function to each column as needed  
   pvalue_cols  <-   colnames (summary_df)[ - ( 1  :  3 )] 
    for (col  in  pvalue_cols){ 
     summary_df[[col]]  <-   format_pvalue (summary_df[[col]]) 
   } 
    
    
    # Create the flextable  
   ft  <-  flextable ::  flextable (summary_df) 
    
    # Apply zebra theme  
   ft  <-  flextable ::  theme_zebra (ft) 
    
    # Add a caption to the table  
   ft  <-  flextable ::  add_header_lines (ft,  "Table 1: Mean and Median Percent Mismatch by Comparison. The P-values are from a pairwise Wilcoxon test with Holm adjustment for multiple comparisons. An asterisk (*) next to a P-value indicates a statistically significant difference (P < 0.05)." ) 
    
    # Save it to a Word document  
   officer ::  read_docx ()  |>  
      body_add_flextable (ft)  |>  
      print ( target =  here ::  here ( "output" ,  "wgs_vs_chip" ,  "figures" ,  "summary_table.docx" )) 
    
   ft    
        Table 1: Mean and Median Percent Mismatch by Comparison. The P-values are from a pairwise Wilcoxon test with Holm adjustment for multiple comparisons. An asterisk (*) next to a P-value indicates a statistically significant difference (P < 0.05).        Comparison      Mean_Percent_Mismatch      Median_Percent_Mismatch      P_Value      ac      bc      xy      wy      wx      ay      bx          ab      1.16      1.19                                                        ac      1.29      1.34      0.3841                                                  bc      0.41      0.43      1.0098e-05*      1.0098e-05*                                            xy      1.35      1.27      0.6937      1      1.0098e-05*                                      wy      3.09      2.67      1.0098e-05*      1.0098e-05*      1.0098e-05*      1.1608e-05*                                wx      2.99      2.58      1.0098e-05*      1.0098e-05*      1.0098e-05*      1.1608e-05*      1                          ay      8.11      8.71      1.0098e-05*      1.0098e-05*      1.0098e-05*      1.0098e-05*      1.0098e-05*      1.0098e-05*                    bx      7.14      7.58      1.0098e-05*      1.0098e-05*      1.0098e-05*      1.0098e-05*      2.0980e-06*      7.6958e-07*      0.3841              cw      6.70      7.10      1.0098e-05*      1.0098e-05*      1.0098e-05*      1.0098e-05*      2.7127e-06*      2.0980e-06*      0.0760      1       
 
 
 14. Check how many samples a SNP might have errors 
 We can look at each population or across all samples. The code below
assumes you have all the data loaded. 
 
 14.1 For chip (ac) 
 The “ac” comparison is for when we genotype 18 samples alone or use
around 500 samples in the genotype call. 
 We extracted the 18 samples out of the full data set to compare with
the 18 samples genotyped alone. 
 How many SNPs have discrepancies in the genotypes in 1 or more
samples (out of the 18 samples) 
       # Discrepancies in 1 or more samples  
    # How many SNPs we tested  
   tested_snps  <-   length ( unique (data_ac_dt $ SNP_id)) 
    cat ( "Number of SNPs tested:" , tested_snps,  "  \n  " )    
  ## Number of SNPs tested: 90252  
       # How many SNPs failed  
   failed_snpsR  <-  
      length ( 
        unique (data_ac_dt[data_ac_dt $ REF_mismatch_count  >=   1 ,] $ SNP_id 
              ) 
            ) 
    cat ( "REF mismatch at in 1 sample:" , failed_snpsR,  "  \n  " )    
  ## REF mismatch at in 1 sample: 7329  
       # How many SNPs failed  
   failed_snpsA  <-  
      length ( 
        unique (data_ac_dt[data_ac_dt $ ALT_mismatch_count  >=   1 ,,] $ SNP_id 
              ) 
            ) 
    cat ( "ALT mismatch at least in 1 sample:" , failed_snpsA,  "  \n  " )    
  ## ALT mismatch at least in 1 sample: 3782  
       # How many SNPs failed zygosity  
   failed_snps  <-  
      length ( 
        unique (data_ac_dt[data_ac_dt $ Zigo_mismatch_count  >=   1 ,,] $ SNP_id 
              ) 
            ) 
    cat ( "Zygosity mismatch in at least 1 sample:" , failed_snps,  "  \n  " )    
  ## Zygosity mismatch in at least 1 sample: 10545  
       # Calculate percentage  
   percentage_failed  <-   round (failed_snps  /  tested_snps  *   100 ,  2 ) 
    cat ( "Percentage of failed SNPs in 1 or more samples:" , percentage_failed,  "%  \n  " )    
  ## Percentage of failed SNPs in 1 or more samples: 11.68 %  
 When we look at the Zygosity of each SNP we find that 10,545 SNPs
have mismatches (11.68%). However, we see from the previous plot that we
have SNPs showing discrepancies in only 1 sample out of the 18
samples. 
 Check how many SNPs have erros in 2 or more samples 
       # Discrepancies in 2 or more samples  
    # How many SNPs we tested  
   tested_snps  <-   length ( unique (data_ac_dt $ SNP_id)) 
    cat ( "Number of SNPs tested:" , tested_snps,  "  \n  " )    
  ## Number of SNPs tested: 90252  
       # How many SNPs failed  
   failed_snpsR  <-  
      length ( 
        unique (data_ac_dt[data_ac_dt $ REF_mismatch_count  >=   2 ,] $ SNP_id 
              ) 
            ) 
    cat ( "REF mismatch in 2 or more samples:" , failed_snpsR,  "  \n  " )    
  ## REF mismatch in 2 or more samples: 3002  
       # How many SNPs failed  
   failed_snpsA  <-  
      length ( 
        unique (data_ac_dt[data_ac_dt $ ALT_mismatch_count  >=   2 ,] $ SNP_id 
              ) 
            ) 
    cat ( "ALT mismatch in 2 or more samples:" , failed_snpsA,  "  \n  " )    
  ## ALT mismatch in 2 or more samples: 1417  
       # How many SNPs failed  
   failed_snps  <-  
      length ( 
        unique (data_ac_dt[data_ac_dt $ Zigo_mismatch_count  >=   2 ,] $ SNP_id 
              ) 
            ) 
    cat ( "Zygosity mismatch in 2 or more samples:" , failed_snps,  "  \n  " )    
  ## Zygosity mismatch in 2 or more samples: 4396  
       # Calculate percentage  
   percentage_failed  <-   round (failed_snps  /  tested_snps  *   100 ,  2 ) 
    cat ( "Percentage of failed SNPs in 2 or more samples:" , percentage_failed,  "%  \n  " )    
  ## Percentage of failed SNPs in 2 or more samples: 4.87 %  
 We can check how many times a SNP has mismatching Zygosity or alleles
across the 18 samples. 
       # Number of samples you want to iterate over  
   num_samples  <-   18  
    
    # Create an empty data frame to store results  
   results2  <-   data.frame () 
    
    # How many SNPs we tested  
   tested_snps  <-   length ( unique (data_ac_dt $ SNP_id)) 
    
    for (i  in   1  : num_samples){ 
      
      # How many SNPs failed REF  
     failed_snpsR  <-   length ( unique (data_ac_dt[data_ac_dt $ REF_mismatch_count  >=  i,] $ SNP_id)) 
      
      # How many SNPs failed ALT  
     failed_snpsA  <-   length ( unique (data_ac_dt[data_ac_dt $ ALT_mismatch_count  >=  i,] $ SNP_id)) 
      
      # How many SNPs failed zygosity  
     failed_snpsZ  <-   length ( unique (data_ac_dt[data_ac_dt $ Zigo_mismatch_count  >=  i,] $ SNP_id)) 
      
      # Calculate percentage  
     percentage_failed  <-   round (failed_snpsZ  /  tested_snps  *   100 ,  2 ) 
      
      # Create a data frame with results for this number of samples  
     temp_results  <-   data.frame ( 
        'Samples'   =  i, 
        'SNPs'   =  tested_snps, 
        'Mismatch_REF'   =  failed_snpsR, 
        'Mismatch_ALT'   =  failed_snpsA, 
        'Mismatch_Zygosity'   =  failed_snpsZ, 
        'Mismatch_Zygosity_perc'   =  percentage_failed 
     ) 
      
      # Append the results to the main results data frame  
     results2  <-   rbind (results2, temp_results) 
      
   } 
    
    # Create the flextable  
   ft  <-   flextable (results2) 
    
    # Apply zebra theme  
   ft  <-   theme_zebra (ft) 
    
    # Add a caption to the table  
   ft  <-   add_header_lines (ft,  "Table 2: Summary of the SNP mismatch rate for the 18 samples genotyped alone or with 500 samples. " ) 
    
    # Save it to a Word document  
   officer ::  read_docx ()  |>  
      body_add_flextable (ft)  |>  
      print ( target =  here ::  here ( "output" ,  "wgs_vs_chip" ,  "figures" ,  "summary_ac.docx" ))    
 KAT 
       # Discrepancies in 1 or more samples  
    # How many SNPs we tested  
   tested_snps_ac  <-   length ( unique (summary_kat_ac $ SNP_id)) 
    cat ( "Number of SNPs tested:" , tested_snps_ac,  "  \n  " )    
  ## Number of SNPs tested: 90252  
       # How many SNPs failed  
   failed_kat_ac  <-  
      length ( unique (summary_kat_ac[summary_kat_ac $ REF_mismatch  >   0   |  
                              summary_kat_ac $ ALT_mismatch  >   0   |  
                              summary_kat_ac $ Zigo_mismatch  >   0 , ] $ SNP_id)) 
    cat ( "Number of SNPs failed:" , failed_kat_ac,  "  \n  " )    
  ## Number of SNPs failed: 3029  
       # Calculate percentage  
   percentage_failed_ac  <-   round (failed_kat_ac  /  tested_snps_ac  *   100 ,  2 ) 
    cat ( "Percentage of failed SNPs:" , percentage_failed_ac,  "%  \n  " )    
  ## Percentage of failed SNPs: 3.36 %  
       # How many SNPs failed KAT  
   failed_kat_ac  <-  
      length ( unique (summary_kat_ac[summary_kat_ac $ REF_mismatch  >   0   |  
                              summary_kat_ac $ ALT_mismatch  >   0   |  
                              summary_kat_ac $ Zigo_mismatch  >   0 , ] $ SNP_id)) 
    cat ( "Number of SNPs failed:" , failed_kat_ac,  "  \n  " )    
  ## Number of SNPs failed: 3029  
       # How many SNPs failed SAI  
   failed_sai_ac  <-  
      length ( unique (summary_sai_ac[summary_sai_ac $ REF_mismatch  >   0   |  
                              summary_sai_ac $ ALT_mismatch  >   0   |  
                              summary_sai_ac $ Zigo_mismatch  >   0 , ] $ SNP_id)) 
    cat ( "Number of SNPs failed:" , failed_sai_ac,  "  \n  " )    
  ## Number of SNPs failed: 8579  
       # Calculate percentage  
   percentage_kat_ac  <-   round (failed_kat_ac  /  tested_snps_ac  *   100 ,  2 ) 
    cat ( "Percentage of failed SNPs:" , percentage_kat_ac,  "%  \n  " )    
  ## Percentage of failed SNPs: 3.36 %  
      percentage_sai_ac  <-   round (failed_sai_ac  /  tested_snps_ac  *   100 ,  2 ) 
    cat ( "Percentage of failed SNPs:" , percentage_sai_ac,  "%  \n  " )    
  ## Percentage of failed SNPs: 9.51 %  
 Summary 
       # Create an empty data frame to store results  
   results_ac  <-   data.frame () 
    
    # How many SNPs we tested  
   tested_snps_ac  <-   length ( unique (summary_kat_ac $ SNP_id)) 
    
    # Datasets and corresponding number of samples  
   datasets_ac  <-   list ( KAT=  list ( data= summary_kat_ac,  num_samples=  6 ),  SAI=  list ( data= summary_sai_ac,  num_samples=  12 )) 
    
    for (name  in   names (datasets_ac)){ 
     data  <-  datasets_ac[[name]] $ data 
     num_samples  <-  datasets_ac[[name]] $ num_samples 
      
      for (i  in   1  : num_samples){ 
        
        # How many SNPs failed  
       failed_snps  <-   length ( unique (data[data $ REF_mismatch  >=  i  |  
                                         data $ ALT_mismatch  >=  i  |
[truncated: 310,370 more chars]
