# Supplementary material for: A genotyping array for the globally invasive vector mosquito, Aedes albopictus
Source: Parasit Vectors. 2024 Mar 4;17:106. doi: 10.1186/s13071-024-06158-z (PMC10910840; doi:10.1186/s13071-024-06158-z)
Supplement: Supplementary file 5 — Additional file 5. Comparing the genotypes of replicate samples. [file 13071_2024_6158_MOESM5_ESM.html]

Aedes albopictus SNP chip - Comparying the genotypes of replicates.


# Aedes albopictus SNP chip - Comparying the genotypes of replicates.

#### Luciano V Cosme

#### 2023-08-23

- Comparison of genotypes
  of triplicates
- Analytic
  approach
  - 1. Load
    libraries
  - 2. Data
    preparation
    - 2.1 Use Plink2 to subset the
      data
  - 3. Calculate
    mismatches using a Python script
    - 3.1 Generate vcfs with two
      samples each
    - 3.2 Script to
      compare alleles between the replicates
    - 3.3.
      Import the csv into R
    - 3.4 Calculate total
      error rate per population

# Comparison of genotypes of triplicates

We genotyped the same samples with the chip 3 times. We initially
selected 5 samples from different populations but one of them, from
Vietnam, did not work. We have 4 samples that were genotyped 3 times
each. I genotyped the samples with the wild animals and we will subset
the data set to perform the comparisons. I used the default prior.

# Analytic approach

1. **Data Preparation**
   1. Genotyped around 500 samples including the triplicates with the
      triplicates.
   2. Set the reference allele to match the ‘AalbF3’ genome assembly for
      both WGS and SNP chip data.
   3. Convert the genotyping data into VCF format
2. **Pairwise Comparisons**
   1. Develop Python to perform pairwise comparisons of the genotypes from
      each duplicates in the vcf file
   2. Check the concordance between the two sample pairs.
3. **Results Summarization**
   1. Compile the results of the pairwise comparisons into a
      comprehensible format (e.g., a table or graph).
   2. Calculate summary statistics that capture the level of agreement or
      discrepancy between the duplicates

## 1. Load libraries

```
library(tidyverse)
library(here)
library(colorout)
library(flextable)
library(reticulate)
library(dplyr)
library(officer)
```

## 2. Data preparation

Check the fam file of the full data set

```
# Check head of data
head data/raw_data/albo/populations/chip_500_dp_01.fam
```

```
## OKI  1001    0   0   2   -9
## OKI  1002    0   0   2   -9
## OKI  1003    0   0   2   -9
## OKI  1004    0   0   2   -9
## OKI  1005    0   0   2   -9
## OKI  1006    0   0   1   -9
## OKI  1007    0   0   1   -9
## OKI  1008    0   0   1   -9
## OKI  1009    0   0   1   -9
## OKI  1010    0   0   1   -9
```

We can check the triplicates

```
# Check triplicates
grep "a\|b\|c" data/raw_data/albo/populations/chip_500_dp_01.fam
```

```
## KAG  a   0   0   0   -9
## KAG  b   0   0   0   -9
## KAG  c   0   0   0   -9
## BEN  a   0   0   0   -9
## BEN  b   0   0   0   -9
## BEN  c   0   0   0   -9
## BAR  a   0   0   0   -9
## BAR  b   0   0   0   -9
## BAR  c   0   0   0   -9
## LOS  a   0   0   0   -9
## LOS  b   0   0   0   -9
## LOS  c   0   0   0   -9
```

### 2.1 Use Plink2 to subset the data

Create output directory

```
# Create main directory
dir.create(
  here("output", "populations", "triplicates"),
  showWarnings = FALSE,
  recursive = FALSE
)
```

To subset the data we need to create a list of samples with family id
and individual ids

```
grep "a\|b\|c" data/raw_data/albo/populations/chip_500_dp_01.fam | awk '{print $1, $2}' > output/populations/triplicates/triplicates.txt;
head output/populations/triplicates/triplicates.txt
```

```
## KAG a
## KAG b
## KAG c
## BEN a
## BEN b
## BEN c
## BAR a
## BAR b
## BAR c
## LOS a
```

Create a new bed file with the triplicates

```
# I created a fam file with the information about each sample, but first we import the data and create a bed file setting the family id constant
plink2 \
--allow-extra-chr \
--bfile data/raw_data/albo/populations/chip_500_dp_01 \
--make-bed \
--fa data/genome/albo.fasta.gz \
--ref-from-fa 'force' `# sets REF alleles when it can be done unambiguously, we use force to change the alleles` \
--keep output/populations/triplicates/triplicates.txt \
--out output/populations/triplicates/triplicates `# dp - default priors` \
--silent;
# --keep-allele-order \ if you use Plink 1.9
grep "samples\|variants" output/populations/triplicates/triplicates.log # to get the number of variants from the log file.
```

```
## 479 samples (138 females, 130 males, 211 ambiguous; 479 founders) loaded from
## 107294 variants loaded from data/raw_data/albo/populations/chip_500_dp_01.bim.
## --keep: 12 samples remaining.
## 12 samples (0 females, 0 males, 12 ambiguous; 12 founders) remaining after main
## --ref-from-fa force: 0 variants changed, 107294 validated.
```

Using the default prior we obtained 107,294 SNPs. All the reference
alleles matched the reference genome (AalbF3).

Check the headings of the the files we will work on.

```
head -n 12 output/populations/triplicates/triplicates.fam
```

```
## KAG  a   0   0   0   -9
## KAG  b   0   0   0   -9
## KAG  c   0   0   0   -9
## BEN  a   0   0   0   -9
## BEN  b   0   0   0   -9
## BEN  c   0   0   0   -9
## BAR  a   0   0   0   -9
## BAR  b   0   0   0   -9
## BAR  c   0   0   0   -9
## LOS  a   0   0   0   -9
## LOS  b   0   0   0   -9
## LOS  c   0   0   0   -9
```

Lets add a number to the individual ids

```
# Merge ids
awk 'BEGIN {i=1; last=""} {if ($1 != last) {last=$1; i++} $2=i$2; print}' output/populations/triplicates/triplicates.fam | head
```

```
## KAG 2a 0 0 0 -9
## KAG 2b 0 0 0 -9
## KAG 2c 0 0 0 -9
## BEN 3a 0 0 0 -9
## BEN 3b 0 0 0 -9
## BEN 3c 0 0 0 -9
## BAR 4a 0 0 0 -9
## BAR 4b 0 0 0 -9
## BAR 4c 0 0 0 -9
## LOS 5a 0 0 0 -9
```

Do it in place

```
# Run this only once
awk 'BEGIN {i=1; last=""} {if ($1 != last) {last=$1; i++} $2=i$2; print}' output/populations/triplicates/triplicates.fam > output/populations/triplicates/tmpfile && mv output/populations/triplicates/tmpfile output/populations/triplicates/triplicates.fam;
head output/populations/triplicates/triplicates.fam
```

```
## KAG 2a 0 0 0 -9
## KAG 2b 0 0 0 -9
## KAG 2c 0 0 0 -9
## BEN 3a 0 0 0 -9
## BEN 3b 0 0 0 -9
## BEN 3c 0 0 0 -9
## BAR 4a 0 0 0 -9
## BAR 4b 0 0 0 -9
## BAR 4c 0 0 0 -9
## LOS 5a 0 0 0 -9
```

## 3. Calculate mismatches using a Python script

Now we can subset the samples and keep the pairs we are
interested.

**Code Explanation:**

1. **Variable Initialization:**
   - The code defines a variable “input\_file” with the value
     “output/populations/triplicates/triplicates.fam”.
   - It defines a variable “output\_dir” with the value
     “output/populations/triplicates/vcfs”.
   - It defines a variable “bfile” with the value
     “output/populations/triplicates/triplicates”.
2. **Create Output Directory:**
   - The code creates the output directory if it does not exist using the
     following command: mkdir -p $output\_dir
3. **Retrieve Unique Families:**
   - It retrieves the unique families from the input file specified by
     “input\_file”.
   - The “awk” command extracts the first column from the input
     file.
   - The “sort” command sorts the extracted column.
   - The “uniq” command filters out duplicate entries.
   - The resulting unique families are stored in the “families”
     variable.
4. **Loop Over Families:**
   - The code enters a loop over each family (“famid”) in the “families”
     variable.
5. **Retrieve Base Sample IDs:**
   - Within the family loop, it retrieves the base sample IDs (without
     ‘a’, ‘b’, or ‘c’ suffixes) for the current family.
   - The “grep” command filters the input file based on the current
     family.
   - The “awk” command extracts the second column (base sample IDs) from
     the filtered lines.
   - The “sed” command removes the ‘a’, ‘b’, or ‘w’ suffixes from the
     base sample IDs.
   - The “uniq” command filters out duplicate entries.
   - The resulting base sample IDs are stored in the “base\_iids”
     variable.
6. **Nested Loop Over Base Sample IDs and Combinations:**
   - The code enters another loop over each base sample ID (“base\_iid”)
     in the “base\_iids” variable.
   - Within the base sample ID loop, it enters a nested loop over three
     combinations: “ab”, “ac”, and “bc”.
7. **Check Sample Existence:**
   - For each combination, the code checks if both samples exist in the
     input file.
   - It uses the “grep” command with regular expressions and the “-q”
     option to suppress output.
   - If both samples exist, it proceeds with the following steps.
8. **Create Temporary File:**
   - It creates a temporary file using the “mktemp” command to store the
     relevant lines from the input file.
9. **Extract Relevant Lines:**
   - The code uses the “grep” command to extract the lines from the input
     file that match the family, base sample ID, and current
     combination.
   - The matching lines are appended to the temporary file.
10. **Execute plink2:**
    - It executes the “plink2” command with various options and arguments
      to perform specific operations on the data.
    - The command performs tasks such as allowing extra chromosomes,
      preserving allele order, using the specified binary file (“bfile”),
      applying filters, and specifying the output format.
    - The output is saved to a VCF file with a name based on the family,
      base sample ID, and combination.
    - The “–silent” option suppresses unnecessary output.
11. **Remove Temporary File:**
    - After executing “plink2”, it removes the temporary file using the
      “rm” command.
12. **Continuation of Nested Loops:**
    - The code continues the nested loops until all combinations and base
      sample IDs have been processed.

Check the fam file to make sure we have it in the right format

```
head output/populations/triplicates/triplicates.fam
```

```
## KAG 2a 0 0 0 -9
## KAG 2b 0 0 0 -9
## KAG 2c 0 0 0 -9
## BEN 3a 0 0 0 -9
## BEN 3b 0 0 0 -9
## BEN 3c 0 0 0 -9
## BAR 4a 0 0 0 -9
## BAR 4b 0 0 0 -9
## BAR 4c 0 0 0 -9
## LOS 5a 0 0 0 -9
```

### 3.1 Generate vcfs with two samples each

```
input_file="output/populations/triplicates/triplicates.fam"
output_dir="output/populations/triplicates/vcfs"
bfile="output/populations/triplicates/triplicates"

# create the output directory if it does not exist
mkdir -p $output_dir

# get unique families
families=$(awk '{print $1}' $input_file | sort | uniq)

for famid in $families; do
  # get the base sample ids (without a, b, w)
  base_iids=$(grep "$famid" $input_file | awk '{print $2}' | sed 's/[abc]$//' | uniq)
  
  for base_iid in $base_iids; do
    for combination in "ab" "ac" "bc"; do
      # Check if both samples exist
      if grep -qE "${famid}\s${base_iid}[${combination:0:1}]\s" "$input_file" && 
         grep -qE "${famid}\s${base_iid}[${combination:1:1}]\s" "$input_file"; then
        # Create temporary file
        tmp_file=$(mktemp)
        grep -E "${famid}\s${base_iid}[${combination:0:1}]\s" "$input_file" > "$tmp_file"
        grep -E "${famid}\s${base_iid}[${combination:1:1}]\s" "$input_file" >> "$tmp_file"
  
        # Execute plink2
        plink2 \
        --allow-extra-chr \
        --keep-allele-order \
        --bfile $bfile \
        --keep "$tmp_file" \
        --recode vcf-iid \
        --geno 0 \
        --out "$output_dir/${famid}_${base_iid}${combination}" \
        --silent
  
        # Remove temporary file
        rm "$tmp_file"
      fi
    done
  done
done
```

Check how many SNPs per vcf

```
# Define directory with the vcfs
output_dir="output/populations/triplicates/vcfs"
# Count how many SNPs we have in each vcf file
for file in ${output_dir}/*.vcf; do
    echo $(basename $file): $(grep -v '^#' $file | wc -l)
done
```

```
## BAR_4ab.vcf: 102924
## BAR_4ac.vcf: 102772
## BAR_4bc.vcf: 103194
## BEN_3ab.vcf: 101645
## BEN_3ac.vcf: 102831
## BEN_3bc.vcf: 101799
## KAG_2ab.vcf: 104078
## KAG_2ac.vcf: 104025
## KAG_2bc.vcf: 103989
## LOS_5ab.vcf: 103430
## LOS_5ac.vcf: 103237
## LOS_5bc.vcf: 103201
```

Check sample names to see if our code created the vcfs with two
samples

```
# Define directory with the VCFs
output_dir="output/populations/triplicates/vcfs"

# Iterate over each VCF file
for file in "${output_dir}"/*.vcf; do
    # Extract the file name without the directory path
    file_name=$(basename "${file}")

    # Use bcftools query to retrieve the sample names
    sample_names=$(bcftools query -l "${file}")
    
    # Print the file name and the sample names
    echo "${file_name}: ${sample_names}"
done
```

```
## BAR_4ab.vcf: 4a
## 4b
## BAR_4ac.vcf: 4a
## 4c
## BAR_4bc.vcf: 4b
## 4c
## BEN_3ab.vcf: 3a
## 3b
## BEN_3ac.vcf: 3a
## 3c
## BEN_3bc.vcf: 3b
## 3c
## KAG_2ab.vcf: 2a
## 2b
## KAG_2ac.vcf: 2a
## 2c
## KAG_2bc.vcf: 2b
## 2c
## LOS_5ab.vcf: 5a
## 5b
## LOS_5ac.vcf: 5a
## 5c
## LOS_5bc.vcf: 5b
## 5c
```

### 3.2 Script to compare alleles between the replicates

Code summary: The provided code performs the following steps:

1. **Import the necessary libraries** The code imports
   the required libraries: “allel”, “pandas”, “os”, and “numpy”.
2. **Create an empty DataFrame** The code initializes
   an empty DataFrame called “output\_df” to store the output results
   obtained from the analysis.
3. **Specify the directory** The code defines the
   directory path where the VCF files are located using the “dir\_name”
   variable.
4. **Retrieve a list of VCF files** The code uses the
   “os.listdir()” function and list comprehension to create a list of all
   VCF files in the specified directory that end with ‘.vcf’.
5. **Iterate over each VCF file** The code sets up a
   loop to iterate over each VCF file found in the previous step.
6. **Construct the file path** The code constructs the
   full file path for the current VCF file by combining the directory path
   and the file name using “os.path.join()”.
7. **Read the VCF file** The code reads the VCF file
   using “allel.read\_vcf()” from the “allel” library, specifying to load
   all available fields (’\*’).
8. **Extract the genotype data** The code extracts the
   genotype data from the VCF file using
   “allel.GenotypeArray(callset[‘calldata/GT’])”.
9. **Check sample count** The code verifies if the VCF
   file contains two samples by checking the shape of the genotype array
   using the “assert” statement. If the shape doesn’t match the expected
   number of samples, an assertion error is raised.
10. **Count total SNPs** The code determines the total
    number of SNPs in the genotype data by calculating the length of the
    genotype array using “len(gt)”.
11. **Calculate counts of homozygous and heterozygous
    SNPs** The code uses “np.count\_nonzero()” and relevant methods of
    the “gt” object to count the number of homozygous reference, homozygous
    alternate, and heterozygous SNPs for each sample.
12. **Compute counts of mismatched homozygous and heterozygous
    SNPs** The code compares the genotypes between the two samples
    using “np.sum()” to calculate the counts of mismatched homozygous
    reference, homozygous alternate, and heterozygous SNPs.
13. **Extract reference and alternative alleles** The
    code retrieves the reference and alternative alleles for each SNP from
    the VCF file.
14. **Count mismatching reference and alternative
    alleles** The code compares the alleles between the two samples
    and counts the number of SNPs with mismatching reference alleles and the
    number of SNPs with mismatching alternative alleles.
15. **Calculate counts of A, T, C, and G alleles** The
    code computes the counts of A, T, C, and G alleles for each sample based
    on the genotype data and the corresponding reference and alternative
    alleles.
16. **Create and append result to output dataframe** The
    code creates a DataFrame called “result” to store the calculated
    statistics for the current VCF file and appends it to the “output\_df”
    DataFrame using “pd.concat()”.
17. **Repeat for each VCF file** The code repeats steps
    5 to 16 for each VCF file in the directory, processing and appending the
    results to the “output\_df” DataFrame.
18. **Write the output to a CSV file** The code writes
    the final “output\_df” DataFrame to a CSV file named
    ‘allele\_comparison\_stats\_2.csv’ using the “to\_csv()” method of
    pandas.

```
import allel
import pandas as pd
import os
import numpy as np

# Initialize the output dataframe
output_df = pd.DataFrame()

# Directory with vcf files
dir_name = "output/populations/triplicates/vcfs"

# Get list of all vcf files in the directory
vcf_files = [f for f in os.listdir(dir_name) if f.endswith('.vcf')]

# Iterate over VCF files
for vcf_file in vcf_files:
    file_path = os.path.join(dir_name, vcf_file)
    callset = allel.read_vcf(file_path, fields=['*'])

    # Get genotype
    gt = allel.GenotypeArray(callset['calldata/GT'])
    
    # Verify the vcf contains two samples
    assert gt.shape[1] == 2, f"Expected 2 samples in {vcf_file}, found {gt.shape[1]}"

    # Count SNPs
    n_snps = len(gt)

    # Count homozygous and heterozygous SNPs for each sample
    n_homo_ref = np.count_nonzero(gt.is_hom_ref(), axis=0)
    n_homo_alt = np.count_nonzero(gt.is_hom_alt(), axis=0)
    n_hetero = np.count_nonzero(gt.is_het(), axis=0)
    
    # Count homozygous and heterozygous SNPs mismatches
    n_homo_ref_mismatch = np.sum(gt.is_hom_ref()[:, 0] != gt.is_hom_ref()[:, 1])
    n_homo_alt_mismatch = np.sum(gt.is_hom_alt()[:, 0] != gt.is_hom_alt()[:, 1])
    n_hetero_mismatch = np.sum(gt.is_het()[:, 0] != gt.is_het()[:, 1])

    # Get alleles
    ref_alleles = callset['variants/REF']
    alt_alleles = callset['variants/ALT'][:, 0]  # assuming bi-allelic

    # Count mismatching reference and alternative alleles
    n_snps_ref_mismatch = np.count_nonzero(ref_alleles[gt[:,0]] != ref_alleles[gt[:,1]])
    n_snps_alt_mismatch = np.count_nonzero(alt_alleles[gt[:,0]] != alt_alleles[gt[:,1]])

    # Count alleles for each sample
    n_a = sum(np.count_nonzero(gt == i, axis=0) for i in range(4) if ref_alleles[i] == 'A' or alt_alleles[i] == 'A')
    n_t = sum(np.count_nonzero(gt == i, axis=0) for i in range(4) if ref_alleles[i] == 'T' or alt_alleles[i] == 'T')
    n_c = sum(np.count_nonzero(gt == i, axis=0) for i in range(4) if ref_alleles[i] == 'C' or alt_alleles[i] == 'C')
    n_g = sum(np.count_nonzero(gt == i, axis=0) for i in range(4) if ref_alleles[i] == 'G' or alt_alleles[i] == 'G')

    # Append results to the output dataframe
    result = pd.DataFrame({
        'vcf_file': [file_path],
        'n_SNPs': [n_snps],
        'n_SNPs_ref_mismatch': [n_snps_ref_mismatch],
        'n_SNPs_alt_mismatch': [n_snps_alt_mismatch],
        'n_A': [n_a],
        'n_T': [n_t],
        'n_C': [n_c],
        'n_G': [n_g],
        'n_homo_ref': [n_homo_ref],
        'n_homo_alt': [n_homo_alt],
        'n_hetero': [n_hetero],
        'n_homo_ref_mismatch': [n_homo_ref_mismatch],
        'n_homo_alt_mismatch': [n_homo_alt_mismatch],
        'n_hetero_mismatch': [n_hetero_mismatch]
    })

    output_df = pd.concat([output_df, result])

# Write the result to a csv file
output_df.to_csv('output/populations/triplicates/allele_comparison_stats.csv', index=False)
```

Clean env

```
# python
py_run_string("import gc; gc.collect()")
```

### 3.3. Import the csv into R

Import the data

```
data <-
  read_delim(
    "output/populations/triplicates/allele_comparison_stats.csv",
    delim = ",",
    show_col_types = FALSE
  )

data <-
  data |>
  mutate(vcf_file = str_remove(vcf_file, "output/populations/triplicates/vcfs/")) |>
  separate(
    vcf_file,
    into = c("Population", "Sample_Comparison"),
    sep = "_",
    extra = "drop"
  ) |>
  separate(
    Sample_Comparison,
    into = c("Sample", "Comparison"),
    sep = "(?<=\\d)(?=[a-z])",
    convert = TRUE
  ) |>
  mutate(Comparison = str_remove(Comparison, ".vcf")) |>
  arrange(Comparison)

# Split the "Comparison" column into "Sample1" and "Sample2"
data <- 
  data |>
  separate(
    Comparison,
    into = c("Sample1", "Sample2"),
    sep = 1,
    # because each comparison has two characters
    remove = FALSE
  ) |> # keep the original comparison column
  relocate(Sample1, Sample2, .after = Comparison) # move the new columns right after Comparison

cols_to_split <-
  c("n_A",
    "n_T",
    "n_C",
    "n_G",
    "n_homo_ref",
    "n_homo_alt",
    "n_hetero")

# Remove unwanted characters from the columns
for (col_name in cols_to_split) {
  data[[col_name]] <- gsub("\\[\\[|]\\n", "", data[[col_name]])
}

# Split the columns
for (col_name in cols_to_split) {
  # Create new column names based on 'Sample1' and 'Sample2'
  new_col_names <- paste0(col_name, "_sample", 1:2)
  
  data <- data |>
    separate(
      col = col_name,
      into = new_col_names,
      sep = " ",
      extra = "drop"
    )
}

# Clean the new columns
cols_to_clean <- 
  grep("^n_", names(data), value = TRUE)

for (col_name in cols_to_clean) {
  # Remove unwanted characters '[', ']', and '\n'
  data[[col_name]] <- gsub("\\[|]|\\n", "", data[[col_name]])
}

# Split the column names into "Sample" and numeric value
data <- 
  data |>
  separate(
    col = Comparison,
    into = c("Sample1", "Sample2"),
    sep = 1,
    remove = FALSE
  ) |>
  relocate(Sample1, Sample2, .after = Comparison)

# Convert columns to numeric
# Specify the column names to convert to numeric
columns_to_convert <-
  c(
    # "Population",
    "Sample",
    # "Comparison",
    # "Sample1",
    # "Sample2",
    "n_SNPs",
    "n_SNPs_ref_mismatch",
    "n_SNPs_alt_mismatch",
    "n_A_sample1",
    "n_A_sample2",
    "n_T_sample1",
    "n_T_sample2",
    "n_C_sample1",
    "n_C_sample2",
    "n_G_sample1",
    "n_G_sample2",
    "n_homo_ref_sample1",
    "n_homo_ref_sample2",
    "n_homo_alt_sample1",
    "n_homo_alt_sample2",
    "n_hetero_sample1",
    "n_hetero_sample2",
    "n_homo_ref_mismatch",
    "n_homo_alt_mismatch",
    "n_hetero_mismatch"
  )

# Convert columns to numeric
data[columns_to_convert] <-
  lapply(data[columns_to_convert], function(x)
    as.numeric(as.character(x)))

# Verify the column types
print(sapply(data[columns_to_convert], class))
```

```
##              Sample              n_SNPs n_SNPs_ref_mismatch n_SNPs_alt_mismatch 
##           "numeric"           "numeric"           "numeric"           "numeric" 
##         n_A_sample1         n_A_sample2         n_T_sample1         n_T_sample2 
##           "numeric"           "numeric"           "numeric"           "numeric" 
##         n_C_sample1         n_C_sample2         n_G_sample1         n_G_sample2 
##           "numeric"           "numeric"           "numeric"           "numeric" 
##  n_homo_ref_sample1  n_homo_ref_sample2  n_homo_alt_sample1  n_homo_alt_sample2 
##           "numeric"           "numeric"           "numeric"           "numeric" 
##    n_hetero_sample1    n_hetero_sample2 n_homo_ref_mismatch n_homo_alt_mismatch 
##           "numeric"           "numeric"           "numeric"           "numeric" 
##   n_hetero_mismatch 
##           "numeric"
```

Now we can subset the data to have more meaningful comparisons and
visualizations.

Check the output

```
head(data)
```

```
## # A tibble: 6 × 25
##   Population Sample Comparison Sample1 Sample2 n_SNPs n_SNPs_ref_mismatch
##   <chr>       <dbl> <chr>      <chr>   <chr>    <dbl>               <dbl>
## 1 BEN             3 ab         a       b       101645                 624
## 2 KAG             2 ab         a       b       104078                 145
## 3 BAR             4 ab         a       b       102924                 207
## 4 LOS             5 ab         a       b       103430                 127
## 5 BEN             3 ac         a       c       102831                 301
## 6 KAG             2 ac         a       c       104025                 169
## # ℹ 18 more variables: n_SNPs_alt_mismatch <dbl>, n_A_sample1 <dbl>,
## #   n_A_sample2 <dbl>, n_T_sample1 <dbl>, n_T_sample2 <dbl>, n_C_sample1 <dbl>,
## #   n_C_sample2 <dbl>, n_G_sample1 <dbl>, n_G_sample2 <dbl>,
## #   n_homo_ref_sample1 <dbl>, n_homo_ref_sample2 <dbl>,
## #   n_homo_alt_sample1 <dbl>, n_homo_alt_sample2 <dbl>, n_hetero_sample1 <dbl>,
## #   n_hetero_sample2 <dbl>, n_homo_ref_mismatch <dbl>,
## #   n_homo_alt_mismatch <dbl>, n_hetero_mismatch <dbl>
```

Check the column names

```
colnames(data)
```

```
##  [1] "Population"          "Sample"              "Comparison"         
##  [4] "Sample1"             "Sample2"             "n_SNPs"             
##  [7] "n_SNPs_ref_mismatch" "n_SNPs_alt_mismatch" "n_A_sample1"        
## [10] "n_A_sample2"         "n_T_sample1"         "n_T_sample2"        
## [13] "n_C_sample1"         "n_C_sample2"         "n_G_sample1"        
## [16] "n_G_sample2"         "n_homo_ref_sample1"  "n_homo_ref_sample2" 
## [19] "n_homo_alt_sample1"  "n_homo_alt_sample2"  "n_hetero_sample1"   
## [22] "n_hetero_sample2"    "n_homo_ref_mismatch" "n_homo_alt_mismatch"
## [25] "n_hetero_mismatch"
```

We can select the columns that we want to compare

```
# Select the specified columns
selected_data <- 
  data |>
  dplyr::select(Population, Sample, Comparison, n_SNPs, n_homo_ref_mismatch, n_homo_alt_mismatch, n_hetero_mismatch)

# Print the selected_data
print(selected_data)
```

```
## # A tibble: 12 × 7
##    Population Sample Comparison n_SNPs n_homo_ref_mismatch n_homo_alt_mismatch
##    <chr>       <dbl> <chr>       <dbl>               <dbl>               <dbl>
##  1 BEN             3 ab         101645                 377                 247
##  2 KAG             2 ab         104078                  86                  59
##  3 BAR             4 ab         102924                 116                  91
##  4 LOS             5 ab         103430                  77                  50
##  5 BEN             3 ac         102831                 171                 130
##  6 KAG             2 ac         104025                  87                  82
##  7 BAR             4 ac         102772                 186                 141
##  8 LOS             5 ac         103237                 111                  61
##  9 LOS             5 bc         103201                 133                  92
## 10 BAR             4 bc         103194                  91                  74
## 11 KAG             2 bc         103989                 114                  95
## 12 BEN             3 bc         101799                 404                 223
## # ℹ 1 more variable: n_hetero_mismatch <dbl>
```

We can calculate the percentage of mismatches

```
# Compute the percentages and create new columns
selected_data <- 
  selected_data |>
  mutate(
    pct_homo_ref_mismatch = round((n_homo_ref_mismatch / n_SNPs) * 100, 2),
    pct_homo_alt_mismatch = round((n_homo_alt_mismatch / n_SNPs) * 100, 2),
    pct_hetero_mismatch = round((n_hetero_mismatch / n_SNPs) * 100, 2)
  )

# Print the modified selected_data
print(selected_data)
```

```
## # A tibble: 12 × 10
##    Population Sample Comparison n_SNPs n_homo_ref_mismatch n_homo_alt_mismatch
##    <chr>       <dbl> <chr>       <dbl>               <dbl>               <dbl>
##  1 BEN             3 ab         101645                 377                 247
##  2 KAG             2 ab         104078                  86                  59
##  3 BAR             4 ab         102924                 116                  91
##  4 LOS             5 ab         103430                  77                  50
##  5 BEN             3 ac         102831                 171                 130
##  6 KAG             2 ac         104025                  87                  82
##  7 BAR             4 ac         102772                 186                 141
##  8 LOS             5 ac         103237                 111                  61
##  9 LOS             5 bc         103201                 133                  92
## 10 BAR             4 bc         103194                  91                  74
## 11 KAG             2 bc         103989                 114                  95
## 12 BEN             3 bc         101799                 404                 223
## # ℹ 4 more variables: n_hetero_mismatch <dbl>, pct_homo_ref_mismatch <dbl>,
## #   pct_homo_alt_mismatch <dbl>, pct_hetero_mismatch <dbl>
```

Lets move the columns

```
# Reorder the columns
selected_data <- 
  selected_data |>
  dplyr::select(Population, Sample, Comparison, n_SNPs,
         n_homo_ref_mismatch, pct_homo_ref_mismatch, 
         n_homo_alt_mismatch, pct_homo_alt_mismatch, 
         n_hetero_mismatch, pct_hetero_mismatch) |>
  arrange(
    Sample, Comparison 
  )

# Print the reordered selected_data
print(selected_data)
```

```
## # A tibble: 12 × 10
##    Population Sample Comparison n_SNPs n_homo_ref_mismatch pct_homo_ref_mismatch
##    <chr>       <dbl> <chr>       <dbl>               <dbl>                 <dbl>
##  1 KAG             2 ab         104078                  86                  0.08
##  2 KAG             2 ac         104025                  87                  0.08
##  3 KAG             2 bc         103989                 114                  0.11
##  4 BEN             3 ab         101645                 377                  0.37
##  5 BEN             3 ac         102831                 171                  0.17
##  6 BEN             3 bc         101799                 404                  0.4 
##  7 BAR             4 ab         102924                 116                  0.11
##  8 BAR             4 ac         102772                 186                  0.18
##  9 BAR             4 bc         103194                  91                  0.09
## 10 LOS             5 ab         103430                  77                  0.07
## 11 LOS             5 ac         103237                 111                  0.11
## 12 LOS             5 bc         103201                 133                  0.13
## # ℹ 4 more variables: n_homo_alt_mismatch <dbl>, pct_homo_alt_mismatch <dbl>,
## #   n_hetero_mismatch <dbl>, pct_hetero_mismatch <dbl>
```

### 3.4 Calculate total error rate per population

We can add up the mismatches to get a total mismatch rate

```
# Compute the total mismatches and percentage of total mismatches
selected_data <- 
  selected_data |>
  mutate(
    total_mismatches = n_homo_ref_mismatch + n_homo_alt_mismatch + n_hetero_mismatch,
    pct_total_mismatches = round((total_mismatches / n_SNPs) * 100, 2)
  )

# Print the modified selected_data
print(selected_data)
```

```
## # A tibble: 12 × 12
##    Population Sample Comparison n_SNPs n_homo_ref_mismatch pct_homo_ref_mismatch
##    <chr>       <dbl> <chr>       <dbl>               <dbl>                 <dbl>
##  1 KAG             2 ab         104078                  86                  0.08
##  2 KAG             2 ac         104025                  87                  0.08
##  3 KAG             2 bc         103989                 114                  0.11
##  4 BEN             3 ab         101645                 377                  0.37
##  5 BEN             3 ac         102831                 171                  0.17
##  6 BEN             3 bc         101799                 404                  0.4 
##  7 BAR             4 ab         102924                 116                  0.11
##  8 BAR             4 ac         102772                 186                  0.18
##  9 BAR             4 bc         103194                  91                  0.09
## 10 LOS             5 ab         103430                  77                  0.07
## 11 LOS             5 ac         103237                 111                  0.11
## 12 LOS             5 bc         103201                 133                  0.13
## # ℹ 6 more variables: n_homo_alt_mismatch <dbl>, pct_homo_alt_mismatch <dbl>,
## #   n_hetero_mismatch <dbl>, pct_hetero_mismatch <dbl>, total_mismatches <dbl>,
## #   pct_total_mismatches <dbl>
```

We can now calculate the mean percentage of mismatches for each
population

```
# Group by Population and compute the mean of pct_total_mismatches
summary_data <- selected_data |>
  group_by(Population) |>
  summarise(mean_pct_total_mismatches = round(mean(pct_total_mismatches), 2))

# Create the flextable
ft <- flextable::flextable(summary_data)

# Apply zebra theme
ft <- flextable::theme_zebra(ft)

# Add a caption to the table
ft <- flextable::add_header_lines(ft, "Table 1: Mean error rate between the 3 replicates for each population")

# Save it to a Word document
officer::read_docx() |>
  body_add_flextable(ft) |>
  print(target = here::here("output", "populations", "triplicates", "summary_error_rate_per_population.docx"))

ft
```

| Table 1: Mean error rate between the 3 replicates for each population | |
| --- | --- |
| Population | mean\_pct\_total\_mismatches |
| BAR | 0.45 |
| BEN | 1.02 |
| KAG | 0.33 |
| LOS | 0.34 |

We observe low mismatch rate for most of the populations. Only BEN
from India has an error rate of 1.02% while the other 3 populations have
an error rate less than 0.5%
