# Supplementary material for: A genotyping array for the globally invasive vector mosquito, Aedes albopictus
Source: Parasit Vectors. 2024 Mar 4;17:106. doi: 10.1186/s13071-024-06158-z (PMC10910840; doi:10.1186/s13071-024-06158-z)
Supplement: Supplementary file 6 — Additional file 6. Functional annotation of SNPs and chip bias evaluation. [file 13071_2024_6158_MOESM6_ESM.html]

Aedes albopictus SNP chip - Functional annotation of SNPs using SnpEff


# Aedes albopictus SNP chip - Functional annotation of SNPs using SnpEff

#### Luciano V Cosme

#### 2023-09-21

- 1. Load
  the libraries
- 2. Download
  SnpEff
  - 2.1
    SnpEff on the cluster
- 3. Use
  SnpEff laptop
- 4. Perform variant annotation
  - 4.1 Prepare
    data
  - 4.2 Run
    annotation
  - 4.3 Parsing vcf instead
    of using bash loops
- 5.
  Getting SNPs and their effects on genes relevant for mosquito life
  history traits
  - 5.1 Diapause
    genes
  - 5.2 Immunity
    Genes
  - 5.3 Summary of
    SNPs on diapause and immunity genes
- 6. WGS variants functional
  annotation
- 7. Compare WGS and Chip
  variants by effect
- 8. Create
  pipeline to select random sets of SNPs
- 9. Create
  new files following WGS SNP effect proportions

## 1. Load the libraries

```
library(tidyverse)
library(here)
library(colorout)
library(dplyr)
library(flextable)
library(ggstatsplot)
library(ggplot2)
library(scales)
library(reticulate)
library(extrafont)
library(stringr)
library(flextable)
library(officer)
library(vroom)
library(gt)
library(ggrepel)
library(janitor)
```

## 2. Download SnpEff

We will used SnpEff to perform a functional annotation of our SNPs
You can download the software HERE. They have great
documentation that it is worth checking it HERE.

```
# move to the directory you want to download it
# Download and install SnpEff
curl -v -L 'https://snpeff.blob.core.windows.net/versions/snpEff_latest_core.zip' > snpEff_latest_core.zip
unzip snpEff_latest_core.zip
```

### 2.1 SnpEff on the cluster

We have two options to compare the functional annotation of data
sets. We can do the annotation on each data set and parse the results
with bash or R. A different approach would be run one annotation once,
and then remove those that do not pass our MAF threshold from the
annotated data set.

We also want to do LD pruning to remove close linked loci. We can do
the LD filtering before or after the functional annotation. Perhaps, we
want to compare the effect of LD pruning on the variant numbers by
effect or region. It seems possible that pruning might effect more
variants on coding regions, whose minor allele frequencies might be
low.

I decided to do the filtering (LD and MAF) before the functional
annotation, avoiding any bias based on the SNP location or it biological
implications. Therefore, the filtering will be based solely on the SNP
frequencies and locations. Previous analysis of linkage disequilibrium
in Ae. albopictus showed considerable less linkage than in Ae aegypti.
While in Ae. aegypti there are long linkage blocks (1/2 dist maximum r2
between a pair of SNPs vary from 40kb up to 120kb in aegypti),
albopictus have shorter blocks (1/2 dist maximum r2 between a pair of
SNPs vary from less than 1 to 2kb). However, using a chromosomal scale,
we found similar linkage patterns in Ae. albopictus.

While WGS LD pruning using number of SNPs is the usual choice, the
chip data needs a different approach. For example, we can we look at 50
SNPs, estimate r2 among all SNP pairs, and remove one SNP from pairs
where the estimate is above 0.1 (remove the SNP whose minor allele
frequency is smaller), and then move the window one SNP, and repeat the
operation.

This is the case in Plink when we use the flag –indep-pairwise 50 1
0.1 (it is a bit more complicated than I explained, check to learn about
VIF https://www.cog-genomics.org/plink/2.0/ld or https://www.cog-genomics.org/plink/1.9/ld).

For the chip data, we have long stretches of the genome without any
data. If we compare 50 SNPs they can come from difference genomic
regions, the average distance of SNPs on the chip is a bit above 10kb,
however it is variable. Therefore, if we use 20kb and maximum distance
we want to compare the SNPs, we will be conservative since there is low
linkage among variants. We can also test different linkage filterings
and their effect on the data sets we end up with.

To do all these tests, it is easier to have access to a cluster. The
main problem turns out to be storage for all the different data sets.
Therefore, we can install SnpEff on the cluster.

```
# Go to home dir
cd

# Download latest version
wget https://snpeff.blob.core.windows.net/versions/snpEff_latest_core.zip

# Unzip file
unzip snpEff_latest_core.zip

# pwd
# /home/lvc26/snpEff
```

```
# on the cluster
srun --pty -N 1 -n 1 -c 2 -p interactive zsh
module load miniconda/4.12.0
# check conda
which conda
/gpfs/ycga/apps/hpc/software/miniconda/4.12.0/condabin/conda
#
# install GFF3Toolkit https://github.com/NAL-i5K/GFF3toolkit
# on the base env using --user
pip install gff3tool --user
# 
#
# QC the annotation from current and previous assembly
#   ____________________________________________________________________________
#   Boyle et al, 2021                                                       ####
#
# To run the QC on the annotation we can try different strategies:
# 1. use the gff file as it is
# 2. sort by scaffold and physical location
# 3. sort by transcript id. 
# I am not sure which one would be the recommended option, therefore I will test all 3. 
# GFF3toolkit has a couple options https://github.com/NAL-i5K/GFF3toolkit#sort-a-gff3-file-back
# https://gff3toolkit.readthedocs.io/en/latest/gff3_sort.html#inputs
#
# we need to decompress the gff file and rename it to use it with snpEff later
cd $HOME/snpEff/data/albo_v3;
zcat aedes_albopictus_LA2_20200826.gff3.gz > genes.gff
#
##  ............................................................................
##  1. use gff as it is                                                     ####
# run the qc to get errors
logsave -a $HOME/snpEff/data/albo_v3/log_1.txt gff3_QC \
-g $HOME/snpEff/data/albo_v3/genes.gff \
-f $HOME/snpEff/data/genomes/albo_v3.fa \
-o $HOME/snpEff/data/albo_v3/error_1.txt \
-s $HOME/snpEff/data/albo_v3/statistic_1.txt
#
# run the qc to get errors but use flag -noncg –noncanonical_gene
# check https://www.uniprot.org/help/canonical_and_isoforms to see what it means
logsave -a $HOME/snpEff/data/albo_v3/log_1nc.txt gff3_QC \
-noncg \
-g $HOME/snpEff/data/albo_v3/genes.gff \
-f $HOME/snpEff/data/genomes/albo_v3.fa \
-o $HOME/snpEff/data/albo_v3/error_1nc.txt \
-s $HOME/snpEff/data/albo_v3/statistic_1nc.txt
#
#
##  ............................................................................
##  2. sort gff by scaffolds and positions                                  ####
#
# Sort a GFF3 file according to the order of Scaffold, coordinates on a Scaffold, and parent-child feature relationships
# check https://github.com/NAL-i5K/GFF3toolkit/blob/master/docs/gff3_sort.md
# we can also use a template to sort by feature
echo "gene pseudogene
mRNA
exon
CDS" > $HOME/snpEff/data/albo_v3/template.txt
# Note:
# If not all the feature type are documented in the sort template file. gff3_sort will sort features by level(1st-level, 2nd-level, and etc) and then by the order in sort template file.
# 
# sort
logsave -a $HOME/snpEff/data/albo_v3/log_sort.txt gff3_sort \
--sort_template $HOME/snpEff/data/albo_v3/template.txt \
-g $HOME/snpEff/data/albo_v3/genes.gff \
-og $HOME/snpEff/data/albo_v3/sorted_genes.gff
#
# now we can run the QC again on the sorted gff file
logsave -a $HOME/snpEff/data/albo_v3/log_2.txt gff3_QC \
-g $HOME/snpEff/data/albo_v3/sorted_genes.gff \
-f $HOME/snpEff/data/genomes/albo_v3.fa \
-o $HOME/snpEff/data/albo_v3/error_2.txt \
-s $HOME/snpEff/data/albo_v3/statistic_2.txt
#
##  ............................................................................
##  3. sort gff by transcript                                               ####
#
# sort gff3 file with --isoform_sort
logsave -a $HOME/snpEff/data/albo_v3/log_sort_isoform.txt gff3_sort \
--isoform_sort \
--sort_template $HOME/snpEff/data/albo_v3/template.txt \
-g $HOME/snpEff/data/albo_v3/genes.gff \
-og $HOME/snpEff/data/albo_v3/isoform_sorted_genes.gff
#
# now we can run the QC again on the sorted gff file
logsave -a $HOME/snpEff/data/albo_v3/log_3.txt gff3_QC \
-g $HOME/snpEff/data/albo_v3/isoform_sorted_genes.gff \
-f $HOME/snpEff/data/genomes/albo_v3.fa \
-o $HOME/snpEff/data/albo_v3/error_3.txt \
-s $HOME/snpEff/data/albo_v3/statistic_3.txt
#
# run the qc to get errors
gff3_QC \
-g $HOME/snpEff/data/albo_v3/genes_sorted.gff3 \
-f $HOME/snpEff/data/genomes/albo_v3.fa \
-o $HOME/snpEff/data/albo_v3/error.txt \
-s $HOME/snpEff/data/albo_v3/statistic.txt
#
# when sorting it creates another error - duplicated ids
# therefore sorting does not seem to solve the errors that cannot be fixed automatically
#
```

```
#   ____________________________________________________________________________
#   AalboF2 - Palinni et al, 2020                                           ####
#
# genome at https://ftp.ncbi.nlm.nih.gov/genomes/all/annotation_releases/7160/102/GCF_006496715.1_Aalbo_primary.1/
#
# decompress the fasta file and name it as albo_v2
zcat GCF_006496715.1_Aalbo_primary.1_genomic.fna.gz > albo_v2.fa
#
# index the genome
module load SAMtools/1.16-GCCcore-10.2.0
samtools faidx $HOME/snpEff/data/genomes/albo_v2.fa
#
# decompress gff file, name it as genes.gff, and place it in a directory called albo_v2 inside snpEff directory
mkdir $HOME/snpEff/data/genomes/albo_v2;
zcat GCF_006496715.1_Aalbo_primary.1_genomic.gff.gz > genes.gff
#
# create interactive session with 4CPU and 20GB memory (10 was not enough)
srun --pty -N 1 -n 1 -c 4 -p interactive zsh
#
# load GATK, it will automatic load the python I installed gfftoolkit
module load GATK/4.2.6.1-GCCcore-10.2.0-Java-11
#
##  ............................................................................
##  1. use gff as it is                                                     ####
# run the qc to get errors
logsave -a $HOME/snpEff/data/albo_v2/log_1.txt gff3_QC \
-g $HOME/snpEff/data/albo_v2/genes.gff \
-f $HOME/snpEff/data/genomes/albo_v2.fa \
-o $HOME/snpEff/data/albo_v2/error_1.txt \
-s $HOME/snpEff/data/albo_v2/statistic_1.txt
#
# with flag non canonical
logsave -a $HOME/snpEff/data/albo_v2/log_1nc.txt gff3_QC \
-noncg \
-g $HOME/snpEff/data/albo_v2/genes.gff \
-f $HOME/snpEff/data/genomes/albo_v2.fa \
-o $HOME/snpEff/data/albo_v2/error_1nc.txt \
-s $HOME/snpEff/data/albo_v2/statistic_1nc.txt
#
#
# lets sort it as well to see if there is a difference
# it seems some genes are missing some features, but those genes might be non coding genes
# now we can run the QC again on the sorted gff file
# 
# sort
logsave -a $HOME/snpEff/data/albo_v2/log_sort.txt gff3_sort \
--sort_template $HOME/snpEff/data/albo_v3/template.txt \
-g $HOME/snpEff/data/albo_v2/genes.gff \
-og $HOME/snpEff/data/albo_v2/sorted_genes.gff
#
# now we can run the QC again on the sorted gff file
logsave -a $HOME/snpEff/data/albo_v2/log_2.txt gff3_QC \
-g $HOME/snpEff/data/albo_v2/sorted_genes.gff \
-f $HOME/snpEff/data/genomes/albo_v2.fa \
-o $HOME/snpEff/data/albo_v2/error_2.txt \
-s $HOME/snpEff/data/albo_v2/statistic_2.txt
#
# sorting also does not help resolving some of the errors, as it happens to our current assembly.
```

We can try to fix some of the errors

```
# check documentation at https://github.com/NAL-i5K/GFF3toolkit/blob/master/docs/gff3_fix.py-documentation.md
# only 30 types of errors can be corrected but there are 22 errors that there is no automatic correction
#
##  ............................................................................
##  1. use gff as it is                                                     ####
#
# run gff3_fix
logsave -a $HOME/snpEff/data/albo_v3/log_fix_1.txt gff3_fix \
-qc_r $HOME/snpEff/data/albo_v3/error_1.txt \
-g $HOME/snpEff/data/albo_v3/genes.gff \
-og $HOME/snpEff/data/albo_v3/corrected_genes.gff
# no luck here it throws an error
# UnboundLocalError: local variable 'CDS_list' referenced before assignment
# gff3_fix died with exit status 1
# if you check the type of errors, you can see that some errors cannot be fixed automatically
# list of errors that can or cannot be fixed at https://github.com/NAL-i5K/GFF3toolkit/blob/master/docs/gff3_fix.py-documentation.md
head -n 100 statistic_1.txt
# Error_code    Number_of_problematic_models    Error_level Error_tag
# Esf0014   1   Error   ##gff-version" missing from the first line
# Esf0036   1060    Error   Value of a attribute contains unescaped ","
# Ema0006   26  Info    Wrong phase
# Ema0007   110 Warning CDS and parent feature on different strands
# Ema0004   5079    Info    Incomplete gene feature that should contain at least one mRNA, exon, and CDS
# Ema0005   1739    Info    Pseudogene has invalid child feature type
# Ema0009   1957    Warning Incorrectly merged gene parent? Isoforms that do not share coding sequences are found
# Ema0002   118 Warning Protein sequence contains internal stop codons
# Ema0008   17  Warning for distinct isoforms that do not share any regions
# Emr0002   39  Warning Incorrectly split gene parent?
#
# using the non canonical option
logsave -a $HOME/snpEff/data/albo_v3/log_fix_1nc.txt gff3_fix \
-qc_r $HOME/snpEff/data/albo_v3/error_1nc.txt \
-g $HOME/snpEff/data/albo_v3/genes.gff \
-og $HOME/snpEff/data/albo_v3/corrected_genes_nc.gff
# then it works
# if you check the type of errors it had
head -n 100 statistic_1nc.txt
# Error_code    Number_of_problematic_models    Error_level Error_tag
# Esf0014   1   Error   ##gff-version" missing from the first line
# Esf0036   1060    Error   Value of a attribute contains unescaped ","
# Ema0005   1739    Info    Pseudogene has invalid child feature type
# 
# therefore we are able to fix these errors. The other "errors" is because the annotation was not curated,
# we do not have data supporting all gene models, exons and introns boundaries. For example,
# we can have exons of a gene that are actually 2, but the intron was not identified. We also have
# exons without parent feature - the software expect exons to be in order by transcript. If we have
# interspaced exons of transcripts, it results in "orphan" exons. It did not follow what our gene 
# models expected
# we can use this version to build the snpEff database
#
# we can try the sorted files
##  ............................................................................
##  2. sort gff by scaffolds and positions                                  ####
#
logsave -a $HOME/snpEff/data/albo_v3/log_fix_2.txt gff3_fix \
-qc_r $HOME/snpEff/data/albo_v3/error_2.txt \
-g $HOME/snpEff/data/albo_v3/sorted_genes.gff \
-og $HOME/snpEff/data/albo_v3/corrected_sorted_genes.gff
# we get a different error
# ValueError: max() arg is an empty sequence
# gff3_fix died with exit status 1
#
##  ............................................................................
##  3. sort gff by transcript                                               ####
#
logsave -a $HOME/snpEff/data/albo_v3/log_fix_3.txt gff3_fix \
-qc_r $HOME/snpEff/data/albo_v3/error_3.txt \
-g $HOME/snpEff/data/albo_v3/isoform_sorted_genes.gff \
-og $HOME/snpEff/data/albo_v3/corrected_isoform_sorted_genes.gff
# ufortunately we get the error again
# ValueError: max() arg is an empty sequence
# gff3_fix died with exit status 1
#
### . . . . . . . . . . . . . . . . . . . . . . . . . . . . . . . . . . . . . ..
### count errors from log files                                             ####
#
# original
grep -c 'There are no CDSs' log_1.txt
# 12677
grep -c 'Parent feature missing' log_1.txt
# 11560 - we have 11,560 orphan exons
# sorted by scaffold following template
grep -c 'There are no CDSs' log_2.txt
# 12677
grep -c 'Parent feature missing' log_2.txt
# 0
# sorted by isoform following template
grep -c 'There are no CDSs' log_3.txt
# 12677
grep -c 'Parent feature missing' log_3.txt
# 0
# sorting removes the orphan exons but we still have 12,677 genes with incomplete or missing CDS
# sorting is not having the impact I expected.
#
```

We can check the Ensembl annotation files for the first genome
assembly of Ae. albopictus and check the latest annotation of Aedes
aegypti genome and compare it to the Ae. albopictus

```
# we can check the data in Ensembl, they curated their annotations very well.
# check the paper about annotations, what a canonical transcript is and they find it https://www.ncbi.nlm.nih.gov/pmc/articles/PMC4919035/pdf/baw093.pdf
#
# we have aegypti and albopictus genome on ensembl. The albopictus genome is AaloF1
https://metazoa.ensembl.org/info/data/ftp/index.html
#
# aegypti
mkdir $HOME/snpEff/data/aegypti_v5
# decompress gff
zcat Aedes_aegypti_lvpagwg.AaegL5.55.gff3.gz > genes.gff
# decompress genome and index it
zcat Aedes_aegypti_lvpagwg.AaegL5.dna.toplevel.fa.gz > aegypti_v5.fa
samtools faidx $HOME/snpEff/data/genomes/aegypti_v5.fa
#
# Ensembl albopictus AaloF1 - first genome assembly Chen et al, 2015 - 154,782 scaffolds
zcat Aedes_albopictus.AaloF1.dna.toplevel.fa.gz > AaloF1.fa
samtools faidx $HOME/snpEff/data/genomes/AaloF1.fa
zcat Aedes_albopictus.AaloF1.55.gff3.gz > genes.gff
```

Run QC on Ensembl annotations

```
# we will not try to sort the files since it creates other artifacts
#
# Ae. aegypti
#
##  ............................................................................
##  1. use gff as it is                                                     ####
# run the qc to get errors
logsave -a $HOME/snpEff/data/aegypti_v5/log_1.txt gff3_QC \
-g $HOME/snpEff/data/aegypti_v5/genes.gff \
-f $HOME/snpEff/data/genomes/aegypti_v5.fa \
-o $HOME/snpEff/data/aegypti_v5/error_1.txt \
-s $HOME/snpEff/data/aegypti_v5/statistic_1.txt
#
# with flag non canonical
logsave -a $HOME/snpEff/data/aegypti_v5/log_1nc.txt gff3_QC \
-noncg \
-g $HOME/snpEff/data/aegypti_v5/genes.gff \
-f $HOME/snpEff/data/genomes/aegypti_v5.fa \
-o $HOME/snpEff/data/aegypti_v5/error_1nc.txt \
-s $HOME/snpEff/data/aegypti_v5/statistic_1nc.txt
#
# AaloF1
logsave -a $HOME/snpEff/data/AaloF1/log_1.txt gff3_QC \
-g $HOME/snpEff/data/AaloF1/genes.gff \
-f $HOME/snpEff/data/genomes/AaloF1.fa \
-o $HOME/snpEff/data/AaloF1/error_1.txt \
-s $HOME/snpEff/data/AaloF1/statistic_1.txt
#
# with flag non canonical
logsave -a $HOME/snpEff/data/AaloF1/log_1nc.txt gff3_QC \
-noncg \
-g $HOME/snpEff/data/AaloF1/genes.gff \
-f $HOME/snpEff/data/genomes/AaloF1.fa \
-o $HOME/snpEff/data/AaloF1/error_1nc.txt \
-s $HOME/snpEff/data/AaloF1/statistic_1nc.txt
```

We can parse the data from the QC we did with the different
genomes

```
# create dir and transfer the data from cluster to it
mkdir -p output/SnpEff/gff_qc/{albo_v1,albo_v2,albo_v3,aegypti_v5}
```

Import data into R

```
# I also included the logs and the error descriptions in the output directory for each genome assembly
# import data
##  ............................................................................
##  aegypti_v5                                                              ####
#
aegypti_v5_c <-  # using canonical gene models
  read_delim(
    here(
      "output", "SnpEff", "gff_qc", "aegypti_v5", "statistic_1.txt"
    ),
    col_names      = TRUE,
    show_col_types = FALSE,
    col_types      = "cnccc"
  ) |>
  mutate(        # create column with info about assembly and flags
    assembly       = "AaegL5",
    model          = "Canonical"
  )
#
aegypti_v5_nc <- # using non canonical gene models
  read_delim(
    here(
      "output", "SnpEff", "gff_qc", "aegypti_v5", "statistic_1nc.txt"
    ),
    col_names      = TRUE,
    show_col_types = FALSE,
    col_types      = "cnccc"
  ) |>
mutate(
  assembly       = "AaegL5",
  model          = "Non-canonical"
)
#
##  ............................................................................
##  albo_v1                                                                 ####
#
albo_v1_c <-     # using canonical gene models
  read_delim(
    here(
      "output", "SnpEff", "gff_qc", "albo_v1", "statistic_1.txt"
    ),
    col_names      = TRUE,
    show_col_types = FALSE,
    col_types      = "cnccc"
  ) |>
  mutate(
    assembly       = "AalbF1",
    model          = "Canonical"
  )
#
albo_v1_nc <-    # using non canonical gene models
  read_delim(
    here(
      "output", "SnpEff", "gff_qc", "albo_v1", "statistic_1nc.txt"
    ),
    col_names      = TRUE,
    show_col_types = FALSE,
    col_types      = "cnccc"
  ) |>
  mutate(
    assembly       = "AalbF1",
    model          = "Non-canonical"
  )
#
##  ............................................................................
##  albo_v2                                                                 ####
#
albo_v2_c <-     # using canonical gene models
  read_delim(
    here(
      "output", "SnpEff", "gff_qc", "albo_v2", "statistic_1.txt"
    ),
    col_names      = TRUE,
    show_col_types = FALSE,
    col_types      = "cnccc"
  ) |>
  mutate(
    assembly       = "AalbF2",
    model          = "Canonical"
  )
#
albo_v2_nc <-    # using non canonical gene models
  read_delim(
    here(
      "output", "SnpEff", "gff_qc", "albo_v2", "statistic_1nc.txt"
    ),
    col_names      = TRUE,
    show_col_types = FALSE,
    col_types      = "cnccc"
  ) |>
  mutate(
    assembly       = "AalbF2",
    model          = "Non-canonical"
  )
#
##  ............................................................................
##  albo_v3                                                                 ####
#
albo_v3_c <-     # using canonical gene models
  read_delim(
    here(
      "output", "SnpEff", "gff_qc", "albo_v3", "statistic_1.txt"
    ),
    col_names      = TRUE,
    show_col_types = FALSE,
    col_types      = "cnccc"
  ) |>
  mutate(
    assembly       = "AalbF3",
    model          = "Canonical"
  )
#
albo_v3_nc <-    # using non canonical gene models
  read_delim(
    here(
      "output", "SnpEff", "gff_qc", "albo_v3", "statistic_1nc.txt"
    ),
    col_names      = TRUE,
    show_col_types = FALSE,
    col_types      = "cnccc"
  ) |>
  mutate(
    assembly       = "AalbF3",
    model          = "Non-canonical"
  )
#
```

Merge the data sets and calculate totals

```
# row bind the tibbles
gff_qc <-
  bind_rows(
    list(
      aegypti_v5_c, aegypti_v5_nc, albo_v1_c, albo_v1_nc, albo_v2_c, albo_v2_nc, albo_v3_c, albo_v3_nc
    )
  ) 
#
# calculate total for each group
gff_qc2 <-
  gff_qc |>
  group_by(
    assembly, model
  ) |>
  group_split() |>
  purrr::map_df(
    janitor::adorn_totals
  )
#
# summary errors
gff_qc3 <-
  gff_qc |>
  group_by(assembly,model) |>
  summarise(Total = sum(Number_of_problematic_models), .groups = "keep") # |>
  # knitr::kable()
```

Create table with gff QC data

```
#
# make table
ftab_gff <- flextable(
  gff_qc2 |>
    rename(
      "Error code"                     = 1,
      "Number of problematic models"   = 2,
      "Error level"                    = 3,
      "Error tag"                      = 4,
      Assembly                         = 5,
      Model                            = 6
    ) |>
    select( # change the order of the columns by index
      c(
        5, 1, 6, 2, 3, 4
      )
    )
) |>
  set_caption(
    as_paragraph(
      as_chunk(
        "Errors detected in genome annotations files according to the gene model by GFF3ToolKit",
        props                          = fp_text_default(
          font.family                  = "Cambria"
        )
      )
    ),
    word_stylename                     = "Table Caption"
  )
#
# check it
ftab_gff |>
  autofit()
```

Errors detected in genome annotations files according to the gene model by GFF3ToolKit

| Assembly | Error code | Model | Number of problematic models | Error level | Error tag |
| --- | --- | --- | --- | --- | --- |
| AaegL5 | Ema0009 | Canonical | 447 | Warning | Incorrectly merged gene parent? Isoforms that do not share coding sequences are found |
| AaegL5 | Ema0004 | Canonical | 366 | Info | Incomplete gene feature that should contain at least one mRNA, exon, and CDS |
| AaegL5 | Ema0008 | Canonical | 28 | Warning | Warning for distinct isoforms that do not share any regions |
| AaegL5 | Ema0002 | Canonical | 91 | Warning | Protein sequence contains internal stop codons |
| AaegL5 | Esf0003 | Canonical | 2,310 | Error | Strand information missing |
| - | Total | - | 3,242 | - | - |
| AaegL5 | Esf0003 | Non-canonical | 2,310 | Error | Strand information missing |
| - | Total | - | 2,310 | - | - |
| AalbF1 | Esf0012 | Canonical | 82 | Info | Found Ns in a feature using the external FASTA |
| AalbF1 | Esf0003 | Canonical | 154,782 | Error | Strand information missing |
| - | Total | - | 154,864 | - | - |
| AalbF1 | Esf0012 | Non-canonical | 82 | Info | Found Ns in a feature using the external FASTA |
| AalbF1 | Esf0003 | Non-canonical | 154,782 | Error | Strand information missing |
| - | Total | - | 154,864 | - | - |
| AalbF2 | Esf0036 | Canonical | 2,234 | Error | Value of a attribute contains unescaped "," |
| AalbF2 | Ema0004 | Canonical | 9,530 | Info | Incomplete gene feature that should contain at least one mRNA, exon, and CDS |
| AalbF2 | Ema0005 | Canonical | 3,994 | Info | Pseudogene has invalid child feature type |
| AalbF2 | Ema0009 | Canonical | 4,285 | Warning | Incorrectly merged gene parent? Isoforms that do not share coding sequences are found |
| AalbF2 | Ema0002 | Canonical | 235 | Warning | Protein sequence contains internal stop codons |
| AalbF2 | Ema0008 | Canonical | 38 | Warning | Warning for distinct isoforms that do not share any regions |
| AalbF2 | Emr0001 | Canonical | 1 | Warning | Duplicate transcript found |
| AalbF2 | Emr0002 | Canonical | 137 | Warning | Incorrectly split gene parent? |
| - | Total | - | 20,454 | - | - |
| AalbF2 | Esf0036 | Non-canonical | 2,234 | Error | Value of a attribute contains unescaped "," |
| AalbF2 | Ema0005 | Non-canonical | 3,994 | Info | Pseudogene has invalid child feature type |
| - | Total | - | 6,228 | - | - |
| AalbF3 | Esf0014 | Canonical | 1 | Error | ##gff-version" missing from the first line |
| AalbF3 | Esf0036 | Canonical | 1,060 | Error | Value of a attribute contains unescaped "," |
| AalbF3 | Ema0006 | Canonical | 26 | Info | Wrong phase |
| AalbF3 | Ema0007 | Canonical | 110 | Warning | CDS and parent feature on different strands |
| AalbF3 | Ema0004 | Canonical | 5,079 | Info | Incomplete gene feature that should contain at least one mRNA, exon, and CDS |
| AalbF3 | Ema0005 | Canonical | 1,739 | Info | Pseudogene has invalid child feature type |
| AalbF3 | Ema0009 | Canonical | 1,957 | Warning | Incorrectly merged gene parent? Isoforms that do not share coding sequences are found |
| AalbF3 | Ema0002 | Canonical | 118 | Warning | Protein sequence contains internal stop codons |
| AalbF3 | Ema0008 | Canonical | 17 | Warning | Warning for distinct isoforms that do not share any regions |
| AalbF3 | Emr0002 | Canonical | 39 | Warning | Incorrectly split gene parent? |
| - | Total | - | 10,146 | - | - |
| AalbF3 | Esf0014 | Non-canonical | 1 | Error | ##gff-version" missing from the first line |
| AalbF3 | Esf0036 | Non-canonical | 1,060 | Error | Value of a attribute contains unescaped "," |
| AalbF3 | Ema0005 | Non-canonical | 1,739 | Info | Pseudogene has invalid child feature type |
| - | Total | - | 2,800 | - | - |

```
# save
# set doc properties
sect_properties <-
  prop_section(
    page_size                          = page_size(
      orient                           = "landscape",
      width                            = 8.3,
      height                           = 11.7
    ),
    type                               = "continuous",
    page_margins                       = page_mar()
  )
#
# save it using the properties
ftab_gff |>
  autofit() |>
  save_as_docx(
    path                               = here(
      "output", "SnpEff", "Errors_annotations_GFF3ToolKit.docx"
    ),
    pr_section                         = sect_properties
  )
```

Summary of errors

```
# summary errors
ftab_gff2 <- flextable(
  gff_qc3 |>
    rename(
      Assembly                            = 1,
      Model                            = 2
    )
  ) |>
  set_caption(
    as_paragraph(
      as_chunk(
        "Summary of errors detected in genome annotations files by gene model using GFF3ToolKit",
        props                          = fp_text_default(
          font.family                  = "Cambria"
        )
      )
    ),
    word_stylename                     = "Table Caption"
  )
#
# check it
ftab_gff2 |>
  autofit()
```

Summary of errors detected in genome annotations files by gene model using GFF3ToolKit

| Assembly | Model | Total |
| --- | --- | --- |
| AaegL5 | Canonical | 3,242 |
| AaegL5 | Non-canonical | 2,310 |
| AalbF1 | Canonical | 154,864 |
| AalbF1 | Non-canonical | 154,864 |
| AalbF2 | Canonical | 20,454 |
| AalbF2 | Non-canonical | 6,228 |
| AalbF3 | Canonical | 10,146 |
| AalbF3 | Non-canonical | 2,800 |

```
# save
# set doc properties
sect_properties <-
  prop_section(
    page_size                          = page_size(
      orient                           = "portrait",
      width                            = 8.3,
      height                           = 11.7
    ),
    type                               = "continuous",
    page_margins                       = page_mar()
  )
#
# save it using the properties
ftab_gff2 |>
  autofit() |>
  save_as_docx(
    path                               = here(
      "output", "SnpEff", "Summary_errors_annotations_GFF3ToolKit.docx"
    ),
    pr_section                         = sect_properties
  )
```

Generate peptide sequences

```
logsave -a $HOME/snpEff/data/albo_v3/log_protein_1.txt gff3_to_fasta \
--gff $HOME/snpEff/data/albo_v3/genes.gff \
--fasta $HOME/snpEff/data/genomes/albo_v3.fa \
--sequence_type pep \
--defline simple \
--output_prefix $HOME/snpEff/data/albo_v3/albo
# it will create a peptide file names albo_pep.fa, we need to change the name of the file for snpEff database building
mv $HOME/snpEff/data/albo_v3/albo_pep.fa $HOME/snpEff/data/albo_v3/protein.fa
# check the file
head $HOME/snpEff/data/albo_v3/protein.fa
wc -l $HOME/snpEff/data/albo_v3/protein.fa
# 47990 sequences
```

Create data base for genome on the cluster

```
# we need to create a dir for the database at the SnpEff home directory
# create interactive session ( 2CPU 10Gb)
srun --pty -N 1 -n 1 -c 2 -p interactive zsh
cd /home/lvc26/snpEff;
mkdir -p data/{genomes,albo_v3} 
#
# create protein sequences from gff 
# check http://ccb.jhu.edu/software/stringtie/gff.shtml#gffread
git clone https://github.com/gpertea/gffread
cd gffread
make release
# export path
export PATH=$PATH:/gpfs/ycga/home/lvc26/gffread
#
# check gff file
# In order to see the GTF2 version of the same transcripts, the -T option should be added
gffread -E $HOME/snpEff/data/albo_v3/genes.gff -T -o- | more
# The examples above also show that gffread can be used to convert a file between GTF2 and GFF3 file formats.

# now we have to put the gff the genome file inside the directories we create the data base
# name the them accordingly, your directories show look like this
$HOME/snpEff/data/albo_v3/genes.gff
$HOME/snpEff/data/albo_v3/genes.gtf
$HOME/snpEff/data/genomes/albo_v3.fa
# 
# index the genome
module load SAMtools/1.16-GCCcore-10.2.0
samtools faidx $HOME/snpEff/data/genomes/albo_v3.fa
# 
# earlier I created a fasta file with protein sequences
$HOME/snpEff/data/albo_v3/protein.fa
#
# then run chmod on the executable file
chmod 775 snpEff.jar
#
# load gatk to automatic load java 11, I tried some versions and it did not work, saying it the version was too old
module load GATK/4.2.6.1-GCCcore-10.2.0-Java-11
#
##  ............................................................................
##  create database                                                         ####
#
# when I first run it, I used 4 CPUs and 20GB of memory, however, I got the message "java died with exit status 255"
# at the end of the database build. It means that I did not have enough memory. Fix it requesting more memory
# the command below requests 1CP with 40GB of memory for 30 minutes
salloc -t 0:30:00 --mem=40G
# it also failed
# submit batch job instead
# we can use dsq, first we create script, we can use echo and save it as file
# check snpEff options
java -Xmx2g -jar $HOME/snpEff/snpEff.jar build
# load gatk again
module load GATK/4.2.6.1-GCCcore-10.2.0-Java-11
#
# build database
logsave -a $HOME/snpEff/data/albo_v3/log_database_1.txt java \
-Xmx40g \
-jar $HOME/snpEff/snpEff.jar \
build \
-gff3 \
-v albo_v3
#
# create command file
echo "module load GATK/4.2.6.1-GCCcore-10.2.0-Java-11; java -Xmx50g -jar /home/lvc26/snpEff/snpEff.jar build -noCheckProtein -noCheckCds -gff3 -v albo_v3" > $HOME/snpEff/data/albo_v3/1.snpEff_build_database.txt
#
# load dsq
module load dSQ/1.05
# run dsq
dsq \
--job-file $HOME/snpEff/data/albo_v3/1.snpEff_build_database.txt \
--mem-per-cpu 50g \
--cpus-per-task=1 \
-t 00-00:15:00 \
--mail-type END \
--partition=scavenge \
--job-name snpEff \
--output $HOME/snpEff/data/albo_v3/dsq-jobfile-%A_%a-%N.txt \
--batch-file $HOME/snpEff/data/albo_v3/1.snpEff.sh
#
# submit it
sbatch /home/lvc26/snpEff/data/albo_v3/1.snpEff.sh
# autopsy
dsqa -j 19329159
# completed and database is ready for use.
# I checked the email of the job and it only used less than 2GB of memory, so it was failing because of the database checks that failed.
# then next time we need to use it check help
java -Xmx2g -jar $HOME/snpEff/snpEff.jar
#
# to submit a batch job it is probably to load gatk to load a recent java version, and then use snpEff. It will probably be easier to submit an array job with all diffent parameters.
module load GATK/4.2.6.1-GCCcore-10.2.0-Java-11
java -Xmx8g -jar $HOME/snpEff/snpEff.jar
```

## 3. Use SnpEff laptop

Our first step is to create a database using the AalbF3 using the
annotation file (gff format). There is one database for Aedes albopictus
on the SnpEff databases, but it is for the AalbF1. We cannot use it.

```
# lets check our annotation file
head -n 2 output/files/genes.gff
```

```
## chr3.9   Gnomon  gene    10376   142706  .   -   .   ID=gene-LOC109400916;Dbxref=GeneID:109400916;Name=LOC109400916;gbkey=Gene;gene=LOC109400916;gene_biotype=protein_coding;partial=true;start_range=.,10376
## chr3.9   Gnomon  mRNA    10376   142706  .   -   .   ID=rna-XM_029851713.1;Parent=gene-LOC109400916;Dbxref=GeneID:109400916,Genbank:XM_029851713.1;Name=XM_029851713.1;gbkey=mRNA;gene=LOC109400916;model_evidence=Supporting evidence includes similarity to: 1 Protein%2C and 97%25 coverage of the annotated genomic feature by RNAseq alignments%2C including 57 samples with support for all annotated introns;partial=true;product=UPF0565 protein C2orf69 homolog;start_range=.,10376;transcript_id=XM_029851713.1
```

Create database for AalbF3. SnpEff documentation HERE:

```
There are three main steps when building a database:

Step 1: Configure a new genome in SnpEff's config file snpEff.config.
Step 2: Build using gene annotations and reference sequences
Step 3: Checking the database: SnpEff will check the database by comparing predicted protein sequences and CDS sequences with ones provided by the user.
```

Documentation regarding building database using GFF file:

```
GFF File name

SnpEff expects the GFF file to be located at


$SNPEFF_HOME/data/GENOME_NAME/genes.gff
where:

$SNPEFF_HOME is the directory where SnpEff is installed (usually $HOME/snpEff)
GENOME_NAME is the genome name of the genome you are trying to build, which MUST match the name you added in the config file snpEff.config
Note: The file name can be genes.gff.gz if it's compressed using gzip.
```

```
# change the path to the location you have SnpEff
# make sure you move the gene.gff to the directory you have SnpEff
# also add the genome to snpEff.config at SnpEff home directory
echo "albo_v3.genome : Albopictus" >> snpEff.config
#
# then build the database, in my case I had a few databases that I used previously. You can name it anyway you want.
java -Xmx8g -jar /Users/lucianocosme/Packages/snpEff/snpEff.jar build -gff3 -v albo_v3
```

Check SnpEff options

```
# This will show a 'help' message
java -jar /Users/lucianocosme/Packages/snpEff/snpEff.jar
```

## 4. Perform variant annotation

### 4.1 Prepare data

We will annotate only SNPs that passed quality control. Our first
step is to convert it to vcf file format

```
# We will first annotate the SNPs from the wild caught animals. We will use the file after quality control.
plink2 \
--bfile data/raw_data/albo/variant_annotation/file7 \
--allow-extra-chr \
--fa data/genome/albo.fasta.gz \
--ref-from-fa 'force' \
--recode vcf \
--out output/SnpEff/wild \
--silent;
grep 'variants' output/SnpEff/wild.log;
```

Check sample names on the vcf

```
bcftools query -l output/SnpEff/wild.vcf | head
```

```
## OKI_1001
## OKI_1002
## OKI_1003
## OKI_1004
## OKI_1005
## OKI_1006
## OKI_1007
## OKI_1008
## OKI_1009
## OKI_1011
```

### 4.2 Run annotation

```
# then annotate the vcf with the v4
java -Xmx8g -jar /Users/lucianocosme/Packages/snpEff/snpEff.jar albo_v3 output/SnpEff/wild.vcf -v > output/SnpEff/chip_ann.vcf
#
# we will get some warnings or errors because the annotation was lifted from AalbF2 to the AalbF3, with some transcripts missing some features.
# therefore we need to focus on genes with id starting with LOC
```

Move files to output

```
# SnpEff will create a .txt and a .html file in our project root directory, we can move them to the right place
mv snpEff_* output/SnpEff/
# you can double click the file snpEff_summary.html to see the summary of our annotation
```

Get types of variants

```
# i wrote a bash script to get the number of variants we have, you might have to run chmod 775 if you get permission denied
./scripts/analysis/variants_by_effect.sh output/SnpEff/chip_ann.vcf
```

Alternatively, we can create a file with the effects and how they are
annotated in the vcf file

```
echo "Nonsynonymous missense_variant
Synonymous synonymous_variant
Intron intron_variant
Intergenic intergenic_region
3UTR 3_prime_UTR_variant
5UTR 5_prime_UTR" > output/SnpEff/variant_types.txt
```

```
# not the most elegant but works
cat output/SnpEff/variant_types.txt | while IFS=" " read -r variant effect
do
  echo "$variant"; cat output/SnpEff/chip_ann.vcf | sed '/##/d' | grep -c "$effect"
done | xargs -n2 > output/SnpEff/annotations_summary.txt
```

Now, we can import it into R and make a table

```
# import data
var_effects <- read_delim(
  here(
    "output", "SnpEff", "annotations_summary.txt"
  ),
  col_names = FALSE,
  show_col_types = FALSE,
  col_types = "ci"
) |>
  rename(
    Effect            = 1,
    Number            = 2
  ) |>
  mutate(
    Effect = as.factor(
      Effect
    ),
    Percentage = prop.table(
      Number
    ) * 100,
    across(
      3, ~round(., 2)
    )
  ) |>
  arrange(
    -Percentage
  )

# make table
ftabv <- flextable(
  var_effects |>
  rename(
    Effect            = 1,
    "Number of SNPs"  = 2
  )
) |>
  set_caption(
    as_paragraph(
      as_chunk(
        "Variant effects for SNPs genotyped with the Chip",
        props         = fp_text_default(
          font.family = "Cambria"
        )
      )
    ),
    word_stylename    = "Table Caption"
  )

# Set theme
ftabv <- flextable::theme_zebra(ftabv)


# print(ftab, preview = "docx") # to open and see it in Microsoft Word.
ftabv
```

Variant effects for SNPs genotyped with the Chip

| Effect | Number of SNPs | Percentage |
| --- | --- | --- |
| Intron | 24,387 | 39.49 |
| Synonymous | 14,323 | 23.20 |
| Intergenic | 13,758 | 22.28 |
| 3UTR | 3,697 | 5.99 |
| 5UTR | 3,642 | 5.90 |
| Nonsynonymous | 1,942 | 3.14 |

```
# save
# then you can open MS Word and make adjustments
ftabv |>
  autofit() |>
  save_as_docx(
    path = here(
      "output", "SnpEff", "effect_type_and_number_SNPs.docx"
    )
  )
```

We can also make a plot of the effects

```
# create vector of colors for our plot
# Wong, B. Points of view: Color blindness. Nat Methods (2011).
bla <- '#000000'
blu <- '#0072b2'
grb <- '#56b4e9'
lir <- '#cc79a7'
gre <- '#009e73'
red <- '#d55e00'
org <- '#e69f00'
yel <- '#f0e442'
gry <- '#BBBBBB'
#
# create vector
# option1
# special_colors <- list(
#   "Intron"        = "#BBBBBB",
#   "Intergenic"    = "#0072b2",
#   "Synonymous"    = "#009e73",
#   "5UTR"          = "#56b4e9",
#   "3UTR"          = "#cc79a7",
#   "Nonsynonymous" = "#d55e00"
# )
# 
# Option 2
special_colors <- list(
  "Intron"        = "#C1CDCD",
  "Intergenic"    = "#1AC722",
  "Synonymous"    = "#FF8C00",
  "5UTR"          = "#87CEFA",
  "3UTR"          = "#FF3030",
  "Nonsynonymous" = "pink"
)
#
# make plot
var_effects |>
  mutate(                                   # we can use mutate to change the class of column
    Effect            = as.factor(          # we make column "Effect" factor
      Effect
      ),
    Effect = fct_reorder(                   # library forcats to re-order by the number of variants
       Effect, Number
      )
  ) |>
  ggplot() +
  geom_col(                                 # to make bar plot with different colors
    aes(                                    # mapping
      x               = Effect,
      y               = Number,
      fill            = Effect
    ),
    color             = "yellow"            # for the border
  ) +
  labs(                                     # to change the ylab and tittle
    title             = "Chip variants per SnpEff annotation",
    y                 = "Number of annotated variants",
    x                 = "Annotated effect",
    caption           = "Functional annotation of the SNPs in the CHIP after QC (~60k)"
  ) +
  hrbrthemes::theme_ipsum(                  # I like this theme, but you can use any
    base_family       = "Roboto Condensed", # needs to have extrafont library
    axis_text_size    = 12,
    axis_title_size   = 14,
    plot_margin       = margin(
      10, 10, 10, 10
    ),
    grid              = TRUE,
    grid_col          = "#fabbe2"
  ) +
   scale_fill_manual(
    values = unlist(
    special_colors
    )
  ) +
  scale_color_manual(
    values            = unlist(
    special_colors
    )
  ) +
  theme(
    legend.position   = "none",
    panel.grid.major  = element_line(
      linetype        = "dashed",
      linewidth       = 0.2,
    ),
    panel.grid.minor  = element_line(
      linetype        = "dashed",
      linewidth       = 0.2
    )
  ) +
  scale_y_continuous(
        trans = "log10",
        breaks = scales::log_breaks(
          n = 10
        ),
        labels = label_comma()
    ) +
  annotation_logticks(
    sides = "b"
  ) +
  geom_label(
    aes(
      x               = Effect,
      y               = Number,
      color           = Effect,
      label=paste(
        formatC(
          Number, format="f", big.mark = ",", digits=0
          ),"\n","(",Percentage,"%)"
      )
    ),
    face              = "bold",
    size              = 2,
    family            = "",
    alpha             = .9,
  ) +
  coord_flip()                             # to make it easier to read
```

```
#
# save plot
ggsave(
  here(
    "output", "SnpEff", "figures", "SNPs_effect_chip.pdf"
  ),
  width               = 10,
  height              = 4,
  units               = "in"
)
```

We can make variant lists based on their effect. It is useful for
other analysis. However, since the annotation is not optimal, we have to
be careful.

```
# variants from intergenic regions
sed '/##/d' output/SnpEff/chip_ann.vcf | grep "intergenic_region" | awk '{print $3}' > output/SnpEff/intergenic_SNPs.txt
#
# variants from introns
sed '/##/d' output/SnpEff/chip_ann.vcf | grep "intron_variant" | awk '{print $3}' > output/SnpEff/intron_SNPs.txt
#
# variants from 3'UTR
sed '/##/d' output/SnpEff/chip_ann.vcf | grep "3_prime_UTR_variant" | awk '{print $3}' > output/SnpEff/3UTR_SNPs.txt
#
# variants from 5'UTR
sed '/##/d' output/SnpEff/chip_ann.vcf | grep "5_prime_UTR" | awk '{print $3}' > output/SnpEff/5UTR_SNPs.txt
#
# variants from both 3 and 5'UTRs
sed '/##/d' output/SnpEff/chip_ann.vcf | grep "3_prime_UTR_variant\|5_prime_UTR" | awk '{print $3}' > output/SnpEff/UTRs_SNPs.txt
#
# synonymous
sed '/##/d' output/SnpEff/chip_ann.vcf | grep "synonymous_variant" | awk '{print $3}' > output/SnpEff/Synonymous_SNPs.txt
#
# nonsynonymous
sed '/##/d' output/SnpEff/chip_ann.vcf | grep "missense_variant" | awk '{print $3}' > output/SnpEff/Nonsynonymous_SNPs.txt
```

We can also import the output of SnpEff into R

```
# import SnpEff output
snpEff <-
  read_delim(
    here(
      "output", "SnpEff", "snpEff_genes.txt"
    ),
    delim = '\t',
     comment = "# ",
    col_names = TRUE,
    show_col_types = FALSE
  ) |>
  rename(
    GeneName = 1
  )
# we can check the name of the columns
colnames(snpEff)
```

```
##  [1] "GeneName"                                                      
##  [2] "GeneId"                                                        
##  [3] "TranscriptId"                                                  
##  [4] "BioType"                                                       
##  [5] "variants_impact_HIGH"                                          
##  [6] "variants_impact_LOW"                                           
##  [7] "variants_impact_MODERATE"                                      
##  [8] "variants_impact_MODIFIER"                                      
##  [9] "variants_effect_3_prime_UTR_variant"                           
## [10] "variants_effect_5_prime_UTR_premature_start_codon_gain_variant"
## [11] "variants_effect_5_prime_UTR_variant"                           
## [12] "variants_effect_downstream_gene_variant"                       
## [13] "variants_effect_initiator_codon_variant"                       
## [14] "variants_effect_intron_variant"                                
## [15] "variants_effect_missense_variant"                              
## [16] "variants_effect_non_coding_transcript_exon_variant"            
## [17] "variants_effect_non_coding_transcript_variant"                 
## [18] "variants_effect_splice_acceptor_variant"                       
## [19] "variants_effect_splice_donor_variant"                          
## [20] "variants_effect_splice_region_variant"                         
## [21] "variants_effect_stop_gained"                                   
## [22] "variants_effect_stop_lost"                                     
## [23] "variants_effect_stop_retained_variant"                         
## [24] "variants_effect_synonymous_variant"                            
## [25] "variants_effect_upstream_gene_variant"
```

Lets get some gene counts

```
# because the annotation was lifted from one genome assembly to a new one, we have some issues with the functional annotation. For example, we have genes missing start or stop codons, exons, etc. Therefore, we can focus on genes with the initial ID. SnpEff ties to annotate genes with missing exons or that have been broken up due to lifting.
# lets count how many genes we have starting with the letter LOC and remove rows with exons only
protein <-
  snpEff |>
  filter(
    !grepl(
      "exon",
      GeneName
    )
  ) |>
  filter(
    grepl(
      "LOC",
      GeneName
    )
  ) |>
  count(
    BioType,
    sort = TRUE
  ) |>
  rename(
    "BioType of the gene"       = 1,
    "Number of genes with SNPs" = 2
  )
#
# make table
set_flextable_defaults(na_str = "NA", big.mark = ",")
ftabp <-
  flextable(
    protein
  ) |>
  set_caption(
    as_paragraph(
      as_chunk(
        "Number of genes with SNPs",
        props = fp_text_default(
          font.family = "Cambria"
        )
      )
    ),
    word_stylename = "Table Caption"
  )
#
# Apply zebra theme
ftabp <- flextable::theme_zebra(ftabp)
# print(ftab, preview = "docx") # to open and see it in Microsoft Word.
ftabp
```

Number of genes with SNPs

| BioType of the gene | Number of genes with SNPs |
| --- | --- |
| protein\_coding | 17,461 |
| pseudogene | 982 |
| NA | 448 |
| snRNA | 13 |
| snoRNA | 5 |
| rRNA | 2 |

```
# save
# then you can open MS Word and make adjustments
ftabp |>
  autofit() |>
  save_as_docx(
    path = here(
      "output", "SnpEff", "Number_of_genes_with_SNPs.docx"
    )
  )
```

### 4.3 Parsing vcf instead of using bash loops

The plot and statistics above are an approximation since the
annotation was lifted, and we have more variant types I did not list. We
could import the vcf to R, and parse it.

Import vcf to R.

```
# following the code shared here https://joemcgirr.github.io/files/code_tutorials/my_genome/SnpEFF.html
start_time <- Sys.time()
# Wong, B. Points of view: Color blindness. Nat Methods (2011).
bla <- '#000000'
blu <- '#0072b2'
grb <- '#56b4e9'
lir <- '#cc79a7'
gre <- '#009e73'
red <- '#d55e00'
org <- '#e69f00'
yel <- '#f0e442'
gry <- '#BBBBBB'
#
# this step we did it earlier
# wget https://snpeff.blob.core.windows.net/versions/snpEff_latest_core.zip
# unzip snpEff_latest_core.zip
```

We need to get gatk to convert vcf to table

```
# you can run it on the terminal
wget \
https://github.com/broadinstitute/gatk/releases/download/4.3.0.0/gatk-4.3.0.0.zip \
--directory-prefix output/files
#
# decompress it
unzip \
/Users/lucianocosme/Dropbox/Albopictus/manuscript_chip/data/no_autogenous/albo_chip/output/files/gatk-4.3.0.0.zip \
-d /Users/lucianocosme/Packages
# export path
export PATH=$PATH:/Users/lucianocosme/Packages/gatk-4.3.0.0
#
```

Convert vcf to table

```
# export path
export PATH=$PATH:/Users/lucianocosme/Packages/gatk-4.3.0.0;

gatk \
VariantsToTable \
-V output/SnpEff/chip_ann.vcf \
-F CHROM -F POS -F TYPE -F ID -F ANN -F LOF -F NMD -GF AD -GF DP -GF GQ -GF GT \
-O output/SnpEff/chip_ann.txt
```

Import data to R

```
# read the data
ann <- vroom(
  here(
    "output", "SnpEff", "chip_ann.txt"
  ),
  guess_max = 1000000,
  show_col_types = FALSE
)
#
```

Create annotation types

```
# table based on SnpEFF documentation
ann_types <- data.frame(
  impact = c(
    "HIGH", "HIGH", "HIGH", "HIGH", "HIGH", "HIGH", "HIGH", "HIGH", "HIGH", "HIGH", "MODERATE", "MODERATE", "MODERATE", "MODERATE", "MODERATE", "MODERATE", "MODERATE", "MODERATE", "MODERATE", "MODERATE", "MODERATE", "LOW", "LOW", "LOW", "LOW", "LOW", "LOW", "MODIFIER", "MODIFIER", "MODIFIER", "MODIFIER", "MODIFIER", "MODIFIER", "MODIFIER", "MODIFIER", "MODIFIER", "MODIFIER", "MODIFIER", "MODIFIER", "MODIFIER", "MODIFIER", "MODIFIER", "MODIFIER", "MODIFIER", "MODIFIER", "MODIFIER", "MODIFIER", "MODIFIER", "MODIFIER", "MODIFIER", "MODIFIER", "MODIFIER"
  ),
  ontology_term = c(
    "chromosome_number_variation", "exon_loss_variant", "frameshift_variant", "rare_amino_acid_variant", "splice_acceptor_variant", "splice_donor_variant", "start_lost", "stop_gained", "stop_lost", "transcript_ablation", "3_prime_UTR_truncation&exon_loss", "5_prime_UTR_truncation&exon_loss_variant", "coding_sequence_variant-moderate", "conservative_inframe_deletion", "conservative_inframe_insertion", "disruptive_inframe_deletion", "disruptive_inframe_insertion", "missense_variant", "regulatory_region_ablation", "splice_region_variant-moderate", "TFBS_ablation", "5_prime_UTR_premature_start_codon_gain_variant", "initiator_codon_variant", "splice_region_variant-low", "start_retained", "stop_retained_variant", "synonymous_variant", "3_prime_UTR_variant", "5_prime_UTR_variant", "coding_sequence_variant-modifier", "conserved_intergenic_variant", "conserved_intron_variant", "downstream_gene_variant", "exon_variant", "feature_elongation", "feature_truncation", "gene_variant", "intergenic_region", "intragenic_variant", "intron_variant", "mature_miRNA_variant", "miRNA", "NMD_transcript_variant", "non_coding_transcript_exon_variant", "non_coding_transcript_variant", "regulatory_region_amplification", "regulatory_region_variant", "TF_binding_site_variant", "TFBS_amplification", "transcript_amplification", "transcript_variant", "upstream_gene_variant"
  )
)
# check the possible variant annotations
ann_types |>
  gt() |>
  tab_header(
    title = "Putative impact for sequence ontology terms output by SnpEFF"
  )
```

|  |  |
| --- | --- |
| Putative impact for sequence ontology terms output by SnpEFF | |
| impact | ontology\_term |
| HIGH | chromosome\_number\_variation |
| HIGH | exon\_loss\_variant |
| HIGH | frameshift\_variant |
| HIGH | rare\_amino\_acid\_variant |
| HIGH | splice\_acceptor\_variant |
| HIGH | splice\_donor\_variant |
| HIGH | start\_lost |
| HIGH | stop\_gained |
| HIGH | stop\_lost |
| HIGH | transcript\_ablation |
| MODERATE | 3\_prime\_UTR\_truncation&exon\_loss |
| MODERATE | 5\_prime\_UTR\_truncation&exon\_loss\_variant |
| MODERATE | coding\_sequence\_variant-moderate |
| MODERATE | conservative\_inframe\_deletion |
| MODERATE | conservative\_inframe\_insertion |
| MODERATE | disruptive\_inframe\_deletion |
| MODERATE | disruptive\_inframe\_insertion |
| MODERATE | missense\_variant |
| MODERATE | regulatory\_region\_ablation |
| MODERATE | splice\_region\_variant-moderate |
| MODERATE | TFBS\_ablation |
| LOW | 5\_prime\_UTR\_premature\_start\_codon\_gain\_variant |
| LOW | initiator\_codon\_variant |
| LOW | splice\_region\_variant-low |
| LOW | start\_retained |
| LOW | stop\_retained\_variant |
| LOW | synonymous\_variant |
| MODIFIER | 3\_prime\_UTR\_variant |
| MODIFIER | 5\_prime\_UTR\_variant |
| MODIFIER | coding\_sequence\_variant-modifier |
| MODIFIER | conserved\_intergenic\_variant |
| MODIFIER | conserved\_intron\_variant |
| MODIFIER | downstream\_gene\_variant |
| MODIFIER | exon\_variant |
| MODIFIER | feature\_elongation |
| MODIFIER | feature\_truncation |
| MODIFIER | gene\_variant |
| MODIFIER | intergenic\_region |
| MODIFIER | intragenic\_variant |
| MODIFIER | intron\_variant |
| MODIFIER | mature\_miRNA\_variant |
| MODIFIER | miRNA |
| MODIFIER | NMD\_transcript\_variant |
| MODIFIER | non\_coding\_transcript\_exon\_variant |
| MODIFIER | non\_coding\_transcript\_variant |
| MODIFIER | regulatory\_region\_amplification |
| MODIFIER | regulatory\_region\_variant |
| MODIFIER | TF\_binding\_site\_variant |
| MODIFIER | TFBS\_amplification |
| MODIFIER | transcript\_amplification |
| MODIFIER | transcript\_variant |
| MODIFIER | upstream\_gene\_variant |

Make variant plots. First, get variant effect numbers

```
# function to calculate the number of variants annotated for each ontology
# not my code, it is from https://joemcgirr.github.io/files/code_tutorials/my_genome/SnpEFF.html
n_variants <- c()
for (ot in ann_types$ontology_term) {
  n_variants <- c(n_variants, (filter(ann, grepl(ot, ANN)) |> nrow()))
  # print(ot)
}
#
# create new column and sort
ann_types$n_variants <- n_variants
ann_types <- arrange(ann_types, n_variants)
#
# set factors
ann_types$ontology_term <- factor(ann_types$ontology_term, levels = ann_types$ontology_term)
ann_types$impact <- factor(ann_types$impact, levels = c("HIGH","MODERATE","LOW","MODIFIER"))
```

Prepare data for plotting

```
# arrange and remove those with those with no variants
ann_types2 <-
  ann_types |>
  mutate(
    ontology_term = str_remove_all(  # to remove the _variant from the column
      ontology_term, "_variant"
    )
  ) |>
  arrange(
    -n_variants, ann_types
  ) |>
  filter(
    !row_number() %in% c(1, 12)      # remove row with by index, we remove those that have the same values
  ) |>
  filter(
    n_variants > 0
  ) |>
  mutate(
    impact        = tolower(         # we use base R to convert all values on column Effect to lower case
      impact
    ),
    impact        = str_to_sentence( # here we use library stringr to convert the first letter to upper case
      impact
    ),
    ontology_term = str_to_sentence( # here we use library stringr to convert the first letter to upper case
      ontology_term
    ),
    across("ontology_term", ~str_replace(., "_", " ")),
    across("ontology_term", ~str_replace(., " prime_utr", "'UTR")),
    across("ontology_term", ~str_replace(., "_premature_start_codon_gain", " start codon gain")),
    across("ontology_term", ~str_replace(., "Missense", "Nonsynonymous")),
    ontology_term = factor(          # set ontology_term as factor
      ontology_term
    ),
    impact        = factor(          # set impact as factor
      impact
    ),
    Percentage    = prop.table(      # we use prop.table to calculate percentages
      n_variants
    ) * 100,
    across(                          # we use across to set column 3 as 2 decimals
      4, round, 2
    )
  ) |>
  rename(                            # to make impact start with capital letter
    Impact        = 1
  )
```

Make plot

```
# Wong, B. Points of view: Color blindness. Nat Methods (2011).
bla <- '#000000'
blu <- '#0072b2'
grb <- '#56b4e9'
lir <- '#cc79a7'
gre <- '#009e73'
red <- '#d55e00'
org <- '#e69f00'
yel <- '#f0e442'
gry <- '#BBBBBB'
#
# create colors
my_colors2 <- c(
  '#d55e00', '#f0e442', '#009e73','#56b4e9'
)
#
# create plot
p1 <-
  ann_types2 |>
  mutate(
    ontology_term     = fct_reorder(              # library forcats to re-order by the number of variants
      ontology_term, n_variants
    ),
    Impact            = fct_relevel(
      Impact,
      "High", "Moderate", "Low", "Modifier"
    ),
  ) |>
  ggplot(
    aes()
  ) +
  geom_col(                                       # to make colorful bar plot
    aes(
      x               = ontology_term,
      y               = n_variants,
      fill            = Impact
    ),
    color             = "pink",                   # for the border
    linewidth         = .3
  ) +
  coord_flip() +                                  # to make it easier to read
  hrbrthemes::theme_ipsum(
    base_family       = "Roboto Condensed",
    axis_text_size    = 12,
    axis_title_size   = 12,
    plot_margin       = margin(
      10, 10, 10, 10                              # top, right, bottom, left
    ),
    grid              = TRUE,
    grid_col          = "#fabbe2"
  ) +
  labs(
    y                 = "Number of annotated variants",
    x                 = "",
    title             = "Chip variants per SnpEff annotation"
  ) +
  geom_text(
    aes(
      y               = n_variants,
      x               = ontology_term,
      color           = Impact,
      label           = percent(
        Percentage / 100
      )                                          # we use library scales function to show percentage
    ),
    size              = 3.5,
    family            = "Roboto Condensed",
    hjust             = -0.01
  ) +
  scale_fill_manual(
    values            = my_colors2
  ) +
  scale_color_manual(
    values            = my_colors2
  ) +
  scale_y_continuous(
    labels            = comma
  ) +
  theme(
    legend.title      = element_text(
      family          = "Roboto Condensed",
      face            = "bold",
    ),
    legend.position   = "top",
    axis.title.x      = element_text(
      size            = 12
    ),
    axis.title.y      = element_text(
      size            = 12
    ),
    axis.title        = element_text(
      size            = 12
    ),
    axis.text         = element_text(
      size            = 12
    ),
    plot.title        = element_text(
      size            = 18,
      face            = "bold"
    ),
    panel.grid.major  = element_line(
      linetype        = "dashed",
      linewidth       = 0.2,
    ),
    panel.grid.minor  = element_line(
      linetype        = "dashed",
      linewidth       = 0.2
    )
  )
#
p1
```

```
#
# save plot
ggsave(
  here(
    "output", "figures", "SNPs_effect_chip_plot2.pdf"
  ),
  width  = 11,
  height = 5,
  units  = "in"
)
```

The downside of this approach is importing the vcf into R. If you
have millions of SNPs it is not doable.

## 5. Getting SNPs and their effects on genes relevant for mosquito life history traits

### 5.1 Diapause genes

We can obtain the list of genes from Boyle, et al. (2021). There is
some indication that these may be associated with the climatic
adaptation of Ae. albopictus in cold regions, where winter conditions do
not allow breeding or survival of mosquitoes for a few months. We will
use genes with FDR < 0.05; check their paper for more details
(supplemental material).

Transpose file with the diapause genes

```
# copied the genes from the paper
# if you are in a MacOS, you can install datamash, and on the terminal, run the command below (but don't worry, we have both files in the repository)
# brew install datamash
# then we can easily transpose the file 
cat output/files/diapause_genes_fdr_05_boyle_et_al_2021.txt | datamash -W transpose > output/files/fdr_05_diapause_genes.txt
```

Get the SNPs on the diapause genes

```
cat <(echo 'Gene Number_SNPs') \
<(cat output/files/fdr_05_diapause_genes.txt | while IFS=" " read -r diapause
do
    echo $diapause; cat output/SnpEff/chip_ann.vcf | sed '/##/d' | grep $diapause | wc -l
done | xargs -n2) > output/files/number_snps_per_diapause_gene.txt;
```

```
# import data
diapause_genes <-
  read_delim(
    here(
      "output", "files", "number_snps_per_diapause_gene.txt"
    ),
    col_names      = TRUE,
    show_col_types = FALSE,
    col_types      = "ci"
  ) |>
  rename(
    Gene           = 1,
    SNPs           = 2
  ) |>
  arrange(           # here we sort by descending value
    desc(
      SNPs
    )
  )
#
# make table
ftabd <-
  flextable(
    diapause_genes
  ) |>
  set_caption(
    as_paragraph(
      as_chunk(
        "Number of SNPs on diapause genes",
        props         = fp_text_default(
          font.family = "Cambria"
        )
      )
    ),
    word_stylename    = "Table Caption"
  )
#

# Apply zebra theme
ftabd <- flextable::theme_zebra(ftabd)
# print(ftab, preview = "docx") # to open and see it in Microsoft Word.
ftabd
```

Number of SNPs on diapause genes

| Gene | SNPs |
| --- | --- |
| LOC109411232 | 19 |
| LOC109398746 | 4 |
| LOC109402178 | 3 |
| LOC109398750 | 3 |
| LOC109402174 | 2 |
| LOC109398749 | 2 |
| LOC109398756 | 2 |
| LOC109398753 | 1 |
| LOC109398754 | 1 |
| LOC109413239 | 0 |
| LOC109424986 | 0 |

```
# save
# then you can open MS Word and make adjustments
ftabd |>
  autofit() |>
  save_as_docx(
    path = here(
      "output", "SnpEff", "Number_SNPs_on_diapause_genes.docx"
    )
  )
```

Now lets get the SNPs associated on diapause genes

```
# we can bash tools to get some information about our SNPs
# to make our command to run faster, we can grep the rows with the diapause genes from our vcf and create a new file. Then we can use it to parse our results
# create new file with only the genes of interest - we could use # grep -Ff file1 file2
# ripgrep https://formulae.brew.sh/formula/ripgrep  is faster - it will take long if we have long gene lists
# on MacOS you can use brew to install it - brew install ripgrip
# the -Ff are option to search fixed patter (-F), and file (-f) - check man rg
rg -Ff \
output/files/fdr_05_diapause_genes.txt \
output/SnpEff/chip_ann.vcf \
> output/SnpEff/fdr_05_diapause_snps.txt
```

Now use get the SNPs

```
# we can now parse the results to make a table later
grep "missense\|synonymous\|5_prime_UTR_variant\|3_prime_UTR_variant" output/SnpEff/fdr_05_diapause_snps.txt \
| awk '{print $1,$2,$3,$4,$5,$8}' \
| sed 's/|/\t/g' \
| awk '{print $1,$2,$3,$4,$5,$8,$7,$13,$15,$16,$23}' \
| sed 's|exon-XM_019671140.2-1|LOC109398756|' \
| sed 's|XM_019684743.2|LOC109411232|' \
| sed 's|XM_019686967.2|LOC109413239|' \
| sed 's|XM_019671140.2|LOC109398750|' \
| sed 's|rna-XM_029870393.1|LOC109398750|' \
| sed 's|XM_029870418.1|LOC109424986|' \
| sed 's|exon-XM_029870393.1-1|LOC109398750|' \
> output/SnpEff/snps_diapause_genes_1.txt
# the script above is not perfect, and we end up missing some gene IDs, we can use grep to get the rows with the missing ids. For example,
# we get a SNPs with gene id rna-XM_029870393.1, we can use grep "rna-XM_029870393.1" output/SnpEff/chip_ann.vcf to find its id. I did that and added that to the file
# we can use sed to replace them in our file
# you can use grep to get the gene names from the gff file.
# for example
# grep "exon-XM_029870393.1-1" output/files/genes.gff 
# chr3.19   Gnomon  exon    2234864 2235110 .   -   .   ID=exon-XM_029870393.1-1;Parent=rna-XM_029870393.1;Dbxref=GeneID:109398750,Genbank:XM_029870393.1;gbkey=mRNA;gene=LOC109398750;product=methyl-CpG-binding domain protein 3%2C transcript variant X2;transcript_id=XM_029870393.1
# I did it for all genes missing ID
# LOC109398756 # exon-XM_019671140.2-1
# LOC109411232 # XM_019684743.2
# LOC109413239 # XM_019686967.2
# LOC109398750 # XM_019671140.2
# LOC109398750 # rna-XM_029870393.1
# LOC109424986 # XM_029870418.1
```

SNPs on introns

```
# the command in previous chunk does not work well for the SNPs on introns, we can do it in two steps
paste -d ' ' <(
# step 1
grep "intron_variant" output/SnpEff/fdr_05_diapause_snps.txt \
| awk '{print $1,$2,$3,$4,$5,$8}' \
| sed 's/,/\t/g' \
| awk '{print $1,$2,$3,$4,$5,$6}' \
| sed 's/|/\t/g' \
| awk '{print $1,$2,$3,$4,$5,$8}') \
<(
# step 2
grep "intron_variant" output/SnpEff/fdr_05_diapause_snps.txt\
| awk '{print $1,$2,$3,$4,$5,$8}' \
| sed 's/,/\t/g' \
| grep "protein_coding\|intron_variant" \
| awk '{
    for (i = 1; i <= NF; i++) {
        if ($i ~ /intron_variant/) {
            printf "%s %s ", $i, $(i + 1)
        }
    }
    print ""
}' | sed 's/|/\t/g' | awk '{print $2,$8,$10,$13,$15}') > output/SnpEff/snps_diapause_genes_2.txt
```

We can make a table from our data

```
# import data
snps_diapause1 <-                         # name of the tibble
  read_delim(                             # function to read the file, check options typing ?read_delim on the console
    here(                                 # use library here to find the file
      "output", "SnpEff", "snps_diapause_genes_1.txt" # the location of the file and it's name, works in all plattaforms
    ),
    col_names            = FALSE,         # we don't have columns names
    show_col_types       = FALSE,         # to hide message from tidyverse library
  ) |>
  rename(                                 # we use rename to specify the names of each column
    Scaffold             = 1,             # the numbers are the columns numbers from left to right, the the first word is the name of the columns
    Position             = 2,
    SNP                  = 3,
    "Reference allele"   = 4,             # if the name of the columns is more than one word, we have to use " "
    "Alternative allele" = 5,
    Effect               = 6,
    Type                 = 7,
    Biotype              = 8,
    "Nucleotide change"  = 9,
    "Aminoacid change"   = 10,
    GeneID               = 11
  ) |>
  mutate(
    Scaffold             = as.character(  # make scaffold as character, we will manipulate it later
      Scaffold
    )
  )
# sendond file
snps_diapause2 <-
  read_delim(
    here(
      "output", "SnpEff", "snps_diapause_genes_2.txt"
    ),
    col_names            = FALSE,
    show_col_types       = FALSE,
  ) |>
  rename(
    Scaffold             = 1,
    Position             = 2,
    SNP                  = 3,
    "Reference allele"   = 4,
    "Alternative allele" = 5,
    Effect               = 6,
    Type                 = 7,
    Biotype              = 8,
    "Nucleotide change"  = 9,
    "Aminoacid change"   = 10,
    GeneID               = 11
  ) |>
  mutate(
    "Aminoacid change"   = " "              # adds a columns since SNPs on introns don't cause aa changes
  ) |>
  mutate(
    Scaffold             = as.character(    # make scaffold as character, we will manipulate it later
      Scaffold
    )
  )
#
# rbind them
snps_diapause3 <-
  rbind(                                    # use rbind combine the objects
    snps_diapause1, snps_diapause2          # the name of the objects
  ) |>
  arrange(                                  # use arrange to sort by scaffold and type
    Scaffold, Type
  ) |>
  mutate(                                   # use mutate to change geneIDs
    GeneID = str_remove(                    # use library stringr to remove strings
      GeneID, "gene-"                       # we remove gene- since some genes have the extra word
    )
  ) |>
  mutate_at(                                # we use mutate_at to only make changes in one column
    vars(                                   # to select column or variable
      "Aminoacid change"                    # name of the column
    ), funs(                                # list function calls
      replace(                              # to replace
        ., !grepl(                          # we use !grepl to remove fields without the pattern
          "p", .                            # all amino acid changes have the letter p on them, so we remove those without it
        ),
        ""                                  # here we replace it with nothing using ''
      )
    )
  ) |>
  mutate(
  Effect   = tolower(                       # we use base R to convert all values on column Effect to lower case
    Effect
  )
  ) |>
  mutate(
    Effect = str_to_sentence(               # here we use library stringr to convert the first letter to upper case, just like in a sentence
      Effect
    )
  )
#
#
# make table
ftab.snps <-
  flextable(
    snps_diapause3
  ) |>
  set_caption(
    as_paragraph(
      as_chunk(
        "SNPs on diapause genes",
        props      = fp_text_default(
          font.fam = "Cambria"
        )
      )
    ),
    word_stylena   = "Table Caption"
  )
#
# Apply zebra theme
ftab.snps <- flextable::theme_zebra(ftab.snps)

# print(ftab.snps, preview = "docx") # to open and see it in Microsoft Word.
ftab.snps |>
  autofit()
```

SNPs on diapause genes

| Scaffold | Position | SNP | Reference allele | Alternative allele | Effect | Type | Biotype | Nucleotide change | Aminoacid change | GeneID |
| --- | --- | --- | --- | --- | --- | --- | --- | --- | --- | --- |
| 1.5 | 384,768 | AX-583746747 | G | A | Modifier | intron\_variant | protein\_coding | c.145+30C>T |  | LOC109402178 |
| 1.5 | 374,571 | AX-583746721 | A | C | Low | synonymous\_variant | protein\_coding | c.456T>G | p.Ala152Ala | LOC109413238 |
| 1.5 | 374,775 | AX-583750651 | A | G | Low | synonymous\_variant | protein\_coding | c.252T>C | p.Asp84Asp | LOC109413238 |
| 1.5 | 460,009 | AX-583747442 | G | A | Low | synonymous\_variant | protein\_coding | c.1212C>T | p.Pro404Pro | LOC109402173 |
| 1.5 | 460,904 | AX-583747485 | T | C | Low | synonymous\_variant | protein\_coding | c.432A>G | p.Leu144Leu | LOC109402173 |
| 3.1 | 3,300,814 | AX-580336192 | A | T | Modifier | 3\_prime\_UTR\_variant | protein\_coding | c.\*729T>A |  | LOC109411232 |
| 3.1 | 3,463,101 | AX-580336870 | T | G | Modifier | 5\_prime\_UTR\_variant | protein\_coding | c.-341A>C |  | LOC109411232 |
| 3.1 | 3,463,774 | AX-580336878 | T | C | Modifier | 5\_prime\_UTR\_variant | protein\_coding | c.-1014A>G |  | LOC109411232 |
| 3.1 | 3,304,574 | AX-580338227 | C | T | Modifier | intron\_variant | protein\_coding | c.961+594G>A |  | LOC109411232 |
| 3.1 | 3,304,968 | AX-580338236 | A | G | Modifier | intron\_variant | protein\_coding | c.961+200T>C |  | LOC109411232 |
| 3.1 | 3,339,542 | AX-580336357 | T | A | Modifier | intron\_variant | protein\_coding | c.679-30795A>T |  | LOC109411232 |
| 3.1 | 3,340,340 | AX-580338346 | G | A | Modifier | intron\_variant | protein\_coding | c.679-31593C>T |  | LOC109411232 |
| 3.1 | 3,340,788 | AX-580336395 | A | T | Modifier | intron\_variant | protein\_coding | c.679-32041T>A |  | LOC109411232 |
| 3.1 | 3,377,418 | AX-580338434 | G | C | Modifier | intron\_variant | protein\_coding | c.678+13167C>G |  | LOC109411232 |
| 3.1 | 3,389,566 | AX-580336499 | A | T | Modifier | intron\_variant | protein\_coding | c.678+1019T>A |  | LOC109411232 |
| 3.1 | 3,389,812 | AX-580338487 | C | T | Modifier | intron\_variant | protein\_coding | c.678+773G>A |  | LOC109411232 |
| 3.1 | 3,390,039 | AX-580336526 | T | C | Modifier | intron\_variant | protein\_coding | c.678+546A>G |  | LOC109411232 |
| 3.1 | 3,401,309 | AX-580336571 | A | C | Modifier | intron\_variant | protein\_coding | c.392-170T>G |  | LOC109411232 |
| 3.1 | 3,407,679 | AX-580338576 | T | C | Modifier | intron\_variant | protein\_coding | c.392-6540A>G |  | LOC109411232 |
| 3.1 | 3,408,533 | AX-580338597 | A | G | Modifier | intron\_variant | protein\_coding | c.392-7394T>C |  | LOC109411232 |
| 3.1 | 3,427,972 | AX-580338657 | A | G | Modifier | intron\_variant | protein\_coding | c.392-26833T>C |  | LOC109411232 |
| 3.1 | 3,439,964 | AX-580338775 | T | C | Modifier | intron\_variant | protein\_coding | c.391+22406A>G |  | LOC109411232 |
| 3.1 | 3,440,228 | AX-580338784 | G | A | Modifier | intron\_variant | protein\_coding | c.391+22142C>T |  | LOC109411232 |
| 3.1 | 3,462,068 | AX-580336816 | T | C | Modifier | intron\_variant | protein\_coding | c.391+302A>G |  | LOC109411232 |
| 3.19 | 2,229,464 | AX-581509085 | A | G | Modifier | 3\_prime\_UTR\_variant | protein\_coding | c.\*44A>G |  | LOC109398750 |
| 3.19 | 2,270,349 | AX-581511297 | G | T | Modifier | intron\_variant | protein\_coding | c.-8+9G>T |  | LOC109398746 |
| 3.19 | 2,289,280 | AX-581511654 | A | G | Modifier | intron\_variant | protein\_coding | c.972+18T>C |  | LOC109398754 |
| 3.19 | 2,233,284 | AX-581509100 | G | A | Low | synonymous\_variant | protein\_coding | c.498C>T | p.Asn166Asn | LOC109398750 |
| 3.19 | 2,235,931 | AX-581510984 | G | T | Low | synonymous\_variant | protein\_coding | c.306G>T | p.Pro102Pro | LOC109398756 |
| 3.19 | 2,260,721 | AX-581509393 | G | A | Low | synonymous\_variant | protein\_coding | c.1296C>T | p.Thr432Thr | LOC109398753 |
| 3.19 | 2,271,368 | AX-581509585 | C | T | Low | synonymous\_variant | protein\_coding | c.888C>T | p.Ser296Ser | LOC109398746 |
| 3.19 | 2,271,680 | AX-581511336 | C | T | Low | synonymous\_variant | protein\_coding | c.1200C>T | p.Asn400Asn | LOC109398746 |

```
#
# save it
# create settings - check ??save_as_docx on console
sect_properties <- 
  prop_section(
  page_size    = page_size(
    orient     = "landscape",
    width      = 8.3,
    height     = 11.7
  ),
  type         = "continuous",
  page_margins = page_mar()
)
# save
# then you can open MS Word and make adjustments
ftab.snps |>
  save_as_docx(
    path       = here(
      "output", "SnpEff", "SNPs_on_diapause_genes.docx"
    ),
    pr_section = sect_properties
  )
```

### 5.2 Immunity Genes

I downloaded the supplemental material from Palinni et al, 2020, and
got the list of immunity genes. Let’s check how many SNPs we have on
them.

```
# the list of genes
wc -l output/files/immunity_genes_Palinni_et_al_2020.txt # 662 genes
```

```
# we can bash tools to get some information about our SNPs
# to make our command to run faster, we can grep the rows with the diapause genes from our vcf and create a new file. Then we can use it to parse our results
# this is slow, I already have the output in the repository
# create new file with only the genes of interest
# this time only use ripgrip since we have more than 600 genes, it will take a long time for grep to do it
rg -Ff  \
output/files/immunity_genes_Palinni_et_al_2020.txt \
output/SnpEff/chip_ann.vcf \
> output/SnpEff/immunity_snps.txt
```

We can use the same code from the diapause genes, however, since we
have many more genes now, I will not fix the geneID field. We could
write a loop to fix it later.

```
# we can now parse the results to make a table later
grep "missense\|synonymous\|5_prime_UTR_variant\|3_prime_UTR_variant" output/SnpEff/immunity_snps.txt \
| awk '{print $1,$2,$3,$4,$5,$8}' \
| sed 's/|/\t/g' \
| awk '{print $1,$2,$3,$4,$5,$8,$7,$13,$15,$16,$23}' \
> output/SnpEff/snps_immunity_genes_1.txt
```

SNPs on introns. We just replace the file names. You can use your own
gene list of interest.

```
# the command in previous chunk does not work well for the SNPs on introns, we can do it in two steps
paste -d ' ' <(
# step 1
grep "intron_variant" output/SnpEff/immunity_snps.txt \
| awk '{print $1,$2,$3,$4,$5,$8}' \
| sed 's/,/\t/g' \
| awk '{print $1,$2,$3,$4,$5,$6}' \
| sed 's/|/\t/g' \
| awk '{print $1,$2,$3,$4,$5,$8}') \
<(
# step 2
grep "intron_variant" output/SnpEff/immunity_snps.txt\
| awk '{print $1,$2,$3,$4,$5,$8}' \
| sed 's/,/\t/g' \
| grep "protein_coding\|intron_variant" \
| awk '{
    for (i = 1; i <= NF; i++) {
        if ($i ~ /intron_variant/) {
            printf "%s %s ", $i, $(i + 1)
        }
    }
    print ""
}' | sed 's/|/\t/g' | awk '{print $2,$8,$10,$13,$15}') > output/SnpEff/snps_immunity_genes_2.txt
```

Using the same code from the diapause genes, we can create a table
with our immunity genes SNPs. We could write a function to repeat the
entire process for a gene list. First write a bash pipeline to get the
files ready for R, then write a R function to import the data.

```
# import data
snps_immunity1 <-                                     # name of the tibble
  read_delim(                                         # function to read the file, check options typing ?read_delim on the console
    here(                                             # use library here to find the file
      "output", "SnpEff", "snps_immunity_genes_1.txt" # the location of the file and it's name, works in all plattaforms
    ),
    col_names            = FALSE,                     # we don't have columns names
    show_col_types       = FALSE,                     # to hide message from tidyverse library
  ) |>
  rename(                                             # we use rename to specify the names of each column
    Scaffold             = 1,                         # the numbers are the columns numbers from left to right, the the first word is the name of the columns
    Position             = 2,
    SNP                  = 3,
    "Reference allele"   = 4,                         # if the name of the columns is more than one word, we have to use " "
    "Alternative allele" = 5,
    Effect               = 6,
    Type                 = 7,
    Biotype              = 8,
    "Nucleotide change"  = 9,
    "Aminoacid change"   = 10,
    GeneID               = 11
  ) |>
  mutate(
    Scaffold             = as.character(              # make scaffold as character, we will manipulate it later
      Scaffold
    )
  )
# sendond file
snps_immunity2 <-
  read_delim(
    here(
      "output", "SnpEff", "snps_immunity_genes_2.txt"
    ),
    col_names            = FALSE,
    show_col_types       = FALSE,
  ) |>
  rename(
    Scaffold             = 1,
    Position             = 2,
    SNP                  = 3,
    "Reference allele"   = 4,
    "Alternative allele" = 5,
    Effect               = 6,
    Type                 = 7,
    Biotype              = 8,
    "Nucleotide change"  = 9,
    "Aminoacid change"   = 10,
    GeneID               = 11
  ) |>
  mutate(
    "Aminoacid change"   = " "           # adds a columns since SNPs on introns don't cause aa changes
  ) |>
  mutate(
    Scaffold = as.character(            # make scaffold as character, we will manipulate it later
      Scaffold
    )
  )
#
# rbind them
snps_immunity3 <-
  rbind(                                # use rbind combine the objects
    snps_immunity1, snps_immunity2      # the name of the objects
  ) |>
  arrange(                              # use arrange to sort by Scaffold and Type
    Scaffold, Type
  ) |>
  mutate(                               # use mutate to change geneIDs
    GeneID = str_remove(                # use library stringr to remove strings
      GeneID, "gene-"                   # we remove gene- since some genes have the extra word
    )
  ) |>
  mutate_at(                            # we use mutate_at to only make changes in one column
    vars(                               # to select column or variable
      "Aminoacid change"                # name of the column
    ), funs(                            # list function calls
      replace(                          # to replace
        ., !grepl(                      # we use !grepl to remove fields without the pattern, we only want nonsynonymous changes starting with p
          "p", .                        # all amino acid changes have the letter p on them, so we remove those without it
        ),
        ""                              # here we replace it with nothing using ''
      )
    )
  ) |>
  mutate(
  Effect   = tolower(                   # we use base R to convert all values on column Effect to lower case
    Effect
  )
  ) |>
  mutate(
    Effect = str_to_sentence(           # here we use library stringr to convert the first letter to upper case, just like in a sentence
      Effect
    )
  )
#
#
# make table
ftai.snps <-
  flextable(
    snps_immunity3 |>
      filter(                          # the table would be too long (1,689 SNPs on immunity genes)
        Type == "missense_variant"
      )
  ) |>
  set_caption(
    as_paragraph(
      as_chunk(
        "SNPs resulting in nonsynonymous changes in immunity genes",
        props         = fp_text_default(
          font.family = "Cambria"
        )
      )
    ),
    word_stylename    = "Table Caption"
  )
#
# Apply zebra theme
ftai.snps <- flextable::theme_zebra(ftai.snps)
# print(ftab.snps, preview = "docx") # to open and see it in Microsoft Word.
# it is a long table, we have almost 1700 SNPs on immunity genes.
ftai.snps |>
  autofit()
```

SNPs resulting in nonsynonymous changes in immunity genes

| Scaffold | Position | SNP | Reference allele | Alternative allele | Effect | Type | Biotype | Nucleotide change | Aminoacid change | GeneID |
| --- | --- | --- | --- | --- | --- | --- | --- | --- | --- | --- |
| 1.1 | 10,697,741 | AX-583056929 | G | A | Moderate | missense\_variant | protein\_coding | c.491G>A | p.Arg164His | exon-XM\_029855176.1-1 |
| 1.1 | 10,699,650 | AX-583058970 | A | C | Moderate | missense\_variant | protein\_coding | c.196T>G | p.Ser66Ala | LOC109430086 |
| 1.106 | 2,512,509 | AX-583109537 | C | A | Moderate | missense\_variant | protein\_coding | c.1405C>A | p.His469Asn | LOC115257625 |
| 1.113 | 5,762,762 | AX-583151909 | A | G | Moderate | missense\_variant | protein\_coding | c.623A>G | p.Glu208Gly | LOC109415428 |
| 1.113 | 5,763,562 | AX-583152046 | A | G | Moderate | missense\_variant | protein\_coding | c.1423A>G | p.Ile475Val | LOC109415428 |
| 1.131 | 5,279,847 | AX-583254712 | C | T | Moderate | missense\_variant | protein\_coding | c.626G>A | p.Ser209Asn | LOC109399009 |
| 1.147 | 5,839,558 | AX-583431723 | T | C | Moderate | missense\_variant | protein\_coding | c.775A>G | p.Ile259Val | exon-XM\_029868010.1-1 |
| 1.147 | 5,840,353 | AX-583434252 | T | G | Moderate | missense\_variant | protein\_coding | c.193A>C | p.Thr65Pro | exon-XM\_029868010.1-1 |
| 1.25 | 1,021,102 | AX-583588144 | A | G | Moderate | missense\_variant | protein\_coding | c.424A>G | p.Lys142Glu | LOC109402615 |
| 1.36 | 4,880,818 | AX-583694151 | T | C | Moderate | missense\_variant | protein\_coding | c.445A>G | p.Lys149Glu | LOC109397206 |
| 1.79 | 54,655 | AX-583972510 | G | A | Moderate | missense\_variant | protein\_coding | c.599C>T | p.Ser200Leu | LOC115260154 |
| 1.85 | 17,046,403 | AX-584044965 | C | T | Moderate | missense\_variant | protein\_coding | c.524C>T | p.Thr175Met | LOC109424643 |
| 1.85 | 23,680,098 | AX-584075828 | A | G | Moderate | missense\_variant | protein\_coding | c.4819A>G | p.Lys1607Glu | exon-XM\_029862069.1-1 |
| 1.85 | 23,681,315 | AX-584075910 | A | G | Moderate | missense\_variant | protein\_coding | c.5722A>G | p.Met1908Val | exon-XM\_029862069.1-1 |
| 1.85 | 27,167,461 | AX-584094434 | G | C | Moderate | missense\_variant | protein\_coding | c.27C>G | p.Asp9Glu | LOC109431512 |
| 1.91 | 3,380,846 | AX-584233670 | A | G | Moderate | missense\_variant | protein\_coding | c.2011A>G | p.Asn671Asp | LOC109412409 |
| 1.91 | 5,039,401 | AX-584245979 | A | G | Moderate | missense\_variant | protein\_coding | c.1156A>G | p.Ser386Gly | LOC109430356 |
| 1.91 | 8,897,363 | AX-584263120 | G | T | Moderate | missense\_variant | protein\_coding | c.880C>A | p.Leu294Met | LOC109621626 |
| 1.91 | 8,897,632 | AX-584265058 | A | G | Moderate | missense\_variant | protein\_coding | c.611T>C | p.Met204Thr | LOC109621626 |
| 2.103 | 2,573,871 | AX-584332894 | G | C | Moderate | missense\_variant | protein\_coding | c.471C>G | p.His157Gln | LOC115266575 |
| 2.103 | 2,574,411 | AX-584332912 | A | T | Moderate | missense\_variant | protein\_coding | c.28T>A | p.Ser10Thr | LOC115266575 |
| 2.138 | 335,263 | AX-584531554 | A | G | Moderate | missense\_variant | protein\_coding | c.172A>G | p.Ser58Gly | LOC109409282 |
| 2.14 | 12,158,966 | AX-585060557 | T | C | Moderate | missense\_variant | protein\_coding | c.7A>G | p.Thr3Ala | exon-XM\_029859293.1-1 |
| 2.14 | 12,647,106 | AX-584552253 | G | C | Moderate | missense\_variant | protein\_coding | c.964G>C | p.Gly322Arg | LOC109409279 |
| 2.146 | 419,711 | AX-585089323 | A | T | Moderate | missense\_variant | protein\_coding | c.701A>T | p.Asp234Val | LOC109407295 |
| 2.167 | 1,433,370 | AX-585168461 | G | A | Moderate | missense\_variant | protein\_coding | c.199G>A | p.Val67Ile | exon-XM\_029853094.1-1 |
| 2.17 | 3,924,782 | AX-585190434 | G | A | Moderate | missense\_variant | protein\_coding | c.587G>A | p.Arg196Gln | exon-XM\_019684995.2-1 |
| 2.17 | 3,926,378 | AX-584681782 | C | G | Moderate | missense\_variant | protein\_coding | c.2050C>G | p.Pro684Ala | exon-XM\_019684995.2-1 |
| 2.17 | 4,004,422 | AX-584682128 | C | T | Moderate | missense\_variant | protein\_coding | c.11C>T | p.Thr4Met | exon-XM\_019684993.2-1 |
| 2.17 | 13,726,115 | AX-582441613 | T | C | Moderate | missense\_variant | protein\_coding | c.671A>G | p.Lys224Arg | LOC109410220 |
| 2.175 | 780,098 | AX-585206011 | C | T | Moderate | missense\_variant | protein\_coding | c.956C>T | p.Ser319Leu | exon-XM\_029852847.1-1 |
| 2.175 | 800,927 | AX-584697392 | C | A | Moderate | missense\_variant | protein\_coding | c.3090C>A | p.Ser1030Arg | exon-XM\_029852847.1-1 |
| 2.27 | 1,560,585 | AX-579525195 | A | T | Moderate | missense\_variant | protein\_coding | c.1326T>A | p.Asp442Glu | LOC109419663 |
| 2.42 | 12,014,338 | AX-579673469 | T | A | Moderate | missense\_variant | protein\_coding | c.961T>A | p.Ser321Thr | LOC109426828 |
| 2.6 | 16,276,549 | AX-579809339 | T | A | Moderate | missense\_variant | protein\_coding | c.490T>A | p.Ser164Thr | exon-XM\_029878835.1-1 |
| 2.6 | 16,296,793 | AX-579809489 | C | T | Moderate | missense\_variant | protein\_coding | c.586C>T | p.Pro196Ser | exon-XM\_019700969.2-1 |
| 2.68 | 5,446,027 | AX-579896611 | G | T | Moderate | missense\_variant | protein\_coding | c.1060C>A | p.Leu354Ile | LOC109622615 |
| 2.68 | 5,446,516 | AX-579896667 | A | G | Moderate | missense\_variant | protein\_coding | c.571T>C | p.Trp191Arg | LOC109622615 |
| 2.87 | 2,149,902 | AX-580019993 | G | A | Moderate | missense\_variant | protein\_coding | c.49G>A | p.Ala17Thr | LOC109398084 |
| 2.87 | 2,150,355 | AX-580020020 | A | G | Moderate | missense\_variant | protein\_coding | c.370A>G | p.Ile124Val | LOC109398084 |
| 2.87 | 2,174,851 | AX-580020260 | A | G | Moderate | missense\_variant | protein\_coding | c.1162A>G | p.Thr388Ala | LOC109398030 |
| 2.95 | 352,322 | AX-580191265 | T | A | Moderate | missense\_variant | protein\_coding | c.109A>T | p.Thr37Ser | LOC115269749 |
| 3.116 | 5,061,100 | AX-580503305 | T | C | Moderate | missense\_variant | protein\_coding | c.619A>G | p.Arg207Gly | exon-XM\_029857358.1-1 |
| 3.122 | 5,261,799 | AX-580598894 | A | C | Moderate | missense\_variant | protein\_coding | c.801T>G | p.Asp267Glu | exon-XM\_029865713.1-1 |
| 3.151 | 3,436,049 | AX-580842317 | T | C | Moderate | missense\_variant | protein\_coding | c.1045A>G | p.Thr349Ala | exon-XM\_029875170.1-1 |
| 3.159 | 1,104,660 | AX-580902042 | A | G | Moderate | missense\_variant | protein\_coding | c.638T>C | p.Val213Ala | LOC109409956 |
| 3.17 | 18,459,269 | AX-581338723 | T | C | Moderate | missense\_variant | protein\_coding | c.28A>G | p.Thr10Ala | exon-XM\_029875820.1-1 |
| 3.18 | 2,874,117 | AX-581428184 | T | C | Moderate | missense\_variant | protein\_coding | c.1267A>G | p.Ser423Gly | exon-XM\_029875979.1-1 |
| 3.2 | 8,846,714 | AX-581596089 | T | C | Moderate | missense\_variant | protein\_coding | c.287A>G | p.Asn96Ser | LOC109413737 |
| 3.32 | 3,322,424 | AX-581763570 | T | C | Moderate | missense\_variant | protein\_coding | c.1193T>C | p.Val398Ala | LOC109415245 |
| 3.32 | 3,408,703 | AX-581765674 | G | A | Moderate | missense\_variant | protein\_coding | c.592G>A | p.Gly198Ser | LOC109414897 |
| 3.32 | 3,418,991 | AX-581765806 | T | G | Moderate | missense\_variant | protein\_coding | c.324T>G | p.Asp108Glu | LOC109416024 |
| 3.4 | 5,487,127 | AX-581830792 | T | A | Moderate | missense\_variant | protein\_coding | c.295A>T | p.Thr99Ser | LOC109399310 |
| 3.58 | 5,278,791 | AX-582051662 | C | A | Moderate | missense\_variant | protein\_coding | c.316G>T | p.Ala106Ser | exon-XM\_019673611.2-1 |
| 3.75 | 16,587,391 | AX-582760777 | A | G | Moderate | missense\_variant | protein\_coding | c.334A>G | p.Asn112Asp | LOC109398879 |
| 3.75 | 16,589,393 | AX-582761776 | C | T | Moderate | missense\_variant | protein\_coding | c.317G>A | p.Arg106Lys | LOC109398826 |
| 3.77 | 6,092,180 | AX-582825108 | G | A | Moderate | missense\_variant | protein\_coding | c.92G>A | p.Arg31Lys | LOC109420317 |
| 3.93 | 5,614,846 | AX-583002481 | G | A | Moderate | missense\_variant | protein\_coding | c.1039G>A | p.Asp347Asn | exon-XM\_029860991.1-1 |

```
#
# save it
# create settings - check ??save_as_docx on console
sect_properties <- prop_section(
  page_size    = page_size(
    orient     = "landscape",
    width      = 8.3, height = 11.7
  ),
  type         = "continuous",
  page_margins = page_mar()
)
# save
# then you can open MS Word and make adjustments
ftai.snps |>
  save_as_docx(
    path       = here(
      "output", "SnpEff", "SNPs_nonsynonymous_immunity_genes.docx"
    ),
    pr_section = sect_properties
  )
```

### 5.3 Summary of SNPs on diapause and immunity genes

We can also get the sum totals for the type of SNPs we have in
diapause and immunity genes

```
# row bind both tibbles
diap_immun <-
  bind_rows(                       # we use bind_rows and not rbind from base R - both would work here (same number of columns)
    snps_diapause3, snps_immunity3 # the objects we want to bind
  ) |>
  count(                           # here we count based on the column Type
    Type,
    sort = TRUE                    # we sort high > low
  ) |>
  filter(                          # here we filter n > 90 because we only want to show the types with more SNPs, and the other ones are mixed types
    n > 90
  ) |>
  mutate_if(                       # here we replace all underscores with spaces
    is.character,                  # all columns as characters
    str_replace_all,               # replace all
    "_",                           # underscore
    " "                            # with space
  ) |>
    mutate(
    Type = str_to_sentence(        # here we use library stringr to convert the first letter to upper case, just like in a sentence
      Type
    )
  ) |>
  rename(                          # we can finally rename the columns
    "SNP effect" = 1,
    "Number"     = 2
  ) |>
  mutate_if(                       # here we replace Missense with Synonymous
    is.character,                  # all columns as characters
    str_replace_all,               # replace all
    "Missense",                    # 
    "Nonsynonymous"
  ) |>
  mutate_if(                       # here we replace prime with 'Synonymous' prime
    is.character,                  # all columns as characters
    str_replace_all,               # replace all
    " prime",
    "' prime"
  )
#
# lets make a table
set_flextable_defaults(na_str = "NA", big.mark = ",")
ftabdi <-
  flextable(
    diap_immun
  ) |>
  set_caption(
    as_paragraph(
      as_chunk(
        "The effects of SNPs from the Chip on immunity and diapause genes",
        props         = fp_text_default(
          font.family = "Cambria"
        )
      )
    ),
    word_stylename    = "Table Caption"
  )
#
# Apply zebra theme
ftabdi <- flextable::theme_zebra(ftabdi)
# print(ftabdi, preview = "docx") # to open and see it in Microsoft Word.
# check the table
ftabdi |>
  autofit()
```

The effects of SNPs from the Chip on immunity and diapause genes

| SNP effect | Number |
| --- | --- |
| Synonymous variant | 415 |
| Intron variant | 409 |
| 5' prime utr variant | 91 |

```
#
# save it
ftabdi |>
  autofit() |>
  save_as_docx(
    path              = here(
      "output",
      "SnpEff",
      "summary_SNP_effect_diapause_immunity_genes.docx"
    )
  )
```

## 6. WGS variants functional annotation

To get an idea of the variant distribution across the genome of Ae.
albopictus, I will do the functional annotation using the WGS data set I
used to select variants for the chip. The initial data set had around 34
million variants, but after MAF filter of 10%, we obtained a data set
with approximately 2.5 million variants. Because we do not have
permission to share the WGS data set, I will not include it in the
repository.

```
# the data is located at
/ycga-gpfs/project/caccone/lvc26/september_2020/snp_calls/chunk_calls/plink/merged_files/albo_MAF_0.01
#
# our first step is to set reference allele as the reference genome, and covert to vcf using Plink2
# the genome is at
/ycga-gpfs/project/caccone/lvc26/september_2020/genome/aedes_albopictus_LA2_20200826.fasta
#
# create interactive session with 2 CPU
srun --pty -N 1 -n 1 -c 2 -p interactive zsh
#
# Plink2 
export PATH=$PATH:/ycga-gpfs/project/caccone/lvc26/plink2
#
# get plink2 version
plink2 --version
# PLINK v2.00a3.6LM 64-bit Intel (14 Aug 2022)
```

```
## bash: line 1: /ycga-gpfs/project/caccone/lvc26/september_2020/snp_calls/chunk_calls/plink/merged_files/albo_MAF_0.01: No such file or directory
## bash: line 5: /ycga-gpfs/project/caccone/lvc26/september_2020/genome/aedes_albopictus_LA2_20200826.fasta: No such file or directory
## bash: line 8: srun: command not found
## PLINK v2.00a3.3 64-bit (3 Jun 2022)
```

On the cluster, set reference allele, and make vcf file

```
# Plink 1.x sets major alleles as reference alleles, therefore it can change based on the allele frequency. We can use the reference genome with Plink to set the reference alleles correctly.
#
# MAF 1% is the default of plink.
##  ............................................................................
##  MAF 1%                                                                  ####
# MAF 0.01 - default plink
plink2 \
--bfile /ycga-gpfs/project/caccone/lvc26/september_2020/snp_calls/chunk_calls/plink/merged_files/albo_MAF_0.01 \
--allow-extra-chr \
--fa /ycga-gpfs/project/caccone/lvc26/september_2020/genome/aedes_albopictus_LA2_20200826.fasta \
--ref-from-fa 'force'  \
--make-bed \
--export vcf id-delim='-' ref-first bgz \
--out /ycga-gpfs/project/caccone/lvc26/SnpEff/albo_wgs \
--silent;
grep 'variants' /ycga-gpfs/project/caccone/lvc26/SnpEff/albo_wgs.log
#
# 24616205 variants loaded from
# --ref-from-fa force: 720711 variants changed, 23895494 validated.
#
##  ............................................................................
##  MAF 5%                                                                  ####
plink2 \
--bfile /ycga-gpfs/project/caccone/lvc26/september_2020/snp_calls/chunk_calls/plink/merged_files/albo_MAF_0.01 \
--allow-extra-chr \
--fa /ycga-gpfs/project/caccone/lvc26/september_2020/genome/aedes_albopictus_LA2_20200826.fasta \
--ref-from-fa 'force'  \
--make-bed \
--maf 0.05 \
--export vcf id-delim='-' ref-first bgz \
--out /ycga-gpfs/project/caccone/lvc26/SnpEff/albo_wgs_MAF_0.05 \
--silent;
grep 'variants' /ycga-gpfs/project/caccone/lvc26/SnpEff/albo_wgs_MAF_0.05.log
#
# 24616205 variants loaded from
# 18671088 variants removed due to allele frequency threshold(s)
# 5945117 variants remaining after main filters.
# --ref-from-fa force: 606703 variants changed, 5338414 validated.
#
##  ............................................................................
##  MAF 10%                                                                 ####
plink2 \
--bfile /ycga-gpfs/project/caccone/lvc26/september_2020/snp_calls/chunk_calls/plink/merged_files/albo_MAF_0.01 \
--allow-extra-chr \
--fa /ycga-gpfs/project/caccone/lvc26/september_2020/genome/aedes_albopictus_LA2_20200826.fasta \
--ref-from-fa 'force'  \
--make-bed \
--maf 0.1 \
--export vcf id-delim='-' ref-first bgz \
--out /ycga-gpfs/project/caccone/lvc26/SnpEff/albo_wgs_MAF_0.1 \
--silent;
grep 'variants' /ycga-gpfs/project/caccone/lvc26/SnpEff/albo_wgs_MAF_0.1.log
#
# 24616205 variants loaded from
# 21819313 variants removed due to allele frequency threshold(s)
# 2796892 variants remaining after main filters.
# --ref-from-fa force: 503613 variants changed, 2293279 validated.
#
##  ............................................................................
##  MAF 15%                                                                 ####
plink2 \
--bfile /ycga-gpfs/project/caccone/lvc26/september_2020/snp_calls/chunk_calls/plink/merged_files/albo_MAF_0.01 \
--allow-extra-chr \
--fa /ycga-gpfs/project/caccone/lvc26/september_2020/genome/aedes_albopictus_LA2_20200826.fasta \
--ref-from-fa 'force'  \
--make-bed \
--maf 0.15 \
--export vcf id-delim='-' ref-first bgz \
--out /ycga-gpfs/project/caccone/lvc26/SnpEff/albo_wgs_MAF_0.15 \
--silent;
grep 'variants' /ycga-gpfs/project/caccone/lvc26/SnpEff/albo_wgs_MAF_0.15.log
#
# 24616205 variants loaded from
# 22917110 variants removed due to allele frequency threshold(s)
# 1699095 variants remaining after main filters.
# --ref-from-fa force: 422154 variants changed, 1276941 validated.
#
##  ............................................................................
##  MAF 25%                                                                 ####
plink2 \
--bfile /ycga-gpfs/project/caccone/lvc26/september_2020/snp_calls/chunk_calls/plink/merged_files/albo_MAF_0.01 \
--allow-extra-chr \
--fa /ycga-gpfs/project/caccone/lvc26/september_2020/genome/aedes_albopictus_LA2_20200826.fasta \
--ref-from-fa 'force'  \
--make-bed \
--maf 0.20 \
--export vcf id-delim='-' ref-first bgz \
--out /ycga-gpfs/project/caccone/lvc26/SnpEff/albo_wgs_MAF_0.20 \
--silent;
grep 'variants' /ycga-gpfs/project/caccone/lvc26/SnpEff/albo_wgs_MAF_0.20.log
#
# 24616205 variants loaded from
# 23446040 variants removed due to allele frequency threshold(s)
# 1170165 variants remaining after main filters.
# --ref-from-fa force: 351474 variants changed, 818691 validated.
```

Transfer data to SSD

```
# run this command in the terminal and not R studio
# I put transferred the data to the external SSD drive because it more than 8Gb
rsync -chavzP --stats lvc26@ruddle.hpc.yale.edu:/ycga-gpfs/project/caccone/lvc26/SnpEff/ /Volumes/evo2/data_chip_design
```

6.1 Run annotation

```
# on SSD
##  ............................................................................
##  MAF 1%                                                                  ####
# then annotate the vcf with the v4
# we first run annotation for MAF 0.01
java -Xmx8g -jar /Users/lucianocosme/Packages/snpEff/snpEff.jar albo_v3 \
/Volumes/evo2/data_chip_design/albo_wgs.vcf.gz \
-v \
-stats /Volumes/evo2/data_chip_design/MAF_0.01.html \
-csvStats /Volumes/evo2/data_chip_design/MAF_0.01.csv \
> /Volumes/evo2/data_chip_design/albo_wgs.ann_MAF_0.01.vcf
#
# we will get some warnings or errors because the annotation was lifted from AalbF2 to the AalbF3, with some transcripts missing some features.
# therefore we need to focus on genes with id starting with LOC
#
# because I need to run multiple annotations, I used the cluster to do the annotations, the bottleneck was the storage, the 2Tb SSD did not have enough space to save all the vcfs.
# 
# on the cluster
srun --pty -N 1 -n 1 -c 2 -p interactive zsh
module load GATK/4.2.6.1-GCCcore-10.2.0-Java-11 # for java to be loaded
java -Xmx8g -jar $HOME/snpEff/snpEff.jar --help
#
##  ............................................................................
##  MAF 1%                                                                  ####
# then annotate the vcf with the v4
# we first run annotation for MAF 0.01
java -Xmx10g -jar $HOME/snpEff/snpEff.jar albo_v3 \
/ycga-gpfs/project/caccone/lvc26/SnpEff/albo_wgs.vcf.gz \
-v \
-stats /ycga-gpfs/project/caccone/lvc26/SnpEff/MAF_0.01.html \
-csvStats /ycga-gpfs/project/caccone/lvc26/SnpEff/MAF_0.01.csv \
> /ycga-gpfs/project/caccone/lvc26/SnpEff/albo_wgs.ann_MAF_0.01.vcf
##  ............................................................................
##  MAF 5%                                                                  ####
# then annotate the vcf with the v4
# we first run annotation for MAF 0.05
java -Xmx8g -jar /Users/lucianocosme/Packages/snpEff/snpEff.jar albo_v3 \
/Volumes/evo2/data_chip_design/albo_wgs_MAF_0.05.vcf.gz \
-v \
-stats /Volumes/evo2/data_chip_design/MAF_0.05.html \
-csvStats /Volumes/evo2/data_chip_design/MAF_0.05.csv \
> /Volumes/evo2/data_chip_design/albo_wgs.ann_MAF_0.05.vcf
#
#
##  ............................................................................
##  MAF 10%                                                                 ####
# then annotate the vcf with the v4
# we first run annotation for MAF 0.1
java -Xmx8g -jar /Users/lucianocosme/Packages/snpEff/snpEff.jar albo_v3 /Volumes/evo2/data_chip_design/albo_wgs_MAF_0.1.vcf.gz \
-v \
-stats /Volumes/evo2/data_chip_design/MAF_0.1.html \
-csvStats /Volumes/evo2/data_chip_design/MAF_0.1.csv \
> /Volumes/evo2/data_chip_design/albo_wgs.ann_MAF_0.1.vcf
#
#
##  ............................................................................
##  MAF 15%                                                                 ####
# then annotate the vcf with the v4
# we first run annotation for MAF 0.15
java -Xmx8g -jar /Users/lucianocosme/Packages/snpEff/snpEff.jar albo_v3 \
/Volumes/evo2/data_chip_design/albo_wgs_MAF_0.15.vcf.gz \
-v \
-stats /Volumes/evo2/data_chip_design/MAF_0.15.html \
-csvStats /Volumes/evo2/data_chip_design/MAF_0.15.csv \
> /Volumes/evo2/data_chip_design/albo_wgs.ann_MAF_0.15.vcf
#
#
##  ............................................................................
##  MAF 20%                                                                ####
# then annotate the vcf with the v4
# we first run annotation for MAF 0.2
java -Xmx8g -jar /Users/lucianocosme/Packages/snpEff/snpEff.jar albo_v3 \
/Volumes/evo2/data_chip_design/albo_wgs_MAF_0.20.vcf.gz \
-v \
-stats /Volumes/evo2/data_chip_design/MAF_0.2.html \
-csvStats /Volumes/evo2/data_chip_design/MAF_0.2.csv \
> /Volumes/evo2/data_chip_design/albo_wgs.ann_MAF_0.2.vcf
#
```

Move files to output

```
# SnpEff will create a .txt and a .html file in our project root directory, we can move them to the right place
mv snpEff_* output/SnpEff/
# you can double click the file snpEff_summary.html to see the summary of our annotation
```

Get types of variants

```
# I wrote a bash script to get the number of variants we have, you might have to run chmod 775 if you get permission denied
./scripts/analysis/variants_by_effect.sh /Volumes/evo2/data_chip_design/albo_wgs.ann_MAF_0.1.vcf
```

Counting variats from
/Volumes/evo2/data\_chip\_design/albo\_wgs.ann\_MAF\_0.1.vcf Nonsynonymous:
67217 Synonymous: 384482 Intron: 1281482 Intergenic: 993796 3’UTR: 64912
5’UTR: 67744

Alternatively, we can create a file with the effects and how they are
annotated in the vcf file

```
echo "Nonsynonymous missense_variant
Synonymous synonymous_variant
Intron intron_variant
Intergenic intergenic_region
3UTR 3_prime_UTR_variant
5UTR 5_prime_UTR" > output/SnpEff/variant_types.txt
```

Count effects of variants

```
# not the most elegant but works
cat output/SnpEff/variant_types.txt | while IFS=" " read -r variant effect
do
  echo "$variant"; cat /Volumes/evo2/data_chip_design/albo_wgs.ann_MAF_0.1.vcf | sed '/##/d' | grep -c "$effect"
done | xargs -n2 > output/SnpEff/wgs_annotations_summary_MAF_0.1.txt
```

Make a table

```
# import data
wgs_var_effects <- read_delim(
  here(
    "output", "SnpEff", "wgs_annotations_summary_MAF_0.1.txt"
  ),
  col_names           = FALSE,
  show_col_types      = FALSE,
  col_types           = "cn"
) |>
  rename(
    Effect            = 1,
    Number            = 2
  ) |>
  mutate(
    Effect = as.factor(
      Effect
    ),
    Percentage = prop.table( # we use prop.table to calculate percentages
      Number
    ) * 100,
    across(        # we use across to set column 3 as 2 decimals
      3, round, 2
    )
  ) |>
  arrange(     # we want to sort column percentage from higher to low 
    -Percentage
  )
#
# make table
ftabv <- flextable(
  wgs_var_effects |>
  rename(
    Effect            = 1,
    "Number of SNPs"  = 2,
    Percentage        = 3
  )
) |>
  set_caption(
    as_paragraph(
      as_chunk(
        "Variant effects for 2,796,892 SNPs from WGS data set - MAF 10%",
        props         = fp_text_default(
          font.family = "Cambria"
        )
      )
    ),
    word_stylename    = "Table Caption"
  )
#

# Apply zebra theme
ftabv <- flextable::theme_zebra(ftabv)
# print(ftab, preview = "docx") # to open and see it in Microsoft Word.
ftabv
```

Variant effects for 2,796,892 SNPs from WGS data set - MAF 10%

| Effect | Number of SNPs | Percentage |
| --- | --- | --- |
| Intron | 1,281,482 | 44.81 |
| Intergenic | 993,796 | 34.75 |
| Synonymous | 384,482 | 13.45 |
| 5UTR | 67,744 | 2.37 |
| Nonsynonymous | 67,217 | 2.35 |
| 3UTR | 64,912 | 2.27 |

```
# save
# then you can open MS Word and make adjustments
ftabv |>
  autofit() |>
  save_as_docx(
    path = here(
      "output", "SnpEff", "wgs_effect_type_and_number_SNPs_MAF_0.1.docx"
    )
  )
```

Plot of the effects

```
# create vector of colors for our plot
# option1
# special_colors <- list(
#   "Intron"        = "#BBBBBB",
#   "Intergenic"    = "#0072b2",
#   "Synonymous"    = "#009e73",
#   "5UTR"          = "#56b4e9",
#   "3UTR"          = "#cc79a7",
#   "Nonsynonymous" = "#d55e00"
# )
# 
# Option 2
special_colors <- list(
  "Intron"        = "#C1CDCD",
  "Intergenic"    = "#1AC722",
  "Synonymous"    = "#FF8C00",
  "5UTR"          = "#87CEFA",
  "3UTR"          = "#FF3030",
  "Nonsynonymous" = "pink"
)
#
# make plot
wgs_var_effects |>
    mutate(                                 # we can use mutate to change the class of column
    Effect            = as.factor(          # we make column "Effect" factor
      Effect
      ),
    Effect            = fct_reorder(        # library forcats to re-order by the number of variants
      Effect, Number
    )
  ) |>
  ggplot() +
  geom_col(                                 # to make bar plot with different colors
    aes(                                    # mapping
      x               = Effect,
      y               = Number,
      fill            = Effect
    ),
    color             = "yellow"            # for the border
  ) +
  labs(                                     # to change the ylab and tittle
    title             = "WGS variants per SnpEff annotation",
    y                 = "Number of annotated variants",
    x                 = "Annotated effect",
    caption           = "Functional annotation of SNPs in WGS data set (~2.8m - MAF 10%)"
  ) +
  hrbrthemes::theme_ipsum(                  # I like this theme, but you can use any
    base_family       = "", # needs to have extrafont library
    axis_text_size    = 12,
    axis_title_size   = 14,
    plot_margin       = margin(
      10, 10, 10, 10
    ),
    grid              = TRUE,
    grid_col          = "#fabbe2"
  ) +
  scale_fill_manual(
    values            = unlist(
    special_colors
    )
  ) +
  scale_color_manual(
    values            = unlist(
    special_colors
    )
  ) +
  theme(
    legend.position   = "none",
    panel.grid.major  = element_line(
      linetype        = "dashed",
      linewidth       = 0.2,
    ),
    panel.grid.minor  = element_line(
      linetype        = "dashed",
      linewidth       = 0.2
    )
  ) +
  scale_y_continuous(
        trans         = "log10",
        breaks        = scales::log_breaks(
          n           = 7
        ),
        labels        = label_comma()
    ) +
  annotation_logticks(
    sides             = "b"
  ) +
  geom_label(
    aes(
      x               = Effect,
      y               = Number,
      color           = Effect,
      label           = paste(
        formatC(
          Number,
          format      = "f", 
          big.mark    = ",",
          digits      = 0
          ),"\n","(",Percentage,"%)"
      )
    ),
    face              = "bold",
    size              = 2,
    family            = "Roboto Condensed",
    alpha             = .9,
  ) +
  coord_flip()                             # to make it easier to read
```

```
#
# save plot
ggsave(
  here(
    "output", "SnpEff", "figures", "wgs_SNPs_effect_chip.pdf"
  ),
  width               = 9,
  height              = 5,
  units               = "in"
)
```

## 7. Compare WGS and Chip variants by effect

```
# chip data
chip_data <- read_delim(
  here(
    "output", "SnpEff", "annotations_summary.txt"
  ),
  col_names           = FALSE,
  show_col_types      = FALSE,
  col_types           = "cn"
) |>
  rename(
    Effect            = 1,
    Number            = 2
  ) |>
  mutate(
    Effect            = as.factor(
      Effect
    ),
    Percentage        = prop.table(              # we use prop.table to calculate percentages
      Number
    ) * 100,
    across(                                      # we use acrros to set column 3 as 2 decimals
      3, round, 2
    )
  ) |>
  arrange(                                       # we want to sort column percentage from higher to low
    -Percentage
  ) |>
  mutate(Data         = "Chip")
#
# wgs data
wgs_data <- read_delim(
  here(
    "output", "SnpEff", "wgs_annotations_summary_MAF_0.1.txt"
  ),
  col_names           = FALSE,
  show_col_types      = FALSE,
  col_types           = "cn"
) |>
  rename(
    Effect            = 1,
    Number            = 2
  ) |>
  mutate(
    Effect            = as.factor(
      Effect
    ),
    Percentage        = prop.table(              # we use prop.table to calculate percentages
      Number
    ) * 100,
    across(                                      # we use across to set column 3 as 2 decimals
      3, round, 2
    )
  ) |>
  arrange(                                       # we want to sort column percentage from higher to low
    -Percentage
  ) |>
  mutate(Data         = "WGS")
#
# merge data sets
wgs_chip_data <-
    rbind(
  chip_data, wgs_data
)
```

Create plot

```
# 
# plot it
wgs_chip_data |>
    mutate(                                      # we can use mutate to change the class of column
    Effect            = as.factor(               # we make column "Effect" factor
      Effect
      ),
    Effect            = fct_reorder(             # library forcats to re-order by the number of variants
      Effect, Number
    )
  ) |>
  ggplot() +
  geom_col(                                      # to make bar plot with different colors
    aes(                                         # mapping
      x               = Effect,
      y               = Number,
      color           = Effect,
      fill            = Data
    ),
    show.legend = TRUE,
    position = position_dodge(width = 0.7),
    width = .6,
    linewidth = .01,
    alpha=0.8
  ) +
  labs(                                          # to change the ylab and tittle
    title             = "Variants effects per SnpEff annotation",
    y                 = bquote(bold(.("Number of annotated variants effects"~(Log[10])))),
    x                 = "Annotated effect",
    caption           = "Functional annotation of WGS SNPs used for chip design (~2.8m) and Chip recovered SNPs (~60k) - MAF = 0.1"
  ) +
  hrbrthemes::theme_ipsum(                       # I like this theme, but you can use any
    base_family            = "", # needs to have extrafont library
    axis_text_size         = 12,
    axis_title_size        = 14,
    plot_margin            = margin(
      10, 10, 10, 10
    ),
    grid                   = TRUE,
    grid_col               = "#fabbe2"
  ) +
  scale_color_manual(
    name                   = "Effect",
    breaks                 = c(
      "Intron", "Intergenic", "Synonymous", "5UTR", "3UTR", "Nonsynonymous"
    ),
    values                 = c(
      "Intron"             = "#7A7070",
      "Intergenic"         = "#4674D9",
      "Synonymous"         = "#17D13C",
      "5UTR"               = "purple",
      "3UTR"               = "#F7AB1E",
      "Nonsynonymous"      = "#ED1717"
    ),
    guide                  = "none"
  ) +  
  scale_fill_manual(
   name                    = "Data",
   breaks                  = c(
     "WGS", "Chip"
   ),
   values                  = c(
     "Chip"                = "#bab9b1",
     "WGS"                 = "#d2f59e"
   )
 ) +
  theme(
    axis.text.y            = element_text(
      colour               = "black"
      ),
    axis.title.y           = element_text(
      colour               = "black",
      face                 = "bold"
      ),
    legend.position        = "right",
    panel.grid.major       = element_line(
      linetype             = "dashed",
      linewidth            = 0.2,
    ),
    panel.grid.minor       = element_line(
      linetype             = "dashed",
      linewidth            = 0.2
    )
  ) +
  scale_y_continuous(
    trans                  = "log10",
    breaks                 = scales::log_breaks(
      n                    = 7
    ),
    labels                 = label_comma()
  ) +
    annotation_logticks(
      sides                = "b"
    ) +
  geom_label(
    aes(
      x                    = Effect,
      y                    = Number,
      color                = Effect,
      label                = paste(
        formatC(
          Number,
          format           = "f", 
          big.mark         = ",",
          digits           = 0
        ), "\n", "(", Percentage, "%)"
      )
    ),
    fill                   = alpha(
      c(
        "white"
      ),
      0.8
    ),
    size                   = 2.5,
    family                 = "Roboto Condensed",
    label.size             = NA,                 # to remove border
    nudge_x                = -.05
  ) +
    coord_flip()                                 # to make it easier to read
```

```
  #
# save plot
ggsave(
  here(
    "output", "SnpEff","figures", "wgs_vs_chip_SnpEff.pdf"
  ),
  width                    = 8,
  height                   = 5,
  units                    = "in"
)
```

We can make a table with the percentage differences

```
# calculate the differences
percetage_diff <-
  wgs_data |>
  left_join(              # use left join to join by column Effect (common column)
    chip_data,
    by               = "Effect",
    suffix           = c( # we add wgs and chip to keep track of data
      ".wgs", ".chip"
    ),
  ) |>
  mutate(                 # we create a new column and calculate the difference
    difference       =  Percentage.chip - Percentage.wgs
  ) |>
  select(                 # we select only the columns we want in our table
    Effect, Number.wgs, Percentage.wgs, Number.chip, Percentage.chip, difference
  ) |>
  rename(                 # we rename the columns by index
    "Variant effect" = 1,
    "WGS (N)"        = 2,
    "WGS (%)"        = 3,
    "Chip (N)"       = 4,
    "Chip (%)"       = 5,
    "Chip bias (%)"  = 6
  )
#
# make table
ftab_diff <-
  flextable(
    percetage_diff
  ) |>
  set_caption(
    as_paragraph(
      as_chunk(
        "Percentage of variants by their effects in WGS and chip data sets (%)",
        props      = fp_text_default(
          font.fam = "Cambria"
        )
      )
    ),
    word_stylena   = "Table Caption"
  )
#

# Apply zebra theme
ftab_diff <- flextable::theme_zebra(ftab_diff)
# print(ftab.snps, preview = "docx") # to open and see it in Microsoft Word.
ftab_diff |>
  autofit()
```

Percentage of variants by their effects in WGS and chip data sets (%)

| Variant effect | WGS (N) | WGS (%) | Chip (N) | Chip (%) | Chip bias (%) |
| --- | --- | --- | --- | --- | --- |
| Intron | 1,281,482 | 44.81 | 24,387 | 39.49 | -5.32 |
| Intergenic | 993,796 | 34.75 | 13,758 | 22.28 | -12.47 |
| Synonymous | 384,482 | 13.45 | 14,323 | 23.20 | 9.75 |
| 5UTR | 67,744 | 2.37 | 3,642 | 5.90 | 3.53 |
| Nonsynonymous | 67,217 | 2.35 | 1,942 | 3.14 | 0.79 |
| 3UTR | 64,912 | 2.27 | 3,697 | 5.99 | 3.72 |

```
#
# save
ftab_diff |>
  autofit() |>
  save_as_docx(
    path       = here(
      "output", "SnpEff", "difference_wgs_chip.docx"
    )
  )
```

## 8. Create pipeline to select random sets of SNPs

We do not know how the differences in the proportion of each variant
by effect in the chip data set affects ancestry analysis. We can create
SNP lists of the chip data matching the proportions of the WGS data set.
For example, in the chip we have bias towards coding regions.

In the WGS data set, SNPs in introns are the highest proportion in
the data set. Therefore, the chip data set should also have SNPs in
introns with the highest proportion. The same idea is valid for other
SNP effect types. Overall, we have an under-representation of variants
in introns (-6.96%) and intergenic regions (-23.30%) in the chip data
set, with an over representation of synonymous (+18.36%), 3’UTR
(-4.47%), 5’UTR (+5.19%), and nonsynonymous (+2.23%). We do not know how
selection is acting on each set of SNPs. Presumably, variants in coding
region may be under stronger selection than those variants in intergenic
regions.

```
# I used Excel to calculate the new proportions of the chip variants to match the WGS proportions
# then I used the RStudio addin datapasta to paste here as tibble, you can also import the Excel file or do it in a different way
new_proportions <- tibble::tribble(
  ~Variant.effect, ~`WGS.(N)`, ~`WGS.(%)`, ~`Chip.(N)`, ~`Chip.(%)`, ~`Chip.bias.(%)`, ~`Chip.Possible.(N)`, ~`Chip.new.(%)`,
         "Intron",    1281482,      44.81,       24387,       39.49,            -5.32,           17741,           44.81,
     "Intergenic",     993796,      34.75,       13758,       22.28,           -12.47,           13758,           34.75,
     "Synonymous",     384482,      13.45,       14323,        23.2,             9.75,            5325,           13.45,
           "5UTR",      67744,       2.37,        3642,         5.9,             3.53,             938,            2.37,
           "3UTR",      64912,       2.27,        3697,        5.99,             3.72,             899,            2.27,
  "Nonsynonymous",      67217,       2.35,        1942,        3.14,             0.79,             930,            2.35,
               NA,    2859633,        100,       61749,         100,                0,           39591,             100
  )

# create table
# make table
ftab_prop <-
  flextable(
    new_proportions
  ) |>
  set_caption(
    as_paragraph(
      as_chunk(
        "New proportion of variants from Chip data set to match WGS variant proportions",
        props      = fp_text_default(
          font.fam = "Cambria"
        )
      )
    ),
    word_stylena   = "Table Caption"
  )
#
# Apply zebra theme
ftab_prop <- flextable::theme_zebra(ftab_prop)
# print(ftab.snps, preview = "docx") # to open and see it in Microsoft Word.
ftab_prop |>
  autofit()
```

New proportion of variants from Chip data set to match WGS variant proportions

| Variant.effect | WGS.(N) | WGS.(%) | Chip.(N) | Chip.(%) | Chip.bias.(%) | Chip.Possible.(N) | Chip.new.(%) |
| --- | --- | --- | --- | --- | --- | --- | --- |
| Intron | 1,281,482 | 44.81 | 24,387 | 39.49 | -5.32 | 17,741 | 44.81 |
| Intergenic | 993,796 | 34.75 | 13,758 | 22.28 | -12.47 | 13,758 | 34.75 |
| Synonymous | 384,482 | 13.45 | 14,323 | 23.20 | 9.75 | 5,325 | 13.45 |
| 5UTR | 67,744 | 2.37 | 3,642 | 5.90 | 3.53 | 938 | 2.37 |
| 3UTR | 64,912 | 2.27 | 3,697 | 5.99 | 3.72 | 899 | 2.27 |
| Nonsynonymous | 67,217 | 2.35 | 1,942 | 3.14 | 0.79 | 930 | 2.35 |
| NA | 2,859,633 | 100.00 | 61,749 | 100.00 | 0.00 | 39,591 | 100.00 |

```
#
# save
ftab_prop |>
  autofit() |>
  save_as_docx(
    path       = here(
      "output", "SnpEff", "new_proportions_chip.docx"
    )
  )
```

The challenge now is to subset the Chip data set by variant effect
and generate a new data set with similar variant proportions to the WGS
data set. We can randomly select variants from each effect group, then
we can perform a new variant effect annotation, and calculate the
proportion of each variant type by their effect. However, since some
variants have more than one effect, we might not get the exact
proportion we want.

We created a SNP list from the chip data set earlier. We can use
those to create the new chip data set. We can use bash tools to randomly
select SNPs, then we merge the lists of SNPs, and use Plink2 to generate
the new data set. We then perform the functional annotation of the new
data set. Perhaps, we can generate a few data sets, randomly selecting
the same number of SNPs, and compare the robustness of our approach.

We can create a new directory inside the SnpEff directory, and move
the files there

```
# I created a new directory and copy the variant lists there. I have a file with both 3 and 5'UTR SNPs, that is why we have more than 61k total
# mkdir output/SnpEff/chip_new_proportions
wc -l output/SnpEff/chip_new_proportions/*SNPs.txt
```

```
##     3697 output/SnpEff/chip_new_proportions/3UTR_SNPs.txt
##     3642 output/SnpEff/chip_new_proportions/5UTR_SNPs.txt
##     1942 output/SnpEff/chip_new_proportions/Nonsynonymous_SNPs.txt
##    14323 output/SnpEff/chip_new_proportions/Synonymous_SNPs.txt
##     7334 output/SnpEff/chip_new_proportions/UTRs_SNPs.txt
##    13758 output/SnpEff/chip_new_proportions/intergenic_SNPs.txt
##    24387 output/SnpEff/chip_new_proportions/intron_SNPs.txt
##    69083 total
```

We can create a bash pipeline to randomly sample SNPs and create a
new data set, following the WGS variant proportions.

```
# the first step of the pipeline is to get the number of SNPs we need to randomly sample from the list of SNPs of each effect group. This information is in the table we created recently. 
head(new_proportions)
```

```
## # A tibble: 6 × 8
##   Variant.effect `WGS.(N)` `WGS.(%)` `Chip.(N)` `Chip.(%)` `Chip.bias.(%)`
##   <chr>              <dbl>     <dbl>      <dbl>      <dbl>           <dbl>
## 1 Intron           1281482     44.8       24387      39.5            -5.32
## 2 Intergenic        993796     34.8       13758      22.3           -12.5 
## 3 Synonymous        384482     13.4       14323      23.2             9.75
## 4 5UTR               67744      2.37       3642       5.9             3.53
## 5 3UTR               64912      2.27       3697       5.99            3.72
## 6 Nonsynonymous      67217      2.35       1942       3.14            0.79
## # ℹ 2 more variables: `Chip.Possible.(N)` <dbl>, `Chip.new.(%)` <dbl>
```

We use all the intergenic SNPs we have since they are the most under
represented in the chip data set.

We have 13758 intergenic SNPs and we will always include them in all
data sets. For all other categories of SNPs, we will always randomly
sample the SNPs in that category. We have more than we need to achieve
the WGS data set proportions.

For example, we will randomly sample 17741 SNPs out of the 39591 for
SNPs on introns to end with a proportion of 44.81% for Chip data
set.

We will have similar proportions to the WGS data set, but since we
have access to more SNPs in coding regions, we can sample them and see
if there are differences in the output of the analysis.

We don’t know if the the allele frequency differences we see in each
data set are the result of different selection pressures, for example,
due to local adaptation of populations.

We might see a swift in the allele frequency of populations if
certain SNPs are sampled. For example, we have 24387 on introns, and we
will randomly sample 17741, more than half of the data set will always
be re-sampled.

In other SNPs sets we have a different scenario. We have 1942 SNPs
with nonsynonymous effects, but we will always sample 930 SNPs, which is
about 50% of the data set.

If variants with nonsynonymous effects have a strong effect on the
allele frequency of a population, they might bias the output of our
analysis.

We might conclude that SNPs sampling following the WGS variants
proportions according to their effect, is influenced by the selection
pressures on each variant. Then, even an even sampling of the variants
according the their effect is not sufficient to replicate the allele
frequencies using whole genome sequencing.

If a set of variants have strong effects on the allele frequency of
populations, then we will be able to have a holistic view of the current
populations’ genetic architecture, if we have high density data across
the genome. It will also tell us that the selection pressure on variants
is strong enough to bias the output of the analysis using the chip data.
Then, precaution would be advised. Perhaps, limiting or avoiding certain
applications or analysis, or valuing the outcome differently.

We wanted to include as many as possible SNPs on exons. If the SNPs
on the exons are under strong selection, they might have low minor
allele frequency. For example, for a variant causing a nonsynonymous
change in a protein coding gene of vital importance, the polymorphic
might exist only in the heterozygous state in a few individuals in the
populations.

An example would be a variant causing a ineffective enzyme. The
transcription machinery might not have adapted yet to the new mutation.
Both chromosomes would be transcribed, but only RNA molecules from one
chromosome would be translated to an normal enzyme.

The lower transcription rate of the RNA of the gene will result in
lower levels of the enzyme. If the lower activity of this enzyme causes
changes in the organism, positively or negatively, the mutation will be
maintained or not in the population.

For example, the lower level of the enzyme, results in changes in
behavior that increases the reproductive fitness of the individual.

If the offspring of this individual carrying the new mutation also
have a higher reproductive fitness, the allele frequency of the
population might change over generations. We observe these changes in
frequency via linkage network analysis. For example, an influx of
migrants in a population might change the allele frequencies in the next
generations.

These changes can be visualized by measuring linkage differences
between two populations, and of course, only if there are differences in
recombination rate during meiosis. A general increase in linkage might
be observed.

Create pipeline

```
# the first step in our pipeline is to create a file with how many SNPs we have to sample from each group.
# we can select it from the object we created earlier
random_variants <-
  new_proportions |>
  select(
    "Variant.effect", "Chip.Possible.(N)"
  ) |>
  filter(
    !row_number() %in% c(7) # to remove the row "Total"
  ) |>
  rename(                   # new names for the columns
    effect = 1,
    number = 2
  ) |>
  mutate(                   # we need to change the cases of the rows to match the files
    effect = tolower(       # we use base R to change everything to lowercase
      effect
    ),
    across(                 # however we need to change utr back capital letters UTR
      "effect", ~str_replace(., "utr", "UTR")
    )
  )
#
# save it to a file to use in the bash pipeline
write_delim(
  random_variants,
  here(
    "output", "SnpEff", "chip_new_proportions", "n_variant_for_sampling_by_effect.txt"
  ),
  delim     = " ",
  quote     = "none",
  col_names = FALSE
)
head(random_variants)
```

```
## # A tibble: 6 × 2
##   effect        number
##   <chr>          <dbl>
## 1 intron         17741
## 2 intergenic     13758
## 3 synonymous      5325
## 4 5UTR             938
## 5 3UTR             899
## 6 nonsynonymous    930
```

Loop to get sets of randomly selected variants

```
# a loop to generate random sets of SNPs
for set in $(seq 1 10); do
  cat output/SnpEff/chip_new_proportions/n_variant_for_sampling_by_effect.txt | while IFS=" " read -r effect number
  do
    cat output/SnpEff/chip_new_proportions/$effect\_SNPs.txt | shuf -n $number
  done > output/SnpEff/chip_new_proportions/sets/set$set.txt
done
```

Count how many SNPs we have in each set

```
wc -l output/SnpEff/chip_new_proportions/sets/set*.txt
```

```
##    39591 output/SnpEff/chip_new_proportions/sets/set1.txt
##    39591 output/SnpEff/chip_new_proportions/sets/set10.txt
##    39591 output/SnpEff/chip_new_proportions/sets/set2.txt
##    39591 output/SnpEff/chip_new_proportions/sets/set3.txt
##    39591 output/SnpEff/chip_new_proportions/sets/set4.txt
##    39591 output/SnpEff/chip_new_proportions/sets/set5.txt
##    39591 output/SnpEff/chip_new_proportions/sets/set6.txt
##    39591 output/SnpEff/chip_new_proportions/sets/set7.txt
##    39591 output/SnpEff/chip_new_proportions/sets/set8.txt
##    39591 output/SnpEff/chip_new_proportions/sets/set9.txt
##   395910 total
```

Compare the SNPs on two data sets (1 and 2)

```
# from man comm
#     -1      Suppress printing of column 1, lines only in file1.
#     -2      Suppress printing of column 2, lines only in file2.
#     -3      Suppress printing of column 3, lines common to both.
#
# comparing set1 and set2
# to get the shared number of SNPs
echo "Comparing set1 and set2:";
echo "shared number SNPs";
comm -12 <(sort output/SnpEff/chip_new_proportions/sets/set1.txt) <(sort output/SnpEff/chip_new_proportions/sets/set2.txt) | wc -l;
comm -12 <(sort output/SnpEff/chip_new_proportions/sets/set1.txt) <(sort output/SnpEff/chip_new_proportions/sets/set2.txt) > output/SnpEff/chip_new_proportions/comm_set1_2.txt
#
# to get number SNPs unique to set1
echo "SNPs unique to set1";
comm -23 <(sort output/SnpEff/chip_new_proportions/sets/set1.txt) <(sort output/SnpEff/chip_new_proportions/sets/set2.txt) | wc -l;
#
# to get number SNPs unique to set2
echo "SNPs unique to set2";
comm -13 <(sort output/SnpEff/chip_new_proportions/sets/set1.txt) <(sort output/SnpEff/chip_new_proportions/sets/set2.txt) | wc -l;
```

```
## Comparing set1 and set2:
## shared number SNPs
##    29969
## SNPs unique to set1
##     9622
## SNPs unique to set2
##     9622
```

Compare the SNPs on two data sets (3 and 4)

```
echo "Comparing set3 and set4:";
echo "shared number SNPs";
comm -12 <(sort output/SnpEff/chip_new_proportions/sets/set3.txt) <(sort output/SnpEff/chip_new_proportions/sets/set4.txt) | wc -l;
comm -12 <(sort output/SnpEff/chip_new_proportions/sets/set3.txt) <(sort output/SnpEff/chip_new_proportions/sets/set4.txt) > output/SnpEff/chip_new_proportions/comm_set3_4.txt
#
# to get number SNPs unique to set1
echo "SNPs unique to set1";
comm -23 <(sort output/SnpEff/chip_new_proportions/sets/set3.txt) <(sort output/SnpEff/chip_new_proportions/sets/set4.txt) | wc -l;
#
# to get number SNPs unique to set2
echo "SNPs unique to set2";
comm -13 <(sort output/SnpEff/chip_new_proportions/sets/set3.txt) <(sort output/SnpEff/chip_new_proportions/sets/set4.txt) | wc -l;
```

```
## Comparing set3 and set4:
## shared number SNPs
##    29966
## SNPs unique to set1
##     9625
## SNPs unique to set2
##     9625
```

Check SNPs that are shared between set1 and 2 and and those from set
3 and 4

```
echo "shared SNPs for each comparison:"
echo " number SNPs shared set1 and set2"
wc -l output/SnpEff/chip_new_proportions/comm_set1_2.txt;
#
echo " number SNPs shared set3 and set4"
wc -l output/SnpEff/chip_new_proportions/comm_set3_4.txt;
#
echo "Comparing shared SNPs from set1_2 and set3_4:";
echo "shared SNPs - we got them twice";
comm -12 <(sort output/SnpEff/chip_new_proportions/comm_set1_2.txt) <(sort output/SnpEff/chip_new_proportions/comm_set3_4.txt) | wc -l;
#comm -12 <(sort output/SnpEff/chip_new_proportions/comm_set1_2.txt) <(sort output/SnpEff/chip_new_proportions/comm_set3_4.txt) > output/SnpEff/chip_new_proportions/comm_set3_4.txt
#
# to get number SNPs unique to set1
echo "SNPs unique to set1_2 - we did not sample then twice";
comm -23 <(sort output/SnpEff/chip_new_proportions/comm_set1_2.txt) <(sort output/SnpEff/chip_new_proportions/comm_set3_4.txt) | wc -l;
#
# to get number SNPs unique to set2
echo "SNPs unique to set3_4 - we did not sample then twice";
comm -13 <(sort output/SnpEff/chip_new_proportions/comm_set1_2.txt) <(sort output/SnpEff/chip_new_proportions/comm_set3_4.txt) | wc -l;
```

```
## shared SNPs for each comparison:
##  number SNPs shared set1 and set2
##    29969 output/SnpEff/chip_new_proportions/comm_set1_2.txt
##  number SNPs shared set3 and set4
##    29966 output/SnpEff/chip_new_proportions/comm_set3_4.txt
## Comparing shared SNPs from set1_2 and set3_4:
## shared SNPs - we got them twice
##    21697
## SNPs unique to set1_2 - we did not sample then twice
##     8272
## SNPs unique to set3_4 - we did not sample then twice
##     8269
```

Out of the 39591 SNPs from our comparison, we obtained 29943 shared
between set1 and set3 and 29889 shared between set3 and set4.

From these two list of shared SNPs, we got 21683 that were sampled 4
times (they were in all 4 sets), while 16466 (8260 + 8206 unique to each
re-sampling) were not re sampled in all 4 sets. We get around 8200 SNPs
unique to each set.

Now we can use the sets of SNPs to create new files for our
analyses.

```
# since we cannot repeat test all SNPs sets, we will choose 3 out of the 10 randomly
# you can run the comman below several time if you want. Each time, 3 random SNPs sets will be choose
ls -1 output/SnpEff/chip_new_proportions/sets/*.txt | shuf -n3
```

```
## output/SnpEff/chip_new_proportions/sets/random3.txt
## output/SnpEff/chip_new_proportions/sets/random5.txt
## output/SnpEff/chip_new_proportions/sets/random10.txt
```

When I first run it, I got set3, 5, and 10. We can also create
another 3 sets with random SNPs from the full data set. Our goal is to
see if the our approach is sensible to biological factors and not only
due to dimensional reduction of the data set.

```
# the bed file7 in our population directory has all the 59384 SNPs passing QC
# we can create the 3 random SNP list from it. We need 39591 SNPs to match the number of SNPs in our previous SNP sets.
# note about each SNP set about LD pruning and MAF: we will have to perform filtering depending on the type of analysis we will perform with the data. For example, for Admixture, we will use LD pruning, which might create SNP sets with slight different proportions from what we wanted. Minor allele frequency filters will also affect our data set's SNP numbers
#
# we can use a loop to create 10 sets, then we randomly choose 3 of them
# when we extract the set of snps with plink we obtain around 33400 SNPs
for set in $(seq 1 10); do
  awk '{print $2}' output/populations/file7.bim | shuf -n 33400 > output/SnpEff/chip_new_proportions/sets/random$set.txt
done
```

Select 3 sets from the 10 random sets

```
# since we cannot repeat test all SNPs sets, we will choose 3 out of the 10 randomly
# you can run the comm below several time if you want. Each time, 3 random SNPs sets will be choose
ls -1 output/SnpEff/chip_new_proportions/sets/random*.txt | shuf -n3
```

```
## output/SnpEff/chip_new_proportions/sets/random5.txt
## output/SnpEff/chip_new_proportions/sets/random1.txt
## output/SnpEff/chip_new_proportions/sets/random6.txt
```

Each time we run the command above a different set of SNPs will be
selected. In my case, it selected random9, 4, and 5.

We can make Venn diagrams of our sets. Let’s do 3 way Venn
diagrams

```
# install the library we will need to create the Venn diagrams
pip install matplotlib-venn
```

We will be using the R library reticulate, which allows integration
of python and R

```
# once you import it, you will see the functions in our R environment
import numpy as np
import scipy as sp
import matplotlib.pyplot as plt
from matplotlib_venn import venn2, venn3, venn3_circles
```

Create Venn diagram from SNP sets

```
import numpy as np
import scipy as sp
import matplotlib.pyplot as plt
from matplotlib_venn import venn2, venn3, venn3_circles
import sys
sys.stdout = open('output.log', 'w')

# compare sets with SNP sampling following the WGS proportions
# we will compare set3, 5, and 10
set3  = open('output/SnpEff/chip_new_proportions/sets/set3.txt').read().split()
set5  = open('output/SnpEff/chip_new_proportions/sets/set5.txt').read().split()
set10 = open('output/SnpEff/chip_new_proportions/sets/set10.txt').read().split()
#
## count total number of SNPs
total = len(list(set(set3+set5+set10)))
#
# plot Venn
fig = plt.figure()
fig, ax = plt.subplots(1, figsize=(11,6))
#
v = venn3(subsets=([set(set3), set(set5), set(set10)]),
set_labels = ('set3', 'set5', 'set10'),
set_colors=("#ff88c0", "#4fd3ff", "#FF8C00"),
subset_label_formatter=lambda x: str(x) + "\n(" + f"{(x/total):.2%}" + ")",
alpha=0.5)
#
# add circles
c = venn3_circles(([set(set3), set(set5), set(set10)]),
linestyle='dashed',
linewidth=1,
color="#cfcac6")
#
# the aesthetics
overlap = v.get_patch_by_id("C")  # get the overlap
overlap.set_edgecolor("white")    # set the edge color
overlap.set_alpha(1)              # set the alpha to 1 otherwise 
overlap.set_linestyle("dashed")   # set a dashed line
overlap.set_linewidth(2)          # set same line width as the circles
overlap.set_zorder(2)             # bump overlap up a level so we can 
#
# Set titles for the figure and the subplot respectively
fig.suptitle('SNP sets following WGS proportions', fontsize=14, fontweight='bold');
ax.set_title('~34k SNPs each set');
fig.tight_layout()
#
plt.savefig('output/SnpEff/chip_new_proportions/sets/sets_following_wgs_prop.pdf', bbox_inches='tight') 
plt.show()
```


From the Venn diagram, we can see that the 3 SNP sets share almost
25k SNPs (~48% of the SNPs), but they have about 4k unique SNPs (~8%),
while sharing ~4.8k SNPs, and the other set (~9%). It will be
interesting to see if the difference among sets will result in
differences in the population or individual based statistics.

Create Venn diagram from random SNP sets

```
# compare sets with SNP sampling following the WGS proportions
# we will compare set3, 5, and 10
random2 = open('output/SnpEff/chip_new_proportions/sets/random2.txt').read().split()
random8 = open('output/SnpEff/chip_new_proportions/sets/random8.txt').read().split()
random9 = open('output/SnpEff/chip_new_proportions/sets/random9.txt').read().split()
#
## count total number of SNPs
total = len(list(set(random2+random8+random9)))
#
# plot Venn
fig = plt.figure()
fig, ax = plt.subplots(1, figsize=(11,6))
#
v = venn3(subsets=([set(random2), set(random8), set(random9)]),
set_labels = ('random2', 'random8', 'random9'),
set_colors=("#ff88c0", "#4fd3ff", "#FF8C00"),
subset_label_formatter=lambda x: str(x) + "\n(" + f"{(x/total):.2%}" + ")",
alpha=0.5)
#
# add circles
c = venn3_circles(([set(random2), set(random8), set(random9)]),
linestyle='dashed',
linewidth=1,
color="#cfcac6")
#
# the aesthetics
overlap = v.get_patch_by_id("C")  # get the overlap
overlap.set_edgecolor("white")    # set the edge color
overlap.set_alpha(1)              # set the alpha to 1 otherwise 
overlap.set_linestyle("dashed")   # set a dashed line
overlap.set_linewidth(2)          # set same line width as the circles
overlap.set_zorder(2)             # bump overlap up a level so we can 
#
# Set titles for the figure and the subplot respectively
fig.suptitle('Random SNP sets not following WGS proportions', fontsize=14, fontweight='bold')
ax.set_title('~34k SNPs each set')
fig.tight_layout()
#
plt.savefig('output/SnpEff/chip_new_proportions/sets/sets_random_not_following_WGS_prop.pdf', bbox_inches='tight') 
plt.show()
```


To exit python, type quit or exit in the console.

We can see that the shared number of SNPs among the 3 randoms sets
are different than when we follow the WGS proportions. Now we have about
30% shared SNPs instead of 48%. We see a similar number of unique SNPs
for each set (8%), and each set shared more with other sets, increasing
from 9% to 15%.

## 9. Create new files following WGS SNP effect proportions

We will use the sets following the WGS SNP effect proportions to
create new data sets to run with fastStructure.

Choose 2 sets randomly

```
# since we cannot repeat test all SNPs sets, we will choose 2 out of the 10 randomly
# you can run the comman below several time if you want. Each time, 2 random SNPs sets will be choose
ls -1 output/SnpEff/chip_new_proportions/sets/set*.txt | shuf -n2
```

```
## output/SnpEff/chip_new_proportions/sets/set8.txt
## output/SnpEff/chip_new_proportions/sets/set6.txt
```

I will use set4.txt and set10.txt.

We can extract these SNPs from the file7 using Plink

set4

```
plink2 \
--allow-extra-chr \
--bfile output/populations/file7 \
--extract output/SnpEff/chip_new_proportions/sets/set4.txt \
--make-bed \
--out output/populations/set4 \
--silent;
grep 'variants\|samples' output/populations/set4.log
```

```
## 237 samples (30 females, 67 males, 140 ambiguous; 237 founders) loaded from
## 82731 variants loaded from output/populations/file7.bim.
## --extract: 33447 variants remaining.
## 33447 variants remaining after main filters.
```

set10

```
plink2 \
--allow-extra-chr \
--bfile output/populations/file7 \
--extract output/SnpEff/chip_new_proportions/sets/set10.txt \
--make-bed \
--out output/populations/set10 \
--silent;
grep 'variants\|samples' output/populations/set10.log
```

```
## 237 samples (30 females, 67 males, 140 ambiguous; 237 founders) loaded from
## 82731 variants loaded from output/populations/file7.bim.
## --extract: 33380 variants remaining.
## 33380 variants remaining after main filters.
```

We can select two random sets of SNPs to compare with these two
sets

```
# since we cannot repeat test all SNPs sets, we will choose 3 out of the 10 randomly
# you can run the comm below several time if you want. Each time, 3 random SNPs sets will be choose
ls -1 output/SnpEff/chip_new_proportions/sets/random*.txt | shuf -n2
```

```
## output/SnpEff/chip_new_proportions/sets/random5.txt
## output/SnpEff/chip_new_proportions/sets/random4.txt
```

random8

```
plink2 \
--allow-extra-chr \
--bfile output/populations/file7 \
--extract output/SnpEff/chip_new_proportions/sets/random8.txt \
--make-bed \
--out output/populations/random8 \
--silent;
grep 'variants\|samples' output/populations/random8.log
```

```
## 237 samples (30 females, 67 males, 140 ambiguous; 237 founders) loaded from
## 82731 variants loaded from output/populations/file7.bim.
## --extract: 39591 variants remaining.
## 39591 variants remaining after main filters.
```

random4

```
plink2 \
--allow-extra-chr \
--bfile output/populations/file7 \
--extract output/SnpEff/chip_new_proportions/sets/random4.txt \
--make-bed \
--out output/populations/random4 \
--silent;
grep 'variants\|samples' output/populations/random4.log
```

```
## 237 samples (30 females, 67 males, 140 ambiguous; 237 founders) loaded from
## 82731 variants loaded from output/populations/file7.bim.
## --extract: 39591 variants remaining.
## 39591 variants remaining after main filters.
```

We did run ancestry analysis with these different sets, but there was
no significant differences.
