# Supplementary material for: A genotyping array for the globally invasive vector mosquito, Aedes albopictus
Source: Parasit Vectors. 2024 Mar 4;17:106. doi: 10.1186/s13071-024-06158-z (PMC10910840; doi:10.1186/s13071-024-06158-z)
Supplement: Supplementary file 7 — Additional file 7. Quality control for wild samples genotyped with the chip. [file 13071_2024_6158_MOESM7_ESM.html]

Aedes albopictus SNP chip - Chip use: genotyping wild populations - quality control.


# Aedes albopictus SNP chip - Chip use: genotyping wild populations - quality control.

#### Luciano V Cosme

#### 2023-10-27

- 1. Getting
  started: R libraries and software for QC
  - 1.1 R libraries and software
  - 1.2 Check directory
    structure for the project
  - 1.3 Download software for
    quality control
- 2. The data
  - 2.1 Use Plink2 to convert
    to bed format
  - 2.2 Use R to update the .fam
    file
  - 2.3 Remove
    duplicates
  - 2.4 Checking the
    number of samples and localities
- 3.
  Quality control steps
  - 3.1 Missingness
  - 3.2 Minor allele frequency
  - 3.3 HWE test
  - 3.4 LD pruning
  - 3.4
    Heterozygosity
  - 3.6 Relatedness
  - 3.7 Quick PCA
    with Plink using the LD pruned data
- 4. Create chromosomal scale
  - 4.1 Estimate LD
    blocks using chromosomal scale
- 5. SNP
  annotation lifting to different genome assemblies
  - 5.1 AalbF2
- 6. Plot SNP
  density
- 7. LD pruning using the
  chromosomal scale
- 8. Intergenic unlinked SNPs
- 9. Pruning r2
  0.01
- 10. Pruning r2
  0.1

# 1. Getting started: R libraries and software for QC

## 1.1 R libraries and software

```
library(tidyverse)
library(here)
library(dplyr)
library(ggplot2)
library(colorout)
library(extrafont)
library(reticulate)
library(scales)
library(stringr)
library(grid)
library(flextable)
library(devtools)
library(readr)
library(purrr)
library(ggtext)
library(ggvenn)
library(data.table)
library(RColorBrewer)
library(ggrepel)
```

## 1.2 Check directory structure for the project

**Note:** make sure you download the raw data and place
it in a new directory. You can check the directory structure using a
tool called Tree.

```
# check all the Tree options with the command in your terminal: man tree
tree -L 2 -d --charset=ascii . # -L set the directory depth to show; the flag -d tells tree to return directories only. and the period at the end tells tree to use the current directory; --charset=ascii is to show nicely here.
# if you want to see the files you can use the code below.
# tree --dirsfirst .
```

```
|-- data
|   |-- genome
|   |-- raw_data
|-- output
|   |-- SnpEff
|   |-- crosses
|   |-- files
|   `-- populations
`-- scripts
    |-- RMarkdown_files
    `-- analysis"
```

## 1.3 Download software for quality control

I used PLINK v2.00a3.3 64-bit (3 Jun 2022) for all the analysis.
However, the Affymetrix software does not export the files directly into
a format we can import into Plink2. Therefore, we need first to use
Plink 1.9 to convert the `.ped` and `.map` file
format to `.bed`, `.bin`, and `.fam`.
You can install it with `conda`. You can download Miniconda or Anaconda.
`conda install -c bioconda plink2`. If you have problems, you
can also get it from GitHub, and compile it for your OS (both
versions, 1.9 and 2). Please check Marees et. al, 2018 for more
information about quality control using Plink 1.9. I used some of their
code for quality control, and their general guidelines.

Check your Plink2 version.

```
plink2 --version
```

```
## PLINK v2.00a3.3 64-bit (3 Jun 2022)
```

**First check if the data is in the correct
location:**

```
ls data/raw_data/albo/populations/* # we use * to truncate the name of the file showing all files
```

```
## data/raw_data/albo/populations/chip_500_dp_01.bed
## data/raw_data/albo/populations/chip_500_dp_01.bim
## data/raw_data/albo/populations/chip_500_dp_01.fam
## data/raw_data/albo/populations/chip_500_dp_01.log
## data/raw_data/albo/populations/manuscript_august10_2023.vcf
```

Note about the bash multiline comments.  
 Since we will use the
Plink command with one option per line separated by backslash
`/` , and not one single line command, we add comments using
`` # ` `` to open and `` ` `` to close.  
 For example,
we can split the `plink2 –version` into a multiline command
with a comment.

```
plink2 `# this still works` \
--version
```

```
## PLINK v2.00a3.3 64-bit (3 Jun 2022)
```

# 2. The data

I prepared the data with the families and individual ids. I also set
the reference alleles to match the AlbF3 genome assembly

We can check how many sample names we have in our vcf

```
# make sure you have all the .CEL samples in your family file
bcftools query -l data/raw_data/albo/populations/manuscript_august10_2023.vcf | wc -l
```

```
##      243
```

## 2.1 Use Plink2 to convert to bed format

```
plink2 \
--allow-extra-chr \
--vcf data/raw_data/albo/populations/manuscript_august10_2023.vcf \
--const-fid \
--make-bed \
--exclude output/files/albopictus_SNPs_fail_segregation.txt \
--fa data/genome/albo.fasta.gz \
--ref-from-fa 'force' `# sets REF alleles when it can be done unambiguously, we use force to change the alleles` \
--out output/populations/file1 \
--silent;
# --keep-allele-order \ if you use Plink 1.9
grep "variants" output/populations/file1.log; # to get the number of variants from the log file.
```

```
## --vcf: 115346 variants scanned.
## 115346 variants loaded from output/populations/file1-temporary.pvar.zst.
## --exclude: 113864 variants remaining.
## 113864 variants remaining after main filters.
## --ref-from-fa force: 0 variants changed, 113864 validated.
```

Check the fam file

```
# Check head of the data
head output/populations/file1.fam
```

```
## OKI  1001    0   0   2   -9
## OKI  1002    0   0   2   -9
## OKI  1003    0   0   2   -9
## OKI  1004    0   0   2   -9
## OKI  1005    0   0   2   -9
## OKI  1006    0   0   1   -9
## OKI  1007    0   0   1   -9
## OKI  1008    0   0   1   -9
## OKI  1009    0   0   1   -9
## OKI  1010    0   0   1   -9
```

```
head -n 5 data/raw_data/albo/wgs_vs_chip/sample_ped_info.txt
```

```
## Sample Filename  Family_ID   Individual_ID   Father_ID   Mother_ID   Sex Affection Status
## 608_Debug027_B5.CEL  KAT 12a 0   0   2   -9
## 616_Debug028_H10.CEL SAI 16a 0   0   2   -9
## 615_Debug028_G9.CEL  SAI 3a  0   0   2   -9
## 607_Debug027_B4.CEL  KAT 11a 0   0   2   -9
```

## 2.2 Use R to update the .fam file

Import the fam file we use with Axiom Suite

```
# the order of the rows in this file does not matter
samples <-
  read.delim(
    file   = here(
      "data",
      "raw_data",
      "albo",
      "wgs_vs_chip",
      "sample_ped_info_ALLPOPS_for_comparisons.txt"
    ),
    header = TRUE
  )
head(samples)
```

```
##     Sample.Filename Family_ID Individual_ID Father_ID Mother_ID Sex
## 1  8_MAN_Brazil.CEL       MAU             8         0         0   0
## 2  9_MAN_Brazil.CEL       MAU             9         0         0   0
## 3 16_MAN_Brazil.CEL       MAU            16         0         0   0
## 4 17_MAN_Brazil.CEL       MAU            17         0         0   0
## 5 18_MAN_Brazil.CEL       MAU            18         0         0   0
## 6 60_MAN_Brazil.CEL       MAU            60         0         0   0
##   Affection.Status
## 1               -9
## 2               -9
## 3               -9
## 4               -9
## 5               -9
## 6               -9
```

Import .fam file we created once we created the bed file using
Plink2

```
# The fam file is the same for both data sets with the default or new priors
fam1 <-
  read.delim(
    file   = here(
      "output", "populations", "file1.fam"
    ),
    header = FALSE,
    
  )
head(fam1)
```

```
##   V1           V2 V3 V4 V5 V6
## 1  0 1001_OKI.CEL  0  0  0 -9
## 2  0 1002_OKI.CEL  0  0  0 -9
## 3  0 1003_OKI.CEL  0  0  0 -9
## 4  0 1004_OKI.CEL  0  0  0 -9
## 5  0 1005_OKI.CEL  0  0  0 -9
## 6  0 1006_OKI.CEL  0  0  0 -9
```

We can merge the tibbles.

```
# Extract the number part from the columns
fam1_temp <- fam1 |>
  mutate(num_id = as.numeric(str_extract(V2, "^\\d+")))

samples_temp <- samples |>
  mutate(num_id = as.numeric(str_extract(Sample.Filename, "^\\d+")))

# Perform the left join using the num_id columns and keep the order of fam1
df <- fam1_temp |>
  dplyr::left_join(samples_temp, by = "num_id") |>
  dplyr::select(-num_id) |>
  dplyr::select(8:13)

# check the data frame
head(df)
```

```
##   Family_ID Individual_ID Father_ID Mother_ID Sex Affection.Status
## 1       OKI          1001         0         0   2               -9
## 2       OKI          1002         0         0   2               -9
## 3       OKI          1003         0         0   2               -9
## 4       OKI          1004         0         0   2               -9
## 5       OKI          1005         0         0   2               -9
## 6       OKI          1006         0         0   1               -9
```

We can check how many samples we have in our file

```
nrow(df)
```

```
## [1] 243
```

Before you save the new fam file, you can change the original file to
a different name, to compare the order later. If you want to repeat the
steps above after you saving the new file1.fam, you will need to import
the vcf again.

```
# Save and override the .fam file for dp
write.table(
  df,
  file      = here(
    "output", "populations", "file1.fam"
  ),
  sep       = "\t",
  row.names = FALSE,
  col.names = FALSE,
  quote     = FALSE
)
```

Check the new .fam file to see if has the order and the sample
attributes we want.

```
# you can open the file on a text editor and double check the sample order and information.
head -n 5 output/populations/file1.fam
```

```
## OKI  1001    0   0   2   -9
## OKI  1002    0   0   2   -9
## OKI  1003    0   0   2   -9
## OKI  1004    0   0   2   -9
## OKI  1005    0   0   2   -9
```

## 2.3 Remove duplicates

We have 4 samples genotyped 3 times each. We can keep only one of
them

We can check the triplicates

```
# Check triplicates
grep "a\|b\|c" output/populations/file1.fam
```

```
## KAG  a   0   0   0   -9
## KAG  b   0   0   0   -9
## KAG  c   0   0   0   -9
## BEN  a   0   0   0   -9
## BEN  b   0   0   0   -9
## BEN  c   0   0   0   -9
```

We can keep the first sample and remove b and c

To subset the data we need to create a list of samples with family id
and individual ids

```
grep "b\|c" output/populations/file1.fam | awk '{print $1, $2}' > output/populations/duplicates_to_remove.txt;
head output/populations/duplicates_to_remove.txt
```

```
## KAG b
## KAG c
## BEN b
## BEN c
```

Create a new bed removing the duplicated samples. We can also make
sure the reference alleles match the reference genome

```
# I created a fam file with the information about each sample, but first we import the data and create a bed file setting the family id constant
plink2 \
--allow-extra-chr \
--bfile output/populations/file1 \
--make-bed \
--fa data/genome/albo.fasta.gz \
--ref-from-fa 'force' `# sets REF alleles when it can be done unambiguously, we use force to change the alleles` \
--exclude output/files/albopictus_SNPs_fail_segregation.txt \
--remove output/populations/duplicates_to_remove.txt \
--out output/populations/file1b \
--silent;
# --keep-allele-order \ if you use Plink 1.9
grep "samples\|variants" output/populations/file1b.log # to get the number of variants from the log file.
```

```
## 243 samples (30 females, 69 males, 144 ambiguous; 243 founders) loaded from
## 113864 variants loaded from output/populations/file1.bim.
## --exclude: 113864 variants remaining.
## --remove: 239 samples remaining.
## 239 samples (30 females, 69 males, 140 ambiguous; 239 founders) remaining after
## 113864 variants remaining after main filters.
## --ref-from-fa force: 0 variants changed, 113864 validated.
```

Using the default prior we obtained 107,294 SNPs. All the reference
alleles matched the reference genome (AalbF3). We removed the duplicates
and kept only 1 sample

Check the headings of the the files we will work on.

```
head -n 5 output/populations/file1b.fam
```

```
## OKI  1001    0   0   2   -9
## OKI  1002    0   0   2   -9
## OKI  1003    0   0   2   -9
## OKI  1004    0   0   2   -9
## OKI  1005    0   0   2   -9
```

Check how many samples are in the .fam file

```
wc -l output/populations/file1b.fam
```

```
##      239 output/populations/file1b.fam
```

## 2.4 Checking the number of samples and localities

```
# this part is very important. Make sure there are no NAs or something that is not what you would expect. For example, the number of mosquitoes per population.
awk '{print $1}' output/populations/file1b.fam | sort | uniq -c | awk '{print $2, $1}' # here we use awk to print the first column of the file1.fam. Then we sort it using sort, and count the unique occurrences with uniq. The second awk command is for aesthetic reasons, I prefer showing the family name on the left and the counts on the right. You can always check the manual to see explanations for all the tools. For example, type man awk, man sort, or man uniq to see all the operations or options available. The pipe operator (|) passes the output (stdout) of one command as input (stdin) to another. Since we create a command using the pipe operator, we can call it a pipeline.
```

```
## BEN 12
## CAM 12
## CHA 12
## GEL 2
## HAI 12
## HAN 4
## HOC 7
## HUN 12
## INJ 11
## INW 4
## JAF 2
## KAC 6
## KAG 12
## KAN 12
## KAT 10
## KLP 4
## KUN 4
## LAM 9
## MAT 12
## OKI 12
## QNC 12
## SON 3
## SSK 12
## SUF 6
## SUU 6
## TAI 8
## UTS 12
## YUN 9
```

In Linux/Unix we have 3 `I/O streams`: Standard input
(`stdin`) - this is the file handle that your process reads
to get information from you.  
 Standard output (`stdout`) -
your process writes conventional output to this file handle.  
Standard error (`sterr`) - your process writes diagnostic
output to this file handle.  
 Most programs need to read input, write
output, and log errors, so `stdin`, `stdout`, and
`stderr` are predefined as a programming convenience.  
 An
easy way to access any file is by using the unique file descriptor
number associated with it. In the case of these streams, there are
unique values assigned to each one of them:  
 0:
`stdin`  
 1: `stdout`  
 2:
`stderr`

# 3. Quality control steps

## 3.1 Missingness

```
plink2 \
--allow-extra-chr \
--bfile output/populations/file1b \
--geno 0.1                `# we set genotype missiningness to 10% with this option` \
--make-bed \
--out output/populations/file2 \
--silent \
--missing;                 # --missing produces sample-based and variant-based missing data reports. If run with --within/--family, the variant-based report is stratified by cluster.
grep "variants" output/populations/file2.log
```

```
## 113864 variants loaded from output/populations/file1b.bim.
## --geno: 9725 variants removed due to missing genotype data.
## 104139 variants remaining after main filters.
```

Make plot

```
#   ____________________________________________________________________________
#   import individual missingness                                           ####
indmiss <-                                              # name of the data frame we are creating
  read.delim(                                           # use function read table
    file   = here(
      "output", "populations", "file2.smiss"
    ),                                                  # we use library here to import file2.imiss from data/QC
    header = TRUE                                       # we do have headers in our file
  )
#   ____________________________________________________________________________
#   import SNP missingness                                                  ####
snpmiss <-
  read.delim(
    file   = here(
      "output", "populations", "file2.vmiss"
    ),
    header = TRUE
  )
#
```

Plot individual missingness

```
# load plotting theme
source(
  here(
    "scripts", "analysis", "my_theme2.R"
  )
)

ggplot( # Start a ggplot object with the data and aesthetic mappings
  indmiss,
  aes(
    x = F_MISS
  )
) +
  geom_histogram( # Add a histogram layer
    color            = "black",
    fill             = "#B6FAD7",
    bins             = 6
  ) +
  geom_text(
    # Add text labels for bin counts
    stat             = "bin",
    aes(
    label = after_stat(count)
  ),
    vjust            = -0.5,
    color            = "purple",
    size             = 3,
    bins             = 6
  ) +
  geom_vline(
    # Add a vertical line at the mean of F_MISS
    aes(
    xintercept = mean(F_MISS)),
    color            = "orange",
    linetype         = "dotted",
    linewidth        = .5
  ) +
  geom_text(
    # Add a text label for the mean of F_MISS
    aes(
      x = mean(F_MISS),
      y = 75,
      label = paste0(
        "Mean \n",
        scales::percent(mean(F_MISS),
          accuracy = 0.01
        )
      )
    ),
    size = 3,
    color = "orange",
    hjust = -.1
  ) +
  labs( # Add axis labels
    x                = "Individual Missingness (%)",
    y                = "Frequency (n)"
  ) +
  my_theme() +
  scale_x_continuous( # Scale the x-axis to display percentages
    labels           = scales::percent,
    n.breaks         = 6
  )
```

```
#
# save the plot
ggsave(
  here(
    "output", "populations", "figures" , "individual_missingness.pdf"
  ),
  width              = 7,
  height             = 5,
  units              = "in"
)
```

The function my\_theme() that we imported above

```
# Define a function to customize the theme
my_theme <- function() {
  theme_minimal(base_size = 12, base_family = "") +
    theme(
      panel.grid.major = element_line(
        linetype = "dashed",
        linewidth = 0.2,
        color = "pink"
      ),
      panel.grid.minor = element_line(
        linetype = "dashed",
        linewidth = 0.2,
        color = "pink"
      ),
      # Customize the x-axis label
      axis.title.x = element_text(
        angle          = 0,
        hjust          = 1,
        face           = "bold"
      ),
      # Customize the y-axis label
      axis.title.y = element_text(
        angle          = 90,
        hjust          = 1,
        face           = "bold"
      )
    )
}
# we can save the function to source it later
dump(                                                    # check ?dump for more information
  "my_theme",                                            # the object we want to save
  here(
    "scripts", "analysis", "my_theme2.R")                # use here to save it our function as .R 
)
```

Plot variant missingness

```
# This plot takes a while to compute
# This code creates a histogram from the indmiss data set using the F_MISS column.
# ggplot builds a histogram of individual missingness data
ggplot(
  snpmiss,
  aes(
    x = F_MISS
  )
) +
  geom_histogram(
    color = "black",
    fill = "#B6FAD7",
    bins = 6
  ) +
  stat_bin(
    geom = "text",
    aes(
      label = format(
        after_stat(count),
        big.mark = ",",
        scientific = FALSE
      )
    ),
    vjust = -0.5,
    color = "purple",
    size = 2,
    bins = 6
  ) +
  geom_vline(
    aes(
      xintercept = mean(F_MISS)
    ),
    color = "orange",
    linetype = "dotted",
    linewidth = 0.5
  ) +
  geom_text(
    aes(
      x = mean(F_MISS),
      y = 16000,
      label = paste0(
        "Mean \n",
        scales::percent(mean(F_MISS),
          accuracy = 0.01
        )
      )
    ),
    size = 3,
    color = "orange",
    # hjust = 1.5,
    vjust = -.2
  ) +
  labs(
    x = "Variant Missingness (%)",
    y = "Frequency (n)"
  ) +
  # theme_minimal(
  #   base_size = 12,
  #   base_family = "Roboto Condensed"
  # ) +
  scale_x_continuous(
    labels = scales::percent,
    n.breaks = 6
  ) +
  scale_y_continuous(
    labels = scales::label_comma(),
    n.breaks = 5
  ) +
  my_theme()
```

```
# save the plot
ggsave(
  here(
    "output", "populations", "figures", "SNPs_missingness.pdf"
  ),
  width  = 7,
  height = 5,
  units  = "in"
)
```

Remove individuals missing more than 20% of SNPs. You can use the
threshold you want, change the flag –mind

```
plink2 \
--allow-extra-chr \
--bfile output/populations/file2 \
--mind 0.2               `# here we set the individual missingness threshold of 20%`\
--make-bed \
--out output/populations/file3 \
--silent;
grep "samples\|variants" output/populations/file3.log
```

```
## 239 samples (30 females, 69 males, 140 ambiguous; 239 founders) loaded from
## 104139 variants loaded from output/populations/file2.bim.
## 0 samples removed due to missing genotype data (--mind).
## 239 samples (30 females, 69 males, 140 ambiguous; 239 founders) remaining after
```

We did not remove any SNP due to individual missingness

## 3.2 Minor allele frequency

First lets make a plot of the MAF. First, we estimate the allele
frequency with Plink.

```
plink2 \
--allow-extra-chr \
--bfile output/populations/file3 \
--freq \
--out output/populations/MAF_check \
--silent
```

Then we plot it with ggplot.

```
#   ____________________________________________________________________________
#   Import MAF data                                                         ####
maf_freq <-
  read.delim(
    here(
      "output", "populations", "MAF_check.afreq"
    ),
    header = TRUE
  )
```

Make MAF plot

```
#   ____________________________________________________________________________
#   make the plot                                                           ####
ggplot(
  maf_freq,
  aes(ALT_FREQS)
) +
  geom_histogram(
    colour = "black",
    fill = "#C4F3F5",
    bins = 40
  ) +
  labs(
    x = "Minor Allele Frequency (MAF)",
    y = "Frequency (n)",
    caption = "<span style='color:red;'><i>Red</i></span> <span style='color:black;'><i>line at</i></span><span style='color:red;'><i> MAF 10%</i></span><span style='color:black;'><i> threshold</i></span>."
  ) +
  geom_text(
    aes(
      x = .1,
      y = 8000,
      label = paste0("11,314 SNPs")
    ),
    size = 3,
    color = "blue",
    vjust = -.2
  ) +
  geom_vline(xintercept = 0.1, color = "red") +
  my_theme() +
  theme(plot.caption = element_markdown()) +
  scale_y_continuous(label = scales::number_format(big.mark = ",")) +
  scale_x_continuous(breaks = c(0, 0.1, 0.2, 0.4, 0.6, 0.8, 1))
```

```
#   ____________________________________________________________________________
#   save the plot                                                           ####
ggsave(
  here(
    "output", "populations", "figures", "MAF.pdf"
  ),
  width  = 5,
  height = 4,
  units  = "in"
)
```

Now we apply the MAF filter.

```
# We will use MAF of 10%
plink2 \
--allow-extra-chr \
--bfile output/populations/file3 \
--maf 0.1 \
--make-bed \
--out output/populations/file4 \
--silent;
grep "variants" output/populations/file4.log
```

We removed 11,314 variants due to the MAF filter. Next we will
excludes markers which deviate from Hardy–Weinberg equilibrium (HWE). It
is a common indicator of genotyping error, but may also indicate
evolutionary selection. We have to do it for each population
individually. We cannot do it for all populations at once. Therefore,
the first step is to create a new bed file with Plink keeping only one
population. I like to create a new directory and name it “hardy”, and
copy the “file4” there.

```
mkdir -p output/populations/hardy;
cp output/populations/file4.* output/populations/hardy/
```

## 3.3 HWE test

Now we can run the HWE test. However, we will need to apply the SNP
missingness again for each population. If we do not, the HWE will vary
widely. With the bash script below, we will create a new file for each
population, run the HWE test with HWE p value <1e‐6 (HWE p value
<1e‐6). Then, we ask Plink to generate a list of SNPs that passed the
test for each population.

```
for fam in $(awk '{print $1}' output/populations/hardy/file4.fam | sort | uniq); 
do 
echo $fam | \
plink2 \
--allow-extra-chr \
--silent \
--keep-allele-order \
--bfile output/populations/hardy/file4 \
--keep-fam /dev/stdin \
--make-bed \
--out output/populations/hardy/$fam \
--hwe 0.000001 \
--geno 0.1 \
--write-snplist; \
done
```

Next, we use “cat” and “`awk`” to concatenate the SNP list
from all populations, and remove duplicates. Once we have a list of SNPs
that passed the test for each population, we can use Plink to create a
new bed file keeping only the SNPs that passed the test in each
population. First, lets get the list of SNPs, and count how many
passed:

```
cat output/populations/hardy/*.snplist | awk '!a[$0]++' > output/populations/passed_hwe.txt;
wc -l output/populations/passed_hwe.txt
```

How many variants we had before

```
cat output/populations/file4.bim | awk '{print $2}'| awk '!a[$0]++' | wc -l
```

```
##    82731
```

Variants not passing HWE test

```
82731 - 82731
```

```
## [1] 0
```

All variants passed HWE test. If some failed, next time we could
remove the variants that did not pass HWE test, using the –extract flag,
extracting only those that passed HWE.

## 3.4 LD pruning

Since we do not have to remove any SNPs due to deviation from HWE, we
can proceed with heterozygosity estimates. The first step is to “prune”
our data set. We will check the pairwise linkage estimates for all SNPs.
We can work with file4. We will use “`indep-pairwise`” to
check if there are SNPs above a certain linkage disequilibrium (LD)
threshold. Check Plink documentation for more details https://www.cog-genomics.org/plink/1.9/ld I used
“`--indep-pairwise 5 1 0.1`” , which indicates according to
the documentation:
`--indep-pairphase <window size>['kb'] <step size (variant ct)> <r^2 threshold>`
We will check in a window of 5kb if there is any pair of SNPs with r2
estimates above 0.1, then we will move our window 1 SNP and check again
for SNPs above the threshold. We will repeat this procedure until we
check the entire genome.

```
plink2 \
--allow-extra-chr \
--bfile output/populations/file4 \
--extract output/populations/passed_hwe.txt \
--indep-pairwise 5 1 0.1 \
--out output/populations/indepSNP \
--silent;
grep 'pairwise\|variants\|samples' output/populations/indepSNP.log
```

```
##   --indep-pairwise 5 1 0.1
## 239 samples (30 females, 69 males, 140 ambiguous; 239 founders) loaded from
## 82731 variants loaded from output/populations/file4.bim.
## --extract: 82731 variants remaining.
## 82731 variants remaining after main filters.
## --indep-pairwise (11 compute threads): 22057/82731 variants removed.
```

Remember, the SNPs are not removed from our data set. Plink created 3
files when we ran the code above. One is the “indepSNP.log” file, and
the other two are:  
“**indepSNP.prune.in**” -> list of
SNPs with squared correlation smaller than our r2 threshold of
0.1.  
“i**ndepSNP.prune.out**” -> list of SNPs with
squared correlation greater than our r2 threshold of 0.1. For our
heterozygosity estimates, we want to use the set of SNPs that are below
our r2 threshold of 0.1. We consider that they are randomly associated.
We can use Plink to estimate the heterozygosity using the
“indepSNP.prune.in” file.

## 3.4 Heterozygosity

```
plink2 \
--allow-extra-chr \
--bfile output/populations/file4 \
--extract output/populations/indepSNP.prune.in \
--het \
--out output/populations/R_check \
--silent;
grep 'variants' output/populations/R_check.log
```

```
## 82731 variants loaded from output/populations/file4.bim.
## --extract: 60674 variants remaining.
## 60674 variants remaining after main filters.
```

We can see that we started with 92,693 SNPs, then we only extract
those that are not “linked” from the “indepSNP.prune.in” file. We used
these SNPs to estimate heterozygosity. Now we can use R to parse the
R\_check.het, to find the individuals with excess heterozygosity. We will
remove any individual that deviates more than 4 standard deviations from
the mean heterozygosity of the data set. The code below will create a
list of individuals with excess heterozygosity (file named
“`fail-het-qc.txt`”), and make a heterozygosity plot for the
entire data set.

```
#   ____________________________________________________________________________
#   find individuals with high heterozygosity                              ####
# import the data from Plink
het <- read.delim(
  here(
    "output", "populations", "R_check.het"
  ),
  head = TRUE
)
#
# check head of the file
colnames(het)
```

```
## [1] "X.FID"  "IID"    "O.HOM." "E.HOM." "OBS_CT" "F"
```

Estimate het

```
# create a column named HET_RATE and calculate the heterozygosity rate
het$HET_RATE <- (het$"OBS_CT" - het$"O.HOM") / het$"OBS_CT"
#
# use subset function to get values deviating from 4sd of the mean heterozygosity rate.
het_fail <-
  subset(
    het, (het$HET_RATE < mean(
      het$HET_RATE
    ) - 4 * sd(
      het$HET_RATE
    )) |
      (het$HET_RATE > mean(
        het$HET_RATE
      ) + 4 * sd(
        het$HET_RATE
      ))
  )
#
# get the list of individuals that failed our threshold of 4sd from the mean.
het_fail$HET_DST <-
  (het_fail$HET_RATE - mean(
    het$HET_RATE
  )) / sd(
    het$HET_RATE
  )
```

Save the files to use with Plink

```
#   ____________________________________________________________________________
#   save the data to use with Plink2                                        ####
#
write.table(
  het_fail,
  here(
    "output", "populations", "fail-het-qc.txt"
  ),
  row.names = FALSE
)
```

Make plot

```
#   ____________________________________________________________________________
#   make a heterozygosity plot                                              ####
#
ggplot(
  het,
  aes(
    HET_RATE
  )
) +
  geom_histogram(
    colour           = "black",
    fill             = "#CDFAF8",
    bins             = 40
  ) +
  labs(
    x                = "Heterozygosity Rate",
    y                = "Number of Individuals"
  ) +
  geom_vline(
    aes(
      xintercept     = mean(
        HET_RATE
      )
    ),
    col              = "#F2C46F",
    linewidth        = 1.5
  ) +
  geom_vline(
    aes(
      xintercept     = mean(
        HET_RATE
      ) + 4 * sd(
        HET_RATE
      )
    ),
    col              = "#BFB9B9",
    linewidth        = 1
  ) +
  geom_vline(
    aes(
      xintercept     = mean(
        HET_RATE
      ) - 4 * sd(
        HET_RATE
      )
    ),
    col              = "#BFB9B9",
    linewidth        = 1
  ) + 
  my_theme() +
  scale_y_continuous(
    labels           = comma
  )
```

```
#   ____________________________________________________________________________
#   save the heterozygosity plot                                            ####
ggsave(
  here(
    "output", "populations", "figures", "Heterozygosity.pdf"
  ),
  width  = 5,
  height = 4,
  units  = "in"
)
```

The orange line in the plot above indicates the mean, and the gray
lines indicate 4 standard deviation from the mean. We can see that some
mosquitoes do have excess heterozygous sites. We will remove them. We
can get their ID from the file “`fail-het-qc.txt`”. We can
use the bash script below to parse the file to use with Plink

```
sed 's/"// g' output/populations/fail-het-qc.txt | awk '{print$1, $2}'> output/populations/het_fail_ind.txt;
echo 'How many mosquitoes we need to remove from our data set:';
cat output/populations/het_fail_ind.txt | tail -n +2 | wc -l;
echo 'Which mosquitoes we have to remove:';
tail -n +2 output/populations/het_fail_ind.txt
```

```
## How many mosquitoes we need to remove from our data set:
##        2
## Which mosquitoes we have to remove:
## KAT 9
## KAT 12
```

The population from Nepal has high heterozygosity rate. We will
remove 2 individuals.

Next, we will remove these mosquitoes from our data set using
Plink:

```
plink2 \
--allow-extra-chr \
--bfile output/populations/file4 \
--remove output/populations/het_fail_ind.txt \
--make-bed \
--out output/populations/file5 \
--silent;
grep 'variants\|samples' output/populations/file5.log
```

## 3.6 Relatedness

Check for cryptic relatedness. Check Plink2 documentation https://www.cog-genomics.org/plink/2.0/distance You can
download King directly https://www.kingrelatedness.com/manual.shtml or check
their manuscript https://www.ncbi.nlm.populations.gov/pmc/articles/PMC3025716/pdf/btq559.pdf
From Plink2 documentation: “Note that KING kinship coefficients are
scaled such that duplicate samples have kinship 0.5, not 1. First-degree
relations (parent-child, full siblings) correspond to ~0.25,
second-degree relations correspond to ~0.125, etc. It is conventional to
use a cutoff of ~0.354 (the geometric mean of 0.5 and 0.25) to screen
for monozygotic twins and duplicate samples, ~0.177 to add first-degree
relations, etc.” There two options. One is to run only –make-king and
another one is to use –make-king-table We will use the threshold of
0.354 and create a table.

```
# Plink2 will create a file with extension .king
plink2 \
--allow-extra-chr \
--bfile output/populations/file4 \
--extract output/populations/indepSNP.prune.in \
--make-king-table rel-check \
--king-table-filter 0.354 \
--out output/populations/file5 \
--silent;
grep 'variants\|samples' output/populations/file5.log
```

```
## 239 samples (30 females, 69 males, 140 ambiguous; 239 founders) loaded from
## 82731 variants loaded from output/populations/file4.bim.
## --extract: 60674 variants remaining.
## 60674 variants remaining after main filters.
```

Check the individuals that did not pass our filtering.

```
head output/populations/file5.kin0
```

```
## #FID1    IID1    FID2    IID2    NSNP    HETHET  IBS0    KINSHIP
## KAN  1327    KAN 1325    58810   0.226679    0.00851896  0.400443
## OKI  1010    OKI 1006    57912   0.219903    0.0134687   0.356474
```

We want to remove one of the individuals of the pairs.

```
plink2 \
--allow-extra-chr \
--bfile output/populations/file4 \
--extract output/populations/indepSNP.prune.in \
--make-king triangle bin \
--out output/populations/file6 \
--silent;
grep 'variants\|samples' output/populations/file6.log
```

```
## 239 samples (30 females, 69 males, 140 ambiguous; 239 founders) loaded from
## 82731 variants loaded from output/populations/file4.bim.
## --extract: 60674 variants remaining.
## 60674 variants remaining after main filters.
## --make-king pass 1/1: Scanning for rare variants... done.
## 0 variants handled by initial scan (60674 remaining).
## --make-king: 60674 variants processed.
```

Now we can use Plink2 to remove one of the mosquitoes from the pair
with high kinship. It will remove 2 samples since we had 2 samples in
some populations with high relatedness, and we could remove 1 and keep
the other two. Plink2 always tries to maximize the number of samples
passing the filters.

```
plink2 \
--allow-extra-chr \
--bfile output/populations/file4 \
--king-cutoff output/populations/file6 0.354 \
--make-bed \
--out output/populations/file7 \
--silent;
grep 'samples\|variants\|remaining' output/populations/file7.log
```

```
## 239 samples (30 females, 69 males, 140 ambiguous; 239 founders) loaded from
## 82731 variants loaded from output/populations/file4.bim.
## output/populations/file7.king.cutoff.out.id , and 237 remaining sample IDs
```

Now we can check the individuals that were removed.

```
head -n 10 output/populations/file7.king.cutoff.out.id
```

```
## #FID IID
## OKI  1010
## KAN  1327
```

## 3.7 Quick PCA with Plink using the LD pruned data

```
plink2 \
--allow-extra-chr \
--bfile output/populations/file7 \
--pca allele-wts \
--freq \
--out output/populations/pca_pops \
--silent;
grep 'samples\|variants' output/populations/pca_pops.log
```

```
## 237 samples (30 females, 67 males, 140 ambiguous; 237 founders) loaded from
## 82731 variants loaded from output/populations/file7.bim.
```

Check the files

```
head -n 2 output/populations/pca_pops.eigenvec
```

```
## #FID IID PC1 PC2 PC3 PC4 PC5 PC6 PC7 PC8 PC9 PC10
## OKI  1001    -0.0666771  0.0189132   -0.0894577  -0.076237   0.0751274   -0.0756819  0.0651951   -0.00236584 -0.0160601  0.0183014
```

```
head -n 2 output/populations/pca_pops.eigenval
```

```
## 28.7344
## 17.64
```

Import PCA data

```
# import the data from Plink
pca <- read.delim(
  here(
    "output", "populations", "pca_pops.eigenvec"
  ),
  head = TRUE
)
 
# check head of the file
head(pca)
```

```
##   X.FID  IID        PC1       PC2        PC3        PC4       PC5        PC6
## 1   OKI 1001 -0.0666771 0.0189132 -0.0894577 -0.0762370 0.0751274 -0.0756819
## 2   OKI 1002 -0.0630430 0.0168808 -0.1023800 -0.0339297 0.0427102 -0.0504430
## 3   OKI 1003 -0.0666289 0.0200056 -0.1154830 -0.1028510 0.1169400 -0.1083940
## 4   OKI 1004 -0.0680138 0.0191789 -0.1149250 -0.1342530 0.1607270 -0.1773920
## 5   OKI 1005 -0.0693594 0.0190276 -0.1246080 -0.1467700 0.1794630 -0.1976020
## 6   OKI 1006 -0.0647995 0.0173923 -0.1150670 -0.1035240 0.1062590 -0.1032130
##         PC7         PC8         PC9        PC10
## 1 0.0651951 -0.00236584 -0.01606010  0.01830140
## 2 0.0527060  0.00424887 -0.00187660  0.00163174
## 3 0.1058350  0.01470530 -0.03409440  0.01087970
## 4 0.1894420  0.03108510  0.00170166 -0.04975590
## 5 0.2134870  0.03209460 -0.00140403 -0.05358850
## 6 0.1113970  0.01577530 -0.03972560  0.01148020
```

Import sample attributes

```
# import sample attributes
samples2 <- read.delim(
  here(
    "output", "populations", "Population_meta_data.txt"
  ),
  head = TRUE
)
#
# check head of the file
head(samples2)
```

```
##   geo  range continent    region country pop
## 1   1 native      Asia East Asia   China HAI
## 2   1 Native      Asia East Asia   China HUN
## 3   1 Native      Asia East Asia   China YUN
## 4   1 Native      Asia East Asia   Japan AIZ
## 5   1 Native      Asia East Asia   Japan HIR
## 6   1 Native      Asia East Asia   Japan JAT
```

Check number of samples per population

```
pops_pca <- 
  pca |>
  group_by(X.FID) |>
  summarize(count_distinct = n_distinct(IID))

# check it
head(pops_pca)
```

```
## # A tibble: 6 × 2
##   X.FID count_distinct
##   <chr>          <int>
## 1 BEN               12
## 2 CAM               12
## 3 CHA               12
## 4 GEL                2
## 5 HAI               12
## 6 HAN                4
```

Merge the data

```
df4 <-
  merge(
    pca,
    samples2,
    by.x = 1,
    by.y = 6,
    all.x = T,
    all.y = F
  ) |>
  na.omit()
head(df4)
```

```
##   X.FID IID       PC1        PC2       PC3        PC4       PC5       PC6
## 1   BEN   a 0.0332425 -0.0535430 0.0252149 0.01158490 0.0324649 0.0536790
## 2   BEN 257 0.0328290 -0.0552763 0.0285761 0.01009050 0.0368262 0.0519362
## 3   BEN 261 0.0334594 -0.0538783 0.0269419 0.01730230 0.0373530 0.0485611
## 4   BEN 263 0.0341014 -0.0570405 0.0301381 0.01291350 0.0416342 0.0574442
## 5   BEN 264 0.0338284 -0.0547246 0.0246010 0.00753497 0.0326306 0.0495816
## 6   BEN 262 0.0330521 -0.0568827 0.0283885 0.01373890 0.0414787 0.0565339
##         PC7         PC8       PC9      PC10 geo  range continent     region
## 1 0.0440326 -0.00890912 0.0217571 0.0439923   2 Native      Asia South Asia
## 2 0.0508604 -0.00314432 0.0215693 0.0476881   2 Native      Asia South Asia
## 3 0.0523802 -0.01025400 0.0184817 0.0431707   2 Native      Asia South Asia
## 4 0.0480215 -0.00989342 0.0220571 0.0536394   2 Native      Asia South Asia
## 5 0.0459036 -0.00580143 0.0197725 0.0404343   2 Native      Asia South Asia
## 6 0.0536163 -0.01271660 0.0228105 0.0595990   2 Native      Asia South Asia
##   country
## 1   India
## 2   India
## 3   India
## 4   India
## 5   India
## 6   India
```

Get some data for the PCA plot

```
# How many populations
length(unique(df4$X.FID))
```

```
## [1] 27
```

```
# How many countries
length(unique(df4$country))
```

```
## [1] 12
```

```
# How many regions
length(unique(df4$region))
```

```
## [1] 3
```

```
# How many continents
length(unique(df4$continent))
```

```
## [1] 1
```

Check the countries

```
unique(df4$country)
```

```
##  [1] "India"     "Thailand"  "Bhutan"    "China"     "Vietnam"   "Indonesia"
##  [7] "Sri Lanka" "Japan"     "Nepal"     "Malaysia"  "Maldives"  "Taiwan"
```

Check the regions

```
unique(df4$region)
```

```
## [1] "South Asia"     "Southeast Asia" "East Asia"
```

Make plot1

```
# source the plotting function
source(here("scripts", "analysis", "my_theme2.R"))

# Shapes
N = 100
M = 1000
good.shapes = c(1:25, 33:127)

# Colors
palette1 <- brewer.pal(12, "Paired")
palette2 <- brewer.pal(11, "Set3")
palette23 <- c(palette1, palette2)

# Compute the count for each country
country_count <- df4 |>
  group_by(country) |>
  summarize(count = n())

# Merge the count back to the main data
df4 <- df4 |>
  left_join(country_count, by = "country")

# Create a custom label for the legend
df4$country_label <-
  paste(df4$country, " (", df4$count, ")", sep = "")

# Define the color and shape manually
palette23 <- c(palette1, palette2)
colors <- setNames(palette23, unique(df4$country_label))
shapes <-
  setNames(good.shapes[c(1:25, 58:67)], unique(df4$country_label))

# Replace " Asia" with an empty string
df4$region <- str_replace(df4$region, " Asia", "")

# Compute the center of ellipses for each continent
ellipse_centers <- df4 |>
  group_by(region) |>
  summarise(PC1_center = mean(PC1), PC2_center = mean(PC2))

# # Calculate the number of samples per region
continent_count <- df4 |>
  group_by(region) |>
  summarise(count = n())
continent_labels <-
  setNames(
    paste(continent_count$region, " (", continent_count$count, ")", sep = ""),
    continent_count$region
  )

# Define the colors you want for each continent
continent_colors <-
  c("red", "blue", "green") # Adjust these colors to your preference

# Create the plot
ggplot(df4, aes(PC1, PC2)) +
  geom_point(aes(shape = country_label, color = country_label)) +
  stat_ellipse(
    aes(fill = region, group = region),
    geom = "polygon",
    alpha = 0.2,
    level = 0.8,
    segments = 40,
    color = "pink"
  ) +
  stat_ellipse(
    aes(group = region),
    geom = "path",
    level = 0.8,
    segments = 40,
    color = "pink"
  ) + # Line around the ellipse
  geom_text_repel(
    data = ellipse_centers,
    aes(x = PC1_center, y = PC2_center, label = region),
    color = "magenta"
  ) + # Add labels using ggrepel
  xlab("PC1 (18.04% Variance)") +
  ylab("PC2 (12.25% Variance)") +
  labs(caption = "Principal Component Analysis with 60,674 SNPs \n of mosquitoes from 26 localities across 12 countries in Asia.") +
  guides(
    color = guide_legend(
      title = "Country",
      order = 2,
      ncol = 1
    ),
    shape = guide_legend(
      title = "Country",
      order = 2,
      ncol = 1
    ),
    fill = guide_legend(
      title = "Continent",
      order = 1,
      ncol = 1
    )
  ) +
  scale_fill_manual(values = continent_colors, labels = continent_labels) +
  scale_color_manual(values = colors) +
  scale_shape_manual(values = shapes) +
  my_theme() +
  theme(
    plot.caption = element_text(face = "italic"),
    legend.position = "right",
    legend.justification = "top",
    legend.box.just = "center",
    legend.box.background = element_blank(),
    plot.margin = margin(5.5, 30, 5.5, 5.5, "points"),
    legend.margin = margin(10, 10, 10, 10) # move the legend a bit up
  )
```

```
#   ____________________________________________________________________________
#   save the pca plot                                                       ####
ggsave(
  here(
    "output", "populations", "figures", "PCA_continent_asia_plink.pdf"
  ),
  width  = 10,
  height = 6,
  units  = "in"
)
```

# 4. Create chromosomal scale

Import the .bim file with the SNPs to create a new chromosomal
scale.

```
#   ____________________________________________________________________________
#   import the bim file with the SNP data                                   ####
snps <-
  read_delim(                    # to learn about the options use here, run ?read_delim on the console.
    here(
      "output", "populations", "file7.bim"
    ),                           # use library here to load it
    col_names      = FALSE,      # we don't have header in the input file
    show_col_types = FALSE,      # suppress message from read_delim
    col_types      = "ccidcc"    # set the class of each column
  )
#
# set column names
colnames(
  snps
) <-                             # to add a header in our tibble
  c(
    "Scaffold", "SNP", "Cm", "Position", "Allele1", "Allele2"
  )
#
# check the tibble
head(snps)
```

```
## # A tibble: 6 × 6
##   Scaffold SNP             Cm Position Allele1 Allele2
##   <chr>    <chr>        <int>    <dbl> <chr>   <chr>  
## 1 1        AX-583033342     0   315059 C       G      
## 2 1        AX-583033370     0   330057 G       A      
## 3 1        AX-583035194     0   330265 A       G      
## 4 1        AX-583033387     0   331288 C       T      
## 5 1        AX-583035257     0   442875 T       C      
## 6 1        AX-583035355     0   540754 G       C
```

We can write a function to import the bim files.

```
#   ____________________________________________________________________________
#   function to import bim files                                            ####
#
import_bim <- function(file) {
  # import as a tibble and set columns as integers
  bim <-
    read_delim(
      file,
      col_names      = FALSE,
      show_col_types = FALSE,
      col_types      = "ccidcc"
    )
  # rename the columns by index
  bim <- bim |>
    rename(
      Scaffold = 1,
      SNP      = 2,
      Cm       = 3,
      Position = 4,
      Allele1  = 5,
      Allele2  = 6
    )
  return(bim)
}
# we can save the function to source it later
dump(                                                     # check ?dump for more information
  "import_bim",                                           # the object we want to save
  here(
    "scripts", "analysis", "import_bim.R")       # use here to save it our function as .R 
)
```

Separate the tibbles into each chromosome.

```
#   ____________________________________________________________________________
#   separate the SNP data per chromosome                                    ####
# chr1
chr1_snps <-
  snps |>
  filter(
    str_detect(
      Scaffold, "^1."
    )
  ) |> # here we get only Scaffold rows starting with 1
  as_tibble() # save as tibble
#
# chr2
chr2_snps <-
  snps |>
  filter(
    str_detect(
      Scaffold, "^2."
    )
  ) |>
  as_tibble()
#
# chr3
chr3_snps <-
  snps |>
  filter(
    str_detect(
      Scaffold, "^3."
    )
  ) |>
  as_tibble()
```

Now we can index the reference genome with the new scaffold names
that match our .bim file

```
# index the genome
samtools faidx data/genome/albo.fasta.gz
```

Transfer genome to the cluster.

```
# change the paths to match your directories
rsync -chavzP --stats /Users/lucianocosme/Dropbox/Albopictus/manuscript_chip/data/no_autogenous/albo_chip/data/genome/* lvc26@ruddle.hpc.yale.edu:/ycga-gpfs/project/caccone/lvc26/albo_manuscript/updated_genome
```

Now we can get the scaffold order and their size from the
`.fai` file. Check the about it at `Samtools`
documentation HERE.

```
# check the head of the .fai file
head data/genome/albo.fasta.gz.fai
# For each row:
# Column 1: The scaffold name. In your FASTA file, this is preceded by '>'
# Column 2: The number of bases in the scaffol
# Column 3: The byte index of the file where the scaffold sequence begins.
# Column 4: bases per line in the FASTA file
# Column 5: bytes per line in the FASTA file
# 
# we can use awk to get the first two columns, I also change the field separator.
cat data/genome/albo.fasta.gz.fai | awk 'BEGIN{FS=" "; OFS="\t"} {print $1, $2}' > data/genome/scaffold_sizes.txt;
echo "scaffold sizes";
# check the output
head data/genome/scaffold_sizes.txt
# since we fixed the scaffold order previous, and also moved the scaffold 1.86 to its correct position, we can move ahead and calculate the new scale for our SNPs.
```

Import the file with sizes of each scaffold.

```
#   ____________________________________________________________________________
#   import the file with the scaffold sizes                                 ####
sizes <-
  read_delim(
    here(
      "data", "genome", "scaffold_sizes.txt"
    ),
    col_names      = FALSE,
    show_col_types = FALSE,
    col_types      = "cd"
  )
#
# set column names
colnames(
  sizes
) <- c(
  "Scaffold", "Size"
)
#   ____________________________________________________________________________
#   create new column with the chromosome number                            ####
sizes <- 
  sizes |>
  mutate(
    Chromosome = case_when( # we use mutate to create a new column called Chromosome
      startsWith(
        Scaffold, "1"
      ) ~ "1", # use startsWith to get Scaffold rows starting with 1 and output 1 on Chromosome column
      startsWith(
        Scaffold, "2"
      ) ~ "2",
      startsWith(
        Scaffold, "3"
      ) ~ "3"
    )
  ) |>
  arrange(
    Scaffold
  )                   # to sort the order of the scaffolds, fixing the problem we have with scaffold 1.86
# check it
head(sizes)
```

```
## # A tibble: 6 × 3
##   Scaffold     Size Chromosome
##   <chr>       <dbl> <chr>     
## 1 1.1        351198 1         
## 2 1.10     11939576 1         
## 3 1.100     3389100 1         
## 4 1.101      470438 1         
## 5 1.102     2525157 1         
## 6 1.103      150026 1
```

Create new scale. Get the scaffolds for each chromosome.

```
#   ____________________________________________________________________________
#   separate the scaffold sizes tibble per chromosome                       ####
# chr1
chr1_scaffolds <- 
  sizes |>
  filter(
    str_detect(
      Scaffold, "^1" # we use library stringr to get scaffolds starting with 1 (chromosome 1)
    )
  ) |> 
  as_tibble()
#
# chr2
chr2_scaffolds <-
  sizes |>
  filter(
    str_detect(
      Scaffold, "^2" # we use library stringr to get scaffolds starting with 2 (chromosome 2)
    )
  ) |> 
  as_tibble()
#
# # chr3
chr3_scaffolds <-
  sizes |>
  filter(
    str_detect(
      Scaffold, "^3" # we use library stringr to get scaffolds starting with 3 (chromosome 3)
    )
  ) |>
  as_tibble()
```

Create a scale for each chromosome.

```
#   ____________________________________________________________________________
#   create a new scale for each chromosome                                  ####
# chr1
chr1_scaffolds$overall_size_before_bp <-
  0                                                                        # we create a new column with zeros
for (i in 2:nrow(
  chr1_scaffolds
)
) {                                                                        # loop to start on second line
  chr1_scaffolds$overall_size_before_bp[i] <-                              # set position on the scale
    chr1_scaffolds$overall_size_before_bp[i - 1] + chr1_scaffolds$Size[i - # add the scaffold size and the location to get position on new scale
      1]
}
#
# chr2
chr2_scaffolds$overall_size_before_bp <- 0
for (i in 2:nrow(
  chr2_scaffolds
)
) {
  chr2_scaffolds$overall_size_before_bp[i] <-
    chr2_scaffolds$overall_size_before_bp[i - 1] + chr2_scaffolds$Size[i -
      1]
}
#
# chr3
chr3_scaffolds$overall_size_before_bp <- 0
for (i in 2:nrow(
  chr3_scaffolds
)
) {
  chr3_scaffolds$overall_size_before_bp[i] <-
    chr3_scaffolds$overall_size_before_bp[i - 1] + chr3_scaffolds$Size[i -
      1]
}
```

Merge the data frames scaffolds and SNPs.

```
#   ____________________________________________________________________________
#   merge the data sets using the tidyverse function left_join              ####
# chr1
chr1_scale <-
  chr1_snps |>          # create data frame for each chromosome, get chr1_snps
  left_join(            # use lef_join function to merge it with chr1_scaffolds
    chr1_scaffolds,
    by = "Scaffold"
  ) |>                  # set column to use for merging (Scaffold in this case)
  na.omit() |>          # remove NAs, we don't have SNPs in every scaffold
  mutate(
    midPos_fullseq = as.numeric(
      Position
    ) +                 # make new columns numeric
      as.numeric(
        overall_size_before_bp
      )
  )
#
# chr2
chr2_scale <-
  chr2_snps |>
  left_join(
    chr2_scaffolds,
    by = "Scaffold"
  ) |>
  na.omit() |>
  mutate(
    midPos_fullseq = as.numeric(
      Position
    ) +
      as.numeric(
        overall_size_before_bp
      )
  )
#
# chr3
chr3_scale <-
  chr3_snps |>
  left_join(
    chr3_scaffolds,
    by = "Scaffold"
  ) |>
  na.omit() |>
  mutate(
    midPos_fullseq = as.numeric(
      Position
    ) +
      as.numeric(
        overall_size_before_bp
      )
  )
```

Merge all chromosome scales.

```
#   ____________________________________________________________________________
#   merge the data sets, and select only the columns we need                ####
chroms <- rbind(
  chr1_scale, chr2_scale, chr3_scale
) |>
  dplyr::select(
    Chromosome, SNP, Cm, midPos_fullseq, Allele1, Allele2
  )
# check it
head(chroms)
```

```
## # A tibble: 6 × 6
##   Chromosome SNP             Cm midPos_fullseq Allele1 Allele2
##   <chr>      <chr>        <int>          <dbl> <chr>   <chr>  
## 1 1          AX-583033342     0         315059 C       G      
## 2 1          AX-583033370     0         330057 G       A      
## 3 1          AX-583035194     0         330265 A       G      
## 4 1          AX-583033387     0         331288 C       T      
## 5 1          AX-583035257     0         442875 T       C      
## 6 1          AX-583035355     0         540754 G       C
```

Save the new .bim file

```
#   ____________________________________________________________________________
#   save the new bim file with a new name, I added "B"                      ####
write.table(
  chroms,
  file      = here(
    "output", "populations", "file7B.bim"
  ),
  sep       = "\t",
  row.names = FALSE,
  col.names = FALSE,
  quote     = FALSE
)
```

Rename the .bim files

```
# change the name of the first .bim file, for example, append _backup.bim, and then replace the original file
mv output/populations/file7.bim output/populations/file7_backup.bim;
# than change the new bim we create to the original name (do it only once, otherwise it will mess up)
mv output/populations/file7B.bim output/populations/file7.bim
```

Create a new bed file with Plink2 to see if it works. For example, to
see if the variants are in the right order. Plink2 will give us a
warning.

```
plink2 \
--bfile output/populations/file7 \
--make-bed \
--out output/populations/test01;
# then we remove the files 
rm output/populations/test01.*
```

```
## PLINK v2.00a3.3 64-bit (3 Jun 2022)            www.cog-genomics.org/plink/2.0/
## (C) 2005-2022 Shaun Purcell, Christopher Chang   GNU General Public License v3
## Logging to output/populations/test01.log.
## Options in effect:
##   --bfile output/populations/file7
##   --make-bed
##   --out output/populations/test01
## 
## Start time: Fri Oct 27 15:38:43 2023
## 32768 MiB RAM detected; reserving 16384 MiB for main workspace.
## Using up to 12 threads (change this with --threads).
## 237 samples (30 females, 67 males, 140 ambiguous; 237 founders) loaded from
## output/populations/file7.fam.
## 82731 variants loaded from output/populations/file7.bim.
## Note: No phenotype data present.
## Writing output/populations/test01.fam ... done.
## Writing output/populations/test01.bim ... done.
## Writing output/populations/test01.bed ... 0%79%done.
## End time: Fri Oct 27 15:38:43 2023
```

No warnings from Plink2. Now, we can go ahead with our analysis.

## 4.1 Estimate LD blocks using chromosomal scale

```
mkdir -p output/populations/ld/blocks_chr
```

We can use Plink1.9 to estimate LD blocks for the populations with
more than 12 individuals. We will use the entire genome for this part
instead of the larger scaffolds only. We will set max distance of LD
blocks of 500kb. We found out that the average half distance of r^2 max
is small, from 1 to 5kb

```
for fam in $(awk '{print $1}' output/populations/ld/pops_4ld.txt | sort | uniq); 
do 
echo $fam | \
plink \
--allow-extra-chr \
--keep-allele-order \
--bfile output/populations/file7 \
--keep-fam /dev/stdin \
--maf 0.1 \
--blocks no-pheno-req \
--blocks-max-kb 200 \
--out output/populations/ld/blocks_chr/$fam \
--geno 0.1 \
--silent
done;
#
rm output/populations/ld/blocks_chr/*.nosex
```

Now we can get some data from our .log files

```
echo "Population,n,nVariants,geno,maf,passQC" > output/populations/ld/blocks_chr/table_ld_stats.csv
for file in output/populations/ld/blocks_chr/*.log
do
  variants=$(grep -oE '([0-9]+) variants loaded from \.bim file' $file | grep -oE '[0-9]+')
  geno=$(grep -oE '([0-9]+) variants removed due to missing genotype data \(--geno\)' $file | grep -oE '[0-9]+')
  maf=$(grep -oE '([0-9]+) variants removed due to minor allele threshold\(s\)' $file | grep -oE '[0-9]+')
  pass=$(grep -oE '([0-9]+) variants and [0-9]+ people pass filters and QC\.' $file | grep -oE '[0-9]+' | head -1)
  n=$(grep -oE '([0-9]+) variants and [0-9]+ people pass filters and QC\.' $file | grep -oE '[0-9]+' | tail -1)
  filename=$(basename $file .log)
  echo "$filename,$n,$variants,$geno,$maf,$pass" >> output/populations/ld/blocks_chr/table_ld_stats.csv
done;
head -n 5 output/populations/ld/blocks_chr/table_ld_stats.csv
```

```
## Population,n,nVariants,geno,maf,passQC
## BEN,12,82731,4630,26625,51476
## CAM,12,82731,6183,21869,54679
## CHA,12,82731,3541,24580,54610
## HAI,12,82731,4016,18267,60448
```

We can check it out

```
# Load data from the table_ld_stats.csv file
ld_blocks <- read.csv(
  here(
    "output", "populations", "ld", "blocks_chr", "table_ld_stats.csv"
    ),
  header = TRUE,
  sep = ","
)

# Create the flextable
ft <- flextable(ld_blocks)

# Apply zebra theme
ft <- theme_zebra(ft)

# Add a caption to the table
ft <- flextable::add_header_lines(ft, "Table 1: Summary of quality control for population data.")

# Save it to a Word document
officer::read_docx() |>
  body_add_flextable(ft) |>
  print(target = here::here("output", "populations", "summary_blocks_chr.docx"))

ft
```

| Table 1: Summary of quality control for population data. | | | | | |
| --- | --- | --- | --- | --- | --- |
| Population | n | nVariants | geno | maf | passQC |
| BEN | 12 | 82,731 | 4,630 | 26,625 | 51,476 |
| CAM | 12 | 82,731 | 6,183 | 21,869 | 54,679 |
| CHA | 12 | 82,731 | 3,541 | 24,580 | 54,610 |
| HAI | 12 | 82,731 | 4,016 | 18,267 | 60,448 |
| HUN | 12 | 82,731 | 3,971 | 17,380 | 61,380 |
| KAG | 12 | 82,731 | 5,015 | 22,815 | 54,901 |
| KAN | 11 | 82,731 | 4,522 | 29,212 | 48,997 |
| MAT | 12 | 82,731 | 3,949 | 25,368 | 53,414 |
| OKI | 11 | 82,731 | 5,146 | 24,667 | 52,918 |
| QNC | 12 | 82,731 | 5,212 | 31,599 | 45,920 |
| SSK | 12 | 82,731 | 3,685 | 24,847 | 54,199 |
| UTS | 12 | 82,731 | 4,215 | 23,866 | 54,650 |

Now we can count how many blocks we found in each population

```
wc -l output/populations/ld/blocks_chr/*.blocks | \
  awk '{population = gensub(/\.blocks_chr/, "", "g", $2); print population "\t" $1}' | \
  sed 's#output/populations/ld/blocks_chr/##' | \
  sed 's/.blocks//' | \
  sed '$d' > output/populations/ld/blocks_chr/populations_block_counts.csv;
head -n 30 output/populations/ld/blocks_chr/populations_block_counts.csv
```

```
## BEN  10
## CAM  14
## CHA  8
## HAI  49
## HUN  38
## KAG  107
## KAN  158
## MAT  16
## OKI  86
## QNC  41
## SSK  8
## UTS  97
```

Now we can add the number of blocks to the table we made

```
# Load data from the table_ld_stats.csv file
ld_blocks <- read.csv(
  here(
    "output", "populations", "ld", "blocks_chr", "table_ld_stats.csv"
    ),
  header = TRUE,
  sep = ","
)

# Load the population counts data from the CSV file
pop_counts <-
  read.delim(
    here(
      "output",
      "populations",
      "ld",
      "blocks_chr",
      "populations_block_counts.csv"
    ),
    header = F,
    sep = "\t"
  ) |>
  rename(
    Population       = 1,
    nBlocks          = 2
  )

# Merge the population counts with the table data
ld_blocks <- merge(ld_blocks, pop_counts, by = "Population")

# Create the flextable
ft <- flextable(ld_blocks)

# Apply zebra theme
ft <- theme_zebra(ft)

# Add a caption to the table
ft <- flextable::add_header_lines(ft, "Table 2: Number of linkage blocks detected with Plink for populations with at least 10 individuals.")

# Save it to a Word document
officer::read_docx() |>
  body_add_flextable(ft) |>
  print(target = here::here("output", "populations", "summary_ld_blocks_chr.docx"))

ft
```

| Table 2: Number of linkage blocks detected with Plink for populations with at least 10 individuals. | | | | | | |
| --- | --- | --- | --- | --- | --- | --- |
| Population | n | nVariants | geno | maf | passQC | nBlocks |
| BEN | 12 | 82,731 | 4,630 | 26,625 | 51,476 | 10 |
| CAM | 12 | 82,731 | 6,183 | 21,869 | 54,679 | 14 |
| CHA | 12 | 82,731 | 3,541 | 24,580 | 54,610 | 8 |
| HAI | 12 | 82,731 | 4,016 | 18,267 | 60,448 | 49 |
| HUN | 12 | 82,731 | 3,971 | 17,380 | 61,380 | 38 |
| KAG | 12 | 82,731 | 5,015 | 22,815 | 54,901 | 107 |
| KAN | 11 | 82,731 | 4,522 | 29,212 | 48,997 | 158 |
| MAT | 12 | 82,731 | 3,949 | 25,368 | 53,414 | 16 |
| OKI | 11 | 82,731 | 5,146 | 24,667 | 52,918 | 86 |
| QNC | 12 | 82,731 | 5,212 | 31,599 | 45,920 | 41 |
| SSK | 12 | 82,731 | 3,685 | 24,847 | 54,199 | 8 |
| UTS | 12 | 82,731 | 4,215 | 23,866 | 54,650 | 97 |

Get the size of each block from the .block.det files

```
get_kb_column <- function(dir_path) {
  # obtain the list of files with extension .blocks.det
  file_names <- list.files(path = dir_path, pattern = "\\.blocks\\.det$", full.names = TRUE)
  
  # create an empty list to hold the data frames
  block_list <- list()
  
  # loop through the files and read the data into the list
  for (file in file_names) {
    df <- read.table(file, header = TRUE, check.names = FALSE, stringsAsFactors = FALSE)
    # select only the KB column and add it to the block_list with the file name
    block_list[[file]] <- df |> dplyr::select(KB) |> add_column(file = file, .before = 1)
  }
  
  # combine the data frames in the block_list into a single data frame
  blocks <- bind_rows(block_list)
  
  # clean up the file name column
  blocks$file <- str_remove(blocks$file, "^.*\\/ld\\/blocks\\/")
  
  return(blocks)
}

# example usage: replace dir_path with your directory path
dir_path <- here("output", "populations", "ld", "blocks_chr")

blocks<- 
  get_kb_column(dir_path) |>
  mutate(file = str_remove(file, "/Users/lucianocosme/Library/CloudStorage/Dropbox/Albopictus/manuscript_chip/data/no_autogenous/albo_chip/output/populations/ld/blocks_chr/")) |>
  mutate(file = str_remove(file, ".blocks.det")) |>

  as_tibble() |>
  rename(
    Population = 1
  )
```

Create density plot of the size of the LD blocks Plink found

```
# to check how many colors we need
# n_distinct(blocks$Population)

# define the color palette with 63 color blind colors
colors <-
  c("#0072B2", "#E69F00", "#009E73", "#F0E442", "#56B4E9", "#D55E00", "#CC79A7", "#000000", "#999999", "#E5A8B0", "#B5BB5E", "#5F9E6E", "#E6C9AF", "#A98D8E", "#C4B6D7", "#88CCEE", "#CC6677", "#DDCC77", "#117733", "#332288", "#44AA99", "#999933", "#CC4452", "#88B3A3", "#FFFF99", "#E69F00", "#56B4E9", "#D55E00", "#CC79A7", "#0072B2", "#009E73", "#F0E442", "#000000", "#999999", "#E5A8B0", "#B5BB5E", "#5F9E6E", "#E6C9AF", "#A98D8E", "#C4B6D7", "#88CCEE", "#CC6677", "#DDCC77", "#117733", "#332288", "#44AA99", "#999933", "#CC4452", "#88B3A3", "#FFFF99", "#E69F00", "#56B4E9", "#D55E00", "#CC79A7", "#0072B2", "#009E73", "#F0E442", "#000000", "#999999", "#E5A8B0", "#B5BB5E", "#5F9E6E", "#E6C9AF", "#A98D8E", "#C4B6D7", "#88CCEE")

# make plot using the sample y scale for all populations
ggplot(blocks, aes(x = KB)) +
  stat_density(
    aes(y = after_stat(count), fill = factor(Population)),
    linewidth = .5,
    alpha = .4,
    position = "identity"
  ) +
  scale_fill_manual(values = colors) +
  scale_x_continuous(labels = comma, name = "Block length (kb)") +
  scale_y_continuous(labels = comma, name = "Count") +
  theme(
    plot.title = element_text(hjust = 0.5, size = 18, face = "bold"),
    axis.text = element_text(size = 12),
    axis.title = element_text(size = 16, face = "bold"),
    strip.text = element_text(size = 14, face = "bold"),
    plot.background = element_rect(fill = "white"),
    panel.background = element_rect(fill = 'white', colour = 'black')
  ) +
  guides(fill = "none") +
  facet_wrap( ~ Population, ncol = 3) + my_theme()
```

```
# save the plot as a PDF using ggsave
ggsave(
  here(
    "output",
    "populations",
    "figures",
    "block_density_y_scale_fixed_chr.pdf"
  ),
  width = 10,
  height = 10,
  units = "in"
)
```

Make plot allowing the y axis scale free

```
ggplot(blocks, aes(x = KB)) +
  stat_density(
    aes(y = after_stat(count), fill = factor(Population)),
    linewidth = .5,
    alpha = .4,
    position = "identity"
  ) +
  scale_fill_manual(values = colors) +
  scale_x_continuous(labels = comma, name = "Block length (kb)") +
  scale_y_continuous(labels = comma, name = "Count") +
  theme(
    plot.title = element_text(hjust = 0.5, size = 18, face = "bold"),
    axis.text = element_text(size = 12),
    axis.title = element_text(size = 16, face = "bold"),
    strip.text = element_text(size = 14, face = "bold"),
    plot.background = element_rect(fill = "white"),
    panel.background = element_rect(fill = 'white', colour = 'black')
  ) +
  guides(fill = "none") +
  facet_wrap( ~ Population, ncol = 3, scales = "free_y") +
  my_theme()
```

```
# save the plot as a PDF using ggsave
ggsave(
  here(
    "output",
    "populations",
    "figures",
    "block_density_free_scale_chr.pdf"
  ),
  width = 20,
  height = 18,
  units = "in"
)
```

# 5. SNP annotation lifting to different genome assemblies

We mapped the probe sequences to all available in NCBI and
VectorBase. We can lift the annotation of the SNPs to all genomes.

## 5.1 AalbF2

Check the results of the mapping.

```
head output/probes/results_cluster/bwa/bwa_mem_default_results/AalbF2_ncbi.albopictus_Ref_F.txt
```

```
## Read_Name Ref_Name Start End SNP Strand Mapping_Quality Mismatches Indels
## AX-582945972_F NW_021837045 11080 11150 11115 - 60 0 0
## AX-582191512_F NW_021837045 20143 20143 20178 - 0 1 0
## AX-583160724_F NW_021837045 21036 21036 21071 - 0 0 0
## AX-582943846_F NW_021837045 22286 22356 22321 + 18 0 0
## AX-582943848_F NW_021837045 22597 22667 22632 + 60 0 0
## AX-583160720_F NW_021837045 23055 23055 23090 - 0 0 0
## AX-584286814_F NW_021837045 45905 45905 45940 + 0 6 1
## AX-582943855_F NW_021837045 78569 78639 78604 + 7 0 0
## AX-583994837_F NW_021837045 109750 109750 109785 - 0 1 0
```

We can import the data into R

Mapping results for the probe sequences with the reference allele
(AalbF3)

```
# Load the data into a data table directly using `here` in `fread`
AalbF2_ref_F <-
  fread(
    here(
      "output",
      "probes",
      "results_cluster",
      "bwa",
      "bwa_mem_default_results",
      "AalbF2_ncbi.albopictus_Ref_F.txt"
    ),
    sep = " "
  )

# Remove the "_F" from the end of the strings in the "Read_Name" column
AalbF2_ref_F$Read_Name <- sub("_F$", "", AalbF2_ref_F$Read_Name)

# Rename the "Reference" column to "SNP_id"
setnames(AalbF2_ref_F, "Read_Name", "SNP_id")

# Rename the "SNP" column to "Position"
setnames(AalbF2_ref_F, "SNP", "Position")

head(AalbF2_ref_F)
```

```
##          SNP_id     Ref_Name Start   End Position Strand Mapping_Quality
## 1: AX-582945972 NW_021837045 11080 11150    11115      -              60
## 2: AX-582191512 NW_021837045 20143 20143    20178      -               0
## 3: AX-583160724 NW_021837045 21036 21036    21071      -               0
## 4: AX-582943846 NW_021837045 22286 22356    22321      +              18
## 5: AX-582943848 NW_021837045 22597 22667    22632      +              60
## 6: AX-583160720 NW_021837045 23055 23055    23090      -               0
##    Mismatches Indels
## 1:          0      0
## 2:          1      0
## 3:          0      0
## 4:          0      0
## 5:          0      0
## 6:          0      0
```

I created a list of SNPs with unique mapping and zero mismatches. We
can import it

```
# Load the data into a data table directly using `here` in `fread`
AalbF2_ref_u <- fread(
  here(
    "output",
    "probes",
    "results_cluster",
    "bwa",
    "bwa_mem_default_results",
    "AalbF2_ncbi.albopictus_Ref_F_unique_no_mismatches_ids.txt"
  ),
  sep = " ",
  header = FALSE # Treat the first line as data
)

# Name the column as "SNP_id"
setnames(AalbF2_ref_u, "V1", "SNP_id")

# Remove the "_F" from the end of the strings in the "Read_Name" column
AalbF2_ref_u$SNP_id <- sub("_F$", "", AalbF2_ref_u$SNP_id)

head(AalbF2_ref_u)
```

```
##          SNP_id
## 1: AX-579436016
## 2: AX-579436089
## 3: AX-579436102
## 4: AX-579436125
## 5: AX-579436149
## 6: AX-579436196
```

Before we do any data tidying we can check how many probe sequences
mapped with mismatches, low quality, or have indels.

```
# Count the number of SNP_id with Mapping_Quality less than 20
count_MQ_lt_20_ref <- nrow(AalbF2_ref_F[Mapping_Quality < 20])

# Count the number of SNP_id with Mismatches greater than 0
count_MM_gt_0_ref <- nrow(AalbF2_ref_F[Mismatches > 0])

# Count the number of SNP_id with Indels greater than 0
count_IN_gt_0_ref <- nrow(AalbF2_ref_F[Indels > 0])

# Create a table of the counts of each SNP_id
SNP_id_counts_ref <- table(AalbF2_ref_F$SNP_id)

# Find which SNP_ids appear more than once and count them
SNP_id_more_than_once_ref <- length(which(SNP_id_counts_ref > 1))

# Find which SNP_ids appear exactly once and count them
SNP_id_only_once_ref <- length(which(SNP_id_counts_ref == 1))


# Print the counts
print(paste("Number of SNP_id with Mapping_Quality < 20: ", count_MQ_lt_20_ref))
```

```
## [1] "Number of SNP_id with Mapping_Quality < 20:  223561"
```

```
print(paste("Number of SNP_id with Mismatches > 0: ", count_MM_gt_0_ref))
```

```
## [1] "Number of SNP_id with Mismatches > 0:  109614"
```

```
print(paste("Number of SNP_id with Indels > 0: ", count_IN_gt_0_ref))
```

```
## [1] "Number of SNP_id with Indels > 0:  23311"
```

```
print(paste("Number of SNP_id appearing more than once: ", SNP_id_more_than_once_ref))
```

```
## [1] "Number of SNP_id appearing more than once:  102442"
```

```
print(paste("Number of SNP_id appearing only once: ", SNP_id_only_once_ref))
```

```
## [1] "Number of SNP_id appearing only once:  72954"
```

Some probe sequences mapped in more than one place. Lets filter the
data by mapping quality > 20, allowing no mismatches or indels. The
genome assembly AalbF2 is around 2.5Gb while the AalbF3 is around 1.5Gb.
We used AalbF3 to design the probe sequences.

```
# Filter the data based on your conditions
filtered_AalbF2_ref_F <- AalbF2_ref_F[Mapping_Quality >= 20 & Mismatches == 0 & Indels == 0]
```

Lets compare before and after filtering

```
print(paste("Number of SNP before filtering: ", length(unique(AalbF2_ref_F$SNP_id))))
```

```
## [1] "Number of SNP before filtering:  175396"
```

```
print(paste("Number of SNP after filtering: ", length(unique(filtered_AalbF2_ref_F$SNP_id))))
```

```
## [1] "Number of SNP after filtering:  118480"
```

Only 118,480 out of the 175,396 SNPs had unique mapping with zero
mismatches or indels in the AalbF2.

We can check the output of the mapping of the probe sequence with the
alternative allele. We can allow 1 mismatch since it has the alternative
allele.

Merge the data tables

```
# Perform the inner join
AalbF2_ref <- AalbF2_ref_F[AalbF2_ref_u, on = "SNP_id"]
head(AalbF2_ref)
```

```
##          SNP_id     Ref_Name    Start      End Position Strand Mapping_Quality
## 1: AX-579436016 NW_021837378 32463395 32463465 32463430      -              60
## 2: AX-579436089 NW_021837378 32462227 32462297 32462262      -              60
## 3: AX-579436102 NW_021837378 32461993 32462063 32462028      -              60
## 4: AX-579436125 NW_021837378 32461571 32461641 32461606      -              60
## 5: AX-579436149 NW_021837378 32461088 32461158 32461123      -              60
## 6: AX-579436196 NW_021837378 32460378 32460448 32460413      -              60
##    Mismatches Indels
## 1:          0      0
## 2:          0      0
## 3:          0      0
## 4:          0      0
## 5:          0      0
## 6:          0      0
```

Mapping results for the probe sequences with the alternative allele
(AalbF3)

```
# Load the data into a data table directly using `here` in `fread`
AalbF2_alt_F <-
  fread(
    here(
      "output",
      "probes",
      "results_cluster",
      "bwa",
      "bwa_mem_default_results",
      "AalbF2_ncbi.albopictus_alt_F.txt"
    ),
    sep = " "
  )


# Remove the "_F" from the end of the strings in the "Read_Name" column
AalbF2_alt_F$Read_Name <- sub("_F$", "", AalbF2_alt_F$Read_Name)

# # Create a new column "Reference" by appending "_alt" to the "Read_Name" values
# AalbF2_alt_F[, Reference := paste0(Read_Name, "_alt")]

# Rename the "Reference" column to "SNP_id"
setnames(AalbF2_alt_F, "Read_Name", "SNP_id")

# Rename the "SNP" column to "Position"
setnames(AalbF2_alt_F, "SNP", "Position")

head(AalbF2_alt_F)
```

```
##          SNP_id     Ref_Name Start   End Position Strand Mapping_Quality
## 1: AX-582945972 NW_021837045 11080 11150    11115      -              56
## 2: AX-582191512 NW_021837045 20143 20143    20178      -               0
## 3: AX-583160724 NW_021837045 21036 21036    21071      -               0
## 4: AX-582943846 NW_021837045 22286 22356    22321      +              17
## 5: AX-582943848 NW_021837045 22597 22667    22632      +              48
## 6: AX-583160720 NW_021837045 23055 23055    23090      -               0
##    Mismatches Indels
## 1:          1      0
## 2:          2      0
## 3:          1      0
## 4:          1      0
## 5:          1      0
## 6:          1      0
```

Lets filter the alt allele object

```
# Filter the data based on your conditions
filtered_AalbF2_alt_F <- AalbF2_alt_F[Mapping_Quality >= 20 & Mismatches == 1 & Indels == 0]
```

Lets compare before and after filtering

```
print(paste("Number of SNP before filtering: ", length(unique(AalbF2_alt_F$SNP_id))))
```

```
## [1] "Number of SNP before filtering:  175396"
```

```
print(paste("Number of SNP after filtering: ", length(unique(filtered_AalbF2_alt_F$SNP_id))))
```

```
## [1] "Number of SNP after filtering:  108830"
```

Although we do not know for sure if the mismatch is at the SNP
position, we can see that we have 108,830 probe sequences with the
alternative allele mapping uniquely to AalbF2.

We can get the overlap of the SNP ids between the two objects

```
# Calculate shared values
AalbF2_SNPs <- intersect(filtered_AalbF2_ref_F$SNP_id, filtered_AalbF2_alt_F$SNP_id)

library(ggvenn)
# Create Venn diagram
venn_AalbF2 <- list(
  "Reference" = filtered_AalbF2_ref_F$SNP_id,
  "Alternative" = filtered_AalbF2_alt_F$SNP_id
)
venn_plot2 <- ggvenn(venn_AalbF2, fill_color = c("steelblue", "darkorange"), show_percentage = TRUE)

# Add a title
venn_plot2 <- venn_plot2 +
  ggtitle("AalbF2 unique mapping") +
  theme(plot.title = element_text(hjust = .5))

# Display the Venn diagram
print(venn_plot2)
```

We have 108,812 SNPs that we can use with AalbF2. Before we move
ahead, we can extract this SNPs from each data set and see if their
coordinates match.

Lets filter the data tables to keep only the shared SNP\_id

```
# Filter the data table to keep only the rows where SNP_id is in SNP_id_vector
filtered_AalbF2_ref <- filtered_AalbF2_ref_F[SNP_id %in% AalbF2_SNPs]
filtered_AalbF2_alt <- filtered_AalbF2_alt_F[SNP_id %in% AalbF2_SNPs]
```

Now we can compare the SNP positions

```
# Merge the data tables based on the "SNP_id" column
compare_pos <- merge(filtered_AalbF2_ref, filtered_AalbF2_alt, by = "SNP_id", suffixes = c("_ref", "_alt"))

# Create a new column to check if "Position" matches in both data tables
compare_pos[, Position_match := Position_ref == Position_alt]

# We can keep only the rows with "TRUE" on Position_match
matched_SNPs <- compare_pos[Position_match == TRUE]

# We can create a vetor with the SNP ids
SNP_id_vector <- matched_SNPs$SNP_id

# How many SNPs we have
length(SNP_id_vector)
```

```
## [1] 108812
```

All probe sequences with reference and alternative alleles mapped to
the same region.

Now we can use either filtered\_AalbF2\_ref or filtered\_AalbF2\_alt to
get the position of the SNPs in the AalbF2

```
# Columns to select
AalbF2_SNP_pos <- filtered_AalbF2_ref[, .(SNP_id, Ref_Name, Position)]

# Rename the "Ref_Name" column to "Scaffold"
setnames(AalbF2_SNP_pos, "Ref_Name", "Scaffold")

# Check the first few rows of the new data table
print(head(AalbF2_SNP_pos))
```

```
##          SNP_id     Scaffold Position
## 1: AX-582945972 NW_021837045    11115
## 2: AX-582943848 NW_021837045    22632
## 3: AX-582943896 NW_021837045   133778
## 4: AX-582943915 NW_021837045   161360
## 5: AX-582943937 NW_021837045   197704
## 6: AX-582946090 NW_021837045   214418
```

Now we can check the file7.bim that we created earlier using a
chromosomal scale for AalbF3

```
head output/populations/file7.bim
```

```
## 1    AX-583033342    0   315059  C   G
## 1    AX-583033370    0   330057  G   A
## 1    AX-583035194    0   330265  A   G
## 1    AX-583033387    0   331288  C   T
## 1    AX-583035257    0   442875  T   C
## 1    AX-583035355    0   540754  G   C
## 1    AX-583033631    0   713019  T   A
## 1    AX-583034838    0   1138155 T   C
## 1    AX-583034951    0   1155151 T   C
## 1    AX-583034959    0   1155381 C   A
```

We need to import the .bim file and merge it with the
AalbF2\_SNP\_pos

```
# load the function that we saved earlier
source(
  here(
    "scripts", "analysis", "import_bim.R"
  ),
  local = knitr::knit_global()
)
# import the file
file7 <- import_bim(
  here(
    "output", "populations", "file7.bim"
  )
)

# Convert to data table
setDT(file7)

# Rename columns
setnames(file7, "SNP", "SNP_id")
setnames(file7, "Scaffold", "Scaffold_AalbF3")
setnames(file7, "Position", "Position_AalbF3")

head(file7)
```

```
##    Scaffold_AalbF3       SNP_id Cm Position_AalbF3 Allele1 Allele2
## 1:               1 AX-583033342  0          315059       C       G
## 2:               1 AX-583033370  0          330057       G       A
## 3:               1 AX-583035194  0          330265       A       G
## 4:               1 AX-583033387  0          331288       C       T
## 5:               1 AX-583035257  0          442875       T       C
## 6:               1 AX-583035355  0          540754       G       C
```

Merge the data

```
# Merge the data tables based on the "SNP_id" column
merged_data <- merge(AalbF2_SNP_pos, file7, by = "SNP_id")
head(merged_data)
```

```
##          SNP_id     Scaffold Position Scaffold_AalbF3 Cm Position_AalbF3
## 1: AX-579436149 NW_021837378 32461123               2  0       359888898
## 2: AX-579436196 NW_021837378 32460413               2  0       359889608
## 3: AX-579436243 NW_021837378 32459083               2  0       359890938
## 4: AX-579436308 NW_021837378 32455721               2  0       359894300
## 5: AX-579436317 NW_021837378 32455487               2  0       359894534
## 6: AX-579436508 NW_021837378 32450497               2  0       359899524
##    Allele1 Allele2
## 1:       A       C
## 2:       C       T
## 3:       A       G
## 4:       A       C
## 5:       G       T
## 6:       A       G
```

```
# Select the columns we need for bim file
merged_data <- 
  merged_data |>
  dplyr::select(
    Scaffold, SNP_id, Cm, Position, Allele1, Allele2
  )
# check it
head(merged_data)
```

```
##        Scaffold       SNP_id Cm Position Allele1 Allele2
## 1: NW_021837378 AX-579436149  0 32461123       A       C
## 2: NW_021837378 AX-579436196  0 32460413       C       T
## 3: NW_021837378 AX-579436243  0 32459083       A       G
## 4: NW_021837378 AX-579436308  0 32455721       A       C
## 5: NW_021837378 AX-579436317  0 32455487       G       T
## 6: NW_021837378 AX-579436508  0 32450497       A       G
```

We can save the SNP ids to file and extract it from file 7

```
write.table(
  merged_data$SNP_id,
  file      = here(
    "output", "populations", "AalbF2_SNPs.txt"
  ),
  sep       = "\t",
  row.names = FALSE,
  col.names = FALSE,
  quote     = FALSE
)
```

Now we can create a new file

```
plink2 \
--bfile output/populations/file7 \
--make-bed \
--extract output/populations/AalbF2_SNPs.txt \
--allow-extra-chr \
--out output/populations/file8 \
--silent;

grep "variants" output/populations/file8.log
```

```
## 82731 variants loaded from output/populations/file7.bim.
## --extract: 44697 variants remaining.
## 44697 variants remaining after main filters.
```

Import file8.bim

```
# Import the file with explicit column names
file8 <- fread(
  here("output", "populations", "file8.bim"),
  col.names = c("Chr_AalbF3", "SNP_id", "Cm", "Position_AalbF3", "Allele1", "Allele2")
)

head(file8)
```

```
##    Chr_AalbF3       SNP_id Cm Position_AalbF3 Allele1 Allele2
## 1:          1 AX-583033631  0          713019       T       A
## 2:          1 AX-583034838  0         1138155       T       C
## 3:          1 AX-583037044  0         1277398       G       A
## 4:          1 AX-583035342  0         1278179       T       C
## 5:          1 AX-583037429  0         1310198       T       C
## 6:          1 AX-583037492  0         1310753       T       G
```

We have to merge it again

```
# Merge the data tables based on the "SNP_id" column
merged_data2 <- merge(AalbF2_SNP_pos, file8, by = "SNP_id")
head(merged_data2)
```

```
##          SNP_id     Scaffold Position Chr_AalbF3 Cm Position_AalbF3 Allele1
## 1: AX-579436149 NW_021837378 32461123          2  0       359888898       A
## 2: AX-579436196 NW_021837378 32460413          2  0       359889608       C
## 3: AX-579436243 NW_021837378 32459083          2  0       359890938       A
## 4: AX-579436308 NW_021837378 32455721          2  0       359894300       A
## 5: AX-579436317 NW_021837378 32455487          2  0       359894534       G
## 6: AX-579436508 NW_021837378 32450497          2  0       359899524       A
##    Allele2
## 1:       C
## 2:       T
## 3:       G
## 4:       C
## 5:       T
## 6:       G
```

```
# Select the columns we need for the bim file
merged_data2 <- 
  merged_data2 |>
  dplyr::select(
    Scaffold, SNP_id, Cm, Position, Allele1, Allele2
  )
# check it
head(merged_data2)
```

```
##        Scaffold       SNP_id Cm Position Allele1 Allele2
## 1: NW_021837378 AX-579436149  0 32461123       A       C
## 2: NW_021837378 AX-579436196  0 32460413       C       T
## 3: NW_021837378 AX-579436243  0 32459083       A       G
## 4: NW_021837378 AX-579436308  0 32455721       A       C
## 5: NW_021837378 AX-579436317  0 32455487       G       T
## 6: NW_021837378 AX-579436508  0 32450497       A       G
```

We need to set the SNP order to match the file 8 because of the
encoding of the files

```
# Merge the data and keep the order based on SNP_id in file8
merged_data_ordered <- merge(file8, merged_data2, by = "SNP_id", sort = FALSE)

# Select the columns we need for bim file
merged_data_ordered <- 
  merged_data_ordered |>
  dplyr::select(
    Scaffold, SNP_id, Cm.x, Position, Allele1.x, Allele2.x
  )

# Show the first few rows of the merged data
head(merged_data_ordered)
```

```
##        Scaffold       SNP_id Cm.x Position Allele1.x Allele2.x
## 1: NW_021838798 AX-583033631    0 73277244         T         A
## 2: NW_021838798 AX-583034838    0 72852108         T         C
## 3: NW_021838798 AX-583037044    0 72712865         G         A
## 4: NW_021838798 AX-583035342    0 72712084         T         C
## 5: NW_021838798 AX-583037429    0 72680065         T         C
## 6: NW_021838798 AX-583037492    0 72679510         T         G
```

Check if the order of the SNPs match the file8.bim

```
head output/populations/file8.bim
```

```
## 1    AX-583033631    0   713019  T   A
## 1    AX-583034838    0   1138155 T   C
## 1    AX-583037044    0   1277398 G   A
## 1    AX-583035342    0   1278179 T   C
## 1    AX-583037429    0   1310198 T   C
## 1    AX-583037492    0   1310753 T   G
## 1    AX-583037870    0   1346607 A   G
## 1    AX-583037899    0   1346850 G   A
## 1    AX-583036374    0   1353955 T   C
## 1    AX-583036635    0   1359862 C   T
```

Save the new .bim file

```
write.table(
  merged_data_ordered,
  file      = here(
    "output", "populations", "file8B.bim"
  ),
  sep       = "\t",
  row.names = FALSE,
  col.names = FALSE,
  quote     = FALSE
)
```

Rename the .bim files

```
# change the name of the first .bim file, for example, append _backup.bim, and then replace the original file
mv output/populations/file8.bim output/populations/file8_backup.bim;
# than change the new bim we create to the original name (do it only once, otherwise it will mess up)
mv output/populations/file8B.bim output/populations/file8.bim
```

Create a new bed file with Plink2 to see if it works. For example, to
see if the variants are in the right order. Plink2 will give us a
warning.

```
plink \
--keep-allele-order \
--bfile output/populations/file8 \
--make-bed \
--allow-extra-chr \
--out output/populations/file9;
```

```
## PLINK v1.90b6.26 64-bit (2 Apr 2022)           www.cog-genomics.org/plink/1.9/
## (C) 2005-2022 Shaun Purcell, Christopher Chang   GNU General Public License v3
## Logging to output/populations/file9.log.
## Options in effect:
##   --allow-extra-chr
##   --bfile output/populations/file8
##   --keep-allele-order
##   --make-bed
##   --out output/populations/file9
## 
## 32768 MB RAM detected; reserving 16384 MB for main workspace.
## 44697 variants loaded from .bim file.
## 237 people (67 males, 30 females, 140 ambiguous) loaded from .fam.
## Ambiguous sex IDs written to output/populations/file9.nosex .
## Using 1 thread (no multithreaded calculations invoked).
## Before main variant filters, 237 founders and 0 nonfounders present.
## Calculating allele frequencies... 0%1%2%3%4%5%6%7%8%9%10%11%12%13%14%15%16%17%18%19%20%21%22%23%24%25%26%27%28%29%30%31%32%33%34%35%36%37%38%39%40%41%42%43%44%45%46%47%48%49%50%51%52%53%54%55%56%57%58%59%60%61%62%63%64%65%66%67%68%69%70%71%72%73%74%75%76%77%78%79%80%81%82%83%84%85%86%87%88%89%90%91%92%93%94%95%96%97%98%99% done.
## Total genotyping rate is 0.972329.
## 44697 variants and 237 people pass filters and QC.
## Note: No phenotypes present.
## --make-bed to output/populations/file9.bed + output/populations/file9.bim +
## output/populations/file9.fam ... 0%1%2%3%4%5%6%7%8%9%10%11%12%13%14%15%16%17%18%19%20%21%22%23%24%25%26%27%28%29%30%31%32%33%34%35%36%37%38%39%40%41%42%43%44%45%46%47%48%49%50%51%52%53%54%55%56%57%58%59%60%61%62%63%64%65%66%67%68%69%70%71%72%73%74%75%76%77%78%79%80%81%82%83%84%85%86%87%88%89%90%91%92%93%94%95%96%97%98%99%done.
```

Check the new file9.bim

```
head output/populations/file9.bim
```

```
## 1    AX-583033631    0   713019  T   A
## 1    AX-583034838    0   1138155 T   C
## 1    AX-583037044    0   1277398 G   A
## 1    AX-583035342    0   1278179 T   C
## 1    AX-583037429    0   1310198 T   C
## 1    AX-583037492    0   1310753 T   G
## 1    AX-583037870    0   1346607 A   G
## 1    AX-583037899    0   1346850 G   A
## 1    AX-583036374    0   1353955 T   C
## 1    AX-583036635    0   1359862 C   T
```

```
head(AalbF2_SNP_pos)
```

```
##          SNP_id     Scaffold Position
## 1: AX-582945972 NW_021837045    11115
## 2: AX-582943848 NW_021837045    22632
## 3: AX-582943896 NW_021837045   133778
## 4: AX-582943915 NW_021837045   161360
## 5: AX-582943937 NW_021837045   197704
## 6: AX-582946090 NW_021837045   214418
```

Check a few SNPs to see if they match

```
merged_data_ordered |>
  dplyr::filter(
    SNP_id == "AX-583787677"
  )
```

```
##        Scaffold       SNP_id Cm.x Position Allele1.x Allele2.x
## 1: NW_021838798 AX-583787677    0  1587755         A         G
```

All looks good.

Please check the manual and manuscript of Admixture. We do not need
all variants to run the analysis: we opted to reduce the data set, and
use cross validation and bootstrapping to find the optimal number of
ancestral populations (k). We have a maximum wall time in our clusters
of 5 days. Using the entire data set with cross validation and
bootstrapping would require longer time for high k values. Check the R
Markdown file 02.Admixture\_analysis.Rmd to see how we set up the
analysis. From the Admixture manual page 6:

```
As a rule of thumb, we have found that 10,000 markers suffice to perform GWAS correction for
continentally separated populations (for example, African, Asian, and European populations FST >
.05) while more like 100,000 markers are necessary when the populations are within a continent
(Europe, for instance, FST < 0.01).
```

# 6. Plot SNP density

After quality control with approximately 60k SNPs

```
# load the function that we saved earlier
source(
  here(
    "scripts", "analysis", "import_bim.R"
  ),
  local = knitr::knit_global()
)
# import the file
snp_den_qc <- import_bim(
  here(
    "output", "populations", "file7.bim"
  )
)
```

Make plot of the SNP density

```
#   ____________________________________________________________________________
#   plot SNP density after QC                                               ####
snp_den_qc |>
  rename(
    Chromosome = 1
  ) |>
  mutate(
    Position           = as.numeric(
      Position
    )
  ) |>
  ggplot(
    aes(
      x                = Position
    ),
    label              = sprintf(
      "%0.2f",
      round(
        a,
        digits         = 0
      )
    )
  ) +
  geom_histogram(
    aes(
      y                = after_stat(
        count
      )
    ),
    binwidth           = 1e6
  ) +
  facet_wrap(
    vars(
      Chromosome
    ),
    scales             = "free_x"
  ) +
  labs(
    title              = "SNP Density after QC",
    x                  = expression(
      "Position in the genome (Mb)"
    ),
    y                  = expression(
      "Number of SNPs"
    )
  ) +
  scale_x_continuous(
    labels             = function(x) {
      format(
        x / 1e6,
        big.mark       = ",", 
        scientific     = FALSE
      )
    }
  ) +
  geom_density(
  aes(
    y = 1e6 * after_stat(count)
  ),
  color = "red",
  linewidth = .75,
  alpha = .4,
  fill = "pink"
  ) +
  hrbrthemes::theme_ipsum(
    base_family        = "Roboto Condensed",
    axis_text_size     = 12,
    axis_title_size    = 14,
    plot_margin        = margin(
      10, 10, 10, 10
    ),
    grid               = TRUE,
    grid_col           = "#fabbe2"
  ) +
  theme(
    panel.grid.major   = element_line(
      linetype         = "dashed",
      linewidth        = 0.2
    ),
    panel.grid.minor   = element_line(
      linetype         = "dashed",
      linewidth        = 0.2
    ),
    panel.spacing      = unit(0.5, "lines"),
    strip.text         = element_text(
      face             = "bold", hjust = .5
    ),
    strip.background.x = element_rect(
      color            = "gray"
    )
  )
```

```
#   ____________________________________________________________________________
#   save the density plot                                            ####
ggsave(
  here(
    "output", "populations","figures", "snp_density_after_qc.pdf"
  ),
  width  = 10,
  height = 6,
  units  = "in"
)
```

SNPs per chromosome

```
# we can use dplyr "count" to get the number of SNPs for each chromosome
# lets get the data we need
snps_per_chrm <- 
  snp_den_qc |>
  count(
    Scaffold) |>
  rename(
    Chromosome = 1,
    "SNPs (N) " = 2
  )

# Create the flextable
ft <- flextable::flextable(snps_per_chrm)

# Apply zebra theme
ft <- flextable::theme_zebra(ft)

# Add a caption to the table
ft <- flextable::add_header_lines(ft, "SNPs per chromosome after quality control")
ft
```

| SNPs per chromosome after quality control | |
| --- | --- |
| Chromosome | SNPs (N) |
| 1 | 18,602 |
| 2 | 34,636 |
| 3 | 29,493 |

We can get the mean number of SNPs per chromosome or the entire
genome

```
# we first use dplyr cut_width to get the number of SNPs per 1Mb window
albo_den <- 
  snp_den_qc |>
  dplyr::select(
    Scaffold, Position
  ) |>
  group_by(
    Scaffold,
    windows               = cut_width(
      Position,
      width               = 1e6,
      boundary            = 0
    )
  ) |>
  summarise(
    n                     = n(),
    .groups               = "keep"
  ) |>
  group_by(
    Scaffold
  ) |>
  summarise(
    mean                  = mean(n),
    n                     = n(),
    .groups               = "keep"
  ) |>
  rename(
    Chromosome            = 1,
    "SNPs per 1Mb window" = 2,
    "Number of windows"   = 3
  )
#
# check the results
snp_table <-
  flextable(
    albo_den
  )
snp_table <- colformat_double(
  x        = snp_table,
  big.mark = ",",
  digits   = 2,
  na_str   = "N/A"
)
snp_table
```

| Chromosome | SNPs per 1Mb window | Number of windows |
| --- | --- | --- |
| 1 | 50.55 | 368 |
| 2 | 59.92 | 578 |
| 3 | 60.56 | 487 |

Merge objects

```
# we can merge the two data sets we created above into one table
after_qc <-
  snps_per_chrm |>
  left_join(
    albo_den,
    by = "Chromosome"
  )
snp_table2 <- flextable(
  after_qc)
snp_table2 <- colformat_double(
  x        = snp_table2,
  big.mark = ",",
  digits   = 2, 
  na_str   = "N/A"
  )
snp_table2
```

| Chromosome | SNPs (N) | SNPs per 1Mb window | Number of windows |
| --- | --- | --- | --- |
| 1 | 18,602 | 50.55 | 368 |
| 2 | 34,636 | 59.92 | 578 |
| 3 | 29,493 | 60.56 | 487 |

# 7. LD pruning using the chromosomal scale

```
# we set a window of variants of 5 and move the window 1 variant per time, removing 1 of the variants with lowest MAF from a pair above the threshold of r^2 > 0.1
# the mean distance is 203kb across the tested populations. Try --indep-pairwise 200kb 1 0.1
plink2 \
--allow-extra-chr \
--bfile output/populations/file10 \
--indep-pairwise 5 1 0.1 \
--out output/populations/indepSNP_chr \
--silent;
grep 'pairwise\|variants\|samples' output/populations/indepSNP_chr.log
```

Lets do the scaffold again

```
# we set a window of variants of 5 and move the window 1 variant each time, removing 1 of the variants with lowest MAF from a pair above the threshold of r^2 > 0.1
# the mean distance is 203kb across the tested populations. We used --indep-pairwise 5 1 0.1 before. We can use the same values from the mean half distance max r2
plink2 \
--allow-extra-chr \
--bfile output/populations/file11 \
--indep-pairwise 5 1 0.1 \
--out output/populations/indepSNP_scaffolds \
--silent;
grep 'pairwise\|variants\|samples' output/populations/indepSNP_scaffolds.log
```

Now we can compare the two sets of SNPs using scaffolds or
chromosomal scale

Create Venn diagram of SNPs removed due to LD

```
# Read in the two files as vectors
prunout_chr <-
  read_delim(
    here(
      "output", "populations", "indepSNP_chr.prune.out"
    ),
    delim = " ",
    col_names = FALSE,
    show_col_types = FALSE
  )

prunout_scaffolds <-
  read_delim(
    here(
      "output", "populations", "indepSNP_scaffolds.prune.out"
    ),
    delim = " ",
    col_names = FALSE,
    show_col_types = FALSE
  )

# Convert the list to vector
prunout_scaffolds <- unlist(prunout_scaffolds)
prunout_chr <- unlist(prunout_chr)


# Calculate shared values
prunout <- intersect(prunout_chr, prunout_scaffolds)

# Create Venn diagram
venn_data1 <- list(
  "Chromosomal" = prunout_chr,
  "Scaffolds" = prunout_scaffolds
)

# create plot
venn_plot1 <- ggvenn(venn_data1, fill_color = c("steelblue", "darkorange"), show_percentage = TRUE)

# Add a title
venn_plot1 <- venn_plot1 +
  ggtitle("Comparison of genomic scales for linked SNPs") +
  theme(plot.title = element_text(hjust = .5))

# Display the Venn diagram
print(venn_plot1)
```

```
# Save Venn diagram to PDF
output_path <- here("output", "populations", "figures", "SNPs_linked_comparison.pdf")
ggsave(output_path, venn_plot1, height = 5, width = 5, dpi = 300)
```

Create a venn diagram of SNPs kept

```
# Read in the two files as vectors
prunin_chr <-
  read_delim(
    here(
      "output", "populations", "indepSNP_chr.prune.in"
    ),
    delim = " ",
    col_names = FALSE,
    show_col_types = FALSE
  )

prunin_scaffolds <-
  read_delim(
    here(
      "output", "populations", "indepSNP_scaffolds.prune.in"
    ),
    delim = " ",
    col_names = FALSE,
    show_col_types = FALSE
  )

# Convert the list to vector
prunin_chr <- unlist(prunin_chr)
prunin_scaffolds <- unlist(prunin_scaffolds)


# Calculate shared values
prunout <- intersect(prunin_chr, prunin_scaffolds)

# Create Venn diagram
venn_data2 <- list(
  "Chromosomal" = prunin_chr,
  "Scaffolds" = prunin_scaffolds
)

# create plot
venn_plot2 <- ggvenn(venn_data2, fill_color = c("steelblue", "darkorange"), show_percentage = TRUE)

# Add a title
venn_plot2 <- venn_plot2 +
  ggtitle("Comparison of genomic scales for unlinked SNPs") +
  theme(plot.title = element_text(hjust = .5))

# Display the Venn diagram
print(venn_plot2)
```

```
# Save Venn diagram to PDF
output_path <- here("output", "populations", "figures", "SNPs_unlinked_comparison.pdf")
ggsave(output_path, venn_plot2, height = 5, width = 5, dpi = 300)
```

The pruning is almost identical using the chromosomal or scaffold
scale. We will keep the previous pruning using 5 1 0.1 which resulted in
approximately 59k SNPs.

# 8. Intergenic unlinked SNPs

We can also create a file to run the ancestry analyses using only
intergenic SNPs

We can use the intergenic SNPs as “neutral” SNPs’

```
plink2 \
--allow-extra-chr \
--bfile output/populations/file7 \
--geno 0.2 \
--maf 0.1 \
--make-bed \
--extract output/SnpEff/intergenic_SNPs.txt \
--out output/populations/intergenic \
--silent;
grep "samples\|variants" output/populations/intergenic.log
```

```
## 237 samples (30 females, 67 males, 140 ambiguous; 237 founders) loaded from
## 82731 variants loaded from output/populations/file7.bim.
## --extract: 11652 variants remaining.
## --geno: 0 variants removed due to missing genotype data.
## 35 variants removed due to allele frequency threshold(s)
## 11617 variants remaining after main filters.
```

Perform LD prunning

```
plink2 \
--allow-extra-chr \
--bfile output/populations/intergenic \
--indep-pairwise 5 1 0.1 \
--out output/populations/inter \
--silent;
grep "samples\|variants" output/populations/inter.log
```

```
## 237 samples (30 females, 67 males, 140 ambiguous; 237 founders) loaded from
## 11617 variants loaded from output/populations/intergenic.bim.
## --indep-pairwise (3 compute threads): 777/11617 variants removed.
```

Keep unlinked SNPs

```
plink2 \
--allow-extra-chr \
--bfile output/populations/intergenic \
--geno 0.2 \
--maf 0.1 \
--export vcf \
--make-bed \
--extract output/populations/inter.prune.in \
--out output/populations/intergenic2 \
--silent;
grep "samples\|variants" output/populations/intergenic2.log
```

```
## 237 samples (30 females, 67 males, 140 ambiguous; 237 founders) loaded from
## 11617 variants loaded from output/populations/intergenic.bim.
## --extract: 10840 variants remaining.
## --geno: 0 variants removed due to missing genotype data.
## 0 variants removed due to allele frequency threshold(s)
## 10840 variants remaining after main filters.
```

OutFLANK analysis with quasi-independent set of SNPs

We need to import the data

```
library(OutFLANK)
library(vcfR)
```

```
# Read your vcf file
vcf <- read.vcfR(here("output","populations","intergenic2.vcf"))
```

```
## Scanning file to determine attributes.
## File attributes:
##   meta lines: 8
##   header_line: 9
##   variant count: 10840
##   column count: 246
## 
Meta line 8 read in.
## All meta lines processed.
## gt matrix initialized.
## Character matrix gt created.
##   Character matrix gt rows: 10840
##   Character matrix gt cols: 246
##   skip: 0
##   nrows: 10840
##   row_num: 0
## 
Processed variant 1000
Processed variant 2000
Processed variant 3000
Processed variant 4000
Processed variant 5000
Processed variant 6000
Processed variant 7000
Processed variant 8000
Processed variant 9000
Processed variant 10000
Processed variant: 10840
## All variants processed
```

```
# Extract genotypes
geno <- vcfR::extract.gt(vcf)

# Extract locus names (positions)
locusNames <- vcfR::getPOS(vcf)

# Samples
sampleNames <- colnames(geno)

# Extract population names by splitting the sample names
# If your sample names contain population information.
popNames <- sapply(strsplit(sampleNames, "_"), "[", 1)

# Initialize new matrix for transformed genotypes
G <- matrix(NA, nrow = nrow(geno), ncol = ncol(geno))

# Recode genotypes
G[geno %in% c("0/0", "0|0")] <- 0
G[geno %in% c("0/1", "1/0", "1|0", "0|1")] <- 1
G[geno %in% c("1/1", "1|1")] <- 2

# Replace NA with 9 for missing data
G <- replace(G, is.na(G), 9)

# Check the counts of each genotype
table(as.vector(G))
```

```
## 
##       0       1       2       9 
## 1299261  641241  550218   78360
```

```
# Extract SNP names
snpNames <- vcfR::getID(vcf)

# Calculate FST with SNP names
my_fst <- MakeDiploidFSTMat(t(G), locusNames = snpNames, popNames = popNames)
```

```
## Calculating FSTs, may take a few minutes...
## [1] "10000 done of 10840"
```

```
head(my_fst)
```

```
##      LocusName        He        FST          T1        T2  FSTNoCorr   T1NoCorr
## 1 AX-583034838 0.3574763 0.48417163 0.088444174 0.1826711 0.51657843 0.09438423
## 2 AX-583037866 0.4825236 0.29768562 0.072871445 0.2447933 0.34827829 0.08528626
## 3 AX-583038083 0.2187500 0.21973808 0.024304449 0.1106065 0.27279637 0.03018364
## 4 AX-583038481 0.3402647 0.03578489 0.006107068 0.1706605 0.08055462 0.01375156
## 5 AX-583044095 0.3597194 0.07029925 0.012725141 0.1810139 0.16476779 0.02984661
## 6 AX-583044062 0.4993328 0.14815959 0.037320744 0.2518956 0.21980594 0.05539633
##    T2NoCorr meanAlleleFreq
## 1 0.1827104      0.7669492
## 2 0.2448796      0.5934783
## 3 0.1106453      0.8750000
## 4 0.1707110      0.7826087
## 5 0.1811435      0.7648402
## 6 0.2520238      0.4817352
```

We can select the SNPs with low FST to use as neutral SNPs

```
my_fst |>
  filter(FST<=0.20) |>
  dplyr::select(LocusName) -> neutral_snps

length(neutral_snps$LocusName)
```

```
## [1] 9483
```

```
write.table(
  neutral_snps$LocusName,
  file = here("output", "populations", "neutral_SNPs.txt"),
  row.names = FALSE,
  quote = FALSE,
  col.names = FALSE,
  sep = "\n"
)
```

Out of the 1245 1220 have FST values < 0.2, which is in contrast
with the SNPs on other genomic regions. We could filter the 25 SNPs with
FST higher than 0.2 but lets try without removing them first. The code
below is only if you want to remove them.

Now we can repeat the first step using the unliked SNPs with low FST
values Create a vcf using the unlinked loci with no missing data

```
plink2 \
--allow-extra-chr \
--bfile output/populations/intergenic \
--export vcf \
--make-bed \
--extract output/populations/neutral_SNPs.txt \
--out output/populations/neutral \
--silent;
grep "samples\|variants" output/populations/neutral.log
```

```
## 237 samples (30 females, 67 males, 140 ambiguous; 237 founders) loaded from
## 11617 variants loaded from output/populations/intergenic.bim.
## --extract: 9483 variants remaining.
## 9483 variants remaining after main filters.
```

Move to dir

```
cp output/populations/neutral.bed output/populations/snps_sets;
cp output/populations/neutral.bim output/populations/snps_sets;
cp output/populations/neutral.fam output/populations/snps_sets;
cp output/populations/neutral.log output/populations/snps_sets;
cp output/populations/neutral.vcf output/populations/snps_sets;
ls output/populations/snps_sets
```

```
## neutral.bed
## neutral.bim
## neutral.fam
## neutral.geno
## neutral.lfmm
## neutral.log
## neutral.pca
## neutral.removed
## neutral.snmf
## neutral.snmfProject
## neutral.vcf
## neutral.vcfsnp
## r2_0.01.bed
## r2_0.01.bim
## r2_0.01.fam
## r2_0.01.geno
## r2_0.01.lfmm
## r2_0.01.log
## r2_0.01.nosex
## r2_0.01.pca
## r2_0.01.removed
## r2_0.01.snmf
## r2_0.01.snmfProject
## r2_0.01.vcf
## r2_0.01.vcfsnp
## r2_0.1.bed
## r2_0.1.bim
## r2_0.1.fam
## r2_0.1.geno
## r2_0.1.lfmm
## r2_0.1.log
## r2_0.1.nosex
## r2_0.1.pca
## r2_0.1.removed
## r2_0.1.snmf
## r2_0.1.snmfProject
## r2_0.1.vcf
## r2_0.1.vcfsnp
```

Import the data.

```
# Read your vcf file
vcf <- read.vcfR(here("output","local_adaptation","neutral.vcf"))
```

```
## Scanning file to determine attributes.
## File attributes:
##   meta lines: 8
##   header_line: 9
##   variant count: 1220
##   column count: 128
## 
Meta line 8 read in.
## All meta lines processed.
## gt matrix initialized.
## Character matrix gt created.
##   Character matrix gt rows: 1220
##   Character matrix gt cols: 128
##   skip: 0
##   nrows: 1220
##   row_num: 0
## 
Processed variant 1000
Processed variant: 1220
## All variants processed
```

```
# Extract genotypes
geno <- vcfR::extract.gt(vcf)

# Extract locus names (positions)
locusNames <- vcfR::getPOS(vcf)

# Samples
sampleNames <- colnames(geno)

# Extract population names by splitting the sample names
# If your sample names contain population information.
popNames <- sapply(strsplit(sampleNames, "_"), "[", 1)

# Initialize new matrix for transformed genotypes
G <- matrix(NA, nrow = nrow(geno), ncol = ncol(geno))

# Recode genotypes
G[geno %in% c("0/0", "0|0")] <- 0
G[geno %in% c("0/1", "1/0", "1|0", "0|1")] <- 1
G[geno %in% c("1/1", "1|1")] <- 2

# Check the counts of each genotype
table(as.vector(G))
```

```
## 
##     0     1     2 
## 64251 49967 30962
```

```
# Extract SNP names
snpNames <- vcfR::getID(vcf)

# Calculate FST with SNP names
my_fst <- MakeDiploidFSTMat(t(G), locusNames = snpNames, popNames = popNames)
```

```
## Calculating FSTs, may take a few minutes...
```

```
head(my_fst)
```

```
##      LocusName        He         FST          T1        T2  FSTNoCorr
## 1 AX-583034838 0.4008192 0.164885011 0.033709186 0.2044406 0.19706911
## 2 AX-583049532 0.3169621 0.019158983 0.003051440 0.1592694 0.04948776
## 3 AX-583056767 0.3415013 0.047884271 0.008249681 0.1722837 0.08895758
## 4 AX-583061613 0.3415013 0.009390761 0.001611444 0.1715989 0.04942826
## 5 AX-583061163 0.3642751 0.074842092 0.013795884 0.1843332 0.11906870
## 6 AX-583061282 0.2327519 0.138246271 0.016434909 0.1188814 0.21066659
##      T1NoCorr  T2NoCorr meanAlleleFreq
## 1 0.040289013 0.2044410      0.2773109
## 2 0.007881903 0.1592697      0.8025210
## 3 0.015325988 0.1722842      0.2184874
## 4 0.008481858 0.1715994      0.2184874
## 5 0.021948386 0.1843338      0.7605042
## 6 0.025044465 0.1188820      0.1344538
```

Data prep: decide which SNPs to use for calibrating the null
distribution of Fst

```
# Run OutFLANK to estimate the neutral FST distribution
out_trim <- OutFLANK(FstDataFrame = my_fst, Hmin = 0.1, NumberOfSamples = 10, qthreshold = 0.05)
str(out_trim)
```

```
## List of 6
##  $ FSTbar               : num 0.055
##  $ FSTNoCorrbar         : num 0.0978
##  $ dfInferred           : num 8.67
##  $ numberLowFstOutliers : int 0
##  $ numberHighFstOutliers: int 0
##  $ results              :'data.frame':   1220 obs. of  15 variables:
##   ..$ LocusName       : chr [1:1220] "AX-583034838" "AX-583049532" "AX-583056767" "AX-583061613" ...
##   ..$ He              : num [1:1220] 0.401 0.317 0.342 0.342 0.364 ...
##   ..$ FST             : num [1:1220] 0.16489 0.01916 0.04788 0.00939 0.07484 ...
##   ..$ T1              : num [1:1220] 0.03371 0.00305 0.00825 0.00161 0.0138 ...
##   ..$ T2              : num [1:1220] 0.204 0.159 0.172 0.172 0.184 ...
##   ..$ FSTNoCorr       : num [1:1220] 0.1971 0.0495 0.089 0.0494 0.1191 ...
##   ..$ T1NoCorr        : num [1:1220] 0.04029 0.00788 0.01533 0.00848 0.02195 ...
##   ..$ T2NoCorr        : num [1:1220] 0.204 0.159 0.172 0.172 0.184 ...
##   ..$ meanAlleleFreq  : num [1:1220] 0.277 0.803 0.218 0.218 0.761 ...
##   ..$ indexOrder      : int [1:1220] 1 2 3 4 5 6 7 8 9 10 ...
##   ..$ GoodH           : chr [1:1220] "goodH" "goodH" "goodH" "goodH" ...
##   ..$ qvalues         : num [1:1220] 0.838 0.982 0.971 0.982 0.971 ...
##   ..$ pvalues         : num [1:1220] 0.0716 0.2688 0.9742 0.2679 0.5608 ...
##   ..$ pvaluesRightTail: num [1:1220] 0.0358 0.8656 0.5129 0.866 0.2804 ...
##   ..$ OutlierFlag     : logi [1:1220] FALSE FALSE FALSE FALSE FALSE FALSE ...
```

Check the fit and make sure it looks good, especially in the right
tail:

```
OutFLANKResultsPlotter(
  out_trim,
  withOutliers = TRUE,
  NoCorr = TRUE,
  Hmin = 0.1,
  binwidth = 0.001,
  Zoom = FALSE,
  RightZoomFraction = 0.05,
  titletext = NULL
)
```

```
## Zoom in on right tail
OutFLANKResultsPlotter(
  out_trim ,
  withOutliers = TRUE,
  NoCorr = TRUE,
  Hmin = 0.1,
  binwidth = 0.001,
  Zoom =
    TRUE,
  RightZoomFraction = 0.15,
  titletext = NULL
)
```

Also check the P-value histogram: Here, we plot the “right-tailed”
P-values, which means that outliers in the right tail of the FST
distribution will have a P-value near zero. Because we ran the algorithm
on a trimmed set of SNPs, this will remove some of the signal around
selected sites. So we expect this histogram to be flat and maybe have a
bump near 0 for selected sites. This histogram looks pretty good.

```
hist(out_trim$results$pvaluesRightTail)
```

# 9. Pruning r2 0.01

We can use the SNP set from neuro-admixture training. We will extract
it from file7

```
plink \
--keep-allele-order \
--bfile output/populations/file7 \
--make-bed \
--export vcf \
--out output/populations/snps_sets/r2_0.01 \
--extract output/populations/nadmix/train/train_r_0.01.snplist \
--silent
grep "variants\|samples" output/populations/snps_sets/r2_0.01.log
```

```
## 82731 variants loaded from .bim file.
## --extract: 21095 variants remaining.
## 21095 variants and 237 people pass filters and QC.
```

# 10. Pruning r2 0.1

We can use the SNP set from neuro-admixture training. We will extract
it from file7

```
# we use the same snps that we use with neuro admxiture
plink \
--keep-allele-order \
--bfile output/populations/file7 \
--make-bed \
--export vcf \
--out output/populations/snps_sets/r2_0.1 \
--extract output/populations/nadmix/train/train_r_0.1.snplist \
--silent
grep "variants\|samples" output/populations/snps_sets/r2_0.1.log
```

```
## 82731 variants loaded from .bim file.
## --extract: 58152 variants remaining.
## 58152 variants and 237 people pass filters and QC.
```
