# Supplementary material for: A genotyping array for the globally invasive vector mosquito, Aedes albopictus
Source: Parasit Vectors. 2024 Mar 4;17:106. doi: 10.1186/s13071-024-06158-z (PMC10910840; doi:10.1186/s13071-024-06158-z)
Supplement: Supplementary file 8 — Additional file 8. Linkage analysis with PopLDdecay. [file 13071_2024_6158_MOESM8_ESM.html]

Aedes albopictus SNP chip - Chip use: Linkage.


# Aedes albopictus SNP chip - Chip use: Linkage.

#### Luciano V Cosme

#### 2023-10-08

- 1. R libraries and software
- 2. Prepare the
  data
- 3. Create chromosomal scale
- 3. Chromosomal level LD
  estimates
  - 3.1 Chromosome 1
    - 3.1.1 run
      PopLDdecay
    - 3.1.2 Plot using PopLDdecay
    - 3.1.3 Plot
      using ggplot
  - 3.2 Chromosome 2
    - 3.1.1 run
      PopLDdecay
    - 3.1.2 Plot using PopLDdecay
    - 3.1.3
      Plot using ggplot
  - 3.3 Chromosome 3
    - 3.1.1 run
      PopLDdecay
    - 3.1.2 Plot using PopLDdecay
    - 3.1.3
      Plot using ggplot
- 4. Plot comparying
  chromosomes

## 1. R libraries and software

```
library(tidyverse)
library(here)
library(dplyr)
library(ggplot2)
library(colorout)
library(extrafont)
library(scales)
library(stringr)
library(flextable)
library(ggtext)
library(RColorBrewer)
library(ggrepel)
library(grid)
library(forcats)
library(officer)
library(ggstatsplot)
```

I will use the PopLDdecay for the estimates

https://github.com/BGI-shenzhen/PopLDdecay and the
manuscript DOI:10.1093/bioinformatics/bty875

Clean env and memory

```
# Remove all objects from the environment
rm(list = ls())

# Run the garbage collector to free up memory
gc()
```

```
##           used  (Mb) gc trigger  (Mb) limit (Mb) max used  (Mb)
## Ncells 2419409 129.3    4551360 243.1         NA  4551360 243.1
## Vcells 4078901  31.2    8388608  64.0      32768  6637402  50.7
```

## 2. Prepare the data

Mosquitoes removed due excess heterozygosity

```
tail -n +2 output/populations/het_fail_ind.txt
```

```
## KAT 9
## KAT 12
```

Mosquitoes removed duo to relatedness

```
head -n 10 output/populations/file7.king.cutoff.out.id
```

```
## #FID IID
## OKI  1010
## KAN  1327
```

Create a list of individuals to remove

```
echo 'KAT 9
KAT 12
OKI 1010
KAN 1327' > output/populations/linkage/remove.txt
```

Create file

```
plink2 \
--allow-extra-chr \
--remove output/populations/linkage/remove.txt \
--bfile output/populations/file3 \
--make-bed \
--out output/populations/linkage/ld1 \
--silent;
grep 'samples\|variants\|remaining' output/populations/linkage/ld1.log
```

```
## 239 samples (30 females, 69 males, 140 ambiguous; 239 founders) loaded from
## 104139 variants loaded from output/populations/file3.bim.
## --remove: 235 samples remaining.
## 235 samples (30 females, 65 males, 140 ambiguous; 235 founders) remaining after
```

```
awk '{print $1}' output/populations/linkage/ld1.fam | sort | uniq -c | awk '{print $2, $1}'
```

```
## BEN 12
## CAM 12
## CHA 12
## GEL 2
## HAI 12
## HAN 4
## HOC 7
## HUN 12
## INJ 11
## INW 4
## JAF 2
## KAC 6
## KAG 12
## KAN 11
## KAT 8
## KLP 4
## KUN 4
## LAM 9
## MAT 12
## OKI 11
## QNC 12
## SON 3
## SSK 12
## SUF 6
## SUU 6
## TAI 8
## UTS 12
## YUN 9
```

Create list of populations

```
awk '{print $1}' output/populations/linkage/ld1.fam | sort | uniq -c | awk '{print $2, $1}' | awk '$2 >= 6 {print}' | awk '{print $1}' > output/populations/linkage/pops_4ld.txt;
head -n100 output/populations/linkage/pops_4ld.txt
```

```
## BEN
## CAM
## CHA
## HAI
## HOC
## HUN
## INJ
## KAC
## KAG
## KAN
## KAT
## LAM
## MAT
## OKI
## QNC
## SSK
## SUF
## SUU
## TAI
## UTS
## YUN
```

## 3. Create chromosomal scale

Import the .bim file with the SNPs to create a new chromosomal
scale.

```
#   ____________________________________________________________________________
#   import the bim file with the SNP data                                   ####
snps <-
  read_delim(                    # to learn about the options use here, run ?read_delim on the console.
    here(
      "output", "populations", "linkage", "ld1.bim"
    ),                           # use library here to load it
    col_names      = FALSE,      # we don't have header in the input file
    show_col_types = FALSE,      # suppress message from read_delim
    col_types      = "ccidcc"    # set the class of each column
  )
#
# set column names
colnames(
  snps
) <-                             # to add a header in our tibble
  c(
    "Scaffold", "SNP", "Cm", "Position", "Allele1", "Allele2"
  )
#
# check the tibble
head(snps)
```

```
## # A tibble: 6 × 6
##   Scaffold SNP             Cm Position Allele1 Allele2
##   <chr>    <chr>        <int>    <dbl> <chr>   <chr>  
## 1 1.1      AX-583033226     0   161729 A       G      
## 2 1.1      AX-583033342     0   315059 C       G      
## 3 1.1      AX-583033370     0   330057 G       A      
## 4 1.1      AX-583035194     0   330265 A       G      
## 5 1.1      AX-583033387     0   331288 C       T      
## 6 1.1      AX-583035211     0   345197 C       T
```

Separate the tibbles into each chromosome

```
#   ____________________________________________________________________________
#   separate the SNP data per chromosome                                    ####
# chr1
chr1_snps <-
  snps |>
  filter(
    str_detect(
      Scaffold, "^1."
    )
  ) |> # here we get only Scaffold rows starting with 1
  as_tibble() # save as tibble
#
# chr2
chr2_snps <-
  snps |>
  filter(
    str_detect(
      Scaffold, "^2."
    )
  ) |>
  as_tibble()
#
# chr3
chr3_snps <-
  snps |>
  filter(
    str_detect(
      Scaffold, "^3."
    )
  ) |>
  as_tibble()
```

Import the file with sizes of each scaffold.

```
#   ____________________________________________________________________________
#   import the file with the scaffold sizes                                 ####
sizes <-
  read_delim(
    here(
      "data", "genome", "scaffold_sizes.txt"
    ),
    col_names      = FALSE,
    show_col_types = FALSE,
    col_types      = "cd"
  )
#
# set column names
colnames(
  sizes
) <- c(
  "Scaffold", "Size"
)
#   ____________________________________________________________________________
#   create new column with the chromosome number                            ####
sizes <- 
  sizes |>
  mutate(
    Chromosome = case_when( # we use mutate to create a new column called Chromosome
      startsWith(
        Scaffold, "1"
      ) ~ "1", # use startsWith to get Scaffold rows starting with 1 and output 1 on Chromosome column
      startsWith(
        Scaffold, "2"
      ) ~ "2",
      startsWith(
        Scaffold, "3"
      ) ~ "3"
    )
  ) |>
  arrange(
    Scaffold
  )                   # to sort the order of the scaffolds, fixing the problem we have with scaffold 1.86
# check it
head(sizes)
```

```
## # A tibble: 6 × 3
##   Scaffold     Size Chromosome
##   <chr>       <dbl> <chr>     
## 1 1.1        351198 1         
## 2 1.10     11939576 1         
## 3 1.100     3389100 1         
## 4 1.101      470438 1         
## 5 1.102     2525157 1         
## 6 1.103      150026 1
```

Create new scale. Get the scaffolds for each chromosome.

```
#   ____________________________________________________________________________
#   separate the scaffold sizes tibble per chromosome                       ####
# chr1
chr1_scaffolds <- 
  sizes |>
  filter(
    str_detect(
      Scaffold, "^1" # we use library stringr to get scaffolds starting with 1 (chromosome 1)
    )
  ) |> 
  as_tibble()
#
# chr2
chr2_scaffolds <-
  sizes |>
  filter(
    str_detect(
      Scaffold, "^2" # we use library stringr to get scaffolds starting with 2 (chromosome 2)
    )
  ) |> 
  as_tibble()
#
# # chr3
chr3_scaffolds <-
  sizes |>
  filter(
    str_detect(
      Scaffold, "^3" # we use library stringr to get scaffolds starting with 3 (chromosome 3)
    )
  ) |>
  as_tibble()
```

Create a scale for each chromosome.

```
#   ____________________________________________________________________________
#   create a new scale for each chromosome                                  ####
# chr1
chr1_scaffolds$overall_size_before_bp <-
  0                                                                        # we create a new column with zeros
for (i in 2:nrow(
  chr1_scaffolds
)
) {                                                                        # loop to start on second line
  chr1_scaffolds$overall_size_before_bp[i] <-                              # set position on the scale
    chr1_scaffolds$overall_size_before_bp[i - 1] + chr1_scaffolds$Size[i - # add the scaffold size and the location to get position on new scale
      1]
}
#
# chr2
chr2_scaffolds$overall_size_before_bp <- 0
for (i in 2:nrow(
  chr2_scaffolds
)
) {
  chr2_scaffolds$overall_size_before_bp[i] <-
    chr2_scaffolds$overall_size_before_bp[i - 1] + chr2_scaffolds$Size[i -
      1]
}
#
# chr3
chr3_scaffolds$overall_size_before_bp <- 0
for (i in 2:nrow(
  chr3_scaffolds
)
) {
  chr3_scaffolds$overall_size_before_bp[i] <-
    chr3_scaffolds$overall_size_before_bp[i - 1] + chr3_scaffolds$Size[i -
      1]
}
```

Merge the data frames scaffolds and SNPs.

```
#   ____________________________________________________________________________
#   merge the data sets using the tidyverse function left_join              ####
# chr1
chr1_scale <-
  chr1_snps |>          # create data frame for each chromosome, get chr1_snps
  left_join(            # use lef_join function to merge it with chr1_scaffolds
    chr1_scaffolds,
    by = "Scaffold"
  ) |>                  # set column to use for merging (Scaffold in this case)
  na.omit() |>          # remove NAs, we don't have SNPs in every scaffold
  mutate(
    midPos_fullseq = as.numeric(
      Position
    ) +                 # make new columns numeric
      as.numeric(
        overall_size_before_bp
      )
  )
#
# chr2
chr2_scale <-
  chr2_snps |>
  left_join(
    chr2_scaffolds,
    by = "Scaffold"
  ) |>
  na.omit() |>
  mutate(
    midPos_fullseq = as.numeric(
      Position
    ) +
      as.numeric(
        overall_size_before_bp
      )
  )
#
# chr3
chr3_scale <-
  chr3_snps |>
  left_join(
    chr3_scaffolds,
    by = "Scaffold"
  ) |>
  na.omit() |>
  mutate(
    midPos_fullseq = as.numeric(
      Position
    ) +
      as.numeric(
        overall_size_before_bp
      )
  )
```

Merge all chromosome scales.

```
#   ____________________________________________________________________________
#   merge the data sets, and select only the columns we need                ####
chroms <- rbind(
  chr1_scale, chr2_scale, chr3_scale
) |>
  dplyr::select(
    Chromosome, SNP, Cm, midPos_fullseq, Allele1, Allele2
  )
# check it
head(chroms)
```

```
## # A tibble: 6 × 6
##   Chromosome SNP             Cm midPos_fullseq Allele1 Allele2
##   <chr>      <chr>        <int>          <dbl> <chr>   <chr>  
## 1 1          AX-583033226     0         161729 A       G      
## 2 1          AX-583033342     0         315059 C       G      
## 3 1          AX-583033370     0         330057 G       A      
## 4 1          AX-583035194     0         330265 A       G      
## 5 1          AX-583033387     0         331288 C       T      
## 6 1          AX-583035211     0         345197 C       T
```

Save the new .bim file

```
#   ____________________________________________________________________________
#   save the new bim file with a new name, I added "B"                      ####
write.table(
  chroms,
  file      = here(
    "output", "populations", "linkage", "ld1B.bim"
  ),
  sep       = "\t",
  row.names = FALSE,
  col.names = FALSE,
  quote     = FALSE
)
```

Rename the .bim files

```
# change the name of the first .bim file, for example, append _backup.bim, and then replace the original file
mv output/populations/linkage/ld1.bim output/populations/linkage/ld1_backup.bim;
# than change the new bim we create to the original name (do it only once, otherwise it will mess up)
mv output/populations/linkage/ld1B.bim output/populations/linkage/ld1.bim
```

Create a new bed file with Plink2 to see if it works. For example, to
see if the variants are in the right order. Plink2 will give us a
warning.

```
plink2 \
--bfile output/populations/linkage/ld1 \
--make-bed \
--out output/populations/linkage/test01;
# then we remove the files 
rm output/populations/linkage/test01.*
```

```
## PLINK v2.00a3.3 64-bit (3 Jun 2022)            www.cog-genomics.org/plink/2.0/
## (C) 2005-2022 Shaun Purcell, Christopher Chang   GNU General Public License v3
## Logging to output/populations/linkage/test01.log.
## Options in effect:
##   --bfile output/populations/linkage/ld1
##   --make-bed
##   --out output/populations/linkage/test01
## 
## Start time: Sun Oct  8 14:53:48 2023
## 32768 MiB RAM detected; reserving 16384 MiB for main workspace.
## Using up to 12 threads (change this with --threads).
## 235 samples (30 females, 65 males, 140 ambiguous; 235 founders) loaded from
## output/populations/linkage/ld1.fam.
## 104139 variants loaded from output/populations/linkage/ld1.bim.
## Note: No phenotype data present.
## Writing output/populations/linkage/test01.fam ... done.
## Writing output/populations/linkage/test01.bim ... done.
## Writing output/populations/linkage/test01.bed ... 0%62%done.
## End time: Sun Oct  8 14:53:48 2023
```

No warnings from Plink2. Now, we can go ahead with our analysis.

## 3. Chromosomal level LD estimates

We will use MAF 0.005 for each chromosome (default from PopLDdecay)
Clean env and memory

```
# Remove all objects from the environment
rm(list = ls())

# Run the garbage collector to free up memory
gc()
```

```
##           used  (Mb) gc trigger  (Mb) limit (Mb) max used  (Mb)
## Ncells 2556968 136.6    4548762 243.0         NA  4548762 243.0
## Vcells 4432572  33.9   10825744  82.6      32768 10752723  82.1
```

We can estimate LD for each chromosome separated.

Import the .bim file with the SNPs to create a new chromosomal
scale.

```
#   ____________________________________________________________________________
#   import the bim file with the SNP data                                   ####
snps <-
  read_delim(                    # to learn about the options use here, run ?read_delim on the console.
    here(
      "output", "populations", "linkage", "ld1.bim"
    ),                           # use library here to load it
    col_names      = FALSE,      # we don't have header in the input file
    show_col_types = FALSE,      # suppress message from read_delim
    col_types      = "ccidcc"    # set the class of each column
  )
#
# set column names
colnames(
  snps
) <-                             # to add a header in our tibble
  c(
    "Chromosome", "SNP", "Cm", "Position", "Allele1", "Allele2"
  )
#
# check the tibble
head(snps)
```

```
## # A tibble: 6 × 6
##   Chromosome SNP             Cm Position Allele1 Allele2
##   <chr>      <chr>        <int>    <dbl> <chr>   <chr>  
## 1 1          AX-583033226     0   161729 A       G      
## 2 1          AX-583033342     0   315059 C       G      
## 3 1          AX-583033370     0   330057 G       A      
## 4 1          AX-583035194     0   330265 A       G      
## 5 1          AX-583033387     0   331288 C       T      
## 6 1          AX-583035211     0   345197 C       T
```

Separate the tibbles into each chromosome.

```
#   ____________________________________________________________________________
#   separate the SNP data per chromosome                                    ####
# chr1
chr1_snps <-
  snps |>
  filter(Chromosome == 1) |> # here we get only Chromosome rows starting with 1
  as_tibble() # save as tibble
#
# chr2
chr2_snps <-
  snps |>
  filter(Chromosome == 2) |>
  as_tibble()
#
# chr3
chr3_snps <-
  snps |>
  filter(Chromosome == 3) |>
  as_tibble()
```

We have objects with the SNPs for each chromosome

```
head(chr1_snps)
```

```
## # A tibble: 6 × 6
##   Chromosome SNP             Cm Position Allele1 Allele2
##   <chr>      <chr>        <int>    <dbl> <chr>   <chr>  
## 1 1          AX-583033226     0   161729 A       G      
## 2 1          AX-583033342     0   315059 C       G      
## 3 1          AX-583033370     0   330057 G       A      
## 4 1          AX-583035194     0   330265 A       G      
## 5 1          AX-583033387     0   331288 C       T      
## 6 1          AX-583035211     0   345197 C       T
```

Filter the data by chromosome

Chromosome 1

```
write.table(
  chr1_snps$SNP,
  file      = here(
    "output", "populations", "linkage", "chr1_ld_SNPs.txt"
  ),
  sep       = "\t",
  row.names = FALSE,
  col.names = FALSE,
  quote     = FALSE
)
```

Chromosome 2

```
write.table(
  chr2_snps$SNP,
  file      = here(
    "output", "populations", "linkage", "chr2_ld_SNPs.txt"
  ),
  sep       = "\t",
  row.names = FALSE,
  col.names = FALSE,
  quote     = FALSE
)
```

Chromosome 3

```
write.table(
  chr3_snps$SNP,
  file      = here(
    "output", "populations", "linkage", "chr3_ld_SNPs.txt"
  ),
  sep       = "\t",
  row.names = FALSE,
  col.names = FALSE,
  quote     = FALSE
)
```

Now create new files for each chromosome

Create dirs

```
# make directory
mkdir -p output/populations/linkage/chr1;
mkdir -p output/populations/linkage/chr2;
mkdir -p output/populations/linkage/chr3;
```

Chromosome 1

```
plink2 \
--allow-extra-chr \
--bfile output/populations/linkage/ld1 \
--keep-fam output/populations/linkage/pops_4ld.txt \
--extract output/populations/linkage/chr1_ld_SNPs.txt \
--make-bed \
--maf 0.01 \
--export vcf \
--out output/populations/linkage/chr1/ld1 \
--silent;
grep 'samples\|variants\|remaining' output/populations/linkage/chr1/ld1.log
```

```
## 235 samples (30 females, 65 males, 140 ambiguous; 235 founders) loaded from
## 104139 variants loaded from output/populations/linkage/ld1.bim.
## --extract: 23397 variants remaining.
## --keep-fam: 212 samples remaining.
## 212 samples (28 females, 61 males, 123 ambiguous; 212 founders) remaining after
## 1044 variants removed due to allele frequency threshold(s)
## 22353 variants remaining after main filters.
```

Chromosome 2

```
plink2 \
--allow-extra-chr \
--bfile output/populations/linkage/ld1 \
--keep-fam output/populations/linkage/pops_4ld.txt \
--extract output/populations/linkage/chr2_ld_SNPs.txt \
--make-bed \
--maf 0.01 \
--export vcf \
--out output/populations/linkage/chr2/ld1 \
--silent;
grep 'samples\|variants\|remaining' output/populations/linkage/chr2/ld1.log
```

```
## 235 samples (30 females, 65 males, 140 ambiguous; 235 founders) loaded from
## 104139 variants loaded from output/populations/linkage/ld1.bim.
## --extract: 43518 variants remaining.
## --keep-fam: 212 samples remaining.
## 212 samples (28 females, 61 males, 123 ambiguous; 212 founders) remaining after
## 1786 variants removed due to allele frequency threshold(s)
## 41732 variants remaining after main filters.
```

Chromosome 3

```
plink2 \
--allow-extra-chr \
--bfile output/populations/linkage/ld1 \
--keep-fam output/populations/linkage/pops_4ld.txt \
--extract output/populations/linkage/chr3_ld_SNPs.txt \
--make-bed \
--maf 0.01 \
--export vcf \
--out output/populations/linkage/chr3/ld1 \
--silent;
grep 'samples\|variants\|remaining' output/populations/linkage/chr3/ld1.log
```

```
## 235 samples (30 females, 65 males, 140 ambiguous; 235 founders) loaded from
## 104139 variants loaded from output/populations/linkage/ld1.bim.
## --extract: 37224 variants remaining.
## --keep-fam: 212 samples remaining.
## 212 samples (28 females, 61 males, 123 ambiguous; 212 founders) remaining after
## 1557 variants removed due to allele frequency threshold(s)
## 35667 variants remaining after main filters.
```

Check the vcfs

```
ls output/populations/linkage/chr*/ld1.vcf
```

```
## output/populations/linkage/chr1/ld1.vcf
## output/populations/linkage/chr2/ld1.vcf
## output/populations/linkage/chr3/ld1.vcf
```

### 3.1 Chromosome 1

Clean env and memory

```
# Remove all objects from the environment
rm(list = ls())

# Run the garbage collector to free up memory
gc()
```

```
##           used  (Mb) gc trigger  (Mb) limit (Mb) max used  (Mb)
## Ncells 2557457 136.6    4548762 243.0         NA  4548762 243.0
## Vcells 4434440  33.9   10825744  82.6      32768 10752723  82.1
```

#### 3.1.1 run PopLDdecay

Prepare the files required by PopLDdecay

```
bcftools query -l output/populations/linkage/chr1/ld1.vcf > output/populations/linkage/vcf_samples.txt

bcftools query -l output/populations/linkage/chr1/ld1.vcf | cut -d'_' -f1 | sort | uniq > output/populations/linkage/unique_pops_from_vcf.txt
while read pop; do
    grep "^${pop}_" output/populations/linkage/vcf_samples.txt > output/populations/linkage/${pop}_samples.txt
done < output/populations/linkage/unique_pops_from_vcf.txt
```

```
while read pop; do
    PopLDdecay -InVCF output/populations/linkage/chr1/ld1.vcf \
               -OutType 4 \
               -MaxDist 1000 \
               -MAF 0.01 \
               -OutFilterSNP \
               -OutStat output/populations/linkage/chr1/${pop}_LDdecay.gz \
               -SubPop output/populations/linkage/${pop}_samples.txt
done < output/populations/linkage/unique_pops_from_vcf.txt
```

#### 3.1.2 Plot using PopLDdecay

Get the files we need

```
for file in output/populations/linkage/chr1/*_LDdecay.stat.gz; do 
    pop_name=$(basename $file _LDdecay.stat.gz)
    echo "$file    $pop_name"
done > output/populations/linkage/chr1/ld_decay_results_list.txt

head -100 output/populations/linkage/chr1/ld_decay_results_list.txt
```

Plot using PopLDdecay. It will create files that we can use to plot
with ggplot

```
perl /Users/lucianocosme/Packages/PopLDdecay/bin/Plot_MultiPop.pl --help
```

```
perl /Users/lucianocosme/Packages/PopLDdecay/bin/Plot_MultiPop.pl \
      -in output/populations/linkage/chr1/ld_decay_results_list.txt \
      -output output/populations/linkage/chr1/all_pops \
      -bin1 10 \
      -bin2 100 \
      -break 100 \
      -maxX 1000 \
      -measure r2 \
      -method MeanBin \
      -keepR
```

#### 3.1.3 Plot using ggplot

```
# Define the path to the .fam file using here
fam_file_path <- here("output", "populations", "linkage", "chr1", "ld1.fam")

# Read the .fam file
fam_data <- read.table(fam_file_path, header = FALSE, stringsAsFactors = FALSE)

# Extract the family names from the first column
populations <- unique(fam_data$V1)

# Determine the number of unique populations
num_populations <- length(unique(populations))

# Fetch colors accordingly
colors_set3 <- brewer.pal(min(12, num_populations), "Set3")
colors_pastel2 <- brewer.pal(min(8, num_populations - length(colors_set3)), "Pastel2")
colors_paired <- brewer.pal(min(12, num_populations - length(colors_set3) - length(colors_pastel2)), "Paired")

# Combine the colors
colors <- c(colors_set3, colors_pastel2, colors_paired)

# Ensure we're only taking as many colors as there are populations
colors <- colors[1:num_populations]

# Create a named vector for populations and their colors
color_mapping <- setNames(colors, populations)

# Initialize an empty list to store data frames
data_frames <- list()

# Read data from each population file and store it in the list
for (pop in populations) {
  file_path <- paste("output/populations/linkage/chr1/all_pops.", pop, sep = "")
  data <- read.table(file_path)
  data$population <- pop  # Add a column for population name
  data_frames[[pop]] <- data
}

# Combine all data frames into a single data frame
combined_data <- do.call(rbind, data_frames)

# Create a named vector for populations and their colors
color_mapping <- setNames(colors, populations)

# Calculate half distances
half_distances <- combined_data |>
  group_by(population) |>
  mutate(max_r2 = max(V2)) |>
  filter(V2 <= max_r2 / 2) |>
  arrange(V1) |>
  slice(1) |>
  select(population, half_distance = V1)

# Calculate the maximum V2 value for each population
max_values <- combined_data |>
  group_by(population) |>
  summarize(max_V2 = max(V2))

# Merge the maximum V2 values with the half_distances dataframe
half_distances <- merge(half_distances, max_values, by = "population")

# Create the ggplot
p <- ggplot(combined_data, aes(x = V1 / 1000, y = V2, group = population, color = population)) +
  geom_point(size = .3, alpha = 0.1) +
  geom_smooth(method = "loess", se = FALSE, span = 0.3, color = "black", linewidth = 0.5) +
  geom_vline(data = half_distances, aes(xintercept = half_distance / 1000), color = "blue", linetype = "dashed") +
  geom_text_repel(data = half_distances, aes(x = half_distance / 1000 + 130, y = max_V2, label = paste0(round(half_distance / 1000, 1), "Kb")), 
            color = "blue", vjust = -1, size = 3) +
  labs(
    title = "",
    x = "Distance(Kb)",
    y = expression(r^2)
  ) +
  scale_color_manual(values = color_mapping) +
  scale_y_continuous(labels = label_comma(accuracy = 0.01)) +
  scale_x_continuous(labels = label_comma()) +
  theme_minimal() +
  theme(
    legend.position = "none",
    axis.title.x = element_text(face = "bold", size = 14),
    axis.title.y = element_text(face = "bold", size = 14),
    strip.text = element_text(face = "bold")
  ) +
  facet_wrap(~ population, scales = "free_y", ncol = 2) +
  coord_cartesian(xlim = c(0, 1000))

p
```

```
## `geom_smooth()` using formula = 'y ~ x'
```

```
ggsave("output/populations/linkage/chr1/decay_chr1.pdf", plot = p, width = 5, height = 8)
```

```
## `geom_smooth()` using formula = 'y ~ x'
```

Save the data to use later

```
saveRDS(half_distances, here(
  "output", "populations", "linkage","chr1", "chr1.rds"
))
```

### 3.2 Chromosome 2

Clean env and memory

```
# Remove all objects from the environment
rm(list = ls())

# Run the garbage collector to free up memory
gc()
```

```
##           used  (Mb) gc trigger (Mb) limit (Mb) max used (Mb)
## Ncells 3000593 160.3    5804207  310         NA  5804207  310
## Vcells 7553105  57.7   23063803  176      32768 23063803  176
```

#### 3.1.1 run PopLDdecay

```
while read pop; do
    PopLDdecay -InVCF output/populations/linkage/chr2/ld1.vcf \
               -OutType 4 \
               -MaxDist 1000 \
               -MAF 0.01 \
               -OutFilterSNP \
               -OutStat output/populations/linkage/chr2/${pop}_LDdecay.gz \
               -SubPop output/populations/linkage/${pop}_samples.txt
done < output/populations/linkage/unique_pops_from_vcf.txt
```

#### 3.1.2 Plot using PopLDdecay

Get the files we need

```
for file in output/populations/linkage/chr2/*_LDdecay.stat.gz; do 
    pop_name=$(basename $file _LDdecay.stat.gz)
    echo "$file    $pop_name"
done > output/populations/linkage/chr2/ld_decay_results_list.txt

head -100 output/populations/linkage/chr2/ld_decay_results_list.txt
```

Plot using PopLDdecay. It will create files that we can use to plot
with ggplot

```
perl /Users/lucianocosme/Packages/PopLDdecay/bin/Plot_MultiPop.pl \
      -in output/populations/linkage/chr2/ld_decay_results_list.txt \
      -output output/populations/linkage/chr2/all_pops \
      -bin1 10 \
      -bin2 100 \
      -break 100 \
      -maxX 1000 \
      -measure r2 \
      -method MeanBin \
      -keepR
```

#### 3.1.3 Plot using ggplot

```
# Define the path to the .fam file using here
fam_file_path <- here("output", "populations", "linkage", "chr2", "ld1.fam")

# Read the .fam file
fam_data <- read.table(fam_file_path, header = FALSE, stringsAsFactors = FALSE)

# Extract the family names from the first column
populations <- unique(fam_data$V1)

# Determine the number of unique populations
num_populations <- length(unique(populations))

# Fetch colors accordingly
colors_set3 <- brewer.pal(min(12, num_populations), "Set3")
colors_pastel2 <- brewer.pal(min(8, num_populations - length(colors_set3)), "Pastel2")
colors_paired <- brewer.pal(min(12, num_populations - length(colors_set3) - length(colors_pastel2)), "Paired")

# Combine the colors
colors <- c(colors_set3, colors_pastel2, colors_paired)

# Ensure we're only taking as many colors as there are populations
colors <- colors[1:num_populations]

# Create a named vector for populations and their colors
color_mapping <- setNames(colors, populations)

# Initialize an empty list to store data frames
data_frames <- list()

# Read data from each population file and store it in the list
for (pop in populations) {
  file_path <- paste("output/populations/linkage/chr2/all_pops.", pop, sep = "")
  data <- read.table(file_path)
  data$population <- pop  # Add a column for population name
  data_frames[[pop]] <- data
}

# Combine all data frames into a single data frame
combined_data <- do.call(rbind, data_frames)

# Create a named vector for populations and their colors
color_mapping <- setNames(colors, populations)

# Calculate half distances
half_distances <- combined_data |>
  group_by(population) |>
  mutate(max_r2 = max(V2)) |>
  filter(V2 <= max_r2 / 2) |>
  arrange(V1) |>
  slice(1) |>
  select(population, half_distance = V1)

# Calculate the maximum V2 value for each population
max_values <- combined_data |>
  group_by(population) |>
  summarize(max_V2 = max(V2))

# Merge the maximum V2 values with the half_distances dataframe
half_distances <- merge(half_distances, max_values, by = "population")

# Create the ggplot
p <- ggplot(combined_data, aes(x = V1 / 1000, y = V2, group = population, color = population)) +
  geom_point(size = .3, alpha = 0.1) +
  geom_smooth(method = "loess", se = FALSE, span = 0.3, color = "black", linewidth = 0.5) +
  geom_vline(data = half_distances, aes(xintercept = half_distance / 1000), color = "blue", linetype = "dashed") +
  geom_text_repel(data = half_distances, aes(x = half_distance / 1000 + 130, y = max_V2, label = paste0(round(half_distance / 1000, 1), "Kb")), 
            color = "blue", vjust = -1, size = 3) +
  labs(
    title = "",
    x = "Distance(Kb)",
    y = expression(r^2)
  ) +
  scale_color_manual(values = color_mapping) +
  scale_y_continuous(labels = label_comma(accuracy = 0.01)) +
  scale_x_continuous(labels = label_comma()) +
  theme_minimal() +
  theme(
    legend.position = "none",
    axis.title.x = element_text(face = "bold", size = 14),
    axis.title.y = element_text(face = "bold", size = 14),
    strip.text = element_text(face = "bold")
  ) +
  facet_wrap(~ population, scales = "free_y", ncol = 2) +
  coord_cartesian(xlim = c(0, 1000))

p
```

```
## `geom_smooth()` using formula = 'y ~ x'
```

```
ggsave("output/populations/linkage/chr2/decay_chr2.pdf", plot = p, width = 5, height = 8)
```

```
## `geom_smooth()` using formula = 'y ~ x'
```

Save the data to use later

```
saveRDS(half_distances, here(
  "output", "populations", "linkage","chr2", "chr2.rds"
))
```

### 3.3 Chromosome 3

Clean env and memory

```
# Remove all objects from the environment
rm(list = ls())

# Run the garbage collector to free up memory
gc()
```

```
##           used  (Mb) gc trigger  (Mb) limit (Mb) max used  (Mb)
## Ncells 3001341 160.3    5804207 310.0         NA  5804207 310.0
## Vcells 7753626  59.2   27849300 212.5      32768 24156306 184.3
```

#### 3.1.1 run PopLDdecay

```
while read pop; do
    PopLDdecay -InVCF output/populations/linkage/chr3/ld1.vcf \
               -OutType 4 \
               -MaxDist 1000 \
               -MAF 0.01 \
               -OutFilterSNP \
               -OutStat output/populations/linkage/chr3/${pop}_LDdecay.gz \
               -SubPop output/populations/linkage/${pop}_samples.txt
done < output/populations/linkage/unique_pops_from_vcf.txt
```

#### 3.1.2 Plot using PopLDdecay

Get the files we need

```
for file in output/populations/linkage/chr3/*_LDdecay.stat.gz; do 
    pop_name=$(basename $file _LDdecay.stat.gz)
    echo "$file    $pop_name"
done > output/populations/linkage/chr3/ld_decay_results_list.txt

head -100 output/populations/linkage/chr3/ld_decay_results_list.txt
```

Plot using PopLDdecay. It will create files that we can use to plot
with ggplot

```
perl /Users/lucianocosme/Packages/PopLDdecay/bin/Plot_MultiPop.pl \
      -in output/populations/linkage/chr3/ld_decay_results_list.txt \
      -output output/populations/linkage/chr3/all_pops \
      -bin1 10 \
      -bin2 100 \
      -break 100 \
      -maxX 1000 \
      -measure r2 \
      -method MeanBin \
      -keepR
```

#### 3.1.3 Plot using ggplot

```
# Define the path to the .fam file using here
fam_file_path <- here("output", "populations", "linkage", "chr3", "ld1.fam")

# Read the .fam file
fam_data <- read.table(fam_file_path, header = FALSE, stringsAsFactors = FALSE)

# Extract the family names from the first column
populations <- unique(fam_data$V1)

# Determine the number of unique populations
num_populations <- length(unique(populations))

# Fetch colors accordingly
colors_set3 <- brewer.pal(min(12, num_populations), "Set3")
colors_pastel2 <- brewer.pal(min(8, num_populations - length(colors_set3)), "Pastel2")
colors_paired <- brewer.pal(min(12, num_populations - length(colors_set3) - length(colors_pastel2)), "Paired")

# Combine the colors
colors <- c(colors_set3, colors_pastel2, colors_paired)

# Ensure we're only taking as many colors as there are populations
colors <- colors[1:num_populations]

# Create a named vector for populations and their colors
color_mapping <- setNames(colors, populations)

# Initialize an empty list to store data frames
data_frames <- list()

# Read data from each population file and store it in the list
for (pop in populations) {
  file_path <- paste("output/populations/linkage/chr3/all_pops.", pop, sep = "")
  data <- read.table(file_path)
  data$population <- pop  # Add a column for population name
  data_frames[[pop]] <- data
}

# Combine all data frames into a single data frame
combined_data <- do.call(rbind, data_frames)

# Create a named vector for populations and their colors
color_mapping <- setNames(colors, populations)

# Calculate half distances
half_distances <- combined_data |>
  group_by(population) |>
  mutate(max_r2 = max(V2)) |>
  filter(V2 <= max_r2 / 2) |>
  arrange(V1) |>
  slice(1) |>
  select(population, half_distance = V1)

# Calculate the maximum V2 value for each population
max_values <- combined_data |>
  group_by(population) |>
  summarize(max_V2 = max(V2))

# Merge the maximum V2 values with the half_distances dataframe
half_distances <- merge(half_distances, max_values, by = "population")

# Create the ggplot
p <- ggplot(combined_data, aes(x = V1 / 1000, y = V2, group = population, color = population)) +
  geom_point(size = .3, alpha = 0.1) +
  geom_smooth(method = "loess", se = FALSE, span = 0.3, color = "black", linewidth = 0.5) +
  geom_vline(data = half_distances, aes(xintercept = half_distance / 1000), color = "blue", linetype = "dashed") +
  geom_text_repel(data = half_distances, aes(x = half_distance / 1000 + 130, y = max_V2, label = paste0(round(half_distance / 1000, 1), "Kb")), 
            color = "blue", vjust = -1, size = 3) +
  labs(
    title = "",
    x = "Distance(Kb)",
    y = expression(r^2)
  ) +
  scale_color_manual(values = color_mapping) +
  scale_y_continuous(labels = label_comma(accuracy = 0.01)) +
  scale_x_continuous(labels = label_comma()) +
  theme_minimal() +
  theme(
    legend.position = "none",
    axis.title.x = element_text(face = "bold", size = 14),
    axis.title.y = element_text(face = "bold", size = 14),
    strip.text = element_text(face = "bold")
  ) +
  facet_wrap(~ population, scales = "free_y", ncol = 2) +
  coord_cartesian(xlim = c(0, 1000))

p
```

```
## `geom_smooth()` using formula = 'y ~ x'
```

```
ggsave("output/populations/linkage/chr3/decay_chr3.pdf", plot = p, width = 5, height = 8)
```

```
## `geom_smooth()` using formula = 'y ~ x'
```

Save the data to use later

```
saveRDS(half_distances, here(
  "output", "populations", "linkage", "chr3", "chr3.rds"
))
```

## 4. Plot comparying chromosomes

Clean env and memory

```
# Remove all objects from the environment
rm(list = ls())

# Run the garbage collector to free up memory
gc()
```

```
##           used  (Mb) gc trigger  (Mb) limit (Mb) max used  (Mb)
## Ncells 3002096 160.4    5804207 310.0         NA  5804207 310.0
## Vcells 7818001  59.7   27920698 213.1      32768 27920698 213.1
```

Create data frame with the data from each chromosome

Import the data

```
# Chromosome 1
chr1 <- readRDS(here(
  "output", "populations", "linkage", "chr1", "chr1.rds"
)) |> dplyr::select(population, half_distance
) |> dplyr::rename(
  chr1 = half_distance
) |>
  mutate(chr1 = chr1/1000)

# Chromosome 2
chr2 <- readRDS(here(
  "output", "populations", "linkage", "chr2", "chr2.rds"
)) |> dplyr::select(population, half_distance
) |> dplyr::rename(
  chr2 = half_distance
) |>
  mutate(chr2 = chr2/1000)

# Chromosome 3
chr3 <- readRDS(here(
  "output", "populations", "linkage", "chr3", "chr3.rds"
)) |> dplyr::select(population, half_distance
) |> dplyr::rename(
  chr3 = half_distance
) |>
  mutate(chr3 = chr3/1000)

# Merge
distance <- merge(merge(chr1, chr2, by="population"), chr3, by="population")

# Convert data from wide to long format
data_long <- gather(distance, key = "chr", value = "value", -population)

# Make it capital
data_long$chr <- tools::toTitleCase(data_long$chr)

# Remove KAT because it has mosquitoes collected from different source and had higher linkage due it
data_long <- subset(data_long, population != "KAT")
```

Make one plot

```
# Define a custom color palette
custom_palette <- c(
  "Chr1" = "#edb975", 
  "Chr2" = "#c1f5ff", 
  "Chr3" = "#ff72f3"
)

# Reordering the levels of the chr factor
data_long$chr <- factor(data_long$chr, levels = c("Chr3", "Chr2", "Chr1"))

# source the plotting function
source(here("scripts", "analysis", "my_theme2.R"))

# Plotting half_distance with borders and spaced bars
ggplot(data_long, aes(x = population, y = value, fill = chr)) +
  geom_bar(stat = "identity", position = position_dodge(width = 0.9), color = "black", width = 0.7) +
  labs(title = "", y = "Half Distance (kb)", x = "Population") +
  geom_text(aes(label = round(value, 0)), position = position_dodge(width = 0.8), vjust = 0.5, hjust = -0.2, size = 4) +
  my_theme() +
  coord_flip() +
  scale_fill_manual(values = custom_palette, name = "Chromosome", breaks = c("Chr1", "Chr2", "Chr3")) +
  theme(legend.position = "top",
        plot.margin = margin(t = 10, r = 30, b = 10, l = 10, unit = "pt"))
```

Import sample locations

```
sampling_loc <- readRDS(here("output", "populations", "sampling_loc.rds"))
head(sampling_loc)
```

```
## # A tibble: 6 × 6
##   Pop_City   Country  Latitude Longitude Region Abbreviation
##   <chr>      <chr>       <dbl>     <dbl> <chr>  <chr>       
## 1 Gelephu    Bhutan       26.9      90.5 Asia   GEL         
## 2 Phnom Penh Cambodia     11.6     105.  Asia   CAM         
## 3 Hainan     China        19.2     110.  Asia   HAI         
## 4 Yunnan     China        24.5     101.  Asia   YUN         
## 5 Hunan      China        27.6     112.  Asia   HUN         
## 6 Bengaluru  India        13.0      77.6 Asia   BEN
```

```
head(data_long)
```

```
##   population  chr value
## 1        BEN Chr1  18.9
## 2        CAM Chr1  18.9
## 3        CHA Chr1  12.3
## 4        HAI Chr1   1.5
## 5        HOC Chr1  54.6
## 6        HUN Chr1  12.7
```

```
# Join with sampling_loc to get sampling localities
distance2 <- data_long |>
  left_join(sampling_loc, by = c("population" = "Abbreviation"))
head(distance2)
```

```
##   population  chr value         Pop_City  Country Latitude Longitude Region
## 1        BEN Chr1  18.9        Bengaluru    India  12.9716   77.5946   Asia
## 2        CAM Chr1  18.9       Phnom Penh Cambodia  11.5564  104.9282   Asia
## 3        CHA Chr1  12.3      Chanthaburi Thailand  12.6097  102.1045   Asia
## 4        HAI Chr1   1.5           Hainan    China  19.2000  109.6000   Asia
## 5        HOC Chr1  54.6 Ho Chi Minh City  Vietnam  10.7626  106.6602   Asia
## 6        HUN Chr1  12.7            Hunan    China  27.6253  111.8569   Asia
```

Add the name of the city/countries to the plot

```
# Creating the label with population, city, and country for the y-axis
distance2$pop_city_country_label <- paste(distance2$population, "\n", distance2$Pop_City, "\n(", distance2$Country, ")", sep = "")

# Sorting by Country, then City, and then by Population to ensure populations from the same country (and city) are plotted together
distance2 <- distance2 %>% arrange(Country, Pop_City, population)

# Adjusting the factor levels for plotting in the desired order
distance2$pop_city_country_label <- fct_inorder(distance2$pop_city_country_label)


# Plotting the data
ggplot(distance2, aes(x = pop_city_country_label, y = value, fill = chr)) +
  geom_bar(stat = "identity", position = position_dodge(width = 0.9), color = "black", width = 0.7) +
  labs(title = "", y = "Half Distance (kb)", x = "") +
  geom_text(aes(label = round(value, 0)), position = position_dodge(width = 0.8), vjust = 0.5, hjust = -0.2, size = 3.5) +
  my_theme() +
  coord_flip() +
  labs(title = "", y = "Half Distance (kb)", x = "Population") +
  scale_fill_manual(values = custom_palette, name = "Chromosome", breaks = c("Chr1", "Chr2", "Chr3")) +
  theme(legend.position = "top",
        plot.margin = margin(t = 10, r = 30, b = 10, l = 10, unit = "pt"),
        axis.text.y = element_text(angle = 0, hjust = 1))
```

```
ggsave("output/populations/linkage/decay_albopictus.pdf", width = 6, height = 12)
```

```
# Create table
dist_table <- distance2 |>
  dplyr::select(
    population, Pop_City, Country, chr, value
  ) |>
  dplyr::rename(
    City = Pop_City,
    Population = population
  )

# Spread and arrange
dist_table_condensed <- dist_table %>%
  spread(key = chr, value = value) %>%
  dplyr::select(Population, Country, City, Chr1, Chr2, Chr3) |>
  dplyr::arrange(Country, City)


# Create table
ft <- flextable::flextable(dist_table_condensed)


# Apply zebra striping with bg
ft <- flextable::theme_zebra(ft)

# Show it
ft
```

| Population | Country | City | Chr1 | Chr2 | Chr3 |
| --- | --- | --- | --- | --- | --- |
| CAM | Cambodia | Phnom Penh | 18.9 | 115.2 | 139.4 |
| HAI | China | Hainan | 1.5 | 63.8 | 66.0 |
| HUN | China | Hunan | 12.7 | 57.9 | 57.0 |
| YUN | China | Yunnan | 16.1 | 335.6 | 1.0 |
| BEN | India | Bengaluru | 18.9 | 194.6 | 207.6 |
| INJ | Indonesia | Jakarta | 37.3 | 91.1 | 58.8 |
| SUF | Indonesia | Sulawesi (Forest) | 39.9 | 333.7 | 163.1 |
| SUU | Indonesia | Sulawesi (Urban) | 63.8 | 353.5 | 278.7 |
| KAG | Japan | Kagoshima | 45.7 | 30.3 | 3.9 |
| KAN | Japan | Kanazawa | 42.0 | 46.4 | 57.7 |
| OKI | Japan | Okinawa | 6.1 | 21.4 | 63.0 |
| UTS | Japan | Utsunomiya | 33.9 | 14.6 | 25.0 |
| MAT | Malaysia | Tambun | 25.5 | 157.4 | 168.3 |
| TAI | Taiwan | Tainan | 9.9 | 261.2 | 121.4 |
| CHA | Thailand | Chanthaburi | 12.3 | 174.9 | 96.6 |
| KAC | Thailand | Kanchanaburi | 22.3 | 378.1 | 46.7 |
| LAM | Thailand | Lampang | 18.9 | 281.7 | 88.8 |
| SSK | Thailand | Sisaket | 3.1 | 170.6 | 147.8 |
| HOC | Vietnam | Ho Chi Minh City | 54.6 | 182.5 | 146.0 |
| QNC | Vietnam | Quy Nhon City | 3.5 | 118.2 | 6.6 |

```
# Create a new Word document
doc <- read_docx()

# Add the flextable
doc <- body_add_flextable(doc, value = ft)

# Save the document to a file
# Define the path for saving the Word document
file_path <- here("output", "populations", "linkage", "decay_albopictus.docx")

print(doc, target = file_path)
```

Get counts

```
# Define the path to the .fam file using here
fam_file_path <- here("output", "populations", "linkage", "chr3", "ld1.fam")

# Read the .fam file
fam_data <- read.table(fam_file_path, header = FALSE, stringsAsFactors = FALSE)

# Get the number of individuals
individuals_count <- table(fam_data$V1)
individuals_count
```

```
## 
## BEN CAM CHA HAI HOC HUN INJ KAC KAG KAN KAT LAM MAT OKI QNC SSK SUF SUU TAI UTS 
##  12  12  12  12   7  12  11   6  12  11   8   9  12  11  12  12   6   6   8  12 
## YUN 
##   9
```

Merge the data

```
# Convert individuals_count to a data frame
individuals_df <- as.data.frame(individuals_count)
colnames(individuals_df) <- c("Population", "Count")

# Merge with dist_table_condensed
merged_data <- merge(dist_table_condensed, individuals_df, by="Population")
```

Check correlation

```
# Chr1 vs Count
plot_Chr1 <- ggplot(merged_data, aes(x = Count, y = Chr1)) +
  geom_point() +
  geom_smooth(method = "lm", col = "red") +
  ggtitle("Correlation between Count and Chr1") +
  labs(x = "Count", y = "LD Half Distance - Chr1")

# Chr2 vs Count
plot_Chr2 <- ggplot(merged_data, aes(x = Count, y = Chr2)) +
  geom_point() +
  geom_smooth(method = "lm", col = "blue") +
  ggtitle("Correlation between Count and Chr2") +
  labs(x = "Count", y = "LD Half Distance - Chr2")

# Chr3 vs Count
plot_Chr3 <- ggplot(merged_data, aes(x = Count, y = Chr3)) +
  geom_point() +
  geom_smooth(method = "lm", col = "green") +
  ggtitle("Correlation between Count and Chr3") +
  labs(x = "Count", y = "LD Half Distance - Chr3")

# Display the plots
print(plot_Chr1)
```

```
## `geom_smooth()` using formula = 'y ~ x'
```

```
print(plot_Chr2)
```

```
## `geom_smooth()` using formula = 'y ~ x'
```

```
print(plot_Chr3)
```

```
## `geom_smooth()` using formula = 'y ~ x'
```

Estimate r2

```
# For Chr1
model_Chr1 <- lm(Chr1 ~ Count, data = merged_data)
summary_Chr1 <- summary(model_Chr1)
R2_Chr1 <- summary_Chr1$r.squared
p_value_Chr1 <- coef(summary_Chr1)[2,4]

# For Chr2
model_Chr2 <- lm(Chr2 ~ Count, data = merged_data)
summary_Chr2 <- summary(model_Chr2)
R2_Chr2 <- summary_Chr2$r.squared
p_value_Chr2 <- coef(summary_Chr2)[2,4]

# For Chr3
model_Chr3 <- lm(Chr3 ~ Count, data = merged_data)
summary_Chr3 <- summary(model_Chr3)
R2_Chr3 <- summary_Chr3$r.squared
p_value_Chr3 <- coef(summary_Chr3)[2,4]

# Display R2 and p-values
cat("For Chr1: R2 =", R2_Chr1, ", p-value =", p_value_Chr1, "\n")
```

```
## For Chr1: R2 = 0.2268475 , p-value = 0.03375708
```

```
cat("For Chr2: R2 =", R2_Chr2, ", p-value =", p_value_Chr2, "\n")
```

```
## For Chr2: R2 = 0.6429323 , p-value = 2.125989e-05
```

```
cat("For Chr3: R2 =", R2_Chr3, ", p-value =", p_value_Chr3, "\n")
```

```
## For Chr3: R2 = 0.1115358 , p-value = 0.150124
```

```
# For Chr1
model_Chr1 <- lm(Chr1 ~ Count, data = merged_data)
summary_Chr1 <- summary(model_Chr1)
R2_Chr1 <- summary_Chr1$r.squared
p_value_Chr1 <- coef(summary_Chr1)[2,4]

# For Chr2
model_Chr2 <- lm(Chr2 ~ Count, data = merged_data)
summary_Chr2 <- summary(model_Chr2)
R2_Chr2 <- summary_Chr2$r.squared
p_value_Chr2 <- coef(summary_Chr2)[2,4]

# For Chr3
model_Chr3 <- lm(Chr3 ~ Count, data = merged_data)
summary_Chr3 <- summary(model_Chr3)
R2_Chr3 <- summary_Chr3$r.squared
p_value_Chr3 <- coef(summary_Chr3)[2,4]

# Display R2 and p-values
cat("For Chr1: R2 =", R2_Chr1, ", p-value =", p_value_Chr1, "\n")
```

```
## For Chr1: R2 = 0.2268475 , p-value = 0.03375708
```

```
cat("For Chr2: R2 =", R2_Chr2, ", p-value =", p_value_Chr2, "\n")
```

```
## For Chr2: R2 = 0.6429323 , p-value = 2.125989e-05
```

```
cat("For Chr3: R2 =", R2_Chr3, ", p-value =", p_value_Chr3, "\n")
```

```
## For Chr3: R2 = 0.1115358 , p-value = 0.150124
```

Annotate the plots

```
# Helper function to extract coefficients, R2, and create the label
get_annotation <- function(model) {
  coefs <- coef(model)
  eq <- sprintf("y = %.2fx + %.2f", coefs[2], coefs[1])
  r2 <- sprintf("R^2 = %.2f", summary(model)$r.squared)
  return(paste(eq, r2, sep = "\n"))
}

# Annotations for each chromosome
annotate_Chr1 <- get_annotation(model_Chr1)
annotate_Chr2 <- get_annotation(model_Chr2)
annotate_Chr3 <- get_annotation(model_Chr3)

# Plot with annotations
plot_with_annotation <- function(data, yvar, label) {
  ggplot(data, aes(x = Count, y = !!sym(yvar))) +
    geom_point() +
    geom_smooth(method = "lm", col = "red") +
    annotate("text", x = max(data$Count) * 0.7, y = max(data[[yvar]]) * 0.1, label = label, hjust = 0) +
    labs(x = "Count", y = yvar)
}

plot1 <- plot_with_annotation(merged_data, "Chr1", annotate_Chr1)
plot2 <- plot_with_annotation(merged_data, "Chr2", annotate_Chr2)
plot3 <- plot_with_annotation(merged_data, "Chr3", annotate_Chr3)

# Display the plots with annotations
print(plot1)
```

```
## `geom_smooth()` using formula = 'y ~ x'
```

```
print(plot2)
```

```
## `geom_smooth()` using formula = 'y ~ x'
```

```
print(plot3)
```

```
## `geom_smooth()` using formula = 'y ~ x'
```

Create a facet plot

```
# Read the data
merged_data_long <- readRDS(here("output", "populations", "linkage", "merged_data_long.rds"))

# Function to compute the linear model details
get_lm_details <- function(data, yvar) {
  model <- lm(data[[yvar]] ~ data$Count)
  coefs <- coef(model)
  eq <- sprintf("y = %.2fx + %.2f", coefs[2], coefs[1])
  r2 <- sprintf("R^2 = %.2f", summary(model)$r.squared)
  return(tibble(Chromosome = yvar, Equation = eq, R2 = r2))
}

# Compute details for each chromosome and bind rows
annotations <- bind_rows(get_lm_details(merged_data, "Chr1"), 
                         get_lm_details(merged_data, "Chr2"), 
                         get_lm_details(merged_data, "Chr3"))

# Compute maximum y for annotations for each chromosome
annotations <- annotations %>%
  left_join(merged_data_long %>% group_by(Chromosome) %>% summarise(MaxY = max(LD_Half_Distance, na.rm = TRUE)), by = "Chromosome")

# Plotting with annotations correctly positioned in the top right corner
ggplot(merged_data_long, aes(x = Count, y = LD_Half_Distance)) +
  geom_point() +
  geom_smooth(method = "lm", col = "red") +
  geom_text(data = annotations, aes(label = paste(Equation, R2, sep = "\n"), y = MaxY), 
            x = max(merged_data$Count), hjust = 1, vjust = 1) +
  facet_wrap(~ Chromosome, scales = "free_y", ncol = 1) +
  labs(x = "Number of Samples", y = "LD Half Distance (kb)") +
  my_theme()
```

```
## `geom_smooth()` using formula = 'y ~ x'
```

```
# Save the plot
ggsave(here("output", "populations", "linkage", "decay_sample_size.pdf"), width = 4, height = 6)
```

```
## `geom_smooth()` using formula = 'y ~ x'
```

Plot all countries

```
# Calculate mean for each Country and Chromosome
mean_data <- merged_data_long %>%
  group_by(Country, Chromosome) %>%
  summarise(mean_value = mean(LD_Half_Distance, na.rm = TRUE), .groups = "drop")

# Jitter plot overlaid on boxplot for each chromosome by country without legend
ggplot(merged_data_long, aes(x = Country, y = LD_Half_Distance)) +
  geom_boxplot(alpha = 0.5) +
  geom_jitter(aes(color = Country), width = 0.2) +
  geom_text(data = mean_data, aes(y = mean_value, label = round(mean_value, 2)), vjust = 0.5, hjust = -0.5, size = 3) + # Annotate mean value
  facet_wrap(~ Chromosome, scales = "free_y", ncol = 1) +
  theme_minimal() +
  labs(title = "", y = "LD Half Distance (kb)") +
  my_theme() +
  coord_flip() +
  theme(axis.text.x = element_text(angle = 0, hjust = 1), legend.position = "none")
```

```
# Save the plot
ggsave(here("output", "populations", "linkage", "decay_mean_by_country.pdf"), width = 6, height = 6)
```

Legend: The Linkage Disequilibrium (LD) half distance values in
kilobases (kb) for the Aedes albopictus mosquito across various Asian
countries, grouped by chromosome. Each boxplot displays the
interquartile range of the LD Half Distance values, with the horizontal
line in the box marking the median. Colored jittered points represent
individual data points, with each color corresponding to a specific
country. The plot also annotates each country-chromosome combination’s
mean LD half distance. Distinct facets separate the values for different
chromosomes to offer clear visualization.

Use ggstatplot to test if there is any significant differences
Chr1

```
# Reorder the levels of Country based on the median of Chr1
merged_data <- merged_data %>%
  mutate(Country = fct_reorder(Country, Chr1, .fun = median))

# Plot
ggbetweenstats(
  data = merged_data,
  x = Country,
  y = Chr1,
  title = "Chr1",
  xlab = "Country",
  ylab = "LD Half Distance (kb)",
  ggplot.component = list(theme(legend.position = "none"))
) + my_theme() + theme(legend.position = "none")
```

```
# Save the plot
ggsave(here("output", "populations", "linkage", "decay_chr1_test.pdf"), width = 7, height = 6)
```

Chr2

```
# Reorder the levels of Country based on the median of Chr1
merged_data <- merged_data %>%
  mutate(Country = fct_reorder(Country, Chr2, .fun = median))

# Plot
ggbetweenstats(
  data = merged_data,
  x = Country,
  y = Chr2,
  title = "Chr2",
  xlab = "Country",
  ylab = "LD Half Distance (kb)",
  ggplot.component = list(theme(legend.position = "none"))
) + my_theme() + theme(legend.position = "none")
```

```
# Save the plot
ggsave(here("output", "populations", "linkage", "decay_chr2_test.pdf"), width = 7, height = 6)
```

Chr3

```
# Reorder the levels of Country based on the median of Chr1
merged_data <- merged_data %>%
  mutate(Country = fct_reorder(Country, Chr3, .fun = median))

# Plot
ggbetweenstats(
  data = merged_data,
  x = Country,
  y = Chr3,
  title = "Chr3",
  xlab = "Country",
  ylab = "LD Half Distance (kb)",
  ggplot.component = list(theme(legend.position = "none"))
) + my_theme() + theme(legend.position = "none")
```

```
# Save the plot
ggsave(here("output", "populations", "linkage", "decay_chr3_test.pdf"), width = 7, height = 6)
```
