# Supplementary material for: A genotyping array for the globally invasive vector mosquito, Aedes albopictus
Source: Parasit Vectors. 2024 Mar 4;17:106. doi: 10.1186/s13071-024-06158-z (PMC10910840; doi:10.1186/s13071-024-06158-z)
Supplement: Supplementary file 9 — Additional file 9. Admixture analysis. [file 13071_2024_6158_MOESM9_ESM.html]

Aedes albopictus SNP chip - Ancetry analysis with Admixture.


# Aedes albopictus SNP chip - Ancetry analysis with Admixture.

#### Luciano V Cosme

#### 2023-08-28

- 1. Load
  the libraries
- 2.
  Admixture runs setup
  - 2.1 Neutral set
  - 2.2 SNPs set
    r2 0.01
  - 2.3 SNPs set r2
    0.1
- 3. Run Admixture
  - 3. Neutral
    set
  - 3.2 r2\_0.01
  - 3.3 r2\_0.1
- 4. Get the cross validation
  values
  - 4.1 Neutral
    set
  - 4.2 r2\_0.01 snp
    set
  - 4.3 r2\_0.1 snp
    set
- 5. Structure
  plots
  - 5.1 Neutral set
    - 5.1.1 Create plot with Q
      matrix
  - 5.2 r2 0.01 set
    - 5.2.1 Create plot with Q
      matrix
  - 5.3 r2 0.1 set
    - 5.3.1 Create plot with Q
      matrix

## 1. Load the libraries

```
library(tidyverse)
library(here)
library(colorout)
library(dplyr)
library(flextable)
library(ggstatsplot)
library(extrafont)
library(stringr)
library(flextable)
library(officer)
```

For Admixture we can load the package in the cluster.

```
get clone https://github.com/NovembreLab/admixture.git

# check it
./admixture/releases/admixture_linux-1.3.0/admixture -h
```

Export path

```
# Add this in your script
export PATH=$PATH:/gpfs/ycga/project/caccone/lvc26/albo_manuscript/admixture/admixture/releases/admixture_linux-1.3.0/

# Then call admixture from anywhere
admixture -h
```

## 2. Admixture runs setup

We created 3 sets of SNPs. One with intergenic SNPs that we called
neutral. Then, one with pruning using r2 of 0.01 and other with r2 of
0.1. They have different number of SNPs but the same 237 samples. ’

```
awk '{print $1}' output/populations/snps_sets/neutral.fam | sort | uniq -c | awk '{print $2}'
```

```
## BEN
## CAM
## CHA
## GEL
## HAI
## HAN
## HOC
## HUN
## INJ
## INW
## JAF
## KAC
## KAG
## KAN
## KAT
## KLP
## KUN
## LAM
## MAT
## OKI
## QNC
## SON
## SSK
## SUF
## SUU
## TAI
## UTS
## YUN
```

Some of these sampling localities are very close to each other but we
will consider to count the total number of sample groups.

```
awk '{print $1}' output/populations/snps_sets/neutral.fam | sort | uniq -c | wc -l
```

```
##       28
```

First we need to test if our command will create the directories we
want. We can use `echo` instead of `mkdir` to test
it.

```
for i in $(seq -w 1 10)
do
  echo run$i
done             # it works
```

Change echo to `mkdir` and create all directories. First
option with seq using -w flag to have leading zeros (check
“`man seq`”, -w, –equal-width).

```
for i in $(seq 1 5)
do
  mkdir run$i
done
```

Next we have to create the script to run Admixture. First, lets test
the loop using k=1 to k=5 and 5 runs for each k. Note that for runs we
use seq -w (we get run01, run02, etc., when we have 10 runs). For K, we
don’t want the leading zeros, so we don’t use the -w flag.

```
for run in $(seq 1 2); do
    for k in $(seq 1 2); do
        echo "run= $run k= $k seed= $run"
    done
done
```

```
## run= 1 k= 1 seed= 1
## run= 1 k= 2 seed= 1
## run= 2 k= 1 seed= 2
## run= 2 k= 2 seed= 2
```

Check the Admixture options.

```
admixture --help
****                   ADMIXTURE Version 1.3.0                  ****
****                    Copyright 2008-2015                     ****
****           David Alexander, Suyash Shringarpure,            ****
****                John  Novembre, Ken Lange                   ****
****                                                            ****
****                 Please cite our paper!                     ****
****   Information at www.genetics.ucla.edu/software/admixture  ****


  ADMIXTURE basic usage:  (see manual for complete reference)
    % admixture [options] inputFile K

  where:
    K is the number of populations; and
    inputFile may be:
      - a PLINK .bed file
      - a PLINK "12" coded .ped file

  Output will be in files inputBasename.K.Q, inputBasename.K.P

  General options:
    -jX          : do computation on X threads
    --seed=X     : use random seed X for initialization

  Algorithm options:
     -m=
    --method=[em|block]     : set method.  block is default

     -a=
    --acceleration=none   |
                   sqs<X> |
                   qn<X>      : set acceleration

  Convergence criteria:
    -C=X : set major convergence criterion (for point estimation)
    -c=x : set minor convergence criterion (for bootstrap and CV reestimates)

  Bootstrap standard errors:
    -B[X]      : do bootstrapping [with X replicates]
```

Generate script for Admixture. We will use 500 bootstraps, and
cv=10.

```
admixture file.bed $name -j10 # we will run with the default settings (200 bootstraps) -j10 means we will run with 10 threads, 200 bootstraps
admixture file.bed $name -j20 --cv=10 --B1000 # we will run with 1000 bootstraps, cross-validation 10, using 20 threads
```

Test loop to make script for `dsq`.

```
# 2000 bootstraps and cv 10
for run in $(seq 1); do
    for k in $(seq 1 2); do
        echo "cd /gpfs/ycga/project/caccone/lvc26/admixture/neutral/run$run; \
export PATH=$PATH:/gpfs/ycga/project/caccone/lvc26/albo_manuscript/admixture/admixture/releases/admixture_linux-1.3.0/; \
admixture -s $run --cv=10 -B2000 -j20 /gpfs/ycga/project/caccone/lvc26/neuroadmix/snps_sets/neutral.bed $k | tee log_k$k.txt"
    done
done | head
```

```
## cd /gpfs/ycga/project/caccone/lvc26/admixture/neutral/run1; export PATH=/Users/lucianocosme/opt/miniconda3/bin:/Users/lucianocosme/opt/miniconda3/condabin:/Users/lucianocosme/opt/miniconda3/bin:/Users/lucianocosme/opt/miniconda3/bin:/usr/local/bin:/System/Cryptexes/App/usr/bin:/usr/bin:/bin:/usr/sbin:/sbin:/Library/TeX/texbin:/usr/local/go/bin:/opt/X11/bin:/Library/Apple/usr/bin:/var/run/com.apple.security.cryptexd/codex.system/bootstrap/usr/local/bin:/var/run/com.apple.security.cryptexd/codex.system/bootstrap/usr/bin:/var/run/com.apple.security.cryptexd/codex.system/bootstrap/usr/appleinternal/bin:/Users/lucianocosme/Applications/quarto/bin:/usr/texbin:/Applications/RStudio.app/Contents/Resources/app/bin/postback:/gpfs/ycga/project/caccone/lvc26/albo_manuscript/admixture/admixture/releases/admixture_linux-1.3.0/; admixture -s 1 --cv=10 -B2000 -j20 /gpfs/ycga/project/caccone/lvc26/neuroadmix/snps_sets/neutral.bed 1 | tee log_k1.txt
## cd /gpfs/ycga/project/caccone/lvc26/admixture/neutral/run1; export PATH=/Users/lucianocosme/opt/miniconda3/bin:/Users/lucianocosme/opt/miniconda3/condabin:/Users/lucianocosme/opt/miniconda3/bin:/Users/lucianocosme/opt/miniconda3/bin:/usr/local/bin:/System/Cryptexes/App/usr/bin:/usr/bin:/bin:/usr/sbin:/sbin:/Library/TeX/texbin:/usr/local/go/bin:/opt/X11/bin:/Library/Apple/usr/bin:/var/run/com.apple.security.cryptexd/codex.system/bootstrap/usr/local/bin:/var/run/com.apple.security.cryptexd/codex.system/bootstrap/usr/bin:/var/run/com.apple.security.cryptexd/codex.system/bootstrap/usr/appleinternal/bin:/Users/lucianocosme/Applications/quarto/bin:/usr/texbin:/Applications/RStudio.app/Contents/Resources/app/bin/postback:/gpfs/ycga/project/caccone/lvc26/albo_manuscript/admixture/admixture/releases/admixture_linux-1.3.0/; admixture -s 1 --cv=10 -B2000 -j20 /gpfs/ycga/project/caccone/lvc26/neuroadmix/snps_sets/neutral.bed 2 | tee log_k2.txt
```

Now we can generate the full script for dsq.

### 2.1 Neutral set

We tested Admixture with several runs for each data set. Admixture
always returned k=5 or k=6 as the number of ancestral populations. We
will do one more run with the same SNP sets we used for all
algorithms.

```
for run in $(seq 1); do
    for k in $(seq 1 25); do
        echo "cd /gpfs/ycga/project/caccone/lvc26/admixture/neutral/run$run; \
export PATH=$PATH:/gpfs/ycga/project/caccone/lvc26/albo_manuscript/admixture/admixture/releases/admixture_linux-1.3.0/; \
admixture -s $run --cv=10 -B1000 -j20 /gpfs/ycga/project/caccone/lvc26/neuroadmix/snps_sets/neutral.bed $k | tee log_k$k.txt"
    done
done > dsq1.txt
```

Generate the dsq batch files on the cluster.

```
module load dSQ/1.05
# make script
# https://docs.ycrc.yale.edu/clusters-at-yale/job-scheduling/dsq/
#
# create dsq file
dsq \
--job-file dsq1.txt \
--output /gpfs/ycga/project/caccone/lvc26/admixture/neutral/logs/admix-%A_%3a-%N.txt \
--mem-per-cpu 1g \
-t 24:00:00 \
--cpus-per-task=10 \
--mail-type ALL \
--job-name admixtureN \
--batch-file admixture.sh \
--partition=scavenge
#
```

Before we submit all jobs, it is a good practice to submit only one
job of the array and see if it runs without problems.

```
# create a directory for the logs
cd /gpfs/ycga/project/caccone/lvc26/admixture/neutral
mkdir logs
#
# submit 1the first job to see if it works.
sbatch --array=0 admixture.sh
# Submitted batch job 6272630
# check status
dsqa -j 6272630  # no errors, we can go ahead and submit all jobs
```

### 2.2 SNPs set r2 0.01

We tested admixture with several runs for each data set. Admixture
always returned k=5 or k=6 as the number of ancestral populations. We
will do one more run with the same SNP sets we used for all
algorithms

```
for run in $(seq 1); do
    for k in $(seq 1 25); do
        echo "cd /gpfs/ycga/project/caccone/lvc26/admixture/r2_0.01/run$run; \
export PATH=$PATH:/gpfs/ycga/project/caccone/lvc26/albo_manuscript/admixture/admixture/releases/admixture_linux-1.3.0/; \
admixture -s $run --cv=10 -B1000 -j20 /gpfs/ycga/project/caccone/lvc26/neuroadmix/snps_sets/r2_0.01.bed $k | tee log_k$k.txt"
    done
done > dsq1.txt
```

Generate the dsq batch files on the cluster.

```
module load dSQ/1.05
# make script
# https://docs.ycrc.yale.edu/clusters-at-yale/job-scheduling/dsq/
#
# create dsq file
dsq \
--job-file dsq1.txt \
--output /gpfs/ycga/project/caccone/lvc26/admixture/r2_0.01/logs/admix-%A_%3a-%N.txt \
--mem-per-cpu 1g \
-t 24:00:00 \
--cpus-per-task=10 \
--mail-type ALL \
--job-name admixture_r2_0.01 \
--batch-file admixture.sh \
--partition=scavenge
#
```

### 2.3 SNPs set r2 0.1

We tested admixture with several runs for each data set. Admixture
always returned k=5 or k=6 as the number of ancestral populations. We
will do one more run with the same SNP sets we used for all
algorithms

```
for run in $(seq 1); do
    for k in $(seq 1 25); do
        echo "cd /gpfs/ycga/project/caccone/lvc26/admixture/r2_0.1/run$run; \
export PATH=$PATH:/gpfs/ycga/project/caccone/lvc26/albo_manuscript/admixture/admixture/releases/admixture_linux-1.3.0/; \
admixture -s $run --cv=10 -B1000 -j20 /gpfs/ycga/project/caccone/lvc26/neuroadmix/snps_sets/r2_0.1.bed $k | tee log_k$k.txt"
    done
done > dsq1.txt
```

Generate the dsq batch files on the cluster.

```
module load dSQ/1.05
# make script
# https://docs.ycrc.yale.edu/clusters-at-yale/job-scheduling/dsq/
#
# create dsq file
dsq \
--job-file dsq1.txt \
--output /gpfs/ycga/project/caccone/lvc26/admixture/r2_0.1/logs/admix-%A_%3a-%N.txt \
--mem-per-cpu 1g \
-t 24:00:00 \
--cpus-per-task=10 \
--mail-type ALL \
--job-name admixture_r2_0.1 \
--batch-file admixture.sh \
--partition=scavenge
#
```

## 3. Run Admixture

### 3. Neutral set

If it runs without problems, we can submit all jobs.

```
sbatch --array=1-24 admixture.sh
# Submitted batch job 6272667
```

Check the jobs status.

```
# check status
dsqa -j 6272667 # all finished
```

### 3.2 r2\_0.01

```
# create a directory for the logs
cd /gpfs/ycga/project/caccone/lvc26/admixture/r2_0.01
mkdir logs

sbatch admixture.sh
# Submitted batch job 6272808
# check status
dsqa -j 6272808
```

### 3.3 r2\_0.1

```
# create a directory for the logs
cd /gpfs/ycga/project/caccone/lvc26/admixture/r2_0.1
mkdir logs

sbatch admixture.sh
# Submitted batch job 6272864
# check status
dsqa -j 6272864  # PREEMPTED      5     11,16,18-20
```

Run autopsy once it is done.

```
dsqa -j 6272864 -f dsq1.txt -s PREEMPTED > rerun_jobs.txt; wc -l rerun_jobs.txt
# re submit the 30 jobs 
dsq \
--job-file rerun_jobs.txt \
--mem-per-cpu 1g \
--cpus-per-task=10 \
--time 120:00:00 \
--mail-type END \
--partition=week \
--job-name admixture2 \
--output /gpfs/ycga/project/caccone/lvc26/admixture/r2_0.1/logs/admix-%A_%3a-%N.txt \
--batch-file admixture2.sh \
--submit #job 6350311

# check status
dsqa -j 6350311
```

## 4. Get the cross validation values

### 4.1 Neutral set

Collect the cross validation information from all the log files. We
have to do it for each run. We need to get the CV and the K from each
log file.

```
# navigate to the data directory
cd /gpfs/ycga/project/caccone/lvc26/admixture/neutral
# let's check the log files using grep. We need to get the line with the CV values. We can grep CV
grep CV run1/log*.txt | head
```

Make loop to get summary

```
cd /gpfs/ycga/project/caccone/lvc26/admixture/neutral
#
for i in $(ls -1 run1/log*.txt); do
    grep -H CV $i | sed 's|run| |' | sed 's|/log_k| |' | sed 's|.txt:CV error (K=| |' | sed 's|):||' | sed 's|_||' | awk '{print $1, $3, $4}'
done > cross_validation_neutral_set.txt
```

Download the data

```
rsync -chavzP --stats lvc26@mccleary.ycrc.yale.edu:/gpfs/ycga/project/caccone/lvc26/admixture/neutral/cross_validation_neutral_set.txt /Users/lucianocosme/Library/CloudStorage/Dropbox/Albopictus/manuscript_chip/data/no_autogenous/albo_chip/output/populations/admixture/neutral
```

Import the cross validation values into R

```
cross_val_default <-
  read_delim(
    here(
      "output", "populations", "admixture", "neutral", "cross_validation_neutral_set.txt"
    ),
    col_names      = FALSE,
    show_col_types = FALSE,
    col_types      = "cin"
  ) |>
  rename(
    run = 1,
    k   = 2,
    cv  = 3
  )
```

Lets make cv plot for the default run

```
cross_val_default |>
  ggplot() +
  geom_line(
    aes(
      x        = k,
      y        = cv
    ),
    linewidth  = .75,
    color = "magenta"
  ) +
  labs(
    x        = "K",
    y        = "Cross-validation error",
    title    = "Admixture Cross-validation neutral SNPs",
    subtitle = "1000 bootstraps and cv = 10 ",
    caption  = "algorithm runs for choices of K ranging from 1 to 25"
  ) +
  hrbrthemes::theme_ipsum(
    base_family      = "",
    axis_text_size   = 12,
    axis_title_size  = 14,
    plot_margin      = margin(10, 10, 10, 10),
    grid             = TRUE,
    grid_col         = "#fabbe2"
  ) +
  theme(
    panel.grid.major = element_line(
      linetype       = "dashed",
      linewidth      = 0.2,
    ),
    legend.title     = element_text(
        size           = 14,
        face           = "bold"
      ),
    panel.grid.minor = element_line(
      linetype       = "dashed",
      linewidth      = 0.2
    )
  )
```

```
#
# save the plot
ggsave(
  here(
    "output", "populations","figures", "admixuture_default_run_k1_k25.pdf"
  ),
  width  = 8,
  height = 6,
  units  = "in"
)
```

Now we can find the K with the lowest cross-validation error

```
cross_val_default |>
  summarize(
    LowestCVerror = min(
      cv,
      na.rm       = TRUE
    )
  ) -> cv_min_default

cross_val_default |>
  slice(
    which.min(
      cv
    )
  ) |>
  select(
    run, k, cv
  ) |>
  rename(
    LowestCVerror = 3
  ) -> k_with_lowest_cv_default


k_with_lowest_cv_default
```

```
## # A tibble: 1 × 3
##   run       k LowestCVerror
##   <chr> <int>         <dbl>
## 1 1         5         0.699
```

k=5

### 4.2 r2\_0.01 snp set

Collect the cross validation information from the all the log files.
We have to do it for each run. We need to get the CV and the K from each
log file.

```
cd /gpfs/ycga/project/caccone/lvc26/admixture/r2_0.01
grep CV run1/log*.txt | head
```

Make loop to get the summary

```
cd /gpfs/ycga/project/caccone/lvc26/admixture/r2_0.01
#
for i in $(ls -1 run1/log*.txt); do
    grep -H CV $i | sed 's|run| |' | sed 's|/log_k| |' | sed 's|.txt:CV error (K=| |' | sed 's|):||' | sed 's|_||' | awk '{print $1, $3, $4}'
done > cross_validation_r2_0.01_set.txt
```

Download the data

```
rsync -chavzP --stats lvc26@mccleary.ycrc.yale.edu:/gpfs/ycga/project/caccone/lvc26/admixture/r2_0.01/cross_validation_r2_0.01_set.txt /Users/lucianocosme/Library/CloudStorage/Dropbox/Albopictus/manuscript_chip/data/no_autogenous/albo_chip/output/populations/admixture/r2_0.01
```

Import the cross validation values into R

```
cross_val_default <-
  read_delim(
    here(
      "output", "populations", "admixture", "r2_0.01", "cross_validation_r2_0.01_set.txt"
    ),
    col_names      = FALSE,
    show_col_types = FALSE,
    col_types      = "cin"
  ) |>
  rename(
    run = 1,
    k   = 2,
    cv  = 3
  )
```

Lets make cv plot for the default run

```
# make plot
cross_val_default |>
  ggplot() +
  geom_line(
    aes(
      x        = k,
      y        = cv
    ),
    linewidth  = .75,
    color = "magenta"
  ) +
  labs(
    x        = "K",
    y        = "Cross-validation error",
    title    = "Admixture Cross-validation r2_0.01 SNPs",
    subtitle = "1000 bootstraps and cv = 10 ",
    caption  = "algorithm runs for choices of K ranging from 1 to 25"
  ) +
  hrbrthemes::theme_ipsum(
    base_family      = "",
    axis_text_size   = 12,
    axis_title_size  = 14,
    plot_margin      = margin(10, 10, 10, 10),
    grid             = TRUE,
    grid_col         = "#fabbe2"
  ) +
  theme(
    panel.grid.major = element_line(
      linetype       = "dashed",
      linewidth      = 0.2,
    ),
    legend.title     = element_text(
        size           = 14,
        face           = "bold"
      ),
    panel.grid.minor = element_line(
      linetype       = "dashed",
      linewidth      = 0.2
    )
  )
```

```
#
# save the plot
ggsave(
  here(
    "output", "populations", "figures", "admixuture_r2_0.01_run_k1_k25.pdf"
  ),
  width  = 8,
  height = 6,
  units  = "in"
)
```

Now we can find the K with the lowest cross-validation error

```
cross_val_default |>
  summarize(
    LowestCVerror = min(
      cv,
      na.rm       = TRUE
    )
  ) -> cv_min_default

cross_val_default |>
  slice(
    which.min(
      cv
    )
  ) |>
  select(
    run, k, cv
  ) |>
  rename(
    LowestCVerror = 3
  ) -> k_with_lowest_cv_default


k_with_lowest_cv_default
```

```
## # A tibble: 1 × 3
##   run       k LowestCVerror
##   <chr> <int>         <dbl>
## 1 1         5         0.746
```

k=5

### 4.3 r2\_0.1 snp set

Collect the cross validation information from the all the log files.
We have to do it for each run. We need to get the CV and the K from each
log file.

```
cd /gpfs/ycga/project/caccone/lvc26/admixture/r2_0.1
grep CV run1/log*.txt | head
```

Make loop to get the summary

```
cd /gpfs/ycga/project/caccone/lvc26/admixture/r2_0.1
#
for i in $(ls -1 run1/log*.txt); do
    grep -H CV $i | sed 's|run| |' | sed 's|/log_k| |' | sed 's|.txt:CV error (K=| |' | sed 's|):||' | sed 's|_||' | awk '{print $1, $3, $4}'
done > cross_validation_r2_0.1_set.txt
```

Download the data

```
rsync -chavzP --stats lvc26@mccleary.ycrc.yale.edu:/gpfs/ycga/project/caccone/lvc26/admixture/r2_0.1/cross_validation_r2_0.1_set.txt /Users/lucianocosme/Library/CloudStorage/Dropbox/Albopictus/manuscript_chip/data/no_autogenous/albo_chip/output/populations/admixture/r2_0.1
```

Import the cross validation values into R

```
cross_val_default <-
  read_delim(
    here(
      "output", "populations", "admixture", "r2_0.1", "cross_validation_r2_0.1_set.txt"
    ),
    col_names      = FALSE,
    show_col_types = FALSE,
    col_types      = "cin"
  ) |>
  rename(
    run = 1,
    k   = 2,
    cv  = 3
  )
```

Lets make cv plot for the default run

```
# make plot
cross_val_default |>
  ggplot() +
  geom_line(
    aes(
      x        = k,
      y        = cv
    ),
    linewidth  = .75,
    color = "magenta"
  ) +
  labs(
    x        = "K",
    y        = "Cross-validation error",
    title    = "Admixture Cross-validation r2_0.01 SNPs",
    subtitle = "1000 bootstraps and cv = 10 ",
    caption  = "algorithm runs for choices of K ranging from 1 to 25"
  ) +
  hrbrthemes::theme_ipsum(
    base_family      = "",
    axis_text_size   = 12,
    axis_title_size  = 14,
    plot_margin      = margin(10, 10, 10, 10),
    grid             = TRUE,
    grid_col         = "#fabbe2"
  ) +
  theme(
    panel.grid.major = element_line(
      linetype       = "dashed",
      linewidth      = 0.2,
    ),
    legend.title     = element_text(
        size           = 14,
        face           = "bold"
      ),
    panel.grid.minor = element_line(
      linetype       = "dashed",
      linewidth      = 0.2
    )
  )
```

```
#
# save the plot
ggsave(
  here(
    "output", "populations", "figures", "admixuture_r2_0.1_run_k1_k25.pdf"
  ),
  width  = 8,
  height = 6,
  units  = "in"
)
```

Now we can find the K with the lowest cross-validation error

```
cross_val_default |>
  summarize(
    LowestCVerror = min(
      cv,
      na.rm       = TRUE
    )
  ) -> cv_min_default

cross_val_default |>
  slice(
    which.min(
      cv
    )
  ) |>
  dplyr::select(
    run, k, cv
  ) |>
  rename(
    LowestCVerror = 3
  ) -> k_with_lowest_cv_default


k_with_lowest_cv_default
```

```
## # A tibble: 1 × 3
##   run       k LowestCVerror
##   <chr> <int>         <dbl>
## 1 1         5         0.676
```

k=5

## 5. Structure plots

### 5.1 Neutral set

Download the data

```
rsync -chavzP --stats --include='*/' --include='*.Q' --exclude='*'  lvc26@mccleary.ycrc.yale.edu:/gpfs/ycga/project/caccone/lvc26/admixture/neutral/* /Users/lucianocosme/Library/CloudStorage/Dropbox/Albopictus/manuscript_chip/data/no_autogenous/albo_chip/output/populations/admixture/neutral
```

Check the downloaded data

```
ls -1 output/populations/admixture/neutral/run1 | tail
```

#### 5.1.1 Create plot with Q matrix

Make plot

```
# Extract ancestry coefficients
k5run1 <- read_delim(
  here("output", "populations", "admixture", "neutral", "run1", "neutral.5.Q"),
  delim = " ", # Specify the delimiter if different from the default (comma)
  col_names = FALSE,
  show_col_types = FALSE
) 

head(k5run1)
```

```
## # A tibble: 6 × 5
##      X1    X2      X3      X4    X5
##   <dbl> <dbl>   <dbl>   <dbl> <dbl>
## 1 0.349 0.251 0.0197  0.0318  0.349
## 2 0.314 0.252 0.0244  0.00540 0.405
## 3 0.321 0.225 0.0554  0.00001 0.399
## 4 0.314 0.235 0.0173  0.0558  0.379
## 5 0.300 0.244 0.00001 0.0461  0.410
## 6 0.317 0.226 0.0539  0.0105  0.393
```

The fam file

```
fam_file <- here(
  "output", "populations", "snps_sets", "neutral.fam"
)

# Read the .fam file
fam_data <- read.table(fam_file, 
                       header = FALSE,
                       col.names = c("FamilyID", "IndividualID", "PaternalID", "MaternalID", "Sex", "Phenotype"))

# View the first few rows
head(fam_data)
```

```
##   FamilyID IndividualID PaternalID MaternalID Sex Phenotype
## 1      OKI         1001          0          0   2        -9
## 2      OKI         1002          0          0   2        -9
## 3      OKI         1003          0          0   2        -9
## 4      OKI         1004          0          0   2        -9
## 5      OKI         1005          0          0   2        -9
## 6      OKI         1006          0          0   1        -9
```

Create ID column

```
# Change column name
colnames(fam_data)[colnames(fam_data) == "IndividualID"] <- "ind"


# Merge columns "FamilyID" and "IndividualID" with an underscore
# fam_data$ind <- paste(fam_data$FamilyID, fam_data$IndividualID, sep = "_")


# Change column name
colnames(fam_data)[colnames(fam_data) == "FamilyID"] <- "pop"

# Select ID
fam_data <- fam_data |>
  dplyr::select("ind", "pop")

# View the first few rows
head(fam_data)
```

```
##    ind pop
## 1 1001 OKI
## 2 1002 OKI
## 3 1003 OKI
## 4 1004 OKI
## 5 1005 OKI
## 6 1006 OKI
```

Add it to matrix

```
k5run1 <- fam_data |>
  dplyr::select(ind, pop) |>
  bind_cols(k5run1)

head(k5run1)
```

```
##    ind pop       X1       X2       X3       X4       X5
## 1 1001 OKI 0.348771 0.250551 0.019674 0.031750 0.349254
## 2 1002 OKI 0.313650 0.251651 0.024403 0.005396 0.404899
## 3 1003 OKI 0.321265 0.224595 0.055417 0.000010 0.398712
## 4 1004 OKI 0.313674 0.234637 0.017323 0.055846 0.378520
## 5 1005 OKI 0.299965 0.244414 0.000010 0.046103 0.409507
## 6 1006 OKI 0.317372 0.225772 0.053897 0.010453 0.392506
```

Rename the columns

```
# Rename the columns starting from the third one
k5run1 <- k5run1 |>
  rename_with(~paste0("v", seq_along(.x)), .cols = -c(ind, pop))

# View the first few rows
head(k5run1)
```

```
##    ind pop       v1       v2       v3       v4       v5
## 1 1001 OKI 0.348771 0.250551 0.019674 0.031750 0.349254
## 2 1002 OKI 0.313650 0.251651 0.024403 0.005396 0.404899
## 3 1003 OKI 0.321265 0.224595 0.055417 0.000010 0.398712
## 4 1004 OKI 0.313674 0.234637 0.017323 0.055846 0.378520
## 5 1005 OKI 0.299965 0.244414 0.000010 0.046103 0.409507
## 6 1006 OKI 0.317372 0.225772 0.053897 0.010453 0.392506
```

Import sample locations

```
sampling_loc <- readRDS(here("output", "populations", "sampling_loc.rds"))
head(sampling_loc)
```

```
## # A tibble: 6 × 6
##   Pop_City   Country  Latitude Longitude Region Abbreviation
##   <chr>      <chr>       <dbl>     <dbl> <chr>  <chr>       
## 1 Gelephu    Bhutan       26.9      90.5 Asia   GEL         
## 2 Phnom Penh Cambodia     11.6     105.  Asia   CAM         
## 3 Hainan     China        19.2     110.  Asia   HAI         
## 4 Yunnan     China        24.5     101.  Asia   YUN         
## 5 Hunan      China        27.6     112.  Asia   HUN         
## 6 Bengaluru  India        13.0      77.6 Asia   BEN
```

```
source(
  here(
    "scripts", "analysis", "my_theme3.R"
  )
)

# Create a named vector to map countries to regions
country_to_region <- c(
  "Bhutan" = "South Asia",
  "Cambodia" = "Southeast Asia",
  "China" = "East Asia",
  "India" = "South Asia",
  "Indonesia" = "Southeast Asia",
  "Japan" = "East Asia",
  "Malaysia" = "Southeast Asia",
  "Maldives" = "South Asia",
  "Nepal" = "South Asia",
  "Sri Lanka" = "South Asia",
  "Taiwan" = "East Asia",
  "Thailand" = "Southeast Asia",
  "Vietnam" = "Southeast Asia"
)

# Add the region to the data frame
sampling_loc$Region2 <- country_to_region[sampling_loc$Country]

# Melt the data frame for plotting
Q_melted <- k5run1 |>
  pivot_longer(
    cols = -c(ind, pop),
    names_to = "variable",
    values_to = "value"
  )
# Join with sampling_loc to get sampling localities
Q_joined <- Q_melted |>
  left_join(sampling_loc, by = c("pop" = "Abbreviation"))

# Create a combined variable for Region and Country
Q_joined <- Q_joined |>
  mutate(Region_Country = interaction(Region, Country, sep = "_"))

# Order the combined variable by Region and Country, then by individual
Q_ordered <- Q_joined |>
  arrange(Region, Region2, Country, ind) |>
  mutate(ind = factor(ind, levels = unique(ind)))  # Convert ind to a factor with levels in the desired order

# Add labels: country names for the first individual in each country, NA for all other individuals
Q_ordered <- Q_ordered |>
  group_by(Region_Country) |>
  mutate(label = ifelse(row_number() == 1, as.character(Country), NA))

# Group by individual and variable, calculate mean ancestry proportions
Q_grouped <- Q_ordered |>
  group_by(ind, variable) |>
  summarise(value = mean(value), .groups = "drop")

# Create a data frame for borders
borders <-
  data.frame(Region_Country = unique(Q_ordered$Region_Country))

# Add the order of the last individual of each country to ensure correct placement of borders
borders$order <-
  sapply(borders$Region_Country, function(rc)
    max(which(Q_ordered$Region_Country == rc))) + 0.5  # Shift borders to the right edge of the bars

# Select only the first occurrence of each country in the ordered data
label_df <- Q_ordered |>
  filter(!is.na(label)) |>
  distinct(label, .keep_all = TRUE)

# Create a custom label function
label_func <- function(x) {
  labels <- rep("", length(x))
  labels[x %in% label_df$ind] <- label_df$label
  labels
}

# Calculate the position of lines
border_positions <- Q_ordered |>
  group_by(Country) |>
  summarise(pos = max(as.numeric(ind)) + 0)

# Calculate the position of population labels and bars
pop_labels <- Q_ordered |>
  mutate(Name = paste(pop, Pop_City, sep = " - ")) |>
  group_by(pop) |>
  slice_head(n = 1) |>
  ungroup() |>
  dplyr::select(ind, Pop_City, Country, Name) |>
  mutate(pos = as.numeric(ind))  # calculate position of population labels

pop_labels_bars <- pop_labels |>
  mutate(pos = as.numeric(ind)  - .5)


# Calculate the position of lines
border_positions <- Q_ordered |>
  group_by(Country) |>
  summarise(pos = max(as.numeric(ind)) - 1)


pop_labels_bars <- pop_labels |>
  mutate(pos = as.numeric(ind)  - .5)

# Function to filter and normalize data
normalize_data <- function(df, min_value) {
  df |>
    filter(value > min_value) |>
    group_by(ind) |>
    mutate(value = value / sum(value))
}

# Use the function
Q_grouped_filtered <- normalize_data(Q_grouped, 0.1)
# 

color_palette <-
  c(
    "v1" = "#FFB347",
    "v2" = "#F49AC2",
    "v3" = "red",
    "v4" = "#FFFF99",
    "v5" = "#AE9393"
  )
# Generate all potential variable names
all_variables <- paste0("v", 1:5)

# Map each variable to a name
color_mapping <- data.frame(variable = all_variables,
                            color = names(color_palette))

# Merge with Q_grouped_filtered
Q_grouped_filtered <- merge(Q_grouped_filtered, color_mapping, by = "variable")

# Create the plot
ggplot(Q_grouped_filtered, aes(x = as.factor(ind), y = value, fill = color)) +
  geom_bar(stat = 'identity', width = 1) +
  geom_vline(
    data = pop_labels_bars,
    aes(xintercept = pos),
    color = "#2C444A",
    linewidth = .2
  ) +
  geom_text(
    data = pop_labels,
    aes(x = as.numeric(ind), y = 1, label = Name),
    vjust = 1.5,
    hjust = 0,
    size = 2,
    angle = 90,
    inherit.aes = FALSE
  ) +
  my_theme() +
  theme(
    axis.text.x = element_text(
      angle = 90,
      hjust = 1,
      size = 12
    ),
    legend.position = "none",
    plot.margin = unit(c(3, 0.5, 0.5, 0.5), "cm")
  ) +
  xlab("Admixture matrix") +
  ylab("Ancestry proportions") +
  labs(caption = "Each bar represents the ancestry proportions for an individual for k=5.\n Admixture for k1:25 with 9,047 SNPs.") +
  scale_x_discrete(labels = label_func) +
  scale_fill_manual(values = color_palette) +
  expand_limits(y = c(0, 1.5))
```

```
# # save it
ggsave(
  here("output", "populations", "figures", "admixture_neutral_k5.pdf"),
  width  = 12,
  height = 6,
  units  = "in",
  device = cairo_pdf
)
```

### 5.2 r2 0.01 set

Download the data

```
rsync -chavzP --stats --include='*/' --include='*.Q' --exclude='*'  lvc26@mccleary.ycrc.yale.edu:/gpfs/ycga/project/caccone/lvc26/admixture/r2_0.01/* /Users/lucianocosme/Library/CloudStorage/Dropbox/Albopictus/manuscript_chip/data/no_autogenous/albo_chip/output/populations/admixture/r2_0.01
```

Check the downloaded data

```
ls -1 output/populations/admixture/r2_0.01/run1 | tail
```

```
## r2_0.01.23.Q
## r2_0.01.24.Q
## r2_0.01.25.Q
## r2_0.01.3.Q
## r2_0.01.4.Q
## r2_0.01.5.Q
## r2_0.01.6.Q
## r2_0.01.7.Q
## r2_0.01.8.Q
## r2_0.01.9.Q
```

#### 5.2.1 Create plot with Q matrix

Make plot

```
# Extract ancestry coefficients
k5run1 <- read_delim(
  here("output", "populations", "admixture", "r2_0.01", "run1", "r2_0.01.5.Q"),
  delim = " ", # Specify the delimiter if different from the default (comma)
  col_names = FALSE,
  show_col_types = FALSE
) 

head(k5run1)
```

```
## # A tibble: 6 × 5
##      X1      X2      X3     X4    X5
##   <dbl>   <dbl>   <dbl>  <dbl> <dbl>
## 1 0.317 0.0707  0.00016 0.0522 0.560
## 2 0.279 0.0959  0.0108  0.0445 0.570
## 3 0.249 0.0636  0.0118  0.0126 0.663
## 4 0.243 0.0109  0.00547 0.0332 0.707
## 5 0.232 0.00151 0.00001 0.0344 0.732
## 6 0.250 0.0409  0.0151  0.0381 0.656
```

The fam file

```
fam_file <- here(
  "output", "populations", "snps_sets", "r2_0.01.fam"
)

# Read the .fam file
fam_data <- read.table(fam_file, 
                       header = FALSE,
                       col.names = c("FamilyID", "IndividualID", "PaternalID", "MaternalID", "Sex", "Phenotype"))

# View the first few rows
head(fam_data)
```

```
##   FamilyID IndividualID PaternalID MaternalID Sex Phenotype
## 1      OKI         1001          0          0   2        -9
## 2      OKI         1002          0          0   2        -9
## 3      OKI         1003          0          0   2        -9
## 4      OKI         1004          0          0   2        -9
## 5      OKI         1005          0          0   2        -9
## 6      OKI         1006          0          0   1        -9
```

Create ID column

```
# Change column name
colnames(fam_data)[colnames(fam_data) == "IndividualID"] <- "ind"

# Change column name
colnames(fam_data)[colnames(fam_data) == "FamilyID"] <- "pop"

# Select ID
fam_data <- fam_data |>
  dplyr::select("ind", "pop")

# View the first few rows
head(fam_data)
```

```
##    ind pop
## 1 1001 OKI
## 2 1002 OKI
## 3 1003 OKI
## 4 1004 OKI
## 5 1005 OKI
## 6 1006 OKI
```

Add it to matrix

```
k5run1 <- fam_data |>
  dplyr::select(ind, pop) |>
  bind_cols(k5run1)

head(k5run1)
```

```
##    ind pop       X1       X2       X3       X4       X5
## 1 1001 OKI 0.317249 0.070658 0.000160 0.052193 0.559741
## 2 1002 OKI 0.279230 0.095881 0.010824 0.044486 0.569579
## 3 1003 OKI 0.248873 0.063590 0.011806 0.012550 0.663181
## 4 1004 OKI 0.243349 0.010864 0.005469 0.033171 0.707148
## 5 1005 OKI 0.232079 0.001506 0.000010 0.034445 0.731960
## 6 1006 OKI 0.249958 0.040899 0.015095 0.038120 0.655927
```

Rename the columns

```
# Rename the columns starting from the third one
k5run1 <- k5run1 |>
  rename_with(~paste0("v", seq_along(.x)), .cols = -c(ind, pop))

# View the first few rows
head(k5run1)
```

```
##    ind pop       v1       v2       v3       v4       v5
## 1 1001 OKI 0.317249 0.070658 0.000160 0.052193 0.559741
## 2 1002 OKI 0.279230 0.095881 0.010824 0.044486 0.569579
## 3 1003 OKI 0.248873 0.063590 0.011806 0.012550 0.663181
## 4 1004 OKI 0.243349 0.010864 0.005469 0.033171 0.707148
## 5 1005 OKI 0.232079 0.001506 0.000010 0.034445 0.731960
## 6 1006 OKI 0.249958 0.040899 0.015095 0.038120 0.655927
```

Import sample locations

```
sampling_loc <- readRDS(here("output", "populations", "sampling_loc.rds"))
head(sampling_loc)
```

```
## # A tibble: 6 × 6
##   Pop_City   Country  Latitude Longitude Region Abbreviation
##   <chr>      <chr>       <dbl>     <dbl> <chr>  <chr>       
## 1 Gelephu    Bhutan       26.9      90.5 Asia   GEL         
## 2 Phnom Penh Cambodia     11.6     105.  Asia   CAM         
## 3 Hainan     China        19.2     110.  Asia   HAI         
## 4 Yunnan     China        24.5     101.  Asia   YUN         
## 5 Hunan      China        27.6     112.  Asia   HUN         
## 6 Bengaluru  India        13.0      77.6 Asia   BEN
```

```
source(
  here(
    "scripts", "analysis", "my_theme3.R"
  )
)

# Create a named vector to map countries to regions
country_to_region <- c(
  "Bhutan" = "South Asia",
  "Cambodia" = "Southeast Asia",
  "China" = "East Asia",
  "India" = "South Asia",
  "Indonesia" = "Southeast Asia",
  "Japan" = "East Asia",
  "Malaysia" = "Southeast Asia",
  "Maldives" = "South Asia",
  "Nepal" = "South Asia",
  "Sri Lanka" = "South Asia",
  "Taiwan" = "East Asia",
  "Thailand" = "Southeast Asia",
  "Vietnam" = "Southeast Asia"
)

# Add the region to the data frame
sampling_loc$Region2 <- country_to_region[sampling_loc$Country]

# Melt the data frame for plotting
Q_melted <- k5run1 |>
  pivot_longer(
    cols = -c(ind, pop),
    names_to = "variable",
    values_to = "value"
  )
# Join with sampling_loc to get sampling localities
Q_joined <- Q_melted |>
  left_join(sampling_loc, by = c("pop" = "Abbreviation"))

# Create a combined variable for Region and Country
Q_joined <- Q_joined |>
  mutate(Region_Country = interaction(Region, Country, sep = "_"))

# Order the combined variable by Region and Country, then by individual
Q_ordered <- Q_joined |>
  arrange(Region, Region2, Country, ind) |>
  mutate(ind = factor(ind, levels = unique(ind)))  # Convert ind to a factor with levels in the desired order

# Add labels: country names for the first individual in each country, NA for all other individuals
Q_ordered <- Q_ordered |>
  group_by(Region_Country) |>
  mutate(label = ifelse(row_number() == 1, as.character(Country), NA))

# Group by individual and variable, calculate mean ancestry proportions
Q_grouped <- Q_ordered |>
  group_by(ind, variable) |>
  summarise(value = mean(value), .groups = "drop")

# Create a data frame for borders
borders <-
  data.frame(Region_Country = unique(Q_ordered$Region_Country))

# Add the order of the last individual of each country to ensure correct placement of borders
borders$order <-
  sapply(borders$Region_Country, function(rc)
    max(which(Q_ordered$Region_Country == rc))) + 0.5  # Shift borders to the right edge of the bars

# Select only the first occurrence of each country in the ordered data
label_df <- Q_ordered |>
  filter(!is.na(label)) |>
  distinct(label, .keep_all = TRUE)

# Create a custom label function
label_func <- function(x) {
  labels <- rep("", length(x))
  labels[x %in% label_df$ind] <- label_df$label
  labels
}

# Calculate the position of lines
border_positions <- Q_ordered |>
  group_by(Country) |>
  summarise(pos = max(as.numeric(ind)) + 0)

# Calculate the position of population labels and bars
pop_labels <- Q_ordered |>
  mutate(Name = paste(pop, Pop_City, sep = " - ")) |>
  group_by(pop) |>
  slice_head(n = 1) |>
  ungroup() |>
  dplyr::select(ind, Pop_City, Country, Name) |>
  mutate(pos = as.numeric(ind))  # calculate position of population labels

pop_labels_bars <- pop_labels |>
  mutate(pos = as.numeric(ind)  - .5)


# Calculate the position of lines
border_positions <- Q_ordered |>
  group_by(Country) |>
  summarise(pos = max(as.numeric(ind)) - 1)


pop_labels_bars <- pop_labels |>
  mutate(pos = as.numeric(ind)  - .5)

# Function to filter and normalize data
normalize_data <- function(df, min_value) {
  df |>
    filter(value > min_value) |>
    group_by(ind) |>
    mutate(value = value / sum(value))
}

# Use the function
Q_grouped_filtered <- normalize_data(Q_grouped, 0.1)
# 

color_palette <-
  c(
    "v1" = "#FFB347",
    "v2" = "#F49AC2",
    "v3" = "red",
    "v4" = "#FFFF99",
    "v5" = "#AE9393"
  )
# Generate all potential variable names
all_variables <- paste0("v", 1:5)

# Map each variable to a name
color_mapping <- data.frame(variable = all_variables,
                            color = names(color_palette))

# Merge with Q_grouped_filtered
Q_grouped_filtered <- merge(Q_grouped_filtered, color_mapping, by = "variable")

# Create the plot
ggplot(Q_grouped_filtered, aes(x = as.factor(ind), y = value, fill = color)) +
  geom_bar(stat = 'identity', width = 1) +
  geom_vline(
    data = pop_labels_bars,
    aes(xintercept = pos),
    color = "#2C444A",
    linewidth = .2
  ) +
  geom_text(
    data = pop_labels,
    aes(x = as.numeric(ind), y = 1, label = Name),
    vjust = 1.5,
    hjust = 0,
    size = 2,
    angle = 90,
    inherit.aes = FALSE
  ) +
  my_theme() +
  theme(
    axis.text.x = element_text(
      angle = 90,
      hjust = 1,
      size = 12
    ),
    legend.position = "none",
    plot.margin = unit(c(3, 0.5, 0.5, 0.5), "cm")
  ) +
  xlab("Admixture matrix") +
  ylab("Ancestry proportions") +
  labs(caption = "Each bar represents the ancestry proportions for an individual for k=5.\n Admixture for k1:25 with 20,931 SNPs.") +
  scale_x_discrete(labels = label_func) +
  scale_fill_manual(values = color_palette) +
  expand_limits(y = c(0, 1.5))
```

```
# # save it
ggsave(
  here("output", "populations", "figures", "admixture_r2_0.01_k5.pdf"),
  width  = 12,
  height = 6,
  units  = "in",
  device = cairo_pdf
)
```

### 5.3 r2 0.1 set

Download the data

```
rsync -chavzP --stats --include='*/' --include='*.Q' --exclude='*'  lvc26@mccleary.ycrc.yale.edu:/gpfs/ycga/project/caccone/lvc26/admixture/r2_0.1/* /Users/lucianocosme/Library/CloudStorage/Dropbox/Albopictus/manuscript_chip/data/no_autogenous/albo_chip/output/populations/admixture/r2_0.1
```

Check the downloaded data

```
ls -1 output/populations/admixture/r2_0.1/run1 | tail
```

#### 5.3.1 Create plot with Q matrix

Make plot

```
# Extract ancestry coefficients
k5run1 <- read_delim(
  here("output", "populations", "admixture", "r2_0.1", "run1", "r2_0.1.5.Q"),
  delim = " ", # Specify the delimiter if different from the default (comma)
  col_names = FALSE,
  show_col_types = FALSE
) 

head(k5run1)
```

```
## # A tibble: 6 × 5
##      X1      X2      X3      X4    X5
##   <dbl>   <dbl>   <dbl>   <dbl> <dbl>
## 1 0.248 0.129   0.00001 0.00912 0.614
## 2 0.220 0.145   0.00001 0.0184  0.616
## 3 0.181 0.0975  0.00001 0.00001 0.722
## 4 0.147 0.0112  0.00001 0.00516 0.837
## 5 0.105 0.00001 0.00001 0.00001 0.895
## 6 0.171 0.0981  0.00001 0.00001 0.731
```

The fam file

```
fam_file <- here("output", "populations", "snps_sets", "r2_0.1.fam")

# Read the .fam file
fam_data <- read.table(
  fam_file,
  header = FALSE,
  col.names = c(
    "FamilyID",
    "IndividualID",
    "PaternalID",
    "MaternalID",
    "Sex",
    "Phenotype"
  )
)

# View the first few rows
head(fam_data)
```

```
##   FamilyID IndividualID PaternalID MaternalID Sex Phenotype
## 1      OKI         1001          0          0   2        -9
## 2      OKI         1002          0          0   2        -9
## 3      OKI         1003          0          0   2        -9
## 4      OKI         1004          0          0   2        -9
## 5      OKI         1005          0          0   2        -9
## 6      OKI         1006          0          0   1        -9
```

Create ID column

```
# Change column name
colnames(fam_data)[colnames(fam_data) == "IndividualID"] <- "ind"

# Change column name
colnames(fam_data)[colnames(fam_data) == "FamilyID"] <- "pop"

# Select ID
fam_data <- fam_data |>
  dplyr::select("ind", "pop")

# View the first few rows
head(fam_data)
```

```
##    ind pop
## 1 1001 OKI
## 2 1002 OKI
## 3 1003 OKI
## 4 1004 OKI
## 5 1005 OKI
## 6 1006 OKI
```

Add it to matrix

```
k5run1 <- fam_data |>
  dplyr::select(ind, pop) |>
  bind_cols(k5run1)

head(k5run1)
```

```
##    ind pop       X1       X2    X3       X4       X5
## 1 1001 OKI 0.247788 0.128696 1e-05 0.009116 0.614391
## 2 1002 OKI 0.220143 0.145400 1e-05 0.018377 0.616069
## 3 1003 OKI 0.180728 0.097481 1e-05 0.000010 0.721772
## 4 1004 OKI 0.146729 0.011211 1e-05 0.005160 0.836890
## 5 1005 OKI 0.105331 0.000010 1e-05 0.000010 0.894639
## 6 1006 OKI 0.171413 0.098054 1e-05 0.000010 0.730513
```

Rename the columns

```
# Rename the columns starting from the third one
k5run1 <- k5run1 |>
  rename_with(~paste0("v", seq_along(.x)), .cols = -c(ind, pop))

# View the first few rows
head(k5run1)
```

```
##    ind pop       v1       v2    v3       v4       v5
## 1 1001 OKI 0.247788 0.128696 1e-05 0.009116 0.614391
## 2 1002 OKI 0.220143 0.145400 1e-05 0.018377 0.616069
## 3 1003 OKI 0.180728 0.097481 1e-05 0.000010 0.721772
## 4 1004 OKI 0.146729 0.011211 1e-05 0.005160 0.836890
## 5 1005 OKI 0.105331 0.000010 1e-05 0.000010 0.894639
## 6 1006 OKI 0.171413 0.098054 1e-05 0.000010 0.730513
```

Import sample locations

```
sampling_loc <- readRDS(here("output", "populations", "sampling_loc.rds"))
head(sampling_loc)
```

```
## # A tibble: 6 × 6
##   Pop_City   Country  Latitude Longitude Region Abbreviation
##   <chr>      <chr>       <dbl>     <dbl> <chr>  <chr>       
## 1 Gelephu    Bhutan       26.9      90.5 Asia   GEL         
## 2 Phnom Penh Cambodia     11.6     105.  Asia   CAM         
## 3 Hainan     China        19.2     110.  Asia   HAI         
## 4 Yunnan     China        24.5     101.  Asia   YUN         
## 5 Hunan      China        27.6     112.  Asia   HUN         
## 6 Bengaluru  India        13.0      77.6 Asia   BEN
```

```
source(
  here(
    "scripts", "analysis", "my_theme3.R"
  )
)

# Create a named vector to map countries to regions
country_to_region <- c(
  "Bhutan" = "South Asia",
  "Cambodia" = "Southeast Asia",
  "China" = "East Asia",
  "India" = "South Asia",
  "Indonesia" = "Southeast Asia",
  "Japan" = "East Asia",
  "Malaysia" = "Southeast Asia",
  "Maldives" = "South Asia",
  "Nepal" = "South Asia",
  "Sri Lanka" = "South Asia",
  "Taiwan" = "East Asia",
  "Thailand" = "Southeast Asia",
  "Vietnam" = "Southeast Asia"
)

# Add the region to the data frame
sampling_loc$Region2 <- country_to_region[sampling_loc$Country]

# Melt the data frame for plotting
Q_melted <- k5run1 |>
  pivot_longer(
    cols = -c(ind, pop),
    names_to = "variable",
    values_to = "value"
  )
# Join with sampling_loc to get sampling localities
Q_joined <- Q_melted |>
  left_join(sampling_loc, by = c("pop" = "Abbreviation"))

# Create a combined variable for Region and Country
Q_joined <- Q_joined |>
  mutate(Region_Country = interaction(Region, Country, sep = "_"))

# Order the combined variable by Region and Country, then by individual
Q_ordered <- Q_joined |>
  arrange(Region, Region2, Country, ind) |>
  mutate(ind = factor(ind, levels = unique(ind)))  # Convert ind to a factor with levels in the desired order

# Add labels: country names for the first individual in each country, NA for all other individuals
Q_ordered <- Q_ordered |>
  group_by(Region_Country) |>
  mutate(label = ifelse(row_number() == 1, as.character(Country), NA))

# Group by individual and variable, calculate mean ancestry proportions
Q_grouped <- Q_ordered |>
  group_by(ind, variable) |>
  summarise(value = mean(value), .groups = "drop")

# Create a data frame for borders
borders <-
  data.frame(Region_Country = unique(Q_ordered$Region_Country))

# Add the order of the last individual of each country to ensure correct placement of borders
borders$order <-
  sapply(borders$Region_Country, function(rc)
    max(which(Q_ordered$Region_Country == rc))) + 0.5  # Shift borders to the right edge of the bars

# Select only the first occurrence of each country in the ordered data
label_df <- Q_ordered |>
  filter(!is.na(label)) |>
  distinct(label, .keep_all = TRUE)

# Create a custom label function
label_func <- function(x) {
  labels <- rep("", length(x))
  labels[x %in% label_df$ind] <- label_df$label
  labels
}

# Calculate the position of lines
border_positions <- Q_ordered |>
  group_by(Country) |>
  summarise(pos = max(as.numeric(ind)) + 0)

# Calculate the position of population labels and bars
pop_labels <- Q_ordered |>
  mutate(Name = paste(pop, Pop_City, sep = " - ")) |>
  group_by(pop) |>
  slice_head(n = 1) |>
  ungroup() |>
  dplyr::select(ind, Pop_City, Country, Name) |>
  mutate(pos = as.numeric(ind))  # calculate position of population labels

pop_labels_bars <- pop_labels |>
  mutate(pos = as.numeric(ind)  - .5)


# Calculate the position of lines
border_positions <- Q_ordered |>
  group_by(Country) |>
  summarise(pos = max(as.numeric(ind)) - 1)


pop_labels_bars <- pop_labels |>
  mutate(pos = as.numeric(ind)  - .5)

# Function to filter and normalize data
normalize_data <- function(df, min_value) {
  df |>
    filter(value > min_value) |>
    group_by(ind) |>
    mutate(value = value / sum(value))
}

# Use the function
Q_grouped_filtered <- normalize_data(Q_grouped, 0.1)
# 

color_palette <-
  c(
    "v1" = "#FFB347",
    "v2" = "#F49AC2",
    "v3" = "red",
    "v4" = "#FFFF99",
    "v5" = "#AE9393"
  )

# Generate all potential variable names
all_variables <- paste0("v", 1:5)

# Map each variable to a name
color_mapping <- data.frame(variable = all_variables,
                            color = names(color_palette))

# Merge with Q_grouped_filtered
Q_grouped_filtered <- merge(Q_grouped_filtered, color_mapping, by = "variable")

# Create the plot
ggplot(Q_grouped_filtered, aes(x = as.factor(ind), y = value, fill = color)) +
  geom_bar(stat = 'identity', width = 1) +
  geom_vline(
    data = pop_labels_bars,
    aes(xintercept = pos),
    color = "#2C444A",
    linewidth = .2
  ) +
  geom_text(
    data = pop_labels,
    aes(x = as.numeric(ind), y = 1, label = Name),
    vjust = 1.5,
    hjust = 0,
    size = 2,
    angle = 90,
    inherit.aes = FALSE
  ) +
  my_theme() +
  theme(
    axis.text.x = element_text(
      angle = 90,
      hjust = 1,
      size = 12
    ),
    legend.position = "none",
    plot.margin = unit(c(3, 0.5, 0.5, 0.5), "cm")
  ) +
  xlab("Admixture matrix") +
  ylab("Ancestry proportions") +
  labs(caption = "Each bar represents the ancestry proportions for an individual for k=5.\n Admixture for k1:25 with 57,780 SNPs.") +
  scale_x_discrete(labels = label_func) +
  scale_fill_manual(values = color_palette) +
  expand_limits(y = c(0, 1.5))
```

```
# # save it
ggsave(
  here("output", "populations", "figures", "admixture_r2_0.1_k5.pdf"),
  width  = 12,
  height = 6,
  units  = "in",
  device = cairo_pdf
)
```
