# Supplementary material for: A genotyping array for the globally invasive vector mosquito, Aedes albopictus
Source: Parasit Vectors. 2024 Mar 4;17:106. doi: 10.1186/s13071-024-06158-z (PMC10910840; doi:10.1186/s13071-024-06158-z)
Supplement: Supplementary file 10 — Additional file 10. LEA analysis. [file 13071_2024_6158_MOESM10_ESM.html]

Aedes albopictus SNP chip - Ancestry analysis with LEA.


# Aedes albopictus SNP chip - Ancestry analysis with LEA.

#### Luciano V Cosme

#### 2023-08-28

- 1 Neutral SNP
  set
  - 1.1 k7
  - 1.2 k5
- 2 SNP set with prunning
  using r2 0.01
  - 2.1 k5
- 3 SNP set with prunning
  using r2 0.1
  - 3.1 k5

##LEA

Libraries

```
library(LEA)
library(vcfR)
library(RColorBrewer)
library(ggplot2)
library(adegenet)
library(ape)
library(tidyverse)
library(here)
library(dplyr)
library(ggplot2)
library(colorout)
library(extrafont)
library(scales)
library(stringr)
library(ggtext)
```

### 1 Neutral SNP set

Check the data

```
ls output/populations/snps_sets/*.vcf
```

```
## output/populations/snps_sets/neutral.vcf
## output/populations/snps_sets/r2_0.01.vcf
## output/populations/snps_sets/r2_0.1.vcf
```

Import the data

```
genotype <- here(
    "output", "populations", "snps_sets", "neutral.vcf"
  )

d <- read.vcfR(
  genotype
)
```

```
## Scanning file to determine attributes.
## File attributes:
##   meta lines: 8
##   header_line: 9
##   variant count: 9483
##   column count: 246
## 
Meta line 8 read in.
## All meta lines processed.
## gt matrix initialized.
## Character matrix gt created.
##   Character matrix gt rows: 9483
##   Character matrix gt cols: 246
##   skip: 0
##   nrows: 9483
##   row_num: 0
## 
Processed variant 1000
Processed variant 2000
Processed variant 3000
Processed variant 4000
Processed variant 5000
Processed variant 6000
Processed variant 7000
Processed variant 8000
Processed variant 9000
Processed variant: 9483
## All variants processed
```

Population and individuals information

```
inds_full <- attr(d@gt,"dimnames")[[2]]
inds_full <- inds_full[-1]
a <- strsplit(inds_full, '_')
pops <- unname(sapply(a, FUN = function(x) return(as.character(x[1])))) 
table(pops)
```

```
## pops
## BEN CAM CHA GEL HAI HAN HOC HUN INJ INW JAF KAC KAG KAN KAT KLP KUN LAM MAT OKI 
##  12  12  12   2  12   4   7  12  11   4   2   6  12  11  10   4   4   9  12  11 
## QNC SON SSK SUF SUU TAI UTS YUN 
##  12   3  12   6   6   8  12   9
```

```
pops <- factor(pops)
inds <- unname(sapply(a, FUN = function(x) return(as.character(x[2]))))
```

Convert format

```
vcf2geno(genotype, gsub(".vcf", ".geno", genotype))
```

```
## 
##  - number of detected individuals:   237
##  - number of detected loci:      9483
## 
## For SNP info, please check /Users/lucianocosme/Library/CloudStorage/Dropbox/Albopictus/manuscript_chip/data/no_autogenous/albo_chip/output/populations/snps_sets/neutral.vcfsnp.
## 
## 0 line(s) were removed because these are not SNPs.
## Please, check /Users/lucianocosme/Library/CloudStorage/Dropbox/Albopictus/manuscript_chip/data/no_autogenous/albo_chip/output/populations/snps_sets/neutral.removed file, for more informations.
```

```
## [1] "/Users/lucianocosme/Library/CloudStorage/Dropbox/Albopictus/manuscript_chip/data/no_autogenous/albo_chip/output/populations/snps_sets/neutral.geno"
```

```
vcf2lfmm(genotype, gsub(".vcf", ".lfmm", genotype))
```

```
## 
##  - number of detected individuals:   237
##  - number of detected loci:      9483
## 
## For SNP info, please check /Users/lucianocosme/Library/CloudStorage/Dropbox/Albopictus/manuscript_chip/data/no_autogenous/albo_chip/output/populations/snps_sets/neutral.vcfsnp.
## 
## 0 line(s) were removed because these are not SNPs.
## Please, check /Users/lucianocosme/Library/CloudStorage/Dropbox/Albopictus/manuscript_chip/data/no_autogenous/albo_chip/output/populations/snps_sets/neutral.removed file, for more informations.
## 
## 
##  - number of detected individuals:   237
##  - number of detected loci:      9483
```

```
## [1] "/Users/lucianocosme/Library/CloudStorage/Dropbox/Albopictus/manuscript_chip/data/no_autogenous/albo_chip/output/populations/snps_sets/neutral.lfmm"
```

PCA

```
setwd(
  here(
    "output", "populations"
  )
)
nPC <- length(inds)
pc <- pca(gsub(".vcf", ".lfmm", genotype), K = nPC)
```

```
## [1] "******************************"
## [1] " Principal Component Analysis "
## [1] "******************************"
## summary of the options:
## 
##         -n (number of individuals)          237
##         -L (number of loci)                 9483
##         -K (number of principal components) 237
##         -x (genotype file)                  /Users/lucianocosme/Library/CloudStorage/Dropbox/Albopictus/manuscript_chip/data/no_autogenous/albo_chip/output/populations/snps_sets/neutral.lfmm
##         -a (eigenvalue file)                /Users/lucianocosme/Library/CloudStorage/Dropbox/Albopictus/manuscript_chip/data/no_autogenous/albo_chip/output/populations/snps_sets/neutral.pca/neutral.eigenvalues
##         -e (eigenvector file)               /Users/lucianocosme/Library/CloudStorage/Dropbox/Albopictus/manuscript_chip/data/no_autogenous/albo_chip/output/populations/snps_sets/neutral.pca/neutral.eigenvectors
##         -d (standard deviation file)        /Users/lucianocosme/Library/CloudStorage/Dropbox/Albopictus/manuscript_chip/data/no_autogenous/albo_chip/output/populations/snps_sets/neutral.pca/neutral.sdev
##         -p (projection file)                /Users/lucianocosme/Library/CloudStorage/Dropbox/Albopictus/manuscript_chip/data/no_autogenous/albo_chip/output/populations/snps_sets/neutral.pca/neutral.projections
##         -c data centered
```

```
show(pc)
```

```
## * pca class *
## 
## project directory:               /Users/lucianocosme/Library/CloudStorage/Dropbox/Albopictus/manuscript_chip/data/no_autogenous/albo_chip/output/populations/snps_sets/ 
## pca result directory:            neutral.pca/ 
## input file:                      neutral.lfmm 
## eigenvalue file:                 neutral.eigenvalues 
## eigenvector file:                neutral.eigenvectors 
## standard deviation file:         neutral.sdev 
## projection file:                 neutral.projections 
## pcaProject file:                   neutral.pcaProject 
## number of individuals:           237 
## number of loci:                  9483 
## number of principal components:  237 
## centered:                        TRUE 
## scaled:                          FALSE
```

Test

```
# PC significant test: tracy-widom test
tw <- tracy.widom(pc)
```

```
## [1] "*******************"
## [1] " Tracy-Widom tests "
## [1] "*******************"
## summary of the options:
## 
##         -n (number of eigenvalues)          237
##         -i (input file)                     /Users/lucianocosme/Library/CloudStorage/Dropbox/Albopictus/manuscript_chip/data/no_autogenous/albo_chip/output/populations/snps_sets/neutral.pca/neutral.eigenvalues
##         -o (output file)                    /Users/lucianocosme/Library/CloudStorage/Dropbox/Albopictus/manuscript_chip/data/no_autogenous/albo_chip/output/populations/snps_sets/neutral.pca/neutral.tracywidom
```

```
# tw$pvalues
# plot the percentage of variance explained by each component
plot(tw$percentage, pch = 19, col = "pink", cex = .8)
```

Get values

```
# plot preparation
pc.coord <- as.data.frame(pc$projections)
colnames(pc.coord) <- paste0("PC", 1:nPC)
pc.coord$Individual <- inds
pc.coord$Population <- pops
# perc1 <- paste0(round(tw$percentage, digits = 3) * 100, "%")
perc <- paste0(round(pc$eigenvalues/sum(pc$eigenvalues), digits = 3) * 100, "%")
nb.cols <- 40
mycolors <- colorRampPalette(brewer.pal(8, "Set2"))(nb.cols)
```

Check R symbols for plot

```
#to see all shapes -> plot shapes - para escolher os simbolos
N = 100; M = 1000
good.shapes = c(1:25,33:127)
foo = data.frame( x = rnorm(M), y = rnorm(M), s = factor( sample(1:N, M, replace = TRUE) ) )
ggplot(aes(x,y,shape=s ), data=foo ) +
  scale_shape_manual(values=good.shapes[1:N]) +
  geom_point()
```

Sample data

```
sampling_loc <- readRDS(here("output", "local_adaptation", "sampling_loc.rds"))
head(sampling_loc)
```

```
## # A tibble: 6 × 6
##   Pop_City   Country  Latitude Longitude Region Abbreviation
##   <chr>      <chr>       <dbl>     <dbl> <chr>  <chr>       
## 1 Gelephu    Bhutan       26.9      90.5 Asia   GEL         
## 2 Phnom Penh Cambodia     11.6     105.  Asia   CAM         
## 3 Hainan     China        19.2     110.  Asia   HAI         
## 4 Yunnan     China        24.5     101.  Asia   YUN         
## 5 Hunan      China        27.6     112.  Asia   HUN         
## 6 Bengaluru  India        13.0      77.6 Asia   BEN
```

More sample data

```
# import sample attributes
samples2 <- read.delim(
  here(
    "output", "populations", "Population_meta_data.txt"
  ),
  head = TRUE
)

samples2<- samples2 |>
  dplyr::select(
    region, pop
  )

# check head of the file
head(samples2)
```

```
##      region pop
## 1 East Asia HAI
## 2 East Asia HUN
## 3 East Asia YUN
## 4 East Asia AIZ
## 5 East Asia HIR
## 6 East Asia JAT
```

Merge with sampling\_loc

```
merged_loc <- merge(samples2, sampling_loc, by.x = "pop", by.y = "Abbreviation")
head(merged_loc)
```

```
##   pop          region           Pop_City    Country Latitude Longitude Region
## 1 AIZ       East Asia Aizuwakamatsu City      Japan  37.4924  139.9936   Asia
## 2 ALV Southern Europe              Vlore    Albania  40.4660   19.4897 Europe
## 3 ANT     East Africa       Antananarivo Madagascar -18.8792   47.5079 Africa
## 4 AWK     West Africa               Awka    Nigeria   6.2105    7.0741 Africa
## 5 BAR Southern Europe          Barcelona      Spain  41.3851    2.1734 Europe
## 6 BEN      South Asia          Bengaluru      India  12.9716   77.5946   Asia
```

Check pops

```
head(pc.coord$Population)
```

```
## [1] OKI OKI OKI OKI OKI OKI
## 28 Levels: BEN CAM CHA GEL HAI HAN HOC HUN INJ INW JAF KAC KAG KAN KAT ... YUN
```

Check how many sampling localities

```
length(unique(pc.coord$Population))
```

```
## [1] 28
```

Merge

```
merged_data <- merge(pc.coord, merged_loc, by.x = "Population", by.y = "pop")
head(merged_data)
```

```
##   Population     PC1       PC2       PC3     PC4       PC5       PC6     PC7
## 1        BEN 4.57996  -8.46826 -1.404020 6.29358 -0.895919  1.026170 5.34578
## 2        BEN 3.42742  -9.59001 -2.434760 7.23352  1.857060  1.270250 6.33774
## 3        BEN 4.36662  -9.52450 -0.244327 7.01980 -2.193670 -0.088839 4.33631
## 4        BEN 3.53481 -10.45140 -1.601640 6.40133 -0.720671 -0.337122 3.66928
## 5        BEN 3.50260  -8.09649  0.214005 4.98309 -0.421659  2.180020 2.94658
## 6        BEN 3.94516  -8.56587 -0.962068 6.00269 -1.399860 -0.656488 3.63677
##         PC8       PC9     PC10      PC11       PC12      PC13    PC14
## 1 -0.734776  0.827422 2.175930 -0.672215  0.9154580 -0.872199 3.56459
## 2  3.217130 -0.114101 1.743730  2.057730 -0.0171675 -6.529640 6.36593
## 3  0.679631 -0.217482 2.044000  1.836600  5.8199700 -5.536860 4.84849
## 4  3.203120  1.497300 0.763997 -2.094320  9.5748000 -9.191710 5.57817
## 5  1.808640 -0.120933 0.502904 -0.329695  0.7755190 -5.659440 2.24705
## 6  3.646310 -0.434618 0.541424 -1.643670 10.0397000 -8.894240 7.25533
##         PC15     PC16      PC17      PC18     PC19     PC20     PC21       PC22
## 1 -2.6509600 2.778680 -0.349752 -1.932020 -1.89687  7.94412 0.952197  1.7345400
## 2  1.2460900 1.895820 -6.181630  6.020260  6.89707 15.55060 3.895330  0.6852910
## 3 -0.6813360 0.851376 -1.754790  3.096370  1.04406  7.34535 2.424900 -3.5431700
## 4 -3.2099000 1.063180 -7.405340  2.391010  5.69693 23.01180 0.194000  6.4317700
## 5  0.0574468 2.072370 -5.635120  0.177878  1.55157  9.83820 0.419880  0.0141712
## 6 -2.3431800 0.619264 -7.735810  5.119290  5.38221 25.46190 0.576386  5.7393800
##         PC23       PC24      PC25       PC26      PC27      PC28      PC29
## 1  -2.790940 -0.9050710 -5.751830  -0.859881 -0.858893  1.139170 -1.160160
## 2  -0.673888 -0.0113921  0.244525   3.525560  8.062390  3.457410 -2.054240
## 3  -4.608170  1.1624000  1.784870  -1.357500  1.289740 -0.798501  4.303110
## 4 -13.474400  5.4488500 -6.198020 -18.306100 -5.899060  4.533830 -1.030990
## 5  -6.121900 -0.5481160  3.674890  -1.242210  0.824387 -0.414703 -0.810455
## 6 -12.484100  6.7499200 -6.424430 -18.611200 -5.366750  7.701110  2.496500
##        PC30     PC31      PC32      PC33      PC34      PC35      PC36
## 1 -2.140580  2.24295 -1.088790   7.72842   2.51183  0.006931  1.386570
## 2  8.973560  7.54727  7.264020  16.96730   7.94077 -9.373450 -2.260190
## 3  3.644120 -1.23790  2.720960   6.94766   1.77635  1.130270 -0.993544
## 4  0.644840 -7.59288 -2.099660 -14.31290  -8.29424 13.348900  3.218330
## 5  2.044370  7.17860 -2.467730   5.75457   1.78376 -3.800920  1.335230
## 6 -0.683019 -8.92816 -0.866001 -14.42910 -10.64030 12.204600  3.040980
##        PC37     PC38     PC39       PC40     PC41     PC42     PC43      PC44
## 1 -1.251940 -1.89531 -5.04150  0.0446239 -3.88161 -3.39001  4.67923  0.401226
## 2 -8.883420 -4.68357 -6.78437  1.4218100 16.64300  3.87165 -2.94172 -9.558130
## 3 -3.317560 -6.36436 -4.96064  7.9415800 -5.83716 -1.22192  2.68288  3.110870
## 4  1.217990 10.59340  5.54107 -4.2230900  5.85331 -1.74483 -1.23341 -3.846340
## 5  3.875980 -7.22173  2.43871 -4.4495300 -6.24042 -5.09396 -5.68967 -5.075980
## 6 -0.412429 11.13930  6.82738 -2.7678000  7.00684 -1.37748 -1.68561 -5.460210
##        PC45      PC46      PC47      PC48      PC49      PC50     PC51
## 1 -0.835357  5.842220  3.621740  0.400475  6.572430 -2.350260 -1.51243
## 2  3.978170  3.055060 -0.078807 -0.539642 -5.728380  2.906890  1.66235
## 3 -0.243200 -3.237580  1.315170  3.462140  0.923088  2.339800 -4.93367
## 4  3.374070 -4.183550 -3.994580 -0.547504  0.559999  2.079480 -0.10646
## 5  0.726620 -0.581764  8.805300 -0.354395 -2.589490 -0.991979  4.04414
## 6  1.025640 -4.146370 -2.702300 -1.943690 -0.379108 -0.690208  1.29618
##        PC52      PC53      PC54      PC55      PC56      PC57      PC58
## 1 -4.893320  7.508390  11.22130 -5.098000  5.592370 -8.697050  0.381041
## 2  5.514990 -7.304680 -10.06130  0.547658 -1.073170 -3.701520 -3.211260
## 3 -0.998673  4.150710   5.82323 -1.374120 -5.095920  3.574980  2.524190
## 4  1.498550 -0.797559   2.06011  1.600990  0.806038  0.809481  1.884970
## 5 -6.067220  7.278030   1.01929  0.165037 -3.861110  0.126088 -1.833300
## 6  1.385330 -1.531220   2.11721  1.472420  1.562550  0.725633  2.076060
##         PC59       PC60     PC61      PC62     PC63     PC64      PC65
## 1 -0.0375329   7.026850  2.04609  8.179260 -1.83539 -1.68141 -5.576690
## 2  5.0391100   1.767620 -2.84385 -4.756310  3.56739  2.00781  2.788390
## 3  1.0689600  -4.267310 -5.25547  0.885596 -6.15616 -1.73641 -0.752738
## 4 -2.9131200  -2.181750  1.40171 -1.891460 -3.36196  1.65256 -0.625289
## 5  3.5052100 -10.589500 -8.06865  4.775040  1.72490  1.85574 -1.778550
## 6 -2.5165900  -0.551139  2.80556 -3.099600 -1.38656  4.58187 -1.806420
##        PC66      PC67     PC68     PC69       PC70      PC71       PC72
## 1  9.062520  5.434270  2.19221  4.27117  2.8204000  2.112900 -1.7277900
## 2 -3.069730  1.520510 -3.33412 -3.41416  0.0222933 -2.394520 -0.0107178
## 3  9.465430 -0.514028 -4.53219 -5.90288  9.1496400  6.022360  7.9095900
## 4 -0.202468  2.615780 -1.59211 -3.23965 -4.0246200  1.754340 -1.3225000
## 5  7.440810 -1.762260  6.77406  4.81224 -5.9873500  4.525950  1.6574700
## 6 -2.710450  2.177410 -1.69677 -3.32682 -2.9779700 -0.733516 -0.4738600
##         PC73      PC74       PC75      PC76      PC77      PC78      PC79
## 1  0.1314290 -3.349410 -6.8332700  9.079940 -5.181650  -4.40176  4.169410
## 2  1.5160200  6.318320 -2.5242900 -7.094460 -1.298230  -4.01622  1.437360
## 3 -1.3178300 -5.545940 -6.3289400 -2.668530 -3.477580  -3.98455  1.023260
## 4 -1.7655700  1.463670 -0.7682090  0.461624  0.464319   2.67902  0.844333
## 5 -7.1443200  2.603760  2.4969300  5.720090  5.431270 -10.01970 -4.660510
## 6  0.0576997  0.564183 -0.0556533 -0.351028 -0.039929   2.23972 -1.244720
##       PC80      PC81      PC82      PC83      PC84      PC85      PC86
## 1 -1.80837 -0.208523  0.770581  4.289180  1.769280  0.352918 -3.593880
## 2 -1.98845  1.895420  5.217430 -1.572510  2.437250  0.472080 -0.553244
## 3  6.93311  5.753460 -3.947820 -4.975360 -7.930300 11.567100 -4.852190
## 4 -2.22573 -2.366260 -0.970764  1.910080 -0.838514  0.946982 -2.611640
## 5 -5.46028 -6.298160 -6.962570  7.424240  0.199716 -6.507910 -0.202901
## 6 -1.25249 -1.851460 -1.357920  0.966614 -0.140411  1.404400 -0.396954
##        PC87      PC88      PC89      PC90       PC91       PC92      PC93
## 1 -4.310900 -1.142550  2.109860 -3.605580  1.5636600 -6.7838900 -2.423850
## 2 -1.852580 -3.833810 -0.796682 -1.174070 -0.8028770 -1.1808900 -0.535813
## 3  2.967220 -4.509960 -1.412240 -2.800630  5.0910800 -2.6419000 13.590700
## 4 -0.235049 -0.208065  0.872347  1.399070  1.0034100  0.8288940 -2.944500
## 5  3.788100  8.335070  2.603150  4.914720 -9.9251000 -2.8354100  3.179280
## 6 -1.272870 -1.139700  1.084680 -0.377294 -0.0944503 -0.0863461 -2.959740
##       PC94      PC95      PC96      PC97      PC98      PC99      PC100
## 1 -5.27384 -0.302843 -6.312740 -4.221570 -7.441820 -9.094380 -7.8827200
## 2  0.97801 -1.397600  0.188338  1.521910  0.907088  1.849080  0.1271120
## 3 -1.10238  4.827280 -5.134680  4.663740  8.389180 -0.491622 -0.0498673
## 4  2.02056 -0.126858  5.840570 -3.306600 -0.115136  2.688190 -2.3926700
## 5 -5.12849  4.918410 -8.152770 -7.186960 -9.283610 -2.013620 -0.8972520
## 6  3.24105  0.921298  4.721970  0.438298 -0.531698  0.327161 -1.6903800
##       PC101      PC102    PC103     PC104     PC105     PC106    PC107
## 1 -1.287380 -1.8603600 -1.98593  4.714330  1.982410 -6.766560  6.76273
## 2  3.013510  0.0652664  1.33153  0.517874 -2.034060 -3.001790  3.35229
## 3  4.097080 -3.7352800 -6.24417 -5.847250  2.341870 -2.955710 -4.60267
## 4 -0.492997  2.8367800  2.20463 -3.074570 -0.494408  0.359379  2.62553
## 5 11.616400 -9.1616800  2.69873 -1.962370 11.758500 -2.022880 -7.35019
## 6 -0.513965 -1.2922100  2.40567 -2.483580 -1.765750 -1.510710  2.83200
##        PC108     PC109     PC110     PC111     PC112      PC113     PC114
## 1 -1.9031200  1.284830  5.453610 -2.580780  0.247373 -0.4931660 -4.930220
## 2 -2.2862200  2.986280  1.155250  0.820412  4.833090  3.1999600 -0.938987
## 3  3.3009300  3.854440 -8.275010  5.626140  0.648937 -3.4762900  3.095420
## 4 -0.0964178 -1.464060 -0.562084 -0.237009 -1.198630  2.1534000 -1.521260
## 5  4.1041800 -5.435820 -9.025330 -1.483870 -0.547153  7.3323300  1.812140
## 6  0.1665780 -0.810283  0.202820  1.582230 -2.652370  0.0081031  0.317752
##       PC115     PC116     PC117    PC118     PC119     PC120    PC121     PC122
## 1  0.743795 15.689900  1.412370  1.58981  2.027680 -8.313040  3.44822  3.473710
## 2 -2.334580 -2.197740 -1.739380 -1.36773  4.897370 -0.330686  1.50212  3.747340
## 3  6.760830 -5.217520  1.110880 -4.69682  4.194020 10.810900 -4.73073 -1.159710
## 4 -0.169477  0.588849  0.628267  1.87245 -1.236950 -2.478520 -1.36757  0.780662
## 5 -5.621230 -3.711460  4.797130  5.00545  0.191472 -5.538070 -2.13436 -2.751460
## 6  0.256149 -3.286150 -0.766875  1.41630  1.088660 -0.127513  2.14799 -0.488837
##       PC123    PC124     PC125    PC126     PC127     PC128     PC129     PC130
## 1 13.859400 -9.00006 -2.466840  6.44602  4.085790  1.431030 -3.006080 -3.471630
## 2 -5.836450 -2.20018  0.438695 -2.01169  0.307142 -0.824239  2.166150  2.630240
## 3  2.777990 -3.30201 -0.587463  3.56187 -3.555150 -2.400780 -5.115630  2.292310
## 4  1.543020  4.13079 -0.387638  1.49734  1.569490 -0.416921 -0.653099  0.797651
## 5  5.300660 -5.83707  0.285852 -7.66589 -1.439840 -4.179560  6.777070  1.048010
## 6 -0.508681  1.07531  0.380045  1.56422  0.197597  0.930739  0.451697  0.205135
##       PC131     PC132    PC133    PC134     PC135      PC136     PC137
## 1  2.030800  5.258830 -6.88549 -5.38959 -0.625571 -0.2568600 -2.050310
## 2 -1.480370  1.444780 -1.21193 -2.93565 -0.482316  1.6037500 -1.969420
## 3  1.682220 -3.116800 -7.82713  8.00762 -0.473220 -6.9708900  8.511560
## 4 -1.730610  0.463225 -2.10235  2.09603 -0.847681  0.0890304  0.885858
## 5 -0.887427 -1.047150 16.50850 -7.16659  2.768720 -1.6916400  2.487000
## 6 -0.181767  0.323391 -0.37344  2.32448  2.202720 -0.6687170 -3.273510
##       PC138       PC139     PC140      PC141     PC142     PC143        PC144
## 1  3.830090  14.6624000  6.024610 -4.8455300 -3.907270  6.783820  0.587011000
## 2  3.063830  -0.0668679  0.951377 -0.0665816 -1.011540 -1.975330 -3.170620000
## 3 -4.113550   3.9600000 -3.742620  9.5494200 -1.317490 -2.253360  2.367640000
## 4  0.869234   0.8548580  0.811307 -1.3509700  0.259230 -1.070750 -1.849580000
## 5  1.654070 -10.0831000 -5.007780  4.3362900 -4.923820  0.156295 -7.000790000
## 6  2.471560   0.2377650 -0.242716 -1.0164100  0.685717 -0.279365  0.000223943
##      PC145      PC146      PC147      PC148     PC149    PC150     PC151
## 1 -1.65453 -9.4433300  1.0589900 -6.1206400  1.240270 -8.02217 -3.805540
## 2  3.03497  0.3170340 -4.0708100  0.1453410 -3.188370  2.43724  0.609072
## 3  5.36599 -0.8266660 -1.2056300 -3.7755200  8.779900  9.70629 -4.376820
## 4  3.12171 -1.8629600 -0.7333280  1.5257600  0.383873  0.87531  3.306060
## 5 -3.72853  1.9991400  0.0744462  0.0989937  1.052370 -5.41301  1.668390
## 6  2.93746  0.0767604 -2.0434300  0.1984310  1.538020 -1.96368 -1.594270
##       PC152     PC153      PC154     PC155     PC156      PC157      PC158
## 1 -3.025040 -3.227340   5.017140  3.016770  4.375770  9.8344800 -3.5442900
## 2 -1.487080 -1.674140   1.777580  2.729600 -2.164090  2.2104600 -0.9471240
## 3 -1.055580  8.789380 -11.634700 -1.535430  4.208860  0.2844560  6.2664100
## 4  0.399710  0.491537   0.268903  1.035180  1.066200 -0.2473650  1.7780200
## 5  0.706334  3.788590   1.206470  0.583790 -1.227800 -4.0825700  2.6013900
## 6  1.770380  0.959574  -1.585110  0.674055 -0.962817 -0.0206288 -0.0832623
##       PC159    PC160     PC161      PC162     PC163     PC164     PC165
## 1 -5.832130 3.745470 -3.474630  6.1105500  5.839560  1.041840 -0.659596
## 2 -0.993847 2.233560  0.688248  1.3332900 -0.965502 -0.417391 -0.493454
## 3  0.152343 3.998380 -1.516970 -0.0951598  1.691180 -5.445340  6.588220
## 4 -0.367277 1.700770 -1.758280 -1.1212700  1.556810  2.004140  2.242460
## 5 -2.828740 4.078350 -2.318610 -8.8425700 -1.467300 -3.091810  0.488471
## 6 -1.829460 0.928212  0.505748 -1.9036700 -1.221910 -0.964471  0.154070
##       PC166     PC167    PC168     PC169     PC170     PC171     PC172
## 1 -6.579700 -2.877170  5.67673 -5.107130 -1.278470  1.939510 -5.534640
## 2 -5.957720  0.697465 -1.81422 -1.938320 -0.732630 -1.032480 -1.178780
## 3 -5.710580 -9.788720  3.87049  5.232480  2.155170 -0.692042 -1.682630
## 4 -1.227600 -0.817042  1.96052 -1.182370 -0.263681  0.295107 -0.603652
## 5 -1.347240 -2.285890  1.07846 -1.113530  1.134280 -3.400990 -1.506130
## 6 -0.969369 -1.995620 -1.74181  0.416151 -1.458030 -3.150780 -1.524870
##       PC173    PC174     PC175     PC176      PC177     PC178    PC179
## 1 -0.302660  0.51569  2.343320 -0.459102 -2.5272600 -2.681040 -3.21702
## 2  1.632600 -3.70284 -0.880800  0.125875 -0.8801960 -0.041944 -2.16779
## 3  3.519560  4.15595  4.854130  2.304480 -0.4639740 -1.881340 -1.00016
## 4 -0.149579 -2.39171  0.295326  0.601013 -0.0443523 -1.092060  1.90115
## 5  3.108750  3.85168 -1.298940  0.894044 -0.6068260  2.045400  0.17046
## 6  0.559315  4.24254 -1.084810  0.885966 -2.0424100  2.222140 -1.08872
##       PC180     PC181     PC182     PC183      PC184     PC185     PC186
## 1  2.136390  1.323000 -1.192750  1.201140  -2.919020  0.410820  1.358930
## 2  3.627160 -1.186110 -4.001720  4.873340 -18.388100 -4.509770  1.060130
## 3 -2.706750  2.750490 -0.698758  0.397674  -0.539422  1.637540  0.773583
## 4 -2.146720 -1.694580  1.546460 -1.542370   3.376270  4.168890 -0.208302
## 5 -0.554476 -0.595617  0.137068 -6.922230  -0.165856 -0.601471 -0.835563
## 6  2.221090 -1.320370  0.738334  1.143910  -2.623810 -5.438260 -1.853960
##      PC187     PC188     PC189     PC190     PC191      PC192     PC193
## 1 -3.34196 -1.892870 -0.789579 -3.317690  0.163351 -2.2623400  1.368820
## 2  2.61045 -0.935033  2.817180 17.995900 11.713700 -1.7631300  6.358490
## 3 -1.29694  0.384745  0.568082  0.861722 -1.535170 -0.0737759  1.544330
## 4 -3.93136 -0.185045 -0.403137 -0.926154 -0.811572 -1.8708900 -1.923990
## 5  2.71361  1.607560  2.588820  1.336380 -1.195110  0.1977410 -0.432085
## 6  2.47985  1.624080  1.977110 -0.312762  2.214540  1.8460300  1.022560
##       PC194     PC195      PC196     PC197      PC198     PC199     PC200
## 1  0.186048 -1.453480  0.3377660 -0.101624 -0.5858380 -1.138000 -0.672646
## 2 -4.393460  0.936576  2.1100700 -1.163800 -0.0813825 -8.161380  0.354375
## 3 -0.280315  2.809440  0.0818684 -2.344160 -2.1846700  0.837199  1.951400
## 4 -2.213340  6.013600 -0.8085700 -0.578112  0.4714010  0.338804 -2.510040
## 5  1.871320  0.308286  1.2153800  1.010110 -1.1301600  0.791923  0.418986
## 6  3.097320 -3.944520  0.3773920 -0.199169  0.0161010 -0.220407  3.527170
##       PC201     PC202     PC203     PC204      PC205       PC206      PC207
## 1  0.207949  1.543610  1.801580 -0.711546  -0.101880  -0.1143940  -0.335628
## 2 -4.840260 -0.331452 -0.988763  0.708973  -1.830470  -4.1519600   1.618490
## 3 -0.429272  0.208902  0.315807 -0.153456   0.889328   0.0306363  -1.146990
## 4  2.346460 -1.967980 -3.574780  0.263925 -10.572100 -18.9115000  16.194300
## 5 -0.158045 -1.884350 -0.154247  1.743620   0.198980  -1.8327400  -0.371477
## 6 -0.128442  1.064270  3.309210 -1.356000  10.124000  17.8492000 -16.137700
##       PC208      PC209      PC210     PC211     PC212     PC213     PC214
## 1 -2.210480 -0.2199330 -0.2615640 -0.869482 -0.863335 -0.385652  1.282480
## 2 -0.439339 -2.8734500 -1.7514600  0.449532  0.398645  0.753011 -0.408048
## 3  0.885223 -0.0149758  0.0218576  1.336250  1.844720  0.159873 -1.004360
## 4  8.408930 -5.4124400  6.0920200  5.177420  5.233280 -1.317570 -0.914162
## 5 -1.134550 -1.0336800 -0.3128450 -0.245478 -1.312510 -0.393133  0.388746
## 6 -9.048220  3.7959200 -5.0949900 -4.876600 -4.914900  1.366220  0.961978
##       PC215     PC216      PC217      PC218     PC219     PC220     PC221
## 1 -1.070310  0.464808  0.0395216 -0.0965031  0.491164  0.226526  0.297618
## 2  0.254589 -0.510467 -1.2161100 -1.1225900 -0.305435 -0.395907 -0.150726
## 3  0.689689 -0.338267 -0.0646241 -0.4521020  0.141887  0.529634 -0.049206
## 4 -0.485768 -1.483960  0.1420910  0.2088280 -0.833057  1.427150  1.390510
## 5 -1.156300 -0.843181 -0.3253270  0.2572840 -0.457759  0.161429 -0.469240
## 6  0.696919  0.802042  0.1545610 -0.3143780  1.084670 -1.884270 -1.338360
##       PC222      PC223      PC224      PC225      PC226      PC227      PC228
## 1 -0.357814  1.2039800 -0.2057590  0.1882880  0.0630144  0.5939770 -0.0909304
## 2 -0.805678  0.3128100 -1.0455500 -0.6390600 -0.1924250  0.8151870  1.1846300
## 3  0.844642 -0.0632818  0.6327610  0.6268330  0.3956970  0.1852140 -0.0253538
## 4  0.172224 -0.8479920 -0.4243320 -1.4838000 -0.6782690  0.3461670 -0.2532290
## 5 -0.647450 -0.2876820  0.0736936 -0.0427597 -0.2243120 -0.0291385  0.2494760
## 6 -0.135926  0.1316710  0.8108620  1.5793400  0.4819340 -0.5086290 -0.9216490
##       PC229     PC230      PC231      PC232      PC233       PC234      PC235
## 1  0.397232 -0.180931 -0.1889410  0.0431406 -0.0602863 -0.05985860 -0.1357400
## 2  0.532124  0.347892  0.0912547  0.4114500  0.3059110 -0.00943952  0.0710615
## 3  0.585886  0.582795 -0.4806230 -0.4611580 -0.1876160 -0.11030700 -0.0257094
## 4  1.096840  0.709397 -0.0229951 -0.3989080 -0.2071280  0.43099500 -0.5143800
## 5 -0.339824 -0.156057 -0.5281150  0.1687720 -0.1566490  0.00538047  0.1628910
## 6 -1.146130 -0.693639  0.1868260  0.0490921 -0.0768905 -0.39545700  0.3110580
##         PC236 PC237 Individual     region  Pop_City Country Latitude Longitude
## 1 -0.21673600     0          a South Asia Bengaluru   India  12.9716   77.5946
## 2 -0.00814409     0        257 South Asia Bengaluru   India  12.9716   77.5946
## 3  0.18336900     0        261 South Asia Bengaluru   India  12.9716   77.5946
## 4 -0.00340600     0        263 South Asia Bengaluru   India  12.9716   77.5946
## 5 -0.00961880     0        264 South Asia Bengaluru   India  12.9716   77.5946
## 6 -0.05149640     0        262 South Asia Bengaluru   India  12.9716   77.5946
##   Region
## 1   Asia
## 2   Asia
## 3   Asia
## 4   Asia
## 5   Asia
## 6   Asia
```

Create PCA plot

```
# source the plotting function
source(
  here(
    "scripts", "analysis", "my_theme2.R"
  )
)

distinct_palette <- c(
  "#E69F00",
  "#009E73",
  "#0072B2",
  "#CC79A7",
  "#800000",
  "#808080",
  "#CCEBC5",
  "#FFB5B8",
  "#99CCFF",
  "#8E8BFF",
  "#F781BF",
  "#FFFF33",
  "#A65628",
  "#984EA3",
  "#D2D2D2",
  "#4DAF4A",
  "#DEB887",
  "#7FFFD4",
  "#D2691E",
  "#DC143C",
  "#8B008B"
)


# make plot by continent and range
ggplot(merged_data, aes(PC1, PC2)) +
  geom_point(aes(colour = Country, shape = Country), size = 1) +
  xlab(paste0("PC1 (", perc[1], " Variance)")) +
  ylab(paste0("PC2 (", perc[2], " Variance)")) +
  labs(
    caption = "PCA with 9,047 intergenic SNPs of 237 mosquitoes from 28 localities in Asia."
  ) +
  guides(
    color = guide_legend(title = "Country", ncol = 3),
    shape = guide_legend(title = "Country", ncol = 3),
    fill = guide_legend(title = "Region", ncol = 1)
  ) +
  stat_ellipse(aes(fill = region, group = region), geom = "polygon", alpha = 0.2, level = 0.8) +
  scale_color_manual(values = distinct_palette) +
  scale_shape_manual(values=good.shapes[c(1:25, 58:67)]) +
  my_theme() +
  theme(
    plot.caption = element_text(face = "italic"),
    legend.position = "top",
    legend.justification = "top",
    legend.box.just = "center",
    legend.box.background = element_blank(),
    plot.margin = margin(5.5, 25, 5.5, 5.5, "points"),
    legend.margin = margin(10,10,10,10)
  )
```

```
# #   ____________________________________________________________________________
# #   save the pca plot                                                       ####
ggsave(
  here(
    "output", "populations", "figures", "PCA_lea_pc1_pc2_neutral.pdf"
  ),
  width  = 8,
  height = 6,
  units  = "in"
)
```

PC1 and PC3

```
# source the plotting function
source(
  here(
    "scripts", "analysis", "my_theme2.R"
  )
)

distinct_palette <- c(
  "#E69F00",
  "#009E73",
  "#0072B2",
  "#CC79A7",
  "#800000",
  "#808080",
  "#CCEBC5",
  "#FFB5B8",
  "#99CCFF",
  "#8E8BFF",
  "#F781BF",
  "#FFFF33",
  "#A65628",
  "#984EA3",
  "#D2D2D2",
  "#4DAF4A",
  "#DEB887",
  "#7FFFD4",
  "#D2691E",
  "#DC143C",
  "#8B008B" 
)


# make plot by continent and range
ggplot(merged_data, aes(PC1, PC2)) +
  geom_point(aes(colour = Country, shape = Country), size = 1) +
  xlab(paste0("PC1 (", perc[1], " Variance)")) +
  ylab(paste0("PC3 (", perc[3], " Variance)")) +
  labs(
    caption = "PCA with 9,047 intergenic SNPs of 237 mosquitoes from 28 localities in Asia."
  ) +
  guides(
    color = guide_legend(title = "Country", ncol = 3),
    shape = guide_legend(title = "Country", ncol = 3),
    fill = guide_legend(title = "Region", ncol = 1)
  ) +
  stat_ellipse(aes(fill = region, group = region), geom = "polygon", alpha = 0.2, level = 0.8) +
  scale_color_manual(values = distinct_palette) +
  scale_shape_manual(values=good.shapes[c(1:25, 58:67)]) +
  my_theme() +
  theme(
    plot.caption = element_text(face = "italic"),
    legend.position = "top",
    legend.justification = "top",
    legend.box.just = "center",
    legend.box.background = element_blank(),
    plot.margin = margin(5.5, 25, 5.5, 5.5, "points"),
    legend.margin = margin(10,10,10,10)
  )
```

```
# #   ____________________________________________________________________________
# #   save the pca plot                                                       ####
ggsave(
  here(
    "output", "populations", "figures", "PCA_lea_pc1_pc3_neutral.pdf"
  ),
  width  = 8,
  height = 6,
  units  = "in"
)
```

Run LEA

```
# set output dir
setwd(
  here(
    "output", "populations"
  )
)
# main options
# K = number of ancestral populations
# entropy = TRUE computes the cross-entropy criterion, # CPU = 4 is the number of CPU used (hidden input) project = NULL
project = snmf(
  genotype,
  K = 1:15,
  project = "new",
  repetitions = 5,
  CPU = 4,
  entropy = TRUE
)
```

```
project = load.snmfProject("output/populations/snps_sets/neutral.snmfProject")
```

Cross entropy

```
# Open a new pdf file
pdf(here("output","populations","figures","lea_cross_entropy_neutral.pdf"), width = 6, height = 4)

# Create your plot
plot(project, col = "pink", pch = 19, cex = 1.2)

# Close the pdf file
dev.off()
```

```
## quartz_off_screen 
##                 2
```

```
plot(project, col = "pink", pch = 19, cex = 1.2)
```

#### 1.1 k7

Default plot

```
# select the best run for K = 4 clusters
best = which.min(cross.entropy(project, K = 7))
# best is run 3
barchart(project, K = 7, run = best,
        border = NA, space = 0,
        col = distinct_palette,
        xlab = "Individuals",
        ylab = "Ancestry proportions",
        main = "Ancestry matrix") -> bp
axis(1, at = 1:length(bp$order),
     labels = bp$order, las=1,
     cex.axis = .4)
```

Mean admixture by country using ggplot

```
distinct_palette <-
  c(
    "#AE9393",
    "#FFB347",
    "#FFFF99",
    "#D0F0C0",
    "#AEC6CF",
    "#008080",
    "#F49AC2",
    "#1E90FF",
    "#75FAFF",
    "#77DD77",
    "#B22222",
    "#B8B800",
    "#B20CD9",
    "#FFD1DC",
    "#779ECB",
    "#FF8C1A"
  )

sampling_loc <- readRDS(here("output", "local_adaptation", "sampling_loc.rds"))


# Extract ancestry coefficients
Q_values <- as.data.frame(Q(project, K = 7, run = best))

# Create a named vector to map countries to regions
country_to_region <- c(
  "Bhutan" = "South Asia",
  "Cambodia" = "Southeast Asia",
  "China" = "East Asia",
  "India" = "South Asia",
  "Indonesia" = "Southeast Asia",
  "Japan" = "East Asia",
  "Malaysia" = "Southeast Asia",
  "Maldives" = "South Asia",
  "Nepal" = "South Asia",
  "Sri Lanka" = "South Asia",
  "Taiwan" = "East Asia",
  "Thailand" = "Southeast Asia",
  "Vietnam" = "Southeast Asia",
  "Madagascar" = "Africa",
  "Nigeria" = "Africa",
  "Albania" = "Europe",
  "Russia" = "Europe",
  "Spain" = "Europe",
  "USA" = "North America",
  "Argentina" = "South America",
  "Trinidad and Tobago" = "North America"
)

# Add the region to the data frame
sampling_loc$Region2 <- country_to_region[sampling_loc$Country]


# Add individual IDs and pops ids
Q_values$ind <- inds
Q_values$pop <- pops

# Melt the data frame for plotting
Q_melted <- melt(Q_values, id.vars = c("ind", "pop"))

# Join with sampling_loc to get sampling localities
Q_joined <- Q_melted |>
  left_join(sampling_loc, by = c("pop" = "Abbreviation"))

# Create a combined variable for Region and Country
Q_joined <- Q_joined |>
  mutate(Region_Country = interaction(Region, Country, sep = "_"))

# Order the combined variable by Region and Country
Q_ordered <- Q_joined |>
  arrange(Region, Region2, Country) |>
  mutate(Region_Country = factor(Region_Country, levels = unique(Region_Country)))

# Group by Country and calculate mean ancestry proportions
Q_grouped <- Q_ordered |>
  group_by(Region_Country, variable) |>
  summarise(value = mean(value), .groups = "drop")

# Create a data frame for borders
borders <- data.frame(Region_Country = unique(Q_grouped$Region_Country))

# Add the order of each country to ensure correct placement of borders
borders$order <- 1:nrow(borders) + 0.5  # Shift borders to the right edge of the bars


# Function to filter and normalize data
normalize_data <- function(df, min_value) {
  df |>
    filter(value > min_value) |>
    group_by(Region_Country) |>
    mutate(value = value / sum(value))
}

# Use the function
Q_grouped_filtered <- normalize_data(Q_grouped, 0.1)

# source the plotting function
source(
  here(
    "scripts", "analysis", "my_theme2.R"
  )
)

# Generate all potential variable names
all_variables <- paste0("V", 1:16)

# Create a data frame that pairs each potential variable with a color
color_mapping <- data.frame(variable = all_variables,
                            color = distinct_palette[1:length(all_variables)])

# Merge this data frame with Q_grouped_filtered to create the new color column
Q_grouped_filtered <- merge(Q_grouped_filtered, color_mapping, by = "variable")

# Create bar chart
ggplot(Q_grouped_filtered, aes(x = Region_Country, y = value, fill = color)) +
  geom_bar(stat = 'identity', width = 1) +
  geom_segment(data = borders, aes(x = order, xend = order, y = 0, yend = 1, fill = NULL), linetype = "solid", color = "#2C444A") +  # Add borders
  my_theme() +
  theme(axis.text.x = element_text(angle = 90, hjust = 1),
        legend.position = "none") +  # Hide legend
  xlab("") +  # Suppress x-axis label
  ylab("Ancestry proportions") +
  ggtitle("Ancestry matrix") +
  labs(caption = "Each bar represents the average ancestry proportions for individuals in a given country for k=7.") +
  # scale_fill_manual(values = color) +
  scale_x_discrete(labels = function(x) gsub(".*_", "", x))  # Remove Region prefix from labels


#   ____________________________________________________________________________
#   save the pca plot                                                       ####
# ggsave(
#   here(
#     "output", "populations", "figures", "LEA_k=7_neutral.pdf"
#   ),
#   width  = 12,
#   height = 6,
#   units  = "in",
#   device = cairo_pdf
# )
```

Using ggplot2 for individual admixtures

```
# Extract ancestry coefficients
leak7 <- read_delim(
  here("output", "populations", "snps_sets", "neutral.snmf", "K7", "run3","neutral_r3.7.Q"),
  delim = " ", # Specify the delimiter if different from the default (comma)
  col_names = FALSE,
  show_col_types = FALSE
) 
# unseen_pckmeans.7.Q
# pckmeans.7.Q
head(leak7)
```

```
## # A tibble: 6 × 7
##      X1       X2       X3       X4       X5       X6       X7
##   <dbl>    <dbl>    <dbl>    <dbl>    <dbl>    <dbl>    <dbl>
## 1 0.481 0.0708   0.222    0.00569  0.0413   0.0686   0.110   
## 2 0.241 0.0661   0.271    0.0172   0.0506   0.162    0.192   
## 3 0.730 0.0596   0.129    0.00957  0.000100 0.000100 0.0716  
## 4 0.999 0.000100 0.000100 0.000100 0.000100 0.000100 0.000100
## 5 0.999 0.000100 0.000100 0.000100 0.000100 0.000100 0.000100
## 6 0.721 0.0666   0.110    0.0293   0.000100 0.000100 0.0729
```

The fam file

```
fam_file <- here(
  "output", "populations", "snps_sets", "neutral.fam"
)

# Read the .fam file
fam_data <- read.table(fam_file, 
                       header = FALSE,
                       col.names = c("FamilyID", "IndividualID", "PaternalID", "MaternalID", "Sex", "Phenotype"))

# View the first few rows
head(fam_data)
```

```
##   FamilyID IndividualID PaternalID MaternalID Sex Phenotype
## 1      OKI         1001          0          0   2        -9
## 2      OKI         1002          0          0   2        -9
## 3      OKI         1003          0          0   2        -9
## 4      OKI         1004          0          0   2        -9
## 5      OKI         1005          0          0   2        -9
## 6      OKI         1006          0          0   1        -9
```

Create ID column

```
# Change column name
colnames(fam_data)[colnames(fam_data) == "IndividualID"] <- "ind"

# Change column name
colnames(fam_data)[colnames(fam_data) == "FamilyID"] <- "pop"

# Select ID
fam_data <- fam_data |>
  dplyr::select("ind", "pop")

# View the first few rows
head(fam_data)
```

```
##    ind pop
## 1 1001 OKI
## 2 1002 OKI
## 3 1003 OKI
## 4 1004 OKI
## 5 1005 OKI
## 6 1006 OKI
```

Add it to matrix

```
leak7 <- fam_data |>
  dplyr::select(ind, pop) |>
  bind_cols(leak7)

head(leak7)
```

```
##    ind pop       X1         X2         X3         X4          X5          X6
## 1 1001 OKI 0.481261 0.07082050 0.22190700 0.00569292 0.041312000 0.068599300
## 2 1002 OKI 0.240552 0.06607170 0.27105700 0.01724490 0.050601700 0.162441000
## 3 1003 OKI 0.729612 0.05958920 0.12941900 0.00956504 0.000099982 0.000099982
## 4 1004 OKI 0.999400 0.00009995 0.00009995 0.00009995 0.000099950 0.000099950
## 5 1005 OKI 0.999400 0.00009995 0.00009995 0.00009995 0.000099950 0.000099950
## 6 1006 OKI 0.721117 0.06660400 0.10982200 0.02930780 0.000099982 0.000099982
##           X7
## 1 0.11040800
## 2 0.19203200
## 3 0.07161450
## 4 0.00009995
## 5 0.00009995
## 6 0.07294920
```

Rename the columns

```
# Rename the columns starting from the third one
leak7 <- leak7 |>
  rename_with(~paste0("v", seq_along(.x)), .cols = -c(ind, pop))

# View the first few rows
head(leak7)
```

```
##    ind pop       v1         v2         v3         v4          v5          v6
## 1 1001 OKI 0.481261 0.07082050 0.22190700 0.00569292 0.041312000 0.068599300
## 2 1002 OKI 0.240552 0.06607170 0.27105700 0.01724490 0.050601700 0.162441000
## 3 1003 OKI 0.729612 0.05958920 0.12941900 0.00956504 0.000099982 0.000099982
## 4 1004 OKI 0.999400 0.00009995 0.00009995 0.00009995 0.000099950 0.000099950
## 5 1005 OKI 0.999400 0.00009995 0.00009995 0.00009995 0.000099950 0.000099950
## 6 1006 OKI 0.721117 0.06660400 0.10982200 0.02930780 0.000099982 0.000099982
##           v7
## 1 0.11040800
## 2 0.19203200
## 3 0.07161450
## 4 0.00009995
## 5 0.00009995
## 6 0.07294920
```

Import sample locations

```
sampling_loc <- readRDS(here("output", "local_adaptation", "sampling_loc.rds"))
head(sampling_loc)
```

```
## # A tibble: 6 × 6
##   Pop_City   Country  Latitude Longitude Region Abbreviation
##   <chr>      <chr>       <dbl>     <dbl> <chr>  <chr>       
## 1 Gelephu    Bhutan       26.9      90.5 Asia   GEL         
## 2 Phnom Penh Cambodia     11.6     105.  Asia   CAM         
## 3 Hainan     China        19.2     110.  Asia   HAI         
## 4 Yunnan     China        24.5     101.  Asia   YUN         
## 5 Hunan      China        27.6     112.  Asia   HUN         
## 6 Bengaluru  India        13.0      77.6 Asia   BEN
```

```
source(
  here(
    "scripts", "analysis", "my_theme3.R"
  )
)

# Create a named vector to map countries to regions
country_to_region <- c(
  "Bhutan" = "South Asia",
  "Cambodia" = "Southeast Asia",
  "China" = "East Asia",
  "India" = "South Asia",
  "Indonesia" = "Southeast Asia",
  "Japan" = "East Asia",
  "Malaysia" = "Southeast Asia",
  "Maldives" = "South Asia",
  "Nepal" = "South Asia",
  "Sri Lanka" = "South Asia",
  "Taiwan" = "East Asia",
  "Thailand" = "Southeast Asia",
  "Vietnam" = "Southeast Asia"
)

# Add the region to the data frame
sampling_loc$Region2 <- country_to_region[sampling_loc$Country]

# Melt the data frame for plotting
Q_melted <- leak7 |>
  pivot_longer(
    cols = -c(ind, pop),
    names_to = "variable",
    values_to = "value"
  )
# Join with sampling_loc to get sampling localities
Q_joined <- Q_melted |>
  left_join(sampling_loc, by = c("pop" = "Abbreviation"))

# Create a combined variable for Region and Country
Q_joined <- Q_joined |>
  mutate(Region_Country = interaction(Region, Country, sep = "_"))

# Order the combined variable by Region and Country, then by individual
Q_ordered <- Q_joined |>
  arrange(Region, Region2, Country, ind) |>
  mutate(ind = factor(ind, levels = unique(ind)))  # Convert ind to a factor with levels in the desired order

# Add labels: country names for the first individual in each country, NA for all other individuals
Q_ordered <- Q_ordered |>
  group_by(Region_Country) |>
  mutate(label = ifelse(row_number() == 1, as.character(Country), NA))

# Group by individual and variable, calculate mean ancestry proportions
Q_grouped <- Q_ordered |>
  group_by(ind, variable) |>
  summarise(value = mean(value), .groups = "drop")

# Create a data frame for borders
borders <-
  data.frame(Region_Country = unique(Q_ordered$Region_Country))

# Add the order of the last individual of each country to ensure correct placement of borders
borders$order <-
  sapply(borders$Region_Country, function(rc)
    max(which(Q_ordered$Region_Country == rc))) + 0.5  # Shift borders to the right edge of the bars

# Select only the first occurrence of each country in the ordered data
label_df <- Q_ordered |>
  filter(!is.na(label)) |>
  distinct(label, .keep_all = TRUE)

# Create a custom label function
label_func <- function(x) {
  labels <- rep("", length(x))
  labels[x %in% label_df$ind] <- label_df$label
  labels
}

# Calculate the position of lines
border_positions <- Q_ordered |>
  group_by(Country) |>
  summarise(pos = max(as.numeric(ind)) + 0)

# Calculate the position of population labels and bars
pop_labels <- Q_ordered |>
  mutate(Name = paste(pop, Pop_City, sep = " - ")) |>
  group_by(pop) |>
  slice_head(n = 1) |>
  ungroup() |>
  dplyr::select(ind, Pop_City, Country, Name) |>
  mutate(pos = as.numeric(ind))  # calculate position of population labels

pop_labels_bars <- pop_labels |>
  mutate(pos = as.numeric(ind)  - .5)


# Calculate the position of lines
border_positions <- Q_ordered |>
  group_by(Country) |>
  summarise(pos = max(as.numeric(ind)) - 1)


pop_labels_bars <- pop_labels |>
  mutate(pos = as.numeric(ind)  - .5)

# Function to filter and normalize data
normalize_data <- function(df, min_value) {
  df |>
    filter(value > min_value) |>
    group_by(ind) |>
    mutate(value = value / sum(value))
}

# Use the function
Q_grouped_filtered <- normalize_data(Q_grouped, 0.1)
# 
color_palette <-
  c(
    "v1" = "#AE9393",
    "v2" = "red",
    "v3" = "#FFFF99",
    "v4" = "#D0F0C0",
    "v5" = "#FFB347",
    "v6" = "#F49AC2",
    "v7" = "#AEC6CF"
  )


# Generate all potential variable names
all_variables <- paste0("v", 1:7)

# Map each variable to a name
color_mapping <- data.frame(variable = all_variables,
                            color = names(color_palette))

# Merge with Q_grouped_filtered
Q_grouped_filtered <- merge(Q_grouped_filtered, color_mapping, by = "variable")

# Create the plot
ggplot(Q_grouped_filtered, aes(x = as.factor(ind), y = value, fill = color)) +
  geom_bar(stat = 'identity', width = 1) +
  geom_vline(
    data = pop_labels_bars,
    aes(xintercept = pos),
    color = "#2C444A",
    linewidth = .2
  ) +
  geom_text(
    data = pop_labels,
    aes(x = as.numeric(ind), y = 1, label = Name),
    vjust = 1.5,
    hjust = 0,
    size = 2,
    angle = 90,
    inherit.aes = FALSE
  ) +
  my_theme() +
  theme(
    axis.text.x = element_text(
      angle = 90,
      hjust = 1,
      size = 12
    ),
    legend.position = "none",
    plot.margin = unit(c(3, 0.5, 0.5, 0.5), "cm")
  ) +
  xlab("Admixture matrix") +
  ylab("Ancestry proportions") +
  labs(caption = "Each bar represents the ancestry proportions for an individual for k=7.\n LEA inference for k7 with intergenic SNPs.") +
  scale_x_discrete(labels = label_func) +
  scale_fill_manual(values = color_palette) +
  expand_limits(y = c(0, 1.5))
```

```
ggsave(
  here("output", "populations", "figures", "lea_k=7_neutral.pdf"),
  width  = 12,
  height = 6,
  units  = "in",
  device = cairo_pdf
)
```

#### 1.2 k5

We can plot k=5 to compare to other algorithms.

Find the best run

```
best = which.min(cross.entropy(project, K = 5))
best
```

```
## [1] 3
```

Run 3 for k=5

```
# Extract ancestry coefficients
leak5 <- read_delim(
  here("output", "populations", "snps_sets", "neutral.snmf", "K5", "run3","neutral_r3.5.Q"),
  delim = " ",
  col_names = FALSE,
  show_col_types = FALSE
) 
# unseen_pckmeans.7.Q
# pckmeans.7.Q
head(leak5)
```

```
## # A tibble: 6 × 5
##      X1     X2     X3     X4     X5
##   <dbl>  <dbl>  <dbl>  <dbl>  <dbl>
## 1 0.486 0.156  0.0171 0.0872 0.255 
## 2 0.413 0.221  0.0428 0.0816 0.241 
## 3 0.609 0.0970 0.0268 0.102  0.165 
## 4 0.711 0.123  0.0448 0.0523 0.0687
## 5 0.769 0.128  0.0445 0.0295 0.0289
## 6 0.595 0.117  0.0503 0.0863 0.151
```

The fam file

```
fam_file <- here(
  "output", "populations", "snps_sets", "neutral.fam"
)

# Read the .fam file
fam_data <- read.table(fam_file, 
                       header = FALSE,
                       col.names = c("FamilyID", "IndividualID", "PaternalID", "MaternalID", "Sex", "Phenotype"))

# View the first few rows
head(fam_data)
```

```
##   FamilyID IndividualID PaternalID MaternalID Sex Phenotype
## 1      OKI         1001          0          0   2        -9
## 2      OKI         1002          0          0   2        -9
## 3      OKI         1003          0          0   2        -9
## 4      OKI         1004          0          0   2        -9
## 5      OKI         1005          0          0   2        -9
## 6      OKI         1006          0          0   1        -9
```

Create ID column

```
# Change column name
colnames(fam_data)[colnames(fam_data) == "IndividualID"] <- "ind"

# Change column name
colnames(fam_data)[colnames(fam_data) == "FamilyID"] <- "pop"

# Select ID
fam_data <- fam_data |>
  dplyr::select("ind", "pop")

# View the first few rows
head(fam_data)
```

```
##    ind pop
## 1 1001 OKI
## 2 1002 OKI
## 3 1003 OKI
## 4 1004 OKI
## 5 1005 OKI
## 6 1006 OKI
```

Add it to matrix

```
leak5 <- fam_data |>
  dplyr::select(ind, pop) |>
  bind_cols(leak5)

head(leak5)
```

```
##    ind pop       X1        X2        X3        X4        X5
## 1 1001 OKI 0.485589 0.1555300 0.0171214 0.0872414 0.2545190
## 2 1002 OKI 0.413323 0.2213730 0.0427845 0.0816402 0.2408790
## 3 1003 OKI 0.609096 0.0969784 0.0268309 0.1019500 0.1651450
## 4 1004 OKI 0.711456 0.1227820 0.0447598 0.0522809 0.0687215
## 5 1005 OKI 0.769209 0.1279830 0.0444730 0.0294702 0.0288648
## 6 1006 OKI 0.595327 0.1167970 0.0502820 0.0862996 0.1512940
```

Rename the columns

```
# Rename the columns starting from the third one
leak5 <- leak5 |>
  rename_with(~paste0("v", seq_along(.x)), .cols = -c(ind, pop))

# View the first few rows
head(leak5)
```

```
##    ind pop       v1        v2        v3        v4        v5
## 1 1001 OKI 0.485589 0.1555300 0.0171214 0.0872414 0.2545190
## 2 1002 OKI 0.413323 0.2213730 0.0427845 0.0816402 0.2408790
## 3 1003 OKI 0.609096 0.0969784 0.0268309 0.1019500 0.1651450
## 4 1004 OKI 0.711456 0.1227820 0.0447598 0.0522809 0.0687215
## 5 1005 OKI 0.769209 0.1279830 0.0444730 0.0294702 0.0288648
## 6 1006 OKI 0.595327 0.1167970 0.0502820 0.0862996 0.1512940
```

Import sample locations

```
sampling_loc <- readRDS(here("output", "local_adaptation", "sampling_loc.rds"))
head(sampling_loc)
```

```
## # A tibble: 6 × 6
##   Pop_City   Country  Latitude Longitude Region Abbreviation
##   <chr>      <chr>       <dbl>     <dbl> <chr>  <chr>       
## 1 Gelephu    Bhutan       26.9      90.5 Asia   GEL         
## 2 Phnom Penh Cambodia     11.6     105.  Asia   CAM         
## 3 Hainan     China        19.2     110.  Asia   HAI         
## 4 Yunnan     China        24.5     101.  Asia   YUN         
## 5 Hunan      China        27.6     112.  Asia   HUN         
## 6 Bengaluru  India        13.0      77.6 Asia   BEN
```

Structure plot

```
source(
  here(
    "scripts", "analysis", "my_theme3.R"
  )
)

# Create a named vector to map countries to regions
country_to_region <- c(
  "Bhutan" = "South Asia",
  "Cambodia" = "Southeast Asia",
  "China" = "East Asia",
  "India" = "South Asia",
  "Indonesia" = "Southeast Asia",
  "Japan" = "East Asia",
  "Malaysia" = "Southeast Asia",
  "Maldives" = "South Asia",
  "Nepal" = "South Asia",
  "Sri Lanka" = "South Asia",
  "Taiwan" = "East Asia",
  "Thailand" = "Southeast Asia",
  "Vietnam" = "Southeast Asia"
)

# Add the region to the data frame
sampling_loc$Region2 <- country_to_region[sampling_loc$Country]

# Melt the data frame for plotting
Q_melted <- leak5 |>
  pivot_longer(
    cols = -c(ind, pop),
    names_to = "variable",
    values_to = "value"
  )
# Join with sampling_loc to get sampling localities
Q_joined <- Q_melted |>
  left_join(sampling_loc, by = c("pop" = "Abbreviation"))

# Create a combined variable for Region and Country
Q_joined <- Q_joined |>
  mutate(Region_Country = interaction(Region, Country, sep = "_"))

# Order the combined variable by Region and Country, then by individual
Q_ordered <- Q_joined |>
  arrange(Region, Region2, Country, ind) |>
  mutate(ind = factor(ind, levels = unique(ind)))  # Convert ind to a factor with levels in the desired order

# Add labels: country names for the first individual in each country, NA for all other individuals
Q_ordered <- Q_ordered |>
  group_by(Region_Country) |>
  mutate(label = ifelse(row_number() == 1, as.character(Country), NA))

# Group by individual and variable, calculate mean ancestry proportions
Q_grouped <- Q_ordered |>
  group_by(ind, variable) |>
  summarise(value = mean(value), .groups = "drop")

# Create a data frame for borders
borders <-
  data.frame(Region_Country = unique(Q_ordered$Region_Country))

# Add the order of the last individual of each country to ensure correct placement of borders
borders$order <-
  sapply(borders$Region_Country, function(rc)
    max(which(Q_ordered$Region_Country == rc))) + 0.5  # Shift borders to the right edge of the bars

# Select only the first occurrence of each country in the ordered data
label_df <- Q_ordered |>
  filter(!is.na(label)) |>
  distinct(label, .keep_all = TRUE)

# Create a custom label function
label_func <- function(x) {
  labels <- rep("", length(x))
  labels[x %in% label_df$ind] <- label_df$label
  labels
}

# Calculate the position of lines
border_positions <- Q_ordered |>
  group_by(Country) |>
  summarise(pos = max(as.numeric(ind)) + 0)

# Calculate the position of population labels and bars
pop_labels <- Q_ordered |>
  mutate(Name = paste(pop, Pop_City, sep = " - ")) |>
  group_by(pop) |>
  slice_head(n = 1) |>
  ungroup() |>
  dplyr::select(ind, Pop_City, Country, Name) |>
  mutate(pos = as.numeric(ind))  # calculate position of population labels

pop_labels_bars <- pop_labels |>
  mutate(pos = as.numeric(ind)  - .5)


# Calculate the position of lines
border_positions <- Q_ordered |>
  group_by(Country) |>
  summarise(pos = max(as.numeric(ind)) - 1)


pop_labels_bars <- pop_labels |>
  mutate(pos = as.numeric(ind)  - .5)

# Function to filter and normalize data
normalize_data <- function(df, min_value) {
  df |>
    filter(value > min_value) |>
    group_by(ind) |>
    mutate(value = value / sum(value))
}

# Use the function
Q_grouped_filtered <- normalize_data(Q_grouped, 0.1)
# 
color_palette <-
  c(
    "v1" = "#AE9393",
    "v2" = "#F49AC2",
    "v3" = "#FFFF99",
    "v4" = "red",
    "v5" = "#FFB347"
  )

# Generate all potential variable names
all_variables <- paste0("v", 1:5)

# Map each variable to a name
color_mapping <- data.frame(variable = all_variables,
                            color = names(color_palette))

# Merge with Q_grouped_filtered
Q_grouped_filtered <- merge(Q_grouped_filtered, color_mapping, by = "variable")

# Create the plot
ggplot(Q_grouped_filtered, aes(x = as.factor(ind), y = value, fill = color)) +
  geom_bar(stat = 'identity', width = 1) +
  geom_vline(
    data = pop_labels_bars,
    aes(xintercept = pos),
    color = "#2C444A",
    linewidth = .2
  ) +
  geom_text(
    data = pop_labels,
    aes(x = as.numeric(ind), y = 1, label = Name),
    vjust = 1.5,
    hjust = 0,
    size = 2,
    angle = 90,
    inherit.aes = FALSE
  ) +
  my_theme() +
  theme(
    axis.text.x = element_text(
      angle = 90,
      hjust = 1,
      size = 12
    ),
    legend.position = "none",
    plot.margin = unit(c(3, 0.5, 0.5, 0.5), "cm")
  ) +
  xlab("Admixture matrix") +
  ylab("Ancestry proportions") +
  labs(caption = "Each bar represents the ancestry proportions for an individual for k=5.\n LEA inference for k1:15 with intergenic SNPs") +
  scale_x_discrete(labels = label_func) +
  scale_fill_manual(values = color_palette) +
  expand_limits(y = c(0, 1.5))
```

```
ggsave(
  here("output", "populations", "figures", "lea_k=5_neutral.pdf"),
  width  = 12,
  height = 6,
  units  = "in",
  device = cairo_pdf
)
```

### 2 SNP set with prunning using r2 0.01

Import the data

```
genotype <- here(
    "output", "populations", "snps_sets", "r2_0.01.vcf"
  )

d <- read.vcfR(
  genotype
)
```

```
## Scanning file to determine attributes.
## File attributes:
##   meta lines: 8
##   header_line: 9
##   variant count: 20931
##   column count: 246
## 
Meta line 8 read in.
## All meta lines processed.
## gt matrix initialized.
## Character matrix gt created.
##   Character matrix gt rows: 20931
##   Character matrix gt cols: 246
##   skip: 0
##   nrows: 20931
##   row_num: 0
## 
Processed variant 1000
Processed variant 2000
Processed variant 3000
Processed variant 4000
Processed variant 5000
Processed variant 6000
Processed variant 7000
Processed variant 8000
Processed variant 9000
Processed variant 10000
Processed variant 11000
Processed variant 12000
Processed variant 13000
Processed variant 14000
Processed variant 15000
Processed variant 16000
Processed variant 17000
Processed variant 18000
Processed variant 19000
Processed variant 20000
Processed variant: 20931
## All variants processed
```

Samples ids

```
#create vectors of individual names and population attribution
inds_full <- attr(d@gt,"dimnames")[[2]]
inds_full <- inds_full[-1]
a <- strsplit(inds_full, '_')
pops <- unname(sapply(a, FUN = function(x) return(as.character(x[1])))) 
table(pops)
```

```
## pops
## BEN CAM CHA GEL HAI HAN HOC HUN INJ INW JAF KAC KAG KAN KAT KLP KUN LAM MAT OKI 
##  12  12  12   2  12   4   7  12  11   4   2   6  12  11  10   4   4   9  12  11 
## QNC SON SSK SUF SUU TAI UTS YUN 
##  12   3  12   6   6   8  12   9
```

```
pops <- factor(pops)
inds <- unname(sapply(a, FUN = function(x) return(as.character(x[2]))))
```

Convert

```
#  convert the vcf file, which should be located in the working directory, into a geno file in the working directory
vcf2geno(genotype, gsub(".vcf", ".geno", genotype))
```

```
## 
##  - number of detected individuals:   237
##  - number of detected loci:      20931
## 
## For SNP info, please check /Users/lucianocosme/Library/CloudStorage/Dropbox/Albopictus/manuscript_chip/data/no_autogenous/albo_chip/output/populations/snps_sets/r2_0.01.vcfsnp.
## 
## 0 line(s) were removed because these are not SNPs.
## Please, check /Users/lucianocosme/Library/CloudStorage/Dropbox/Albopictus/manuscript_chip/data/no_autogenous/albo_chip/output/populations/snps_sets/r2_0.01.removed file, for more informations.
```

```
## [1] "/Users/lucianocosme/Library/CloudStorage/Dropbox/Albopictus/manuscript_chip/data/no_autogenous/albo_chip/output/populations/snps_sets/r2_0.01.geno"
```

```
vcf2lfmm(genotype, gsub(".vcf", ".lfmm", genotype))
```

```
## 
##  - number of detected individuals:   237
##  - number of detected loci:      20931
## 
## For SNP info, please check /Users/lucianocosme/Library/CloudStorage/Dropbox/Albopictus/manuscript_chip/data/no_autogenous/albo_chip/output/populations/snps_sets/r2_0.01.vcfsnp.
## 
## 0 line(s) were removed because these are not SNPs.
## Please, check /Users/lucianocosme/Library/CloudStorage/Dropbox/Albopictus/manuscript_chip/data/no_autogenous/albo_chip/output/populations/snps_sets/r2_0.01.removed file, for more informations.
## 
## 
##  - number of detected individuals:   237
##  - number of detected loci:      20931
```

```
## [1] "/Users/lucianocosme/Library/CloudStorage/Dropbox/Albopictus/manuscript_chip/data/no_autogenous/albo_chip/output/populations/snps_sets/r2_0.01.lfmm"
```

PCA

```
### PCA #####
# set output dir
setwd(
  here(
    "output", "populations"
  )
)
nPC <- length(inds)
pc <- pca(gsub(".vcf", ".lfmm", genotype), K = nPC)
```

```
## [1] "******************************"
## [1] " Principal Component Analysis "
## [1] "******************************"
## summary of the options:
## 
##         -n (number of individuals)          237
##         -L (number of loci)                 20931
##         -K (number of principal components) 237
##         -x (genotype file)                  /Users/lucianocosme/Library/CloudStorage/Dropbox/Albopictus/manuscript_chip/data/no_autogenous/albo_chip/output/populations/snps_sets/r2_0.01.lfmm
##         -a (eigenvalue file)                /Users/lucianocosme/Library/CloudStorage/Dropbox/Albopictus/manuscript_chip/data/no_autogenous/albo_chip/output/populations/snps_sets/r2_0.01.pca/r2_0.01.eigenvalues
##         -e (eigenvector file)               /Users/lucianocosme/Library/CloudStorage/Dropbox/Albopictus/manuscript_chip/data/no_autogenous/albo_chip/output/populations/snps_sets/r2_0.01.pca/r2_0.01.eigenvectors
##         -d (standard deviation file)        /Users/lucianocosme/Library/CloudStorage/Dropbox/Albopictus/manuscript_chip/data/no_autogenous/albo_chip/output/populations/snps_sets/r2_0.01.pca/r2_0.01.sdev
##         -p (projection file)                /Users/lucianocosme/Library/CloudStorage/Dropbox/Albopictus/manuscript_chip/data/no_autogenous/albo_chip/output/populations/snps_sets/r2_0.01.pca/r2_0.01.projections
##         -c data centered
```

```
show(pc)
```

```
## * pca class *
## 
## project directory:               /Users/lucianocosme/Library/CloudStorage/Dropbox/Albopictus/manuscript_chip/data/no_autogenous/albo_chip/output/populations/snps_sets/ 
## pca result directory:            r2_0.01.pca/ 
## input file:                      r2_0.01.lfmm 
## eigenvalue file:                 r2_0.01.eigenvalues 
## eigenvector file:                r2_0.01.eigenvectors 
## standard deviation file:         r2_0.01.sdev 
## projection file:                 r2_0.01.projections 
## pcaProject file:                   r2_0.01.pcaProject 
## number of individuals:           237 
## number of loci:                  20931 
## number of principal components:  237 
## centered:                        TRUE 
## scaled:                          FALSE
```

Test

```
# PC significant test: tracy-widom test
tw <- tracy.widom(pc)
```

```
## [1] "*******************"
## [1] " Tracy-Widom tests "
## [1] "*******************"
## summary of the options:
## 
##         -n (number of eigenvalues)          237
##         -i (input file)                     /Users/lucianocosme/Library/CloudStorage/Dropbox/Albopictus/manuscript_chip/data/no_autogenous/albo_chip/output/populations/snps_sets/r2_0.01.pca/r2_0.01.eigenvalues
##         -o (output file)                    /Users/lucianocosme/Library/CloudStorage/Dropbox/Albopictus/manuscript_chip/data/no_autogenous/albo_chip/output/populations/snps_sets/r2_0.01.pca/r2_0.01.tracywidom
```

```
# tw$pvalues
# plot the percentage of variance explained by each component
plot(tw$percentage, pch = 19, col = "pink", cex = .8)
```

Values

```
# plot preparation
pc.coord <- as.data.frame(pc$projections)
colnames(pc.coord) <- paste0("PC", 1:nPC)
pc.coord$Individual <- inds
pc.coord$Population <- pops
# perc1 <- paste0(round(tw$percentage, digits = 3) * 100, "%")
perc <- paste0(round(pc$eigenvalues/sum(pc$eigenvalues), digits = 3) * 100, "%")
nb.cols <- 40
mycolors <- colorRampPalette(brewer.pal(8, "Set2"))(nb.cols)
```

Shapes

```
#to see all shapes -> plot shapes - para escolher os simbolos
N = 100; M = 1000
good.shapes = c(1:25,33:127)
foo = data.frame( x = rnorm(M), y = rnorm(M), s = factor( sample(1:N, M, replace = TRUE) ) )
# ggplot(aes(x,y,shape=s ), data=foo ) +
#   scale_shape_manual(values=good.shapes[1:N]) +
#   geom_point()
```

```
sampling_loc <- readRDS(here("output", "local_adaptation", "sampling_loc.rds"))
head(sampling_loc)
```

```
## # A tibble: 6 × 6
##   Pop_City   Country  Latitude Longitude Region Abbreviation
##   <chr>      <chr>       <dbl>     <dbl> <chr>  <chr>       
## 1 Gelephu    Bhutan       26.9      90.5 Asia   GEL         
## 2 Phnom Penh Cambodia     11.6     105.  Asia   CAM         
## 3 Hainan     China        19.2     110.  Asia   HAI         
## 4 Yunnan     China        24.5     101.  Asia   YUN         
## 5 Hunan      China        27.6     112.  Asia   HUN         
## 6 Bengaluru  India        13.0      77.6 Asia   BEN
```

```
# import sample attributes
samples2 <- read.delim(
  here(
    "output", "populations", "Population_meta_data.txt"
  ),
  head = TRUE
)

samples2<- samples2 |>
  dplyr::select(
    region, pop
  )

# check head of the file
head(samples2)
```

```
##      region pop
## 1 East Asia HAI
## 2 East Asia HUN
## 3 East Asia YUN
## 4 East Asia AIZ
## 5 East Asia HIR
## 6 East Asia JAT
```

Merge with sampling\_loc

```
merged_loc <- merge(samples2, sampling_loc, by.x = "pop", by.y = "Abbreviation")
head(merged_loc)
```

```
##   pop          region           Pop_City    Country Latitude Longitude Region
## 1 AIZ       East Asia Aizuwakamatsu City      Japan  37.4924  139.9936   Asia
## 2 ALV Southern Europe              Vlore    Albania  40.4660   19.4897 Europe
## 3 ANT     East Africa       Antananarivo Madagascar -18.8792   47.5079 Africa
## 4 AWK     West Africa               Awka    Nigeria   6.2105    7.0741 Africa
## 5 BAR Southern Europe          Barcelona      Spain  41.3851    2.1734 Europe
## 6 BEN      South Asia          Bengaluru      India  12.9716   77.5946   Asia
```

```
head(pc.coord$Population)
```

```
## [1] OKI OKI OKI OKI OKI OKI
## 28 Levels: BEN CAM CHA GEL HAI HAN HOC HUN INJ INW JAF KAC KAG KAN KAT ... YUN
```

Check how many sampling localities

```
length(unique(pc.coord$Population))
```

```
## [1] 28
```

Merge

```
merged_data <- merge(pc.coord, merged_loc, by.x = "Population", by.y = "pop")
head(merged_data)
```

```
##   Population       PC1        PC2         PC3        PC4         PC5
## 1        BEN -17.90190 -13.002500  -7.2670900 -11.918100   1.0964700
## 2        BEN -17.93450 -14.717300  -6.3516000 -10.708700  -0.0449077
## 3        BEN -18.22870 -14.240900  -4.4810400 -10.268100   2.9889300
## 4        BEN -19.67310 -14.695200  -6.1351400 -12.530900   0.6038590
## 5        BEN -19.01670 -14.440200  -6.0434600 -10.188900   0.4559690
## 6        BEN -19.43160 -15.309400  -6.3463000 -11.194800   1.8665300
##           PC6         PC7         PC8         PC9         PC10        PC11
## 1  11.3607000   3.6325200   0.5615690   0.7443210   3.56790000  -1.2186300
## 2  11.7923000   6.1032400   0.9970240   1.2984900   3.62965000   1.3514300
## 3  12.0954000   3.8806900   0.4328050   4.1457800   0.44179200  -1.1241700
## 4  12.6324000   4.7203700   3.0257500  -0.4835580   3.13575000   1.4538800
## 5  10.3163000   4.9066400  -1.3510300   1.8632900   0.57243500   1.7469600
## 6  14.2087000   5.4421200   1.3146500   0.3335790   4.30928000   2.3163900
##          PC12        PC13         PC14        PC15        PC16        PC17
## 1   5.0798800  10.8528000  -1.62618000   1.5962100  -2.3794600   7.7308200
## 2   6.8340400  17.3541000  -3.41404000   3.9840700  -8.7519700  11.1157000
## 3   6.0387700   8.8210700   1.56500000   1.7374900  -6.1850600   4.3241800
## 4   9.5661100  23.8264000  -2.17051000   1.3617000 -19.8542000  21.7801000
## 5   4.9803800  11.0009000  -1.08150000   2.2197600  -9.7946900   9.1312500
## 6  10.4980000  23.8454000  -2.72740000  -0.9202820 -19.4283000  23.0033000
##          PC18        PC19         PC20         PC21        PC22        PC23
## 1   8.6297600  -7.2383600  -1.72834000  -2.29163000  -1.5999600  -0.9595880
## 2  12.3018000 -16.5695000  -4.05063000   4.61595000   7.6653600   8.0522900
## 3   7.1417500  -4.5098100  -0.89364100  -0.18754700   2.9355700  -0.8048980
## 4  29.8949000 -25.7393000  -6.26344000 -17.30780000  -4.8246000   6.9430100
## 5  12.6089000  -9.2004900   0.16270700   1.02105000   2.7359800  -1.1633200
## 6  30.7327000 -26.2560000  -6.58537000 -18.25270000  -6.4104500   5.7672900
##          PC24        PC25        PC26        PC27        PC28        PC29
## 1   0.2011810   0.9980390   4.8263200   0.1325070   8.5312600   4.8973400
## 2  -2.6400800  -8.0084100   3.4078600   0.5875850  41.7643000  -2.9045500
## 3  -1.2316800  -2.6352300   0.6075140  -3.4183700  14.3696000   2.2585800
## 4  -1.0752500  -1.3561500  -3.7509800   7.4906100 -53.4306000  -0.8970300
## 5  -2.0157800  -3.0357300   4.9004800  -3.9299400  13.1434000   1.1177900
## 6   1.1187500  -0.6441630  -4.6809100   7.3379300 -53.3781000  -1.9565300
##          PC30        PC31        PC32       PC33         PC34        PC35
## 1  -1.0993600   1.3776500  -3.1590300   8.197340 -3.28310e+00   0.6942100
## 2   9.6170300   0.4396210 -11.5739000  -6.502880 -2.98107e+00 -12.8954000
## 3   1.9207300   0.6430770   0.6475940   6.318330 -1.45904e+00   5.7210000
## 4  -5.2056400  -0.7765100   0.1151770  -3.516580  8.54274e+00   3.5054600
## 5   4.9757300   2.8238400   3.3264400   3.782140 -2.01291e+00   2.7278200
## 6  -6.3261300  -0.3028110   0.2640330  -4.705340  8.46390e+00   2.7145000
##         PC36        PC37        PC38        PC39        PC40         PC41
## 1 -19.546100   1.6307400   7.9670900  12.1926000  -7.5127000  -6.71680000
## 2  35.259100 -12.6680000 -19.7221000  -7.4981500   4.1988300   9.48769000
## 3  -4.952760   9.1514800   3.9724600   5.3001000  -0.6516530  -8.97442000
## 4   9.030310  -5.3780200  -5.8836200  -2.0504600   2.7857100   0.99269000
## 5  -6.012780   5.1401600   5.6471700   2.4481400  -8.8134900   3.06722000
## 6  10.026200  -6.1831900  -3.4579000  -1.5059100   3.7721300   0.92876900
##           PC42         PC43         PC44        PC45         PC46        PC47
## 1   0.27363700   0.48858400   6.65987000  -2.0698800   2.19308000   3.2132200
## 2  -3.37954000   3.00714000 -11.10240000 -11.4410000  -1.34877000  -6.4843800
## 3   3.89341000   8.49724000   7.65705000  -0.6474010  -4.21483000   9.8374400
## 4   0.24127500   2.73943000  -7.27468000   1.0411900   1.94592000   0.0667949
## 5   5.79418000 -10.21430000  10.86950000   6.4989500  -8.09734000   6.3903500
## 6  -0.40454900   3.95591000  -3.62748000   1.9212600  -0.11949000  -0.0968189
##          PC48        PC49        PC50        PC51        PC52         PC53
## 1   3.4432700  -3.0286700   0.3809270   2.8016300  -0.9819770   5.59108000
## 2  -2.7165900  -0.1707690   2.1610400  -3.2642200   0.1060190  -4.97748000
## 3   1.8423600  -6.1604300   9.0706000   4.3074200  -4.0256700   2.67980000
## 4  -4.3389700   1.6031800  -0.2220430   4.7514700  -0.3281900  -0.85157900
## 5  -7.9248600   0.9392220   1.3949800  -0.2696310  -6.9665900   3.21643000
## 6  -4.0341500   1.1811200  -0.1944490   3.0525400  -2.5135200   0.03861260
##          PC54        PC55        PC56        PC57        PC58        PC59
## 1  -0.6117950  10.0651000   9.6576100   4.7893900   1.3430800  -0.2409040
## 2   4.5585500   0.8849700   3.1219600   1.8669000   2.5932400   1.0940000
## 3   3.3207900   8.3311700  -6.7989200   1.0066300  -5.1416500  -4.2421800
## 4   0.8164820   2.7990500  -0.3478110  -4.0807900  -2.2665200   1.5128700
## 5  -9.3075500  -1.3163200  -0.4588830  -5.4090400   0.2336710   7.8980000
## 6   1.8932200   1.3383700  -1.6295700  -1.9759700  -3.2515500   1.8249000
##          PC60         PC61         PC62         PC63        PC64        PC65
## 1  14.5442000   6.75771000  -4.16275000  -3.34383000  -4.0519500  -8.9364100
## 2   2.8806000   0.79244800  -6.11226000  -2.09776000   0.8313180   0.8872920
## 3   3.2729200   5.45797000   4.79916000 -11.29980000  18.3176000  -6.7797600
## 4   0.2076770   2.27738000  -0.19930500   1.08452000   0.5369900   0.3139800
## 5  -1.7100100 -20.47780000   5.00979000  -2.12069000  -8.8987000  13.4305000
## 6  -0.1712560   0.00267691   0.19289800   2.12909000  -1.0451100  -0.6928220
##          PC66        PC67        PC68        PC69        PC70         PC71
## 1  -0.3976430   4.2047500  -5.8997100  11.1053000  -8.2240800  -6.56877000
## 2   3.8146500   0.1732010   4.7255700   1.8232100  -4.8239700   1.92682000
## 3   3.9141400 -11.9011000  -7.6325900  -1.5422200   5.0635000 -19.40920000
## 4   1.6778900   1.4802400  -0.7630900  -2.3272700  -2.8085800   2.42192000
## 5  -1.3309900   8.1064900   4.8434100  -5.6680500  -3.5060700   0.11156900
## 6  -1.5918200   2.4971500  -0.2498380   1.0470100  -2.4386300   3.74709000
##           PC72        PC73        PC74        PC75        PC76        PC77
## 1  10.17420000 -11.3514000  12.1511000   7.3504900  -3.4114200  11.4614000
## 2   6.65587000  -1.7871800   0.8773190   6.6931100  -3.8885100   4.5592300
## 3  11.23100000   2.5372800   6.0350200 -16.4688000   6.0996400  -1.9770300
## 4   1.26418000   3.5485400   2.5055700  -1.3571900  -0.2802030  -2.4362500
## 5  -9.67074000  -3.5692000   0.7541080   8.9538000   9.9158600   5.7044100
## 6   1.68754000   1.4240800   1.1316700  -0.5093640   0.6560440  -2.4503900
##          PC78         PC79         PC80        PC81       PC82         PC83
## 1  -1.2538500  12.10260000  -8.48566000  -0.1041340  -3.066780  14.98330000
## 2  -3.9005900  -0.76464200  -2.58456000  -1.3075800  -0.986590   4.14678000
## 3  -5.2103500 -14.80720000   0.70741700 -12.2276000  -5.550970  -0.01035090
## 4   0.3489420   1.81816000   1.12405000   0.5692800  -1.314130   0.66222800
## 5  -5.6811100   9.44320000   3.11869000  -7.1019500   3.851270   8.07150000
## 6  -1.7167500   0.35873200   0.95966900  -1.6488600   2.060370  -0.81767900
##          PC84         PC85         PC86        PC87         PC88        PC89
## 1   2.3149900   3.89791000  12.47620000  -0.5768750 -10.94860000   8.3926800
## 2   3.5540500  -0.54512000  -0.56611100  -3.7005800  -3.49611000   5.1526000
## 3  -4.3325200  -7.16474000 -17.07000000  11.7084000 -19.53300000   3.3667300
## 4  -0.0312164   1.63946000  -2.52175000   0.6181420  -3.65607000   1.7798800
## 5  -4.5457500 -20.50650000   7.88814000   4.2336200   5.41318000   2.4999300
## 6   0.4372570   0.02794520  -1.31700000  -0.0425701  -4.42135000   2.9181500
##           PC90        PC91         PC92        PC93         PC94        PC95
## 1  5.79364e+00   8.5922300   0.14517200  -8.6384300  6.56127e+00  15.7541000
## 2 -5.08055e+00  -1.0193600   1.22284000   2.9416000  2.18153e+00   0.9014790
## 3  3.40803e+00 -12.0594000   7.34224000   5.5900500 -1.04445e+01   2.9026900
## 4 -6.27443e-01  -0.0617235  -1.85707000   2.2907300 -1.08001e+00  -1.0537700
## 5  4.08469e-01  -3.2443700  -7.11269000 -15.5868000  1.34640e+01  -6.6347000
## 6  1.27792e-01   0.2317580  -3.22602000   4.5194700 -6.87758e-01   1.0373500
##           PC96        PC97        PC98        PC99        PC100        PC101
## 1  -2.17798000   9.6451900  -2.9397800   3.7278100  15.45490000  -1.93906000
## 2  -1.52268000   1.4386800   4.4276200   5.1092400  -1.73627000   0.58899600
## 3   7.82575000  -6.6286800  -4.8165300  -3.7488100   8.43801000  -5.59910000
## 4  -3.78208000  -1.4896300   1.2138600  -0.2799580   3.42677000   1.98463000
## 5  -4.68281000  19.6298000  -2.5686300 -10.2652000   4.34823000  -5.26535000
## 6  -3.15895000  -0.9490340  -1.2296200  -1.3242200   0.81849900  -0.37831200
##         PC102       PC103       PC104       PC105        PC106      PC107
## 1 -11.0084000   2.6904800   7.3263300 -11.4519000 -7.58281e+00  -5.480940
## 2   0.8405100   1.7065200  -3.1779200   3.3342600  4.04050e+00  -1.574890
## 3 -10.2820000  -2.5442500   0.4606190   7.9754500  1.51699e+01   4.276550
## 4  -1.4471700  -0.3787470  -1.4323000   0.6866820 -6.73745e-01   0.172906
## 5   7.8624800 -25.7274000   2.5803600  -5.8653900  5.34167e+00  12.105200
## 6  -0.7867700  -3.2531500  -1.1306500  -1.3517000  9.83633e-01   1.013040
##          PC108        PC109        PC110        PC111       PC112       PC113
## 1   5.54389000   2.90407000 -1.04296e+00  -9.08223000   0.9924240  -5.4654500
## 2   3.21862000  -2.68425000  9.06628e-01  -0.06374850  -4.1879300   9.1203200
## 3  -7.25316000 -16.91270000 -2.83988e+00  -0.20587000   2.3416300   9.2018600
## 4  -1.58892000  -1.86825000  1.11053e-01  -1.35199000   0.6076020   0.7638030
## 5   5.53872000   7.16990000  2.32264e+00   6.74998000   5.7820500 -10.2362000
## 6  -1.70615000  -1.50396000  9.25306e-01   1.41573000   0.7468450   0.9168010
##         PC114        PC115       PC116       PC117       PC118        PC119
## 1  16.0245000  2.00140e-01   2.2682500  30.9071000 -14.0176000  14.49490000
## 2  -2.9589500  1.11736e+00   1.0987300   0.2075210   2.7354000   3.58570000
## 3  -1.7522100  3.60816e+00  -5.5661300   0.3417380  -2.0877900 -11.84230000
## 4  -3.0624800  2.11320e-01  -1.8161900   1.2055500   0.0216941  -1.15648000
## 5   3.0396500 -5.68673e+00   3.0634800 -21.3337000  14.7475000   1.74105000
## 6  -4.4599900  3.44836e-01   0.6655690  -0.3938190   0.8635360   0.09947570
##         PC120       PC121       PC122       PC123       PC124       PC125
## 1  11.5170000   3.7715500  -5.3076400  14.1398000   0.3237060  14.3916000
## 2  -1.2288700  -3.3643500   3.1099900   1.3271100   2.0985700   1.7774900
## 3 -26.4610000  -0.1960150   2.3575800 -11.9602000  -8.8435700  -2.2920000
## 4   2.3529400  -2.3316600  -0.5208220  -0.1174560   0.3076740   0.5477080
## 5  -8.1251300   3.7056300   4.9470600   8.1311400  -2.4845400  -7.0165700
## 6  -0.6484200  -1.3230400   1.4381300   0.7894440  -0.3390400   1.5554200
##          PC126       PC127       PC128        PC129       PC130        PC131
## 1 -13.37340000  10.5410000  -8.9874000   0.56791200  -2.5380600  -3.79983000
## 2  -3.80600000   1.8182000  -3.6902000  -0.95580000  -0.0548548  -0.13123200
## 3  -1.64595000  -1.3426600  -6.9094600   6.19054000  -5.6844800   2.30288000
## 4   2.04812000  -0.6915380   1.5297000  -1.88508000   0.4136230   0.06409940
## 5   4.79757000  -0.4246760 -23.9412000   4.02440000  -3.8891400  -0.12459900
## 6   0.48874100   0.3708300   0.5948270  -2.95115000   0.2731490  -2.79498000
##         PC132       PC133       PC134        PC135       PC136        PC137
## 1  -6.5030200  -2.6551700  -3.0083000  1.71730e+00 -15.7980000  -3.30561000
## 2   3.3670800  -1.2812100  -3.9863700 -3.59722e+00   3.6396300  -0.29263400
## 3  -3.8112700  -6.5208300  -2.2786300  5.91427e+00 -10.5297000   5.75885000
## 4   1.9161800   0.3483600  -1.6408100  6.24563e-01   1.9145900  -3.35105000
## 5  -9.9697800   9.6634700  16.4622000 -7.43107e+00 -13.1349000  -3.12213000
## 6   0.4723780  -1.7892500  -0.7034340  9.17912e-02   0.6926220  -0.43424800
##         PC138       PC139       PC140        PC141       PC142       PC143
## 1  -3.4260900  -6.2798900 -18.2417000  18.43020000   8.3856500   8.3382400
## 2   2.0531300  -1.9245000   5.3901200   3.73856000   0.8868380  -0.2223570
## 3  -2.3776000 -15.5123000  -3.4239300  -5.39212000   9.8656200 -14.1335000
## 4   1.9526400  -0.1817410  -1.1137700  -0.06987900  -1.2474000   1.1433900
## 5  -9.4054400  -0.4743670   6.8887700  -6.01800000  -8.0882000  -2.1503700
## 6   0.3406310   1.8611000   1.3291800  -1.37300000  -0.0787373  -0.5188240
##         PC144        PC145       PC146        PC147       PC148       PC149
## 1   5.5408400  -3.53930000   7.4150600 -3.42937e+00 -14.0967000  -0.2905990
## 2  -1.4229900  -1.28928000  -0.1623980 -1.42335e+00  -0.9474570  -0.4728770
## 3 -19.5236000  -5.99970000  -2.0135600  3.70539e+00 -12.8249000  -4.6990300
## 4  -3.7148500   2.61356000  -0.0552736  1.90159e-01  -1.5400000  -0.5712190
## 5  12.6267000   5.53412000  -3.4007200  1.10638e+01   2.7785500  -6.2069600
## 6  -2.4687700   1.81950000   0.6855870  9.36018e-01  -1.4877200  -0.9258230
##         PC150       PC151       PC152       PC153        PC154       PC155
## 1  -3.6434800  -2.5165500   8.6529600   6.8365100   3.97002000  -2.4990600
## 2   3.2271500  -0.6025260   0.2413790  -2.1465900  -0.17821300  -2.1719900
## 3  -4.8782900  -3.0064800  11.3089000 -18.0655000   3.58072000   3.4149300
## 4   2.5511700  -0.7835840  -1.8468500   2.1208400   0.86718000   1.3280200
## 5   5.5223400 -15.3104000  12.5198000   0.2354350   4.09006000  -4.6936100
## 6   1.3914800  -1.6093300   0.0225369  -0.2142720   0.16645600  -0.8581280
##         PC156       PC157        PC158       PC159        PC160       PC161
## 1   3.4061500   5.6563400 -14.58930000  -2.2798100   2.55155000  -4.1559500
## 2   3.1256100   5.4330800   1.86930000  -1.3366600   2.41087000   0.5058550
## 3  -7.5334500 -11.3179000   8.14216000   8.1905900  -3.37938000  -3.3158400
## 4  -0.4268840   0.1669370   1.33259000   0.1056740   1.84925000  -0.7278290
## 5   5.9555400  -0.2620110  15.39410000  -2.8111400   2.83182000  -7.7175000
## 6   2.6887400   1.7441300  -0.42150600   0.0936997   1.10580000   0.8256850
##          PC162        PC163       PC164        PC165        PC166       PC167
## 1  -3.51141000   6.82734000  -3.0286500  -6.18625000  -1.88673000   3.0251000
## 2   1.09072000   2.31460000   0.2269610   3.22892000  -0.24556700  -2.2675200
## 3  -3.55780000  -6.88406000  -4.2120400  -7.34124000   1.14787000   9.3826900
## 4  -2.60971000  -0.09920390  -1.2460100   1.19306000   0.28925800  -0.8751480
## 5   8.38805000  -2.39690000  -4.3334900  -5.83379000   4.37561000   4.9103300
## 6   3.39013000  -1.42401000   2.5435600  -1.37272000  -0.44609500  -0.5880850
##          PC168       PC169       PC170       PC171       PC172       PC173
## 1 -10.22260000   0.0996273   0.7942900  -2.4458000   1.2247300   1.5337200
## 2   1.92965000  -5.1401600   4.8208800  -0.3862410  -0.9916400   8.7602700
## 3  -6.04788000  -2.7664900   0.7856120  -2.4417500   4.5045000  -2.2243100
## 4  -1.16024000   2.2261500   0.9775040  -1.6839200   1.5442200   1.6330500
## 5  -1.68581000  -0.3800320   1.9521600  -0.0222831  -1.6690500  -3.2809600
## 6  -0.67821100  -0.4590530  -3.1786200  -0.6266000  -1.8906000  -1.6988800
##         PC174       PC175        PC176       PC177        PC178        PC179
## 1  -5.7806100   0.7983410   0.00877461   1.8108500   3.85221000   1.66574000
## 2   4.7133100  -9.0851500  -5.14256000   1.9136200 -10.10670000  21.35360000
## 3   5.5615300  -0.0637149   5.04407000   3.1295600  -1.06508000  -0.58003900
## 4   1.5073600  -1.2129000  -0.41484000   1.9049900   0.55288900  -2.34546000
## 5   1.8357800   1.3384100  -0.30506900   2.0971600  -0.37432500  -1.72155000
## 6  -0.8807790  -0.6410000  -1.13724000  -1.7205800  -0.35331700   1.65000000
##         PC180        PC181       PC182       PC183       PC184       PC185
## 1  -1.1177500   1.68266000   0.1625360  -0.6873690  -0.1991950  -2.1421400
## 2  -0.3818160 -21.89650000 -30.0915000  -2.8223600 -20.5764000 -16.0535000
## 3   0.4946190   1.76407000   5.7782900  -0.2852550  -2.4422900  -1.4630100
## 4  -1.1191600   2.44442000   2.4140900   0.5945130   2.3770900   0.0210807
## 5   0.9931850  -0.00366152   1.1296100  -0.7329420   1.3697700  -0.8306430
## 6   2.1824700  -0.86152400  -2.9240000  -1.7888400  -2.5250800  -0.3417910
##          PC186       PC187        PC188       PC189        PC190       PC191
## 1  -0.58769500   0.5035180   0.27533300   0.6882360   0.75877800  -2.0961300
## 2  -4.91425000  -3.2009500  10.19500000  -1.4866600  10.16390000   3.5570200
## 3  -3.75322000   0.5434730   2.09033000  -0.5993340   0.34070400   1.0701100
## 4  -1.32761000   1.9706000   0.80817500  -0.7095880   0.81718400   2.2280800
## 5   0.72703800   1.1247100   2.51569000   2.2309500  -0.56147900   0.9927260
## 6   0.87559200  -1.0830600  -3.87144000   1.2436500   0.29100400  -1.3358200
##         PC192        PC193        PC194        PC195        PC196        PC197
## 1  -0.0238817  -0.38964400  -0.49024500   0.61366800  9.26965e-01   0.78885700
## 2   4.2008800  -3.81343000   0.87417200   2.64868000 -6.84628e+00   0.86509400
## 3  -1.0110900   0.92613700  -1.26355000   1.40622000 -1.29062e+00   0.24960800
## 4  -1.9902700  -3.14291000   0.62826800  -0.91398500 -1.03266e+00  -2.28230000
## 5  -2.4911500  -0.11211700   1.64198000   2.27782000 -7.43295e-02   0.79224700
## 6   1.8620200   2.24947000  -1.46699000   0.42436500  9.79570e-01   1.80249000
##          PC198        PC199        PC200       PC201        PC202        PC203
## 1  -0.27983500  1.36257e+00   0.88625200   0.3675210  -0.02983140  -0.74587700
## 2   1.74120000 -7.01877e-01  -1.48442000  -3.9125000  -0.33859300  -1.14249000
## 3   0.00583607  8.62176e-01  -2.03765000  -0.0407867  -1.18543000  -0.95655900
## 4  -1.14747000  3.38411e-01   0.00306267  -3.9818500  -0.34079700  -0.96115700
## 5  -0.87461500 -8.37404e-01   0.67335100  -0.4522920  -0.09074620   0.59809600
## 6   1.80796000 -2.26957e-01   0.57860000   3.9909200  -0.33454100   1.16419000
##         PC204        PC205       PC206       PC207        PC208       PC209
## 1  -1.3294200   0.94916000   0.0299917  -0.4342240  -0.42708400   0.6010560
## 2   0.4091430   0.45188200   1.8599100   0.0502472   0.66675500   0.1485050
## 3   0.5120000   1.21180000  -0.5175010  -0.9309760  -0.78552000   0.3290820
## 4  11.0084000  13.71620000  34.0681000  11.6234000  28.36580000   7.2110000
## 5  -0.3647070   2.75853000   1.4452400  -0.8239570   1.61747000   0.8434370
## 6 -10.5223000 -13.46310000 -33.7128000 -10.8713000 -28.31130000  -6.9543700
##          PC210        PC211        PC212        PC213        PC214       PC215
## 1  1.16840e+00   1.66659000 -7.50885e-01  -0.39230300  -0.15339200   0.0859478
## 2 -2.17571e+00  -0.74063500 -2.77119e-02  -0.09033910   1.16402000  -0.4931860
## 3 -4.75291e-01  -0.64450000  9.10209e-01  -0.24600000  -0.66132600  -0.2668270
## 4 -8.49697e+00   1.72123000  1.52825e+00  -6.57878000  -4.05028000   0.8376290
## 5 -2.94385e-01  -0.72402900 -2.28893e+00   0.15191700  -0.75391700  -0.2944950
## 6  8.41046e+00  -2.62148000 -2.72686e+00   6.21112000   4.14061000  -2.1600800
##          PC216        PC217       PC218        PC219        PC220        PC221
## 1  -0.25909800  -0.75609300  -0.6732210   0.35182400  -0.39490900  -0.73818500
## 2  -0.63981300  -0.40078500   0.4307900   1.70623000   1.31312000  -0.12043900
## 3   0.62269100  -0.03747600   0.1398690  -0.03076260   0.36565100   0.50693400
## 4   3.40488000  -0.86605800   0.9283760  -2.32685000   0.29134700  -2.06623000
## 5   0.62887400  -1.06951000  -0.5426910  -0.22257500   0.37298500  -0.16970000
## 6  -2.96618000   0.31438700  -1.0794000   1.12584000  -0.51228200   1.47176000
##          PC222        PC223        PC224        PC225        PC226        PC227
## 1   0.49605300   0.31054800   0.21872000   0.55312500   0.41663800  -0.03724110
## 2  -0.83249600  -0.02255420  -0.28774000  -0.05444470   0.23562300   0.66069000
## 3  -0.03452660   0.19564500   1.29158000  -0.11675400  -0.37066100   0.34605900
## 4  -1.38360000   0.29058500   0.96982300   0.61571200   0.22156800  -0.16733400
## 5  -0.00491723  -0.19795800   0.18460600   0.18539800  -0.12778200  -0.43623000
## 6   2.10633000  -0.04304780  -1.57466000  -1.22561000   0.16357800   0.16727400
##          PC228        PC229        PC230        PC231        PC232        PC233
## 1  -0.57371700  7.87250e-02 -3.38842e-01  -0.03936270 -2.10701e-01   0.16224100
## 2   0.76586500  4.29712e-01  6.41845e-01   0.13673200  2.28730e-01   0.12963500
## 3  -0.02058750  5.59669e-01 -2.72200e-01  -0.17554100 -2.98125e-02  -0.02614160
## 4   0.97662700 -3.41863e-01  7.25576e-01   0.07196790 -2.81714e-01  -0.06426680
## 5   0.25871900 -7.09960e-01 -8.71551e-02  -0.52434000  1.39599e-01   0.08441400
## 6  -1.11175000  8.19412e-01 -8.58836e-01   0.98445500  5.90318e-01  -0.20273500
##          PC234        PC235        PC236 PC237 Individual     region  Pop_City
## 1  -0.07677560  9.53793e-02 -2.30538e-02   NAN          a South Asia Bengaluru
## 2  -0.05388450  2.44945e-01  6.48216e-02   NAN        257 South Asia Bengaluru
## 3  -0.08380570 -7.71649e-02  1.85948e-02   NAN        261 South Asia Bengaluru
## 4   0.20849300  1.91257e-01  8.65440e-02   NAN        263 South Asia Bengaluru
## 5  -0.33321400  2.16569e-01  1.62813e-01   NAN        264 South Asia Bengaluru
## 6   0.10208400 -3.33634e-01 -2.70744e-02   NAN        262 South Asia Bengaluru
##   Country Latitude Longitude Region
## 1   India  12.9716   77.5946   Asia
## 2   India  12.9716   77.5946   Asia
## 3   India  12.9716   77.5946   Asia
## 4   India  12.9716   77.5946   Asia
## 5   India  12.9716   77.5946   Asia
## 6   India  12.9716   77.5946   Asia
```

Plot

```
# source the plotting function
source(
  here(
    "scripts", "analysis", "my_theme2.R"
  )
)

distinct_palette <- c(
  "#E69F00",
  "#009E73",
  "#0072B2",
  "#CC79A7",
  "#800000",
  "#808080",
  "#CCEBC5",
  "#FFB5B8",
  "#99CCFF",
  "#8E8BFF",
  "#F781BF",
  "#FFFF33",
  "#A65628",
  "#984EA3",
  "#D2D2D2",
  "#4DAF4A",
  "#DEB887",
  "#7FFFD4",
  "#D2691E",
  "#DC143C",
  "#8B008B"
)
merged_data$PC1 <- as.numeric(as.character(merged_data$PC1))
merged_data$PC2 <- as.numeric(as.character(merged_data$PC2))
merged_data$PC3 <- as.numeric(as.character(merged_data$PC3))

# make plot by continent and range
ggplot(merged_data, aes(PC1, PC2)) +
  geom_point(aes(colour = Country, shape = Country), size = 1) +
  xlab(paste0("PC1 (", perc[1], " Variance)")) +
  ylab(paste0("PC2 (", perc[2], " Variance)")) +
  labs(
    caption = "PCA with 20,931 SNPs of 237 mosquitoes from 28 localities in Asia."
  ) +
  guides(
    color = guide_legend(title = "Country", ncol = 3),
    shape = guide_legend(title = "Country", ncol = 3),
    fill = guide_legend(title = "Region", ncol = 1)
  ) +
  stat_ellipse(aes(fill = region, group = region), geom = "polygon", alpha = 0.2, level = 0.8) +
  scale_color_manual(values = distinct_palette) +
  scale_shape_manual(values=good.shapes[c(1:25, 58:67)]) +
  my_theme() +
  theme(
    plot.caption = element_text(face = "italic"),
    legend.position = "top",
    legend.justification = "top",
    legend.box.just = "center",
    legend.box.background = element_blank(),
    plot.margin = margin(5.5, 25, 5.5, 5.5, "points"),
    legend.margin = margin(10,10,10,10)
  )
```

```
# #   ____________________________________________________________________________
# #   save the pca plot                                                       ####
ggsave(
  here(
    "output", "populations", "figures", "PCA_lea_pc1_pc2_r2_0.01.pdf"
  ),
  width  = 8,
  height = 6,
  units  = "in"
)
```

PC1 an PC3

```
# source the plotting function
source(
  here(
    "scripts", "analysis", "my_theme2.R"
  )
)

distinct_palette <- c(
  "#E69F00",
  "#009E73",
  "#0072B2",
  "#CC79A7",
  "#800000",
  "#808080",
  "#CCEBC5",
  "#FFB5B8",
  "#99CCFF",
  "#8E8BFF",
  "#F781BF",
  "#FFFF33",
  "#A65628",
  "#984EA3",
  "#D2D2D2",
  "#4DAF4A",
  "#DEB887",
  "#7FFFD4",
  "#D2691E",
  "#DC143C",
  "#8B008B" 
)


# make plot by continent and range
ggplot(merged_data, aes(PC1, PC2)) +
  geom_point(aes(colour = Country, shape = Country), size = 1) +
  xlab(paste0("PC1 (", perc[1], " Variance)")) +
  ylab(paste0("PC3 (", perc[3], " Variance)")) +
  labs(
    caption = "PCA with 20,931 SNPs of 237 mosquitoes from 28 localities in Asia."
  ) +
  guides(
    color = guide_legend(title = "Country", ncol = 3),
    shape = guide_legend(title = "Country", ncol = 3),
    fill = guide_legend(title = "Region", ncol = 1)
  ) +
  stat_ellipse(aes(fill = region, group = region), geom = "polygon", alpha = 0.2, level = 0.8) +
  scale_color_manual(values = distinct_palette) +
  scale_shape_manual(values=good.shapes[c(1:25, 58:67)]) +
  my_theme() +
  theme(
    plot.caption = element_text(face = "italic"),
    legend.position = "top",
    legend.justification = "top",
    legend.box.just = "center",
    legend.box.background = element_blank(),
    plot.margin = margin(5.5, 25, 5.5, 5.5, "points"),
    legend.margin = margin(10,10,10,10)
  )
```

```
# #   ____________________________________________________________________________
# #   save the pca plot                                                       ####
ggsave(
  here(
    "output", "populations", "figures", "PCA_lea_pc1_pc3_r2_0.01.pdf"
  ),
  width  = 8,
  height = 6,
  units  = "in"
)
```

```
# set output dir
setwd(
  here(
    "output", "populations"
  )
)
# main options
# K = number of ancestral populations
# entropy = TRUE computes the cross-entropy criterion, # CPU = 4 is the number of CPU used (hidden input) project = NULL
project = snmf(
  genotype,
  K = 1:15,
  project = "new",
  repetitions = 5,
  CPU = 4,
  entropy = TRUE
)
```

Load project

```
# To load the project, use:
project = load.snmfProject("output/populations/snps_sets/r2_0.01.snmfProject")

# To remove the project, use:
 # remove.snmfProject("snps_sets/r2_0.01.snmfProject")
```

Cross entropy

```
# Open a new pdf file
pdf(here("output","populations","figures","lea_cross_entropy_r2_0.01.pdf"), width = 6, height = 4)

# Create your plot
plot(project, col = "pink", pch = 19, cex = 1.2)

# Close the pdf file
dev.off()
plot(project, col = "pink", pch = 19, cex = 1.2)
```

We will not plot k=8 but only the k=5 to compare to admixture

#### 2.1 k5

We can plot k=5 to compare to other algorithms

Find the best run

```
best8 = which.min(cross.entropy(project, K = 8)) # 5
best5 = which.min(cross.entropy(project, K = 5)) # 2
best8
```

```
## [1] 5
```

```
best5
```

```
## [1] 2
```

Run 3 for k=5

```
# Extract ancestry coefficients
leak5 <- read_delim(
  here("output", "populations", "snps_sets", "r2_0.01.snmf", "K5", "run2","r2_0.01_r2.5.Q"),
  delim = " ", # Specify the delimiter if different from the default (comma)
  col_names = FALSE,
  show_col_types = FALSE
) 
# unseen_pckmeans.7.Q
# pckmeans.7.Q
head(leak5)
```

```
## # A tibble: 6 × 5
##         X1      X2     X3       X4    X5
##      <dbl>   <dbl>  <dbl>    <dbl> <dbl>
## 1 0.0574   0.0290  0.240  0.0219   0.651
## 2 0.0936   0.0212  0.239  0.0460   0.600
## 3 0.0114   0.00895 0.156  0.0202   0.804
## 4 0.000100 0.0106  0.117  0.000920 0.872
## 5 0.000100 0.0174  0.0831 0.000100 0.899
## 6 0.000100 0.0305  0.154  0.0105   0.805
```

The fam file

```
fam_file <- here(
  "output", "populations", "snps_sets", "r2_0.01.fam"
)

# Read the .fam file
fam_data <- read.table(fam_file, 
                       header = FALSE,
                       col.names = c("FamilyID", "IndividualID", "PaternalID", "MaternalID", "Sex", "Phenotype"))

# View the first few rows
head(fam_data)
```

```
##   FamilyID IndividualID PaternalID MaternalID Sex Phenotype
## 1      OKI         1001          0          0   2        -9
## 2      OKI         1002          0          0   2        -9
## 3      OKI         1003          0          0   2        -9
## 4      OKI         1004          0          0   2        -9
## 5      OKI         1005          0          0   2        -9
## 6      OKI         1006          0          0   1        -9
```

Create ID column

```
# Change column name
colnames(fam_data)[colnames(fam_data) == "IndividualID"] <- "ind"


# Merge columns "FamilyID" and "IndividualID" with an underscore
# fam_data$ind <- paste(fam_data$FamilyID, fam_data$IndividualID, sep = "_")


# Change column name
colnames(fam_data)[colnames(fam_data) == "FamilyID"] <- "pop"

# Select ID
fam_data <- fam_data |>
  dplyr::select("ind", "pop")

# View the first few rows
head(fam_data)
```

```
##    ind pop
## 1 1001 OKI
## 2 1002 OKI
## 3 1003 OKI
## 4 1004 OKI
## 5 1005 OKI
## 6 1006 OKI
```

Add it to matrix

```
leak5 <- fam_data |>
  dplyr::select(ind, pop) |>
  bind_cols(leak5)

head(leak5)
```

```
##    ind pop          X1         X2       X3          X4       X5
## 1 1001 OKI 0.057351200 0.02903100 0.240333 0.021857400 0.651427
## 2 1002 OKI 0.093595600 0.02123130 0.239314 0.045972700 0.599886
## 3 1003 OKI 0.011381600 0.00894785 0.155884 0.020222400 0.803564
## 4 1004 OKI 0.000099990 0.01060030 0.116732 0.000919858 0.871648
## 5 1005 OKI 0.000099982 0.01739940 0.083069 0.000099982 0.899332
## 6 1006 OKI 0.000099990 0.03047430 0.153502 0.010503400 0.805420
```

Rename the columns

```
# Rename the columns starting from the third one
leak5 <- leak5 |>
  rename_with(~paste0("v", seq_along(.x)), .cols = -c(ind, pop))

# View the first few rows
head(leak5)
```

```
##    ind pop          v1         v2       v3          v4       v5
## 1 1001 OKI 0.057351200 0.02903100 0.240333 0.021857400 0.651427
## 2 1002 OKI 0.093595600 0.02123130 0.239314 0.045972700 0.599886
## 3 1003 OKI 0.011381600 0.00894785 0.155884 0.020222400 0.803564
## 4 1004 OKI 0.000099990 0.01060030 0.116732 0.000919858 0.871648
## 5 1005 OKI 0.000099982 0.01739940 0.083069 0.000099982 0.899332
## 6 1006 OKI 0.000099990 0.03047430 0.153502 0.010503400 0.805420
```

Import sample locations

```
sampling_loc <- readRDS(here("output", "local_adaptation", "sampling_loc.rds"))
head(sampling_loc)
```

```
## # A tibble: 6 × 6
##   Pop_City   Country  Latitude Longitude Region Abbreviation
##   <chr>      <chr>       <dbl>     <dbl> <chr>  <chr>       
## 1 Gelephu    Bhutan       26.9      90.5 Asia   GEL         
## 2 Phnom Penh Cambodia     11.6     105.  Asia   CAM         
## 3 Hainan     China        19.2     110.  Asia   HAI         
## 4 Yunnan     China        24.5     101.  Asia   YUN         
## 5 Hunan      China        27.6     112.  Asia   HUN         
## 6 Bengaluru  India        13.0      77.6 Asia   BEN
```

```
source(
  here(
    "scripts", "analysis", "my_theme3.R"
  )
)

# Create a named vector to map countries to regions
country_to_region <- c(
  "Bhutan" = "South Asia",
  "Cambodia" = "Southeast Asia",
  "China" = "East Asia",
  "India" = "South Asia",
  "Indonesia" = "Southeast Asia",
  "Japan" = "East Asia",
  "Malaysia" = "Southeast Asia",
  "Maldives" = "South Asia",
  "Nepal" = "South Asia",
  "Sri Lanka" = "South Asia",
  "Taiwan" = "East Asia",
  "Thailand" = "Southeast Asia",
  "Vietnam" = "Southeast Asia"
)

# Add the region to the data frame
sampling_loc$Region2 <- country_to_region[sampling_loc$Country]

# Melt the data frame for plotting
Q_melted <- leak5 |>
  pivot_longer(
    cols = -c(ind, pop),
    names_to = "variable",
    values_to = "value"
  )
# Join with sampling_loc to get sampling localities
Q_joined <- Q_melted |>
  left_join(sampling_loc, by = c("pop" = "Abbreviation"))

# Create a combined variable for Region and Country
Q_joined <- Q_joined |>
  mutate(Region_Country = interaction(Region, Country, sep = "_"))

# Order the combined variable by Region and Country, then by individual
Q_ordered <- Q_joined |>
  arrange(Region, Region2, Country, ind) |>
  mutate(ind = factor(ind, levels = unique(ind)))  # Convert ind to a factor with levels in the desired order

# Add labels: country names for the first individual in each country, NA for all other individuals
Q_ordered <- Q_ordered |>
  group_by(Region_Country) |>
  mutate(label = ifelse(row_number() == 1, as.character(Country), NA))

# Group by individual and variable, calculate mean ancestry proportions
Q_grouped <- Q_ordered |>
  group_by(ind, variable) |>
  summarise(value = mean(value), .groups = "drop")

# Create a data frame for borders
borders <-
  data.frame(Region_Country = unique(Q_ordered$Region_Country))

# Add the order of the last individual of each country to ensure correct placement of borders
borders$order <-
  sapply(borders$Region_Country, function(rc)
    max(which(Q_ordered$Region_Country == rc))) + 0.5  # Shift borders to the right edge of the bars

# Select only the first occurrence of each country in the ordered data
label_df <- Q_ordered |>
  filter(!is.na(label)) |>
  distinct(label, .keep_all = TRUE)

# Create a custom label function
label_func <- function(x) {
  labels <- rep("", length(x))
  labels[x %in% label_df$ind] <- label_df$label
  labels
}

# Calculate the position of lines
border_positions <- Q_ordered |>
  group_by(Country) |>
  summarise(pos = max(as.numeric(ind)) + 0)

# Calculate the position of population labels and bars
pop_labels <- Q_ordered |>
  mutate(Name = paste(pop, Pop_City, sep = " - ")) |>
  group_by(pop) |>
  slice_head(n = 1) |>
  ungroup() |>
  dplyr::select(ind, Pop_City, Country, Name) |>
  mutate(pos = as.numeric(ind))  # calculate position of population labels

pop_labels_bars <- pop_labels |>
  mutate(pos = as.numeric(ind)  - .5)


# Calculate the position of lines
border_positions <- Q_ordered |>
  group_by(Country) |>
  summarise(pos = max(as.numeric(ind)) - 1)


pop_labels_bars <- pop_labels |>
  mutate(pos = as.numeric(ind)  - .5)

# Function to filter and normalize data
normalize_data <- function(df, min_value) {
  df |>
    filter(value > min_value) |>
    group_by(ind) |>
    mutate(value = value / sum(value))
}

# Use the function
Q_grouped_filtered <- normalize_data(Q_grouped, 0.1)
# 
color_palette <-
  c(
    "v1" = "#F49AC2",
    "v2" = "#FFFF99",
    "v3" = "#FFB347",
    "v4" = "red",
    "v5" = "#AE9393"
  )


# Generate all potential variable names
all_variables <- paste0("v", 1:5)

# Map each variable to a name
color_mapping <- data.frame(variable = all_variables,
                            color = names(color_palette))

# Merge with Q_grouped_filtered
Q_grouped_filtered <- merge(Q_grouped_filtered, color_mapping, by = "variable")

# Create the plot
ggplot(Q_grouped_filtered, aes(x = as.factor(ind), y = value, fill = color)) +
  geom_bar(stat = 'identity', width = 1) +
  geom_vline(
    data = pop_labels_bars,
    aes(xintercept = pos),
    color = "#2C444A",
    linewidth = .2
  ) +
  geom_text(
    data = pop_labels,
    aes(x = as.numeric(ind), y = 1, label = Name),
    vjust = 1.5,
    hjust = 0,
    size = 2,
    angle = 90,
    inherit.aes = FALSE
  ) +
  my_theme() +
  theme(
    axis.text.x = element_text(
      angle = 90,
      hjust = 1,
      size = 12
    ),
    legend.position = "none",
    plot.margin = unit(c(3, 0.5, 0.5, 0.5), "cm")
  ) +
  xlab("Admixture matrix") +
  ylab("Ancestry proportions") +
  labs(caption = "Each bar represents the ancestry proportions for an individual for k=5.\n LEA inference for k1:15 with SNPs pruned with r2 0.01.") +
  scale_x_discrete(labels = label_func) +
  scale_fill_manual(values = color_palette) +
  expand_limits(y = c(0, 1.5))
```

```
ggsave(
  here("output", "populations", "figures", "lea_k=5_r2_0.01.pdf"),
  width  = 12,
  height = 6,
  units  = "in",
  device = cairo_pdf
)
```

### 3 SNP set with prunning using r2 0.1

Import the data

```
genotype <- here(
    "output", "populations", "snps_sets", "r2_0.1.vcf"
  )

d <- read.vcfR(
  genotype
)
```

```
## Scanning file to determine attributes.
## File attributes:
##   meta lines: 8
##   header_line: 9
##   variant count: 57780
##   column count: 246
## 
Meta line 8 read in.
## All meta lines processed.
## gt matrix initialized.
## Character matrix gt created.
##   Character matrix gt rows: 57780
##   Character matrix gt cols: 246
##   skip: 0
##   nrows: 57780
##   row_num: 0
## 
Processed variant 1000
Processed variant 2000
Processed variant 3000
Processed variant 4000
Processed variant 5000
Processed variant 6000
Processed variant 7000
Processed variant 8000
Processed variant 9000
Processed variant 10000
Processed variant 11000
Processed variant 12000
Processed variant 13000
Processed variant 14000
Processed variant 15000
Processed variant 16000
Processed variant 17000
Processed variant 18000
Processed variant 19000
Processed variant 20000
Processed variant 21000
Processed variant 22000
Processed variant 23000
Processed variant 24000
Processed variant 25000
Processed variant 26000
Processed variant 27000
Processed variant 28000
Processed variant 29000
Processed variant 30000
Processed variant 31000
Processed variant 32000
Processed variant 33000
Processed variant 34000
Processed variant 35000
Processed variant 36000
Processed variant 37000
Processed variant 38000
Processed variant 39000
Processed variant 40000
Processed variant 41000
Processed variant 42000
Processed variant 43000
Processed variant 44000
Processed variant 45000
Processed variant 46000
Processed variant 47000
Processed variant 48000
Processed variant 49000
Processed variant 50000
Processed variant 51000
Processed variant 52000
Processed variant 53000
Processed variant 54000
Processed variant 55000
Processed variant 56000
Processed variant 57000
Processed variant: 57780
## All variants processed
```

```
#create vectors of individual names and population attribution
inds_full <- attr(d@gt,"dimnames")[[2]]
inds_full <- inds_full[-1]
a <- strsplit(inds_full, '_')
pops <- unname(sapply(a, FUN = function(x) return(as.character(x[1])))) 
table(pops)
```

```
## pops
## BEN CAM CHA GEL HAI HAN HOC HUN INJ INW JAF KAC KAG KAN KAT KLP KUN LAM MAT OKI 
##  12  12  12   2  12   4   7  12  11   4   2   6  12  11  10   4   4   9  12  11 
## QNC SON SSK SUF SUU TAI UTS YUN 
##  12   3  12   6   6   8  12   9
```

```
pops <- factor(pops)
inds <- unname(sapply(a, FUN = function(x) return(as.character(x[2]))))
```

```
#convert the vcf file, which should be located in the working directory, into a geno file in the working directory
vcf2geno(genotype, gsub(".vcf", ".geno", genotype))
```

```
## 
##  - number of detected individuals:   237
##  - number of detected loci:      57780
## 
## For SNP info, please check /Users/lucianocosme/Library/CloudStorage/Dropbox/Albopictus/manuscript_chip/data/no_autogenous/albo_chip/output/populations/snps_sets/r2_0.1.vcfsnp.
## 
## 0 line(s) were removed because these are not SNPs.
## Please, check /Users/lucianocosme/Library/CloudStorage/Dropbox/Albopictus/manuscript_chip/data/no_autogenous/albo_chip/output/populations/snps_sets/r2_0.1.removed file, for more informations.
```

```
## [1] "/Users/lucianocosme/Library/CloudStorage/Dropbox/Albopictus/manuscript_chip/data/no_autogenous/albo_chip/output/populations/snps_sets/r2_0.1.geno"
```

```
vcf2lfmm(genotype, gsub(".vcf", ".lfmm", genotype))
```

```
## 
##  - number of detected individuals:   237
##  - number of detected loci:      57780
## 
## For SNP info, please check /Users/lucianocosme/Library/CloudStorage/Dropbox/Albopictus/manuscript_chip/data/no_autogenous/albo_chip/output/populations/snps_sets/r2_0.1.vcfsnp.
## 
## 0 line(s) were removed because these are not SNPs.
## Please, check /Users/lucianocosme/Library/CloudStorage/Dropbox/Albopictus/manuscript_chip/data/no_autogenous/albo_chip/output/populations/snps_sets/r2_0.1.removed file, for more informations.
## 
## 
##  - number of detected individuals:   237
##  - number of detected loci:      57780
```

```
## [1] "/Users/lucianocosme/Library/CloudStorage/Dropbox/Albopictus/manuscript_chip/data/no_autogenous/albo_chip/output/populations/snps_sets/r2_0.1.lfmm"
```

PCA

```
### PCA #####
# set output dir
setwd(
  here(
    "output", "populations"
  )
)
nPC <- length(inds)
pc <- pca(gsub(".vcf", ".lfmm", genotype), K = nPC)
```

```
## [1] "******************************"
## [1] " Principal Component Analysis "
## [1] "******************************"
## summary of the options:
## 
##         -n (number of individuals)          237
##         -L (number of loci)                 57780
##         -K (number of principal components) 237
##         -x (genotype file)                  /Users/lucianocosme/Library/CloudStorage/Dropbox/Albopictus/manuscript_chip/data/no_autogenous/albo_chip/output/populations/snps_sets/r2_0.1.lfmm
##         -a (eigenvalue file)                /Users/lucianocosme/Library/CloudStorage/Dropbox/Albopictus/manuscript_chip/data/no_autogenous/albo_chip/output/populations/snps_sets/r2_0.1.pca/r2_0.1.eigenvalues
##         -e (eigenvector file)               /Users/lucianocosme/Library/CloudStorage/Dropbox/Albopictus/manuscript_chip/data/no_autogenous/albo_chip/output/populations/snps_sets/r2_0.1.pca/r2_0.1.eigenvectors
##         -d (standard deviation file)        /Users/lucianocosme/Library/CloudStorage/Dropbox/Albopictus/manuscript_chip/data/no_autogenous/albo_chip/output/populations/snps_sets/r2_0.1.pca/r2_0.1.sdev
##         -p (projection file)                /Users/lucianocosme/Library/CloudStorage/Dropbox/Albopictus/manuscript_chip/data/no_autogenous/albo_chip/output/populations/snps_sets/r2_0.1.pca/r2_0.1.projections
##         -c data centered
```

```
show(pc)
```

```
## * pca class *
## 
## project directory:               /Users/lucianocosme/Library/CloudStorage/Dropbox/Albopictus/manuscript_chip/data/no_autogenous/albo_chip/output/populations/snps_sets/ 
## pca result directory:            r2_0.1.pca/ 
## input file:                      r2_0.1.lfmm 
## eigenvalue file:                 r2_0.1.eigenvalues 
## eigenvector file:                r2_0.1.eigenvectors 
## standard deviation file:         r2_0.1.sdev 
## projection file:                 r2_0.1.projections 
## pcaProject file:                   r2_0.1.pcaProject 
## number of individuals:           237 
## number of loci:                  57780 
## number of principal components:  237 
## centered:                        TRUE 
## scaled:                          FALSE
```

Test

```
# PC significant test: tracy-widom test
tw <- tracy.widom(pc)
```

```
## [1] "*******************"
## [1] " Tracy-Widom tests "
## [1] "*******************"
## summary of the options:
## 
##         -n (number of eigenvalues)          237
##         -i (input file)                     /Users/lucianocosme/Library/CloudStorage/Dropbox/Albopictus/manuscript_chip/data/no_autogenous/albo_chip/output/populations/snps_sets/r2_0.1.pca/r2_0.1.eigenvalues
##         -o (output file)                    /Users/lucianocosme/Library/CloudStorage/Dropbox/Albopictus/manuscript_chip/data/no_autogenous/albo_chip/output/populations/snps_sets/r2_0.1.pca/r2_0.1.tracywidom
```

```
# tw$pvalues
# plot the percentage of variance explained by each component
plot(tw$percentage, pch = 19, col = "pink", cex = .8)
```

Values

```
# plot preparation
pc.coord <- as.data.frame(pc$projections)
colnames(pc.coord) <- paste0("PC", 1:nPC)
pc.coord$Individual <- inds
pc.coord$Population <- pops
# perc1 <- paste0(round(tw$percentage, digits = 3) * 100, "%")
perc <- paste0(round(pc$eigenvalues/sum(pc$eigenvalues), digits = 3) * 100, "%")
nb.cols <- 40
mycolors <- colorRampPalette(brewer.pal(8, "Set2"))(nb.cols)
```

Symbols

```
#to see all shapes -> plot shapes - para escolher os simbolos
N = 100; M = 1000
good.shapes = c(1:25,33:127)
foo = data.frame( x = rnorm(M), y = rnorm(M), s = factor( sample(1:N, M, replace = TRUE) ) )
# ggplot(aes(x,y,shape=s ), data=foo ) +
#   scale_shape_manual(values=good.shapes[1:N]) +
#   geom_point()
```

Meta data

```
sampling_loc <- readRDS(here("output", "local_adaptation", "sampling_loc.rds"))
head(sampling_loc)
```

```
## # A tibble: 6 × 6
##   Pop_City   Country  Latitude Longitude Region Abbreviation
##   <chr>      <chr>       <dbl>     <dbl> <chr>  <chr>       
## 1 Gelephu    Bhutan       26.9      90.5 Asia   GEL         
## 2 Phnom Penh Cambodia     11.6     105.  Asia   CAM         
## 3 Hainan     China        19.2     110.  Asia   HAI         
## 4 Yunnan     China        24.5     101.  Asia   YUN         
## 5 Hunan      China        27.6     112.  Asia   HUN         
## 6 Bengaluru  India        13.0      77.6 Asia   BEN
```

More meta data

```
# import sample attributes
samples2 <- read.delim(
  here(
    "output", "populations", "Population_meta_data.txt"
  ),
  head = TRUE
)

samples2<- samples2 |>
  dplyr::select(
    region, pop
  )

# check head of the file
head(samples2)
```

```
##      region pop
## 1 East Asia HAI
## 2 East Asia HUN
## 3 East Asia YUN
## 4 East Asia AIZ
## 5 East Asia HIR
## 6 East Asia JAT
```

Merge with sampling\_loc

```
merged_loc <- merge(samples2, sampling_loc, by.x = "pop", by.y = "Abbreviation")
head(merged_loc)
```

```
##   pop          region           Pop_City    Country Latitude Longitude Region
## 1 AIZ       East Asia Aizuwakamatsu City      Japan  37.4924  139.9936   Asia
## 2 ALV Southern Europe              Vlore    Albania  40.4660   19.4897 Europe
## 3 ANT     East Africa       Antananarivo Madagascar -18.8792   47.5079 Africa
## 4 AWK     West Africa               Awka    Nigeria   6.2105    7.0741 Africa
## 5 BAR Southern Europe          Barcelona      Spain  41.3851    2.1734 Europe
## 6 BEN      South Asia          Bengaluru      India  12.9716   77.5946   Asia
```

Check pops

```
head(pc.coord$Population)
```

```
## [1] OKI OKI OKI OKI OKI OKI
## 28 Levels: BEN CAM CHA GEL HAI HAN HOC HUN INJ INW JAF KAC KAG KAN KAT ... YUN
```

Check how many sampling localities

```
length(unique(pc.coord$Population))
```

```
## [1] 28
```

Merge

```
merged_data <- merge(pc.coord, merged_loc, by.x = "Population", by.y = "pop")
head(merged_data)
```

```
##   Population       PC1        PC2         PC3         PC4          PC5
## 1        BEN -21.12510 -26.216700   8.4870200 -17.6548000  -2.71430000
## 2        BEN -20.99630 -27.566100   9.5577600 -18.2048000  -1.75778000
## 3        BEN -21.89770 -26.721400   8.5344700 -17.3222000  -3.71905000
## 4        BEN -22.71840 -29.186500   9.6005500 -19.6170000  -2.94831000
## 5        BEN -22.30890 -26.759800   7.6865200 -16.9286000  -0.77658300
## 6        BEN -22.42740 -29.049900   8.8043200 -18.9412000  -3.03286000
##            PC6         PC7         PC8          PC9        PC10         PC11
## 1   8.29477000  15.3405000  -0.2912250  -0.68459200  -0.3446610  -0.26423300
## 2   6.13050000  17.4734000  -0.9379350  -2.89122000  -0.4220160   1.65387000
## 3   6.14484000  16.3245000  -2.3025600   0.00718294  -3.2412500  -1.57941000
## 4   7.98096000  18.2228000  -1.6091300  -0.46856300   1.4654400   1.39153000
## 5   6.74858000  14.1221000  -1.9330800  -1.17370000  -2.0345600   1.61394000
## 6   7.86098000  20.2294000  -1.5138600  -0.04767160   0.2625480   2.38508000
##           PC12        PC13       PC14        PC15        PC16         PC17
## 1  15.28600000   0.2208010  -5.662690  -4.8364100  -3.2559600   8.52019000
## 2  25.48600000   0.8603530 -10.570600  -8.2537900  -8.4960800  16.38500000
## 3  17.21570000  -3.0425900  -4.455310  -3.9310800  -3.4042500   9.31844000
## 4  34.69670000  -1.7214400 -15.253700  -8.4099900 -12.8067000  26.61120000
## 5  17.70870000  -1.4226600  -6.968290  -5.9499100  -5.4339300  12.97110000
## 6  35.84950000  -1.8396600 -16.279800  -8.6482000 -10.5491000  27.06640000
##           PC18        PC19        PC20        PC21        PC22        PC23
## 1  -7.45547000 -12.9980000 -13.1721000  -2.7336000   5.5838900   0.2235280
## 2 -11.72490000 -22.1228000 -26.2750000   2.3413900  -0.9125150  -1.6070700
## 3  -5.58948000 -14.7010000 -10.4966000   2.7146200   5.8382500  -2.4851100
## 4 -24.83950000 -45.5056000 -40.5812000  -2.1702300  26.0265000  12.2099000
## 5  -9.64556000 -16.4420000 -16.4344000   1.7525900   3.8517700   1.0994000
## 6 -25.10490000 -46.8974000 -42.7536000  -3.0108400  26.4644000  11.8691000
##          PC24        PC25        PC26         PC27         PC28         PC29
## 1  -0.0977660   0.3519740   0.3603840  -1.29177000   2.52289000 -12.38320000
## 2  -6.4712800  -2.6704100   2.9100200  -2.31946000  13.14690000 -63.11520000
## 3  -2.3157800   1.0943200  -3.2507600  -0.68217800   4.88222000 -16.34230000
## 4   3.4801200  -1.9031300  10.7983000  15.84350000 -18.35750000  80.11600000
## 5  -1.1579000   0.6650960  -1.4165100  -1.71280000   2.89425000 -19.94890000
## 6   4.2589900  -3.7204100  11.0701000  16.35230000 -18.82040000  79.70180000
##           PC30        PC31        PC32        PC33        PC34        PC35
## 1   2.23894000  -4.8685000  -8.4121700  -2.9099100  -1.0632500   1.5417900
## 2  -1.48853000 -14.3530000 -11.5816000 -18.3954000  14.1691000   1.4895900
## 3  -2.75103000  -7.8402500  -7.1135300   1.0838200  -5.1373900   3.2087600
## 4   2.43782000  15.5151000  12.3956000  11.7816000  -3.6741600  -2.5404900
## 5  -3.92099000  -3.5588200  -4.4419800   0.3473710  -4.0952200   7.2271100
## 6   3.02210000  15.6442000  14.6329000  11.7776000  -2.0662700  -2.1780200
##           PC36         PC37        PC38        PC39         PC40        PC41
## 1  -6.02427000  -2.18806000  17.0480000 -11.7336000   1.64507000 -11.9023000
## 2   2.23907000  22.85220000 -54.2922000  33.3448000   7.78461000  30.2898000
## 3  -4.07089000  -4.33324000  14.3811000 -16.9921000  -8.89495000  -7.1035100
## 4  -0.13431900   8.78196000 -12.1345000  16.7969000  -1.83524000   8.6346400
## 5   1.37942000  -5.03079000   8.7141000 -18.6334000   1.41477000 -11.8812000
## 6  -1.16036000   8.89992000 -13.0480000  14.9684000   0.29230200   8.5124000
##           PC42        PC43        PC44        PC45        PC46         PC47
## 1  -1.84303000 -10.7653000  -0.6590260   0.2295630  -3.9737800  -1.48550000
## 2   4.82095000   4.3053900  10.4447000   7.0499000   3.3149700   4.67117000
## 3  -8.91135000  -7.9826900  -3.6077400  -6.9097100  -9.6182200  -6.19984000
## 4   5.16088000   0.3050340   6.5686600   2.2856900  -0.4083280   5.16049000
## 5   0.65145100   0.8891180  -9.8058600  -5.4994900   6.2744200   3.50539000
## 6   4.04394000   1.1642700   4.5592000   0.8154110   0.5976930   3.92659000
##          PC48       PC49        PC50         PC51        PC52        PC53
## 1   0.7704700   4.271930  -2.5711300  -4.23315000  17.8502000  -2.6153400
## 2  -6.3118600 -12.516600  -6.8601300  -2.76627000  -3.6622300   5.0692700
## 3  14.8582000  -9.614380   9.2264300 -14.45710000  -8.1461300   2.5570400
## 4  -0.7178890  -3.005620  -2.0844100  -1.80783000  -0.8996430  -4.4966200
## 5   4.8818200  10.317700  13.7773000  -2.66042000   8.4127100  -9.4883600
## 6  -0.2814910  -3.157850  -0.4583980  -1.90958000   0.8868460  -2.7746300
##          PC54        PC55        PC56         PC57         PC58        PC59
## 1 -17.3511000   7.5609200  -8.5034300   9.98055000   0.17205400  -5.0724600
## 2  13.4889000   2.6367000   8.5229200  -5.80035000  -1.16575000  -1.4937300
## 3 -21.9908000   1.2428000 -15.3251000  -3.54401000 -11.59130000   6.5279400
## 4   3.2565100  -1.1877700   2.1426200  -0.35587700   0.14634200   2.1414300
## 5  -2.7345600   0.4717490 -12.1945000  -4.05523000   3.51862000  -8.1935300
## 6   2.7136900  -0.2296190   1.6437500  -1.21208000   1.54248000   0.4463520
##          PC60        PC61        PC62         PC63         PC64         PC65
## 1   4.9486100  14.9490000  -1.5199000   7.06942000  11.65770000   0.70127900
## 2   5.5261500   3.8559400   2.3336500  -0.06036980  -2.45348000   3.19507000
## 3   2.0776200 -15.9403000   2.6667400   0.65687500  17.96120000   1.77015000
## 4   0.5873710   0.5500820   1.3890500   1.40572000  -1.61840000   3.19547000
## 5  -8.2266700   6.0298700 -19.8921000 -11.43200000   7.87088000 -22.04100000
## 6   1.2863000   1.4565100   0.5566610   0.46422100  -1.74822000   2.34437000
##           PC66        PC67         PC68        PC69         PC70        PC71
## 1   3.95704000  -7.8182000  13.63140000   5.1656200  -6.23350000   5.0587300
## 2   4.06759000  -4.1492200   2.43565000  -1.5840000  -6.93106000  -1.4605500
## 3  -4.41046000  -5.8493100   5.79765000   6.6740300  -6.07599000   9.6663100
## 4  -3.41262000  -3.3405300  -4.82349000  -1.0385000   1.02439000   0.3746210
## 5 -15.23610000   8.0330900   0.02494700   7.7890700  13.33950000  -1.2509100
## 6  -3.16960000  -2.1834500  -3.75185000  -1.3745800  -1.67620000  -0.8916860
##          PC72        PC73        PC74        PC75        PC76        PC77
## 1   5.3244100  -3.1439600   3.0601400 -12.9284000  -6.5948200   7.4553900
## 2  -4.3822100  -1.7575100  -5.7120100   4.4509400  -1.1125200   2.9793100
## 3  -3.5307500  -7.6236300 -15.7160000  17.9821000   1.5306500  16.6400000
## 4  -4.2089300  -0.7173410  -1.4582100   0.1425180   3.4637800   0.4420400
## 5  -0.6459200   4.1916900 -15.9249000  -1.1244700  -5.9023600 -11.9137000
## 6  -0.8438680   1.5446700   0.0408936   1.1384000   4.4380700   0.9679600
##          PC78        PC79        PC80        PC81        PC82        PC83
## 1  11.4180000  -4.1493100  -3.6194500  -7.5809200  -4.6613400 -29.7529000
## 2   0.3131760   1.3386600   1.4717200  -4.3463000   6.6829500  -0.0579343
## 3  -2.6814100  19.8327000   6.7309800  -5.1156500  -1.8507200   2.2913400
## 4   1.9045100   1.2774700   0.6844840   5.1533500   0.0925460   2.0507700
## 5  -3.2946200  -6.4985000   4.4015200 -14.9760000  -2.7248800   6.2544100
## 6  -0.0339263   1.5444500   1.9815200   4.1914900   0.8058800   2.7782300
##          PC84        PC85        PC86         PC87         PC88        PC89
## 1   2.8692600 -25.4671000   6.0299900 -24.25180000  -5.72269000  -0.1573810
## 2   3.4088300  -6.3397400   1.1540700  -3.63834000  -1.18003000   0.8212640
## 3  -6.9321300   4.9161500   8.9574800   0.02197780  -6.47049000  -0.7118300
## 4   3.8437200  -3.5312600   0.7478770  -0.38932500   0.37388700   0.7160080
## 5   1.0019100  20.3686000  -9.0571300  -1.74955000 -20.06520000 -13.0160000
## 6   3.5902700  -1.1994800   2.8760600   0.48492000  -1.47314000   0.7491990
##          PC90        PC91        PC92         PC93        PC94        PC95
## 1   4.9594800   1.5646300  25.2200000  10.04800000  -4.9090000  -6.6310000
## 2  -1.0611800   3.2943600  -6.5117900  -1.14887000   0.2463340   3.8931500
## 3  12.5858000   8.1533800   3.1850400  -7.91924000  37.7466000   6.3894000
## 4  -1.1176400   2.2843500  -3.0233800   2.47949000   0.8149600   1.3470900
## 5   2.9434100  -1.8421400  19.8871000 -19.37690000 -39.7354000  -8.2599300
## 6  -2.4755900  -0.3221850   1.0127300   0.58408300   1.3288200   0.9929450
##           PC96        PC97         PC98         PC99       PC100        PC101
## 1   1.06250000  -6.5147100 -3.30110e+01   2.45852000   7.3502700   5.54790000
## 2  -2.68238000   3.4172000 -3.75100e+00  -2.12078000   6.9060000   3.39591000
## 3  -1.49894000   9.7979500 -8.86757e+00  10.92240000  -4.1584500   4.64532000
## 4   0.47074300  -1.9753400  4.41828e+00  -0.08571420   3.2456600  -1.42437000
## 5  10.93620000  -2.7180600  8.47520e+00  -6.68774000   2.2402400 -13.10420000
## 6  -1.09000000   0.1293680  2.86652e+00  -0.87396900   0.3758100   1.55233000
##         PC102       PC103       PC104       PC105       PC106        PC107
## 1  17.7989000   2.9088600  -5.3209300   0.9298870  23.9096000   4.48179000
## 2  -0.8674370  -3.0078200  -1.8284700   3.0924500  -2.5779800  -3.28218000
## 3  23.7569000 -22.1894000  12.2133000 -20.1064000   7.3497200 -10.94570000
## 4  -0.9084140   1.5868700  -0.8982620  -1.1205500   0.7306400  -0.08758340
## 5  -1.6384400  17.5297000 -11.0310000  20.8293000 -20.0768000  18.16110000
## 6  -1.8220200   2.5812500  -3.0949400   0.3999790   1.2467100  -0.98033700
##         PC108        PC109       PC110       PC111       PC112       PC113
## 1 -14.8711000   7.42536000   5.2356700  15.1303000   4.7381300 -19.1832000
## 2  -0.2399960   0.33220600  -1.7960900   0.0425830  -2.4179400  -0.0886193
## 3   1.3638300   4.97378000  14.8264000 -17.6628000   3.9320800  -0.4203780
## 4  -0.6447630  -0.25084400   0.6140920   0.4052730  -2.2312800  -0.5270050
## 5 -12.5424000  -2.55056000  -8.9035400  -4.7021300   3.5133400  -4.8712800
## 6  -0.1677480   0.68691200  -0.6489940  -1.9357400   0.4753190   0.6299610
##         PC114       PC115        PC116       PC117       PC118        PC119
## 1  11.8697000 -16.5024000  -1.38657000  31.2129000  -2.5141500  1.55982e+01
## 2  -2.9395900  -2.6109800  -3.86882000  -2.6676500   4.8992000  1.55189e+00
## 3   8.5462400 -28.5863000  -6.94436000 -15.0042000  -3.9719400  3.50293e+00
## 4  -0.4712090  -1.2481700   1.43497000   1.3055000   0.2856130  9.66012e-01
## 5  17.3365000  -9.7901600  23.75140000 -21.9441000  29.8410000 -1.43594e+01
## 6   1.2300000  -2.2284100  -0.16898300  -0.6973090   3.0099000  7.22438e-03
##         PC120       PC121       PC122       PC123       PC124       PC125
## 1  -0.9381380   7.9233100  -7.7881200  11.4158000   4.5487900   1.2090600
## 2  -2.2910600   4.5672000   2.2435800   1.5907700  -0.2113100  -3.8570800
## 3 -26.1128000 -50.5424000  28.3420000 -16.2748000 -11.9804000  -9.0010900
## 4  -2.0107000   1.7252100   2.3525900   0.6548940  -0.2827160  -1.8095000
## 5  27.2832000   7.3588700   8.0147300   0.1358340   0.7245760  -5.0220800
## 6  -2.0911600   4.2355800   1.6387500   1.1441900  -2.3761100  -0.9229370
##          PC126        PC127        PC128       PC129       PC130       PC131
## 1  -5.66124000 -2.45241e+01  -0.94408000  -7.6509700  14.0592000  -6.7854100
## 2  -0.40686200 -1.02914e+00   1.29407000   1.7118800  -5.6673500   2.2435300
## 3   2.53832000  1.44299e+01  21.78290000  15.1964000 -29.2608000 -10.2528000
## 4   2.52369000  2.10891e+00  -1.08602000   1.3815300   0.0961440  -0.2154240
## 5 -20.50900000 -1.41735e+01  16.23380000  12.2509000 -38.6337000   9.2813200
## 6   1.11999000  4.80594e-01  -2.94598000   0.2351530  -1.6577600   0.6989100
##         PC132      PC133       PC134       PC135       PC136       PC137
## 1   3.3694000   2.374760  15.3837000  43.4299000  24.8567000  -4.5770600
## 2  -1.4535400  -0.369758   4.1663600   0.7569120  -1.4598400   2.8074300
## 3  -3.4540200  24.004000 -10.6336000 -16.6615000 -12.2395000 -20.4951000
## 4  -2.8547200  -0.237152  -3.6989100   1.7526100  -0.8467190  -0.9620770
## 5 -10.6143000  16.481200  -4.4333800   5.8114600 -12.2945000  -0.7365450
## 6  -1.7013100  -0.418307  -0.6489500   1.9126300  -2.0562300  -2.2321800
##          PC138       PC139        PC140       PC141       PC142       PC143
## 1  22.81840000   7.2535600 -24.79900000  -6.9311400  20.5485000 -30.0183000
## 2   2.72467000  -3.8703400  -2.12796000  -0.5092630  -4.1400400   0.3546790
## 3  -5.77881000  -9.8572000  -7.81972000  19.4081000 -10.2392000 -23.8906000
## 4  -0.63932900  -0.7312300  -0.24709300  -0.9064190  -2.5458000   0.7104400
## 5 -34.06140000  12.6227000   1.94264000   2.8129800  22.4516000   6.1937600
## 6  -1.57383000  -1.5379900   0.52125800  -0.7354850  -2.0646400  -1.0045100
##         PC144       PC145       PC146       PC147        PC148        PC149
## 1  28.3809000 -13.4795000  -0.8916330 -22.3339000 -35.47070000  2.64935e+01
## 2   1.0807600  -1.8851000   4.2552600   2.0318200   0.76320100  2.91314e+00
## 3 -14.0132000   1.2430400   4.2750000 -11.3812000  -7.56045000  8.49027e+00
## 4   0.8641170  -0.3380390   1.1405500  -1.9988700   2.28772000 -2.00982e+00
## 5  -6.2880600 -20.0530000  -8.8176100   0.4599260   9.19823000  1.56251e+01
## 6  -0.9878170   2.2425300   1.5324700  -1.0766500  -0.46756600 -9.26350e-01
##          PC150       PC151       PC152       PC153        PC154       PC155
## 1  14.76540000  -4.7179000   1.1529700 -10.0477000  -7.64873000   6.5217700
## 2  -0.96250400   1.1217800  -3.4778100   2.8493100  -4.85280000   2.7480900
## 3 -16.76270000  14.6486000   5.9606800 -13.5170000   5.54083000 -18.2164000
## 4  -0.23838300  -0.1063110   1.2425900   0.3399560  -1.90513000  -0.8189280
## 5  31.03950000  -3.1570900   4.7337200  21.8747000  15.65050000  -7.6675200
## 6  -3.43059000   0.1652030  -1.4355700   2.5930300   0.00541175  -0.2905870
##         PC156       PC157       PC158        PC159       PC160        PC161
## 1   9.1039300   5.5282200 -14.6385000   5.80290000   2.2640300  -2.07999000
## 2   0.4768470  -0.2338240  -0.7892600   0.23414500   2.2022900  -2.38962000
## 3  10.3792000   0.2274320   7.6679900  12.14660000   2.6239400   0.77791800
## 4  -1.7881600  -1.8496800  -1.2532500   1.32108000  -0.1423960  -0.12892000
## 5   9.5528900  -5.1418100  13.5768000  -3.92305000  10.2640000  -6.64926000
## 6  -0.3840940  -1.2611900  -0.2745640  -1.09414000   0.7413980  -3.67161000
##         PC162       PC163      PC164       PC165        PC166       PC167
## 1  -5.0150200   5.8248400  -3.722770  -0.4940910 -10.82150000  -0.9388300
## 2  -1.8125600   2.2831000   0.673383   2.8220100   4.50647000   2.6851500
## 3   3.9746700  -1.0814800  -5.647200  -0.9363040  -4.92348000   1.5099100
## 4  -2.7971600   0.2045050   1.025040   0.2495590   0.73708600   0.3986850
## 5   1.8564500   9.2686000  -6.952180   2.0798400   1.55571000  -0.2951240
## 6   1.5246700  -0.4823120  -0.676185  -0.0864281  -2.20714000   0.2351740
##          PC168       PC169       PC170        PC171        PC172       PC173
## 1  -0.55442200  -0.3743910   6.3452300  -7.13375000   0.43970100  -0.1253670
## 2  -2.59928000  -0.1307130   3.9138000  -1.89943000  -0.33665400   1.6222900
## 3   4.57266000   3.2703200   1.8131000   1.01558000   5.39459000  -5.5055100
## 4   0.05411580   1.1003500   0.3403380  -0.74050100   2.04745000   2.3980400
## 5  -0.99827800   6.0520200   4.8501700   0.40237900   1.92762000  -0.5799570
## 6   1.53756000   0.7213230  -0.1994150  -0.34776100   0.26710100  -0.4932810
##          PC174        PC175       PC176       PC177       PC178        PC179
## 1 -3.81927e-01  -2.11304000   2.9439400   2.4441800   1.2042500  -0.51521100
## 2 -5.40784e-01  -5.00470000   3.9466100  -8.0002700  -5.7376500  -3.98934000
## 3  5.17499e+00   1.64256000   0.1699090   1.6542200  -0.2425450   4.36133000
## 4  9.56254e-01   1.56088000   0.9974650  -3.2671800  -0.9238730  -0.50113900
## 5  3.68929e+00   2.86528000  -1.0041900  -0.8197480   1.5807100  -2.30744000
## 6 -3.76693e+00  -2.22085000  -2.9309000   4.4110100   1.4632700  -1.42867000
##          PC180        PC181       PC182        PC183        PC184       PC185
## 1   1.40223000  -0.65724700  -2.2840700  -4.18823000  -0.86105800   0.8483300
## 2  11.85860000  -7.14679000   4.6139300  24.99140000  13.90510000  53.3265000
## 3  -5.55482000  -1.98449000  -0.1362050  -3.22393000   1.89783000  -0.3886590
## 4  -2.59195000   0.47564500  -2.9618000   0.50321000   0.12141000  -3.6282800
## 5  -0.09715940   1.55499000   0.7971850  -2.64665000  -1.08346000  -1.4480800
## 6   2.96903000   0.99152700   0.2385350   0.40988500   0.56802300   2.0789500
##          PC186        PC187        PC188       PC189        PC190       PC191
## 1  -0.24489900   1.84136000 -2.80263e-01   1.8546400 -4.43113e-01   1.9208400
## 2  51.53680000 -17.32120000  5.02855e+00 -26.5415000  1.46635e+01  -2.2318900
## 3   0.44920700   0.65721700  8.46501e-01   1.5952600 -2.57029e+00   0.7854340
## 4  -1.44676000   2.56409000 -1.87040e+00   0.4244260  5.71903e-01   1.0444200
## 5  -0.56481600  -0.91672900 -2.27838e+00   0.5412340  1.78439e+00  -1.1764100
## 6  -0.31211400  -2.49429000 -9.40577e-01  -1.1041800  7.28286e-01   0.9416920
##          PC192        PC193        PC194       PC195       PC196       PC197
## 1  -0.87984600   0.50451800  -2.86036000   0.7874190   0.9720960  -0.7877650
## 2  -4.55282000   2.50351000  12.19390000  -3.3179900  -3.4685000   2.2320300
## 3   1.38094000   3.15353000  -0.76531500   0.8585230   0.7344950  -0.4920300
## 4   0.82732100   2.63986000  -1.71353000  -0.0159541   0.9678460   3.5549800
## 5   1.19707000   0.22894700  -1.24979000   0.3317780   0.1297840  -0.3758370
## 6   0.08370250  -0.85934200   1.24761000  -0.2231790   0.0428804  -1.0484100
##          PC198        PC199       PC200        PC201       PC202        PC203
## 1 -1.99684e-01  -1.11885000   0.9397460  -0.45154300  -1.3621900 -5.14614e-01
## 2 -3.49332e+00   0.73142000  -6.2420500   1.74009000  -1.5648700 -3.18236e-01
## 3 -2.44411e+00  -2.22062000   0.2857870   0.76019000  -0.5174860  2.58793e-02
## 4 -1.04568e+00  -1.31075000  -2.4926200   1.27123000  -1.0163900 -1.87660e+00
## 5  4.07572e-01   1.98122000  -0.3409920  -0.34483100   0.5092150 -8.46287e-02
## 6  8.17099e-01  -0.50323400   1.9966000  -0.97070300   1.3177200  1.33346e+00
##         PC204        PC205        PC206       PC207        PC208        PC209
## 1   0.9098230  -0.41043800  -2.00797000   1.1352300  -1.11808000  5.81459e-01
## 2   1.0411400   1.46901000  -0.23460100   1.2740300   2.81294000  1.93087e+00
## 3   0.2211800  -0.46630100   0.44417200   0.1508940   0.41571600 -1.31405e-02
## 4   0.7608390   5.58871000   9.62258000   2.0937900   9.72991000  7.71622e+01
## 5   1.4843300  -1.00699000   0.45052500   1.7733600  -1.58494000  1.68549e+00
## 6  -0.4368020  -5.70147000  -8.55191000  -2.6887200  -9.77064000 -7.68781e+01
##          PC210        PC211        PC212        PC213        PC214       PC215
## 1  -0.65222600   0.14640400  -0.22558600   0.16811200  1.18610e+00   0.5856490
## 2  -0.56022600   0.26494000   0.25803000  -1.62597000 -3.15615e-02  -1.1726300
## 3   1.32290000   0.40683800   0.08621740   0.65804400 -4.99558e-01  -0.4401960
## 4   4.45557000  -5.12672000  -7.00580000  -8.29989000 -2.84811e-01  -4.3650700
## 5  -1.10715000  -0.05929860  -1.35709000  -1.03973000 -1.02182e-01   0.2050010
## 6  -5.22369000   4.88191000   6.94937000   7.20167000 -7.07169e-02   3.0153300
##          PC216        PC217        PC218        PC219        PC220        PC221
## 1  -0.16960000   1.37399000   0.83040600 -2.48006e-01   0.47006400  1.02705e-01
## 2   0.74501200   0.63887700   0.24709000 -7.38958e-01  -0.02827680  2.72054e-01
## 3   0.35711800  -0.71013400   0.07146780  6.25675e-01  -0.30353300 -8.36693e-01
## 4   4.38679000  -1.62975000   2.37741000  1.13742e+00   1.77996000 -1.13534e+00
## 5   0.58847100   0.45009100   0.30454700  8.72864e-01   0.36281200 -1.44273e-01
## 6  -3.34138000   1.43260000  -1.57846000 -9.23672e-01  -1.83440000  8.54572e-01
##          PC222        PC223        PC224        PC225        PC226        PC227
## 1   0.13071500  -0.66060400  2.64907e-01   0.05866200   0.15490500  9.96340e-02
## 2   1.03223000  -0.67367400 -3.89672e-01   0.42516600   0.39389900  7.26387e-01
## 3  -0.24599200  -0.99939100  8.09871e-01  -0.37959700   0.27758400  2.05464e-01
## 4   1.21815000  -1.68822000  3.75583e-01   1.17819000  -1.01624000 -7.51697e-01
## 5  -0.43971500  -0.22348700 -3.23138e-01  -0.40193300  -0.13333400 -2.01302e-03
## 6  -1.65523000   1.56887000 -2.17489e-01  -0.12973700   1.10500000  2.48154e-01
##          PC228        PC229        PC230        PC231        PC232        PC233
## 1  8.94705e-01 -7.89690e-01   0.62239100   0.30400600  2.63599e-01  -0.04869920
## 2 -5.05877e-02  8.53152e-01  -0.00692285   0.03505550  4.56803e-01   0.03960260
## 3 -3.60440e-01  1.54412e-01  -0.03874390   0.23335300  2.13369e-01  -0.02447320
## 4  1.80893e-01  1.68740e-01   0.92470400  -0.67433500 -1.41894e-01  -0.17159700
## 5 -5.95439e-01 -8.82056e-02   0.59316400  -0.12300700 -2.30047e-01   0.03627420
## 6 -8.42116e-01 -3.43788e-02  -1.97393000   0.75467800 -7.31736e-01   0.08200520
##          PC234        PC235        PC236 PC237 Individual     region  Pop_City
## 1   0.07203700 -4.28714e-02   0.05709050   NAN          a South Asia Bengaluru
## 2   0.20814300  3.70834e-01  -0.16512800   NAN        257 South Asia Bengaluru
## 3  -0.24580000 -2.51930e-01   0.02853340   NAN        261 South Asia Bengaluru
## 4   0.08117620  4.23416e-02  -0.03686370   NAN        263 South Asia Bengaluru
## 5   0.06813150  1.74180e-01  -0.12070000   NAN        264 South Asia Bengaluru
## 6  -0.38657400 -2.79999e-01   0.03980820   NAN        262 South Asia Bengaluru
##   Country Latitude Longitude Region
## 1   India  12.9716   77.5946   Asia
## 2   India  12.9716   77.5946   Asia
## 3   India  12.9716   77.5946   Asia
## 4   India  12.9716   77.5946   Asia
## 5   India  12.9716   77.5946   Asia
## 6   India  12.9716   77.5946   Asia
```

```
# source the plotting function
source(
  here(
    "scripts", "analysis", "my_theme2.R"
  )
)

distinct_palette <- c(
  "#E69F00",
  "#009E73",
  "#0072B2",
  "#CC79A7",
  "#800000",
  "#808080",
  "#CCEBC5",
  "#FFB5B8",
  "#99CCFF",
  "#8E8BFF",
  "#F781BF",
  "#FFFF33",
  "#A65628",
  "#984EA3",
  "#D2D2D2",
  "#4DAF4A",
  "#DEB887",  # BurlyWood
  "#7FFFD4",  # Aquamarine
  "#D2691E",  # Chocolate
  "#DC143C",  # Crimson
  "#8B008B"   # DarkMagenta
)
merged_data$PC1 <- as.numeric(as.character(merged_data$PC1))
merged_data$PC2 <- as.numeric(as.character(merged_data$PC2))
merged_data$PC3 <- as.numeric(as.character(merged_data$PC3))

# make plot by continent and range
ggplot(merged_data, aes(PC1, PC2)) +
  geom_point(aes(colour = Country, shape = Country), size = 1) +
  xlab(paste0("PC1 (", perc[1], " Variance)")) +
  ylab(paste0("PC2 (", perc[2], " Variance)")) +
  labs(
    caption = "PCA with 57,780 SNPs of 237 mosquitoes from 28 localities in Asia."
  ) +
  guides(
    color = guide_legend(title = "Country", ncol = 3),
    shape = guide_legend(title = "Country", ncol = 3),
    fill = guide_legend(title = "Region", ncol = 1)
  ) +
  stat_ellipse(aes(fill = region, group = region), geom = "polygon", alpha = 0.2, level = 0.8) +
  scale_color_manual(values = distinct_palette) +
  scale_shape_manual(values=good.shapes[c(1:25, 58:67)]) +
  my_theme() +
  theme(
    plot.caption = element_text(face = "italic"),
    legend.position = "top",
    legend.justification = "top",
    legend.box.just = "center",
    legend.box.background = element_blank(),
    plot.margin = margin(5.5, 25, 5.5, 5.5, "points"),
    legend.margin = margin(10,10,10,10)
  )
```

```
# #   ____________________________________________________________________________
# #   save the pca plot                                                       ####
ggsave(
  here(
    "output", "populations", "figures", "PCA_lea_pc1_pc2_r2_0.1.pdf"
  ),
  width  = 8,
  height = 6,
  units  = "in"
)
```

PC1 an PC3

```
# source the plotting function
source(
  here(
    "scripts", "analysis", "my_theme2.R"
  )
)

distinct_palette <- c(
  "#E69F00",
  "#009E73",
  "#0072B2",
  "#CC79A7",
  "#800000",
  "#808080",
  "#CCEBC5",
  "#FFB5B8",
  "#99CCFF",
  "#8E8BFF",
  "#F781BF",
  "#FFFF33",
  "#A65628",
  "#984EA3",
  "#D2D2D2",
  "#4DAF4A",
  "#DEB887",  # BurlyWood
  "#7FFFD4",  # Aquamarine
  "#D2691E",  # Chocolate
  "#DC143C",  # Crimson
  "#8B008B"   # DarkMagenta
)


# make plot by continent and range
ggplot(merged_data, aes(PC1, PC2)) +
  geom_point(aes(colour = Country, shape = Country), size = 1) +
  xlab(paste0("PC1 (", perc[1], " Variance)")) +
  ylab(paste0("PC3 (", perc[3], " Variance)")) +
  labs(
    caption = "PCA with 57,780 SNPs of 237 mosquitoes from 28 localities in Asia."
  ) +
  guides(
    color = guide_legend(title = "Country", ncol = 3),
    shape = guide_legend(title = "Country", ncol = 3),
    fill = guide_legend(title = "Region", ncol = 1)
  ) +
  stat_ellipse(aes(fill = region, group = region), geom = "polygon", alpha = 0.2, level = 0.8) +
  scale_color_manual(values = distinct_palette) +
  scale_shape_manual(values=good.shapes[c(1:25, 58:67)]) +
  my_theme() +
  theme(
    plot.caption = element_text(face = "italic"),
    legend.position = "top",
    legend.justification = "top",
    legend.box.just = "center",
    legend.box.background = element_blank(),
    plot.margin = margin(5.5, 25, 5.5, 5.5, "points"),
    legend.margin = margin(10,10,10,10)
  )
```

```
# #   ____________________________________________________________________________
# #   save the pca plot                                                       ####
ggsave(
  here(
    "output", "populations", "figures", "PCA_lea_pc1_pc3_r2_0.1.pdf"
  ),
  width  = 8,
  height = 6,
  units  = "in"
)
```

Run LEA

```
# set output dir
setwd(
  here(
    "output", "populations"
  )
)
# main options
# K = number of ancestral populations
# entropy = TRUE computes the cross-entropy criterion, # CPU = 6 is the number of CPU used (hidden input) project = NULL
project = snmf(
  genotype,
  K = 1:15,
  project = "new",
  repetitions = 5,
  CPU = 4,
  entropy = TRUE
)
```

Load project

```
# To load the project, use:
project = load.snmfProject("output/populations/snps_sets/r2_0.1.snmfProject")

# To remove the project, use:
 # remove.snmfProject("snps_sets/r2_0.1.snmfProject")
```

Cross entropy

```
# Open a new pdf file
pdf(here("output","populations","figures","lea_cross_entropy_r2_0.1.pdf"), width = 6, height = 4)

# Create your plot
plot(project, col = "pink", pch = 19, cex = 1.2)

# Close the pdf file
dev.off()
plot(project, col = "pink", pch = 19, cex = 1.2)
```

We will not plot k=8 but only the k=5 to compare to admixture

#### 3.1 k5

We can plot k=5 to compare to other algorithms

Find the best run

```
best8 = which.min(cross.entropy(project, K = 8)) # 5
best5 = which.min(cross.entropy(project, K = 5)) # 5
best8
```

```
## [1] 5
```

```
best5
```

```
## [1] 5
```

Run 3 for k=5

```
# Extract ancestry coefficients
leak5 <- read_delim(
  here("output", "populations", "snps_sets", "r2_0.1.snmf", "K5", "run5","r2_0.1_r5.5.Q"),
  delim = " ", # Specify the delimiter if different from the default (comma)
  col_names = FALSE,
  show_col_types = FALSE
) 
# unseen_pckmeans.7.Q
# pckmeans.7.Q
head(leak5)
```

```
## # A tibble: 6 × 5
##        X1     X2    X3       X4    X5
##     <dbl>  <dbl> <dbl>    <dbl> <dbl>
## 1 0.00901 0.134  0.601 0.0199   0.236
## 2 0.0206  0.155  0.568 0.0289   0.227
## 3 0.0175  0.103  0.711 0.000100 0.169
## 4 0.00882 0.0695 0.777 0.0136   0.131
## 5 0.00145 0.0634 0.808 0.0136   0.113
## 6 0.0128  0.104  0.723 0.000100 0.160
```

The fam file

```
fam_file <- here(
  "output", "populations", "snps_sets", "r2_0.1.fam"
)

# Read the .fam file
fam_data <- read.table(fam_file, 
                       header = FALSE,
                       col.names = c("FamilyID", "IndividualID", "PaternalID", "MaternalID", "Sex", "Phenotype"))

# View the first few rows
head(fam_data)
```

```
##   FamilyID IndividualID PaternalID MaternalID Sex Phenotype
## 1      OKI         1001          0          0   2        -9
## 2      OKI         1002          0          0   2        -9
## 3      OKI         1003          0          0   2        -9
## 4      OKI         1004          0          0   2        -9
## 5      OKI         1005          0          0   2        -9
## 6      OKI         1006          0          0   1        -9
```

Create ID column

```
# Change column name
colnames(fam_data)[colnames(fam_data) == "IndividualID"] <- "ind"


# Merge columns "FamilyID" and "IndividualID" with an underscore
# fam_data$ind <- paste(fam_data$FamilyID, fam_data$IndividualID, sep = "_")


# Change column name
colnames(fam_data)[colnames(fam_data) == "FamilyID"] <- "pop"

# Select ID
fam_data <- fam_data |>
  dplyr::select("ind", "pop")

# View the first few rows
head(fam_data)
```

```
##    ind pop
## 1 1001 OKI
## 2 1002 OKI
## 3 1003 OKI
## 4 1004 OKI
## 5 1005 OKI
## 6 1006 OKI
```

Add it to matrix

```
leak5 <- fam_data |>
  dplyr::select(ind, pop) |>
  bind_cols(leak5)

head(leak5)
```

```
##    ind pop         X1        X2       X3          X4       X5
## 1 1001 OKI 0.00901176 0.1344870 0.600824 0.019856000 0.235822
## 2 1002 OKI 0.02059050 0.1549340 0.568124 0.028927700 0.227423
## 3 1003 OKI 0.01745480 0.1030830 0.710789 0.000099991 0.168573
## 4 1004 OKI 0.00881626 0.0694981 0.777402 0.013613100 0.130670
## 5 1005 OKI 0.00145040 0.0633980 0.808295 0.013615700 0.113241
## 6 1006 OKI 0.01277640 0.1043520 0.723096 0.000099991 0.159675
```

Rename the columns

```
# Rename the columns starting from the third one
leak5 <- leak5 |>
  rename_with(~paste0("v", seq_along(.x)), .cols = -c(ind, pop))

# View the first few rows
head(leak5)
```

```
##    ind pop         v1        v2       v3          v4       v5
## 1 1001 OKI 0.00901176 0.1344870 0.600824 0.019856000 0.235822
## 2 1002 OKI 0.02059050 0.1549340 0.568124 0.028927700 0.227423
## 3 1003 OKI 0.01745480 0.1030830 0.710789 0.000099991 0.168573
## 4 1004 OKI 0.00881626 0.0694981 0.777402 0.013613100 0.130670
## 5 1005 OKI 0.00145040 0.0633980 0.808295 0.013615700 0.113241
## 6 1006 OKI 0.01277640 0.1043520 0.723096 0.000099991 0.159675
```

Import sample locations

```
sampling_loc <- readRDS(here("output", "local_adaptation", "sampling_loc.rds"))
head(sampling_loc)
```

```
## # A tibble: 6 × 6
##   Pop_City   Country  Latitude Longitude Region Abbreviation
##   <chr>      <chr>       <dbl>     <dbl> <chr>  <chr>       
## 1 Gelephu    Bhutan       26.9      90.5 Asia   GEL         
## 2 Phnom Penh Cambodia     11.6     105.  Asia   CAM         
## 3 Hainan     China        19.2     110.  Asia   HAI         
## 4 Yunnan     China        24.5     101.  Asia   YUN         
## 5 Hunan      China        27.6     112.  Asia   HUN         
## 6 Bengaluru  India        13.0      77.6 Asia   BEN
```

```
source(
  here(
    "scripts", "analysis", "my_theme3.R"
  )
)

# Create a named vector to map countries to regions
country_to_region <- c(
  "Bhutan" = "South Asia",
  "Cambodia" = "Southeast Asia",
  "China" = "East Asia",
  "India" = "South Asia",
  "Indonesia" = "Southeast Asia",
  "Japan" = "East Asia",
  "Malaysia" = "Southeast Asia",
  "Maldives" = "South Asia",
  "Nepal" = "South Asia",
  "Sri Lanka" = "South Asia",
  "Taiwan" = "East Asia",
  "Thailand" = "Southeast Asia",
  "Vietnam" = "Southeast Asia"
)

# Add the region to the data frame
sampling_loc$Region2 <- country_to_region[sampling_loc$Country]

# Melt the data frame for plotting
Q_melted <- leak5 |>
  pivot_longer(
    cols = -c(ind, pop),
    names_to = "variable",
    values_to = "value"
  )
# Join with sampling_loc to get sampling localities
Q_joined <- Q_melted |>
  left_join(sampling_loc, by = c("pop" = "Abbreviation"))

# Create a combined variable for Region and Country
Q_joined <- Q_joined |>
  mutate(Region_Country = interaction(Region, Country, sep = "_"))

# Order the combined variable by Region and Country, then by individual
Q_ordered <- Q_joined |>
  arrange(Region, Region2, Country, ind) |>
  mutate(ind = factor(ind, levels = unique(ind)))  # Convert ind to a factor with levels in the desired order

# Add labels: country names for the first individual in each country, NA for all other individuals
Q_ordered <- Q_ordered |>
  group_by(Region_Country) |>
  mutate(label = ifelse(row_number() == 1, as.character(Country), NA))

# Group by individual and variable, calculate mean ancestry proportions
Q_grouped <- Q_ordered |>
  group_by(ind, variable) |>
  summarise(value = mean(value), .groups = "drop")

# Create a data frame for borders
borders <-
  data.frame(Region_Country = unique(Q_ordered$Region_Country))

# Add the order of the last individual of each country to ensure correct placement of borders
borders$order <-
  sapply(borders$Region_Country, function(rc)
    max(which(Q_ordered$Region_Country == rc))) + 0.5  # Shift borders to the right edge of the bars

# Select only the first occurrence of each country in the ordered data
label_df <- Q_ordered |>
  filter(!is.na(label)) |>
  distinct(label, .keep_all = TRUE)

# Create a custom label function
label_func <- function(x) {
  labels <- rep("", length(x))
  labels[x %in% label_df$ind] <- label_df$label
  labels
}

# Calculate the position of lines
border_positions <- Q_ordered |>
  group_by(Country) |>
  summarise(pos = max(as.numeric(ind)) + 0)

# Calculate the position of population labels and bars
pop_labels <- Q_ordered |>
  mutate(Name = paste(pop, Pop_City, sep = " - ")) |>
  group_by(pop) |>
  slice_head(n = 1) |>
  ungroup() |>
  dplyr::select(ind, Pop_City, Country, Name) |>
  mutate(pos = as.numeric(ind))  # calculate position of population labels

pop_labels_bars <- pop_labels |>
  mutate(pos = as.numeric(ind)  - .5)


# Calculate the position of lines
border_positions <- Q_ordered |>
  group_by(Country) |>
  summarise(pos = max(as.numeric(ind)) - 1)


pop_labels_bars <- pop_labels |>
  mutate(pos = as.numeric(ind)  - .5)

# Function to filter and normalize data
normalize_data <- function(df, min_value) {
  df |>
    filter(value > min_value) |>
    group_by(ind) |>
    mutate(value = value / sum(value))
}

# Use the function
Q_grouped_filtered <- normalize_data(Q_grouped, 0.1)
# 
color_palette <-
  c(
    "v1" = "red",
    "v2" = "#F49AC2",
    "v3" = "#AE9393",
    "v4" = "#FFFF99",
    "v5" = "#FFB347"
  )


# Generate all potential variable names
all_variables <- paste0("v", 1:5)

# Map each variable to a name
color_mapping <- data.frame(variable = all_variables,
                            color = names(color_palette))

# Merge with Q_grouped_filtered
Q_grouped_filtered <- merge(Q_grouped_filtered, color_mapping, by = "variable")

# Create the plot
ggplot(Q_grouped_filtered, aes(x = as.factor(ind), y = value, fill = color)) +
  geom_bar(stat = 'identity', width = 1) +
  geom_vline(
    data = pop_labels_bars,
    aes(xintercept = pos),
    color = "#2C444A",
    linewidth = .2
  ) +
  geom_text(
    data = pop_labels,
    aes(x = as.numeric(ind), y = 1, label = Name),
    vjust = 1.5,
    hjust = 0,
    size = 2,
    angle = 90,
    inherit.aes = FALSE
  ) +
  my_theme() +
  theme(
    axis.text.x = element_text(
      angle = 90,
      hjust = 1,
      size = 12
    ),
    legend.position = "none",
    plot.margin = unit(c(3, 0.5, 0.5, 0.5), "cm")
  ) +
  xlab("Admixture matrix") +
  ylab("Ancestry proportions") +
  labs(caption = "Each bar represents the ancestry proportions for an individual for k=5.\n LEA inference for k1:15 with SNPs prunned with r2 0.1.") +
  scale_x_discrete(labels = label_func) +
  scale_fill_manual(values = color_palette) +
  expand_limits(y = c(0, 1.5))
```

```
ggsave(
  here("output", "populations", "figures", "lea_k=5_r2_0.1.pdf"),
  width  = 12,
  height = 6,
  units  = "in",
  device = cairo_pdf
)
```
