# Supplementary material for: A genotyping array for the globally invasive vector mosquito, Aedes albopictus
Source: Parasit Vectors. 2024 Mar 4;17:106. doi: 10.1186/s13071-024-06158-z (PMC10910840; doi:10.1186/s13071-024-06158-z)
Supplement: Supplementary file 11 — Additional file 11. fastStructure analysis. [file 13071_2024_6158_MOESM11_ESM.html]

Aedes albopictus SNP chip - Ancestry analysis with fastStructure


# Aedes albopictus SNP chip - Ancestry analysis with fastStructure

#### Luciano V Cosme

#### 2023-08-28

- 1. R libraries and
  installing fastStructure
- 2. Run
  fastStructure
  - 2.1 Neutral set
    - 2.1.1 Simple
      prior
    - 2.1.2 Logistic
      prior
    - 2.1.3 Generate the dsq batch
      files
    - 2.1.4 Parse files from runs
    - 2.1.5
      Choose k simple plot
    - 2.1.6 Choose k logistic plot
    - 2.1.7
      Statistical tests - comparing simple and logistic prior runs
    - 2.1.8 pong
      - 2.1.8.1 simple
        prior
      - 2.1.8.2
        logistic prior
  - 2.2 r2 0.01 set
    - 2.2.1 Simple
      prior
    - 2.2.2 Logistic
      prior
    - 2.2.3 Generate the dsq batch
      files
    - 2.2.4 Parse files from runs
    - 2.2.5 Choose k simple plot
    - 2.2.6 Choose
      k logistic
    - 2.2.7
      Statistical tests - comparing simple and logistic prior runs
    - 2.2.8 pong
      - 2.2.8.1 simple
        prior
      - 2.2.8.1
        logistic prior
  - 2.3 r2 0.1 set
    - 2.3.1 Simple
      prior
    - 2.3.2 Logistic
      prior
    - 2.3.3 Generate the dsq batch
      files
    - 2.3.4 Parse files from runs
    - 2.3.5 Choose k simple plot
    - 2.3.6
      Choose k logistic
    - 2.3.7
      Statistical tests - comparing simple and logistic prior runs
    - 2.3.8 pong
      - 2.3.8.1 simple
        prior
      - 2.3.8.1
        logistic prior

## 1. R libraries and installing fastStructure

```
library(tidyverse)
library(here)
library(colorout)
library(dplyr)
library(ggplot2)
library(extrafont)
library(hrbrthemes)
library(hrbrmisc)
library(ggstatsplot)
library(flextable)
library(officer)
```

Our first step is to create a conda environment and install
fastStructure on the cluster.

```
# first create a interactive session with 4 CPU's and 20GB memory
salloc --time=01:00:00 --nodes=1 --ntasks=1 --cpus-per-task=1 --mem-per-cpu=5120
cd /home/lvc26/project
# download fastStructure in a directory.
git clone https://github.com/rajanil/fastStructure
# navigate to fastStructure dir
cd fastStructure
# then load miniconda
module load miniconda/23.3.1
# create new environment
conda create --name fastStructure -c bioconda python=2.7
# activate the new env
conda activate fastStructure
# to deactivate it later
conda deactivate
# if you want to remove it
# conda env remove -n fastStructure
```

Check the required fastStructure dependencies at https://github.com/rajanil/fastStructure

```
fastStructure depends on

Numpy
Scipy
Cython
GNU Scientific Library
```

Install dependencies.

```
# scipy and numpy (it already installs numpy)
pip install scipy
# Successfully installed numpy-1.16.6 scipy-1.2.3
# cython
pip install cython==0.27.3
# gsl
conda install gsl
#
# identify the path to the library files libgsl.so and libgslcblas.so, and header file gsl/gsl_sf_psi.h that are part of your GSL installation.
# run to check the options
gsl-config -
#################################################################################################
# get paths - on Mccleary
gsl-config --prefix
# /gpfs/gibbs/project/powell/lvc26/conda_envs/fastStructure
gsl-config --libs
# -L/gpfs/gibbs/project/powell/lvc26/conda_envs/fastStructure/lib -lgsl -lgslcblas -lm
gsl-config --cflags
# -I/gpfs/gibbs/project/powell/lvc26/conda_envs/fastStructure/include
# export their paths
export LD_LIBRARY_PATH=$LD_LIBRARY_PATH:/gpfs/gibbs/project/powell/lvc26/conda_envs/fastStructure
export CFLAGS="-I/gpfs/gibbs/project/powell/lvc26/conda_envs/fastStructure/include"
export LDFLAGS="-L/gpfs/gibbs/project/powell/lvc26/conda_envs/fastStructure/lib -lgsl -lgslcblas -lm"

#
# Then set these environment variables
source ~/.bashrc
# navigate to the directory to compile inside faststructure dir
cd /home/lvc26/project/fastStructure/vars
# compile
python setup.py build_ext -f --inplace
# it might give you some warnings
# To compile the main cython scripts, navigate to fastStructure directory (cd .. from the vars directory)
cd ..
python setup.py build_ext -f --inplace
# it will give you several warnings
# last step is to check if it works
python structure.py
# change rights
chmod 775 structure.py chooseK.py
# check python path
echo $PYTHONPATH
# then to run it from other places
# add fastStructure to the python path
export PYTHONPATH=/home/lvc26/project/fastStructure
# Then set these environment variables
source ~/.bashrc
# now from any directory you can call fastStructure using -m (module)
python -m structure # your parameters
```

```
Here is how you can use this script

Usage: python structure.py
     -K <int> (number of populations)
     --input=<file> (/path/to/input/file)
     --output=<file> (/path/to/output/file)
     --tol=<float> (convergence criterion; default: 10e-6)
     --prior={simple,logistic} (choice of prior; default: simple)
     --cv=<int> (number of test sets for cross-validation, 0 implies no CV step; default: 0)
     --format={bed,str} (format of input file; default: bed)
     --full (to output all variational parameters; optional)
     --seed=<int> (manually specify seed for random number generator; optional)
```

How to use fastStructure interactively or via batch script.

```
salloc --time=01:00:00 --nodes=1 --ntasks=1 --cpus-per-task=1 --mem-per-cpu=5120
module load miniconda/23.3.1
conda activate fastStructure
export PYTHONPATH=/home/lvc26/project/fastStructure
# export PYTHONPATH=/home/lvc26/project/fastStructure
python -m structure # rest of the command
# export PATH=$PATH:/ycga-gpfs/project/powell/lvc26/fastStructure
# then you are ready to run it.
# if you are using a batch script, then just use:
source /vast/palmer/apps/avx2/software/miniconda/23.3.1/etc/profile.d/conda.sh;
module load miniconda/23.3.1
conda activate fastStructure
export PYTHONPATH=/home/lvc26/project/fastStructure
# if it does not work, check your miniconda installation
# to get information about your miniconda on the clusters (probably is similar to mine)
conda info | grep -i 'base environment'
```

## 2. Run fastStructure

We ran fastStructure with several data sets testing k1:30 with 100
runs. Now, we can run fastStructure with the final data sets

### 2.1 Neutral set

Create a directory for each run

```
cd /gpfs/ycga/project/caccone/lvc26/faststructure/neutral
for i in $(seq -w 1 10)
do
  mkdir run$i
done
```

Now we can generate the full script for dsq (not showing here,
because it has 2,000 lines).

#### 2.1.1 Simple prior

```
for k in $(seq 1 30); do
    for run in $(seq -w 1 10); do
        echo "cd /gpfs/ycga/project/caccone/lvc26/faststructure; source /vast/palmer/apps/avx2/software/miniconda/23.3.1/etc/profile.d/conda.sh;module load miniconda/23.3.1; conda activate fastStructure; export PYTHONPATH=/home/lvc26/project/fastStructure; python -m structure -K $k --input=/gpfs/ycga/project/caccone/lvc26/neuroadmix/snps_sets/neutral --output=/gpfs/ycga/project/caccone/lvc26/faststructure/neutral/run$run/simple --prior=simple --full --cv=10 --tol=10e-6"
    done
done > dsq1.txt
```

#### 2.1.2 Logistic prior

I used 10 runs for the neutral set because it is around 9k SNPs. For
other data sets we can do less runs because it takes too long.

```
for k in $(seq 1 30); do
    for run in $(seq -w 1 10); do
        echo "cd /gpfs/ycga/project/caccone/lvc26/faststructure; source /vast/palmer/apps/avx2/software/miniconda/23.3.1/etc/profile.d/conda.sh;module load miniconda/23.3.1; conda activate fastStructure; export PYTHONPATH=/home/lvc26/project/fastStructure; python -m structure -K $k --input=/gpfs/ycga/project/caccone/lvc26/neuroadmix/snps_sets/neutral --output=/gpfs/ycga/project/caccone/lvc26/faststructure/neutral/run$run/logistic --prior=logistic --full --cv=10 --tol=10e-6"
    done
done > dsq2.txt
```

Load dsq, and then create the batch file. Check the dsq –help

#### 2.1.3 Generate the dsq batch files

```
module load dSQ/1.05
##############################################
#                 Simple
##############################################
dsq \
--job-file dsq1.txt \
--mem-per-cpu 1g \
--cpus-per-task=1 \
-t 24:00:00 \
--mail-type END \
--partition=scavenge \
--job-name fastStr_s \
--output /dev/null \
--batch-file 1.simple.sh

##############################################
#                 Logistic
##############################################
dsq \
--job-file dsq2.txt \
--mem-per-cpu 1g \
--cpus-per-task=1 \
--time 7-00:00:00 \
--mail-type END \
--partition=week \
--job-name fastStr_L \
--output /dev/null \
--batch-file 2.logistic.sh
```

Before we submit all jobs, it is a good practice to submit only one
job of the array and see if it runs without problems.

```
# We will run a random job from our array to see if works
#sbatch --array=$((1 + $RANDOM % 2000)) 1.simple.sh  # for random job
# or 
sbatch --array=1 1.simple.sh  # to run the first job
# check status
dsqa -j 6273617 # add job number here
```

If it runs without problems, we can submit all jobs.

```
sbatch 1.simple.sh
# Submitted batch job 6273630
sbatch 2.logistic.sh
# Submitted batch job 36273940
```

Check the jobs status.

```
# check status
dsqa -j 6273630 # simple
dsqa -j 6273940 # logistic all completed :)
```

Run autopsy once it is done.

```
# simple, all completed
# logistic, some jobs timed out
# get the jobs we need to re-run
dsqa -j 37569748 -f dsq2.txt -s TIMEOUT > rerun_jobs.txt; wc -l rerun_jobs.txt # 30 jobs
# re submit the 30 jobs 
dsq \
--job-file rerun_jobs.txt \
--mem-per-cpu 1g \
--cpus-per-task=1 \
-t 120:00:00 \
--status-dir /gpfs/ysm/project/powell/lvc26/albopictus/fastStructure/logs \
--mail-type END \
--partition=general \
--job-name logistic2 \
--output logs/logistic_rerun-%A_%a-%N.txt \
--batch-file 2.1logistic.sh \
--submit
#
# check status
dsqa -j 38086185
```

#### 2.1.4 Parse files from runs

Next, we will need to parse the results and make a plot. Our fist
step is done on the cluster. We need to grab the Q matrices and the log
files from our runs. We start wit the simple priors. We first find the
optimal number of Ks. Then we use pong to parse all runs and generate
one structure like plot. You can get pong here https://github.com/ramachandran-lab/pong. I installed
pong on my base environment in my laptop. You can create am environment
if you want: check their manual to follow instructions.

Data location

```
cd /gpfs/ycga/project/caccone/lvc26/faststructure/neutral
```

#### 2.1.5 Choose k simple plot

Our first step is to use fastStructure script chooseK.py to find the
number of ancestral populations. The main reason we did 10 runs is
because we don’t find the same optimal k for every run. Since we already
created a conda environment to run fastStructure, we can activate it,
and run chooseK.

First for our runs with simple prior.

```
salloc --time=06:00:00 --nodes=1 --ntasks=1 --cpus-per-task=1 --mem-per-cpu=5120
module load miniconda/23.3.1
conda activate fastStructure
export PYTHONPATH=/home/lvc26/project/fastStructure


# simple 10 runs
cat <(echo 'run likelihood structure') \
<(for i in /gpfs/ycga/project/caccone/lvc26/faststructure/neutral/run*
do
    echo $i | sed 's@.*/@@' | sed 's/run/run /' ; python -m chooseK --input=$i/simple | grep 'likelihood' | awk '{print $6, $8}'; python -m chooseK --input=$i/simple | grep 'structure' |  awk '{print $6, $10}'
done | xargs -n6 | awk '{print $2, $4, $6}') > simple_prior_models.txt

# logistic 10 runs
cat <(echo 'run likelihood structure') \
<(for i in /gpfs/ycga/project/caccone/lvc26/faststructure/neutral/run*
do
    echo $i | sed 's@.*/@@' | sed 's/run/run /' ; python -m chooseK --input=$i/logistic | grep 'likelihood' | awk '{print $6, $8}'; python -m chooseK --input=$i/logistic | grep 'structure' |  awk '{print $6, $10}'
done | xargs -n6 | awk '{print $2, $4, $6}') > logistic_prior_models.txt
```

Download the data

```
rsync -chavzP --stats lvc26@mccleary.ycrc.yale.edu:/gpfs/ycga/project/caccone/lvc26/faststructure/neutral/*_prior_models.txt /Users/lucianocosme/Library/CloudStorage/Dropbox/Albopictus/manuscript_chip/data/no_autogenous/albo_chip/output/populations/faststructure/neutral
```

We can make a plot with the Ks. Let’s import the data in R.

```
# function to import our choosek.py data
import_fastStructure <- function(file) {
  # import as a tibble and set columns as integers
  dat <- read_delim(
    file,
    col_names      = TRUE,
    show_col_types = FALSE,
    col_types      = "iii"
  )

  # get columns we need and make it long for plotting
  dat <- dat |>
    gather(
      structure, likelihood, -run
    )

  # rename the columns by index
  dat <- dat |>
    rename(
      run   = 1,
      model = 2,
      k     = 3
    )
  return(dat)
}
# we can save the function to source it later
dump(
  "import_fastStructure",
  here(
    "scripts", "analysis", "import_fastStructure.R"
  ),
  append = TRUE
)
```

Now we can use the function to import the data

```
# we can use our function with here to import the data
choose_k_simple <- 
  import_fastStructure(
  here(
    "output", "populations","fastStructure", "neutral","simple_prior_models.txt"
  )
)
```

Function to plot fastStructure choosek.py results

```
# function to plot our choosek.py data
plot_fastStructure <- function(df) {
  df |>
    ggplot() +
    geom_line(
      aes(
        x              = run,
        y              = k,
        color          = model
      ),
      linewidth = 1
    ) +
    scale_colour_manual(
      "model",
      values = c(
        structure      = "#9d60ff",
        likelihood     = "#ffc08c"
      ),
      labels = c(
        "Maximizes \n Likelihood \n", "Explain \n Structure"
      )
    ) +
    labs(
      x                = "Run",
      y                = "K",
      title            = "fastStructure",
      caption          = "algorithm runs for choices of K ranging from 1 to 30"
    ) +
    hrbrthemes::theme_ipsum(
      base_family = "",
      axis_text_size = 12,
      axis_title_size = 14,
      plot_margin = margin(
        10, 10, 10, 10
      ),
      grid = TRUE,
      grid_col = "#fabbe2"
    ) +
    theme(
      panel.grid.major = element_line(
        linetype       = "dashed",
        linewidth      = 0.2
      ),
      panel.grid.minor = element_line(
        linetype       = "dashed",
        linewidth      = 0.2
      ),
      legend.text = element_text(
        size = 12
      ),
      legend.title = element_text(
        size           = 14,
        face           = "bold"
      ),
      legend.position = "right"
    )
}
# we can save the function to source it later
dump(
  "plot_fastStructure",
  here(
    "scripts", "analysis", "plot_fastStructure.R"
  ),
  append = TRUE
)
```

Use our function to plot k values

```
plot_fastStructure(
  choose_k_simple
) +
  labs(
    subtitle = "simple prior"
  )
```

```
# save plot
ggsave(
  here(
    "output", "figures", "fastStructure_neutral_simple_prior_k1_k30.pdf"
  ),
  width  = 8,
  height = 6,
  units  = "in"
)
```

#### 2.1.6 Choose k logistic plot

Import logistic data

```
# import the data
choose_k_logistic <- import_fastStructure(
  here(
    "output", "populations","fastStructure", "neutral","logistic_prior_models.txt"
  )
)
```

Make plot of the data

```
# make plot
plot_fastStructure(
  choose_k_logistic
) +
  labs(
    subtitle = "logistic prior"
  )
```

```
#
# save plot
ggsave(
  here(
    "output", "figures", "fastStructure_neutral_logistic_prior_k1_k30.pdf"
  ),
  width  = 8,
  height = 6,
  units  = "in"
)
```

#### 2.1.7 Statistical tests - comparing simple and logistic prior runs

```
set.seed(123)  # for reproducibility
# import data
# simple
simple1 <- import_fastStructure(
  here(
    "output", "populations","fastStructure", "neutral", "simple_prior_models.txt"
  )
) |>
  mutate(
    prior = "simple"
  )
#
# logistic
logistic1 <- import_fastStructure(
  here(
    "output", "populations","fastStructure", "neutral", "logistic_prior_models.txt"
  )
) |>
  mutate(
    prior = "logistic"
  )
#
# rbind data set
fastStruc_prior <- rbind(
  simple1, logistic1
) |>
  mutate(
    prior = factor(
      prior
    )
  )
#
# run stats
grouped_ggbetweenstats(
  data = fastStruc_prior,
  x = prior,
  y = k,
  grouping.var = model,
  p.adjust.method = "bonferroni",
  palette = "default_jama",
  package = "ggsci",
  plotgrid.args = list(
    nrow = 1
  ),
  annotation.args = list(
    title = "Comparison of fastStructure prior"
  )
)
```

```
# # save plot
ggsave(
  here(
    "output", "populations", "figures", "welch_faststructure_neutral_prior_comparisons.pdf"
  ),
  width = 11,
  height = 8,
  units = "in"
)
```

The number of model components that explain the structure in the data
is different from Admixture. For our models explaining the structure in
the data, we have simple: k=9, and logistic: k=12, whereas Admixture
indicated k=5. We can plot k=5 to see how it compares to the Admxiture,
LEA, and neuro-admixture.

#### 2.1.8 pong

We first need to find the most common mode among our runs. We do not
need to plot all matrices, we can plot the first 12 using pong.

Download the data

```
rsync -chavzP --stats --include='*/' --include='*.meanQ' --exclude='*' lvc26@mccleary.ycrc.yale.edu:/gpfs/ycga/project/caccone/lvc26/faststructure/neutral* /Users/lucianocosme/Library/CloudStorage/Dropbox/Albopictus/manuscript_chip/data/no_autogenous/albo_chip/output/populations/faststructure/
```

##### 2.1.8.1 simple prior

Check the downloaded data

```
ls -1 output/populations/faststructure/neutral/run*/simple* | tail
```

Create filemap2.txt

```
for k in $(seq 2 12); do
    for run in $(seq -w 1 10); do
        echo "k$k""r$run $k faststructure/neutral/run$run/simple.$k.meanQ" | tr ' ' '\t'
    done
done > output/populations/filemap2.txt;
head -n 5 output/populations/filemap2.txt
```

```
wc -l output/populations/filemap2.txt
```

Create ind2pop

```
rm output/populations/ind2pop.txt;
cat output/populations/snps_sets/neutral.fam | awk '{print $1}' > output/populations/ind2pop.txt;
head -n 5 output/populations/ind2pop.txt
```

Run on the terminal and not on RStudio.

```
# on your computer to visualize the results
pong \
--filemap output/populations/filemap2.txt \
--ind2pop output/populations/ind2pop.txt  \
--dist_metric jaccard \
--sim_threshold 0.82 \
--greedy \
--force \
--output_dir output/populations/sim_threshold_0.82 \
--verbose
```

Check for the modes for k=5. Two modes: one with 8 runs and 1 with 2
runs. We can use run01 for our structure plot.

###### 2.1.8.1.1 Create plot simple prior with Q matrix identified with pong

Make plot

```
# Extract ancestry coefficients
k5run1 <- read_delim(
  here("output", "populations", "faststructure", "neutral", "run01", "simple.5.meanQ"),
  delim = "  ", # Specify the delimiter if different from the default (comma)
  col_names = FALSE,
  show_col_types = FALSE
) 

head(k5run1)
```

```
## # A tibble: 6 × 5
##      X1       X2       X3    X4       X5
##   <dbl>    <dbl>    <dbl> <dbl>    <dbl>
## 1 0.561 0.000012 0.000012 0.439 0.000012
## 2 0.479 0.109    0.000012 0.412 0.000012
## 3 0.575 0.000012 0.000012 0.425 0.000012
## 4 0.527 0.000012 0.000012 0.473 0.000012
## 5 0.527 0.000012 0.000012 0.473 0.000012
## 6 0.561 0.000012 0.000012 0.438 0.000012
```

The fam file

```
fam_file <- here(
  "output", "populations", "snps_sets", "neutral.fam"
)

# Read the .fam file
fam_data <- read.table(fam_file, 
                       header = FALSE,
                       col.names = c("FamilyID", "IndividualID", "PaternalID", "MaternalID", "Sex", "Phenotype"))

# View the first few rows
head(fam_data)
```

```
##   FamilyID IndividualID PaternalID MaternalID Sex Phenotype
## 1      OKI         1001          0          0   2        -9
## 2      OKI         1002          0          0   2        -9
## 3      OKI         1003          0          0   2        -9
## 4      OKI         1004          0          0   2        -9
## 5      OKI         1005          0          0   2        -9
## 6      OKI         1006          0          0   1        -9
```

Create ID column

```
# Change column name
colnames(fam_data)[colnames(fam_data) == "IndividualID"] <- "ind"


# Change column name
colnames(fam_data)[colnames(fam_data) == "FamilyID"] <- "pop"

# Select ID
fam_data <- fam_data |>
  dplyr::select("ind", "pop")

# View the first few rows
head(fam_data)
```

```
##    ind pop
## 1 1001 OKI
## 2 1002 OKI
## 3 1003 OKI
## 4 1004 OKI
## 5 1005 OKI
## 6 1006 OKI
```

Add it to matrix

```
k5run1 <- fam_data |>
  dplyr::select(ind, pop) |>
  bind_cols(k5run1)

head(k5run1)
```

```
##    ind pop       X1       X2      X3       X4      X5
## 1 1001 OKI 0.560940 0.000012 1.2e-05 0.439025 1.2e-05
## 2 1002 OKI 0.479157 0.109106 1.2e-05 0.411713 1.2e-05
## 3 1003 OKI 0.574956 0.000012 1.2e-05 0.425009 1.2e-05
## 4 1004 OKI 0.526625 0.000012 1.2e-05 0.473340 1.2e-05
## 5 1005 OKI 0.526835 0.000012 1.2e-05 0.473130 1.2e-05
## 6 1006 OKI 0.561466 0.000012 1.2e-05 0.438499 1.2e-05
```

Rename the columns

```
# Rename the columns starting from the third one
k5run1 <- k5run1 |>
  rename_with(~paste0("v", seq_along(.x)), .cols = -c(ind, pop))

# View the first few rows
head(k5run1)
```

```
##    ind pop       v1       v2      v3       v4      v5
## 1 1001 OKI 0.560940 0.000012 1.2e-05 0.439025 1.2e-05
## 2 1002 OKI 0.479157 0.109106 1.2e-05 0.411713 1.2e-05
## 3 1003 OKI 0.574956 0.000012 1.2e-05 0.425009 1.2e-05
## 4 1004 OKI 0.526625 0.000012 1.2e-05 0.473340 1.2e-05
## 5 1005 OKI 0.526835 0.000012 1.2e-05 0.473130 1.2e-05
## 6 1006 OKI 0.561466 0.000012 1.2e-05 0.438499 1.2e-05
```

Import sample locations

```
sampling_loc <- readRDS(here("output", "local_adaptation", "sampling_loc.rds"))
head(sampling_loc)
```

```
## # A tibble: 6 × 6
##   Pop_City   Country  Latitude Longitude Region Abbreviation
##   <chr>      <chr>       <dbl>     <dbl> <chr>  <chr>       
## 1 Gelephu    Bhutan       26.9      90.5 Asia   GEL         
## 2 Phnom Penh Cambodia     11.6     105.  Asia   CAM         
## 3 Hainan     China        19.2     110.  Asia   HAI         
## 4 Yunnan     China        24.5     101.  Asia   YUN         
## 5 Hunan      China        27.6     112.  Asia   HUN         
## 6 Bengaluru  India        13.0      77.6 Asia   BEN
```

```
source(
  here(
    "scripts", "analysis", "my_theme3.R"
  )
)

# Create a named vector to map countries to regions
country_to_region <- c(
  "Bhutan" = "South Asia",
  "Cambodia" = "Southeast Asia",
  "China" = "East Asia",
  "India" = "South Asia",
  "Indonesia" = "Southeast Asia",
  "Japan" = "East Asia",
  "Malaysia" = "Southeast Asia",
  "Maldives" = "South Asia",
  "Nepal" = "South Asia",
  "Sri Lanka" = "South Asia",
  "Taiwan" = "East Asia",
  "Thailand" = "Southeast Asia",
  "Vietnam" = "Southeast Asia"
)

# Add the region to the data frame
sampling_loc$Region2 <- country_to_region[sampling_loc$Country]

# Melt the data frame for plotting
Q_melted <- k5run1 |>
  pivot_longer(
    cols = -c(ind, pop),
    names_to = "variable",
    values_to = "value"
  )
# Join with sampling_loc to get sampling localities
Q_joined <- Q_melted |>
  left_join(sampling_loc, by = c("pop" = "Abbreviation"))

# Create a combined variable for Region and Country
Q_joined <- Q_joined |>
  mutate(Region_Country = interaction(Region, Country, sep = "_"))

# Order the combined variable by Region and Country, then by individual
Q_ordered <- Q_joined |>
  arrange(Region, Region2, Country, ind) |>
  mutate(ind = factor(ind, levels = unique(ind)))  # Convert ind to a factor with levels in the desired order

# Add labels: country names for the first individual in each country, NA for all other individuals
Q_ordered <- Q_ordered |>
  group_by(Region_Country) |>
  mutate(label = ifelse(row_number() == 1, as.character(Country), NA))

# Group by individual and variable, calculate mean ancestry proportions
Q_grouped <- Q_ordered |>
  group_by(ind, variable) |>
  summarise(value = mean(value), .groups = "drop")

# Create a data frame for borders
borders <-
  data.frame(Region_Country = unique(Q_ordered$Region_Country))

# Add the order of the last individual of each country to ensure correct placement of borders
borders$order <-
  sapply(borders$Region_Country, function(rc)
    max(which(Q_ordered$Region_Country == rc))) + 0.5  # Shift borders to the right edge of the bars

# Select only the first occurrence of each country in the ordered data
label_df <- Q_ordered |>
  filter(!is.na(label)) |>
  distinct(label, .keep_all = TRUE)

# Create a custom label function
label_func <- function(x) {
  labels <- rep("", length(x))
  labels[x %in% label_df$ind] <- label_df$label
  labels
}

# Calculate the position of lines
border_positions <- Q_ordered |>
  group_by(Country) |>
  summarise(pos = max(as.numeric(ind)) + 0)

# Calculate the position of population labels and bars
pop_labels <- Q_ordered |>
  mutate(Name = paste(pop, Pop_City, sep = " - ")) |>
  group_by(pop) |>
  slice_head(n = 1) |>
  ungroup() |>
  dplyr::select(ind, Pop_City, Country, Name) |>
  mutate(pos = as.numeric(ind))  # calculate position of population labels

pop_labels_bars <- pop_labels |>
  mutate(pos = as.numeric(ind)  - .5)


# Calculate the position of lines
border_positions <- Q_ordered |>
  group_by(Country) |>
  summarise(pos = max(as.numeric(ind)) - 1)


pop_labels_bars <- pop_labels |>
  mutate(pos = as.numeric(ind)  - .5)

# Function to filter and normalize data
normalize_data <- function(df, min_value) {
  df |>
    filter(value > min_value) |>
    group_by(ind) |>
    mutate(value = value / sum(value))
}

# Use the function
Q_grouped_filtered <- normalize_data(Q_grouped, 0.1)
# 

color_palette <-
  c(
    "v1" = "#FFB347",
    "v2" = "#AE9393",
    "v3" = "#FFFF99",
    "v4" = "#F49AC2",
    "v5" = "red"
    )

# Generate all potential variable names
all_variables <- paste0("v", 1:5)

# Map each variable to a name
color_mapping <- data.frame(variable = all_variables,
                            color = names(color_palette))

# Merge with Q_grouped_filtered
Q_grouped_filtered <- merge(Q_grouped_filtered, color_mapping, by = "variable")

# Create the plot
ggplot(Q_grouped_filtered, aes(x = as.factor(ind), y = value, fill = color)) +
  geom_bar(stat = 'identity', width = 1) +
  geom_vline(
    data = pop_labels_bars,
    aes(xintercept = pos),
    color = "#2C444A",
    linewidth = .2
  ) +
  geom_text(
    data = pop_labels,
    aes(x = as.numeric(ind), y = 1, label = Name),
    vjust = 1.5,
    hjust = 0,
    size = 2,
    angle = 90,
    inherit.aes = FALSE
  ) +
  my_theme() +
  theme(
    axis.text.x = element_text(
      angle = 90,
      hjust = 1,
      size = 12
    ),
    legend.position = "none",
    plot.margin = unit(c(3, 0.5, 0.5, 0.5), "cm")
  ) +
  xlab("Admixture matrix") +
  ylab("Ancestry proportions") +
  labs(caption = "Each bar represents the ancestry proportions for an individual for k=5.\n fastStructure simple prior for k1:30 with 9,047 SNPs.") +
  scale_x_discrete(labels = label_func) +
  scale_fill_manual(values = color_palette) +
  expand_limits(y = c(0, 1.5))
```

```
# # save it
ggsave(
  here("output", "populations", "figures", "faststructure_simple_neutral_k5.pdf"),
  width  = 12,
  height = 6,
  units  = "in",
  device = cairo_pdf
)
```

##### 2.1.8.2 logistic prior

Check the downloaded data

```
ls -1 output/populations/faststructure/neutral/run*/logistic* | tail
```

```
## output/populations/faststructure/neutral/run10/logistic.28.meanQ
## output/populations/faststructure/neutral/run10/logistic.29.meanQ
## output/populations/faststructure/neutral/run10/logistic.3.meanQ
## output/populations/faststructure/neutral/run10/logistic.30.meanQ
## output/populations/faststructure/neutral/run10/logistic.4.meanQ
## output/populations/faststructure/neutral/run10/logistic.5.meanQ
## output/populations/faststructure/neutral/run10/logistic.6.meanQ
## output/populations/faststructure/neutral/run10/logistic.7.meanQ
## output/populations/faststructure/neutral/run10/logistic.8.meanQ
## output/populations/faststructure/neutral/run10/logistic.9.meanQ
```

Create filemap2.txt

```
# we will use the 10 runs to make the plot. 
# loop to generate filemap2.txt with k values from 2 to 12. I use tab instead of space. We need that for pong.
for k in $(seq 2 12); do
    for run in $(seq -w 1 10); do
        echo "k$k""r$run $k faststructure/neutral/run$run/logistic.$k.meanQ" | tr ' ' '\t'
    done
done > output/populations/filemap2.txt;
head -n 5 output/populations/filemap2.txt
```

```
## k2r01    2   faststructure/neutral/run01/logistic.2.meanQ
## k2r02    2   faststructure/neutral/run02/logistic.2.meanQ
## k2r03    2   faststructure/neutral/run03/logistic.2.meanQ
## k2r04    2   faststructure/neutral/run04/logistic.2.meanQ
## k2r05    2   faststructure/neutral/run05/logistic.2.meanQ
```

```
wc -l output/populations/filemap2.txt
```

```
##      110 output/populations/filemap2.txt
```

Create ind2pop

```
rm output/populations/ind2pop.txt;
cat output/populations/snps_sets/neutral.fam | awk '{print $1}' > output/populations/ind2pop.txt;
head -n 5 output/populations/ind2pop.txt
```

```
## OKI
## OKI
## OKI
## OKI
## OKI
```

Run on the terminal and not on RStudio.

```
# on your computer to visualize the results
pong \
--filemap output/populations/filemap2.txt \
--ind2pop output/populations/ind2pop.txt  \
--dist_metric jaccard \
--sim_threshold 0.82 \
--greedy \
--force \
--output_dir output/populations/sim_threshold_0.82 \
--verbose
```

Check for the modes for k=5. Seven modes. The mode with the most runs
had 3 runs. We can plot run02

###### 2.1.8.2.1 Create plot simple prior with Q matrix identified with pong

Make plot

```
# Extract ancestry coefficients
k5run1 <- read_delim(
  here("output", "populations", "faststructure", "neutral", "run02", "logistic.5.meanQ"),
  delim = "  ", # Specify the delimiter if different from the default (comma)
  col_names = FALSE,
  show_col_types = FALSE
) 

head(k5run1)
```

```
## # A tibble: 6 × 5
##         X1       X2    X3       X4       X5
##      <dbl>    <dbl> <dbl>    <dbl>    <dbl>
## 1 0.000012 0.000012 1.00  0.000183 0.000035
## 2 0.872    0.000012 0.128 0.000012 0.000012
## 3 0.000012 0.000063 1.00  0.000014 0.000012
## 4 0.000012 0.000012 1.00  0.000011 0.000015
## 5 0.000012 0.000034 1.00  0.000029 0.000012
## 6 0.000012 0.000013 1.00  0.000046 0.000012
```

The fam file

```
fam_file <- here(
  "output", "populations", "snps_sets", "neutral.fam"
)

# Read the .fam file
fam_data <- read.table(fam_file, 
                       header = FALSE,
                       col.names = c("FamilyID", "IndividualID", "PaternalID", "MaternalID", "Sex", "Phenotype"))

# View the first few rows
head(fam_data)
```

```
##   FamilyID IndividualID PaternalID MaternalID Sex Phenotype
## 1      OKI         1001          0          0   2        -9
## 2      OKI         1002          0          0   2        -9
## 3      OKI         1003          0          0   2        -9
## 4      OKI         1004          0          0   2        -9
## 5      OKI         1005          0          0   2        -9
## 6      OKI         1006          0          0   1        -9
```

Create ID column

```
# Change column name
colnames(fam_data)[colnames(fam_data) == "IndividualID"] <- "ind"


# Merge columns "FamilyID" and "IndividualID" with an underscore
# fam_data$ind <- paste(fam_data$FamilyID, fam_data$IndividualID, sep = "_")


# Change column name
colnames(fam_data)[colnames(fam_data) == "FamilyID"] <- "pop"

# Select ID
fam_data <- fam_data |>
  dplyr::select("ind", "pop")

# View the first few rows
head(fam_data)
```

```
##    ind pop
## 1 1001 OKI
## 2 1002 OKI
## 3 1003 OKI
## 4 1004 OKI
## 5 1005 OKI
## 6 1006 OKI
```

Add it to matrix

```
k5run1 <- fam_data |>
  dplyr::select(ind, pop) |>
  bind_cols(k5run1)

head(k5run1)
```

```
##    ind pop       X1      X2       X3       X4      X5
## 1 1001 OKI 0.000012 1.2e-05 0.999760 0.000183 3.5e-05
## 2 1002 OKI 0.871549 1.2e-05 0.128416 0.000012 1.2e-05
## 3 1003 OKI 0.000012 6.3e-05 0.999899 0.000014 1.2e-05
## 4 1004 OKI 0.000012 1.2e-05 0.999950 0.000011 1.5e-05
## 5 1005 OKI 0.000012 3.4e-05 0.999913 0.000029 1.2e-05
## 6 1006 OKI 0.000012 1.3e-05 0.999918 0.000046 1.2e-05
```

Rename the columns

```
# Rename the columns starting from the third one
k5run1 <- k5run1 |>
  rename_with(~paste0("v", seq_along(.x)), .cols = -c(ind, pop))

# View the first few rows
head(k5run1)
```

```
##    ind pop       v1      v2       v3       v4      v5
## 1 1001 OKI 0.000012 1.2e-05 0.999760 0.000183 3.5e-05
## 2 1002 OKI 0.871549 1.2e-05 0.128416 0.000012 1.2e-05
## 3 1003 OKI 0.000012 6.3e-05 0.999899 0.000014 1.2e-05
## 4 1004 OKI 0.000012 1.2e-05 0.999950 0.000011 1.5e-05
## 5 1005 OKI 0.000012 3.4e-05 0.999913 0.000029 1.2e-05
## 6 1006 OKI 0.000012 1.3e-05 0.999918 0.000046 1.2e-05
```

Import sample locations

```
sampling_loc <- readRDS(here("output", "local_adaptation", "sampling_loc.rds"))
head(sampling_loc)
```

```
## # A tibble: 6 × 6
##   Pop_City   Country  Latitude Longitude Region Abbreviation
##   <chr>      <chr>       <dbl>     <dbl> <chr>  <chr>       
## 1 Gelephu    Bhutan       26.9      90.5 Asia   GEL         
## 2 Phnom Penh Cambodia     11.6     105.  Asia   CAM         
## 3 Hainan     China        19.2     110.  Asia   HAI         
## 4 Yunnan     China        24.5     101.  Asia   YUN         
## 5 Hunan      China        27.6     112.  Asia   HUN         
## 6 Bengaluru  India        13.0      77.6 Asia   BEN
```

```
source(
  here(
    "scripts", "analysis", "my_theme3.R"
  )
)

# Create a named vector to map countries to regions
country_to_region <- c(
  "Bhutan" = "South Asia",
  "Cambodia" = "Southeast Asia",
  "China" = "East Asia",
  "India" = "South Asia",
  "Indonesia" = "Southeast Asia",
  "Japan" = "East Asia",
  "Malaysia" = "Southeast Asia",
  "Maldives" = "South Asia",
  "Nepal" = "South Asia",
  "Sri Lanka" = "South Asia",
  "Taiwan" = "East Asia",
  "Thailand" = "Southeast Asia",
  "Vietnam" = "Southeast Asia"
)

# Add the region to the data frame
sampling_loc$Region2 <- country_to_region[sampling_loc$Country]

# Melt the data frame for plotting
Q_melted <- k5run1 |>
  pivot_longer(
    cols = -c(ind, pop),
    names_to = "variable",
    values_to = "value"
  )
# Join with sampling_loc to get sampling localities
Q_joined <- Q_melted |>
  left_join(sampling_loc, by = c("pop" = "Abbreviation"))

# Create a combined variable for Region and Country
Q_joined <- Q_joined |>
  mutate(Region_Country = interaction(Region, Country, sep = "_"))

# Order the combined variable by Region and Country, then by individual
Q_ordered <- Q_joined |>
  arrange(Region, Region2, Country, ind) |>
  mutate(ind = factor(ind, levels = unique(ind)))  # Convert ind to a factor with levels in the desired order

# Add labels: country names for the first individual in each country, NA for all other individuals
Q_ordered <- Q_ordered |>
  group_by(Region_Country) |>
  mutate(label = ifelse(row_number() == 1, as.character(Country), NA))

# Group by individual and variable, calculate mean ancestry proportions
Q_grouped <- Q_ordered |>
  group_by(ind, variable) |>
  summarise(value = mean(value), .groups = "drop")

# Create a data frame for borders
borders <-
  data.frame(Region_Country = unique(Q_ordered$Region_Country))

# Add the order of the last individual of each country to ensure correct placement of borders
borders$order <-
  sapply(borders$Region_Country, function(rc)
    max(which(Q_ordered$Region_Country == rc))) + 0.5  # Shift borders to the right edge of the bars

# Select only the first occurrence of each country in the ordered data
label_df <- Q_ordered |>
  filter(!is.na(label)) |>
  distinct(label, .keep_all = TRUE)

# Create a custom label function
label_func <- function(x) {
  labels <- rep("", length(x))
  labels[x %in% label_df$ind] <- label_df$label
  labels
}

# Calculate the position of lines
border_positions <- Q_ordered |>
  group_by(Country) |>
  summarise(pos = max(as.numeric(ind)) + 0)

# Calculate the position of population labels and bars
pop_labels <- Q_ordered |>
  mutate(Name = paste(pop, Pop_City, sep = " - ")) |>
  group_by(pop) |>
  slice_head(n = 1) |>
  ungroup() |>
  dplyr::select(ind, Pop_City, Country, Name) |>
  mutate(pos = as.numeric(ind))  # calculate position of population labels

pop_labels_bars <- pop_labels |>
  mutate(pos = as.numeric(ind)  - .5)


# Calculate the position of lines
border_positions <- Q_ordered |>
  group_by(Country) |>
  summarise(pos = max(as.numeric(ind)) - 1)


pop_labels_bars <- pop_labels |>
  mutate(pos = as.numeric(ind)  - .5)

# Function to filter and normalize data
normalize_data <- function(df, min_value) {
  df |>
    filter(value > min_value) |>
    group_by(ind) |>
    mutate(value = value / sum(value))
}

# Use the function
Q_grouped_filtered <- normalize_data(Q_grouped, 0.1)
# 

color_palette <-
  c(
    "v1" = "#AE9393",
    "v2" = "red",
    "v3" = "#FFFF99",
    "v4" = "#F49AC2",
    "v5" = "#FFB347"
  )
# Generate all potential variable names
all_variables <- paste0("v", 1:5)

# Map each variable to a name
color_mapping <- data.frame(variable = all_variables,
                            color = names(color_palette))

# Merge with Q_grouped_filtered
Q_grouped_filtered <- merge(Q_grouped_filtered, color_mapping, by = "variable")

# Create the plot
ggplot(Q_grouped_filtered, aes(x = as.factor(ind), y = value, fill = color)) +
  geom_bar(stat = 'identity', width = 1) +
  geom_vline(
    data = pop_labels_bars,
    aes(xintercept = pos),
    color = "#2C444A",
    linewidth = .2
  ) +
  geom_text(
    data = pop_labels,
    aes(x = as.numeric(ind), y = 1, label = Name),
    vjust = 1.5,
    hjust = 0,
    size = 2,
    angle = 90,
    inherit.aes = FALSE
  ) +
  my_theme() +
  theme(
    axis.text.x = element_text(
      angle = 90,
      hjust = 1,
      size = 12
    ),
    legend.position = "none",
    plot.margin = unit(c(3, 0.5, 0.5, 0.5), "cm")
  ) +
  xlab("Admixture matrix") +
  ylab("Ancestry proportions") +
  labs(caption = "Each bar represents the ancestry proportions for an individual for k=5.\n fastStructure logistic prior for k1:30 with 9,047 SNPs.") +
  scale_x_discrete(labels = label_func) +
  scale_fill_manual(values = color_palette) +
  expand_limits(y = c(0, 1.5))
```

```
# # save it
ggsave(
  here("output", "populations", "figures", "faststructure_logistic_neutral_k5.pdf"),
  width  = 12,
  height = 6,
  units  = "in",
  device = cairo_pdf
)
```

### 2.2 r2 0.01 set

Create a directory for each run

```
cd /gpfs/ycga/project/caccone/lvc26/faststructure/r2_0.01
for i in $(seq 1 10) # we will not use -w because we will run 1 run with the logistic prior
do
  mkdir run$i
done
```

Now we can generate the full script for dsq (not showing here,
because it has 2,000 lines).

#### 2.2.1 Simple prior

```
for k in $(seq 1 30); do
    for run in $(seq 1 10); do
        echo "cd /gpfs/ycga/project/caccone/lvc26/faststructure; source /vast/palmer/apps/avx2/software/miniconda/23.3.1/etc/profile.d/conda.sh;module load miniconda/23.3.1; conda activate fastStructure; export PYTHONPATH=/home/lvc26/project/fastStructure; python -m structure -K $k --input=/gpfs/ycga/project/caccone/lvc26/neuroadmix/snps_sets/r2_0.01 --output=/gpfs/ycga/project/caccone/lvc26/faststructure/r2_0.01/run$run/simple --prior=simple --full --cv=10 --tol=10e-6"
    done
done > dsq1.txt
```

#### 2.2.2 Logistic prior

Only 1 run I used 10 runs for the neutral set because it is around 9k
SNPs. For other data sets we can do less runs because it takes too
long.

```
for k in $(seq 1 30); do
    for run in $(seq 1); do
        echo "cd /gpfs/ycga/project/caccone/lvc26/faststructure; source /vast/palmer/apps/avx2/software/miniconda/23.3.1/etc/profile.d/conda.sh;module load miniconda/23.3.1; conda activate fastStructure; export PYTHONPATH=/home/lvc26/project/fastStructure; python -m structure -K $k --input=/gpfs/ycga/project/caccone/lvc26/neuroadmix/snps_sets/r2_0.01 --output=/gpfs/ycga/project/caccone/lvc26/faststructure/r2_0.01/run$run/logistic --prior=logistic --full --cv=10 --tol=10e-6"
    done
done > dsq2.txt
```

Load dsq, and then create the batch file. Check the dsq –help

#### 2.2.3 Generate the dsq batch files

```
module load dSQ/1.05
##############################################
#                 Simple
##############################################
dsq \
--job-file dsq1.txt \
--mem-per-cpu 1g \
--cpus-per-task=1 \
-t 24:00:00 \
--mail-type END \
--partition=scavenge \
--job-name fastStr_s01 \
--output /dev/null \
--batch-file 1.simple.sh


##############################################
#                 Logistic
##############################################
dsq \
--job-file dsq2.txt \
--mem-per-cpu 1g \
--cpus-per-task=1 \
--time 7-00:00:00 \
--mail-type END \
--partition=week \
--job-name fastStr_L01 \
--output /dev/null \
--batch-file 2.logistic.sh
```

Submit

```
sbatch 1.simple.sh
dsqa -j 6274417 

sbatch 2.logistic.sh
dsqa -j 6274725
# completed
```

#### 2.2.4 Parse files from runs

Next, we will need to parse the results and make a plot. Our fist
step is done on the cluster. We need to grab the Q matrices and the log
files from our runs. We start wit the simple priors. We first find the
optimal number of Ks. Then we use pong to parse all runs and generate
one structure like plot. You can get pong here https://github.com/ramachandran-lab/pong. I installed
pong on my base environment in my laptop. You can create a environment
if you want, check their manual to follow instructions.

Data location

```
cd /gpfs/ycga/project/caccone/lvc26/faststructure/r2_0.01
```

#### 2.2.5 Choose k simple plot

Our first step is to use fastStructure script chooseK.py to find the
number of ancestral populations. The main reason we did 10 runs is
because we don’t find the same optimal k for every run. Since we already
created a conda environment to run fastStructure, we can activate it,
and run chooseK.

First for our runs with simple prior.

```
salloc --time=06:00:00 --nodes=1 --ntasks=1 --cpus-per-task=1 --mem-per-cpu=5120
module load miniconda/23.3.1
conda activate fastStructure
export PYTHONPATH=/home/lvc26/project/fastStructure


# simple 10 runs
cat <(echo 'run likelihood structure') \
<(for i in /gpfs/ycga/project/caccone/lvc26/faststructure/r2_0.01/run*
do
    echo $i | sed 's@.*/@@' | sed 's/run/run /' ; python -m chooseK --input=$i/simple | grep 'likelihood' | awk '{print $6, $8}'; python -m chooseK --input=$i/simple | grep 'structure' |  awk '{print $6, $10}'
done | xargs -n6 | awk '{print $2, $4, $6}') > simple_prior_models.txt

# logistic 1 run
cat <(echo 'run likelihood structure') \
<(for i in /gpfs/ycga/project/caccone/lvc26/faststructure/r2_0.01/run1
do
    echo $i | sed 's@.*/@@' | sed 's/run/run /' ; python -m chooseK --input=$i/logistic | grep 'likelihood' | awk '{print $6, $8}'; python -m chooseK --input=$i/logistic | grep 'structure' |  awk '{print $6, $10}'
done | xargs -n6 | awk '{print $2, $4, $6}') > logistic_prior_models.txt
```

Download the data

```
rsync -chavzP --stats lvc26@mccleary.ycrc.yale.edu:/gpfs/ycga/project/caccone/lvc26/faststructure/r2_0.01/*_prior_models.txt /Users/lucianocosme/Library/CloudStorage/Dropbox/Albopictus/manuscript_chip/data/no_autogenous/albo_chip/output/populations/faststructure/r2_0.01
```

We can make a plot with the Ks. Let’s import the data in R.

```
# function to import our choosek.py data
import_fastStructure <- function(file) {
  # import as a tibble and set columns as integers
  dat <- read_delim(
    file,
    col_names      = TRUE,
    show_col_types = FALSE,
    col_types      = "iii"
  )

  # get columns we need and make it long for plotting
  dat <- dat |>
    gather(
      structure, likelihood, -run
    )

  # rename the columns by index
  dat <- dat |>
    rename(
      run   = 1,
      model = 2,
      k     = 3
    )
  return(dat)
}
# we can save the function to source it later
dump(
  "import_fastStructure",
  here(
    "scripts", "analysis", "import_fastStructure.R"
  ),
  append = TRUE
)
```

Now we can use the function to import the data

```
# we can use our function with here to import the data
choose_k_simple <- 
  import_fastStructure(
  here(
    "output", "populations","fastStructure", "r2_0.01","simple_prior_models.txt"
  )
)
```

Function to plot fastStructure choosek.py results

```
# function to plot our choosek.py data
plot_fastStructure <- function(df) {
  df |>
    ggplot() +
    geom_line(
      aes(
        x              = run,
        y              = k,
        color          = model
      ),
      linewidth = 1
    ) +
    scale_colour_manual(
      "model",
      values = c(
        structure      = "#9d60ff",
        likelihood     = "#ffc08c"
      ),
      labels = c(
        "Maximizes \n Likelihood \n", "Explain \n Structure"
      )
    ) +
    labs(
      x                = "Run",
      y                = "K",
      title            = "fastStructure",
      caption          = "algorithm runs for choices of K ranging from 1 to 30"
    ) +
    hrbrthemes::theme_ipsum(
      base_family = "",
      axis_text_size = 12,
      axis_title_size = 14,
      plot_margin = margin(
        10, 10, 10, 10
      ),
      grid = TRUE,
      grid_col = "#fabbe2"
    ) +
    theme(
      panel.grid.major = element_line(
        linetype       = "dashed",
        linewidth      = 0.2
      ),
      panel.grid.minor = element_line(
        linetype       = "dashed",
        linewidth      = 0.2
      ),
      legend.text = element_text(
        size = 12
      ),
      legend.title = element_text(
        size           = 14,
        face           = "bold"
      ),
      legend.position = "right"
    )
}
# we can save the function to source it later
dump(
  "plot_fastStructure",
  here(
    "scripts", "analysis", "plot_fastStructure.R"
  ),
  append = TRUE
)
```

Use our function to plot k values

```
plot_fastStructure(
  choose_k_simple
) +
  labs(
    subtitle = "simple prior"
  )
```

```
# save plot
ggsave(
  here(
    "output", "figures", "fastStructure_r2_0.01_simple_prior_k1_k30.pdf"
  ),
  width  = 8,
  height = 6,
  units  = "in"
)
```

#### 2.2.6 Choose k logistic

Import logistic data

```
# import the data
choose_k_logistic <- import_fastStructure(
  here(
    "output", "populations","fastStructure", "r2_0.01", "logistic_prior_models.txt"
  )
)
# only 1 run
choose_k_logistic
```

```
## # A tibble: 2 × 3
##     run model          k
##   <int> <chr>      <int>
## 1     1 likelihood    30
## 2     1 structure     13
```

#### 2.2.7 Statistical tests - comparing simple and logistic prior runs

```
set.seed(123)  # for reproducibility
# import data
# simple
simple1 <- import_fastStructure(
  here(
    "output", "populations","fastStructure", "r2_0.01", "simple_prior_models.txt"
  )
) |>
  mutate(
    prior = "simple"
  )
#
# logistic
logistic1 <- import_fastStructure(
  here(
    "output", "populations","fastStructure", "r2_0.01", "logistic_prior_models.txt"
  )
) |>
  mutate(
    prior = "logistic"
  )
#
# rbind data set
fastStruc_prior <- rbind(
  simple1, logistic1
) |>
  mutate(
    prior = factor(
      prior
    )
  )
#
# run stats
grouped_ggbetweenstats(
  data = fastStruc_prior,
  x = prior,
  y = k,
  grouping.var = model,
  p.adjust.method = "bonferroni",
  palette = "default_jama",
  package = "ggsci",
  plotgrid.args = list(
    nrow = 1
  ),
  annotation.args = list(
    title = "Comparison of fastStructure prior"
  )
)
```

```
# # save plot
ggsave(
  here(
    "output", "populations", "figures", "welch_faststructure_r2_0.01_prior_comparisons.pdf"
  ),
  width = 11,
  height = 8,
  units = "in"
)
```

The number of model components that explain the structure in the data
is different from Admixture. For our simple model explaining the
structure in the data we have k=13, and for logistic k=20, whereas
Admixture indicated k=5. We can plot k=5 to see how it compares to the
Admixture, LEA, and neuro-admixture.

#### 2.2.8 pong

We first need to find the most common mode among runs. We do not need
to plot all matrices, we can plot the first 12 using pong.

Download the data

```
# next we can download the files to our computer, run the command below in your computer, not in the cluster
rsync -chavzP --stats --include='*/' --include='*.meanQ' --exclude='*' lvc26@mccleary.ycrc.yale.edu:/gpfs/ycga/project/caccone/lvc26/faststructure/r2_0.01* /Users/lucianocosme/Library/CloudStorage/Dropbox/Albopictus/manuscript_chip/data/no_autogenous/albo_chip/output/populations/faststructure/
```

##### 2.2.8.1 simple prior

Check the downloaded data

```
ls -1 output/populations/faststructure/r2_0.01/run*/simple* | tail
```

Create filemap2.txt

```
# we will use the 10 runs to make the plot. 
# loop to generate filemap2.txt with k values from 2 to 12. I use tab instead of space. We need that for pong.
for k in $(seq 2 12); do
    for run in $(seq 1 10); do
        echo "k$k""r$run $k faststructure/r2_0.01/run$run/simple.$k.meanQ" | tr ' ' '\t'
    done
done > output/populations/filemap2.txt;
head -n 5 output/populations/filemap2.txt
```

```
wc -l output/populations/filemap2.txt
```

Create ind2pop

```
rm output/populations/ind2pop.txt;
cat output/populations/snps_sets/r2_0.01.fam | awk '{print $1}' > output/populations/ind2pop.txt;
head -n 5 output/populations/ind2pop.txt
```

Run on the terminal and not on RStudio.

```
# on your computer to visualize the results
pong \
--filemap output/populations/filemap2.txt \
--ind2pop output/populations/ind2pop.txt  \
--dist_metric jaccard \
--sim_threshold 0.82 \
--greedy \
--force \
--output_dir output/populations/sim_threshold_0.82 \
--verbose
```

1 mode for k=5, so any run works.

###### 2.2.8.1.1 Create plot of the simple prior with Q matrix identified with pong

Make plot

```
# Extract ancestry coefficients
k5run1 <- read_delim(
  here("output", "populations", "faststructure", "r2_0.01", "run1", "simple.5.meanQ"),
  delim = "  ", # Specify the delimiter if different from the default (comma)
  col_names = FALSE,
  show_col_types = FALSE
) 

head(k5run1)
```

```
## # A tibble: 6 × 5
##         X1     X2       X3    X4       X5
##      <dbl>  <dbl>    <dbl> <dbl>    <dbl>
## 1 0.0926   0.274  0.000005 0.633 0.000005
## 2 0.126    0.245  0.000005 0.629 0.000005
## 3 0.0385   0.172  0.000005 0.789 0.000005
## 4 0.000005 0.123  0.000005 0.877 0.000005
## 5 0.000005 0.0852 0.000005 0.915 0.000005
## 6 0.000005 0.188  0.000005 0.800 0.0128
```

The fam file

```
fam_file <- here(
  "output", "populations", "snps_sets", "r2_0.01.fam"
)

# Read the .fam file
fam_data <- read.table(fam_file, 
                       header = FALSE,
                       col.names = c("FamilyID", "IndividualID", "PaternalID", "MaternalID", "Sex", "Phenotype"))

# View the first few rows
head(fam_data)
```

```
##   FamilyID IndividualID PaternalID MaternalID Sex Phenotype
## 1      OKI         1001          0          0   2        -9
## 2      OKI         1002          0          0   2        -9
## 3      OKI         1003          0          0   2        -9
## 4      OKI         1004          0          0   2        -9
## 5      OKI         1005          0          0   2        -9
## 6      OKI         1006          0          0   1        -9
```

Create ID column

```
# Change column name
colnames(fam_data)[colnames(fam_data) == "IndividualID"] <- "ind"


# Merge columns "FamilyID" and "IndividualID" with an underscore
# fam_data$ind <- paste(fam_data$FamilyID, fam_data$IndividualID, sep = "_")


# Change column name
colnames(fam_data)[colnames(fam_data) == "FamilyID"] <- "pop"

# Select ID
fam_data <- fam_data |>
  dplyr::select("ind", "pop")

# View the first few rows
head(fam_data)
```

```
##    ind pop
## 1 1001 OKI
## 2 1002 OKI
## 3 1003 OKI
## 4 1004 OKI
## 5 1005 OKI
## 6 1006 OKI
```

Add it to matrix

```
k5run1 <- fam_data |>
  dplyr::select(ind, pop) |>
  bind_cols(k5run1)

head(k5run1)
```

```
##    ind pop       X1       X2    X3       X4       X5
## 1 1001 OKI 0.092564 0.274411 5e-06 0.633015 0.000005
## 2 1002 OKI 0.125992 0.244922 5e-06 0.629076 0.000005
## 3 1003 OKI 0.038454 0.172095 5e-06 0.789440 0.000005
## 4 1004 OKI 0.000005 0.123287 5e-06 0.876698 0.000005
## 5 1005 OKI 0.000005 0.085162 5e-06 0.914823 0.000005
## 6 1006 OKI 0.000005 0.187602 5e-06 0.799554 0.012834
```

Rename the columns

```
# Rename the columns starting from the third one
k5run1 <- k5run1 |>
  rename_with(~paste0("v", seq_along(.x)), .cols = -c(ind, pop))

# View the first few rows
head(k5run1)
```

```
##    ind pop       v1       v2    v3       v4       v5
## 1 1001 OKI 0.092564 0.274411 5e-06 0.633015 0.000005
## 2 1002 OKI 0.125992 0.244922 5e-06 0.629076 0.000005
## 3 1003 OKI 0.038454 0.172095 5e-06 0.789440 0.000005
## 4 1004 OKI 0.000005 0.123287 5e-06 0.876698 0.000005
## 5 1005 OKI 0.000005 0.085162 5e-06 0.914823 0.000005
## 6 1006 OKI 0.000005 0.187602 5e-06 0.799554 0.012834
```

Import sample locations

```
sampling_loc <- readRDS(here("output", "local_adaptation", "sampling_loc.rds"))
head(sampling_loc)
```

```
## # A tibble: 6 × 6
##   Pop_City   Country  Latitude Longitude Region Abbreviation
##   <chr>      <chr>       <dbl>     <dbl> <chr>  <chr>       
## 1 Gelephu    Bhutan       26.9      90.5 Asia   GEL         
## 2 Phnom Penh Cambodia     11.6     105.  Asia   CAM         
## 3 Hainan     China        19.2     110.  Asia   HAI         
## 4 Yunnan     China        24.5     101.  Asia   YUN         
## 5 Hunan      China        27.6     112.  Asia   HUN         
## 6 Bengaluru  India        13.0      77.6 Asia   BEN
```

```
source(
  here(
    "scripts", "analysis", "my_theme3.R"
  )
)

# Create a named vector to map countries to regions
country_to_region <- c(
  "Bhutan" = "South Asia",
  "Cambodia" = "Southeast Asia",
  "China" = "East Asia",
  "India" = "South Asia",
  "Indonesia" = "Southeast Asia",
  "Japan" = "East Asia",
  "Malaysia" = "Southeast Asia",
  "Maldives" = "South Asia",
  "Nepal" = "South Asia",
  "Sri Lanka" = "South Asia",
  "Taiwan" = "East Asia",
  "Thailand" = "Southeast Asia",
  "Vietnam" = "Southeast Asia"
)

# Add the region to the data frame
sampling_loc$Region2 <- country_to_region[sampling_loc$Country]

# Melt the data frame for plotting
Q_melted <- k5run1 |>
  pivot_longer(
    cols = -c(ind, pop),
    names_to = "variable",
    values_to = "value"
  )
# Join with sampling_loc to get sampling localities
Q_joined <- Q_melted |>
  left_join(sampling_loc, by = c("pop" = "Abbreviation"))

# Create a combined variable for Region and Country
Q_joined <- Q_joined |>
  mutate(Region_Country = interaction(Region, Country, sep = "_"))

# Order the combined variable by Region and Country, then by individual
Q_ordered <- Q_joined |>
  arrange(Region, Region2, Country, ind) |>
  mutate(ind = factor(ind, levels = unique(ind)))  # Convert ind to a factor with levels in the desired order

# Add labels: country names for the first individual in each country, NA for all other individuals
Q_ordered <- Q_ordered |>
  group_by(Region_Country) |>
  mutate(label = ifelse(row_number() == 1, as.character(Country), NA))

# Group by individual and variable, calculate mean ancestry proportions
Q_grouped <- Q_ordered |>
  group_by(ind, variable) |>
  summarise(value = mean(value), .groups = "drop")

# Create a data frame for borders
borders <-
  data.frame(Region_Country = unique(Q_ordered$Region_Country))

# Add the order of the last individual of each country to ensure correct placement of borders
borders$order <-
  sapply(borders$Region_Country, function(rc)
    max(which(Q_ordered$Region_Country == rc))) + 0.5  # Shift borders to the right edge of the bars

# Select only the first occurrence of each country in the ordered data
label_df <- Q_ordered |>
  filter(!is.na(label)) |>
  distinct(label, .keep_all = TRUE)

# Create a custom label function
label_func <- function(x) {
  labels <- rep("", length(x))
  labels[x %in% label_df$ind] <- label_df$label
  labels
}

# Calculate the position of lines
border_positions <- Q_ordered |>
  group_by(Country) |>
  summarise(pos = max(as.numeric(ind)) + 0)

# Calculate the position of population labels and bars
pop_labels <- Q_ordered |>
  mutate(Name = paste(pop, Pop_City, sep = " - ")) |>
  group_by(pop) |>
  slice_head(n = 1) |>
  ungroup() |>
  dplyr::select(ind, Pop_City, Country, Name) |>
  mutate(pos = as.numeric(ind))  # calculate position of population labels

pop_labels_bars <- pop_labels |>
  mutate(pos = as.numeric(ind)  - .5)


# Calculate the position of lines
border_positions <- Q_ordered |>
  group_by(Country) |>
  summarise(pos = max(as.numeric(ind)) - 1)


pop_labels_bars <- pop_labels |>
  mutate(pos = as.numeric(ind)  - .5)

# Function to filter and normalize data
normalize_data <- function(df, min_value) {
  df |>
    filter(value > min_value) |>
    group_by(ind) |>
    mutate(value = value / sum(value))
}

# Use the function
Q_grouped_filtered <- normalize_data(Q_grouped, 0.1)
# 

color_palette <-
  c(
    "v1" = "#F49AC2",
    "v2" = "#FFB347",
    "v3" = "#FFFF99",
    "v4" = "#AE9393",
    "v5" = "red"
  )
# Generate all potential variable names
all_variables <- paste0("v", 1:5)

# Map each variable to a name
color_mapping <- data.frame(variable = all_variables,
                            color = names(color_palette))

# Merge with Q_grouped_filtered
Q_grouped_filtered <- merge(Q_grouped_filtered, color_mapping, by = "variable")

# Create the plot
ggplot(Q_grouped_filtered, aes(x = as.factor(ind), y = value, fill = color)) +
  geom_bar(stat = 'identity', width = 1) +
  geom_vline(
    data = pop_labels_bars,
    aes(xintercept = pos),
    color = "#2C444A",
    linewidth = .2
  ) +
  geom_text(
    data = pop_labels,
    aes(x = as.numeric(ind), y = 1, label = Name),
    vjust = 1.5,
    hjust = 0,
    size = 2,
    angle = 90,
    inherit.aes = FALSE
  ) +
  my_theme() +
  theme(
    axis.text.x = element_text(
      angle = 90,
      hjust = 1,
      size = 12
    ),
    legend.position = "none",
    plot.margin = unit(c(3, 0.5, 0.5, 0.5), "cm")
  ) +
  xlab("Admixture matrix") +
  ylab("Ancestry proportions") +
  labs(caption = "Each bar represents the ancestry proportions for an individual for k=5.\n fastStructure simple prior for k1:30 with 20,931 SNPs.") +
  scale_x_discrete(labels = label_func) +
  scale_fill_manual(values = color_palette) +
  expand_limits(y = c(0, 1.5))
```

```
# # save it
ggsave(
  here("output", "populations", "figures", "faststructure_simple_r2_0.01_k5.pdf"),
  width  = 12,
  height = 6,
  units  = "in",
  device = cairo_pdf
)
```

##### 2.2.8.1 logistic prior

Check the downloaded data

```
ls -1 output/populations/faststructure/r2_0.01/run*/logistic* | tail
```

We do not need to use pong because we have only 1 run for the
logistic.

###### 2.2.8.1.1 Create plot simple prior with Q matrix identified with pong

Make plot

```
# Extract ancestry coefficients
k5run1 <- read_delim(
  here("output", "populations", "faststructure", "r2_0.01", "run1", "logistic.5.meanQ"),
  delim = "  ", # Specify the delimiter if different from the default (comma)
  col_names = FALSE,
  show_col_types = FALSE
) 

head(k5run1)
```

```
## # A tibble: 6 × 5
##         X1       X2       X3    X4       X5
##      <dbl>    <dbl>    <dbl> <dbl>    <dbl>
## 1 0.000005 0.000005 0.000005  1.00 0.000005
## 2 0.000005 0.000005 0.000005  1.00 0.000005
## 3 0.000005 0.000005 0.000005  1.00 0.000005
## 4 0.000005 0.000005 0.000005  1.00 0.000005
## 5 0.000005 0.000005 0.000005  1.00 0.000005
## 6 0.000005 0.000005 0.000005  1.00 0.000005
```

The fam file

```
fam_file <- here(
  "output", "populations", "snps_sets", "r2_0.01.fam"
)

# Read the .fam file
fam_data <- read.table(fam_file, 
                       header = FALSE,
                       col.names = c("FamilyID", "IndividualID", "PaternalID", "MaternalID", "Sex", "Phenotype"))

# View the first few rows
head(fam_data)
```

```
##   FamilyID IndividualID PaternalID MaternalID Sex Phenotype
## 1      OKI         1001          0          0   2        -9
## 2      OKI         1002          0          0   2        -9
## 3      OKI         1003          0          0   2        -9
## 4      OKI         1004          0          0   2        -9
## 5      OKI         1005          0          0   2        -9
## 6      OKI         1006          0          0   1        -9
```

Create ID column

```
# Change column name
colnames(fam_data)[colnames(fam_data) == "IndividualID"] <- "ind"


# Merge columns "FamilyID" and "IndividualID" with an underscore
# fam_data$ind <- paste(fam_data$FamilyID, fam_data$IndividualID, sep = "_")


# Change column name
colnames(fam_data)[colnames(fam_data) == "FamilyID"] <- "pop"

# Select ID
fam_data <- fam_data |>
  dplyr::select("ind", "pop")

# View the first few rows
head(fam_data)
```

```
##    ind pop
## 1 1001 OKI
## 2 1002 OKI
## 3 1003 OKI
## 4 1004 OKI
## 5 1005 OKI
## 6 1006 OKI
```

Add it to matrix

```
k5run1 <- fam_data |>
  dplyr::select(ind, pop) |>
  bind_cols(k5run1)

head(k5run1)
```

```
##    ind pop    X1    X2    X3      X4    X5
## 1 1001 OKI 5e-06 5e-06 5e-06 0.99998 5e-06
## 2 1002 OKI 5e-06 5e-06 5e-06 0.99998 5e-06
## 3 1003 OKI 5e-06 5e-06 5e-06 0.99998 5e-06
## 4 1004 OKI 5e-06 5e-06 5e-06 0.99998 5e-06
## 5 1005 OKI 5e-06 5e-06 5e-06 0.99998 5e-06
## 6 1006 OKI 5e-06 5e-06 5e-06 0.99998 5e-06
```

Rename the columns

```
# Rename the columns starting from the third one
k5run1 <- k5run1 |>
  rename_with(~paste0("v", seq_along(.x)), .cols = -c(ind, pop))

# View the first few rows
head(k5run1)
```

```
##    ind pop    v1    v2    v3      v4    v5
## 1 1001 OKI 5e-06 5e-06 5e-06 0.99998 5e-06
## 2 1002 OKI 5e-06 5e-06 5e-06 0.99998 5e-06
## 3 1003 OKI 5e-06 5e-06 5e-06 0.99998 5e-06
## 4 1004 OKI 5e-06 5e-06 5e-06 0.99998 5e-06
## 5 1005 OKI 5e-06 5e-06 5e-06 0.99998 5e-06
## 6 1006 OKI 5e-06 5e-06 5e-06 0.99998 5e-06
```

Import sample locations

```
sampling_loc <- readRDS(here("output", "local_adaptation", "sampling_loc.rds"))
head(sampling_loc)
```

```
## # A tibble: 6 × 6
##   Pop_City   Country  Latitude Longitude Region Abbreviation
##   <chr>      <chr>       <dbl>     <dbl> <chr>  <chr>       
## 1 Gelephu    Bhutan       26.9      90.5 Asia   GEL         
## 2 Phnom Penh Cambodia     11.6     105.  Asia   CAM         
## 3 Hainan     China        19.2     110.  Asia   HAI         
## 4 Yunnan     China        24.5     101.  Asia   YUN         
## 5 Hunan      China        27.6     112.  Asia   HUN         
## 6 Bengaluru  India        13.0      77.6 Asia   BEN
```

```
source(
  here(
    "scripts", "analysis", "my_theme3.R"
  )
)

# Create a named vector to map countries to regions
country_to_region <- c(
  "Bhutan" = "South Asia",
  "Cambodia" = "Southeast Asia",
  "China" = "East Asia",
  "India" = "South Asia",
  "Indonesia" = "Southeast Asia",
  "Japan" = "East Asia",
  "Malaysia" = "Southeast Asia",
  "Maldives" = "South Asia",
  "Nepal" = "South Asia",
  "Sri Lanka" = "South Asia",
  "Taiwan" = "East Asia",
  "Thailand" = "Southeast Asia",
  "Vietnam" = "Southeast Asia"
)

# Add the region to the data frame
sampling_loc$Region2 <- country_to_region[sampling_loc$Country]

# Melt the data frame for plotting
Q_melted <- k5run1 |>
  pivot_longer(
    cols = -c(ind, pop),
    names_to = "variable",
    values_to = "value"
  )
# Join with sampling_loc to get sampling localities
Q_joined <- Q_melted |>
  left_join(sampling_loc, by = c("pop" = "Abbreviation"))

# Create a combined variable for Region and Country
Q_joined <- Q_joined |>
  mutate(Region_Country = interaction(Region, Country, sep = "_"))

# Order the combined variable by Region and Country, then by individual
Q_ordered <- Q_joined |>
  arrange(Region, Region2, Country, ind) |>
  mutate(ind = factor(ind, levels = unique(ind)))  # Convert ind to a factor with levels in the desired order

# Add labels: country names for the first individual in each country, NA for all other individuals
Q_ordered <- Q_ordered |>
  group_by(Region_Country) |>
  mutate(label = ifelse(row_number() == 1, as.character(Country), NA))

# Group by individual and variable, calculate mean ancestry proportions
Q_grouped <- Q_ordered |>
  group_by(ind, variable) |>
  summarise(value = mean(value), .groups = "drop")

# Create a data frame for borders
borders <-
  data.frame(Region_Country = unique(Q_ordered$Region_Country))

# Add the order of the last individual of each country to ensure correct placement of borders
borders$order <-
  sapply(borders$Region_Country, function(rc)
    max(which(Q_ordered$Region_Country == rc))) + 0.5  # Shift borders to the right edge of the bars

# Select only the first occurrence of each country in the ordered data
label_df <- Q_ordered |>
  filter(!is.na(label)) |>
  distinct(label, .keep_all = TRUE)

# Create a custom label function
label_func <- function(x) {
  labels <- rep("", length(x))
  labels[x %in% label_df$ind] <- label_df$label
  labels
}

# Calculate the position of lines
border_positions <- Q_ordered |>
  group_by(Country) |>
  summarise(pos = max(as.numeric(ind)) + 0)

# Calculate the position of population labels and bars
pop_labels <- Q_ordered |>
  mutate(Name = paste(pop, Pop_City, sep = " - ")) |>
  group_by(pop) |>
  slice_head(n = 1) |>
  ungroup() |>
  dplyr::select(ind, Pop_City, Country, Name) |>
  mutate(pos = as.numeric(ind))  # calculate position of population labels

pop_labels_bars <- pop_labels |>
  mutate(pos = as.numeric(ind)  - .5)


# Calculate the position of lines
border_positions <- Q_ordered |>
  group_by(Country) |>
  summarise(pos = max(as.numeric(ind)) - 1)


pop_labels_bars <- pop_labels |>
  mutate(pos = as.numeric(ind)  - .5)

# Function to filter and normalize data
normalize_data <- function(df, min_value) {
  df |>
    filter(value > min_value) |>
    group_by(ind) |>
    mutate(value = value / sum(value))
}

# Use the function
Q_grouped_filtered <- normalize_data(Q_grouped, 0.1)
# 

color_palette <-
  c(
    "v1" = "#FFB347",
    "v2" = "red",
    "v3" = "#F49AC2",
    "v4" = "#FFFF99",
    "v5" = "#AE9393"
    )
# Generate all potential variable names
all_variables <- paste0("v", 1:5)

# Map each variable to a name
color_mapping <- data.frame(variable = all_variables,
                            color = names(color_palette))

# Merge with Q_grouped_filtered
Q_grouped_filtered <- merge(Q_grouped_filtered, color_mapping, by = "variable")

# Create the plot
ggplot(Q_grouped_filtered, aes(x = as.factor(ind), y = value, fill = color)) +
  geom_bar(stat = 'identity', width = 1) +
  geom_vline(
    data = pop_labels_bars,
    aes(xintercept = pos),
    color = "#2C444A",
    linewidth = .2
  ) +
  geom_text(
    data = pop_labels,
    aes(x = as.numeric(ind), y = 1, label = Name),
    vjust = 1.5,
    hjust = 0,
    size = 2,
    angle = 90,
    inherit.aes = FALSE
  ) +
  my_theme() +
  theme(
    axis.text.x = element_text(
      angle = 90,
      hjust = 1,
      size = 12
    ),
    legend.position = "none",
    plot.margin = unit(c(3, 0.5, 0.5, 0.5), "cm")
  ) +
  xlab("Admixture matrix") +
  ylab("Ancestry proportions") +
  labs(caption = "Each bar represents the ancestry proportions for an individual for k=5.\n fastStructure logistic priopr for k1:30 with 20,931 SNPs.") +
  scale_x_discrete(labels = label_func) +
  scale_fill_manual(values = color_palette) +
  expand_limits(y = c(0, 1.5))
```

```
# # save it
ggsave(
  here("output", "populations", "figures", "faststructure_logistic_r2_0.01_k5.pdf"),
  width  = 12,
  height = 6,
  units  = "in",
  device = cairo_pdf
)
```

### 2.3 r2 0.1 set

Create a directory for each run

```
cd /gpfs/ycga/project/caccone/lvc26/faststructure/r2_0.1
for i in $(seq 1 10) # we will not use -w because we will run 1 run with the logistic prior
do
  mkdir run$i
done
```

Now we can generate the full script for dsq (not showing here,
because it has 2,000 lines).

#### 2.3.1 Simple prior

```
for k in $(seq 1 30); do
    for run in $(seq 1 10); do
        echo "cd /gpfs/ycga/project/caccone/lvc26/faststructure; source /vast/palmer/apps/avx2/software/miniconda/23.3.1/etc/profile.d/conda.sh;module load miniconda/23.3.1; conda activate fastStructure; export PYTHONPATH=/home/lvc26/project/fastStructure; python -m structure -K $k --input=/gpfs/ycga/project/caccone/lvc26/neuroadmix/snps_sets/r2_0.1 --output=/gpfs/ycga/project/caccone/lvc26/faststructure/r2_0.1/run$run/simple --prior=simple --full --cv=10 --tol=10e-6"
    done
done > dsq1.txt
```

#### 2.3.2 Logistic prior

Only 1 run I used 10 runs for the neutral set because it is around 9k
SNPs. For other data sets we can do less runs because it takes too
long.

```
for k in $(seq 1 30); do
    for run in $(seq 1); do
        echo "cd /gpfs/ycga/project/caccone/lvc26/faststructure; source /vast/palmer/apps/avx2/software/miniconda/23.3.1/etc/profile.d/conda.sh;module load miniconda/23.3.1; conda activate fastStructure; export PYTHONPATH=/home/lvc26/project/fastStructure; python -m structure -K $k --input=/gpfs/ycga/project/caccone/lvc26/neuroadmix/snps_sets/r2_0.1 --output=/gpfs/ycga/project/caccone/lvc26/faststructure/r2_0.1/run$run/logistic --prior=logistic --full --cv=10 --tol=10e-6"
    done
done > dsq2.txt
```

Load dsq, and then create the batch file. Check the dsq –help

#### 2.3.3 Generate the dsq batch files

```
module load dSQ/1.05
##############################################
#                 Simple
##############################################
dsq \
--job-file dsq1.txt \
--mem-per-cpu 1g \
--cpus-per-task=1 \
-t 24:00:00 \
--mail-type END \
--partition=scavenge \
--job-name fastStr_s1 \
--output /dev/null \
--batch-file 1.simple.sh


##############################################
#                 Logistic
##############################################
dsq \
--job-file dsq2.txt \
--mem-per-cpu 1g \
--cpus-per-task=1 \
--time 7-00:00:00 \
--mail-type END \
--partition=week \
--job-name fastStr_L1 \
--output /dev/null \
--batch-file 2.logistic.sh
```

Submit

```
sbatch 1.simple.sh
dsqa -j 6274779

sbatch 2.logistic.sh
dsqa -j 6274782
```

#### 2.3.4 Parse files from runs

Next, we will need to parse the results and make a plot. Our fist
step is done on the cluster. We need to grab the Q matrices and the log
files from our runs. We start wit the simple priors. We first find the
optimal number of Ks. Then we use pong to parse all 10 runs and generate
one structure like plot. You can get pong here https://github.com/ramachandran-lab/pong. I installed
pong on my base environment in my laptop. You can create a environment
if you want, check their easy to follow instructions.

Data location

```
cd /gpfs/ycga/project/caccone/lvc26/faststructure/r2_0.1
```

#### 2.3.5 Choose k simple plot

Our first step is to use fastStructure script chooseK.py to find the
number of ancestral populations. The main reason we did 10 runs is
because we don’t find the same optimal k for every run. Since we already
created a conda environment to run fastStructure, we can activate it,
and run chooseK.

First for our runs with the simple prior.

```
salloc --time=06:00:00 --nodes=1 --ntasks=1 --cpus-per-task=1 --mem-per-cpu=5120
module load miniconda/23.3.1
conda activate fastStructure
export PYTHONPATH=/home/lvc26/project/fastStructure


# simple 10 runs
cat <(echo 'run likelihood structure') \
<(for i in /gpfs/ycga/project/caccone/lvc26/faststructure/r2_0.1/run*
do
    echo $i | sed 's@.*/@@' | sed 's/run/run /' ; python -m chooseK --input=$i/simple | grep 'likelihood' | awk '{print $6, $8}'; python -m chooseK --input=$i/simple | grep 'structure' |  awk '{print $6, $10}'
done | xargs -n6 | awk '{print $2, $4, $6}') > simple_prior_models.txt

# logistic 1 run
cat <(echo 'run likelihood structure') \
<(for i in /gpfs/ycga/project/caccone/lvc26/faststructure/r2_0.01/run1
do
    echo $i | sed 's@.*/@@' | sed 's/run/run /' ; python -m chooseK --input=$i/logistic | grep 'likelihood' | awk '{print $6, $8}'; python -m chooseK --input=$i/logistic | grep 'structure' |  awk '{print $6, $10}'
done | xargs -n6 | awk '{print $2, $4, $6}') > logistic_prior_models.txt
```

Download the data

```
rsync -chavzP --stats lvc26@mccleary.ycrc.yale.edu:/gpfs/ycga/project/caccone/lvc26/faststructure/r2_0.1/*_prior_models.txt /Users/lucianocosme/Library/CloudStorage/Dropbox/Albopictus/manuscript_chip/data/no_autogenous/albo_chip/output/populations/faststructure/r2_0.1
```

We can make a plot with the Ks. Let’s import the data in R.

```
# function to import our choosek.py data
import_fastStructure <- function(file) {
  # import as a tibble and set columns as integers
  dat <- read_delim(
    file,
    col_names      = TRUE,
    show_col_types = FALSE,
    col_types      = "iii"
  )

  # get columns we need and make it long for plotting
  dat <- dat |>
    gather(
      structure, likelihood, -run
    )

  # rename the columns by index
  dat <- dat |>
    rename(
      run   = 1,
      model = 2,
      k     = 3
    )
  return(dat)
}
# we can save the function to source it later
dump(
  "import_fastStructure",
  here(
    "scripts", "analysis", "import_fastStructure.R"
  ),
  append = TRUE
)
```

Now we can use the function to import the data

```
# we can use our function with here to import the data
choose_k_simple <- 
  import_fastStructure(
  here(
    "output", "populations","fastStructure", "r2_0.1","simple_prior_models.txt"
  )
)
```

Function to plot fastStructure choosek.py results

```
# function to plot our choosek.py data
plot_fastStructure <- function(df) {
  df |>
    ggplot() +
    geom_line(
      aes(
        x              = run,
        y              = k,
        color          = model
      ),
      linewidth = 1
    ) +
    scale_colour_manual(
      "model",
      values = c(
        structure      = "#9d60ff",
        likelihood     = "#ffc08c"
      ),
      labels = c(
        "Maximizes \n Likelihood \n", "Explain \n Structure"
      )
    ) +
    labs(
      x                = "Run",
      y                = "K",
      title            = "fastStructure",
      caption          = "algorithm runs for choices of K ranging from 1 to 30"
    ) +
    hrbrthemes::theme_ipsum(
      base_family = "",
      axis_text_size = 12,
      axis_title_size = 14,
      plot_margin = margin(
        10, 10, 10, 10
      ),
      grid = TRUE,
      grid_col = "#fabbe2"
    ) +
    theme(
      panel.grid.major = element_line(
        linetype       = "dashed",
        linewidth      = 0.2
      ),
      panel.grid.minor = element_line(
        linetype       = "dashed",
        linewidth      = 0.2
      ),
      legend.text = element_text(
        size = 12
      ),
      legend.title = element_text(
        size           = 14,
        face           = "bold"
      ),
      legend.position = "right"
    )
}
# we can save the function to source it later
dump(
  "plot_fastStructure",
  here(
    "scripts", "analysis", "plot_fastStructure.R"
  ),
  append = TRUE
)
```

Use our function to plot k values

```
plot_fastStructure(
  choose_k_simple
) +
  labs(
    subtitle = "simple prior"
  )
```

```
# save plot
ggsave(
  here(
    "output", "figures", "fastStructure_r2_0.1_simple_prior_k1_k30.pdf"
  ),
  width  = 8,
  height = 6,
  units  = "in"
)
```

#### 2.3.6 Choose k logistic

Import logistic data

```
# import the data
choose_k_logistic <- import_fastStructure(
  here(
    "output", "populations","fastStructure", "r2_0.1","logistic_prior_models.txt"
  )
)
# only 1 run
choose_k_logistic
```

```
## # A tibble: 2 × 3
##     run model          k
##   <int> <chr>      <int>
## 1     1 likelihood    30
## 2     1 structure     13
```

#### 2.3.7 Statistical tests - comparing simple and logistic prior runs

```
set.seed(123)  # for reproducibility
# import data
# simple
simple1 <- import_fastStructure(
  here(
    "output", "populations","fastStructure", "r2_0.1", "simple_prior_models.txt"
  )
) |>
  mutate(
    prior = "simple"
  )
#
# logistic
logistic1 <- import_fastStructure(
  here(
    "output", "populations","fastStructure", "r2_0.1", "logistic_prior_models.txt"
  )
) |>
  mutate(
    prior = "logistic"
  )
#
# rbind data set
fastStruc_prior <- rbind(
  simple1, logistic1
) |>
  mutate(
    prior = factor(
      prior
    )
  )
#
# run stats
grouped_ggbetweenstats(
  data = fastStruc_prior,
  x = prior,
  y = k,
  grouping.var = model,
  p.adjust.method = "bonferroni",
  palette = "default_jama",
  package = "ggsci",
  plotgrid.args = list(
    nrow = 1
  ),
  annotation.args = list(
    title = "Comparison of fastStructure prior"
  )
)
```

```
# # save plot
ggsave(
  here(
    "output", "populations", "figures", "welch_faststructure_r2_0.1_prior_comparisons.pdf"
  ),
  width = 11,
  height = 8,
  units = "in"
)
```

The number of model components that explain the structure in the data
is different from Admixture. For our simple model explaining the
structure in the data we have k=13, and logistic k=16, whereas Admixture
indicated k=5. We can plot k=5 to see how it compares to the Admixture,
LEA, and neuro-admixture.

#### 2.3.8 pong

We first need to find the most common mode among runs. We do not need
to plot all matrices, we can plot the first 12 using pong.

Download the data

```
# next we can download the files to our computer, run the command below in your computer, not in the cluster
rsync -chavzP --stats --include='*/' --include='*.meanQ' --exclude='*' lvc26@mccleary.ycrc.yale.edu:/gpfs/ycga/project/caccone/lvc26/faststructure/r2_0.1* /Users/lucianocosme/Library/CloudStorage/Dropbox/Albopictus/manuscript_chip/data/no_autogenous/albo_chip/output/populations/faststructure/
```

##### 2.3.8.1 simple prior

Check the downloaded data

```
ls -1 output/populations/faststructure/r2_0.1/run*/simple* | tail
```

Create filemap2.txt

```
# we will use the 10 runs to make the plot. 
# loop to generate filemap2.txt with k values from 2 to 12. I use tab instead of space. We need that for pong.
for k in $(seq 2 12); do
    for run in $(seq 1 10); do
        echo "k$k""r$run $k faststructure/r2_0.1/run$run/simple.$k.meanQ" | tr ' ' '\t'
    done
done > output/populations/filemap2.txt;
head -n 5 output/populations/filemap2.txt
```

```
wc -l output/populations/filemap2.txt
```

Create ind2pop

```
rm output/populations/ind2pop.txt;
cat output/populations/snps_sets/r2_0.1.fam | awk '{print $1}' > output/populations/ind2pop.txt;
head -n 5 output/populations/ind2pop.txt
```

Run on the terminal and not on RStudio.

```
# on your computer to visualize the results
pong \
--filemap output/populations/filemap2.txt \
--ind2pop output/populations/ind2pop.txt  \
--dist_metric jaccard \
--sim_threshold 0.82 \
--greedy \
--force \
--output_dir output/populations/sim_threshold_0.82 \
--verbose
```

1 mode for k=5, so any run works.

###### 2.3.8.1.1 Create plot simple prior with Q matrix identified with pong

Make plot

```
# Extract ancestry coefficients
k5run1 <- read_delim(
  here("output", "populations", "faststructure", "r2_0.1", "run1", "simple.5.meanQ"),
  delim = "  ", # Specify the delimiter if different from the default (comma)
  col_names = FALSE,
  show_col_types = FALSE
) 

head(k5run1)
```

```
## # A tibble: 6 × 5
##         X1       X2       X3    X4       X5
##      <dbl>    <dbl>    <dbl> <dbl>    <dbl>
## 1 0.000002 0.000002 0.0114   0.791 0.198   
## 2 0.146    0.000002 0.000002 0.694 0.160   
## 3 0.000002 0.000002 0.000002 0.949 0.0510  
## 4 0.000002 0.000002 0.000002 1.00  0.000002
## 5 0.000002 0.000002 0.000002 1.00  0.000002
## 6 0.000002 0.000002 0.000002 1.00  0.000002
```

The fam file

```
fam_file <- here(
  "output", "populations", "snps_sets", "r2_0.1.fam"
)

# Read the .fam file
fam_data <- read.table(fam_file, 
                       header = FALSE,
                       col.names = c("FamilyID", "IndividualID", "PaternalID", "MaternalID", "Sex", "Phenotype"))

# View the first few rows
head(fam_data)
```

```
##   FamilyID IndividualID PaternalID MaternalID Sex Phenotype
## 1      OKI         1001          0          0   2        -9
## 2      OKI         1002          0          0   2        -9
## 3      OKI         1003          0          0   2        -9
## 4      OKI         1004          0          0   2        -9
## 5      OKI         1005          0          0   2        -9
## 6      OKI         1006          0          0   1        -9
```

Create ID column

```
# Change column name
colnames(fam_data)[colnames(fam_data) == "IndividualID"] <- "ind"


# Merge columns "FamilyID" and "IndividualID" with an underscore
# fam_data$ind <- paste(fam_data$FamilyID, fam_data$IndividualID, sep = "_")


# Change column name
colnames(fam_data)[colnames(fam_data) == "FamilyID"] <- "pop"

# Select ID
fam_data <- fam_data |>
  dplyr::select("ind", "pop")

# View the first few rows
head(fam_data)
```

```
##    ind pop
## 1 1001 OKI
## 2 1002 OKI
## 3 1003 OKI
## 4 1004 OKI
## 5 1005 OKI
## 6 1006 OKI
```

Add it to matrix

```
k5run1 <- fam_data |>
  dplyr::select(ind, pop) |>
  bind_cols(k5run1)

head(k5run1)
```

```
##    ind pop       X1    X2       X3       X4       X5
## 1 1001 OKI 0.000002 2e-06 0.011353 0.790797 0.197846
## 2 1002 OKI 0.146314 2e-06 0.000002 0.694085 0.159598
## 3 1003 OKI 0.000002 2e-06 0.000002 0.949043 0.050951
## 4 1004 OKI 0.000002 2e-06 0.000002 0.999993 0.000002
## 5 1005 OKI 0.000002 2e-06 0.000002 0.999993 0.000002
## 6 1006 OKI 0.000002 2e-06 0.000002 0.999993 0.000002
```

Rename the columns

```
# Rename the columns starting from the third one
k5run1 <- k5run1 |>
  rename_with(~paste0("v", seq_along(.x)), .cols = -c(ind, pop))

# View the first few rows
head(k5run1)
```

```
##    ind pop       v1    v2       v3       v4       v5
## 1 1001 OKI 0.000002 2e-06 0.011353 0.790797 0.197846
## 2 1002 OKI 0.146314 2e-06 0.000002 0.694085 0.159598
## 3 1003 OKI 0.000002 2e-06 0.000002 0.949043 0.050951
## 4 1004 OKI 0.000002 2e-06 0.000002 0.999993 0.000002
## 5 1005 OKI 0.000002 2e-06 0.000002 0.999993 0.000002
## 6 1006 OKI 0.000002 2e-06 0.000002 0.999993 0.000002
```

Import sample locations

```
sampling_loc <- readRDS(here("output", "local_adaptation", "sampling_loc.rds"))
head(sampling_loc)
```

```
## # A tibble: 6 × 6
##   Pop_City   Country  Latitude Longitude Region Abbreviation
##   <chr>      <chr>       <dbl>     <dbl> <chr>  <chr>       
## 1 Gelephu    Bhutan       26.9      90.5 Asia   GEL         
## 2 Phnom Penh Cambodia     11.6     105.  Asia   CAM         
## 3 Hainan     China        19.2     110.  Asia   HAI         
## 4 Yunnan     China        24.5     101.  Asia   YUN         
## 5 Hunan      China        27.6     112.  Asia   HUN         
## 6 Bengaluru  India        13.0      77.6 Asia   BEN
```

```
source(
  here(
    "scripts", "analysis", "my_theme3.R"
  )
)

# Create a named vector to map countries to regions
country_to_region <- c(
  "Bhutan" = "South Asia",
  "Cambodia" = "Southeast Asia",
  "China" = "East Asia",
  "India" = "South Asia",
  "Indonesia" = "Southeast Asia",
  "Japan" = "East Asia",
  "Malaysia" = "Southeast Asia",
  "Maldives" = "South Asia",
  "Nepal" = "South Asia",
  "Sri Lanka" = "South Asia",
  "Taiwan" = "East Asia",
  "Thailand" = "Southeast Asia",
  "Vietnam" = "Southeast Asia"
)

# Add the region to the data frame
sampling_loc$Region2 <- country_to_region[sampling_loc$Country]

# Melt the data frame for plotting
Q_melted <- k5run1 |>
  pivot_longer(
    cols = -c(ind, pop),
    names_to = "variable",
    values_to = "value"
  )
# Join with sampling_loc to get sampling localities
Q_joined <- Q_melted |>
  left_join(sampling_loc, by = c("pop" = "Abbreviation"))

# Create a combined variable for Region and Country
Q_joined <- Q_joined |>
  mutate(Region_Country = interaction(Region, Country, sep = "_"))

# Order the combined variable by Region and Country, then by individual
Q_ordered <- Q_joined |>
  arrange(Region, Region2, Country, ind) |>
  mutate(ind = factor(ind, levels = unique(ind)))  # Convert ind to a factor with levels in the desired order

# Add labels: country names for the first individual in each country, NA for all other individuals
Q_ordered <- Q_ordered |>
  group_by(Region_Country) |>
  mutate(label = ifelse(row_number() == 1, as.character(Country), NA))

# Group by individual and variable, calculate mean ancestry proportions
Q_grouped <- Q_ordered |>
  group_by(ind, variable) |>
  summarise(value = mean(value), .groups = "drop")

# Create a data frame for borders
borders <-
  data.frame(Region_Country = unique(Q_ordered$Region_Country))

# Add the order of the last individual of each country to ensure correct placement of borders
borders$order <-
  sapply(borders$Region_Country, function(rc)
    max(which(Q_ordered$Region_Country == rc))) + 0.5  # Shift borders to the right edge of the bars

# Select only the first occurrence of each country in the ordered data
label_df <- Q_ordered |>
  filter(!is.na(label)) |>
  distinct(label, .keep_all = TRUE)

# Create a custom label function
label_func <- function(x) {
  labels <- rep("", length(x))
  labels[x %in% label_df$ind] <- label_df$label
  labels
}

# Calculate the position of lines
border_positions <- Q_ordered |>
  group_by(Country) |>
  summarise(pos = max(as.numeric(ind)) + 0)

# Calculate the position of population labels and bars
pop_labels <- Q_ordered |>
  mutate(Name = paste(pop, Pop_City, sep = " - ")) |>
  group_by(pop) |>
  slice_head(n = 1) |>
  ungroup() |>
  dplyr::select(ind, Pop_City, Country, Name) |>
  mutate(pos = as.numeric(ind))  # calculate position of population labels

pop_labels_bars <- pop_labels |>
  mutate(pos = as.numeric(ind)  - .5)


# Calculate the position of lines
border_positions <- Q_ordered |>
  group_by(Country) |>
  summarise(pos = max(as.numeric(ind)) - 1)


pop_labels_bars <- pop_labels |>
  mutate(pos = as.numeric(ind)  - .5)

# Function to filter and normalize data
normalize_data <- function(df, min_value) {
  df |>
    filter(value > min_value) |>
    group_by(ind) |>
    mutate(value = value / sum(value))
}

# Use the function
Q_grouped_filtered <- normalize_data(Q_grouped, 0.1)
# 

color_palette <-
  c(
    "v1" = "#F49AC2",
    "v2" = "red",
    "v3" = "#FFFF99",
    "v4" = "#AE9393",
    "v5" = "#FFB347"
  )
# Generate all potential variable names
all_variables <- paste0("v", 1:5)

# Map each variable to a name
color_mapping <- data.frame(variable = all_variables,
                            color = names(color_palette))

# Merge with Q_grouped_filtered
Q_grouped_filtered <- merge(Q_grouped_filtered, color_mapping, by = "variable")

# Create the plot
ggplot(Q_grouped_filtered, aes(x = as.factor(ind), y = value, fill = color)) +
  geom_bar(stat = 'identity', width = 1) +
  geom_vline(
    data = pop_labels_bars,
    aes(xintercept = pos),
    color = "#2C444A",
    linewidth = .2
  ) +
  geom_text(
    data = pop_labels,
    aes(x = as.numeric(ind), y = 1, label = Name),
    vjust = 1.5,
    hjust = 0,
    size = 2,
    angle = 90,
    inherit.aes = FALSE
  ) +
  my_theme() +
  theme(
    axis.text.x = element_text(
      angle = 90,
      hjust = 1,
      size = 12
    ),
    legend.position = "none",
    plot.margin = unit(c(3, 0.5, 0.5, 0.5), "cm")
  ) +
  xlab("Admixture matrix") +
  ylab("Ancestry proportions") +
  labs(caption = "Each bar represents the ancestry proportions for an individual for k=5.\n fastStructure simple prior for k1:30 with 57,780 SNPs.") +
  scale_x_discrete(labels = label_func) +
  scale_fill_manual(values = color_palette) +
  expand_limits(y = c(0, 1.5))
```

```
# # save it
ggsave(
  here("output", "populations", "figures", "faststructure_simple_r2_0.1_k5.pdf"),
  width  = 12,
  height = 6,
  units  = "in",
  device = cairo_pdf
)
```

##### 2.3.8.1 logistic prior

Check the downloaded data

```
ls -1 output/populations/faststructure/r2_0.1/run*/logistic* | tail
```

We do not need to use pong because we have only 1 run for the
logistic.

###### 2.3.8.1.1 Create plot simple prior with Q matrix identified with pong

Make plot

```
# Extract ancestry coefficients
k5run1 <- read_delim(
  here("output", "populations", "faststructure", "r2_0.1", "run1", "logistic.5.meanQ"),
  delim = "  ", # Specify the delimiter if different from the default (comma)
  col_names = FALSE,
  show_col_types = FALSE
) 

head(k5run1)
```

```
## # A tibble: 6 × 5
##         X1       X2     X3       X4    X5
##      <dbl>    <dbl>  <dbl>    <dbl> <dbl>
## 1 0.000002 0.000002 0.102  0.000002 0.898
## 2 0.000002 0.000002 0.0529 0.0395   0.908
## 3 0.000002 0.000002 0.0328 0.000002 0.967
## 4 0.000002 0.000002 0.0318 0.000002 0.968
## 5 0.000002 0.000002 0.0112 0.000002 0.989
## 6 0.000002 0.000002 0.0348 0.000002 0.965
```

The fam file

```
fam_file <- here(
  "output", "populations", "snps_sets", "r2_0.1.fam"
)

# Read the .fam file
fam_data <- read.table(fam_file, 
                       header = FALSE,
                       col.names = c("FamilyID", "IndividualID", "PaternalID", "MaternalID", "Sex", "Phenotype"))

# View the first few rows
head(fam_data)
```

```
##   FamilyID IndividualID PaternalID MaternalID Sex Phenotype
## 1      OKI         1001          0          0   2        -9
## 2      OKI         1002          0          0   2        -9
## 3      OKI         1003          0          0   2        -9
## 4      OKI         1004          0          0   2        -9
## 5      OKI         1005          0          0   2        -9
## 6      OKI         1006          0          0   1        -9
```

Create ID column

```
# Change column name
colnames(fam_data)[colnames(fam_data) == "IndividualID"] <- "ind"


# Merge columns "FamilyID" and "IndividualID" with an underscore
# fam_data$ind <- paste(fam_data$FamilyID, fam_data$IndividualID, sep = "_")


# Change column name
colnames(fam_data)[colnames(fam_data) == "FamilyID"] <- "pop"

# Select ID
fam_data <- fam_data |>
  dplyr::select("ind", "pop")

# View the first few rows
head(fam_data)
```

```
##    ind pop
## 1 1001 OKI
## 2 1002 OKI
## 3 1003 OKI
## 4 1004 OKI
## 5 1005 OKI
## 6 1006 OKI
```

Add it to matrix

```
k5run1 <- fam_data |>
  dplyr::select(ind, pop) |>
  bind_cols(k5run1)

head(k5run1)
```

```
##    ind pop    X1    X2       X3       X4       X5
## 1 1001 OKI 2e-06 2e-06 0.102183 0.000002 0.897812
## 2 1002 OKI 2e-06 2e-06 0.052898 0.039471 0.907627
## 3 1003 OKI 2e-06 2e-06 0.032833 0.000002 0.967162
## 4 1004 OKI 2e-06 2e-06 0.031840 0.000002 0.968155
## 5 1005 OKI 2e-06 2e-06 0.011165 0.000002 0.988830
## 6 1006 OKI 2e-06 2e-06 0.034841 0.000002 0.965154
```

Rename the columns

```
# Rename the columns starting from the third one
k5run1 <- k5run1 |>
  rename_with(~paste0("v", seq_along(.x)), .cols = -c(ind, pop))

# View the first few rows
head(k5run1)
```

```
##    ind pop    v1    v2       v3       v4       v5
## 1 1001 OKI 2e-06 2e-06 0.102183 0.000002 0.897812
## 2 1002 OKI 2e-06 2e-06 0.052898 0.039471 0.907627
## 3 1003 OKI 2e-06 2e-06 0.032833 0.000002 0.967162
## 4 1004 OKI 2e-06 2e-06 0.031840 0.000002 0.968155
## 5 1005 OKI 2e-06 2e-06 0.011165 0.000002 0.988830
## 6 1006 OKI 2e-06 2e-06 0.034841 0.000002 0.965154
```

Import sample locations

```
sampling_loc <- readRDS(here("output", "local_adaptation", "sampling_loc.rds"))
head(sampling_loc)
```

```
## # A tibble: 6 × 6
##   Pop_City   Country  Latitude Longitude Region Abbreviation
##   <chr>      <chr>       <dbl>     <dbl> <chr>  <chr>       
## 1 Gelephu    Bhutan       26.9      90.5 Asia   GEL         
## 2 Phnom Penh Cambodia     11.6     105.  Asia   CAM         
## 3 Hainan     China        19.2     110.  Asia   HAI         
## 4 Yunnan     China        24.5     101.  Asia   YUN         
## 5 Hunan      China        27.6     112.  Asia   HUN         
## 6 Bengaluru  India        13.0      77.6 Asia   BEN
```

```
source(
  here(
    "scripts", "analysis", "my_theme3.R"
  )
)

# Create a named vector to map countries to regions
country_to_region <- c(
  "Bhutan" = "South Asia",
  "Cambodia" = "Southeast Asia",
  "China" = "East Asia",
  "India" = "South Asia",
  "Indonesia" = "Southeast Asia",
  "Japan" = "East Asia",
  "Malaysia" = "Southeast Asia",
  "Maldives" = "South Asia",
  "Nepal" = "South Asia",
  "Sri Lanka" = "South Asia",
  "Taiwan" = "East Asia",
  "Thailand" = "Southeast Asia",
  "Vietnam" = "Southeast Asia"
)

# Add the region to the data frame
sampling_loc$Region2 <- country_to_region[sampling_loc$Country]

# Melt the data frame for plotting
Q_melted <- k5run1 |>
  pivot_longer(
    cols = -c(ind, pop),
    names_to = "variable",
    values_to = "value"
  )
# Join with sampling_loc to get sampling localities
Q_joined <- Q_melted |>
  left_join(sampling_loc, by = c("pop" = "Abbreviation"))

# Create a combined variable for Region and Country
Q_joined <- Q_joined |>
  mutate(Region_Country = interaction(Region, Country, sep = "_"))

# Order the combined variable by Region and Country, then by individual
Q_ordered <- Q_joined |>
  arrange(Region, Region2, Country, ind) |>
  mutate(ind = factor(ind, levels = unique(ind)))  # Convert ind to a factor with levels in the desired order

# Add labels: country names for the first individual in each country, NA for all other individuals
Q_ordered <- Q_ordered |>
  group_by(Region_Country) |>
  mutate(label = ifelse(row_number() == 1, as.character(Country), NA))

# Group by individual and variable, calculate mean ancestry proportions
Q_grouped <- Q_ordered |>
  group_by(ind, variable) |>
  summarise(value = mean(value), .groups = "drop")

# Create a data frame for borders
borders <-
  data.frame(Region_Country = unique(Q_ordered$Region_Country))

# Add the order of the last individual of each country to ensure correct placement of borders
borders$order <-
  sapply(borders$Region_Country, function(rc)
    max(which(Q_ordered$Region_Country == rc))) + 0.5  # Shift borders to the right edge of the bars

# Select only the first occurrence of each country in the ordered data
label_df <- Q_ordered |>
  filter(!is.na(label)) |>
  distinct(label, .keep_all = TRUE)

# Create a custom label function
label_func <- function(x) {
  labels <- rep("", length(x))
  labels[x %in% label_df$ind] <- label_df$label
  labels
}

# Calculate the position of lines
border_positions <- Q_ordered |>
  group_by(Country) |>
  summarise(pos = max(as.numeric(ind)) + 0)

# Calculate the position of population labels and bars
pop_labels <- Q_ordered |>
  mutate(Name = paste(pop, Pop_City, sep = " - ")) |>
  group_by(pop) |>
  slice_head(n = 1) |>
  ungroup() |>
  dplyr::select(ind, Pop_City, Country, Name) |>
  mutate(pos = as.numeric(ind))  # calculate position of population labels

pop_labels_bars <- pop_labels |>
  mutate(pos = as.numeric(ind)  - .5)


# Calculate the position of lines
border_positions <- Q_ordered |>
  group_by(Country) |>
  summarise(pos = max(as.numeric(ind)) - 1)


pop_labels_bars <- pop_labels |>
  mutate(pos = as.numeric(ind)  - .5)

# Function to filter and normalize data
normalize_data <- function(df, min_value) {
  df |>
    filter(value > min_value) |>
    group_by(ind) |>
    mutate(value = value / sum(value))
}

# Use the function
Q_grouped_filtered <- normalize_data(Q_grouped, 0.1)
# 

color_palette <-
  c(
    "v1" = "#F49AC2",
    "v2" = "red",
    "v3" = "#FFB347",
    "v4" = "#AE9393",
    "v5" = "#FFFF99"
  )
# Generate all potential variable names
all_variables <- paste0("v", 1:5)

# Map each variable to a name
color_mapping <- data.frame(variable = all_variables,
                            color = names(color_palette))

# Merge with Q_grouped_filtered
Q_grouped_filtered <- merge(Q_grouped_filtered, color_mapping, by = "variable")

# Create the plot
ggplot(Q_grouped_filtered, aes(x = as.factor(ind), y = value, fill = color)) +
  geom_bar(stat = 'identity', width = 1) +
  geom_vline(
    data = pop_labels_bars,
    aes(xintercept = pos),
    color = "#2C444A",
    linewidth = .2
  ) +
  geom_text(
    data = pop_labels,
    aes(x = as.numeric(ind), y = 1, label = Name),
    vjust = 1.5,
    hjust = 0,
    size = 2,
    angle = 90,
    inherit.aes = FALSE
  ) +
  my_theme() +
  theme(
    axis.text.x = element_text(
      angle = 90,
      hjust = 1,
      size = 12
    ),
    legend.position = "none",
    plot.margin = unit(c(3, 0.5, 0.5, 0.5), "cm")
  ) +
  xlab("Admixture matrix") +
  ylab("Ancestry proportions") +
  labs(caption = "Each bar represents the ancestry proportions for an individual for k=5.\n fastStructure logistic prior for k1:30 with 57,780 SNPs.") +
  scale_x_discrete(labels = label_func) +
  scale_fill_manual(values = color_palette) +
  expand_limits(y = c(0, 1.5))
```

```
# # save it
ggsave(
  here("output", "populations", "figures", "faststructure_logistic_r2_0.1_k5.pdf"),
  width  = 12,
  height = 6,
  units  = "in",
  device = cairo_pdf
)
```
