# Supplementary material for: A genotyping array for the globally invasive vector mosquito, Aedes albopictus
Source: Parasit Vectors. 2024 Mar 4;17:106. doi: 10.1186/s13071-024-06158-z (PMC10910840; doi:10.1186/s13071-024-06158-z)
Supplement: Supplementary file 12 — Additional file 12. Neural Admixture analysis. [file 13071_2024_6158_MOESM12_ESM.html]

Aedes albopictus SNP chip - Ancestry analysis with Neuro-admixture


# Aedes albopictus SNP chip - Ancestry analysis with Neuro-admixture

#### Luciano V Cosme

#### 2023-08-29

- A. Get
  neuro-admixture and set up a run to test it
- 1. Train with r2 0.01 filter
  - 1.1.
    Plot after training
  - 1.2 Inference with all
    populations
  - 1.3 Plot after
    inference with all populations
  - 1.4 Plot
    inference
- 2. Train neuro admixture
  with SNPS r2 0.1
  - 2.1.
    Plot after training
  - 2.2 Inference with all
    populations
  - 2.3 Plot after
    inference with all populations
  - 2.4 Plot
    inference
- 3. Train with neutral SNPs
  - 3.1.
    Plot after training
  - 3.2 Inference with all
    populations
  - 3.3 Plot after
    inference with all populations
  - 3.4 Plot
    inference

## A. Get neuro-admixture and set up a run to test it

The software is available at https://github.com/ai-sandbox/neural-admixture

On the HPC we first need to create an interactive session

```
# Create and navigate to the neuroadmixture directory
cd /gpfs/ycga/project/caccone/lvc26/neuroadmix;

# Create interactive session with 1 CPUs
salloc --partition devel --time=06:00:00 --nodes=1 --ntasks=1 --cpus-per-task=4 --mem-per-cpu=5120 zsh;

# Load miniconda (do not use the default it did not work for me)
module load miniconda/23.1.0

# Create a conda environment
conda create --name nadmenv python=3.9
# environment location: /gpfs/gibbs/project/powell/lvc26/conda_envs/nadmenv

# Activate the new env
conda activate nadmenv

# Install neuro-admixture
pip install neural-admixture --user

# To deactivate it later
conda deactivate

# If you want to remove it
# conda env remove -n nadmenv

source /vast/palmer/apps/avx2/software/miniconda/23.1.0/etc/profile.d/conda.sh;
module load miniconda/23.1.0; 
conda activate nadmenv; 
export PYTHONPATH=/home/lvc26/project/conda_envs/nadmenv
```

On the mac laptop

```
pip install neural-admixture
export KMP_DUPLICATE_LIB_OK=TRUE

neural-admixture train --help
```

Libraries

```
library(tidyverse)
library(here)
library(colorout)
library(dplyr)
library(ggplot2)
library(extrafont)
```

## 1. Train with r2 0.01 filter

Prune the data

```
plink2 \
--allow-extra-chr \
--bfile output/populations/file7 \
--indep-pairwise 5 1 0.01 \
--out output/populations/nadmix/indepSNP \
--silent;
grep 'pairwise\|variants\|samples' output/populations/nadmix/indepSNP.log
```

```
##   --indep-pairwise 5 1 0.01
## 237 samples (30 females, 67 males, 140 ambiguous; 237 founders) loaded from
## 82731 variants loaded from output/populations/file7.bim.
## --indep-pairwise (3 compute threads): 61277/82731 variants removed.
```

Look at the Admixture plot with k=5 and choose the populations with
individuals with single ancestry or low admixture (more than 50% of
single ancestry)

```
color_palette <-
  c(
    "V1" = "#AE9393", # KAG
    "V2" = "#F49AC2", # YUN, SSK, BEN
    "V3" = "red", # INJ, INW, SUF, SUU
    "V4" = "#FFB347", # KAN
    "V5" = "#FFFF99"# TAI

  )
```

Create a new bed file for training. Then we use the 60 SNPs with all
populations to test neuro-admixture

```
echo "KAG
YUN
SKK
BEN
INJ
INW
SUF
SUU
KAN
TAI
" > output/populations/nadmix/pops_2_train.txt
```

Subset with plink

```
plink \
--keep-allele-order \
--keep-fam output/populations/nadmix/pops_2_train.txt \
--bfile output/populations/file7 \
--maf 0.1 \
--make-bed \
--out output/populations/nadmix/train/train_r_0.01 \
--extract output/populations/nadmix/indepSNP.prune.in \
--geno 0.2 \
--silent \
--write-snplist
grep "samples\|variants" output/populations/nadmix/train/train_r_0.01.log
```

```
## 82731 variants loaded from .bim file.
## --extract: 21454 variants remaining.
## Total genotyping rate in remaining samples is 0.969198.
## 11 variants removed due to missing genotype data (--geno).
## 512 variants removed due to minor allele threshold(s)
## 20931 variants and 79 people pass filters and QC.
```

Transfer data to cluster (all the data sets are here) - files for
inference

```
rsync -chavzP --stats /Users/lucianocosme/Library/CloudStorage/Dropbox/Albopictus/manuscript_chip/data/no_autogenous/albo_chip/output/populations/nadmix/train lvc26@mccleary.ycrc.yale.edu:/gpfs/ycga/project/caccone/lvc26/neuroadmix
```

We can also transfer the files for training

```
rsync -chavzP --stats /Users/lucianocosme/Library/CloudStorage/Dropbox/Albopictus/manuscript_chip/data/no_autogenous/albo_chip/output/populations/snps_sets lvc26@mccleary.ycrc.yale.edu:/gpfs/ycga/project/caccone/lvc26/neuroadmix
```

Train neuroadmix with a range of ks Get two gpu/cpus

```
salloc --cpus-per-gpu=2 --gpus=2 --time=30:00 --partition gpu_devel
```

Unsupervised (multi-head)

We run admixture with these populations and the recommended k=4
instead of k=5. It is probably because KAN shows admixture with KAG.
Therefore, we can train neuro-admixture with k=4 and see if it will make
the same assignments as admixture did.

```
cd /gpfs/ycga/project/caccone/lvc26/neuroadmix;
module load miniconda/23.1.0;
conda activate nadmenv;

# pckmeans r_0.01
neural-admixture train --seed 1234 --initialization pckmeans --warmup_epochs 1000 --max_epochs 1000 --activation relu --optimizer adam --learning_rate 1e-7 --min_k 2 --max_k 10 --name r_0.01 --data_path /gpfs/ycga/project/caccone/lvc26/neuroadmix/train/train_r_0.01.bed --save_dir /gpfs/ycga/project/caccone/lvc26/neuroadmix/r_0.01 | tee r_0.01.log
```

Download the data

```
rsync -chavzP --stats lvc26@mccleary.ycrc.yale.edu:/gpfs/ycga/project/caccone/lvc26/neuroadmix/r_0.01 /Users/lucianocosme/Library/CloudStorage/Dropbox/Albopictus/manuscript_chip/data/no_autogenous/albo_chip/output/populations/nadmix/results
```

### 1.1. Plot after training

```
# Extract ancestry coefficients
nadmixk4 <- read_delim(
  here("output", "populations", "nadmix", "results", "r_0.01","r_0.01.4.Q"),
  delim = " ", # Specify the delimiter if different from the default (comma)
  col_names = FALSE,
  show_col_types = FALSE
) 

head(nadmixk4)
```

```
## # A tibble: 6 × 4
##        X1    X2      X3       X4
##     <dbl> <dbl>   <dbl>    <dbl>
## 1 0.0356  0.953 0.0111  0.000395
## 2 0.0164  0.965 0.0182  0.000605
## 3 0.00566 0.985 0.00518 0.00430 
## 4 0.00513 0.984 0.00459 0.00583 
## 5 0.0186  0.976 0.00422 0.00122 
## 6 0.0179  0.976 0.00457 0.00191
```

The fam file

```
fam_file <- here(
  "output", "populations", "nadmix", "train","train_r_0.01.fam"
)

# Read the .fam file
fam_data <- read.table(fam_file, 
                       header = FALSE,
                       col.names = c("FamilyID", "IndividualID", "PaternalID", "MaternalID", "Sex", "Phenotype"))

# View the first few rows
head(fam_data)
```

```
##   FamilyID IndividualID PaternalID MaternalID Sex Phenotype
## 1      YUN         1089          0          0   1        -9
## 2      YUN         1090          0          0   1        -9
## 3      YUN         1091          0          0   1        -9
## 4      YUN         1092          0          0   1        -9
## 5      YUN         1093          0          0   1        -9
## 6      YUN         1094          0          0   1        -9
```

Create ID column

```
# Change column name
colnames(fam_data)[colnames(fam_data) == "IndividualID"] <- "ind"


# Merge columns "FamilyID" and "IndividualID" with an underscore
# fam_data$ind <- paste(fam_data$FamilyID, fam_data$IndividualID, sep = "_")


# Change column name
colnames(fam_data)[colnames(fam_data) == "FamilyID"] <- "pop"

# Select ID
fam_data <- fam_data |>
  dplyr::select("ind", "pop")

# View the first few rows
head(fam_data)
```

```
##    ind pop
## 1 1089 YUN
## 2 1090 YUN
## 3 1091 YUN
## 4 1092 YUN
## 5 1093 YUN
## 6 1094 YUN
```

Add it to matrix

```
nadmixk4 <- fam_data |>
  dplyr::select(ind, pop) |>
  bind_cols(nadmixk4)

head(nadmixk4)
```

```
##    ind pop          X1        X2          X3           X4
## 1 1089 YUN 0.035645261 0.9528779 0.011082007 0.0003948842
## 2 1090 YUN 0.016425438 0.9647899 0.018179640 0.0006049462
## 3 1091 YUN 0.005662931 0.9848562 0.005178256 0.0043026549
## 4 1092 YUN 0.005127336 0.9844576 0.004587282 0.0058277580
## 5 1093 YUN 0.018613618 0.9759459 0.004223889 0.0012165380
## 6 1094 YUN 0.017912805 0.9756008 0.004573460 0.0019128845
```

Rename the columns

```
# Rename the columns starting from the third one
nadmixk4 <- nadmixk4 |>
  rename_with(~paste0("v", seq_along(.x)), .cols = -c(ind, pop))

# View the first few rows
head(nadmixk4)
```

```
##    ind pop          v1        v2          v3           v4
## 1 1089 YUN 0.035645261 0.9528779 0.011082007 0.0003948842
## 2 1090 YUN 0.016425438 0.9647899 0.018179640 0.0006049462
## 3 1091 YUN 0.005662931 0.9848562 0.005178256 0.0043026549
## 4 1092 YUN 0.005127336 0.9844576 0.004587282 0.0058277580
## 5 1093 YUN 0.018613618 0.9759459 0.004223889 0.0012165380
## 6 1094 YUN 0.017912805 0.9756008 0.004573460 0.0019128845
```

Import sample locations

```
sampling_loc <- readRDS(here("output", "local_adaptation", "sampling_loc.rds"))
head(sampling_loc)
```

```
## # A tibble: 6 × 6
##   Pop_City   Country  Latitude Longitude Region Abbreviation
##   <chr>      <chr>       <dbl>     <dbl> <chr>  <chr>       
## 1 Gelephu    Bhutan       26.9      90.5 Asia   GEL         
## 2 Phnom Penh Cambodia     11.6     105.  Asia   CAM         
## 3 Hainan     China        19.2     110.  Asia   HAI         
## 4 Yunnan     China        24.5     101.  Asia   YUN         
## 5 Hunan      China        27.6     112.  Asia   HUN         
## 6 Bengaluru  India        13.0      77.6 Asia   BEN
```

```
source(
  here(
    "scripts", "analysis", "my_theme3.R"
  )
)

# Create a named vector to map countries to regions
country_to_region <- c(
  "Bhutan" = "South Asia",
  "Cambodia" = "Southeast Asia",
  "China" = "East Asia",
  "India" = "South Asia",
  "Indonesia" = "Southeast Asia",
  "Japan" = "East Asia",
  "Malaysia" = "Southeast Asia",
  "Maldives" = "South Asia",
  "Nepal" = "South Asia",
  "Sri Lanka" = "South Asia",
  "Taiwan" = "East Asia",
  "Thailand" = "Southeast Asia",
  "Vietnam" = "Southeast Asia"
)

# Add the region to the data frame
sampling_loc$Region2 <- country_to_region[sampling_loc$Country]


# Melt the data frame for plotting
Q_melted <- nadmixk4 |>
  pivot_longer(
    cols = -c(ind, pop),
    names_to = "variable",
    values_to = "value"
  )
# Join with sampling_loc to get sampling localities
Q_joined <- Q_melted |>
  left_join(sampling_loc, by = c("pop" = "Abbreviation"))

# Create a combined variable for Region and Country
Q_joined <- Q_joined |>
  mutate(Region_Country = interaction(Region, Country, sep = "_"))

# Order the combined variable by Region and Country, then by individual
Q_ordered <- Q_joined |>
  arrange(Region, Region2, Country, ind) |>
  mutate(ind = factor(ind, levels = unique(ind)))  # Convert ind to a factor with levels in the desired order

# Add labels: country names for the first individual in each country, NA for all other individuals
Q_ordered <- Q_ordered |>
  group_by(Region_Country) |>
  mutate(label = ifelse(row_number() == 1, as.character(Country), NA))

# Group by individual and variable, calculate mean ancestry proportions
Q_grouped <- Q_ordered |>
  group_by(ind, variable) |>
  summarise(value = mean(value), .groups = "drop")

# Create a data frame for borders
borders <-
  data.frame(Region_Country = unique(Q_ordered$Region_Country))

# Add the order of the last individual of each country to ensure correct placement of borders
borders$order <-
  sapply(borders$Region_Country, function(rc)
    max(which(Q_ordered$Region_Country == rc))) + 0.5  # Shift borders to the right edge of the bars

# Select only the first occurrence of each country in the ordered data
label_df <- Q_ordered |>
  filter(!is.na(label)) |>
  distinct(label, .keep_all = TRUE)

# Create a custom label function
label_func <- function(x) {
  labels <- rep("", length(x))
  labels[x %in% label_df$ind] <- label_df$label
  labels
}

# Calculate the position of lines
border_positions <- Q_ordered |>
  group_by(Country) |>
  summarise(pos = max(as.numeric(ind)) + 0)

# Calculate the position of population labels and bars
pop_labels <- Q_ordered |>
  mutate(Name = paste(pop, Pop_City, sep = " - ")) |>
  group_by(pop) |>
  slice_head(n = 1) |>
  ungroup() |>
  dplyr::select(ind, Pop_City, Country, Name) |>
  mutate(pos = as.numeric(ind))  # calculate position of population labels

pop_labels_bars <- pop_labels |>
  mutate(pos = as.numeric(ind)  - .5)


# Calculate the position of lines
border_positions <- Q_ordered |>
  group_by(Country) |>
  summarise(pos = max(as.numeric(ind)) - 1)


pop_labels_bars <- pop_labels |>
  mutate(pos = as.numeric(ind)  - .5)

# Function to filter and normalize data
normalize_data <- function(df, min_value) {
  df |>
    filter(value > min_value) |>
    group_by(ind) |>
    mutate(value = value / sum(value))
}

# Use the function
Q_grouped_filtered <- normalize_data(Q_grouped, 0.1)

color_palette <-
  c(
    "V1" = "#FFB347",
    "V2" = "#F49AC2",
    "V3" = "red",
    "V4" = "#AE9393"
  )

# Generate all potential variable names
all_variables <- paste0("v", 1:4)

# Map each variable to a name
color_mapping <- data.frame(variable = all_variables,
                            color = names(color_palette))

# Merge with Q_grouped_filtered
Q_grouped_filtered <- merge(Q_grouped_filtered, color_mapping, by = "variable")

# Create the plot
ggplot(Q_grouped_filtered, aes(x = as.factor(ind), y = value, fill = color)) +
  geom_bar(stat = 'identity', width = 1) +
  geom_vline(
    data = pop_labels_bars,
    aes(xintercept = pos),
    color = "#2C444A",
    linewidth = .2
  ) +
  geom_text(
    data = pop_labels,
    aes(x = as.numeric(ind), y = 1, label = Name),
    vjust = 1.5,
    hjust = 0,
    size = 2,
    angle = 90,
    inherit.aes = FALSE
  ) +
  my_theme() +
  theme(
    axis.text.x = element_text(
      angle = 90,
      hjust = 1,
      size = 12
    ),
    legend.position = "none",
    plot.margin = unit(c(3, 0.5, 0.5, 0.5), "cm")
  ) +
  xlab("Admixture matrix") +
  ylab("Ancestry proportions") +
  labs(caption = "Each bar represents the ancestry proportions for an individual for k=4.\n Neuro-admixture training k4 with 20,931 SNPs.") +
  scale_x_discrete(labels = label_func) +
  scale_fill_manual(values = color_palette) +
  expand_limits(y = c(0, 1.5))
```

```
# # # save it
ggsave(
  here("output", "populations", "figures", "neuro_admixture_k=4_r_0.01_trained.pdf"),
  width  = 12,
  height = 6,
  units  = "in",
  device = cairo_pdf
)
```

### 1.2 Inference with all populations

Now we can use all populations but extract the SNPs we trained

```
plink \
--keep-allele-order \
--bfile output/populations/file7 \
--make-bed \
--export vcf \
--out output/populations/snps_sets/r2_0.01 \
--extract output/populations/nadmix/train/train_r_0.01.snplist \
--silent
grep "samples\|variants" output/populations/snps_sets/r2_0.01.log
```

```
## 82731 variants loaded from .bim file.
## --extract: 20931 variants remaining.
## 20931 variants and 237 people pass filters and QC.
```

Transfer data to cluster

```
rsync -chavzP --stats /Users/lucianocosme/Library/CloudStorage/Dropbox/Albopictus/manuscript_chip/data/no_autogenous/albo_chip/output/populations/nadmix lvc26@mccleary.ycrc.yale.edu:/gpfs/ycga/project/caccone/lvc26/
```

Inference mode (projective analysis)

```
neural-admixture infer --name r_0.01 --save_dir /gpfs/ycga/project/caccone/lvc26/neuroadmix/r_0.01 --out_name r_0.01_inference --data_path /gpfs/ycga/project/caccone/lvc26/neuroadmix/snps_sets/r2_0.01.bed | tee r_0.01_inference.log
```

### 1.3 Plot after inference with all populations

Download the data

```
rsync -chavzP --stats lvc26@mccleary.ycrc.yale.edu:/gpfs/ycga/project/caccone/lvc26/neuroadmix/r_0.01 /Users/lucianocosme/Library/CloudStorage/Dropbox/Albopictus/manuscript_chip/data/no_autogenous/albo_chip/output/populations/nadmix/results
```

### 1.4 Plot inference

```
# Extract ancestry coefficients
nadmixk5 <- read_delim(
  here("output", "populations", "nadmix", "results", "r_0.01","r_0.01_inference.5.Q"),
  delim = " ", # Specify the delimiter if different from the default (comma)
  col_names = FALSE,
  show_col_types = FALSE
) 
# unseen_pckmeans.7.Q
# pckmeans.7.Q
head(nadmixk5)
```

```
## # A tibble: 6 × 5
##       X1    X2    X3     X4     X5
##    <dbl> <dbl> <dbl>  <dbl>  <dbl>
## 1 0.0203 0.701 0.152 0.109  0.0173
## 2 0.0191 0.682 0.184 0.102  0.0130
## 3 0.0163 0.706 0.151 0.0845 0.0416
## 4 0.0341 0.671 0.167 0.109  0.0193
## 5 0.0389 0.692 0.168 0.0892 0.0128
## 6 0.0162 0.714 0.171 0.0784 0.0202
```

The fam file

```
fam_file <- here(
  "output", "populations", "snps_sets", "r2_0.01.fam"
)

# Read the .fam file
fam_data <- read.table(fam_file, 
                       header = FALSE,
                       col.names = c("FamilyID", "IndividualID", "PaternalID", "MaternalID", "Sex", "Phenotype"))

# View the first few rows
head(fam_data)
```

```
##   FamilyID IndividualID PaternalID MaternalID Sex Phenotype
## 1      OKI         1001          0          0   2        -9
## 2      OKI         1002          0          0   2        -9
## 3      OKI         1003          0          0   2        -9
## 4      OKI         1004          0          0   2        -9
## 5      OKI         1005          0          0   2        -9
## 6      OKI         1006          0          0   1        -9
```

Create ID column

```
# Change column name
colnames(fam_data)[colnames(fam_data) == "IndividualID"] <- "ind"


# Merge columns "FamilyID" and "IndividualID" with an underscore
# fam_data$ind <- paste(fam_data$FamilyID, fam_data$IndividualID, sep = "_")


# Change column name
colnames(fam_data)[colnames(fam_data) == "FamilyID"] <- "pop"

# Select ID
fam_data <- fam_data |>
  dplyr::select("ind", "pop")

# View the first few rows
head(fam_data)
```

```
##    ind pop
## 1 1001 OKI
## 2 1002 OKI
## 3 1003 OKI
## 4 1004 OKI
## 5 1005 OKI
## 6 1006 OKI
```

Add it to matrix

```
nadmixk5 <- fam_data |>
  dplyr::select(ind, pop) |>
  bind_cols(nadmixk5)

head(nadmixk5)
```

```
##    ind pop         X1        X2        X3         X4         X5
## 1 1001 OKI 0.02026024 0.7009754 0.1522250 0.10923769 0.01730163
## 2 1002 OKI 0.01906374 0.6816174 0.1840338 0.10228224 0.01300276
## 3 1003 OKI 0.01627573 0.7062859 0.1513138 0.08453301 0.04159156
## 4 1004 OKI 0.03413134 0.6708513 0.1668813 0.10887521 0.01926081
## 5 1005 OKI 0.03889918 0.6916250 0.1675111 0.08921071 0.01275407
## 6 1006 OKI 0.01619347 0.7141496 0.1710819 0.07837918 0.02019598
```

Rename the columns

```
# Rename the columns starting from the third one
nadmixk5 <- nadmixk5 |>
  rename_with(~paste0("v", seq_along(.x)), .cols = -c(ind, pop))

# View the first few rows
head(nadmixk5)
```

```
##    ind pop         v1        v2        v3         v4         v5
## 1 1001 OKI 0.02026024 0.7009754 0.1522250 0.10923769 0.01730163
## 2 1002 OKI 0.01906374 0.6816174 0.1840338 0.10228224 0.01300276
## 3 1003 OKI 0.01627573 0.7062859 0.1513138 0.08453301 0.04159156
## 4 1004 OKI 0.03413134 0.6708513 0.1668813 0.10887521 0.01926081
## 5 1005 OKI 0.03889918 0.6916250 0.1675111 0.08921071 0.01275407
## 6 1006 OKI 0.01619347 0.7141496 0.1710819 0.07837918 0.02019598
```

Import sample locations

```
sampling_loc <- readRDS(here("output", "local_adaptation", "sampling_loc.rds"))
head(sampling_loc)
```

```
## # A tibble: 6 × 6
##   Pop_City   Country  Latitude Longitude Region Abbreviation
##   <chr>      <chr>       <dbl>     <dbl> <chr>  <chr>       
## 1 Gelephu    Bhutan       26.9      90.5 Asia   GEL         
## 2 Phnom Penh Cambodia     11.6     105.  Asia   CAM         
## 3 Hainan     China        19.2     110.  Asia   HAI         
## 4 Yunnan     China        24.5     101.  Asia   YUN         
## 5 Hunan      China        27.6     112.  Asia   HUN         
## 6 Bengaluru  India        13.0      77.6 Asia   BEN
```

```
source(
  here(
    "scripts", "analysis", "my_theme3.R"
  )
)

# Create a named vector to map countries to regions
country_to_region <- c(
  "Bhutan" = "South Asia",
  "Cambodia" = "Southeast Asia",
  "China" = "East Asia",
  "India" = "South Asia",
  "Indonesia" = "Southeast Asia",
  "Japan" = "East Asia",
  "Malaysia" = "Southeast Asia",
  "Maldives" = "South Asia",
  "Nepal" = "South Asia",
  "Sri Lanka" = "South Asia",
  "Taiwan" = "East Asia",
  "Thailand" = "Southeast Asia",
  "Vietnam" = "Southeast Asia"
)

# Add the region to the data frame
sampling_loc$Region2 <- country_to_region[sampling_loc$Country]

# Melt the data frame for plotting
Q_melted <- nadmixk5 |>
  pivot_longer(
    cols = -c(ind, pop),
    names_to = "variable",
    values_to = "value"
  )
# Join with sampling_loc to get sampling localities
Q_joined <- Q_melted |>
  left_join(sampling_loc, by = c("pop" = "Abbreviation"))

# Create a combined variable for Region and Country
Q_joined <- Q_joined |>
  mutate(Region_Country = interaction(Region, Country, sep = "_"))

# Order the combined variable by Region and Country, then by individual
Q_ordered <- Q_joined |>
  arrange(Region, Region2, Country, ind) |>
  mutate(ind = factor(ind, levels = unique(ind)))  # Convert ind to a factor with levels in the desired order

# Add labels: country names for the first individual in each country, NA for all other individuals
Q_ordered <- Q_ordered |>
  group_by(Region_Country) |>
  mutate(label = ifelse(row_number() == 1, as.character(Country), NA))

# Group by individual and variable, calculate mean ancestry proportions
Q_grouped <- Q_ordered |>
  group_by(ind, variable) |>
  summarise(value = mean(value), .groups = "drop")

# Create a data frame for borders
borders <-
  data.frame(Region_Country = unique(Q_ordered$Region_Country))

# Add the order of the last individual of each country to ensure correct placement of borders
borders$order <-
  sapply(borders$Region_Country, function(rc)
    max(which(Q_ordered$Region_Country == rc))) + 0.5  # Shift borders to the right edge of the bars

# Select only the first occurrence of each country in the ordered data
label_df <- Q_ordered |>
  filter(!is.na(label)) |>
  distinct(label, .keep_all = TRUE)

# Create a custom label function
label_func <- function(x) {
  labels <- rep("", length(x))
  labels[x %in% label_df$ind] <- label_df$label
  labels
}

# Calculate the position of lines
border_positions <- Q_ordered |>
  group_by(Country) |>
  summarise(pos = max(as.numeric(ind)) + 0)

# Calculate the position of population labels and bars
pop_labels <- Q_ordered |>
  mutate(Name = paste(pop, Pop_City, sep = " - ")) |>
  group_by(pop) |>
  slice_head(n = 1) |>
  ungroup() |>
  dplyr::select(ind, Pop_City, Country, Name) |>
  mutate(pos = as.numeric(ind))  # calculate position of population labels

pop_labels_bars <- pop_labels |>
  mutate(pos = as.numeric(ind)  - .5)


# Calculate the position of lines
border_positions <- Q_ordered |>
  group_by(Country) |>
  summarise(pos = max(as.numeric(ind)) - 1)


pop_labels_bars <- pop_labels |>
  mutate(pos = as.numeric(ind)  - .5)

# Function to filter and normalize data
normalize_data <- function(df, min_value) {
  df |>
    filter(value > min_value) |>
    group_by(ind) |>
    mutate(value = value / sum(value))
}

# Use the function
Q_grouped_filtered <- normalize_data(Q_grouped, 0.1)

color_palette <-
  c(
    "V1" = "red",
    "V2" = "#AE9393",
    "V3" = "#F49AC2",
    "V4" = "#FFB347",
    "V5" = "#FFFF99"
  )

# Generate all potential variable names
all_variables <- paste0("v", 1:5)

# Map each variable to a name
color_mapping <- data.frame(variable = all_variables,
                            color = names(color_palette))

# Merge with Q_grouped_filtered
Q_grouped_filtered <- merge(Q_grouped_filtered, color_mapping, by = "variable")

# Create the plot
ggplot(Q_grouped_filtered, aes(x = as.factor(ind), y = value, fill = color)) +
  geom_bar(stat = 'identity', width = 1) +
  geom_vline(
    data = pop_labels_bars,
    aes(xintercept = pos),
    color = "#2C444A",
    linewidth = .2
  ) +
  geom_text(
    data = pop_labels,
    aes(x = as.numeric(ind), y = 1, label = Name),
    vjust = 1.5,
    hjust = 0,
    size = 2,
    angle = 90,
    inherit.aes = FALSE
  ) +
  my_theme() +
  theme(
    axis.text.x = element_text(
      angle = 90,
      hjust = 1,
      size = 12
    ),
    legend.position = "none",
    plot.margin = unit(c(3, 0.5, 0.5, 0.5), "cm")
  ) +
  xlab("Admixture matrix") +
  ylab("Ancestry proportions") +
  labs(caption = "Each bar represents the ancestry proportions for an individual for k=5.\n Neuro-admixture inference for k5 with 20,931 SNPs.") +
  scale_x_discrete(labels = label_func) +
  scale_fill_manual(values = color_palette) +
  expand_limits(y = c(0, 1.5))
```

```
ggsave(
  here("output", "populations", "figures", "neuro_admixture_k=5_r_0.01_inference.pdf"),
  width  = 12,
  height = 6,
  units  = "in",
  device = cairo_pdf
)
```

## 2. Train neuro admixture with SNPS r2 0.1

We can choose a few populations representing each genetic cluster
from k=5 from our admixture analysis

```
color_palette <-
  c(
    "V1" = "#AE9393", # KAG
    "V2" = "#F49AC2", # MAT, YUN, BEN
    "V3" = "red", # INJ, INW
    "V4" = "#FFB347", # KAN
    "V5" = "#FFFF99"# TAI

  )
```

Create a new bed file for training. Then we use the 60 SNPs with all
populations to test neuro-admixture

```
echo "KAG
MAT
YUN
BEN
INJ
INW
KAN
TAI
" > output/populations/nadmix/pops_4_training.txt
```

Subset with plink

```
plink \
--keep-allele-order \
--keep-fam output/populations/nadmix/pops_4_training.txt \
--bfile output/populations/file7 \
--maf 0.1 \
--make-bed \
--out output/populations/nadmix/train/train_r_0.1 \
--extract output/populations/indepSNP_chr.prune.in \
--geno 0.2 \
--silent \
--write-snplist
# 57k SNPs
grep "variants\|samples" output/populations/nadmix/train/train_r_0.1.log
```

```
## 82731 variants loaded from .bim file.
## --extract: 60988 variants remaining.
## Total genotyping rate in remaining samples is 0.972429.
## 5 variants removed due to missing genotype data (--geno).
## 3203 variants removed due to minor allele threshold(s)
## 57780 variants and 79 people pass filters and QC.
```

Train with the pckmeans initialization

Get two gpu/cpus

```
salloc --cpus-per-gpu=2 --gpus=2 --time=30:00 --partition gpu_devel
```

Train

```
cd /gpfs/ycga/project/caccone/lvc26/neuroadmix;
module load miniconda/23.1.0;
conda activate nadmenv;

# pckmeans r_0.1
neural-admixture train --seed 1234 --initialization pckmeans --warmup_epochs 1000 --max_epochs 1000 --activation relu --optimizer adam --learning_rate 1e-7 --min_k 2 --max_k 10 --name r_0.1 --data_path /gpfs/ycga/project/caccone/lvc26/neuroadmix/train/train_r_0.1.bed --save_dir /gpfs/ycga/project/caccone/lvc26/neuroadmix/r_0.1 | tee r_0.1.log
```

Download the data

```
rsync -chavzP --stats lvc26@mccleary.ycrc.yale.edu:/gpfs/ycga/project/caccone/lvc26/neuroadmix/r_0.1 /Users/lucianocosme/Library/CloudStorage/Dropbox/Albopictus/manuscript_chip/data/no_autogenous/albo_chip/output/populations/nadmix/results
```

### 2.1. Plot after training

```
# Extract ancestry coefficients
nadmixk4 <- read_delim(
  here("output", "populations", "nadmix", "results", "r_0.1","r_0.1.4.Q"),
  delim = " ", # Specify the delimiter if different from the default (comma)
  col_names = FALSE,
  show_col_types = FALSE
) 


head(nadmixk4)
```

```
## # A tibble: 6 × 4
##      X1          X2        X3       X4
##   <dbl>       <dbl>     <dbl>    <dbl>
## 1 1.00  0.00000466  0.0000627 8.43e-12
## 2 0.999 0.000000451 0.00103   6.41e-13
## 3 0.999 0.000000108 0.00124   2.52e-11
## 4 0.999 0.000000578 0.00112   6.43e-13
## 5 1.00  0.000000487 0.0000991 2.62e-12
## 6 1.00  0.00000499  0.000410  5.08e-12
```

The fam file

```
fam_file <- here(
  "output", "populations", "nadmix", "train","train_r_0.1.fam"
)

# Read the .fam file
fam_data <- read.table(fam_file, 
                       header = FALSE,
                       col.names = c("FamilyID", "IndividualID", "PaternalID", "MaternalID", "Sex", "Phenotype"))

# View the first few rows
head(fam_data)
```

```
##   FamilyID IndividualID PaternalID MaternalID Sex Phenotype
## 1      YUN         1089          0          0   1        -9
## 2      YUN         1090          0          0   1        -9
## 3      YUN         1091          0          0   1        -9
## 4      YUN         1092          0          0   1        -9
## 5      YUN         1093          0          0   1        -9
## 6      YUN         1094          0          0   1        -9
```

Create ID column

```
# Change column name
colnames(fam_data)[colnames(fam_data) == "IndividualID"] <- "ind"


# Change column name
colnames(fam_data)[colnames(fam_data) == "FamilyID"] <- "pop"

# Select ID
fam_data <- fam_data |>
  dplyr::select("ind", "pop")

# View the first few rows
head(fam_data)
```

```
##    ind pop
## 1 1089 YUN
## 2 1090 YUN
## 3 1091 YUN
## 4 1092 YUN
## 5 1093 YUN
## 6 1094 YUN
```

Add it to matrix

```
nadmixk4 <- fam_data |>
  dplyr::select(ind, pop) |>
  bind_cols(nadmixk4)

head(nadmixk4)
```

```
##    ind pop        X1           X2           X3           X4
## 1 1089 YUN 0.9999326 4.662390e-06 6.269065e-05 8.431109e-12
## 2 1090 YUN 0.9989667 4.512044e-07 1.032777e-03 6.409134e-13
## 3 1091 YUN 0.9987565 1.082062e-07 1.243371e-03 2.517938e-11
## 4 1092 YUN 0.9988790 5.775292e-07 1.120423e-03 6.426688e-13
## 5 1093 YUN 0.9999003 4.874677e-07 9.912007e-05 2.622454e-12
## 6 1094 YUN 0.9995853 4.991595e-06 4.097095e-04 5.084839e-12
```

Rename the columns

```
# Rename the columns starting from the third one
nadmixk4 <- nadmixk4 |>
  rename_with(~paste0("v", seq_along(.x)), .cols = -c(ind, pop))

# View the first few rows
head(nadmixk4)
```

```
##    ind pop        v1           v2           v3           v4
## 1 1089 YUN 0.9999326 4.662390e-06 6.269065e-05 8.431109e-12
## 2 1090 YUN 0.9989667 4.512044e-07 1.032777e-03 6.409134e-13
## 3 1091 YUN 0.9987565 1.082062e-07 1.243371e-03 2.517938e-11
## 4 1092 YUN 0.9988790 5.775292e-07 1.120423e-03 6.426688e-13
## 5 1093 YUN 0.9999003 4.874677e-07 9.912007e-05 2.622454e-12
## 6 1094 YUN 0.9995853 4.991595e-06 4.097095e-04 5.084839e-12
```

Import sample locations

```
sampling_loc <- readRDS(here("output", "local_adaptation", "sampling_loc.rds"))
head(sampling_loc)
```

```
## # A tibble: 6 × 6
##   Pop_City   Country  Latitude Longitude Region Abbreviation
##   <chr>      <chr>       <dbl>     <dbl> <chr>  <chr>       
## 1 Gelephu    Bhutan       26.9      90.5 Asia   GEL         
## 2 Phnom Penh Cambodia     11.6     105.  Asia   CAM         
## 3 Hainan     China        19.2     110.  Asia   HAI         
## 4 Yunnan     China        24.5     101.  Asia   YUN         
## 5 Hunan      China        27.6     112.  Asia   HUN         
## 6 Bengaluru  India        13.0      77.6 Asia   BEN
```

```
source(
  here(
    "scripts", "analysis", "my_theme3.R"
  )
)

# Create a named vector to map countries to regions
country_to_region <- c(
  "Bhutan" = "South Asia",
  "Cambodia" = "Southeast Asia",
  "China" = "East Asia",
  "India" = "South Asia",
  "Indonesia" = "Southeast Asia",
  "Japan" = "East Asia",
  "Malaysia" = "Southeast Asia",
  "Maldives" = "South Asia",
  "Nepal" = "South Asia",
  "Sri Lanka" = "South Asia",
  "Taiwan" = "East Asia",
  "Thailand" = "Southeast Asia",
  "Vietnam" = "Southeast Asia"
)

# Add the region to the data frame
sampling_loc$Region2 <- country_to_region[sampling_loc$Country]

# Melt the data frame for plotting
# Q_melted <- melt(nadmixk5, id.vars = c("ind", "pop"))
# Melt the data frame for plotting
Q_melted <- nadmixk4 |>
  pivot_longer(
    cols = -c(ind, pop),
    names_to = "variable",
    values_to = "value"
  )
# Join with sampling_loc to get sampling localities
Q_joined <- Q_melted |>
  left_join(sampling_loc, by = c("pop" = "Abbreviation"))

# Create a combined variable for Region and Country
Q_joined <- Q_joined |>
  mutate(Region_Country = interaction(Region, Country, sep = "_"))

# Order the combined variable by Region and Country, then by individual
Q_ordered <- Q_joined |>
  arrange(Region, Region2, Country, ind) |>
  mutate(ind = factor(ind, levels = unique(ind)))  # Convert ind to a factor with levels in the desired order

# Add labels: country names for the first individual in each country, NA for all other individuals
Q_ordered <- Q_ordered |>
  group_by(Region_Country) |>
  mutate(label = ifelse(row_number() == 1, as.character(Country), NA))

# Group by individual and variable, calculate mean ancestry proportions
Q_grouped <- Q_ordered |>
  group_by(ind, variable) |>
  summarise(value = mean(value), .groups = "drop")

# Create a data frame for borders
borders <-
  data.frame(Region_Country = unique(Q_ordered$Region_Country))

# Add the order of the last individual of each country to ensure correct placement of borders
borders$order <-
  sapply(borders$Region_Country, function(rc)
    max(which(Q_ordered$Region_Country == rc))) + 0.5  # Shift borders to the right edge of the bars

# Select only the first occurrence of each country in the ordered data
label_df <- Q_ordered |>
  filter(!is.na(label)) |>
  distinct(label, .keep_all = TRUE)

# Create a custom label function
label_func <- function(x) {
  labels <- rep("", length(x))
  labels[x %in% label_df$ind] <- label_df$label
  labels
}

# Calculate the position of lines
border_positions <- Q_ordered |>
  group_by(Country) |>
  summarise(pos = max(as.numeric(ind)) + 0)

# Calculate the position of population labels and bars
pop_labels <- Q_ordered |>
  mutate(Name = paste(pop, Pop_City, sep = " - ")) |>
  group_by(pop) |>
  slice_head(n = 1) |>
  ungroup() |>
  dplyr::select(ind, Pop_City, Country, Name) |>
  mutate(pos = as.numeric(ind))  # calculate position of population labels

pop_labels_bars <- pop_labels |>
  mutate(pos = as.numeric(ind)  - .5)


# Calculate the position of lines
border_positions <- Q_ordered |>
  group_by(Country) |>
  summarise(pos = max(as.numeric(ind)) - 1)


pop_labels_bars <- pop_labels |>
  mutate(pos = as.numeric(ind)  - .5)

# Function to filter and normalize data
normalize_data <- function(df, min_value) {
  df |>
    filter(value > min_value) |>
    group_by(ind) |>
    mutate(value = value / sum(value))
}

# Use the function
Q_grouped_filtered <- normalize_data(Q_grouped, 0.1)

color_palette <-
  c(
    "V1" = "#FFB347",
    "V2" = "#F49AC2",
    "V3" = "red",
    "V4" = "#AE9393"
  )

# Generate all potential variable names
all_variables <- paste0("v", 1:4)

# Map each variable to a name
color_mapping <- data.frame(variable = all_variables,
                            color = names(color_palette))

# Merge with Q_grouped_filtered
Q_grouped_filtered <- merge(Q_grouped_filtered, color_mapping, by = "variable")

# Create the plot
ggplot(Q_grouped_filtered, aes(x = as.factor(ind), y = value, fill = color)) +
  geom_bar(stat = 'identity', width = 1) +
  geom_vline(
    data = pop_labels_bars,
    aes(xintercept = pos),
    color = "#2C444A",
    linewidth = .2
  ) +
  geom_text(
    data = pop_labels,
    aes(x = as.numeric(ind), y = 1, label = Name),
    vjust = 1.5,
    hjust = 0,
    size = 2,
    angle = 90,
    inherit.aes = FALSE
  ) +
  my_theme() +
  theme(
    axis.text.x = element_text(
      angle = 90,
      hjust = 1,
      size = 12
    ),
    legend.position = "none",
    plot.margin = unit(c(3, 0.5, 0.5, 0.5), "cm")
  ) +
  xlab("Admixture matrix") +
  ylab("Ancestry proportions") +
  labs(caption = "Each bar represents the ancestry proportions for an individual for k=4.\n Neuro-admixture training k4 with 20,931 SNPs.") +
  scale_x_discrete(labels = label_func) +
  scale_fill_manual(values = color_palette) +
  expand_limits(y = c(0, 1.5))
```

```
# # # save it
ggsave(
  here("output", "populations", "figures", "neuro_admixture_k=4_r_0.1_trained.pdf"),
  width  = 12,
  height = 6,
  units  = "in",
  device = cairo_pdf
)
```

### 2.2 Inference with all populations

Now we can use all populations but extract the SNPs we trained

```
plink \
--keep-allele-order \
--bfile output/populations/file7 \
--make-bed \
--export vcf \
--out output/populations/snps_sets/r2_0.1 \
--extract output/populations/nadmix/train/train_r_0.1.snplist \
--silent
grep "samples\|variants" output/populations/snps_sets/r2_0.1.log
```

```
## 82731 variants loaded from .bim file.
## --extract: 57780 variants remaining.
## 57780 variants and 237 people pass filters and QC.
```

Transfer data to cluster

```
rsync -chavzP --stats /Users/lucianocosme/Library/CloudStorage/Dropbox/Albopictus/manuscript_chip/data/no_autogenous/albo_chip/output/populations/nadmix lvc26@mccleary.ycrc.yale.edu:/gpfs/ycga/project/caccone/lvc26/
```

Inference mode (projective analysis)

```
neural-admixture infer --name r_0.1 --save_dir /gpfs/ycga/project/caccone/lvc26/neuroadmix/r_0.1 --out_name r_0.1_inference --data_path /gpfs/ycga/project/caccone/lvc26/neuroadmix/snps_sets/r2_0.1.bed | tee r_0.1_inference.log
```

### 2.3 Plot after inference with all populations

Download the data

```
rsync -chavzP --stats lvc26@mccleary.ycrc.yale.edu:/gpfs/ycga/project/caccone/lvc26/neuroadmix/r_0.1 /Users/lucianocosme/Library/CloudStorage/Dropbox/Albopictus/manuscript_chip/data/no_autogenous/albo_chip/output/populations/nadmix/results
```

### 2.4 Plot inference

```
# Extract ancestry coefficients
nadmixk5 <- read_delim(
  here("output", "populations", "nadmix", "results", "r_0.1","r_0.1_inference.5.Q"),
  delim = " ", # Specify the delimiter if different from the default (comma)
  col_names = FALSE,
  show_col_types = FALSE
) 
# unseen_pckmeans.7.Q
# pckmeans.7.Q
head(nadmixk5)
```

```
## # A tibble: 6 × 5
##         X1    X2     X3     X4      X5
##      <dbl> <dbl>  <dbl>  <dbl>   <dbl>
## 1 0.00224  0.827 0.0444 0.113  0.0130 
## 2 0.000621 0.937 0.0307 0.0280 0.00339
## 3 0.00297  0.844 0.0415 0.103  0.00833
## 4 0.00247  0.784 0.0591 0.146  0.00809
## 5 0.00279  0.757 0.0703 0.159  0.0113 
## 6 0.00192  0.845 0.0404 0.101  0.0120
```

The fam file

```
fam_file <- here(
  "output", "populations", "snps_sets", "r2_0.1.fam"
)

# Read the .fam file
fam_data <- read.table(fam_file, 
                       header = FALSE,
                       col.names = c("FamilyID", "IndividualID", "PaternalID", "MaternalID", "Sex", "Phenotype"))

# View the first few rows
head(fam_data)
```

```
##   FamilyID IndividualID PaternalID MaternalID Sex Phenotype
## 1      OKI         1001          0          0   2        -9
## 2      OKI         1002          0          0   2        -9
## 3      OKI         1003          0          0   2        -9
## 4      OKI         1004          0          0   2        -9
## 5      OKI         1005          0          0   2        -9
## 6      OKI         1006          0          0   1        -9
```

Create ID column

```
# Change column name
colnames(fam_data)[colnames(fam_data) == "IndividualID"] <- "ind"


# Merge columns "FamilyID" and "IndividualID" with an underscore
# fam_data$ind <- paste(fam_data$FamilyID, fam_data$IndividualID, sep = "_")


# Change column name
colnames(fam_data)[colnames(fam_data) == "FamilyID"] <- "pop"

# Select ID
fam_data <- fam_data |>
  dplyr::select("ind", "pop")

# View the first few rows
head(fam_data)
```

```
##    ind pop
## 1 1001 OKI
## 2 1002 OKI
## 3 1003 OKI
## 4 1004 OKI
## 5 1005 OKI
## 6 1006 OKI
```

Add it to matrix

```
nadmixk5 <- fam_data |>
  dplyr::select(ind, pop) |>
  bind_cols(nadmixk5)

head(nadmixk5)
```

```
##    ind pop           X1        X2         X3         X4          X5
## 1 1001 OKI 0.0022390699 0.8273290 0.04438876 0.11306997 0.012973181
## 2 1002 OKI 0.0006207919 0.9372209 0.03072406 0.02804254 0.003391742
## 3 1003 OKI 0.0029661185 0.8442711 0.04149344 0.10293802 0.008331385
## 4 1004 OKI 0.0024678661 0.7841588 0.05913275 0.14614794 0.008092790
## 5 1005 OKI 0.0027905770 0.7570373 0.07026709 0.15857665 0.011328328
## 6 1006 OKI 0.0019242963 0.8448561 0.04041177 0.10083950 0.011968327
```

Rename the columns

```
# Rename the columns starting from the third one
nadmixk5 <- nadmixk5 |>
  rename_with(~paste0("v", seq_along(.x)), .cols = -c(ind, pop))

# View the first few rows
head(nadmixk5)
```

```
##    ind pop           v1        v2         v3         v4          v5
## 1 1001 OKI 0.0022390699 0.8273290 0.04438876 0.11306997 0.012973181
## 2 1002 OKI 0.0006207919 0.9372209 0.03072406 0.02804254 0.003391742
## 3 1003 OKI 0.0029661185 0.8442711 0.04149344 0.10293802 0.008331385
## 4 1004 OKI 0.0024678661 0.7841588 0.05913275 0.14614794 0.008092790
## 5 1005 OKI 0.0027905770 0.7570373 0.07026709 0.15857665 0.011328328
## 6 1006 OKI 0.0019242963 0.8448561 0.04041177 0.10083950 0.011968327
```

Import sample locations

```
sampling_loc <- readRDS(here("output", "local_adaptation", "sampling_loc.rds"))
head(sampling_loc)
```

```
## # A tibble: 6 × 6
##   Pop_City   Country  Latitude Longitude Region Abbreviation
##   <chr>      <chr>       <dbl>     <dbl> <chr>  <chr>       
## 1 Gelephu    Bhutan       26.9      90.5 Asia   GEL         
## 2 Phnom Penh Cambodia     11.6     105.  Asia   CAM         
## 3 Hainan     China        19.2     110.  Asia   HAI         
## 4 Yunnan     China        24.5     101.  Asia   YUN         
## 5 Hunan      China        27.6     112.  Asia   HUN         
## 6 Bengaluru  India        13.0      77.6 Asia   BEN
```

```
source(
  here(
    "scripts", "analysis", "my_theme3.R"
  )
)

# Create a named vector to map countries to regions
country_to_region <- c(
  "Bhutan" = "South Asia",
  "Cambodia" = "Southeast Asia",
  "China" = "East Asia",
  "India" = "South Asia",
  "Indonesia" = "Southeast Asia",
  "Japan" = "East Asia",
  "Malaysia" = "Southeast Asia",
  "Maldives" = "South Asia",
  "Nepal" = "South Asia",
  "Sri Lanka" = "South Asia",
  "Taiwan" = "East Asia",
  "Thailand" = "Southeast Asia",
  "Vietnam" = "Southeast Asia"
)

# Add the region to the data frame
sampling_loc$Region2 <- country_to_region[sampling_loc$Country]

# Melt the data frame for plotting
# Q_melted <- melt(nadmixk5, id.vars = c("ind", "pop"))
# Melt the data frame for plotting
Q_melted <- nadmixk5 |>
  pivot_longer(
    cols = -c(ind, pop),
    names_to = "variable",
    values_to = "value"
  )
# Join with sampling_loc to get sampling localities
Q_joined <- Q_melted |>
  left_join(sampling_loc, by = c("pop" = "Abbreviation"))

# Create a combined variable for Region and Country
Q_joined <- Q_joined |>
  mutate(Region_Country = interaction(Region, Country, sep = "_"))

# Order the combined variable by Region and Country, then by individual
Q_ordered <- Q_joined |>
  arrange(Region, Region2, Country, ind) |>
  mutate(ind = factor(ind, levels = unique(ind)))  # Convert ind to a factor with levels in the desired order

# Add labels: country names for the first individual in each country, NA for all other individuals
Q_ordered <- Q_ordered |>
  group_by(Region_Country) |>
  mutate(label = ifelse(row_number() == 1, as.character(Country), NA))

# Group by individual and variable, calculate mean ancestry proportions
Q_grouped <- Q_ordered |>
  group_by(ind, variable) |>
  summarise(value = mean(value), .groups = "drop")

# Create a data frame for borders
borders <-
  data.frame(Region_Country = unique(Q_ordered$Region_Country))

# Add the order of the last individual of each country to ensure correct placement of borders
borders$order <-
  sapply(borders$Region_Country, function(rc)
    max(which(Q_ordered$Region_Country == rc))) + 0.5  # Shift borders to the right edge of the bars

# Select only the first occurrence of each country in the ordered data
label_df <- Q_ordered |>
  filter(!is.na(label)) |>
  distinct(label, .keep_all = TRUE)

# Create a custom label function
label_func <- function(x) {
  labels <- rep("", length(x))
  labels[x %in% label_df$ind] <- label_df$label
  labels
}

# Calculate the position of lines
border_positions <- Q_ordered |>
  group_by(Country) |>
  summarise(pos = max(as.numeric(ind)) + 0)

# Calculate the position of population labels and bars
pop_labels <- Q_ordered |>
  mutate(Name = paste(pop, Pop_City, sep = " - ")) |>
  group_by(pop) |>
  slice_head(n = 1) |>
  ungroup() |>
  dplyr::select(ind, Pop_City, Country, Name) |>
  mutate(pos = as.numeric(ind))  # calculate position of population labels

pop_labels_bars <- pop_labels |>
  mutate(pos = as.numeric(ind)  - .5)


# Calculate the position of lines
border_positions <- Q_ordered |>
  group_by(Country) |>
  summarise(pos = max(as.numeric(ind)) - 1)


pop_labels_bars <- pop_labels |>
  mutate(pos = as.numeric(ind)  - .5)

# Function to filter and normalize data
normalize_data <- function(df, min_value) {
  df |>
    filter(value > min_value) |>
    group_by(ind) |>
    mutate(value = value / sum(value))
}

# Use the function
Q_grouped_filtered <- normalize_data(Q_grouped, 0.1)

color_palette <-
  c(
    "V1" = "red",
    "V2" = "#AE9393",
    "V3" = "#FFFF99",
    "V4" = "#FFB347",
    "V5" = "#F49AC2"
  )

# Generate all potential variable names
all_variables <- paste0("v", 1:5)

# Map each variable to a name
color_mapping <- data.frame(variable = all_variables,
                            color = names(color_palette))

# Merge with Q_grouped_filtered
Q_grouped_filtered <- merge(Q_grouped_filtered, color_mapping, by = "variable")

# Create the plot
ggplot(Q_grouped_filtered, aes(x = as.factor(ind), y = value, fill = color)) +
  geom_bar(stat = 'identity', width = 1) +
  geom_vline(
    data = pop_labels_bars,
    aes(xintercept = pos),
    color = "#2C444A",
    linewidth = .2
  ) +
  geom_text(
    data = pop_labels,
    aes(x = as.numeric(ind), y = 1, label = Name),
    vjust = 1.5,
    hjust = 0,
    size = 2,
    angle = 90,
    inherit.aes = FALSE
  ) +
  my_theme() +
  theme(
    axis.text.x = element_text(
      angle = 90,
      hjust = 1,
      size = 12
    ),
    legend.position = "none",
    plot.margin = unit(c(3, 0.5, 0.5, 0.5), "cm")
  ) +
  xlab("Admixture matrix") +
  ylab("Ancestry proportions") +
  labs(caption = "Each bar represents the ancestry proportions for an individual for k=5.\n Neuro-admixture inference for k5 with 20,931 SNPs.") +
  scale_x_discrete(labels = label_func) +
  scale_fill_manual(values = color_palette) +
  expand_limits(y = c(0, 1.5))
```

```
ggsave(
  here("output", "populations", "figures", "neuro_admixture_k=5_r_0.1_inference.pdf"),
  width  = 12,
  height = 6,
  units  = "in",
  device = cairo_pdf
)
```

## 3. Train with neutral SNPs

```
plink2 \
--allow-extra-chr \
--bfile output/populations/neutral \
--make-bed \
--maf 0.1 \
--geno 0.2 \
--extract output/populations/neutral_SNPs.txt \
--out output/populations/nadmix/train/neutral_train \
--keep-fam output/populations/nadmix/pops_4_training.txt \
--write-snplist \
--silent;
grep "samples\|variants" output/populations/nadmix/train/neutral_train.log
```

```
## 237 samples (30 females, 67 males, 140 ambiguous; 237 founders) loaded from
## 9483 variants loaded from output/populations/neutral.bim.
## --extract: 9483 variants remaining.
## --keep-fam: 79 samples remaining.
## 79 samples (14 females, 14 males, 51 ambiguous; 79 founders) remaining after
## 435 variants removed due to allele frequency threshold(s)
## 9047 variants remaining after main filters.
```

Now use the snp list to create the same data set with all the
samples. Neuroadmixture expects the same SNPs for training and
inference. Because we subseted the data with filter –maf 0.1, we ended
up with less SNPs. Then, we can create the full data set with the SNPs
that we have after sub-setting.

```
plink2 \
--allow-extra-chr \
--bfile output/populations/neutral \
--export vcf \
--make-bed \
--maf 0.1 \
--geno 0.2 \
--out output/populations/snps_sets/neutral \
--extract output/populations/nadmix/train/neutral_train.snplist \
--silent;
grep "samples\|variants" output/populations/snps_sets/neutral.log
```

```
## 237 samples (30 females, 67 males, 140 ambiguous; 237 founders) loaded from
## 9483 variants loaded from output/populations/neutral.bim.
## --extract: 9047 variants remaining.
## --geno: 0 variants removed due to missing genotype data.
## 0 variants removed due to allele frequency threshold(s)
## 9047 variants remaining after main filters.
```

Train with the pckmeans initialization Get two gpu/cpus

```
salloc --cpus-per-gpu=2 --gpus=2 --time=30:00 --partition gpu_devel
```

Train

```
cd /gpfs/ycga/project/caccone/lvc26/neuroadmix;
module load miniconda/23.1.0;
conda activate nadmenv;

# pckmeans r_0.1
neural-admixture train --seed 1234 --initialization pckmeans --warmup_epochs 1000 --max_epochs 1000 --activation relu --optimizer adam --learning_rate 1e-7 --min_k 2 --max_k 10 --name neutral --data_path /gpfs/ycga/project/caccone/lvc26/neuroadmix/train/neutral_train.bed --save_dir /gpfs/ycga/project/caccone/lvc26/neuroadmix/neutral | tee neutral.log
```

Download the data

```
rsync -chavzP --stats lvc26@mccleary.ycrc.yale.edu:/gpfs/ycga/project/caccone/lvc26/neuroadmix/neutral /Users/lucianocosme/Library/CloudStorage/Dropbox/Albopictus/manuscript_chip/data/no_autogenous/albo_chip/output/populations/nadmix/results
```

### 3.1. Plot after training

```
# Extract ancestry coefficients
nadmixk4 <- read_delim(
  here("output", "populations", "nadmix", "results", "neutral","neutral.4.Q"),
  delim = " ", # Specify the delimiter if different from the default (comma)
  col_names = FALSE,
  show_col_types = FALSE
) 
# unseen_pckmeans.7.Q
# pckmeans.7.Q
head(nadmixk4)
```

```
## # A tibble: 6 × 4
##      X1       X2     X3    X4
##   <dbl>    <dbl>  <dbl> <dbl>
## 1 0.838 0.000436 0.0381 0.123
## 2 0.867 0.00224  0.0160 0.115
## 3 0.770 0.000612 0.0724 0.157
## 4 0.799 0.000301 0.0347 0.166
## 5 0.755 0.00228  0.0647 0.178
## 6 0.814 0.0196   0.0331 0.134
```

The fam file

```
fam_file <- here(
  "output", "populations", "nadmix", "train","neutral_train.fam"
)

# Read the .fam file
fam_data <- read.table(fam_file, 
                       header = FALSE,
                       col.names = c("FamilyID", "IndividualID", "PaternalID", "MaternalID", "Sex", "Phenotype"))

# View the first few rows
head(fam_data)
```

```
##   FamilyID IndividualID PaternalID MaternalID Sex Phenotype
## 1      YUN         1089          0          0   1        -9
## 2      YUN         1090          0          0   1        -9
## 3      YUN         1091          0          0   1        -9
## 4      YUN         1092          0          0   1        -9
## 5      YUN         1093          0          0   1        -9
## 6      YUN         1094          0          0   1        -9
```

Create ID column

```
# Change column name
colnames(fam_data)[colnames(fam_data) == "IndividualID"] <- "ind"


# Merge columns "FamilyID" and "IndividualID" with an underscore
# fam_data$ind <- paste(fam_data$FamilyID, fam_data$IndividualID, sep = "_")


# Change column name
colnames(fam_data)[colnames(fam_data) == "FamilyID"] <- "pop"

# Select ID
fam_data <- fam_data |>
  dplyr::select("ind", "pop")

# View the first few rows
head(fam_data)
```

```
##    ind pop
## 1 1089 YUN
## 2 1090 YUN
## 3 1091 YUN
## 4 1092 YUN
## 5 1093 YUN
## 6 1094 YUN
```

Add it to matrix

```
nadmixk4 <- fam_data |>
  dplyr::select(ind, pop) |>
  bind_cols(nadmixk4)

head(nadmixk4)
```

```
##    ind pop        X1           X2         X3        X4
## 1 1089 YUN 0.8380623 0.0004355365 0.03813944 0.1233627
## 2 1090 YUN 0.8666582 0.0022393065 0.01604919 0.1150533
## 3 1091 YUN 0.7700980 0.0006116223 0.07239948 0.1568909
## 4 1092 YUN 0.7988410 0.0003007596 0.03468554 0.1661727
## 5 1093 YUN 0.7551251 0.0022792281 0.06472636 0.1778693
## 6 1094 YUN 0.8135621 0.0196489729 0.03308360 0.1337053
```

Rename the columns

```
# Rename the columns starting from the third one
nadmixk4 <- nadmixk4 |>
  rename_with(~paste0("v", seq_along(.x)), .cols = -c(ind, pop))

# View the first few rows
head(nadmixk4)
```

```
##    ind pop        v1           v2         v3        v4
## 1 1089 YUN 0.8380623 0.0004355365 0.03813944 0.1233627
## 2 1090 YUN 0.8666582 0.0022393065 0.01604919 0.1150533
## 3 1091 YUN 0.7700980 0.0006116223 0.07239948 0.1568909
## 4 1092 YUN 0.7988410 0.0003007596 0.03468554 0.1661727
## 5 1093 YUN 0.7551251 0.0022792281 0.06472636 0.1778693
## 6 1094 YUN 0.8135621 0.0196489729 0.03308360 0.1337053
```

Import sample locations

```
sampling_loc <- readRDS(here("output", "local_adaptation", "sampling_loc.rds"))
head(sampling_loc)
```

```
## # A tibble: 6 × 6
##   Pop_City   Country  Latitude Longitude Region Abbreviation
##   <chr>      <chr>       <dbl>     <dbl> <chr>  <chr>       
## 1 Gelephu    Bhutan       26.9      90.5 Asia   GEL         
## 2 Phnom Penh Cambodia     11.6     105.  Asia   CAM         
## 3 Hainan     China        19.2     110.  Asia   HAI         
## 4 Yunnan     China        24.5     101.  Asia   YUN         
## 5 Hunan      China        27.6     112.  Asia   HUN         
## 6 Bengaluru  India        13.0      77.6 Asia   BEN
```

```
source(
  here(
    "scripts", "analysis", "my_theme3.R"
  )
)

# Create a named vector to map countries to regions
country_to_region <- c(
  "Bhutan" = "South Asia",
  "Cambodia" = "Southeast Asia",
  "China" = "East Asia",
  "India" = "South Asia",
  "Indonesia" = "Southeast Asia",
  "Japan" = "East Asia",
  "Malaysia" = "Southeast Asia",
  "Maldives" = "South Asia",
  "Nepal" = "South Asia",
  "Sri Lanka" = "South Asia",
  "Taiwan" = "East Asia",
  "Thailand" = "Southeast Asia",
  "Vietnam" = "Southeast Asia"
)

# Add the region to the data frame
sampling_loc$Region2 <- country_to_region[sampling_loc$Country]

# Melt the data frame for plotting
Q_melted <- nadmixk4 |>
  pivot_longer(
    cols = -c(ind, pop),
    names_to = "variable",
    values_to = "value"
  )
# Join with sampling_loc to get sampling localities
Q_joined <- Q_melted |>
  left_join(sampling_loc, by = c("pop" = "Abbreviation"))

# Create a combined variable for Region and Country
Q_joined <- Q_joined |>
  mutate(Region_Country = interaction(Region, Country, sep = "_"))

# Order the combined variable by Region and Country, then by individual
Q_ordered <- Q_joined |>
  arrange(Region, Region2, Country, ind) |>
  mutate(ind = factor(ind, levels = unique(ind)))  # Convert ind to a factor with levels in the desired order

# Add labels: country names for the first individual in each country, NA for all other individuals
Q_ordered <- Q_ordered |>
  group_by(Region_Country) |>
  mutate(label = ifelse(row_number() == 1, as.character(Country), NA))

# Group by individual and variable, calculate mean ancestry proportions
Q_grouped <- Q_ordered |>
  group_by(ind, variable) |>
  summarise(value = mean(value), .groups = "drop")

# Create a data frame for borders
borders <-
  data.frame(Region_Country = unique(Q_ordered$Region_Country))

# Add the order of the last individual of each country to ensure correct placement of borders
borders$order <-
  sapply(borders$Region_Country, function(rc)
    max(which(Q_ordered$Region_Country == rc))) + 0.5  # Shift borders to the right edge of the bars

# Select only the first occurrence of each country in the ordered data
label_df <- Q_ordered |>
  filter(!is.na(label)) |>
  distinct(label, .keep_all = TRUE)

# Create a custom label function
label_func <- function(x) {
  labels <- rep("", length(x))
  labels[x %in% label_df$ind] <- label_df$label
  labels
}

# Calculate the position of lines
border_positions <- Q_ordered |>
  group_by(Country) |>
  summarise(pos = max(as.numeric(ind)) + 0)

# Calculate the position of population labels and bars
pop_labels <- Q_ordered |>
  mutate(Name = paste(pop, Pop_City, sep = " - ")) |>
  group_by(pop) |>
  slice_head(n = 1) |>
  ungroup() |>
  dplyr::select(ind, Pop_City, Country, Name) |>
  mutate(pos = as.numeric(ind))  # calculate position of population labels

pop_labels_bars <- pop_labels |>
  mutate(pos = as.numeric(ind)  - .5)


# Calculate the position of lines
border_positions <- Q_ordered |>
  group_by(Country) |>
  summarise(pos = max(as.numeric(ind)) - 1)


pop_labels_bars <- pop_labels |>
  mutate(pos = as.numeric(ind)  - .5)

# Function to filter and normalize data
normalize_data <- function(df, min_value) {
  df |>
    filter(value > min_value) |>
    group_by(ind) |>
    mutate(value = value / sum(value))
}

# Use the function
Q_grouped_filtered <- normalize_data(Q_grouped, 0.1)

color_palette <-
  c(
    "V1" = "#FFB347",
    "V2" = "#F49AC2",
    "V3" = "#AE9393",
    "V4" = "red"
  )

# Generate all potential variable names
all_variables <- paste0("v", 1:4)

# Map each variable to a name
color_mapping <- data.frame(variable = all_variables,
                            color = names(color_palette))

# Merge with Q_grouped_filtered
Q_grouped_filtered <- merge(Q_grouped_filtered, color_mapping, by = "variable")

# Create the plot
ggplot(Q_grouped_filtered, aes(x = as.factor(ind), y = value, fill = color)) +
  geom_bar(stat = 'identity', width = 1) +
  geom_vline(
    data = pop_labels_bars,
    aes(xintercept = pos),
    color = "#2C444A",
    linewidth = .2
  ) +
  geom_text(
    data = pop_labels,
    aes(x = as.numeric(ind), y = 1, label = Name),
    vjust = 1.5,
    hjust = 0,
    size = 2,
    angle = 90,
    inherit.aes = FALSE
  ) +
  my_theme() +
  theme(
    axis.text.x = element_text(
      angle = 90,
      hjust = 1,
      size = 12
    ),
    legend.position = "none",
    plot.margin = unit(c(3, 0.5, 0.5, 0.5), "cm")
  ) +
  xlab("Admixture matrix") +
  ylab("Ancestry proportions") +
  labs(caption = "Each bar represents the ancestry proportions for an individual for k=4.\n Neuro-admixture training k4 with 20,931 SNPs.") +
  scale_x_discrete(labels = label_func) +
  scale_fill_manual(values = color_palette) +
  expand_limits(y = c(0, 1.5))
```

```
# # # save it
ggsave(
  here("output", "populations", "figures", "neuro_admixture_k=4_neutral_trained.pdf"),
  width  = 12,
  height = 6,
  units  = "in",
  device = cairo_pdf
)
```

### 3.2 Inference with all populations

Transfer data to cluster

```
rsync -chavzP --stats /Users/lucianocosme/Library/CloudStorage/Dropbox/Albopictus/manuscript_chip/data/no_autogenous/albo_chip/output/populations/nadmix lvc26@mccleary.ycrc.yale.edu:/gpfs/ycga/project/caccone/lvc26/
```

Inference mode (projective analysis)

```
neural-admixture infer --name neutral --save_dir /gpfs/ycga/project/caccone/lvc26/neuroadmix/neutral --out_name neutral_inference --data_path /gpfs/ycga/project/caccone/lvc26/neuroadmix/snps_sets/neutral.bed | tee neutral_inference.log
```

### 3.3 Plot after inference with all populations

Download the data

```
rsync -chavzP --stats lvc26@mccleary.ycrc.yale.edu:/gpfs/ycga/project/caccone/lvc26/neuroadmix/neutral /Users/lucianocosme/Library/CloudStorage/Dropbox/Albopictus/manuscript_chip/data/no_autogenous/albo_chip/output/populations/nadmix/results
```

### 3.4 Plot inference

```
# Extract ancestry coefficients
nadmixk5 <- read_delim(
  here("output", "populations", "nadmix", "results", "neutral","neutral_inference.5.Q"),
  delim = " ", # Specify the delimiter if different from the default (comma)
  col_names = FALSE,
  show_col_types = FALSE
) 
# unseen_pckmeans.7.Q
# pckmeans.7.Q
head(nadmixk5)
```

```
## # A tibble: 6 × 5
##      X1     X2    X3    X4    X5
##   <dbl>  <dbl> <dbl> <dbl> <dbl>
## 1 0.247 0.182  0.210 0.154 0.208
## 2 0.214 0.0904 0.306 0.208 0.182
## 3 0.257 0.179  0.191 0.161 0.211
## 4 0.373 0.109  0.117 0.257 0.144
## 5 0.356 0.110  0.138 0.255 0.141
## 6 0.324 0.162  0.168 0.165 0.181
```

The fam file

```
fam_file <- here(
  "output", "populations", "snps_sets", "neutral.fam"
)

# Read the .fam file
fam_data <- read.table(fam_file, 
                       header = FALSE,
                       col.names = c("FamilyID", "IndividualID", "PaternalID", "MaternalID", "Sex", "Phenotype"))

# View the first few rows
head(fam_data)
```

```
##   FamilyID IndividualID PaternalID MaternalID Sex Phenotype
## 1      OKI         1001          0          0   2        -9
## 2      OKI         1002          0          0   2        -9
## 3      OKI         1003          0          0   2        -9
## 4      OKI         1004          0          0   2        -9
## 5      OKI         1005          0          0   2        -9
## 6      OKI         1006          0          0   1        -9
```

Create ID column

```
# Change column name
colnames(fam_data)[colnames(fam_data) == "IndividualID"] <- "ind"


# Merge columns "FamilyID" and "IndividualID" with an underscore
# fam_data$ind <- paste(fam_data$FamilyID, fam_data$IndividualID, sep = "_")


# Change column name
colnames(fam_data)[colnames(fam_data) == "FamilyID"] <- "pop"

# Select ID
fam_data <- fam_data |>
  dplyr::select("ind", "pop")

# View the first few rows
head(fam_data)
```

```
##    ind pop
## 1 1001 OKI
## 2 1002 OKI
## 3 1003 OKI
## 4 1004 OKI
## 5 1005 OKI
## 6 1006 OKI
```

Add it to matrix

```
nadmixk5 <- fam_data |>
  dplyr::select(ind, pop) |>
  bind_cols(nadmixk5)

head(nadmixk5)
```

```
##    ind pop        X1         X2        X3        X4        X5
## 1 1001 OKI 0.2465583 0.18223503 0.2096153 0.1539955 0.2075958
## 2 1002 OKI 0.2139250 0.09040003 0.3061219 0.2078033 0.1817497
## 3 1003 OKI 0.2573991 0.17856130 0.1912381 0.1614681 0.2113334
## 4 1004 OKI 0.3727467 0.10854463 0.1174154 0.2574833 0.1438100
## 5 1005 OKI 0.3558274 0.10966873 0.1382425 0.2551490 0.1411124
## 6 1006 OKI 0.3244200 0.16158162 0.1678030 0.1649402 0.1812551
```

Rename the columns

```
# Rename the columns starting from the third one
nadmixk5 <- nadmixk5 |>
  rename_with(~paste0("v", seq_along(.x)), .cols = -c(ind, pop))

# View the first few rows
head(nadmixk5)
```

```
##    ind pop        v1         v2        v3        v4        v5
## 1 1001 OKI 0.2465583 0.18223503 0.2096153 0.1539955 0.2075958
## 2 1002 OKI 0.2139250 0.09040003 0.3061219 0.2078033 0.1817497
## 3 1003 OKI 0.2573991 0.17856130 0.1912381 0.1614681 0.2113334
## 4 1004 OKI 0.3727467 0.10854463 0.1174154 0.2574833 0.1438100
## 5 1005 OKI 0.3558274 0.10966873 0.1382425 0.2551490 0.1411124
## 6 1006 OKI 0.3244200 0.16158162 0.1678030 0.1649402 0.1812551
```

Import sample locations

```
sampling_loc <- readRDS(here("output", "local_adaptation", "sampling_loc.rds"))
head(sampling_loc)
```

```
## # A tibble: 6 × 6
##   Pop_City   Country  Latitude Longitude Region Abbreviation
##   <chr>      <chr>       <dbl>     <dbl> <chr>  <chr>       
## 1 Gelephu    Bhutan       26.9      90.5 Asia   GEL         
## 2 Phnom Penh Cambodia     11.6     105.  Asia   CAM         
## 3 Hainan     China        19.2     110.  Asia   HAI         
## 4 Yunnan     China        24.5     101.  Asia   YUN         
## 5 Hunan      China        27.6     112.  Asia   HUN         
## 6 Bengaluru  India        13.0      77.6 Asia   BEN
```

```
source(
  here(
    "scripts", "analysis", "my_theme3.R"
  )
)

# Create a named vector to map countries to regions
country_to_region <- c(
  "Bhutan" = "South Asia",
  "Cambodia" = "Southeast Asia",
  "China" = "East Asia",
  "India" = "South Asia",
  "Indonesia" = "Southeast Asia",
  "Japan" = "East Asia",
  "Malaysia" = "Southeast Asia",
  "Maldives" = "South Asia",
  "Nepal" = "South Asia",
  "Sri Lanka" = "South Asia",
  "Taiwan" = "East Asia",
  "Thailand" = "Southeast Asia",
  "Vietnam" = "Southeast Asia"
)

# Add the region to the data frame
sampling_loc$Region2 <- country_to_region[sampling_loc$Country]

# Melt the data frame for plotting
Q_melted <- nadmixk5 |>
  pivot_longer(
    cols = -c(ind, pop),
    names_to = "variable",
    values_to = "value"
  )
# Join with sampling_loc to get sampling localities
Q_joined <- Q_melted |>
  left_join(sampling_loc, by = c("pop" = "Abbreviation"))

# Create a combined variable for Region and Country
Q_joined <- Q_joined |>
  mutate(Region_Country = interaction(Region, Country, sep = "_"))

# Order the combined variable by Region and Country, then by individual
Q_ordered <- Q_joined |>
  arrange(Region, Region2, Country, ind) |>
  mutate(ind = factor(ind, levels = unique(ind)))  # Convert ind to a factor with levels in the desired order

# Add labels: country names for the first individual in each country, NA for all other individuals
Q_ordered <- Q_ordered |>
  group_by(Region_Country) |>
  mutate(label = ifelse(row_number() == 1, as.character(Country), NA))

# Group by individual and variable, calculate mean ancestry proportions
Q_grouped <- Q_ordered |>
  group_by(ind, variable) |>
  summarise(value = mean(value), .groups = "drop")

# Create a data frame for borders
borders <-
  data.frame(Region_Country = unique(Q_ordered$Region_Country))

# Add the order of the last individual of each country to ensure correct placement of borders
borders$order <-
  sapply(borders$Region_Country, function(rc)
    max(which(Q_ordered$Region_Country == rc))) + 0.5  # Shift borders to the right edge of the bars

# Select only the first occurrence of each country in the ordered data
label_df <- Q_ordered |>
  filter(!is.na(label)) |>
  distinct(label, .keep_all = TRUE)

# Create a custom label function
label_func <- function(x) {
  labels <- rep("", length(x))
  labels[x %in% label_df$ind] <- label_df$label
  labels
}

# Calculate the position of lines
border_positions <- Q_ordered |>
  group_by(Country) |>
  summarise(pos = max(as.numeric(ind)) + 0)

# Calculate the position of population labels and bars
pop_labels <- Q_ordered |>
  mutate(Name = paste(pop, Pop_City, sep = " - ")) |>
  group_by(pop) |>
  slice_head(n = 1) |>
  ungroup() |>
  dplyr::select(ind, Pop_City, Country, Name) |>
  mutate(pos = as.numeric(ind))  # calculate position of population labels

pop_labels_bars <- pop_labels |>
  mutate(pos = as.numeric(ind)  - .5)


# Calculate the position of lines
border_positions <- Q_ordered |>
  group_by(Country) |>
  summarise(pos = max(as.numeric(ind)) - 1)


pop_labels_bars <- pop_labels |>
  mutate(pos = as.numeric(ind)  - .5)

# Function to filter and normalize data
normalize_data <- function(df, min_value) {
  df |>
    filter(value > min_value) |>
    group_by(ind) |>
    mutate(value = value / sum(value))
}

# Use the function
Q_grouped_filtered <- normalize_data(Q_grouped, 0.1)

color_palette <-
  c(
    "V1" = "#AE9393",
    "V2" = "red",
    "V3" = "#FFFF99",
    "V4" = "#F49AC2",
    "V5" = "#FFB347"
  )

# Generate all potential variable names
all_variables <- paste0("v", 1:5)

# Map each variable to a name
color_mapping <- data.frame(variable = all_variables,
                            color = names(color_palette))

# Merge with Q_grouped_filtered
Q_grouped_filtered <- merge(Q_grouped_filtered, color_mapping, by = "variable")

# Create the plot
ggplot(Q_grouped_filtered, aes(x = as.factor(ind), y = value, fill = color)) +
  geom_bar(stat = 'identity', width = 1) +
  geom_vline(
    data = pop_labels_bars,
    aes(xintercept = pos),
    color = "#2C444A",
    linewidth = .2
  ) +
  geom_text(
    data = pop_labels,
    aes(x = as.numeric(ind), y = 1, label = Name),
    vjust = 1.5,
    hjust = 0,
    size = 2,
    angle = 90,
    inherit.aes = FALSE
  ) +
  my_theme() +
  theme(
    axis.text.x = element_text(
      angle = 90,
      hjust = 1,
      size = 12
    ),
    legend.position = "none",
    plot.margin = unit(c(3, 0.5, 0.5, 0.5), "cm")
  ) +
  xlab("Admixture matrix") +
  ylab("Ancestry proportions") +
  labs(caption = "Each bar represents the ancestry proportions for an individual for k=5.\n Neuro-admixture inference for k5 with intergenic SNPs.") +
  scale_x_discrete(labels = label_func) +
  scale_fill_manual(values = color_palette) +
  expand_limits(y = c(0, 1.5))
```

```
ggsave(
  here("output", "populations", "figures", "neuro_admixture_k=5_neutral_inference.pdf"),
  width  = 12,
  height = 6,
  units  = "in",
  device = cairo_pdf
)
```
