# Supplementary material for: A genotyping array for the globally invasive vector mosquito, Aedes albopictus
Source: Parasit Vectors. 2024 Mar 4;17:106. doi: 10.1186/s13071-024-06158-z (PMC10910840; doi:10.1186/s13071-024-06158-z)
Supplement: Supplementary file 13 — Additional file 13. Interpolation of admixture matrices over Asia. [file 13071_2024_6158_MOESM13_ESM.html]

 

 

 

 
 
 


 

 

 Aedes albopictus SNP chip - Interpolation of Q matrices over Asia. 

 
 
 
 
 
 
 
 
 

 


 
 
 


 


 


 

 

 


 

 


 


 


 Aedes albopictus SNP chip - Interpolation
of Q matrices over Asia. 
 Luciano V Cosme 
 2023-08-29 

 

 
 
  Overview  
  Packages 
 
  Non-standard Packages  
  
  Standard
Packages 
 
  1. Load the
Packages  
  2. LEA neutral
k=7 
 
  2.1 Q-values  
  2.2 Preparing the
data for tess3Q_map_rasters  
  2.3 Make a matrix of the Q
values  
  2.4 Interpolate the
Q-values by Kriging  
  2.5 Scaling and
cleaning the genoscape_brick  
  
  3. LEA neutral
k=5 
 
  3.1 Q-values  
  3.2 Preparing the
data for tess3Q_map_rasters  
  3.3 make a matrix of the Q
values  
  3.4 Interpolate the
Q-values by Kriging  
  3.5 Scaling and
cleaning the genoscape_brick  
  
  4. LEA r2 0.01
k=5 
 
  4.1 Q-values  
  4.2 Preparing the
data for tess3Q_map_rasters  
  4.3 make a matrix of the Q
values  
  4.4 Interpolate the
Q-values by Kriging  
  4.5 Scaling and
cleaning the genoscape_brick  
  
  5. LEA r2 0.1
k=5 
 
  5.1 Q-values  
  5.2 Preparing the
data for tess3Q_map_rasters  
  5.3 make a matrix of the Q
values  
  5.4 Interpolate the
Q-values by Kriging  
  5.5 Scaling and
cleaning the genoscape_brick  
  
  6. fastStructure
neutral SNPs simple prior 
 
  6.1 Preparing the
data for tess3Q_map_rasters  
  6.2 make a matrix of the Q
values  
  6.3 Interpolate the
Q-values by Kriging  
  6.4 Scaling and
cleaning the genoscape_brick  
  
  7. fastStructure
neutral SNPs logistic prior 
 
  7.1 Preparing the
data for tess3Q_map_rasters  
  7.2 make a matrix of the Q
values  
  7.3 Interpolate the
Q-values by Kriging  
  7.4 Scaling and
cleaning the genoscape_brick  
  
  8. fastStructure r2
0.01 SNPs simple prior 
 
  8.1 Preparing the
data for tess3Q_map_rasters  
  8.2 make a matrix of the Q
values  
  8.3 Interpolate the
Q-values by Kriging  
  8.4 Scaling and
cleaning the genoscape_brick  
  
  9. fastStructure r2
0.01 SNPs logistic prior 
 
  9.1 Preparing the
data for tess3Q_map_rasters  
  9.2 make a matrix of the Q
values  
  9.3 Interpolate the
Q-values by Kriging  
  9.4 Scaling and
cleaning the genoscape_brick  
  
  10. fastStructure r2 0.1
SNPs simple prior 
 
  10.1 Preparing the
data for tess3Q_map_rasters  
  10.2 make a matrix of the Q
values  
  10.3 Interpolate the
Q-values by Kriging  
  10.4 Scaling and
cleaning the genoscape_brick  
  
  11. fastStructure r2
0.1 SNPs logistic prior 
 
  11.1 Preparing the
data for tess3Q_map_rasters  
  11.2 make a matrix of the Q
values  
  11.3 Interpolate the
Q-values by Kriging  
  11.4 Scaling and
cleaning the genoscape_brick  
  
  12. Admixture neutral SNPs k5 
 
  12.1 Preparing the
data for tess3Q_map_rasters  
  12.2 make a matrix of the Q
values  
  12.3 Interpolate the
Q-values by Kriging  
  12.4 Scaling and
cleaning the genoscape_brick  
  
  13. Admixture r2 0.01 SNPs k5 
 
  13.1 Preparing the
data for tess3Q_map_rasters  
  13.2 make a matrix of the Q
values  
  13.3 Interpolate the
Q-values by Kriging  
  13.4 Scaling and
cleaning the genoscape_brick  
  
  14. Admixture r2 0.1 SNPs k5 
 
  14.1 Preparing the
data for tess3Q_map_rasters  
  14.2 make a matrix of the Q
values  
  14.3 Interpolate the
Q-values by Kriging  
  14.4 Scaling and
cleaning the genoscape_brick  
  
  15. neuro-admxiture r2 0.01
SNPs k5 
 
  15.1 Q-values  
  15.2 Preparing the
data for tess3Q_map_rasters  
  15.3 make a matrix of the Q
values  
  15.4 Interpolate the
Q-values by Kriging  
  15.5 Scaling and
cleaning the genoscape_brick  
  
  16. neuro-admxiture r2 0.1 SNPs
k5 
 
  16.1 Q-values  
  16.2 Preparing the
data for tess3Q_map_rasters  
  16.3 make a matrix of the Q
values  
  16.4 Interpolate the
Q-values by Kriging  
  16.5 Scaling and
cleaning the genoscape_brick  
  
  17. neuro-admxiture
neutral r2 0.1 SNPs k5 
 
  17.1 Q-values  
  17.2 Preparing the
data for tess3Q_map_rasters  
  17.3 make a matrix of the Q
values  
  17.4 Interpolate the
Q-values by Kriging  
  17.5 Scaling and
cleaning the genoscape_brick  
  
  
 
 

 
 Overview 
 To work through this, clone the repository (an RStudio project) at  https://github.com/eriqande/make-a-BGP-map 
to get all the necessary input files, etc. Then open up the RStudio
project and run through ‘Make-a-BGP-map-Notebook.Rmd’. 
 
 
 Packages 
 
 Non-standard Packages 
 
  fork  of  tess3r . Note that you can’t
use the default version of  tess3r , you have to use my fork
of it, which has some extra functionality. 
 the package  genoscapeRtools  
 
 Get those packages like this: 
      remotes ::  install_github ( "eriqande/TESS3_encho_sen" )   # for special version of tess3r  
   remotes ::  install_github ( "eriqande/genoscapeRtools" )   # for Eric's genoscapeRtools     
 
 
 
 Standard Packages 
 The rest of the packages you need can be downloaded from CRAN. If you
don’t have them you should get them:  raster ,
 sf ,  fields ,  downloader , and
 tidyverse . The last one there gets ggplot2 and a number of
other packages by Hadley Wickham. 
 You can get those like this: 
       install.packages ( c ( "raster" ,  "sf" ,  "tidyverse" ,  "fields" ,  "downloader" ))    
 
 1. Load the Packages 
       library (raster)   # important to load before tidyverse, otherwise it masks select()  
    library (tidyverse) 
    library (sf) 
    library (ggspatial) 
    library (ggplot2) 
    library (dplyr) 
    library (colorout) 
    library (here) 
    library (scatterpie) 
    library (rnaturalearth) 
    library (rnaturalearthdata) 
    library (ggrepel) 
    library (Cairo)    
 
 
 2. LEA neutral k=7 
 Clear memory and environment 
       # Clear entire environment  
    rm ( list =   ls ()) 
    # Forcefully trigger garbage collection  
    gc ()    
  ##           used  (Mb) gc trigger  (Mb) limit (Mb) max used  (Mb)
## Ncells 2908731 155.4    5488260 293.2         NA  4203397 224.5
## Vcells 3993455  30.5    8388608  64.0      32768  5745292  43.9  
 
 2.1 Q-values 
       # Extract ancestry coefficients  
   leak7  <-   read_delim ( 
      here ( "output" ,  "populations" ,  "snps_sets" ,  "neutral.snmf" ,  "K7" ,  "run3" , "neutral_r3.7.Q" ), 
      delim =   " " ,  # Specify the delimiter if different from the default (comma)  
      col_names =   FALSE , 
      show_col_types =   FALSE  
   )  
    # unseen_pckmeans.7.Q  
    # pckmeans.7.Q  
    head (leak7)    
  ## # A tibble: 6 × 7
##      X1       X2       X3       X4       X5       X6       X7
##   <dbl>    <dbl>    <dbl>    <dbl>    <dbl>    <dbl>    <dbl>
## 1 0.481 0.0708   0.222    0.00569  0.0413   0.0686   0.110   
## 2 0.241 0.0661   0.271    0.0172   0.0506   0.162    0.192   
## 3 0.730 0.0596   0.129    0.00957  0.000100 0.000100 0.0716  
## 4 0.999 0.000100 0.000100 0.000100 0.000100 0.000100 0.000100
## 5 0.999 0.000100 0.000100 0.000100 0.000100 0.000100 0.000100
## 6 0.721 0.0666   0.110    0.0293   0.000100 0.000100 0.0729  
 The fam file 
      fam_file  <-   here ( 
      "output" ,  "populations" ,  "snps_sets" ,  "neutral.fam"  
   ) 
    
    # Read the .fam file  
   fam_data  <-   read.table (fam_file,  
                           header =   FALSE , 
                           col.names =   c ( "FamilyID" ,  "IndividualID" ,  "PaternalID" ,  "MaternalID" ,  "Sex" ,  "Phenotype" )) 
    
    # View the first few rows  
    head (fam_data)    
  ##   FamilyID IndividualID PaternalID MaternalID Sex Phenotype
## 1      OKI         1001          0          0   2        -9
## 2      OKI         1002          0          0   2        -9
## 3      OKI         1003          0          0   2        -9
## 4      OKI         1004          0          0   2        -9
## 5      OKI         1005          0          0   2        -9
## 6      OKI         1006          0          0   1        -9  
 Create ID column 
       # Change column name  
    colnames (fam_data)[ colnames (fam_data)  ==   "IndividualID" ]  <-   "ind"  
    
    
    # Change column name  
    colnames (fam_data)[ colnames (fam_data)  ==   "FamilyID" ]  <-   "pop"  
    
    # Select ID  
   fam_data  <-  fam_data  |>  
     dplyr ::  select ( "ind" ,  "pop" ) 
    
    # View the first few rows  
    head (fam_data)    
  ##    ind pop
## 1 1001 OKI
## 2 1002 OKI
## 3 1003 OKI
## 4 1004 OKI
## 5 1005 OKI
## 6 1006 OKI  
 Add it to matrix 
      leak7  <-  fam_data  |>  
     dplyr ::  select (ind, pop)  |>  
      bind_cols (leak7) 
    
    head (leak7)    
  ##    ind pop       X1         X2         X3         X4          X5          X6
## 1 1001 OKI 0.481261 0.07082050 0.22190700 0.00569292 0.041312000 0.068599300
## 2 1002 OKI 0.240552 0.06607170 0.27105700 0.01724490 0.050601700 0.162441000
## 3 1003 OKI 0.729612 0.05958920 0.12941900 0.00956504 0.000099982 0.000099982
## 4 1004 OKI 0.999400 0.00009995 0.00009995 0.00009995 0.000099950 0.000099950
## 5 1005 OKI 0.999400 0.00009995 0.00009995 0.00009995 0.000099950 0.000099950
## 6 1006 OKI 0.721117 0.06660400 0.10982200 0.02930780 0.000099982 0.000099982
##           X7
## 1 0.11040800
## 2 0.19203200
## 3 0.07161450
## 4 0.00009995
## 5 0.00009995
## 6 0.07294920  
 Rename the columns 
       # Rename the columns starting from the third one  
   leak7  <-  leak7  |>  
      rename_with ( ~  paste0 ( "v" ,  seq_along (.x)),  .cols =   -  c (ind, pop)) 
    
    # View the first few rows  
    head (leak7)    
  ##    ind pop       v1         v2         v3         v4          v5          v6
## 1 1001 OKI 0.481261 0.07082050 0.22190700 0.00569292 0.041312000 0.068599300
## 2 1002 OKI 0.240552 0.06607170 0.27105700 0.01724490 0.050601700 0.162441000
## 3 1003 OKI 0.729612 0.05958920 0.12941900 0.00956504 0.000099982 0.000099982
## 4 1004 OKI 0.999400 0.00009995 0.00009995 0.00009995 0.000099950 0.000099950
## 5 1005 OKI 0.999400 0.00009995 0.00009995 0.00009995 0.000099950 0.000099950
## 6 1006 OKI 0.721117 0.06660400 0.10982200 0.02930780 0.000099982 0.000099982
##           v7
## 1 0.11040800
## 2 0.19203200
## 3 0.07161450
## 4 0.00009995
## 5 0.00009995
## 6 0.07294920  
 Import samples attributes 
      sampling_loc  <-   readRDS ( here ( "output" ,  "populations" ,  "sampling_loc.rds" )) 
    # head(sampling_loc)  
    
   pops  <-  sampling_loc  |>  
      filter ( 
       Region  ==   "Asia"  
     )  |>  
     dplyr ::  select ( 
       Abbreviation, Latitude, Longitude, Pop_City, Country 
     ) 
    
    head (pops)    
  ## # A tibble: 6 × 5
##   Abbreviation Latitude Longitude Pop_City   Country 
##   <chr>           <dbl>     <dbl> <chr>      <chr>   
## 1 GEL              26.9      90.5 Gelephu    Bhutan  
## 2 CAM              11.6     105.  Phnom Penh Cambodia
## 3 HAI              19.2     110.  Hainan     China   
## 4 YUN              24.5     101.  Yunnan     China   
## 5 HUN              27.6     112.  Hunan      China   
## 6 BEN              13.0      77.6 Bengaluru  India  
 Merge with pops 
       # Add an index column to Q_tibble  
   leak7 $ index  <-   seq_len ( nrow (leak7)) 
    
    # Perform the merge as before  
   df1  <-  
      merge ( 
       leak7, 
       pops, 
        by.x =   2 , 
        by.y =   1 , 
        all.x =  T, 
        all.y =  F 
     )  |>  
      na.omit () 
    
    # Order by the index column to ensure the order matches the original Q_tibble  
   df1  <-  df1[ order (df1 $ index),] 
    
    # Optionally, you can remove the index column if it's no longer needed  
   df1 $ index  <-   NULL  
    
    # Now the rows of df1 should be in the same order as the original Q_tibble  
    head (df1)    
  ##     pop  ind       v1         v2         v3         v4          v5          v6
## 159 OKI 1001 0.481261 0.07082050 0.22190700 0.00569292 0.041312000 0.068599300
## 160 OKI 1002 0.240552 0.06607170 0.27105700 0.01724490 0.050601700 0.162441000
## 161 OKI 1003 0.729612 0.05958920 0.12941900 0.00956504 0.000099982 0.000099982
## 162 OKI 1004 0.999400 0.00009995 0.00009995 0.00009995 0.000099950 0.000099950
## 163 OKI 1005 0.999400 0.00009995 0.00009995 0.00009995 0.000099950 0.000099950
## 164 OKI 1006 0.721117 0.06660400 0.10982200 0.02930780 0.000099982 0.000099982
##             v7 Latitude Longitude Pop_City Country
## 159 0.11040800  26.5013  127.9454  Okinawa   Japan
## 160 0.19203200  26.5013  127.9454  Okinawa   Japan
## 161 0.07161450  26.5013  127.9454  Okinawa   Japan
## 162 0.00009995  26.5013  127.9454  Okinawa   Japan
## 163 0.00009995  26.5013  127.9454  Okinawa   Japan
## 164 0.07294920  26.5013  127.9454  Okinawa   Japan  
 We used this color palette to make the “structure” plot 
      color_palette2  <-  
      c ( 
        "v1"   =   "#AE9393" , 
        "v2"   =   "red" , 
        "v3"   =   "#FFFF99" , 
        "v4"   =   "#D0F0C0" , 
        "v5"   =   "#FFB347" , 
        "v6"   =   "#F49AC2" , 
        "v7"   =   "#AEC6CF"  
     )    
 Make pie plot 
      world  <-   ne_countries ( scale =   "medium" ,  returnclass =   "sf" ) 
   countries_with_data  <-   unique (df1 $ Country) 
    
    # Filtering the world data to include only the countries in your data  
   selected_countries  <-  world  |>  
      filter (admin  %in%  countries_with_data) 
    
    # Calculate mean proportions for each population  
   df_mean  <-  df1  |>  
      group_by (pop)  |>  
      summarise ( across ( starts_with ( "v" ), \(x)  mean (x,  na.rm =   TRUE )),  
                Longitude =   mean (Longitude), 
                Latitude =   mean (Latitude)) 
    
    
    source ( 
      here ( 
        "scripts" ,  "analysis" ,  "my_theme2.R"  
     ) 
   ) 
    
    ggplot ()  +  
      geom_sf ( data =  selected_countries,  fill=  "white" )  +  
      geom_scatterpie ( data =  df_mean,  
                      aes ( x =  Longitude,  y =  Latitude,  r =   1.5 ),  
                      cols =   c ( "v1" ,  "v2" ,  "v3" ,  "v4" ,  "v5" ,  "v6" ,  "v7" ),  color =   NA )  +  
      geom_text_repel ( data =  df_mean, 
                      aes ( x =  Longitude,  y =  Latitude,  label =  pop),  
                      size =   3 ,  
                      box.padding =   unit ( 0.5 ,  "lines" ), 
                      max.overlaps =   50 )  +  
      scale_fill_manual ( values =  color_palette2)  +  
      guides ( fill =   "none" )  +    # Hide legend  
      # coord_sf() +  
      coord_sf ( xlim =   c ( 60 ,  150 ),  ylim =   c ( -  10 ,  60 ))  +  
      my_theme ()    
   
       # #   
    ggsave ( 
      here ( "output" ,  "populations" ,  "figures" ,  "lea_neutral_k7_pie.pdf" ), 
      width  =   12 , 
      height =   6 , 
      units  =   "in" , 
      device =  cairo_pdf 
   )    
 
 
 2.2 Preparing the data for tess3Q_map_rasters 
 Within Eric’s fork of tess3r is a function called tess3Q_map_rasters.
It takes input from the objects we have above, but it takes that input
as matrices rather than data frames, etc. so there is a little finagling
to be done. 
 Make sure the lat longs are in the correct order and arrangement 
      df2  <-  df1  |>  
     dplyr ::  rename ( 
        Long =  Longitude, 
        Lat =  Latitude 
     ) 
    
   long_lat_tibble  <-  df2  |>  
     dplyr ::  select (Long, Lat) 
    
    
   long_lat_matrix  <-  long_lat_tibble  |>  
      as.matrix () 
    
    head (long_lat_matrix)    
  ##         Long     Lat
## 159 127.9454 26.5013
## 160 127.9454 26.5013
## 161 127.9454 26.5013
## 162 127.9454 26.5013
## 163 127.9454 26.5013
## 164 127.9454 26.5013  
 
 
 2.3 Make a matrix of the Q values 
 Pull off the names of individuals and make a matrix of it: 
      Q_matrix  <-  leak7  |>  
     dplyr ::  select ( - ind,  - pop,  - index)  |>  
      as.matrix () 
    head (Q_matrix)    
  ##            v1         v2         v3         v4          v5          v6
## [1,] 0.481261 0.07082050 0.22190700 0.00569292 0.041312000 0.068599300
## [2,] 0.240552 0.06607170 0.27105700 0.01724490 0.050601700 0.162441000
## [3,] 0.729612 0.05958920 0.12941900 0.00956504 0.000099982 0.000099982
## [4,] 0.999400 0.00009995 0.00009995 0.00009995 0.000099950 0.000099950
## [5,] 0.999400 0.00009995 0.00009995 0.00009995 0.000099950 0.000099950
## [6,] 0.721117 0.06660400 0.10982200 0.02930780 0.000099982 0.000099982
##              v7
## [1,] 0.11040800
## [2,] 0.19203200
## [3,] 0.07161450
## [4,] 0.00009995
## [5,] 0.00009995
## [6,] 0.07294920  
 
 
 2.4 Interpolate the Q-values by Kriging 
 For this, we use the above variables in tess3r::tess3Q_map_rasters().
Note the use of namespace addressing for this function rather than
loading the whole tess3r package with the library() command. 
       print ( ncol (Q_matrix)  ==   length (color_palette2))    
  ## [1] TRUE  
 Create brick 
      genoscape_brick  <-  tess3r ::  tess3Q_map_rasters ( 
      x =  Q_matrix,  
      coord =  long_lat_matrix,   
      map.polygon =  selected_countries, 
      window =   extent (selected_countries)[ 1  :  4 ], 
      # window = combined_extent,  
      resolution =   c ( 600 , 600 ),  # if you want more cells in your raster, set higher  
      # this next lines need to to be here, but don't do much...  
      col.palette =  tess3r ::  CreatePalette (color_palette2,  length (color_palette2)), 
      method =   "map.max" ,  
      interpol =  tess3r ::  FieldsKrigModel ( 80 ),   
      main =   "Ancestry coefficients" , 
      xlab =   "Longitude" ,  
      ylab =   "Latitude" ,  
      cex =  . 4  
   )    
  ## Warning: 
## Grid searches over lambda (nugget and sill variances) with  minima at the endpoints: 
##   (REML) Restricted maximum likelihood 
##    minimum at  right endpoint  lambda  =  0.02118404 (eff. df= 26.59999 )
## Warning: 
## Grid searches over lambda (nugget and sill variances) with  minima at the endpoints: 
##   (REML) Restricted maximum likelihood 
##    minimum at  right endpoint  lambda  =  0.02118404 (eff. df= 26.59999 )
## Warning: 
## Grid searches over lambda (nugget and sill variances) with  minima at the endpoints: 
##   (REML) Restricted maximum likelihood 
##    minimum at  right endpoint  lambda  =  31378.74 (eff. df= 3.00096 )
## Warning: 
## Grid searches over lambda (nugget and sill variances) with  minima at the endpoints: 
##   (REML) Restricted maximum likelihood 
##    minimum at  right endpoint  lambda  =  0.02118404 (eff. df= 26.59999 )
## Warning: 
## Grid searches over lambda (nugget and sill variances) with  minima at the endpoints: 
##   (REML) Restricted maximum likelihood 
##    minimum at  right endpoint  lambda  =  0.02118404 (eff. df= 26.59999 )
## Warning: 
## Grid searches over lambda (nugget and sill variances) with  minima at the endpoints: 
##   (REML) Restricted maximum likelihood 
##    minimum at  right endpoint  lambda  =  0.02118404 (eff. df= 26.59999 )
## Warning: 
## Grid searches over lambda (nugget and sill variances) with  minima at the endpoints: 
##   (REML) Restricted maximum likelihood 
##    minimum at  right endpoint  lambda  =  0.02118404 (eff. df= 26.59999 )  
       # after that, we need to add names of the clusters back onto this raster brick  
   Q_tibble2  <-  leak7  |>  
     dplyr ::  select ( 
        - pop,  - ind,  - index 
     ) 
    names (genoscape_brick)  <-   names (Q_tibble2)[]    
 That gives us a raster brick of Q-values associated with each cell in
the raster, but those values are not always constrained between 0 and 1,
so we have to massage them a little bit in the next section. 
 
 
 2.5 Scaling and cleaning the genoscape_brick 
 For this we use the function ‘genoscapeRtools::qprob_rando_raster()’.
This takes the raster brick that comes out of tess3Q_map_rasters() and
does some rescaling and (maybe) some random sampling to return a raster
of colors that I hope will do a reliable job of representing (in some
way) predicted assignment accuracy over space. See
‘?genoscapeRtools::qprob_rando_raster’ to learn about the scaling
options, etc. (However, I am not convinced that all of those options are
reliably estimated.) 
 This will squash the raster brick down to a single RGBA (i.e., four
channels, red, green, blue and alpha) raster brick. 
      genoscape_rgba  <-  genoscapeRtools ::  qprob_rando_raster ( 
      TRB =  genoscape_brick, 
      cols =  color_palette2, 
      alpha_scale =   2.0 , 
      abs_thresh =   0.0 , 
      alpha_exp =   1.55 , 
      alpha_chop_max =   255  
   ) 
    
    # This adds the info for a regular lat-long projection  
    crs (genoscape_rgba)  <-   "+proj=longlat +datum=WGS84 +no_defs +ellps=WGS84 +towgs84=0,0,0"     
 We can easily plot this with the function layer_spatial from the
ggspatial package: 
       ggplot ()  +   
     ggspatial ::  layer_spatial (genoscape_rgba)  +   
      my_theme ()  +  
      coord_sf ()    
 With pies 
       ggplot ()  +  
      layer_spatial (genoscape_rgba)  +  
      geom_spatial_point ( data =  long_lat_tibble, 
                         mapping =   aes ( x =  Long,  y =  Lat), 
                         size =  . 2 )  +  
      geom_text_repel ( 
        data =  df_mean, 
        aes ( x =  Longitude,  y =  Latitude,  label =  pop), 
        size =   3 , 
        box.padding =   unit ( 0.5 ,  "lines" ) 
     )  +  
      labs ( x =   "Longitude" , 
           y =   "Latitude" )  +  
      geom_scatterpie ( data =  df_mean,  
                      aes ( x =  Longitude,  y =  Latitude,  r =   1 ),  
                      cols =   c ( "v1" ,  "v2" ,  "v3" ,  "v4" ,  "v5" ,  "v6" ,  "v7" ),  color =   NA )  +  
      my_theme ()  +  
      scale_fill_manual ( values =  color_palette2)  +  
      guides ( fill =   "none" )  +    # Hide legend  
      coord_sf ()    
  ## Assuming `crs = 4326` in stat_spatial_identity()  
   
       # #   
    ggsave ( 
      here ( "output" ,  "populations" ,  "figures" ,  "lea_neutral_k7_interpolated_pie.pdf" ), 
      width  =   12 , 
      height =   6 , 
      units  =   "in" , 
      device =  cairo_pdf 
   )    
  ## Assuming `crs = 4326` in stat_spatial_identity()  
 
 
 
 3. LEA neutral k=5 
 Clear memory and environment 
       # Clear entire environment  
    rm ( list =   ls ()) 
    # Forcefully trigger garbage collection  
    gc ()    
  ##           used  (Mb) gc trigger  (Mb) limit (Mb) max used  (Mb)
## Ncells 4695143 250.8    8968841 479.0         NA  8968841 479.0
## Vcells 8784693  67.1   43993373 335.7      32768 68732428 524.4  
 
 3.1 Q-values 
       # Extract ancestry coefficients  
   leak5  <-   read_delim ( 
      here ( "output" ,  "populations" ,  "snps_sets" ,  "neutral.snmf" ,  "K5" ,  "run3" , "neutral_r3.5.Q" ), 
      delim =   " " ,  # Specify the delimiter if different from the default (comma)  
      col_names =   FALSE , 
      show_col_types =   FALSE  
   )  
    # unseen_pckmeans.7.Q  
    # pckmeans.7.Q  
    head (leak5)    
  ## # A tibble: 6 × 5
##      X1     X2     X3     X4     X5
##   <dbl>  <dbl>  <dbl>  <dbl>  <dbl>
## 1 0.486 0.156  0.0171 0.0872 0.255 
## 2 0.413 0.221  0.0428 0.0816 0.241 
## 3 0.609 0.0970 0.0268 0.102  0.165 
## 4 0.711 0.123  0.0448 0.0523 0.0687
## 5 0.769 0.128  0.0445 0.0295 0.0289
## 6 0.595 0.117  0.0503 0.0863 0.151  
 The fam file 
      fam_file  <-   here ( 
      "output" ,  "populations" ,  "snps_sets" ,  "neutral.fam"  
   ) 
    
    # Read the .fam file  
   fam_data  <-   read.table (fam_file,  
                           header =   FALSE , 
                           col.names =   c ( "FamilyID" ,  "IndividualID" ,  "PaternalID" ,  "MaternalID" ,  "Sex" ,  "Phenotype" )) 
    
    # View the first few rows  
    head (fam_data)    
  ##   FamilyID IndividualID PaternalID MaternalID Sex Phenotype
## 1      OKI         1001          0          0   2        -9
## 2      OKI         1002          0          0   2        -9
## 3      OKI         1003          0          0   2        -9
## 4      OKI         1004          0          0   2        -9
## 5      OKI         1005          0          0   2        -9
## 6      OKI         1006          0          0   1        -9  
 Create ID column 
       # Change column name  
    colnames (fam_data)[ colnames (fam_data)  ==   "IndividualID" ]  <-   "ind"  
    
    # Change column name  
    colnames (fam_data)[ colnames (fam_data)  ==   "FamilyID" ]  <-   "pop"  
    
    # Select ID  
   fam_data  <-  fam_data  |>  
     dplyr ::  select ( "ind" ,  "pop" ) 
    
    # View the first few rows  
    head (fam_data)    
  ##    ind pop
## 1 1001 OKI
## 2 1002 OKI
## 3 1003 OKI
## 4 1004 OKI
## 5 1005 OKI
## 6 1006 OKI  
 Add it to matrix 
      leak5  <-  fam_data  |>  
     dplyr ::  select (ind, pop)  |>  
      bind_cols (leak5) 
    
    head (leak5)    
  ##    ind pop       X1        X2        X3        X4        X5
## 1 1001 OKI 0.485589 0.1555300 0.0171214 0.0872414 0.2545190
## 2 1002 OKI 0.413323 0.2213730 0.0427845 0.0816402 0.2408790
## 3 1003 OKI 0.609096 0.0969784 0.0268309 0.1019500 0.1651450
## 4 1004 OKI 0.711456 0.1227820 0.0447598 0.0522809 0.0687215
## 5 1005 OKI 0.769209 0.1279830 0.0444730 0.0294702 0.0288648
## 6 1006 OKI 0.595327 0.1167970 0.0502820 0.0862996 0.1512940  
 Rename the columns 
       # Rename the columns starting from the third one  
   leak5  <-  leak5  |>  
      rename_with ( ~  paste0 ( "v" ,  seq_along (.x)),  .cols =   -  c (ind, pop)) 
    
    # View the first few rows  
    head (leak5)    
  ##    ind pop       v1        v2        v3        v4        v5
## 1 1001 OKI 0.485589 0.1555300 0.0171214 0.0872414 0.2545190
## 2 1002 OKI 0.413323 0.2213730 0.0427845 0.0816402 0.2408790
## 3 1003 OKI 0.609096 0.0969784 0.0268309 0.1019500 0.1651450
## 4 1004 OKI 0.711456 0.1227820 0.0447598 0.0522809 0.0687215
## 5 1005 OKI 0.769209 0.1279830 0.0444730 0.0294702 0.0288648
## 6 1006 OKI 0.595327 0.1167970 0.0502820 0.0862996 0.1512940  
 Import samples attributes 
      sampling_loc  <-   readRDS ( here ( "output" ,  "populations" ,  "sampling_loc.rds" )) 
    # head(sampling_loc)  
    
   pops  <-  sampling_loc  |>  
      filter ( 
       Region  ==   "Asia"  
     )  |>  
     dplyr ::  select ( 
       Abbreviation, Latitude, Longitude, Pop_City, Country 
     ) 
    
    head (pops)    
  ## # A tibble: 6 × 5
##   Abbreviation Latitude Longitude Pop_City   Country 
##   <chr>           <dbl>     <dbl> <chr>      <chr>   
## 1 GEL              26.9      90.5 Gelephu    Bhutan  
## 2 CAM              11.6     105.  Phnom Penh Cambodia
## 3 HAI              19.2     110.  Hainan     China   
## 4 YUN              24.5     101.  Yunnan     China   
## 5 HUN              27.6     112.  Hunan      China   
## 6 BEN              13.0      77.6 Bengaluru  India  
 Merge with pops 
       # Add an index column to Q_tibble  
   leak5 $ index  <-   seq_len ( nrow (leak5)) 
    
    # Perform the merge as before  
   df1  <-  
      merge ( 
       leak5, 
       pops, 
        by.x =   2 , 
        by.y =   1 , 
        all.x =  T, 
        all.y =  F 
     )  |>  
      na.omit () 
    
    # Order by the index column to ensure the order matches the original Q_tibble  
   df1  <-  df1[ order (df1 $ index),] 
    
    # Optionally, you can remove the index column if it's no longer needed  
   df1 $ index  <-   NULL  
    
    # Now the rows of df1 should be in the same order as the original Q_tibble  
    head (df1)    
  ##     pop  ind       v1        v2        v3        v4        v5 Latitude
## 159 OKI 1001 0.485589 0.1555300 0.0171214 0.0872414 0.2545190  26.5013
## 160 OKI 1002 0.413323 0.2213730 0.0427845 0.0816402 0.2408790  26.5013
## 161 OKI 1003 0.609096 0.0969784 0.0268309 0.1019500 0.1651450  26.5013
## 162 OKI 1004 0.711456 0.1227820 0.0447598 0.0522809 0.0687215  26.5013
## 163 OKI 1005 0.769209 0.1279830 0.0444730 0.0294702 0.0288648  26.5013
## 164 OKI 1006 0.595327 0.1167970 0.0502820 0.0862996 0.1512940  26.5013
##     Longitude Pop_City Country
## 159  127.9454  Okinawa   Japan
## 160  127.9454  Okinawa   Japan
## 161  127.9454  Okinawa   Japan
## 162  127.9454  Okinawa   Japan
## 163  127.9454  Okinawa   Japan
## 164  127.9454  Okinawa   Japan  
 We used this color palette to make the “structure” plot 
      color_palette2  <-  
      c ( 
        "v1"   =   "#AE9393" , 
        "v2"   =   "#F49AC2" , 
        "v3"   =   "#FFFF99" , 
        "v4"   =   "red" , 
        "v5"   =   "#FFB347"  
     )    
 Make pie plot 
      world  <-   ne_countries ( scale =   "medium" ,  returnclass =   "sf" ) 
   countries_with_data  <-   unique (df1 $ Country) 
    
    # Filtering the world data to include only the countries in your data  
   selected_countries  <-  world  |>  
      filter (admin  %in%  countries_with_data) 
    
    # Calculate mean proportions for each population  
   df_mean  <-  df1  |>  
      group_by (pop)  |>  
      summarise ( across ( starts_with ( "v" ), \(x)  mean (x,  na.rm =   TRUE )),  
                Longitude =   mean (Longitude), 
                Latitude =   mean (Latitude)) 
    
    
    source ( 
      here ( 
        "scripts" ,  "analysis" ,  "my_theme2.R"  
     ) 
   ) 
    
    ggplot ()  +  
      geom_sf ( data =  selected_countries,  fill=  "white" )  +  
      geom_scatterpie ( data =  df_mean,  
                      aes ( x =  Longitude,  y =  Latitude,  r =   1.5 ),  
                      cols =   c ( "v1" ,  "v2" ,  "v3" ,  "v4" ,  "v5" ),  color =   NA )  +  
      geom_text_repel ( data =  df_mean, 
                      aes ( x =  Longitude,  y =  Latitude,  label =  pop),  
                      size =   3 ,  
                      box.padding =   unit ( 0.5 ,  "lines" ), 
                      max.overlaps =   50 )  +  
      scale_fill_manual ( values =  color_palette2)  +  
      guides ( fill =   "none" )  +    # Hide legend  
      # coord_sf() +  
      coord_sf ( xlim =   c ( 60 ,  150 ),  ylim =   c ( -  10 ,  60 ))  +  
      my_theme ()    
   
       # #   
    ggsave ( 
      here ( "output" ,  "populations" ,  "figures" ,  "lea_neutral_k5_pie.pdf" ), 
      width  =   12 , 
      height =   6 , 
      units  =   "in" , 
      device =  cairo_pdf 
   )    
 
 
 3.2 Preparing the data for tess3Q_map_rasters 
 Make sure the lat longs are in the correct order and arrangement 
      df2  <-  df1  |>  
     dplyr ::  rename ( 
        Long =  Longitude, 
        Lat =  Latitude 
     ) 
    
   long_lat_tibble  <-  df2  |>  
     dplyr ::  select (Long, Lat) 
    
    
   long_lat_matrix  <-  long_lat_tibble  |>  
      as.matrix () 
    
    head (long_lat_matrix)    
  ##         Long     Lat
## 159 127.9454 26.5013
## 160 127.9454 26.5013
## 161 127.9454 26.5013
## 162 127.9454 26.5013
## 163 127.9454 26.5013
## 164 127.9454 26.5013  
 
 
 3.3 make a matrix of the Q values 
 Pull off the names of individuals and make a matrix of it: 
      Q_matrix  <-  leak5  |>  
     dplyr ::  select ( - ind,  - pop,  - index)  |>  
      as.matrix () 
    head (Q_matrix)    
  ##            v1        v2        v3        v4        v5
## [1,] 0.485589 0.1555300 0.0171214 0.0872414 0.2545190
## [2,] 0.413323 0.2213730 0.0427845 0.0816402 0.2408790
## [3,] 0.609096 0.0969784 0.0268309 0.1019500 0.1651450
## [4,] 0.711456 0.1227820 0.0447598 0.0522809 0.0687215
## [5,] 0.769209 0.1279830 0.0444730 0.0294702 0.0288648
## [6,] 0.595327 0.1167970 0.0502820 0.0862996 0.1512940  
 
 
 3.4 Interpolate the Q-values by Kriging 
       print ( ncol (Q_matrix)  ==   length (color_palette2))    
  ## [1] TRUE  
 Create brick 
      genoscape_brick  <-  tess3r ::  tess3Q_map_rasters ( 
      x =  Q_matrix,  
      coord =  long_lat_matrix,   
      map.polygon =  selected_countries, 
      window =   extent (selected_countries)[ 1  :  4 ], 
      # window = combined_extent,  
      resolution =   c ( 600 , 600 ),  # if you want more cells in your raster, set higher  
      # this next lines need to to be here, but don't do much...  
      col.palette =  tess3r ::  CreatePalette (color_palette2,  length (color_palette2)), 
      method =   "map.max" ,  
      interpol =  tess3r ::  FieldsKrigModel ( 80 ),   
      main =   "Ancestry coefficients" , 
      xlab =   "Longitude" ,  
      ylab =   "Latitude" ,  
      cex =  . 4  
   )    
  ## Warning: 
## Grid searches over lambda (nugget and sill variances) with  minima at the endpoints: 
##   (REML) Restricted maximum likelihood 
##    minimum at  right endpoint  lambda  =  0.02118404 (eff. df= 26.59999 )
## Warning: 
## Grid searches over lambda (nugget and sill variances) with  minima at the endpoints: 
##   (REML) Restricted maximum likelihood 
##    minimum at  right endpoint  lambda  =  0.02118404 (eff. df= 26.59999 )
## Warning: 
## Grid searches over lambda (nugget and sill variances) with  minima at the endpoints: 
##   (REML) Restricted maximum likelihood 
##    minimum at  right endpoint  lambda  =  0.02118404 (eff. df= 26.59999 )
## Warning: 
## Grid searches over lambda (nugget and sill variances) with  minima at the endpoints: 
##   (REML) Restricted maximum likelihood 
##    minimum at  right endpoint  lambda  =  0.02118404 (eff. df= 26.59999 )
## Warning: 
## Grid searches over lambda (nugget and sill variances) with  minima at the endpoints: 
##   (REML) Restricted maximum likelihood 
##    minimum at  right endpoint  lambda  =  0.02118404 (eff. df= 26.59999 )  
       # after that, we need to add names of the clusters back onto this raster brick  
   Q_tibble2  <-  leak5  |>  
     dplyr ::  select ( 
        - pop,  - ind,  - index 
     ) 
    names (genoscape_brick)  <-   names (Q_tibble2)[]    
 
 
 3.5 Scaling and cleaning the genoscape_brick 
      genoscape_rgba  <-  genoscapeRtools ::  qprob_rando_raster ( 
      TRB =  genoscape_brick, 
      cols =  color_palette2, 
      alpha_scale =   2.0 , 
      abs_thresh =   0.0 , 
      alpha_exp =   1.55 , 
      alpha_chop_max =   255  
   ) 
    
    # This adds the info for a regular lat-long projection  
    crs (genoscape_rgba)  <-   "+proj=longlat +datum=WGS84 +no_defs +ellps=WGS84 +towgs84=0,0,0"     
 We can easily plot this with the function layer_spatial from the
ggspatial package: 
       ggplot ()  +   
     ggspatial ::  layer_spatial (genoscape_rgba)  +   
      my_theme ()  +  
      coord_sf ()    
 With pies 
       ggplot ()  +  
      layer_spatial (genoscape_rgba)  +  
      geom_spatial_point ( data =  long_lat_tibble, 
                         mapping =   aes ( x =  Long,  y =  Lat), 
                         size =  . 2 )  +  
      geom_text_repel ( 
        data =  df_mean, 
        aes ( x =  Longitude,  y =  Latitude,  label =  pop), 
        size =   3 , 
        box.padding =   unit ( 0.5 ,  "lines" ) 
     )  +  
      labs ( x =   "Longitude" , 
           y =   "Latitude" )  +  
      geom_scatterpie ( data =  df_mean,  
                      aes ( x =  Longitude,  y =  Latitude,  r =   1 ),  
                      cols =   c ( "v1" ,  "v2" ,  "v3" ,  "v4" ,  "v5" ),  color =   NA )  +  
      my_theme ()  +  
      scale_fill_manual ( values =  color_palette2)  +  
      guides ( fill =   "none" )  +    # Hide legend  
      coord_sf ()    
  ## Assuming `crs = 4326` in stat_spatial_identity()  
   
       # #   
    ggsave ( 
      here ( "output" ,  "populations" ,  "figures" ,  "lea_neutral_k5_interpolated_pie.pdf" ), 
      width  =   12 , 
      height =   6 , 
      units  =   "in" , 
      device =  cairo_pdf 
   )    
  ## Assuming `crs = 4326` in stat_spatial_identity()  
 
 
 
 4. LEA r2 0.01 k=5 
 Clear memory and environment 
       # Clear entire environment  
    rm ( list =   ls ()) 
    # Forcefully trigger garbage collection  
    gc ()    
  ##           used  (Mb) gc trigger  (Mb) limit (Mb) max used  (Mb)
## Ncells 4695813 250.8    8968841 479.0         NA  8968841 479.0
## Vcells 8787767  67.1   42297638 322.8      32768 68732428 524.4  
 
 4.1 Q-values 
      leak5  <-   read_delim ( 
      here ( "output" ,  "populations" ,  "snps_sets" ,  "r2_0.01.snmf" ,  "K5" ,  "run2" , "r2_0.01_r2.5.Q" ), 
      delim =   " " ,  # Specify the delimiter if different from the default (comma)  
      col_names =   FALSE , 
      show_col_types =   FALSE  
   )  
    # unseen_pckmeans.7.Q  
    # pckmeans.7.Q  
    head (leak5)    
  ## # A tibble: 6 × 5
##         X1      X2     X3       X4    X5
##      <dbl>   <dbl>  <dbl>    <dbl> <dbl>
## 1 0.0574   0.0290  0.240  0.0219   0.651
## 2 0.0936   0.0212  0.239  0.0460   0.600
## 3 0.0114   0.00895 0.156  0.0202   0.804
## 4 0.000100 0.0106  0.117  0.000920 0.872
## 5 0.000100 0.0174  0.0831 0.000100 0.899
## 6 0.000100 0.0305  0.154  0.0105   0.805  
 The fam file 
      fam_file  <-   here ( 
      "output" ,  "populations" ,  "snps_sets" ,  "r2_0.1.fam"  
   ) 
    
    # Read the .fam file  
   fam_data  <-   read.table (fam_file,  
                           header =   FALSE , 
                           col.names =   c ( "FamilyID" ,  "IndividualID" ,  "PaternalID" ,  "MaternalID" ,  "Sex" ,  "Phenotype" )) 
    
    # View the first few rows  
    head (fam_data)    
  ##   FamilyID IndividualID PaternalID MaternalID Sex Phenotype
## 1      OKI         1001          0          0   2        -9
## 2      OKI         1002          0          0   2        -9
## 3      OKI         1003          0          0   2        -9
## 4      OKI         1004          0          0   2        -9
## 5      OKI         1005          0          0   2        -9
## 6      OKI         1006          0          0   1        -9  
 Create ID column 
       # Change column name  
    colnames (fam_data)[ colnames (fam_data)  ==   "IndividualID" ]  <-   "ind"  
    
    # Change column name  
    colnames (fam_data)[ colnames (fam_data)  ==   "FamilyID" ]  <-   "pop"  
    
    # Select ID  
   fam_data  <-  fam_data  |>  
     dplyr ::  select ( "ind" ,  "pop" ) 
    
    # View the first few rows  
    head (fam_data)    
  ##    ind pop
## 1 1001 OKI
## 2 1002 OKI
## 3 1003 OKI
## 4 1004 OKI
## 5 1005 OKI
## 6 1006 OKI  
 Add it to matrix 
      leak5  <-  fam_data  |>  
     dplyr ::  select (ind, pop)  |>  
      bind_cols (leak5) 
    
    head (leak5)    
  ##    ind pop          X1         X2       X3          X4       X5
## 1 1001 OKI 0.057351200 0.02903100 0.240333 0.021857400 0.651427
## 2 1002 OKI 0.093595600 0.02123130 0.239314 0.045972700 0.599886
## 3 1003 OKI 0.011381600 0.00894785 0.155884 0.020222400 0.803564
## 4 1004 OKI 0.000099990 0.01060030 0.116732 0.000919858 0.871648
## 5 1005 OKI 0.000099982 0.01739940 0.083069 0.000099982 0.899332
## 6 1006 OKI 0.000099990 0.03047430 0.153502 0.010503400 0.805420  
 Rename the columns 
       # Rename the columns starting from the third one  
   leak5  <-  leak5  |>  
      rename_with ( ~  paste0 ( "v" ,  seq_along (.x)),  .cols =   -  c (ind, pop)) 
    
    # View the first few rows  
    head (leak5)    
  ##    ind pop          v1         v2       v3          v4       v5
## 1 1001 OKI 0.057351200 0.02903100 0.240333 0.021857400 0.651427
## 2 1002 OKI 0.093595600 0.02123130 0.239314 0.045972700 0.599886
## 3 1003 OKI 0.011381600 0.00894785 0.155884 0.020222400 0.803564
## 4 1004 OKI 0.000099990 0.01060030 0.116732 0.000919858 0.871648
## 5 1005 OKI 0.000099982 0.01739940 0.083069 0.000099982 0.899332
## 6 1006 OKI 0.000099990 0.03047430 0.153502 0.010503400 0.805420  
 Import samples attributes 
      sampling_loc  <-   readRDS ( here ( "output" ,  "populations" ,  "sampling_loc.rds" )) 
    # head(sampling_loc)  
    
   pops  <-  sampling_loc  |>  
      filter ( 
       Region  ==   "Asia"  
     )  |>  
     dplyr ::  select ( 
       Abbreviation, Latitude, Longitude, Pop_City, Country 
     ) 
    
    head (pops)    
  ## # A tibble: 6 × 5
##   Abbreviation Latitude Longitude Pop_City   Country 
##   <chr>           <dbl>     <dbl> <chr>      <chr>   
## 1 GEL              26.9      90.5 Gelephu    Bhutan  
## 2 CAM              11.6     105.  Phnom Penh Cambodia
## 3 HAI              19.2     110.  Hainan     China   
## 4 YUN              24.5     101.  Yunnan     China   
## 5 HUN              27.6     112.  Hunan      China   
## 6 BEN              13.0      77.6 Bengaluru  India  
 Merge with pops 
       # Add an index column to Q_tibble  
   leak5 $ index  <-   seq_len ( nrow (leak5)) 
    
    # Perform the merge as before  
   df1  <-  
      merge ( 
       leak5, 
       pops, 
        by.x =   2 , 
        by.y =   1 , 
        all.x =  T, 
        all.y =  F 
     )  |>  
      na.omit () 
    
    # Order by the index column to ensure the order matches the original Q_tibble  
   df1  <-  df1[ order (df1 $ index),] 
    
    # Optionally, you can remove the index column if it's no longer needed  
   df1 $ index  <-   NULL  
    
    # Now the rows of df1 should be in the same order as the original Q_tibble  
    head (df1)    
  ##     pop  ind          v1         v2       v3          v4       v5 Latitude
## 159 OKI 1001 0.057351200 0.02903100 0.240333 0.021857400 0.651427  26.5013
## 160 OKI 1002 0.093595600 0.02123130 0.239314 0.045972700 0.599886  26.5013
## 161 OKI 1003 0.011381600 0.00894785 0.155884 0.020222400 0.803564  26.5013
## 162 OKI 1004 0.000099990 0.01060030 0.116732 0.000919858 0.871648  26.5013
## 163 OKI 1005 0.000099982 0.01739940 0.083069 0.000099982 0.899332  26.5013
## 164 OKI 1006 0.000099990 0.03047430 0.153502 0.010503400 0.805420  26.5013
##     Longitude Pop_City Country
## 159  127.9454  Okinawa   Japan
## 160  127.9454  Okinawa   Japan
## 161  127.9454  Okinawa   Japan
## 162  127.9454  Okinawa   Japan
## 163  127.9454  Okinawa   Japan
## 164  127.9454  Okinawa   Japan  
 We used this color palette to make the “structure” plot 
      color_palette2  <-  
      c ( 
        "v1"   =   "#F49AC2" , 
        "v2"   =   "#FFFF99" , 
        "v3"   =   "#FFB347" , 
        "v4"   =   "red" , 
        "v5"   =   "#AE9393"  
     )    
 Make pie plot 
      world  <-   ne_countries ( scale =   "medium" ,  returnclass =   "sf" ) 
   countries_with_data  <-   unique (df1 $ Country) 
    
    # Filtering the world data to include only the countries in your data  
   selected_countries  <-  world  |>  
      filter (admin  %in%  countries_with_data) 
    
    # Calculate mean proportions for each population  
   df_mean  <-  df1  |>  
      group_by (pop)  |>  
      summarise ( across ( starts_with ( "v" ), \(x)  mean (x,  na.rm =   TRUE )),  
                Longitude =   mean (Longitude), 
                Latitude =   mean (Latitude)) 
    
    
    source ( 
      here ( 
        "scripts" ,  "analysis" ,  "my_theme2.R"  
     ) 
   ) 
    
    ggplot ()  +  
      geom_sf ( data =  selected_countries,  fill=  "white" )  +  
      geom_scatterpie ( data =  df_mean,  
                      aes ( x =  Longitude,  y =  Latitude,  r =   1.5 ),  
                      cols =   c ( "v1" ,  "v2" ,  "v3" ,  "v4" ,  "v5" ),  color =   NA )  +  
      geom_text_repel ( data =  df_mean, 
                      aes ( x =  Longitude,  y =  Latitude,  label =  pop),  
                      size =   3 ,  
                      box.padding =   unit ( 0.5 ,  "lines" ), 
                      max.overlaps =   50 )  +  
      scale_fill_manual ( values =  color_palette2)  +  
      guides ( fill =   "none" )  +    # Hide legend  
      # coord_sf() +  
      coord_sf ( xlim =   c ( 60 ,  150 ),  ylim =   c ( -  10 ,  60 ))  +  
      my_theme ()    
   
       # #   
    ggsave ( 
      here ( "output" ,  "populations" ,  "figures" ,  "lea_r2_0.01_k5_pie.pdf" ), 
      width  =   12 , 
      height =   6 , 
      units  =   "in" , 
      device =  cairo_pdf 
   )    
 
 
 4.2 Preparing the data for tess3Q_map_rasters 
 Make sure the lat longs are in the correct order and arrangement 
      df2  <-  df1  |>  
     dplyr ::  rename ( 
        Long =  Longitude, 
        Lat =  Latitude 
     ) 
    
   long_lat_tibble  <-  df2  |>  
     dplyr ::  select (Long, Lat) 
    
    
   long_lat_matrix  <-  long_lat_tibble  |>  
      as.matrix () 
    
    head (long_lat_matrix)    
  ##         Long     Lat
## 159 127.9454 26.5013
## 160 127.9454 26.5013
## 161 127.9454 26.5013
## 162 127.9454 26.5013
## 163 127.9454 26.5013
## 164 127.9454 26.5013  
 
 
 4.3 make a matrix of the Q values 
 Pull off the names of individuals and make a matrix of it: 
      Q_matrix  <-  leak5  |>  
     dplyr ::  select ( - ind,  - pop,  - index)  |>  
      as.matrix () 
    head (Q_matrix)    
  ##               v1         v2       v3          v4       v5
## [1,] 0.057351200 0.02903100 0.240333 0.021857400 0.651427
## [2,] 0.093595600 0.02123130 0.239314 0.045972700 0.599886
## [3,] 0.011381600 0.00894785 0.155884 0.020222400 0.803564
## [4,] 0.000099990 0.01060030 0.116732 0.000919858 0.871648
## [5,] 0.000099982 0.01739940 0.083069 0.000099982 0.899332
## [6,] 0.000099990 0.03047430 0.153502 0.010503400 0.805420  
 
 
 4.4 Interpolate the Q-values by Kriging 
       print ( ncol (Q_matrix)  ==   length (color_palette2))    
  ## [1] TRUE  
 Create brick 
      genoscape_brick  <-  tess3r ::  tess3Q_map_rasters ( 
      x =  Q_matrix,  
      coord =  long_lat_matrix,   
      map.polygon =  selected_countries, 
      window =   extent (selected_countries)[ 1  :  4 ], 
      # window = combined_extent,  
      resolution =   c ( 600 , 600 ),  # if you want more cells in your raster, set higher  
      # this next lines need to to be here, but don't do much...  
      col.palette =  tess3r ::  CreatePalette (color_palette2,  length (color_palette2)), 
      method =   "map.max" ,  
      interpol =  tess3r ::  FieldsKrigModel ( 80 ),   
      main =   "Ancestry coefficients" , 
      xlab =   "Longitude" ,  
      ylab =   "Latitude" ,  
      cex =  . 4  
   )    
  ## Warning: 
## Grid searches over lambda (nugget and sill variances) with  minima at the endpoints: 
##   (REML) Restricted maximum likelihood 
##    minimum at  right endpoint  lambda  =  0.02118404 (eff. df= 26.59999 )
## Warning: 
## Grid searches over lambda (nugget and sill variances) with  minima at the endpoints: 
##   (REML) Restricted maximum likelihood 
##    minimum at  right endpoint  lambda  =  0.02118404 (eff. df= 26.59999 )
## Warning: 
## Grid searches over lambda (nugget and sill variances) with  minima at the endpoints: 
##   (REML) Restricted maximum likelihood 
##    minimum at  right endpoint  lambda  =  0.02118404 (eff. df= 26.59999 )
## Warning: 
## Grid searches over lambda (nugget and sill variances) with  minima at the endpoints: 
##   (REML) Restricted maximum likelihood 
##    minimum at  right endpoint  lambda  =  0.02118404 (eff. df= 26.59999 )
## Warning: 
## Grid searches over lambda (nugget and sill variances) with  minima at the endpoints: 
##   (REML) Restricted maximum likelihood 
##    minimum at  right endpoint  lambda  =  0.02118404 (eff. df= 26.59999 )  
       # after that, we need to add names of the clusters back onto this raster brick  
   Q_tibble2  <-  leak5  |>  
     dplyr ::  select ( 
        - pop,  - ind,  - index 
     ) 
    names (genoscape_brick)  <-   names (Q_tibble2)[]    
 
 
 4.5 Scaling and cleaning the genoscape_brick 
      genoscape_rgba  <-  genoscapeRtools ::  qprob_rando_raster ( 
      TRB =  genoscape_brick, 
      cols =  color_palette2, 
      alpha_scale =   2.0 , 
      abs_thresh =   0.0 , 
      alpha_exp =   1.55 , 
      alpha_chop_max =   255  
   ) 
    
    # This adds the info for a regular lat-long projection  
    crs (genoscape_rgba)  <-   "+proj=longlat +datum=WGS84 +no_defs +ellps=WGS84 +towgs84=0,0,0"     
 We can easily plot this with the function layer_spatial from the
ggspatial package: 
       ggplot ()  +   
     ggspatial ::  layer_spatial (genoscape_rgba)  +   
      my_theme ()  +  
      coord_sf ()    
 With pies 
       ggplot ()  +  
      layer_spatial (genoscape_rgba)  +  
      geom_spatial_point ( data =  long_lat_tibble, 
                         mapping =   aes ( x =  Long,  y =  Lat), 
                         size =  . 2 )  +  
      geom_text_repel ( 
        data =  df_mean, 
        aes ( x =  Longitude,  y =  Latitude,  label =  pop), 
        size =   3 , 
        box.padding =   unit ( 0.5 ,  "lines" ) 
     )  +  
      labs ( x =   "Longitude" , 
           y =   "Latitude" )  +  
      geom_scatterpie ( data =  df_mean,  
                      aes ( x =  Longitude,  y =  Latitude,  r =   1 ),  
                      cols =   c ( "v1" ,  "v2" ,  "v3" ,  "v4" ,  "v5" ),  color =   NA )  +  
      my_theme ()  +  
      scale_fill_manual ( values =  color_palette2)  +  
      guides ( fill =   "none" )  +    # Hide legend  
      coord_sf ()    
  ## Assuming `crs = 4326` in stat_spatial_identity()  
   
       # #   
    ggsave ( 
      here ( "output" ,  "populations" ,  "figures" ,  "lea_r2_0.01_k5_interpolated_pie.pdf" ), 
      width  =   12 , 
      height =   6 , 
      units  =   "in" , 
      device =  cairo_pdf 
   )    
  ## Assuming `crs = 4326` in stat_spatial_identity()  
 
 
 
 5. LEA r2 0.1 k=5 
 Clear memory and environment 
       # Clear entire environment  
    rm ( list =   ls ()) 
    # Forcefully trigger garbage collection  
    gc ()    
  ##           used  (Mb) gc trigger  (Mb) limit (Mb) max used  (Mb)
## Ncells 4696100 250.8    8968841 479.0         NA  8968841 479.0
## Vcells 8789418  67.1   40868549 311.9      32768 68732428 524.4  
 
 5.1 Q-values 
       # Extract ancestry coefficients  
   leak5  <-   read_delim ( 
      here ( "output" ,  "populations" ,  "snps_sets" ,  "r2_0.1.snmf" ,  "K5" ,  "run5" , "r2_0.1_r5.5.Q" ), 
      delim =   " " ,  # Specify the delimiter if different from the default (comma)  
      col_names =   FALSE , 
      show_col_types =   FALSE  
   )  
    # unseen_pckmeans.7.Q  
    # pckmeans.7.Q  
    head (leak5)    
  ## # A tibble: 6 × 5
##        X1     X2    X3       X4    X5
##     <dbl>  <dbl> <dbl>    <dbl> <dbl>
## 1 0.00901 0.134  0.601 0.0199   0.236
## 2 0.0206  0.155  0.568 0.0289   0.227
## 3 0.0175  0.103  0.711 0.000100 0.169
## 4 0.00882 0.0695 0.777 0.0136   0.131
## 5 0.00145 0.0634 0.808 0.0136   0.113
## 6 0.0128  0.104  0.723 0.000100 0.160  
 The fam file 
      fam_file  <-   here ( 
      "output" ,  "populations" ,  "snps_sets" ,  "r2_0.01.fam"  
   ) 
    
    # Read the .fam file  
   fam_data  <-   read.table (fam_file,  
                           header =   FALSE , 
                           col.names =   c ( "FamilyID" ,  "IndividualID" ,  "PaternalID" ,  "MaternalID" ,  "Sex" ,  "Phenotype" )) 
    
    # View the first few rows  
    head (fam_data)    
  ##   FamilyID IndividualID PaternalID MaternalID Sex Phenotype
## 1      OKI         1001          0          0   2        -9
## 2      OKI         1002          0          0   2        -9
## 3      OKI         1003          0          0   2        -9
## 4      OKI         1004          0          0   2        -9
## 5      OKI         1005          0          0   2        -9
## 6      OKI         1006          0          0   1        -9  
 Create ID column 
       # Change column name  
    colnames (fam_data)[ colnames (fam_data)  ==   "IndividualID" ]  <-   "ind"  
    
    # Change column name  
    colnames (fam_data)[ colnames (fam_data)  ==   "FamilyID" ]  <-   "pop"  
    
    # Select ID  
   fam_data  <-  fam_data  |>  
     dplyr ::  select ( "ind" ,  "pop" ) 
    
    # View the first few rows  
    head (fam_data)    
  ##    ind pop
## 1 1001 OKI
## 2 1002 OKI
## 3 1003 OKI
## 4 1004 OKI
## 5 1005 OKI
## 6 1006 OKI  
 Add it to matrix 
      leak5  <-  fam_data  |>  
     dplyr ::  select (ind, pop)  |>  
      bind_cols (leak5) 
    
    head (leak5)    
  ##    ind pop         X1        X2       X3          X4       X5
## 1 1001 OKI 0.00901176 0.1344870 0.600824 0.019856000 0.235822
## 2 1002 OKI 0.02059050 0.1549340 0.568124 0.028927700 0.227423
## 3 1003 OKI 0.01745480 0.1030830 0.710789 0.000099991 0.168573
## 4 1004 OKI 0.00881626 0.0694981 0.777402 0.013613100 0.130670
## 5 1005 OKI 0.00145040 0.0633980 0.808295 0.013615700 0.113241
## 6 1006 OKI 0.01277640 0.1043520 0.723096 0.000099991 0.159675  
 Rename the columns 
       # Rename the columns starting from the third one  
   leak5  <-  leak5  |>  
      rename_with ( ~  paste0 ( "v" ,  seq_along (.x)),  .cols =   -  c (ind, pop)) 
    
    # View the first few rows  
    head (leak5)    
  ##    ind pop         v1        v2       v3          v4       v5
## 1 1001 OKI 0.00901176 0.1344870 0.600824 0.019856000 0.235822
## 2 1002 OKI 0.02059050 0.1549340 0.568124 0.028927700 0.227423
## 3 1003 OKI 0.01745480 0.1030830 0.710789 0.000099991 0.168573
## 4 1004 OKI 0.00881626 0.0694981 0.777402 0.013613100 0.130670
## 5 1005 OKI 0.00145040 0.0633980 0.808295 0.013615700 0.113241
## 6 1006 OKI 0.01277640 0.1043520 0.723096 0.000099991 0.159675  
 Import samples attributes 
      sampling_loc  <-   readRDS ( here ( "output" ,  "populations" ,  "sampling_loc.rds" )) 
    # head(sampling_loc)  
    
   pops  <-  sampling_loc  |>  
      filter ( 
       Region  ==   "Asia"  
     )  |>  
     dplyr ::  select ( 
       Abbreviation, Latitude, Longitude, Pop_City, Country 
     ) 
    
    head (pops)    
  ## # A tibble: 6 × 5
##   Abbreviation Latitude Longitude Pop_City   Country 
##   <chr>           <dbl>     <dbl> <chr>      <chr>   
## 1 GEL              26.9      90.5 Gelephu    Bhutan  
## 2 CAM              11.6     105.  Phnom Penh Cambodia
## 3 HAI              19.2     110.  Hainan     China   
## 4 YUN              24.5     101.  Yunnan     China   
## 5 HUN              27.6     112.  Hunan      China   
## 6 BEN              13.0      77.6 Bengaluru  India  
 Merge with pops 
       # Add an index column to Q_tibble  
   leak5 $ index  <-   seq_len ( nrow (leak5)) 
    
    # Perform the merge as before  
   df1  <-  
      merge ( 
       leak5, 
       pops, 
        by.x =   2 , 
        by.y =   1 , 
        all.x =  T, 
        all.y =  F 
     )  |>  
      na.omit () 
    
    # Order by the index column to ensure the order matches the original Q_tibble  
   df1  <-  df1[ order (df1 $ index),] 
    
    # Optionally, you can remove the index column if it's no longer needed  
   df1 $ index  <-   NULL  
    
    # Now the rows of df1 should be in the same order as the original Q_tibble  
    head (df1)    
  ##     pop  ind         v1        v2       v3          v4       v5 Latitude
## 159 OKI 1001 0.00901176 0.1344870 0.600824 0.019856000 0.235822  26.5013
## 160 OKI 1002 0.02059050 0.1549340 0.568124 0.028927700 0.227423  26.5013
## 161 OKI 1003 0.01745480 0.1030830 0.710789 0.000099991 0.168573  26.5013
## 162 OKI 1004 0.00881626 0.0694981 0.777402 0.013613100 0.130670  26.5013
## 163 OKI 1005 0.00145040 0.0633980 0.808295 0.013615700 0.113241  26.5013
## 164 OKI 1006 0.01277640 0.1043520 0.723096 0.000099991 0.159675  26.5013
##     Longitude Pop_City Country
## 159  127.9454  Okinawa   Japan
## 160  127.9454  Okinawa   Japan
## 161  127.9454  Okinawa   Japan
## 162  127.9454  Okinawa   Japan
## 163  127.9454  Okinawa   Japan
## 164  127.9454  Okinawa   Japan  
 We used this color palette to make the “structure” plot 
      color_palette2  <-  
      c ( 
        "v1"   =   "red" , 
        "v2"   =   "#F49AC2" , 
        "v3"   =   "#AE9393" , 
        "v4"   =   "#FFFF99" , 
        "v5"   =   "#FFB347"  
     )    
 Make pie plot 
      world  <-   ne_countries ( scale =   "medium" ,  returnclass =   "sf" ) 
   countries_with_data  <-   unique (df1 $ Country) 
    
    # Filtering the world data to include only the countries in your data  
   selected_countries  <-  world  |>  
      filter (admin  %in%  countries_with_data) 
    
    # Calculate mean proportions for each population  
   df_mean  <-  df1  |>  
      group_by (pop)  |>  
      summarise ( across ( starts_with ( "v" ), \(x)  mean (x,  na.rm =   TRUE )),  
                Longitude =   mean (Longitude), 
                Latitude =   mean (Latitude)) 
    
    
    source ( 
      here ( 
        "scripts" ,  "analysis" ,  "my_theme2.R"  
     ) 
   ) 
    
    ggplot ()  +  
      geom_sf ( data =  selected_countries,  fill=  "white" )  +  
      geom_scatterpie ( data =  df_mean,  
                      aes ( x =  Longitude,  y =  Latitude,  r =   1.5 ),  
                      cols =   c ( "v1" ,  "v2" ,  "v3" ,  "v4" ,  "v5" ),  color =   NA )  +  
      geom_text_repel ( data =  df_mean, 
                      aes ( x =  Longitude,  y =  Latitude,  label =  pop),  
                      size =   3 ,  
                      box.padding =   unit ( 0.5 ,  "lines" ), 
                      max.overlaps =   50 )  +  
      scale_fill_manual ( values =  color_palette2)  +  
      guides ( fill =   "none" )  +    # Hide legend  
      # coord_sf() +  
      coord_sf ( xlim =   c ( 60 ,  150 ),  ylim =   c ( -  10 ,  60 ))  +  
      my_theme ()    
   
       # #   
    ggsave ( 
      here ( "output" ,  "populations" ,  "figures" ,  "lea_r2_0.1_k5_pie.pdf" ), 
      width  =   12 , 
      height =   6 , 
      units  =   "in" , 
      device =  cairo_pdf 
   )    
 
 
 5.2 Preparing the data for tess3Q_map_rasters 
      df2  <-  df1  |>  
     dplyr ::  rename ( 
        Long =  Longitude, 
        Lat =  Latitude 
     ) 
    
   long_lat_tibble  <-  df2  |>  
     dplyr ::  select (Long, Lat) 
    
    
   long_lat_matrix  <-  long_lat_tibble  |>  
      as.matrix () 
    
    head (long_lat_matrix)    
  ##         Long     Lat
## 159 127.9454 26.5013
## 160 127.9454 26.5013
## 161 127.9454 26.5013
## 162 127.9454 26.5013
## 163 127.9454 26.5013
## 164 127.9454 26.5013  
 
 
 5.3 make a matrix of the Q values 
 Pull off the names of individuals and make a matrix of it: 
      Q_matrix  <-  leak5  |>  
     dplyr ::  select ( - ind,  - pop,  - index)  |>  
      as.matrix () 
    head (Q_matrix)    
  ##              v1        v2       v3          v4       v5
## [1,] 0.00901176 0.1344870 0.600824 0.019856000 0.235822
## [2,] 0.02059050 0.1549340 0.568124 0.028927700 0.227423
## [3,] 0.01745480 0.1030830 0.710789 0.000099991 0.168573
## [4,] 0.00881626 0.0694981 0.777402 0.013613100 0.130670
## [5,] 0.00145040 0.0633980 0.808295 0.013615700 0.113241
## [6,] 0.01277640 0.1043520 0.723096 0.000099991 0.159675  
 
 
 5.4 Interpolate the Q-values by Kriging 
       print ( ncol (Q_matrix)  ==   length (color_palette2))    
  ## [1] TRUE  
 Create brick 
      genoscape_brick  <-  tess3r ::  tess3Q_map_rasters ( 
      x =  Q_matrix,  
      coord =  long_lat_matrix,   
      map.polygon =  selected_countries, 
      window =   extent (selected_countries)[ 1  :  4 ], 
      # window = combined_extent,  
      resolution =   c ( 600 , 600 ),  # if you want more cells in your raster, set higher  
      # this next lines need to to be here, but don't do much...  
      col.palette =  tess3r ::  CreatePalette (color_palette2,  length (color_palette2)), 
      method =   "map.max" ,  
      interpol =  tess3r ::  FieldsKrigModel ( 80 ),   
      main =   "Ancestry coefficients" , 
      xlab =   "Longitude" ,  
      ylab =   "Latitude" ,  
      cex =  . 4  
   )    
  ## Warning: 
## Grid searches over lambda (nugget and sill variances) with  minima at the endpoints: 
##   (REML) Restricted maximum likelihood 
##    minimum at  right endpoint  lambda  =  0.02118404 (eff. df= 26.59999 )
## Warning: 
## Grid searches over lambda (nugget and sill variances) with  minima at the endpoints: 
##   (REML) Restricted maximum likelihood 
##    minimum at  right endpoint  lambda  =  0.02118404 (eff. df= 26.59999 )
## Warning: 
## Grid searches over lambda (nugget and sill variances) with  minima at the endpoints: 
##   (REML) Restricted maximum likelihood 
##    minimum at  right endpoint  lambda  =  0.02118404 (eff. df= 26.59999 )
## Warning: 
## Grid searches over lambda (nugget and sill variances) with  minima at the endpoints: 
##   (REML) Restricted maximum likelihood 
##    minimum at  right endpoint  lambda  =  0.02118404 (eff. df= 26.59999 )
## Warning: 
## Grid searches over lambda (nugget and sill variances) with  minima at the endpoints: 
##   (REML) Restricted maximum likelihood 
##    minimum at  right endpoint  lambda  =  0.02118404 (eff. df= 26.59999 )  
       # after that, we need to add names of the clusters back onto this raster brick  
   Q_tibble2  <-  leak5  |>  
     dplyr ::  select ( 
        - pop,  - ind,  - index 
     ) 
    names (genoscape_brick)  <-   names (Q_tibble2)[]    
 
 
 5.5 Scaling and cleaning the genoscape_brick 
      genoscape_rgba  <-  genoscapeRtools ::  qprob_rando_raster ( 
      TRB =  genoscape_brick, 
      cols =  color_palette2, 
      alpha_scale =   2.0 , 
      abs_thresh =   0.0 , 
      alpha_exp =   1.55 , 
      alpha_chop_max =   255  
   ) 
    
    # This adds the info for a regular lat-long projection  
    crs (genoscape_rgba)  <-   "+proj=longlat +datum=WGS84 +no_defs +ellps=WGS84 +towgs84=0,0,0"     
 We can easily plot this with the function layer_spatial from the
ggspatial package: 
       ggplot ()  +   
     ggspatial ::  layer_spatial (genoscape_rgba)  +   
      my_theme ()  +  
      coord_sf ()    
 With pies 
       ggplot ()  +  
      layer_spatial (genoscape_rgba)  +  
      geom_spatial_point ( data =  long_lat_tibble, 
                         mapping =   aes ( x =  Long,  y =  Lat), 
                         size =  . 2 )  +  
      geom_text_repel ( 
        data =  df_mean, 
        aes ( x =  Longitude,  y =  Latitude,  label =  pop), 
        size =   3 , 
        box.padding =   unit ( 0.5 ,  "lines" ) 
     )  +  
      labs ( x =   "Longitude" , 
           y =   "Latitude" )  +  
      geom_scatterpie ( data =  df_mean,  
                      aes ( x =  Longitude,  y =  Latitude,  r =   1 ),  
                      cols =   c ( "v1" ,  "v2" ,  "v3" ,  "v4" ,  "v5" ),  color =   NA )  +  
      my_theme ()  +  
      scale_fill_manual ( values =  color_palette2)  +  
      guides ( fill =   "none" )  +    # Hide legend  
      coord_sf ()    
  ## Assuming `crs = 4326` in stat_spatial_identity()  
   
       # #   
    ggsave ( 
      here ( "output" ,  "populations" ,  "figures" ,  "lea_r2_0.1_k5_interpolated_pie.pdf" ), 
      width  =   12 , 
      height =   6 , 
      units  =   "in" , 
      device =  cairo_pdf 
   )    
  ## Assuming `crs = 4326` in stat_spatial_identity()  
 
 
 
 6. fastStructure neutral SNPs simple prior 
 Clear memory and environment 
       # Clear entire environment  
    rm ( list =   ls ()) 
    # Forcefully trigger garbage collection  
    gc ()    
  ##           used  (Mb) gc trigger  (Mb) limit (Mb) max used  (Mb)
## Ncells 4696390 250.9   12802251 683.8         NA  8968841 479.0
## Vcells 8791073  67.1   39349927 300.3      32768 68732428 524.4  
 Make plot 
       # Extract ancestry coefficients  
   k5run1  <-   read_delim ( 
      here ( "output" ,  "populations" ,  "faststructure" ,  "neutral" ,  "run01" ,  "simple.5.meanQ" ), 
      delim =   "  " ,  # Specify the delimiter if different from the default (comma)  
      col_names =   FALSE , 
      show_col_types =   FALSE  
   )  
    
    head (k5run1)    
  ## # A tibble: 6 × 5
##      X1       X2       X3    X4       X5
##   <dbl>    <dbl>    <dbl> <dbl>    <dbl>
## 1 0.561 0.000012 0.000012 0.439 0.000012
## 2 0.479 0.109    0.000012 0.412 0.000012
## 3 0.575 0.000012 0.000012 0.425 0.000012
## 4 0.527 0.000012 0.000012 0.473 0.000012
## 5 0.527 0.000012 0.000012 0.473 0.000012
## 6 0.561 0.000012 0.000012 0.438 0.000012  
 The fam file 
      fam_file  <-   here ( 
      "output" ,  "populations" ,  "snps_sets" ,  "neutral.fam"  
   ) 
    
    # Read the .fam file  
   fam_data  <-   read.table (fam_file,  
                           header =   FALSE , 
                           col.names =   c ( "FamilyID" ,  "IndividualID" ,  "PaternalID" ,  "MaternalID" ,  "Sex" ,  "Phenotype" )) 
    
    # View the first few rows  
    head (fam_data)    
  ##   FamilyID IndividualID PaternalID MaternalID Sex Phenotype
## 1      OKI         1001          0          0   2        -9
## 2      OKI         1002          0          0   2        -9
## 3      OKI         1003          0          0   2        -9
## 4      OKI         1004          0          0   2        -9
## 5      OKI         1005          0          0   2        -9
## 6      OKI         1006          0          0   1        -9  
 Create ID column 
       # Change column name  
    colnames (fam_data)[ colnames (fam_data)  ==   "IndividualID" ]  <-   "ind"  
    
    
    # Change column name  
    colnames (fam_data)[ colnames (fam_data)  ==   "FamilyID" ]  <-   "pop"  
    
    # Select ID  
   fam_data  <-  fam_data  |>  
     dplyr ::  select ( "ind" ,  "pop" ) 
    
    # View the first few rows  
    head (fam_data)    
  ##    ind pop
## 1 1001 OKI
## 2 1002 OKI
## 3 1003 OKI
## 4 1004 OKI
## 5 1005 OKI
## 6 1006 OKI  
 Add it to matrix 
      k5run1  <-  fam_data  |>  
     dplyr ::  select (ind, pop)  |>  
      bind_cols (k5run1) 
    
    head (k5run1)    
  ##    ind pop       X1       X2      X3       X4      X5
## 1 1001 OKI 0.560940 0.000012 1.2e-05 0.439025 1.2e-05
## 2 1002 OKI 0.479157 0.109106 1.2e-05 0.411713 1.2e-05
## 3 1003 OKI 0.574956 0.000012 1.2e-05 0.425009 1.2e-05
## 4 1004 OKI 0.526625 0.000012 1.2e-05 0.473340 1.2e-05
## 5 1005 OKI 0.526835 0.000012 1.2e-05 0.473130 1.2e-05
## 6 1006 OKI 0.561466 0.000012 1.2e-05 0.438499 1.2e-05  
 Rename the columns 
       # Rename the columns starting from the third one  
   k5run1  <-  k5run1  |>  
      rename_with ( ~  paste0 ( "v" ,  seq_along (.x)),  .cols =   -  c (ind, pop)) 
    
    # View the first few rows  
    head (k5run1)    
  ##    ind pop       v1       v2      v3       v4      v5
## 1 1001 OKI 0.560940 0.000012 1.2e-05 0.439025 1.2e-05
## 2 1002 OKI 0.479157 0.109106 1.2e-05 0.411713 1.2e-05
## 3 1003 OKI 0.574956 0.000012 1.2e-05 0.425009 1.2e-05
## 4 1004 OKI 0.526625 0.000012 1.2e-05 0.473340 1.2e-05
## 5 1005 OKI 0.526835 0.000012 1.2e-05 0.473130 1.2e-05
## 6 1006 OKI 0.561466 0.000012 1.2e-05 0.438499 1.2e-05  
 Import samples attributes 
      sampling_loc  <-   readRDS ( here ( "output" ,  "populations" ,  "sampling_loc.rds" )) 
    # head(sampling_loc)  
    
   pops  <-  sampling_loc  |>  
      filter ( 
       Region  ==   "Asia"  
     )  |>  
     dplyr ::  select ( 
       Abbreviation, Latitude, Longitude, Pop_City, Country 
     ) 
    
    head (pops)    
  ## # A tibble: 6 × 5
##   Abbreviation Latitude Longitude Pop_City   Country 
##   <chr>           <dbl>     <dbl> <chr>      <chr>   
## 1 GEL              26.9      90.5 Gelephu    Bhutan  
## 2 CAM              11.6     105.  Phnom Penh Cambodia
## 3 HAI              19.2     110.  Hainan     China   
## 4 YUN              24.5     101.  Yunnan     China   
## 5 HUN              27.6     112.  Hunan      China   
## 6 BEN              13.0      77.6 Bengaluru  India  
       # Add an index column to Q_tibble  
   k5run1 $ index  <-   seq_len ( nrow (k5run1)) 
    
    # Perform the merge as before  
   df1  <-  
      merge ( 
       k5run1, 
       pops, 
        by.x =   2 , 
        by.y =   1 , 
        all.x =  T, 
        all.y =  F 
     )  |>  
      na.omit () 
    
    # Order by the index column to ensure the order matches the original Q_tibble  
   df1  <-  df1[ order (df1 $ index),] 
    
    # Optionally, you can remove the index column if it's no longer needed  
   df1 $ index  <-   NULL  
    
    # Now the rows of df1 should be in the same order as the original Q_tibble  
    head (df1)    
  ##     pop  ind       v1       v2      v3       v4      v5 Latitude Longitude
## 159 OKI 1001 0.560940 0.000012 1.2e-05 0.439025 1.2e-05  26.5013  127.9454
## 160 OKI 1002 0.479157 0.109106 1.2e-05 0.411713 1.2e-05  26.5013  127.9454
## 161 OKI 1003 0.574956 0.000012 1.2e-05 0.425009 1.2e-05  26.5013  127.9454
## 162 OKI 1004 0.526625 0.000012 1.2e-05 0.473340 1.2e-05  26.5013  127.9454
## 163 OKI 1005 0.526835 0.000012 1.2e-05 0.473130 1.2e-05  26.5013  127.9454
## 164 OKI 1006 0.561466 0.000012 1.2e-05 0.438499 1.2e-05  26.5013  127.9454
##     Pop_City Country
## 159  Okinawa   Japan
## 160  Okinawa   Japan
## 161  Okinawa   Japan
## 162  Okinawa   Japan
## 163  Okinawa   Japan
## 164  Okinawa   Japan  
 We used this color palette to make the “structure” plot 
      color_palette2  <-  
      c ( 
        "v1"   =   "#FFB347" , 
        "v2"   =   "#AE9393" , 
        "v3"   =   "#FFFF99" , 
        "v4"   =   "#F49AC2" , 
        "v5"   =   "red"  
     )    
      world  <-   ne_countries ( scale =   "medium" ,  returnclass =   "sf" ) 
   countries_with_data  <-   unique (df1 $ Country) 
    
    # Filtering the world data to include only the countries in your data  
   selected_countries  <-  world  |>  
      filter (admin  %in%  countries_with_data) 
    
    # Calculate mean proportions for each population  
   df_mean  <-  df1  |>  
      group_by (pop)  |>  
      summarise ( across ( starts_with ( "v" ), \(x)  mean (x,  na.rm =   TRUE )),  
                Longitude =   mean (Longitude), 
                Latitude =   mean (Latitude)) 
    
    
    source ( 
      here ( 
        "scripts" ,  "analysis" ,  "my_theme2.R"  
     ) 
   ) 
    
    ggplot ()  +  
      geom_sf ( data =  selected_countries,  fill=  "white" )  +  
      geom_scatterpie ( data =  df_mean,  
                      aes ( x =  Longitude,  y =  Latitude,  r =   1.5 ),  
                      cols =   c ( "v1" ,  "v2" ,  "v3" ,  "v4" ,  "v5" ),  color =   NA )  +  
      geom_text_repel ( data =  df_mean, 
                      aes ( x =  Longitude,  y =  Latitude,  label =  pop),  
                      size =   3 ,  
                      box.padding =   unit ( 0.5 ,  "lines" ), 
                      max.overlaps =   50 )  +  
      scale_fill_manual ( values =  color_palette2)  +  
      guides ( fill =   "none" )  +    # Hide legend  
      # coord_sf() +  
      coord_sf ( xlim =   c ( 60 ,  150 ),  ylim =   c ( -  10 ,  60 ))  +  
      my_theme ()    
   
       # #   
    ggsave ( 
      here ( "output" ,  "populations" ,  "figures" ,  "fastStructure_neutral_simple_k5_pie.pdf" ), 
      width  =   12 , 
      height =   6 , 
      units  =   "in" , 
      device =  cairo_pdf 
   )    
 
 6.1 Preparing the data for tess3Q_map_rasters 
 Make sure the lat longs are in the correct order and arrangement 
      df2  <-  df1  |>  
     dplyr ::  rename ( 
        Long =  Longitude, 
        Lat =  Latitude 
     ) 
    
   long_lat_tibble  <-  df2  |>  
     dplyr ::  select (Long, Lat) 
    
    
   long_lat_matrix  <-  long_lat_tibble  |>  
      as.matrix () 
    
    head (long_lat_matrix)    
  ##         Long     Lat
## 159 127.9454 26.5013
## 160 127.9454 26.5013
## 161 127.9454 26.5013
## 162 127.9454 26.5013
## 163 127.9454 26.5013
## 164 127.9454 26.5013  
 
 
 6.2 make a matrix of the Q values 
 Pull off the names of individuals and make a matrix of it: 
      Q_matrix  <-  k5run1  |>  
     dplyr ::  select ( - ind,  - pop,  - index)  |>  
      as.matrix () 
    head (Q_matrix)    
  ##            v1       v2      v3       v4      v5
## [1,] 0.560940 0.000012 1.2e-05 0.439025 1.2e-05
## [2,] 0.479157 0.109106 1.2e-05 0.411713 1.2e-05
## [3,] 0.574956 0.000012 1.2e-05 0.425009 1.2e-05
## [4,] 0.526625 0.000012 1.2e-05 0.473340 1.2e-05
## [5,] 0.526835 0.000012 1.2e-05 0.473130 1.2e-05
## [6,] 0.561466 0.000012 1.2e-05 0.438499 1.2e-05  
 
 
 6.3 Interpolate the Q-values by Kriging 
       print ( ncol (Q_matrix)  ==   length (color_palette2))    
  ## [1] TRUE  
 Create brick 
      genoscape_brick  <-  tess3r ::  tess3Q_map_rasters ( 
      x =  Q_matrix,  
      coord =  long_lat_matrix,   
      map.polygon =  selected_countries, 
      window =   extent (selected_countries)[ 1  :  4 ], 
      # window = combined_extent,  
      resolution =   c ( 600 , 600 ),  # if you want more cells in your raster, set higher  
      # this next lines need to to be here, but don't do much...  
      col.palette =  tess3r ::  CreatePalette (color_palette2,  length (color_palette2)), 
      method =   "map.max" ,  
      interpol =  tess3r ::  FieldsKrigModel ( 40 ),   
      main =   "Ancestry coefficients" , 
      xlab =   "Longitude" ,  
      ylab =   "Latitude" ,  
      cex =  . 4  
   )    
  ## Warning: 
## Grid searches over lambda (nugget and sill variances) with  minima at the endpoints: 
##   (REML) Restricted maximum likelihood 
##    minimum at  right endpoint  lambda  =  0.04206056 (eff. df= 26.60001 )
## Warning: 
## Grid searches over lambda (nugget and sill variances) with  minima at the endpoints: 
##   (REML) Restricted maximum likelihood 
##    minimum at  right endpoint  lambda  =  0.04206056 (eff. df= 26.60001 )
## Warning: 
## Grid searches over lambda (nugget and sill variances) with  minima at the endpoints: 
##   (REML) Restricted maximum likelihood 
##    minimum at  right endpoint  lambda  =  0.04206056 (eff. df= 26.60001 )
## Warning: 
## Grid searches over lambda (nugget and sill variances) with  minima at the endpoints: 
##   (REML) Restricted maximum likelihood 
##    minimum at  right endpoint  lambda  =  0.04206056 (eff. df= 26.60001 )  
       # after that, we need to add names of the clusters back onto this raster brick  
   Q_tibble2  <-  k5run1  |>  
     dplyr ::  select ( 
        - pop,  - ind,  - index 
     ) 
    names (genoscape_brick)  <-   names (Q_tibble2)[]    
 
 
 6.4 Scaling and cleaning the genoscape_brick 
      genoscape_rgba  <-  genoscapeRtools ::  qprob_rando_raster ( 
      TRB =  genoscape_brick, 
      cols =  color_palette2, 
      alpha_scale =   2.0 , 
      abs_thresh =   0.0 , 
      alpha_exp =   1.55 , 
      alpha_chop_max =   255  
   ) 
    
    crs (genoscape_rgba)  <-   "+proj=longlat +datum=WGS84 +no_defs +ellps=WGS84 +towgs84=0,0,0"     
 With pies 
       ggplot ()  +  
      layer_spatial (genoscape_rgba)  +  
      geom_spatial_point ( data =  long_lat_tibble, 
                         mapping =   aes ( x =  Long,  y =  Lat), 
                         size =  . 2 )  +  
      geom_text_repel ( 
        data =  df_mean, 
        aes ( x =  Longitude,  y =  Latitude,  label =  pop), 
        size =   3 , 
        box.padding =   unit ( 0.5 ,  "lines" ) 
     )  +  
      labs ( x =   "Longitude" , 
           y =   "Latitude" )  +  
      geom_scatterpie ( 
        data =  df_mean, 
        aes ( x =  Longitude,  y =  Latitude,  r =   1 ), 
        cols =   c ( "v1" ,  "v2" ,  "v3" ,  "v4" ,  "v5" ), 
        color =   NA  
     )  +  
      my_theme ()  +  
      scale_fill_manual ( values =  color_palette2)  +  
      guides ( fill =   "none" )  +    # Hide legend  
      coord_sf ()    
  ## Assuming `crs = 4326` in stat_spatial_identity()  
   
       ggsave ( 
      here ( "output" ,  "populations" ,  "figures" ,  "fastStructure_neutral_simple_k5_interpolated_pie.pdf" ), 
      width  =   12 , 
      height =   6 , 
      units  =   "in" , 
      device =  cairo_pdf 
   )    
  ## Assuming `crs = 4326` in stat_spatial_identity()  
 
 
 
 7. fastStructure neutral SNPs logistic prior 
 Clear memory and environment 
       # Clear entire environment  
    rm ( list =   ls ()) 
    # Forcefully trigger garbage collection  
    gc ()    
  ##           used  (Mb) gc trigger  (Mb) limit (Mb) max used  (Mb)
## Ncells 4696653 250.9   12802251 683.8         NA 12802251 683.8
## Vcells 8792780  67.1   45471916 347.0      32768 68732428 524.4  
 Make plot 
       # Extract ancestry coefficients  
   k5run1  <-   read_delim ( 
      here ( "output" ,  "populations" ,  "faststructure" ,  "neutral" ,  "run02" ,  "logistic.5.meanQ" ), 
      delim =   "  " ,  # Specify the delimiter if different from the default (comma)  
      col_names =   FALSE , 
      show_col_types =   FALSE  
   )  
    
    head (k5run1)    
  ## # A tibble: 6 × 5
##         X1       X2    X3       X4       X5
##      <dbl>    <dbl> <dbl>    <dbl>    <dbl>
## 1 0.000012 0.000012 1.00  0.000183 0.000035
## 2 0.872    0.000012 0.128 0.000012 0.000012
## 3 0.000012 0.000063 1.00  0.000014 0.000012
## 4 0.000012 0.000012 1.00  0.000011 0.000015
## 5 0.000012 0.000034 1.00  0.000029 0.000012
## 6 0.000012 0.000013 1.00  0.000046 0.000012  
 The fam file 
      fam_file  <-   here ( 
      "output" ,  "populations" ,  "snps_sets" ,  "neutral.fam"  
   ) 
    
    # Read the .fam file  
   fam_data  <-   read.table (fam_file,  
                           header =   FALSE , 
                           col.names =   c ( "FamilyID" ,  "IndividualID" ,  "PaternalID" ,  "MaternalID" ,  "Sex" ,  "Phenotype" )) 
    
    # View the first few rows  
    head (fam_data)    
  ##   FamilyID IndividualID PaternalID MaternalID Sex Phenotype
## 1      OKI         1001          0          0   2        -9
## 2      OKI         1002          0          0   2        -9
## 3      OKI         1003          0          0   2        -9
## 4      OKI         1004          0          0   2        -9
## 5      OKI         1005          0          0   2        -9
## 6      OKI         1006          0          0   1        -9  
 Create ID column 
       # Change column name  
    colnames (fam_data)[ colnames (fam_data)  ==   "IndividualID" ]  <-   "ind"  
    
    # Change column name  
    colnames (fam_data)[ colnames (fam_data)  ==   "FamilyID" ]  <-   "pop"  
    
    # Select ID  
   fam_data  <-  fam_data  |>  
     dplyr ::  select ( "ind" ,  "pop" ) 
    
    # View the first few rows  
    head (fam_data)    
  ##    ind pop
## 1 1001 OKI
## 2 1002 OKI
## 3 1003 OKI
## 4 1004 OKI
## 5 1005 OKI
## 6 1006 OKI  
 Add it to matrix 
      k5run1  <-  fam_data  |>  
     dplyr ::  select (ind, pop)  |>  
      bind_cols (k5run1) 
    
    head (k5run1)    
  ##    ind pop       X1      X2       X3       X4      X5
## 1 1001 OKI 0.000012 1.2e-05 0.999760 0.000183 3.5e-05
## 2 1002 OKI 0.871549 1.2e-05 0.128416 0.000012 1.2e-05
## 3 1003 OKI 0.000012 6.3e-05 0.999899 0.000014 1.2e-05
## 4 1004 OKI 0.000012 1.2e-05 0.999950 0.000011 1.5e-05
## 5 1005 OKI 0.000012 3.4e-05 0.999913 0.000029 1.2e-05
## 6 1006 OKI 0.000012 1.3e-05 0.999918 0.000046 1.2e-05  
 Rename the columns 
       # Rename the columns starting from the third one  
   k5run1  <-  k5run1  |>  
      rename_with ( ~  paste0 ( "v" ,  seq_along (.x)),  .cols =   -  c (ind, pop)) 
    
    # View the first few rows  
    head (k5run1)    
  ##    ind pop       v1      v2       v3       v4      v5
## 1 1001 OKI 0.000012 1.2e-05 0.999760 0.000183 3.5e-05
## 2 1002 OKI 0.871549 1.2e-05 0.128416 0.000012 1.2e-05
## 3 1003 OKI 0.000012 6.3e-05 0.999899 0.000014 1.2e-05
## 4 1004 OKI 0.000012 1.2e-05 0.999950 0.000011 1.5e-05
## 5 1005 OKI 0.000012 3.4e-05 0.999913 0.000029 1.2e-05
## 6 1006 OKI 0.000012 1.3e-05 0.999918 0.000046 1.2e-05  
 Import samples attributes 
      sampling_loc  <-   readRDS ( here ( "output" ,  "populations" ,  "sampling_loc.rds" )) 
    # head(sampling_loc)  
    
   pops  <-  sampling_loc  |>  
      filter ( 
       Region  ==   "Asia"  
     )  |>  
     dplyr ::  select ( 
       Abbreviation, Latitude, Longitude, Pop_City, Country 
     ) 
    
    head (pops)    
  ## # A tibble: 6 × 5
##   Abbreviation Latitude Longitude Pop_City   Country 
##   <chr>           <dbl>     <dbl> <chr>      <chr>   
## 1 GEL              26.9      90.5 Gelephu    Bhutan  
## 2 CAM              11.6     105.  Phnom Penh Cambodia
## 3 HAI              19.2     110.  Hainan     China   
## 4 YUN              24.5     101.  Yunnan     China   
## 5 HUN              27.6     112.  Hunan      China   
## 6 BEN              13.0      77.6 Bengaluru  India  
       # Add an index column to Q_tibble  
   k5run1 $ index  <-   seq_len ( nrow (k5run1)) 
    
    # Perform the merge as before  
   df1  <-  
      merge ( 
       k5run1, 
       pops, 
        by.x =   2 , 
        by.y =   1 , 
        all.x =  T, 
        all.y =  F 
     )  |>  
      na.omit () 
    
    # Order by the index column to ensure the order matches the original Q_tibble  
   df1  <-  df1[ order (df1 $ index),] 
    
    # Optionally, you can remove the index column if it's no longer needed  
   df1 $ index  <-   NULL  
    
    # Now the rows of df1 should be in the same order as the original Q_tibble  
    head (df1)    
  ##     pop  ind       v1      v2       v3       v4      v5 Latitude Longitude
## 159 OKI 1001 0.000012 1.2e-05 0.999760 0.000183 3.5e-05  26.5013  127.9454
## 160 OKI 1002 0.871549 1.2e-05 0.128416 0.000012 1.2e-05  26.5013  127.9454
## 161 OKI 1003 0.000012 6.3e-05 0.999899 0.000014 1.2e-05  26.5013  127.9454
## 162 OKI 1004 0.000012 1.2e-05 0.999950 0.000011 1.5e-05  26.5013  127.9454
## 163 OKI 1005 0.000012 3.4e-05 0.999913 0.000029 1.2e-05  26.5013  127.9454
## 164 OKI 1006 0.000012 1.3e-05 0.999918 0.000046 1.2e-05  26.5013  127.9454
##     Pop_City Country
## 159  Okinawa   Japan
## 160  Okinawa   Japan
## 161  Okinawa   Japan
## 162  Okinawa   Japan
## 163  Okinawa   Japan
## 164  Okinawa   Japan  
 We used this color palette to make the “structure” plot 
      color_palette2  <-  
      c ( 
        "v1"   =   "#AE9393" , 
        "v2"   =   "red" , 
        "v3"   =   "#FFFF99" , 
        "v4"   =   "#F49AC2" , 
        "v5"   =   "#FFB347"  #,  
        # "v6" = "#008080",  
        # "v7" = "#FFFF99"  
     )    
      world  <-   ne_countries ( scale =   "medium" ,  returnclass =   "sf" ) 
   countries_with_data  <-   unique (df1 $ Country) 
    
    # Filtering the world data to include only the countries in your data  
   selected_countries  <-  world  |>  
      filter (admin  %in%  countries_with_data) 
    
    # Calculate mean proportions for each population  
   df_mean  <-  df1  |>  
      group_by (pop)  |>  
      summarise ( across ( starts_with ( "v" ), \(x)  mean (x,  na.rm =   TRUE )),  
                Longitude =   mean (Longitude), 
                Latitude =   mean (Latitude)) 
    
    
    source ( 
      here ( 
        "scripts" ,  "analysis" ,  "my_theme2.R"  
     ) 
   ) 
    
    ggplot ()  +  
      geom_sf ( data =  selected_countries,  fill=  "white" )  +  
      geom_scatterpie ( data =  df_mean,  
                      aes ( x =  Longitude,  y =  Latitude,  r =   1.5 ),  
                      cols =   c ( "v1" ,  "v2" ,  "v3" ,  "v4" ,  "v5" ),  color =   NA )  +  
      geom_text_repel ( data =  df_mean, 
                      aes ( x =  Longitude,  y =  Latitude,  label =  pop),  
                      size =   3 ,  
                      box.padding =   unit ( 0.5 ,  "lines" ), 
                      max.overlaps =   50 )  +  
      scale_fill_manual ( values =  color_palette2)  +  
      guides ( fill =   "none" )  +    # Hide legend  
      # coord_sf() +  
      coord_sf ( xlim =   c ( 60 ,  150 ),  ylim =   c ( -  10 ,  60 ))  +  
      my_theme ()    
   
       # #   
    ggsave ( 
      here ( "output" ,  "populations" ,  "figures" ,  "fastStructure_neutral_logistic_k5_pie.pdf" ), 
      width  =   12 , 
      height =   6 , 
      units  =   "in" , 
      device =  cairo_pdf 
   )    
 
 7.1 Preparing the data for tess3Q_map_rasters 
      df2  <-  df1  |>  
     dplyr ::  rename ( 
        Long =  Longitude, 
        Lat =  Latitude 
     ) 
    
   long_lat_tibble  <-  df2  |>  
     dplyr ::  select (Long, Lat) 
    
    
   long_lat_matrix  <-  long_lat_tibble  |>  
      as.matrix () 
    
    head (long_lat_matrix)    
  ##         Long     Lat
## 159 127.9454 26.5013
## 160 127.9454 26.5013
## 161 127.9454 26.5013
## 162 127.9454 26.5013
## 163 127.9454 26.5013
## 164 127.9454 26.5013  
 
 
 7.2 make a matrix of the Q values 
 Pull off the names of individuals and make a matrix 
      Q_matrix  <-  k5run1  |>  
     dplyr ::  select ( - ind,  - pop,  - index)  |>  
      as.matrix () 
    head (Q_matrix)    
  ##            v1      v2       v3       v4      v5
## [1,] 0.000012 1.2e-05 0.999760 0.000183 3.5e-05
## [2,] 0.871549 1.2e-05 0.128416 0.000012 1.2e-05
## [3,] 0.000012 6.3e-05 0.999899 0.000014 1.2e-05
## [4,] 0.000012 1.2e-05 0.999950 0.000011 1.5e-05
## [5,] 0.000012 3.4e-05 0.999913 0.000029 1.2e-05
## [6,] 0.000012 1.3e-05 0.999918 0.000046 1.2e-05  
 
 
 7.3 Interpolate the Q-values by Kriging 
       print ( ncol (Q_matrix)  ==   length (color_palette2))    
  ## [1] TRUE  
 Create brick 
      genoscape_brick  <-  tess3r ::  tess3Q_map_rasters ( 
      x =  Q_matrix,  
      coord =  long_lat_matrix,   
      map.polygon =  selected_countries, 
      window =   extent (selected_countries)[ 1  :  4 ], 
      # window = combined_extent,  
      resolution =   c ( 600 , 600 ),  # if you want more cells in your raster, set higher  
      # this next lines need to to be here, but don't do much...  
      col.palette =  tess3r ::  CreatePalette (color_palette2,  length (color_palette2)), 
      method =   "map.max" ,  
      interpol =  tess3r ::  FieldsKrigModel ( 40 ),   
      main =   "Ancestry coefficients" , 
      xlab =   "Longitude" ,  
      ylab =   "Latitude" ,  
      cex =  . 4  
   )    
  ## Warning: 
## Grid searches over lambda (nugget and sill variances) with  minima at the endpoints: 
##   (REML) Restricted maximum likelihood 
##    minimum at  right endpoint  lambda  =  0.04206056 (eff. df= 26.60001 )
## Warning: 
## Grid searches over lambda (nugget and sill variances) with  minima at the endpoints: 
##   (REML) Restricted maximum likelihood 
##    minimum at  right endpoint  lambda  =  0.04206056 (eff. df= 26.60001 )
## Warning: 
## Grid searches over lambda (nugget and sill variances) with  minima at the endpoints: 
##   (REML) Restricted maximum likelihood 
##    minimum at  right endpoint  lambda  =  0.04206056 (eff. df= 26.60001 )  
       # after that, we need to add names of the clusters back onto this raster brick  
   Q_tibble2  <-  k5run1  |>  
     dplyr ::  select ( 
        - pop,  - ind,  - index 
     ) 
    names (genoscape_brick)  <-   names (Q_tibble2)[]    
 
 
 7.4 Scaling and cleaning the genoscape_brick 
      genoscape_rgba  <-  genoscapeRtools ::  qprob_rando_raster ( 
      TRB =  genoscape_brick, 
      cols =  color_palette2, 
      alpha_scale =   2.0 , 
      abs_thresh =   0.0 , 
      alpha_exp =   1.55 , 
      alpha_chop_max =   255  
   ) 
    
    crs (genoscape_rgba)  <-   "+proj=longlat +datum=WGS84 +no_defs +ellps=WGS84 +towgs84=0,0,0"     
 We can easily plot this with the function layer_spatial from the
ggspatial package: 
       ggplot ()  +   
     ggspatial ::  layer_spatial (genoscape_rgba)  +   
      my_theme ()  +  
      coord_sf ()    
 Plot 
       ggplot ()  +  
      layer_spatial (genoscape_rgba)  +  
      geom_spatial_point ( data =  long_lat_tibble, 
                         mapping =   aes ( x =  Long,  y =  Lat), 
                         size =  . 2 )  +  
      geom_text_repel ( 
        data =  df_mean, 
        aes ( x =  Longitude,  y =  Latitude,  label =  pop), 
        size =   3 , 
        box.padding =   unit ( 0.5 ,  "lines" ) 
     )  +  
      labs ( x =   "Longitude" , 
           y =   "Latitude" )  +  
      geom_scatterpie ( 
        data =  df_mean, 
        aes ( x =  Longitude,  y =  Latitude,  r =   1 ), 
        cols =   c ( "v1" ,  "v2" ,  "v3" ,  "v4" ,  "v5" ), 
        color =   NA  
     )  +  
      my_theme ()  +  
      scale_fill_manual ( values =  color_palette2)  +  
      guides ( fill =   "none" )  +    # Hide legend  
      coord_sf ()    
  ## Assuming `crs = 4326` in stat_spatial_identity()  
   
       ggsave ( 
      here ( "output" ,  "populations" ,  "figures" ,  "fastStructure_neutral_logistic_k5_interpolated_pie.pdf" ), 
      width  =   12 , 
      height =   6 , 
      units  =   "in" , 
      device =  cairo_pdf 
   )    
  ## Assuming `crs = 4326` in stat_spatial_identity()  
 
 
 
 8. fastStructure r2 0.01 SNPs simple prior 
 Clear memory and environment 
       # Clear entire environment  
    rm ( list =   ls ()) 
    # Forcefully trigger garbage collection  
    gc ()    
  ##           used  (Mb) gc trigger  (Mb) limit (Mb) max used  (Mb)
## Ncells 4696934 250.9   12802251 683.8         NA 12802251 683.8
## Vcells 8794491  67.1   43717040 333.6      32768 68732428 524.4  
 Make plot 
       # Extract ancestry coefficients  
   k5run1  <-   read_delim ( 
      here ( "output" ,  "populations" ,  "faststructure" ,  "r2_0.01" ,  "run1" ,  "simple.5.meanQ" ), 
      delim =   "  " ,  # Specify the delimiter if different from the default (comma)  
      col_names =   FALSE , 
      show_col_types =   FALSE  
   )  
    
    head (k5run1)    
  ## # A tibble: 6 × 5
##         X1     X2       X3    X4       X5
##      <dbl>  <dbl>    <dbl> <dbl>    <dbl>
## 1 0.0926   0.274  0.000005 0.633 0.000005
## 2 0.126    0.245  0.000005 0.629 0.000005
## 3 0.0385   0.172  0.000005 0.789 0.000005
## 4 0.000005 0.123  0.000005 0.877 0.000005
## 5 0.000005 0.0852 0.000005 0.915 0.000005
## 6 0.000005 0.188  0.000005 0.800 0.0128  
 The fam file 
      fam_file  <-   here ( 
      "output" ,  "populations" ,  "snps_sets" ,  "r2_0.01.fam"  
   ) 
    
    # Read the .fam file  
   fam_data  <-   read.table (fam_file,  
                           header =   FALSE , 
                           col.names =   c ( "FamilyID" ,  "IndividualID" ,  "PaternalID" ,  "MaternalID" ,  "Sex" ,  "Phenotype" )) 
    
    # View the first few rows  
    head (fam_data)    
  ##   FamilyID IndividualID PaternalID MaternalID Sex Phenotype
## 1      OKI         1001          0          0   2        -9
## 2      OKI         1002          0          0   2        -9
## 3      OKI         1003          0          0   2        -9
## 4      OKI         1004          0          0   2        -9
## 5      OKI         1005          0          0   2        -9
## 6      OKI         1006          0          0   1        -9  
 Create ID column 
       # Change column name  
    colnames (fam_data)[ colnames (fam_data)  ==   "IndividualID" ]  <-   "ind"  
    
    
    # Merge columns "FamilyID" and "IndividualID" with an underscore  
    # fam_data$ind <- paste(fam_data$FamilyID, fam_data$IndividualID, sep = "_")  
    
    
    # Change column name  
    colnames (fam_data)[ colnames (fam_data)  ==   "FamilyID" ]  <-   "pop"  
    
    # Select ID  
   fam_data  <-  fam_data  |>  
     dplyr ::  select ( "ind" ,  "pop" ) 
    
    # View the first few rows  
    head (fam_data)    
  ##    ind pop
## 1 1001 OKI
## 2 1002 OKI
## 3 1003 OKI
## 4 1004 OKI
## 5 1005 OKI
## 6 1006 OKI  
 Add it to matrix 
      k5run1  <-  fam_data  |>  
     dplyr ::  select (ind, pop)  |>  
      bind_cols (k5run1) 
    
    head (k5run1)    
  ##    ind pop       X1       X2    X3       X4       X5
## 1 1001 OKI 0.092564 0.274411 5e-06 0.633015 0.000005
## 2 1002 OKI 0.125992 0.244922 5e-06 0.629076 0.000005
## 3 1003 OKI 0.038454 0.172095 5e-06 0.789440 0.000005
## 4 1004 OKI 0.000005 0.123287 5e-06 0.876698 0.000005
## 5 1005 OKI 0.000005 0.085162 5e-06 0.914823 0.000005
## 6 1006 OKI 0.000005 0.187602 5e-06 0.799554 0.012834  
 Rename the columns 
       # Rename the columns starting from the third one  
   k5run1  <-  k5run1  |>  
      rename_with ( ~  paste0 ( "v" ,  seq_along (.x)),  .cols =   -  c (ind, pop)) 
    
    # View the first few rows  
    head (k5run1)    
  ##    ind pop       v1       v2    v3       v4       v5
## 1 1001 OKI 0.092564 0.274411 5e-06 0.633015 0.000005
## 2 1002 OKI 0.125992 0.244922 5e-06 0.629076 0.000005
## 3 1003 OKI 0.038454 0.172095 5e-06 0.789440 0.000005
## 4 1004 OKI 0.000005 0.123287 5e-06 0.876698 0.000005
## 5 1005 OKI 0.000005 0.085162 5e-06 0.914823 0.000005
## 6 1006 OKI 0.000005 0.187602 5e-06 0.799554 0.012834  
 Import samples attributes 
      sampling_loc  <-   readRDS ( here ( "output" ,  "populations" ,  "sampling_loc.rds" )) 
    # head(sampling_loc)  
    
   pops  <-  sampling_loc  |>  
      filter ( 
       Region  ==   "Asia"  
     )  |>  
     dplyr ::  select ( 
       Abbreviation, Latitude, Longitude, Pop_City, Country 
     ) 
    
    head (pops)    
  ## # A tibble: 6 × 5
##   Abbreviation Latitude Longitude Pop_City   Country 
##   <chr>           <dbl>     <dbl> <chr>      <chr>   
## 1 GEL              26.9      90.5 Gelephu    Bhutan  
## 2 CAM              11.6     105.  Phnom Penh Cambodia
## 3 HAI              19.2     110.  Hainan     China   
## 4 YUN              24.5     101.  Yunnan     China   
## 5 HUN              27.6     112.  Hunan      China   
## 6 BEN              13.0      77.6 Bengaluru  India  
       # Add an index column to Q_tibble  
   k5run1 $ index  <-   seq_len ( nrow (k5run1)) 
    
    # Perform the merge as before  
   df1  <-  
      merge ( 
       k5run1, 
       pops, 
        by.x =   2 , 
        by.y =   1 , 
        all.x =  T, 
        all.y =  F 
     )  |>  
      na.omit () 
    
    # Order by the index column to ensure the order matches the original Q_tibble  
   df1  <-  df1[ order (df1 $ index),] 
    
    # Optionally, you can remove the index column if it's no longer needed  
   df1 $ index  <-   NULL  
    
    # Now the rows of df1 should be in the same order as the original Q_tibble  
    head (df1)    
  ##     pop  ind       v1       v2    v3       v4       v5 Latitude Longitude
## 159 OKI 1001 0.092564 0.274411 5e-06 0.633015 0.000005  26.5013  127.9454
## 160 OKI 1002 0.125992 0.244922 5e-06 0.629076 0.000005  26.5013  127.9454
## 161 OKI 1003 0.038454 0.172095 5e-06 0.789440 0.000005  26.5013  127.9454
## 162 OKI 1004 0.000005 0.123287 5e-06 0.876698 0.000005  26.5013  127.9454
## 163 OKI 1005 0.000005 0.085162 5e-06 0.914823 0.000005  26.5013  127.9454
## 164 OKI 1006 0.000005 0.187602 5e-06 0.799554 0.012834  26.5013  127.9454
##     Pop_City Country
## 159  Okinawa   Japan
## 160  Okinawa   Japan
## 161  Okinawa   Japan
## 162  Okinawa   Japan
## 163  Okinawa   Japan
## 164  Okinawa   Japan  
 We used this color palette to make the “structure” plot 
      color_palette2  <-  
      c ( 
        "v1"   =   "#F49AC2" , 
        "v2"   =   "#FFB347" , 
        "v3"   =   "#FFFF99" , 
        "v4"   =   "#AE9393" , 
        "v5"   =   "red"  #,  
        # "v6" = "#008080",  
        # "v7" = "#FFFF99"  
     )    
 Plot 
      world  <-   ne_countries ( scale =   "medium" ,  returnclass =   "sf" ) 
   countries_with_data  <-   unique (df1 $ Country) 
    
    # Filtering the world data to include only the countries in your data  
   selected_countries  <-  world  |>  
      filter (admin  %in%  countries_with_data) 
    
    # Calculate mean proportions for each population  
   df_mean  <-  df1  |>  
      group_by (pop)  |>  
      summarise ( across ( starts_with ( "v" ), \(x)  mean (x,  na.rm =   TRUE )),  
                Longitude =   mean (Longitude), 
                Latitude =   mean (Latitude)) 
    
    
    source ( 
      here ( 
        "scripts" ,  "analysis" ,  "my_theme2.R"  
     ) 
   ) 
    
    ggplot ()  +  
      geom_sf ( data =  selected_countries,  fill=  "white" )  +  
      geom_scatterpie ( data =  df_mean,  
                      aes ( x =  Longitude,  y =  Latitude,  r =   1.5 ),  
                      cols =   c ( "v1" ,  "v2" ,  "v3" ,  "v4" ,  "v5" ),  color =   NA )  +  
      geom_text_repel ( data =  df_mean, 
                      aes ( x =  Longitude,  y =  Latitude,  label =  pop),  
                      size =   3 ,  
                      box.padding =   unit ( 0.5 ,  "lines" ), 
                      max.overlaps =   50 )  +  
      scale_fill_manual ( values =  color_palette2)  +  
      guides ( fill =   "none" )  +    # Hide legend  
      # coord_sf() +  
      coord_sf ( xlim =   c ( 60 ,  150 ),  ylim =   c ( -  10 ,  60 ))  +  
      my_theme ()    
   
       # #   
    ggsave ( 
      here ( "output" ,  "populations" ,  "figures" ,  "fastStructure_r2_0.01_simple_k5_pie.pdf" ), 
      width  =   12 , 
      height =   6 , 
      units  =   "in" , 
      device =  cairo_pdf 
   )    
 
 8.1 Preparing the data for tess3Q_map_rasters 
      df2  <-  df1  |>  
     dplyr ::  rename ( 
        Long =  Longitude, 
        Lat =  Latitude 
     ) 
    
   long_lat_tibble  <-  df2  |>  
     dplyr ::  select (Long, Lat) 
    
    
   long_lat_matrix  <-  long_lat_tibble  |>  
      as.matrix () 
    
    head (long_lat_matrix)    
  ##         Long     Lat
## 159 127.9454 26.5013
## 160 127.9454 26.5013
## 161 127.9454 26.5013
## 162 127.9454 26.5013
## 163 127.9454 26.5013
## 164 127.9454 26.5013  
 
 
 8.2 make a matrix of the Q values 
      Q_matrix  <-  k5run1  |>  
     dplyr ::  select ( - ind,  - pop,  - index)  |>  
      as.matrix () 
    head (Q_matrix)    
  ##            v1       v2    v3       v4       v5
## [1,] 0.092564 0.274411 5e-06 0.633015 0.000005
## [2,] 0.125992 0.244922 5e-06 0.629076 0.000005
## [3,] 0.038454 0.172095 5e-06 0.789440 0.000005
## [4,] 0.000005 0.123287 5e-06 0.876698 0.000005
## [5,] 0.000005 0.085162 5e-06 0.914823 0.000005
## [6,] 0.000005 0.187602 5e-06 0.799554 0.012834  
 
 
 8.3 Interpolate the Q-values by Kriging 
       print ( ncol (Q_matrix)  ==   length (color_palette2))    
  ## [1] TRUE  
 Create brick 
      genoscape_brick  <-  tess3r ::  tess3Q_map_rasters ( 
      x =  Q_matrix,  
      coord =  long_lat_matrix,   
      map.polygon =  selected_countries, 
      window =   extent (selected_countries)[ 1  :  4 ], 
      # window = combined_extent,  
      resolution =   c ( 600 , 600 ),  # if you want more cells in your raster, set higher  
      # this next lines need to to be here, but don't do much...  
      col.palette =  tess3r ::  CreatePalette (color_palette2,  length (color_palette2)), 
      method =   "map.max" ,  
      interpol =  tess3r ::  FieldsKrigModel ( 40 ),   
      main =   "Ancestry coefficients" , 
      xlab =   "Longitude" ,  
      ylab =   "Latitude" ,  
      cex =  . 4  
   )    
  ## Warning: 
## Grid searches over lambda (nugget and sill variances) with  minima at the endpoints: 
##   (REML) Restricted maximum likelihood 
##    minimum at  right endpoint  lambda  =  0.04206056 (eff. df= 26.60001 )
## Warning: 
## Grid searches over lambda (nugget and sill variances) with  minima at the endpoints: 
##   (REML) Restricted maximum likelihood 
##    minimum at  right endpoint  lambda  =  0.04206056 (eff. df= 26.60001 )
## Warning: 
## Grid searches over lambda (nugget and sill variances) with  minima at the endpoints: 
##   (REML) Restricted maximum likelihood 
##    minimum at  right endpoint  lambda  =  0.04206056 (eff. df= 26.60001 )
## Warning: 
## Grid searches over lambda (nugget and sill variances) with  minima at the endpoints: 
##   (REML) Restricted maximum likelihood 
##    minimum at  right endpoint  lambda  =  0.04206056 (eff. df= 26.60001 )
## Warning: 
## Grid searches over lambda (nugget and sill variances) with  minima at the endpoints: 
##   (REML) Restricted maximum likelihood 
##    minimum at  right endpoint  lambda  =  0.04206056 (eff. df= 26.60001 )  
       # after that, we need to add names of the clusters back onto this raster brick  
   Q_tibble2  <-  k5run1  |>  
     dplyr ::  select ( 
        - pop,  - ind,  - index 
     ) 
    names (genoscape_brick)  <-   names (Q_tibble2)[]    
 
 
 8.4 Scaling and cleaning the genoscape_brick 
      genoscape_rgba  <-  genoscapeRtools ::  qprob_rando_raster ( 
      TRB =  genoscape_brick, 
      cols =  color_palette2, 
      alpha_scale =   2.0 , 
      abs_thresh =   0.0 , 
      alpha_exp =   1.55 , 
      alpha_chop_max =   255  
   ) 
    
    crs (genoscape_rgba)  <-   "+proj=longlat +datum=WGS84 +no_defs +ellps=WGS84 +towgs84=0,0,0"     
       ggplot ()  +   
     ggspatial ::  layer_spatial (genoscape_rgba)  +   
      my_theme ()  +  
      coord_sf ()    
 Plot 
       ggplot ()  +  
      layer_spatial (genoscape_rgba)  +  
      geom_spatial_point ( data =  long_lat_tibble, 
                         mapping =   aes ( x =  Long,  y =  Lat), 
                         size =  . 2 )  +  
      geom_text_repel ( 
        data =  df_mean, 
        aes ( x =  Longitude,  y =  Latitude,  label =  pop), 
        size =   3 , 
        box.padding =   unit ( 0.5 ,  "lines" ) 
     )  +  
      labs ( x =   "Longitude" , 
           y =   "Latitude" )  +  
      geom_scatterpie ( 
        data =  df_mean, 
        aes ( x =  Longitude,  y =  Latitude,  r =   1 ), 
        cols =   c ( "v1" ,  "v2" ,  "v3" ,  "v4" ,  "v5" ), 
        color =   NA  
     )  +  
      my_theme ()  +  
      scale_fill_manual ( values =  color_palette2)  +  
      guides ( fill =   "none" )  +    # Hide legend  
      coord_sf ()    
  ## Assuming `crs = 4326` in stat_spatial_identity()  
   
       ggsave ( 
      here ( "output" ,  "populations" ,  "figures" ,  "fastStructure_r2_0.01_simple_k5_interpolated_pie.pdf" ), 
      width  =   12 , 
      height =   6 , 
      units  =   "in" , 
      device =  cairo_pdf 
   )    
  ## Assuming `crs = 4326` in stat_spatial_identity()  
 
 
 
 9. fastStructure r2 0.01 SNPs logistic prior 
 Clear memory and environment 
       # Clear entire environment  
    rm ( list =   ls ()) 
    # Forcefully trigger garbage collection  
    gc ()    
  ##           used  (Mb) gc trigger  (Mb) limit (Mb) max used  (Mb)
## Ncells 4697216 250.9   12802251 683.8         NA 12802251 683.8
## Vcells 8796347  67.2   40492156 309.0      32768 68732428 524.4  
 Make plot 
       # Extract ancestry coefficients  
   k5run1  <-   read_delim ( 
      here ( "output" ,  "populations" ,  "faststructure" ,  "r2_0.01" ,  "run1" ,  "logistic.5.meanQ" ), 
      delim =   "  " ,  # Specify the delimiter if different from the default (comma)  
      col_names =   FALSE , 
      show_col_types =   FALSE  
   )  
    
    head (k5run1)    
  ## # A tibble: 6 × 5
##         X1       X2       X3    X4       X5
##      <dbl>    <dbl>    <dbl> <dbl>    <dbl>
## 1 0.000005 0.000005 0.000005  1.00 0.000005
## 2 0.000005 0.000005 0.000005  1.00 0.000005
## 3 0.000005 0.000005 0.000005  1.00 0.000005
## 4 0.000005 0.000005 0.000005  1.00 0.000005
## 5 0.000005 0.000005 0.000005  1.00 0.000005
## 6 0.000005 0.000005 0.000005  1.00 0.000005  
 The fam file 
      fam_file  <-   here ( 
      "output" ,  "populations" ,  "snps_sets" ,  "r2_0.01.fam"  
   ) 
    
    # Read the .fam file  
   fam_data  <-   read.table (fam_file,  
                           header =   FALSE , 
                           col.names =   c ( "FamilyID" ,  "IndividualID" ,  "PaternalID" ,  "MaternalID" ,  "Sex" ,  "Phenotype" )) 
    
    # View the first few rows  
    head (fam_data)    
  ##   FamilyID IndividualID PaternalID MaternalID Sex Phenotype
## 1      OKI         1001          0          0   2        -9
## 2      OKI         1002          0          0   2        -9
## 3      OKI         1003          0          0   2        -9
## 4      OKI         1004          0          0   2        -9
## 5      OKI         1005          0          0   2        -9
## 6      OKI         1006          0          0   1        -9  
 Create ID column 
       # Change column name  
    colnames (fam_data)[ colnames (fam_data)  ==   "IndividualID" ]  <-   "ind"  
    
    
    # Merge columns "FamilyID" and "IndividualID" with an underscore  
    # fam_data$ind <- paste(fam_data$FamilyID, fam_data$IndividualID, sep = "_")  
    
    
    # Change column name  
    colnames (fam_data)[ colnames (fam_data)  ==   "FamilyID" ]  <-   "pop"  
    
    # Select ID  
   fam_data  <-  fam_data  |>  
     dplyr ::  select ( "ind" ,  "pop" ) 
    
    # View the first few rows  
    head (fam_data)    
  ##    ind pop
## 1 1001 OKI
## 2 1002 OKI
## 3 1003 OKI
## 4 1004 OKI
## 5 1005 OKI
## 6 1006 OKI  
 Add it to matrix 
      k5run1  <-  fam_data  |>  
     dplyr ::  select (ind, pop)  |>  
      bind_cols (k5run1) 
    
    head (k5run1)    
  ##    ind pop    X1    X2    X3      X4    X5
## 1 1001 OKI 5e-06 5e-06 5e-06 0.99998 5e-06
## 2 1002 OKI 5e-06 5e-06 5e-06 0.99998 5e-06
## 3 1003 OKI 5e-06 5e-06 5e-06 0.99998 5e-06
## 4 1004 OKI 5e-06 5e-06 5e-06 0.99998 5e-06
## 5 1005 OKI 5e-06 5e-06 5e-06 0.99998 5e-06
## 6 1006 OKI 5e-06 5e-06 5e-06 0.99998 5e-06  
 Rename the columns 
       # Rename the columns starting from the third one  
   k5run1  <-  k5run1  |>  
      rename_with ( ~  paste0 ( "v" ,  seq_along (.x)),  .cols =   -  c (ind, pop)) 
    
    # View the first few rows  
    head (k5run1)    
  ##    ind pop    v1    v2    v3      v4    v5
## 1 1001 OKI 5e-06 5e-06 5e-06 0.99998 5e-06
## 2 1002 OKI 5e-06 5e-06 5e-06 0.99998 5e-06
## 3 1003 OKI 5e-06 5e-06 5e-06 0.99998 5e-06
## 4 1004 OKI 5e-06 5e-06 5e-06 0.99998 5e-06
## 5 1005 OKI 5e-06 5e-06 5e-06 0.99998 5e-06
## 6 1006 OKI 5e-06 5e-06 5e-06 0.99998 5e-06  
 Import samples attributes 
      sampling_loc  <-   readRDS ( here ( "output" ,  "populations" ,  "sampling_loc.rds" )) 
    # head(sampling_loc)  
    
   pops  <-  sampling_loc  |>  
      filter ( 
       Region  ==   "Asia"  
     )  |>  
     dplyr ::  select ( 
       Abbreviation, Latitude, Longitude, Pop_City, Country 
     ) 
    
    head (pops)    
  ## # A tibble: 6 × 5
##   Abbreviation Latitude Longitude Pop_City   Country 
##   <chr>           <dbl>     <dbl> <chr>      <chr>   
## 1 GEL              26.9      90.5 Gelephu    Bhutan  
## 2 CAM              11.6     105.  Phnom Penh Cambodia
## 3 HAI              19.2     110.  Hainan     China   
## 4 YUN              24.5     101.  Yunnan     China   
## 5 HUN              27.6     112.  Hunan      China   
## 6 BEN              13.0      77.6 Bengaluru  India  
       # Add an index column to Q_tibble  
   k5run1 $ index  <-   seq_len ( nrow (k5run1)) 
    
    # Perform the merge as before  
   df1  <-  
      merge ( 
       k5run1, 
       pops, 
        by.x =   2 , 
        by.y =   1 , 
        all.x =  T, 
        all.y =  F 
     )  |>  
      na.omit () 
    
    # Order by the index column to ensure the order matches the original Q_tibble  
   df1  <-  df1[ order (df1 $ index),] 
    
    # Optionally, you can remove the index column if it's no longer needed  
   df1 $ index  <-   NULL  
    
    # Now the rows of df1 should be in the same order as the original Q_tibble  
    head (df1)    
  ##     pop  ind    v1    v2    v3      v4    v5 Latitude Longitude Pop_City
## 159 OKI 1001 5e-06 5e-06 5e-06 0.99998 5e-06  26.5013  127.9454  Okinawa
## 160 OKI 1002 5e-06 5e-06 5e-06 0.99998 5e-06  26.5013  127.9454  Okinawa
## 161 OKI 1003 5e-06 5e-06 5e-06 0.99998 5e-06  26.5013  127.9454  Okinawa
## 162 OKI 1004 5e-06 5e-06 5e-06 0.99998 5e-06  26.5013  127.9454  Okinawa
## 163 OKI 1005 5e-06 5e-06 5e-06 0.99998 5e-06  26.5013  127.9454  Okinawa
## 164 OKI 1006 5e-06 5e-06 5e-06 0.99998 5e-06  26.5013  127.9454  Okinawa
##     Country
## 159   Japan
## 160   Japan
## 161   Japan
## 162   Japan
## 163   Japan
## 164   Japan  
 We used this color palette to make the “structure” plot 
      color_palette2  <-  
      c ( 
        "v1"   =   "#FFB347" , 
        "v2"   =   "red" , 
        "v3"   =   "#F49AC2" , 
        "v4"   =   "#FFFF99" , 
        "v5"   =   "#AE9393"  #,  
        # "v6" = "#008080",  
        # "v7" = "#FFFF99"  
     )    
      world  <-   ne_countries ( scale =   "medium" ,  returnclass =   "sf" ) 
   countries_with_data  <-   unique (df1 $ Country) 
    
    # Filtering the world data to include only the countries in your data  
   selected_countries  <-  world  |>  
      filter (admin  %in%  countries_with_data) 
    
    # Calculate mean proportions for each population  
   df_mean  <-  df1  |>  
      group_by (pop)  |>  
      summarise ( across ( starts_with ( "v" ), \(x)  mean (x,  na.rm =   TRUE )),  
                Longitude =   mean (Longitude), 
                Latitude =   mean (Latitude)) 
    
    
    source ( 
      here ( 
        "scripts" ,  "analysis" ,  "my_theme2.R"  
     ) 
   ) 
    
    ggplot ()  +  
      geom_sf ( data =  selected_countries,  fill=  "white" )  +  
      geom_scatterpie ( data =  df_mean,  
                      aes ( x =  Longitude,  y =  Latitude,  r =   1.5 ),  
                      cols =   c ( "v1" ,  "v2" ,  "v3" ,  "v4" ,  "v5" ),  color =   NA )  +  
      geom_text_repel ( data =  df_mean, 
                      aes ( x =  Longitude,  y =  Latitude,  label =  pop),  
                      size =   3 ,  
                      box.padding =   unit ( 0.5 ,  "lines" ), 
                      max.overlaps =   50 )  +  
      scale_fill_manual ( values =  color_palette2)  +  
      guides ( fill =   "none" )  +    # Hide legend  
      # coord_sf() +  
      coord_sf ( xlim =   c ( 60 ,  150 ),  ylim =   c ( -  10 ,  60 ))  +  
      my_theme ()    
   
       # #   
    ggsave ( 
      here ( "output" ,  "populations" ,  "figures" ,  "fastStructure_r2_0.01_logistic_k5_pie.pdf" ), 
      width  =   12 , 
      height =   6 , 
      units  =   "in" , 
      device =  cairo_pdf 
   )    
 
 9.1 Preparing the data for tess3Q_map_rasters 
 Make sure the lat longs are in the correct order and arrangement 
      df2  <-  df1  |>  
     dplyr ::  rename ( 
        Long =  Longitude, 
        Lat =  Latitude 
     ) 
    
   long_lat_tibble  <-  df2  |>  
     dplyr ::  select (Long, Lat) 
    
    
   long_lat_matrix  <-  long_lat_tibble  |>  
      as.matrix () 
    
    head (long_lat_matrix)    
  ##         Long     Lat
## 159 127.9454 26.5013
## 160 127.9454 26.5013
## 161 127.9454 26.5013
## 162 127.9454 26.5013
## 163 127.9454 26.5013
## 164 127.9454 26.5013  
 
 
 9.2 make a matrix of the Q values 
 Pull off the names of individuals and make a matrix of it 
      Q_matrix  <-  k5run1  |>  
     dplyr ::  select ( - ind,  - pop,  - index)  |>  
      as.matrix () 
    head (Q_matrix)    
  ##         v1    v2    v3      v4    v5
## [1,] 5e-06 5e-06 5e-06 0.99998 5e-06
## [2,] 5e-06 5e-06 5e-06 0.99998 5e-06
## [3,] 5e-06 5e-06 5e-06 0.99998 5e-06
## [4,] 5e-06 5e-06 5e-06 0.99998 5e-06
## [5,] 5e-06 5e-06 5e-06 0.99998 5e-06
## [6,] 5e-06 5e-06 5e-06 0.99998 5e-06  
 
 
 9.3 Interpolate the Q-values by Kriging 
       print ( ncol (Q_matrix)  ==   length (color_palette2))    
  ## [1] TRUE  
 Create brick 
      genoscape_brick  <-  tess3r ::  tess3Q_map_rasters ( 
      x =  Q_matrix,  
      coord =  long_lat_matrix,   
      map.polygon =  selected_countries, 
      window =   extent (selected_countries)[ 1  :  4 ], 
      # window = combined_extent,  
      resolution =   c ( 600 , 600 ),  # if you want more cells in your raster, set higher  
      # this next lines need to to be here, but don't do much...  
      col.palette =  tess3r ::  CreatePalette (color_palette2,  length (color_palette2)), 
      method =   "map.max" ,  
      interpol =  tess3r ::  FieldsKrigModel ( 40 ),   
      main =   "Ancestry coefficients" , 
      xlab =   "Longitude" ,  
      ylab =   "Latitude" ,  
      cex =  . 4  
   )    
  ## Warning: 
## Grid searches over lambda (nugget and sill variances) with  minima at the endpoints: 
##   (REML) Restricted maximum likelihood 
##    minimum at  right endpoint  lambda  =  0.04206056 (eff. df= 26.60001 )
## Warning: 
## Grid searches over lambda (nugget and sill variances) with  minima at the endpoints: 
##   (REML) Restricted maximum likelihood 
##    minimum at  right endpoint  lambda  =  0.04206056 (eff. df= 26.60001 )
## Warning: 
## Grid searches over lambda (nugget and sill variances) with  minima at the endpoints: 
##   (REML) Restricted maximum likelihood 
##    minimum at  right endpoint  lambda  =  0.04206056 (eff. df= 26.60001 )  
       # after that, we need to add names of the clusters back onto this raster brick  
   Q_tibble2  <-  k5run1  |>  
     dplyr ::  select ( 
        - pop,  - ind,  - index 
     ) 
    names (genoscape_brick)  <-   names (Q_tibble2)[]    
 
 
 9.4 Scaling and cleaning the genoscape_brick 
      genoscape_rgba  <-  genoscapeRtools ::  qprob_rando_raster ( 
      TRB =  genoscape_brick, 
      cols =  color_palette2, 
      alpha_scale =   2.0 , 
      abs_thresh =   0.0 , 
      alpha_exp =   1.55 , 
      alpha_chop_max =   255  
   ) 
    
    crs (genoscape_rgba)  <-   "+proj=longlat +datum=WGS84 +no_defs +ellps=WGS84 +towgs84=0,0,0"     
       ggplot ()  +   
     ggspatial ::  layer_spatial (genoscape_rgba)  +   
      my_theme ()  +  
      coord_sf ()    
 Plot 
       ggplot ()  +  
      layer_spatial (genoscape_rgba)  +  
      geom_spatial_point ( data =  long_lat_tibble, 
                         mapping =   aes ( x =  Long,  y =  Lat), 
                         size =  . 2 )  +  
      geom_text_repel ( 
        data =  df_mean, 
        aes ( x =  Longitude,  y =  Latitude,  label =  pop), 
        size =   3 , 
        box.padding =   unit ( 0.5 ,  "lines" ) 
     )  +  
      labs ( x =   "Longitude" , 
           y =   "Latitude" )  +  
      geom_scatterpie ( 
        data =  df_mean, 
        aes ( x =  Longitude,  y =  Latitude,  r =   1 ), 
        cols =   c ( "v1" ,  "v2" ,  "v3" ,  "v4" ,  "v5" ), 
        color =   NA  
     )  +  
      my_theme ()  +  
      scale_fill_manual ( values =  color_palette2)  +  
      guides ( fill =   "none" )  +    # Hide legend  
      coord_sf ()    
  ## Assuming `crs = 4326` in stat_spatial_identity()  
   
       ggsave ( 
      here ( "output" ,  "populations" ,  "figures" ,  "fastStructure_r2_0.01_logistic_k5_interpolated_pie.pdf" ), 
      width  =   12 , 
      height =   6 , 
      units  =   "in" , 
      device =  cairo_pdf 
   )    
  ## Assuming `crs = 4326` in stat_spatial_identity()  
 
 
 
 10. fastStructure r2 0.1 SNPs simple prior 
 Clear memory and environment 
       # Clear entire environment  
    rm ( list =   ls ()) 
    # Forcefully trigger garbage collection  
    gc ()    
  ##           used  (Mb) gc trigger  (Mb) limit (Mb) max used  (Mb)
## Ncells 4697490 250.9   12802251 683.8         NA 12802251 683.8
## Vcells 8797829  67.2   38936470 297.1      32768 68732428 524.4  
 Make plot 
       # Extract ancestry coefficients  
   k5run1  <-   read_delim ( 
      here ( "output" ,  "populations" ,  "faststructure" ,  "r2_0.1" ,  "run1" ,  "simple.5.meanQ" ), 
      delim =   "  " ,  # Specify the delimiter if different from the default (comma)  
      col_names =   FALSE , 
      show_col_types =   FALSE  
   )  
    
    head (k5run1)    
  ## # A tibble: 6 × 5
##         X1       X2       X3    X4       X5
##      <dbl>    <dbl>    <dbl> <dbl>    <dbl>
## 1 0.000002 0.000002 0.0114   0.791 0.198   
## 2 0.146    0.000002 0.000002 0.694 0.160   
## 3 0.000002 0.000002 0.000002 0.949 0.0510  
## 4 0.000002 0.000002 0.000002 1.00  0.000002
## 5 0.000002 0.000002 0.000002 1.00  0.000002
## 6 0.000002 0.000002 0.000002 1.00  0.000002  
 The fam file 
      fam_file  <-   here ( 
      "output" ,  "populations" ,  "snps_sets" ,  "r2_0.1.fam"  
   ) 
    
    # Read the .fam file  
   fam_data  <-   read.table (fam_file,  
                           header =   FALSE , 
                           col.names =   c ( "FamilyID" ,  "IndividualID" ,  "PaternalID" ,  "MaternalID" ,  "Sex" ,  "Phenotype" )) 
    
    # View the first few rows  
    head (fam_data)    
  ##   FamilyID IndividualID PaternalID MaternalID Sex Phenotype
## 1      OKI         1001          0          0   2        -9
## 2      OKI         1002          0          0   2        -9
## 3      OKI         1003          0          0   2        -9
## 4      OKI         1004          0          0   2        -9
## 5      OKI         1005          0          0   2        -9
## 6      OKI         1006          0          0   1        -9  
 Create ID column 
       # Change column name  
    colnames (fam_data)[ colnames (fam_data)  ==   "IndividualID" ]  <-   "ind"  
    
    # Change column name  
    colnames (fam_data)[ colnames (fam_data)  ==   "FamilyID" ]  <-   "pop"  
    
    # Select ID  
   fam_data  <-  fam_data  |>  
     dplyr ::  select ( "ind" ,  "pop" ) 
    
    # View the first few rows  
    head (fam_data)    
  ##    ind pop
## 1 1001 OKI
## 2 1002 OKI
## 3 1003 OKI
## 4 1004 OKI
## 5 1005 OKI
## 6 1006 OKI  
 Add it to matrix 
      k5run1  <-  fam_data  |>  
     dplyr ::  select (ind, pop)  |>  
      bind_cols (k5run1) 
    
    head (k5run1)    
  ##    ind pop       X1    X2       X3       X4       X5
## 1 1001 OKI 0.000002 2e-06 0.011353 0.790797 0.197846
## 2 1002 OKI 0.146314 2e-06 0.000002 0.694085 0.159598
## 3 1003 OKI 0.000002 2e-06 0.000002 0.949043 0.050951
## 4 1004 OKI 0.000002 2e-06 0.000002 0.999993 0.000002
## 5 1005 OKI 0.000002 2e-06 0.000002 0.999993 0.000002
## 6 1006 OKI 0.000002 2e-06 0.000002 0.999993 0.000002  
 Rename the columns 
       # Rename the columns starting from the third one  
   k5run1  <-  k5run1  |>  
      rename_with ( ~  paste0 ( "v" ,  seq_along (.x)),  .cols =   -  c (ind, pop)) 
    
    # View the first few rows  
    head (k5run1)    
  ##    ind pop       v1    v2       v3       v4       v5
## 1 1001 OKI 0.000002 2e-06 0.011353 0.790797 0.197846
## 2 1002 OKI 0.146314 2e-06 0.000002 0.694085 0.159598
## 3 1003 OKI 0.000002 2e-06 0.000002 0.949043 0.050951
## 4 1004 OKI 0.000002 2e-06 0.000002 0.999993 0.000002
## 5 1005 OKI 0.000002 2e-06 0.000002 0.999993 0.000002
## 6 1006 OKI 0.000002 2e-06 0.000002 0.999993 0.000002  
 Import samples attributes 
      sampling_loc  <-   readRDS ( here ( "output" ,  "populations" ,  "sampling_loc.rds" )) 
    # head(sampling_loc)  
    
   pops  <-  sampling_loc  |>  
      filter ( 
       Region  ==   "Asia"  
     )  |>  
     dplyr ::  select ( 
       Abbreviation, Latitude, Longitude, Pop_City, Country 
     ) 
    
    head (pops)    
  ## # A tibble: 6 × 5
##   Abbreviation Latitude Longitude Pop_City   Country 
##   <chr>           <dbl>     <dbl> <chr>      <chr>   
## 1 GEL              26.9      90.5 Gelephu    Bhutan  
## 2 CAM              11.6     105.  Phnom Penh Cambodia
## 3 HAI              19.2     110.  Hainan     China   
## 4 YUN              24.5     101.  Yunnan     China   
## 5 HUN              27.6     112.  Hunan      China   
## 6 BEN              13.0      77.6 Bengaluru  India  
       # Add an index column to Q_tibble  
   k5run1 $ index  <-   seq_len ( nrow (k5run1)) 
    
    # Perform the merge as before  
   df1  <-  
      merge ( 
       k5run1, 
       pops, 
        by.x =   2 , 
        by.y =   1 , 
        all.x =  T, 
        all.y =  F 
     )  |>  
      na.omit () 
    
    # Order by the index column to ensure the order matches the original Q_tibble  
   df1  <-  df1[ order (df1 $ index),] 
    
    # Optionally, you can remove the index column if it's no longer needed  
   df1 $ index  <-   NULL  
    
    # Now the rows of df1 should be in the same order as the original Q_tibble  
    head (df1)    
  ##     pop  ind       v1    v2       v3       v4       v5 Latitude Longitude
## 159 OKI 1001 0.000002 2e-06 0.011353 0.790797 0.197846  26.5013  127.9454
## 160 OKI 1002 0.146314 2e-06 0.000002 0.694085 0.159598  26.5013  127.9454
## 161 OKI 1003 0.000002 2e-06 0.000002 0.949043 0.050951  26.5013  127.9454
## 162 OKI 1004 0.000002 2e-06 0.000002 0.999993 0.000002  26.5013  127.9454
## 163 OKI 1005 0.000002 2e-06 0.000002 0.999993 0.000002  26.5013  127.9454
## 164 OKI 1006 0.000002 2e-06 0.000002 0.999993 0.000002  26.5013  127.9454
##     Pop_City Country
## 159  Okinawa   Japan
## 160  Okinawa   Japan
## 161  Okinawa   Japan
## 162  Okinawa   Japan
## 163  Okinawa   Japan
## 164  Okinawa   Japan  
 We used this color palette to make the “structure” plot 
      color_palette2  <-  
      c ( 
        "v1"   =   "#F49AC2" , 
        "v2"   =   "red" , 
        "v3"   =   "#FFFF99" , 
        "v4"   =   "#AE9393" , 
        "v5"   =   "#FFB347"  
     )    
 Plot 
      world  <-   ne_countries ( scale =   "medium" ,  returnclass =   "sf" ) 
   countries_with_data  <-   unique (df1 $ Country) 
    
    # Filtering the world data to include only the countries in your data  
   selected_countries  <-  world  |>  
      filter (admin  %in%  countries_with_data) 
    
    # Calculate mean proportions for each population  
   df_mean  <-  df1  |>  
      group_by (pop)  |>  
      summarise ( across ( starts_with ( "v" ), \(x)  mean (x,  na.rm =   TRUE )),  
                Longitude =   mean (Longitude), 
                Latitude =   mean (Latitude)) 
    
    
    source ( 
      here ( 
        "scripts" ,  "analysis" ,  "my_theme2.R"  
     ) 
   ) 
    
    ggplot ()  +  
      geom_sf ( data =  selected_countries,  fill=  "white" )  +  
      geom_scatterpie ( data =  df_mean,  
                      aes ( x =  Longitude,  y =  Latitude,  r =   1.5 ),  
                      cols =   c ( "v1" ,  "v2" ,  "v3" ,  "v4" ,  "v5" ),  color =   NA )  +  
      geom_text_repel ( data =  df_mean, 
                      aes ( x =  Longitude,  y =  Latitude,  label =  pop),  
                      size =   3 ,  
                      box.padding =   unit ( 0.5 ,  "lines" ), 
                      max.overlaps =   50 )  +  
      scale_fill_manual ( values =  color_palette2)  +  
      guides ( fill =   "none" )  +    # Hide legend  
      # coord_sf() +  
      coord_sf ( xlim =   c ( 60 ,  150 ),  ylim =   c ( -  10 ,  60 ))  +  
      my_theme ()    
   
       # #   
    ggsave ( 
      here ( "output" ,  "populations" ,  "figures" ,  "fastStructure_r2_0.1_simple_k5_pie.pdf" ), 
      width  =   12 , 
      height =   6 , 
      units  =   "in" , 
      device =  cairo_pdf 
   )    
 
 10.1 Preparing the data for tess3Q_map_rasters 
      df2  <-  df1  |>  
     dplyr ::  rename ( 
        Long =  Longitude, 
        Lat =  Latitude 
     ) 
    
   long_lat_tibble  <-  df2  |>  
     dplyr ::  select (Long, Lat) 
    
    
   long_lat_matrix  <-  long_lat_tibble  |>  
      as.matrix () 
    
    head (long_lat_matrix)    
  ##         Long     Lat
## 159 127.9454 26.5013
## 160 127.9454 26.5013
## 161 127.9454 26.5013
## 162 127.9454 26.5013
## 163 127.9454 26.5013
## 164 127.9454 26.5013  
 
 
 10.2 make a matrix of the Q values 
 Pull off the names of individuals and make a matrix of it: 
      Q_matrix  <-  k5run1  |>  
     dplyr ::  select ( - ind,  - pop,  - index)  |>  
      as.matrix () 
    head (Q_matrix)    
  ##            v1    v2       v3       v4       v5
## [1,] 0.000002 2e-06 0.011353 0.790797 0.197846
## [2,] 0.146314 2e-06 0.000002 0.694085 0.159598
## [3,] 0.000002 2e-06 0.000002 0.949043 0.050951
## [4,] 0.000002 2e-06 0.000002 0.999993 0.000002
## [5,] 0.000002 2e-06 0.000002 0.999993 0.000002
## [6,] 0.000002 2e-06 0.000002 0.999993 0.000002  
 
 
 10.3 Interpolate the Q-values by Kriging 
       print ( ncol (Q_matrix)  ==   length (color_palette2))    
  ## [1] TRUE  
 Create brick 
      genoscape_brick  <-  tess3r ::  tess3Q_map_rasters ( 
      x =  Q_matrix,  
      coord =  long_lat_matrix,   
      map.polygon =  selected_countries, 
      window =   extent (selected_countries)[ 1  :  4 ], 
      # window = combined_extent,  
      resolution =   c ( 600 , 600 ),  # if you want more cells in your raster, set higher  
      # this next lines need to to be here, but don't do much...  
      col.palette =  tess3r ::  CreatePalette (color_palette2,  length (color_palette2)), 
      method =   "map.max" ,  
      interpol =  tess3r ::  FieldsKrigModel ( 40 ),   
      main =   "Ancestry coefficients" , 
      xlab =   "Longitude" ,  
      ylab =   "Latitude" ,  
      cex =  . 4  
   )    
  ## Warning: 
## Grid searches over lambda (nugget and sill variances) with  minima at the endpoints: 
##   (REML) Restricted maximum likelihood 
##    minimum at  right endpoint  lambda  =  0.04206056 (eff. df= 26.60001 )
## Warning: 
## Grid searches over lambda (nugget and sill variances) with  minima at the endpoints: 
##   (REML) Restricted maximum likelihood 
##    minimum at  right endpoint  lambda  =  0.04206056 (eff. df= 26.60001 )
## Warning: 
## Grid searches over lambda (nugget and sill variances) with  minima at the endpoints: 
##   (REML) Restricted maximum likelihood 
##    minimum at  right endpoint  lambda  =  0.04206056 (eff. df= 26.60001 )
## Warning: 
## Grid searches over lambda (nugget and sill variances) with  minima at the endpoints: 
##   (REML) Restricted maximum likelihood 
##    minimum at  right endpoint  lambda  =  0.04206056 (eff. df= 26.60001 )
## Warning: 
## Grid searches over lambda (nugget and sill variances) with  minima at the endpoints: 
##   (REML) Restricted maximum likelihood 
##    minimum at  right endpoint  lambda  =  0.04206056 (eff. df= 26.60001 )  
       # after that, we need to add names of the clusters back onto this raster brick  
   Q_tibble2  <-  k5run1  |>  
     dplyr ::  select ( 
        - pop,  - ind,  - index 
     ) 
    names (genoscape_brick)  <-   names (Q_tibble2)[]    
 
 
 10.4 Scaling and cleaning the genoscape_brick 
      genoscape_rgba  <-  genoscapeRtools ::  qprob_rando_raster ( 
      TRB =  genoscape_brick, 
      cols =  color_palette2, 
      alpha_scale =   2.0 , 
      abs_thresh =   0.0 , 
      alpha_exp =   1.55 , 
      alpha_chop_max =   255  
   ) 
    
    crs (genoscape_rgba)  <-   "+proj=longlat +datum=WGS84 +no_defs +ellps=WGS84 +towgs84=0,0,0"     
 Plot 
       ggplot ()  +   
     ggspatial ::  layer_spatial (genoscape_rgba)  +   
      my_theme ()  +  
      coord_sf ()    
 Plot 
       ggplot ()  +  
      layer_spatial (genoscape_rgba)  +  
      geom_spatial_point ( data =  long_lat_tibble, 
                         mapping =   aes ( x =  Long,  y =  Lat), 
                         size =  . 2 )  +  
      geom_text_repel ( 
        data =  df_mean, 
        aes ( x =  Longitude,  y =  Latitude,  label =  pop), 
        size =   3 , 
        box.padding =   unit ( 0.5 ,  "lines" ) 
     )  +  
      labs ( x =   "Longitude" , 
           y =   "Latitude" )  +  
      geom_scatterpie ( 
        data =  df_mean, 
        aes ( x =  Longitude,  y =  Latitude,  r =   1 ), 
        cols =   c ( "v1" ,  "v2" ,  "v3" ,  "v4" ,  "v5" ), 
        color =   NA  
     )  +  
      my_theme ()  +  
      scale_fill_manual ( values =  color_palette2)  +  
      guides ( fill =   "none" )  +    # Hide legend  
      coord_sf ()    
  ## Assuming `crs = 4326` in stat_spatial_identity()  
   
       ggsave ( 
      here ( "output" ,  "populations" ,  "figures" ,  "fastStructure_r2_0.1_simple_k5_interpolated_pie.pdf" ), 
      width  =   12 , 
      height =   6 , 
      units  =   "in" , 
      device =  cairo_pdf 
   )    
  ## Assuming `crs = 4326` in stat_spatial_identity()  
 
 
 
 11. fastStructure r2 0.1 SNPs logistic prior 
 Clear memory and environment 
       # Clear entire environment  
    rm ( list =   ls ()) 
    # Forcefully trigger garbage collection  
    gc ()    
  ##           used  (Mb) gc trigger  (Mb) limit (Mb) max used  (Mb)
## Ncells 4697767 250.9   12802251 683.8         NA 12802251 683.8
## Vcells 8799348  67.2   37633222 287.2      32768 68732428 524.4  
 Make plot 
       # Extract ancestry coefficients  
   k5run1  <-   read_delim ( 
      here ( "output" ,  "populations" ,  "faststructure" ,  "r2_0.1" ,  "run1" ,  "logistic.5.meanQ" ), 
      delim =   "  " ,  # Specify the delimiter if different from the default (comma)  
      col_names =   FALSE , 
      show_col_types =   FALSE  
   )  
    
    head (k5run1)    
  ## # A tibble: 6 × 5
##         X1       X2     X3       X4    X5
##      <dbl>    <dbl>  <dbl>    <dbl> <dbl>
## 1 0.000002 0.000002 0.102  0.000002 0.898
## 2 0.000002 0.000002 0.0529 0.0395   0.908
## 3 0.000002 0.000002 0.0328 0.000002 0.967
## 4 0.000002 0.000002 0.0318 0.000002 0.968
## 5 0.000002 0.000002 0.0112 0.000002 0.989
## 6 0.000002 0.000002 0.0348 0.000002 0.965  
 The fam file 
      fam_file  <-   here ( 
      "output" ,  "populations" ,  "snps_sets" ,  "r2_0.1.fam"  
   ) 
    
    # Read the .fam file  
   fam_data  <-   read.table (fam_file,  
                           header =   FALSE , 
                           col.names =   c ( "FamilyID" ,  "IndividualID" ,  "PaternalID" ,  "MaternalID" ,  "Sex" ,  "Phenotype" )) 
    
    # View the first few rows  
    head (fam_data)    
  ##   FamilyID IndividualID PaternalID MaternalID Sex Phenotype
## 1      OKI         1001          0          0   2        -9
## 2      OKI         1002          0          0   2        -9
## 3      OKI         1003          0          0   2        -9
## 4      OKI         1004          0          0   2        -9
## 5      OKI         1005          0          0   2        -9
## 6      OKI         1006          0          0   1        -9  
 Create ID column 
       # Change column name  
    colnames (fam_data)[ colnames (fam_data)  ==   "IndividualID" ]  <-   "ind"  
    
    # Change column name  
    colnames (fam_data)[ colnames (fam_data)  ==   "FamilyID" ]  <-   "pop"  
    
    # Select ID  
   fam_data  <-  fam_data  |>  
     dplyr ::  select ( "ind" ,  "pop" ) 
    
    # View the first few rows  
    head (fam_data)    
  ##    ind pop
## 1 1001 OKI
## 2 1002 OKI
## 3 1003 OKI
## 4 1004 OKI
## 5 1005 OKI
## 6 1006 OKI  
 Add it to matrix 
      k5run1  <-  fam_data  |>  
     dplyr ::  select (ind, pop)  |>  
      bind_cols (k5run1) 
    
    head (k5run1)    
  ##    ind pop    X1    X2       X3       X4       X5
## 1 1001 OKI 2e-06 2e-06 0.102183 0.000002 0.897812
## 2 1002 OKI 2e-06 2e-06 0.052898 0.039471 0.907627
## 3 1003 OKI 2e-06 2e-06 0.032833 0.000002 0.967162
## 4 1004 OKI 2e-06 2e-06 0.031840 0.000002 0.968155
## 5 1005 OKI 2e-06 2e-06 0.011165 0.000002 0.988830
## 6 1006 OKI 2e-06 2e-06 0.034841 0.000002 0.965154  
 Rename the columns 
       # Rename the columns starting from the third one  
   k5run1  <-  k5run1  |>  
      rename_with ( ~  paste0 ( "v" ,  seq_along (.x)),  .cols =   -  c (ind, pop)) 
    
    # View the first few rows  
    head (k5run1)    
  ##    ind pop    v1    v2       v3       v4       v5
## 1 1001 OKI 2e-06 2e-06 0.102183 0.000002 0.897812
## 2 1002 OKI 2e-06 2e-06 0.052898 0.039471 0.907627
## 3 1003 OKI 2e-06 2e-06 0.032833 0.000002 0.967162
## 4 1004 OKI 2e-06 2e-06 0.031840 0.000002 0.968155
## 5 1005 OKI 2e-06 2e-06 0.011165 0.000002 0.988830
## 6 1006 OKI 2e-06 2e-06 0.034841 0.000002 0.965154  
 Import samples attributes 
      sampling_loc  <-   readRDS ( here ( "output" ,  "populations" ,  "sampling_loc.rds" )) 
    # head(sampling_loc)  
    
   pops  <-  sampling_loc  |>  
      filter ( 
       Region  ==   "Asia"  
     )  |>  
     dplyr ::  select ( 
       Abbreviation, Latitude, Longitude, Pop_City, Country 
     ) 
    
    head (pops)    
  ## # A tibble: 6 × 5
##   Abbreviation Latitude Longitude Pop_City   Country 
##   <chr>           <dbl>     <dbl> <chr>      <chr>   
## 1 GEL              26.9      90.5 Gelephu    Bhutan  
## 2 CAM              11.6     105.  Phnom Penh Cambodia
## 3 HAI              19.2     110.  Hainan     China   
## 4 YUN              24.5     101.  Yunnan     China   
## 5 HUN              27.6     112.  Hunan      China   
## 6 BEN              13.0      77.6 Bengaluru  India  
       # Add an index column to Q_tibble  
   k5run1 $ index  <-   seq_len ( nrow (k5run1)) 
    
    # Perform the merge as before  
   df1  <-  
      merge ( 
       k5run1, 
       pops, 
        by.x =   2 , 
        by.y =   1 , 
        all.x =  T, 
        all.y =  F 
     )  |>  
      na.omit () 
    
    # Order by the index column to ensure the order matches the original Q_tibble  
   df1  <-  df1[ order (df1 $ index),] 
    
    # Optionally, you can remove the index column if it's no longer needed  
   df1 $ index  <-   NULL  
    
    # Now the rows of df1 should be in the same order as the original Q_tibble  
    head (df1)    
  ##     pop  ind    v1    v2       v3       v4       v5 Latitude Longitude Pop_City
## 159 OKI 1001 2e-06 2e-06 0.102183 0.000002 0.897812  26.5013  127.9454  Okinawa
## 160 OKI 1002 2e-06 2e-06 0.052898 0.039471 0.907627  26.5013  127.9454  Okinawa
## 161 OKI 1003 2e-06 2e-06 0.032833 0.000002 0.967162  26.5013  127.9454  Okinawa
## 162 OKI 1004 2e-06 2e-06 0.031840 0.000002 0.968155  26.5013  127.9454  Okinawa
## 163 OKI 1005 2e-06 2e-06 0.011165 0.000002 0.988830  26.5013  127.9454  Okinawa
## 164 OKI 1006 2e-06 2e-06 0.034841 0.000002 0.965154  26.5013  127.9454  Okinawa
##     Country
## 159   Japan
## 160   Japan
## 161   Japan
## 162   Japan
## 163   Japan
## 164   Japan  
 We used this color palette to make the “structure” plot 
      color_palette2  <-  
      c ( 
        "v1"   =   "#F49AC2" , 
        "v2"   =   "red" , 
        "v3"   =   "#FFB347" , 
        "v4"   =   "#AE9393" , 
        "v5"   =   "#FFFF99"  
     )    
      world  <-   ne_countries ( scale =   "medium" ,  returnclass =   "sf" ) 
   countries_with_data  <-   unique (df1 $ Country) 
    
    # Filtering the world data to include only the countries in your data  
   selected_countries  <-  world  |>  
      filter (admin  %in%  countries_with_data) 
    
    # Calculate mean proportions for each population  
   df_mean  <-  df1  |>  
      group_by (pop)  |>  
      summarise ( across ( starts_with ( "v" ), \(x)  mean (x,  na.rm =   TRUE )),  
                Longitude =   mean (Longitude), 
                Latitude =   mean (Latitude)) 
    
    
    source ( 
      here ( 
        "scripts" ,  "analysis" ,  "my_theme2.R"  
     ) 
   ) 
    
    ggplot ()  +  
      geom_sf ( data =  selected_countries,  fill=  "white" )  +  
      geom_scatterpie ( data =  df_mean,  
                      aes ( x =  Longitude,  y =  Latitude,  r =   1.5 ),  
                      cols =   c ( "v1" ,  "v2" ,  "v3" ,  "v4" ,  "v5" ),  color =   NA )  +  
      geom_text_repel ( data =  df_mean, 
                      aes ( x =  Longitude,  y =  Latitude,  label =  pop),  
                      size =   3 ,  
                      box.padding =   unit ( 0.5 ,  "lines" ), 
                      max.overlaps =   50 )  +  
      scale_fill_manual ( values =  color_palette2)  +  
      guides ( fill =   "none" )  +    # Hide legend  
      # coord_sf() +  
      coord_sf ( xlim =   c ( 60 ,  150 ),  ylim =   c ( -  10 ,  60 ))  +  
      my_theme ()    
   
       # #   
    ggsave ( 
      here ( "output" ,  "populations" ,  "figures" ,  "fastStructure_r2_0.1_logistic_k5_pie.pdf" ), 
      width  =   12 , 
      height =   6 , 
      units  =   "in" , 
      device =  cairo_pdf 
   )    
 
 11.1 Preparing the data for tess3Q_map_rasters 
      df2  <-  df1  |>  
     dplyr ::  rename ( 
        Long =  Longitude, 
        Lat =  Latitude 
     ) 
    
   long_lat_tibble  <-  df2  |>  
     dplyr ::  select (Long, Lat) 
    
    
   long_lat_matrix  <-  long_lat_tibble  |>  
      as.matrix () 
    
    head (long_lat_matrix)    
  ##         Long     Lat
## 159 127.9454 26.5013
## 160 127.9454 26.5013
## 161 127.9454 26.5013
## 162 127.9454 26.5013
## 163 127.9454 26.5013
## 164 127.9454 26.5013  
 
 
 11.2 make a matrix of the Q values 
 Pull off the names of individuals and make a matrix of it 
      Q_matrix  <-  k5run1  |>  
     dplyr ::  select ( - ind,  - pop,  - index)  |>  
      as.matrix () 
    head (Q_matrix)    
  ##         v1    v2       v3       v4       v5
## [1,] 2e-06 2e-06 0.102183 0.000002 0.897812
## [2,] 2e-06 2e-06 0.052898 0.039471 0.907627
## [3,] 2e-06 2e-06 0.032833 0.000002 0.967162
## [4,] 2e-06 2e-06 0.031840 0.000002 0.968155
## [5,] 2e-06 2e-06 0.011165 0.000002 0.988830
## [6,] 2e-06 2e-06 0.034841 0.000002 0.965154  
 
 
 11.3 Interpolate the Q-values by Kriging 
       print ( ncol (Q_matrix)  ==   length (color_palette2))    
  ## [1] TRUE  
 Create brick 
      genoscape_brick  <-  tess3r ::  tess3Q_map_rasters ( 
      x =  Q_matrix,  
      coord =  long_lat_matrix,   
      map.polygon =  selected_countries, 
      window =   extent (selected_countries)[ 1  :  4 ], 
      # window = combined_extent,  
      resolution =   c ( 600 , 600 ),  # if you want more cells in your raster, set higher  
      # this next lines need to to be here, but don't do much...  
      col.palette =  tess3r ::  CreatePalette (color_palette2,  length (color_palette2)), 
      method =   "map.max" ,  
      interpol =  tess3r ::  FieldsKrigModel ( 40 ),   
      main =   "Ancestry coefficients" , 
      xlab =   "Longitude" ,  
      ylab =   "Latitude" ,  
      cex =  . 4  
   )    
  ## Warning: 
## Grid searches over lambda (nugget and sill variances) with  minima at the endpoints: 
##   (REML) Restricted maximum likelihood 
##    minimum at  right endpoint  lambda  =  0.04206056 (eff. df= 26.60001 )
## Warning: 
## Grid searches over lambda (nugget and sill variances) with  minima at the endpoints: 
##   (REML) Restricted maximum likelihood 
##    minimum at  right endpoint  lambda  =  0.04206056 (eff. df= 26.60001 )
## Warning: 
## Grid searches over lambda (nugget and sill variances) with  minima at the endpoints: 
##   (REML) Restricted maximum likelihood 
##    minimum at  right endpoint  lambda  =  0.04206056 (eff. df= 26.60001 )  
       # after that, we need to add names of the clusters back onto this raster brick  
   Q_tibble2  <-  k5run1  |>  
     dplyr ::  select ( 
        - pop,  - ind,  - index 
     ) 
    names (genoscape_brick)  <-   names (Q_tibble2)[]    
 
 
 11.4 Scaling and cleaning the genoscape_brick 
      genoscape_rgba  <-  genoscapeRtools ::  qprob_rando_raster ( 
      TRB =  genoscape_brick, 
      cols =  color_palette2, 
      alpha_scale =   2.0 , 
      abs_thresh =   0.0 , 
      alpha_exp =   1.55 , 
      alpha_chop_max =   255  
   ) 
    
    crs (genoscape_rgba)  <-   "+proj=longlat +datum=WGS84 +no_defs +ellps=WGS84 +towgs84=0,0,0"     
 Plot 
       ggplot ()  +   
     ggspatial ::  layer_spatial (genoscape_rgba)  +   
      my_theme ()  +  
      coord_sf ()    
   
 Plot 
       ggplot ()  +  
      layer_spatial (genoscape_rgba)  +  
      geom_spatial_point ( data =  long_lat_tibble, 
                         mapping =   aes ( x =  Long,  y =  Lat), 
                         size =  . 2 )  +  
      geom_text_repel ( 
        data =  df_mean, 
        aes ( x =  Longitude,  y =  Latitude,  label =  pop), 
        size =   3 , 
        box.padding =   unit ( 0.5 ,  "lines" ) 
     )  +  
      labs ( x =   "Longitude" , 
           y =   "Latitude" )  +  
      geom_scatterpie ( 
        data =  df_mean, 
        aes ( x =  Longitude,  y =  Latitude,  r =   1 ), 
        cols =   c ( "v1" ,  "v2" ,  "v3" ,  "v4" ,  "v5" ), 
        color =   NA  
     )  +  
      my_theme ()  +  
      scale_fill_manual ( values =  color_palette2)  +  
      guides ( fill =   "none" )  +    # Hide legend  
      coord_sf ()    
  ## Assuming `crs = 4326` in stat_spatial_identity()  
   
       ggsave ( 
      here ( "output" ,  "populations" ,  "figures" ,  "fastStructure_r2_0.1_logistic_k5_interpolated_pie.pdf" ), 
      width  =   12 , 
      height =   6 , 
      units  =   "in" , 
      device =  cairo_pdf 
   )    
  ## Assuming `crs = 4326` in stat_spatial_identity()  
 
 
 
 12. Admixture neutral SNPs k5 
 Clear memory and environment 
       # Clear entire environment  
    rm ( list =   ls ()) 
    # Forcefully trigger garbage collection  
    gc ()    
  ##           used  (Mb) gc trigger  (Mb) limit (Mb) max used  (Mb)
## Ncells 4698041 251.0   12802251 683.8         NA 12802251 683.8
## Vcells 8800849  67.2   36191893 276.2      32768 68732428 524.4  
 Make plot 
       # Extract ancestry coefficients  
   k5run1  <-   read_delim ( 
      here ( "output" ,  "populations" ,  "admixture" ,  "neutral" ,  "run1" ,  "neutral.5.Q" ), 
      delim =   " " ,  # Specify the delimiter if different from the default (comma)  
      col_names =   FALSE , 
      show_col_types =   FALSE  
   )  
    
    head (k5run1)    
  ## # A tibble: 6 × 5
##      X1    X2      X3      X4    X5
##   <dbl> <dbl>   <dbl>   <dbl> <dbl>
## 1 0.349 0.251 0.0197  0.0318  0.349
## 2 0.314 0.252 0.0244  0.00540 0.405
## 3 0.321 0.225 0.0554  0.00001 0.399
## 4 0.314 0.235 0.0173  0.0558  0.379
## 5 0.300 0.244 0.00001 0.0461  0.410
## 6 0.317 0.226 0.0539  0.0105  0.393  
 The fam file 
      fam_file  <-   here ( 
      "output" ,  "populations" ,  "snps_sets" ,  "neutral.fam"  
   ) 
    
    # Read the .fam file  
   fam_data  <-   read.table (fam_file,  
                           header =   FALSE , 
                           col.names =   c ( "FamilyID" ,  "IndividualID" ,  "PaternalID" ,  "MaternalID" ,  "Sex" ,  "Phenotype" )) 
    
    # View the first few rows  
    head (fam_data)    
  ##   FamilyID IndividualID PaternalID MaternalID Sex Phenotype
## 1      OKI         1001          0          0   2        -9
## 2      OKI         1002          0          0   2        -9
## 3      OKI         1003          0          0   2        -9
## 4      OKI         1004          0          0   2        -9
## 5      OKI         1005          0          0   2        -9
## 6      OKI         1006          0          0   1        -9  
 Create ID column 
       # Change column name  
    colnames (fam_data)[ colnames (fam_data)  ==   "IndividualID" ]  <-   "ind"  
    
    # Change column name  
    colnames (fam_data)[ colnames (fam_data)  ==   "FamilyID" ]  <-   "pop"  
    
    # Select ID  
   fam_data  <-  fam_data  |>  
     dplyr ::  select ( "ind" ,  "pop" ) 
    
    # View the first few rows  
    head (fam_data)    
  ##    ind pop
## 1 1001 OKI
## 2 1002 OKI
## 3 1003 OKI
## 4 1004 OKI
## 5 1005 OKI
## 6 1006 OKI  
 Add it to matrix 
      k5run1  <-  fam_data  |>  
     dplyr ::  select (ind, pop)  |>  
      bind_cols (k5run1) 
    
    head (k5run1)    
  ##    ind pop       X1       X2       X3       X4       X5
## 1 1001 OKI 0.348771 0.250551 0.019674 0.031750 0.349254
## 2 1002 OKI 0.313650 0.251651 0.024403 0.005396 0.404899
## 3 1003 OKI 0.321265 0.224595 0.055417 0.000010 0.398712
## 4 1004 OKI 0.313674 0.234637 0.017323 0.055846 0.378520
## 5 1005 OKI 0.299965 0.244414 0.000010 0.046103 0.409507
## 6 1006 OKI 0.317372 0.225772 0.053897 0.010453 0.392506  
 Rename the columns 
       # Rename the columns starting from the third one  
   k5run1  <-  k5run1  |>  
      rename_with ( ~  paste0 ( "v" ,  seq_along (.x)),  .cols =   -  c (ind, pop)) 
    
    # View the first few rows  
    head (k5run1)    
  ##    ind pop       v1       v2       v3       v4       v5
## 1 1001 OKI 0.348771 0.250551 0.019674 0.031750 0.349254
## 2 1002 OKI 0.313650 0.251651 0.024403 0.005396 0.404899
## 3 1003 OKI 0.321265 0.224595 0.055417 0.000010 0.398712
## 4 1004 OKI 0.313674 0.234637 0.017323 0.055846 0.378520
## 5 1005 OKI 0.299965 0.244414 0.000010 0.046103 0.409507
## 6 1006 OKI 0.317372 0.225772 0.053897 0.010453 0.392506  
 Import samples attributes 
      sampling_loc  <-   readRDS ( here ( "output" ,  "populations" ,  "sampling_loc.rds" )) 
    # head(sampling_loc)  
    
   pops  <-  sampling_loc  |>  
      filter ( 
       Region  ==   "Asia"  
     )  |>  
     dplyr ::  select ( 
       Abbreviation, Latitude, Longitude, Pop_City, Country 
     ) 
    
    head (pops)    
  ## # A tibble: 6 × 5
##   Abbreviation Latitude Longitude Pop_City   Country 
##   <chr>           <dbl>     <dbl> <chr>      <chr>   
## 1 GEL              26.9      90.5 Gelephu    Bhutan  
## 2 CAM              11.6     105.  Phnom Penh Cambodia
## 3 HAI              19.2     110.  Hainan     China   
## 4 YUN              24.5     101.  Yunnan     China   
## 5 HUN              27.6     112.  Hunan      China   
## 6 BEN              13.0      77.6 Bengaluru  India  
       # Add an index column to Q_tibble  
   k5run1 $ index  <-   seq_len ( nrow (k5run1)) 
    
    # Perform the merge as before  
   df1  <-  
      merge ( 
       k5run1, 
       pops, 
        by.x =   2 , 
        by.y =   1 , 
        all.x =  T, 
        all.y =  F 
     )  |>  
      na.omit () 
    
    # Order by the index column to ensure the order matches the original Q_tibble  
   df1  <-  df1[ order (df1 $ index),] 
    
    # Optionally, you can remove the index column if it's no longer needed  
   df1 $ index  <-   NULL  
    
    # Now the rows of df1 should be in the same order as the original Q_tibble  
    head (df1)    
  ##     pop  ind       v1       v2       v3       v4       v5 Latitude Longitude
## 159 OKI 1001 0.348771 0.250551 0.019674 0.031750 0.349254  26.5013  127.9454
## 160 OKI 1002 0.313650 0.251651 0.024403 0.005396 0.404899  26.5013  127.9454
## 161 OKI 1003 0.321265 0.224595 0.055417 0.000010 0.398712  26.5013  127.9454
## 162 OKI 1004 0.313674 0.234637 0.017323 0.055846 0.378520  26.5013  127.9454
## 163 OKI 1005 0.299965 0.244414 0.000010 0.046103 0.409507  26.5013  127.9454
## 164 OKI 1006 0.317372 0.225772 0.053897 0.010453 0.392506  26.5013  127.9454
##     Pop_City Country
## 159  Okinawa   Japan
## 160  Okinawa   Japan
## 161  Okinawa   Japan
## 162  Okinawa   Japan
## 163  Okinawa   Japan
## 164  Okinawa   Japan  
 We used this color palette to make the “structure” plot 
      color_palette2  <-  
      c ( 
        "v1"   =   "#FFB347" , 
        "v2"   =   "#F49AC2" , 
        "v3"   =   "red" , 
        "v4"   =   "#FFFF99" , 
        "v5"   =   "#AE9393"  
     )    
      world  <-   ne_countries ( scale =   "medium" ,  returnclass =   "sf" ) 
   countries_with_data  <-   unique (df1 $ Country) 
    
    # Filtering the world data to include only the countries in your data  
   selected_countries  <-  world  |>  
      filter (admin  %in%  countries_with_data) 
    
    # Calculate mean proportions for each population  
   df_mean  <-  df1  |>  
      group_by (pop)  |>  
      summarise ( across ( starts_with ( "v" ), \(x)  mean (x,  na.rm =   TRUE )),  
                Longitude =   mean (Longitude), 
                Latitude =   mean (Latitude)) 
    
    
    source ( 
      here ( 
        "scripts" ,  "analysis" ,  "my_theme2.R"  
     ) 
   ) 
    
    ggplot ()  +  
      geom_sf ( data =  selected_countries,  fill=  "white" )  +  
      geom_scatterpie ( data =  df_mean,  
                      aes ( x =  Longitude,  y =  Latitude,  r =   1.5 ),  
                      cols =   c ( "v1" ,  "v2" ,  "v3" ,  "v4" ,  "v5" ),  color =   NA )  +  
      geom_text_repel ( data =  df_mean, 
                      aes ( x =  Longitude,  y =  Latitude,  label =  pop),  
                      size =   3 ,  
                      box.padding =   unit ( 0.5 ,  "lines" ), 
                      max.overlaps =   50 )  +  
      scale_fill_manual ( values =  color_palette2)  +  
      guides ( fill =   "none" )  +    # Hide legend  
      # coord_sf() +  
      coord_sf ( xlim =   c ( 60 ,  150 ),  ylim =   c ( -  10 ,  60 ))  +  
      my_theme ()    
   
       # #   
    ggsave ( 
      here ( "output" ,  "populations" ,  "figures" ,  "admixture_neutral_k5_pie.pdf" ), 
      width  =   12 , 
      height =   6 , 
      units  =   "in" , 
      device =  cairo_pdf 
   )    
 
 12.1 Preparing the data for tess3Q_map_rasters 
 Make sure the lat longs are in the correct order and arrangement 
      df2  <-  df1  |>  
     dplyr ::  rename ( 
        Long =  Longitude, 
        Lat =  Latitude 
     ) 
    
   long_lat_tibble  <-  df2  |>  
     dplyr ::  select (Long, Lat) 
    
    
   long_lat_matrix  <-  long_lat_tibble  |>  
      as.matrix () 
    
    head (long_lat_matrix)    
  ##         Long     Lat
## 159 127.9454 26.5013
## 160 127.9454 26.5013
## 161 127.9454 26.5013
## 162 127.9454 26.5013
## 163 127.9454 26.5013
## 164 127.9454 26.5013  
 
 
 12.2 make a matrix of the Q values 
 Pull off the names of individuals and make a matrix of it: 
      Q_matrix  <-  k5run1  |>  
     dplyr ::  select ( - ind,  - pop,  - index)  |>  
      as.matrix () 
    head (Q_matrix)    
  ##            v1       v2       v3       v4       v5
## [1,] 0.348771 0.250551 0.019674 0.031750 0.349254
## [2,] 0.313650 0.251651 0.024403 0.005396 0.404899
## [3,] 0.321265 0.224595 0.055417 0.000010 0.398712
## [4,] 0.313674 0.234637 0.017323 0.055846 0.378520
## [5,] 0.299965 0.244414 0.000010 0.046103 0.409507
## [6,] 0.317372 0.225772 0.053897 0.010453 0.392506  
 
 
 12.3 Interpolate the Q-values by Kriging 
       print ( ncol (Q_matrix)  ==   length (color_palette2))    
  ## [1] TRUE  
 Create brick 
      genoscape_brick  <-  tess3r ::  tess3Q_map_rasters ( 
      x =  Q_matrix,  
      coord =  long_lat_matrix,   
      map.polygon =  selected_countries, 
      window =   extent (selected_countries)[ 1  :  4 ], 
      # window = combined_extent,  
      resolution =   c ( 600 , 600 ),  # if you want more cells in your raster, set higher  
      # this next lines need to to be here, but don't do much...  
      col.palette =  tess3r ::  CreatePalette (color_palette2,  length (color_palette2)), 
      method =   "map.max" ,  
      interpol =  tess3r ::  FieldsKrigModel ( 40 ),   
      main =   "Ancestry coefficients" , 
      xlab =   "Longitude" ,  
      ylab =   "Latitude" ,  
      cex =  . 4  
   )    
  ## Warning: 
## Grid searches over lambda (nugget and sill variances) with  minima at the endpoints: 
##   (REML) Restricted maximum likelihood 
##    minimum at  right endpoint  lambda  =  0.04206056 (eff. df= 26.60001 )
## Warning: 
## Grid searches over lambda (nugget and sill variances) with  minima at the endpoints: 
##   (REML) Restricted maximum likelihood 
##    minimum at  right endpoint  lambda  =  0.04206056 (eff. df= 26.60001 )
## Warning: 
## Grid searches over lambda (nugget and sill variances) with  minima at the endpoints: 
##   (REML) Restricted maximum likelihood 
##    minimum at  right endpoint  lambda  =  0.04206056 (eff. df= 26.60001 )
## Warning: 
## Grid searches over lambda (nugget and sill variances) with  minima at the endpoints: 
##   (REML) Restricted maximum likelihood 
##    minimum at  right endpoint  lambda  =  0.04206056 (eff. df= 26.60001 )
## Warning: 
## Grid searches over lambda (nugget and sill variances) with  minima at the endpoints: 
##   (REML) Restricted maximum likelihood 
##    minimum at  right endpoint  lambda  =  0.04206056 (eff. df= 26.60001 )  
       # after that, we need to add names of the clusters back onto this raster brick  
   Q_tibble2  <-  k5run1  |>  
     dplyr ::  select ( 
        - pop,  - ind,  - index 
     ) 
    names (genoscape_brick)  <-   names (Q_tibble2)[]    
 
 
 12.4 Scaling and cleaning the genoscape_brick 
      genoscape_rgba  <-  genoscapeRtools ::  qprob_rando_raster ( 
      TRB =  genoscape_brick, 
      cols =  color_palette2, 
      alpha_scale =   2.0 , 
      abs_thresh =   0.0 , 
      alpha_exp =   1.55 , 
      alpha_chop_max =   255  
   ) 
    
    # This adds the info for a regular lat-long projection  
    crs (genoscape_rgba)  <-   "+proj=longlat +datum=WGS84 +no_defs +ellps=WGS84 +towgs84=0,0,0"     
 Plot 
       ggplot ()  +   
     ggspatial ::  layer_spatial (genoscape_rgba)  +   
      my_theme ()  +  
      coord_sf ()    
 Plot 
       ggplot ()  +  
      layer_spatial (genoscape_rgba)  +  
      geom_spatial_point ( data =  long_lat_tibble, 
                         mapping =   aes ( x =  Long,  y =  Lat), 
                         size =  . 2 )  +  
      geom_text_repel ( 
        data =  df_mean, 
        aes ( x =  Longitude,  y =  Latitude,  label =  pop), 
        size =   3 , 
        box.padding =   unit ( 0.5 ,  "lines" ) 
     )  +  
      labs ( x =   "Longitude" , 
           y =   "Latitude" )  +  
      geom_scatterpie ( 
        data =  df_mean, 
        aes ( x =  Longitude,  y =  Latitude,  r =   1 ), 
        cols =   c ( "v1" ,  "v2" ,  "v3" ,  "v4" ,  "v5" ), 
        color =   NA  
     )  +  
      my_theme ()  +  
      scale_fill_manual ( values =  color_palette2)  +  
      guides ( fill =   "none" )  +    # Hide legend  
      coord_sf ()    
  ## Assuming `crs = 4326` in stat_spatial_identity()  
   
       ggsave ( 
      here ( "output" ,  "populations" ,  "figures" ,  "admixture_neutral_k5_interpolated_pie.pdf" ), 
      width  =   12 , 
      height =   6 , 
      units  =   "in" , 
      device =  cairo_pdf 
   )    
  ## Assuming `crs = 4326` in stat_spatial_identity()  
 
 
 
 13. Admixture r2 0.01 SNPs k5 
 Clear memory and environment 
       # Clear entire environment  
    rm ( list =   ls ()) 
    # Forcefully trigger garbage collection  
    gc ()    
  ##           used  (Mb) gc trigger  (Mb) limit (Mb) max used  (Mb)
## Ncells 4698321 251.0   12802251 683.8         NA 12802251 683.8
## Vcells 8802360  67.2   40473282 308.8      32768 68732428 524.4  
 Make plot 
       # Extract ancestry coefficients  
   k5run1  <-   read_delim ( 
      here ( "output" ,  "populations" ,  "admixture" ,  "r2_0.01" ,  "run1" ,  "r2_0.01.5.Q" ), 
      delim =   " " ,  # Specify the delimiter if different from the default (comma)  
      col_names =   FALSE , 
      show_col_types =   FALSE  
   )  
    
    head (k5run1)    
  ## # A tibble: 6 × 5
##      X1      X2      X3     X4    X5
##   <dbl>   <dbl>   <dbl>  <dbl> <dbl>
## 1 0.317 0.0707  0.00016 0.0522 0.560
## 2 0.279 0.0959  0.0108  0.0445 0.570
## 3 0.249 0.0636  0.0118  0.0126 0.663
## 4 0.243 0.0109  0.00547 0.0332 0.707
## 5 0.232 0.00151 0.00001 0.0344 0.732
## 6 0.250 0.0409  0.0151  0.0381 0.656  
 The fam file 
      fam_file  <-   here ( 
      "output" ,  "populations" ,  "snps_sets" ,  "r2_0.01.fam"  
   ) 
    
    # Read the .fam file  
   fam_data  <-   read.table (fam_file,  
                           header =   FALSE , 
                           col.names =   c ( "FamilyID" ,  "IndividualID" ,  "PaternalID" ,  "MaternalID" ,  "Sex" ,  "Phenotype" )) 
    
    # View the first few rows  
    head (fam_data)    
  ##   FamilyID IndividualID PaternalID MaternalID Sex Phenotype
## 1      OKI         1001          0          0   2        -9
## 2      OKI         1002          0          0   2        -9
## 3      OKI         1003          0          0   2        -9
## 4      OKI         1004          0          0   2        -9
## 5      OKI         1005          0          0   2        -9
## 6      OKI         1006          0          0   1        -9  
 Create ID column 
       # Change column name  
    colnames (fam_data)[ colnames (fam_data)  ==   "IndividualID" ]  <-   "ind"  
    
    # Change column name  
    colnames (fam_data)[ colnames (fam_data)  ==   "FamilyID" ]  <-   "pop"  
    
    # Select ID  
   fam_data  <-  fam_data  |>  
     dplyr ::  select ( "ind" ,  "pop" ) 
    
    # View the first few rows  
    head (fam_data)    
  ##    ind pop
## 1 1001 OKI
## 2 1002 OKI
## 3 1003 OKI
## 4 1004 OKI
## 5 1005 OKI
## 6 1006 OKI  
 Add it to matrix 
      k5run1  <-  fam_data  |>  
     dplyr ::  select (ind, pop)  |>  
      bind_cols (k5run1) 
    
    head (k5run1)    
  ##    ind pop       X1       X2       X3       X4       X5
## 1 1001 OKI 0.317249 0.070658 0.000160 0.052193 0.559741
## 2 1002 OKI 0.279230 0.095881 0.010824 0.044486 0.569579
## 3 1003 OKI 0.248873 0.063590 0.011806 0.012550 0.663181
## 4 1004 OKI 0.243349 0.010864 0.005469 0.033171 0.707148
## 5 1005 OKI 0.232079 0.001506 0.000010 0.034445 0.731960
## 6 1006 OKI 0.249958 0.040899 0.015095 0.038120 0.655927  
 Rename the columns 
       # Rename the columns starting from the third one  
   k5run1  <-  k5run1  |>  
      rename_with ( ~  paste0 ( "v" ,  seq_along (.x)),  .cols =   -  c (ind, pop)) 
    
    # View the first few rows  
    head (k5run1)    
  ##    ind pop       v1       v2       v3       v4       v5
## 1 1001 OKI 0.317249 0.070658 0.000160 0.052193 0.559741
## 2 1002 OKI 0.279230 0.095881 0.010824 0.044486 0.569579
## 3 1003 OKI 0.248873 0.063590 0.011806 0.012550 0.663181
## 4 1004 OKI 0.243349 0.010864 0.005469 0.033171 0.707148
## 5 1005 OKI 0.232079 0.001506 0.000010 0.034445 0.731960
## 6 1006 OKI 0.249958 0.040899 0.015095 0.038120 0.655927  
 Import samples attributes 
      sampling_loc  <-   readRDS ( here ( "output" ,  "populations" ,  "sampling_loc.rds" )) 
    # head(sampling_loc)  
    
   pops  <-  sampling_loc  |>  
      filter ( 
       Region  ==   "Asia"  
     )  |>  
     dplyr ::  select ( 
       Abbreviation, Latitude, Longitude, Pop_City, Country 
     ) 
    
    head (pops)    
  ## # A tibble: 6 × 5
##   Abbreviation Latitude Longitude Pop_City   Country 
##   <chr>           <dbl>     <dbl> <chr>      <chr>   
## 1 GEL              26.9      90.5 Gelephu    Bhutan  
## 2 CAM              11.6     105.  Phnom Penh Cambodia
## 3 HAI              19.2     110.  Hainan     China   
## 4 YUN              24.5     101.  Yunnan     China   
## 5 HUN              27.6     112.  Hunan      China   
## 6 BEN              13.0      77.6 Bengaluru  India  
       # Add an index column to Q_tibble  
   k5run1 $ index  <-   seq_len ( nrow (k5run1)) 
    
    # Perform the merge as before  
   df1  <-  
      merge ( 
       k5run1, 
       pops, 
        by.x =   2 , 
        by.y =   1 , 
        all.x =  T, 
        all.y =  F 
     )  |>  
      na.omit () 
    
    # Order by the index column to ensure the order matches the original Q_tibble  
   df1  <-  df1[ order (df1 $ index),] 
    
    # Optionally, you can remove the index column if it's no longer needed  
   df1 $ index  <-   NULL  
    
    # Now the rows of df1 should be in the same order as the original Q_tibble  
    head (df1)    
  ##     pop  ind       v1       v2       v3       v4       v5 Latitude Longitude
## 159 OKI 1001 0.317249 0.070658 0.000160 0.052193 0.559741  26.5013  127.9454
## 160 OKI 1002 0.279230 0.095881 0.010824 0.044486 0.569579  26.5013  127.9454
## 161 OKI 1003 0.248873 0.063590 0.011806 0.012550 0.663181  26.5013  127.9454
## 162 OKI 1004 0.243349 0.010864 0.005469 0.033171 0.707148  26.5013  127.9454
## 163 OKI 1005 0.232079 0.001506 0.000010 0.034445 0.731960  26.5013  127.9454
## 164 OKI 1006 0.249958 0.040899 0.015095 0.038120 0.655927  26.5013  127.9454
##     Pop_City Country
## 159  Okinawa   Japan
## 160  Okinawa   Japan
## 161  Okinawa   Japan
## 162  Okinawa   Japan
## 163  Okinawa   Japan
## 164  Okinawa   Japan  
 We used this color palette to make the “structure” plot 
      color_palette2  <-  
      c ( 
        "v1"   =   "#FFB347" , 
        "v2"   =   "#F49AC2" , 
        "v3"   =   "red" , 
        "v4"   =   "#FFFF99" , 
        "v5"   =   "#AE9393"  
     )    
      world  <-   ne_countries ( scale =   "medium" ,  returnclass =   "sf" ) 
   countries_with_data  <-   unique (df1 $ Country) 
    
    # Filtering the world data to include only the countries in your data  
   selected_countries  <-  world  |>  
      filter (admin  %in%  countries_with_data) 
    
    # Calculate mean proportions for each population  
   df_mean  <-  df1  |>  
      group_by (pop)  |>  
      summarise ( across ( starts_with ( "v" ), \(x)  mean (x,  na.rm =   TRUE )),  
                Longitude =   mean (Longitude), 
                Latitude =   mean (Latitude)) 
    
    
    source ( 
      here ( 
        "scripts" ,  "analysis" ,  "my_theme2.R"  
     ) 
   ) 
    
    ggplot ()  +  
      geom_sf ( data =  selected_countries,  fill=  "white" )  +  
      geom_scatterpie ( data =  df_mean,  
                      aes ( x =  Longitude,  y =  Latitude,  r =   1.5 ),  
                      cols =   c ( "v1" ,  "v2" ,  "v3" ,  "v4" ,  "v5" ),  color =   NA )  +  
      geom_text_repel ( data =  df_mean, 
                      aes ( x =  Longitude,  y =  Latitude,  label =  pop),  
                      size =   3 ,  
                      box.padding =   unit ( 0.5 ,  "lines" ), 
                      max.overlaps =   50 )  +  
      scale_fill_manual ( values =  color_palette2)  +  
      guides ( fill =   "none" )  +    # Hide legend  
      # coord_sf() +  
      coord_sf ( xlim =   c ( 60 ,  150 ),  ylim =   c ( -  10 ,  60 ))  +  
      my_theme ()    
   
       # #   
    ggsave ( 
      here ( "output" ,  "populations" ,  "figures" ,  "admixture_r2_0.01_k5_pie.pdf" ), 
      width  =   12 , 
      height =   6 , 
      units  =   "in" , 
      device =  cairo_pdf 
   )    
 
 13.1 Preparing the data for tess3Q_map_rasters 
 Make sure the lat longs are in the correct order and arrangement 
      df2  <-  df1  |>  
     dplyr ::  rename ( 
        Long =  Longitude, 
        Lat =  Latitude 
     ) 
    
   long_lat_tibble  <-  df2  |>  
     dplyr ::  select (Long, Lat) 
    
    
   long_lat_matrix  <-  long_lat_tibble  |>  
      as.matrix () 
    
    head (long_lat_matrix)    
  ##         Long     Lat
## 159 127.9454 26.5013
## 160 127.9454 26.5013
## 161 127.9454 26.5013
## 162 127.9454 26.5013
## 163 127.9454 26.5013
## 164 127.9454 26.5013  
 
 
 13.2 make a matrix of the Q values 
 Pull off the names of individuals and make a matrix of it: 
      Q_matrix  <-  k5run1  |>  
     dplyr ::  select ( - ind,  - pop,  - index)  |>  
      as.matrix () 
    head (Q_matrix)    
  ##            v1       v2       v3       v4       v5
## [1,] 0.317249 0.070658 0.000160 0.052193 0.559741
## [2,] 0.279230 0.095881 0.010824 0.044486 0.569579
## [3,] 0.248873 0.063590 0.011806 0.012550 0.663181
## [4,] 0.243349 0.010864 0.005469 0.033171 0.707148
## [5,] 0.232079 0.001506 0.000010 0.034445 0.731960
## [6,] 0.249958 0.040899 0.015095 0.038120 0.655927  
 
 
 13.3 Interpolate the Q-values by Kriging 
       print ( ncol (Q_matrix)  ==   length (color_palette2))    
  ## [1] TRUE  
 Create brick 
      genoscape_brick  <-  tess3r ::  tess3Q_map_rasters ( 
      x =  Q_matrix,  
      coord =  long_lat_matrix,   
      map.polygon =  selected_countries, 
      window =   extent (selected_countries)[ 1  :  4 ], 
      # window = combined_extent,  
      resolution =   c ( 600 , 600 ),  # if you want more cells in your raster, set higher  
      # this next lines need to to be here, but don't do much...  
      col.palette =  tess3r ::  CreatePalette (color_palette2,  length (color_palette2)), 
      method =   "map.max" ,  
      interpol =  tess3r ::  FieldsKrigModel ( 40 ),   
      main =   "Ancestry coefficients" , 
      xlab =   "Longitude" ,  
      ylab =   "Latitude" ,  
      cex =  . 4  
   )    
  ## Warning: 
## Grid searches over lambda (nugget and sill variances) with  minima at the endpoints: 
##   (REML) Restricted maximum likelihood 
##    minimum at  right endpoint  lambda  =  0.04206056 (eff. df= 26.60001 )
## Warning: 
## Grid searches over lambda (nugget and sill variances) with  minima at the endpoints: 
##   (REML) Restricted maximum likelihood 
##    minimum at  right endpoint  lambda  =  0.04206056 (eff. df= 26.60001 )
## Warning: 
## Grid searches over lambda (nugget and sill variances) with  minima at the endpoints: 
##   (REML) Restricted maximum likelihood 
##    minimum at  right endpoint  lambda  =  0.04206056 (eff. df= 26.60001 )
## Warning: 
## Grid searches over lambda (nugget and sill variances) with  minima at the endpoints: 
##   (REML) Restricted maximum likelihood 
##    minimum at  right endpoint  lambda  =  0.04206056 (eff. df= 26.60001 )
## Warning: 
## Grid searches over lambda (nugget and sill variances) with  minima at the endpoints: 
##   (REML) Restricted maximum likelihood 
##    minimum at  right endpoint  lambda  =  0.04206056 (eff. df= 26.60001 )  
       # after that, we need to add names of the clusters back onto this raster brick  
   Q_tibble2  <-  k5run1  |>  
     dplyr ::  select ( 
        - pop,  - ind,  - index 
     ) 
    names (genoscape_brick)  <-   names (Q_tibble2)[]    
 
 
 13.4 Scaling and cleaning the genoscape_brick 
      genoscape_rgba  <-  genoscapeRtools ::  qprob_rando_raster ( 
      TRB =  genoscape_brick, 
      cols =  color_palette2, 
      alpha_scale =   2.0 , 
      abs_thresh =   0.0 , 
      alpha_exp =   1.55 , 
      alpha_chop_max =   255  
   ) 
    
    # This adds the info for a regular lat-long projection  
    crs (genoscape_rgba)  <-   "+proj=longlat +datum=WGS84 +no_defs +ellps=WGS84 +towgs84=0,0,0"     
 We can easily plot this with the function layer_spatial from the
ggspatial package: 
       ggplot ()  +   
     ggspatial ::  layer_spatial (genoscape_rgba)  +   
      my_theme ()  +  
      coord_sf ()    
 Plot 
       ggplot ()  +  
      layer_spatial (genoscape_rgba)  +  
      geom_spatial_point ( data =  long_lat_tibble, 
                         mapping =   aes ( x =  Long,  y =  Lat), 
                         size =  . 2 )  +  
      geom_text_repel ( 
        data =  df_mean, 
        aes ( x =  Longitude,  y =  Latitude,  label =  pop), 
        size =   3 , 
        box.padding =   unit ( 0.5 ,  "lines" ) 
     )  +  
      labs ( x =   "Longitude" , 
           y =   "Latitude" )  +  
      geom_scatterpie ( 
        data =  df_mean, 
        aes ( x =  Longitude,  y =  Latitude,  r =   1 ), 
        cols =   c ( "v1" ,  "v2" ,  "v3" ,  "v4" ,  "v5" ), 
        color =   NA  
     )  +  
      my_theme ()  +  
      scale_fill_manual ( values =  color_palette2)  +  
      guides ( fill =   "none" )  +    # Hide legend  
      coord_sf ()    
  ## Assuming `crs = 4326` in stat_spatial_identity()  
   
       ggsave ( 
      here ( "output" ,  "populations" ,  "figures" ,  "admixture_r2_0.01_k5_interpolated_pie.pdf" ), 
      width  =   12 , 
      height =   6 , 
      units  =   "in" , 
      device =  cairo_pdf 
   )    
  ## Assuming `crs = 4326` in stat_spatial_identity()  
 
 
 
 14. Admixture r2 0.1 SNPs k5 
 Clear memory and environment 
       # Clear entire environment  
    rm ( list =   ls ()) 
    # Forcefully trigger garbage collection  
    gc ()    
  ##           used  (Mb) gc trigger  (Mb) limit (Mb) max used  (Mb)
## Ncells 4698558 251.0   12802251 683.8         NA 12802251 683.8
## Vcells 8803798  67.2   38932664 297.1      32768 68732428 524.4  
 Make plot 
       # Extract ancestry coefficients  
   k5run1  <-   read_delim ( 
      here ( "output" ,  "populations" ,  "admixture" ,  "r2_0.1" ,  "run1" ,  "r2_0.1.5.Q" ), 
      delim =   " " ,  # Specify the delimiter if different from the default (comma)  
      col_names =   FALSE , 
      show_col_types =   FALSE  
   )  
    
    head (k5run1)    
  ## # A tibble: 6 × 5
##      X1      X2      X3      X4    X5
##   <dbl>   <dbl>   <dbl>   <dbl> <dbl>
## 1 0.248 0.129   0.00001 0.00912 0.614
## 2 0.220 0.145   0.00001 0.0184  0.616
## 3 0.181 0.0975  0.00001 0.00001 0.722
## 4 0.147 0.0112  0.00001 0.00516 0.837
## 5 0.105 0.00001 0.00001 0.00001 0.895
## 6 0.171 0.0981  0.00001 0.00001 0.731  
 The fam file 
      fam_file  <-   here ( 
      "output" ,  "populations" ,  "snps_sets" ,  "r2_0.1.fam"  
   ) 
    
    # Read the .fam file  
   fam_data  <-   read.table (fam_file,  
                           header =   FALSE , 
                           col.names =   c ( "FamilyID" ,  "IndividualID" ,  "PaternalID" ,  "MaternalID" ,  "Sex" ,  "Phenotype" )) 
    
    # View the first few rows  
    head (fam_data)    
  ##   FamilyID IndividualID PaternalID MaternalID Sex Phenotype
## 1      OKI         1001          0          0   2        -9
## 2      OKI         1002          0          0   2        -9
## 3      OKI         1003          0          0   2        -9
## 4      OKI         1004          0          0   2        -9
## 5      OKI         1005          0          0   2        -9
## 6      OKI         1006          0          0   1        -9  
 Create ID column 
       # Change column name  
    colnames (fam_data)[ colnames (fam_data)  ==   "IndividualID" ]  <-   "ind"  
    
    # Change column name  
    colnames (fam_data)[ colnames (fam_data)  ==   "FamilyID" ]  <-   "pop"  
    
    # Select ID  
   fam_data  <-  fam_data  |>  
     dplyr ::  select ( "ind" ,  "pop" ) 
    
    # View the first few rows  
    head (fam_data)    
  ##    ind pop
## 1 1001 OKI
## 2 1002 OKI
## 3 1003 OKI
## 4 1004 OKI
## 5 1005 OKI
## 6 1006 OKI  
 Add it to matrix 
      k5run1  <-  fam_data  |>  
     dplyr ::  select (ind, pop)  |>  
      bind_cols (k5run1) 
    
    head (k5run1)    
  ##    ind pop       X1       X2    X3       X4       X5
## 1 1001 OKI 0.247788 0.128696 1e-05 0.009116 0.614391
## 2 1002 OKI 0.220143 0.145400 1e-05 0.018377 0.616069
## 3 1003 OKI 0.180728 0.097481 1e-05 0.000010 0.721772
## 4 1004 OKI 0.146729 0.011211 1e-05 0.005160 0.836890
## 5 1005 OKI 0.105331 0.000010 1e-05 0.000010 0.894639
## 6 1006 OKI 0.171413 0.098054 1e-05 0.000010 0.730513  
 Rename the columns 
       # Rename the columns starting from the third one  
   k5run1  <-  k5run1  |>  
      rename_with ( ~  paste0 ( "v" ,  seq_along (.x)),  .cols =   -  c (ind, pop)) 
    
    # View the first few rows  
    head (k5run1)    
  ##    ind pop       v1       v2    v3       v4       v5
## 1 1001 OKI 0.247788 0.128696 1e-05 0.009116 0.614391
## 2 1002 OKI 0.220143 0.145400 1e-05 0.018377 0.616069
## 3 1003 OKI 0.180728 0.097481 1e-05 0.000010 0.721772
## 4 1004 OKI 0.146729 0.011211 1e-05 0.005160 0.836890
## 5 1005 OKI 0.105331 0.000010 1e-05 0.000010 0.894639
## 6 1006 OKI 0.171413 0.098054 1e-05 0.000010 0.730513  
 Import samples attributes 
      sampling_loc  <-   readRDS ( here ( "output" ,  "populations" ,  "sampling_loc.rds" )) 
    # head(sampling_loc)  
    
   pops  <-  sampling_loc  |>  
      filter ( 
       Region  ==   "Asia"  
     )  |>  
     dplyr ::  select ( 
       Abbreviation, Latitude, Longitude, Pop_City, Country 
     ) 
    
    head (pops)    
  ## # A tibble: 6 × 5
##   Abbreviation Latitude Longitude Pop_City   Country 
##   <chr>           <dbl>     <dbl> <chr>      <chr>   
## 1 GEL              26.9      90.5 Gelephu    Bhutan  
## 2 CAM              11.6     105.  Phnom Penh Cambodia
## 3 HAI              19.2     110.  Hainan     China   
## 4 YUN              24.5     101.  Yunnan     China   
## 5 HUN              27.6     112.  Hunan      China   
## 6 BEN              13.0      77.6 Bengaluru  India  
       # Add an index column to Q_tibble  
   k5run1 $ index  <-   seq_len ( nrow (k5run1)) 
    
    # Perform the merge as before  
   df1  <-  
      merge ( 
       k5run1, 
       pops, 
        by.x =   2 , 
        by.y =   1 , 
        all.x =  T, 
        all.y =  F 
     )  |>  
      na.omit () 
    
    # Order by the index column to ensure the order matches the original Q_tibble  
   df1  <-  df1[ order (df1 $ index),] 
    
    # Optionally, you can remove the index column if it's no longer needed  
   df1 $ index  <-   NULL  
    
    # Now the rows of df1 should be in the same order as the original Q_tibble  
    head (df1)    
  ##     pop  ind       v1       v2    v3       v4       v5 Latitude Longitude
## 159 OKI 1001 0.247788 0.128696 1e-05 0.009116 0.614391  26.5013  127.9454
## 160 OKI 1002 0.220143 0.145400 1e-05 0.018377 0.616069  26.5013  127.9454
## 161 OKI 1003 0.180728 0.097481 1e-05 0.000010 0.721772  26.5013  127.9454
## 162 OKI 1004 0.146729 0.011211 1e-05 0.005160 0.836890  26.5013  127.9454
## 163 OKI 1005 0.105331 0.000010 1e-05 0.000010 0.894639  26.5013  127.9454
## 164 OKI 1006 0.171413 0.098054 1e-05 0.000010 0.730513  26.5013  127.9454
##     Pop_City Country
## 159  Okinawa   Japan
## 160  Okinawa   Japan
## 161  Okinawa   Japan
## 162  Okinawa   Japan
## 163  Okinawa   Japan
## 164  Okinawa   Japan  
 We used this color palette to make the “structure” plot 
      color_palette2  <-  
      c ( 
        "v1"   =   "#FFB347" , 
        "v2"   =   "#F49AC2" , 
        "v3"   =   "red" , 
        "v4"   =   "#FFFF99" , 
        "v5"   =   "#AE9393"  
     )    
      world  <-   ne_countries ( scale =   "medium" ,  returnclass =   "sf" ) 
   countries_with_data  <-   unique (df1 $ Country) 
    
    # Filtering the world data to include only the countries in your data  
   selected_countries  <-  world  |>  
      filter (admin  %in%  countries_with_data) 
    
    # Calculate mean proportions for each population  
   df_mean  <-  df1  |>  
      group_by (pop)  |>  
      summarise ( across ( starts_with ( "v" ), \(x)  mean (x,  na.rm =   TRUE )),  
                Longitude =   mean (Longitude), 
                Latitude =   mean (Latitude)) 
    
    
    source ( 
      here ( 
        "scripts" ,  "analysis" ,  "my_theme2.R"  
     ) 
   ) 
    
    ggplot ()  +  
      geom_sf ( data =  selected_countries,  fill=  "white" )  +  
      geom_scatterpie ( data =  df_mean,  
                      aes ( x =  Longitude,  y =  Latitude,  r =   1.5 ),  
                      cols =   c ( "v1" ,  "v2" ,  "v3" ,  "v4" ,  "v5" ),  color =   NA )  +  
      geom_text_repel ( data =  df_mean, 
                      aes ( x =  Longitude,  y =  Latitude,  label =  pop),  
                      size =   3 ,  
                      box.padding =   unit ( 0.5 ,  "lines" ), 
                      max.overlaps =   50 )  +  
      scale_fill_manual ( values =  color_palette2)  +  
      guides ( fill =   "none" )  +    # Hide legend  
      # coord_sf() +  
      coord_sf ( xlim =   c ( 60 ,  150 ),  ylim =   c ( -  10 ,  60 ))  +  
      my_theme ()    
   
       # #   
    ggsave ( 
      here ( "output" ,  "populations" ,  "figures" ,  "admixture_r2_0.1_k5_pie.pdf" ), 
      width  =   12 , 
      height =   6 , 
      units  =   "in" , 
      device =  cairo_pdf 
   )    
 
 14.1 Preparing the data for tess3Q_map_rasters 
 Make sure the lat longs are in the correct order and arrangement 
      df2  <-  df1  |>  
     dplyr ::  rename ( 
        Long =  Longitude, 
        Lat =  Latitude 
     ) 
    
   long_lat_tibble  <-  df2  |>  
     dplyr ::  select (Long, Lat) 
    
    
   long_lat_matrix  <-  long_lat_tibble  |>  
      as.matrix () 
    
    head (long_lat_matrix)    
  ##         Long     Lat
## 159 127.9454 26.5013
## 160 127.9454 26.5013
## 161 127.9454 26.5013
## 162 127.9454 26.5013
## 163 127.9454 26.5013
## 164 127.9454 26.5013  
 
 
 14.2 make a matrix of the Q values 
 Pull off the names of individuals and make a matrix of it: 
      Q_matrix  <-  k5run1  |>  
     dplyr ::  select ( - ind,  - pop,  - index)  |>  
      as.matrix () 
    head (Q_matrix)    
  ##            v1       v2    v3       v4       v5
## [1,] 0.247788 0.128696 1e-05 0.009116 0.614391
## [2,] 0.220143 0.145400 1e-05 0.018377 0.616069
## [3,] 0.180728 0.097481 1e-05 0.000010 0.721772
## [4,] 0.146729 0.011211 1e-05 0.005160 0.836890
## [5,] 0.105331 0.000010 1e-05 0.000010 0.894639
## [6,] 0.171413 0.098054 1e-05 0.000010 0.730513  
 
 
 14.3 Interpolate the Q-values by Kriging 
       print ( ncol (Q_matrix)  ==   length (color_palette2))    
  ## [1] TRUE  
 Create brick 
      genoscape_brick  <-  tess3r ::  tess3Q_map_rasters ( 
      x =  Q_matrix,  
      coord =  long_lat_matrix,   
      map.polygon =  selected_countries, 
      window =   extent (selected_countries)[ 1  :  4 ], 
      # window = combined_extent,  
      resolution =   c ( 600 , 600 ),  # if you want more cells in your raster, set higher  
      # this next lines need to to be here, but don't do much...  
      col.palette =  tess3r ::  CreatePalette (color_palette2,  length (color_palette2)), 
      method =   "map.max" ,  
      interpol =  tess3r ::  FieldsKrigModel ( 40 ),   
      main =   "Ancestry coefficients" , 
      xlab =   "Longitude" ,  
      ylab =   "Latitude" ,  
      cex =  . 4  
   )    
  ## Warning: 
## Grid searches over lambda (nugget and sill variances) with  minima at the endpoints: 
##   (REML) Restricted maximum likelihood 
##    minimum at  right endpoint  lambda  =  0.04206056 (eff. df= 26.60001 )
## Warning: 
## Grid searches over lambda (nugget and sill variances) with  minima at the endpoints: 
##   (REML) Restricted maximum likelihood 
##    minimum at  right endpoint  lambda  =  0.04206056 (eff. df= 26.60001 )
## Warning: 
## Grid searches over lambda (nugget and sill variances) with  minima at the endpoints: 
##   (REML) Restricted maximum likelihood 
##    minimum at  right endpoint  lambda  =  0.04206056 (eff. df= 26.60001 )
## Warning: 
## Grid searches over lambda (nugget and sill variances) with  minima at the endpoints: 
##   (REML) Restricted maximum likelihood 
##    minimum at  right endpoint  lambda  =  0.04206056 (eff. df= 26.60001 )
## Warning: 
## Grid searches over lambda (nugget and sill variances) with  minima at the endpoints: 
##   (REML) Restricted maximum likelihood 
##    minimum at  right endpoint  lambda  =  0.04206056 (eff. df= 26.60001 )  
       # after that, we need to add names of the clusters back onto this raster brick  
   Q_tibble2  <-  k5run1  |>  
     dplyr ::  select ( 
        - pop,  - ind,  - index 
     ) 
    names (genoscape_brick)  <-   names (Q_tibble2)[]    
 
 
 14.4 Scaling and cleaning the genoscape_brick 
      genoscape_rgba  <-  genoscapeRtools ::  qprob_rando_raster ( 
      TRB =  genoscape_brick, 
      cols =  color_palette2, 
      alpha_scale =   2.0 , 
      abs_thresh =   0.0 , 
      alpha_exp =   1.55 , 
      alpha_chop_max =   255  
   ) 
    
    crs (genoscape_rgba)  <-   "+proj=longlat +datum=WGS84 +no_defs +ellps=WGS84 +towgs84=0,0,0"     
 Plot 
       ggplot ()  +   
     ggspatial ::  layer_spatial (genoscape_rgba)  +   
      my_theme ()  +  
      coord_sf ()    
 Plot 
       ggplot ()  +  
      layer_spatial (genoscape_rgba)  +  
      geom_spatial_point ( data =  long_lat_tibble, 
                         mapping =   aes ( x =  Long,  y =  Lat), 
                         size =  . 2 )  +  
      geom_text_repel ( 
        data =  df_mean, 
        aes ( x =  Longitude,  y =  Latitude,  label =  pop), 
        size =   3 , 
        box.padding =   unit ( 0.5 ,  "lines" ) 
     )  +  
      labs ( x =   "Longitude" , 
           y =   "Latitude" )  +  
      geom_scatterpie ( 
        data =  df_mean, 
        aes ( x =  Longitude,  y =  Latitude,  r =   1 ), 
        cols =   c ( "v1" ,  "v2" ,  "v3" ,  "v4" ,  "v5" ), 
        color =   NA  
     )  +  
      my_theme ()  +  
      scale_fill_manual ( values =  color_palette2)  +  
      guides ( fill =   "none" )  +    # Hide legend  
      coord_sf ()    
  ## Assuming `crs = 4326` in stat_spatial_identity()  
   
       ggsave ( 
      here ( "output" ,  "populations" ,  "figures" ,  "admixture_r2_0.1_k5_interpolated_pie.pdf" ), 
      width  =   12 , 
      height =   6 , 
      units  =   "in" , 
      device =  cairo_pdf 
   )    
  ## Assuming `crs = 4326` in stat_spatial_identity()  
 
 
 
 15. neuro-admxiture r2 0.01 SNPs k5 
 Clear memory and environment 
       # Clear entire environment  
    rm ( list =   ls ()) 
    # Forcefully trigger garbage collection  
    gc ()    
  ##           used  (Mb) gc trigger  (Mb) limit (Mb) max used  (Mb)
## Ncells 4698874 251.0   12802251 683.8         NA 12802251 683.8
## Vcells 8805369  67.2   36115607 275.6      32768 68732428 524.4  
 
 15.1 Q-values 
 Import Q 
       # Extract ancestry coefficients  
   nadmixk5  <-   read_delim ( 
      here ( "output" ,  "populations" ,  "nadmix" ,  "results" ,  "r_0.01" , "r_0.01_inference.5.Q" ), 
      delim =   " " ,  # Specify the delimiter if different from the default (comma)  
      col_names =   FALSE , 
      show_col_types =   FALSE  
   )  
    # unseen_pckmeans.7.Q  
    # pckmeans.7.Q  
    head (nadmixk5)    
  ## # A tibble: 6 × 5
##       X1    X2    X3     X4     X5
##    <dbl> <dbl> <dbl>  <dbl>  <dbl>
## 1 0.0203 0.701 0.152 0.109  0.0173
## 2 0.0191 0.682 0.184 0.102  0.0130
## 3 0.0163 0.706 0.151 0.0845 0.0416
## 4 0.0341 0.671 0.167 0.109  0.0193
## 5 0.0389 0.692 0.168 0.0892 0.0128
## 6 0.0162 0.714 0.171 0.0784 0.0202  
 The fam file 
      fam_file  <-   here ( 
      "output" ,  "populations" ,  "snps_sets" ,  "r2_0.01.fam"  
   ) 
    
    # Read the .fam file  
   fam_data  <-   read.table (fam_file,  
                           header =   FALSE , 
                           col.names =   c ( "FamilyID" ,  "IndividualID" ,  "PaternalID" ,  "MaternalID" ,  "Sex" ,  "Phenotype" )) 
    
    # View the first few rows  
    head (fam_data)    
  ##   FamilyID IndividualID PaternalID MaternalID Sex Phenotype
## 1      OKI         1001          0          0   2        -9
## 2      OKI         1002          0          0   2        -9
## 3      OKI         1003          0          0   2        -9
## 4      OKI         1004          0          0   2        -9
## 5      OKI         1005          0          0   2        -9
## 6      OKI         1006          0          0   1        -9  
 Create ID column 
       # Change column name  
    colnames (fam_data)[ colnames (fam_data)  ==   "IndividualID" ]  <-   "ind"  
    
    
    # Merge columns "FamilyID" and "IndividualID" with an underscore  
    # fam_data$ind <- paste(fam_data$FamilyID, fam_data$IndividualID, sep = "_")  
    
    
    # Change column name  
    colnames (fam_data)[ colnames (fam_data)  ==   "FamilyID" ]  <-   "pop"  
    
    # Select ID  
   fam_data  <-  fam_data  |>  
     dplyr ::  select ( "ind" ,  "pop" ) 
    
    # View the first few rows  
    head (fam_data)    
  ##    ind pop
## 1 1001 OKI
## 2 1002 OKI
## 3 1003 OKI
## 4 1004 OKI
## 5 1005 OKI
## 6 1006 OKI  
 Add it to matrix 
      nadmixk5  <-  fam_data  |>  
     dplyr ::  select (ind, pop)  |>  
      bind_cols (nadmixk5) 
    
    head (nadmixk5)    
  ##    ind pop         X1        X2        X3         X4         X5
## 1 1001 OKI 0.02026024 0.7009754 0.1522250 0.10923769 0.01730163
## 2 1002 OKI 0.01906374 0.6816174 0.1840338 0.10228224 0.01300276
## 3 1003 OKI 0.01627573 0.7062859 0.1513138 0.08453301 0.04159156
## 4 1004 OKI 0.03413134 0.6708513 0.1668813 0.10887521 0.01926081
## 5 1005 OKI 0.03889918 0.6916250 0.1675111 0.08921071 0.01275407
## 6 1006 OKI 0.01619347 0.7141496 0.1710819 0.07837918 0.02019598  
 Rename the columns 
       # Rename the columns starting from the third one  
   nadmixk5  <-  nadmixk5  |>  
      rename_with ( ~  paste0 ( "v" ,  seq_along (.x)),  .cols =   -  c (ind, pop)) 
    
    # View the first few rows  
    head (nadmixk5)    
  ##    ind pop         v1        v2        v3         v4         v5
## 1 1001 OKI 0.02026024 0.7009754 0.1522250 0.10923769 0.01730163
## 2 1002 OKI 0.01906374 0.6816174 0.1840338 0.10228224 0.01300276
## 3 1003 OKI 0.01627573 0.7062859 0.1513138 0.08453301 0.04159156
## 4 1004 OKI 0.03413134 0.6708513 0.1668813 0.10887521 0.01926081
## 5 1005 OKI 0.03889918 0.6916250 0.1675111 0.08921071 0.01275407
## 6 1006 OKI 0.01619347 0.7141496 0.1710819 0.07837918 0.02019598  
 Import samples attributes 
      sampling_loc  <-   readRDS ( here ( "output" ,  "populations" ,  "sampling_loc.rds" )) 
    # head(sampling_loc)  
    
   pops  <-  sampling_loc  |>  
      filter ( 
       Region  ==   "Asia"  
     )  |>  
     dplyr ::  select ( 
       Abbreviation, Latitude, Longitude, Pop_City, Country 
     ) 
    
    head (pops)    
  ## # A tibble: 6 × 5
##   Abbreviation Latitude Longitude Pop_City   Country 
##   <chr>           <dbl>     <dbl> <chr>      <chr>   
## 1 GEL              26.9      90.5 Gelephu    Bhutan  
## 2 CAM              11.6     105.  Phnom Penh Cambodia
## 3 HAI              19.2     110.  Hainan     China   
## 4 YUN              24.5     101.  Yunnan     China   
## 5 HUN              27.6     112.  Hunan      China   
## 6 BEN              13.0      77.6 Bengaluru  India  
 Merge with pops 
       # Add an index column to Q_tibble  
   nadmixk5 $ index  <-   seq_len ( nrow (nadmixk5)) 
    
    # Perform the merge as before  
   df1  <-  
      merge ( 
       nadmixk5, 
       pops, 
        by.x =   2 , 
        by.y =   1 , 
        all.x =  T, 
        all.y =  F 
     )  |>  
      na.omit () 
    
    # Order by the index column to ensure the order matches the original Q_tibble  
   df1  <-  df1[ order (df1 $ index),] 
    
    # Optionally, you can remove the index column if it's no longer needed  
   df1 $ index  <-   NULL  
    
    # Now the rows of df1 should be in the same order as the original Q_tibble  
    head (df1)    
  ##     pop  ind         v1        v2        v3         v4         v5 Latitude
## 159 OKI 1001 0.02026024 0.7009754 0.1522250 0.10923769 0.01730163  26.5013
## 160 OKI 1002 0.01906374 0.6816174 0.1840338 0.10228224 0.01300276  26.5013
## 161 OKI 1003 0.01627573 0.7062859 0.1513138 0.08453301 0.04159156  26.5013
## 162 OKI 1004 0.03413134 0.6708513 0.1668813 0.10887521 0.01926081  26.5013
## 163 OKI 1005 0.03889918 0.6916250 0.1675111 0.08921071 0.01275407  26.5013
## 164 OKI 1006 0.01619347 0.7141496 0.1710819 0.07837918 0.02019598  26.5013
##     Longitude Pop_City Country
## 159  127.9454  Okinawa   Japan
## 160  127.9454  Okinawa   Japan
## 161  127.9454  Okinawa   Japan
## 162  127.9454  Okinawa   Japan
## 163  127.9454  Okinawa   Japan
## 164  127.9454  Okinawa   Japan  
 We used this color palette to make the “structure” plot 
      color_palette2  <-  
      c ( 
        "v1"   =   "red" , 
        "v2"   =   "#AE9393" , 
        "v3"   =   "#F49AC2" , 
        "v4"   =   "#FFB347" , 
        "v5"   =   "#FFFF99"  
     )    
 Make pie plot 
      world  <-   ne_countries ( scale =   "medium" ,  returnclass =   "sf" ) 
   countries_with_data  <-   unique (df1 $ Country) 
    
    # Filtering the world data to include only the countries in your data  
   selected_countries  <-  world  |>  
      filter (admin  %in%  countries_with_data) 
    
    # Calculate mean proportions for each population  
   df_mean  <-  df1  |>  
      group_by (pop)  |>  
      summarise ( across ( starts_with ( "v" ), \(x)  mean (x,  na.rm =   TRUE )),  
                Longitude =   mean (Longitude), 
                Latitude =   mean (Latitude)) 
    
    source ( 
      here ( 
        "scripts" ,  "analysis" ,  "my_theme2.R"  
     ) 
   ) 
    
    ggplot ()  +  
      geom_sf ( data =  selected_countries,  fill=  "white" )  +  
      geom_scatterpie ( data =  df_mean,  
                      aes ( x =  Longitude,  y =  Latitude,  r =   1.5 ),  
                      cols =   c ( "v1" ,  "v2" ,  "v3" ,  "v4" ,  "v5" ),  color =   NA )  +  
      geom_text_repel ( data =  df_mean, 
                      aes ( x =  Longitude,  y =  Latitude,  label =  pop),  
                      size =   3 ,  
                      box.padding =   unit ( 0.5 ,  "lines" ), 
                      max.overlaps =   50 )  +  
      scale_fill_manual ( values =  color_palette2)  +  
      guides ( fill =   "none" )  +    # Hide legend  
      # coord_sf() +  
      coord_sf ( xlim =   c ( 60 ,  150 ),  ylim =   c ( -  10 ,  60 ))  +  
      my_theme ()    
   
       # #   
    ggsave ( 
      here ( "output" ,  "populations" ,  "figures" ,  "neuro-admixture_r_0.01_k5_pie.pdf" ), 
      width  =   12 , 
      height =   6 , 
      units  =   "in" , 
      device =  cairo_pdf 
   )    
 
 
 15.2 Preparing the data for tess3Q_map_rasters 
      df2  <-  df1  |>  
     dplyr ::  rename ( 
        Long =  Longitude, 
        Lat =  Latitude 
     ) 
    
   long_lat_tibble  <-  df2  |>  
     dplyr ::  select (Long, Lat) 
    
    
   long_lat_matrix  <-  long_lat_tibble  |>  
      as.matrix () 
    
    head (long_lat_matrix)    
  ##         Long     Lat
## 159 127.9454 26.5013
## 160 127.9454 26.5013
## 161 127.9454 26.5013
## 162 127.9454 26.5013
## 163 127.9454 26.5013
## 164 127.9454 26.5013  
 
 
 15.3 make a matrix of the Q values 
 Pull off the names of individuals and make a matrix of it: 
      Q_matrix  <-  nadmixk5  |>  
     dplyr ::  select ( - ind,  - pop,  - index)  |>  
      as.matrix () 
    head (Q_matrix)    
  ##              v1        v2        v3         v4         v5
## [1,] 0.02026024 0.7009754 0.1522250 0.10923769 0.01730163
## [2,] 0.01906374 0.6816174 0.1840338 0.10228224 0.01300276
## [3,] 0.01627573 0.7062859 0.1513138 0.08453301 0.04159156
## [4,] 0.03413134 0.6708513 0.1668813 0.10887521 0.01926081
## [5,] 0.03889918 0.6916250 0.1675111 0.08921071 0.01275407
## [6,] 0.01619347 0.7141496 0.1710819 0.07837918 0.02019598  
 
 
 15.4 Interpolate the Q-values by Kriging 
       print ( ncol (Q_matrix)  ==   length (color_palette2))    
  ## [1] TRUE  
 Create brick 
      genoscape_brick  <-  tess3r ::  tess3Q_map_rasters ( 
      x =  Q_matrix,  
      coord =  long_lat_matrix,   
      map.polygon =  selected_countries, 
      window =   extent (selected_countries)[ 1  :  4 ], 
      # window = combined_extent,  
      resolution =   c ( 600 , 600 ),  # if you want more cells in your raster, set higher  
      # this next lines need to to be here, but don't do much...  
      col.palette =  tess3r ::  CreatePalette (color_palette2,  length (color_palette2)), 
      method =   "map.max" ,  
      interpol =  tess3r ::  FieldsKrigModel ( 80 ),   
      main =   "Ancestry coefficients" , 
      xlab =   "Longitude" ,  
      ylab =   "Latitude" ,  
      cex =  . 4  
   )    
  ## Warning: 
## Grid searches over lambda (nugget and sill variances) with  minima at the endpoints: 
##   (REML) Restricted maximum likelihood 
##    minimum at  right endpoint  lambda  =  0.02118404 (eff. df= 26.59999 )
## Warning: 
## Grid searches over lambda (nugget and sill variances) with  minima at the endpoints: 
##   (REML) Restricted maximum likelihood 
##    minimum at  right endpoint  lambda  =  0.02118404 (eff. df= 26.59999 )
## Warning: 
## Grid searches over lambda (nugget and sill variances) with  minima at the endpoints: 
##   (REML) Restricted maximum likelihood 
##    minimum at  right endpoint  lambda  =  0.02118404 (eff. df= 26.59999 )
## Warning: 
## Grid searches over lambda (nugget and sill variances) with  minima at the endpoints: 
##   (REML) Restricted maximum likelihood 
##    minimum at  right endpoint  lambda  =  0.02118404 (eff. df= 26.59999 )  
       # after that, we need to add names of the clusters back onto this raster brick  
   Q_tibble2  <-  nadmixk5  |>  
     dplyr ::  select ( 
        - pop,  - ind,  - index 
     ) 
    names (genoscape_brick)  <-   names (Q_tibble2)[]    
 
 
 15.5 Scaling and cleaning the genoscape_brick 
      genoscape_rgba  <-  genoscapeRtools ::  qprob_rando_raster ( 
      TRB =  genoscape_brick, 
      cols =  color_palette2, 
      alpha_scale =   2.0 , 
      abs_thresh =   0.0 , 
      alpha_exp =   1.55 , 
      alpha_chop_max =   255  
   ) 
    
    # This adds the info for a regular lat-long projection  
    crs (genoscape_rgba)  <-   "+proj=longlat +datum=WGS84 +no_defs +ellps=WGS84 +towgs84=0,0,0"     
 With pies 
       ggplot ()  +  
      layer_spatial (genoscape_rgba)  +  
      geom_spatial_point ( data =  long_lat_tibble, 
                         mapping =   aes ( x =  Long,  y =  Lat), 
                         size =  . 2 )  +  
      geom_text_repel ( 
        data =  df_mean, 
        aes ( x =  Longitude,  y =  Latitude,  label =  pop), 
        size =   3 , 
        box.padding =   unit ( 0.5 ,  "lines" ) 
     )  +  
      labs ( x =   "Longitude" , 
           y =   "Latitude" )  +  
      geom_scatterpie ( data =  df_mean,  
                      aes ( x =  Longitude,  y =  Latitude,  r =   1 ),  
                      cols =   c ( "v1" ,  "v2" ,  "v3" ,  "v4" ,  "v5" ),  color =   NA )  +  
      my_theme ()  +  
      scale_fill_manual ( values =  color_palette2)  +  
      guides ( fill =   "none" )  +    # Hide legend  
      coord_sf ()    
  ## Assuming `crs = 4326` in stat_spatial_identity()  
   
       # #   
    ggsave ( 
      here ( "output" ,  "populations" ,  "figures" ,  "neuro-admixture_r_0.01_k5_interpolated_pie.pdf" ), 
      width  =   12 , 
      height =   6 , 
      units  =   "in" , 
      device =  cairo_pdf 
   )    
  ## Assuming `crs = 4326` in stat_spatial_identity()  
 
 
 
 16. neuro-admxiture r2 0.1 SNPs k5 
 Clear memory and environment 
       # Clear entire environment  
    rm ( list =   ls ()) 
    # Forcefully trigger garbage collection  
    gc ()    
  ##           used  (Mb) gc trigger  (Mb) limit (Mb) max used  (Mb)
## Ncells 4699139 251.0   12802251 683.8         NA 12802251 683.8
## Vcells 8807075  67.2   41867203 319.5      32768 68732428 524.4  
 
 16.1 Q-values 
 Import Q 
       # Extract ancestry coefficients  
   nadmixk5  <-   read_delim ( 
      here ( "output" ,  "populations" ,  "nadmix" ,  "results" ,  "r_0.1" , "r_0.1_inference.5.Q" ), 
      delim =   " " ,  # Specify the delimiter if different from the default (comma)  
      col_names =   FALSE , 
      show_col_types =   FALSE  
   )  
    # unseen_pckmeans.7.Q  
    # pckmeans.7.Q  
    head (nadmixk5)    
  ## # A tibble: 6 × 5
##         X1    X2     X3     X4      X5
##      <dbl> <dbl>  <dbl>  <dbl>   <dbl>
## 1 0.00224  0.827 0.0444 0.113  0.0130 
## 2 0.000621 0.937 0.0307 0.0280 0.00339
## 3 0.00297  0.844 0.0415 0.103  0.00833
## 4 0.00247  0.784 0.0591 0.146  0.00809
## 5 0.00279  0.757 0.0703 0.159  0.0113 
## 6 0.00192  0.845 0.0404 0.101  0.0120  
 The fam file 
      fam_file  <-   here ( 
      "output" ,  "populations" ,  "snps_sets" ,  "r2_0.1.fam"  
   ) 
    
    # Read the .fam file  
   fam_data  <-   read.table (fam_file,  
                           header =   FALSE , 
                           col.names =   c ( "FamilyID" ,  "IndividualID" ,  "PaternalID" ,  "MaternalID" ,  "Sex" ,  "Phenotype" )) 
    
    # View the first few rows  
    head (fam_data)    
  ##   FamilyID IndividualID PaternalID MaternalID Sex Phenotype
## 1      OKI         1001          0          0   2        -9
## 2      OKI         1002          0          0   2        -9
## 3      OKI         1003          0          0   2        -9
## 4      OKI         1004          0          0   2        -9
## 5      OKI         1005          0          0   2        -9
## 6      OKI         1006          0          0   1        -9  
 Create ID column 
       # Change column name  
    colnames (fam_data)[ colnames (fam_data)  ==   "IndividualID" ]  <-   "ind"  
    
    # Change column name  
    colnames (fam_data)[ colnames (fam_data)  ==   "FamilyID" ]  <-   "pop"  
    
    # Select ID  
   fam_data  <-  fam_data  |>  
     dplyr ::  select ( "ind" ,  "pop" ) 
    
    # View the first few rows  
    head (fam_data)    
  ##    ind pop
## 1 1001 OKI
## 2 1002 OKI
## 3 1003 OKI
## 4 1004 OKI
## 5 1005 OKI
## 6 1006 OKI  
 Add it to matrix 
      nadmixk5  <-  fam_data  |>  
     dplyr ::  select (ind, pop)  |>  
      bind_cols (nadmixk5) 
    
    head (nadmixk5)    
  ##    ind pop           X1        X2         X3         X4          X5
## 1 1001 OKI 0.0022390699 0.8273290 0.04438876 0.11306997 0.012973181
## 2 1002 OKI 0.0006207919 0.9372209 0.03072406 0.02804254 0.003391742
## 3 1003 OKI 0.0029661185 0.8442711 0.04149344 0.10293802 0.008331385
## 4 1004 OKI 0.0024678661 0.7841588 0.05913275 0.14614794 0.008092790
## 5 1005 OKI 0.0027905770 0.7570373 0.07026709 0.15857665 0.011328328
## 6 1006 OKI 0.0019242963 0.8448561 0.04041177 0.10083950 0.011968327  
 Rename the columns 
       # Rename the columns starting from the third one  
   nadmixk5  <-  nadmixk5  |>  
      rename_with ( ~  paste0 ( "v" ,  seq_along (.x)),  .cols =   -  c (ind, pop)) 
    
    # View the first few rows  
    head (nadmixk5)    
  ##    ind pop           v1        v2         v3         v4          v5
## 1 1001 OKI 0.0022390699 0.8273290 0.04438876 0.11306997 0.012973181
## 2 1002 OKI 0.0006207919 0.9372209 0.03072406 0.02804254 0.003391742
## 3 1003 OKI 0.0029661185 0.8442711 0.04149344 0.10293802 0.008331385
## 4 1004 OKI 0.0024678661 0.7841588 0.05913275 0.14614794 0.008092790
## 5 1005 OKI 0.0027905770 0.7570373 0.07026709 0.15857665 0.011328328
## 6 1006 OKI 0.0019242963 0.8448561 0.04041177 0.10083950 0.011968327  
 Import samples attributes 
      sampling_loc  <-   readRDS ( here ( "output" ,  "populations" ,  "sampling_loc.rds" )) 
    # head(sampling_loc)  
    
   pops  <-  sampling_loc  |>  
      filter ( 
       Region  ==   "Asia"  
     )  |>  
     dplyr ::  select ( 
       Abbreviation, Latitude, Longitude, Pop_City, Country 
     ) 
    
    head (pops)    
  ## # A tibble: 6 × 5
##   Abbreviation Latitude Longitude Pop_City   Country 
##   <chr>           <dbl>     <dbl> <chr>      <chr>   
## 1 GEL              26.9      90.5 Gelephu    Bhutan  
## 2 CAM              11.6     105.  Phnom Penh Cambodia
## 3 HAI              19.2     110.  Hainan     China   
## 4 YUN              24.5     101.  Yunnan     China   
## 5 HUN              27.6     112.  Hunan      China   
## 6 BEN              13.0      77.6 Bengaluru  India  
 Merge with pops 
       # Add an index column to Q_tibble  
   nadmixk5 $ index  <-   seq_len ( nrow (nadmixk5)) 
    
    # Perform the merge as before  
   df1  <-  
      merge ( 
       nadmixk5, 
       pops, 
        by.x =   2 , 
        by.y =   1 , 
        all.x =  T, 
        all.y =  F 
     )  |>  
      na.omit () 
    
    # Order by the index column to ensure the order matches the original Q_tibble  
   df1  <-  df1[ order (df1 $ index),] 
    
    # Optionally, you can remove the index column if it's no longer needed  
   df1 $ index  <-   NULL  
    
    # Now the rows of df1 should be in the same order as the original Q_tibble  
    head (df1)    
  ##     pop  ind           v1        v2         v3         v4          v5 Latitude
## 159 OKI 1001 0.0022390699 0.8273290 0.04438876 0.11306997 0.012973181  26.5013
## 160 OKI 1002 0.0006207919 0.9372209 0.03072406 0.02804254 0.003391742  26.5013
## 161 OKI 1003 0.0029661185 0.8442711 0.04149344 0.10293802 0.008331385  26.5013
## 162 OKI 1004 0.0024678661 0.7841588 0.05913275 0.14614794 0.008092790  26.5013
## 163 OKI 1005 0.0027905770 0.7570373 0.07026709 0.15857665 0.011328328  26.5013
## 164 OKI 1006 0.0019242963 0.8448561 0.04041177 0.10083950 0.011968327  26.5013
##     Longitude Pop_City Country
## 159  127.9454  Okinawa   Japan
## 160  127.9454  Okinawa   Japan
## 161  127.9454  Okinawa   Japan
## 162  127.9454  Okinawa   Japan
## 163  127.9454  Okinawa   Japan
## 164  127.9454  Okinawa   Japan  
 We used this color palette to make the “structure” plot 
      color_palette2  <-  
   color_palette  <-  
      c ( 
        "v1"   =   "red" , 
        "v2"   =   "#AE9393" , 
        "v3"   =   "#FFFF99" , 
        "v4"   =   "#FFB347" , 
        "v5"   =   "#F49AC2"  
     )    
 Make pie plot 
      world  <-   ne_countries ( scale =   "medium" ,  returnclass =   "sf" ) 
   countries_with_data  <-   unique (df1 $ Country) 
    
    # Filtering the world data to include only the countries in your data  
   selected_countries  <-  world  |>  
      filter (admin  %in%  countries_with_data) 
    
    # Calculate mean proportions for each population  
   df_mean  <-  df1  |>  
      group_by (pop)  |>  
      summarise ( across ( starts_with ( "v" ), \(x)  mean (x,  na.rm =   TRUE )),  
                Longitude =   mean (Longitude), 
                Latitude =   mean (Latitude)) 
    
    
    source ( 
      here ( 
        "scripts" ,  "analysis" ,  "my_theme2.R"  
     ) 
   ) 
    
    ggplot ()  +  
      geom_sf ( data =  selected_countries,  fill=  "white" )  +  
      geom_scatterpie ( data =  df_mean,  
                      aes ( x =  Longitude,  y =  Latitude,  r =   1.5 ),  
                      cols =   c ( "v1" ,  "v2" ,  "v3" ,  "v4" ,  "v5" ),  color =   NA )  +  
      geom_text_repel ( data =  df_mean, 
                      aes ( x =  Longitude,  y =  Latitude,  label =  pop),  
                      size =   3 ,  
                      box.padding =   unit ( 0.5 ,  "lines" ), 
                      max.overlaps =   50 )  +  
      scale_fill_manual ( values =  color_palette2)  +  
      guides ( fill =   "none" )  +    # Hide legend  
      # coord_sf() +  
      coord_sf ( xlim =   c ( 60 ,  150 ),  ylim =   c ( -  10 ,  60 ))  +  
      my_theme ()    
   
       # #   
    ggsave ( 
      here ( "output" ,  "populations" ,  "figures" ,  "neuro-admixture_r_0.1_k5_pie.pdf" ), 
      width  =   12 , 
      height =   6 , 
      units  =   "in" , 
      device =  cairo_pdf 
   )    
 
 
 16.2 Preparing the data for tess3Q_map_rasters 
 Make sure the lat longs are in the correct order and arrangement 
      df2  <-  df1  |>  
     dplyr ::  rename ( 
        Long =  Longitude, 
        Lat =  Latitude 
     ) 
    
   long_lat_tibble  <-  df2  |>  
     dplyr ::  select (Long, Lat) 
    
    
   long_lat_matrix  <-  long_lat_tibble  |>  
      as.matrix () 
    
    head (long_lat_matrix)    
  ##         Long     Lat
## 159 127.9454 26.5013
## 160 127.9454 26.5013
## 161 127.9454 26.5013
## 162 127.9454 26.5013
## 163 127.9454 26.5013
## 164 127.9454 26.5013  
 
 
 16.3 make a matrix of the Q values 
 Pull off the names of individuals and make a matrix of it: 
      Q_matrix  <-  nadmixk5  |>  
     dplyr ::  select ( - ind,  - pop,  - index)  |>  
      as.matrix () 
    head (Q_matrix)    
  ##                v1        v2         v3         v4          v5
## [1,] 0.0022390699 0.8273290 0.04438876 0.11306997 0.012973181
## [2,] 0.0006207919 0.9372209 0.03072406 0.02804254 0.003391742
## [3,] 0.0029661185 0.8442711 0.04149344 0.10293802 0.008331385
## [4,] 0.0024678661 0.7841588 0.05913275 0.14614794 0.008092790
## [5,] 0.0027905770 0.7570373 0.07026709 0.15857665 0.011328328
## [6,] 0.0019242963 0.8448561 0.04041177 0.10083950 0.011968327  
 
 
 16.4 Interpolate the Q-values by Kriging 
       print ( ncol (Q_matrix)  ==   length (color_palette2))    
  ## [1] TRUE  
 Create brick 
      genoscape_brick  <-  tess3r ::  tess3Q_map_rasters ( 
      x =  Q_matrix,  
      coord =  long_lat_matrix,   
      map.polygon =  selected_countries, 
      window =   extent (selected_countries)[ 1  :  4 ], 
      # window = combined_extent,  
      resolution =   c ( 600 , 600 ),  # if you want more cells in your raster, set higher  
      # this next lines need to to be here, but don't do much...  
      col.palette =  tess3r ::  CreatePalette (color_palette2,  length (color_palette2)), 
      method =   "map.max" ,  
      interpol =  tess3r ::  FieldsKrigModel ( 80 ),   
      main =   "Ancestry coefficients" , 
      xlab =   "Longitude" ,  
      ylab =   "Latitude" ,  
      cex =  . 4  
   )    
  ## Warning: 
## Grid searches over lambda (nugget and sill variances) with  minima at the endpoints: 
##   (REML) Restricted maximum likelihood 
##    minimum at  right endpoint  lambda  =  0.02118404 (eff. df= 26.59999 )
## Warning: 
## Grid searches over lambda (nugget and sill variances) with  minima at the endpoints: 
##   (REML) Restricted maximum likelihood 
##    minimum at  right endpoint  lambda  =  0.02118404 (eff. df= 26.59999 )
## Warning: 
## Grid searches over lambda (nugget and sill variances) with  minima at the endpoints: 
##   (REML) Restricted maximum likelihood 
##    minimum at  right endpoint  lambda  =  0.02118404 (eff. df= 26.59999 )  
       # after that, we need to add names of the clusters back onto this raster brick  
   Q_tibble2  <-  nadmixk5  |>  
     dplyr ::  select ( 
        - pop,  - ind,  - index 
     ) 
    names (genoscape_brick)  <-   names (Q_tibble2)[]    
 
 
 16.5 Scaling and cleaning the genoscape_brick 
      genoscape_rgba  <-  genoscapeRtools ::  qprob_rando_raster ( 
      TRB =  genoscape_brick, 
      cols =  color_palette2, 
      alpha_scale =   2.0 , 
      abs_thresh =   0.0 , 
      alpha_exp =   1.55 , 
      alpha_chop_max =   255  
   ) 
    
    # This adds the info for a regular lat-long projection  
    crs (genoscape_rgba)  <-   "+proj=longlat +datum=WGS84 +no_defs +ellps=WGS84 +towgs84=0,0,0"     
 We can easily plot this with the function layer_spatial from the
ggspatial package: 
       ggplot ()  +   
     ggspatial ::  layer_spatial (genoscape_rgba)  +   
      my_theme ()  +  
      coord_sf ()    
 With pies 
       ggplot ()  +  
      layer_spatial (genoscape_rgba)  +  
      geom_spatial_point ( data =  long_lat_tibble, 
                         mapping =   aes ( x =  Long,  y =  Lat), 
                         size =  . 2 )  +  
      geom_text_repel ( 
        data =  df_mean, 
        aes ( x =  Longitude,  y =  Latitude,  label =  pop), 
        size =   3 , 
        box.padding =   unit ( 0.5 ,  "lines" ) 
     )  +  
      labs ( x =   "Longitude" , 
           y =   "Latitude" )  +  
      geom_scatterpie ( data =  df_mean,  
                      aes ( x =  Longitude,  y =  Latitude,  r =   1 ),  
                      cols =   c ( "v1" ,  "v2" ,  "v3" ,  "v4" ,  "v5" ),  color =   NA )  +  
      my_theme ()  +  
      scale_fill_manual ( values =  color_palette2)  +  
      guides ( fill =   "none" )  +    # Hide legend  
      coord_sf ()    
  ## Assuming `crs = 4326` in stat_spatial_identity()  
   
       # #   
    ggsave ( 
      here ( "output" ,  "populations" ,  "figures" ,  "neuro-admixture_r_0.1_k5_interpolated_pie.pdf" ), 
      width  =   12 , 
      height =   6 , 
      units  =   "in" , 
      device =  cairo_pdf 
   )    
  ## Assuming `crs = 4326` in stat_spatial_identity()  
 
 
 
 17. neuro-admxiture neutral r2 0.1 SNPs k5 
 Clear memory and environment 
       # Clear entire environment  
    rm ( list =   ls ()) 
    # Forcefully trigger garbage collection  
    gc ()    
  ##           used  (Mb) gc trigger  (Mb) limit (Mb) max used  (Mb)
## Ncells 4699423 251.0   12802251 683.8         NA 12802251 683.8
## Vcells 8808839  67.3   40256515 307.2      32768 68732428 524.4  
 
 17.1 Q-values 
 Import Q 
       # Extract ancestry coefficients  
   nadmixk5  <-   read_delim ( 
      here ( "output" ,  "populations" ,  "nadmix" ,  "results" ,  "neutral" , "neutral_inference.5.Q" ), 
      delim =   " " ,  # Specify the delimiter if different from the default (comma)  
      col_names =   FALSE , 
      show_col_types =   FALSE  
   )  
    
    head (nadmixk5)    
  ## # A tibble: 6 × 5
##      X1     X2    X3    X4    X5
##   <dbl>  <dbl> <dbl> <dbl> <dbl>
## 1 0.247 0.182  0.210 0.154 0.208
## 2 0.214 0.0904 0.306 0.208 0.182
## 3 0.257 0.179  0.191 0.161 0.211
## 4 0.373 0.109  0.117 0.257 0.144
## 5 0.356 0.110  0.138 0.255 0.141
## 6 0.324 0.162  0.168 0.165 0.181  
 The fam file 
      fam_file  <-   here ( 
      "output" ,  "populations" ,  "snps_sets" ,  "neutral.fam"  
   ) 
    
    # Read the .fam file  
   fam_data  <-   read.table (fam_file,  
                           header =   FALSE , 
                           col.names =   c ( "FamilyID" ,  "IndividualID" ,  "PaternalID" ,  "MaternalID" ,  "Sex" ,  "Phenotype" )) 
    
    # View the first few rows  
    head (fam_data)    
  ##   FamilyID IndividualID PaternalID MaternalID Sex Phenotype
## 1      OKI         1001          0          0   2        -9
## 2      OKI         1002          0          0   2        -9
## 3      OKI         1003          0          0   2        -9
## 4      OKI         1004          0          0   2        -9
## 5      OKI         1005          0          0   2        -9
## 6      OKI         1006          0          0   1        -9  
 Create ID column 
       # Change column name  
    colnames (fam_data)[ colnames (fam_data)  ==   "IndividualID" ]  <-   "ind"  
    
    # Change column name  
    colnames (fam_data)[ colnames (fam_data)  ==   "FamilyID" ]  <-   "pop"  
    
    # Select ID  
   fam_data  <-  fam_data  |>  
     dplyr ::  select ( "ind" ,  "pop" ) 
    
    # View the first few rows  
    head (fam_data)    
  ##    ind pop
## 1 1001 OKI
## 2 1002 OKI
## 3 1003 OKI
## 4 1004 OKI
## 5 1005 OKI
## 6 1006 OKI  
 Add it to matrix 
      nadmixk5  <-  fam_data  |>  
     dplyr ::  select (ind, pop)  |>  
      bind_cols (nadmixk5) 
    
    head (nadmixk5)    
  ##    ind pop        X1         X2        X3        X4        X5
## 1 1001 OKI 0.2465583 0.18223503 0.2096153 0.1539955 0.2075958
## 2 1002 OKI 0.2139250 0.09040003 0.3061219 0.2078033 0.1817497
## 3 1003 OKI 0.2573991 0.17856130 0.1912381 0.1614681 0.2113334
## 4 1004 OKI 0.3727467 0.10854463 0.1174154 0.2574833 0.1438100
## 5 1005 OKI 0.3558274 0.10966873 0.1382425 0.2551490 0.1411124
## 6 1006 OKI 0.3244200 0.16158162 0.1678030 0.1649402 0.1812551  
 Rename the columns 
       # Rename the columns starting from the third one  
   nadmixk5  <-  nadmixk5  |>  
      rename_with ( ~  paste0 ( "v" ,  seq_along (.x)),  .cols =   -  c (ind, pop)) 
    
    # View the first few rows  
    head (nadmixk5)    
  ##    ind pop        v1         v2        v3        v4        v5
## 1 1001 OKI 0.2465583 0.18223503 0.2096153 0.1539955 0.2075958
## 2 1002 OKI 0.2139250 0.09040003 0.3061219 0.2078033 0.1817497
## 3 1003 OKI 0.2573991 0.17856130 0.1912381 0.1614681 0.2113334
## 4 1004 OKI 0.3727467 0.10854463 0.1174154 0.2574833 0.1438100
## 5 1005 OKI 0.3558274 0.10966873 0.1382425 0.2551490 0.1411124
## 6 1006 OKI 0.3244200 0.16158162 0.1678030 0.1649402 0.1812551  
 Import samples attributes 
      sampling_loc  <-   readRDS ( here ( "output" ,  "populations" ,  "sampling_loc.rds" )) 
    # head(sampling_loc)  
    
   pops  <-  sampling_loc  |>  
      filter ( 
       Region  ==   "Asia"  
     )  |>  
     dplyr ::  select ( 
       Abbreviation, Latitude, Longitude, Pop_City, Country 
     ) 
    
    head (pops)    
  ## # A tibble: 6 × 5
##   Abbreviation Latitude Longitude Pop_City   Country 
##   <chr>           <dbl>     <dbl> <chr>      <chr>   
## 1 GEL              26.9      90.5 Gelephu    Bhutan  
## 2 CAM              11.6     105.  Phnom Penh Cambodia
## 3 HAI              19.2     110.  Hainan     China   
## 4 YUN              24.5     101.  Yunnan     China   
## 5 HUN              27.6     112.  Hunan      China   
## 6 BEN              13.0      77.6 Bengaluru  India  
 Merge with pops 
       # Add an index column to Q_tibble  
   nadmixk5 $ index  <-   seq_len ( nrow (nadmixk5)) 
    
    # Perform the merge as before  
   df1  <-  
      merge ( 
       nadmixk5, 
       pops, 
        by.x =   2 , 
        by.y =   1 , 
        all.x =  T, 
        all.y =  F 
     )  |>  
      na.omit () 
    
    # Order by the index column to ensure the order matches the original Q_tibble  
   df1  <-  df1[ order (df1 $ index),] 
    
    # Optionally, you can remove the index column if it's no longer needed  
   df1 $ index  <-   NULL  
    
    # Now the rows of df1 should be in the same order as the original Q_tibble  
    head (df1)    
  ##     pop  ind        v1         v2        v3        v4        v5 Latitude
## 159 OKI 1001 0.2465583 0.18223503 0.2096153 0.1539955 0.2075958  26.5013
## 160 OKI 1002 0.2139250 0.09040003 0.3061219 0.2078033 0.1817497  26.5013
## 161 OKI 1003 0.2573991 0.17856130 0.1912381 0.1614681 0.2113334  26.5013
## 162 OKI 1004 0.3727467 0.10854463 0.1174154 0.2574833 0.1438100  26.5013
## 163 OKI 1005 0.3558274 0.10966873 0.1382425 0.2551490 0.1411124  26.5013
## 164 OKI 1006 0.3244200 0.16158162 0.1678030 0.1649402 0.1812551  26.5013
##     Longitude Pop_City Country
## 159  127.9454  Okinawa   Japan
## 160  127.9454  Okinawa   Japan
## 161  127.9454  Okinawa   Japan
## 162  127.9454  Okinawa   Japan
## 163  127.9454  Okinawa   Japan
## 164  127.9454  Okinawa   Japan  
 We used this color palette to make the “structure” plot 
      color_palette2  <-  
   color_palette  <-  
      c ( 
        "v1"   =   "#AE9393" , 
        "v2"   =   "red" , 
        "v3"   =   "#FFFF99" , 
        "v4"   =   "#F49AC2" , 
        "v5"   =   "#FFB347"  
     )    
 Make pie plot 
      world  <-   ne_countries ( scale =   "medium" ,  returnclass =   "sf" ) 
   countries_with_data  <-   unique (df1 $ Country) 
    
    # Filtering the world data to include only the countries in your data  
   selected_countries  <-  world  |>  
      filter (admin  %in%  countries_with_data) 
    
    # Calculate mean proportions for each population  
   df_mean  <-  df1  |>  
      group_by (pop)  |>  
      summarise ( across ( starts_with ( "v" ), \(x)  mean (x,  na.rm =   TRUE )),  
                Longitude =   mean (Longitude), 
                Latitude =   mean (Latitude)) 
    
    
    source ( 
      here ( 
        "scripts" ,  "analysis" ,  "my_theme2.R"  
     ) 
   ) 
    
    ggplot ()  +  
      geom_sf ( data =  selected_countries,  fill=  "white" )  +  
      geom_scatterpie ( data =  df_mean,  
                      aes ( x =  Longitude,  y =  Latitude,  r =   1.5 ),  
                      cols =   c ( "v1" ,  "v2" ,  "v3" ,  "v4" ,  "v5" ),  color =   NA )  +  
      geom_text_repel ( data =  df_mean, 
                      aes ( x =  Longitude,  y =  Latitude,  label =  pop),  
                      size =   3 ,  
                      box.padding =   unit ( 0.5 ,  "lines" ), 
                      max.overlaps =   50 )  +  
      scale_fill_manual ( values =  color_palette2)  +  
      guides ( fill =   "none" )  +    # Hide legend  
      # coord_sf() +  
      coord_sf ( xlim =   c ( 60 ,  150 ),  ylim =   c ( -  10 ,  60 ))  +  
      my_theme ()    
   
       # #   
    ggsave ( 
      here ( "output" ,  "populations" ,  "figures" ,  "neuro-admixture_neutral_k5_pie.pdf" ), 
      width  =   12 , 
      height =   6 , 
      units  =   "in" , 
      device =  cairo_pdf 
   )    
 
 
 17.2 Preparing the data for tess3Q_map_rasters 
      df2  <-  df1  |>  
     dplyr ::  rename ( 
        Long =  Longitude, 
        Lat =  Latitude 
     ) 
    
   long_lat_tibble  <-  df2  |>  
     dplyr ::  select (Long, Lat) 
    
    
   long_lat_matrix  <-  long_lat_tibble  |>  
      as.matrix () 
    
    head (long_lat_matrix)    
  ##         Long     Lat
## 159 127.9454 26.5013
## 160 127.9454 26.5013
## 161 127.9454 26.5013
## 162 127.9454 26.5013
## 163 127.9454 26.5013
## 164 127.9454 26.5013  
 
 
 17.3 make a matrix of the Q values 
 Pull off the names of individuals and make a matrix of it: 
      Q_matrix  <-  nadmixk5  |>  
     dplyr ::  select ( - ind,  - pop,  - index)  |>  
      as.matrix () 
    head (Q_matrix)    
  ##             v1         v2        v3        v4        v5
## [1,] 0.2465583 0.18223503 0.2096153 0.1539955 0.2075958
## [2,] 0.2139250 0.09040003 0.3061219 0.2078033 0.1817497
## [3,] 0.2573991 0.17856130 0.1912381 0.1614681 0.2113334
## [4,] 0.3727467 0.10854463 0.1174154 0.2574833 0.1438100
## [5,] 0.3558274 0.10966873 0.1382425 0.2551490 0.1411124
## [6,] 0.3244200 0.16158162 0.1678030 0.1649402 0.1812551  
 
 
 17.4 Interpolate the Q-values by Kriging 
       print ( ncol (Q_matrix)  ==   length (color_palette2))    
  ## [1] TRUE  
 Create brick 
      genoscape_brick  <-  tess3r ::  tess3Q_map_rasters ( 
      x =  Q_matrix,  
      coord =  long_lat_matrix,   
      map.polygon =  selected_countries, 
      window =   extent (selected_countries)[ 1  :  4 ], 
      # window = combined_extent,  
      resolution =   c ( 600 , 600 ),  # if you want more cells in your raster, set higher  
      # this next lines need to to be here, but don't do much...  
      col.palette =  tess3r ::  CreatePalette (color_palette2,  length (color_palette2)), 
      method =   "map.max" ,  
      interpol =  tess3r ::  FieldsKrigModel ( 80 ),   
      main =   "Ancestry coefficients" , 
      xlab =   "Longitude" ,  
      ylab =   "Latitude" ,  
      cex =  . 4  
   )    
  ## Warning: 
## Grid searches over lambda (nugget and sill variances) with  minima at the endpoints: 
##   (REML) Restricted maximum likelihood 
##    minimum at  right endpoint  lambda  =  0.02118404 (eff. df= 26.59999 )
## Warning: 
## Grid searches over lambda (nugget and sill variances) with  minima at the endpoints: 
##   (REML) Restricted maximum likelihood 
##    minimum at  right endpoint  lambda  =  0.02118404 (eff. df= 26.59999 )
## Warning: 
## Grid searches over lambda (nugget and sill variances) with  minima at the endpoints: 
##   (REML) Restricted maximum likelihood 
##    minimum at  right endpoint  lambda  =  0.02118404 (eff. df= 26.59999 )
## Warning: 
## Grid searches over lambda (nugget and sill variances) with  minima at the endpoints: 
##   (REML) Restricted maximum likelihood 
##    minimum at  right endpoint  lambda  =  0.02118404 (eff. df= 26.59999 )  
       # after that, we need to add names of the clusters back onto this raster brick  
   Q_tibble2  <-  nadmixk5  |>  
     dplyr ::  select ( 
        - pop,  - ind,  - index 
     ) 
    names (genoscape_brick)  <-   names (Q_tibble2)[]    
 
 
 17.5 Scaling and cleaning the genoscape_brick 
      genoscape_rgba  <-  genoscapeRtools ::  qprob_rando_raster ( 
      TRB =  genoscape_brick, 
      cols =  color_palette2, 
      alpha_scale =   2.0 , 
      abs_thresh =   0.0 , 
      alpha_exp =   1.55 , 
      alpha_chop_max =   255  
   ) 
    
    # This adds the info for a regular lat-long projection  
    crs (genoscape_rgba)  <-   "+proj=longlat +datum=WGS84 +no_defs +ellps=WGS84 +towgs84=0,0,0"     
 We can easily plot this with the function layer_spatial from the
ggspatial package: 
       ggplot ()  +   
     ggspatial ::  layer_spatial (genoscape_rgba)  +   
      my_theme ()  +  
      coord_sf ()    
 With pies 
       ggplot ()  +  
      layer_spatial (genoscape_rgba)  +  
      geom_spatial_point ( data =  long_lat_tibble, 
                         mapping =   aes ( x =  Long,  y =  Lat), 
                         size =  . 2 )  +  
      geom_text_repel ( 
        data =  df_mean, 
        aes ( x =  Longitude,  y =  Latitude,  label =  pop), 
        size =   3 , 
        box.padding =   unit ( 0.5 ,  "lines" ) 
     )  +  
      labs ( x =   "Longitude" , 
           y =   "Latitude" )  +  
      geom_scatterpie ( data =  df_mean,  
                      aes ( x =  Longitude,  y =  Latitude,  r =   1 ),  
                      cols =   c ( "v1" ,  "v2" ,  "v3" ,  "v4" ,  "v5" ),  color =   NA )  +  
      my_theme ()  +  
      scale_fill_manual ( values =  color_palette2)  +  
      guides ( fill =   "none" )  +    # Hide legend  
      coord_sf ()    
  ## Assuming `crs = 4326` in stat_spatial_identity()  
   
       # #   
    ggsave ( 
      here ( "output" ,  "populations" ,  "figures" ,  "neuro-admixture_neutral_k5_interpolated_pie.pdf" ), 
      width  =   12 , 
      height =   6 , 
      units  =   "in" , 
      device =  cairo_pdf 
   )    
  ## Assuming `crs = 4326` in stat_spatial_identity()  
 
 
 


 

 

 

 

 


 
 

 
 
