# Supplementary material for: A genotyping array for the globally invasive vector mosquito, Aedes albopictus
Source: Parasit Vectors. 2024 Mar 4;17:106. doi: 10.1186/s13071-024-06158-z (PMC10910840; doi:10.1186/s13071-024-06158-z)
Supplement: Supplementary file 14 — Additional file 14. Evaluating the impact of chip bias with LEA and PCA. [file 13071_2024_6158_MOESM14_ESM.html]

Aedes albopictus SNP chip - Ancestry analysis with LEA for the SNP sets comparying the proportion of SNPs by effect.


# Aedes albopictus SNP chip - Ancestry analysis with LEA for the SNP sets comparying the proportion of SNPs by effect.

#### Luciano V Cosme

#### 2023-09-27

- Libraries
  - 1. SNPs sets
  - 2. PCA
  - 3. Acestry
    analysis
    - 3.1 set3
      - 3.1.1 Structure
        plot
      - 3.1.2 Interploation over map
    - 3.2 set5
      - 3.2.1
        Structure plot
      - 3.2.2 Interploation over map
    - 3.3 set10
      - 3.3.1
        Structure plot
      - 3.3.2 Interploation over map
    - 3.4 random2
      - 3.4.1
        Structure plot
      - 3.4.2 Interploation over map
    - 3.5 random8
      - 3.5.1
        Structure plot
      - 3.5.2 Interploation over map
    - 3.6 random9
      - 3.6.1
        Structure plot
      - 3.6.2 Interploation over map

# Libraries

```
library(LEA)
library(vcfR)
library(RColorBrewer)
library(ggplot2)
library(adegenet)
library(ape)
library(tidyverse)
library(here)
library(dplyr)
library(ggplot2)
library(colorout)
library(extrafont)
library(scales)
library(stringr)
library(ggtext)
library(flextable)
library(officer)
library(raster)
library(sf)
library(ggspatial)
library(scatterpie)
library(rnaturalearth)
library(rnaturalearthdata)
library(ggrepel)
library(Cairo)
```

## 1. SNPs sets

At the end of the functional annotation Markdown, we created 3 sets
of SNPs following the WGS proportions by effect and 3 sets of SNPs using
the same number of SNPs but randomly selected.

Check the sets

```
ls output/SnpEff/chip_new_proportions/sets
```

```
## random1.txt
## random10.txt
## random2.txt
## random3.txt
## random4.txt
## random5.txt
## random6.txt
## random7.txt
## random8.txt
## random9.txt
## set1.txt
## set10.txt
## set2.txt
## set3.txt
## set4.txt
## set5.txt
## set6.txt
## set7.txt
## set8.txt
## set9.txt
## sets_following_wgs_prop.pdf
## sets_following_wgs_prop_selected.pdf
## sets_random_not_following_WGS_prop.pdf
## sets_random_not_following_WGS_prop_selected.pdf
```

We wrote code to randomly select 3 sets for each group (random and
set). The sets we used: Following wgs proportion: set3, set5 and set10
Now following wgs proportions: random2, random8 and random9

Now we can create vcf to use with LEA

Create vcfs and files for PCA

Set3

```
plink2 \
--allow-extra-chr \
--bfile output/populations/file7 \
--extract output/SnpEff/chip_new_proportions/sets/set3.txt \
--export vcf \
--pca allele-wts \
--freq \
--out output/variant_proportions/set3 \
--silent;
grep 'variants\|samples' output/variant_proportions/set3.log
```

```
## 237 samples (30 females, 67 males, 140 ambiguous; 237 founders) loaded from
## 82731 variants loaded from output/populations/file7.bim.
## --extract: 33413 variants remaining.
## 33413 variants remaining after main filters.
```

Set5

```
plink2 \
--allow-extra-chr \
--bfile output/populations/file7 \
--extract output/SnpEff/chip_new_proportions/sets/set5.txt \
--export vcf \
--pca allele-wts \
--freq \
--out output/variant_proportions/set5 \
--silent;
grep 'variants\|samples' output/variant_proportions/set5.log
```

```
## 237 samples (30 females, 67 males, 140 ambiguous; 237 founders) loaded from
## 82731 variants loaded from output/populations/file7.bim.
## --extract: 33456 variants remaining.
## 33456 variants remaining after main filters.
```

Set10

```
plink2 \
--allow-extra-chr \
--bfile output/populations/file7 \
--extract output/SnpEff/chip_new_proportions/sets/set10.txt \
--export vcf \
--pca allele-wts \
--freq \
--out output/variant_proportions/set10 \
--silent;
grep 'variants\|samples' output/variant_proportions/set10.log
```

```
## 237 samples (30 females, 67 males, 140 ambiguous; 237 founders) loaded from
## 82731 variants loaded from output/populations/file7.bim.
## --extract: 33392 variants remaining.
## 33392 variants remaining after main filters.
```

random2

```
plink2 \
--allow-extra-chr \
--bfile output/populations/file7 \
--extract output/SnpEff/chip_new_proportions/sets/random2.txt \
--export vcf \
--pca allele-wts \
--freq \
--out output/variant_proportions/random2 \
--silent;
grep 'variants\|samples' output/variant_proportions/random2.log
```

```
## 237 samples (30 females, 67 males, 140 ambiguous; 237 founders) loaded from
## 82731 variants loaded from output/populations/file7.bim.
## --extract: 33400 variants remaining.
## 33400 variants remaining after main filters.
```

random8

```
plink2 \
--allow-extra-chr \
--bfile output/populations/file7 \
--extract output/SnpEff/chip_new_proportions/sets/random8.txt \
--export vcf \
--pca allele-wts \
--freq \
--out output/variant_proportions/random8 \
--silent;
grep 'variants\|samples' output/variant_proportions/random8.log
```

```
## 237 samples (30 females, 67 males, 140 ambiguous; 237 founders) loaded from
## 82731 variants loaded from output/populations/file7.bim.
## --extract: 33400 variants remaining.
## 33400 variants remaining after main filters.
```

random9

```
plink2 \
--allow-extra-chr \
--bfile output/populations/file7 \
--extract output/SnpEff/chip_new_proportions/sets/random9.txt \
--export vcf \
--pca allele-wts \
--freq \
--out output/variant_proportions/random9 \
--silent;
grep 'variants\|samples' output/variant_proportions/random9.log
```

```
## 237 samples (30 females, 67 males, 140 ambiguous; 237 founders) loaded from
## 82731 variants loaded from output/populations/file7.bim.
## --extract: 33400 variants remaining.
## 33400 variants remaining after main filters.
```

Let’s parse the .log file to get the number of SNPs in each file

```
# Load necessary libraries
library(stringr)

# Specify the directory containing the .log files
dir_path <- "output/variant_proportions"

# Get a list of all the .log files in the directory
log_files <- list.files(path = dir_path, pattern = "\\.log$", full.names = TRUE)

# Function to extract the number of variants from a given file
extract_variants <- function(file_path) {
    content <- readLines(file_path)
    line <- grep("variants remaining after main filters", content, value = TRUE)
    return(as.numeric(unlist(str_extract_all(line, "\\d+"))))
}

# Get the variants from each log file
variants <- sapply(log_files, extract_variants)

# Get the file name without the .log extension
file_names <- gsub(".log$", "", basename(log_files))

# Create a data frame with the results
result_df <- data.frame(FileName = file_names, Variants = variants)

# Print the result
print(result_df)
```

```
##                                        FileName Variants
## output/variant_proportions/random2.log  random2    33400
## output/variant_proportions/random8.log  random8    33400
## output/variant_proportions/random9.log  random9    33400
## output/variant_proportions/set10.log      set10    33392
## output/variant_proportions/set3.log        set3    33413
## output/variant_proportions/set5.log        set5    33456
```

Let’s create a table and save it

```
# Create a flextable with zebra theme
ft <- flextable(result_df)
ft <- color(ft, color = "black", part = "header")
ft <- bg(ft, bg = "gray", part = "header")
ft <- color(ft, color = "black", part = "body")

# Use zebra theme
ft <- bg(ft, i = seq(2, nrow(result_df), by = 2), bg = "lightgray")
ft
```

| FileName | Variants |
| --- | --- |
| random2 | 33,400 |
| random8 | 33,400 |
| random9 | 33,400 |
| set10 | 33,392 |
| set3 | 33,413 |
| set5 | 33,456 |

```
# Save to DOCX
doc <- read_docx()
doc <- body_add_flextable(doc, ft)
print(doc, target = here("output", "variant_proportions", "result_table.docx"))
```

## 2. PCA

We created PCA files with Plink when we generated the vcf files. We
can import them and create a PCA

Check the files

```
ls output/variant_proportions/*.eigenvec
```

```
## output/variant_proportions/random2.eigenvec
## output/variant_proportions/random8.eigenvec
## output/variant_proportions/random9.eigenvec
## output/variant_proportions/set10.eigenvec
## output/variant_proportions/set3.eigenvec
## output/variant_proportions/set5.eigenvec
```

```
ls output/variant_proportions/*.eigenval
```

```
## output/variant_proportions/random2.eigenval
## output/variant_proportions/random8.eigenval
## output/variant_proportions/random9.eigenval
## output/variant_proportions/set10.eigenval
## output/variant_proportions/set3.eigenval
## output/variant_proportions/set5.eigenval
```

Import .eigenvec

```
# Define the directory
dir_path <- here("output","variant_proportions")

# List all .eigenvec files in the directory
file_paths <- list.files(path = dir_path, pattern = "\\.eigenvec$", full.names = TRUE)

# Read all files into a list
pca_list <- lapply(file_paths, function(path) {
  read.delim(path, header = TRUE)
})

# Assign names to the list elements based on the filenames
names(pca_list) <- sapply(file_paths, function(path) {
  basename(tools::file_path_sans_ext(path))
})
```

Import sample attributes

```
# import sample attributes
samples2 <- read.delim(
  here(
    "output", "populations", "Population_meta_data.txt"
  ),
  head = TRUE
)
#
# check head of the file
head(samples2)
```

```
##   geo  range continent    region country pop
## 1   1 native      Asia East Asia   China HAI
## 2   1 Native      Asia East Asia   China HUN
## 3   1 Native      Asia East Asia   China YUN
## 4   1 Native      Asia East Asia   Japan AIZ
## 5   1 Native      Asia East Asia   Japan HIR
## 6   1 Native      Asia East Asia   Japan JAT
```

Merge the data

```
# Custom function to merge the PCA data frame with samples2
merge_with_samples2 <- function(df) {
  merged_df <-
    merge(
      df,
      samples2,
      by.x = "X.FID",
      by.y = 6,
      all.x = TRUE,
      all.y = FALSE
    ) %>%
    na.omit()
  return(merged_df)
}

# Apply the function to each dataframe in the list
merged_list <- lapply(pca_list, merge_with_samples2)

# Check the first few rows of one of the merged data frames for verification
head(merged_list$random2)
```

```
##   X.FID IID        PC1        PC2       PC3        PC4       PC5       PC6
## 1   BEN   a -0.0317165 -0.0550937 0.0221005 0.01023110 0.0203769 0.0580362
## 2   BEN 257 -0.0302539 -0.0562667 0.0290389 0.00561612 0.0267604 0.0571630
## 3   BEN 261 -0.0310892 -0.0560538 0.0273201 0.01443210 0.0251756 0.0568556
## 4   BEN 263 -0.0331771 -0.0576573 0.0289612 0.00876081 0.0294055 0.0556967
## 5   BEN 264 -0.0316259 -0.0557791 0.0226878 0.00627924 0.0213318 0.0537480
## 6   BEN 262 -0.0317751 -0.0571210 0.0280904 0.01129510 0.0285958 0.0574392
##          PC7          PC8       PC9      PC10 geo  range continent     region
## 1 -0.0458563 -0.009479380 0.0112923 0.0381346   2 Native      Asia South Asia
## 2 -0.0484142 -0.000547293 0.0172005 0.0428997   2 Native      Asia South Asia
## 3 -0.0572810 -0.012319600 0.0120067 0.0459204   2 Native      Asia South Asia
## 4 -0.0543452 -0.004284200 0.0189452 0.0584283   2 Native      Asia South Asia
## 5 -0.0481374 -0.006275960 0.0182097 0.0400247   2 Native      Asia South Asia
## 6 -0.0577929 -0.005920690 0.0209378 0.0611609   2 Native      Asia South Asia
##   country
## 1   India
## 2   India
## 3   India
## 4   India
## 5   India
## 6   India
```

Create data frames

```
random2 <- merged_list$random2
random8 <- merged_list$random8
random9 <- merged_list$random9
set10 <- merged_list$set10
set3 <- merged_list$set3
set5 <- merged_list$set5

random2$source <- "random2"
random8$source <- "random8"
random9$source <- "random9"
set10$source <- "set10"
set3$source <- "set3"
set5$source <- "set5"
```

Combine them

```
combined_data <- rbind(random2, random8, random9, set10, set3, set5)
head(combined_data)
```

```
##   X.FID IID        PC1        PC2       PC3        PC4       PC5       PC6
## 1   BEN   a -0.0317165 -0.0550937 0.0221005 0.01023110 0.0203769 0.0580362
## 2   BEN 257 -0.0302539 -0.0562667 0.0290389 0.00561612 0.0267604 0.0571630
## 3   BEN 261 -0.0310892 -0.0560538 0.0273201 0.01443210 0.0251756 0.0568556
## 4   BEN 263 -0.0331771 -0.0576573 0.0289612 0.00876081 0.0294055 0.0556967
## 5   BEN 264 -0.0316259 -0.0557791 0.0226878 0.00627924 0.0213318 0.0537480
## 6   BEN 262 -0.0317751 -0.0571210 0.0280904 0.01129510 0.0285958 0.0574392
##          PC7          PC8       PC9      PC10 geo  range continent     region
## 1 -0.0458563 -0.009479380 0.0112923 0.0381346   2 Native      Asia South Asia
## 2 -0.0484142 -0.000547293 0.0172005 0.0428997   2 Native      Asia South Asia
## 3 -0.0572810 -0.012319600 0.0120067 0.0459204   2 Native      Asia South Asia
## 4 -0.0543452 -0.004284200 0.0189452 0.0584283   2 Native      Asia South Asia
## 5 -0.0481374 -0.006275960 0.0182097 0.0400247   2 Native      Asia South Asia
## 6 -0.0577929 -0.005920690 0.0209378 0.0611609   2 Native      Asia South Asia
##   country  source
## 1   India random2
## 2   India random2
## 3   India random2
## 4   India random2
## 5   India random2
## 6   India random2
```

Create the plot

```
# source the plotting function
source(here("scripts", "analysis", "my_theme2.R"))

ggplot(combined_data, aes(x = PC1, y = PC2)) +
  geom_point(
    aes(fill = country),
    size = 1,
    shape = 21,
    color = "gray"
  ) +
  stat_ellipse(
    aes(fill = country, group = country),
    geom = "polygon",
    alpha = 0.2,
    level = 0.8,
    segments = 40,
    color = NA,
    show.legend = FALSE
  ) +
  my_theme() +
  facet_wrap( ~ source, ncol = 3) +
  labs(
    title = "PCA Plot SNP sets",
    x = "PC1",
    y = "PC2",
    fill = "Country",
    caption = "All sets had ~ 34k SNPs. Bottow row following the WGS variant proportions by effect."
  ) +
  theme(
    legend.position = "top",
    panel.spacing = unit(1, "lines"),
    plot.caption = element_text(face = "italic", hjust = 1)  # Make the caption italicized
  )
```

```
## Too few points to calculate an ellipse
## Too few points to calculate an ellipse
## Too few points to calculate an ellipse
## Too few points to calculate an ellipse
## Too few points to calculate an ellipse
## Too few points to calculate an ellipse
## Too few points to calculate an ellipse
## Too few points to calculate an ellipse
## Too few points to calculate an ellipse
## Too few points to calculate an ellipse
## Too few points to calculate an ellipse
## Too few points to calculate an ellipse
```

```
# Save plot
ggsave(
  here(
    "output", "variant_proportions", "figures", "PCA_sets.pdf"
  ),
  width  = 6,
  height = 8,
  units  = "in"
)
```

```
## Too few points to calculate an ellipse
## Too few points to calculate an ellipse
## Too few points to calculate an ellipse
## Too few points to calculate an ellipse
## Too few points to calculate an ellipse
## Too few points to calculate an ellipse
## Too few points to calculate an ellipse
## Too few points to calculate an ellipse
## Too few points to calculate an ellipse
## Too few points to calculate an ellipse
## Too few points to calculate an ellipse
## Too few points to calculate an ellipse
```

## 3. Acestry analysis

### 3.1 set3

Clean env and memory

```
rm(list=ls())
gc()
```

```
##           used  (Mb) gc trigger  (Mb) limit (Mb) max used  (Mb)
## Ncells 5050989 269.8    8748335 467.3         NA  8748335 467.3
## Vcells 7681246  58.7   14786712 112.9      32768 12254856  93.5
```

Import the data

```
genotype <- here(
    "output", "variant_proportions", "set3.vcf"
  )

d <- read.vcfR(
  genotype
)
```

```
## Scanning file to determine attributes.
## File attributes:
##   meta lines: 8
##   header_line: 9
##   variant count: 33413
##   column count: 246
## 
Meta line 8 read in.
## All meta lines processed.
## gt matrix initialized.
## Character matrix gt created.
##   Character matrix gt rows: 33413
##   Character matrix gt cols: 246
##   skip: 0
##   nrows: 33413
##   row_num: 0
## 
Processed variant 1000
Processed variant 2000
Processed variant 3000
Processed variant 4000
Processed variant 5000
Processed variant 6000
Processed variant 7000
Processed variant 8000
Processed variant 9000
Processed variant 10000
Processed variant 11000
Processed variant 12000
Processed variant 13000
Processed variant 14000
Processed variant 15000
Processed variant 16000
Processed variant 17000
Processed variant 18000
Processed variant 19000
Processed variant 20000
Processed variant 21000
Processed variant 22000
Processed variant 23000
Processed variant 24000
Processed variant 25000
Processed variant 26000
Processed variant 27000
Processed variant 28000
Processed variant 29000
Processed variant 30000
Processed variant 31000
Processed variant 32000
Processed variant 33000
Processed variant: 33413
## All variants processed
```

Population and individuals information

```
inds_full <- attr(d@gt,"dimnames")[[2]]
inds_full <- inds_full[-1]
a <- strsplit(inds_full, '_')
pops <- unname(sapply(a, FUN = function(x) return(as.character(x[1])))) 
table(pops)
```

```
## pops
## BEN CAM CHA GEL HAI HAN HOC HUN INJ INW JAF KAC KAG KAN KAT KLP KUN LAM MAT OKI 
##  12  12  12   2  12   4   7  12  11   4   2   6  12  11  10   4   4   9  12  11 
## QNC SON SSK SUF SUU TAI UTS YUN 
##  12   3  12   6   6   8  12   9
```

```
pops <- factor(pops)
inds <- unname(sapply(a, FUN = function(x) return(as.character(x[2]))))
```

Convert format

```
vcf2geno(genotype, gsub(".vcf", ".geno", genotype))
```

```
## 
##  - number of detected individuals:   237
##  - number of detected loci:      33413
## 
## For SNP info, please check /Users/lucianocosme/Library/CloudStorage/Dropbox/Albopictus/manuscript_chip/data/no_autogenous/albo_chip/output/variant_proportions/set3.vcfsnp.
## 
## 0 line(s) were removed because these are not SNPs.
## Please, check /Users/lucianocosme/Library/CloudStorage/Dropbox/Albopictus/manuscript_chip/data/no_autogenous/albo_chip/output/variant_proportions/set3.removed file, for more informations.
```

```
## [1] "/Users/lucianocosme/Library/CloudStorage/Dropbox/Albopictus/manuscript_chip/data/no_autogenous/albo_chip/output/variant_proportions/set3.geno"
```

```
vcf2lfmm(genotype, gsub(".vcf", ".lfmm", genotype))
```

```
## 
##  - number of detected individuals:   237
##  - number of detected loci:      33413
## 
## For SNP info, please check /Users/lucianocosme/Library/CloudStorage/Dropbox/Albopictus/manuscript_chip/data/no_autogenous/albo_chip/output/variant_proportions/set3.vcfsnp.
## 
## 0 line(s) were removed because these are not SNPs.
## Please, check /Users/lucianocosme/Library/CloudStorage/Dropbox/Albopictus/manuscript_chip/data/no_autogenous/albo_chip/output/variant_proportions/set3.removed file, for more informations.
## 
## 
##  - number of detected individuals:   237
##  - number of detected loci:      33413
```

```
## [1] "/Users/lucianocosme/Library/CloudStorage/Dropbox/Albopictus/manuscript_chip/data/no_autogenous/albo_chip/output/variant_proportions/set3.lfmm"
```

Run LEA

```
# set output dir
setwd(
  here(
    "output", "variant_proportions"
  )
)
# main options
# K = number of ancestral populations
# entropy = TRUE computes the cross-entropy criterion, # CPU = 4 is the number of CPU used (hidden input) project = NULL
project = snmf(
  genotype,
  K = 1:25,
  project = "new",
  repetitions = 1,
  alpha = 5,
  tolerance = 0.0001,
  percentage = 0.5,
  iterations = 200, 
  ploidy = 2,
  seed = 13,
  CPU = 4,
  entropy = TRUE
)
```

```
project = load.snmfProject("output/variant_proportions/set3.snmfProject")
```

Cross entropy

```
# Open a new pdf file
pdf(here("output","variant_proportions","figures","cross_entropy_set3.pdf"), width = 6, height = 4)

# Create your plot
plot(project, col = "pink", pch = 19, cex = 1.2)

# Close the pdf file
dev.off()
```

```
## quartz_off_screen 
##                 2
```

```
plot(project, col = "pink", pch = 19, cex = 1.2)
```

#### 3.1.1 Structure plot

```
# Matrix
leak5 <- read_delim(
  here("output", "variant_proportions", "set3.snmf", "K5", "run1","set3_r1.5.Q"),
  delim = " ",
  col_names = FALSE,
  show_col_types = FALSE
) 
# unseen_pckmeans.7.Q
# pckmeans.7.Q
head(leak5)
```

```
## # A tibble: 6 × 5
##         X1       X2     X3    X4    X5
##      <dbl>    <dbl>  <dbl> <dbl> <dbl>
## 1 0.112    0.000100 0.135  0.555 0.198
## 2 0.137    0.0424   0.120  0.490 0.210
## 3 0.000100 0.00934  0.208  0.662 0.121
## 4 0.0106   0.00809  0.112  0.750 0.120
## 5 0.0292   0.0152   0.0892 0.759 0.108
## 6 0.0144   0.0135   0.143  0.716 0.113
```

The fam file

```
fam_file <- here(
  "output", "populations", "snps_sets", "neutral.fam"
)

# Read the .fam file
fam_data <- read.table(fam_file, 
                       header = FALSE,
                       col.names = c("FamilyID", "IndividualID", "PaternalID", "MaternalID", "Sex", "Phenotype"))

# View the first few rows
head(fam_data)
```

```
##   FamilyID IndividualID PaternalID MaternalID Sex Phenotype
## 1      OKI         1001          0          0   2        -9
## 2      OKI         1002          0          0   2        -9
## 3      OKI         1003          0          0   2        -9
## 4      OKI         1004          0          0   2        -9
## 5      OKI         1005          0          0   2        -9
## 6      OKI         1006          0          0   1        -9
```

Create ID column

```
# Change column name
colnames(fam_data)[colnames(fam_data) == "IndividualID"] <- "ind"

# Change column name
colnames(fam_data)[colnames(fam_data) == "FamilyID"] <- "pop"

# Select ID
fam_data <- fam_data |>
  dplyr::select("ind", "pop")

# View the first few rows
head(fam_data)
```

```
##    ind pop
## 1 1001 OKI
## 2 1002 OKI
## 3 1003 OKI
## 4 1004 OKI
## 5 1005 OKI
## 6 1006 OKI
```

Add it to matrix

```
leak5 <- fam_data |>
  dplyr::select(ind, pop) |>
  bind_cols(leak5)

head(leak5)
```

```
##    ind pop         X1          X2        X3       X4       X5
## 1 1001 OKI 0.11158400 0.000099991 0.1353900 0.554775 0.198151
## 2 1002 OKI 0.13734800 0.042359900 0.1202150 0.490371 0.209705
## 3 1003 OKI 0.00009999 0.009335060 0.2081730 0.661789 0.120602
## 4 1004 OKI 0.01060020 0.008091190 0.1115070 0.750293 0.119509
## 5 1005 OKI 0.02924580 0.015215500 0.0891502 0.758603 0.107786
## 6 1006 OKI 0.01443040 0.013455300 0.1430950 0.716448 0.112571
```

Rename the columns

```
# Rename the columns starting from the third one
leak5 <- leak5 |>
  rename_with(~paste0("v", seq_along(.x)), .cols = -c(ind, pop))

# View the first few rows
head(leak5)
```

```
##    ind pop         v1          v2        v3       v4       v5
## 1 1001 OKI 0.11158400 0.000099991 0.1353900 0.554775 0.198151
## 2 1002 OKI 0.13734800 0.042359900 0.1202150 0.490371 0.209705
## 3 1003 OKI 0.00009999 0.009335060 0.2081730 0.661789 0.120602
## 4 1004 OKI 0.01060020 0.008091190 0.1115070 0.750293 0.119509
## 5 1005 OKI 0.02924580 0.015215500 0.0891502 0.758603 0.107786
## 6 1006 OKI 0.01443040 0.013455300 0.1430950 0.716448 0.112571
```

Import sample locations

```
sampling_loc <- readRDS(here("output", "populations", "sampling_loc.rds"))
head(sampling_loc)
```

```
## # A tibble: 6 × 6
##   Pop_City   Country  Latitude Longitude Region Abbreviation
##   <chr>      <chr>       <dbl>     <dbl> <chr>  <chr>       
## 1 Gelephu    Bhutan       26.9      90.5 Asia   GEL         
## 2 Phnom Penh Cambodia     11.6     105.  Asia   CAM         
## 3 Hainan     China        19.2     110.  Asia   HAI         
## 4 Yunnan     China        24.5     101.  Asia   YUN         
## 5 Hunan      China        27.6     112.  Asia   HUN         
## 6 Bengaluru  India        13.0      77.6 Asia   BEN
```

Structure plot

```
source(
  here(
    "scripts", "analysis", "my_theme3.R"
  )
)

# Create a named vector to map countries to regions
country_to_region <- c(
  "Bhutan" = "South Asia",
  "Cambodia" = "Southeast Asia",
  "China" = "East Asia",
  "India" = "South Asia",
  "Indonesia" = "Southeast Asia",
  "Japan" = "East Asia",
  "Malaysia" = "Southeast Asia",
  "Maldives" = "South Asia",
  "Nepal" = "South Asia",
  "Sri Lanka" = "South Asia",
  "Taiwan" = "East Asia",
  "Thailand" = "Southeast Asia",
  "Vietnam" = "Southeast Asia"
)

# Add the region to the data frame
sampling_loc$Region2 <- country_to_region[sampling_loc$Country]

# Melt the data frame for plotting
Q_melted <- leak5 |>
  pivot_longer(
    cols = -c(ind, pop),
    names_to = "variable",
    values_to = "value"
  )
# Join with sampling_loc to get sampling localities
Q_joined <- Q_melted |>
  left_join(sampling_loc, by = c("pop" = "Abbreviation"))

# Create a combined variable for Region and Country
Q_joined <- Q_joined |>
  mutate(Region_Country = interaction(Region, Country, sep = "_"))

# Order the combined variable by Region and Country, then by individual
Q_ordered <- Q_joined |>
  arrange(Region, Region2, Country, ind) |>
  mutate(ind = factor(ind, levels = unique(ind)))  # Convert ind to a factor with levels in the desired order

# Add labels: country names for the first individual in each country, NA for all other individuals
Q_ordered <- Q_ordered |>
  group_by(Region_Country) |>
  mutate(label = ifelse(row_number() == 1, as.character(Country), NA))

# Group by individual and variable, calculate mean ancestry proportions
Q_grouped <- Q_ordered |>
  group_by(ind, variable) |>
  summarise(value = mean(value), .groups = "drop")

# Create a data frame for borders
borders <-
  data.frame(Region_Country = unique(Q_ordered$Region_Country))

# Add the order of the last individual of each country to ensure correct placement of borders
borders$order <-
  sapply(borders$Region_Country, function(rc)
    max(which(Q_ordered$Region_Country == rc))) + 0.5  # Shift borders to the right edge of the bars

# Select only the first occurrence of each country in the ordered data
label_df <- Q_ordered |>
  filter(!is.na(label)) |>
  distinct(label, .keep_all = TRUE)

# Create a custom label function
label_func <- function(x) {
  labels <- rep("", length(x))
  labels[x %in% label_df$ind] <- label_df$label
  labels
}

# Calculate the position of lines
border_positions <- Q_ordered |>
  group_by(Country) |>
  summarise(pos = max(as.numeric(ind)) + 0)

# Calculate the position of population labels and bars
pop_labels <- Q_ordered |>
  mutate(Name = paste(pop, Pop_City, sep = " - ")) |>
  group_by(pop) |>
  slice_head(n = 1) |>
  ungroup() |>
  dplyr::select(ind, Pop_City, Country, Name) |>
  mutate(pos = as.numeric(ind))  # calculate position of population labels

pop_labels_bars <- pop_labels |>
  mutate(pos = as.numeric(ind)  - .5)


# Calculate the position of lines
border_positions <- Q_ordered |>
  group_by(Country) |>
  summarise(pos = max(as.numeric(ind)) - 1)


pop_labels_bars <- pop_labels |>
  mutate(pos = as.numeric(ind)  - .5)

# Function to filter and normalize data
normalize_data <- function(df, min_value) {
  df |>
    filter(value > min_value) |>
    group_by(ind) |>
    mutate(value = value / sum(value))
}

# Use the function
Q_grouped_filtered <- normalize_data(Q_grouped, 0.1)
# 
color_palette <-
  c(
    "v1" = "#FFFF00",
    "v2" = "#0000FF",
    "v3" = "#FF00FF",
    "v4" = "#FF0000",
    "v5" = "#00FF00"
  )

# Generate all potential variable names
all_variables <- paste0("v", 1:5)

# Map each variable to a name
color_mapping <- data.frame(variable = all_variables,
                            color = names(color_palette))

# Merge with Q_grouped_filtered
Q_grouped_filtered <- merge(Q_grouped_filtered, color_mapping, by = "variable")

# Create the plot
ggplot(Q_grouped_filtered, aes(x = as.factor(ind), y = value, fill = color)) +
  geom_bar(stat = 'identity', width = 1) +
  geom_vline(
    data = pop_labels_bars,
    aes(xintercept = pos),
    color = "#2C444A",
    linewidth = .2
  ) +
  geom_text(
    data = pop_labels,
    aes(x = as.numeric(ind), y = 1, label = Name),
    vjust = 1.5,
    hjust = 0,
    size = 2,
    angle = 90,
    inherit.aes = FALSE
  ) +
  my_theme() +
  theme(
    axis.text.x = element_text(
      angle = 90,
      hjust = 1,
      size = 12
    ),
    legend.position = "none",
    plot.margin = unit(c(3, 0.5, 0.5, 0.5), "cm")
  ) +
  xlab("Admixture matrix") +
  ylab("Ancestry proportions") +
  labs(caption = "Each bar represents the ancestry proportions for an individual for k=5.\n LEA inference for k1:25 for set3.") +
  scale_x_discrete(labels = label_func) +
  scale_fill_manual(values = color_palette) +
  expand_limits(y = c(0, 1.5))
```

```
ggsave(
  here("output", "variant_proportions", "figures", "set3.pdf"),
  width  = 12,
  height = 6,
  units  = "in",
  device = cairo_pdf
)
```

#### 3.1.2 Interploation over map

Clear memory and environment

```
# Clear entire environment
rm(list = ls())
# Forcefully trigger garbage collection
gc()
```

```
##           used  (Mb) gc trigger  (Mb) limit (Mb) max used  (Mb)
## Ncells 5254538 280.7    8748335 467.3         NA  8748335 467.3
## Vcells 8115545  62.0   38770990 295.8      32768 43249490 330.0
```

```
# Matrix
leak5 <- read_delim(
  here("output", "variant_proportions", "set3.snmf", "K5", "run1","set3_r1.5.Q"),
  delim = " ",
  col_names = FALSE,
  show_col_types = FALSE
) 
# unseen_pckmeans.7.Q
# pckmeans.7.Q
head(leak5)
```

```
## # A tibble: 6 × 5
##         X1       X2     X3    X4    X5
##      <dbl>    <dbl>  <dbl> <dbl> <dbl>
## 1 0.112    0.000100 0.135  0.555 0.198
## 2 0.137    0.0424   0.120  0.490 0.210
## 3 0.000100 0.00934  0.208  0.662 0.121
## 4 0.0106   0.00809  0.112  0.750 0.120
## 5 0.0292   0.0152   0.0892 0.759 0.108
## 6 0.0144   0.0135   0.143  0.716 0.113
```

The fam file

```
fam_file <- here(
  "output", "populations", "snps_sets", "neutral.fam"
)

# Read the .fam file
fam_data <- read.table(fam_file, 
                       header = FALSE,
                       col.names = c("FamilyID", "IndividualID", "PaternalID", "MaternalID", "Sex", "Phenotype"))

# View the first few rows
head(fam_data)
```

```
##   FamilyID IndividualID PaternalID MaternalID Sex Phenotype
## 1      OKI         1001          0          0   2        -9
## 2      OKI         1002          0          0   2        -9
## 3      OKI         1003          0          0   2        -9
## 4      OKI         1004          0          0   2        -9
## 5      OKI         1005          0          0   2        -9
## 6      OKI         1006          0          0   1        -9
```

Create ID column

```
# Change column name
colnames(fam_data)[colnames(fam_data) == "IndividualID"] <- "ind"

# Change column name
colnames(fam_data)[colnames(fam_data) == "FamilyID"] <- "pop"

# Select ID
fam_data <- fam_data |>
  dplyr::select("ind", "pop")

# View the first few rows
head(fam_data)
```

```
##    ind pop
## 1 1001 OKI
## 2 1002 OKI
## 3 1003 OKI
## 4 1004 OKI
## 5 1005 OKI
## 6 1006 OKI
```

Add it to matrix

```
leak5 <- fam_data |>
  dplyr::select(ind, pop) |>
  bind_cols(leak5)

head(leak5)
```

```
##    ind pop         X1          X2        X3       X4       X5
## 1 1001 OKI 0.11158400 0.000099991 0.1353900 0.554775 0.198151
## 2 1002 OKI 0.13734800 0.042359900 0.1202150 0.490371 0.209705
## 3 1003 OKI 0.00009999 0.009335060 0.2081730 0.661789 0.120602
## 4 1004 OKI 0.01060020 0.008091190 0.1115070 0.750293 0.119509
## 5 1005 OKI 0.02924580 0.015215500 0.0891502 0.758603 0.107786
## 6 1006 OKI 0.01443040 0.013455300 0.1430950 0.716448 0.112571
```

Rename the columns

```
# Rename the columns starting from the third one
leak5 <- leak5 |>
  rename_with(~paste0("v", seq_along(.x)), .cols = -c(ind, pop))

# View the first few rows
head(leak5)
```

```
##    ind pop         v1          v2        v3       v4       v5
## 1 1001 OKI 0.11158400 0.000099991 0.1353900 0.554775 0.198151
## 2 1002 OKI 0.13734800 0.042359900 0.1202150 0.490371 0.209705
## 3 1003 OKI 0.00009999 0.009335060 0.2081730 0.661789 0.120602
## 4 1004 OKI 0.01060020 0.008091190 0.1115070 0.750293 0.119509
## 5 1005 OKI 0.02924580 0.015215500 0.0891502 0.758603 0.107786
## 6 1006 OKI 0.01443040 0.013455300 0.1430950 0.716448 0.112571
```

Import samples attributes

```
sampling_loc <- readRDS(here("output", "populations", "sampling_loc.rds"))
# head(sampling_loc)

pops <- sampling_loc |>
  filter(
    Region == "Asia"
  ) |>
  dplyr::select(
    Abbreviation, Latitude, Longitude, Pop_City, Country
  )

head(pops)
```

```
## # A tibble: 6 × 5
##   Abbreviation Latitude Longitude Pop_City   Country 
##   <chr>           <dbl>     <dbl> <chr>      <chr>   
## 1 GEL              26.9      90.5 Gelephu    Bhutan  
## 2 CAM              11.6     105.  Phnom Penh Cambodia
## 3 HAI              19.2     110.  Hainan     China   
## 4 YUN              24.5     101.  Yunnan     China   
## 5 HUN              27.6     112.  Hunan      China   
## 6 BEN              13.0      77.6 Bengaluru  India
```

Merge with pops

```
# Add an index column to Q_tibble
leak5$index <- seq_len(nrow(leak5))

# Perform the merge as before
df1 <-
  merge(
    leak5,
    pops,
    by.x = 2,
    by.y = 1,
    all.x = T,
    all.y = F
  ) |>
  na.omit()

# Order by the index column to ensure the order matches the original Q_tibble
df1 <- df1[order(df1$index),]

# Optionally, you can remove the index column if it's no longer needed
df1$index <- NULL

# Now the rows of df1 should be in the same order as the original Q_tibble
head(df1)
```

```
##     pop  ind         v1          v2        v3       v4       v5 Latitude
## 159 OKI 1001 0.11158400 0.000099991 0.1353900 0.554775 0.198151  26.5013
## 160 OKI 1002 0.13734800 0.042359900 0.1202150 0.490371 0.209705  26.5013
## 161 OKI 1003 0.00009999 0.009335060 0.2081730 0.661789 0.120602  26.5013
## 162 OKI 1004 0.01060020 0.008091190 0.1115070 0.750293 0.119509  26.5013
## 163 OKI 1005 0.02924580 0.015215500 0.0891502 0.758603 0.107786  26.5013
## 164 OKI 1006 0.01443040 0.013455300 0.1430950 0.716448 0.112571  26.5013
##     Longitude Pop_City Country
## 159  127.9454  Okinawa   Japan
## 160  127.9454  Okinawa   Japan
## 161  127.9454  Okinawa   Japan
## 162  127.9454  Okinawa   Japan
## 163  127.9454  Okinawa   Japan
## 164  127.9454  Okinawa   Japan
```

We used this color palette to make the “structure” plot

```
color_palette2 <-
  c(
    "v1" = "#FFFF00",
    "v2" = "#0000FF",
    "v3" = "#FF00FF",
    "v4" = "#FF0000",
    "v5" = "#00FF00"
  )
```

Preparing the data for tess3Q\_map\_rasters

Make sure the lat longs are in the correct order and arrangement

```
df2 <- df1 |>
  dplyr::rename(
    Long = Longitude,
    Lat = Latitude
  )

long_lat_tibble <- df2 |>
  dplyr::select(Long, Lat)


long_lat_matrix <- long_lat_tibble |>
  as.matrix()

head(long_lat_matrix)
```

```
##         Long     Lat
## 159 127.9454 26.5013
## 160 127.9454 26.5013
## 161 127.9454 26.5013
## 162 127.9454 26.5013
## 163 127.9454 26.5013
## 164 127.9454 26.5013
```

Make a matrix of the Q values

Pull off the names of individuals and make a matrix of it:

```
Q_matrix <- leak5 |>
  dplyr::select(-ind, -pop, -index) |>
  as.matrix()
head(Q_matrix)
```

```
##              v1          v2        v3       v4       v5
## [1,] 0.11158400 0.000099991 0.1353900 0.554775 0.198151
## [2,] 0.13734800 0.042359900 0.1202150 0.490371 0.209705
## [3,] 0.00009999 0.009335060 0.2081730 0.661789 0.120602
## [4,] 0.01060020 0.008091190 0.1115070 0.750293 0.119509
## [5,] 0.02924580 0.015215500 0.0891502 0.758603 0.107786
## [6,] 0.01443040 0.013455300 0.1430950 0.716448 0.112571
```

Interpolate the Q-values by Kriging

```
print(ncol(Q_matrix) == length(color_palette2))
```

```
## [1] TRUE
```

Create brick

```
world <- ne_countries(scale = "medium", returnclass = "sf")
countries_with_data <- unique(df1$Country)

# Filtering the world data to include only the countries in your data
selected_countries <- world |>
  filter(admin %in% countries_with_data)

genoscape_brick <- tess3r::tess3Q_map_rasters(
  x = Q_matrix, 
  coord = long_lat_matrix,  
  map.polygon = selected_countries,
  window = extent(selected_countries)[1:4],
  # window = combined_extent,
  resolution = c(600,600), # if you want more cells in your raster, set higher
  # this next lines need to to be here, but don't do much...
  col.palette = tess3r::CreatePalette(color_palette2, length(color_palette2)),
  method = "map.max", 
  interpol = tess3r::FieldsKrigModel(80),  
  main = "Ancestry coefficients",
  xlab = "Longitude", 
  ylab = "Latitude", 
  cex = .4
)
```

```
## Warning: 
## Grid searches over lambda (nugget and sill variances) with  minima at the endpoints: 
##   (REML) Restricted maximum likelihood 
##    minimum at  right endpoint  lambda  =  0.02118404 (eff. df= 26.59999 )
## Warning: 
## Grid searches over lambda (nugget and sill variances) with  minima at the endpoints: 
##   (REML) Restricted maximum likelihood 
##    minimum at  right endpoint  lambda  =  0.02118404 (eff. df= 26.59999 )
## Warning: 
## Grid searches over lambda (nugget and sill variances) with  minima at the endpoints: 
##   (REML) Restricted maximum likelihood 
##    minimum at  right endpoint  lambda  =  0.02118404 (eff. df= 26.59999 )
## Warning: 
## Grid searches over lambda (nugget and sill variances) with  minima at the endpoints: 
##   (REML) Restricted maximum likelihood 
##    minimum at  right endpoint  lambda  =  0.02118404 (eff. df= 26.59999 )
## Warning: 
## Grid searches over lambda (nugget and sill variances) with  minima at the endpoints: 
##   (REML) Restricted maximum likelihood 
##    minimum at  right endpoint  lambda  =  0.02118404 (eff. df= 26.59999 )
```

```
# after that, we need to add names of the clusters back onto this raster brick
Q_tibble2 <- leak5 |>
  dplyr::select(
    -pop, -ind, -index
  )
names(genoscape_brick) <- names(Q_tibble2)[]
```

Scaling and cleaning the genoscape\_brick

```
genoscape_rgba <- genoscapeRtools::qprob_rando_raster(
  TRB = genoscape_brick,
  cols = color_palette2,
  alpha_scale = 2.0,
  abs_thresh = 0.0,
  alpha_exp = 1.55,
  alpha_chop_max = 255
)

# This adds the info for a regular lat-long projection
crs(genoscape_rgba) <- "+proj=longlat +datum=WGS84 +no_defs +ellps=WGS84 +towgs84=0,0,0"
```

Plot with pies

```
source(
  here(
    "scripts", "analysis", "my_theme2.R"
  )
)

# Calculate mean proportions for each population
df_mean <- df1 |>
  group_by(pop) |>
  summarise(across(starts_with("v"), \(x) mean(x, na.rm = TRUE)), 
            Longitude = mean(Longitude),
            Latitude = mean(Latitude))

ggplot() +
  layer_spatial(genoscape_rgba) +
  geom_spatial_point(data = long_lat_tibble,
                     mapping = aes(x = Long, y = Lat),
                     size = .2) +
  geom_text_repel(
    data = df_mean,
    aes(x = Longitude, y = Latitude, label = pop),
    size = 3,
    box.padding = unit(0.5, "lines")
  ) +
  labs(x = "Longitude",
       y = "Latitude") +
  geom_scatterpie(data = df_mean, 
                  aes(x = Longitude, y = Latitude, r = 1), 
                  cols = c("v1", "v2", "v3", "v4", "v5"), color = NA) +
  my_theme() +
  scale_fill_manual(values = color_palette2) +
  guides(fill = "none") +  # Hide legend
  coord_sf()
```

```
## Assuming `crs = 4326` in stat_spatial_identity()
```

```
# # 
ggsave(
  here("output", "variant_proportions", "figures", "set3_map.pdf"),
  width  = 6,
  height = 6,
  units  = "in",
  device = cairo_pdf
)
```

```
## Assuming `crs = 4326` in stat_spatial_identity()
```

### 3.2 set5

Clean env and memory

```
rm(list=ls())
gc()
```

```
##            used  (Mb) gc trigger  (Mb) limit (Mb) max used  (Mb)
## Ncells  5641735 301.4   15241385 814.0         NA 10604778 566.4
## Vcells 10447887  79.8   45026568 343.6      32768 56276493 429.4
```

Import the data

```
genotype <- here(
    "output", "variant_proportions", "set5.vcf"
  )

d <- read.vcfR(
  genotype
)
```

```
## Scanning file to determine attributes.
## File attributes:
##   meta lines: 8
##   header_line: 9
##   variant count: 33456
##   column count: 246
## 
Meta line 8 read in.
## All meta lines processed.
## gt matrix initialized.
## Character matrix gt created.
##   Character matrix gt rows: 33456
##   Character matrix gt cols: 246
##   skip: 0
##   nrows: 33456
##   row_num: 0
## 
Processed variant 1000
Processed variant 2000
Processed variant 3000
Processed variant 4000
Processed variant 5000
Processed variant 6000
Processed variant 7000
Processed variant 8000
Processed variant 9000
Processed variant 10000
Processed variant 11000
Processed variant 12000
Processed variant 13000
Processed variant 14000
Processed variant 15000
Processed variant 16000
Processed variant 17000
Processed variant 18000
Processed variant 19000
Processed variant 20000
Processed variant 21000
Processed variant 22000
Processed variant 23000
Processed variant 24000
Processed variant 25000
Processed variant 26000
Processed variant 27000
Processed variant 28000
Processed variant 29000
Processed variant 30000
Processed variant 31000
Processed variant 32000
Processed variant 33000
Processed variant: 33456
## All variants processed
```

Population and individuals information

```
inds_full <- attr(d@gt,"dimnames")[[2]]
inds_full <- inds_full[-1]
a <- strsplit(inds_full, '_')
pops <- unname(sapply(a, FUN = function(x) return(as.character(x[1])))) 
table(pops)
```

```
## pops
## BEN CAM CHA GEL HAI HAN HOC HUN INJ INW JAF KAC KAG KAN KAT KLP KUN LAM MAT OKI 
##  12  12  12   2  12   4   7  12  11   4   2   6  12  11  10   4   4   9  12  11 
## QNC SON SSK SUF SUU TAI UTS YUN 
##  12   3  12   6   6   8  12   9
```

```
pops <- factor(pops)
inds <- unname(sapply(a, FUN = function(x) return(as.character(x[2]))))
```

Convert format

```
vcf2geno(genotype, gsub(".vcf", ".geno", genotype))
```

```
## 
##  - number of detected individuals:   237
##  - number of detected loci:      33456
## 
## For SNP info, please check /Users/lucianocosme/Library/CloudStorage/Dropbox/Albopictus/manuscript_chip/data/no_autogenous/albo_chip/output/variant_proportions/set5.vcfsnp.
## 
## 0 line(s) were removed because these are not SNPs.
## Please, check /Users/lucianocosme/Library/CloudStorage/Dropbox/Albopictus/manuscript_chip/data/no_autogenous/albo_chip/output/variant_proportions/set5.removed file, for more informations.
```

```
## [1] "/Users/lucianocosme/Library/CloudStorage/Dropbox/Albopictus/manuscript_chip/data/no_autogenous/albo_chip/output/variant_proportions/set5.geno"
```

```
vcf2lfmm(genotype, gsub(".vcf", ".lfmm", genotype))
```

```
## 
##  - number of detected individuals:   237
##  - number of detected loci:      33456
## 
## For SNP info, please check /Users/lucianocosme/Library/CloudStorage/Dropbox/Albopictus/manuscript_chip/data/no_autogenous/albo_chip/output/variant_proportions/set5.vcfsnp.
## 
## 0 line(s) were removed because these are not SNPs.
## Please, check /Users/lucianocosme/Library/CloudStorage/Dropbox/Albopictus/manuscript_chip/data/no_autogenous/albo_chip/output/variant_proportions/set5.removed file, for more informations.
## 
## 
##  - number of detected individuals:   237
##  - number of detected loci:      33456
```

```
## [1] "/Users/lucianocosme/Library/CloudStorage/Dropbox/Albopictus/manuscript_chip/data/no_autogenous/albo_chip/output/variant_proportions/set5.lfmm"
```

Run LEA

```
# set output dir
setwd(
  here(
    "output", "variant_proportions"
  )
)
# main options
# K = number of ancestral populations
# entropy = TRUE computes the cross-entropy criterion, # CPU = 4 is the number of CPU used (hidden input) project = NULL
project = snmf(
  genotype,
  K = 1:25,
  project = "new",
  repetitions = 1,
  alpha = 5,
  tolerance = 0.0001,
  percentage = 0.5,
  iterations = 200, 
  ploidy = 2,
  seed = 13,
  CPU = 4,
  entropy = TRUE
)
```

```
project = load.snmfProject("output/variant_proportions/set5.snmfProject")
```

Cross entropy

```
# Open a new pdf file
pdf(here("output","variant_proportions","figures","cross_entropy_set5.pdf"), width = 6, height = 4)

# Create your plot
plot(project, col = "pink", pch = 19, cex = 1.2)

# Close the pdf file
dev.off()
```

```
## quartz_off_screen 
##                 2
```

```
plot(project, col = "pink", pch = 19, cex = 1.2)
```

#### 3.2.1 Structure plot

```
# Matrix
leak5 <- read_delim(
  here("output", "variant_proportions", "set5.snmf", "K5", "run1","set5_r1.5.Q"),
  delim = " ",
  col_names = FALSE,
  show_col_types = FALSE
) 
# unseen_pckmeans.7.Q
# pckmeans.7.Q
head(leak5)
```

```
## # A tibble: 6 × 5
##         X1    X2     X3     X4       X5
##      <dbl> <dbl>  <dbl>  <dbl>    <dbl>
## 1 0.175    0.575 0.196  0.0536 0.000100
## 2 0.243    0.499 0.216  0.0290 0.0129  
## 3 0.00335  0.719 0.0845 0.168  0.0253  
## 4 0.000100 0.778 0.0885 0.123  0.0103  
## 5 0.000100 0.781 0.0998 0.106  0.0123  
## 6 0.000100 0.762 0.0459 0.157  0.0358
```

The fam file

```
fam_file <- here(
  "output", "populations", "snps_sets", "neutral.fam"
)

# Read the .fam file
fam_data <- read.table(fam_file, 
                       header = FALSE,
                       col.names = c("FamilyID", "IndividualID", "PaternalID", "MaternalID", "Sex", "Phenotype"))

# View the first few rows
head(fam_data)
```

```
##   FamilyID IndividualID PaternalID MaternalID Sex Phenotype
## 1      OKI         1001          0          0   2        -9
## 2      OKI         1002          0          0   2        -9
## 3      OKI         1003          0          0   2        -9
## 4      OKI         1004          0          0   2        -9
## 5      OKI         1005          0          0   2        -9
## 6      OKI         1006          0          0   1        -9
```

Create ID column

```
# Change column name
colnames(fam_data)[colnames(fam_data) == "IndividualID"] <- "ind"

# Change column name
colnames(fam_data)[colnames(fam_data) == "FamilyID"] <- "pop"

# Select ID
fam_data <- fam_data |>
  dplyr::select("ind", "pop")

# View the first few rows
head(fam_data)
```

```
##    ind pop
## 1 1001 OKI
## 2 1002 OKI
## 3 1003 OKI
## 4 1004 OKI
## 5 1005 OKI
## 6 1006 OKI
```

Add it to matrix

```
leak5 <- fam_data |>
  dplyr::select(ind, pop) |>
  bind_cols(leak5)

head(leak5)
```

```
##    ind pop         X1       X2        X3        X4          X5
## 1 1001 OKI 0.17539600 0.574849 0.1960490 0.0536060 0.000099991
## 2 1002 OKI 0.24330500 0.499288 0.2155520 0.0289508 0.012905000
## 3 1003 OKI 0.00334552 0.719382 0.0844687 0.1675530 0.025250900
## 4 1004 OKI 0.00009999 0.777883 0.0884578 0.1232710 0.010288600
## 5 1005 OKI 0.00009999 0.781404 0.0997505 0.1064060 0.012339100
## 6 1006 OKI 0.00009999 0.761591 0.0459112 0.1566220 0.035775400
```

Rename the columns

```
# Rename the columns starting from the third one
leak5 <- leak5 |>
  rename_with(~paste0("v", seq_along(.x)), .cols = -c(ind, pop))

# View the first few rows
head(leak5)
```

```
##    ind pop         v1       v2        v3        v4          v5
## 1 1001 OKI 0.17539600 0.574849 0.1960490 0.0536060 0.000099991
## 2 1002 OKI 0.24330500 0.499288 0.2155520 0.0289508 0.012905000
## 3 1003 OKI 0.00334552 0.719382 0.0844687 0.1675530 0.025250900
## 4 1004 OKI 0.00009999 0.777883 0.0884578 0.1232710 0.010288600
## 5 1005 OKI 0.00009999 0.781404 0.0997505 0.1064060 0.012339100
## 6 1006 OKI 0.00009999 0.761591 0.0459112 0.1566220 0.035775400
```

Import sample locations

```
sampling_loc <- readRDS(here("output", "populations", "sampling_loc.rds"))
head(sampling_loc)
```

```
## # A tibble: 6 × 6
##   Pop_City   Country  Latitude Longitude Region Abbreviation
##   <chr>      <chr>       <dbl>     <dbl> <chr>  <chr>       
## 1 Gelephu    Bhutan       26.9      90.5 Asia   GEL         
## 2 Phnom Penh Cambodia     11.6     105.  Asia   CAM         
## 3 Hainan     China        19.2     110.  Asia   HAI         
## 4 Yunnan     China        24.5     101.  Asia   YUN         
## 5 Hunan      China        27.6     112.  Asia   HUN         
## 6 Bengaluru  India        13.0      77.6 Asia   BEN
```

Structure plot

```
source(
  here(
    "scripts", "analysis", "my_theme3.R"
  )
)

# Create a named vector to map countries to regions
country_to_region <- c(
  "Bhutan" = "South Asia",
  "Cambodia" = "Southeast Asia",
  "China" = "East Asia",
  "India" = "South Asia",
  "Indonesia" = "Southeast Asia",
  "Japan" = "East Asia",
  "Malaysia" = "Southeast Asia",
  "Maldives" = "South Asia",
  "Nepal" = "South Asia",
  "Sri Lanka" = "South Asia",
  "Taiwan" = "East Asia",
  "Thailand" = "Southeast Asia",
  "Vietnam" = "Southeast Asia"
)

# Add the region to the data frame
sampling_loc$Region2 <- country_to_region[sampling_loc$Country]

# Melt the data frame for plotting
Q_melted <- leak5 |>
  pivot_longer(
    cols = -c(ind, pop),
    names_to = "variable",
    values_to = "value"
  )
# Join with sampling_loc to get sampling localities
Q_joined <- Q_melted |>
  left_join(sampling_loc, by = c("pop" = "Abbreviation"))

# Create a combined variable for Region and Country
Q_joined <- Q_joined |>
  mutate(Region_Country = interaction(Region, Country, sep = "_"))

# Order the combined variable by Region and Country, then by individual
Q_ordered <- Q_joined |>
  arrange(Region, Region2, Country, ind) |>
  mutate(ind = factor(ind, levels = unique(ind)))  # Convert ind to a factor with levels in the desired order

# Add labels: country names for the first individual in each country, NA for all other individuals
Q_ordered <- Q_ordered |>
  group_by(Region_Country) |>
  mutate(label = ifelse(row_number() == 1, as.character(Country), NA))

# Group by individual and variable, calculate mean ancestry proportions
Q_grouped <- Q_ordered |>
  group_by(ind, variable) |>
  summarise(value = mean(value), .groups = "drop")

# Create a data frame for borders
borders <-
  data.frame(Region_Country = unique(Q_ordered$Region_Country))

# Add the order of the last individual of each country to ensure correct placement of borders
borders$order <-
  sapply(borders$Region_Country, function(rc)
    max(which(Q_ordered$Region_Country == rc))) + 0.5  # Shift borders to the right edge of the bars

# Select only the first occurrence of each country in the ordered data
label_df <- Q_ordered |>
  filter(!is.na(label)) |>
  distinct(label, .keep_all = TRUE)

# Create a custom label function
label_func <- function(x) {
  labels <- rep("", length(x))
  labels[x %in% label_df$ind] <- label_df$label
  labels
}

# Calculate the position of lines
border_positions <- Q_ordered |>
  group_by(Country) |>
  summarise(pos = max(as.numeric(ind)) + 0)

# Calculate the position of population labels and bars
pop_labels <- Q_ordered |>
  mutate(Name = paste(pop, Pop_City, sep = " - ")) |>
  group_by(pop) |>
  slice_head(n = 1) |>
  ungroup() |>
  dplyr::select(ind, Pop_City, Country, Name) |>
  mutate(pos = as.numeric(ind))  # calculate position of population labels

pop_labels_bars <- pop_labels |>
  mutate(pos = as.numeric(ind)  - .5)


# Calculate the position of lines
border_positions <- Q_ordered |>
  group_by(Country) |>
  summarise(pos = max(as.numeric(ind)) - 1)


pop_labels_bars <- pop_labels |>
  mutate(pos = as.numeric(ind)  - .5)

# Function to filter and normalize data
normalize_data <- function(df, min_value) {
  df |>
    filter(value > min_value) |>
    group_by(ind) |>
    mutate(value = value / sum(value))
}

# Use the function
Q_grouped_filtered <- normalize_data(Q_grouped, 0.1)
# 
color_palette <-
  c(
    "v1" = "#FFFF00",
    "v2" = "#FF0000",
    "v3" = "#00FF00",
    "v4" = "#FF00FF",
    "v5" = "#0000FF"
  )

# Generate all potential variable names
all_variables <- paste0("v", 1:5)

# Map each variable to a name
color_mapping <- data.frame(variable = all_variables,
                            color = names(color_palette))

# Merge with Q_grouped_filtered
Q_grouped_filtered <- merge(Q_grouped_filtered, color_mapping, by = "variable")

# Create the plot
ggplot(Q_grouped_filtered, aes(x = as.factor(ind), y = value, fill = color)) +
  geom_bar(stat = 'identity', width = 1) +
  geom_vline(
    data = pop_labels_bars,
    aes(xintercept = pos),
    color = "#2C444A",
    linewidth = .2
  ) +
  geom_text(
    data = pop_labels,
    aes(x = as.numeric(ind), y = 1, label = Name),
    vjust = 1.5,
    hjust = 0,
    size = 2,
    angle = 90,
    inherit.aes = FALSE
  ) +
  my_theme() +
  theme(
    axis.text.x = element_text(
      angle = 90,
      hjust = 1,
      size = 12
    ),
    legend.position = "none",
    plot.margin = unit(c(3, 0.5, 0.5, 0.5), "cm")
  ) +
  xlab("Admixture matrix") +
  ylab("Ancestry proportions") +
  labs(caption = "Each bar represents the ancestry proportions for an individual for k=5.\n LEA inference for k1:25 for set3.") +
  scale_x_discrete(labels = label_func) +
  scale_fill_manual(values = color_palette) +
  expand_limits(y = c(0, 1.5))
```

```
ggsave(
  here("output", "variant_proportions", "figures", "set5.pdf"),
  width  = 12,
  height = 6,
  units  = "in",
  device = cairo_pdf
)
```

#### 3.2.2 Interploation over map

Clear memory and environment

```
# Clear entire environment
rm(list = ls())
# Forcefully trigger garbage collection
gc()
```

```
##           used  (Mb) gc trigger  (Mb) limit (Mb) max used  (Mb)
## Ncells 5641980 301.4   15241385 814.0         NA 10604778 566.4
## Vcells 9018152  68.9   36021255 274.9      32768 56276493 429.4
```

```
# Matrix
leak5 <- read_delim(
  here("output", "variant_proportions", "set5.snmf", "K5", "run1","set5_r1.5.Q"),
  delim = " ",
  col_names = FALSE,
  show_col_types = FALSE
) 

head(leak5)
```

```
## # A tibble: 6 × 5
##         X1    X2     X3     X4       X5
##      <dbl> <dbl>  <dbl>  <dbl>    <dbl>
## 1 0.175    0.575 0.196  0.0536 0.000100
## 2 0.243    0.499 0.216  0.0290 0.0129  
## 3 0.00335  0.719 0.0845 0.168  0.0253  
## 4 0.000100 0.778 0.0885 0.123  0.0103  
## 5 0.000100 0.781 0.0998 0.106  0.0123  
## 6 0.000100 0.762 0.0459 0.157  0.0358
```

The fam file

```
fam_file <- here(
  "output", "populations", "snps_sets", "neutral.fam"
)

# Read the .fam file
fam_data <- read.table(fam_file, 
                       header = FALSE,
                       col.names = c("FamilyID", "IndividualID", "PaternalID", "MaternalID", "Sex", "Phenotype"))

# View the first few rows
head(fam_data)
```

```
##   FamilyID IndividualID PaternalID MaternalID Sex Phenotype
## 1      OKI         1001          0          0   2        -9
## 2      OKI         1002          0          0   2        -9
## 3      OKI         1003          0          0   2        -9
## 4      OKI         1004          0          0   2        -9
## 5      OKI         1005          0          0   2        -9
## 6      OKI         1006          0          0   1        -9
```

Create ID column

```
# Change column name
colnames(fam_data)[colnames(fam_data) == "IndividualID"] <- "ind"

# Change column name
colnames(fam_data)[colnames(fam_data) == "FamilyID"] <- "pop"

# Select ID
fam_data <- fam_data |>
  dplyr::select("ind", "pop")

# View the first few rows
head(fam_data)
```

```
##    ind pop
## 1 1001 OKI
## 2 1002 OKI
## 3 1003 OKI
## 4 1004 OKI
## 5 1005 OKI
## 6 1006 OKI
```

Add it to matrix

```
leak5 <- fam_data |>
  dplyr::select(ind, pop) |>
  bind_cols(leak5)

head(leak5)
```

```
##    ind pop         X1       X2        X3        X4          X5
## 1 1001 OKI 0.17539600 0.574849 0.1960490 0.0536060 0.000099991
## 2 1002 OKI 0.24330500 0.499288 0.2155520 0.0289508 0.012905000
## 3 1003 OKI 0.00334552 0.719382 0.0844687 0.1675530 0.025250900
## 4 1004 OKI 0.00009999 0.777883 0.0884578 0.1232710 0.010288600
## 5 1005 OKI 0.00009999 0.781404 0.0997505 0.1064060 0.012339100
## 6 1006 OKI 0.00009999 0.761591 0.0459112 0.1566220 0.035775400
```

Rename the columns

```
# Rename the columns starting from the third one
leak5 <- leak5 |>
  rename_with(~paste0("v", seq_along(.x)), .cols = -c(ind, pop))

# View the first few rows
head(leak5)
```

```
##    ind pop         v1       v2        v3        v4          v5
## 1 1001 OKI 0.17539600 0.574849 0.1960490 0.0536060 0.000099991
## 2 1002 OKI 0.24330500 0.499288 0.2155520 0.0289508 0.012905000
## 3 1003 OKI 0.00334552 0.719382 0.0844687 0.1675530 0.025250900
## 4 1004 OKI 0.00009999 0.777883 0.0884578 0.1232710 0.010288600
## 5 1005 OKI 0.00009999 0.781404 0.0997505 0.1064060 0.012339100
## 6 1006 OKI 0.00009999 0.761591 0.0459112 0.1566220 0.035775400
```

Import samples attributes

```
sampling_loc <- readRDS(here("output", "populations", "sampling_loc.rds"))
# head(sampling_loc)

pops <- sampling_loc |>
  filter(
    Region == "Asia"
  ) |>
  dplyr::select(
    Abbreviation, Latitude, Longitude, Pop_City, Country
  )

head(pops)
```

```
## # A tibble: 6 × 5
##   Abbreviation Latitude Longitude Pop_City   Country 
##   <chr>           <dbl>     <dbl> <chr>      <chr>   
## 1 GEL              26.9      90.5 Gelephu    Bhutan  
## 2 CAM              11.6     105.  Phnom Penh Cambodia
## 3 HAI              19.2     110.  Hainan     China   
## 4 YUN              24.5     101.  Yunnan     China   
## 5 HUN              27.6     112.  Hunan      China   
## 6 BEN              13.0      77.6 Bengaluru  India
```

Merge with pops

```
# Add an index column to Q_tibble
leak5$index <- seq_len(nrow(leak5))

# Perform the merge as before
df1 <-
  merge(
    leak5,
    pops,
    by.x = 2,
    by.y = 1,
    all.x = T,
    all.y = F
  ) |>
  na.omit()

# Order by the index column to ensure the order matches the original Q_tibble
df1 <- df1[order(df1$index),]

# Optionally, you can remove the index column if it's no longer needed
df1$index <- NULL

# Now the rows of df1 should be in the same order as the original Q_tibble
head(df1)
```

```
##     pop  ind         v1       v2        v3        v4          v5 Latitude
## 159 OKI 1001 0.17539600 0.574849 0.1960490 0.0536060 0.000099991  26.5013
## 160 OKI 1002 0.24330500 0.499288 0.2155520 0.0289508 0.012905000  26.5013
## 161 OKI 1003 0.00334552 0.719382 0.0844687 0.1675530 0.025250900  26.5013
## 162 OKI 1004 0.00009999 0.777883 0.0884578 0.1232710 0.010288600  26.5013
## 163 OKI 1005 0.00009999 0.781404 0.0997505 0.1064060 0.012339100  26.5013
## 164 OKI 1006 0.00009999 0.761591 0.0459112 0.1566220 0.035775400  26.5013
##     Longitude Pop_City Country
## 159  127.9454  Okinawa   Japan
## 160  127.9454  Okinawa   Japan
## 161  127.9454  Okinawa   Japan
## 162  127.9454  Okinawa   Japan
## 163  127.9454  Okinawa   Japan
## 164  127.9454  Okinawa   Japan
```

We used this color palette to make the “structure” plot

```
color_palette2 <-
  c(
    "v1" = "#FFFF00",
    "v2" = "#FF0000",
    "v3" = "#00FF00",
    "v4" = "#FF00FF",
    "v5" = "#0000FF"
  )
```

Preparing the data for tess3Q\_map\_rasters

Make sure the lat longs are in the correct order and arrangement

```
df2 <- df1 |>
  dplyr::rename(
    Long = Longitude,
    Lat = Latitude
  )

long_lat_tibble <- df2 |>
  dplyr::select(Long, Lat)


long_lat_matrix <- long_lat_tibble |>
  as.matrix()

head(long_lat_matrix)
```

```
##         Long     Lat
## 159 127.9454 26.5013
## 160 127.9454 26.5013
## 161 127.9454 26.5013
## 162 127.9454 26.5013
## 163 127.9454 26.5013
## 164 127.9454 26.5013
```

Make a matrix of the Q values

Pull off the names of individuals and make a matrix of it:

```
Q_matrix <- leak5 |>
  dplyr::select(-ind, -pop, -index) |>
  as.matrix()
head(Q_matrix)
```

```
##              v1       v2        v3        v4          v5
## [1,] 0.17539600 0.574849 0.1960490 0.0536060 0.000099991
## [2,] 0.24330500 0.499288 0.2155520 0.0289508 0.012905000
## [3,] 0.00334552 0.719382 0.0844687 0.1675530 0.025250900
## [4,] 0.00009999 0.777883 0.0884578 0.1232710 0.010288600
## [5,] 0.00009999 0.781404 0.0997505 0.1064060 0.012339100
## [6,] 0.00009999 0.761591 0.0459112 0.1566220 0.035775400
```

Interpolate the Q-values by Kriging

```
print(ncol(Q_matrix) == length(color_palette2))
```

```
## [1] TRUE
```

Create brick

```
world <- ne_countries(scale = "medium", returnclass = "sf")
countries_with_data <- unique(df1$Country)

# Filtering the world data to include only the countries in your data
selected_countries <- world |>
  filter(admin %in% countries_with_data)

genoscape_brick <- tess3r::tess3Q_map_rasters(
  x = Q_matrix, 
  coord = long_lat_matrix,  
  map.polygon = selected_countries,
  window = extent(selected_countries)[1:4],
  # window = combined_extent,
  resolution = c(600,600), # if you want more cells in your raster, set higher
  # this next lines need to to be here, but don't do much...
  col.palette = tess3r::CreatePalette(color_palette2, length(color_palette2)),
  method = "map.max", 
  interpol = tess3r::FieldsKrigModel(80),  
  main = "Ancestry coefficients",
  xlab = "Longitude", 
  ylab = "Latitude", 
  cex = .4
)
```

```
## Warning: 
## Grid searches over lambda (nugget and sill variances) with  minima at the endpoints: 
##   (REML) Restricted maximum likelihood 
##    minimum at  right endpoint  lambda  =  0.02118404 (eff. df= 26.59999 )
## Warning: 
## Grid searches over lambda (nugget and sill variances) with  minima at the endpoints: 
##   (REML) Restricted maximum likelihood 
##    minimum at  right endpoint  lambda  =  0.02118404 (eff. df= 26.59999 )
## Warning: 
## Grid searches over lambda (nugget and sill variances) with  minima at the endpoints: 
##   (REML) Restricted maximum likelihood 
##    minimum at  right endpoint  lambda  =  0.02118404 (eff. df= 26.59999 )
## Warning: 
## Grid searches over lambda (nugget and sill variances) with  minima at the endpoints: 
##   (REML) Restricted maximum likelihood 
##    minimum at  right endpoint  lambda  =  0.02118404 (eff. df= 26.59999 )
## Warning: 
## Grid searches over lambda (nugget and sill variances) with  minima at the endpoints: 
##   (REML) Restricted maximum likelihood 
##    minimum at  right endpoint  lambda  =  0.02118404 (eff. df= 26.59999 )
```

```
# after that, we need to add names of the clusters back onto this raster brick
Q_tibble2 <- leak5 |>
  dplyr::select(
    -pop, -ind, -index
  )
names(genoscape_brick) <- names(Q_tibble2)[]
```

Scaling and cleaning the genoscape\_brick

```
genoscape_rgba <- genoscapeRtools::qprob_rando_raster(
  TRB = genoscape_brick,
  cols = color_palette2,
  alpha_scale = 2.0,
  abs_thresh = 0.0,
  alpha_exp = 1.55,
  alpha_chop_max = 255
)

# This adds the info for a regular lat-long projection
crs(genoscape_rgba) <- "+proj=longlat +datum=WGS84 +no_defs +ellps=WGS84 +towgs84=0,0,0"
```

Plot with pies

```
source(
  here(
    "scripts", "analysis", "my_theme2.R"
  )
)

# Calculate mean proportions for each population
df_mean <- df1 |>
  group_by(pop) |>
  summarise(across(starts_with("v"), \(x) mean(x, na.rm = TRUE)), 
            Longitude = mean(Longitude),
            Latitude = mean(Latitude))

ggplot() +
  layer_spatial(genoscape_rgba) +
  geom_spatial_point(data = long_lat_tibble,
                     mapping = aes(x = Long, y = Lat),
                     size = .2) +
  geom_text_repel(
    data = df_mean,
    aes(x = Longitude, y = Latitude, label = pop),
    size = 3,
    box.padding = unit(0.5, "lines")
  ) +
  labs(x = "Longitude",
       y = "Latitude") +
  geom_scatterpie(data = df_mean, 
                  aes(x = Longitude, y = Latitude, r = 1), 
                  cols = c("v1", "v2", "v3", "v4", "v5"), color = NA) +
  my_theme() +
  scale_fill_manual(values = color_palette2) +
  guides(fill = "none") +  # Hide legend
  coord_sf()
```

```
## Assuming `crs = 4326` in stat_spatial_identity()
```

```
# # 
ggsave(
  here("output", "variant_proportions", "figures", "set5_map.pdf"),
  width  = 6,
  height = 6,
  units  = "in",
  device = cairo_pdf
)
```

```
## Assuming `crs = 4326` in stat_spatial_identity()
```

### 3.3 set10

Clean env and memory

```
rm(list=ls())
gc()
```

```
##            used  (Mb) gc trigger  (Mb) limit (Mb) max used  (Mb)
## Ncells  5642620 301.4   15241385 814.0         NA 15241385 814.0
## Vcells 10461397  79.9   41647420 317.8      32768 56276493 429.4
```

Import the data

```
genotype <- here(
    "output", "variant_proportions", "set10.vcf"
  )

d <- read.vcfR(
  genotype
)
```

```
## Scanning file to determine attributes.
## File attributes:
##   meta lines: 8
##   header_line: 9
##   variant count: 33392
##   column count: 246
## 
Meta line 8 read in.
## All meta lines processed.
## gt matrix initialized.
## Character matrix gt created.
##   Character matrix gt rows: 33392
##   Character matrix gt cols: 246
##   skip: 0
##   nrows: 33392
##   row_num: 0
## 
Processed variant 1000
Processed variant 2000
Processed variant 3000
Processed variant 4000
Processed variant 5000
Processed variant 6000
Processed variant 7000
Processed variant 8000
Processed variant 9000
Processed variant 10000
Processed variant 11000
Processed variant 12000
Processed variant 13000
Processed variant 14000
Processed variant 15000
Processed variant 16000
Processed variant 17000
Processed variant 18000
Processed variant 19000
Processed variant 20000
Processed variant 21000
Processed variant 22000
Processed variant 23000
Processed variant 24000
Processed variant 25000
Processed variant 26000
Processed variant 27000
Processed variant 28000
Processed variant 29000
Processed variant 30000
Processed variant 31000
Processed variant 32000
Processed variant 33000
Processed variant: 33392
## All variants processed
```

Population and individuals information

```
inds_full <- attr(d@gt,"dimnames")[[2]]
inds_full <- inds_full[-1]
a <- strsplit(inds_full, '_')
pops <- unname(sapply(a, FUN = function(x) return(as.character(x[1])))) 
table(pops)
```

```
## pops
## BEN CAM CHA GEL HAI HAN HOC HUN INJ INW JAF KAC KAG KAN KAT KLP KUN LAM MAT OKI 
##  12  12  12   2  12   4   7  12  11   4   2   6  12  11  10   4   4   9  12  11 
## QNC SON SSK SUF SUU TAI UTS YUN 
##  12   3  12   6   6   8  12   9
```

```
pops <- factor(pops)
inds <- unname(sapply(a, FUN = function(x) return(as.character(x[2]))))
```

Convert format

```
vcf2geno(genotype, gsub(".vcf", ".geno", genotype))
```

```
## 
##  - number of detected individuals:   237
##  - number of detected loci:      33392
## 
## For SNP info, please check /Users/lucianocosme/Library/CloudStorage/Dropbox/Albopictus/manuscript_chip/data/no_autogenous/albo_chip/output/variant_proportions/set10.vcfsnp.
## 
## 0 line(s) were removed because these are not SNPs.
## Please, check /Users/lucianocosme/Library/CloudStorage/Dropbox/Albopictus/manuscript_chip/data/no_autogenous/albo_chip/output/variant_proportions/set10.removed file, for more informations.
```

```
## [1] "/Users/lucianocosme/Library/CloudStorage/Dropbox/Albopictus/manuscript_chip/data/no_autogenous/albo_chip/output/variant_proportions/set10.geno"
```

```
vcf2lfmm(genotype, gsub(".vcf", ".lfmm", genotype))
```

```
## 
##  - number of detected individuals:   237
##  - number of detected loci:      33392
## 
## For SNP info, please check /Users/lucianocosme/Library/CloudStorage/Dropbox/Albopictus/manuscript_chip/data/no_autogenous/albo_chip/output/variant_proportions/set10.vcfsnp.
## 
## 0 line(s) were removed because these are not SNPs.
## Please, check /Users/lucianocosme/Library/CloudStorage/Dropbox/Albopictus/manuscript_chip/data/no_autogenous/albo_chip/output/variant_proportions/set10.removed file, for more informations.
## 
## 
##  - number of detected individuals:   237
##  - number of detected loci:      33392
```

```
## [1] "/Users/lucianocosme/Library/CloudStorage/Dropbox/Albopictus/manuscript_chip/data/no_autogenous/albo_chip/output/variant_proportions/set10.lfmm"
```

Run LEA

```
# set output dir
setwd(
  here(
    "output", "variant_proportions"
  )
)
# main options
# K = number of ancestral populations
# entropy = TRUE computes the cross-entropy criterion, # CPU = 4 is the number of CPU used (hidden input) project = NULL
project = snmf(
  genotype,
  K = 1:25,
  project = "new",
  repetitions = 1,
  alpha = 5,
  tolerance = 0.0001,
  percentage = 0.5,
  iterations = 200, 
  ploidy = 2,
  seed = 13,
  CPU = 4,
  entropy = TRUE
)
```

```
project = load.snmfProject("output/variant_proportions/set10.snmfProject")
```

Cross entropy

```
# Open a new pdf file
pdf(here("output","variant_proportions","figures","cross_entropy_set10.pdf"), width = 6, height = 4)

# Create your plot
plot(project, col = "pink", pch = 19, cex = 1.2)

# Close the pdf file
dev.off()
```

```
## quartz_off_screen 
##                 2
```

```
plot(project, col = "pink", pch = 19, cex = 1.2)
```

#### 3.3.1 Structure plot

```
# Matrix
leak5 <- read_delim(
  here("output", "variant_proportions", "set10.snmf", "K5", "run1","set10_r1.5.Q"),
  delim = " ",
  col_names = FALSE,
  show_col_types = FALSE
) 


head(leak5)
```

```
## # A tibble: 6 × 5
##       X1     X2       X3       X4    X5
##    <dbl>  <dbl>    <dbl>    <dbl> <dbl>
## 1 0.116  0.173  0.0175   0.0708   0.623
## 2 0.0651 0.201  0.0382   0.171    0.524
## 3 0.157  0.146  0.000100 0.000100 0.697
## 4 0.0970 0.0885 0.0196   0.000100 0.795
## 5 0.116  0.0452 0.0236   0.000100 0.815
## 6 0.102  0.107  0.0148   0.0584   0.718
```

The fam file

```
fam_file <- here(
  "output", "populations", "snps_sets", "neutral.fam"
)

# Read the .fam file
fam_data <- read.table(fam_file, 
                       header = FALSE,
                       col.names = c("FamilyID", "IndividualID", "PaternalID", "MaternalID", "Sex", "Phenotype"))

# View the first few rows
head(fam_data)
```

```
##   FamilyID IndividualID PaternalID MaternalID Sex Phenotype
## 1      OKI         1001          0          0   2        -9
## 2      OKI         1002          0          0   2        -9
## 3      OKI         1003          0          0   2        -9
## 4      OKI         1004          0          0   2        -9
## 5      OKI         1005          0          0   2        -9
## 6      OKI         1006          0          0   1        -9
```

Create ID column

```
# Change column name
colnames(fam_data)[colnames(fam_data) == "IndividualID"] <- "ind"

# Change column name
colnames(fam_data)[colnames(fam_data) == "FamilyID"] <- "pop"

# Select ID
fam_data <- fam_data |>
  dplyr::select("ind", "pop")

# View the first few rows
head(fam_data)
```

```
##    ind pop
## 1 1001 OKI
## 2 1002 OKI
## 3 1003 OKI
## 4 1004 OKI
## 5 1005 OKI
## 6 1006 OKI
```

Add it to matrix

```
leak5 <- fam_data |>
  dplyr::select(ind, pop) |>
  bind_cols(leak5)

head(leak5)
```

```
##    ind pop        X1        X2          X3          X4       X5
## 1 1001 OKI 0.1163710 0.1725730 0.017452600 0.070828600 0.622774
## 2 1002 OKI 0.0651225 0.2014900 0.038158600 0.171094000 0.524135
## 3 1003 OKI 0.1570890 0.1460220 0.000099982 0.000099982 0.696689
## 4 1004 OKI 0.0969622 0.0884783 0.019564000 0.000099991 0.794896
## 5 1005 OKI 0.1163020 0.0452408 0.023569900 0.000099991 0.814787
## 6 1006 OKI 0.1021300 0.1066390 0.014846300 0.058365400 0.718019
```

Rename the columns

```
# Rename the columns starting from the third one
leak5 <- leak5 |>
  rename_with(~paste0("v", seq_along(.x)), .cols = -c(ind, pop))

# View the first few rows
head(leak5)
```

```
##    ind pop        v1        v2          v3          v4       v5
## 1 1001 OKI 0.1163710 0.1725730 0.017452600 0.070828600 0.622774
## 2 1002 OKI 0.0651225 0.2014900 0.038158600 0.171094000 0.524135
## 3 1003 OKI 0.1570890 0.1460220 0.000099982 0.000099982 0.696689
## 4 1004 OKI 0.0969622 0.0884783 0.019564000 0.000099991 0.794896
## 5 1005 OKI 0.1163020 0.0452408 0.023569900 0.000099991 0.814787
## 6 1006 OKI 0.1021300 0.1066390 0.014846300 0.058365400 0.718019
```

Import sample locations

```
sampling_loc <- readRDS(here("output", "populations", "sampling_loc.rds"))
head(sampling_loc)
```

```
## # A tibble: 6 × 6
##   Pop_City   Country  Latitude Longitude Region Abbreviation
##   <chr>      <chr>       <dbl>     <dbl> <chr>  <chr>       
## 1 Gelephu    Bhutan       26.9      90.5 Asia   GEL         
## 2 Phnom Penh Cambodia     11.6     105.  Asia   CAM         
## 3 Hainan     China        19.2     110.  Asia   HAI         
## 4 Yunnan     China        24.5     101.  Asia   YUN         
## 5 Hunan      China        27.6     112.  Asia   HUN         
## 6 Bengaluru  India        13.0      77.6 Asia   BEN
```

Structure plot

```
source(
  here(
    "scripts", "analysis", "my_theme3.R"
  )
)

# Create a named vector to map countries to regions
country_to_region <- c(
  "Bhutan" = "South Asia",
  "Cambodia" = "Southeast Asia",
  "China" = "East Asia",
  "India" = "South Asia",
  "Indonesia" = "Southeast Asia",
  "Japan" = "East Asia",
  "Malaysia" = "Southeast Asia",
  "Maldives" = "South Asia",
  "Nepal" = "South Asia",
  "Sri Lanka" = "South Asia",
  "Taiwan" = "East Asia",
  "Thailand" = "Southeast Asia",
  "Vietnam" = "Southeast Asia"
)

# Add the region to the data frame
sampling_loc$Region2 <- country_to_region[sampling_loc$Country]

# Melt the data frame for plotting
Q_melted <- leak5 |>
  pivot_longer(
    cols = -c(ind, pop),
    names_to = "variable",
    values_to = "value"
  )
# Join with sampling_loc to get sampling localities
Q_joined <- Q_melted |>
  left_join(sampling_loc, by = c("pop" = "Abbreviation"))

# Create a combined variable for Region and Country
Q_joined <- Q_joined |>
  mutate(Region_Country = interaction(Region, Country, sep = "_"))

# Order the combined variable by Region and Country, then by individual
Q_ordered <- Q_joined |>
  arrange(Region, Region2, Country, ind) |>
  mutate(ind = factor(ind, levels = unique(ind)))  # Convert ind to a factor with levels in the desired order

# Add labels: country names for the first individual in each country, NA for all other individuals
Q_ordered <- Q_ordered |>
  group_by(Region_Country) |>
  mutate(label = ifelse(row_number() == 1, as.character(Country), NA))

# Group by individual and variable, calculate mean ancestry proportions
Q_grouped <- Q_ordered |>
  group_by(ind, variable) |>
  summarise(value = mean(value), .groups = "drop")

# Create a data frame for borders
borders <-
  data.frame(Region_Country = unique(Q_ordered$Region_Country))

# Add the order of the last individual of each country to ensure correct placement of borders
borders$order <-
  sapply(borders$Region_Country, function(rc)
    max(which(Q_ordered$Region_Country == rc))) + 0.5  # Shift borders to the right edge of the bars

# Select only the first occurrence of each country in the ordered data
label_df <- Q_ordered |>
  filter(!is.na(label)) |>
  distinct(label, .keep_all = TRUE)

# Create a custom label function
label_func <- function(x) {
  labels <- rep("", length(x))
  labels[x %in% label_df$ind] <- label_df$label
  labels
}

# Calculate the position of lines
border_positions <- Q_ordered |>
  group_by(Country) |>
  summarise(pos = max(as.numeric(ind)) + 0)

# Calculate the position of population labels and bars
pop_labels <- Q_ordered |>
  mutate(Name = paste(pop, Pop_City, sep = " - ")) |>
  group_by(pop) |>
  slice_head(n = 1) |>
  ungroup() |>
  dplyr::select(ind, Pop_City, Country, Name) |>
  mutate(pos = as.numeric(ind))  # calculate position of population labels

pop_labels_bars <- pop_labels |>
  mutate(pos = as.numeric(ind)  - .5)


# Calculate the position of lines
border_positions <- Q_ordered |>
  group_by(Country) |>
  summarise(pos = max(as.numeric(ind)) - 1)


pop_labels_bars <- pop_labels |>
  mutate(pos = as.numeric(ind)  - .5)

# Function to filter and normalize data
normalize_data <- function(df, min_value) {
  df |>
    filter(value > min_value) |>
    group_by(ind) |>
    mutate(value = value / sum(value))
}

# Use the function
Q_grouped_filtered <- normalize_data(Q_grouped, 0.1)
# 
color_palette <-
  c(
    "v1" = "#FF00FF",
    "v2" = "#00FF00",
    "v3" = "#0000FF",
    "v4" = "#FFFF00",
    "v5" = "#FF0000"
  )

# Generate all potential variable names
all_variables <- paste0("v", 1:5)

# Map each variable to a name
color_mapping <- data.frame(variable = all_variables,
                            color = names(color_palette))

# Merge with Q_grouped_filtered
Q_grouped_filtered <- merge(Q_grouped_filtered, color_mapping, by = "variable")

# Create the plot
ggplot(Q_grouped_filtered, aes(x = as.factor(ind), y = value, fill = color)) +
  geom_bar(stat = 'identity', width = 1) +
  geom_vline(
    data = pop_labels_bars,
    aes(xintercept = pos),
    color = "#2C444A",
    linewidth = .2
  ) +
  geom_text(
    data = pop_labels,
    aes(x = as.numeric(ind), y = 1, label = Name),
    vjust = 1.5,
    hjust = 0,
    size = 2,
    angle = 90,
    inherit.aes = FALSE
  ) +
  my_theme() +
  theme(
    axis.text.x = element_text(
      angle = 90,
      hjust = 1,
      size = 12
    ),
    legend.position = "none",
    plot.margin = unit(c(3, 0.5, 0.5, 0.5), "cm")
  ) +
  xlab("Admixture matrix") +
  ylab("Ancestry proportions") +
  labs(caption = "Each bar represents the ancestry proportions for an individual for k=5.\n LEA inference for k1:25 for set10.") +
  scale_x_discrete(labels = label_func) +
  scale_fill_manual(values = color_palette) +
  expand_limits(y = c(0, 1.5))
```

```
ggsave(
  here("output", "variant_proportions", "figures", "set10.pdf"),
  width  = 12,
  height = 6,
  units  = "in",
  device = cairo_pdf
)
```

#### 3.3.2 Interploation over map

Clear memory and environment

```
# Clear entire environment
rm(list = ls())
# Forcefully trigger garbage collection
gc()
```

```
##           used  (Mb) gc trigger  (Mb) limit (Mb) max used  (Mb)
## Ncells 5643326 301.4   15241385 814.0         NA 15241385 814.0
## Vcells 9032494  69.0   41727004 318.4      32768 56276493 429.4
```

```
# Matrix
leak5 <- read_delim(
  here("output", "variant_proportions", "set10.snmf", "K5", "run1","set10_r1.5.Q"),
  delim = " ",
  col_names = FALSE,
  show_col_types = FALSE
) 

head(leak5)
```

```
## # A tibble: 6 × 5
##       X1     X2       X3       X4    X5
##    <dbl>  <dbl>    <dbl>    <dbl> <dbl>
## 1 0.116  0.173  0.0175   0.0708   0.623
## 2 0.0651 0.201  0.0382   0.171    0.524
## 3 0.157  0.146  0.000100 0.000100 0.697
## 4 0.0970 0.0885 0.0196   0.000100 0.795
## 5 0.116  0.0452 0.0236   0.000100 0.815
## 6 0.102  0.107  0.0148   0.0584   0.718
```

The fam file

```
fam_file <- here(
  "output", "populations", "snps_sets", "neutral.fam"
)

# Read the .fam file
fam_data <- read.table(fam_file, 
                       header = FALSE,
                       col.names = c("FamilyID", "IndividualID", "PaternalID", "MaternalID", "Sex", "Phenotype"))

# View the first few rows
head(fam_data)
```

```
##   FamilyID IndividualID PaternalID MaternalID Sex Phenotype
## 1      OKI         1001          0          0   2        -9
## 2      OKI         1002          0          0   2        -9
## 3      OKI         1003          0          0   2        -9
## 4      OKI         1004          0          0   2        -9
## 5      OKI         1005          0          0   2        -9
## 6      OKI         1006          0          0   1        -9
```

Create ID column

```
# Change column name
colnames(fam_data)[colnames(fam_data) == "IndividualID"] <- "ind"

# Change column name
colnames(fam_data)[colnames(fam_data) == "FamilyID"] <- "pop"

# Select ID
fam_data <- fam_data |>
  dplyr::select("ind", "pop")

# View the first few rows
head(fam_data)
```

```
##    ind pop
## 1 1001 OKI
## 2 1002 OKI
## 3 1003 OKI
## 4 1004 OKI
## 5 1005 OKI
## 6 1006 OKI
```

Add it to matrix

```
leak5 <- fam_data |>
  dplyr::select(ind, pop) |>
  bind_cols(leak5)

head(leak5)
```

```
##    ind pop        X1        X2          X3          X4       X5
## 1 1001 OKI 0.1163710 0.1725730 0.017452600 0.070828600 0.622774
## 2 1002 OKI 0.0651225 0.2014900 0.038158600 0.171094000 0.524135
## 3 1003 OKI 0.1570890 0.1460220 0.000099982 0.000099982 0.696689
## 4 1004 OKI 0.0969622 0.0884783 0.019564000 0.000099991 0.794896
## 5 1005 OKI 0.1163020 0.0452408 0.023569900 0.000099991 0.814787
## 6 1006 OKI 0.1021300 0.1066390 0.014846300 0.058365400 0.718019
```

Rename the columns

```
# Rename the columns starting from the third one
leak5 <- leak5 |>
  rename_with(~paste0("v", seq_along(.x)), .cols = -c(ind, pop))

# View the first few rows
head(leak5)
```

```
##    ind pop        v1        v2          v3          v4       v5
## 1 1001 OKI 0.1163710 0.1725730 0.017452600 0.070828600 0.622774
## 2 1002 OKI 0.0651225 0.2014900 0.038158600 0.171094000 0.524135
## 3 1003 OKI 0.1570890 0.1460220 0.000099982 0.000099982 0.696689
## 4 1004 OKI 0.0969622 0.0884783 0.019564000 0.000099991 0.794896
## 5 1005 OKI 0.1163020 0.0452408 0.023569900 0.000099991 0.814787
## 6 1006 OKI 0.1021300 0.1066390 0.014846300 0.058365400 0.718019
```

Import samples attributes

```
sampling_loc <- readRDS(here("output", "populations", "sampling_loc.rds"))
# head(sampling_loc)

pops <- sampling_loc |>
  filter(
    Region == "Asia"
  ) |>
  dplyr::select(
    Abbreviation, Latitude, Longitude, Pop_City, Country
  )

head(pops)
```

```
## # A tibble: 6 × 5
##   Abbreviation Latitude Longitude Pop_City   Country 
##   <chr>           <dbl>     <dbl> <chr>      <chr>   
## 1 GEL              26.9      90.5 Gelephu    Bhutan  
## 2 CAM              11.6     105.  Phnom Penh Cambodia
## 3 HAI              19.2     110.  Hainan     China   
## 4 YUN              24.5     101.  Yunnan     China   
## 5 HUN              27.6     112.  Hunan      China   
## 6 BEN              13.0      77.6 Bengaluru  India
```

Merge with pops

```
# Add an index column to Q_tibble
leak5$index <- seq_len(nrow(leak5))

# Perform the merge as before
df1 <-
  merge(
    leak5,
    pops,
    by.x = 2,
    by.y = 1,
    all.x = T,
    all.y = F
  ) |>
  na.omit()

# Order by the index column to ensure the order matches the original Q_tibble
df1 <- df1[order(df1$index),]

# Optionally, you can remove the index column if it's no longer needed
df1$index <- NULL

# Now the rows of df1 should be in the same order as the original Q_tibble
head(df1)
```

```
##     pop  ind        v1        v2          v3          v4       v5 Latitude
## 159 OKI 1001 0.1163710 0.1725730 0.017452600 0.070828600 0.622774  26.5013
## 160 OKI 1002 0.0651225 0.2014900 0.038158600 0.171094000 0.524135  26.5013
## 161 OKI 1003 0.1570890 0.1460220 0.000099982 0.000099982 0.696689  26.5013
## 162 OKI 1004 0.0969622 0.0884783 0.019564000 0.000099991 0.794896  26.5013
## 163 OKI 1005 0.1163020 0.0452408 0.023569900 0.000099991 0.814787  26.5013
## 164 OKI 1006 0.1021300 0.1066390 0.014846300 0.058365400 0.718019  26.5013
##     Longitude Pop_City Country
## 159  127.9454  Okinawa   Japan
## 160  127.9454  Okinawa   Japan
## 161  127.9454  Okinawa   Japan
## 162  127.9454  Okinawa   Japan
## 163  127.9454  Okinawa   Japan
## 164  127.9454  Okinawa   Japan
```

We used this color palette to make the “structure” plot

```
color_palette2 <-
  c(
    "v1" = "#FF00FF",
    "v2" = "#00FF00",
    "v3" = "#0000FF",
    "v4" = "#FFFF00",
    "v5" = "#FF0000"
  )
```

Preparing the data for tess3Q\_map\_rasters

Make sure the lat longs are in the correct order and arrangement

```
df2 <- df1 |>
  dplyr::rename(
    Long = Longitude,
    Lat = Latitude
  )

long_lat_tibble <- df2 |>
  dplyr::select(Long, Lat)


long_lat_matrix <- long_lat_tibble |>
  as.matrix()

head(long_lat_matrix)
```

```
##         Long     Lat
## 159 127.9454 26.5013
## 160 127.9454 26.5013
## 161 127.9454 26.5013
## 162 127.9454 26.5013
## 163 127.9454 26.5013
## 164 127.9454 26.5013
```

Make a matrix of the Q values

Pull off the names of individuals and make a matrix of it:

```
Q_matrix <- leak5 |>
  dplyr::select(-ind, -pop, -index) |>
  as.matrix()
head(Q_matrix)
```

```
##             v1        v2          v3          v4       v5
## [1,] 0.1163710 0.1725730 0.017452600 0.070828600 0.622774
## [2,] 0.0651225 0.2014900 0.038158600 0.171094000 0.524135
## [3,] 0.1570890 0.1460220 0.000099982 0.000099982 0.696689
## [4,] 0.0969622 0.0884783 0.019564000 0.000099991 0.794896
## [5,] 0.1163020 0.0452408 0.023569900 0.000099991 0.814787
## [6,] 0.1021300 0.1066390 0.014846300 0.058365400 0.718019
```

Interpolate the Q-values by Kriging

```
print(ncol(Q_matrix) == length(color_palette2))
```

```
## [1] TRUE
```

Create brick

```
world <- ne_countries(scale = "medium", returnclass = "sf")
countries_with_data <- unique(df1$Country)

# Filtering the world data to include only the countries in your data
selected_countries <- world |>
  filter(admin %in% countries_with_data)

genoscape_brick <- tess3r::tess3Q_map_rasters(
  x = Q_matrix, 
  coord = long_lat_matrix,  
  map.polygon = selected_countries,
  window = extent(selected_countries)[1:4],
  # window = combined_extent,
  resolution = c(600,600), # if you want more cells in your raster, set higher
  # this next lines need to to be here, but don't do much...
  col.palette = tess3r::CreatePalette(color_palette2, length(color_palette2)),
  method = "map.max", 
  interpol = tess3r::FieldsKrigModel(80),  
  main = "Ancestry coefficients",
  xlab = "Longitude", 
  ylab = "Latitude", 
  cex = .4
)
```

```
## Warning: 
## Grid searches over lambda (nugget and sill variances) with  minima at the endpoints: 
##   (REML) Restricted maximum likelihood 
##    minimum at  right endpoint  lambda  =  0.02118404 (eff. df= 26.59999 )
## Warning: 
## Grid searches over lambda (nugget and sill variances) with  minima at the endpoints: 
##   (REML) Restricted maximum likelihood 
##    minimum at  right endpoint  lambda  =  0.02118404 (eff. df= 26.59999 )
## Warning: 
## Grid searches over lambda (nugget and sill variances) with  minima at the endpoints: 
##   (REML) Restricted maximum likelihood 
##    minimum at  right endpoint  lambda  =  0.02118404 (eff. df= 26.59999 )
## Warning: 
## Grid searches over lambda (nugget and sill variances) with  minima at the endpoints: 
##   (REML) Restricted maximum likelihood 
##    minimum at  right endpoint  lambda  =  0.02118404 (eff. df= 26.59999 )
## Warning: 
## Grid searches over lambda (nugget and sill variances) with  minima at the endpoints: 
##   (REML) Restricted maximum likelihood 
##    minimum at  right endpoint  lambda  =  0.02118404 (eff. df= 26.59999 )
```

```
# after that, we need to add names of the clusters back onto this raster brick
Q_tibble2 <- leak5 |>
  dplyr::select(
    -pop, -ind, -index
  )
names(genoscape_brick) <- names(Q_tibble2)[]
```

Scaling and cleaning the genoscape\_brick

```
genoscape_rgba <- genoscapeRtools::qprob_rando_raster(
  TRB = genoscape_brick,
  cols = color_palette2,
  alpha_scale = 2.0,
  abs_thresh = 0.0,
  alpha_exp = 1.55,
  alpha_chop_max = 255
)

# This adds the info for a regular lat-long projection
crs(genoscape_rgba) <- "+proj=longlat +datum=WGS84 +no_defs +ellps=WGS84 +towgs84=0,0,0"
```

Plot with pies

```
source(
  here(
    "scripts", "analysis", "my_theme2.R"
  )
)

# Calculate mean proportions for each population
df_mean <- df1 |>
  group_by(pop) |>
  summarise(across(starts_with("v"), \(x) mean(x, na.rm = TRUE)), 
            Longitude = mean(Longitude),
            Latitude = mean(Latitude))

ggplot() +
  layer_spatial(genoscape_rgba) +
  geom_spatial_point(data = long_lat_tibble,
                     mapping = aes(x = Long, y = Lat),
                     size = .2) +
  geom_text_repel(
    data = df_mean,
    aes(x = Longitude, y = Latitude, label = pop),
    size = 3,
    box.padding = unit(0.5, "lines")
  ) +
  labs(x = "Longitude",
       y = "Latitude") +
  geom_scatterpie(data = df_mean, 
                  aes(x = Longitude, y = Latitude, r = 1), 
                  cols = c("v1", "v2", "v3", "v4", "v5"), color = NA) +
  my_theme() +
  scale_fill_manual(values = color_palette2) +
  guides(fill = "none") +  # Hide legend
  coord_sf()
```

```
## Assuming `crs = 4326` in stat_spatial_identity()
```

```
# # 
ggsave(
  here("output", "variant_proportions", "figures", "set10_map.pdf"),
  width  = 6,
  height = 6,
  units  = "in",
  device = cairo_pdf
)
```

```
## Assuming `crs = 4326` in stat_spatial_identity()
```

### 3.4 random2

Clean env and memory

```
rm(list=ls())
gc()
```

```
##            used  (Mb) gc trigger  (Mb) limit (Mb) max used  (Mb)
## Ncells  5643080 301.4   15241385 814.0         NA 15241385 814.0
## Vcells 10473061  80.0   40121924 306.2      32768 56276493 429.4
```

Import the data

```
genotype <- here(
    "output", "variant_proportions", "random2.vcf"
  )

d <- read.vcfR(
  genotype
)
```

```
## Scanning file to determine attributes.
## File attributes:
##   meta lines: 8
##   header_line: 9
##   variant count: 33400
##   column count: 246
## 
Meta line 8 read in.
## All meta lines processed.
## gt matrix initialized.
## Character matrix gt created.
##   Character matrix gt rows: 33400
##   Character matrix gt cols: 246
##   skip: 0
##   nrows: 33400
##   row_num: 0
## 
Processed variant 1000
Processed variant 2000
Processed variant 3000
Processed variant 4000
Processed variant 5000
Processed variant 6000
Processed variant 7000
Processed variant 8000
Processed variant 9000
Processed variant 10000
Processed variant 11000
Processed variant 12000
Processed variant 13000
Processed variant 14000
Processed variant 15000
Processed variant 16000
Processed variant 17000
Processed variant 18000
Processed variant 19000
Processed variant 20000
Processed variant 21000
Processed variant 22000
Processed variant 23000
Processed variant 24000
Processed variant 25000
Processed variant 26000
Processed variant 27000
Processed variant 28000
Processed variant 29000
Processed variant 30000
Processed variant 31000
Processed variant 32000
Processed variant 33000
Processed variant: 33400
## All variants processed
```

Population and individuals information

```
inds_full <- attr(d@gt,"dimnames")[[2]]
inds_full <- inds_full[-1]
a <- strsplit(inds_full, '_')
pops <- unname(sapply(a, FUN = function(x) return(as.character(x[1])))) 
table(pops)
```

```
## pops
## BEN CAM CHA GEL HAI HAN HOC HUN INJ INW JAF KAC KAG KAN KAT KLP KUN LAM MAT OKI 
##  12  12  12   2  12   4   7  12  11   4   2   6  12  11  10   4   4   9  12  11 
## QNC SON SSK SUF SUU TAI UTS YUN 
##  12   3  12   6   6   8  12   9
```

```
pops <- factor(pops)
inds <- unname(sapply(a, FUN = function(x) return(as.character(x[2]))))
```

Convert format

```
vcf2geno(genotype, gsub(".vcf", ".geno", genotype))
```

```
## 
##  - number of detected individuals:   237
##  - number of detected loci:      33400
## 
## For SNP info, please check /Users/lucianocosme/Library/CloudStorage/Dropbox/Albopictus/manuscript_chip/data/no_autogenous/albo_chip/output/variant_proportions/random2.vcfsnp.
## 
## 0 line(s) were removed because these are not SNPs.
## Please, check /Users/lucianocosme/Library/CloudStorage/Dropbox/Albopictus/manuscript_chip/data/no_autogenous/albo_chip/output/variant_proportions/random2.removed file, for more informations.
```

```
## [1] "/Users/lucianocosme/Library/CloudStorage/Dropbox/Albopictus/manuscript_chip/data/no_autogenous/albo_chip/output/variant_proportions/random2.geno"
```

```
vcf2lfmm(genotype, gsub(".vcf", ".lfmm", genotype))
```

```
## 
##  - number of detected individuals:   237
##  - number of detected loci:      33400
## 
## For SNP info, please check /Users/lucianocosme/Library/CloudStorage/Dropbox/Albopictus/manuscript_chip/data/no_autogenous/albo_chip/output/variant_proportions/random2.vcfsnp.
## 
## 0 line(s) were removed because these are not SNPs.
## Please, check /Users/lucianocosme/Library/CloudStorage/Dropbox/Albopictus/manuscript_chip/data/no_autogenous/albo_chip/output/variant_proportions/random2.removed file, for more informations.
## 
## 
##  - number of detected individuals:   237
##  - number of detected loci:      33400
```

```
## [1] "/Users/lucianocosme/Library/CloudStorage/Dropbox/Albopictus/manuscript_chip/data/no_autogenous/albo_chip/output/variant_proportions/random2.lfmm"
```

Run LEA

```
# set output dir
setwd(
  here(
    "output", "variant_proportions"
  )
)
# main options
# K = number of ancestral populations
# entropy = TRUE computes the cross-entropy criterion, # CPU = 4 is the number of CPU used (hidden input) project = NULL
project = snmf(
  genotype,
  K = 1:25,
  project = "new",
  repetitions = 1,
  alpha = 5,
  tolerance = 0.0001,
  percentage = 0.5,
  iterations = 200, 
  ploidy = 2,
  seed = 13,
  CPU = 4,
  entropy = TRUE
)
```

```
project = load.snmfProject("output/variant_proportions/random2.snmfProject")
```

Cross entropy

```
# Open a new pdf file
pdf(here("output","variant_proportions","figures","cross_entropy_random2.pdf"), width = 6, height = 4)

# Create your plot
plot(project, col = "pink", pch = 19, cex = 1.2)

# Close the pdf file
dev.off()
```

```
## quartz_off_screen 
##                 2
```

```
plot(project, col = "pink", pch = 19, cex = 1.2)
```

#### 3.4.1 Structure plot

```
# Matrix
leak5 <- read_delim(
  here("output", "variant_proportions", "random2.snmf", "K5", "run1","random2_r1.5.Q"),
  delim = " ",
  col_names = FALSE,
  show_col_types = FALSE
) 


head(leak5)
```

```
## # A tibble: 6 × 5
##         X1       X2    X3       X4       X5
##      <dbl>    <dbl> <dbl>    <dbl>    <dbl>
## 1 0.144    0.105    0.712 0.0392   0.000100
## 2 0.125    0.223    0.534 0.104    0.0141  
## 3 0.000100 0.0697   0.872 0.0528   0.00572 
## 4 0.000100 0.000100 1.00  0.000100 0.000100
## 5 0.000100 0.000100 1.00  0.000100 0.000100
## 6 0.0323   0.0531   0.880 0.0341   0.000100
```

The fam file

```
fam_file <- here(
  "output", "populations", "snps_sets", "neutral.fam"
)

# Read the .fam file
fam_data <- read.table(fam_file, 
                       header = FALSE,
                       col.names = c("FamilyID", "IndividualID", "PaternalID", "MaternalID", "Sex", "Phenotype"))

# View the first few rows
head(fam_data)
```

```
##   FamilyID IndividualID PaternalID MaternalID Sex Phenotype
## 1      OKI         1001          0          0   2        -9
## 2      OKI         1002          0          0   2        -9
## 3      OKI         1003          0          0   2        -9
## 4      OKI         1004          0          0   2        -9
## 5      OKI         1005          0          0   2        -9
## 6      OKI         1006          0          0   1        -9
```

Create ID column

```
# Change column name
colnames(fam_data)[colnames(fam_data) == "IndividualID"] <- "ind"

# Change column name
colnames(fam_data)[colnames(fam_data) == "FamilyID"] <- "pop"

# Select ID
fam_data <- fam_data |>
  dplyr::select("ind", "pop")

# View the first few rows
head(fam_data)
```

```
##    ind pop
## 1 1001 OKI
## 2 1002 OKI
## 3 1003 OKI
## 4 1004 OKI
## 5 1005 OKI
## 6 1006 OKI
```

Add it to matrix

```
leak5 <- fam_data |>
  dplyr::select(ind, pop) |>
  bind_cols(leak5)

head(leak5)
```

```
##    ind pop         X1         X2       X3         X4          X5
## 1 1001 OKI 0.14368500 0.10525200 0.711718 0.03924570 0.000099991
## 2 1002 OKI 0.12513600 0.22286800 0.533557 0.10432000 0.014118800
## 3 1003 OKI 0.00009999 0.06966320 0.871695 0.05282660 0.005715380
## 4 1004 OKI 0.00009997 0.00009997 0.999600 0.00009997 0.000099970
## 5 1005 OKI 0.00009997 0.00009997 0.999600 0.00009997 0.000099970
## 6 1006 OKI 0.03229830 0.05308260 0.880423 0.03409590 0.000099991
```

Rename the columns

```
# Rename the columns starting from the third one
leak5 <- leak5 |>
  rename_with(~paste0("v", seq_along(.x)), .cols = -c(ind, pop))

# View the first few rows
head(leak5)
```

```
##    ind pop         v1         v2       v3         v4          v5
## 1 1001 OKI 0.14368500 0.10525200 0.711718 0.03924570 0.000099991
## 2 1002 OKI 0.12513600 0.22286800 0.533557 0.10432000 0.014118800
## 3 1003 OKI 0.00009999 0.06966320 0.871695 0.05282660 0.005715380
## 4 1004 OKI 0.00009997 0.00009997 0.999600 0.00009997 0.000099970
## 5 1005 OKI 0.00009997 0.00009997 0.999600 0.00009997 0.000099970
## 6 1006 OKI 0.03229830 0.05308260 0.880423 0.03409590 0.000099991
```

Import sample locations

```
sampling_loc <- readRDS(here("output", "populations", "sampling_loc.rds"))
head(sampling_loc)
```

```
## # A tibble: 6 × 6
##   Pop_City   Country  Latitude Longitude Region Abbreviation
##   <chr>      <chr>       <dbl>     <dbl> <chr>  <chr>       
## 1 Gelephu    Bhutan       26.9      90.5 Asia   GEL         
## 2 Phnom Penh Cambodia     11.6     105.  Asia   CAM         
## 3 Hainan     China        19.2     110.  Asia   HAI         
## 4 Yunnan     China        24.5     101.  Asia   YUN         
## 5 Hunan      China        27.6     112.  Asia   HUN         
## 6 Bengaluru  India        13.0      77.6 Asia   BEN
```

Structure plot

```
source(
  here(
    "scripts", "analysis", "my_theme3.R"
  )
)

# Create a named vector to map countries to regions
country_to_region <- c(
  "Bhutan" = "South Asia",
  "Cambodia" = "Southeast Asia",
  "China" = "East Asia",
  "India" = "South Asia",
  "Indonesia" = "Southeast Asia",
  "Japan" = "East Asia",
  "Malaysia" = "Southeast Asia",
  "Maldives" = "South Asia",
  "Nepal" = "South Asia",
  "Sri Lanka" = "South Asia",
  "Taiwan" = "East Asia",
  "Thailand" = "Southeast Asia",
  "Vietnam" = "Southeast Asia"
)

# Add the region to the data frame
sampling_loc$Region2 <- country_to_region[sampling_loc$Country]

# Melt the data frame for plotting
Q_melted <- leak5 |>
  pivot_longer(
    cols = -c(ind, pop),
    names_to = "variable",
    values_to = "value"
  )
# Join with sampling_loc to get sampling localities
Q_joined <- Q_melted |>
  left_join(sampling_loc, by = c("pop" = "Abbreviation"))

# Create a combined variable for Region and Country
Q_joined <- Q_joined |>
  mutate(Region_Country = interaction(Region, Country, sep = "_"))

# Order the combined variable by Region and Country, then by individual
Q_ordered <- Q_joined |>
  arrange(Region, Region2, Country, ind) |>
  mutate(ind = factor(ind, levels = unique(ind)))  # Convert ind to a factor with levels in the desired order

# Add labels: country names for the first individual in each country, NA for all other individuals
Q_ordered <- Q_ordered |>
  group_by(Region_Country) |>
  mutate(label = ifelse(row_number() == 1, as.character(Country), NA))

# Group by individual and variable, calculate mean ancestry proportions
Q_grouped <- Q_ordered |>
  group_by(ind, variable) |>
  summarise(value = mean(value), .groups = "drop")

# Create a data frame for borders
borders <-
  data.frame(Region_Country = unique(Q_ordered$Region_Country))

# Add the order of the last individual of each country to ensure correct placement of borders
borders$order <-
  sapply(borders$Region_Country, function(rc)
    max(which(Q_ordered$Region_Country == rc))) + 0.5  # Shift borders to the right edge of the bars

# Select only the first occurrence of each country in the ordered data
label_df <- Q_ordered |>
  filter(!is.na(label)) |>
  distinct(label, .keep_all = TRUE)

# Create a custom label function
label_func <- function(x) {
  labels <- rep("", length(x))
  labels[x %in% label_df$ind] <- label_df$label
  labels
}

# Calculate the position of lines
border_positions <- Q_ordered |>
  group_by(Country) |>
  summarise(pos = max(as.numeric(ind)) + 0)

# Calculate the position of population labels and bars
pop_labels <- Q_ordered |>
  mutate(Name = paste(pop, Pop_City, sep = " - ")) |>
  group_by(pop) |>
  slice_head(n = 1) |>
  ungroup() |>
  dplyr::select(ind, Pop_City, Country, Name) |>
  mutate(pos = as.numeric(ind))  # calculate position of population labels

pop_labels_bars <- pop_labels |>
  mutate(pos = as.numeric(ind)  - .5)


# Calculate the position of lines
border_positions <- Q_ordered |>
  group_by(Country) |>
  summarise(pos = max(as.numeric(ind)) - 1)


pop_labels_bars <- pop_labels |>
  mutate(pos = as.numeric(ind)  - .5)

# Function to filter and normalize data
normalize_data <- function(df, min_value) {
  df |>
    filter(value > min_value) |>
    group_by(ind) |>
    mutate(value = value / sum(value))
}

# Use the function
Q_grouped_filtered <- normalize_data(Q_grouped, 0.1)
# 
color_palette <-
  c(
    "v1" = "#00FF00",
    "v2" = "#FF00FF",
    "v3" = "#FF0000",
    "v4" = "#FFFF00",
    "v5" = "#0000FF"
  )

# Generate all potential variable names
all_variables <- paste0("v", 1:5)

# Map each variable to a name
color_mapping <- data.frame(variable = all_variables,
                            color = names(color_palette))

# Merge with Q_grouped_filtered
Q_grouped_filtered <- merge(Q_grouped_filtered, color_mapping, by = "variable")

# Create the plot
ggplot(Q_grouped_filtered, aes(x = as.factor(ind), y = value, fill = color)) +
  geom_bar(stat = 'identity', width = 1) +
  geom_vline(
    data = pop_labels_bars,
    aes(xintercept = pos),
    color = "#2C444A",
    linewidth = .2
  ) +
  geom_text(
    data = pop_labels,
    aes(x = as.numeric(ind), y = 1, label = Name),
    vjust = 1.5,
    hjust = 0,
    size = 2,
    angle = 90,
    inherit.aes = FALSE
  ) +
  my_theme() +
  theme(
    axis.text.x = element_text(
      angle = 90,
      hjust = 1,
      size = 12
    ),
    legend.position = "none",
    plot.margin = unit(c(3, 0.5, 0.5, 0.5), "cm")
  ) +
  xlab("Admixture matrix") +
  ylab("Ancestry proportions") +
  labs(caption = "Each bar represents the ancestry proportions for an individual for k=5.\n LEA inference for k1:25 for random2.") +
  scale_x_discrete(labels = label_func) +
  scale_fill_manual(values = color_palette) +
  expand_limits(y = c(0, 1.5))
```

```
ggsave(
  here("output", "variant_proportions", "figures", "random2.pdf"),
  width  = 12,
  height = 6,
  units  = "in",
  device = cairo_pdf
)
```

#### 3.4.2 Interploation over map

Clear memory and environment

```
# Clear entire environment
rm(list = ls())
# Forcefully trigger garbage collection
gc()
```

```
##           used  (Mb) gc trigger  (Mb) limit (Mb) max used  (Mb)
## Ncells 5643822 301.5   15241385 814.0         NA 15241385 814.0
## Vcells 9043969  69.1   41306715 315.2      32768 56276493 429.4
```

```
# Matrix
leak5 <- read_delim(
  here("output", "variant_proportions", "random2.snmf", "K5", "run1","random2_r1.5.Q"),
  delim = " ",
  col_names = FALSE,
  show_col_types = FALSE
) 

head(leak5)
```

```
## # A tibble: 6 × 5
##         X1       X2    X3       X4       X5
##      <dbl>    <dbl> <dbl>    <dbl>    <dbl>
## 1 0.144    0.105    0.712 0.0392   0.000100
## 2 0.125    0.223    0.534 0.104    0.0141  
## 3 0.000100 0.0697   0.872 0.0528   0.00572 
## 4 0.000100 0.000100 1.00  0.000100 0.000100
## 5 0.000100 0.000100 1.00  0.000100 0.000100
## 6 0.0323   0.0531   0.880 0.0341   0.000100
```

The fam file

```
fam_file <- here(
  "output", "populations", "snps_sets", "neutral.fam"
)

# Read the .fam file
fam_data <- read.table(fam_file, 
                       header = FALSE,
                       col.names = c("FamilyID", "IndividualID", "PaternalID", "MaternalID", "Sex", "Phenotype"))

# View the first few rows
head(fam_data)
```

```
##   FamilyID IndividualID PaternalID MaternalID Sex Phenotype
## 1      OKI         1001          0          0   2        -9
## 2      OKI         1002          0          0   2        -9
## 3      OKI         1003          0          0   2        -9
## 4      OKI         1004          0          0   2        -9
## 5      OKI         1005          0          0   2        -9
## 6      OKI         1006          0          0   1        -9
```

Create ID column

```
# Change column name
colnames(fam_data)[colnames(fam_data) == "IndividualID"] <- "ind"

# Change column name
colnames(fam_data)[colnames(fam_data) == "FamilyID"] <- "pop"

# Select ID
fam_data <- fam_data |>
  dplyr::select("ind", "pop")

# View the first few rows
head(fam_data)
```

```
##    ind pop
## 1 1001 OKI
## 2 1002 OKI
## 3 1003 OKI
## 4 1004 OKI
## 5 1005 OKI
## 6 1006 OKI
```

Add it to matrix

```
leak5 <- fam_data |>
  dplyr::select(ind, pop) |>
  bind_cols(leak5)

head(leak5)
```

```
##    ind pop         X1         X2       X3         X4          X5
## 1 1001 OKI 0.14368500 0.10525200 0.711718 0.03924570 0.000099991
## 2 1002 OKI 0.12513600 0.22286800 0.533557 0.10432000 0.014118800
## 3 1003 OKI 0.00009999 0.06966320 0.871695 0.05282660 0.005715380
## 4 1004 OKI 0.00009997 0.00009997 0.999600 0.00009997 0.000099970
## 5 1005 OKI 0.00009997 0.00009997 0.999600 0.00009997 0.000099970
## 6 1006 OKI 0.03229830 0.05308260 0.880423 0.03409590 0.000099991
```

Rename the columns

```
# Rename the columns starting from the third one
leak5 <- leak5 |>
  rename_with(~paste0("v", seq_along(.x)), .cols = -c(ind, pop))

# View the first few rows
head(leak5)
```

```
##    ind pop         v1         v2       v3         v4          v5
## 1 1001 OKI 0.14368500 0.10525200 0.711718 0.03924570 0.000099991
## 2 1002 OKI 0.12513600 0.22286800 0.533557 0.10432000 0.014118800
## 3 1003 OKI 0.00009999 0.06966320 0.871695 0.05282660 0.005715380
## 4 1004 OKI 0.00009997 0.00009997 0.999600 0.00009997 0.000099970
## 5 1005 OKI 0.00009997 0.00009997 0.999600 0.00009997 0.000099970
## 6 1006 OKI 0.03229830 0.05308260 0.880423 0.03409590 0.000099991
```

Import samples attributes

```
sampling_loc <- readRDS(here("output", "populations", "sampling_loc.rds"))
# head(sampling_loc)

pops <- sampling_loc |>
  filter(
    Region == "Asia"
  ) |>
  dplyr::select(
    Abbreviation, Latitude, Longitude, Pop_City, Country
  )

head(pops)
```

```
## # A tibble: 6 × 5
##   Abbreviation Latitude Longitude Pop_City   Country 
##   <chr>           <dbl>     <dbl> <chr>      <chr>   
## 1 GEL              26.9      90.5 Gelephu    Bhutan  
## 2 CAM              11.6     105.  Phnom Penh Cambodia
## 3 HAI              19.2     110.  Hainan     China   
## 4 YUN              24.5     101.  Yunnan     China   
## 5 HUN              27.6     112.  Hunan      China   
## 6 BEN              13.0      77.6 Bengaluru  India
```

Merge with pops

```
# Add an index column to Q_tibble
leak5$index <- seq_len(nrow(leak5))

# Perform the merge as before
df1 <-
  merge(
    leak5,
    pops,
    by.x = 2,
    by.y = 1,
    all.x = T,
    all.y = F
  ) |>
  na.omit()

# Order by the index column to ensure the order matches the original Q_tibble
df1 <- df1[order(df1$index),]

# Optionally, you can remove the index column if it's no longer needed
df1$index <- NULL

# Now the rows of df1 should be in the same order as the original Q_tibble
head(df1)
```

```
##     pop  ind         v1         v2       v3         v4          v5 Latitude
## 159 OKI 1001 0.14368500 0.10525200 0.711718 0.03924570 0.000099991  26.5013
## 160 OKI 1002 0.12513600 0.22286800 0.533557 0.10432000 0.014118800  26.5013
## 161 OKI 1003 0.00009999 0.06966320 0.871695 0.05282660 0.005715380  26.5013
## 162 OKI 1004 0.00009997 0.00009997 0.999600 0.00009997 0.000099970  26.5013
## 163 OKI 1005 0.00009997 0.00009997 0.999600 0.00009997 0.000099970  26.5013
## 164 OKI 1006 0.03229830 0.05308260 0.880423 0.03409590 0.000099991  26.5013
##     Longitude Pop_City Country
## 159  127.9454  Okinawa   Japan
## 160  127.9454  Okinawa   Japan
## 161  127.9454  Okinawa   Japan
## 162  127.9454  Okinawa   Japan
## 163  127.9454  Okinawa   Japan
## 164  127.9454  Okinawa   Japan
```

We used this color palette to make the “structure” plot

```
color_palette2 <-
  c(
    "v1" = "#00FF00",
    "v2" = "#FF00FF",
    "v3" = "#FF0000",
    "v4" = "#FFFF00",
    "v5" = "#0000FF"
  )
```

Preparing the data for tess3Q\_map\_rasters

Make sure the lat longs are in the correct order and arrangement

```
df2 <- df1 |>
  dplyr::rename(
    Long = Longitude,
    Lat = Latitude
  )

long_lat_tibble <- df2 |>
  dplyr::select(Long, Lat)


long_lat_matrix <- long_lat_tibble |>
  as.matrix()

head(long_lat_matrix)
```

```
##         Long     Lat
## 159 127.9454 26.5013
## 160 127.9454 26.5013
## 161 127.9454 26.5013
## 162 127.9454 26.5013
## 163 127.9454 26.5013
## 164 127.9454 26.5013
```

Make a matrix of the Q values

Pull off the names of individuals and make a matrix of it:

```
Q_matrix <- leak5 |>
  dplyr::select(-ind, -pop, -index) |>
  as.matrix()
head(Q_matrix)
```

```
##              v1         v2       v3         v4          v5
## [1,] 0.14368500 0.10525200 0.711718 0.03924570 0.000099991
## [2,] 0.12513600 0.22286800 0.533557 0.10432000 0.014118800
## [3,] 0.00009999 0.06966320 0.871695 0.05282660 0.005715380
## [4,] 0.00009997 0.00009997 0.999600 0.00009997 0.000099970
## [5,] 0.00009997 0.00009997 0.999600 0.00009997 0.000099970
## [6,] 0.03229830 0.05308260 0.880423 0.03409590 0.000099991
```

Interpolate the Q-values by Kriging

```
print(ncol(Q_matrix) == length(color_palette2))
```

```
## [1] TRUE
```

Create brick

```
world <- ne_countries(scale = "medium", returnclass = "sf")
countries_with_data <- unique(df1$Country)

# Filtering the world data to include only the countries in your data
selected_countries <- world |>
  filter(admin %in% countries_with_data)

genoscape_brick <- tess3r::tess3Q_map_rasters(
  x = Q_matrix, 
  coord = long_lat_matrix,  
  map.polygon = selected_countries,
  window = extent(selected_countries)[1:4],
  # window = combined_extent,
  resolution = c(600,600), # if you want more cells in your raster, set higher
  # this next lines need to to be here, but don't do much...
  col.palette = tess3r::CreatePalette(color_palette2, length(color_palette2)),
  method = "map.max", 
  interpol = tess3r::FieldsKrigModel(80),  
  main = "Ancestry coefficients",
  xlab = "Longitude", 
  ylab = "Latitude", 
  cex = .4
)
```

```
## Warning: 
## Grid searches over lambda (nugget and sill variances) with  minima at the endpoints: 
##   (REML) Restricted maximum likelihood 
##    minimum at  right endpoint  lambda  =  0.02118404 (eff. df= 26.59999 )
## Warning: 
## Grid searches over lambda (nugget and sill variances) with  minima at the endpoints: 
##   (REML) Restricted maximum likelihood 
##    minimum at  right endpoint  lambda  =  0.02118404 (eff. df= 26.59999 )
## Warning: 
## Grid searches over lambda (nugget and sill variances) with  minima at the endpoints: 
##   (REML) Restricted maximum likelihood 
##    minimum at  right endpoint  lambda  =  0.02118404 (eff. df= 26.59999 )
## Warning: 
## Grid searches over lambda (nugget and sill variances) with  minima at the endpoints: 
##   (REML) Restricted maximum likelihood 
##    minimum at  right endpoint  lambda  =  0.02118404 (eff. df= 26.59999 )
```

```
# after that, we need to add names of the clusters back onto this raster brick
Q_tibble2 <- leak5 |>
  dplyr::select(
    -pop, -ind, -index
  )
names(genoscape_brick) <- names(Q_tibble2)[]
```

Scaling and cleaning the genoscape\_brick

```
genoscape_rgba <- genoscapeRtools::qprob_rando_raster(
  TRB = genoscape_brick,
  cols = color_palette2,
  alpha_scale = 2.0,
  abs_thresh = 0.0,
  alpha_exp = 1.55,
  alpha_chop_max = 255
)

# This adds the info for a regular lat-long projection
crs(genoscape_rgba) <- "+proj=longlat +datum=WGS84 +no_defs +ellps=WGS84 +towgs84=0,0,0"
```

Plot with pies

```
source(
  here(
    "scripts", "analysis", "my_theme2.R"
  )
)

# Calculate mean proportions for each population
df_mean <- df1 |>
  group_by(pop) |>
  summarise(across(starts_with("v"), \(x) mean(x, na.rm = TRUE)), 
            Longitude = mean(Longitude),
            Latitude = mean(Latitude))

ggplot() +
  layer_spatial(genoscape_rgba) +
  geom_spatial_point(data = long_lat_tibble,
                     mapping = aes(x = Long, y = Lat),
                     size = .2) +
  geom_text_repel(
    data = df_mean,
    aes(x = Longitude, y = Latitude, label = pop),
    size = 3,
    box.padding = unit(0.5, "lines")
  ) +
  labs(x = "Longitude",
       y = "Latitude") +
  geom_scatterpie(data = df_mean, 
                  aes(x = Longitude, y = Latitude, r = 1), 
                  cols = c("v1", "v2", "v3", "v4", "v5"), color = NA) +
  my_theme() +
  scale_fill_manual(values = color_palette2) +
  guides(fill = "none") +  # Hide legend
  coord_sf()
```

```
## Assuming `crs = 4326` in stat_spatial_identity()
```

```
# # 
ggsave(
  here("output", "variant_proportions", "figures", "random2_map.pdf"),
  width  = 6,
  height = 6,
  units  = "in",
  device = cairo_pdf
)
```

```
## Assuming `crs = 4326` in stat_spatial_identity()
```

### 3.5 random8

Clean env and memory

```
rm(list=ls())
gc()
```

```
##            used  (Mb) gc trigger  (Mb) limit (Mb) max used  (Mb)
## Ncells  5643571 301.4   15241385 814.0         NA 15241385 814.0
## Vcells 10485037  80.0   39743414 303.3      32768 56276493 429.4
```

Import the data

```
genotype <- here(
    "output", "variant_proportions", "random8.vcf"
  )

d <- read.vcfR(
  genotype
)
```

```
## Scanning file to determine attributes.
## File attributes:
##   meta lines: 8
##   header_line: 9
##   variant count: 33400
##   column count: 246
## 
Meta line 8 read in.
## All meta lines processed.
## gt matrix initialized.
## Character matrix gt created.
##   Character matrix gt rows: 33400
##   Character matrix gt cols: 246
##   skip: 0
##   nrows: 33400
##   row_num: 0
## 
Processed variant 1000
Processed variant 2000
Processed variant 3000
Processed variant 4000
Processed variant 5000
Processed variant 6000
Processed variant 7000
Processed variant 8000
Processed variant 9000
Processed variant 10000
Processed variant 11000
Processed variant 12000
Processed variant 13000
Processed variant 14000
Processed variant 15000
Processed variant 16000
Processed variant 17000
Processed variant 18000
Processed variant 19000
Processed variant 20000
Processed variant 21000
Processed variant 22000
Processed variant 23000
Processed variant 24000
Processed variant 25000
Processed variant 26000
Processed variant 27000
Processed variant 28000
Processed variant 29000
Processed variant 30000
Processed variant 31000
Processed variant 32000
Processed variant 33000
Processed variant: 33400
## All variants processed
```

Population and individuals information

```
inds_full <- attr(d@gt,"dimnames")[[2]]
inds_full <- inds_full[-1]
a <- strsplit(inds_full, '_')
pops <- unname(sapply(a, FUN = function(x) return(as.character(x[1])))) 
table(pops)
```

```
## pops
## BEN CAM CHA GEL HAI HAN HOC HUN INJ INW JAF KAC KAG KAN KAT KLP KUN LAM MAT OKI 
##  12  12  12   2  12   4   7  12  11   4   2   6  12  11  10   4   4   9  12  11 
## QNC SON SSK SUF SUU TAI UTS YUN 
##  12   3  12   6   6   8  12   9
```

```
pops <- factor(pops)
inds <- unname(sapply(a, FUN = function(x) return(as.character(x[2]))))
```

Convert format

```
vcf2geno(genotype, gsub(".vcf", ".geno", genotype))
```

```
## 
##  - number of detected individuals:   237
##  - number of detected loci:      33400
## 
## For SNP info, please check /Users/lucianocosme/Library/CloudStorage/Dropbox/Albopictus/manuscript_chip/data/no_autogenous/albo_chip/output/variant_proportions/random8.vcfsnp.
## 
## 0 line(s) were removed because these are not SNPs.
## Please, check /Users/lucianocosme/Library/CloudStorage/Dropbox/Albopictus/manuscript_chip/data/no_autogenous/albo_chip/output/variant_proportions/random8.removed file, for more informations.
```

```
## [1] "/Users/lucianocosme/Library/CloudStorage/Dropbox/Albopictus/manuscript_chip/data/no_autogenous/albo_chip/output/variant_proportions/random8.geno"
```

```
vcf2lfmm(genotype, gsub(".vcf", ".lfmm", genotype))
```

```
## 
##  - number of detected individuals:   237
##  - number of detected loci:      33400
## 
## For SNP info, please check /Users/lucianocosme/Library/CloudStorage/Dropbox/Albopictus/manuscript_chip/data/no_autogenous/albo_chip/output/variant_proportions/random8.vcfsnp.
## 
## 0 line(s) were removed because these are not SNPs.
## Please, check /Users/lucianocosme/Library/CloudStorage/Dropbox/Albopictus/manuscript_chip/data/no_autogenous/albo_chip/output/variant_proportions/random8.removed file, for more informations.
## 
## 
##  - number of detected individuals:   237
##  - number of detected loci:      33400
```

```
## [1] "/Users/lucianocosme/Library/CloudStorage/Dropbox/Albopictus/manuscript_chip/data/no_autogenous/albo_chip/output/variant_proportions/random8.lfmm"
```

Run LEA

```
# set output dir
setwd(
  here(
    "output", "variant_proportions"
  )
)
# main options
# K = number of ancestral populations
# entropy = TRUE computes the cross-entropy criterion, # CPU = 4 is the number of CPU used (hidden input) project = NULL
project = snmf(
  genotype,
  K = 1:25,
  project = "new",
  repetitions = 1,
  alpha = 5,
  tolerance = 0.0001,
  percentage = 0.5,
  iterations = 200, 
  ploidy = 2,
  seed = 13,
  CPU = 4,
  entropy = TRUE
)
```

```
project = load.snmfProject("output/variant_proportions/random8.snmfProject")
```

Cross entropy

```
# Open a new pdf file
pdf(here("output","variant_proportions","figures","cross_entropy_random8.pdf"), width = 6, height = 4)

# Create your plot
plot(project, col = "pink", pch = 19, cex = 1.2)

# Close the pdf file
dev.off()
```

```
## quartz_off_screen 
##                 2
```

```
plot(project, col = "pink", pch = 19, cex = 1.2)
```

#### 3.5.1 Structure plot

```
# Matrix
leak5 <- read_delim(
  here("output", "variant_proportions", "random8.snmf", "K5", "run1","random8_r1.5.Q"),
  delim = " ",
  col_names = FALSE,
  show_col_types = FALSE
) 


head(leak5)
```

```
## # A tibble: 6 × 5
##         X1       X2       X3    X4       X5
##      <dbl>    <dbl>    <dbl> <dbl>    <dbl>
## 1 0.0223   0.0799   0.0233   0.814 0.0608  
## 2 0.147    0.162    0.0286   0.574 0.0877  
## 3 0.000100 0.0576   0.000100 0.942 0.000100
## 4 0.000100 0.000100 0.000100 1.00  0.000100
## 5 0.000100 0.000100 0.000100 1.00  0.000100
## 6 0.00358  0.0995   0.0122   0.885 0.000100
```

The fam file

```
fam_file <- here(
  "output", "populations", "snps_sets", "neutral.fam"
)

# Read the .fam file
fam_data <- read.table(fam_file, 
                       header = FALSE,
                       col.names = c("FamilyID", "IndividualID", "PaternalID", "MaternalID", "Sex", "Phenotype"))

# View the first few rows
head(fam_data)
```

```
##   FamilyID IndividualID PaternalID MaternalID Sex Phenotype
## 1      OKI         1001          0          0   2        -9
## 2      OKI         1002          0          0   2        -9
## 3      OKI         1003          0          0   2        -9
## 4      OKI         1004          0          0   2        -9
## 5      OKI         1005          0          0   2        -9
## 6      OKI         1006          0          0   1        -9
```

Create ID column

```
# Change column name
colnames(fam_data)[colnames(fam_data) == "IndividualID"] <- "ind"

# Change column name
colnames(fam_data)[colnames(fam_data) == "FamilyID"] <- "pop"

# Select ID
fam_data <- fam_data |>
  dplyr::select("ind", "pop")

# View the first few rows
head(fam_data)
```

```
##    ind pop
## 1 1001 OKI
## 2 1002 OKI
## 3 1003 OKI
## 4 1004 OKI
## 5 1005 OKI
## 6 1006 OKI
```

Add it to matrix

```
leak5 <- fam_data |>
  dplyr::select(ind, pop) |>
  bind_cols(leak5)

head(leak5)
```

```
##    ind pop          X1         X2          X3       X4          X5
## 1 1001 OKI 0.022296300 0.07994870 0.023336900 0.813667 0.060750600
## 2 1002 OKI 0.147284000 0.16191200 0.028616800 0.574449 0.087738100
## 3 1003 OKI 0.000099973 0.05759880 0.000099973 0.942101 0.000099973
## 4 1004 OKI 0.000099970 0.00009997 0.000099970 0.999600 0.000099970
## 5 1005 OKI 0.000099970 0.00009997 0.000099970 0.999600 0.000099970
## 6 1006 OKI 0.003580470 0.09951710 0.012223200 0.884579 0.000099991
```

Rename the columns

```
# Rename the columns starting from the third one
leak5 <- leak5 |>
  rename_with(~paste0("v", seq_along(.x)), .cols = -c(ind, pop))

# View the first few rows
head(leak5)
```

```
##    ind pop          v1         v2          v3       v4          v5
## 1 1001 OKI 0.022296300 0.07994870 0.023336900 0.813667 0.060750600
## 2 1002 OKI 0.147284000 0.16191200 0.028616800 0.574449 0.087738100
## 3 1003 OKI 0.000099973 0.05759880 0.000099973 0.942101 0.000099973
## 4 1004 OKI 0.000099970 0.00009997 0.000099970 0.999600 0.000099970
## 5 1005 OKI 0.000099970 0.00009997 0.000099970 0.999600 0.000099970
## 6 1006 OKI 0.003580470 0.09951710 0.012223200 0.884579 0.000099991
```

Import sample locations

```
sampling_loc <- readRDS(here("output", "populations", "sampling_loc.rds"))
head(sampling_loc)
```

```
## # A tibble: 6 × 6
##   Pop_City   Country  Latitude Longitude Region Abbreviation
##   <chr>      <chr>       <dbl>     <dbl> <chr>  <chr>       
## 1 Gelephu    Bhutan       26.9      90.5 Asia   GEL         
## 2 Phnom Penh Cambodia     11.6     105.  Asia   CAM         
## 3 Hainan     China        19.2     110.  Asia   HAI         
## 4 Yunnan     China        24.5     101.  Asia   YUN         
## 5 Hunan      China        27.6     112.  Asia   HUN         
## 6 Bengaluru  India        13.0      77.6 Asia   BEN
```

Structure plot

```
source(
  here(
    "scripts", "analysis", "my_theme3.R"
  )
)

# Create a named vector to map countries to regions
country_to_region <- c(
  "Bhutan" = "South Asia",
  "Cambodia" = "Southeast Asia",
  "China" = "East Asia",
  "India" = "South Asia",
  "Indonesia" = "Southeast Asia",
  "Japan" = "East Asia",
  "Malaysia" = "Southeast Asia",
  "Maldives" = "South Asia",
  "Nepal" = "South Asia",
  "Sri Lanka" = "South Asia",
  "Taiwan" = "East Asia",
  "Thailand" = "Southeast Asia",
  "Vietnam" = "Southeast Asia"
)

# Add the region to the data frame
sampling_loc$Region2 <- country_to_region[sampling_loc$Country]

# Melt the data frame for plotting
Q_melted <- leak5 |>
  pivot_longer(
    cols = -c(ind, pop),
    names_to = "variable",
    values_to = "value"
  )
# Join with sampling_loc to get sampling localities
Q_joined <- Q_melted |>
  left_join(sampling_loc, by = c("pop" = "Abbreviation"))

# Create a combined variable for Region and Country
Q_joined <- Q_joined |>
  mutate(Region_Country = interaction(Region, Country, sep = "_"))

# Order the combined variable by Region and Country, then by individual
Q_ordered <- Q_joined |>
  arrange(Region, Region2, Country, ind) |>
  mutate(ind = factor(ind, levels = unique(ind)))  # Convert ind to a factor with levels in the desired order

# Add labels: country names for the first individual in each country, NA for all other individuals
Q_ordered <- Q_ordered |>
  group_by(Region_Country) |>
  mutate(label = ifelse(row_number() == 1, as.character(Country), NA))

# Group by individual and variable, calculate mean ancestry proportions
Q_grouped <- Q_ordered |>
  group_by(ind, variable) |>
  summarise(value = mean(value), .groups = "drop")

# Create a data frame for borders
borders <-
  data.frame(Region_Country = unique(Q_ordered$Region_Country))

# Add the order of the last individual of each country to ensure correct placement of borders
borders$order <-
  sapply(borders$Region_Country, function(rc)
    max(which(Q_ordered$Region_Country == rc))) + 0.5  # Shift borders to the right edge of the bars

# Select only the first occurrence of each country in the ordered data
label_df <- Q_ordered |>
  filter(!is.na(label)) |>
  distinct(label, .keep_all = TRUE)

# Create a custom label function
label_func <- function(x) {
  labels <- rep("", length(x))
  labels[x %in% label_df$ind] <- label_df$label
  labels
}

# Calculate the position of lines
border_positions <- Q_ordered |>
  group_by(Country) |>
  summarise(pos = max(as.numeric(ind)) + 0)

# Calculate the position of population labels and bars
pop_labels <- Q_ordered |>
  mutate(Name = paste(pop, Pop_City, sep = " - ")) |>
  group_by(pop) |>
  slice_head(n = 1) |>
  ungroup() |>
  dplyr::select(ind, Pop_City, Country, Name) |>
  mutate(pos = as.numeric(ind))  # calculate position of population labels

pop_labels_bars <- pop_labels |>
  mutate(pos = as.numeric(ind)  - .5)


# Calculate the position of lines
border_positions <- Q_ordered |>
  group_by(Country) |>
  summarise(pos = max(as.numeric(ind)) - 1)


pop_labels_bars <- pop_labels |>
  mutate(pos = as.numeric(ind)  - .5)

# Function to filter and normalize data
normalize_data <- function(df, min_value) {
  df |>
    filter(value > min_value) |>
    group_by(ind) |>
    mutate(value = value / sum(value))
}

# Use the function
Q_grouped_filtered <- normalize_data(Q_grouped, 0.1)
# 
color_palette <-
  c(
    "v1" = "#FFFF00",
    "v2" = "#FF00FF",
    "v3" = "#0000FF",
    "v4" = "#FF0000",
    "v5" = "#00FF00"
  )

# Generate all potential variable names
all_variables <- paste0("v", 1:5)

# Map each variable to a name
color_mapping <- data.frame(variable = all_variables,
                            color = names(color_palette))

# Merge with Q_grouped_filtered
Q_grouped_filtered <- merge(Q_grouped_filtered, color_mapping, by = "variable")

# Create the plot
ggplot(Q_grouped_filtered, aes(x = as.factor(ind), y = value, fill = color)) +
  geom_bar(stat = 'identity', width = 1) +
  geom_vline(
    data = pop_labels_bars,
    aes(xintercept = pos),
    color = "#2C444A",
    linewidth = .2
  ) +
  geom_text(
    data = pop_labels,
    aes(x = as.numeric(ind), y = 1, label = Name),
    vjust = 1.5,
    hjust = 0,
    size = 2,
    angle = 90,
    inherit.aes = FALSE
  ) +
  my_theme() +
  theme(
    axis.text.x = element_text(
      angle = 90,
      hjust = 1,
      size = 12
    ),
    legend.position = "none",
    plot.margin = unit(c(3, 0.5, 0.5, 0.5), "cm")
  ) +
  xlab("Admixture matrix") +
  ylab("Ancestry proportions") +
  labs(caption = "Each bar represents the ancestry proportions for an individual for k=5.\n LEA inference for k1:25 for random8.") +
  scale_x_discrete(labels = label_func) +
  scale_fill_manual(values = color_palette) +
  expand_limits(y = c(0, 1.5))
```

```
ggsave(
  here("output", "variant_proportions", "figures", "random8.pdf"),
  width  = 12,
  height = 6,
  units  = "in",
  device = cairo_pdf
)
```

#### 3.5.2 Interploation over map

Clear memory and environment

```
# Clear entire environment
rm(list = ls())
# Forcefully trigger garbage collection
gc()
```

```
##           used  (Mb) gc trigger  (Mb) limit (Mb) max used  (Mb)
## Ncells 5644312 301.5   15241385 814.0         NA 15241385 814.0
## Vcells 9055855  69.1   41317233 315.3      32768 56276493 429.4
```

```
# Matrix
leak5 <- read_delim(
  here("output", "variant_proportions", "random8.snmf", "K5", "run1","random8_r1.5.Q"),
  delim = " ",
  col_names = FALSE,
  show_col_types = FALSE
) 

head(leak5)
```

```
## # A tibble: 6 × 5
##         X1       X2       X3    X4       X5
##      <dbl>    <dbl>    <dbl> <dbl>    <dbl>
## 1 0.0223   0.0799   0.0233   0.814 0.0608  
## 2 0.147    0.162    0.0286   0.574 0.0877  
## 3 0.000100 0.0576   0.000100 0.942 0.000100
## 4 0.000100 0.000100 0.000100 1.00  0.000100
## 5 0.000100 0.000100 0.000100 1.00  0.000100
## 6 0.00358  0.0995   0.0122   0.885 0.000100
```

The fam file

```
fam_file <- here(
  "output", "populations", "snps_sets", "neutral.fam"
)

# Read the .fam file
fam_data <- read.table(fam_file, 
                       header = FALSE,
                       col.names = c("FamilyID", "IndividualID", "PaternalID", "MaternalID", "Sex", "Phenotype"))

# View the first few rows
head(fam_data)
```

```
##   FamilyID IndividualID PaternalID MaternalID Sex Phenotype
## 1      OKI         1001          0          0   2        -9
## 2      OKI         1002          0          0   2        -9
## 3      OKI         1003          0          0   2        -9
## 4      OKI         1004          0          0   2        -9
## 5      OKI         1005          0          0   2        -9
## 6      OKI         1006          0          0   1        -9
```

Create ID column

```
# Change column name
colnames(fam_data)[colnames(fam_data) == "IndividualID"] <- "ind"

# Change column name
colnames(fam_data)[colnames(fam_data) == "FamilyID"] <- "pop"

# Select ID
fam_data <- fam_data |>
  dplyr::select("ind", "pop")

# View the first few rows
head(fam_data)
```

```
##    ind pop
## 1 1001 OKI
## 2 1002 OKI
## 3 1003 OKI
## 4 1004 OKI
## 5 1005 OKI
## 6 1006 OKI
```

Add it to matrix

```
leak5 <- fam_data |>
  dplyr::select(ind, pop) |>
  bind_cols(leak5)

head(leak5)
```

```
##    ind pop          X1         X2          X3       X4          X5
## 1 1001 OKI 0.022296300 0.07994870 0.023336900 0.813667 0.060750600
## 2 1002 OKI 0.147284000 0.16191200 0.028616800 0.574449 0.087738100
## 3 1003 OKI 0.000099973 0.05759880 0.000099973 0.942101 0.000099973
## 4 1004 OKI 0.000099970 0.00009997 0.000099970 0.999600 0.000099970
## 5 1005 OKI 0.000099970 0.00009997 0.000099970 0.999600 0.000099970
## 6 1006 OKI 0.003580470 0.09951710 0.012223200 0.884579 0.000099991
```

Rename the columns

```
# Rename the columns starting from the third one
leak5 <- leak5 |>
  rename_with(~paste0("v", seq_along(.x)), .cols = -c(ind, pop))

# View the first few rows
head(leak5)
```

```
##    ind pop          v1         v2          v3       v4          v5
## 1 1001 OKI 0.022296300 0.07994870 0.023336900 0.813667 0.060750600
## 2 1002 OKI 0.147284000 0.16191200 0.028616800 0.574449 0.087738100
## 3 1003 OKI 0.000099973 0.05759880 0.000099973 0.942101 0.000099973
## 4 1004 OKI 0.000099970 0.00009997 0.000099970 0.999600 0.000099970
## 5 1005 OKI 0.000099970 0.00009997 0.000099970 0.999600 0.000099970
## 6 1006 OKI 0.003580470 0.09951710 0.012223200 0.884579 0.000099991
```

Import samples attributes

```
sampling_loc <- readRDS(here("output", "populations", "sampling_loc.rds"))
# head(sampling_loc)

pops <- sampling_loc |>
  filter(
    Region == "Asia"
  ) |>
  dplyr::select(
    Abbreviation, Latitude, Longitude, Pop_City, Country
  )

head(pops)
```

```
## # A tibble: 6 × 5
##   Abbreviation Latitude Longitude Pop_City   Country 
##   <chr>           <dbl>     <dbl> <chr>      <chr>   
## 1 GEL              26.9      90.5 Gelephu    Bhutan  
## 2 CAM              11.6     105.  Phnom Penh Cambodia
## 3 HAI              19.2     110.  Hainan     China   
## 4 YUN              24.5     101.  Yunnan     China   
## 5 HUN              27.6     112.  Hunan      China   
## 6 BEN              13.0      77.6 Bengaluru  India
```

Merge with pops

```
# Add an index column to Q_tibble
leak5$index <- seq_len(nrow(leak5))

# Perform the merge as before
df1 <-
  merge(
    leak5,
    pops,
    by.x = 2,
    by.y = 1,
    all.x = T,
    all.y = F
  ) |>
  na.omit()

# Order by the index column to ensure the order matches the original Q_tibble
df1 <- df1[order(df1$index),]

# Optionally, you can remove the index column if it's no longer needed
df1$index <- NULL

# Now the rows of df1 should be in the same order as the original Q_tibble
head(df1)
```

```
##     pop  ind          v1         v2          v3       v4          v5 Latitude
## 159 OKI 1001 0.022296300 0.07994870 0.023336900 0.813667 0.060750600  26.5013
## 160 OKI 1002 0.147284000 0.16191200 0.028616800 0.574449 0.087738100  26.5013
## 161 OKI 1003 0.000099973 0.05759880 0.000099973 0.942101 0.000099973  26.5013
## 162 OKI 1004 0.000099970 0.00009997 0.000099970 0.999600 0.000099970  26.5013
## 163 OKI 1005 0.000099970 0.00009997 0.000099970 0.999600 0.000099970  26.5013
## 164 OKI 1006 0.003580470 0.09951710 0.012223200 0.884579 0.000099991  26.5013
##     Longitude Pop_City Country
## 159  127.9454  Okinawa   Japan
## 160  127.9454  Okinawa   Japan
## 161  127.9454  Okinawa   Japan
## 162  127.9454  Okinawa   Japan
## 163  127.9454  Okinawa   Japan
## 164  127.9454  Okinawa   Japan
```

We used this color palette to make the “structure” plot

```
color_palette2 <-
  c(
    "v1" = "#FFFF00",
    "v2" = "#FF00FF",
    "v3" = "#0000FF",
    "v4" = "#FF0000",
    "v5" = "#00FF00"
  )
```

Preparing the data for tess3Q\_map\_rasters

Make sure the lat longs are in the correct order and arrangement

```
df2 <- df1 |>
  dplyr::rename(
    Long = Longitude,
    Lat = Latitude
  )

long_lat_tibble <- df2 |>
  dplyr::select(Long, Lat)


long_lat_matrix <- long_lat_tibble |>
  as.matrix()

head(long_lat_matrix)
```

```
##         Long     Lat
## 159 127.9454 26.5013
## 160 127.9454 26.5013
## 161 127.9454 26.5013
## 162 127.9454 26.5013
## 163 127.9454 26.5013
## 164 127.9454 26.5013
```

Make a matrix of the Q values

Pull off the names of individuals and make a matrix of it:

```
Q_matrix <- leak5 |>
  dplyr::select(-ind, -pop, -index) |>
  as.matrix()
head(Q_matrix)
```

```
##               v1         v2          v3       v4          v5
## [1,] 0.022296300 0.07994870 0.023336900 0.813667 0.060750600
## [2,] 0.147284000 0.16191200 0.028616800 0.574449 0.087738100
## [3,] 0.000099973 0.05759880 0.000099973 0.942101 0.000099973
## [4,] 0.000099970 0.00009997 0.000099970 0.999600 0.000099970
## [5,] 0.000099970 0.00009997 0.000099970 0.999600 0.000099970
## [6,] 0.003580470 0.09951710 0.012223200 0.884579 0.000099991
```

Interpolate the Q-values by Kriging

```
print(ncol(Q_matrix) == length(color_palette2))
```

```
## [1] TRUE
```

Create brick

```
world <- ne_countries(scale = "medium", returnclass = "sf")
countries_with_data <- unique(df1$Country)

# Filtering the world data to include only the countries in your data
selected_countries <- world |>
  filter(admin %in% countries_with_data)

genoscape_brick <- tess3r::tess3Q_map_rasters(
  x = Q_matrix, 
  coord = long_lat_matrix,  
  map.polygon = selected_countries,
  window = extent(selected_countries)[1:4],
  # window = combined_extent,
  resolution = c(600,600), # if you want more cells in your raster, set higher
  # this next lines need to to be here, but don't do much...
  col.palette = tess3r::CreatePalette(color_palette2, length(color_palette2)),
  method = "map.max", 
  interpol = tess3r::FieldsKrigModel(80),  
  main = "Ancestry coefficients",
  xlab = "Longitude", 
  ylab = "Latitude", 
  cex = .4
)
```

```
## Warning: 
## Grid searches over lambda (nugget and sill variances) with  minima at the endpoints: 
##   (REML) Restricted maximum likelihood 
##    minimum at  right endpoint  lambda  =  0.02118404 (eff. df= 26.59999 )
## Warning: 
## Grid searches over lambda (nugget and sill variances) with  minima at the endpoints: 
##   (REML) Restricted maximum likelihood 
##    minimum at  right endpoint  lambda  =  0.02118404 (eff. df= 26.59999 )
## Warning: 
## Grid searches over lambda (nugget and sill variances) with  minima at the endpoints: 
##   (REML) Restricted maximum likelihood 
##    minimum at  right endpoint  lambda  =  0.02118404 (eff. df= 26.59999 )
## Warning: 
## Grid searches over lambda (nugget and sill variances) with  minima at the endpoints: 
##   (REML) Restricted maximum likelihood 
##    minimum at  right endpoint  lambda  =  0.02118404 (eff. df= 26.59999 )
```

```
# after that, we need to add names of the clusters back onto this raster brick
Q_tibble2 <- leak5 |>
  dplyr::select(
    -pop, -ind, -index
  )
names(genoscape_brick) <- names(Q_tibble2)[]
```

Scaling and cleaning the genoscape\_brick

```
genoscape_rgba <- genoscapeRtools::qprob_rando_raster(
  TRB = genoscape_brick,
  cols = color_palette2,
  alpha_scale = 2.0,
  abs_thresh = 0.0,
  alpha_exp = 1.55,
  alpha_chop_max = 255
)

# This adds the info for a regular lat-long projection
crs(genoscape_rgba) <- "+proj=longlat +datum=WGS84 +no_defs +ellps=WGS84 +towgs84=0,0,0"
```

Plot with pies

```
source(
  here(
    "scripts", "analysis", "my_theme2.R"
  )
)

# Calculate mean proportions for each population
df_mean <- df1 |>
  group_by(pop) |>
  summarise(across(starts_with("v"), \(x) mean(x, na.rm = TRUE)), 
            Longitude = mean(Longitude),
            Latitude = mean(Latitude))

ggplot() +
  layer_spatial(genoscape_rgba) +
  geom_spatial_point(data = long_lat_tibble,
                     mapping = aes(x = Long, y = Lat),
                     size = .2) +
  geom_text_repel(
    data = df_mean,
    aes(x = Longitude, y = Latitude, label = pop),
    size = 3,
    box.padding = unit(0.5, "lines")
  ) +
  labs(x = "Longitude",
       y = "Latitude") +
  geom_scatterpie(data = df_mean, 
                  aes(x = Longitude, y = Latitude, r = 1), 
                  cols = c("v1", "v2", "v3", "v4", "v5"), color = NA) +
  my_theme() +
  scale_fill_manual(values = color_palette2) +
  guides(fill = "none") +  # Hide legend
  coord_sf()
```

```
## Assuming `crs = 4326` in stat_spatial_identity()
```

```
# # 
ggsave(
  here("output", "variant_proportions", "figures", "random8_map.pdf"),
  width  = 6,
  height = 6,
  units  = "in",
  device = cairo_pdf
)
```

```
## Assuming `crs = 4326` in stat_spatial_identity()
```

### 3.6 random9

Clean env and memory

```
rm(list=ls())
gc()
```

```
##            used  (Mb) gc trigger  (Mb) limit (Mb) max used  (Mb)
## Ncells  5644105 301.5   15241385 814.0         NA 15241385 814.0
## Vcells 10496864  80.1   39728544 303.2      32768 56276493 429.4
```

Import the data

```
genotype <- here(
    "output", "variant_proportions", "random9.vcf"
  )

d <- read.vcfR(
  genotype
)
```

```
## Scanning file to determine attributes.
## File attributes:
##   meta lines: 8
##   header_line: 9
##   variant count: 33400
##   column count: 246
## 
Meta line 8 read in.
## All meta lines processed.
## gt matrix initialized.
## Character matrix gt created.
##   Character matrix gt rows: 33400
##   Character matrix gt cols: 246
##   skip: 0
##   nrows: 33400
##   row_num: 0
## 
Processed variant 1000
Processed variant 2000
Processed variant 3000
Processed variant 4000
Processed variant 5000
Processed variant 6000
Processed variant 7000
Processed variant 8000
Processed variant 9000
Processed variant 10000
Processed variant 11000
Processed variant 12000
Processed variant 13000
Processed variant 14000
Processed variant 15000
Processed variant 16000
Processed variant 17000
Processed variant 18000
Processed variant 19000
Processed variant 20000
Processed variant 21000
Processed variant 22000
Processed variant 23000
Processed variant 24000
Processed variant 25000
Processed variant 26000
Processed variant 27000
Processed variant 28000
Processed variant 29000
Processed variant 30000
Processed variant 31000
Processed variant 32000
Processed variant 33000
Processed variant: 33400
## All variants processed
```

Population and individuals information

```
inds_full <- attr(d@gt,"dimnames")[[2]]
inds_full <- inds_full[-1]
a <- strsplit(inds_full, '_')
pops <- unname(sapply(a, FUN = function(x) return(as.character(x[1])))) 
table(pops)
```

```
## pops
## BEN CAM CHA GEL HAI HAN HOC HUN INJ INW JAF KAC KAG KAN KAT KLP KUN LAM MAT OKI 
##  12  12  12   2  12   4   7  12  11   4   2   6  12  11  10   4   4   9  12  11 
## QNC SON SSK SUF SUU TAI UTS YUN 
##  12   3  12   6   6   8  12   9
```

```
pops <- factor(pops)
inds <- unname(sapply(a, FUN = function(x) return(as.character(x[2]))))
```

Convert format

```
vcf2geno(genotype, gsub(".vcf", ".geno", genotype))
```

```
## 
##  - number of detected individuals:   237
##  - number of detected loci:      33400
## 
## For SNP info, please check /Users/lucianocosme/Library/CloudStorage/Dropbox/Albopictus/manuscript_chip/data/no_autogenous/albo_chip/output/variant_proportions/random9.vcfsnp.
## 
## 0 line(s) were removed because these are not SNPs.
## Please, check /Users/lucianocosme/Library/CloudStorage/Dropbox/Albopictus/manuscript_chip/data/no_autogenous/albo_chip/output/variant_proportions/random9.removed file, for more informations.
```

```
## [1] "/Users/lucianocosme/Library/CloudStorage/Dropbox/Albopictus/manuscript_chip/data/no_autogenous/albo_chip/output/variant_proportions/random9.geno"
```

```
vcf2lfmm(genotype, gsub(".vcf", ".lfmm", genotype))
```

```
## 
##  - number of detected individuals:   237
##  - number of detected loci:      33400
## 
## For SNP info, please check /Users/lucianocosme/Library/CloudStorage/Dropbox/Albopictus/manuscript_chip/data/no_autogenous/albo_chip/output/variant_proportions/random9.vcfsnp.
## 
## 0 line(s) were removed because these are not SNPs.
## Please, check /Users/lucianocosme/Library/CloudStorage/Dropbox/Albopictus/manuscript_chip/data/no_autogenous/albo_chip/output/variant_proportions/random9.removed file, for more informations.
## 
## 
##  - number of detected individuals:   237
##  - number of detected loci:      33400
```

```
## [1] "/Users/lucianocosme/Library/CloudStorage/Dropbox/Albopictus/manuscript_chip/data/no_autogenous/albo_chip/output/variant_proportions/random9.lfmm"
```

Run LEA

```
# set output dir
setwd(
  here(
    "output", "variant_proportions"
  )
)
# main options
# K = number of ancestral populations
# entropy = TRUE computes the cross-entropy criterion, # CPU = 4 is the number of CPU used (hidden input) project = NULL
project = snmf(
  genotype,
  K = 1:25,
  project = "new",
  repetitions = 1,
  alpha = 5,
  tolerance = 0.0001,
  percentage = 0.5,
  iterations = 200, 
  ploidy = 2,
  seed = 13,
  CPU = 4,
  entropy = TRUE
)
```

```
project = load.snmfProject("output/variant_proportions/random9.snmfProject")
```

Cross entropy

```
# Open a new pdf file
pdf(here("output","variant_proportions","figures","cross_entropy_random9.pdf"), width = 6, height = 4)

# Create your plot
plot(project, col = "pink", pch = 19, cex = 1.2)

# Close the pdf file
dev.off()
```

```
## quartz_off_screen 
##                 2
```

```
plot(project, col = "pink", pch = 19, cex = 1.2)
```

#### 3.6.1 Structure plot

```
# Matrix
leak5 <- read_delim(
  here("output", "variant_proportions", "random9.snmf", "K5", "run1","random9_r1.5.Q"),
  delim = " ",
  col_names = FALSE,
  show_col_types = FALSE
) 


head(leak5)
```

```
## # A tibble: 6 × 5
##         X1     X2       X3     X4    X5
##      <dbl>  <dbl>    <dbl>  <dbl> <dbl>
## 1 0.00479  0.115  0.111    0.188  0.581
## 2 0.0182   0.125  0.127    0.162  0.568
## 3 0.0273   0.0801 0.0379   0.110  0.744
## 4 0.00827  0.0895 0.0549   0.127  0.720
## 5 0.0176   0.0823 0.0648   0.0740 0.761
## 6 0.000100 0.170  0.000100 0.116  0.713
```

The fam file

```
fam_file <- here(
  "output", "populations", "snps_sets", "neutral.fam"
)

# Read the .fam file
fam_data <- read.table(fam_file, 
                       header = FALSE,
                       col.names = c("FamilyID", "IndividualID", "PaternalID", "MaternalID", "Sex", "Phenotype"))

# View the first few rows
head(fam_data)
```

```
##   FamilyID IndividualID PaternalID MaternalID Sex Phenotype
## 1      OKI         1001          0          0   2        -9
## 2      OKI         1002          0          0   2        -9
## 3      OKI         1003          0          0   2        -9
## 4      OKI         1004          0          0   2        -9
## 5      OKI         1005          0          0   2        -9
## 6      OKI         1006          0          0   1        -9
```

Create ID column

```
# Change column name
colnames(fam_data)[colnames(fam_data) == "IndividualID"] <- "ind"

# Change column name
colnames(fam_data)[colnames(fam_data) == "FamilyID"] <- "pop"

# Select ID
fam_data <- fam_data |>
  dplyr::select("ind", "pop")

# View the first few rows
head(fam_data)
```

```
##    ind pop
## 1 1001 OKI
## 2 1002 OKI
## 3 1003 OKI
## 4 1004 OKI
## 5 1005 OKI
## 6 1006 OKI
```

Add it to matrix

```
leak5 <- fam_data |>
  dplyr::select(ind, pop) |>
  bind_cols(leak5)

head(leak5)
```

```
##    ind pop          X1        X2          X3        X4       X5
## 1 1001 OKI 0.004786660 0.1147900 0.110925000 0.1884980 0.581000
## 2 1002 OKI 0.018223700 0.1249830 0.127298000 0.1617700 0.567725
## 3 1003 OKI 0.027307100 0.0800896 0.037878500 0.1104490 0.744275
## 4 1004 OKI 0.008272000 0.0895303 0.054886200 0.1273670 0.719945
## 5 1005 OKI 0.017599600 0.0823412 0.064786500 0.0739732 0.761300
## 6 1006 OKI 0.000099982 0.1700950 0.000099982 0.1164760 0.713230
```

Rename the columns

```
# Rename the columns starting from the third one
leak5 <- leak5 |>
  rename_with(~paste0("v", seq_along(.x)), .cols = -c(ind, pop))

# View the first few rows
head(leak5)
```

```
##    ind pop          v1        v2          v3        v4       v5
## 1 1001 OKI 0.004786660 0.1147900 0.110925000 0.1884980 0.581000
## 2 1002 OKI 0.018223700 0.1249830 0.127298000 0.1617700 0.567725
## 3 1003 OKI 0.027307100 0.0800896 0.037878500 0.1104490 0.744275
## 4 1004 OKI 0.008272000 0.0895303 0.054886200 0.1273670 0.719945
## 5 1005 OKI 0.017599600 0.0823412 0.064786500 0.0739732 0.761300
## 6 1006 OKI 0.000099982 0.1700950 0.000099982 0.1164760 0.713230
```

Import sample locations

```
sampling_loc <- readRDS(here("output", "populations", "sampling_loc.rds"))
head(sampling_loc)
```

```
## # A tibble: 6 × 6
##   Pop_City   Country  Latitude Longitude Region Abbreviation
##   <chr>      <chr>       <dbl>     <dbl> <chr>  <chr>       
## 1 Gelephu    Bhutan       26.9      90.5 Asia   GEL         
## 2 Phnom Penh Cambodia     11.6     105.  Asia   CAM         
## 3 Hainan     China        19.2     110.  Asia   HAI         
## 4 Yunnan     China        24.5     101.  Asia   YUN         
## 5 Hunan      China        27.6     112.  Asia   HUN         
## 6 Bengaluru  India        13.0      77.6 Asia   BEN
```

Structure plot

```
source(
  here(
    "scripts", "analysis", "my_theme3.R"
  )
)

# Create a named vector to map countries to regions
country_to_region <- c(
  "Bhutan" = "South Asia",
  "Cambodia" = "Southeast Asia",
  "China" = "East Asia",
  "India" = "South Asia",
  "Indonesia" = "Southeast Asia",
  "Japan" = "East Asia",
  "Malaysia" = "Southeast Asia",
  "Maldives" = "South Asia",
  "Nepal" = "South Asia",
  "Sri Lanka" = "South Asia",
  "Taiwan" = "East Asia",
  "Thailand" = "Southeast Asia",
  "Vietnam" = "Southeast Asia"
)

# Add the region to the data frame
sampling_loc$Region2 <- country_to_region[sampling_loc$Country]

# Melt the data frame for plotting
Q_melted <- leak5 |>
  pivot_longer(
    cols = -c(ind, pop),
    names_to = "variable",
    values_to = "value"
  )
# Join with sampling_loc to get sampling localities
Q_joined <- Q_melted |>
  left_join(sampling_loc, by = c("pop" = "Abbreviation"))

# Create a combined variable for Region and Country
Q_joined <- Q_joined |>
  mutate(Region_Country = interaction(Region, Country, sep = "_"))

# Order the combined variable by Region and Country, then by individual
Q_ordered <- Q_joined |>
  arrange(Region, Region2, Country, ind) |>
  mutate(ind = factor(ind, levels = unique(ind)))  # Convert ind to a factor with levels in the desired order

# Add labels: country names for the first individual in each country, NA for all other individuals
Q_ordered <- Q_ordered |>
  group_by(Region_Country) |>
  mutate(label = ifelse(row_number() == 1, as.character(Country), NA))

# Group by individual and variable, calculate mean ancestry proportions
Q_grouped <- Q_ordered |>
  group_by(ind, variable) |>
  summarise(value = mean(value), .groups = "drop")

# Create a data frame for borders
borders <-
  data.frame(Region_Country = unique(Q_ordered$Region_Country))

# Add the order of the last individual of each country to ensure correct placement of borders
borders$order <-
  sapply(borders$Region_Country, function(rc)
    max(which(Q_ordered$Region_Country == rc))) + 0.5  # Shift borders to the right edge of the bars

# Select only the first occurrence of each country in the ordered data
label_df <- Q_ordered |>
  filter(!is.na(label)) |>
  distinct(label, .keep_all = TRUE)

# Create a custom label function
label_func <- function(x) {
  labels <- rep("", length(x))
  labels[x %in% label_df$ind] <- label_df$label
  labels
}

# Calculate the position of lines
border_positions <- Q_ordered |>
  group_by(Country) |>
  summarise(pos = max(as.numeric(ind)) + 0)

# Calculate the position of population labels and bars
pop_labels <- Q_ordered |>
  mutate(Name = paste(pop, Pop_City, sep = " - ")) |>
  group_by(pop) |>
  slice_head(n = 1) |>
  ungroup() |>
  dplyr::select(ind, Pop_City, Country, Name) |>
  mutate(pos = as.numeric(ind))  # calculate position of population labels

pop_labels_bars <- pop_labels |>
  mutate(pos = as.numeric(ind)  - .5)


# Calculate the position of lines
border_positions <- Q_ordered |>
  group_by(Country) |>
  summarise(pos = max(as.numeric(ind)) - 1)


pop_labels_bars <- pop_labels |>
  mutate(pos = as.numeric(ind)  - .5)

# Function to filter and normalize data
normalize_data <- function(df, min_value) {
  df |>
    filter(value > min_value) |>
    group_by(ind) |>
    mutate(value = value / sum(value))
}

# Use the function
Q_grouped_filtered <- normalize_data(Q_grouped, 0.1)
# 
color_palette <-
  c(
    "v1" = "#0000FF",
    "v2" = "#FF00FF",
    "v3" = "#FFFF00",
    "v4" = "#00FF00",
    "v5" = "#FF0000"
  )
 
# Generate all potential variable names
all_variables <- paste0("v", 1:5)

# Map each variable to a name
color_mapping <- data.frame(variable = all_variables,
                            color = names(color_palette))

# Merge with Q_grouped_filtered
Q_grouped_filtered <- merge(Q_grouped_filtered, color_mapping, by = "variable")

# Create the plot
ggplot(Q_grouped_filtered, aes(x = as.factor(ind), y = value, fill = color)) +
  geom_bar(stat = 'identity', width = 1) +
  geom_vline(
    data = pop_labels_bars,
    aes(xintercept = pos),
    color = "#2C444A",
    linewidth = .2
  ) +
  geom_text(
    data = pop_labels,
    aes(x = as.numeric(ind), y = 1, label = Name),
    vjust = 1.5,
    hjust = 0,
    size = 2,
    angle = 90,
    inherit.aes = FALSE
  ) +
  my_theme() +
  theme(
    axis.text.x = element_text(
      angle = 90,
      hjust = 1,
      size = 12
    ),
    legend.position = "none",
    plot.margin = unit(c(3, 0.5, 0.5, 0.5), "cm")
  ) +
  xlab("Admixture matrix") +
  ylab("Ancestry proportions") +
  labs(caption = "Each bar represents the ancestry proportions for an individual for k=5.\n LEA inference for k1:25 for random9.") +
  scale_x_discrete(labels = label_func) +
  scale_fill_manual(values = color_palette) +
  expand_limits(y = c(0, 1.5))
```

```
ggsave(
  here("output", "variant_proportions", "figures", "random9.pdf"),
  width  = 12,
  height = 6,
  units  = "in",
  device = cairo_pdf
)
```

#### 3.6.2 Interploation over map

Clear memory and environment

```
# Clear entire environment
rm(list = ls())
# Forcefully trigger garbage collection
gc()
```

```
##           used  (Mb) gc trigger  (Mb) limit (Mb) max used  (Mb)
## Ncells 5644808 301.5   15241385 814.0         NA 15241385 814.0
## Vcells 9067880  69.2   41328320 315.4      32768 56276493 429.4
```

```
# Matrix
leak5 <- read_delim(
  here("output", "variant_proportions", "random9.snmf", "K5", "run1","random9_r1.5.Q"),
  delim = " ",
  col_names = FALSE,
  show_col_types = FALSE
) 

head(leak5)
```

```
## # A tibble: 6 × 5
##         X1     X2       X3     X4    X5
##      <dbl>  <dbl>    <dbl>  <dbl> <dbl>
## 1 0.00479  0.115  0.111    0.188  0.581
## 2 0.0182   0.125  0.127    0.162  0.568
## 3 0.0273   0.0801 0.0379   0.110  0.744
## 4 0.00827  0.0895 0.0549   0.127  0.720
## 5 0.0176   0.0823 0.0648   0.0740 0.761
## 6 0.000100 0.170  0.000100 0.116  0.713
```

The fam file

```
fam_file <- here(
  "output", "populations", "snps_sets", "neutral.fam"
)

# Read the .fam file
fam_data <- read.table(fam_file, 
                       header = FALSE,
                       col.names = c("FamilyID", "IndividualID", "PaternalID", "MaternalID", "Sex", "Phenotype"))

# View the first few rows
head(fam_data)
```

```
##   FamilyID IndividualID PaternalID MaternalID Sex Phenotype
## 1      OKI         1001          0          0   2        -9
## 2      OKI         1002          0          0   2        -9
## 3      OKI         1003          0          0   2        -9
## 4      OKI         1004          0          0   2        -9
## 5      OKI         1005          0          0   2        -9
## 6      OKI         1006          0          0   1        -9
```

Create ID column

```
# Change column name
colnames(fam_data)[colnames(fam_data) == "IndividualID"] <- "ind"

# Change column name
colnames(fam_data)[colnames(fam_data) == "FamilyID"] <- "pop"

# Select ID
fam_data <- fam_data |>
  dplyr::select("ind", "pop")

# View the first few rows
head(fam_data)
```

```
##    ind pop
## 1 1001 OKI
## 2 1002 OKI
## 3 1003 OKI
## 4 1004 OKI
## 5 1005 OKI
## 6 1006 OKI
```

Add it to matrix

```
leak5 <- fam_data |>
  dplyr::select(ind, pop) |>
  bind_cols(leak5)

head(leak5)
```

```
##    ind pop          X1        X2          X3        X4       X5
## 1 1001 OKI 0.004786660 0.1147900 0.110925000 0.1884980 0.581000
## 2 1002 OKI 0.018223700 0.1249830 0.127298000 0.1617700 0.567725
## 3 1003 OKI 0.027307100 0.0800896 0.037878500 0.1104490 0.744275
## 4 1004 OKI 0.008272000 0.0895303 0.054886200 0.1273670 0.719945
## 5 1005 OKI 0.017599600 0.0823412 0.064786500 0.0739732 0.761300
## 6 1006 OKI 0.000099982 0.1700950 0.000099982 0.1164760 0.713230
```

Rename the columns

```
# Rename the columns starting from the third one
leak5 <- leak5 |>
  rename_with(~paste0("v", seq_along(.x)), .cols = -c(ind, pop))

# View the first few rows
head(leak5)
```

```
##    ind pop          v1        v2          v3        v4       v5
## 1 1001 OKI 0.004786660 0.1147900 0.110925000 0.1884980 0.581000
## 2 1002 OKI 0.018223700 0.1249830 0.127298000 0.1617700 0.567725
## 3 1003 OKI 0.027307100 0.0800896 0.037878500 0.1104490 0.744275
## 4 1004 OKI 0.008272000 0.0895303 0.054886200 0.1273670 0.719945
## 5 1005 OKI 0.017599600 0.0823412 0.064786500 0.0739732 0.761300
## 6 1006 OKI 0.000099982 0.1700950 0.000099982 0.1164760 0.713230
```

Import samples attributes

```
sampling_loc <- readRDS(here("output", "populations", "sampling_loc.rds"))
# head(sampling_loc)

pops <- sampling_loc |>
  filter(
    Region == "Asia"
  ) |>
  dplyr::select(
    Abbreviation, Latitude, Longitude, Pop_City, Country
  )

head(pops)
```

```
## # A tibble: 6 × 5
##   Abbreviation Latitude Longitude Pop_City   Country 
##   <chr>           <dbl>     <dbl> <chr>      <chr>   
## 1 GEL              26.9      90.5 Gelephu    Bhutan  
## 2 CAM              11.6     105.  Phnom Penh Cambodia
## 3 HAI              19.2     110.  Hainan     China   
## 4 YUN              24.5     101.  Yunnan     China   
## 5 HUN              27.6     112.  Hunan      China   
## 6 BEN              13.0      77.6 Bengaluru  India
```

Merge with pops

```
# Add an index column to Q_tibble
leak5$index <- seq_len(nrow(leak5))

# Perform the merge as before
df1 <-
  merge(
    leak5,
    pops,
    by.x = 2,
    by.y = 1,
    all.x = T,
    all.y = F
  ) |>
  na.omit()

# Order by the index column to ensure the order matches the original Q_tibble
df1 <- df1[order(df1$index),]

# Optionally, you can remove the index column if it's no longer needed
df1$index <- NULL

# Now the rows of df1 should be in the same order as the original Q_tibble
head(df1)
```

```
##     pop  ind          v1        v2          v3        v4       v5 Latitude
## 159 OKI 1001 0.004786660 0.1147900 0.110925000 0.1884980 0.581000  26.5013
## 160 OKI 1002 0.018223700 0.1249830 0.127298000 0.1617700 0.567725  26.5013
## 161 OKI 1003 0.027307100 0.0800896 0.037878500 0.1104490 0.744275  26.5013
## 162 OKI 1004 0.008272000 0.0895303 0.054886200 0.1273670 0.719945  26.5013
## 163 OKI 1005 0.017599600 0.0823412 0.064786500 0.0739732 0.761300  26.5013
## 164 OKI 1006 0.000099982 0.1700950 0.000099982 0.1164760 0.713230  26.5013
##     Longitude Pop_City Country
## 159  127.9454  Okinawa   Japan
## 160  127.9454  Okinawa   Japan
## 161  127.9454  Okinawa   Japan
## 162  127.9454  Okinawa   Japan
## 163  127.9454  Okinawa   Japan
## 164  127.9454  Okinawa   Japan
```

We used this color palette to make the “structure” plot

```
color_palette2 <-
  c(
    "v1" = "#0000FF",
    "v2" = "#FF00FF",
    "v3" = "#FFFF00",
    "v4" = "#00FF00",
    "v5" = "#FF0000"
  )
```

Preparing the data for tess3Q\_map\_rasters

Make sure the lat longs are in the correct order and arrangement

```
df2 <- df1 |>
  dplyr::rename(
    Long = Longitude,
    Lat = Latitude
  )

long_lat_tibble <- df2 |>
  dplyr::select(Long, Lat)


long_lat_matrix <- long_lat_tibble |>
  as.matrix()

head(long_lat_matrix)
```

```
##         Long     Lat
## 159 127.9454 26.5013
## 160 127.9454 26.5013
## 161 127.9454 26.5013
## 162 127.9454 26.5013
## 163 127.9454 26.5013
## 164 127.9454 26.5013
```

Make a matrix of the Q values

Pull off the names of individuals and make a matrix of it:

```
Q_matrix <- leak5 |>
  dplyr::select(-ind, -pop, -index) |>
  as.matrix()
head(Q_matrix)
```

```
##               v1        v2          v3        v4       v5
## [1,] 0.004786660 0.1147900 0.110925000 0.1884980 0.581000
## [2,] 0.018223700 0.1249830 0.127298000 0.1617700 0.567725
## [3,] 0.027307100 0.0800896 0.037878500 0.1104490 0.744275
## [4,] 0.008272000 0.0895303 0.054886200 0.1273670 0.719945
## [5,] 0.017599600 0.0823412 0.064786500 0.0739732 0.761300
## [6,] 0.000099982 0.1700950 0.000099982 0.1164760 0.713230
```

Interpolate the Q-values by Kriging

```
print(ncol(Q_matrix) == length(color_palette2))
```

```
## [1] TRUE
```

Create brick

```
world <- ne_countries(scale = "medium", returnclass = "sf")
countries_with_data <- unique(df1$Country)

# Filtering the world data to include only the countries in your data
selected_countries <- world |>
  filter(admin %in% countries_with_data)

genoscape_brick <- tess3r::tess3Q_map_rasters(
  x = Q_matrix, 
  coord = long_lat_matrix,  
  map.polygon = selected_countries,
  window = extent(selected_countries)[1:4],
  # window = combined_extent,
  resolution = c(600,600), # if you want more cells in your raster, set higher
  # this next lines need to to be here, but don't do much...
  col.palette = tess3r::CreatePalette(color_palette2, length(color_palette2)),
  method = "map.max", 
  interpol = tess3r::FieldsKrigModel(80),  
  main = "Ancestry coefficients",
  xlab = "Longitude", 
  ylab = "Latitude", 
  cex = .4
)
```

```
## Warning: 
## Grid searches over lambda (nugget and sill variances) with  minima at the endpoints: 
##   (REML) Restricted maximum likelihood 
##    minimum at  right endpoint  lambda  =  0.02118404 (eff. df= 26.59999 )
## Warning: 
## Grid searches over lambda (nugget and sill variances) with  minima at the endpoints: 
##   (REML) Restricted maximum likelihood 
##    minimum at  right endpoint  lambda  =  0.02118404 (eff. df= 26.59999 )
## Warning: 
## Grid searches over lambda (nugget and sill variances) with  minima at the endpoints: 
##   (REML) Restricted maximum likelihood 
##    minimum at  right endpoint  lambda  =  0.02118404 (eff. df= 26.59999 )
## Warning: 
## Grid searches over lambda (nugget and sill variances) with  minima at the endpoints: 
##   (REML) Restricted maximum likelihood 
##    minimum at  right endpoint  lambda  =  0.02118404 (eff. df= 26.59999 )
```

```
# after that, we need to add names of the clusters back onto this raster brick
Q_tibble2 <- leak5 |>
  dplyr::select(
    -pop, -ind, -index
  )
names(genoscape_brick) <- names(Q_tibble2)[]
```

Scaling and cleaning the genoscape\_brick

```
genoscape_rgba <- genoscapeRtools::qprob_rando_raster(
  TRB = genoscape_brick,
  cols = color_palette2,
  alpha_scale = 2.0,
  abs_thresh = 0.0,
  alpha_exp = 1.55,
  alpha_chop_max = 255
)
 
# This adds the info for a regular lat-long projection
crs(genoscape_rgba) <- "+proj=longlat +datum=WGS84 +no_defs +ellps=WGS84 +towgs84=0,0,0"
```

Plot with pies

```
source(
  here(
    "scripts", "analysis", "my_theme2.R"
  )
)

# Calculate mean proportions for each population
df_mean <- df1 |>
  group_by(pop) |>
  summarise(across(starts_with("v"), \(x) mean(x, na.rm = TRUE)), 
            Longitude = mean(Longitude),
            Latitude = mean(Latitude))

ggplot() +
  layer_spatial(genoscape_rgba) +
  geom_spatial_point(data = long_lat_tibble,
                     mapping = aes(x = Long, y = Lat),
                     size = .2) +
  geom_text_repel(
    data = df_mean,
    aes(x = Longitude, y = Latitude, label = pop),
    size = 3,
    box.padding = unit(0.5, "lines")
  ) +
  labs(x = "Longitude",
       y = "Latitude") +
  geom_scatterpie(data = df_mean, 
                  aes(x = Longitude, y = Latitude, r = 1), 
                  cols = c("v1", "v2", "v3", "v4", "v5"), color = NA) +
  my_theme() +
  scale_fill_manual(values = color_palette2) +
  guides(fill = "none") +  # Hide legend
  coord_sf()
```

```
## Assuming `crs = 4326` in stat_spatial_identity()
```

```
# 
ggsave(
  here("output", "variant_proportions", "figures", "random9_map.pdf"),
  width  = 6,
  height = 6,
  units  = "in",
  device = cairo_pdf
)
```

```
## Assuming `crs = 4326` in stat_spatial_identity()
```

```
library(ggforce)


df_long <- df_mean |>
  gather(key = "segment", value = "value", v1:v5)

# Calculate pie segments for plotting
df_long <- df_long |>
  group_by(pop) |>
  arrange(pop, segment) |>
  mutate(cum_value = cumsum(value),
         total = sum(value),
         end_angle = 2 * pi * cum_value / total,
         start_angle = c(0, head(end_angle, -1))) |>
  ungroup()

# Adding a nudge value for the x-coordinate
nudge_x <- 1.1

ggplot() +
  geom_sf(
  data = world,
  fill = NA,
  color = "gray",
  lwd = 0.1,
  alpha = 0.3
  ) +
  layer_spatial(genoscape_rgba) +
  # geom_point(
  #   data = df_mean,
  #   aes(x = Longitude, y = Latitude),
  #   color = "black",
  #   size = .3
  # ) +
  ggforce::geom_arc_bar(
    data = df_long,
    aes(
      x0 = Longitude + nudge_x,
      y0 = Latitude,
      r0 = 0,
      r = 0.75,
      start = start_angle,
      end = end_angle,
      fill = segment
    ),
    color = NA
  ) +  # This will plot the pie segments without borders
  ggforce::geom_circle(
    data = df_mean,
    aes(x0 = Longitude+ nudge_x, 
        y0 = Latitude, 
        r = 0.75),
    inherit.aes = FALSE,
    color = "black",
    fill = NA,
    size = 0.1
  ) +  # This will plot the outer circle
  geom_point(
    data = df_mean,
    aes(x = Longitude, y = Latitude),
    color = "black",
    size = .3
  ) +
  geom_text_repel(
    data = df_mean,
    aes(x = Longitude, y = Latitude, label = pop),
    size = 2,
    box.padding = unit(.25, "lines"),
    direction = "both",
    max.iter = 2000,
    force = 1,
    max.overlaps = 50,
    nudge_x = 4,
    nudge_y = 0,
    segment.alpha = 0
  ) +
  labs(x = "Longitude",
       y = "Latitude") +
  my_theme() +
  scale_fill_manual(values = color_palette2) +
  guides(fill = "none") +
  coord_sf(xlim = c(60, 150), ylim = c(-10, 60))
```

```
ggsave(
  here("output", "variant_proportions", "figures", "new.pdf"),
  width  = 6,
  height = 6,
  units  = "in",
  device = cairo_pdf
)
```
