# Supplementary material for: A genotyping array for the globally invasive vector mosquito, Aedes albopictus
Source: Parasit Vectors. 2024 Mar 4;17:106. doi: 10.1186/s13071-024-06158-z (PMC10910840; doi:10.1186/s13071-024-06158-z)
Supplement: Supplementary file 15 — Additional file 15. Fst analysis. [file 13071_2024_6158_MOESM15_ESM.html]

Aedes albopictus SNP chip - FST estimates


# Aedes albopictus SNP chip - FST estimates

#### Luciano V Cosme

#### 2023-08-30

- Libraries
- 1. Intergenic
  SNPs
- 2. SNP set r2
  0.01
- 3. SNP set r2
  0.1

## Libraries

```
library(StAMPP)
library(ggplot2)
library(tidyverse)
library(adegenet)
library(here)
library(flextable)
library(officer)
library(reshape2)
```

We can use different data sets to run our fst estimates.

```
# Remove all objects from the environment
rm(list = ls())

# Run the garbage collector to free up memory
gc()
```

```
##           used  (Mb) gc trigger  (Mb) limit (Mb) max used  (Mb)
## Ncells 2702116 144.4    4737691 253.1         NA  4737691 253.1
## Vcells 4494502  34.3   10146329  77.5      32768  7645357  58.4
```

## 1. Intergenic SNPs

We can estimate Fst using only the neutral set of SNPs for
populations with at least 4 individuals.

Create list of populations

```
awk '{print $1}' output/populations/snps_sets/neutral.fam | sort | uniq -c | awk '{print $2, $1}' | awk '$2 >= 4 {print}' | awk '{print $1}' > output/fst/pops_4fst.txt;
head  output/fst/pops_4fst.txt;
wc -l output/fst/pops_4fst.txt
```

```
## BEN
## CAM
## CHA
## HAI
## HAN
## HOC
## HUN
## INJ
## INW
## KAC
##       25 output/fst/pops_4fst.txt
```

We have 25 populations with 4 or more mosquitoes. We can convert to
raw format and subset the bed file

```
plink \
--allow-extra-chr \
--keep-allele-order \
--bfile output/populations/snps_sets/neutral \
--keep-fam output/fst/pops_4fst.txt \
--recodeA \
--out output/fst/neutral \
--silent;
grep 'samples\|variants\|remaining' output/fst/neutral.log
```

```
## 9047 variants loaded from .bim file.
## --keep-fam: 230 people remaining.
## Total genotyping rate in remaining samples is 0.969394.
## 9047 variants and 230 people pass filters and QC.
```

Look at https://rdrr.io/cran/StAMPP/man/stamppFst.html for
details of Fst estimations

```
neutral <-
  read.PLINK(
    here(
      "output", "fst", "neutral.raw"
    ),
    quiet = FALSE,
    chunkSize = 1000,
    parallel = require("parallel"),
    n.cores = 4
  )

summary(neutral)
```

Now lets convert the genlight object to Stampp format, and estimate
pairwide Fst values

The command below would also work, but you can simplify it and put
only the numbers: genome\_equal\_2 <- stamppFst(neutral, nboots=100,
percent=95 + nclusters==10)

This chunk will take a couple minutes to run.

```
# convert
neutral_2 <- stamppConvert(neutral, type="genlight")

# run stampp. If you want to run with bootstraps and nclusters use the HPC. It will run out of memory on a 32Gb laptop
neutral_3 <- stamppFst(neutral_2, 1, 95, 1)
```

Save it

```
saveRDS(
  neutral_3, here(
    "output", "fst", "neutral.rds"
  )
)
```

To load it

```
neutral_3 <- readRDS(
  here(
    "output", "fst", "neutral.rds"
  )
)
```

Now lets look at the object

```
summary(neutral_3)
```

```
##       OKI               HAI               YUN                 KAT         
##  Min.   :0.06693   Min.   :0.02807   Min.   :-0.002161   Min.   :0.06445  
##  1st Qu.:0.08088   1st Qu.:0.03572   1st Qu.: 0.017006   1st Qu.:0.07817  
##  Median :0.09944   Median :0.05998   Median : 0.055814   Median :0.09368  
##  Mean   :0.11276   Mean   :0.06803   Mean   : 0.056186   Mean   :0.11228  
##  3rd Qu.:0.14177   3rd Qu.:0.09611   3rd Qu.: 0.082102   3rd Qu.:0.13943  
##  Max.   :0.21810   Max.   :0.15999   Max.   : 0.143292   Max.   :0.21573  
##  NA's   :1         NA's   :2         NA's   :3           NA's   :4        
##       TAI              HUN               HAN               KLP         
##  Min.   :0.1233   Min.   :0.03590   Min.   :0.03056   Min.   :0.04714  
##  1st Qu.:0.1495   1st Qu.:0.04296   1st Qu.:0.04201   1st Qu.:0.05136  
##  Median :0.1642   Median :0.05908   Median :0.08134   Median :0.10614  
##  Mean   :0.1780   Mean   :0.07034   Mean   :0.08233   Mean   :0.10225  
##  3rd Qu.:0.2007   3rd Qu.:0.09196   3rd Qu.:0.11386   3rd Qu.:0.12868  
##  Max.   :0.3004   Max.   :0.16409   Max.   :0.19157   Max.   :0.21872  
##  NA's   :5        NA's   :6         NA's   :7         NA's   :8        
##       HOC               QNC               SSK                KAC           
##  Min.   :0.03383   Min.   :0.07153   Min.   :0.000295   Min.   :-0.000224  
##  1st Qu.:0.04598   1st Qu.:0.07691   1st Qu.:0.010202   1st Qu.: 0.014289  
##  Median :0.07343   Median :0.13239   Median :0.069284   Median : 0.075309  
##  Mean   :0.08110   Mean   :0.12117   Mean   :0.056040   Mean   : 0.060492  
##  3rd Qu.:0.10792   3rd Qu.:0.14787   3rd Qu.:0.083976   3rd Qu.: 0.085694  
##  Max.   :0.17718   Max.   :0.21538   Max.   :0.134878   Max.   : 0.145295  
##  NA's   :9         NA's   :10        NA's   :11         NA's   :12         
##       CHA                KAN               UTS               CAM          
##  Min.   :0.003151   Min.   :0.09212   Min.   :0.07893   Min.   :0.008014  
##  1st Qu.:0.017775   1st Qu.:0.11198   1st Qu.:0.09267   1st Qu.:0.019739  
##  Median :0.077002   Median :0.12201   Median :0.10527   Median :0.070429  
##  Mean   :0.063989   Mean   :0.14434   Mean   :0.12345   Mean   :0.064043  
##  3rd Qu.:0.087944   3rd Qu.:0.16900   3rd Qu.:0.14159   3rd Qu.:0.081284  
##  Max.   :0.131817   Max.   :0.23897   Max.   :0.21422   Max.   :0.135431  
##  NA's   :13         NA's   :14        NA's   :15        NA's   :16        
##       KAG               BEN               LAM               SUF         
##  Min.   :0.08122   Min.   :0.02333   Min.   :0.01692   Min.   :0.03162  
##  1st Qu.:0.09061   1st Qu.:0.05390   1st Qu.:0.06908   1st Qu.:0.06323  
##  Median :0.12139   Median :0.08997   Median :0.08394   Median :0.06969  
##  Mean   :0.12750   Mean   :0.07831   Mean   :0.08446   Mean   :0.09810  
##  3rd Qu.:0.14741   3rd Qu.:0.10404   3rd Qu.:0.10618   3rd Qu.:0.09388  
##  Max.   :0.20375   Max.   :0.11899   Max.   :0.14422   Max.   :0.23208  
##  NA's   :17        NA's   :18        NA's   :19        NA's   :20       
##       SUU               KUN              INW               INJ         
##  Min.   :0.06603   Min.   :0.1486   Min.   :0.06648   Min.   :0.08835  
##  1st Qu.:0.07088   1st Qu.:0.1799   1st Qu.:0.08016   1st Qu.:0.08835  
##  Median :0.08605   Median :0.2113   Median :0.09384   Median :0.08835  
##  Mean   :0.11206   Mean   :0.2123   Mean   :0.09384   Mean   :0.08835  
##  3rd Qu.:0.12723   3rd Qu.:0.2441   3rd Qu.:0.10751   3rd Qu.:0.08835  
##  Max.   :0.21012   Max.   :0.2769   Max.   :0.12119   Max.   :0.08835  
##  NA's   :21        NA's   :22       NA's   :23        NA's   :24       
##       MAT     
##  Min.   : NA  
##  1st Qu.: NA  
##  Median : NA  
##  Mean   :NaN  
##  3rd Qu.: NA  
##  Max.   : NA  
##  NA's   :25
```

If you want you can save the fst values as csv.

```
# Convert to data frame
neutral_df <- data.frame(neutral_3)

# Save it
write.csv(neutral_df, file = here("output", "fst", "neutral_df.csv"))
```

Check the Fst values

```
head(neutral_df)
```

```
##            OKI        HAI        YUN       KAT       TAI HUN HAN KLP HOC QNC
## OKI         NA         NA         NA        NA        NA  NA  NA  NA  NA  NA
## HAI 0.07268706         NA         NA        NA        NA  NA  NA  NA  NA  NA
## YUN 0.07937617 0.03397451         NA        NA        NA  NA  NA  NA  NA  NA
## KAT 0.14886465 0.10179042 0.07762791        NA        NA  NA  NA  NA  NA  NA
## TAI 0.15614563 0.12689237 0.14329227 0.2157330        NA  NA  NA  NA  NA  NA
## HUN 0.06693096 0.02807325 0.04026851 0.1034478 0.1233325  NA  NA  NA  NA  NA
##     SSK KAC CHA KAN UTS CAM KAG BEN LAM SUF SUU KUN INW INJ MAT
## OKI  NA  NA  NA  NA  NA  NA  NA  NA  NA  NA  NA  NA  NA  NA  NA
## HAI  NA  NA  NA  NA  NA  NA  NA  NA  NA  NA  NA  NA  NA  NA  NA
## YUN  NA  NA  NA  NA  NA  NA  NA  NA  NA  NA  NA  NA  NA  NA  NA
## KAT  NA  NA  NA  NA  NA  NA  NA  NA  NA  NA  NA  NA  NA  NA  NA
## TAI  NA  NA  NA  NA  NA  NA  NA  NA  NA  NA  NA  NA  NA  NA  NA
## HUN  NA  NA  NA  NA  NA  NA  NA  NA  NA  NA  NA  NA  NA  NA  NA
```

We will convert the data into a matrix.

```
aa <- as.matrix(neutral_df)
aa[upper.tri(aa)] <- t(aa)[upper.tri(t(aa))]
head(aa)
```

```
##            OKI        HAI        YUN        KAT       TAI        HUN        HAN
## OKI         NA 0.07268706 0.07937617 0.14886465 0.1561456 0.06693096 0.09294157
## HAI 0.07268706         NA 0.03397451 0.10179042 0.1268924 0.02807325 0.03807541
## YUN 0.07937617 0.03397451         NA 0.07762791 0.1432923 0.04026851 0.03421635
## KAT 0.14886465 0.10179042 0.07762791         NA 0.2157330 0.10344776 0.11088115
## TAI 0.15614563 0.12689237 0.14329227 0.21573302        NA 0.12333248 0.16471928
## HUN 0.06693096 0.02807325 0.04026851 0.10344776 0.1233325         NA 0.04385787
##            KLP        HOC        QNC         SSK          KAC          CHA
## OKI 0.12753263 0.07966691 0.14381991 0.080917127  0.082764633 0.0807573335
## HAI 0.07062727 0.02857376 0.09737992 0.036399112  0.031226075 0.0350480370
## YUN 0.04892423 0.03983731 0.07317901 0.001820884 -0.002160858 0.0007563386
## KAT 0.12941018 0.11252413 0.14504693 0.078173740  0.078066760 0.0766586019
## TAI 0.20155700 0.14637599 0.20875730 0.142121310  0.152625899 0.1397690667
## HUN 0.07440170 0.03618065 0.10249995 0.042059268  0.038117472 0.0413984260
##            KAN        UTS         CAM        KAG        BEN        LAM
## OKI 0.13456942 0.10960357 0.075330223 0.10320709 0.09568234 0.08756987
## HAI 0.09711038 0.07278214 0.030013229 0.06963227 0.04835553 0.03913709
## YUN 0.10828863 0.08297531 0.002435562 0.07597810 0.01756076 0.00724616
## KAT 0.17428707 0.14702796 0.075728451 0.13942968 0.08425904 0.08100140
## TAI 0.19294422 0.17076422 0.136334355 0.16363753 0.15654314 0.15057531
## HUN 0.08888333 0.06669664 0.035903383 0.05908263 0.05334759 0.04549865
##            SUF        SUU       KUN        INW        INJ        MAT
## OKI 0.14108869 0.11063606 0.2181045 0.18145357 0.14398625 0.09257954
## HAI 0.09101011 0.05998414 0.1599904 0.12872924 0.09510255 0.04482997
## YUN 0.08325162 0.06270272 0.1364487 0.10513394 0.07948148 0.01682092
## KAT 0.06444802 0.06708021 0.2034093 0.09367875 0.08599529 0.09162392
## TAI 0.21266171 0.17599097 0.3003592 0.26676013 0.20046117 0.15440513
## HUN 0.09503274 0.06400679 0.1640872 0.13267018 0.09920459 0.05348803
```

Import sample locations

```
sampling_loc <- readRDS(here("output", "populations", "sampling_loc.rds"))

# Create a named vector to map countries to regions
country_to_region <- c(
  "Bhutan" = "South Asia",
  "Cambodia" = "Southeast Asia",
  "China" = "East Asia",
  "India" = "South Asia",
  "Indonesia" = "Southeast Asia",
  "Japan" = "East Asia",
  "Malaysia" = "Southeast Asia",
  "Maldives" = "South Asia",
  "Nepal" = "South Asia",
  "Sri Lanka" = "South Asia",
  "Taiwan" = "East Asia",
  "Thailand" = "Southeast Asia",
  "Vietnam" = "Southeast Asia"
)

# Add the region to the data frame
sampling_loc$Region2 <- country_to_region[sampling_loc$Country]

# Arrange by region 
sampling_loc <- sampling_loc |>
  dplyr::arrange(
    Region2, Country
  )

# Check it
head(sampling_loc)
```

```
## # A tibble: 6 × 7
##   Pop_City Country Latitude Longitude Region Abbreviation Region2  
##   <chr>    <chr>      <dbl>     <dbl> <chr>  <chr>        <chr>    
## 1 Hainan   China       19.2      110. Asia   HAI          East Asia
## 2 Yunnan   China       24.5      101. Asia   YUN          East Asia
## 3 Hunan    China       27.6      112. Asia   HUN          East Asia
## 4 Okinawa  Japan       26.5      128. Asia   OKI          East Asia
## 5 Sendai   Japan       38.3      141. Asia   SEN          East Asia
## 6 Nagasaki Japan       32.8      130. Asia   NAG          East Asia
```

Order

```
order_pops <- as.vector(sampling_loc$Abbreviation)
order_pops
```

```
##  [1] "HAI" "YUN" "HUN" "OKI" "SEN" "NAG" "SAK" "HIR" "KAN" "YAT" "KYO" "NIG"
## [13] "UTS" "AIZ" "KHO" "SAG" "KAG" "JAT" "TAN" "TAI" "GEL" "BEN" "KUN" "KAT"
## [25] "JAF" "CAM" "SUF" "SUU" "INW" "INJ" "KLP" "MAT" "SSK" "KAC" "SON" "CHA"
## [37] "LAM" "HAN" "HOC" "QNC" "ALV" "POR" "ANT" "MAD" "AWK" "TIK" "BAR" "SAI"
## [49] "PAL" "LOS"
```

Create vector with order of populations

```
# Extract the populations that appear in neutral_df
populations_in_neutral <- colnames(neutral_df)

# Reorder the populations based on order_pops
poporder <- populations_in_neutral[populations_in_neutral %in% order_pops]

# Print the reordered populations
print(poporder)
```

```
##  [1] "OKI" "HAI" "YUN" "KAT" "TAI" "HUN" "HAN" "KLP" "HOC" "QNC" "SSK" "KAC"
## [13] "CHA" "KAN" "UTS" "CAM" "KAG" "BEN" "LAM" "SUF" "SUU" "KUN" "INW" "INJ"
## [25] "MAT"
```

Lets check if the matrix is symmetric.

```
isSymmetric(aa)
```

```
## [1] TRUE
```

Now lets order the matrix using poporder. We will also add NA on the
upper left side of the matrix.

```
aa <- aa[poporder, poporder]
aa[lower.tri(aa)] <- NA
```

Now we have to convert the matrix to a data frame to plot it with
ggplot.

```
pairfst.long <- melt(aa)
summary(pairfst.long)
```

```
##       Var1          Var2         value        
##  OKI    : 25   OKI    : 25   Min.   :-0.0022  
##  HAI    : 25   HAI    : 25   1st Qu.: 0.0589  
##  YUN    : 25   YUN    : 25   Median : 0.0871  
##  KAT    : 25   KAT    : 25   Mean   : 0.0959  
##  TAI    : 25   TAI    : 25   3rd Qu.: 0.1325  
##  HUN    : 25   HUN    : 25   Max.   : 0.3004  
##  (Other):475   (Other):475   NA's   :325
```

Now lets plot the data with ggplot. You can click in the little
square on the top left of the plot to open it on a new window. It will
have the right proportions.

```
pairfst.f <- ggplot(pairfst.long, aes(Var1, Var2)) +
  geom_tile(aes(fill = value), colour = "white") +
  scale_fill_gradient(
    low = "white",
    high = "#71b6ff",
    name = "Fst",
    na.value = "white",
    limits = c(0, 0.5)
  ) +
  scale_x_discrete(position = "top") +
  theme_bw() +
  geom_text(aes(label = ifelse(
    is.na(value), "", formatC(value, digits = 2, format = "f")
  )), size = 3) +
  theme(
    axis.text.x = element_text(angle = 90, hjust = 1),
    axis.title = element_blank(),
    panel.grid.major = element_blank(),
    panel.grid.minor = element_blank(),
    panel.border = element_blank(),
    panel.background = element_blank(),
    axis.text.y = element_text(hjust = 0)
  )
pairfst.f
```

```
ggsave(
  filename = here("output", "fst", "fst_matrix_neutral.pdf"),
  pairfst.f,
  width = 10,
  height = 10,
  units = "in"
)
```

Remove NAs and rename columns

```
# remove NAs
fst2 <-
  pairfst.long |>
  drop_na()

# rename columns
fst2 <-
  fst2 |>
  dplyr::rename(pop1 = 1,
         pop2 = 2,
         fst  = 3)


# Split the data into two data frames, one for pop1 and one for pop2
df_pop1 <- fst2 |>
  dplyr::select(pop = pop1, fst)
df_pop2 <- fst2 |>
  dplyr::select(pop = pop2, fst)

# Combine the two data frames
df_combined <- bind_rows(df_pop1, df_pop2)

# Calculate the mean fst for each population
mean_fst <- df_combined |>
  group_by(pop) |>
  summarise(mean_fst = mean(fst))

print(mean_fst)
```

```
## # A tibble: 25 × 2
##    pop   mean_fst
##    <fct>    <dbl>
##  1 OKI     0.113 
##  2 HAI     0.0682
##  3 YUN     0.0562
##  4 KAT     0.112 
##  5 TAI     0.175 
##  6 HUN     0.0708
##  7 HAN     0.0819
##  8 KLP     0.103 
##  9 HOC     0.0777
## 10 QNC     0.123 
## # ℹ 15 more rows
```

Merge

```
fst3 <-
  sampling_loc |>
  left_join(
    mean_fst,
    by = c("Abbreviation" = "pop")
  ) |>
  drop_na() |>
  dplyr::select(
    -Region
  )

# Remove " Asia" from the Region2 column
fst3$Region2 <- gsub(" Asia", "", fst3$Region2)

# Rename the Region2 column to Region
fst3 <- fst3 |>
  dplyr::rename(Region = Region2)

# check output
head(fst3)
```

```
## # A tibble: 6 × 7
##   Pop_City   Country Latitude Longitude Abbreviation Region mean_fst
##   <chr>      <chr>      <dbl>     <dbl> <chr>        <chr>     <dbl>
## 1 Hainan     China       19.2      110. HAI          East     0.0682
## 2 Yunnan     China       24.5      101. YUN          East     0.0562
## 3 Hunan      China       27.6      112. HUN          East     0.0708
## 4 Okinawa    Japan       26.5      128. OKI          East     0.113 
## 5 Kanazawa   Japan       36.6      137. KAN          East     0.136 
## 6 Utsunomiya Japan       36.6      140. UTS          East     0.112
```

Mean by region

```
# Group by Region and calculate the mean_fst by Region
region_means <- fst3 |>
  group_by(Region) |>
  summarize(mean_fst_by_region = round(mean(mean_fst, na.rm = TRUE), 2)) |>
  ungroup()  # Ungroup the data

# Add the mean_fst_by_region column to the fst3 tibble
fst3 <- fst3 |>
  left_join(region_means, by = "Region")

# Print the modified fst3 tibble
print(fst3)
```

```
## # A tibble: 25 × 8
##    Pop_City    Country  Latitude Longitude Abbreviation Region mean_fst
##    <chr>       <chr>       <dbl>     <dbl> <chr>        <chr>     <dbl>
##  1 Hainan      China       19.2      110.  HAI          East     0.0682
##  2 Yunnan      China       24.5      101.  YUN          East     0.0562
##  3 Hunan       China       27.6      112.  HUN          East     0.0708
##  4 Okinawa     Japan       26.5      128.  OKI          East     0.113 
##  5 Kanazawa    Japan       36.6      137.  KAN          East     0.136 
##  6 Utsunomiya  Japan       36.6      140.  UTS          East     0.112 
##  7 Kagoshima   Japan       31.6      131.  KAG          East     0.106 
##  8 Tainan      Taiwan      23.0      120.  TAI          East     0.175 
##  9 Bengaluru   India       13.0       77.6 BEN          South    0.0689
## 10 Kunfunadhoo Maldives     5.67      73   KUN          South    0.189 
## # ℹ 15 more rows
## # ℹ 1 more variable: mean_fst_by_region <dbl>
```

Mean by country

```
# Group by Country and calculate the mean_fst by Country
country_means <- fst3 |>
  group_by(Country) |>
  summarize(mean_fst_by_country = round(mean(mean_fst, na.rm = TRUE), 2)) |>
  ungroup()  # Ungroup the data

# Add the mean_fst_by_country column to the fst3 tibble
fst3 <- fst3 |>
  left_join(country_means, by = "Country")

# Print the modified fst3 tibble
print(fst3)
```

```
## # A tibble: 25 × 9
##    Pop_City    Country  Latitude Longitude Abbreviation Region mean_fst
##    <chr>       <chr>       <dbl>     <dbl> <chr>        <chr>     <dbl>
##  1 Hainan      China       19.2      110.  HAI          East     0.0682
##  2 Yunnan      China       24.5      101.  YUN          East     0.0562
##  3 Hunan       China       27.6      112.  HUN          East     0.0708
##  4 Okinawa     Japan       26.5      128.  OKI          East     0.113 
##  5 Kanazawa    Japan       36.6      137.  KAN          East     0.136 
##  6 Utsunomiya  Japan       36.6      140.  UTS          East     0.112 
##  7 Kagoshima   Japan       31.6      131.  KAG          East     0.106 
##  8 Tainan      Taiwan      23.0      120.  TAI          East     0.175 
##  9 Bengaluru   India       13.0       77.6 BEN          South    0.0689
## 10 Kunfunadhoo Maldives     5.67      73   KUN          South    0.189 
## # ℹ 15 more rows
## # ℹ 2 more variables: mean_fst_by_region <dbl>, mean_fst_by_country <dbl>
```

Mean by latitude

```
# Add a new column to indicate whether the latitude is above or below 30N
fst3 <- fst3 |>
  mutate(Latitude_group = ifelse(Latitude >= 30, "Above 30N", "Below 30N"))

# Summarize the data by Latitude_group and calculate the mean_fst
summary_by_latitude <- fst3 |>
  group_by(Latitude_group) |>
  summarize(mean_fst_by_latitude = mean(mean_fst, na.rm = TRUE)) |>
  ungroup()  # Ungroup the data

# Add the mean_fst_by_latitude column to the fst3 tibble
fst3 <- fst3 |>
  left_join(summary_by_latitude, by = "Latitude_group")


# Rename columns
fst3 <- fst3 |>
  dplyr::rename(
    City = Pop_City
  )

# Print the modified fst3 tibble
print(fst3)
```

```
## # A tibble: 25 × 11
##    City        Country  Latitude Longitude Abbreviation Region mean_fst
##    <chr>       <chr>       <dbl>     <dbl> <chr>        <chr>     <dbl>
##  1 Hainan      China       19.2      110.  HAI          East     0.0682
##  2 Yunnan      China       24.5      101.  YUN          East     0.0562
##  3 Hunan       China       27.6      112.  HUN          East     0.0708
##  4 Okinawa     Japan       26.5      128.  OKI          East     0.113 
##  5 Kanazawa    Japan       36.6      137.  KAN          East     0.136 
##  6 Utsunomiya  Japan       36.6      140.  UTS          East     0.112 
##  7 Kagoshima   Japan       31.6      131.  KAG          East     0.106 
##  8 Tainan      Taiwan      23.0      120.  TAI          East     0.175 
##  9 Bengaluru   India       13.0       77.6 BEN          South    0.0689
## 10 Kunfunadhoo Maldives     5.67      73   KUN          South    0.189 
## # ℹ 15 more rows
## # ℹ 4 more variables: mean_fst_by_region <dbl>, mean_fst_by_country <dbl>,
## #   Latitude_group <chr>, mean_fst_by_latitude <dbl>
```

```
fst4 <- fst3 |>
  dplyr::select(
    Latitude_group, mean_fst_by_latitude, Region, mean_fst_by_region, Country, mean_fst_by_country, City, Abbreviation, mean_fst,
  )

fst4 <- fst4 |>
  arrange(
    Latitude_group, Region, Country, City
  )

# Round
fst4 <- fst4 |>
  mutate_if(is.numeric, ~ round(., 2))

head(fst4)
```

```
## # A tibble: 6 × 9
##   Latitude_group mean_fst_by_latitude Region mean_fst_by_region Country
##   <chr>                         <dbl> <chr>               <dbl> <chr>  
## 1 Above 30N                      0.12 East                  0.1 Japan  
## 2 Above 30N                      0.12 East                  0.1 Japan  
## 3 Above 30N                      0.12 East                  0.1 Japan  
## 4 Below 30N                      0.09 East                  0.1 China  
## 5 Below 30N                      0.09 East                  0.1 China  
## 6 Below 30N                      0.09 East                  0.1 China  
## # ℹ 4 more variables: mean_fst_by_country <dbl>, City <chr>,
## #   Abbreviation <chr>, mean_fst <dbl>
```

```
# Set theme if you want to use something different from the previous table
set_flextable_defaults(
  font.family = "Arial",
  font.size = 9,
  big.mark = ",",
  theme_fun = "theme_zebra" # try the themes: theme_alafoli(), theme_apa(), theme_booktabs(), theme_box(), theme_tron_legacy(), theme_tron(), theme_vader(), theme_vanilla(), theme_zebra()
)

# Then create the flextable object
flex_table <- flextable(fst4) |>
  set_caption(caption = as_paragraph(
    as_chunk(
      "Table 1. Fst values using intergenic SNPs.",
      props = fp_text_default(color = "#000000", font.size = 14)
    )
  ),
  fp_p = fp_par(text.align = "center", padding = 5))

# Print the flextable
flex_table
```

Table 1. Fst values using intergenic SNPs.

| Latitude\_group | mean\_fst\_by\_latitude | Region | mean\_fst\_by\_region | Country | mean\_fst\_by\_country | City | Abbreviation | mean\_fst |
| --- | --- | --- | --- | --- | --- | --- | --- | --- |
| Above 30N | 0.12 | East | 0.10 | Japan | 0.12 | Kagoshima | KAG | 0.11 |
| Above 30N | 0.12 | East | 0.10 | Japan | 0.12 | Kanazawa | KAN | 0.14 |
| Above 30N | 0.12 | East | 0.10 | Japan | 0.12 | Utsunomiya | UTS | 0.11 |
| Below 30N | 0.09 | East | 0.10 | China | 0.07 | Hainan | HAI | 0.07 |
| Below 30N | 0.09 | East | 0.10 | China | 0.07 | Hunan | HUN | 0.07 |
| Below 30N | 0.09 | East | 0.10 | China | 0.07 | Yunnan | YUN | 0.06 |
| Below 30N | 0.09 | East | 0.10 | Japan | 0.12 | Okinawa | OKI | 0.11 |
| Below 30N | 0.09 | East | 0.10 | Taiwan | 0.18 | Tainan | TAI | 0.18 |
| Below 30N | 0.09 | South | 0.12 | India | 0.07 | Bengaluru | BEN | 0.07 |
| Below 30N | 0.09 | South | 0.12 | Maldives | 0.19 | Kunfunadhoo | KUN | 0.19 |
| Below 30N | 0.09 | South | 0.12 | Nepal | 0.11 | Kathmandu | KAT | 0.11 |
| Below 30N | 0.09 | Southeast | 0.09 | Cambodia | 0.05 | Phnom Penh | CAM | 0.05 |
| Below 30N | 0.09 | Southeast | 0.09 | Indonesia | 0.11 | Jakarta | INJ | 0.11 |
| Below 30N | 0.09 | Southeast | 0.09 | Indonesia | 0.11 | Sulawesi (Forest) | SUF | 0.11 |
| Below 30N | 0.09 | Southeast | 0.09 | Indonesia | 0.11 | Sulawesi (Urban) | SUU | 0.09 |
| Below 30N | 0.09 | Southeast | 0.09 | Indonesia | 0.11 | Wainyapu | INW | 0.14 |
| Below 30N | 0.09 | Southeast | 0.09 | Malaysia | 0.09 | Kuala Lumpur | KLP | 0.10 |
| Below 30N | 0.09 | Southeast | 0.09 | Malaysia | 0.09 | Tambun | MAT | 0.07 |
| Below 30N | 0.09 | Southeast | 0.09 | Thailand | 0.06 | Chanthaburi | CHA | 0.06 |
| Below 30N | 0.09 | Southeast | 0.09 | Thailand | 0.06 | Kanchanaburi | KAC | 0.06 |
| Below 30N | 0.09 | Southeast | 0.09 | Thailand | 0.06 | Lampang | LAM | 0.06 |
| Below 30N | 0.09 | Southeast | 0.09 | Thailand | 0.06 | Sisaket | SSK | 0.06 |
| Below 30N | 0.09 | Southeast | 0.09 | Vietnam | 0.09 | Hanoi | HAN | 0.08 |
| Below 30N | 0.09 | Southeast | 0.09 | Vietnam | 0.09 | Ho Chi Minh City | HOC | 0.08 |
| Below 30N | 0.09 | Southeast | 0.09 | Vietnam | 0.09 | Quy Nhon City | QNC | 0.12 |

```
# Initialize Word document
doc <- 
  read_docx() |>
  body_add_flextable(value = flex_table)

# Define the output path with 'here' library
output_path <- here(
  "output",
  "fst", 
  "fst_neutral_SNPS.docx"
  )

# Save the Word document
print(doc, target = output_path)
```

## 2. SNP set r2 0.01

```
# Remove all objects from the environment
rm(list = ls())

# Run the garbage collector to free up memory
gc()
```

```
##           used  (Mb) gc trigger  (Mb) limit (Mb) max used  (Mb)
## Ncells 3099303 165.6    4737691 253.1         NA  4737691 253.1
## Vcells 5373137  41.0   10146329  77.5      32768  9693704  74.0
```

```
plink \
--allow-extra-chr \
--keep-allele-order \
--bfile output/populations/snps_sets/r2_0.01 \
--keep-fam output/fst/pops_4fst.txt \
--recodeA \
--out output/fst/r2_0.01 \
--silent;
grep 'samples\|variants\|remaining' output/fst/r2_0.01.log
```

```
## 20931 variants loaded from .bim file.
## --keep-fam: 230 people remaining.
## Total genotyping rate in remaining samples is 0.969803.
## 20931 variants and 230 people pass filters and QC.
```

Look at https://rdrr.io/cran/StAMPP/man/stamppFst.html for
details of Fst estimations

```
r2_0.01 <-
  read.PLINK(
    here(
      "output", "fst", "r2_0.01.raw"
    ),
    quiet = FALSE,
    chunkSize = 1000,
    parallel = require("parallel"),
    n.cores = 4
  )

summary(r2_0.01)
```

This chunk will take a couple minutes to run.

```
# convert
r2_0.01_2 <- stamppConvert(r2_0.01, type="genlight")

# run stampp. If you want to runn with bootstraps and nclusters use the HPC. It will run out of memory on a 32Gb laptop
r2_0.01_3 <- stamppFst(r2_0.01_2, 1, 95, 1)
```

Save it

```
saveRDS(
  r2_0.01_3, here(
    "output", "fst", "r2_0.01.rds"
  )
)
```

To load it

```
r2_0.01_3 <- readRDS(
  here(
    "output", "fst", "r2_0.01.rds"
  )
)
```

Now lets look at the object

```
summary(r2_0.01_3)
```

```
##       OKI               HAI               YUN                  KAT         
##  Min.   :0.07411   Min.   :0.03305   Min.   :-0.0000684   Min.   :0.05983  
##  1st Qu.:0.12457   1st Qu.:0.05357   1st Qu.: 0.0202758   1st Qu.:0.09938  
##  Median :0.13500   Median :0.08638   Median : 0.0789742   Median :0.11057  
##  Mean   :0.15247   Mean   :0.09338   Mean   : 0.0821429   Mean   :0.14004  
##  3rd Qu.:0.17245   3rd Qu.:0.13393   3rd Qu.: 0.1383007   3rd Qu.:0.17033  
##  Max.   :0.27404   Max.   :0.19267   Max.   : 0.1838969   Max.   :0.25614  
##  NA's   :1         NA's   :2         NA's   :3            NA's   :4        
##       TAI              HUN               HAN               KLP         
##  Min.   :0.1293   Min.   :0.04315   Min.   :0.03769   Min.   :0.05066  
##  1st Qu.:0.1818   1st Qu.:0.07836   1st Qu.:0.05082   1st Qu.:0.06001  
##  Median :0.1963   Median :0.08788   Median :0.09712   Median :0.13557  
##  Mean   :0.2157   Mean   :0.10679   Mean   :0.10658   Mean   :0.13089  
##  3rd Qu.:0.2423   3rd Qu.:0.12706   3rd Qu.:0.15227   3rd Qu.:0.19086  
##  Max.   :0.3508   Max.   :0.21464   Max.   :0.22468   Max.   :0.23994  
##  NA's   :5        NA's   :6         NA's   :7         NA's   :8        
##       HOC               QNC               SSK                KAC          
##  Min.   :0.04799   Min.   :0.07995   Min.   :0.003314   Min.   :0.001008  
##  1st Qu.:0.06683   1st Qu.:0.08570   1st Qu.:0.011973   1st Qu.:0.018685  
##  Median :0.10139   Median :0.17159   Median :0.110002   Median :0.127829  
##  Mean   :0.11328   Mean   :0.15440   Mean   :0.086747   Mean   :0.096343  
##  3rd Qu.:0.15323   3rd Qu.:0.21452   3rd Qu.:0.148341   3rd Qu.:0.161847  
##  Max.   :0.21621   Max.   :0.24560   Max.   :0.182914   Max.   :0.191394  
##  NA's   :9         NA's   :10        NA's   :11         NA's   :12        
##       CHA                KAN              UTS               CAM          
##  Min.   :0.003687   Min.   :0.1015   Min.   :0.08843   Min.   :0.008604  
##  1st Qu.:0.023862   1st Qu.:0.1789   1st Qu.:0.16593   1st Qu.:0.026054  
##  Median :0.125757   Median :0.1975   Median :0.17580   Median :0.118910  
##  Mean   :0.097930   Mean   :0.2137   Mean   :0.19727   Mean   :0.091140  
##  3rd Qu.:0.149282   3rd Qu.:0.2658   3rd Qu.:0.23861   3rd Qu.:0.127924  
##  Max.   :0.179347   Max.   :0.3277   Max.   :0.29881   Max.   :0.153901  
##  NA's   :13         NA's   :14       NA's   :15        NA's   :16        
##       KAG              BEN               LAM               SUF         
##  Min.   :0.1453   Min.   :0.02933   Min.   :0.01885   Min.   :0.03927  
##  1st Qu.:0.1535   1st Qu.:0.07534   1st Qu.:0.10804   1st Qu.:0.08085  
##  Median :0.1918   Median :0.13014   Median :0.13373   Median :0.09416  
##  Mean   :0.1996   Mean   :0.10628   Mean   :0.11948   Mean   :0.12977  
##  3rd Qu.:0.2348   3rd Qu.:0.13699   3rd Qu.:0.15615   3rd Qu.:0.14373  
##  Max.   :0.2757   Max.   :0.15984   Max.   :0.16749   Max.   :0.29085  
##  NA's   :17       NA's   :18        NA's   :19        NA's   :20       
##       SUU               KUN              INW               INJ        
##  Min.   :0.08632   Min.   :0.1695   Min.   :0.06375   Min.   :0.1298  
##  1st Qu.:0.09984   1st Qu.:0.2147   1st Qu.:0.08902   1st Qu.:0.1298  
##  Median :0.11444   Median :0.2599   Median :0.11429   Median :0.1298  
##  Mean   :0.14125   Mean   :0.2548   Mean   :0.11429   Mean   :0.1298  
##  3rd Qu.:0.15586   3rd Qu.:0.2974   3rd Qu.:0.13955   3rd Qu.:0.1298  
##  Max.   :0.24981   Max.   :0.3349   Max.   :0.16482   Max.   :0.1298  
##  NA's   :21        NA's   :22       NA's   :23        NA's   :24      
##       MAT     
##  Min.   : NA  
##  1st Qu.: NA  
##  Median : NA  
##  Mean   :NaN  
##  3rd Qu.: NA  
##  Max.   : NA  
##  NA's   :25
```

If you want you can save the fst values as csv.

```
# Convert to data frame
r2_0.01_df <- data.frame(r2_0.01_3)

# Save it
write.csv(r2_0.01_df, file = here("output", "fst", "r2_0.01_df.csv"))
```

Check the Fst values

```
head(r2_0.01_df)
```

```
##            OKI        HAI        YUN       KAT       TAI HUN HAN KLP HOC QNC
## OKI         NA         NA         NA        NA        NA  NA  NA  NA  NA  NA
## HAI 0.08801929         NA         NA        NA        NA  NA  NA  NA  NA  NA
## YUN 0.12717041 0.05389545         NA        NA        NA  NA  NA  NA  NA  NA
## KAT 0.19706597 0.13069201 0.09803865        NA        NA  NA  NA  NA  NA  NA
## TAI 0.16437593 0.13725088 0.18359315 0.2561412        NA  NA  NA  NA  NA  NA
## HUN 0.07411078 0.03611331 0.07708983 0.1485081 0.1292503  NA  NA  NA  NA  NA
##     SSK KAC CHA KAN UTS CAM KAG BEN LAM SUF SUU KUN INW INJ MAT
## OKI  NA  NA  NA  NA  NA  NA  NA  NA  NA  NA  NA  NA  NA  NA  NA
## HAI  NA  NA  NA  NA  NA  NA  NA  NA  NA  NA  NA  NA  NA  NA  NA
## YUN  NA  NA  NA  NA  NA  NA  NA  NA  NA  NA  NA  NA  NA  NA  NA
## KAT  NA  NA  NA  NA  NA  NA  NA  NA  NA  NA  NA  NA  NA  NA  NA
## TAI  NA  NA  NA  NA  NA  NA  NA  NA  NA  NA  NA  NA  NA  NA  NA
## HUN  NA  NA  NA  NA  NA  NA  NA  NA  NA  NA  NA  NA  NA  NA  NA
```

Now lets get the Fst values from the object albo3. It has the
bootstraps, CI limits, p-values, and Fst values. We will convert the
data into a matrix.

```
aa <- as.matrix(r2_0.01_df)
aa[upper.tri(aa)] <- t(aa)[upper.tri(t(aa))]
head(aa)
```

```
##            OKI        HAI        YUN        KAT       TAI        HUN        HAN
## OKI         NA 0.08801929 0.12717041 0.19706597 0.1643759 0.07411078 0.11064999
## HAI 0.08801929         NA 0.05389545 0.13069201 0.1372509 0.03611331 0.03832073
## YUN 0.12717041 0.05389545         NA 0.09803865 0.1835931 0.07708983 0.04600133
## KAT 0.19706597 0.13069201 0.09803865         NA 0.2561412 0.14850811 0.13423035
## TAI 0.16437593 0.13725088 0.18359315 0.25614121        NA 0.12925031 0.17880081
## HUN 0.07411078 0.03611331 0.07708983 0.14850811 0.1292503         NA 0.05046773
##            KLP        HOC        QNC        SSK           KAC         CHA
## OKI 0.16656985 0.09549009 0.19007520 0.12817076  1.311262e-01 0.125744140
## HAI 0.08638337 0.03305455 0.12288092 0.05630853  5.254290e-02 0.053245910
## YUN 0.05670882 0.06185930 0.08085858 0.00366398 -6.838534e-05 0.001604481
## KAT 0.14708471 0.14244369 0.17033112 0.09937748  1.006626e-01 0.097234843
## TAI 0.23998253 0.15425313 0.24922008 0.18283845  1.967321e-01 0.178141681
## HUN 0.10841416 0.04314995 0.14309475 0.08054483  7.900004e-02 0.077728277
##           KAN        UTS         CAM        KAG        BEN         LAM
## OKI 0.1634040 0.13493024 0.114640596 0.12106263 0.14616521 0.132969333
## HAI 0.1371587 0.11075509 0.043819388 0.09793452 0.07341023 0.058877477
## YUN 0.1838969 0.15870552 0.003279041 0.13988820 0.02448527 0.006938259
## KAT 0.2486329 0.22282018 0.095547956 0.20548615 0.10205812 0.103613929
## TAI 0.2220346 0.19580993 0.169327020 0.18371222 0.19967862 0.191790397
## HUN 0.1110223 0.08527642 0.066905755 0.07356335 0.09705144 0.084689086
##            SUF        SUU       KUN       INW        INJ        MAT
## OKI 0.21643883 0.15398736 0.2740437 0.2564655 0.21162371 0.13506645
## HAI 0.14879059 0.08892088 0.1926687 0.1850661 0.14633947 0.06332853
## YUN 0.13353818 0.09595000 0.1591835 0.1508741 0.12218318 0.01887260
## KAT 0.05982659 0.06964651 0.2290544 0.1035899 0.09395829 0.11057327
## TAI 0.28326958 0.21716118 0.3507787 0.3354167 0.26762867 0.18747764
## HUN 0.16381699 0.10041490 0.2146355 0.2000360 0.16129956 0.08787756
```

Import sample locations

```
sampling_loc <- readRDS(here("output", "populations", "sampling_loc.rds"))

# Create a named vector to map countries to regions
country_to_region <- c(
  "Bhutan" = "South Asia",
  "Cambodia" = "Southeast Asia",
  "China" = "East Asia",
  "India" = "South Asia",
  "Indonesia" = "Southeast Asia",
  "Japan" = "East Asia",
  "Malaysia" = "Southeast Asia",
  "Maldives" = "South Asia",
  "Nepal" = "South Asia",
  "Sri Lanka" = "South Asia",
  "Taiwan" = "East Asia",
  "Thailand" = "Southeast Asia",
  "Vietnam" = "Southeast Asia"
)

# Add the region to the data frame
sampling_loc$Region2 <- country_to_region[sampling_loc$Country]

# Arrange by region 
sampling_loc <- sampling_loc |>
  dplyr::arrange(
    Region2, Country
  )

# Check it
head(sampling_loc)
```

```
## # A tibble: 6 × 7
##   Pop_City Country Latitude Longitude Region Abbreviation Region2  
##   <chr>    <chr>      <dbl>     <dbl> <chr>  <chr>        <chr>    
## 1 Hainan   China       19.2      110. Asia   HAI          East Asia
## 2 Yunnan   China       24.5      101. Asia   YUN          East Asia
## 3 Hunan    China       27.6      112. Asia   HUN          East Asia
## 4 Okinawa  Japan       26.5      128. Asia   OKI          East Asia
## 5 Sendai   Japan       38.3      141. Asia   SEN          East Asia
## 6 Nagasaki Japan       32.8      130. Asia   NAG          East Asia
```

Order

```
order_pops <- as.vector(sampling_loc$Abbreviation)
order_pops
```

```
##  [1] "HAI" "YUN" "HUN" "OKI" "SEN" "NAG" "SAK" "HIR" "KAN" "YAT" "KYO" "NIG"
## [13] "UTS" "AIZ" "KHO" "SAG" "KAG" "JAT" "TAN" "TAI" "GEL" "BEN" "KUN" "KAT"
## [25] "JAF" "CAM" "SUF" "SUU" "INW" "INJ" "KLP" "MAT" "SSK" "KAC" "SON" "CHA"
## [37] "LAM" "HAN" "HOC" "QNC" "ALV" "POR" "ANT" "MAD" "AWK" "TIK" "BAR" "SAI"
## [49] "PAL" "LOS"
```

Create vector with order of populations

```
# Extract the populations that appear in neutral_df
populations_in_r2_0.01 <- colnames(r2_0.01_df)

# Reorder the populations based on order_pops
poporder <- populations_in_r2_0.01[populations_in_r2_0.01 %in% order_pops]

# Print the reordered populations
print(poporder)
```

```
##  [1] "OKI" "HAI" "YUN" "KAT" "TAI" "HUN" "HAN" "KLP" "HOC" "QNC" "SSK" "KAC"
## [13] "CHA" "KAN" "UTS" "CAM" "KAG" "BEN" "LAM" "SUF" "SUU" "KUN" "INW" "INJ"
## [25] "MAT"
```

Lets check if the matrix is symmetric.

```
isSymmetric(aa)
```

```
## [1] TRUE
```

Now lets order the matrix using poporder. We will also add NA on the
upper left side of the matrix.

```
aa <- aa[poporder, poporder]
aa[lower.tri(aa)] <- NA
```

Now we have to convert the matrix to a data frame to plot it with the
ggplot.

```
pairfst.long <- melt(aa)
summary(pairfst.long)
```

```
##       Var1          Var2         value        
##  OKI    : 25   OKI    : 25   Min.   :-0.0001  
##  HAI    : 25   HAI    : 25   1st Qu.: 0.0797  
##  YUN    : 25   YUN    : 25   Median : 0.1309  
##  KAT    : 25   KAT    : 25   Mean   : 0.1312  
##  TAI    : 25   TAI    : 25   3rd Qu.: 0.1735  
##  HUN    : 25   HUN    : 25   Max.   : 0.3508  
##  (Other):475   (Other):475   NA's   :325
```

Now lets plot the data with ggplot. You can click in the little
square on the top left of the plot to open it on a new window. It will
have the right proportions.

```
pairfst.f <- ggplot(pairfst.long, aes(Var1, Var2)) +
  geom_tile(aes(fill = value), colour = "white") +
  scale_fill_gradient(
    low = "white",
    high = "#71b6ff",
    name = "Fst",
    na.value = "white",
    limits = c(0, 0.5)
  ) +
  scale_x_discrete(position = "top") +
  theme_bw() +
  geom_text(aes(label = ifelse(
    is.na(value), "", formatC(value, digits = 2, format = "f")
  )), size = 3) +
  theme(
    axis.text.x = element_text(angle = 90, hjust = 1),
    axis.title = element_blank(),
    panel.grid.major = element_blank(),
    panel.grid.minor = element_blank(),
    panel.border = element_blank(),
    panel.background = element_blank(),
    axis.text.y = element_text(hjust = 0)
  )
pairfst.f
```

```
ggsave(
  filename = here("output", "fst", "fst_matrix_r2_0.01.pdf"),
  pairfst.f,
  width = 10,
  height = 10,
  units = "in"
)
```

Remove NAs and rename columns

```
# remove NAs
fst2 <-
  pairfst.long |>
  drop_na()

# rename columns
fst2 <-
  fst2 |>
  dplyr::rename(pop1 = 1,
         pop2 = 2,
         fst  = 3)


# Split the data into two data frames, one for pop1 and one for pop2
df_pop1 <- fst2 |>
  dplyr::select(pop = pop1, fst)
df_pop2 <- fst2 |>
  dplyr::select(pop = pop2, fst)

# Combine the two data frames
df_combined <- bind_rows(df_pop1, df_pop2)

# Calculate the mean fst for each population
mean_fst <- df_combined |>
  group_by(pop) |>
  summarise(mean_fst = mean(fst))

print(mean_fst)
```

```
## # A tibble: 25 × 2
##    pop   mean_fst
##    <fct>    <dbl>
##  1 OKI     0.152 
##  2 HAI     0.0932
##  3 YUN     0.0828
##  4 KAT     0.140 
##  5 TAI     0.211 
##  6 HUN     0.104 
##  7 HAN     0.103 
##  8 KLP     0.130 
##  9 HOC     0.104 
## 10 QNC     0.153 
## # ℹ 15 more rows
```

Merge

```
fst3 <-
  sampling_loc |>
  left_join(
    mean_fst,
    by = c("Abbreviation" = "pop")
  ) |>
  drop_na() |>
  dplyr::select(
    -Region
  )

# Remove " Asia" from the Region2 column
fst3$Region2 <- gsub(" Asia", "", fst3$Region2)

# Rename the Region2 column to Region
fst3 <- fst3 |>
  dplyr::rename(Region = Region2)

# check output
head(fst3)
```

```
## # A tibble: 6 × 7
##   Pop_City   Country Latitude Longitude Abbreviation Region mean_fst
##   <chr>      <chr>      <dbl>     <dbl> <chr>        <chr>     <dbl>
## 1 Hainan     China       19.2      110. HAI          East     0.0932
## 2 Yunnan     China       24.5      101. YUN          East     0.0828
## 3 Hunan      China       27.6      112. HUN          East     0.104 
## 4 Okinawa    Japan       26.5      128. OKI          East     0.152 
## 5 Kanazawa   Japan       36.6      137. KAN          East     0.198 
## 6 Utsunomiya Japan       36.6      140. UTS          East     0.173
```

Mean by region

```
# Group by Region and calculate the mean_fst by Region
region_means <- fst3 |>
  group_by(Region) |>
  summarize(mean_fst_by_region = round(mean(mean_fst, na.rm = TRUE), 2)) |>
  ungroup()  # Ungroup the data

# Add the mean_fst_by_region column to the fst3 tibble
fst3 <- fst3 |>
  left_join(region_means, by = "Region")

# Print the modified fst3 tibble
print(fst3)
```

```
## # A tibble: 25 × 8
##    Pop_City    Country  Latitude Longitude Abbreviation Region mean_fst
##    <chr>       <chr>       <dbl>     <dbl> <chr>        <chr>     <dbl>
##  1 Hainan      China       19.2      110.  HAI          East     0.0932
##  2 Yunnan      China       24.5      101.  YUN          East     0.0828
##  3 Hunan       China       27.6      112.  HUN          East     0.104 
##  4 Okinawa     Japan       26.5      128.  OKI          East     0.152 
##  5 Kanazawa    Japan       36.6      137.  KAN          East     0.198 
##  6 Utsunomiya  Japan       36.6      140.  UTS          East     0.173 
##  7 Kagoshima   Japan       31.6      131.  KAG          East     0.157 
##  8 Tainan      Taiwan      23.0      120.  TAI          East     0.211 
##  9 Bengaluru   India       13.0       77.6 BEN          South    0.0978
## 10 Kunfunadhoo Maldives     5.67      73   KUN          South    0.228 
## # ℹ 15 more rows
## # ℹ 1 more variable: mean_fst_by_region <dbl>
```

Mean by country

```
# Group by Country and calculate the mean_fst by Country
country_means <- fst3 |>
  group_by(Country) |>
  summarize(mean_fst_by_country = round(mean(mean_fst, na.rm = TRUE), 2)) |>
  ungroup()  # Ungroup the data

# Add the mean_fst_by_country column to the fst3 tibble
fst3 <- fst3 |>
  left_join(country_means, by = "Country")

# Print the modified fst3 tibble
print(fst3)
```

```
## # A tibble: 25 × 9
##    Pop_City    Country  Latitude Longitude Abbreviation Region mean_fst
##    <chr>       <chr>       <dbl>     <dbl> <chr>        <chr>     <dbl>
##  1 Hainan      China       19.2      110.  HAI          East     0.0932
##  2 Yunnan      China       24.5      101.  YUN          East     0.0828
##  3 Hunan       China       27.6      112.  HUN          East     0.104 
##  4 Okinawa     Japan       26.5      128.  OKI          East     0.152 
##  5 Kanazawa    Japan       36.6      137.  KAN          East     0.198 
##  6 Utsunomiya  Japan       36.6      140.  UTS          East     0.173 
##  7 Kagoshima   Japan       31.6      131.  KAG          East     0.157 
##  8 Tainan      Taiwan      23.0      120.  TAI          East     0.211 
##  9 Bengaluru   India       13.0       77.6 BEN          South    0.0978
## 10 Kunfunadhoo Maldives     5.67      73   KUN          South    0.228 
## # ℹ 15 more rows
## # ℹ 2 more variables: mean_fst_by_region <dbl>, mean_fst_by_country <dbl>
```

Mean by latitude

```
# Add a new column to indicate whether the latitude is above or below 30N
fst3 <- fst3 |>
  mutate(Latitude_group = ifelse(Latitude >= 30, "Above 30N", "Below 30N"))

# Summarize the data by Latitude_group and calculate the mean_fst
summary_by_latitude <- fst3 |>
  group_by(Latitude_group) |>
  summarize(mean_fst_by_latitude = mean(mean_fst, na.rm = TRUE)) |>
  ungroup()  # Ungroup the data

# Add the mean_fst_by_latitude column to the fst3 tibble
fst3 <- fst3 |>
  left_join(summary_by_latitude, by = "Latitude_group")


# Rename columns
fst3 <- fst3 |>
  dplyr::rename(
    City = Pop_City
  )

# Print the modified fst3 tibble
print(fst3)
```

```
## # A tibble: 25 × 11
##    City        Country  Latitude Longitude Abbreviation Region mean_fst
##    <chr>       <chr>       <dbl>     <dbl> <chr>        <chr>     <dbl>
##  1 Hainan      China       19.2      110.  HAI          East     0.0932
##  2 Yunnan      China       24.5      101.  YUN          East     0.0828
##  3 Hunan       China       27.6      112.  HUN          East     0.104 
##  4 Okinawa     Japan       26.5      128.  OKI          East     0.152 
##  5 Kanazawa    Japan       36.6      137.  KAN          East     0.198 
##  6 Utsunomiya  Japan       36.6      140.  UTS          East     0.173 
##  7 Kagoshima   Japan       31.6      131.  KAG          East     0.157 
##  8 Tainan      Taiwan      23.0      120.  TAI          East     0.211 
##  9 Bengaluru   India       13.0       77.6 BEN          South    0.0978
## 10 Kunfunadhoo Maldives     5.67      73   KUN          South    0.228 
## # ℹ 15 more rows
## # ℹ 4 more variables: mean_fst_by_region <dbl>, mean_fst_by_country <dbl>,
## #   Latitude_group <chr>, mean_fst_by_latitude <dbl>
```

```
fst4 <- fst3 |>
  dplyr::select(
    Latitude_group, mean_fst_by_latitude, Region, mean_fst_by_region, Country, mean_fst_by_country, City, Abbreviation, mean_fst,
  )

fst4 <- fst4 |>
  arrange(
    Latitude_group, Region, Country, City
  )

# Round
fst4 <- fst4 |>
  mutate_if(is.numeric, ~ round(., 2))

head(fst4)
```

```
## # A tibble: 6 × 9
##   Latitude_group mean_fst_by_latitude Region mean_fst_by_region Country
##   <chr>                         <dbl> <chr>               <dbl> <chr>  
## 1 Above 30N                      0.18 East                 0.15 Japan  
## 2 Above 30N                      0.18 East                 0.15 Japan  
## 3 Above 30N                      0.18 East                 0.15 Japan  
## 4 Below 30N                      0.13 East                 0.15 China  
## 5 Below 30N                      0.13 East                 0.15 China  
## 6 Below 30N                      0.13 East                 0.15 China  
## # ℹ 4 more variables: mean_fst_by_country <dbl>, City <chr>,
## #   Abbreviation <chr>, mean_fst <dbl>
```

```
# Set theme if you want to use something different from the previous table
set_flextable_defaults(
  font.family = "Arial",
  font.size = 9,
  big.mark = ",",
  theme_fun = "theme_zebra" # try the themes: theme_alafoli(), theme_apa(), theme_booktabs(), theme_box(), theme_tron_legacy(), theme_tron(), theme_vader(), theme_vanilla(), theme_zebra()
)

# Then create the flextable object
flex_table <- flextable(fst4) |>
  set_caption(caption = as_paragraph(
    as_chunk(
      "Table 1. Fst values using SNPs after prunning with r2 0.01.",
      props = fp_text_default(color = "#000000", font.size = 14)
    )
  ),
  fp_p = fp_par(text.align = "center", padding = 5))

# Print the flextable
flex_table
```

Table 1. Fst values using SNPs after prunning with r2 0.01.

| Latitude\_group | mean\_fst\_by\_latitude | Region | mean\_fst\_by\_region | Country | mean\_fst\_by\_country | City | Abbreviation | mean\_fst |
| --- | --- | --- | --- | --- | --- | --- | --- | --- |
| Above 30N | 0.18 | East | 0.15 | Japan | 0.17 | Kagoshima | KAG | 0.16 |
| Above 30N | 0.18 | East | 0.15 | Japan | 0.17 | Kanazawa | KAN | 0.20 |
| Above 30N | 0.18 | East | 0.15 | Japan | 0.17 | Utsunomiya | UTS | 0.17 |
| Below 30N | 0.13 | East | 0.15 | China | 0.09 | Hainan | HAI | 0.09 |
| Below 30N | 0.13 | East | 0.15 | China | 0.09 | Hunan | HUN | 0.10 |
| Below 30N | 0.13 | East | 0.15 | China | 0.09 | Yunnan | YUN | 0.08 |
| Below 30N | 0.13 | East | 0.15 | Japan | 0.17 | Okinawa | OKI | 0.15 |
| Below 30N | 0.13 | East | 0.15 | Taiwan | 0.21 | Tainan | TAI | 0.21 |
| Below 30N | 0.13 | South | 0.16 | India | 0.10 | Bengaluru | BEN | 0.10 |
| Below 30N | 0.13 | South | 0.16 | Maldives | 0.23 | Kunfunadhoo | KUN | 0.23 |
| Below 30N | 0.13 | South | 0.16 | Nepal | 0.14 | Kathmandu | KAT | 0.14 |
| Below 30N | 0.13 | Southeast | 0.12 | Cambodia | 0.08 | Phnom Penh | CAM | 0.08 |
| Below 30N | 0.13 | Southeast | 0.12 | Indonesia | 0.16 | Jakarta | INJ | 0.16 |
| Below 30N | 0.13 | Southeast | 0.12 | Indonesia | 0.16 | Sulawesi (Forest) | SUF | 0.16 |
| Below 30N | 0.13 | Southeast | 0.12 | Indonesia | 0.16 | Sulawesi (Urban) | SUU | 0.12 |
| Below 30N | 0.13 | Southeast | 0.12 | Indonesia | 0.16 | Wainyapu | INW | 0.19 |
| Below 30N | 0.13 | Southeast | 0.12 | Malaysia | 0.11 | Kuala Lumpur | KLP | 0.13 |
| Below 30N | 0.13 | Southeast | 0.12 | Malaysia | 0.11 | Tambun | MAT | 0.09 |
| Below 30N | 0.13 | Southeast | 0.12 | Thailand | 0.09 | Chanthaburi | CHA | 0.08 |
| Below 30N | 0.13 | Southeast | 0.12 | Thailand | 0.09 | Kanchanaburi | KAC | 0.09 |
| Below 30N | 0.13 | Southeast | 0.12 | Thailand | 0.09 | Lampang | LAM | 0.09 |
| Below 30N | 0.13 | Southeast | 0.12 | Thailand | 0.09 | Sisaket | SSK | 0.08 |
| Below 30N | 0.13 | Southeast | 0.12 | Vietnam | 0.12 | Hanoi | HAN | 0.10 |
| Below 30N | 0.13 | Southeast | 0.12 | Vietnam | 0.12 | Ho Chi Minh City | HOC | 0.10 |
| Below 30N | 0.13 | Southeast | 0.12 | Vietnam | 0.12 | Quy Nhon City | QNC | 0.15 |

```
# Initialize Word document
doc <- 
  read_docx() |>
  body_add_flextable(value = flex_table)

# Define the output path with 'here' library
output_path <- here(
  "output",
  "fst", 
  "fst_r2_0.01_SNPS.docx"
  )

# Save the Word document
print(doc, target = output_path)
```

## 3. SNP set r2 0.1

```
# Remove all objects from the environment
rm(list = ls())

# Run the garbage collector to free up memory
gc()
```

```
##           used  (Mb) gc trigger  (Mb) limit (Mb) max used  (Mb)
## Ncells 3098651 165.5    4574771 244.4         NA  4574771 244.4
## Vcells 5357442  40.9   10146329  77.5      32768  8388595  64.0
```

```
plink \
--allow-extra-chr \
--keep-allele-order \
--bfile output/populations/snps_sets/r2_0.1 \
--keep-fam output/fst/pops_4fst.txt \
--recodeA \
--out output/fst/r2_0.1 \
--silent;
grep 'samples\|variants\|remaining' output/fst/r2_0.1.log
```

```
## 57780 variants loaded from .bim file.
## --keep-fam: 230 people remaining.
## Total genotyping rate in remaining samples is 0.972118.
## 57780 variants and 230 people pass filters and QC.
```

Look at https://rdrr.io/cran/StAMPP/man/stamppFst.html for
details of Fst estimations

```
r2_0.1 <-
  read.PLINK(
    here(
      "output", "fst", "r2_0.1.raw"
    ),
    quiet = FALSE,
    chunkSize = 1000,
    parallel = require("parallel"),
    n.cores = 4
  )

summary(r2_0.1)
```

This chunk will take a couple minutes to run.

```
# convert
r2_0.1_2 <- stamppConvert(r2_0.1, type="genlight")

# run stampp. If you want to runn with bootstraps and nclusters use the HPC. It will run out of memory on a 32Gb laptop
r2_0.1_3 <- stamppFst(r2_0.1_2, 1, 95, 1)
```

Save it

```
saveRDS(
  r2_0.1_3, here(
    "output", "fst", "r2_0.1.rds"
  )
)
```

To load it

```
r2_0.1_3 <- readRDS(
  here(
    "output", "fst", "r2_0.1.rds"
  )
)
```

Now lets look at the object

```
summary(r2_0.1_3)
```

```
##       OKI               HAI               YUN                 KAT         
##  Min.   :0.07442   Min.   :0.03415   Min.   :-0.000061   Min.   :0.06197  
##  1st Qu.:0.11875   1st Qu.:0.04948   1st Qu.: 0.020653   1st Qu.:0.10390  
##  Median :0.13231   Median :0.08208   Median : 0.074156   Median :0.11475  
##  Mean   :0.14973   Mean   :0.09324   Mean   : 0.081748   Mean   :0.14294  
##  3rd Qu.:0.17241   3rd Qu.:0.13763   3rd Qu.: 0.138696   3rd Qu.:0.17543  
##  Max.   :0.26203   Max.   :0.19423   Max.   : 0.184499   Max.   :0.26064  
##  NA's   :1         NA's   :2         NA's   :3           NA's   :4        
##       TAI              HUN               HAN               KLP         
##  Min.   :0.1335   Min.   :0.04272   Min.   :0.03747   Min.   :0.05153  
##  1st Qu.:0.1827   1st Qu.:0.07029   1st Qu.:0.04754   1st Qu.:0.05847  
##  Median :0.1963   Median :0.08814   Median :0.09929   Median :0.13852  
##  Mean   :0.2179   Mean   :0.10291   Mean   :0.10694   Mean   :0.13005  
##  3rd Qu.:0.2400   3rd Qu.:0.12577   3rd Qu.:0.15623   3rd Qu.:0.18669  
##  Max.   :0.3444   Max.   :0.20465   Max.   :0.22103   Max.   :0.24103  
##  NA's   :5        NA's   :6         NA's   :7         NA's   :8        
##       HOC               QNC               SSK                KAC          
##  Min.   :0.04642   Min.   :0.07948   Min.   :0.002698   Min.   :0.001375  
##  1st Qu.:0.06326   1st Qu.:0.08570   1st Qu.:0.011550   1st Qu.:0.018868  
##  Median :0.10400   Median :0.17367   Median :0.113973   Median :0.132199  
##  Mean   :0.11322   Mean   :0.15507   Mean   :0.087070   Mean   :0.095838  
##  3rd Qu.:0.15567   3rd Qu.:0.21453   3rd Qu.:0.147997   3rd Qu.:0.153453  
##  Max.   :0.21166   Max.   :0.24452   Max.   :0.179032   Max.   :0.184788  
##  NA's   :9         NA's   :10        NA's   :11         NA's   :12        
##       CHA                KAN              UTS               CAM          
##  Min.   :0.004038   Min.   :0.1031   Min.   :0.08997   Min.   :0.008172  
##  1st Qu.:0.023675   1st Qu.:0.1759   1st Qu.:0.15738   1st Qu.:0.025363  
##  Median :0.128982   Median :0.1946   Median :0.17412   Median :0.121331  
##  Mean   :0.099625   Mean   :0.2137   Mean   :0.19413   Mean   :0.094094  
##  3rd Qu.:0.149675   3rd Qu.:0.2701   3rd Qu.:0.24146   3rd Qu.:0.137461  
##  Max.   :0.177767   Max.   :0.3177   Max.   :0.28404   Max.   :0.161974  
##  NA's   :13         NA's   :14       NA's   :15        NA's   :16        
##       KAG              BEN               LAM               SUF         
##  Min.   :0.1362   Min.   :0.02818   Min.   :0.01876   Min.   :0.04022  
##  1st Qu.:0.1471   1st Qu.:0.07735   1st Qu.:0.10946   1st Qu.:0.07931  
##  Median :0.1938   Median :0.13012   Median :0.13912   Median :0.09606  
##  Mean   :0.1963   Mean   :0.11092   Mean   :0.12277   Mean   :0.13239  
##  3rd Qu.:0.2366   3rd Qu.:0.14495   3rd Qu.:0.16100   3rd Qu.:0.15057  
##  Max.   :0.2646   Max.   :0.17353   Max.   :0.17178   Max.   :0.29578  
##  NA's   :17       NA's   :18        NA's   :19        NA's   :20       
##       SUU               KUN              INW               INJ        
##  Min.   :0.08678   Min.   :0.1681   Min.   :0.06515   Min.   :0.1366  
##  1st Qu.:0.10211   1st Qu.:0.2155   1st Qu.:0.09282   1st Qu.:0.1366  
##  Median :0.11800   Median :0.2629   Median :0.12049   Median :0.1366  
##  Mean   :0.14286   Mean   :0.2578   Mean   :0.12049   Mean   :0.1366  
##  3rd Qu.:0.15875   3rd Qu.:0.3027   3rd Qu.:0.14816   3rd Qu.:0.1366  
##  Max.   :0.24867   Max.   :0.3425   Max.   :0.17582   Max.   :0.1366  
##  NA's   :21        NA's   :22       NA's   :23        NA's   :24      
##       MAT     
##  Min.   : NA  
##  1st Qu.: NA  
##  Median : NA  
##  Mean   :NaN  
##  3rd Qu.: NA  
##  Max.   : NA  
##  NA's   :25
```

If you want you can save the fst values as csv.

```
# Convert to data frame
r2_0.1_df <- data.frame(r2_0.1_3)

# Save it
write.csv(r2_0.1_df, file = here("output", "fst", "r2_0.1_df.csv"))
```

Check the Fst values

```
head(r2_0.1_df)
```

```
##            OKI        HAI        YUN       KAT       TAI HUN HAN KLP HOC QNC
## OKI         NA         NA         NA        NA        NA  NA  NA  NA  NA  NA
## HAI 0.08802883         NA         NA        NA        NA  NA  NA  NA  NA  NA
## YUN 0.11878133 0.04941474         NA        NA        NA  NA  NA  NA  NA  NA
## KAT 0.19738113 0.13464192 0.10181861        NA        NA  NA  NA  NA  NA  NA
## TAI 0.16804359 0.14154214 0.18449852 0.2606445        NA  NA  NA  NA  NA  NA
## HUN 0.07441617 0.03534541 0.06802916 0.1476221 0.1334822  NA  NA  NA  NA  NA
##     SSK KAC CHA KAN UTS CAM KAG BEN LAM SUF SUU KUN INW INJ MAT
## OKI  NA  NA  NA  NA  NA  NA  NA  NA  NA  NA  NA  NA  NA  NA  NA
## HAI  NA  NA  NA  NA  NA  NA  NA  NA  NA  NA  NA  NA  NA  NA  NA
## YUN  NA  NA  NA  NA  NA  NA  NA  NA  NA  NA  NA  NA  NA  NA  NA
## KAT  NA  NA  NA  NA  NA  NA  NA  NA  NA  NA  NA  NA  NA  NA  NA
## TAI  NA  NA  NA  NA  NA  NA  NA  NA  NA  NA  NA  NA  NA  NA  NA
## HUN  NA  NA  NA  NA  NA  NA  NA  NA  NA  NA  NA  NA  NA  NA  NA
```

Now lets get the Fst values from the object albo3. It has the
bootstraps, CI limits, p-values, and Fst values. We will convert the
data into a matrix.

```
aa <- as.matrix(r2_0.1_df)
aa[upper.tri(aa)] <- t(aa)[upper.tri(t(aa))]
head(aa)
```

```
##            OKI        HAI        YUN       KAT       TAI        HUN        HAN
## OKI         NA 0.08802883 0.11878133 0.1973811 0.1680436 0.07441617 0.10763474
## HAI 0.08802883         NA 0.04941474 0.1346419 0.1415421 0.03534541 0.03825566
## YUN 0.11878133 0.04941474         NA 0.1018186 0.1844985 0.06802916 0.04338869
## KAT 0.19738113 0.13464192 0.10181861        NA 0.2606445 0.14762208 0.13924461
## TAI 0.16804359 0.14154214 0.18449852 0.2606445        NA 0.13348223 0.18073656
## HUN 0.07441617 0.03534541 0.06802916 0.1476221 0.1334822         NA 0.04712316
##            KLP        HOC       QNC         SSK           KAC        CHA
## OKI 0.15791274 0.09583416 0.1855034 0.120337901  1.222639e-01 0.11864070
## HAI 0.08207801 0.03414611 0.1192940 0.051748990  4.855596e-02 0.04954779
## YUN 0.05497547 0.05872326 0.0802834 0.003470757 -6.095245e-05 0.00216753
## KAT 0.14992931 0.14537279 0.1754255 0.103904742  1.043844e-01 0.10291812
## TAI 0.23665704 0.15932868 0.2500647 0.183363174  1.938385e-01 0.18037855
## HUN 0.09955817 0.04272361 0.1355892 0.070972254  6.854084e-02 0.06961833
##           KAN        UTS         CAM        KAG        BEN         LAM
## OKI 0.1661065 0.13562198 0.109049041 0.12082719 0.13838680 0.123949616
## HAI 0.1406218 0.11163440 0.041994919 0.09877636 0.06924561 0.053818795
## YUN 0.1797822 0.14989155 0.003581621 0.13178798 0.02349872 0.006781344
## KAT 0.2507648 0.22214457 0.101256096 0.20504141 0.10811511 0.106234969
## TAI 0.2282426 0.19867600 0.171069919 0.18782724 0.20034447 0.189514500
## HUN 0.1159453 0.08822364 0.059878361 0.07511422 0.08813833 0.073799872
##            SUF        SUU       KUN       INW        INJ        MAT
## OKI 0.22063500 0.15757698 0.2620293 0.2615279 0.21402287 0.12899644
## HAI 0.15560449 0.09438831 0.1867310 0.1942290 0.15201957 0.06079123
## YUN 0.14099822 0.09767201 0.1564464 0.1629609 0.12806009 0.01970472
## KAT 0.06196652 0.07006897 0.2304904 0.1073455 0.09416537 0.11474970
## TAI 0.29024752 0.22479493 0.3443908 0.3418771 0.27387778 0.18974486
## HUN 0.16664122 0.10268620 0.2017426 0.2046510 0.16392725 0.08049047
```

Import sample locations

```
sampling_loc <- readRDS(here("output", "populations", "sampling_loc.rds"))

# Create a named vector to map countries to regions
country_to_region <- c(
  "Bhutan" = "South Asia",
  "Cambodia" = "Southeast Asia",
  "China" = "East Asia",
  "India" = "South Asia",
  "Indonesia" = "Southeast Asia",
  "Japan" = "East Asia",
  "Malaysia" = "Southeast Asia",
  "Maldives" = "South Asia",
  "Nepal" = "South Asia",
  "Sri Lanka" = "South Asia",
  "Taiwan" = "East Asia",
  "Thailand" = "Southeast Asia",
  "Vietnam" = "Southeast Asia"
)

# Add the region to the data frame
sampling_loc$Region2 <- country_to_region[sampling_loc$Country]

# Arrange by region 
sampling_loc <- sampling_loc |>
  dplyr::arrange(
    Region2, Country
  )

# Check it
head(sampling_loc)
```

```
## # A tibble: 6 × 7
##   Pop_City Country Latitude Longitude Region Abbreviation Region2  
##   <chr>    <chr>      <dbl>     <dbl> <chr>  <chr>        <chr>    
## 1 Hainan   China       19.2      110. Asia   HAI          East Asia
## 2 Yunnan   China       24.5      101. Asia   YUN          East Asia
## 3 Hunan    China       27.6      112. Asia   HUN          East Asia
## 4 Okinawa  Japan       26.5      128. Asia   OKI          East Asia
## 5 Sendai   Japan       38.3      141. Asia   SEN          East Asia
## 6 Nagasaki Japan       32.8      130. Asia   NAG          East Asia
```

Order

```
order_pops <- as.vector(sampling_loc$Abbreviation)
order_pops
```

```
##  [1] "HAI" "YUN" "HUN" "OKI" "SEN" "NAG" "SAK" "HIR" "KAN" "YAT" "KYO" "NIG"
## [13] "UTS" "AIZ" "KHO" "SAG" "KAG" "JAT" "TAN" "TAI" "GEL" "BEN" "KUN" "KAT"
## [25] "JAF" "CAM" "SUF" "SUU" "INW" "INJ" "KLP" "MAT" "SSK" "KAC" "SON" "CHA"
## [37] "LAM" "HAN" "HOC" "QNC" "ALV" "POR" "ANT" "MAD" "AWK" "TIK" "BAR" "SAI"
## [49] "PAL" "LOS"
```

Create vector with order of populations

```
# Extract the populations that appear in neutral_df
populations_in_r2_0.1 <- colnames(r2_0.1_df)

# Reorder the populations based on order_pops
poporder <- populations_in_r2_0.1[populations_in_r2_0.1 %in% order_pops]

# Print the reordered populations
print(poporder)
```

```
##  [1] "OKI" "HAI" "YUN" "KAT" "TAI" "HUN" "HAN" "KLP" "HOC" "QNC" "SSK" "KAC"
## [13] "CHA" "KAN" "UTS" "CAM" "KAG" "BEN" "LAM" "SUF" "SUU" "KUN" "INW" "INJ"
## [25] "MAT"
```

Lets check if the matrix is symmetric.

```
isSymmetric(aa)
```

```
## [1] TRUE
```

Now lets order the matrix using poporder. We will also add NA on the
upper left side of the matrix.

```
aa <- aa[poporder, poporder]
aa[lower.tri(aa)] <- NA
```

Now we have to convert the matrix to a data frame to plot it with
ggplot.

```
pairfst.long <- melt(aa)
summary(pairfst.long)
```

```
##       Var1          Var2         value        
##  OKI    : 25   OKI    : 25   Min.   :-0.0001  
##  HAI    : 25   HAI    : 25   1st Qu.: 0.0749  
##  YUN    : 25   YUN    : 25   Median : 0.1320  
##  KAT    : 25   KAT    : 25   Mean   : 0.1313  
##  TAI    : 25   TAI    : 25   3rd Qu.: 0.1763  
##  HUN    : 25   HUN    : 25   Max.   : 0.3444  
##  (Other):475   (Other):475   NA's   :325
```

Now lets plot the data with ggplot. You can click in the little
square on the top left of the plot to open it on a new window. It will
have the right proportions.

```
pairfst.f <- ggplot(pairfst.long, aes(Var1, Var2)) +
  geom_tile(aes(fill = value), colour = "white") +
  scale_fill_gradient(
    low = "white",
    high = "#71b6ff",
    name = "Fst",
    na.value = "white",
    limits = c(0, 0.5)
  ) +
  scale_x_discrete(position = "top") +
  theme_bw() +
  geom_text(aes(label = ifelse(
    is.na(value), "", formatC(value, digits = 2, format = "f")
  )), size = 3) +
  theme(
    axis.text.x = element_text(angle = 90, hjust = 1),
    axis.title = element_blank(),
    panel.grid.major = element_blank(),
    panel.grid.minor = element_blank(),
    panel.border = element_blank(),
    panel.background = element_blank(),
    axis.text.y = element_text(hjust = 0)
  )
pairfst.f
```

```
ggsave(
  filename = here("output", "fst", "fst_matrix_r2_0.1.pdf"),
  pairfst.f,
  width = 10,
  height = 10,
  units = "in"
)
```

Remove NAs and rename columns

```
# remove NAs
fst2 <-
  pairfst.long |>
  drop_na()

# rename columns
fst2 <-
  fst2 |>
  dplyr::rename(pop1 = 1,
         pop2 = 2,
         fst  = 3)


# Split the data into two data frames, one for pop1 and one for pop2
df_pop1 <- fst2 |>
  dplyr::select(pop = pop1, fst)
df_pop2 <- fst2 |>
  dplyr::select(pop = pop2, fst)

# Combine the two data frames
df_combined <- bind_rows(df_pop1, df_pop2)

# Calculate the mean fst for each population
mean_fst <- df_combined |>
  group_by(pop) |>
  summarise(mean_fst = mean(fst))

print(mean_fst)
```

```
## # A tibble: 25 × 2
##    pop   mean_fst
##    <fct>    <dbl>
##  1 OKI     0.150 
##  2 HAI     0.0930
##  3 YUN     0.0819
##  4 KAT     0.143 
##  5 TAI     0.213 
##  6 HUN     0.101 
##  7 HAN     0.103 
##  8 KLP     0.128 
##  9 HOC     0.104 
## 10 QNC     0.153 
## # ℹ 15 more rows
```

Merge

```
fst3 <-
  sampling_loc |>
  left_join(
    mean_fst,
    by = c("Abbreviation" = "pop")
  ) |>
  drop_na() |>
  dplyr::select(
    -Region
  )

# Remove " Asia" from the Region2 column
fst3$Region2 <- gsub(" Asia", "", fst3$Region2)

# Rename the Region2 column to Region
fst3 <- fst3 |>
  dplyr::rename(Region = Region2)

# check output
head(fst3)
```

```
## # A tibble: 6 × 7
##   Pop_City   Country Latitude Longitude Abbreviation Region mean_fst
##   <chr>      <chr>      <dbl>     <dbl> <chr>        <chr>     <dbl>
## 1 Hainan     China       19.2      110. HAI          East     0.0930
## 2 Yunnan     China       24.5      101. YUN          East     0.0819
## 3 Hunan      China       27.6      112. HUN          East     0.101 
## 4 Okinawa    Japan       26.5      128. OKI          East     0.150 
## 5 Kanazawa   Japan       36.6      137. KAN          East     0.198 
## 6 Utsunomiya Japan       36.6      140. UTS          East     0.169
```

Mean by region

```
# Group by Region and calculate the mean_fst by Region
region_means <- fst3 |>
  group_by(Region) |>
  summarize(mean_fst_by_region = round(mean(mean_fst, na.rm = TRUE), 2)) |>
  ungroup()  # Ungroup the data

# Add the mean_fst_by_region column to the fst3 tibble
fst3 <- fst3 |>
  left_join(region_means, by = "Region")

# Print the modified fst3 tibble
print(fst3)
```

```
## # A tibble: 25 × 8
##    Pop_City    Country  Latitude Longitude Abbreviation Region mean_fst
##    <chr>       <chr>       <dbl>     <dbl> <chr>        <chr>     <dbl>
##  1 Hainan      China       19.2      110.  HAI          East     0.0930
##  2 Yunnan      China       24.5      101.  YUN          East     0.0819
##  3 Hunan       China       27.6      112.  HUN          East     0.101 
##  4 Okinawa     Japan       26.5      128.  OKI          East     0.150 
##  5 Kanazawa    Japan       36.6      137.  KAN          East     0.198 
##  6 Utsunomiya  Japan       36.6      140.  UTS          East     0.169 
##  7 Kagoshima   Japan       31.6      131.  KAG          East     0.154 
##  8 Tainan      Taiwan      23.0      120.  TAI          East     0.213 
##  9 Bengaluru   India       13.0       77.6 BEN          South    0.0974
## 10 Kunfunadhoo Maldives     5.67      73   KUN          South    0.225 
## # ℹ 15 more rows
## # ℹ 1 more variable: mean_fst_by_region <dbl>
```

Mean by country

```
# Group by Country and calculate the mean_fst by Country
country_means <- fst3 |>
  group_by(Country) |>
  summarize(mean_fst_by_country = round(mean(mean_fst, na.rm = TRUE), 2)) |>
  ungroup()  # Ungroup the data

# Add the mean_fst_by_country column to the fst3 tibble
fst3 <- fst3 |>
  left_join(country_means, by = "Country")

# Print the modified fst3 tibble
print(fst3)
```

```
## # A tibble: 25 × 9
##    Pop_City    Country  Latitude Longitude Abbreviation Region mean_fst
##    <chr>       <chr>       <dbl>     <dbl> <chr>        <chr>     <dbl>
##  1 Hainan      China       19.2      110.  HAI          East     0.0930
##  2 Yunnan      China       24.5      101.  YUN          East     0.0819
##  3 Hunan       China       27.6      112.  HUN          East     0.101 
##  4 Okinawa     Japan       26.5      128.  OKI          East     0.150 
##  5 Kanazawa    Japan       36.6      137.  KAN          East     0.198 
##  6 Utsunomiya  Japan       36.6      140.  UTS          East     0.169 
##  7 Kagoshima   Japan       31.6      131.  KAG          East     0.154 
##  8 Tainan      Taiwan      23.0      120.  TAI          East     0.213 
##  9 Bengaluru   India       13.0       77.6 BEN          South    0.0974
## 10 Kunfunadhoo Maldives     5.67      73   KUN          South    0.225 
## # ℹ 15 more rows
## # ℹ 2 more variables: mean_fst_by_region <dbl>, mean_fst_by_country <dbl>
```

Mean by latitude

```
# Add a new column to indicate whether the latitude is above or below 30N
fst3 <- fst3 |>
  mutate(Latitude_group = ifelse(Latitude >= 30, "Above 30N", "Below 30N"))

# Summarize the data by Latitude_group and calculate the mean_fst
summary_by_latitude <- fst3 |>
  group_by(Latitude_group) |>
  summarize(mean_fst_by_latitude = mean(mean_fst, na.rm = TRUE)) |>
  ungroup()  # Ungroup the data

# Add the mean_fst_by_latitude column to the fst3 tibble
fst3 <- fst3 |>
  left_join(summary_by_latitude, by = "Latitude_group")


# Rename columns
fst3 <- fst3 |>
  dplyr::rename(
    City = Pop_City
  )

# Print the modified fst3 tibble
print(fst3)
```

```
## # A tibble: 25 × 11
##    City        Country  Latitude Longitude Abbreviation Region mean_fst
##    <chr>       <chr>       <dbl>     <dbl> <chr>        <chr>     <dbl>
##  1 Hainan      China       19.2      110.  HAI          East     0.0930
##  2 Yunnan      China       24.5      101.  YUN          East     0.0819
##  3 Hunan       China       27.6      112.  HUN          East     0.101 
##  4 Okinawa     Japan       26.5      128.  OKI          East     0.150 
##  5 Kanazawa    Japan       36.6      137.  KAN          East     0.198 
##  6 Utsunomiya  Japan       36.6      140.  UTS          East     0.169 
##  7 Kagoshima   Japan       31.6      131.  KAG          East     0.154 
##  8 Tainan      Taiwan      23.0      120.  TAI          East     0.213 
##  9 Bengaluru   India       13.0       77.6 BEN          South    0.0974
## 10 Kunfunadhoo Maldives     5.67      73   KUN          South    0.225 
## # ℹ 15 more rows
## # ℹ 4 more variables: mean_fst_by_region <dbl>, mean_fst_by_country <dbl>,
## #   Latitude_group <chr>, mean_fst_by_latitude <dbl>
```

```
fst4 <- fst3 |>
  dplyr::select(
    Latitude_group, mean_fst_by_latitude, Region, mean_fst_by_region, Country, mean_fst_by_country, City, Abbreviation, mean_fst,
  )

fst4 <- fst4 |>
  arrange(
    Latitude_group, Region, Country, City
  )

# Round
fst4 <- fst4 |>
  mutate_if(is.numeric, ~ round(., 2))

head(fst4)
```

```
## # A tibble: 6 × 9
##   Latitude_group mean_fst_by_latitude Region mean_fst_by_region Country
##   <chr>                         <dbl> <chr>               <dbl> <chr>  
## 1 Above 30N                      0.17 East                 0.14 Japan  
## 2 Above 30N                      0.17 East                 0.14 Japan  
## 3 Above 30N                      0.17 East                 0.14 Japan  
## 4 Below 30N                      0.13 East                 0.14 China  
## 5 Below 30N                      0.13 East                 0.14 China  
## 6 Below 30N                      0.13 East                 0.14 China  
## # ℹ 4 more variables: mean_fst_by_country <dbl>, City <chr>,
## #   Abbreviation <chr>, mean_fst <dbl>
```

```
# Set theme if you want to use something different from the previous table
set_flextable_defaults(
  font.family = "Arial",
  font.size = 9,
  big.mark = ",",
  theme_fun = "theme_zebra" # try the themes: theme_alafoli(), theme_apa(), theme_booktabs(), theme_box(), theme_tron_legacy(), theme_tron(), theme_vader(), theme_vanilla(), theme_zebra()
)

# Then create the flextable object
flex_table <- flextable(fst4) |>
  set_caption(caption = as_paragraph(
    as_chunk(
      "Table 1. Fst values using SNPs after prunning with r2 0.1.",
      props = fp_text_default(color = "#000000", font.size = 14)
    )
  ),
  fp_p = fp_par(text.align = "center", padding = 5))

# Print the flextable
flex_table
```

Table 1. Fst values using SNPs after prunning with r2 0.1.

| Latitude\_group | mean\_fst\_by\_latitude | Region | mean\_fst\_by\_region | Country | mean\_fst\_by\_country | City | Abbreviation | mean\_fst |
| --- | --- | --- | --- | --- | --- | --- | --- | --- |
| Above 30N | 0.17 | East | 0.14 | Japan | 0.17 | Kagoshima | KAG | 0.15 |
| Above 30N | 0.17 | East | 0.14 | Japan | 0.17 | Kanazawa | KAN | 0.20 |
| Above 30N | 0.17 | East | 0.14 | Japan | 0.17 | Utsunomiya | UTS | 0.17 |
| Below 30N | 0.13 | East | 0.14 | China | 0.09 | Hainan | HAI | 0.09 |
| Below 30N | 0.13 | East | 0.14 | China | 0.09 | Hunan | HUN | 0.10 |
| Below 30N | 0.13 | East | 0.14 | China | 0.09 | Yunnan | YUN | 0.08 |
| Below 30N | 0.13 | East | 0.14 | Japan | 0.17 | Okinawa | OKI | 0.15 |
| Below 30N | 0.13 | East | 0.14 | Taiwan | 0.21 | Tainan | TAI | 0.21 |
| Below 30N | 0.13 | South | 0.16 | India | 0.10 | Bengaluru | BEN | 0.10 |
| Below 30N | 0.13 | South | 0.16 | Maldives | 0.23 | Kunfunadhoo | KUN | 0.23 |
| Below 30N | 0.13 | South | 0.16 | Nepal | 0.14 | Kathmandu | KAT | 0.14 |
| Below 30N | 0.13 | Southeast | 0.12 | Cambodia | 0.08 | Phnom Penh | CAM | 0.08 |
| Below 30N | 0.13 | Southeast | 0.12 | Indonesia | 0.17 | Jakarta | INJ | 0.16 |
| Below 30N | 0.13 | Southeast | 0.12 | Indonesia | 0.17 | Sulawesi (Forest) | SUF | 0.17 |
| Below 30N | 0.13 | Southeast | 0.12 | Indonesia | 0.17 | Sulawesi (Urban) | SUU | 0.13 |
| Below 30N | 0.13 | Southeast | 0.12 | Indonesia | 0.17 | Wainyapu | INW | 0.20 |
| Below 30N | 0.13 | Southeast | 0.12 | Malaysia | 0.11 | Kuala Lumpur | KLP | 0.13 |
| Below 30N | 0.13 | Southeast | 0.12 | Malaysia | 0.11 | Tambun | MAT | 0.09 |
| Below 30N | 0.13 | Southeast | 0.12 | Thailand | 0.08 | Chanthaburi | CHA | 0.08 |
| Below 30N | 0.13 | Southeast | 0.12 | Thailand | 0.08 | Kanchanaburi | KAC | 0.08 |
| Below 30N | 0.13 | Southeast | 0.12 | Thailand | 0.08 | Lampang | LAM | 0.09 |
| Below 30N | 0.13 | Southeast | 0.12 | Thailand | 0.08 | Sisaket | SSK | 0.08 |
| Below 30N | 0.13 | Southeast | 0.12 | Vietnam | 0.12 | Hanoi | HAN | 0.10 |
| Below 30N | 0.13 | Southeast | 0.12 | Vietnam | 0.12 | Ho Chi Minh City | HOC | 0.10 |
| Below 30N | 0.13 | Southeast | 0.12 | Vietnam | 0.12 | Quy Nhon City | QNC | 0.15 |

```
# Initialize Word document
doc <- 
  read_docx() |>
  body_add_flextable(value = flex_table)

# Define the output path with 'here' library
output_path <- here(
  "output",
  "fst", 
  "fst_r2_0.1_SNPS.docx"
  )

# Save the Word document
print(doc, target = output_path)
```
