# Supplementary material for: A genotyping array for the globally invasive vector mosquito, Aedes albopictus
Source: Parasit Vectors. 2024 Mar 4;17:106. doi: 10.1186/s13071-024-06158-z (PMC10910840; doi:10.1186/s13071-024-06158-z)
Supplement: Supplementary file 16 — Additional file 16. Create map with samples. [file 13071_2024_6158_MOESM16_ESM.html]

Aedes albopictus SNP chip - Maps with sampling sites.


# Aedes albopictus SNP chip - Maps with sampling sites.

#### Luciano V Cosme

#### 2023-10-24

- 1. All samples

Libraries

```
library(ggplot2)
library(tidyverse)
library(here)
library(dplyr)
library(colorout)
library(extrafont)
library(scales)
library(stringr)
library(ggtext)
library(rnaturalearth)
library(rnaturalearthdata)
library(ggrepel)
library(Cairo)
library(gstudio)
library(ggmap)
```

## 1. All samples

Clear memory and environment

```
# Clear entire environment
rm(list = ls())
# Forcefully trigger garbage collection
gc()
```

```
##           used  (Mb) gc trigger  (Mb) limit (Mb) max used  (Mb)
## Ncells 4099084 219.0    7028130 375.4         NA  5989809 319.9
## Vcells 5983467  45.7   12255594  93.6      32768 10146326  77.5
```

```
# Enter your key here
register_google(key = "PUT_YOUR_KEY_HERE", write = F)
```

Load the data

```
chip <- readRDS(here("output","maps","sampling_loc.rds"))
                 
wgs<-read.table(here("output","maps","wgs.txt"), header = T, sep = "\t")
```

Check wgs

```
head(wgs)
```

```
##                                City Years label   color  gb
## 1                   Limbe, Cameroon  2018  LIMw #cc41c0 WGS
## 2             Le Tampon, La Reunion  2017  TAMw #cc41c0 WGS
## 3 Puerto Iguazu-Misiones, Argentina  2018  PUEw #cc41c0 WGS
## 4                    Maceio, Brazil  2018  MACw #cc41c0 WGS
## 5                    Cali, Colombia  2013  CALw #cc41c0 WGS
## 6            Jardin Panteon, Mexico  2017  JARw #cc41c0 WGS
```

Get coordinates

```
loc<-geocode(wgs$City)
loc2<-as.data.frame(loc)
wgs2<- cbind(wgs,loc2)
```

```
# Rename
wgs2 <- wgs2 |>
  dplyr::rename(
    Longitude = lon, 
    Latitude = lat,
    Abbreviation = label
    ) |>
  dplyr::select(
    Abbreviation, Longitude, Latitude
  ) |>
  mutate(tech = "WGS")


head(wgs2)
```

```
saveRDS(wgs2, here("output", "maps", "wgs.rds"))
```

```
wgs2 <- readRDS(here("output", "maps", "wgs.rds"))
head(wgs2)
```

```
##   Abbreviation   Longitude   Latitude tech
## 1         LIMw    9.195443   4.022466  WGS
## 2         TAMw   55.515318 -21.279044  WGS
## 3         PUEw  -54.578599 -25.597164  WGS
## 4         MACw  -35.735217  -9.666042  WGS
## 5         CALw  -76.531985   3.451647  WGS
## 6         JARw -101.216730  21.468961  WGS
```

Check chip

```
head(chip)
```

```
## # A tibble: 6 × 6
##   Pop_City   Country  Latitude Longitude Region Abbreviation
##   <chr>      <chr>       <dbl>     <dbl> <chr>  <chr>       
## 1 Gelephu    Bhutan       26.9      90.5 Asia   GEL         
## 2 Phnom Penh Cambodia     11.6     105.  Asia   CAM         
## 3 Hainan     China        19.2     110.  Asia   HAI         
## 4 Yunnan     China        24.5     101.  Asia   YUN         
## 5 Hunan      China        27.6     112.  Asia   HUN         
## 6 Bengaluru  India        13.0      77.6 Asia   BEN
```

We have some extra localities. Let’s get the populations we have from
a fam file

```
fam_file <- here(
  "output", "populations", "snps_sets", "r2_0.1.fam"
)

# Read the .fam file
fam_data <- read.table(fam_file, 
                       header = FALSE,
                       col.names = c("FamilyID", "IndividualID", "PaternalID", "MaternalID", "Sex", "Phenotype"))


fam_data <- fam_data |>
  distinct(FamilyID, .keep_all = TRUE)
# View the first few rows
head(fam_data)
```

```
##   FamilyID IndividualID PaternalID MaternalID Sex Phenotype
## 1      OKI         1001          0          0   2        -9
## 2      HAI         1053          0          0   0        -9
## 3      YUN         1089          0          0   1        -9
## 4      KAT         1103          0          0   2        -9
## 5      TAI         1161          0          0   2        -9
## 6      HUN         1169          0          0   0        -9
```

Now merge with our chip data

```
# Join with sampling_loc to get sampling localities
chip2 <- chip |>
  inner_join(fam_data, by = c("Abbreviation" = "FamilyID"))

chip2 <- chip2 |>
  distinct(Abbreviation, .keep_all = TRUE) |>
  dplyr::select(
    Abbreviation, Longitude, Latitude
  )

chip2 <- chip2 |>
  dplyr::select(
    Abbreviation, Longitude, Latitude
  ) |>
  mutate(tech = "CHIP")

head(chip2)
```

```
## # A tibble: 6 × 4
##   Abbreviation Longitude Latitude tech 
##   <chr>            <dbl>    <dbl> <chr>
## 1 GEL               90.5     26.9 CHIP 
## 2 CAM              105.      11.6 CHIP 
## 3 HAI              110.      19.2 CHIP 
## 4 YUN              101.      24.5 CHIP 
## 5 HUN              112.      27.6 CHIP 
## 6 BEN               77.6     13.0 CHIP
```

Bind objects

```
data <- rbind(wgs2, chip2)

# Remove the "w"
data$Abbreviation <- gsub("w$", "", data$Abbreviation)

head(data)
```

```
##   Abbreviation   Longitude   Latitude tech
## 1          LIM    9.195443   4.022466  WGS
## 2          TAM   55.515318 -21.279044  WGS
## 3          PUE  -54.578599 -25.597164  WGS
## 4          MAC  -35.735217  -9.666042  WGS
## 5          CAL  -76.531985   3.451647  WGS
## 6          JAR -101.216730  21.468961  WGS
```

```
# Identify localities with both "CHIP" and "WGS".
both_techs_localities <- data |>
  group_by(Abbreviation) |>
  summarise(both = n_distinct(tech) > 1) |>
  filter(both) |>
  pull(Abbreviation)

# Add a new column to your data
data$both_techs <- ifelse(data$Abbreviation %in% both_techs_localities, "Both", as.character(data$tech))


world <- ne_countries(scale = "medium", returnclass = "sf")

source(
  here(
    "scripts", "analysis", "my_theme2.R"
  )
)

# Plot
ggplot() +
  geom_sf(data = world, fill = "#e2f5eb", color = "gray") +
  geom_point(
    data = data,
    aes(x = Longitude, y = Latitude, color = both_techs),
    size = 0.3,
    show.legend = FALSE
  ) +
  geom_text_repel(
    data = data,
    aes(
      x = Longitude, 
      y = Latitude, 
      label = Abbreviation,
      color = both_techs  # Use the both_techs column directly for label colors
    ),
    size = 2,
    box.padding = unit(0.01, "lines"),
    max.overlaps = 50,
    show.legend = FALSE
  ) +
  scale_color_manual(values = c("Both" = "blue", "CHIP" = "red", "WGS" = "black")) +
  labs(color = "Genotyped with:") +
  coord_sf(xlim = c(-130, 180), ylim = c(-60, 80)) +
  my_theme() +
  theme(legend.position = "top",
        axis.text.y = element_text(margin = margin(
          t = 0,
          r = 10,
          b = 0,
          l = 0
        )))
```

```
ggsave(
  here("output", "maps", "samples.pdf"),
  width  = 8,
  height = 5,
  units  = "in",
  device = cairo_pdf
)
```

Zoom

```
# Plot
ggplot() +
  geom_sf(data = world, fill = "#e2f5eb", color = "gray") +
  geom_point(
    data = data,
    aes(x = Longitude, y = Latitude, color = both_techs),
    size = 0.3,
    show.legend = FALSE
  ) +
  geom_text_repel(
    data = data,
    aes(
      x = Longitude, 
      y = Latitude, 
      label = Abbreviation,
      color = both_techs  # Use the both_techs column directly for label colors
    ),
    size = 2,
    box.padding = unit(0.01, "lines"),
    max.overlaps = 50,
    show.legend = FALSE
  ) +
  scale_color_manual(values = c("Both" = "blue", "CHIP" = "red", "WGS" = "black")) +
  labs(color = "Genotyped with:") +
  coord_sf(xlim = c(60, 160), ylim = c(-10, 60)) +
  my_theme() +
  theme(legend.position = "top",
        axis.text.y = element_text(margin = margin(
          t = 0,
          r = 10,
          b = 0,
          l = 0
        )))
```

```
ggsave(
  here("output", "maps", "samples_zoom.pdf"),
  width  = 8,
  height = 5,
  units  = "in",
  device = cairo_pdf
)
```

```
library(ggplot2)
library(sf)
```

```
## Linking to GEOS 3.10.2, GDAL 3.4.2, PROJ 8.2.1; sf_use_s2() is TRUE
```

```
# Filter countries for each region
east_asia_countries <- c("China", "Mongolia", "South Korea", "North Korea", "Japan", "Taiwan")
south_asia_countries <- c("India", "Pakistan", "Bangladesh", "Nepal", "Bhutan", "Sri Lanka", "Maldives")
southeast_asia_countries <- c("Myanmar", "Thailand", "Cambodia", "Laos", "Vietnam", "Malaysia", "Singapore", "Indonesia", "Brunei", "Philippines", "East Timor")

east_asia <- world[world$name %in% east_asia_countries, ]
south_asia <- world[world$name %in% south_asia_countries, ]
southeast_asia <- world[world$name %in% southeast_asia_countries, ]

# Your ggplot code
ggplot() +
  geom_sf(data = world,
          fill = "#e2f5eb",
          color = "gray") +
  geom_sf(data = east_asia, aes(fill = "East Asia")) +
  geom_sf(data = south_asia, aes(fill = "South Asia")) +
  geom_sf(data = southeast_asia, aes(fill = "Southeast Asia")) +
  geom_point(
    data = data,
    aes(x = Longitude, y = Latitude, color = both_techs),
    size = 0.3,
    show.legend = FALSE
  ) +
  geom_text_repel(
    data = data,
    aes(
      x = Longitude,
      y = Latitude,
      label = Abbreviation,
      color = both_techs
    ),
    size = 2,
    box.padding = unit(0.01, "lines"),
    max.overlaps = 50,
    show.legend = FALSE
  ) +
  scale_fill_manual(values = c(
    "East Asia" = "#faaea1",
    "South Asia" = "#95f5a8",
    "Southeast Asia" = "#b2c7ff"
  )) +
  scale_color_manual(values = c(
    "Both" = "blue",
    "CHIP" = "red",
    "WGS" = "black"
  )) +
  labs(fill = "Regions", color = "Genotyped with:") +
  coord_sf(xlim = c(60, 160), ylim = c(-10, 60)) +
  my_theme() +
  theme(legend.position = "top",
        axis.text.y = element_text(margin = margin(
          t = 0,
          r = 10,
          b = 0,
          l = 0
        )))
```

```
# Save your ggplot
ggsave(
  here("output", "maps", "samples_zoom.pdf"),
  width  = 8,
  height = 5,
  units  = "in",
  device = cairo_pdf
)
```
