# Supplementary material for: A genotyping array for the globally invasive vector mosquito, Aedes albopictus
Source: Parasit Vectors. 2024 Mar 4;17:106. doi: 10.1186/s13071-024-06158-z (PMC10910840; doi:10.1186/s13071-024-06158-z)
Supplement: Supplementary file 17 — Additional file 17. Microsoft Word file with supplemental methods. [file 13071_2024_6158_MOESM17_ESM.docx]

**Additional file methods**

**Species identification PCR**

PCR reactions were conducted in 13 μl mixtures, consisting of 2.6 μL of 5 × GoTaq Flexi PCR buffer, 1.1 μL of MgCl_2_ (7.5 mM), 1.1 μL of dNTPs (2.5 mM each), 0.5 μL of each primer (10 mM), 0.1 μL of Taq DNA polymerase (Promega GoTaq), 6.1 μL of water, and 1 μL template DNA. The amplification conditions were as follows: an initial denaturation at 94 °C for 3 minutes, followed by 35 cycles of 94 °C for 20 seconds, 55 °C for 45 seconds, and 70 °C for 2 minutes, and a final extension at 72 °C for 5 minutes. PCR products were run on a 1% agarose gel by electrophoresis to confirm amplification and then purified using magnetic beads before sequencing on an ABI 3730xl DNA Analyzer (Applied Biosystems) at the DNA Analysis Facility on Science Hill at Yale University. Sequences were then trimmed and aligned in Geneious v 11.1.5.

**WGS data**

NCBI SRA database under project ID XXXXXXX.

**SNP mining from WGS Data**

We aligned the paired-end reads to the AalbF3 genome assembly ^11^ using BWA-MEM version 0.7.17 ^12^. We discarded the unmapped reads as well as those with mapping quality below a mapQ of 30 using SAMtools version 1.9 ^13^. Next, we used SAMtools to merge and sort the paired- and single-end pseudoreads read alignments into a single BAM file to be used in subsequent analyses. First, we used GATK version 3.8.0 ^14^ to perform realignments around indels. Second, we used Picard tools version 2.9.0 ^15^ to remove optical and PCR duplicates. Third, we generated an uncompressed BCF using SAMtools mpileup version 1.3.1 ^13^ with indel calling disabled, skipping bases with base quality less than 30, and with map quality adjustment set to 30. Fourth, we converted it to a VCF file using bcftools version 1.5Aff ^13^. We filtered out low quality SNPs with SNPcleaner version 2.4.1 ^16^ and removed sites that had a total depth across all individuals less than 1,500 reads or had less than 10 individuals with at least 2 reads each. Finally, additional sites were filtered out based on the default settings within the SNPcleaner script. We obtained a set of robust sites common for all, comprised of sites that passed all our filtering thresholds. We restrict our analyses to these robust sites using the option -sites of ANGSD version 0.929-21 ^17^. Within ANGSD we used uniquely mapped reads with minimum map quality and base quality thresholds of 30 and 20, respectively.

**Genotyping Procedure**

We used a desktop computer with two Intel Xeon CPUs E5-2637 v3@3.50GHs and 256Gb of memory to perform our analysis (Affymetrix recommends 32Gb of memory). Overall, the “Best Practices Workflow” performs quality control (QC) analysis for samples and plates, genotypes those samples that pass the pre-defined QC thresholds, and then categorizes the probe sets to identify those whose genotypes are recommended for statistical test in the study. We ran the off- target variants (OTV) caller algorithm to identify miscalled clusters. The OTV caller function performs post-processing analysis to identify miscalled clustering and identify which samples should be in the OTV cluster and which samples should remain in the AA, AB, or BB clusters. Samples in the OTV cluster are re-labeled as OTV. We also obtained the SSToll from Affymetrix to create population specific priors.
