# Supplementary material for: A genotyping array for the globally invasive vector mosquito, Aedes albopictus
Source: Parasit Vectors. 2024 Mar 4;17:106. doi: 10.1186/s13071-024-06158-z (PMC10910840; doi:10.1186/s13071-024-06158-z)
Supplement: Supplementary file 19 — Additional file 19. PDF file from Axion Suite for the crosses genotype call. [file 13071_2024_6158_MOESM19_ESM.pdf]

Analysis Summary

- **Batch Name:** new_priors_May_30th_2023
- **Array Package Name:** Axiom_Aealbo.r1
- **Array Display Name:** Axiom_Aealbo.r1
- **Workflow Type:** Best Practices Workflow
- **Date Created:** 5/30/2023 10:35:53 AM

Sample Summary

- Number of input samples: 152
- Samples passing DQC: 152 out of 152
- Samples passing DQC and QC CR: 152 out of 152
- Samples passing DQC, QC CR and Plate QC: 152 out of 152 (100%)
- Number of failing samples: 0
- Number of Samples Genotyped: 152
- Average QC CR for the passing samples: 99.604
- Inbred Penalty Applied: no
- Prior Model File: Axiom_Aealbo.r1.20230526.models
- SNP List File: none

Plate QC Summary

Plate Barcode	Result	Number of files in a batch	Number of files failing dish QC	Number of files failing QC Call rate	Number of samples that passed	Percent of passing samples	Average call rate for passing samples	Filtered Call Rate
5513584436722042923428	PASSED	88	0	0	88	100	99.595	97.479
5513584436722042923430	PASSED	64	0	0	64	100	99.617	97.482

ProbeSet Metrics Summary

- Number of ProbeSets: 175396

ConversionType	Count	Percentage
PolyHighResolution	84963	48.441
Other	31735	18.093
NoMinorHom	20613	11.752
OTV	19164	10.926
MonoHighResolution	18388	10.484
CallRateBelowThreshold	533	0.304

Marker Metrics Summary

- Number of Markers: 175396
- Number of BestandRecommended: 123964
- Percent BestandRecommended: 70.677

ConversionType	Count	Percentage
PolyHighResolution	84963	48.441
Other	31735	18.093
NoMinorHom	20613	11.752
OTV	19164	10.926
MonoHighResolution	18388	10.484
CallRateBelowThreshold	533	0.304

Sample QC Thresholds

- DQC: ≥ 0.82
- QC call_rate: ≥ 95
- Average call rate for passing samples: ≥ 95
- Percent of passing samples: ≥ 90

SNP QC Thresholds

- species-type: Diploid
- cr-cutoff: ≥ 90
- fld-cutoff: ≥ 3.6
- het-so-cutoff: ≥ -0.1
- het-so-XChr-cutoff: ≥ -0.1
- het-so-ZChr-cutoff: ≥ -0.1
- het-so-otv-cutoff: ≥ -0.3
- hom-ro-1-cutoff: ≥ 0.6
- hom-ro-2-cutoff: ≥ 0.3
- hom-ro-3-cutoff: ≥ -0.9
- hom-ro: true
- num-minor-allele-cutoff: ≥ 2
- hom-ro-hap-1-XChr-cutoff: ≥ 0.1
- hom-ro-hap-1-MTChr-cutoff: ≥ 0.4
- hom-ro-hap-1-ZChr-cutoff: ≥ 0.1
- hom-ro-hap-2-XChr-cutoff: ≥ 0.05
- hom-ro-hap-2-MTChr-cutoff: ≥ 0.2
- hom-ro-hap-2-ZChr-cutoff: ≥ 0.05
- aaf-XChr-cut: < 0.36
- aaf-ZChr-cut: < 0.36
- fld-XChr-cut: ≥ 4
- fld-ZChr-cut: ≥ 4
- homfld-XChr-cut: ≥ 6.5
- homfld-ZChr-cut: ≥ 6.5
- homfld-YChr-cut: ≥ 6.5
- homfld-WChr-cut: ≥ 6.5
- min-YChr-samples-cut: ≥ 5
- min-WChr-samples-cut: ≥ 5
- priority-order: PolyHighResolution, NoMinorHom, MonoHighResolution, OTV, UnexpectedGenotypeFreq, CallRateBelowThreshold, Other, OtherMA
- recommended: PolyHighResolution, NoMinorHom, MonoHighResolution, Hemizygous
- y-restrict: ≤ 0.2

- min-genotype-freq-samples: ≥ 20
- genotype-p-value-cutoff: ≥ 1E-06

Multi-Allelic SNP QC Thresholds

- HomMMA-cutoff: > 10
- FLD-MA-cutoff: > 5.2
- FLD-MA-2-cutoff: > 5.2
- Min-FLD-MA-cutoff: > 0
- Min-FLD-MA-2-cutoff: > 0
- HetSO-MA-2-cutoff: > -0.1
- HomRO-MA-cutoff: > 0.5
- HomRO-MA-2-cutoff: > 0.5
- HomRO-MA-1-cutoff: > 1
- priority-order-MA: PolyHighResolution, NoMinorHom, MonoHighResolution, Hemizygous, UnexpectedGenotypeFreq, CallRateBelowThreshold, Other, OtherMA
- Best-CR-MA-cutoff: > 90
