# Supplementary material for: A genotyping array for the globally invasive vector mosquito, Aedes albopictus
Source: Parasit Vectors. 2024 Mar 4;17:106. doi: 10.1186/s13071-024-06158-z (PMC10910840; doi:10.1186/s13071-024-06158-z)
Supplement: Supplementary file 20 — Additional file 20. PDF file from Axion Suite for the wild genotype call. [file 13071_2024_6158_MOESM20_ESM.pdf]

## Analysis Summary

- **Batch Name:** manuscript_august10_2023
- **Array Package Name:** Axiom_Aealbo.r1
- **Array Display Name:** Axiom_Aealbo.r1
- **Workflow Type:** Best Practices Workflow
- **Date Created:** 8/10/2023 10:52:37 AM

## Sample Summary

- Number of input samples: 266
- Samples passing DQC: 245 out of 266
- Samples passing DQC and QC CR: 243 out of 266
- Samples passing DQC, QC CR and Plate QC: 243 out of 266 (91.353%)
- Number of failing samples: 23
- Number of Samples Genotyped: 243
- Average QC CR for the passing samples: 98.744
- Inbred Penalty Applied: no
- Prior Model File: Axiom_Aealbo.r1.20230809_manuscript.models.models
- SNP List File: none

## Plate QC Summary

| Plate Barcode | Result | Number of files in a batch | Number of files failing dish QC | Number of files failing QC Call rate | Number of samples that passed | Percent of passing samples | Average call rate for passing samples | Filtered Call Rate |
|---|---|---|---|---|---|---|---|---|
| 5513584436722042923427 | PASSED | 45 | 7 | 1 | 37 | 82.222 | 98.926 | 96.325 |
| 5513584452328010824440 | PASSED | 53 | 8 | 0 | 45 | 84.906 | 98.877 | 96.93 |
| 5513584452328010824441 | PASSED | 24 | 0 | 0 | 24 | 100 | 99.127 | 96.844 |
| 5513584452328010824444 | PASSED | 31 | 0 | 0 | 31 | 100 | 98.969 | 96.861 |
| 5513584452328010824445 | PASSED | 32 | 1 | 1 | 30 | 93.75 | 97.929 | 95.551 |
| 5513584452328010824447 | PASSED | 32 | 5 | 0 | 27 | 84.375 | 98.749 | 96.734 |
| 5513584452328010824448 | PASSED | 37 | 0 | 0 | 37 | 100 | 98.639 | 96.521 |
| 5513584478174020425867 | PASSED | 12 | 0 | 0 | 12 | 100 | 98.69 | 96.083 |

## ProbeSet Metrics Summary

- Number of ProbeSets: 175396

| ConversionType | Count | Percentage |
|---|---|---|
| PolyHighResolution | 105117 | 59.931 |
| Other | 35588 | 20.29 |
| OTV | 23600 | 13.455 |
| NoMinorHom | 7590 | 4.327 |
| MonoHighResolution | 2639 | 1.505 |
| CallRateBelowThreshold | 862 | 0.491 |

## Marker Metrics Summary

- Number of Markers: 175396
- Number of BestandRecommended: 115346
- Percent BestandRecommended: 65.763

| ConversionType | Count | Percentage |
|---|---|---|
| PolyHighResolution | 105117 | 59.931 |
| Other | 35588 | 20.29 |
| OTV | 23600 | 13.455 |
| NoMinorHom | 7590 | 4.327 |
| MonoHighResolution | 2639 | 1.505 |
| CallRateBelowThreshold | 862 | 0.491 |

## Sample QC Thresholds

- DQC: ≥ 0.82
- QC call_rate: ≥ 90
- Average call rate for passing samples: ≥ 90
- Percent of passing samples: ≥ 90

## SNP QC Thresholds

- species-type: Diploid
- cr-cutoff: ≥ 90
- fld-cutoff: ≥ 3.6
- het-so-cutoff: ≥ -0.1
- het-so-XChr-cutoff: ≥ -0.1
- het-so-ZChr-cutoff: ≥ -0.1
- het-so-otv-cutoff: ≥ -0.3
- hom-ro-1-cutoff: ≥ 0.6
- hom-ro-2-cutoff: ≥ 0.3
- hom-ro-3-cutoff: ≥ -0.9
- hom-ro: true
- num-minor-allele-cutoff: ≥ 2
- hom-ro-hap-1-XChr-cutoff: ≥ 0.1
- hom-ro-hap-1-MTChr-cutoff: ≥ 0.4
- hom-ro-hap-1-ZChr-cutoff: ≥ 0.1
- hom-ro-hap-2-XChr-cutoff: ≥ 0.05
- hom-ro-hap-2-MTChr-cutoff: ≥ 0.2
- hom-ro-hap-2-ZChr-cutoff: ≥ 0.05
- aaf-XChr-cut: < 0.36
- aaf-ZChr-cut: < 0.36
- fld-XChr-cut: ≥ 4
- fld-ZChr-cut: ≥ 4
- homfld-XChr-cut: ≥ 6.5
- homfld-ZChr-cut: ≥ 6.5
- homfld-YChr-cut: ≥ 6.5
- homfld-WChr-cut: ≥ 6.5
- min-YChr-samples-cut: ≥ 5
- min-WChr-samples-cut: ≥ 5
- priority-order: PolyHighResolution, NoMinorHom, MonoHighResolution, OTV, UnexpectedGenotypeFreq, CallRateBelowThreshold, Other, OtherMA
- recommended: PolyHighResolution, NoMinorHom, MonoHighResolution, Hemizygous
- y-restrict: ≤ 0.2

- min-genotype-freq-samples: ≥ 20
- genotype-p-value-cutoff: ≥ 1E-06

## Multi-Allelic SNP QC Thresholds

- HomMMA-cutoff: > 10
- FLD-MA-cutoff: > 5.2
- FLD-MA-2-cutoff: > 5.2
- Min-FLD-MA-cutoff: > 0
- Min-FLD-MA-2-cutoff: > 0
- HetSO-MA-2-cutoff: > -0.1
- HomRO-MA-cutoff: > 0.5
- HomRO-MA-2-cutoff: > 0.5
- HomRO-MA-1-cutoff: > 1
- priority-order-MA: PolyHighResolution, NoMinorHom, MonoHighResolution, Hemizygous, UnexpectedGenotypeFreq, CallRateBelowThreshold, Other, OtherMA
- Best-CR-MA-cutoff: > 90
