# Supplementary material for: A genotyping array for the globally invasive vector mosquito, Aedes albopictus
Source: Parasit Vectors. 2024 Mar 4;17:106. doi: 10.1186/s13071-024-06158-z (PMC10910840; doi:10.1186/s13071-024-06158-z)
Supplement: Supplementary file 22 — Additional file 22. Microsoft Word file with the literature review of the population structure of Ae. albopictus in Asia. [file 13071_2024_6158_MOESM22_ESM.docx]

**Supplemental File S22.** Literature review of selected studies on the population structure of *Ae. albopictus* in Asia

We selected studies for which the populations structure of *Ae. albopictus* in Asia was examined. Some studies included samples from Asia but did not analyze the population structure in Asia or focused only on a small area and these were not included in our review. We also included some studies with sampling in the Indian Ocean and Asia. The studies included use different types of genetic markers, which are listed in Figures S26 and S27. We also plotted the countries with sampling locations for each study to help facilitate our interpretation (Figure S28). We first briefly describe each study's conclusion chronologically and compare it to our findings.

Black et al., 1988 ^1^ studied *Ae. albopictus* in Malaysia and Borneo using allozymes as markers. Their results were inconclusive, but indicated two genetic clusters, finding that Peninsular Malaysian and Borneo populations were strongly genetically differentiated, primarily due to differentiation at the a-Gpdh (alpha glyceraldehyde-3-phosphate dehydrogenase) locus. In our study, we found a similar pattern, with the western region of Malaysia belonging to a genetic cluster and southern Malaysia and Indonesia assigned to a different cluster (Figure 6, "yellow" and "blue" clusters).

Kambhampati et al., 1991 ^2^ analyzed samples from China, India, Japan, Malaysia, and Sri Lanka using allozymes. They found five genetic clusters, with the samples from Japan and China in one cluster, while samples from India, Sri Lanka, Malaysia, and Borneo had their own cluster in the PCA space. Although our sampling site were different, we also observed five clusters, but in our analyses, we found two clusters in China, which are distinct from Japan (Figure 6). We also identified a unique cluster in Indonesia. Although the number of the ancestral groups are similar between the studies, the sampling is not similar, and the geographical distribution of the cluster is different.

Urbanelli et al., 2000 ^3^ studied Japan and Indonesia with allozymes. They found two genetic clusters, separating Japan and Indonesia, and observed genetic isolation within Indonesia, supporting our results. Birungi and Munstermann, 2002 ^4^ analyzed samples from Malaysia, Indonesia, and a laboratory strain from Japan using mtDNA ND5. They found one genetic cluster, indicating a genetic similarity between Malaysia, Japan, and Indonesia, in disagreement with our findings, but perhaps the laboratory strain biased their analysis.

Chareonviriyaphap et al., 2004 ^5^ focused on three regions of Thailand using allozymes. They found one genetic cluster, but they also noted higher differences in the population from Bangkok. We also found that our Thailand samples formed one cluster (Figure 6), and likewise detected isolation by distance (Figure S19). While Mousson et al., 2005 ^6^ studied various countries, including Thailand, La Reunion, Vietnam, Cambodia, and Madagascar, using mtDNA COI, they identified one genetic cluster, indicating one phylogenetic group. Delatte et al., 2011 sampled multiple locations, mainly in the Indian Ocean, using mtDNA COI. Their conclusion was four genetic clusters but had no overlap with our sampling localities.

Porretta et al., 2012 ^7^ investigated Bhutan, China, Japan, Taiwan, Thailand, and Vietnam using tmtDNA COI (cytochrome c oxidase subunit I ) and ND5 (nicotinamide adenine dinucleotide dehydrogenase 5) and found one genetic cluster, while we identified four genetic clusters in these countries (Figure 6). Beebe et al., 2013 ^8^ looked at Australia, Papua New Guinea, Torres Strait Islands, Indonesia, and Timor-Leste using microsatellites and mtDNA COI. They found five genetic clusters and identified a single genetic cluster over Indonesia. We only had samples from Indonesia, which formed their genetic cluster ("blue" cluster in Figure 6). This could indicate up to nine ancestral groups in Asia and the Indian Ocean since both studies identified the Indonesian genetic cluster but did not have any additional overlapping locations.

Zhong et al., 2013 ^9^ studied China, Japan, Singapore, and Taiwan using mtDNA COI, finding six genetic clusters, grouping Southern China and Taiwan into three distinct genetic groups. We identified two genetic clusters in this area (Figure 6). Gupta and Preet, 2014 conducted their study in India using RAPD markers. They found one genetic cluster, but larval samples fell into two clusters based on habitat, as we observed one major genetic cluster (“yellow” cluster Figure 6). Zawani et al., 2014's ^10^ research on Malaysia using mtDNA COI led to a conclusion of one genetic cluster, similar to our findings while the sampling localities were different.

Using mtDNA COI, Ismail et al., 2015 ^11^, analyzed samples from multiple countries, including Malaysia, Bhutan, China, India, Indonesia, Japan, Taiwan, Thailand, and Vietnam. They concluded the occurrence of five genetic clusters, identifying five monophyletic groups with varying country compositions, supporting our results.

Kotsakiozi et al., 2017 ^12^ focused on Japan, Malaysia, Singapore, and Vietnam using ddRAD. Their results showed one genetic cluster for the native range, while we identified three genetic clusters in the same area. Gao et al., 2020 conducted their study in China using microsatellites and mtDNA COI, determining two genetic clusters, which agrees with our results.

Schmidt et al., 2020 ^13^ sampled various Asian countries and found five genetic clusters using ddRAD, and their clusters share a similar distribution to ours. Duong et al., 2021’s ^14^ analysis of Vietnamese samples using mtDNA CO1 led to a conclusion of three genetic clusters, and identified latitudinal isolation by distance. In Vietnam we observed isolation by distance (Figure S19) and identified two distinct clusters but detected admixture with the cluster from South China and Taiwan for all Vietnamese sites except Qhui nhon City (Figure 6).

Shan et al., 2022 ^15^ focused on China using microsatellites. They found three genetic clsuters and did not observe isolation by distance, while we found two groups and detected isolation by distance. Sherpa et al., 2022 ^16^ studied populations from multiple Asian countries using ddRAD. They found three genetic clusters, grouping regions into tropical, subtropical temperate, and temperate clusters, aligning with our clustering pattern.

Wei et al., 2022 ^17^ focused on Chinese samples, using mtDNA COI and SNPs from ddRAD data. Their results showed seven genetic clusters, and they observed isolation by distance. We only detected two significant clusters in China, with some admixture with the Japanese cluster. We also detected isolation by distance within mainland China (Figure S19). Ma et al., 2023 ^18^ focused on China using rDNA ITS2 (ribosomal DNA internal transcribed spacer 2). They found one genetic cluster and low genetic differentiation and indicated high gene flow among the sampled sites.

Shin et al., 2023 ^19^ analyzed samples from South Korea, Japan, and Laos using microsatellites and mtDNA COI. They found that South Korea grouped into two clusters, one aligning with Japan. We did not have samples from South Korea, but the interpolation of our admixture matrices is consistent with their findings because South Korea is located between the Chinese and Japanese clusters ("red" and "green" in Figure 6). Finally, Yeo et al., 2023 ^20^ analyzed samples from Singapore using ddRAD and found three genetic clusters, observing fine structure but no isolation by distance, which could be due to the particular sampling effort that was aimed at asking if there where signals of local adaptation to different environments rather than looking at patterns of genetic differentiation over space.

**Literature**

1 Black, W. C., Ferrari, J. A., Rai, K. S. & Sprenger, D. Breeding structure of a colonising species: Aedes albopictus (Skuse) in the United States. *Heredity* **60**, 173-181 (1988). <https://doi.org:10.1038/hdy.1988.29>

2 Kambhampati, S., Black, W. C. & Rai, K. S. Geographic origin of the US and Brazilian Aedes albopictus inferred from allozyme analysis. *Heredity* **67**, 85-94 (1991). <https://doi.org:10.1038/hdy.1991.67>

3 Urbanelli, S., Bellini, R., Carrieri, M., Sallicandro, P. & Celli, G. Population structure of Aedes albopictus (Skuse): the mosquito which is colonizing Mediterranean countries. *Heredity* **84**, 331-337 (2000). <https://doi.org:10.1046/j.1365-2540.2000.00676.x>

4 Birungi, J. & Munstermann, L. E. Genetic structure of Aedes albopictus (Diptera : Culicidae) populations based on mitochondrial ND5 sequences: Evidence for an independent invasion into Brazil and United States. *Ann Entomol Soc Am* **95**, 125-132 (2002). <https://doi.org:Doi> 10.1603/0013-8746(2002)095[0125:Gsoaad]2.0.Co;2

5 Chareonviriyaphap, T., Akratanakul, P., Huntamai, S., Nettanomsak, S. & Prabaripai, A. Allozyme Patterns of Aedes albopictus, a Vector of Dengue in Thailand. *J Med Entomol* **41**, 657-663 (2004). <https://doi.org:10.1603/0022-2585-41.4.657>

6 Mousson, L. *et al.* Phylogeography of Aedes (Stegomyia) aegypti (L.) and Aedes (Stegomyia) albopictus (Skuse) (Diptera: Culicidae) based on mitochondrial DNA variations. *Genet Res* **86**, 1-11 (2005). <https://doi.org:10.1017/S0016672305007627>

7 Porretta, D., Mastrantonio, V., Bellini, R., Somboon, P. & Urbanelli, S. Glacial History of a Modern Invader: Phylogeography and Species Distribution Modelling of the Asian Tiger Mosquito Aedes albopictus. *Plos One* **7**, e44515 (2012). <https://doi.org:10.1371/journal.pone.0044515>

8 Beebe, N. W. *et al.* Tracing the Tiger: Population Genetics Provides Valuable Insights into the Aedes (Stegomyia) albopictus Invasion of the Australasian Region. *Plos Neglect Trop D* **7**, e2361 (2013). <https://doi.org:10.1371/journal.pntd.0002361>

9 Zhong, D. *et al.* Genetic Analysis of Invasive Aedes albopictus Populations in Los Angeles County, California and Its Potential Public Health Impact. *Plos One* **8**, e68586 (2013). <https://doi.org:10.1371/journal.pone.0068586>

10 Zawani, M. K., Abu, H. A., Sazaly, A. B., Zary, S. Y. & Darlina, M. N. Population genetic structure of Aedes albopictus in Penang, Malaysia. *Genet Mol Res* **13**, 8184-8196 (2014). <https://doi.org:10.4238/2014.October.7.13>

11 Ismail, N.-A. *et al.* Mitochondrial Cytochrome Oxidase I Gene Sequence Analysis of Aedes Albopictus in Malaysia. *J Am Mosquito Contr* **31**, 305-312 (2015). <https://doi.org:10.2987/moco-31-04-305-312.1>

12 Kotsakiozi, P. *et al.* Population genomics of the Asian tiger mosquito, Aedes albopictus: insights into the recent worldwide invasion. *Ecol Evol* **7**, 10143-10157 (2017). <https://doi.org:10.1002/ece3.3514>

13 Schmidt, T. L., Chung, J., Honnen, A. C., Weeks, A. R. & Hoffmann, A. A. Population genomics of two invasive mosquitoes (Aedes aegypti and Aedes albopictus) from the Indo-Pacific. *Plos Neglect Trop D* **14** (2020). <https://doi.org:ARTN> e0008463

10.1371/journal.pntd.0008463

14 Duong, C.-V., Kang, J.-H., Nguyen, V.-V. & Bae, Y.-J. Genetic Diversity and Population Structure of the Asian Tiger Mosquito (Aedes albopictus) in Vietnam: Evidence for Genetic Differentiation by Climate Region. *Genes* **12**, 1579 (2021). <https://doi.org:10.3390/genes12101579>

15 Shan, W. *et al.* Population genetic structure of Aedes albopictus (Diptera: Culicidae) in China inferred by microsatellite and association with knockdown resistance mutations. (2022).

16 Sherpa, S. *et al.* Genomic Shifts, Phenotypic Clines, and Fitness Costs Associated With Cold Tolerance in the Asian Tiger Mosquito. *Molecular Biology and Evolution* **39**, msac104 (2022). <https://doi.org:10.1093/molbev/msac104>

17 Wei, Y. *et al.* Genome-wide SNPs reveal novel patterns of spatial genetic structure in Aedes albopictus (Diptera Culicidae) population in China. *Frontiers in Public Health* **10** (2022).

18 Ma, Z. *et al.* Population genetic characterization of (Aedes albopictus) mosquitoes (Diptera: Culicidae) from the Yangtze River Basin of China based on rDNA-ITS2. *Infect Genet Evol* **113**, 105485 (2023). <https://doi.org:10.1016/j.meegid.2023.105485>

19 Shin, J., Rahman, M.-M., Kim, J., Marcombe, S. & Jung, J. Genetic Diversity of Dengue Vector Aedes albopictus Collected from South Korea, Japan, and Laos. *Insects* **14**, 297 (2023). <https://doi.org:10.3390/insects14030297>

20 Yeo, H. *et al.* Dense residential areas promote gene flow in dengue vector mosquito Aedes albopictus. *Iscience* **26** (2023).
