# Supplementary material for: A genotyping array for the globally invasive vector mosquito, Aedes albopictus
Source: Parasit Vectors. 2024 Mar 4;17:106. doi: 10.1186/s13071-024-06158-z (PMC10910840; doi:10.1186/s13071-024-06158-z)
Supplement: Supplementary file 24 — Additional file 24. Cost estimate for the chip. [file 13071_2024_6158_MOESM24_ESM.docx]

**File S24. Cost estimate for the chip**

In our manuscript, we chose not to specify the costs because they are subject to change over time, which could date the paper or provide inaccurate information to future readers. However, to address the concerns regarding the cost, we can provide some current cost figures to illustrate the cost-effectiveness of our approach.

After the initial development of the chip, the costs we incurred were as follows:

- The total cost of ordering our last five chips from Thermo Fisher was $28,800, accommodating 95 samples per chip, totaling 475 samples. It brings the cost per sample to approximately US$60.63.
- The genotyping service per chip amounted to US$1,887.88, averaging US$19.87 per sample.

Consequently, the total cost per sample comes to US$80.60. Large orders may qualify for further discounts, potentially reducing costs even more.

We also obtained an estimate for WGS sequencing at the sequencing facility at Yale University:

Our latest estimates for using WGS sequencing approximately 55 individual mosquitoes at 30x in one Illumina Novaseq S4 lane, costing around $7,000, which equates to about $127 per mosquito. Additional costs include $76 per individual for library preparation. Therefore, the total cost would be US$203 per sample.

The time factor is crucial. Assuming a week for sample processing by a sequencing facility, similar to the SNP chip, we must consider data processing time. For example, by genotyping 1000 mosquitoes using the chip, we can store and analyze the data (less than 100Gb) on a desktop computer in less than 2 hours. In contrast, WGS data (approximately 20Tb for 1000 samples) would not fit on standard lab computers and requires extensive processing, including marking PCR duplicates, indel realignments, and more, even on high-performance computing (HPC) clusters like Yale's. Storage and computational time are significant challenges with WGS.

The analysis duration for WGS also depends on the user's expertise and computing resources. An experienced user with access to extensive computing power (like 300 to 1000 CPUs at Yale's HPC) might complete the analysis within a week. However, the process could take weeks or months for someone less experienced with WGS or HPC.

In contrast, our SNP chip technology is highly efficient. We have used HPC to identify SNPs and design highly specific probes. The genotype calls are a simple correlation between expected and observed fluorescence signals, making the process extremely fast. The user-friendly software runs on Windows with a graphical interface, offering easy visualization and quality control options.

In conclusion, while precise time savings are challenging to quantify due to varying factors, our SNP chip technology offers a significantly faster and more user-friendly alternative for genotyping large sample sizes compared to WGS.
