# Supplementary material for: A genotyping array for the globally invasive vector mosquito, Aedes albopictus
Source: Parasit Vectors. 2024 Mar 4;17:106. doi: 10.1186/s13071-024-06158-z (PMC10910840; doi:10.1186/s13071-024-06158-z)
Supplement: Supplementary file 25 — Additional file 25. Supplementary Figures. [file 13071_2024_6158_MOESM25_ESM.docx]

**Additional file Figures**

| 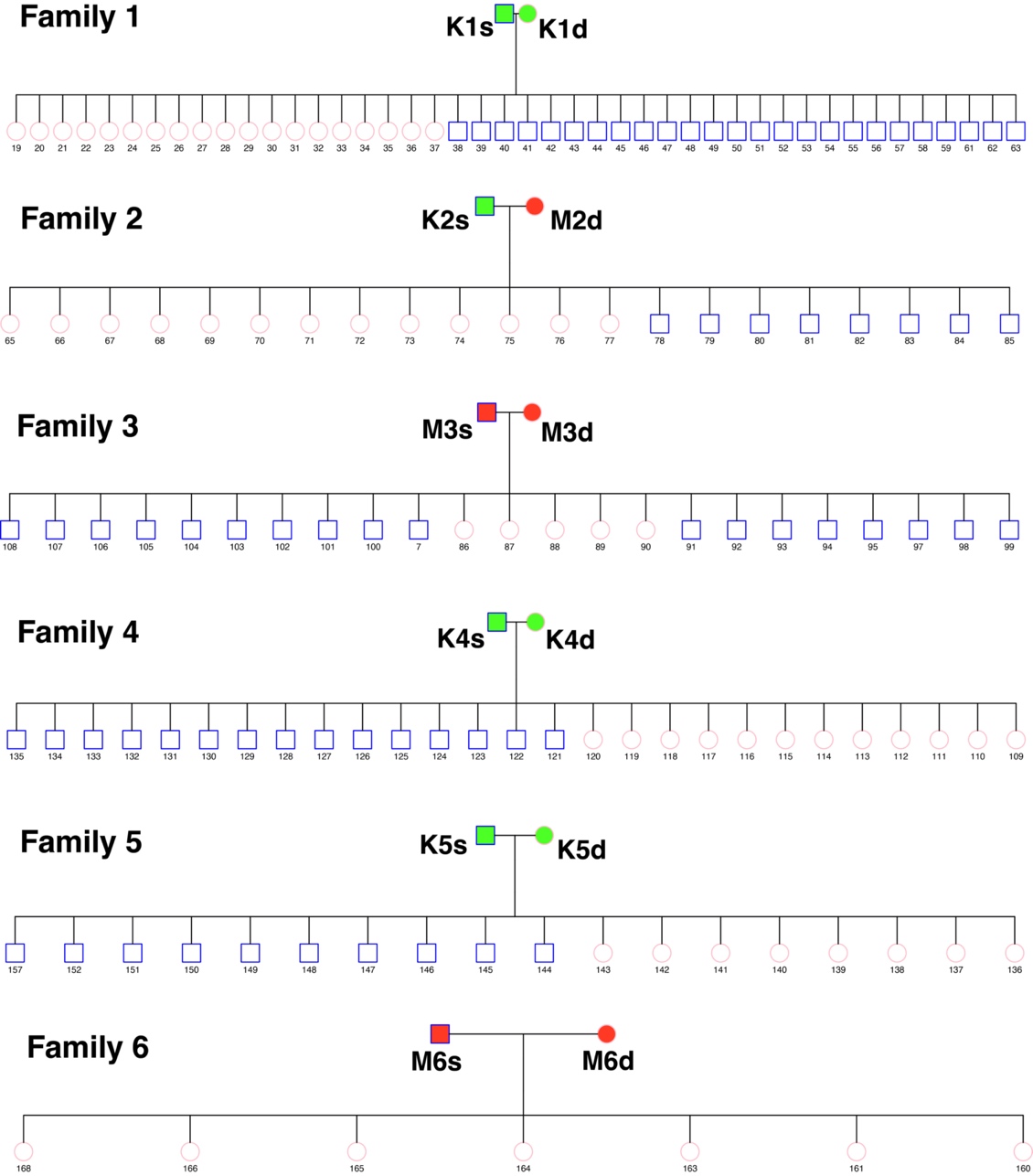 |
| --- |
| **Figure S1**. Pedigrees showing the laboratory colony crosses of *Aedes albopictus*. The squares represent males in each pedigree and the circles represent females, with horizontal lines connecting them to indicate sibling relationships. The color-coded parental labels distinguish their origin; green signifies a native range (K, Kuala Lumpur, Malaysia), while red indicates an invasive range (M, Manassas, USA). The numbers represent the unique identier of each mosquito in the family. |

| 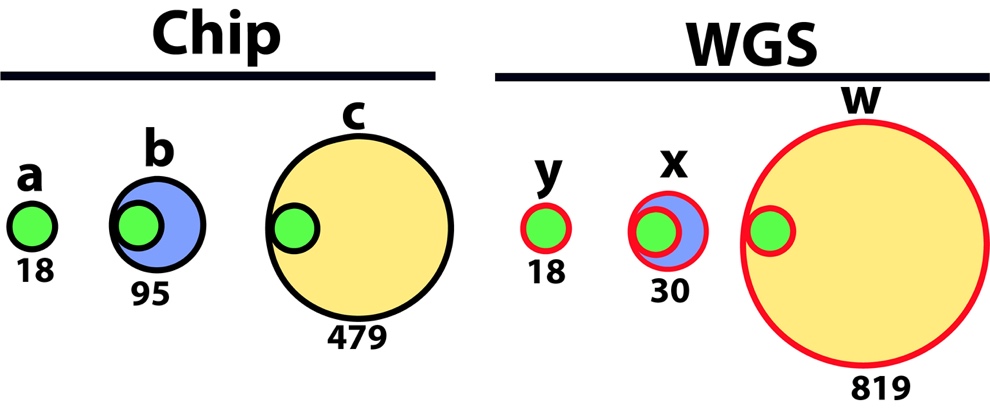 |
| --- |
| **Figure S2**. Overview of the datasets used for the WGS and SNP chip genotype calls comparisons. We used three datasets for each methodology for the genotype calls with the 18 samples from KAT (6) and SAI (12) (WGS, SNP chip). The a, b, and c datasets are to the SNP chip datasets which include 18, 95, and 479 samples, respectively. The x, y, and z datasets are the WGS datasets with 18, 30 and 819 samples, respectively. The a and y datasets include only individuals from the KAT (6) and SAI (12) populations genotyped with both technologies. The b and x datasets also include individuals from the KAT (6) and SAI (12) populations. The c and w datasets include all the samples genotyped with the SNP chip or WGS, respectively. |

| 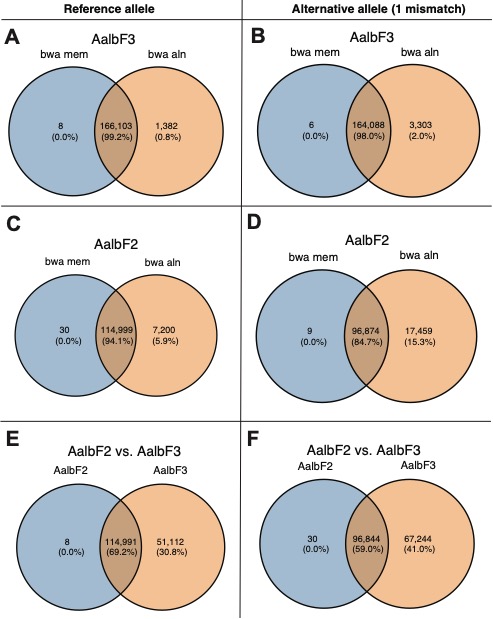 |
| --- |
| **Figure S3**. Venn diagrams comparing the probe sequence alignments with two different methods (BWA MEN and BWA ALN) using two genome assemblies (AalbF3 and AalbF2) for either the reference allele (left panels) or the alternative allele (allowing one mismatch; right panels). The numbers inside the circles represent the number of probe sequences with percentages in parenthesis. The two colors used for the Venn diagrams refer to two alignment methods (bwa mem=blue; bwa aln=orange) in panels A, B, C, and D. The last two panels (E and F) report the number of sequences mapped to one of the two alignments and their overlap for both the reference allele (E) and the alternative allele, allowing one mismatch (F).  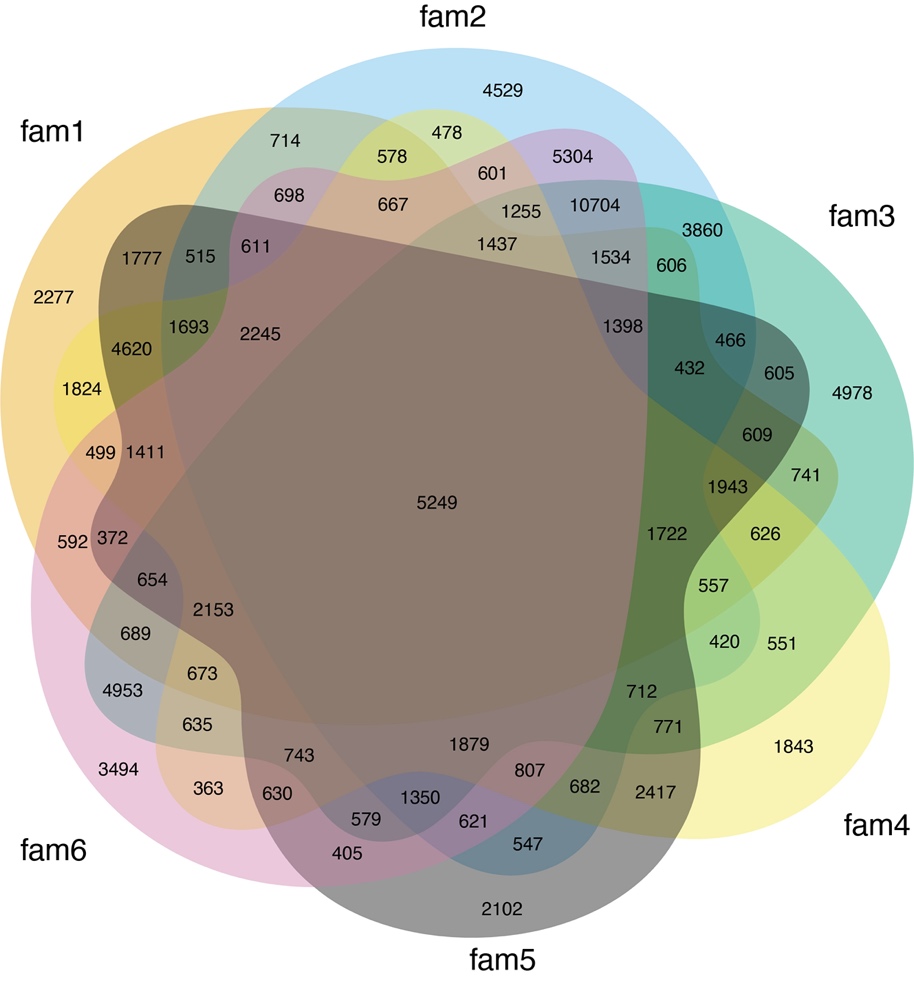 |

| **Figure S4**. Venn diagram showing the number of segregating SNPS for the six families (fam 1-fam 6) used for the laboratory crosses illustrated in Figure S1. Out of 101,376 SNPs available, only 5,249 SNPs, the ones heterozygous in at least one parent, could be used in all the families in the segregation analyses. Table S14 shows how many SNPs were tested in each family. |
| --- |

|  |
| --- |
| **Figure S5**. Comparison of the genotype calls obtained using the WGS and SNP chip platforms for two populations (KAT and SAI), for which datasets of different size were available. The categories for specific dataset comparisons are reported on the far right. The categories include the total number of SNPs (SNPs; purple bar) followed by the number of homozygous SNPs for the reference (Homozygous REF; green bar), the alternative (Homozygous ALT) alleles that did not match, and the number of heterozygous SNPs with mismatching alleles (Heterozygous). The specific dataset comparisons are indicated by letters on the far left. (Figure S2). For all comparisons, the x-axis shows the number of genotype calls (Count) in Log10 scale. Percentages of the total number of SNPs for each comparison are in parenthesis. Note that the total number of SNPs compared for each sample is different as annotated in the figure. |

|  |
| --- |
| **Figure S6.** Cumulative genotype mismatch across the same 18 samples genotyped with the SNP chip (dataset a, Figure S2) or WGS (dataset y, Figure S2). On the y-axis is the individual sample for which a mismatch was detected between the two data sets, either for the reference allele, the alternative allele, or the zygosity (left, middle, and right columns, respectively). The x-axis reports the number of SNPs in thousands (SNP Count, k). The bars show the relative number of SNPs for each category (reference, alternative, zygosity) with actual numbers and their percentages shown next to the bars. The border color of the bars reflects the amount of mismatch for that comparison: no errors (black), SNPs with errors in 1 sample (blue); SNPs with errors in 2 or more samples (orange).   \|  \| \| --- \| \| **Figure S7**. Cumulative genotype mismatch across the same 18 samples genotyped with the SNP chip (dataset a, Figure S2) or WGS (dataset y, Figure S2) for each population. Two populations were genotyped: SAI (N=12) and KAT (N=6). Symbols and conventions as in Figure S6 \| |

|  |
| --- |
| **Figure S8**. The effect of read depth per site when comparing two WGS datasets of different size (dataset y and x, with 18 and 30 samples, respectively; Figure S2). The y-axis is the mean read count (depth) for a genomic position. The x-axis is the number of individuals with mismatches (only 18 samples were present in both datasets). The numbers on top of each bar are the average number of mismatches. |

|  |
| --- |
| **Figure S9**. Pearson correlation between read counts, base quality, and genotype mismatches for the WGS data sets based on 18 and 30 individuals (dataset y and x, Figure S2). The y-axis is the type of mismatches (ALT: the number of SNPs where the alternative allele did not match, REF: the number of SNPs for which the reference allele did not match between the two data sets, Zigo: the number of SNPs that with mismatching zygosity, either homozygous or heterozygous). On the x-axis are the alignment metrics: alt_count is the mean number of reads with the alternative allele; alt_quality: is the mean base quality of the bases; ref_count: is the mean number of reads with the reference allele; ref_quality: is the mean quality of the bases; site_counts: is the mean read count for the position in consideration. The number in each square is the Pearson correlation coefficient, and the color of each square reflects the values of the coefficient (see legend on far right). Red indicates positive correlation and purple indicates negative correlation. The number of mismatches increase as the read count decreases. |

|  |
| --- |
| **Figure S10**. Pearson correlation between WGS and SNP chip metrics with the genotype mismatches using the same 18 individuals (datasets a and y, Figure S2 and Table 1). The first row shows the type of mismatches between the two datasets: Zigosity- the number of SNPs with mismatching zygosity (homozygous or heterozygous); REF- the number of SNPs for which the reference allele did not match; ALT- the number of SNPs where the alternative allele did not match. The columns show the alignment metrics and chip metrics for each comparison: alt_count is the mean number of reads with the alternative allele; alt_quality is the mean base quality of the bases; ref_count is the mean number of reads with the reference allele; ref_quality is the mean quality of the bases; site_counts is the mean read count for a given position. The chip metrics are: CR is the call rate; FLD is the Fisher Linear Discriminant; N_AA is the number of homozygous for reference allele; N_AB is the number of heterozygous SNPs; N_BB is the number of homozygous for the alternative allele; N_NC is the number of variants with no cluster; N_NoCall is the number of loci with no call; N_OTV is the number of off target variants; nMinorAllele is the number of minor alleles. The number in each square is the Pearson correlation coefficient, and the color of each square reflects the value of the coefficient (see legend on far right). Red indicates positive correlation and purple indicates negative correlation. |

| 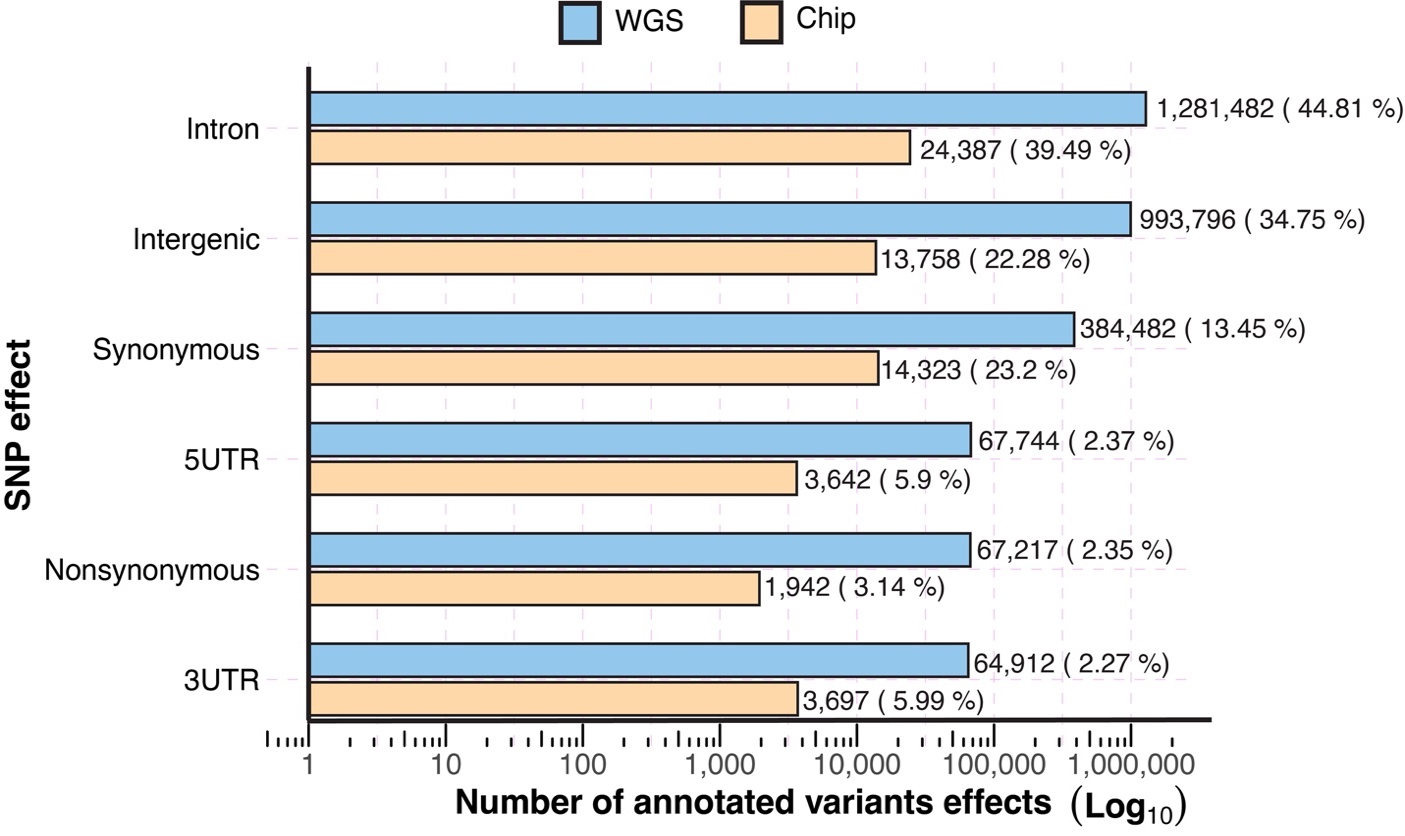 |
| --- |
| **Figure S11**. Functional annotation of variants for the chip and WGS data sets. On the y-axis are the different functional annotation categories from the SnpEff package. On the x-axis is the number of SNPs within each functional annotation category on a Log10 scale. The total number of SNPs and the percentage are listed to the right of each bar. Blue is for the WGS data, orange is for the SNP chip data. The number of SNPs analyzed were 2,727,727 for the WGS data and 59,384 for the chip data. |

| 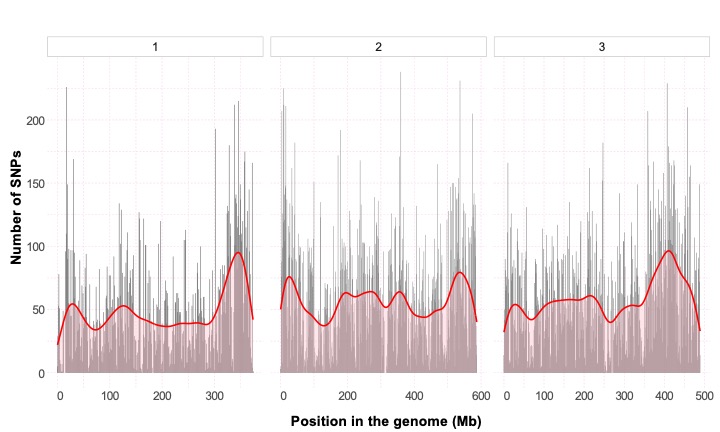 |
| --- |
| **Figure S12**. Plots showing the density of 82,731 SNPs in each chromosome (open bars on top with chromosome number) of the *Ae. albopictus* genome. The 574 scaffolds were merged to create a chromosomal scale following the order described in the AalbF3 genome assembly. The SNP data are from 401 wild samples after quality control. The y-axis represents the number of SNPs, and the x-axis shows the genomic positions in Mb across the three chromosomes. The black vertical bars indicate the number of SNPs at each genome site, and the red line and the red shade are the mean SNP density at each location. |

|  |
| --- |
| **Figure S13**. The linkage disequilibrium (LD) half distance, r^2^, for *Aedes albopictus* populations in the native range. The LD half distance indicates when LD falls to half its initial value, as shown on the x-axis in kilobases (kb). Horizontal bars represent the r^2^ in kb for each chromosome within a population. The bars' colors differentiate the three chromosomes: Chr1 in orange, Chr2 in blue, and Chr3 in pink. Sample sizes (N) for each population are displayed on the right. The LD values were estimated using the PopLDdecay package, and R software analyzed the LD half distance. |

| 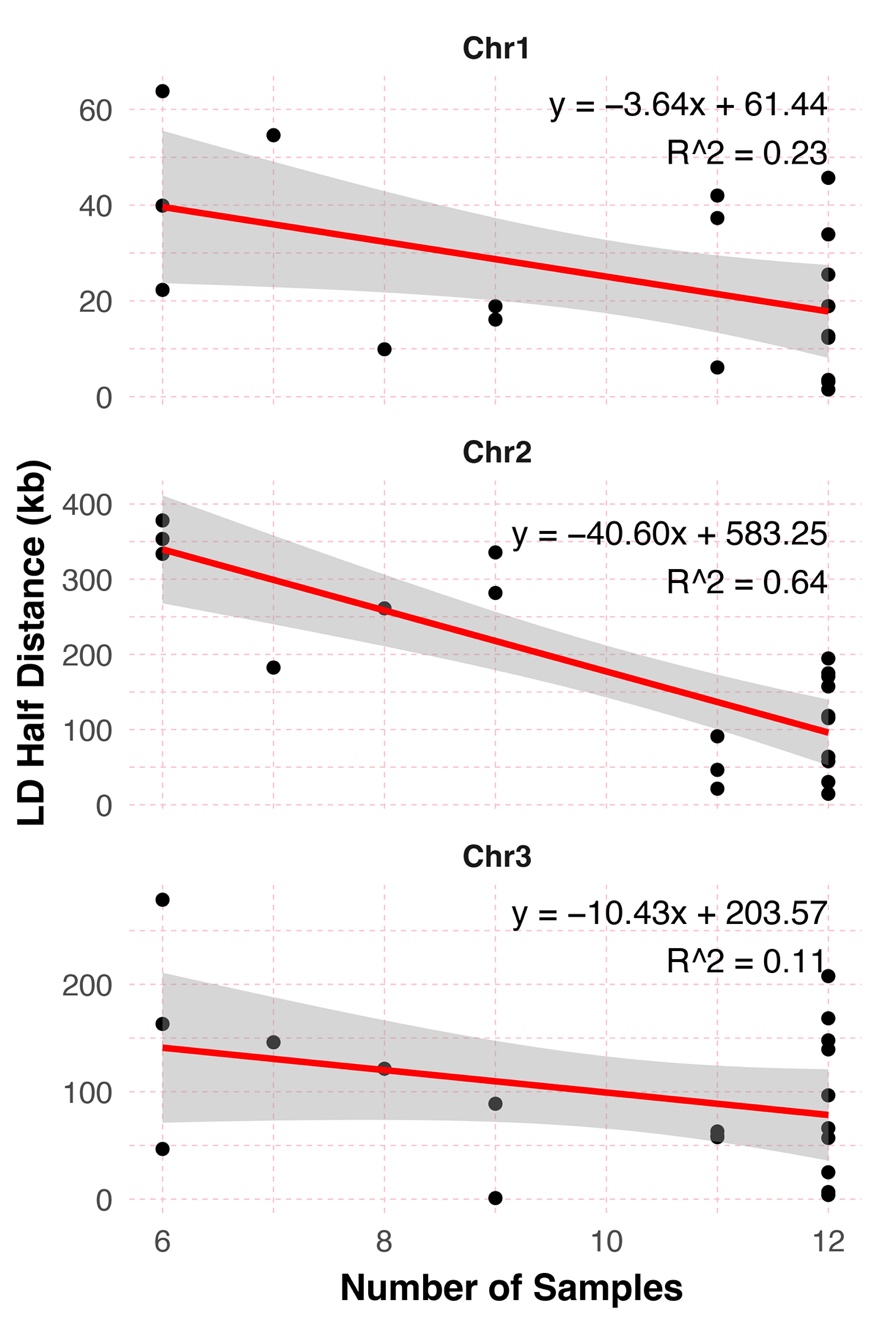 |
| --- |
| **Figure S14**. The relationship between the linkage disequilibrium (LD) half distance, r^2,^, (y-axis) and the number of samples (x-axis) for each population across three chromosomes: Chr1, Chr2, and Chr3. Black dots indicate the observed data points, and the red line shows the linear regression fit, with its confidence interval represented by a gray shaded area. |

| 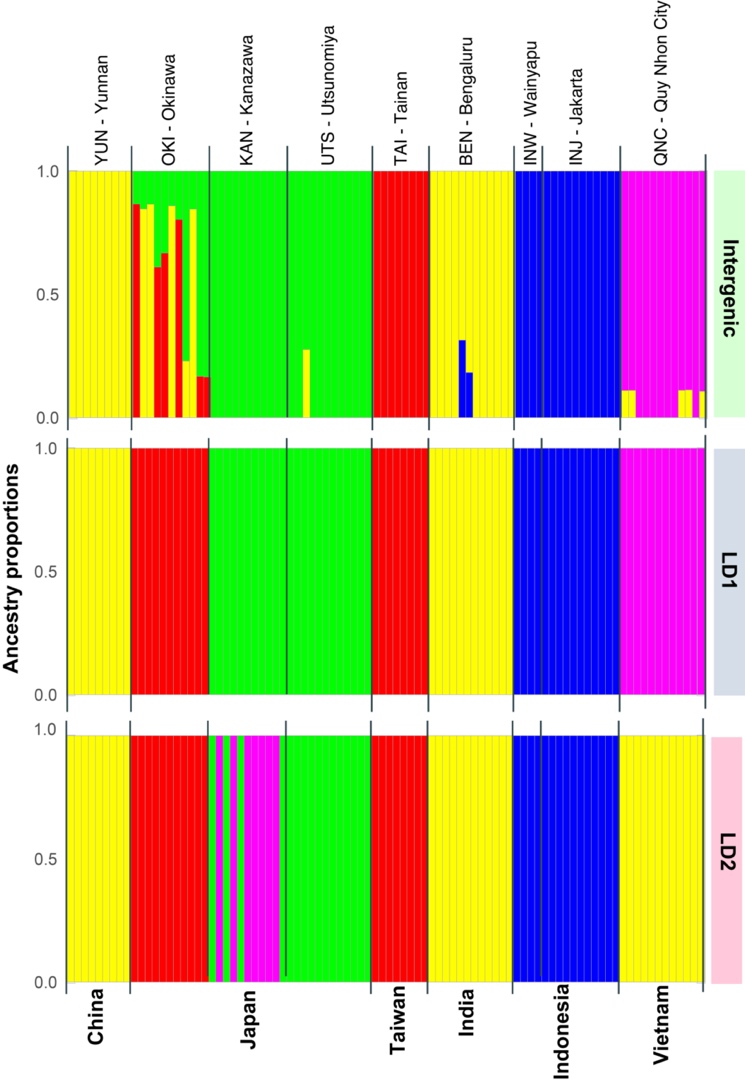 |
| --- |
| **Figure S15**. Structure plots after training a neural admixture model with 90 samples from 9 sampling sites from the native range of *Ae. albopictus* (Figure 2, Table S3). Representative Q matrices for k=5 were plotted of each SNP set (intergenic, LD1 and LD2). For each ancestry plot, each bar on the x-axis represents one mosquito. The height of the colors bars for each bar represents the different clusters, the height of each bar is the probability of the mosquito belonging to one of the five ancestral groups. The y-axis is the admixture proportions of the five ancestral genetic groups for each individual. Population codes and sampling site name are reported on the top. The country of sample origin is reported on the bottom. |

| 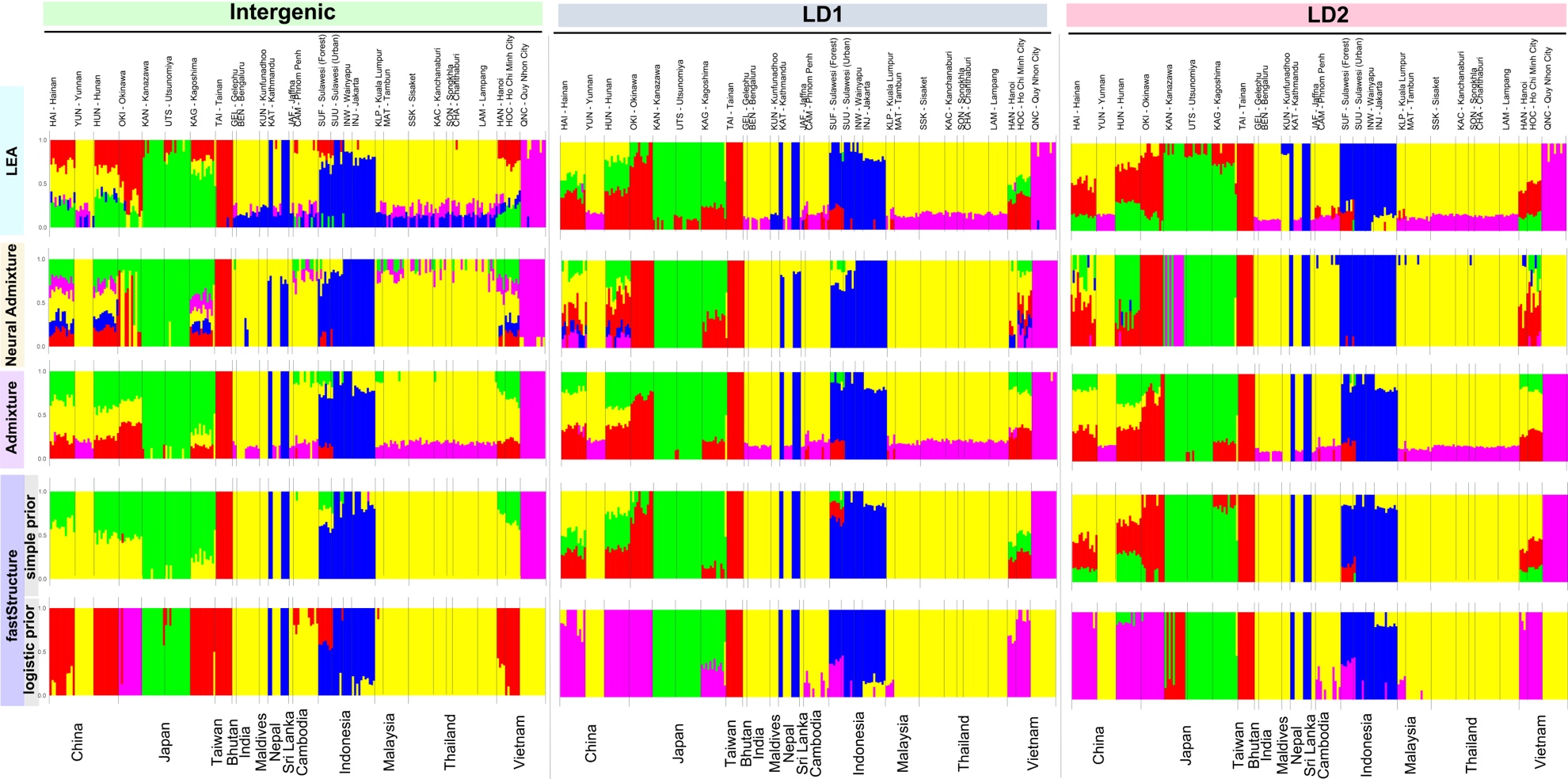 |
| --- |
| **Figure S16**. Population structure of *Ae. albopictus* in the native range using 237 samples from 28 sampling sites and 13 countries (Figure 2, Table S3). Representative Q matrices for k=5 were plotted using different clustering algorithms. The first row shows the LEA analysis results, the second row shows Neural ADMIXTURE results, the third row shows the ADMIXTURE results, the bottom two rows show the fastStructure results run with either simple or logistic priors. Estimates were obtained using three SNP sets (intergenic ~9,483 SNPs, left panel; LD1 ~20,931 SNPs, center panel; LD2 ~ 57, 788 SNPs, right panel). Symbols and conventions as in Figure S15. |

| 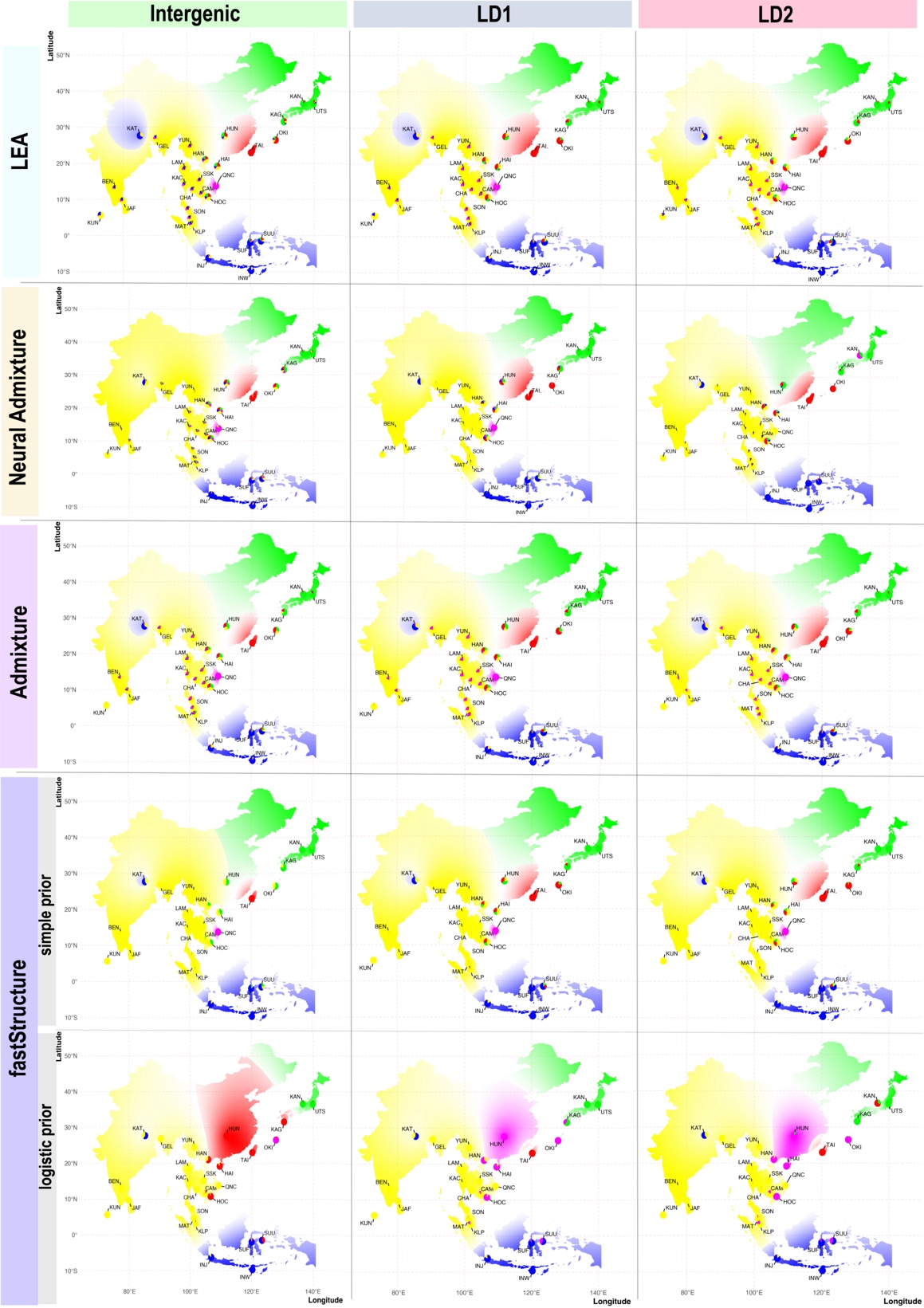 |
| --- |
| **Figure S17**. Population structure of *Ae. albopictus* interpolated over the species’ native range using the R package tess3r using Q matrices for k=5 from each algorithm. All inferences are performed with the three SNP sets (Intergenic, LD1, and LD2). The y-axis is the latitude, and the x-axis is the longitude. Admixture with all SNP sets indicated 5 ancestral groups. The ancestry matrices were interpolated over the entire region for which we have samples. Different shades of the same color reflect different ancestry coefficients. |
| 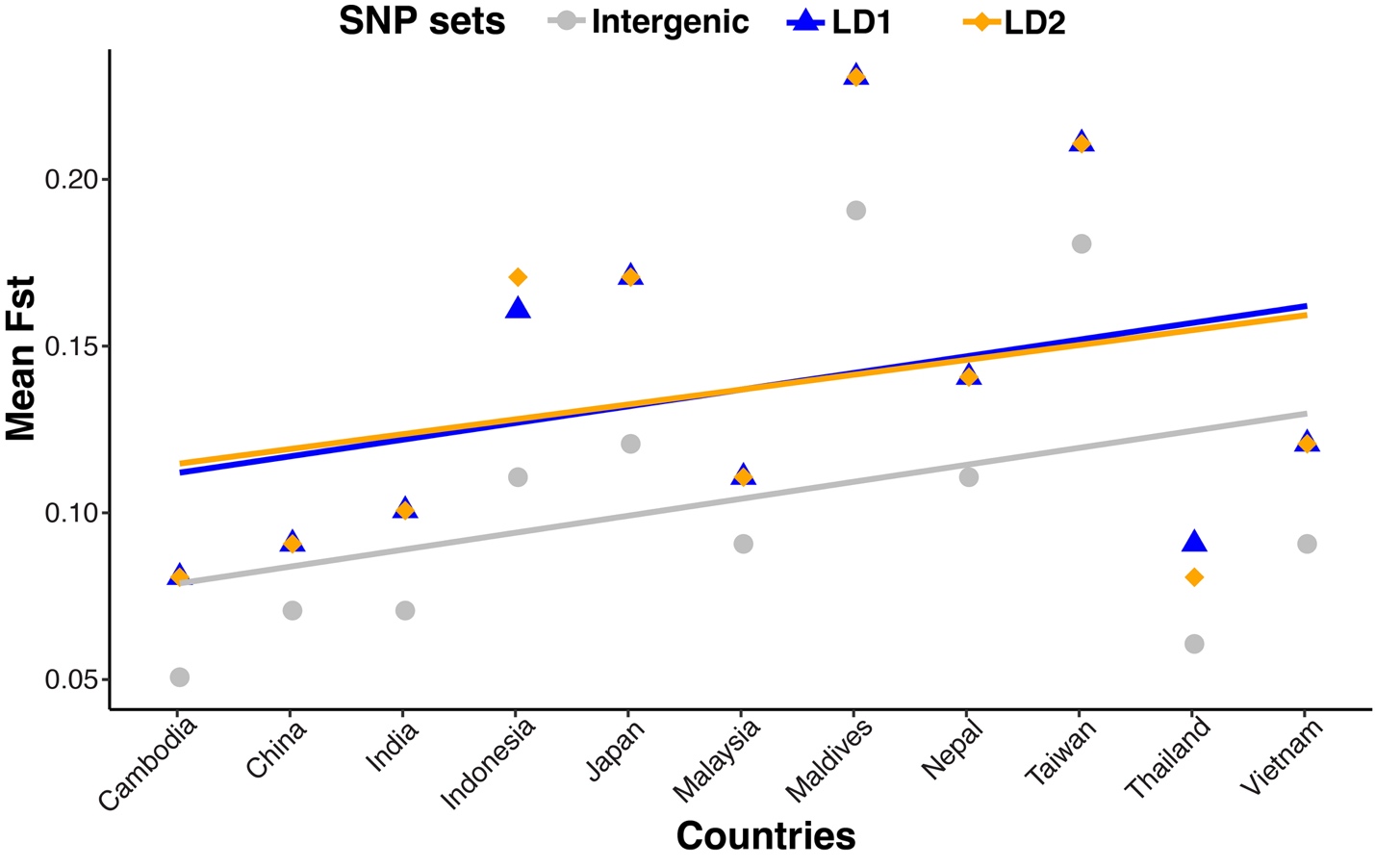 |
| **Figure S18**. Mean Fixation Index (Fst; y-axis) for different countries, using three SNP sets: Intergenic (gray), LD1 (blue), and LD2 (orange). The estimates were computed using the StAMPP package in R. For every SNP set, pairwise Fst values were determined across different populations, which were then averaged to estimate the mean Fst within countries. To visualize the trend in these mean Fst values across countries, we fitted a linear regression line for each SNP set. The countries are listed from left to right based on ascending mean Fst values. |
| \| 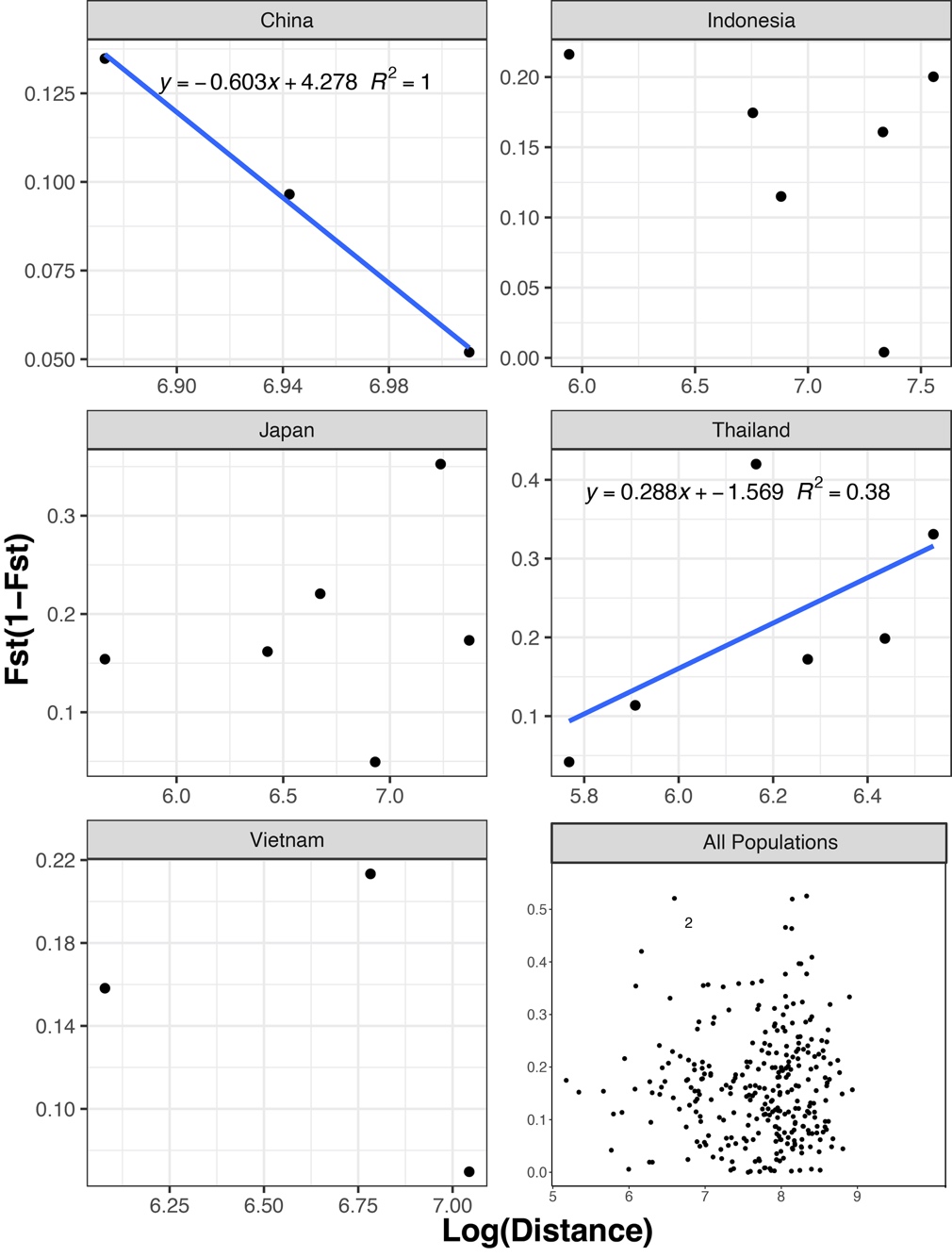 \| \| --- \| \| **Figure S19**. Relationship between genetic (Fst estimates) and geographic distance for countries with at least three sampling localities (first five panels) and for all the populations (last panel). Fst estimates were calculated using the LD2 dataset; Fst values for the LD1 and Integenic were similar (File S14). The geographical distance (Km) was calculated using the R package “geosphere” and the Fst estimates with the R package “StTAMP”. To fit the linear regression we used lm(Fst/(1-Fst) ~ log(Distance). \|  \| 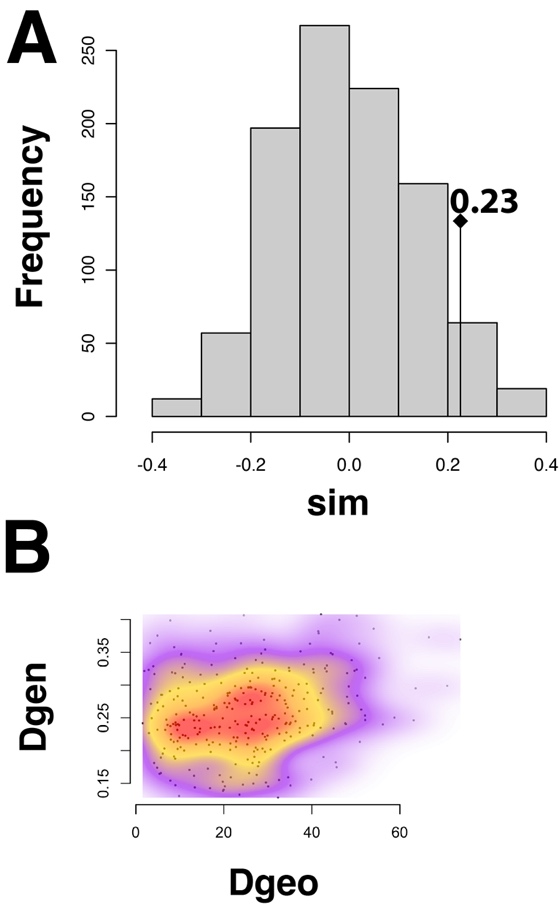 \| \| --- \| \| **Figure S20**. Mantel test for isolation-by-distance using populations with at least four mosquitoes (Table S3) and the LD2 SNP set (57,780 SNPs). (A) Simulated Distribution of Correlations: Histogram illustrating the distribution of correlation coefficients between genetic (Dgen) and geographic (Dgeo) distances obtained from 999 random permutations. The observed correlation coefficient of 0.23, as derived from the actual data, is indicated by the arrow, suggesting not a significant deviation from the random expectations (*p* < 0.053). (B) Isolation by Distance: Scatterplot of geographic distance (Dgeo) against genetic distance (Dgen). The density of overlapping points is represented by color intensity, with darker shades indicating higher overlap. \|  \| 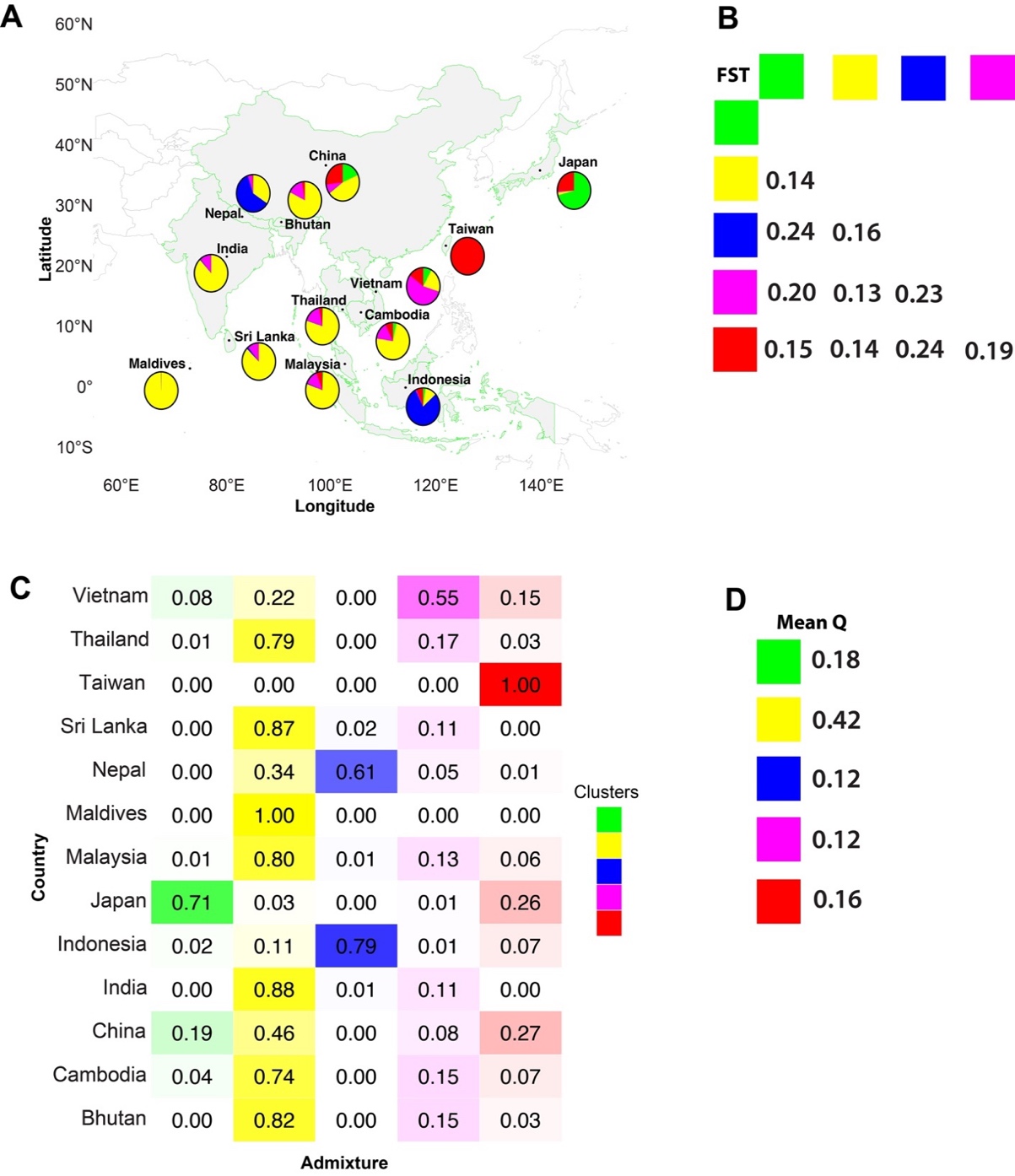 \| \| --- \| \| **Figure S21**. Summary of the admixture proportions per country and Fst between the five genetic clusters identified by ADMIXTURE using the SNP set LD2. **A.** Mean admixture per country (the different colors in each circle represent the percent of each cluster found at that location). **B.** Mean Fst estimates between the genetic clusters. **C.** Mean admixture proportions for each country with each genetic cluster (colors identify clusters). **D.** Mean admixture per genetic cluster. \| |

| 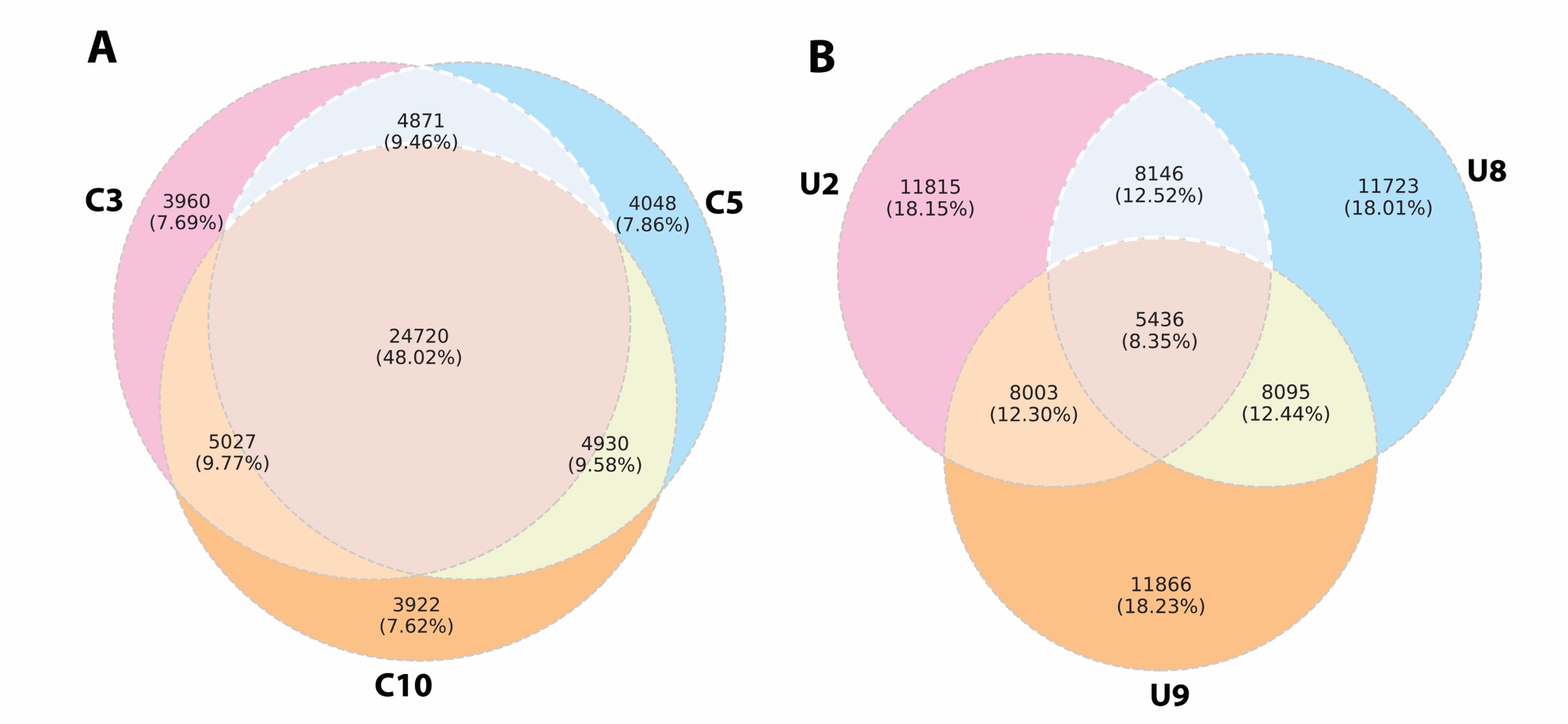 |
| --- |
| **Figure S22**. Venn diagrams displaying how many SNPs are shared between the SNP sets created to evaluate the chip biases towards coding regions. Diagram A represents three SNP sets where the chip bias towards coding regions was corrected (“corrected” sets = C3, C5, and C10). Diagram B shows SNP sets where the bias was not corrected ("uncorrected" sets = U2, U8, and U9; see Table S8). Note that while among the C sets about 48% of SNPs are shared across datasets, and only 8.35% are shared among the U datasets. |

|  |
| --- |
| **Figure S23**. Population structure of *Ae. albopictus* in the native range using corrected (C) and uncorrected (U) datasets. Representative Q matrices for k=5 were plotted. All inferences were performed for each of six SNP sets. The first three rows show the results for sets of randomly sampled SNPs for which the chip bias was not corrected (N=34,500; U2, U8, and U9); the bottom three rows shows the results for the same analysis using SNP sets that reproduce the proportion of SNPs in coding and non-coding regions found in the WGS data (N=~34,500; C10, C3, and C5; see Table S8 for SNPs sets details). Each bar on the x-axis represents one mosquito. The colors and height of each bar represent the probabilities of the mosquito belonging to an ancestral group and the ancestral group. The y-axis is the admixture proportions for the five ancestral genetic clusters. The main difference is the placement of QNC and TAI in two different genetic clusters (pink and yellow or pink and red). |

| 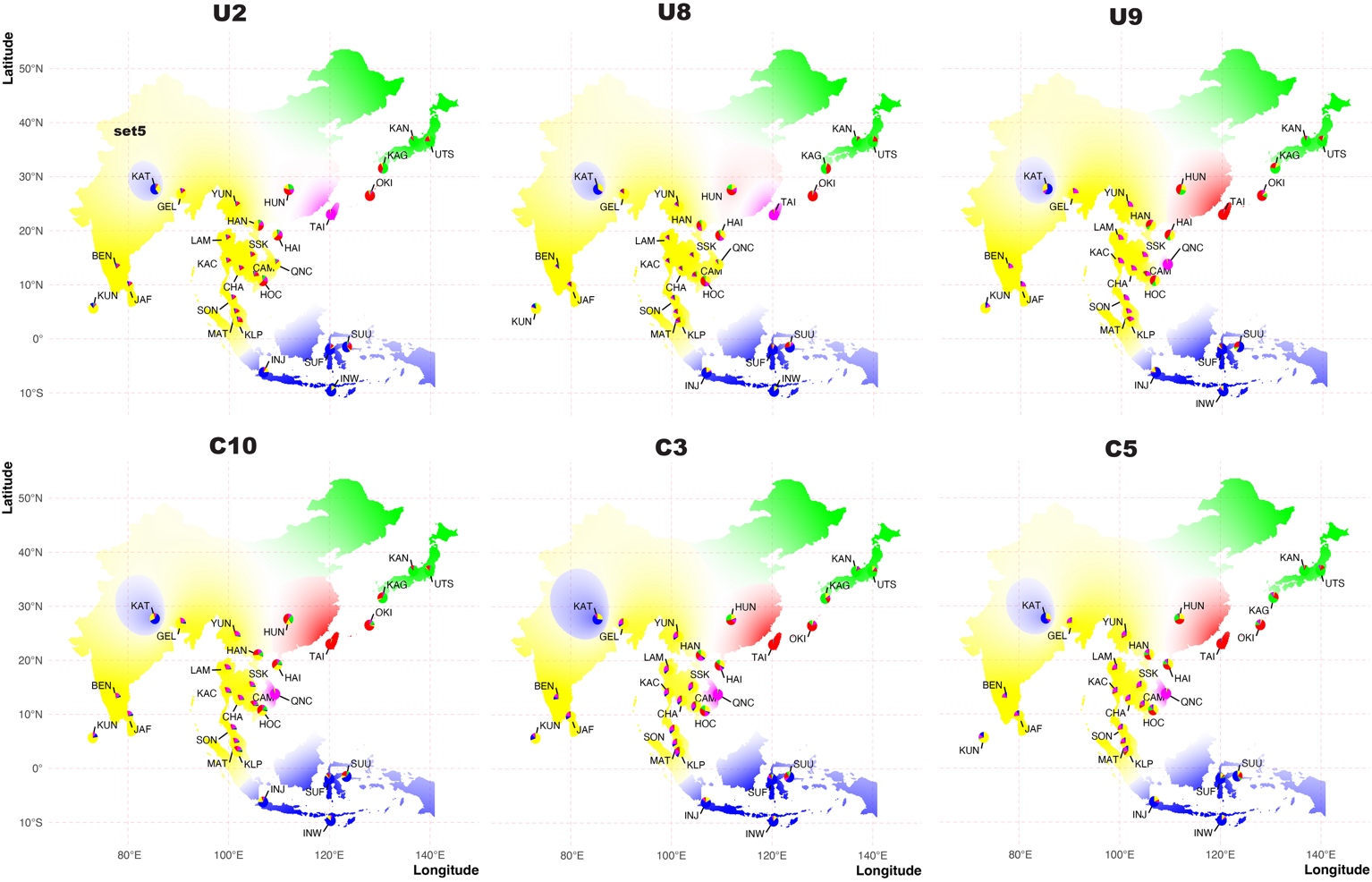 |
| --- |
| **Figure S24**. Populations structure of 237 *Ae. albopictus* from Asia using the clustering algorithm performed by the program LEA using the six SNP chip uncorrected (U) and corrected (C) datasets. Results for k=5 are interpolated over the species’ native range using the R package tess3r. Inferences were carried out using six different SNP sets: the first row shows the results for a set of randomly sampled SNPs for which the chip bias was not correct (N=34,500; U3, U5, and U8); the second row shows the results for the same analysis using a SNPs set that reproduces the proportion of SNPs in coding and non-coding regions found in the WGS data (N=~34,500; C10, C3, and C5; see Table S8 for SNPs sets details). The y-axis is the latitude, and the x-axis is the longitude. Sampling sites are shown as red dots, with associated three letter codes (see Table S3). The ancestry matrices were interpolated over the entire region for which we have samples, with different shades of the same color reflecting different ancestry coefficients. |

| 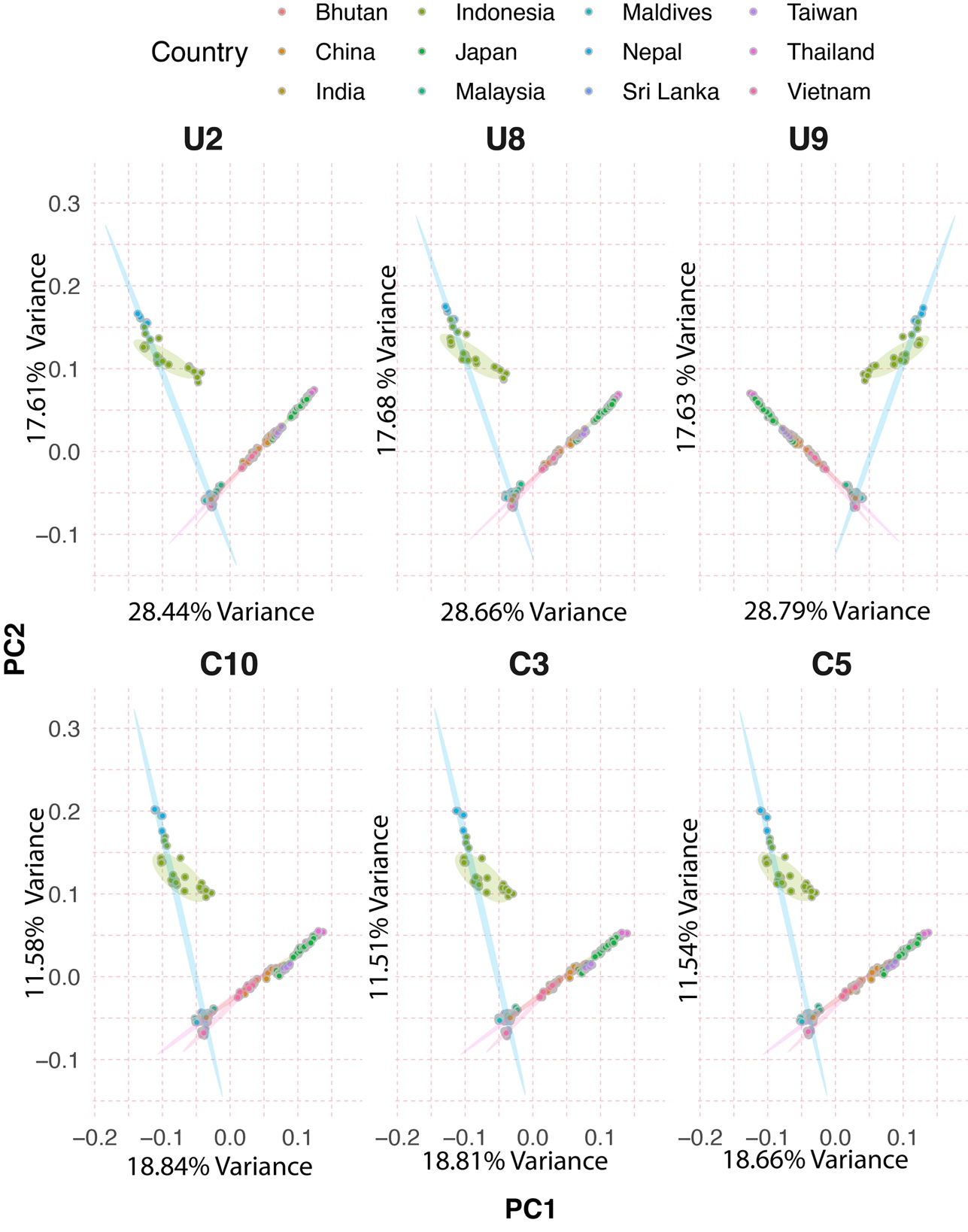 |
| --- |
| **Figure S25**. Principal component analysis of the same six SNPs dataset (uncorrected, U and corrected, C) as in Figures S23 and S24. The y-axis is the principal component 2, and the x-axis is the principal component 1. Each dot represents an individual mosquito, and the color of the dot refers to the country where they were sampled (legend on top). Ellipses mark each country in Asia covering 80% of the samples. See Table S8 for details about each SNP set. The variance explained by each principal component is annotated in the y- and x-axes. |

| 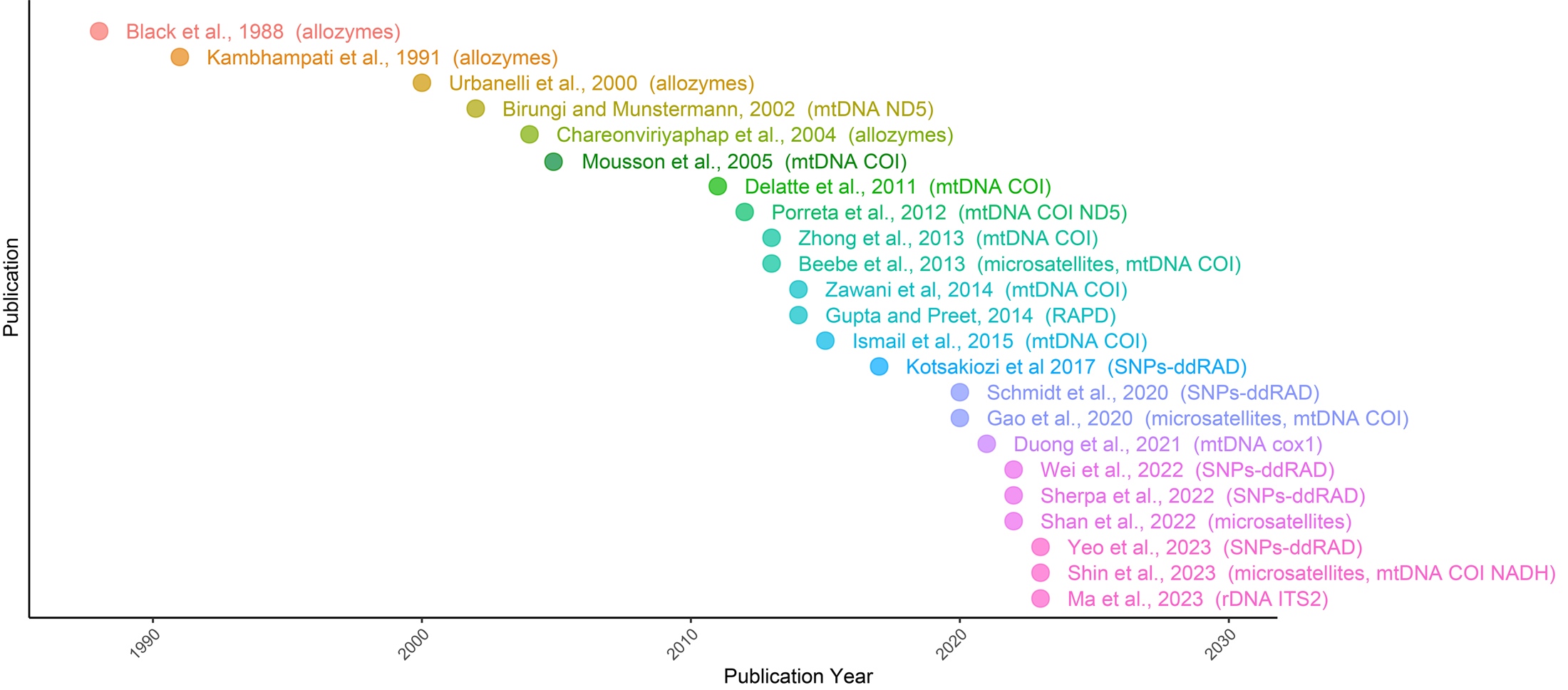 |
| --- |
| **Figure S26**. Timeline of the studies reviewed addressing the population structure of *Ae. albopictus* in Asia and Indian Ocean. The genetic marker(s) used is annotated at the right in parenthesis. |

| 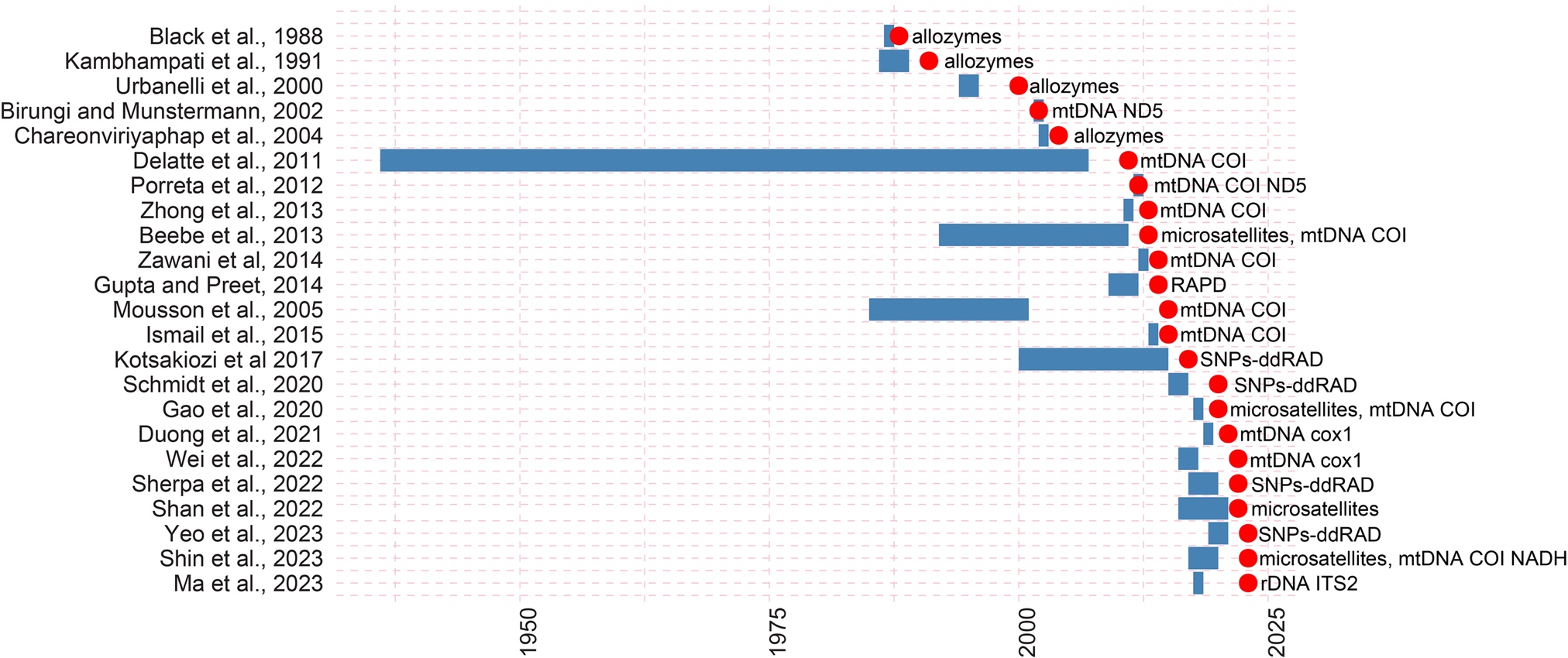 |
| --- |
| **Figure S27**. Gantt plot of the studies reviewed addressing the population structure of *Ae. albopictus* in Asia and Indian Ocean. The y-axis lists the publications, and the x-axis is the year. The genetic marker(s) used is annotated at the right. The blue bars are the range of the sample collection dates used in each study. The red dot is the publication date. |

| 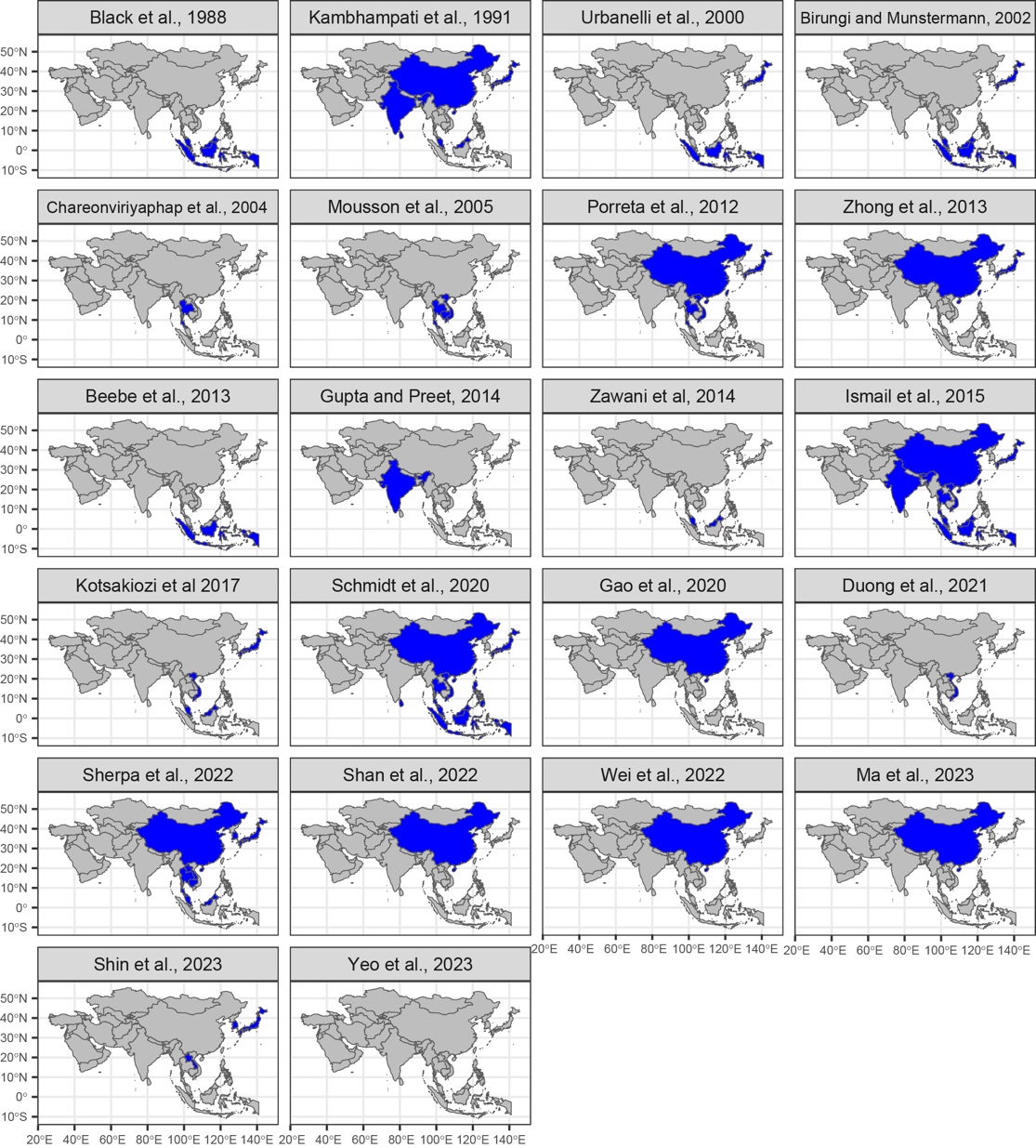 |
| --- |
| **Figure S28**. Sampling sites of studies from figure S27 with the countries sampled highlighted in blue. |
