# Supplementary material for: A genotyping array for the globally invasive vector mosquito, Aedes albopictus
Source: Parasit Vectors. 2024 Mar 4;17:106. doi: 10.1186/s13071-024-06158-z (PMC10910840; doi:10.1186/s13071-024-06158-z)
Supplement: Supplementary file 26 — Additional file 26. Supplementary Tables. [file 13071_2024_6158_MOESM26_ESM.docx]

**Additional file Tables**

**Table S1.** List of the supplemental files with the step-by-step description of all analyses available at [GitHub page](https://lucianocosme.github.io/albo_chip/) or [GitHub project](https://github.com/lucianocosme/albo_chip) and provided in Markdown format. Additional supplemental files present results.

| File | Methods section | Link |
| --- | --- | --- |
| File S1 | WGS analyses and SNP discovery | [File S1](https://lucianocosme.github.io/albo_chip/File_S1_01.SNP_discovery.html) |
| File S2 | Mapping probes to reference genomes | [File S2](https://lucianocosme.github.io/albo_chip/File_S2_02.Map_probes_to_reference_genomes.html) |
| File S3 | Segregation analysis from laboratory crosses | [File S3](https://lucianocosme.github.io/albo_chip/File_S3_03.Evaluating_allele_segregation_from_laboratory_crosses.html) |
| File S4 | Comparing genotypes of samples genotypes with WGS and chip | [File S4](https://lucianocosme.github.io/albo_chip/File_S4_04.Comparison_wgs_chip.html) |
| File S5 | Comparing the genotypes of replicates samples | [File S5](https://lucianocosme.github.io/albo_chip/File_S5_05.Comparying_genotypes_of_replicates.html) |
| File S6 | Functional annotation of SNPs and chip bias evaluation | [File S6](https://lucianocosme.github.io/albo_chip/File_S6_06.SNPs_functional_annotation.html) |
| File S7 | Quality control for wild samples genotyped with the chip | [File S7](https://lucianocosme.github.io/albo_chip/File_S7_07.Chip_use_genotyping_wild_populations_quality_control.html) |
| File S8 | Linkage analysis with PopLDdecay | [File S8](https://lucianocosme.github.io/albo_chip/File_S8_08.Linkage.html) |
| File S9 | Admixture analysis | [File S9](https://lucianocosme.github.io/albo_chip/File_S9_09.Admixture_analysis.html) |
| File S10 | LEA analysis | [File S10](https://lucianocosme.github.io/albo_chip/File_S10_10.LEA_analysis.html) |
| File S11 | fastStructure analysis | [File S11](https://lucianocosme.github.io/albo_chip/File_S11_11.fastStructure_analysis.html) |
| File S12 | Neural Admixture analysis | [File S12](https://lucianocosme.github.io/albo_chip/File_S12_12.neuroAdmixture_analysis.html) |
| File S13 | Interpolation of admixture matrices over Asia | [File S13](https://lucianocosme.github.io/albo_chip/File_S13_13.Q_matrices_interpolation.html) |
| File S14 | Evaluating the impact of chip bias with LEA and PCA | [File S14](https://lucianocosme.github.io/albo_chip/File_S14_14.LEA_SNPs_sets_wgs_chip_proportions.html) |
| File S15 | Fst analysis | [File S15](https://lucianocosme.github.io/albo_chip/File_S15_15.Fst_chip.html) |
| File S16 | Create map with samples | [File S16](https://lucianocosme.github.io/albo_chip/File_S16_16.Maps.html) |
| File S17 | Microsoft Word file with supplemental methods |  |
| File S18 | Microsoft Excel file with probe sequences |  |
| File S19 | PDF file from Axion Suite for the crosses genotype call |  |
| File S20 | PDF file from Axion Suite for the wild genotype call |  |
| File S21 | Microsoft Excel file with the result of segregation test |  |
| File S22 | Microsoft Word file with the literature review of the population structure of *Ae. albopictus* in Asia |  |
| File S23 | Compressed text file with the scores of each polymorphic site for probe design |  |

**Table S2.** Samples provided by Verily Life Sciences for SNP discovery for the SNP chip design. Geographic locations are shown in Figure 2. The first three columns list the city, country, and continent of origin. N refers to the number of samples sequenced. The last two columns show the year of collection and the abbreviation used for each sampling site.

| **Sampling site** | **Country** | **Region** | **N** | **Year** | **Abbreviation** |
| --- | --- | --- | --- | --- | --- |
| Limbe City | Cameroon | Africa | 20 | 2018 | LIM |
| Le Tampon | La Reunion | Africa | 24 | 2017 | TAM |
| Puerto Iguazu-Misiones | Argentina | Americas | 19 | 2018 | PUE |
| Maceio | Brazil | Americas | 20 | 2018 | MAC |
| Cali | Colombia | Americas | 8 | 2013 | CAL |
| Jardin Panteon | Mexico | Americas | 24 | 2017 | JAR |
| Saint Augustine | Trinidad and Tobago | Americas | 18 | 2018 | SAI |
| Houston (TX) | USA | Americas | 19 | 2018 | HOU |
| Jacksonville (FL) | USA | Americas | 20 | 2006 | JAC |
| Los Angeles (CA) | USA | Americas | 20 | 2018 | LOS |
| New Orleans (LA) | USA | Americas | 20 | 2016 | NEO |
| New Zion (SC) | USA | Americas | 19 | 2008 | NEZ |
| Oak Hill (FL) | USA | Americas | 24 | 2008 | OKA |
| Pahokee (FL) | USA | Americas | 20 | 2004 | PAH |
| Palm Beach (FL) | USA | Americas | 20 | 2008 | PAL |
| Salem (NJ) | USA | Americas | 20 | 2006 | SAL |
| Springfield (MO) | USA | Americas | 20 | 2015 | SPR |
| Vero Beach (FL) | USA | Americas | 20 | 1999 | VER |
| Manassas (VI) | USA | Americas | 20 | 2010 | MAN |
| Gelephu | Bhutan | Asia | 4 | 2009 | GEL |
| Beijing | China | Asia | 9 | 2014 | BEI |
| Guangzhou | China | Asia | 20 | 2014 | GUA |
| Hainan | China | Asia | 20 | 2014 | HAI |
| Shanghai | China | Asia | 17 | 2014 | SHA |
| Yunnan | China | Asia | 20 | 2014 | YUN |
| Jakarta | Indonesia | Asia | 24 | 2012 | JAK |
| Amamapare, Papua | Indonesia | Asia | 12 | 2015 | PAP |
| Sumba | Indonesia | Asia | 24 | 2013 | SUM |
| Okinawa | Japan | Asia | 9 | 2007 | OKI |
| Sendai | Japan | Asia | 19 | 2006 | SEN |
| Tanegashima | Japan | Asia | 25 | 2008 | TAN |
| Tokyo | Japan | Asia | 20 | 2006 | TOK |
| Wajima | Japan | Asia | 24 | 2008 | WAJ |
| Ipoh | Malaysia | Asia | 24 | 2013 | IPO |
| Kuala Lumpur | Malaysia | Asia | 19 | 2006 | KUA |
| Kathmandu | Nepal | Asia | 12 | 2000 | KAT |
| Jaffna | Sri Lanka | Asia | 7 | 2018 | JAF |
| Tainan | Taiwan | Asia | 16 | 2010 | TAI |
| Montpelier | France | Europe | 21 | 2015 | MON |
| Imperia | Italy | Europe | 20 | 2017 | IMP |
| Torres Strait Islands | Australia | Pacific | 60 | 12/15 | TOR |
| Honiara | Solomon Islands | Pacific | 18 | 2013 | HON |
| **Total** |  |  | 819 |  |  |

**Table S3.** Samples genotyped with the Aealbo chip. Sample locations are shown in Figure 2. The first three columns list the city, country, and continent of origin. N refers to the number of samples genotyped. The last two columns show the year of collection and the abbreviation used for each sampling site.

| **Sampling site** | **Country** | **Region** | **N** | **Year** | **Abbreviation** |
| --- | --- | --- | --- | --- | --- |
| Hainan | China | East Asia | 12 | 2014 | HAI |
| Hunan | China | East Asia | 12 | 2001 | HUN |
| Yunnan | China | East Asia | 9 | 2014 | YUN |
| Kagoshima | Japan | East Asia | 12 | 2018 | KAG |
| Kanazana | Japan | East Asia | 11 | 2008 | KAN |
| Okinawa | Japan | East Asia | 11 | 2018 | OKI |
| Utsonomyia | Japan | East Asia | 12 | 2008 | UTS |
| Tainan | Taiwan | East Asia | 8 | 2018 | TAI |
| Gelephu | Buthan | South Asia | 2 | 2009 | GEL ^1^ |
| Bengaluru | India | South Asia | 12 |  | BEN |
| Kunfunadhoo | Maldives | South Asia | 4 | 2017 | KUN |
| Kathmandu | Nepal | South Asia | 10 | 2002 | KAT ^2^ |
| Jaffna | Sri Lanka | South Asia | 2 |  | JAF |
| Cambodia | Cambodia | Southeast Asia | 12 |  | CAM |
| Jakarta, Indonesia | Indonesia | Southeast Asia | 11 |  | INJ |
| Wainyapu, Indonesia | Indonesia | Southeast Asia | 4 |  | INW |
| Sulawesi (Forest) | Indonesia | Southeast Asia | 6 |  | SUF |
| Sulawesi (Urban) | Indonesia | Southeast Asia | 6 |  | SUU |
| Kuala Lumpur | Malaysia | Southeast Asia | 4 |  | KLP |
| Tambun | Malaysia | Southeast Asia | 12 |  | MAT |
| Chanthaburi | Thailand | Southeast Asia | 12 |  | CHA |
| Kanchanaburi | Thailand | Southeast Asia | 6 | 2015 | KAC |
| Lampang | Thailand | Southeast Asia | 9 | 2011 | LAM |
| Songkhla | Thailand | Southeast Asia | 3 | 2015 | SON |
| Si Sa Ket | Thailand | Southeast Asia | 12 | 2018 | SSK |
| Hanoi | Vietnam | Southeast Asia | 4 |  | HAN |
| Ho Chi Min | Vietnam | Southeast Asia | 7 | 2018 | HOC |
| Qhui nhon City | Vietnam | Southeast Asia | 12 |  | QNC |
| Saint Augustine | Trinidad and Tobago | South America | 12 |  | SAI ^2^ |
| Laboratory crosses |  |  | 152 |  |  |
| **Total** |  |  | 401 |  |  |

*^1^ DNA extracts from this population were obtained from larvae. All other populations were adult mosquitoes.*

*^2^ These population samples were used for comparison of WGS and chip genotypes (see Table 4).*

**Table S4.** Details on the samples used to evaluate the accuracy of the SNP chip genotype call among three technical replicates.

| **Sampling site** | **Country** | **Region** | **Year** | **Abbreviation** |
| --- | --- | --- | --- | --- |
| Barcelona | Spain | Europe |  | BAR |
| Los Angeles | USA | Americas | 2018 | LOS |
| Ho Chi Min | Vietnam | Asia | 2018 | HOC |
| Kagoshima | Japan | Asia | 2018 | KAG |
| Bengaluru | India | Asia |  | IND |

**Table S5.** The genotyping files for each technical replicate. The first number refers to the sample number in our database, the second column (CEL file) identifies the individual sample on the chip. The first number is the identification number in the data set, the three letters refer to the code for the population, followed by the country and the file extension (.CEL).

| **Replicate ID** | **CEL file** |
| --- | --- |
| 1a | 291_BAR_Spain.CEL |
| 1b | 292_BAR_Spain.CEL |
| 1c | 293_BAR_Spain.CEL |
| 2a | 258_IND_India.CEL |
| 2b | 259_IND_India.CEL |
| 2c | 260_IND_India.CEL |
| 3a | 227_KAG_Japan.CEL |
| 3b | 228_KAG_Japan.CEL |
| 3c | 229_KAG_Japan.CEL |
| 4a | 365_LOS_USA.CEL |
| 4b | 366_LOS_USA.CEL |
| 4c | 367_LOS_USA.CEL |

**Table S6.** Primer sequences used to amplify a 1537 bp fragment of the mitochondrial COI gene for species identification of larval and other unidentified mosquitoes.

| **Name** | **Sequence** | **Annealing Temp** |
| --- | --- | --- |
| COI-Forward | 5’-TTT ACA ATT TAT CGC CTA AAC TTC-3’ | 49.7 ℃ |
| COI-Reverse | 5’-CAT TGC ACT AAT CTG CCA TA-3’ | 50.3 ℃ |

**Table S7.** Key metrics characterizing the genome assemblies. 'Genome size (Gb)' indicates the estimated size of the entire genome in gigabases. 'Number of scaffolds' represents the total count of genomic scaffolds used in the assembly. 'Scaffold N50' and 'Scaffold L50 (N)' offer insights into scaffold length distribution, with N50 representing the median scaffold size and L50 showing the number of scaffolds needed to cover half of the genome. Similarly, 'Number of contigs' counts the total number of contiguous sequences in the assembly, while 'Contig N50' and 'Contig L50 (N)' describe contig length characteristics. 'GC percent (%)' reflects the percentage of guanine and cytosine base pairs in the genome, providing insights into its composition. Lastly, 'Genome coverage (x)' indicates the depth of sequencing coverage achieved during the assembly process.

| Metrics |  |  | Genome |  |  |  |
| --- | --- | --- | --- | --- | --- | --- |
|  | **AalbF1** ^28^ | **AalbF2** ^29^ | **AalbF3** ^24^ | **AalbCell** ^30^ | **AalbRamini** ^31^ | **AaegL5** ^32^ |
| Genome size (Gb) | 1.9 | 2.5 | 1.5 | 2.2 | 1.3 | 1.3 |
| Number of scaffolds | 154,782 | 2,196 | 574 | - | - | 2,309 |
| Scaffold N50 | 201 kb | 55.7 Mb | 10.1 Mb | - | - | 409.8 Mb |
| Scaffold L50 (N) | 2,851 | 13 | 43 | - | - | 2 |
| Number of contigs | 355,061 | 5,472 | 1,134 | 2,434 | 3,342,920 | 2,538 |
| Contig N50 | 18.4 kb | 1.2 Mb | 7.5 Mb | 3.3 Mb | 397 bp | 11.8 Mb |
| Contig L50 (N) | 28,288 | 433 | 56 | 203 | 994,094 | 30 |
| GC percent (%) | 40 | 40 | 40 | 40 | 40 | 38 |
| Genome coverage (x) | 229 | 75 | 75 | 80 | 60 | 110 |

**Table S8.** SNPs sets used to test the effect of the chip bias towards coding regions. The “U” sets are composed of SNPs sampled randomly from the chip data (uncorrected bias). The “C” sets are composed of SNPs sampled from the chip data to obtain a representation of the WGS SNP proportions based on their functional annotation (corrected bias). The second column identified the actual file name in the code. The number of SNPs in each set is reported in the last column.

| Set | Name in the code | SNPs (N) |
| --- | --- | --- |
| U2 | Random2 | 33,400 |
| U8 | Random8 | 33,400 |
| U9 | Random9 | 33,400 |
| C10 | Set10 | 33,392 |
| C3 | Set3 | 33,413 |
| C5 | Set5 | 33,456 |

| **Table S9**. Summary of the number of SNPs used for linkage estimates in each population. Details of population codes in Table S3 and Figure 2. The second column refers to the number of individuals from each population. The next three columns show the number of SNPs for each chromosome (chr1, chr2 and chr3). | | | | |  |
| --- | --- | --- | --- | --- | --- |
| Population | Samples (N) | chr1 | chr2 | chr3 | |
| BEN | 12 | 18,004 | 31,361 | 28,030 | |
| CAM | 12 | 18,883 | 34,800 | 29,800 | |
| CHA | 12 | 18,601 | 33,512 | 28,986 | |
| HAI | 12 | 19,559 | 36,241 | 31,287 | |
| HOC | 7 | 17,243 | 32,285 | 27,557 | |
| HUN | 12 | 19,832 | 36,785 | 31,688 | |
| INJ | 11 | 17,062 | 28,859 | 25,521 | |
| KAC | 6 | 16,095 | 28,260 | 24,433 | |
| KAG | 12 | 17,330 | 34,757 | 28,670 | |
| KAN | 11 | 16,482 | 30,165 | 25,921 | |
| LAM | 9 | 17,275 | 30,930 | 26,655 | |
| MAT | 12 | 18,109 | 32,920 | 28,169 | |
| OKI | 11 | 17,513 | 30,957 | 27,688 | |
| QNC | 12 | 14,365 | 26,019 | 22,670 | |
| SSK | 12 | 18,414 | 33,457 | 28,812 | |
| SUF | 6 | 14,842 | 25,286 | 22,653 | |
| SUU | 6 | 16,417 | 29,709 | 25,373 | |
| TAI | 8 | 13,232 | 26,210 | 21,282 | |
| UTS | 12 | 17,481 | 34,042 | 29,737 | |
| YUN | 9 | 17,655 | 31,546 | 27,383 | |

**Table S10**. SNP sets employed to assess the reproducibility of the admixture, clustering and multivariate analyses using different genomic regions. The "intergenic" set was curated by selecting intergenic SNPs with an Fst value < 0.2, following a quasi-neutral distribution. Sets LD1 and LD2 were derived using distinct linkage pruning strategies. For Set LD1, an r^2^ threshold of 0.01 was applied for pruning, whereas Set LD2 underwent pruning with an r^2^ threshold of 0.1.

| Set | Name in the code | SNPs (N) |
| --- | --- | --- |
| Intergenic | “neutral” | 9,843 |
| LD1 | r2 0.01 | 20,931 |
| LD2 | r2 0.1 | 57,780 |

**Table S11.** Summary of algorithm parameters used for analysis of population structure in the ancestral range with the wild samples. Admixture: the *“run”* parameter identifies the computational run, *“--cv”* enables cross-validation for model assessment, *“-B”* sets the number of bootstrap replicates for robust estimates, *“-j”* defines the number of threads for faster computations. LEA: *“repetitions = 5”* sets the number of times to repeat the analysis for accuracy, *“CPU=4”* allocates four processing threads to speed up the computation, *“entropy=TRUE”* enables the calculation of entropy, providing an additional measure of genetic diversity or information. fastStructure: *“10 runs”* indicates execution of the analysis 10 times for better reliability, *“--prior=simple/logistic”* sets the simple/logistic prior, affecting how the model handles ancestry estimates; *“--full”* computes a full model, rather than a simplified version; *“--cv=10”* enables 10-fold cross-validation for assessing the model's generalizability; *“--tol=10e-6”* sets the tolerance level for convergence at 10^-6, ensuring that the algorithm stops when the estimates are sufficiently accurate. Neural admixture: *“--initialization pckmeans”* sets the initial clustering method to probabilistic k-means, *“--warmup_epochs 1000 runs”* 1,000 warm-up epochs before the main training starts, *“--max_epochs 1000”* sets the maximum number of training epochs, *“--activation relu”* specifies the use of the ReLU activation function in the neural network, *“--optimizer adam”* uses the Adam optimization algorithm for training, *“--learning_rate 1e-7”* sets the learning rate at 10^-7, *“--min_k 2”* and *“--max_k 10”* define the minimum and maximum number of clusters (k) to be estimated.

| **Algorithm** | **k** | **Parameters** |
| --- | --- | --- |
| Admixture | 1:25 | 1 run, --cv=10, -B2000, -j20 |
| LEA | 1:15 | repetitions = 5, CPU=4, entropy=TRUE |
| fastStructure logistic | 1:30 | 10 runs, --prior=logistic --full --cv=10 --tol=10e-6 |
| fastStructure simple | 1:30 | 10 runs, --prior=simple --full --cv=10 --tol=10e-6 |
| Neural admixture train | 2:10 | --initialization pckmeans --warmup_epochs 1000 --max_epochs 1000 --activation relu --optimizer adam --learning_rate 1e-7 --min_k 2 --max_k 10 |

**Table S12**. Probe sequences were aligned using BWA MEM with default parameters for various genome assemblies from NCBI (_ncbi) and VectorBase (_vb) databases. AalbF3 and AalbRamini are exclusive to NCBI. "Mapping Quality > 30" signifies high-confidence alignments. "Mismatches" indicates discrepancies with the reference genome. In the "Alignments" section, "Unique" means a single unique alignment, "Unique + 0 mismatches" is a perfect match, "Multiple" indicates multiple alignments, and "Secondary" and "Supplemental" are suboptimal or additional alignments.

| Genome | Sequence | Number of probe sequences mapped | | | | | | |  |
| --- | --- | --- | --- | --- | --- | --- | --- | --- | --- |
|  |  | Mapping quality>30 | Mismatches | | Alignments | | | | |
|  |  |  | 0 | 1 | Unique | Unique + 0 mismatches | Multiple | Secondary | Supplemental |
| AalbF1_ncbi | Ref | 113,962 | 56,689 | 18,939 | 113,873 | 53,623 | 54,852 | 81,695 | 177 |
|  | Alt | 105,778 | 6,526 | 53,857 | 105,695 | 5,016 | 59,453 | 89,835 | 149 |
|  | N | 106,494 | 327 | 54,983 | 106,410 | 0 | 58,561 | 120,831 | 152 |
| AalbF1_vb | Ref | 113,962 | 56,689 | 18,939 | 113,873 | 53,623 | 54,852 | 81,695 | 177 |
|  | Alt | 105,778 | 6,526 | 53,857 | 105,695 | 5,016 | 59,453 | 89,835 | 149 |
|  | N | 106,494 | 327 | 54,983 | 106,410 | 0 | 58,561 | 120,831 | 152 |
| AalbF2_ncbi | Ref | 115,033 | 115,029 | 0 | 115,033 | 115,029 | 56,912 | 84,164 | 0 |
|  | Alt | 96,906 | 20 | 96,883 | 96,906 | 20 | 66,540 | 115,993 | 0 |
|  | N | 100,803 | 0 | 100,800 | 100,803 | 0 | 61,887 | 148,532 | 0 |
| AalbF2_vb | Ref | 115,032 | 115,028 | 0 | 115,032 | 115,028 | 56,912 | 84,093 | 0 |
|  | Alt | 96,905 | 20 | 96,882 | 96,905 | 20 | 66,540 | 115,980 | 0 |
|  | N | 100,803 | 0 | 100,800 | 100,803 | 0 | 61,887 | 148,506 | 0 |
| AalbF3 | Ref | 166,111 | 166,111 | 0 | 166,111 | 166,111 | 8,621 | 22,070 | 0 |
|  | Alt | 164,095 | 1 | 164,094 | 164,095 | 1 | 8,782 | 11,828 | 0 |
|  | N | 164,194 | 0 | 164,194 | 164,194 | 0 | 8,699 | 11,673 | 0 |
| AalbRamini | Ref | 102,505 | 35,493 | 23,409 | 102,269 | 29,610 | 66,238 | 82,493 | 379 |
|  | Alt | 99,646 | 9,652 | 38,675 | 99,409 | 6,437 | 68,214 | 80,777 | 371 |
|  | N | 99,550 | 1,086 | 39,518 | 99,330 | 0 | 68,505 | 103,143 | 349 |
| AalbCell_ncbi | Ref | 61,734 | 17,691 | 13,669 | 61,677 | 15,205 | 98,651 | 127,218 | 183 |
|  | Alt | 55,438 | 4,991 | 14,932 | 55,398 | 3,877 | 102,692 | 128,574 | 147 |
|  | N | 55,345 | 228 | 14,603 | 55,298 | 0 | 102,741 | 167,040 | 156 |
| AalbCell_vb | Ref | 61,734 | 17,691 | 13,669 | 61,677 | 15,205 | 98,651 | 127,218 | 183 |
|  | Alt | 55,438 | 4,991 | 14,932 | 55,398 | 3,877 | 102,692 | 128,574 | 147 |
|  | N | 55,345 | 228 | 14,603 | 55,298 | 0 | 102,741 | 167,040 | 156 |

**Table S13**. Mapped probe sequences with BWA ALN using default parameters for various genome assemblies from NCBI (_ncbi) and VectorBase (_vb) databases. "Mapping Quality > 30" indicates high-confidence alignment, "Mismatches" reveals discrepancies with the reference genome. In the "Alignments" section, "Unique" signifies a single unique alignment, "Unique + 0 mismatches" indicates a perfect match, and "Multiple" suggests multiple alignments. Sequences are categorized as Ref (mapping uniquely with zero mismatches to AalbF3), Alt (mapping uniquely with one mismatch to AalbF3), and N (mapping uniquely with one mismatch to AalbF3) based on the allele.

| Genome | Sequence | Number of probe sequences mapped | | | |  | | |  |
| --- | --- | --- | --- | --- | --- | --- | --- | --- | --- |
|  |  | Mapping quality>30 | Mismatches | | Alignments | | | | |
|  |  |  | 0 | 1 | Unique | | Unique + 0 mismatches | Multiple | |
| AalbF1_ncbi | Ref | 99,429 | 56,630 | 18,064 | 99,429 | | 55,761 | 56,507 | |
|  | Alt | 93,965 | 5,411 | 58,052 | 93,965 | | 5,150 | 59,624 | |
|  | N | 91,743 | 24 | 59,178 | 91,743 | | 0 | 62,854 | |
| AalbF1_vb | Ref | 99,429 | 56,630 | 18,064 | 99,429 | | 55,761 | 56,507 | |
|  | Alt | 93,965 | 5,411 | 58,052 | 93,965 | | 5,150 | 59,624 | |
|  | N | 91,743 | 24 | 59,178 | 91,743 | | 0 | 62,854 | |
| AalbF2_ncbi | Ref | 122,202 | 122,199 | 0 | 122,202 | | 122,199 | 34,505 | |
|  | Alt | 114,335 | 0 | 114,333 | 114,335 | | 0 | 38,855 | |
|  | N | 117,833 | 0 | 117,831 | 117,833 | | 0 | 39,163 | |
| AalbF2_vb | Ref | 122,202 | 122,199 | 0 | 122,202 | | 122,199 | 34,505 | |
|  | Alt | 114,335 | 0 | 114,333 | 114,335 | | 0 | 38,855 | |
|  | N | 117,833 | 0 | 117,831 | 117,833 | | 0 | 39,163 | |
| AalbF3_ncbi | Ref | 167,485 | 167,485 | 0 | 167,485 | | 167,485 | 4,702 | |
|  | Alt | 167,391 | 0 | 167,391 | 167,391 | | 0 | 4,760 | |
|  | N | 167,434 | 0 | 167,434 | 167,434 | | 0 | 4,739 | |
| AalbRamini_nbi | Ref | 80,151 | 31,542 | 20,880 | 80,151 | | 30,758 | 81,794 | |
|  | Alt | 77,101 | 6,948 | 36,488 | 77,101 | | 6,638 | 83,616 | |
|  | N | 73,552 | 21 | 37,149 | 73,552 | | 0 | 86,916 | |
| AalbCell_ncbi | Ref | 59,704 | 17,538 | 16,657 | 59,704 | | 16,875 | 80,135 | |
|  | Alt | 53,587 | 4,521 | 18,968 | 53,587 | | 4,300 | 84,047 | |
|  | N | 50,150 | 18 | 18,632 | 50,150 | | 0 | 89,822 | |
| AalbCell_vb | Ref | 59,704 | 17,538 | 16,657 | 59,704 | | 16,875 | 80,135 | |
|  | Alt | 53,587 | 4,521 | 18,968 | 53,587 | | 4,300 | 84,047 | |
|  | N | 50,150 | 18 | 18,632 | 50,150 | | 0 | 89,822 | |

| Table S14. Number of segregating SNPs for parents in six families. These are SNPs heterozygous in at least one parent. The number of offspring samples for each family is in parenthesis. The shared SNPs were tested in all families, while the tested SNPs were analyzed in each family. | | | | | | | | |
| --- | --- | --- | --- | --- | --- | --- | --- | --- |
| Family | fam1 (46) | fam2 (23) | fam3 (25) | fam4 (29) | fam5 (20) | fam6 (9) | Shared | Tested |
| fam1 | - | 20,656 | 21,023 | 27,897 | 27,404 | 20,882 | 5,249 | 42,116 |
| fam2 | 20,656 | - | 33,581 | 20,982 | 20,929 | 36,360 | 5,249 | 54,871 |
| fam3 | 21,023 | 33,581 | - | 21,326 | 21,265 | 35,885 | 5,249 | 55,484 |
| fam4 | 27,897 | 20,982 | 21,326 | - | 29,677 | 21,247 | 5,249 | 42,684 |
| fam5 | 27,404 | 20,929 | 21,265 | 29,677 | - | 21,107 | 5,249 | 42,720 |
| fam6 | 20,882 | 36,360 | 35,885 | 21,247 | 21,107 | - | 5,249 | 55,205 |

| **Table S15.** Mean error rate between three replicates of the same individual from four population using a custom code. The individuals for each technical replicate were respectively 227 (1), 258 (2), 291 (3), and 365 (4) (Table S5). | |
| --- | --- |
| **Replicate** | **Mean error rate per replicate (%)** |
| 1 | 0.45 |
| 2 | 1.02 |
| 3 | 0.33 |
| 4 | 0.34 |
| Mean | 0.54 |

Table S16. Genome annotation files containing errors detected by the GFF3ToolKit according to the gene model. "Assembly Used" indicates the genome assembly analyzed, "Error Code" provides specific identifiers for errors, and "Model Used" specifies the gene model. "Problematic Models (n)" quantifies the number of models with issues. "Error Level" categorizes errors by severity, and "Error Tag" offers descriptive labels for context.

| Assembly | Error code | Model  Used | Problematic models (n) | Error level | Error tag |
| --- | --- | --- | --- | --- | --- |
| AaegL5 | Ema0009 | Canonical | 447 | Warning | Incorrectly merged gene parent? Isoforms that do not share coding sequences are found |
| AaegL5 | Ema0004 | Canonical | 366 | Info | Incomplete gene feature that should contain at least one mRNA, exon, and CDS |
| AaegL5 | Ema0008 | Canonical | 28 | Warning | Warning for distinct isoforms that do not share any regions |
| AaegL5 | Ema0002 | Canonical | 91 | Warning | Protein sequence contains internal stop codons |
| AaegL5 | Esf0003 | Canonical | 2,310 | Error | Strand information missing |
| Total |  |  | 3,242 |  |  |
| AaegL5 | Esf0003 | Non-canonical | 2,310 | Error | Strand information missing |
| Total |  |  | 2,310 |  |  |
| AalbF1 | Esf0012 | Canonical | 82 | Info | Found Ns in a feature using the external FASTA |
| AalbF1 | Esf0003 | Canonical | 154,782 | Error | Strand information missing |
| Total |  | - | 154,864 |  |  |
| AalbF1 | Esf0012 | Non-canonical | 82 | Info | Found Ns in a feature using the external FASTA |
| AalbF1 | Esf0003 | Non-canonical | 154,782 | Error | Strand information missing |
| Total |  |  | 154,864 |  |  |
| AalbF2 | Esf0036 | Canonical | 2,234 | Error | Value of a attribute contains unescaped “,” |
| AalbF2 | Ema0004 | Canonical | 9,530 | Info | Incomplete gene feature that should contain at least one mRNA, exon, and CDS |
| AalbF2 | Ema0005 | Canonical | 3,994 | Info | Pseudogene has invalid child feature type |
| AalbF2 | Ema0009 | Canonical | 4,285 | Warning | Incorrectly merged gene parent? Isoforms that do not share coding sequences are found |
| AalbF2 | Ema0002 | Canonical | 235 | Warning | Protein sequence contains internal stop codons |
| AalbF2 | Ema0008 | Canonical | 38 | Warning | Warning for distinct isoforms that do not share any regions |
| AalbF2 | Emr0001 | Canonical | 1 | Warning | Duplicate transcript found |
| AalbF2 | Emr0002 | Canonical | 137 | Warning | Incorrectly split gene parent? |
| Total |  |  | 20,454 |  |  |
| AalbF2 | Esf0036 | Non-canonical | 2,234 | Error | Value of a attribute contains unescaped “,” |
| AalbF2 | Ema0005 | Non-canonical | 3,994 | Info | Pseudogene has invalid child feature type |
| Total |  |  | 6,228 |  |  |
| AalbF3 | Esf0014 | Canonical | 1 | Error | ##gff-version” missing from the first line |
| AalbF3 | Esf0036 | Canonical | 1,060 | Error | Value of a attribute contains unescaped “,” |
| AalbF3 | Ema0006 | Canonical | 26 | Info | Wrong phase |
| AalbF3 | Ema0007 | Canonical | 110 | Warning | CDS and parent feature on different strands |
| AalbF3 | Ema0004 | Canonical | 5,079 | Info | Incomplete gene feature that should contain at least one mRNA, exon, and CDS |
| AalbF3 | Ema0005 | Canonical | 1,739 | Info | Pseudogene has invalid child feature type |
| AalbF3 | Ema0009 | Canonical | 1,957 | Warning | Incorrectly merged gene parent? Isoforms that do not share coding sequences are found |
| AalbF3 | Ema0002 | Canonical | 118 | Warning | Protein sequence contains internal stop codons |
| AalbF3 | Ema0008 | Canonical | 17 | Warning | Warning for distinct isoforms that do not share any regions |
| AalbF3 | Emr0002 | Canonical | 39 | Warning | Incorrectly split gene parent? |
| Total |  |  | 10,146 |  |  |
| AalbF3 | Esf0014 | Non-canonical | 1 | Error | ##gff-version” missing from the first line |
| AalbF3 | Esf0036 | Non-canonical | 1,060 | Error | Value of a attribute contains unescaped “,” |
| AalbF3 | Ema0005 | Non-canonical | 1,739 | Info | Pseudogene has invalid child feature type |
| Total |  |  | 2,800 |  |  |

**Table S17**. Count of genes with SNPs from the chip, based on the AalbF3 annotation, categorized by gene biotype, including protein coding genes, pseudogenes, small nuclear RNAs (snRNA), small nucleolar RNAs (snoRNA), and ribosomal RNAs (rRNA). "NA"= information not available.

| Gene biotype | Number of genes with SNPs |
| --- | --- |
| Protein coding | 17,461 |
| Pseudogene | 982 |
| NA | 448 |
| snRNA | 13 |
| snoRNA | 5 |
| rRNA | 2 |

**Table S1**8. Number of SNPs in putative immunity and diapause genes sorted by their type using the AalbF3 annotation.

| SNP type | Number |
| --- | --- |
| Synonymous variant | 415 |
| Intron variant | 409 |
| 5’ prime UTR variant | 91 |
| Total | 915 |

**Table S19**. SNPs in putative diapause genes, identified using the AalbF3 annotation, with columns indicating scaffold number, SNP position, reference allele, alternative allele, and functional impact levels (High, Moderate, Low, Modifier) determined by SnpEff. "Type of variant" denotes SNP genomic position, and "gene biotype" represents functional gene classification, including protein-coding, long non-coding RNA, and transfer RNA genes.

| Scaffold | Position | SNP | Reference allele | Alternative allele | Effect | Type | Biotype | Nucleotide change | Amino acid change | Gene ID |
| --- | --- | --- | --- | --- | --- | --- | --- | --- | --- | --- |
| 1.5 | 384,768 | AX-583746747 | G | A | Modifier | intron_variant | protein_coding | c.145+30C>T |  | LOC109402178 |
| 1.5 | 374,571 | AX-583746721 | A | C | Low | synonymous_variant | protein_coding | c.456T>G | p.Ala152Ala | LOC109413238 |
| 1.5 | 374,775 | AX-583750651 | A | G | Low | synonymous_variant | protein_coding | c.252T>C | p.Asp84Asp | LOC109413238 |
| 1.5 | 460,009 | AX-583747442 | G | A | Low | synonymous_variant | protein_coding | c.1212C>T | p.Pro404Pro | LOC109402173 |
| 1.5 | 460,904 | AX-583747485 | T | C | Low | synonymous_variant | protein_coding | c.432A>G | p.Leu144Leu | LOC109402173 |
| 3.1 | 3,300,814 | AX-580336192 | A | T | Modifier | 3_prime_UTR_variant | protein_coding | c.*729T>A |  | LOC109411232 |
| 3.1 | 3,463,101 | AX-580336870 | T | G | Modifier | 5_prime_UTR_variant | protein_coding | c.-341A>C |  | LOC109411232 |
| 3.1 | 3,463,774 | AX-580336878 | T | C | Modifier | 5_prime_UTR_variant | protein_coding | c.-1014A>G |  | LOC109411232 |
| 3.1 | 3,304,574 | AX-580338227 | C | T | Modifier | intron_variant | protein_coding | c.961+594G>A |  | LOC109411232 |
| 3.1 | 3,304,968 | AX-580338236 | A | G | Modifier | intron_variant | protein_coding | c.961+200T>C |  | LOC109411232 |
| 3.1 | 3,339,542 | AX-580336357 | T | A | Modifier | intron_variant | protein_coding | c.679-30795A>T |  | LOC109411232 |
| 3.1 | 3,340,340 | AX-580338346 | G | A | Modifier | intron_variant | protein_coding | c.679-31593C>T |  | LOC109411232 |
| 3.1 | 3,340,788 | AX-580336395 | A | T | Modifier | intron_variant | protein_coding | c.679-32041T>A |  | LOC109411232 |
| 3.1 | 3,377,418 | AX-580338434 | G | C | Modifier | intron_variant | protein_coding | c.678+13167C>G |  | LOC109411232 |
| 3.1 | 3,389,566 | AX-580336499 | A | T | Modifier | intron_variant | protein_coding | c.678+1019T>A |  | LOC109411232 |
| 3.1 | 3,389,812 | AX-580338487 | C | T | Modifier | intron_variant | protein_coding | c.678+773G>A |  | LOC109411232 |
| 3.1 | 3,390,039 | AX-580336526 | T | C | Modifier | intron_variant | protein_coding | c.678+546A>G |  | LOC109411232 |
| 3.1 | 3,401,309 | AX-580336571 | A | C | Modifier | intron_variant | protein_coding | c.392-170T>G |  | LOC109411232 |
| 3.1 | 3,407,679 | AX-580338576 | T | C | Modifier | intron_variant | protein_coding | c.392-6540A>G |  | LOC109411232 |
| 3.1 | 3,408,533 | AX-580338597 | A | G | Modifier | intron_variant | protein_coding | c.392-7394T>C |  | LOC109411232 |
| 3.1 | 3,427,972 | AX-580338657 | A | G | Modifier | intron_variant | protein_coding | c.392-26833T>C |  | LOC109411232 |
| 3.1 | 3,439,964 | AX-580338775 | T | C | Modifier | intron_variant | protein_coding | c.391+22406A>G |  | LOC109411232 |
| 3.1 | 3,440,228 | AX-580338784 | G | A | Modifier | intron_variant | protein_coding | c.391+22142C>T |  | LOC109411232 |
| 3.1 | 3,462,068 | AX-580336816 | T | C | Modifier | intron_variant | protein_coding | c.391+302A>G |  | LOC109411232 |
| 3.19 | 2,229,464 | AX-581509085 | A | G | Modifier | 3_prime_UTR_variant | protein_coding | c.*44A>G |  | LOC109398750 |
| 3.19 | 2,270,349 | AX-581511297 | G | T | Modifier | intron_variant | protein_coding | c.-8+9G>T |  | LOC109398746 |
| 3.19 | 2,289,280 | AX-581511654 | A | G | Modifier | intron_variant | protein_coding | c.972+18T>C |  | LOC109398754 |
| 3.19 | 2,233,284 | AX-581509100 | G | A | Low | synonymous_variant | protein_coding | c.498C>T | p.Asn166Asn | LOC109398750 |
| 3.19 | 2,235,931 | AX-581510984 | G | T | Low | synonymous_variant | protein_coding | c.306G>T | p.Pro102Pro | LOC109398756 |
| 3.19 | 2,260,721 | AX-581509393 | G | A | Low | synonymous_variant | protein_coding | c.1296C>T | p.Thr432Thr | LOC109398753 |
| 3.19 | 2,271,368 | AX-581509585 | C | T | Low | synonymous_variant | protein_coding | c.888C>T | p.Ser296Ser | LOC109398746 |
| 3.19 | 2,271,680 | AX-581511336 | C | T | Low | synonymous_variant | protein_coding | c.1200C>T | p.Asn400Asn | LOC109398746 |

**Table S20**. Nonsynonymous SNPs in immunity genes, using the AalbF3 annotation. Columns include scaffold number, SNP position, reference allele, alternative allele, and functional impact levels (High, Moderate, Low, Modifier) determined by SnpEff. "Type of variant" indicates SNP genomic position, and "gene biotype" represents gene functional classification (e.g., protein-coding, long non-coding RNA, transfer RNA).

| Scaffold | Position | SNP | Reference allele | Alternative allele | Effect | Type | Biotype | Nucleotide change | Amino acid change | GeneID |
| --- | --- | --- | --- | --- | --- | --- | --- | --- | --- | --- |
| 1.1 | 10,697,741 | AX-583056929 | G | A | Moderate | missense_variant | protein_coding | c.491G>A | p.Arg164His | XM_029855176.1-1 |
| 1.1 | 10,699,650 | AX-583058970 | A | C | Moderate | missense_variant | protein_coding | c.196T>G | p.Ser66Ala | LOC109430086 |
| 1.106 | 2,512,509 | AX-583109537 | C | A | Moderate | missense_variant | protein_coding | c.1405C>A | p.His469Asn | LOC115257625 |
| 1.113 | 5,762,762 | AX-583151909 | A | G | Moderate | missense_variant | protein_coding | c.623A>G | p.Glu208Gly | LOC109415428 |
| 1.113 | 5,763,562 | AX-583152046 | A | G | Moderate | missense_variant | protein_coding | c.1423A>G | p.Ile475Val | LOC109415428 |
| 1.131 | 5,279,847 | AX-583254712 | C | T | Moderate | missense_variant | protein_coding | c.626G>A | p.Ser209Asn | LOC109399009 |
| 1.147 | 5,839,558 | AX-583431723 | T | C | Moderate | missense_variant | protein_coding | c.775A>G | p.Ile259Val | XM_029868010.1-1 |
| 1.147 | 5,840,353 | AX-583434252 | T | G | Moderate | missense_variant | protein_coding | c.193A>C | p.Thr65Pro | XM_029868010.1-1 |
| 1.25 | 1,021,102 | AX-583588144 | A | G | Moderate | missense_variant | protein_coding | c.424A>G | p.Lys142Glu | LOC109402615 |
| 1.36 | 4,880,818 | AX-583694151 | T | C | Moderate | missense_variant | protein_coding | c.445A>G | p.Lys149Glu | LOC109397206 |
| 1.79 | 54,655 | AX-583972510 | G | A | Moderate | missense_variant | protein_coding | c.599C>T | p.Ser200Leu | LOC115260154 |
| 1.85 | 17,046,403 | AX-584044965 | C | T | Moderate | missense_variant | protein_coding | c.524C>T | p.Thr175Met | LOC109424643 |
| 1.85 | 23,680,098 | AX-584075828 | A | G | Moderate | missense_variant | protein_coding | c.4819A>G | p.Lys1607Glu | XM_029862069.1-1 |
| 1.85 | 23,681,315 | AX-584075910 | A | G | Moderate | missense_variant | protein_coding | c.5722A>G | p.Met1908Val | XM_029862069.1-1 |
| 1.85 | 27,167,461 | AX-584094434 | G | C | Moderate | missense_variant | protein_coding | c.27C>G | p.Asp9Glu | LOC109431512 |
| 1.91 | 3,380,846 | AX-584233670 | A | G | Moderate | missense_variant | protein_coding | c.2011A>G | p.Asn671Asp | LOC109412409 |
| 1.91 | 5,039,401 | AX-584245979 | A | G | Moderate | missense_variant | protein_coding | c.1156A>G | p.Ser386Gly | LOC109430356 |
| 1.91 | 8,897,363 | AX-584263120 | G | T | Moderate | missense_variant | protein_coding | c.880C>A | p.Leu294Met | LOC109621626 |
| 1.91 | 8,897,632 | AX-584265058 | A | G | Moderate | missense_variant | protein_coding | c.611T>C | p.Met204Thr | LOC109621626 |
| 2.103 | 2,573,871 | AX-584332894 | G | C | Moderate | missense_variant | protein_coding | c.471C>G | p.His157Gln | LOC115266575 |
| 2.103 | 2,574,411 | AX-584332912 | A | T | Moderate | missense_variant | protein_coding | c.28T>A | p.Ser10Thr | LOC115266575 |
| 2.138 | 335,263 | AX-584531554 | A | G | Moderate | missense_variant | protein_coding | c.172A>G | p.Ser58Gly | LOC109409282 |
| 2.14 | 12,158,966 | AX-585060557 | T | C | Moderate | missense_variant | protein_coding | c.7A>G | p.Thr3Ala | XM_029859293.1-1 |
| 2.14 | 12,647,106 | AX-584552253 | G | C | Moderate | missense_variant | protein_coding | c.964G>C | p.Gly322Arg | LOC109409279 |
| 2.146 | 419,711 | AX-585089323 | A | T | Moderate | missense_variant | protein_coding | c.701A>T | p.Asp234Val | LOC109407295 |
| 2.167 | 1,433,370 | AX-585168461 | G | A | Moderate | missense_variant | protein_coding | c.199G>A | p.Val67Ile | XM_029853094.1-1 |
| 2.17 | 3,924,782 | AX-585190434 | G | A | Moderate | missense_variant | protein_coding | c.587G>A | p.Arg196Gln | XM_019684995.2-1 |
| 2.17 | 3,926,378 | AX-584681782 | C | G | Moderate | missense_variant | protein_coding | c.2050C>G | p.Pro684Ala | XM_019684995.2-1 |
| 2.17 | 4,004,422 | AX-584682128 | C | T | Moderate | missense_variant | protein_coding | c.11C>T | p.Thr4Met | exon-XM_019684993.2-1 |
| 2.17 | 13,726,115 | AX-582441613 | T | C | Moderate | missense_variant | protein_coding | c.671A>G | p.Lys224Arg | LOC109410220 |
| 2.175 | 780,098 | AX-585206011 | C | T | Moderate | missense_variant | protein_coding | c.956C>T | p.Ser319Leu | -XM_029852847.1-1 |
| 2.175 | 800,927 | AX-584697392 | C | A | Moderate | missense_variant | protein_coding | c.3090C>A | p.Ser1030Arg | exon-XM_029852847.1-1 |
| 2.27 | 1,560,585 | AX-579525195 | A | T | Moderate | missense_variant | protein_coding | c.1326T>A | p.Asp442Glu | LOC109419663 |
| 2.42 | 12,014,338 | AX-579673469 | T | A | Moderate | missense_variant | protein_coding | c.961T>A | p.Ser321Thr | LOC109426828 |
| 2.6 | 16,276,549 | AX-579809339 | T | A | Moderate | missense_variant | protein_coding | c.490T>A | p.Ser164Thr | XM_029878835.1-1 |
| 2.6 | 16,296,793 | AX-579809489 | C | T | Moderate | missense_variant | protein_coding | c.586C>T | p.Pro196Ser | XM_019700969.2-1 |
| 2.68 | 5,446,027 | AX-579896611 | G | T | Moderate | missense_variant | protein_coding | c.1060C>A | p.Leu354Ile | LOC109622615 |
| 2.68 | 5,446,516 | AX-579896667 | A | G | Moderate | missense_variant | protein_coding | c.571T>C | p.Trp191Arg | LOC109622615 |
| 2.87 | 2,149,902 | AX-580019993 | G | A | Moderate | missense_variant | protein_coding | c.49G>A | p.Ala17Thr | LOC109398084 |
| 2.87 | 2,150,355 | AX-580020020 | A | G | Moderate | missense_variant | protein_coding | c.370A>G | p.Ile124Val | LOC109398084 |
| 2.87 | 2,174,851 | AX-580020260 | A | G | Moderate | missense_variant | protein_coding | c.1162A>G | p.Thr388Ala | LOC109398030 |
| 2.95 | 352,322 | AX-580191265 | T | A | Moderate | missense_variant | protein_coding | c.109A>T | p.Thr37Ser | LOC115269749 |
| 3.116 | 5,061,100 | AX-580503305 | T | C | Moderate | missense_variant | protein_coding | c.619A>G | p.Arg207Gly | XM_029857358.1-1 |
| 3.122 | 5,261,799 | AX-580598894 | A | C | Moderate | missense_variant | protein_coding | c.801T>G | p.Asp267Glu | XM_029865713.1-1 |
| 3.151 | 3,436,049 | AX-580842317 | T | C | Moderate | missense_variant | protein_coding | c.1045A>G | p.Thr349Ala | XM_029875170.1-1 |
| 3.159 | 1,104,660 | AX-580902042 | A | G | Moderate | missense_variant | protein_coding | c.638T>C | p.Val213Ala | LOC109409956 |
| 3.17 | 18,459,269 | AX-581338723 | T | C | Moderate | missense_variant | protein_coding | c.28A>G | p.Thr10Ala | XM_029875820.1-1 |
| 3.18 | 2,874,117 | AX-581428184 | T | C | Moderate | missense_variant | protein_coding | c.1267A>G | p.Ser423Gly | XM_029875979.1-1 |
| 3.2 | 8,846,714 | AX-581596089 | T | C | Moderate | missense_variant | protein_coding | c.287A>G | p.Asn96Ser | LOC109413737 |
| 3.32 | 3,322,424 | AX-581763570 | T | C | Moderate | missense_variant | protein_coding | c.1193T>C | p.Val398Ala | LOC109415245 |
| 3.32 | 3,408,703 | AX-581765674 | G | A | Moderate | missense_variant | protein_coding | c.592G>A | p.Gly198Ser | LOC109414897 |
| 3.32 | 3,418,991 | AX-581765806 | T | G | Moderate | missense_variant | protein_coding | c.324T>G | p.Asp108Glu | LOC109416024 |
| 3.4 | 5,487,127 | AX-581830792 | T | A | Moderate | missense_variant | protein_coding | c.295A>T | p.Thr99Ser | LOC109399310 |
| 3.58 | 5,278,791 | AX-582051662 | C | A | Moderate | missense_variant | protein_coding | c.316G>T | p.Ala106Ser | XM_019673611.2-1 |
| 3.75 | 16,587,391 | AX-582760777 | A | G | Moderate | missense_variant | protein_coding | c.334A>G | p.Asn112Asp | LOC109398879 |
| 3.75 | 16,589,393 | AX-582761776 | C | T | Moderate | missense_variant | protein_coding | c.317G>A | p.Arg106Lys | LOC109398826 |
| 3.77 | 6,092,180 | AX-582825108 | G | A | Moderate | missense_variant | protein_coding | c.92G>A | p.Arg31Lys | LOC109420317 |
| 3.93 | 5,614,846 | AX-583002481 | G | A | Moderate | missense_variant | protein_coding | c.1039G>A | p.Asp347Asn | XM_029860991.1-1 |

**Table S21**. Number of SNPs per chromosome and per 1Mb window for SNP chip data from wild samples after quality control (N = number of SNPs)

| Chromosome | SNPs (N) | SNPs per 1Mb window | Number of windows |
| --- | --- | --- | --- |
| 1 | 18,602 | 50.55 | 368 |
| 2 | 34,636 | 59.92 | 578 |
| 3 | 29,493 | 60.56 | 487 |

**Table S22.** Linkage disequilibrium (LD) half-life for native populations of *Ae. albopictus* in Asia. The half-distance in kilobases (kb) is show for each chromosome (Chr1, Chr2, and Chr3).

| **Abbreviation** | **Country** | **City** | **Chr1** | **Chr2** | **Chr3** |
| --- | --- | --- | --- | --- | --- |
| CAM | Cambodia | Phnom Penh | 18.9 | 115.2 | 139.4 |
| HAI | China | Hainan | 1.5 | 63.8 | 66.0 |
| HUN | China | Hunan | 12.7 | 57.9 | 57.0 |
| YUN | China | Yunnan | 16.1 | 335.6 | 1.0 |
| BEN | India | Bengaluru | 18.9 | 194.6 | 207.6 |
| INJ | Indonesia | Jakarta | 37.3 | 91.1 | 58.8 |
| SUF | Indonesia | Sulawesi (Forest) | 39.9 | 333.7 | 163.1 |
| SUU | Indonesia | Sulawesi (Urban) | 63.8 | 353.5 | 278.7 |
| KAG | Japan | Kagoshima | 45.7 | 30.3 | 3.9 |
| KAN | Japan | Kanazawa | 42.0 | 46.4 | 57.7 |
| OKI | Japan | Okinawa | 6.1 | 21.4 | 63.0 |
| UTS | Japan | Utsunomiya | 33.9 | 14.6 | 25.0 |
| MAT | Malaysia | Tambun | 25.5 | 157.4 | 168.3 |
| TAI | Taiwan | Tainan | 9.9 | 261.2 | 121.4 |
| CHA | Thailand | Chanthaburi | 12.3 | 174.9 | 96.6 |
| KAC | Thailand | Kanchanaburi | 22.3 | 378.1 | 46.7 |
| LAM | Thailand | Lampang | 18.9 | 281.7 | 88.8 |
| SSK | Thailand | Sisaket | 3.1 | 170.6 | 147.8 |
| HOC | Vietnam | Ho Chi Minh City | 54.6 | 182.5 | 146.0 |
| QNC | Vietnam | Quy Nhon City | 3.5 | 118.2 | 6.6 |

**Table S23.** Fst estimates for each population using the LD2 SNP set, with 57,780 SNPs, for populations with at least four mosquitoes. On the upper right are the pairwise Fst estimates. On the lower left are the geographical distances in Km, sorted from low to high, from the left to the right. The estimates for the intergenic and LD1 SNP sets are in File S14.

| Population | SSK | QNC | CAM | HOC | CHA | HAI | HAN | KAC | LAM | YUN | MAT | HUN | KLP | TAI | OKI | SUF | INJ | SUU | KAT | KAG | INW | BEN | KAN | UTS | KUN |
| --- | --- | --- | --- | --- | --- | --- | --- | --- | --- | --- | --- | --- | --- | --- | --- | --- | --- | --- | --- | --- | --- | --- | --- | --- | --- |
| SSK |  | 0.08 | 0.01 | 0.06 | 0.00 | 0.05 | 0.05 | 0.00 | 0.01 | 0.00 | 0.02 | 0.07 | 0.06 | 0.18 | 0.12 | 0.14 | 0.13 | 0.10 | 0.10 | 0.13 | 0.16 | 0.03 | 0.18 | 0.15 | 0.16 |
| QNC | 548 |  | 0.08 | 0.13 | 0.08 | 0.12 | 0.13 | 0.08 | 0.09 | 0.08 | 0.10 | 0.14 | 0.14 | 0.25 | 0.19 | 0.22 | 0.20 | 0.17 | 0.18 | 0.19 | 0.24 | 0.10 | 0.24 | 0.21 | 0.24 |
| CAM | 402 | 528 |  | 0.05 | 0.00 | 0.04 | 0.04 | 0.00 | 0.01 | 0.00 | 0.02 | 0.06 | 0.05 | 0.17 | 0.11 | 0.14 | 0.13 | 0.09 | 0.10 | 0.12 | 0.16 | 0.03 | 0.17 | 0.14 | 0.15 |
| HOC | 547 | 436 | 209 |  | 0.06 | 0.03 | 0.05 | 0.06 | 0.06 | 0.06 | 0.07 | 0.04 | 0.09 | 0.16 | 0.10 | 0.17 | 0.17 | 0.10 | 0.15 | 0.10 | 0.21 | 0.08 | 0.15 | 0.12 | 0.21 |
| CHA | 368 | 782 | 329 | 537 |  | 0.05 | 0.05 | 0.00 | 0.01 | 0.00 | 0.02 | 0.07 | 0.06 | 0.18 | 0.12 | 0.14 | 0.13 | 0.10 | 0.10 | 0.13 | 0.16 | 0.02 | 0.18 | 0.15 | 0.15 |
| HAI | 722 | 604 | 987 | 991 | 1087 |  | 0.04 | 0.05 | 0.05 | 0.05 | 0.06 | 0.04 | 0.08 | 0.14 | 0.09 | 0.16 | 0.15 | 0.09 | 0.13 | 0.10 | 0.19 | 0.07 | 0.14 | 0.11 | 0.19 |
| HAN | 678 | 882 | 1059 | 1146 | 1019 | 441 |  | 0.04 | 0.05 | 0.04 | 0.06 | 0.05 | 0.09 | 0.18 | 0.11 | 0.18 | 0.16 | 0.11 | 0.14 | 0.12 | 0.22 | 0.07 | 0.17 | 0.13 | 0.22 |
| KAC | 530 | 1047 | 647 | 856 | 320 | 1218 | 1028 |  | 0.01 | 0.00 | 0.02 | 0.07 | 0.06 | 0.19 | 0.12 | 0.15 | 0.13 | 0.10 | 0.10 | 0.13 | 0.17 | 0.02 | 0.18 | 0.15 | 0.17 |
| LAM | 624 | 1155 | 951 | 1139 | 692 | 1070 | 733 | 475 |  | 0.01 | 0.02 | 0.07 | 0.06 | 0.19 | 0.12 | 0.15 | 0.13 | 0.10 | 0.11 | 0.14 | 0.17 | 0.03 | 0.19 | 0.16 | 0.17 |
| YUN | 1087 | 1449 | 1487 | 1627 | 1323 | 1035 | 601 | 1179 | 714 |  | 0.02 | 0.07 | 0.05 | 0.18 | 0.12 | 0.14 | 0.13 | 0.10 | 0.10 | 0.13 | 0.16 | 0.02 | 0.18 | 0.15 | 0.16 |
| MAT | 1217 | 1348 | 875 | 912 | 893 | 1862 | 1894 | 1060 | 1531 | 2208 |  | 0.08 | 0.06 | 0.19 | 0.13 | 0.15 | 0.14 | 0.11 | 0.11 | 0.14 | 0.18 | 0.04 | 0.19 | 0.16 | 0.17 |
| HUN | 1595 | 1565 | 1929 | 1954 | 1956 | 966 | 954 | 1981 | 1637 | 1108 | 2799 |  | 0.10 | 0.13 | 0.07 | 0.17 | 0.16 | 0.10 | 0.15 | 0.08 | 0.20 | 0.09 | 0.12 | 0.09 | 0.20 |
| KLP | 1365 | 1445 | 1003 | 1011 | 1055 | 1984 | 2042 | 1234 | 1704 | 2375 | 177 | 2932 |  | 0.24 | 0.16 | 0.20 | 0.17 | 0.14 | 0.15 | 0.17 | 0.24 | 0.08 | 0.22 | 0.19 | 0.24 |
| TAI | 1886 | 1548 | 2061 | 1982 | 2237 | 1180 | 1497 | 2397 | 2219 | 1928 | 2894 | 986 | 2979 |  | 0.17 | 0.29 | 0.27 | 0.22 | 0.26 | 0.19 | 0.34 | 0.20 | 0.23 | 0.20 | 0.34 |
| OKI | 2758 | 2410 | 2929 | 2839 | 3110 | 2047 | 2328 | 3265 | 3060 | 2678 | 3747 | 1599 | 3822 | 873 |  | 0.22 | 0.21 | 0.16 | 0.20 | 0.12 | 0.26 | 0.14 | 0.17 | 0.14 | 0.26 |
| SUF | 2567 | 2117 | 2245 | 2046 | 2557 | 2614 | 2986 | 2874 | 3182 | 3566 | 2220 | 3406 | 2115 | 2777 | 3280 |  | 0.08 | 0.04 | 0.06 | 0.23 | 0.10 | 0.15 | 0.27 | 0.24 | 0.30 |
| INJ | 2390 | 2241 | 1989 | 1889 | 2160 | 2845 | 3034 | 2393 | 2844 | 3467 | 1363 | 3805 | 1188 | 3562 | 4294 | 1535 |  | 0.09 | 0.09 | 0.22 | 0.07 | 0.14 | 0.27 | 0.24 | 0.26 |
| SUU | 2797 | 2309 | 2506 | 2300 | 2827 | 2752 | 3151 | 3147 | 3420 | 3744 | 2565 | 3468 | 2469 | 2747 | 3154 | 381 | 1911 |  | 0.07 | 0.17 | 0.13 | 0.11 | 0.21 | 0.18 | 0.25 |
| KAT | 2412 | 2922 | 2724 | 2921 | 2424 | 2648 | 2207 | 2119 | 1789 | 1640 | 3069 | 2611 | 3242 | 3537 | 4204 | 4967 | 4429 | 5209 |  | 0.21 | 0.11 | 0.11 | 0.25 | 0.22 | 0.23 |
| KAG | 3232 | 2942 | 3446 | 3377 | 3595 | 2510 | 2721 | 3713 | 3450 | 2967 | 4289 | 1860 | 4376 | 1397 | 618 | 3893 | 4901 | 3756 | 4367 |  | 0.26 | 0.15 | 0.13 | 0.09 | 0.26 |
| INW | 3271 | 2880 | 2908 | 2727 | 3190 | 3419 | 3762 | 3492 | 3859 | 4320 | 2650 | 4248 | 2505 | 3635 | 4110 | 859 | 1528 | 973 | 5611 | 4718 |  | 0.17 | 0.32 | 0.28 | 0.34 |
| BEN | 2894 | 3424 | 2976 | 3174 | 2660 | 3487 | 3134 | 2377 | 2419 | 2806 | 2751 | 3913 | 2869 | 4631 | 5450 | 4970 | 3876 | 5307 | 1828 | 5773 | 5347 |  | 0.19 | 0.17 | 0.13 |
| KAN | 3991 | 3725 | 4223 | 4161 | 4357 | 3270 | 3444 | 4456 | 4160 | 3619 | 5073 | 2532 | 5163 | 2185 | 1390 | 4621 | 5685 | 4452 | 4880 | 791 | 5424 | 6410 |  | 0.10 | 0.32 |
| UTS | 4249 | 3965 | 4470 | 4399 | 4613 | 3527 | 3715 | 4722 | 4435 | 3902 | 5311 | 2811 | 5396 | 2418 | 1590 | 4755 | 5874 | 4566 | 5167 | 1023 | 5539 | 6696 | 288 |  | 0.28 |
| KUN | 3582 | 4070 | 3572 | 3749 | 3288 | 4241 | 3934 | 3052 | 3202 | 3681 | 3124 | 4777 | 3196 | 5414 | 6262 | 5293 | 3986 | 5658 | 2779 | 6631 | 5513 | 957 | 7300 | 7583 |  |

**Table S24.** Fst estimates amongst populations above 30^o^N and amongst populations below 30^o^N in the ancestral range of *Ae. albopictus* using different SNP data sets. The first value in the “Mean” and Fst columns represents estimates based on only intergenic SNPs (9,483), followed by two values between parentheses. The first value in parentheses is from estimates using the SNP set LD1 (20,931 SNPs), and the second value is from the SNP set LD2 (57,780 SNPs).

| Latitude | Mean | Region | Mean | Country | Mean | City | Code | Fst |
| --- | --- | --- | --- | --- | --- | --- | --- | --- |
| Above 30^0^N | 0.12 (0.18, 0.17) | East | 0.10 (0.15, 0.14) | Japan | 0.12 (0.17, 0.17) | Kagoshima | KAG | 0.11 (0.16, 0.15) |
|  |  |  |  |  |  |  |  |  |
|  |  |  |  |  |  | Kanazawa | KAN | 0.14 (0.20, 0.20) |
|  |  |  |  |  |  |  |  |  |
|  |  |  |  |  |  | Utsunomiya | UTS | 0.11 (0.17, 0.17) |
|  |  |  |  |  |  |  |  |  |
| Below 30^0^N | 0.09 (0.13, 0.13) |  |  |  |  | Okinawa | OKI | 0.11 (0.15, 0.15) |
|  |  |  |  |  |  |  |  |  |
|  |  |  |  | China | 0.07 (0.09, 0.09) | Hainan | HAI | 0.07 (0.09, 0.09) |
|  |  |  |  |  |  |  |  |  |
|  |  |  |  |  |  | Hunan | HUN | 0.07 (0.10, 0.10) |
|  |  |  |  |  |  |  |  |  |
|  |  |  |  |  |  | Yunnan | YUN | 0.06 (0.08, 0.08) |
|  |  |  |  |  |  |  |  |  |
|  |  |  |  | Taiwan | 0.18 (0.21, 0.21) | Tainan | TAI | 0.18 (0.21, 0.21) |
|  |  |  |  |  |  |  |  |  |
|  |  | South | 0.12 (0.16, 0.16) | India | 0.07 (0.10, 0.10) | Bengaluru | BEN | 0.07 (0.10, 0.10) |
|  |  |  |  |  |  |  |  |  |
|  |  |  |  | Maldives | 0.19 (0.23, 0.23) | Kunfunadhoo | KUN | 0.19 (0.23, 0.23) |
|  |  |  |  |  |  |  |  |  |
|  |  |  |  | Nepal | 0.11 (0.14, 0.14) | Kathmandu | KAT | 0.11 (0.14, 0.14) |
|  |  |  |  |  |  |  |  |  |
|  |  | Southeast | 0.09 (0.12, 0.12) | Cambodia | 0.05 (0.08, 0.08) | Phnom Penh | CAM | 0.05 (0.08, 0.08) |
|  |  |  |  |  |  |  |  |  |
|  |  |  |  | Indonesia | 0.11 (0.16, 0.17) | Jakarta | INJ | 0.11 (0.16, 0.16) |
|  |  |  |  |  |  |  |  |  |
|  |  |  |  |  |  | Sulawesi (Forest) | SUF | 0.11 (0.16, 0.17) |
|  |  |  |  |  |  |  |  |  |
|  |  |  |  |  |  | Sulawesi (Urban) | SUU | 0.09 (0.12, 0.13) |
|  |  |  |  |  |  |  |  |  |
|  |  |  |  |  |  | Wainyapu | INW | 0.14 (0.19, 0.20) |
|  |  |  |  |  |  |  |  |  |
|  |  |  |  | Malaysia | 0.09 (0.11, 0.11) | Kuala Lumpur | KLP | 0.1 (0.13, 0.13) |
|  |  |  |  |  |  |  |  |  |
|  |  |  |  |  |  | Tambun | MAT | 0.07 (0.09, 0.09) |
|  |  |  |  |  |  |  |  |  |
|  |  |  |  | Thailand | 0.06 (0.09, 0.08) | Chanthaburi | CHA | 0.06 (0.08, 0.08) |
|  |  |  |  |  |  |  |  |  |
|  |  |  |  |  |  | Kanchanaburi | KAC | 0.06 (0.09, 0.08) |
|  |  |  |  |  |  |  |  |  |
|  |  |  |  |  |  | Lampang | LAM | 0.06 (0.09, 0.09) |
|  |  |  |  |  |  |  |  |  |
|  |  |  |  |  |  | Sisaket | SSK | 0.06 (0.08, 0.08) |
|  |  |  |  |  |  |  |  |  |
|  |  |  |  | Vietnam | 0.09 (0.12, 0.12) | Hanoi | HAN | 0.08 (0.10, 0.10) |
|  |  |  |  |  |  |  |  |  |
|  |  |  |  |  |  | Ho Chi Minh City | HOC | 0.08 (0.10, 0.10) |
|  |  |  |  |  |  |  |  |  |
|  |  |  |  |  |  | Quy Nhon City | QNC | 0.12 (0.15, 0.15) |
|  |  |  |  |  |  |  |  |  |
